# Supplementary material for: Discovery of 20 novel ribosomal leader candidates in bacteria and archaea
Source: BMC Microbiol. 2020 May 24;20:130. doi: 10.1186/s12866-020-01823-6 (PMC7247131; doi:10.1186/s12866-020-01823-6)
Supplement: Supplementary file 2 — Additional file 2. Alignments and information on downstream genes and taxonomy for all predicted r-leader motifs. [file 12866_2020_1823_MOESM2_ESM.pdf]

Supplementary File 1: alignments and information on downstream genes and taxonomy
Discovery of 20 novel ribosomal leader candidates in bacteria and archaea

Iris Eckert & Zasha Weinberg

Note: the presentation and explanatory text of this supplementary data on novel RNA motifs follows the pattern of a presentation of previously found conserved RNA motifs (Weinberg, *et al.*, 2010).

Contents

1 L2-Alphaproteobacteria **4**
1.1 Taxa . . . 4
1.2 Gene contexts . . . 7
1.3 Conserved domains . . . 34
1.4 Multiple-sequence alignment . . . 35

2 L4-Archaeoglobi **49**
2.1 Taxa . . . 49
2.2 Gene contexts . . . 51
2.3 Conserved domains . . . 116
2.4 Multiple-sequence alignment . . . 118

3 L13-Bacteroidia **141**
3.1 Taxa . . . 141
3.2 Gene contexts . . . 143
3.3 Conserved domains . . . 171
3.4 Multiple-sequence alignment . . . 171

4 eL15-Euryarchaeota **199**
4.1 Taxa . . . 199
4.2 Gene contexts . . . 203
4.3 Conserved domains . . . 235
4.4 Multiple-sequence alignment . . . 236

5 L17-Actino-Proteobacteria **261**
5.1 Taxa . . . 261
5.2 Notes . . . 268
5.3 Gene contexts . . . 268
5.4 Conserved domains . . . 280
5.5 Multiple-sequence alignment . . . 281

6 L19-Flavobacteria **302**
6.1 Taxa . . . 302
6.2 Gene contexts . . . 305
6.3 Conserved domains . . . 354
6.4 Multiple-sequence alignment . . . 356

7 L20-Deltaproteobacteria **388**
7.1 Taxa . . . 388
7.2 Gene contexts . . . 388
7.3 Conserved domains . . . 394
7.4 Multiple-sequence alignment . . . 394

8 L25-Gammaproteobacteria **399**
8.1 Taxa . . . 399
8.2 Multiple-sequence alignment . . . 477

9 L31-Actinobacteria **510**
9.1 Taxa . . . 510
9.2 Gene contexts . . . 526
9.3 Conserved domains . . . 579
9.4 Multiple-sequence alignment . . . 581

10 L31-Coriobacteria **622**
10.1 Taxa . . . 622
10.2 Gene contexts . . . 622
10.3 Conserved domains . . . 629
10.4 Multiple-sequence alignment . . . 629

11 L31-Firmicutes **631**
11.1 Taxa . . . 631
11.2 Notes . . . 729
11.3 Gene contexts . . . 729
11.4 Conserved domains . . . 897
11.5 Multiple-sequence alignment . . . 901

12 L31-Gammaproteobacteria **929**
12.1 Taxa . . . 929
12.2 Gene contexts . . . 1024
12.3 Conserved domains . . . 1214
12.4 Multiple-sequence alignment . . . 1216

13 L31-Corynebacteriaceae **1252**
13.1 Taxa . . . 1252
13.2 Gene contexts . . . 1255
13.3 Conserved domains . . . 1262
13.4 Multiple-sequence alignment . . . 1263

14 S4-Bacteroidia **1267**
14.1 Taxa . . . 1267
14.2 Gene contexts . . . 1268
14.3 Conserved domains . . . 1283
14.4 Multiple-sequence alignment . . . 1283

15 S4-Flavobacteria **1297**
15.1 Taxa . . . 1297
15.2 Gene contexts . . . 1299
15.3 Conserved domains . . . 1324
15.4 Multiple-sequence alignment . . . 1325

16 S4-Clostridia **1337**
16.1 Taxa . . . 1337
16.2 Gene contexts . . . 1338
16.3 Conserved domains . . . 1351
16.4 Multiple-sequence alignment . . . 1352

17 S4-Fusobacteriales **1383**
17.1 Taxa . . . 1383
17.2 Gene contexts . . . 1385
17.3 Conserved domains . . . 1402
17.4 Multiple-sequence alignment . . . 1403

18 S6-S18-Chlorobi **1408**
18.1 Taxa . . . 1408
18.2 Gene contexts . . . 1408
18.3 Conserved domains . . . 1410
18.4 Multiple-sequence alignment . . . 1411

19 S10-Clostridia **1412**
19.1 Taxa . . . 1412
19.2 Gene contexts . . . 1414
19.3 Conserved domains . . . 1461
19.4 Multiple-sequence alignment . . . 1462

20 S15-Halobacteria **1499**
20.1 Taxa . . . 1499
20.2 Gene contexts . . . 1501
20.3 Conserved domains . . . 1508
20.4 Multiple-sequence alignment . . . 1509

21 S15-Methanobacteria **1516**
21.1 Taxa . . . 1516
21.2 Gene contexts . . . 1517
21.3 Conserved domains . . . 1522
21.4 Multiple-sequence alignment . . . 1523

22 S15-Flavobacteria **1531**
22.1 Taxa . . . 1531
22.2 Notes . . . 1535
22.3 Gene contexts . . . 1536
22.4 Conserved domains . . . 1586
22.5 Multiple-sequence alignment . . . 1586

23 S16-Flavobacteria **1681**
23.1 Taxa . . . 1681
23.2 Gene contexts . . . 1682
23.3 Conserved domains . . . 1686
23.4 Multiple-sequence alignment . . . 1687

1 L2-Alphaproteobacteria

1.1 Taxa

The taxonomy of each organism containing a putative L2-Alphaproteobacteria RNA is listed, with abbreviations identifying each hit (e.g., "Eco-1-1" and "Eco-1-2" might hypothetically represent two distinct RNAs in *E. coli*). The abbreviations will be used to identify each individual L2-Alphaproteobacteria RNA in Sections 1.2 and 1.4:.

**abbrev. of hits**	**taxonomy of species**
Aat-1-1	Bacteria Proteobacteria Alphaproteobacteria Rhodobacterales Rhodobacteraceae *Actibacterium atlanticum*
Amu-1-1	Bacteria Proteobacteria Alphaproteobacteria Rhodobacterales Rhodobacteraceae *Actibacterium mucosum* KCTC 23349
Aat-2-1	Bacteria Proteobacteria Alphaproteobacteria Rhodobacterales Rhodobacteraceae *Aestuariivita atlantica*
Abo-1-1	Bacteria Proteobacteria Alphaproteobacteria Rhodobacterales Rhodobacteraceae *Aestuariivita boseongensis*

Cba-1-1	Bacteria Proteobacteria Alphaproteobacteria Rhodobacterales Rhodobacteraceae *Celeribacter baekdonensis* B30
Csp-1-1	Bacteria Proteobacteria Alphaproteobacteria Rhodobacterales Rhodobacteraceae *Citreicella sp.* 357
Csp-2-1	Bacteria Proteobacteria Alphaproteobacteria Rhodobacterales Rhodobacteraceae *Citreicella sp.* SE45
Dsp-1-1	Bacteria Proteobacteria Alphaproteobacteria Rhodobacterales Rhodobacteraceae *Defluviimonas sp.* 20V17
Dsh-1-1	Bacteria Proteobacteria Alphaproteobacteria Rhodobacterales Rhodobacteraceae *Dinoroseobacter shibae* DFL 12
Gne-1-1	Bacteria Proteobacteria Alphaproteobacteria Rhodobacterales Rhodobacteraceae *Gemmobacter nectariphilus* DSM 15620
Hma-1-1	Bacteria Proteobacteria Alphaproteobacteria Rhodobacterales Rhodobacteraceae *Haematobacter massiliensis*
Hmi-1-1	Bacteria Proteobacteria Alphaproteobacteria Rhodobacterales Rhodobacteraceae *Haematobacter missouriensis*
Hna-1-1	Bacteria Proteobacteria Alphaproteobacteria Rhodobacterales Rhodobacteraceae *Halocynthiibacter namhaensis*
Hsp-1-1	Bacteria Proteobacteria Alphaproteobacteria Rhodobacterales Rhodobacteraceae *Halocynthiibacter sp.* PAMC 20958
Jaq-1-1	Bacteria Proteobacteria Alphaproteobacteria Rhodobacterales Rhodobacteraceae *Jannaschia aquimarina*
Jsp-1-1	Bacteria Proteobacteria Alphaproteobacteria Rhodobacterales Rhodobacteraceae *Jannaschia sp.* CCS1
Kvu-1-1	Bacteria Proteobacteria Alphaproteobacteria Rhodobacterales Rhodobacteraceae *Ketogulonicigenium vulgare* WSH-001
Kvu-2-1	Bacteria Proteobacteria Alphaproteobacteria Rhodobacterales Rhodobacteraceae *Ketogulonicigenium vulgare* Y25
Laq-1-1	Bacteria Proteobacteria Alphaproteobacteria Rhodobacterales Rhodobacteraceae *Leisingera aquimarina* DSM 24565
Lca-1-1	Bacteria Proteobacteria Alphaproteobacteria Rhodobacterales Rhodobacteraceae *Leisingera caerulea* DSM 24564
Lda-1-1	Bacteria Proteobacteria Alphaproteobacteria Rhodobacterales Rhodobacteraceae *Leisingera daeponensis* DSM 23529
Lme-1-1	Bacteria Proteobacteria Alphaproteobacteria Rhodobacterales Rhodobacteraceae *Leisingera methylohalidivorans* DSM 14336
Lsp-1-1	Bacteria Proteobacteria Alphaproteobacteria Rhodobacterales Rhodobacteraceae *Leisingera sp.* ANG-DT
Lsp-2-1	Bacteria Proteobacteria Alphaproteobacteria Rhodobacterales Rhodobacteraceae *Leisingera sp.* ANG-M1
Lsp-3-1	Bacteria Proteobacteria Alphaproteobacteria Rhodobacterales Rhodobacteraceae *Leisingera sp.* ANG-M6
Lsp-4-1	Bacteria Proteobacteria Alphaproteobacteria Rhodobacterales Rhodobacteraceae *Leisingera sp.* ANG-M7
Lsp-5-1	Bacteria Proteobacteria Alphaproteobacteria Rhodobacterales Rhodobacteraceae *Leisingera sp.* ANG-S
Lsp-6-1	Bacteria Proteobacteria Alphaproteobacteria Rhodobacterales Rhodobacteraceae *Leisingera sp.* ANG-S3
Lsp-7-1	Bacteria Proteobacteria Alphaproteobacteria Rhodobacterales Rhodobacteraceae *Leisingera sp.* ANG-S5
Lsp-8-1	Bacteria Proteobacteria Alphaproteobacteria Rhodobacterales Rhodobacteraceae *Leisingera sp.* ANG-Vp
Lsp-9-1	Bacteria Proteobacteria Alphaproteobacteria Rhodobacterales Rhodobacteraceae *Leisingera sp.* ANG1
Lci-1-1	Bacteria Proteobacteria Alphaproteobacteria Rhodobacterales Rhodobacteraceae *Loktanella cinnabarina* LL-001
Lho-1-1	Bacteria Proteobacteria Alphaproteobacteria Rhodobacterales Rhodobacteraceae *Loktanella hongkongensis* DSM 17492
Lsp-10-1	Bacteria Proteobacteria Alphaproteobacteria Rhodobacterales Rhodobacteraceae *Loktanella sp.* S4079
Lve-1-1	Bacteria Proteobacteria Alphaproteobacteria Rhodobacterales Rhodobacteraceae *Loktanella vestfoldensis* DSM 16212
Lve-2-1	Bacteria Proteobacteria Alphaproteobacteria Rhodobacterales Rhodobacteraceae *Loktanella vestfoldensis* SKA53
Mal-1-1	Bacteria Proteobacteria Alphaproteobacteria Rhodobacterales Rhodobacteraceae *Mameliella alba*
Msp-1-1	Bacteria Proteobacteria Alphaproteobacteria Rhodobacterales Rhodobacteraceae *Maribius sp.* MOLA 401
Mal-2-1	Bacteria Proteobacteria Alphaproteobacteria Rhodobacterales Rhodobacteraceae *Marinovum algicola* DG 898
Nit-1-1 to Nit-1-2	Bacteria Proteobacteria Alphaproteobacteria Rhodobacterales Rhodobacteraceae *Nautella italica*
Nsp-1-1	Bacteria Proteobacteria Alphaproteobacteria Rhodobacterales Rhodobacteraceae *Nautella sp.* ECSMB14104
Nig-1-1	Bacteria Proteobacteria Alphaproteobacteria Rhodobacterales Rhodobacteraceae *Nereida ignava*
Oin-1-1	Bacteria Proteobacteria Alphaproteobacteria Rhodobacterales Rhodobacteraceae *Oceanibulbus indolifex* HEL-45
Oba-1-1	Bacteria Proteobacteria Alphaproteobacteria Rhodobacterales Rhodobacteraceae *Oceanicola batsensis* HTCC2597
Ogr-1-1	Bacteria Proteobacteria Alphaproteobacteria Rhodobacterales Rhodobacteraceae *Oceanicola granulosus* HTCC2516
Osp-1-1	Bacteria Proteobacteria Alphaproteobacteria Rhodobacterales Rhodobacteraceae *Oceanicola sp.* HL-35
Osp-2-1	Bacteria Proteobacteria Alphaproteobacteria Rhodobacterales Rhodobacteraceae *Oceanicola sp.* MCTG156(1a)
Osp-3-1	Bacteria Proteobacteria Alphaproteobacteria Rhodobacterales Rhodobacteraceae *Oceanicola sp.* S124
Ogu-1-1	Bacteria Proteobacteria Alphaproteobacteria Rhodobacterales Rhodobacteraceae *Oceaniovalibus guishaninsula* JLT2003
Oan-1-1	Bacteria Proteobacteria Alphaproteobacteria Rhodobacterales Rhodobacteraceae *Octadecabacter antarcticus* 307
Oar-1-1	Bacteria Proteobacteria Alphaproteobacteria Rhodobacterales Rhodobacteraceae *Octadecabacter arcticus* 238
Ote-1-1	Bacteria Proteobacteria Alphaproteobacteria Rhodobacterales Rhodobacteraceae *Octadecabacter temperatus*
Pde-1-1	Bacteria Proteobacteria Alphaproteobacteria Rhodobacterales Rhodobacteraceae *Paracoccus denitrificans* PD1222
Pga-1-1	Bacteria Proteobacteria Alphaproteobacteria Rhodobacterales Rhodobacteraceae *Phaeobacter gallaeciensis* 2.10
Pga-2-1	Bacteria Proteobacteria Alphaproteobacteria Rhodobacterales Rhodobacteraceae *Phaeobacter gallaeciensis* DSM 17395
Pga-3-1	Bacteria Proteobacteria Alphaproteobacteria Rhodobacterales Rhodobacteraceae *Phaeobacter gallaeciensis* DSM 26640
Pin-1-1	Bacteria Proteobacteria Alphaproteobacteria Rhodobacterales Rhodobacteraceae *Phaeobacter inhibens* DSM 16374
Psp-1-1	Bacteria Proteobacteria Alphaproteobacteria Rhodobacterales Rhodobacteraceae *Phaeobacter sp.* S26
Psp-2-1	Bacteria Proteobacteria Alphaproteobacteria Rhodobacterales Rhodobacteraceae *Phaeobacter sp.* S60
Pte-1-1	Bacteria Proteobacteria Alphaproteobacteria Rhodobacterales Rhodobacteraceae *Planktomarina temperata* RCA23
Pxi-1-1	Bacteria Proteobacteria Alphaproteobacteria Rhodobacterales Rhodobacteraceae *Pseudodonghicola xiamenensis* DSM 18339
Pat-1-1	Bacteria Proteobacteria Alphaproteobacteria Rhodobacterales Rhodobacteraceae *Pseudooceanicola atlanticus*
Pna-1-1	Bacteria Proteobacteria Alphaproteobacteria Rhodobacterales Rhodobacteraceae *Pseudooceanicola nanhaiensis* DSM 18065
Par-1-1	Bacteria Proteobacteria Alphaproteobacteria Rhodobacterales Rhodobacteraceae *Pseudophaeobacter arcticus* DSM 23566
Pan-1-1	Bacteria Proteobacteria Alphaproteobacteria Rhodobacterales Rhodobacteraceae *Pseudorhodobacter antarcticus*
Paq-1-1	Bacteria Proteobacteria Alphaproteobacteria Rhodobacterales Rhodobacteraceae *Pseudorhodobacter aquimaris*
Pfe-1-1	Bacteria Proteobacteria Alphaproteobacteria Rhodobacterales Rhodobacteraceae *Pseudorhodobacter ferrugineus*
Pfe-2-1	Bacteria Proteobacteria Alphaproteobacteria Rhodobacterales Rhodobacteraceae *Pseudorhodobacter ferrugineus* DSM 5888
Pwa-1-1	Bacteria Proteobacteria Alphaproteobacteria Rhodobacterales Rhodobacteraceae *Pseudorhodobacter wandonensis*

Psp-3-1	Bacteria Proteobacteria Alphaproteobacteria Rhodobacterales Rhodobacteraceae *Puniceibacterium sp.* IMCC21224
CRh-1-1	Bacteria Proteobacteria Alphaproteobacteria Rhodobacterales Rhodobacteraceae Rhodobacter *Candidatus* Rhodobacter lobularis
Rca-1-1	Bacteria Proteobacteria Alphaproteobacteria Rhodobacterales Rhodobacteraceae *Rhodobacter capsulatus* B6
Rca-2-1	Bacteria Proteobacteria Alphaproteobacteria Rhodobacterales Rhodobacteraceae *Rhodobacter capsulatus* DE442
Rca-3-1	Bacteria Proteobacteria Alphaproteobacteria Rhodobacterales Rhodobacteraceae *Rhodobacter capsulatus* R121
Rca-4-1	Bacteria Proteobacteria Alphaproteobacteria Rhodobacterales Rhodobacteraceae *Rhodobacter capsulatus* SB 1003
Rca-5-1	Bacteria Proteobacteria Alphaproteobacteria Rhodobacterales Rhodobacteraceae *Rhodobacter capsulatus* Y262
Rca-6-1	Bacteria Proteobacteria Alphaproteobacteria Rhodobacterales Rhodobacteraceae *Rhodobacter capsulatus* YW1
Rca-7-1	Bacteria Proteobacteria Alphaproteobacteria Rhodobacterales Rhodobacteraceae *Rhodobacter capsulatus* YW2
Rsp-1-1	Bacteria Proteobacteria Alphaproteobacteria Rhodobacterales Rhodobacteraceae *Rhodobacter sp.* AKP1
Rsp-2-1	Bacteria Proteobacteria Alphaproteobacteria Rhodobacterales Rhodobacteraceae *Rhodobacter sp.* SW2
Rsp-3-1	Bacteria Proteobacteria Alphaproteobacteria Rhodobacterales Rhodobacteraceae *Rhodobacter sphaeroides* 2.4.1
Rsp-4-1	Bacteria Proteobacteria Alphaproteobacteria Rhodobacterales Rhodobacteraceae *Rhodobacter sphaeroides* ATCC 17025
Rsp-5-1	Bacteria Proteobacteria Alphaproteobacteria Rhodobacterales Rhodobacteraceae *Rhodobacter sphaeroides* ATCC 17029
Rsp-6-1	Bacteria Proteobacteria Alphaproteobacteria Rhodobacterales Rhodobacteraceae *Rhodobacter sphaeroides* KD131
Rsp-7-1	Bacteria Proteobacteria Alphaproteobacteria Rhodobacterales Rhodobacteraceae *Rhodobacter sphaeroides* WS8N
Rba-1-1	Bacteria Proteobacteria Alphaproteobacteria Rhodobacterales *Rhodobacteraceae bacterium* HIMB11
Rba-2-1	Bacteria Proteobacteria Alphaproteobacteria Rhodobacterales *Rhodobacteraceae bacterium* KLH11
Rba-3-1	Bacteria Proteobacteria Alphaproteobacteria Rhodobacterales *Rhodobacteraceae bacterium* PD-2
Rel-1-1	Bacteria Proteobacteria Alphaproteobacteria Rhodobacterales Rhodobacteraceae *Roseibacterium elongatum* DSM 19469
Rat-1-1	Bacteria Proteobacteria Alphaproteobacteria Rhodobacterales Rhodobacteraceae *Roseivivax atlanticus*
Rha-1-1	Bacteria Proteobacteria Alphaproteobacteria Rhodobacterales Rhodobacteraceae *Roseivivax halodurans* JCM 10272
Ris-1-1	Bacteria Proteobacteria Alphaproteobacteria Rhodobacterales Rhodobacteraceae *Roseivivax isoporae* LMG 25204
Rde-1-1	Bacteria Proteobacteria Alphaproteobacteria Rhodobacterales Rhodobacteraceae *Roseobacter denitrificans* OCh 114
Rli-1-1	Bacteria Proteobacteria Alphaproteobacteria Rhodobacterales Rhodobacteraceae *Roseobacter litoralis* Och 149
Rsp-8-1	Bacteria Proteobacteria Alphaproteobacteria Rhodobacterales Rhodobacteraceae *Roseobacter sp.* AzwK-3b
Rsp-9-1	Bacteria Proteobacteria Alphaproteobacteria Rhodobacterales Rhodobacteraceae *Roseobacter sp.* CCS2
Rsp-10-1	Bacteria Proteobacteria Alphaproteobacteria Rhodobacterales Rhodobacteraceae *Roseobacter sp.* GAI101
Rsp-11-1	Bacteria Proteobacteria Alphaproteobacteria Rhodobacterales Rhodobacteraceae *Roseobacter sp.* MED193
Rsp-12-1	Bacteria Proteobacteria Alphaproteobacteria Rhodobacterales Rhodobacteraceae *Roseobacter sp.* SK209-2-6
Rmu-1-1	Bacteria Proteobacteria Alphaproteobacteria Rhodobacterales Rhodobacteraceae *Roseovarius mucosus* DSM 17069
Rnu-1-1	Bacteria Proteobacteria Alphaproteobacteria Rhodobacterales Rhodobacteraceae *Roseovarius nubinhibens* ISM
Rsp-13-1	Bacteria Proteobacteria Alphaproteobacteria Rhodobacterales Rhodobacteraceae *Roseovarius sp.* 217
Rsp-14-1	Bacteria Proteobacteria Alphaproteobacteria Rhodobacterales Rhodobacteraceae *Roseovarius sp.* HTCC2601
Rsp-15-1	Bacteria Proteobacteria Alphaproteobacteria Rhodobacterales Rhodobacteraceae *Roseovarius sp.* MCTG156(2b)
Rsp-16-1	Bacteria Proteobacteria Alphaproteobacteria Rhodobacterales Rhodobacteraceae *Roseovarius sp.* TM1035
Rto-1-1	Bacteria Proteobacteria Alphaproteobacteria Rhodobacterales Rhodobacteraceae *Roseovarius tolerans*
Rme-1-1	Bacteria Proteobacteria Alphaproteobacteria Rhodobacterales Rhodobacteraceae *Rubellimicrobium mesophilum* DSM 19309
Rha-2-1	Bacteria Proteobacteria Alphaproteobacteria Rhodobacterales Rhodobacteraceae *Ruegeria halocynthiae*
Rmo-1-1	Bacteria Proteobacteria Alphaproteobacteria Rhodobacterales Rhodobacteraceae *Ruegeria mobilis*
Rmo-2-1	Bacteria Proteobacteria Alphaproteobacteria Rhodobacterales Rhodobacteraceae *Ruegeria mobilis* F1926
Rpo-1-1	Bacteria Proteobacteria Alphaproteobacteria Rhodobacterales Rhodobacteraceae *Ruegeria pomeroyi* DSS-3
Rsp-17-1	Bacteria Proteobacteria Alphaproteobacteria Rhodobacterales Rhodobacteraceae *Ruegeria sp.* 6PALISEP08
Rsp-18-1	Bacteria Proteobacteria Alphaproteobacteria Rhodobacterales Rhodobacteraceae *Ruegeria sp.* ANG-R
Rsp-19-1	Bacteria Proteobacteria Alphaproteobacteria Rhodobacterales Rhodobacteraceae *Ruegeria sp.* ANG-S4
Rsp-20-1	Bacteria Proteobacteria Alphaproteobacteria Rhodobacterales Rhodobacteraceae *Ruegeria sp.* R11
Rsp-21-1	Bacteria Proteobacteria Alphaproteobacteria Rhodobacterales Rhodobacteraceae *Ruegeria sp.* TM1040
Rsp-22-1	Bacteria Proteobacteria Alphaproteobacteria Rhodobacterales Rhodobacteraceae *Ruegeria sp.* TW15
Sla-1-1	Bacteria Proteobacteria Alphaproteobacteria Rhodobacterales Rhodobacteraceae Ruegeria *Silicibacter lacuscaerulensis* ITI-1157
Ssp-1-1	Bacteria Proteobacteria Alphaproteobacteria Rhodobacterales Rhodobacteraceae Ruegeria *Silicibacter sp.* TrichCH4B
Sst-1-1	Bacteria Proteobacteria Alphaproteobacteria Rhodobacterales Rhodobacteraceae *Sagittula stellata* E-37
Smu-1-1	Bacteria Proteobacteria Alphaproteobacteria Rhodobacterales Rhodobacteraceae *Salipiger mucosus* DSM 16094
Sna-1-1	Bacteria Proteobacteria Alphaproteobacteria Rhodobacterales Rhodobacteraceae *Sedimentitalea nanhaiensis* DSM 24252
Sqi-1-1	Bacteria Proteobacteria Alphaproteobacteria Rhodobacterales Rhodobacteraceae *Sediminimonas qiaohouensis* DSM 21189
Sdo-1-1 to Sdo-1-2	Bacteria Proteobacteria Alphaproteobacteria Rhodobacterales Rhodobacteraceae *Sulfitobacter donghicola* DSW-25 = KCTC 12864 = JCM 14565
Sge-1-1	Bacteria Proteobacteria Alphaproteobacteria Rhodobacterales Rhodobacteraceae *Sulfitobacter geojensis*
Sgu-1-1	Bacteria Proteobacteria Alphaproteobacteria Rhodobacterales Rhodobacteraceae *Sulfitobacter guttiformis* KCTC 32187
Sme-1-1	Bacteria Proteobacteria Alphaproteobacteria Rhodobacterales Rhodobacteraceae *Sulfitobacter mediterraneus*
Sme-2-1	Bacteria Proteobacteria Alphaproteobacteria Rhodobacterales Rhodobacteraceae *Sulfitobacter mediterraneus* KCTC 32188
Sno-1-1	Bacteria Proteobacteria Alphaproteobacteria Rhodobacterales Rhodobacteraceae *Sulfitobacter noctilucae*
Sno-2-1	Bacteria Proteobacteria Alphaproteobacteria Rhodobacterales Rhodobacteraceae *Sulfitobacter noctilucicola*
Spo-1-1	Bacteria Proteobacteria Alphaproteobacteria Rhodobacterales Rhodobacteraceae *Sulfitobacter pontiacus* 3SOLIMAR09
Sps-1-1	Bacteria Proteobacteria Alphaproteobacteria Rhodobacterales Rhodobacteraceae *Sulfitobacter pseudonitzschiae*
Ssp-2-1	Bacteria Proteobacteria Alphaproteobacteria Rhodobacterales Rhodobacteraceae *Sulfitobacter sp.* 20_GPM-1509m
Ssp-3-1	Bacteria Proteobacteria Alphaproteobacteria Rhodobacterales Rhodobacteraceae *Sulfitobacter sp.* CB2047

Ssp-4-1	Bacteria Proteobacteria Alphaproteobacteria Rhodobacterales Rhodobacteraceae *Sulfitobacter sp.* EE-36
Ssp-5-1	Bacteria Proteobacteria Alphaproteobacteria Rhodobacterales Rhodobacteraceae *Sulfitobacter sp.* NAS-14.1
Tsp-1-1	Bacteria Proteobacteria Alphaproteobacteria Rhodobacterales Rhodobacteraceae *Tateyamaria sp.* ANG-S1
Tar-1-1	Bacteria Proteobacteria Alphaproteobacteria Rhodobacterales Rhodobacteraceae *Thalassobacter arenae* DSM 19593
Tsp-2-1	Bacteria Proteobacteria Alphaproteobacteria Rhodobacterales Rhodobacteraceae *Thalassobacter sp.* 16PALIMAR09
Tst-1-1	Bacteria Proteobacteria Alphaproteobacteria Rhodobacterales Rhodobacteraceae *Thalassobacter stenotrophicus*
Tsp-3-1	Bacteria Proteobacteria Alphaproteobacteria Rhodobacterales Rhodobacteraceae Thalassobium *Thalassiobium sp.* R2A62
Tat-1-1	Bacteria Proteobacteria Alphaproteobacteria Rhodobacterales Rhodobacteraceae *Thioclava atlantica*
Tda-1-1	Bacteria Proteobacteria Alphaproteobacteria Rhodobacterales Rhodobacteraceae *Thioclava dalianensis*
Tin-1-1	Bacteria Proteobacteria Alphaproteobacteria Rhodobacterales Rhodobacteraceae *Thioclava indica*
Tpa-1-1	Bacteria Proteobacteria Alphaproteobacteria Rhodobacterales Rhodobacteraceae *Thioclava pacifica* DSM 10166
Wma-1-1	Bacteria Proteobacteria Alphaproteobacteria Rhodobacterales Rhodobacteraceae *Wenxinia marina* DSM 24838
Rba-4-1	Bacteria Proteobacteria Alphaproteobacteria *Rhodobacterales bacterium* HTCC2150
Rba-5-1	Bacteria Proteobacteria Alphaproteobacteria *Rhodobacterales bacterium* HTCC2255
Rba-6-1	Bacteria Proteobacteria Alphaproteobacteria *Rhodobacterales bacterium* HTCC2654
Rba-7-1	Bacteria Proteobacteria Alphaproteobacteria *Rhodobacterales bacterium* Y4I
env-1 to env-656	environmental samples

1.2 Gene contexts

Each L2-Alphaproteobacteria RNA (indicated by "RNA→") is listed. For each hit, the downstream genes predicted to reside in a regulated operon are listed. If the nearest downstream gene is encoding in the opposite strand (and therefore presumed to not be a part of a regulated operon), then that gene is still depicted. Some environmental sequences and some RefSeq entries lack gene annotations, and so no genes are available for such sequences. The direction of each gene is indicated with an arrow (→), and each predicted conserved domain in the gene is named. Conserved domains associated with more than one L2-Alphaproteobacteria RNA are assigned a color; other domains are gray. Information about these conserved domains is given in Section 1.3. The accession of the sequence containing each L2-Alphaproteobacteria RNA is given in the column named "Seq. accession". Accessions beginning with "NC_", "NS_", "NW_" or "NZ_" are contained in RefSeq. Other accession refer to environmental samples. Nucleotide coordinates are given for the 5′ and 3′ boundaries of each L2-Alphaproteobacteria RNA. If the 5′ coordinate is greater than the 3′ coordinate, the RNA is present on the reverse-complement strand of the containing genomic DNA sequence. Each hit is denoted by an abbreviation (like "Eco-1-1") that refers to a taxonomy given in Section 1.1.

**abbrev.**	**Seq. accession**		**5′ at**	**3′ at**	**genes**
env-1	SL_3KL_010_SED_10464608	+	263	326	**RNA**→ RplB (COG0090)rplB (PRK09374)→
Dsh-1-1	NC_009952.1	+	285364	285406	**RNA**→ RplB (COG0090)rplB (PRK09374)→ rpsS (PRK00357)RpsS (COG0185)→ rplV (PRK00565)Ribosomal_L22 (pfam00237)→ RpsC (COG0092)rpsC (PRK00310)→ RplP (COG0197)rplP (PRK09203)→
Pde-1-1	NC_008686.1	+	746785	746857	**RNA**→ RplB (COG0090)rplB (PRK09374)→ rpsS (PRK00357)RpsS (COG0185)→ rplV (PRK00565)Ribosomal_L22 (pfam00237)→ RpsC (COG0092)rpsC (PRK00310)→ RplP (COG0197)rplP (PRK09203)→
env-2	CEVH01071484.1	-	2499	2437	**RNA**→ RplB (COG0090)rplB (PRK09374)→ rpsS (PRK00357)RpsS (COG0185)→ rplV (PRK00565)Ribosomal_L22 (pfam00237)→ RpsC (COG0092)rpsC (PRK00310)→ rplP (PRK09203)Ribosomal_L16 (pfam00252)→
Aat-2-1	NZ_AQQZ01000010.1	+	3052	3115	**RNA**→ RplB (COG0090)rplB (PRK09374)→ rpsS (PRK00357)RpsS (COG0185)→ rplV (PRK00565)Ribosomal_L22 (pfam00237)→ RpsC (COG0092)rpsC (PRK00310)→ RplP (COG0197)rplP (PRK09203)→
Rsp-2-1	NZ_ACYY01000029.1	-	40278	40238	**RNA**→ RplB (COG0090)rplB (PRK09374)→ RplB (COG0090)rplB (PRK09374)→ rpsS (PRK00357)RpsS (COG0185)→ rpsS (PRK00357)RpsS (COG0185)→ rplV (PRK00565)Ribosomal_L22 (pfam00237)→ rplV (PRK00565)Ribosomal_L22 (pfam00237)→ RpsC (COG0092)rpsC (PRK00310)→ RpsC (COG0092)rpsC (PRK00310)→ RplP (COG0197)rplP (PRK09203)→ RplP (COG0197)rplP (PRK09203)→
env-3	JGI20154J14316_10014726	+	1330	1394	**RNA**→ RplB (COG0090)rplB (PRK09374)→ rpsS (PRK00357)RpsS (COG0185)→ rplV (PRK00565)Ribosomal_L22 (pfam00237)→ RpsC (COG0092)rpsC (PRK00310)→ RplP (COG0197)rplP (PRK09203)→ GIY-YIG_SF (cd00719)GIYc (smart00465)→

Amu-1-1	NZ_JFKE01000006.1	-	218957	218896	**RNA**→ RplB (COG0090)rplB (PRK09374)→ rpsS (PRK00357)RpsS (COG0185)→ rplV (PRK00565)Ribosomal_L22 (pfam00237)→ RpsC (COG0092)rpsC (PRK00310)→ RplP (COG0197)rplP (PRK09203)→
env-4	AglaG_F499EG101CWTC2	-	345	276	**RNA**→ rplB (PRK09374)Ribosomal_L2 (pfam00181)→
env-5	AglaG_contig17173	-	368	299	**RNA**→ rplB (PRK09374)Ribosomal_L2 (pfam00181)→
Rat-1-1	NZ_AQQW01000010.1	+	2615	2692	**RNA**→ RplB (COG0090)rplB (PRK09374)→ rpsS (PRK00357)RpsS (COG0185)→ rplV (PRK00565)Ribosomal_L22 (pfam00237)→ RpsC (COG0092)rpsC (PRK00310)→ RplP (COG0197)rplP (PRK09203)→ hypo→ 2OG-FeII_Oxy_5 (pfam13759)→
env-6	JGI24724J26744_10003266	-	8748	8659	**RNA**→ RplB (COG0090)rplB (PRK09374)→ rpsS (PRK00357)RpsS (COG0185)→ rplV (PRK00565)Ribosomal_L22 (pfam00237)→ RpsC (COG0092)rpsC (PRK00310)→ RplP (COG0197)rplP (PRK09203)→
env-7	JGI24025J20009_10035938	-	1770	1681	**RNA**→ RplB (COG0090)rplB (PRK09374)→ rpsS (PRK00357)RpsS (COG0185)→ rplV (PRK00565)Ribosomal_L22 (pfam00237)→ rpsC (PRK00310)30S_S3_KH (cd02412)→
env-8	GX8B9KJ01CH3VN	-	334	271	**RNA**→ rplB (PRK09374)Ribosomal_L2 (pfam00181)→
env-9	JGI12109J20283_12362431	+	37	102	**RNA**→
Tst-1-1	NZ_JGVS01000007.1	+	4252	4317	**RNA**→ RplB (COG0090)rplB (PRK09374)→ rpsS (PRK00357)RpsS (COG0185)→ rplV (PRK00565)Ribosomal_L22 (pfam00237)→ RpsC (COG0092)rpsC (PRK00310)→ RplP (COG0197)rplP (PRK09203)→
Tsp-2-1	NZ_JHAK01000011.1	-	52173	52108	**RNA**→ RplB (COG0090)rplB (PRK09374)→ rpsS (PRK00357)RpsS (COG0185)→ rplV (PRK00565)Ribosomal_L22 (pfam00237)→ RpsC (COG0092)rpsC (PRK00310)→ RplP (COG0197)rplP (PRK09203)→
env-10	P_2C_Liq_1_UnCty_1004676	-	3400	3335	**RNA**→ RplB (COG0090)rplB (PRK09374)→ rpsS (PRK00357)RpsS (COG0185)→ rplV (PRK00565)Ribosomal_L22 (pfam00237)→ RpsC (COG0092)rpsC (PRK00310)→ RplP (COG0197)rplP (PRK09203)→
env-11	P_2C_Liq_2_UnCty_1000207	-	15707	15642	**RNA**→ RplB (COG0090)rplB (PRK09374)→ rpsS (PRK00357)RpsS (COG0185)→ rplV (PRK00565)Ribosomal_L22 (pfam00237)→ RpsC (COG0092)rpsC (PRK00310)→ RplP (COG0197)rplP (PRK09203)→
env-12	P_2C_Liq_2_UnCty_1009999	+	1288	1353	**RNA**→ RplB (COG0090)rplB (PRK09374)→
env-13	ATLU01000110.1	+	1285	1375	**RNA**→ RplB (COG0090)rplB (PRK09374)→ rpsS (PRK00357)RpsS (COG0185)→ rplV (PRK00565)Ribosomal_L22 (pfam00237)→ RpsC (COG0092)rpsC (PRK00310)→ RplP (COG0197)rplP (PRK09203)→
Rca-7-1	NZ_AYPZ01000027.1	+	120952	121013	**RNA**→ RplB (COG0090)rplB (PRK09374)→ rpsS (PRK00357)RpsS (COG0185)→ rplV (PRK00565)Ribosomal_L22 (pfam00237)→ RpsC (COG0092)rpsC (PRK00310)→ RplP (COG0197)rplP (PRK09203)→
Rca-4-1	NC_014034.1	+	342742	342803	**RNA**→ RplB (COG0090)rplB (PRK09374)→ rpsS (PRK00357)RpsS (COG0185)→ rplV (PRK00565)Ribosomal_L22 (pfam00237)→ RpsC (COG0092)rpsC (PRK00310)→ RplP (COG0197)rplP (PRK09203)→
Rca-3-1	NZ_AYQC01000005.1	+	120809	120870	**RNA**→ RplB (COG0090)rplB (PRK09374)→ rpsS (PRK00357)RpsS (COG0185)→ rplV (PRK00565)Ribosomal_L22 (pfam00237)→ RpsC (COG0092)rpsC (PRK00310)→ RplP (COG0197)rplP (PRK09203)→
Rca-5-1	NZ_AYQB01000006.1	+	120842	120903	**RNA**→ RplB (COG0090)rplB (PRK09374)→ rpsS (PRK00357)RpsS (COG0185)→ rplV (PRK00565)Ribosomal_L22 (pfam00237)→ RpsC (COG0092)rpsC (PRK00310)→ RplP (COG0197)rplP (PRK09203)→
Rca-2-1	NZ_AYPR01000005.1	+	120776	120837	**RNA**→ RplB (COG0090)rplB (PRK09374)→ rpsS (PRK00357)RpsS (COG0185)→ rplV (PRK00565)Ribosomal_L22 (pfam00237)→ RpsC (COG0092)rpsC (PRK00310)→ RplP (COG0197)rplP (PRK09203)→
Rca-1-1	NZ_AYQA01000038.1	+	120869	120930	**RNA**→ RplB (COG0090)rplB (PRK09374)→ rpsS (PRK00357)RpsS (COG0185)→ rplV (PRK00565)Ribosomal_L22 (pfam00237)→ RpsC (COG0092)rpsC (PRK00310)→ RplP (COG0197)rplP (PRK09203)→
Rsp-4-1	NC_009428.1	-	2571487	2571448	**RNA**→ RplB (COG0090)rplB (PRK09374)→ rpsS (PRK00357)RpsS (COG0185)→ rplV (PRK00565)Ribosomal_L22 (pfam00237)→ RpsC (COG0092)rpsC (PRK00310)→ RplP (COG0197)rplP (PRK09203)→
Rsp-5-1	NC_009049.1	+	387361	387400	**RNA**→ RplB (COG0090)rplB (PRK09374)→ rpsS (PRK00357)RpsS (COG0185)→ rplV (PRK00565)Ribosomal_L22 (pfam00237)→ RpsC (COG0092)rpsC (PRK00310)→ RplP (COG0197)rplP (PRK09203)→

Rsp-6-1	NC_011963.1	+	19443	19482	**RNA**→ RplB (COG0090)rplB (PRK09374)→ rpsS (PRK00357)RpsS (COG0185)→ rplV (PRK00565)Ribosomal_L22 (pfam00237)→ RpsC (COG0092)rpsC (PRK00310)→ RplP (COG0197)rplP (PRK09203)→
Rsp-7-1	NZ_CM001161.1	+	2879562	2879601	**RNA**→ RplB (COG0090)rplB (PRK09374)→ rpsS (PRK00357)RpsS (COG0185)→ rplV (PRK00565)Ribosomal_L22 (pfam00237)→ RpsC (COG0092)rpsC (PRK00310)→ RplP (COG0197)rplP (PRK09203)→
Rsp-3-1	NC_007493.2	+	309766	309805	**RNA**→ RplB (COG0090)rplB (PRK09374)→ rpsS (PRK00357)RpsS (COG0185)→ rplV (PRK00565)Ribosomal_L22 (pfam00237)→ RpsC (COG0092)rpsC (PRK00310)→ RplP (COG0197)rplP (PRK09203)→
env-14	JQGR01000094.1	+	4628	4725	**RNA**→ RplB (COG0090)rplB (PRK09374)→ rpsS (PRK00357)RpsS (COG0185)→ rplV (PRK00565)Ribosomal_L22 (pfam00237)→ RpsC (COG0092)rpsC (PRK00310)→ RplP (COG0197)rplP (PRK09203)→
env-15	4455295.3.316264	+	69	130	**RNA**→ RplB (COG0090)rplB (PRK09374)→
env-16	AYRF01032015.1	+	99	189	**RNA**→ ←hypo
env-17	3300000052_005679	+	305	368	**RNA**→ RplB (COG0090)rplB (PRK09374)→ rpsS (PRK00357)RpsS (COG0185)→ rplV (PRK00565)Ribosomal_L22 (pfam00237)→ RpsC (COG0092)rpsC (PRK00310)→ rplP (PRK09203)Ribosomal_L16 (pfam00252)→
Sqi-1-1	NZ_AUIJ01000004.1	-	73400	73337	**RNA**→ RplB (COG0090)rplB (PRK09374)→ rpsS (PRK00357)RpsS (COG0185)→ rplV (PRK00565)Ribosomal_L22 (pfam00237)→ RpsC (COG0092)rpsC (PRK00310)→ RplP (COG0197)rplP (PRK09203)→ PRK00306 (PRK00306)Ribosomal_L29 (pfam00831)→ RpsQ (COG0186)rpsQ (PRK05610)→ rplN (PRK05483)Ribosomal_L14 (pfam00238)→ RplX (COG0198)rplX (PRK00004)→ rplE (PRK00010)RplE (COG0094)→ rpsN (PRK08881)Ribosomal_S14 (pfam00253)→ rpsH (PRK00136)Ribosomal_S8 (pfam00410)→ RplF (COG0097)rplF (PRK05498)→ RplR (COG0256)rplR (PRK05593)→ RpsE (COG0098)rpsE (PRK00550)→ Ribosomal_L30 (cd01658)rpmD (PRK05611)→
env-18	AYRH01014365.1	-	242	152	**RNA**→ RplB (COG0090)rplB (PRK09374)→
env-19	JCVI_READ_1105499881630	-	824	763	**RNA**→ RplB (COG0090)rplB (PRK09374)→
env-20	JCVI_SCAF_1101668060777	+	269	332	**RNA**→ RplB (COG0090)rplB (PRK09374)→ rpsS (PRK00357)RpsS (COG0185)→
Hsp-1-1	NZ_JWIE01000061.1	+	80663	80737	**RNA**→ RplB (COG0090)rplB (PRK09374)→ rpsS (PRK00357)RpsS (COG0185)→ rplV (PRK00565)Ribosomal_L22 (pfam00237)→ RpsC (COG0092)rpsC (PRK00310)→ RplP (COG0197)rplP (PRK09203)→
Hna-1-1	NZ_JWIF01000023.1	-	41827	41752	**RNA**→ RplB (COG0090)rplB (PRK09374)→ rpsS (PRK00357)RpsS (COG0185)→ rplV (PRK00565)Ribosomal_L22 (pfam00237)→ RpsC (COG0092)rpsC (PRK00310)→ RplP (COG0197)rplP (PRK09203)→
Rba-6-1	NZ_CH902578.1	-	2097076	2096975	**RNA**→ RplB (COG0090)rplB (PRK09374)→ rpsS (PRK00357)RpsS (COG0185)→ rplV (PRK00565)Ribosomal_L22 (pfam00237)→ RpsC (COG0092)rpsC (PRK00310)→ RplP (COG0197)rplP (PRK09203)→
env-21	CENL01121776.1	+	1754	1825	**RNA**→ RplB (COG0090)rplB (PRK09374)→ rpsS (PRK00357)RpsS (COG0185)→ rplV (PRK00565)Ribosomal_L22 (pfam00237)→ RpsC (COG0092)rpsC (PRK00310)→ RplP (COG0197)rplP (PRK09203)→
Cba-1-1	NZ_AMRK01000018.1	+	2718	2789	**RNA**→ RplB (COG0090)rplB (PRK09374)→ RplB (COG0090)rplB (PRK09374)→ rpsS (PRK00357)RpsS (COG0185)→ rpsS (PRK00357)RpsS (COG0185)→ rplV (PRK00565)Ribosomal_L22 (pfam00237)→ rplV (PRK00565)Ribosomal_L22 (pfam00237)→ RpsC (COG0092)rpsC (PRK00310)→ RpsC (COG0092)rpsC (PRK00310)→ RplP (COG0197)rplP (PRK09203)→ RplP (COG0197)rplP (PRK09203)→
Rsp-1-1	NZ_ANFS01000015.1	+	2126	2188	**RNA**→ RplB (COG0090)rplB (PRK09374)→ rpsS (PRK00357)RpsS (COG0185)→ rplV (PRK00565)Ribosomal_L22 (pfam00237)→ RpsC (COG0092)rpsC (PRK00310)→ RplP (COG0197)rplP (PRK09203)→
env-22	JGI24724J26744_10214823	+	124	189	**RNA**→ rplB (PRK09374)Ribosomal_L2 (pfam00181)→
env-23	NCBI_READ_1112637934486	+	240	306	**RNA**→ RplB (COG0090)rplB (PRK09374)→
env-24	CESE01117110.1	-	1184	1118	**RNA**→ RplB (COG0090)rplB (PRK09374)→ rpsS (PRK00357)RpsS (COG0185)→
env-25	CEST01016851.1	+	2161	2227	**RNA**→ RplB (COG0090)rplB (PRK09374)→ rpsS (PRK00357)RpsS (COG0185)→
env-26	2236876009_187088	+	204	270	**RNA**→ RplB (COG0090)rplB (PRK09374)→
env-27	DelMOSum2010_c10002940	-	12329	12263	**RNA**→ RplB (COG0090)rplB (PRK09374)→ rpsS (PRK00357)RpsS (COG0185)→ rplV (PRK00565)Ribosomal_L22 (pfam00237)→ RpsC (COG0092)rpsC (PRK00310)→ RplP (COG0197)rplP (PRK09203)→

env-28	JGI20152J14361_10100075	-	149	83	**RNA**→ RplB (COG0090)rplB (PRK09374)→
env-29	JGI20151J14362_10028856	+	2610	2676	**RNA**→ RplB (COG0090)rplB (PRK09374)→
env-30	JGI20154J14316_10002806	+	4976	5042	**RNA**→ RplB (COG0090)rplB (PRK09374)→ rpsS (PRK00357)RpsS (COG0185)→<br>rplV (PRK00565)Ribosomal_L22 (pfam00237)→ RpsC (COG0092)rpsC (PRK00310)→<br>RplP (COG0197)rplP (PRK09203)→
env-31	JGI20160J14292_10200265	+	405	471	**RNA**→ RplB (COG0090)rplB (PRK09374)→
env-32	JGI20153J14295_10377309	-	117	51	**RNA**→ hypo→
env-33	JGI20153J14318_10377309	-	117	51	**RNA**→ hypo→
env-34	JGI20157J14317_10000107	-	35008	34942	**RNA**→ RplB (COG0090)rplB (PRK09374)→ rpsS (PRK00357)RpsS (COG0185)→<br>rplV (PRK00565)Ribosomal_L22 (pfam00237)→ RpsC (COG0092)rpsC (PRK00310)→<br>RplP (COG0197)rplP (PRK09203)→
env-35	CEUP01061385.1	-	473	377	**RNA**→ rplB (PRK09374)Ribosomal_L2 (pfam00181)→
Csp-1-1	NZ_AJKJ01000048.1	+	3267	3349	**RNA**→ RplB (COG0090)rplB (PRK09374)→ RplB (COG0090)rplB (PRK09374)→<br>rpsS (PRK00357)RpsS (COG0185)→ rpsS (PRK00357)RpsS (COG0185)→<br>rplV (PRK00565)Ribosomal_L22 (pfam00237)→ rplV (PRK00565)Ribosomal_L22 (pfam00237)→<br>RpsC (COG0092)rpsC (PRK00310)→ RpsC (COG0092)rpsC (PRK00310)→ RplP (COG0197)rplP (PRK09203)→<br>RplP (COG0197)rplP (PRK09203)→ hypo→ hypo→
Pat-1-1	NZ_AQQX01000010.1	-	63842	63774	**RNA**→ RplB (COG0090)rplB (PRK09374)→ rpsS (PRK00357)RpsS (COG0185)→<br>rplV (PRK00565)Ribosomal_L22 (pfam00237)→ RpsC (COG0092)rpsC (PRK00310)→<br>RplP (COG0197)rplP (PRK09203)→
Rba-3-1	NZ_KN714899.1	-	63316	63257	**RNA**→ RplB (COG0090)rplB (PRK09374)→ rpsS (PRK00357)RpsS (COG0185)→<br>rplV (PRK00565)Ribosomal_L22 (pfam00237)→ RpsC (COG0092)rpsC (PRK00310)→<br>RplP (COG0197)rplP (PRK09203)→
env-36	JGI24025J20009_10174089	+	339	403	**RNA**→ RplB (COG0090)rplB (PRK09374)→
env-37	CEQG01114380.1	-	89	29	**RNA**→
Rca-6-1	NZ_AYPY01000003.1	-	128998	128937	**RNA**→ RplB (COG0090)rplB (PRK09374)→ rpsS (PRK00357)RpsS (COG0185)→<br>rplV (PRK00565)Ribosomal_L22 (pfam00237)→ RpsC (COG0092)rpsC (PRK00310)→<br>RplP (COG0197)rplP (PRK09203)→
env-38	JGI24723J26617_10335773	-	212	147	**RNA**→ RplB (COG0090)rplB (PRK09374)→
env-39	JGI24724J26744_10397626	+	94	159	**RNA**→ rplB (PRK09374)Ribosomal_L2 (pfam00181)→
env-40	JGI24723J26617_10265371	+	184	249	**RNA**→ RplB (COG0090)rplB (PRK09374)→
Oba-1-1	NZ_CH724131.1	-	1639797	1639719	**RNA**→ RplB (COG0090)rplB (PRK09374)→ rpsS (PRK00357)RpsS (COG0185)→<br>rplV (PRK00565)Ribosomal_L22 (pfam00237)→ RpsC (COG0092)rpsC (PRK00310)→<br>RplP (COG0197)rplP (PRK09203)→
env-41	JGI12210J13797_10911808	-	154	79	**RNA**→ RplB (COG0090)rplB (PRK09374)→
env-42	SA_S1_NOR08_45m_c10088539	-	163	103	**RNA**→ RplB (COG0090)rplB (PRK09374)→
env-43	SA_S1_NOR05_45m_c10012273	+	805	865	**RNA**→ RplB (COG0090)rplB (PRK09374)→ rpsS (PRK00357)RpsS (COG0185)→<br>rplV (PRK00565)Ribosomal_L22 (pfam00237)→ KH (smart00322)30S_S3_KH (cd02412)→
env-44	SA_S2_NOR15_50m_c10022560	-	1787	1727	**RNA**→ RplB (COG0090)rplB (PRK09374)→ rpsS (PRK00357)Ribosomal_S19 (pfam00203)→<br>rplV (PRK00565)Ribosomal_L22 (pfam00237)→ rpsC (PRK00310)30S_S3_KH (cd02412)→
env-45	KGI_S2_ANT05_2345m_c1008115	+	360	420	**RNA**→ RplB (COG0090)rplB (PRK09374)→ rpsS (PRK00357)RpsS (COG0185)→<br>rplV (PRK00565)Ribosomal_L22 (pfam00237)→ RpsC (COG0092)rpsC (PRK00310)→
env-46	KGI_S1_ANT02_95m_c10175114	-	173	113	**RNA**→ RplB (COG0090)rplB (PRK09374)→
env-47	SA_S2_NOR13_50m_c1107468	-	300	240	**RNA**→ RplB (COG0090)rplB (PRK09374)→
env-48	KGI_S1_ANT03_95m_c1050218	+	27	87	**RNA**→ rplB (PRK09374)Ribosomal_L2 (pfam00181)→
env-49	JGI24723J26617_10562187	-	219	156	**RNA**→ RplB (COG0090)rplB (PRK09374)→
env-50	JGI24724J26744_10017014	-	875	812	**RNA**→ RplB (COG0090)rplB (PRK09374)→
env-51	JGI24025J20009_10010867	-	4900	4837	**RNA**→ RplB (COG0090)rplB (PRK09374)→ rpsS (PRK00357)RpsS (COG0185)→<br>rplV (PRK00565)Ribosomal_L22 (pfam00237)→ RpsC (COG0092)rpsC (PRK00310)→<br>RplP (COG0197)rplP (PRK09203)→
env-52	KGI_S1_ANT01_95m_c10003918	+	2539	2601	**RNA**→ RplB (COG0090)rplB (PRK09374)→ rpsS (PRK00357)RpsS (COG0185)→<br>rplV (PRK00565)Ribosomal_L22 (pfam00237)→ RpsC (COG0092)rpsC (PRK00310)→<br>RplP (COG0197)rplP (PRK09203)→
env-53	KGI_S2_ANT05_2345m_c1031605	-	159	97	**RNA**→ RplB (COG0090)rplB (PRK09374)→
env-54	KGI_S1_ANT02_95m_c10012913	+	2664	2726	**RNA**→ rplB (PRK09374)Ribosomal_L2 (pfam00181)→

env-55	AGBJ01004298.1	-	284	223	**RNA**→ ←hypo
env-56	JGI24025J20009_10035212	-	340	279	**RNA**→ rplB (PRK09374)Ribosomal_L2 (pfam00181)→
env-57	LGVE01157751.1	+	1200	1261	**RNA**→ rplB (PRK09374)Ribosomal_L2 (pfam00181)→
env-58	LGVF01211244.1	-	1626	1565	**RNA**→ RplB (COG0090)rplB (PRK09374)→ rpsS (PRK00357)Ribosomal_S19 (pfam00203)→ rplV (PRK00565)→<br>rplV (PRK00565)Ribosomal_L22 (pfam00237)→ rpsC (PRK00310)30S_S3_KH (cd02412)→
env-59	LGVD01030433.1	+	391	452	**RNA**→ rplB (PRK09374)Ribosomal_L2 (pfam00181)→
env-60	SA_S1_NOR08_45m_c10143553	+	557	618	**RNA**→ RplB (COG0090)rplB (PRK09374)→
env-61	SA_S2_NOR15_50m_c10181039	-	134	73	**RNA**→ RplB (COG0090)rplB (PRK09374)→
env-62	SA_S1_NOR02_45m_c1048433	-	379	318	**RNA**→ rplB (PRK09374)Ribosomal_L2 (pfam00181)→
env-63	SA_S2_NOR18_50m_1043735	+	332	393	**RNA**→ rplB (PRK09374)Ribosomal_L2 (pfam00181)→
env-64	LGOV01019582.1	+	657	718	**RNA**→ RplB (COG0090)rplB (PRK09374)→
Rli-1-1	NC_015730.1	-	3449426	3449359	**RNA**→ RplB (COG0090)rplB (PRK09374)→ rpsS (PRK00357)RpsS (COG0185)→<br>rplV (PRK00565)Ribosomal_L22 (pfam00237)→ RpsC (COG0092)rpsC (PRK00310)→<br>RplP (COG0197)rplP (PRK09203)→
env-65	JGI12109J20283_10879218	-	201	135	**RNA**→ RplB (COG0090)rplB (PRK09374)→
Rde-1-1	NC_008209.1	+	1346820	1346887	**RNA**→ RplB (COG0090)rplB (PRK09374)→ rpsS (PRK00357)RpsS (COG0185)→<br>rplV (PRK00565)Ribosomal_L22 (pfam00237)→ RpsC (COG0092)rpsC (PRK00310)→<br>RplP (COG0197)rplP (PRK09203)→
env-66	JGI11958J13698_1070723	-	120	52	**RNA**→ ←hypo
env-67	CAM_READ_0234679589	-	364	296	**RNA**→ rplB (PRK09374)Ribosomal_L2 (pfam00181)→
env-68	CAM_READ_0234796905	-	364	296	**RNA**→ rplB (PRK09374)Ribosomal_L2 (pfam00181)→
Rsp-15-1	NZ_JQLS01000008.1	+	336402	336479	**RNA**→ RplB (COG0090)rplB (PRK09374)→ rpsS (PRK00357)RpsS (COG0185)→<br>rplV (PRK00565)Ribosomal_L22 (pfam00237)→ RpsC (COG0092)rpsC (PRK00310)→<br>RplP (COG0197)rplP (PRK09203)→
env-69	CESQ01192690.1	-	546	470	**RNA**→ rplB (PRK09374)Ribosomal_L2 (pfam00181)→
Rsp-14-1	NZ_DS022276.1	-	423367	423284	**RNA**→ RplB (COG0090)rplB (PRK09374)→ rpsS (PRK00357)RpsS (COG0185)→<br>rplV (PRK00565)Ribosomal_L22 (pfam00237)→ RpsC (COG0092)rpsC (PRK00310)→<br>RplP (COG0197)rplP (PRK09203)→
env-70	CETR01181359.1	-	335	252	**RNA**→ RplB (COG0090)rplB (PRK09374)→
env-71	JGI11800J13271_1007151	+	1158	1250	**RNA**→
Csp-2-1	NZ_GG704599.1	+	399690	399782	**RNA**→ RplB (COG0090)rplB (PRK09374)→ RplB (COG0090)rplB (PRK09374)→<br>rpsS (PRK00357)RpsS (COG0185)→ rpsS (PRK00357)RpsS (COG0185)→<br>rplV (PRK00565)Ribosomal_L22 (pfam00237)→ rplV (PRK00565)Ribosomal_L22 (pfam00237)→<br>RpsC (COG0092)rpsC (PRK00310)→ RpsC (COG0092)rpsC (PRK00310)→ RplP (COG0197)rplP (PRK09203)→<br>RplP (COG0197)rplP (PRK09203)→
env-72	P_2C_Liq_1_UnCty_1008935	-	2463	2386	**RNA**→ RplB (COG0090)rplB (PRK09374)→ rpsS (PRK00357)RpsS (COG0185)→<br>rplV (PRK00565)Ribosomal_L22 (pfam00237)→ RpsC (COG0092)rpsC (PRK00310)→<br>rplP (PRK09203)Ribosomal_L16 (pfam00252)→
env-73	P_2C_Liq_2_UnCty_1004597	+	433	510	**RNA**→ RplB (COG0090)rplB (PRK09374)→ rpsS (PRK00357)RpsS (COG0185)→<br>rplV (PRK00565)Ribosomal_L22 (pfam00237)→ RpsC (COG0092)rpsC (PRK00310)→<br>rplP (PRK09203)Ribosomal_L16 (pfam00252)→
Nig-1-1	NZ_CVPC01000014.1	-	4708	4631	**RNA**→ RplB (COG0090)rplB (PRK09374)→ rpsS (PRK00357)RpsS (COG0185)→<br>rplV (PRK00565)Ribosomal_L22 (pfam00237)→ RpsC (COG0092)rpsC (PRK00310)→<br>RplP (COG0197)rplP (PRK09203)→
env-74	JGI12273J12029_10524331	+	216	286	**RNA**→
env-75	JGI24025J20009_10000374	-	31294	31182	**RNA**→ RplB (COG0090)rplB (PRK09374)→ rpsS (PRK00357)RpsS (COG0185)→<br>rplV (PRK00565)Ribosomal_L22 (pfam00237)→ RpsC (COG0092)rpsC (PRK00310)→<br>RplP (COG0197)rplP (PRK09203)→
env-76	JGI24025J20009_10010033	+	1689	1756	**RNA**→ RplB (COG0090)rplB (PRK09374)→ rpsS (PRK00357)RpsS (COG0185)→<br>rplV (PRK00565)Ribosomal_L22 (pfam00237)→ RpsC (COG0092)rpsC (PRK00310)→<br>RplP (COG0197)rplP (PRK09203)→
Tsp-1-1	NZ_JWLL01000009.1	-	233099	233024	**RNA**→ RplB (COG0090)rplB (PRK09374)→ rpsS (PRK00357)RpsS (COG0185)→<br>rplV (PRK00565)Ribosomal_L22 (pfam00237)→ RpsC (COG0092)rpsC (PRK00310)→<br>RplP (COG0197)rplP (PRK09203)→
env-77	2236876003_101132	+	388	448	**RNA**→ RplB (COG0090)rplB (PRK09374)→

env-78	PML_READ_00355505	+	143	210	**RNA**→
env-79	JGI20152J14361_10000074	+	12924	12991	**RNA**→ RplB (COG0090)rplB (PRK09374)→ rpsS (PRK00357)RpsS (COG0185)→ rplV (PRK00565)Ribosomal_L22 (pfam00237)→ RpsC (COG0092)rpsC (PRK00310)→ RplP (COG0197)rplP (PRK09203)→ hypo→
env-80	JGI20154J14316_10160884	+	228	295	**RNA**→ rplB (PRK09374)Ribosomal_L2 (pfam00181)→
env-81	JGI20153J14295_10001123	+	7148	7215	**RNA**→ RplB (COG0090)rplB (PRK09374)→ rpsS (PRK00357)RpsS (COG0185)→ rplV (PRK00565)Ribosomal_L22 (pfam00237)→ RpsC (COG0092)rpsC (PRK00310)→ RplP (COG0197)rplP (PRK09203)→ hypo→
env-82	JGI20153J14318_10001123	+	7148	7215	**RNA**→ RplB (COG0090)rplB (PRK09374)→ rpsS (PRK00357)RpsS (COG0185)→ rplV (PRK00565)Ribosomal_L22 (pfam00237)→ RpsC (COG0092)rpsC (PRK00310)→ RplP (COG0197)rplP (PRK09203)→ hypo→
Lve-2-1	NZ_CH672414.1	+	2669496	2669565	**RNA**→ RplB (COG0090)rplB (PRK09374)→ rpsS (PRK00357)RpsS (COG0185)→ rplV (PRK00565)Ribosomal_L22 (pfam00237)→ RpsC (COG0092)rpsC (PRK00310)→ RplP (COG0197)rplP (PRK09203)→
Lve-1-1	NZ_KB907973.1	+	38196	38265	**RNA**→ RplB (COG0090)rplB (PRK09374)→ rpsS (PRK00357)RpsS (COG0185)→ rplV (PRK00565)Ribosomal_L22 (pfam00237)→ RpsC (COG0092)rpsC (PRK00310)→ RplP (COG0197)rplP (PRK09203)→
env-83	JGI12210J13797_10455319	+	174	245	**RNA**→
env-84	JGI12210J13797_10023293	-	122	51	**RNA**→ ←hypo
Ogr-1-1	NZ_CH724107.1	-	1761097	1761017	**RNA**→ RplB (COG0090)rplB (PRK09374)→ rpsS (PRK00357)RpsS (COG0185)→ RplV (COG0091)rplV (PRK00565)→ RpsC (COG0092)rpsC (PRK00310)→ RplP (COG0197)rplP (PRK09203)→
env-85	JCVI_SCAF_1096627353865	+	440	504	**RNA**→ RplB (COG0090)rplB (PRK09374)→ Zw10 (pfam06248)→ rpsS (PRK00357)RpsS (COG0185)→ hypo→ rplV (PRK00565)Ribosomal_L22 (pfam00237)→ RpsC (COG0092)rpsC (PRK00310)→
env-86	PR_CR_10_Liq_1_inCR_1002101	+	720	784	**RNA**→ RplB (COG0090)rplB (PRK09374)→ rpsS (PRK00357)RpsS (COG0185)→ rplV (PRK00565)Ribosomal_L22 (pfam00237)→ RpsC (COG0092)rpsC (PRK00310)→ RplP (COG0197)rplP (PRK09203)→
env-87	PR_CR_10_Liq_3_inCR_1034197	-	612	548	**RNA**→ RplB (COG0090)rplB (PRK09374)→
Lsp-10-1	NZ_JXYE01000005.1	-	18000	17935	**RNA**→ RplB (COG0090)rplB (PRK09374)→ rpsS (PRK00357)RpsS (COG0185)→ rplV (PRK00565)Ribosomal_L22 (pfam00237)→ RpsC (COG0092)rpsC (PRK00310)→ RplP (COG0197)rplP (PRK09203)→
env-88	Antartic2_1216799	+	177	241	**RNA**→ RplB (COG0090)rplB (PRK09374)→
env-89	JGI25920J50251_10282450	-	274	210	**RNA**→ ←hypo
Aat-1-1	NZ_AQQY01000002.1	+	4134	4211	**RNA**→ RplB (COG0090)rplB (PRK09374)→ rpsS (PRK00357)RpsS (COG0185)→ rplV (PRK00565)Ribosomal_L22 (pfam00237)→ RpsC (COG0092)rpsC (PRK00310)→ RplP (COG0197)rplP (PRK09203)→
env-90	HLSNC_1002228	-	3054	2987	**RNA**→ RplB (COG0090)rplB (PRK09374)→ rpsS (PRK00357)RpsS (COG0185)→ rplV (PRK00565)Ribosomal_L22 (pfam00237)→ RpsC (COG0092)rpsC (PRK00310)→ RplP (COG0197)rplP (PRK09203)→ hypo→
env-91	KVRMV2_106490902	+	59	134	**RNA**→ RplB (COG0090)rplB (PRK09374)→
env-92	GYQO9XB02HJOI1	-	85	21	**RNA**→ ←hypo
env-93	4455295.3.416243	+	77	139	**RNA**→ rplB (PRK09374)Ribosomal_L2 (pfam00181)→
env-94	JGI20152J14361_10000792	-	16131	16066	**RNA**→ RplB (COG0090)rplB (PRK09374)→ rpsS (PRK00357)RpsS (COG0185)→ rplV (PRK00565)Ribosomal_L22 (pfam00237)→ RpsC (COG0092)rpsC (PRK00310)→ RplP (COG0197)rplP (PRK09203)→
env-95	JGI20152J14361_10021758	+	662	727	**RNA**→ RplB (COG0090)rplB (PRK09374)→ rpsS (PRK00357)RpsS (COG0185)→ rplV (PRK00565)Ribosomal_L22 (pfam00237)→
Pna-1-1	NZ_JHZF01000011.1	-	1853666	1853592	**RNA**→ RplB (COG0090)rplB (PRK09374)→ rpsS (PRK00357)RpsS (COG0185)→ rplV (PRK00565)Ribosomal_L22 (pfam00237)→ RpsC (COG0092)rpsC (PRK00310)→ RplP (COG0197)rplP (PRK09203)→
env-96	C687J26610_1065482	-	330	262	**RNA**→ rplB (PRK09374)Ribosomal_L2 (pfam00181)→
Sna-1-1	NZ_KI421509.1	-	2029881	2029817	**RNA**→ RplB (COG0090)rplB (PRK09374)→ rpsS (PRK00357)RpsS (COG0185)→ rplV (PRK00565)Ribosomal_L22 (pfam00237)→ RpsC (COG0092)rpsC (PRK00310)→ RplP (COG0197)rplP (PRK09203)→ TIGR02466 (TIGR02466)→
env-97	GYQO9XB02GIUBI	+	54	123	**RNA**→
env-98	DCrCPGB_c116271	+	75	144	**RNA**→ rplB (PRK09374)Ribosomal_L2 (pfam00181)→

env-99	KGI_S2_ANT04_2345m_c1008211	-	1538	1465	**RNA**→ RplB (COG0090)rplB (PRK09374)→ rpsS (PRK00357)RpsS (COG0185)→ rplV (PRK00565)Ribosomal_L22 (pfam00237)→
env-100	KGI_S1_ANT02_95m_c10000174	+	24541	24614	**RNA**→ RplB (COG0090)rplB (PRK09374)→ rpsS (PRK00357)RpsS (COG0185)→ rplV (PRK00565)Ribosomal_L22 (pfam00237)→ RpsC (COG0092)rpsC (PRK00310)→ RplP (COG0197)rplP (PRK09203)→
env-101	KGI_S1_ANT01_95m_c10002670	+	4835	4908	**RNA**→ RplB (COG0090)rplB (PRK09374)→ rpsS (PRK00357)RpsS (COG0185)→ rplV (PRK00565)Ribosomal_L22 (pfam00237)→ RpsC (COG0092)rpsC (PRK00310)→ RplP (COG0197)rplP (PRK09203)→
env-102	KGI_S2_ANT05_2345m_c1002025	-	4049	3976	**RNA**→ RplB (COG0090)rplB (PRK09374)→ rpsS (PRK00357)RpsS (COG0185)→ rplV (PRK00565)Ribosomal_L22 (pfam00237)→ RpsC (COG0092)rpsC (PRK00310)→ RplP (COG0197)rplP (PRK09203)→
env-103	SR_TP_S13sol_1015872	-	193	120	**RNA**→ RplB (COG0090)rplB (PRK09374)→
env-104	HLSNC_1000102	+	4292	4365	**RNA**→ RplB (COG0090)rplB (PRK09374)→ rpsS (PRK00357)RpsS (COG0185)→ rplV (PRK00565)Ribosomal_L22 (pfam00237)→ RpsC (COG0092)rpsC (PRK00310)→ RplP (COG0197)rplP (PRK09203)→
Jsp-1-1	NC_007802.1	+	574447	574519	**RNA**→ RplB (COG0090)rplB (PRK09374)→ rpsS (PRK00357)RpsS (COG0185)→ rplV (PRK00565)Ribosomal_L22 (pfam00237)→ RpsC (COG0092)rpsC (PRK00310)→ RplP (COG0197)rplP (PRK09203)→
env-105	KVRMV2_101198589	+	2281	2349	**RNA**→ rplB (PRK09374)Ribosomal_L2 (pfam00181)→
env-106	PR_CR_10_Liq_1_inCR_1005216	-	2908	2842	**RNA**→ RplB (COG0090)rplB (PRK09374)→ rpsS (PRK00357)RpsS (COG0185)→ rplV (PRK00565)Ribosomal_L22 (pfam00237)→ RpsC (COG0092)rpsC (PRK00310)→ RplP (COG0197)rplP (PRK09203)→
env-107	PR_CR_10_Liq_3_inCR_1001525	-	3425	3359	**RNA**→ RplB (COG0090)rplB (PRK09374)→ rpsS (PRK00357)RpsS (COG0185)→ rplV (PRK00565)Ribosomal_L22 (pfam00237)→ RpsC (COG0092)rpsC (PRK00310)→ RplP (COG0197)rplP (PRK09203)→
env-108	GSLSAAI_contig03181	+	626	692	**RNA**→
env-109	ElkS_mat_CD2A_1005801	-	1817	1752	**RNA**→ RplB (COG0090)rplB (PRK09374)→ rpsS (PRK00357)RpsS (COG0185)→ rplV (PRK00565)Ribosomal_L22 (pfam00237)→ rpsC (PRK00310)30S_S3_KH (cd02412)→
env-110	ElkS_mat_MD6A_1013394	+	164	229	**RNA**→ RplB (COG0090)rplB (PRK09374)→ rpsS (PRK00357)RpsS (COG0185)→ rplV (PRK00565)Ribosomal_L22 (pfam00237)→ rpsC (PRK00310)30S_S3_KH (cd02412)→
env-111	JGI12210J13797_11132003	+	2467	2532	**RNA**→ RplB (COG0090)rplB (PRK09374)→ rpsS (PRK00357)RpsS (COG0185)→ rplV (PRK00565)Ribosomal_L22 (pfam00237)→ RpsC (COG0092)rpsC (PRK00310)→ RplP (COG0197)rplP (PRK09203)→
env-112	JGI12210J13797_11132005	-	3162	3097	**RNA**→ RplB (COG0090)rplB (PRK09374)→ rpsS (PRK00357)RpsS (COG0185)→ rplV (PRK00565)Ribosomal_L22 (pfam00237)→ RpsC (COG0092)rpsC (PRK00310)→ RplP (COG0197)rplP (PRK09203)→
env-113	JGI11876J14442_10003279	-	3251	3186	**RNA**→ RplB (COG0090)rplB (PRK09374)→ rpsS (PRK00357)RpsS (COG0185)→ rplV (PRK00565)Ribosomal_L22 (pfam00237)→ RpsC (COG0092)rpsC (PRK00310)→ RplP (COG0197)rplP (PRK09203)→
env-114	JGI12109J20283_10578297	+	2553	2618	**RNA**→ rplB (PRK09374)Ribosomal_L2 (pfam00181)→
env-115	JGI12109J20283_10578298	-	338	273	**RNA**→ rplB (PRK09374)Ribosomal_L2 (pfam00181)→
env-116	JGI12210J13797_11558909	+	43	103	**RNA**→ ←hypo
env-117	JGI12210J13797_11938347	-	70	10	**RNA**→ ←hypo
env-118	CENR01018324.1	+	1102	1170	**RNA**→ RplB (COG0090)rplB (PRK09374)→
env-119	CENV01010391.1	-	2234	2166	**RNA**→ RplB (COG0090)rplB (PRK09374)→ rpsS (PRK00357)RpsS (COG0185)→ RplV (COG0091)rplV (PRK00565)→ RpsC (COG0092)rpsC (PRK00310)→
env-120	CERO01013343.1	+	295	363	**RNA**→ RplB (COG0090)rplB (PRK09374)→
env-121	JGI24724J26744_10870797	+	32	99	**RNA**→ RplB (COG0090)rplB (PRK09374)→
env-122	JGI12210J13797_10838428	-	914	850	**RNA**→ RplB (COG0090)rplB (PRK09374)→
env-123	JGI12210J13797_10838429	+	87	151	**RNA**→ RplB (COG0090)rplB (PRK09374)→
env-124	JGI20152J14361_10179686	+	269	339	**RNA**→ ←hypo
env-125	SA_S1_NOR08_45m_c10004046	+	4200	4267	**RNA**→ RplB (COG0090)rplB (PRK09374)→ rpsS (PRK00357)RpsS (COG0185)→ rplV (PRK00565)Ribosomal_L22 (pfam00237)→ RpsC (COG0092)rpsC (PRK00310)→ RplP (COG0197)rplP (PRK09203)→

env-126	SA_S2_NOR15_50m_c10018770	+	306	373	RNA→ RplB (COG0090)rplB (PRK09374)→ rpsS (PRK00357)RpsS (COG0185)→<br>rplV (PRK00565)Ribosomal_L22 (pfam00237)→ RpsC (COG0092)rpsC (PRK00310)→<br>RplP (COG0197)rplP (PRK09203)→
env-127	SA_S1_NOR05_45m_c10315404	+	255	322	RNA→
Ote-1-1	NZ_CP012160.1	+	252086	252153	RNA→ RplB (COG0090)rplB (PRK09374)→ rpsS (PRK00357)RpsS (COG0185)→<br>rplV (PRK00565)Ribosomal_L22 (pfam00237)→ RpsC (COG0092)rpsC (PRK00310)→<br>RplP (COG0197)rplP (PRK09203)→
Oan-1-1	NC_020911.1	-	4346201	4346135	RNA→ RplB (COG0090)rplB (PRK09374)→ rpsS (PRK00357)RpsS (COG0185)→<br>rplV (PRK00565)Ribosomal_L22 (pfam00237)→ RpsC (COG0092)rpsC (PRK00310)→<br>RplP (COG0197)rplP (PRK09203)→
Oar-1-1	NC_020908.1	+	3931342	3931406	RNA→ RplB (COG0090)rplB (PRK09374)→ rpsS (PRK00357)RpsS (COG0185)→<br>rplV (PRK00565)Ribosomal_L22 (pfam00237)→ RpsC (COG0092)rpsC (PRK00310)→<br>RplP (COG0197)rplP (PRK09203)→
env-128	KGI_S1_ANT01_95m_c10350259	-	325	258	RNA→ rplB (PRK09374)Ribosomal_L2 (pfam00181)→
env-129	SA_S1_NOR08_45m_c10019190	+	380	447	RNA→ RplB (COG0090)rplB (PRK09374)→ rpsS (PRK00357)RpsS (COG0185)→<br>rplV (PRK00565)Ribosomal_L22 (pfam00237)→ RpsC (COG0092)rpsC (PRK00310)→<br>rplP (PRK09203)Ribosomal_L16 (pfam00252)→
env-130	KGI_S2_ANT04_2345m_c1106538	-	368	301	RNA→ rplB (PRK09374)Ribosomal_L2 (pfam00181)→
env-131	KGI_S2_ANT05_2345m_c1147958	+	34	101	RNA→ rplB (PRK09374)Ribosomal_L2 (pfam00181)→
env-132	KGI_S1_ANT03_95m_c1027476	+	53	120	RNA→ RplB (COG0090)rplB (PRK09374)→
env-133	KGI_S1_ANT02_95m_c10267034	-	323	256	RNA→ RplB (COG0090)rplB (PRK09374)→
env-134	SA_S2_NOR18_50m_1258724	+	183	250	RNA→
env-135	SA_S1_NOR05_45m_c10100002	+	326	393	RNA→ rplB (PRK09374)Ribosomal_L2 (pfam00181)→
Rsp-9-1	NZ_AAYB01000008.1	+	62353	62420	RNA→ RplB (COG0090)rplB (PRK09374)→ RplB (COG0090)rplB (PRK09374)→<br>RplB (COG0090)rplB (PRK09374)→ RplB (COG0090)rplB (PRK09374)→ RplB (COG0090)rplB (PRK09374)→<br>rpsS (PRK00357)RpsS (COG0185)→ rpsS (PRK00357)RpsS (COG0185)→<br>rplV (PRK00565)Ribosomal_L22 (pfam00237)→ rplV (PRK00565)Ribosomal_L22 (pfam00237)→<br>RpsC (COG0092)rpsC (PRK00310)→ RpsC (COG0092)rpsC (PRK00310)→ RplP (COG0197)rplP (PRK09203)→<br>RplP (COG0197)rplP (PRK09203)→
env-136	JGI24724J26744_10208358	+	330	396	RNA→ RplB (COG0090)rplB (PRK09374)→
env-137	JGI24723J26617_10144364	-	304	238	RNA→ RplB (COG0090)rplB (PRK09374)→
Pan-1-1	NZ_LGHU01000123.1	+	98116	98182	RNA→ RplB (COG0090)rplB (PRK09374)→ rpsS (PRK00357)RpsS (COG0185)→<br>rplV (PRK00565)Ribosomal_L22 (pfam00237)→ RpsC (COG0092)rpsC (PRK00310)→<br>RplP (COG0197)rplP (PRK09203)→
Pfe-1-1	NZ_LGHV01000017.1	-	8115	8049	RNA→ RplB (COG0090)rplB (PRK09374)→ rpsS (PRK00357)RpsS (COG0185)→<br>rplV (PRK00565)Ribosomal_L22 (pfam00237)→ RpsC (COG0092)rpsC (PRK00310)→<br>RplP (COG0197)rplP (PRK09203)→
Pfe-2-1	NZ_ATVN01000032.1	-	7903	7837	RNA→ RplB (COG0090)rplB (PRK09374)→ rpsS (PRK00357)RpsS (COG0185)→<br>rplV (PRK00565)Ribosomal_L22 (pfam00237)→ RpsC (COG0092)rpsC (PRK00310)→<br>RplP (COG0197)rplP (PRK09203)→
Lci-1-1	NZ_BATB01000009.1	-	87402	87331	RNA→ RplB (COG0090)rplB (PRK09374)→ rpsS (PRK00357)RpsS (COG0185)→<br>rplV (PRK00565)Ribosomal_L22 (pfam00237)→ RpsC (COG0092)rpsC (PRK00310)→<br>RpsC (COG0092)rpsC (PRK00310)→ hypo→ RplP (COG0197)rplP (PRK09203)→
env-138	JGI24724J26744_10487725	+	233	347	RNA→ ←hypo
Abo-1-1	NZ_JXYH01000044.1	-	31116	31026	RNA→ RplB (COG0090)rplB (PRK09374)→ rpsS (PRK00357)RpsS (COG0185)→<br>rplV (PRK00565)Ribosomal_L22 (pfam00237)→ RpsC (COG0092)rpsC (PRK00310)→<br>RplP (COG0197)rplP (PRK09203)→
Rha-1-1	NZ_JALZ01000013.1	+	2420	2528	RNA→ RplB (COG0090)rplB (PRK09374)→ rpsS (PRK00357)RpsS (COG0185)→<br>rplV (PRK00565)Ribosomal_L22 (pfam00237)→ RpsC (COG0092)rpsC (PRK00310)→<br>RplP (COG0197)rplP (PRK09203)→ hypo→ 2OG-FeII_Oxy_5 (pfam13759)→
Mal-1-1	NZ_JSUQ01000002.1	-	319288	319208	RNA→ RplB (COG0090)rplB (PRK09374)→ rpsS (PRK00357)RpsS (COG0185)→<br>rplV (PRK00565)Ribosomal_L22 (pfam00237)→ RpsC (COG0092)rpsC (PRK00310)→<br>RplP (COG0197)rplP (PRK09203)→
env-139	JGI12075J13256_1001549	+	1682	1762	RNA→ RplB (COG0090)rplB (PRK09374)→ rpsS (PRK00357)RpsS (COG0185)→<br>rplV (PRK00565)Ribosomal_L22 (pfam00237)→
env-140	JGI12112J13074_1001921	-	771	691	RNA→ RplB (COG0090)rplB (PRK09374)→

env-141	JGI24724J26744_10066135	-	1234	1166	**RNA**→ RplB (COG0090)rplB (PRK09374)→ rpsS (PRK00357)RpsS (COG0185)→
env-142	JGI24723J26617_10483647	+	129	197	**RNA**→ RplB (COG0090)rplB (PRK09374)→
env-143	JCVI_SCAF_1101667320527	-	473	406	**RNA**→ rplB (PRK09374)Ribosomal_L2 (pfam00181)→
env-144	APMI01028553.1	-	2484	2421	**RNA**→ RplB (COG0090)rplB (PRK09374)→ rpsS (PRK00357)RpsS (COG0185)→
					rplV (PRK00565)Ribosomal_L22 (pfam00237)→ RpsC (COG0092)rpsC (PRK00310)→
					rplP (PRK09203)Ribosomal_L16 (pfam00252)→
env-145	JGI24724J26744_10205793	-	600	536	**RNA**→ RplB (COG0090)rplB (PRK09374)→
env-146	JGI25321J39296_1078939	-	191	127	**RNA**→ RplB (COG0090)rplB (PRK09374)→
env-147	JGI25321J50212_10013221	-	2584	2520	**RNA**→ RplB (COG0090)rplB (PRK09374)→ rpsS (PRK00357)RpsS (COG0185)→
					rplV (PRK00565)Ribosomal_L22 (pfam00237)→ RpsC (COG0092)rpsC (PRK00310)→
					RplP (COG0197)rplP (PRK09203)→
env-148	JGI24025J20009_10017180	+	442	544	**RNA**→ RplB (COG0090)rplB (PRK09374)→ rpsS (PRK00357)RpsS (COG0185)→
					rplV (PRK00565)Ribosomal_L22 (pfam00237)→ RpsC (COG0092)rpsC (PRK00310)→
					RplP (COG0197)rplP (PRK09203)→
env-149	ATLU01001954.1	+	1098	1219	**RNA**→ rplB (PRK09374)Ribosomal_L2 (pfam00181)→
env-150	JGI12210J13797_10050283	+	46	137	**RNA**→ RplB (COG0090)rplB (PRK09374)→
env-151	JGI12210J13797_10199298	-	104	13	**RNA**→
env-152	JGI11876J14442_10101235	-	251	160	**RNA**→ RplB (COG0090)rplB (PRK09374)→
env-153	JGI12109J20283_10360159	-	6078	5987	**RNA**→ RplB (COG0090)rplB (PRK09374)→ rpsS (PRK00357)RpsS (COG0185)→
					rplV (PRK00565)Ribosomal_L22 (pfam00237)→ RpsC (COG0092)rpsC (PRK00310)→
					RplP (COG0197)rplP (PRK09203)→
env-154	JGI12109J20283_10360162	+	86	177	**RNA**→ RplB (COG0090)rplB (PRK09374)→ rpsS (PRK00357)RpsS (COG0185)→
					rplV (PRK00565)Ribosomal_L22 (pfam00237)→ rpsC (PRK00310)30S_S3_KH (cd02412)→
env-155	ElkS_mat_MD6A_1245876	+	17	108	**RNA**→ RplB (COG0090)rplB (PRK09374)→
env-156	3300001605_10196433	+	760	822	**RNA**→ rplB (PRK09374)Ribosomal_L2 (pfam00181)→
env-157	JGI24723J26617_10002649	-	2300	2237	**RNA**→ RplB (COG0090)rplB (PRK09374)→ rpsS (PRK00357)RpsS (COG0185)→
					rplV (PRK00565)Ribosomal_L22 (pfam00237)→ RpsC (COG0092)rpsC (PRK00310)→
env-158	SA_S1_NOR05_45m_c10038069	-	1091	1029	**RNA**→ RplB (COG0090)rplB (PRK09374)→ rpsS (PRK00357)Ribosomal_S19 (pfam00203)→
env-159	SA_S1_NOR08_45m_c10005006	-	2877	2815	**RNA**→ RplB (COG0090)rplB (PRK09374)→ rpsS (PRK00357)RpsS (COG0185)→
					rplV (PRK00565)Ribosomal_L22 (pfam00237)→ RpsC (COG0092)rpsC (PRK00310)→
					RplP (COG0197)rplP (PRK09203)→
env-160	SA_S2_NOR15_50m_c10038301	+	1693	1755	**RNA**→ rplB (PRK09374)Ribosomal_L2 (pfam00181)→
env-161	SA_S1_NOR02_45m_c1051392	-	97	35	**RNA**→
env-162	KGI_S1_ANT01_95m_c10509536	-	192	130	**RNA**→ RplB (COG0090)rplB (PRK09374)→
Kvu-1-1	NC_017384.1	-	2210425	2210363	**RNA**→ RplB (COG0090)rplB (PRK09374)→ rpsS (PRK00357)RpsS (COG0185)→
					rplV (PRK00565)Ribosomal_L22 (pfam00237)→ RpsC (COG0092)rpsC (PRK00310)→
					RplP (COG0197)rplP (PRK09203)→
Kvu-2-1	NC_014625.1	-	2496662	2496600	**RNA**→ RplB (COG0090)rplB (PRK09374)→ rpsS (PRK00357)RpsS (COG0185)→
env-163	JGI24723J26617_10010575	-	238	176	**RNA**→ RplB (COG0090)rplB (PRK09374)→
Lho-1-1	NZ_KB822996.1	+	454151	454214	**RNA**→ RplB (COG0090)rplB (PRK09374)→ rpsS (PRK00357)RpsS (COG0185)→
					rplV (PRK00565)Ribosomal_L22 (pfam00237)→ RpsC (COG0092)rpsC (PRK00310)→
					RplP (COG0197)rplP (PRK09203)→
CRh-1-1	NZ_LFTY01000002.1	+	138295	138372	**RNA**→ RplB (COG0090)rplB (PRK09374)→ rpsS (PRK00357)RpsS (COG0185)→
					rplV (PRK00565)Ribosomal_L22 (pfam00237)→ RpsC (COG0092)rpsC (PRK00310)→
					RplP (COG0197)rplP (PRK09203)→
env-164	CETY01316910.1	+	295	359	**RNA**→ rplB (PRK09374)Ribosomal_L2 (pfam00181)→
env-165	SI34jun09_120m_1114364	+	319	383	**RNA**→
Ogu-1-1	NZ_AMGO01000054.1	+	2304	2368	**RNA**→ RplB (COG0090)rplB (PRK09374)→ RplB (COG0090)rplB (PRK09374)→
					rpsS (PRK00357)RpsS (COG0185)→ rpsS (PRK00357)RpsS (COG0185)→
					rplV (PRK00565)Ribosomal_L22 (pfam00237)→ rplV (PRK00565)Ribosomal_L22 (pfam00237)→
					RpsC (COG0092)rpsC (PRK00310)→ RpsC (COG0092)rpsC (PRK00310)→ RplP (COG0197)rplP (PRK09203)→
					RplP (COG0197)rplP (PRK09203)→
env-166	_GX4IIKE02G7TTM	+	176	245	**RNA**→ RplB (COG0090)rplB (PRK09374)→
Paq-1-1	NZ_LGHS01000007.1	-	43447	43382	**RNA**→ RplB (COG0090)rplB (PRK09374)→ rpsS (PRK00357)RpsS (COG0185)→
					rplV (PRK00565)Ribosomal_L22 (pfam00237)→ RpsC (COG0092)rpsC (PRK00310)→
					RplP (COG0197)rplP (PRK09203)→

env-167	CERE01101430.1	+	2524	2585	**RNA**→ RplB (COG0090)rplB (PRK09374)→ rpsS (PRK00357)RpsS (COG0185)→<br>rplV (PRK00565)Ribosomal_L22 (pfam00237)→ RpsC (COG0092)rpsC (PRK00310)→
env-168	ATLU01000020.1	+	4112	4189	**RNA**→ RplB (COG0090)rplB (PRK09374)→ rpsS (PRK00357)RpsS (COG0185)→<br>rplV (PRK00565)Ribosomal_L22 (pfam00237)→ RpsC (COG0092)rpsC (PRK00310)→<br>RplP (COG0197)rplP (PRK09203)→
env-169	CERH01017500.1	-	305	240	**RNA**→ RplB (COG0090)rplB (PRK09374)→
Rnu-1-1	NZ_CH724156.1	-	347220	347155	**RNA**→ RplB (COG0090)rplB (PRK09374)→ rpsS (PRK00357)RpsS (COG0185)→<br>RplV (COG0091)rplV (PRK00565)→ RpsC (COG0092)rpsC (PRK00310)→
env-170	CEQZ01016289.1	-	1777	1712	**RNA**→ RplB (COG0090)rplB (PRK09374)→ rpsS (PRK00357)RpsS (COG0185)→<br>rplV (PRK00565)Ribosomal_L22 (pfam00237)→ rpsC (PRK00310)30S_S3_KH (cd02412)→
env-171	KVRMV2_100592709	+	54	115	**RNA**→ RplB (COG0090)rplB (PRK09374)→
env-172	2236876011_238237	+	256	328	**RNA**→ ←hypo
Rba-4-1	NZ_AAXZ01000001.1	+	629695	629767	**RNA**→ RplB (COG0090)rplB (PRK09374)→ RplB (COG0090)rplB (PRK09374)→<br>rpsS (PRK00357)RpsS (COG0185)→ rpsS (PRK00357)RpsS (COG0185)→<br>rplV (PRK00565)Ribosomal_L22 (pfam00237)→ rplV (PRK00565)Ribosomal_L22 (pfam00237)→<br>RpsC (COG0092)rpsC (PRK00310)→ RpsC (COG0092)rpsC (PRK00310)→ RplP (COG0197)rplP (PRK09203)→<br>RplP (COG0197)rplP (PRK09203)→
env-173	JCVI_SCAF_1096628174387	+	532	596	**RNA**→ RplB (COG0090)rplB (PRK09374)→ rpsC (PRK00310)Ribosomal_S3_C (pfam00189)→<br>RplP (COG0197)rplP (PRK09203)→
Sps-1-1	NZ_JAMD01000003.1	-	509244	509179	**RNA**→ RplB (COG0090)rplB (PRK09374)→ rpsS (PRK00357)RpsS (COG0185)→<br>rplV (PRK00565)Ribosomal_L22 (pfam00237)→ RpsC (COG0092)rpsC (PRK00310)→<br>RplP (COG0197)rplP (PRK09203)→
Ssp-2-1	NZ_JIBC01000009.1	+	270672	270737	**RNA**→ RplB (COG0090)rplB (PRK09374)→ rpsS (PRK00357)RpsS (COG0185)→<br>rplV (PRK00565)Ribosomal_L22 (pfam00237)→ RpsC (COG0092)rpsC (PRK00310)→<br>RplP (COG0197)rplP (PRK09203)→
env-174	JGI24723J26617_10018613	+	746	824	**RNA**→ RplB (COG0090)rplB (PRK09374)→ rpsS (PRK00357)RpsS (COG0185)→<br>rplV (PRK00565)Ribosomal_L22 (pfam00237)→ RpsC (COG0092)rpsC (PRK00310)→<br>rplP (PRK09203)Ribosomal_L16 (pfam00252)→
Osp-1-1	NZ_JAFT01000005.1	-	653626	653563	**RNA**→ RplB (COG0090)rplB (PRK09374)→ rpsS (PRK00357)RpsS (COG0185)→<br>rplV (PRK00565)Ribosomal_L22 (pfam00237)→ RpsC (COG0092)rpsC (PRK00310)→<br>RplP (COG0197)rplP (PRK09203)→
Sst-1-1	NZ_AAYA01000001.1	+	200930	200993	**RNA**→ RplB (COG0090)rplB (PRK09374)→ RplB (COG0090)rplB (PRK09374)→<br>rpsS (PRK00357)RpsS (COG0185)→ rpsS (PRK00357)RpsS (COG0185)→<br>rplV (PRK00565)Ribosomal_L22 (pfam00237)→ rplV (PRK00565)Ribosomal_L22 (pfam00237)→<br>RpsC (COG0092)rpsC (PRK00310)→ RpsC (COG0092)rpsC (PRK00310)→ RplP (COG0197)rplP (PRK09203)→<br>RplP (COG0197)rplP (PRK09203)→
env-175	JCVI_READ_1103242074397	+	704	765	**RNA**→
env-176	JGI24724J26744_10049941	+	488	552	**RNA**→ RplB (COG0090)rplB (PRK09374)→ rpsS (PRK00357)RpsS (COG0185)→ hypo→
Rsp-16-1	NZ_ABCL01000006.1	+	350229	350292	**RNA**→ RplB (COG0090)rplB (PRK09374)→ RplB (COG0090)rplB (PRK09374)→<br>rpsS (PRK00357)RpsS (COG0185)→ rpsS (PRK00357)RpsS (COG0185)→<br>rplV (PRK00565)Ribosomal_L22 (pfam00237)→ rplV (PRK00565)Ribosomal_L22 (pfam00237)→<br>RpsC (COG0092)rpsC (PRK00310)→ RpsC (COG0092)rpsC (PRK00310)→ RplP (COG0197)rplP (PRK09203)→<br>RplP (COG0197)rplP (PRK09203)→
env-177	JGI24724J26744_10021376	+	90	155	**RNA**→ RplB (COG0090)rplB (PRK09374)→ rpsS (PRK00357)RpsS (COG0185)→<br>rplV (PRK00565)Ribosomal_L22 (pfam00237)→ RpsC (COG0092)rpsC (PRK00310)→<br>RplP (COG0197)rplP (PRK09203)→
env-178	3300001592_10000630	+	2376	2441	**RNA**→ RplB (COG0090)rplB (PRK09374)→ rpsS (PRK00357)RpsS (COG0185)→<br>rplV (PRK00565)Ribosomal_L22 (pfam00237)→ KH (smart00322)30S_S3_KH (cd02412)→<br>rpsC (PRK00310)Ribosomal_S3_C (pfam00189)→ RplP (COG0197)rplP (PRK09203)→
env-179	JGI25317J35169_1006515	+	816	879	**RNA**→ RplB (COG0090)rplB (PRK09374)→ rpsS (PRK00357)RpsS (COG0185)→<br>rplV (PRK00565)Ribosomal_L22 (pfam00237)→ RpsC (COG0092)rpsC (PRK00310)→<br>RplP (COG0197)rplP (PRK09203)→
env-180	JGI25319J35699_1002819	+	816	879	**RNA**→ RplB (COG0090)rplB (PRK09374)→ rpsS (PRK00357)RpsS (COG0185)→<br>rplV (PRK00565)Ribosomal_L22 (pfam00237)→ RpsC (COG0092)rpsC (PRK00310)→<br>RplP (COG0197)rplP (PRK09203)→

env-181	JGI25322J35698_1003836	+	3368	3431	**RNA**→ RplB (COG0090)rplB (PRK09374)→ rpsS (PRK00357)RpsS (COG0185)→ rplV (PRK00565)Ribosomal_L22 (pfam00237)→ RpsC (COG0092)rpsC (PRK00310)→ RplP (COG0197)rplP (PRK09203)→
env-182	JGI25318J35512_1004712	+	816	879	**RNA**→ RplB (COG0090)rplB (PRK09374)→ rpsS (PRK00357)RpsS (COG0185)→ rplV (PRK00565)Ribosomal_L22 (pfam00237)→ RpsC (COG0092)rpsC (PRK00310)→ RplP (COG0197)rplP (PRK09203)→
env-183	JGI25321J39296_1001477	-	10112	10049	**RNA**→ RplB (COG0090)rplB (PRK09374)→ rpsS (PRK00357)RpsS (COG0185)→ rplV (PRK00565)Ribosomal_L22 (pfam00237)→ RpsC (COG0092)rpsC (PRK00310)→ RplP (COG0197)rplP (PRK09203)→
env-184	JGI25320J50211_1001516	-	2747	2684	**RNA**→ RplB (COG0090)rplB (PRK09374)→ rpsS (PRK00357)RpsS (COG0185)→ rplV (PRK00565)Ribosomal_L22 (pfam00237)→ RpsC (COG0092)rpsC (PRK00310)→ RplP (COG0197)rplP (PRK09203)→
env-185	JGI25321J50212_10005715	-	4273	4210	**RNA**→ RplB (COG0090)rplB (PRK09374)→ rpsS (PRK00357)RpsS (COG0185)→ rplV (PRK00565)Ribosomal_L22 (pfam00237)→ RpsC (COG0092)rpsC (PRK00310)→ RplP (COG0197)rplP (PRK09203)→
env-186	JGI24724J26744_10053916	+	237	300	**RNA**→ RplB (COG0090)rplB (PRK09374)→ rpsS (PRK00357)RpsS (COG0185)→ rplV (PRK00565)Ribosomal_L22 (pfam00237)→
Rsp-13-1	NZ_CH902584.1	+	1991396	1991459	**RNA**→ RplB (COG0090)rplB (PRK09374)→ rpsS (PRK00357)RpsS (COG0185)→ rplV (PRK00565)Ribosomal_L22 (pfam00237)→ RpsC (COG0092)rpsC (PRK00310)→ RplP (COG0197)rplP (PRK09203)→
env-187	CERE01077594.1	+	40	103	**RNA**→ rplB (PRK09374)Ribosomal_L2 (pfam00181)→
Rmu-1-1	NZ_KN293980.1	+	258018	258081	**RNA**→ RplB (COG0090)rplB (PRK09374)→ rpsS (PRK00357)RpsS (COG0185)→ rplV (PRK00565)Ribosomal_L22 (pfam00237)→ RpsC (COG0092)rpsC (PRK00310)→ RplP (COG0197)rplP (PRK09203)→
env-188	GSLSAS_GLMUJHB01ANH3P	-	111	36	**RNA**→ ←hypo
env-189	PR_CR_10_Liq_1_inCR_1002975	-	4609	4543	**RNA**→ RplB (COG0090)rplB (PRK09374)→ rpsS (PRK00357)RpsS (COG0185)→ rplV (PRK00565)Ribosomal_L22 (pfam00237)→ RpsC (COG0092)rpsC (PRK00310)→ RplP (COG0197)rplP (PRK09203)→
env-190	GSLSAS_contig20752	+	91	157	**RNA**→ rplB (PRK09374)Ribosomal_L2 (pfam00181)→
env-191	GSLSAAI_GFP0ZZ301AT1AJ	+	118	184	**RNA**→ RplB (COG0090)rplB (PRK09374)→
env-192	P_2C_Liq_2_UnCty_1112442	+	202	268	**RNA**→ RplB (COG0090)rplB (PRK09374)→
env-193	JCVI_SCAF_1101668363300	-	793	725	**RNA**→ RplB (COG0090)rplB (PRK09374)→
env-194	CESM01031007.1	+	3318	3386	**RNA**→ RplB (COG0090)rplB (PRK09374)→ rpsS (PRK00357)RpsS (COG0185)→ rplV (PRK00565)Ribosomal_L22 (pfam00237)→ rpsC (PRK00310)30S_S3_KH (cd02412)→
env-195	LPaug09P1610m_c1075736	-	346	278	**RNA**→ rplB (PRK09374)Ribosomal_L2 (pfam00181)→
env-196	CENZ01075739.1	-	473	405	**RNA**→ rplB (PRK09374)Ribosomal_L2 (pfam00181)→
env-197	JCVI_SCAF_1096627291439	+	336	404	**RNA**→ RplB (COG0090)rplB (PRK09374)→ rpsS (PRK00357)RpsS (COG0185)→
env-198	CERM01028656.1	+	736	804	**RNA**→ RplB (COG0090)rplB (PRK09374)→
env-199	CEWO01045537.1	+	245	313	**RNA**→ rplB (PRK09374)Ribosomal_L2 (pfam00181)→
env-200	CEVX01073106.1	+	997	1065	**RNA**→ RplB (COG0090)rplB (PRK09374)→ rpsS (PRK00357)RpsS (COG0185)→ rplV (PRK00565)Ribosomal_L22 (pfam00237)→ RpsC (COG0092)rpsC (PRK00310)→ RplP (COG0197)rplP (PRK09203)→
env-201	CEUB01117696.1	+	96	164	**RNA**→ rplB (PRK09374)Ribosomal_L2 (pfam00181)→
env-202	CEUG01069584.1	-	338	270	**RNA**→ rplB (PRK09374)Ribosomal_L2 (pfam00181)→
env-203	CEVF01073172.1	-	937	869	**RNA**→ RplB (COG0090)rplB (PRK09374)→
env-204	CEQW01075709.1	+	117	185	**RNA**→ rplB (PRK09374)Ribosomal_L2 (pfam00181)→
env-205	CESW01144144.1	-	3772	3704	**RNA**→ RplB (COG0090)rplB (PRK09374)→ rpsS (PRK00357)RpsS (COG0185)→ rplV (PRK00565)Ribosomal_L22 (pfam00237)→ RpsC (COG0092)rpsC (PRK00310)→ RplP (COG0197)rplP (PRK09203)→
env-206	CERG01046692.1	-	1707	1639	**RNA**→ RplB (COG0090)rplB (PRK09374)→ rpsS (PRK00357)RpsS (COG0185)→ rplV (PRK00565)Ribosomal_L22 (pfam00237)→ rpsC (PRK00310)30S_S3_KH (cd02412)→
env-207	CENJ01096940.1	+	1807	1875	**RNA**→ RplB (COG0090)rplB (PRK09374)→ rpsS (PRK00357)RpsS (COG0185)→ rplV (PRK00565)Ribosomal_L22 (pfam00237)→ RpsC (COG0092)rpsC (PRK00310)→ RplP (COG0197)rplP (PRK09203)→
env-208	CESN01179177.1	-	635	567	**RNA**→ RplB (COG0090)rplB (PRK09374)→

env-209	CESP01157305.1	-	3522	3454	**RNA**→ RplB (COG0090)rplB (PRK09374)→ rpsS (PRK00357)RpsS (COG0185)→ rplV (PRK00565)Ribosomal_L22 (pfam00237)→ RpsC (COG0092)rpsC (PRK00310)→ RplP (COG0197)rplP (PRK09203)→
env-210	CEVU01049360.1	+	90	158	**RNA**→ RplB (COG0090)rplB (PRK09374)→ rpsS (PRK00357)RpsS (COG0185)→ rplV (PRK00565)Ribosomal_L22 (pfam00237)→ RpsC (COG0092)rpsC (PRK00310)→ RplP (COG0197)rplP (PRK09203)→
env-211	CEVX01093179.1	+	444	512	**RNA**→
env-212	CEWK01264123.1	+	1805	1873	**RNA**→ ←hypo
env-213	CENP01218092.1	-	190	122	**RNA**→ ←hypo
env-214	CEVP01021979.1	+	1007	1075	**RNA**→ ←hypo
env-215	JCVI_READ_1103242821391	+	471	539	**RNA**→ RplB (COG0090)rplB (PRK09374)→
env-216	JCVI_SCAF_1101668667046	+	950	1018	**RNA**→ rplB (PRK09374)Ribosomal_L2 (pfam00181)→
env-217	CETT01105554.1	+	279	347	**RNA**→ rplB (PRK09374)Ribosomal_L2 (pfam00181)→
env-218	CEUU01047001.1	+	1170	1238	**RNA**→ ←hypo
env-219	CEPA01032629.1	-	488	420	**RNA**→ rplB (PRK09374)Ribosomal_L2 (pfam00181)→
env-220	JGI24820J26691_1001055	-	5955	5887	**RNA**→ RplB (COG0090)rplB (PRK09374)→ rpsS (PRK00357)RpsS (COG0185)→ rplV (PRK00565)Ribosomal_L22 (pfam00237)→ RpsC (COG0092)rpsC (PRK00310)→ RplP (COG0197)rplP (PRK09203)→
env-221	CEVE01064005.1	+	149	217	**RNA**→ RplB (COG0090)rplB (PRK09374)→ rpsS (PRK00357)RpsS (COG0185)→ rplV (PRK00565)Ribosomal_L22 (pfam00237)→ rpsC (PRK00310)30S_S3_KH (cd02412)→
env-222	JCVI_SCAF_1096627794593	-	1348	1280	**RNA**→ rplB (PRK09374)Ribosomal_L2 (pfam00181)→
env-223	JCVI_SCAF_1096627334841	+	695	763	**RNA**→ RplB (COG0090)rplB (PRK09374)→ rpsS (PRK00357)RpsS (COG0185)→ rplV (PRK00565)Ribosomal_L22 (pfam00237)→
env-224	JCVI_SCAF_1101668673697	+	999	1067	**RNA**→ rplB (PRK09374)Ribosomal_L2 (pfam00181)→
env-225	CEUQ01027845.1	-	417	349	**RNA**→ rplB (PRK09374)Ribosomal_L2 (pfam00181)→
env-226	CEUK01022496.1	+	194	262	**RNA**→ rplB (PRK09374)Ribosomal_L2 (pfam00181)→
env-227	JCVI_SCAF_1101668339934	-	213	145	**RNA**→
env-228	2236876001_088379	+	363	434	**RNA**→ RplB (COG0090)rplB (PRK09374)→
env-229	JCVI_READ_1103769037343	+	211	279	**RNA**→ RplB (COG0090)rplB (PRK09374)→
env-230	JCVI_READ_1103769614257	+	956	1021	**RNA**→ ←hypo
env-231	CEWK01092565.1	+	421	489	**RNA**→
env-232	CENQ01040580.1	+	79	147	**RNA**→ rplB (PRK09374)Ribosomal_L2 (pfam00181)→
env-233	CEVN01064966.1	-	373	305	**RNA**→ rplB (PRK09374)Ribosomal_L2 (pfam00181)→
env-234	CEPL01019978.1	+	61	129	**RNA**→ hypo→ rplB (PRK09374)Ribosomal_L2 (pfam00181)→
env-235	CEPU01001285.1	+	124	192	**RNA**→ RplB (COG0090)rplB (PRK09374)→
env-236	JCVI_READ_1103769096640	-	624	556	**RNA**→ RplB (COG0090)rplB (PRK09374)→
env-237	CENO01039587.1	-	4337	4269	**RNA**→ RplB (COG0090)rplB (PRK09374)→ rpsS (PRK00357)RpsS (COG0185)→ rplV (PRK00565)Ribosomal_L22 (pfam00237)→ RpsC (COG0092)rpsC (PRK00310)→ RplP (COG0197)rplP (PRK09203)→
env-238	CENW01212150.1	-	201	133	**RNA**→
env-239	CEOJ01093429.1	-	167	99	**RNA**→
env-240	CEOS01129456.1	+	419	487	**RNA**→ RplB (COG0090)rplB (PRK09374)→
env-241	CEOV01114312.1	-	112	44	**RNA**→
env-242	CEOW01046858.1	+	2221	2289	**RNA**→ RplB (COG0090)rplB (PRK09374)→ rpsS (PRK00357)RpsS (COG0185)→ rplV (PRK00565)Ribosomal_L22 (pfam00237)→
env-243	CEPB01039107.1	+	1192	1260	**RNA**→ RplB (COG0090)rplB (PRK09374)→ rpsS (PRK00357)RpsS (COG0185)→ rplV (PRK00565)Ribosomal_L22 (pfam00237)→ RpsC (COG0092)rpsC (PRK00310)→ RplP (COG0197)rplP (PRK09203)→
env-244	CEPC01014332.1	+	2318	2386	**RNA**→ RplB (COG0090)rplB (PRK09374)→ rpsS (PRK00357)RpsS (COG0185)→
env-245	CEVG01269443.1	+	380	448	**RNA**→ rplB (PRK09374)Ribosomal_L2 (pfam00181)→
env-246	CEVY01099477.1	+	2626	2694	**RNA**→ RplB (COG0090)rplB (PRK09374)→ rpsS (PRK00357)RpsS (COG0185)→ rplV (PRK00565)Ribosomal_L22 (pfam00237)→ RpsC (COG0092)rpsC (PRK00310)→ RplP (COG0197)rplP (PRK09203)→
env-247	CEQD01048550.1	-	1102	1034	**RNA**→ RplB (COG0090)rplB (PRK09374)→ rpsS (PRK00357)RpsS (COG0185)→

env-248	CEOC01035558.1	+	378	446	**RNA**→ rplB (PRK09374)Ribosomal_L2 (pfam00181)→
env-249	CEQM01116065.1	+	207	275	**RNA**→ rplB (PRK09374)Ribosomal_L2 (pfam00181)→
env-250	CEWA01125852.1	-	329	261	**RNA**→ rplB (PRK09374)Ribosomal_L2 (pfam00181)→
env-251	CEWO01164779.1	-	401	333	**RNA**→ rplB (PRK09374)Ribosomal_L2 (pfam00181)→
env-252	CENG01071547.1	-	1142	1074	**RNA**→ RplB (COG0090)rplB (PRK09374)→ rpsS (PRK00357)Ribosomal_S19 (pfam00203)→
env-253	CENJ01189867.1	-	96	28	**RNA**→
env-254	CENU01109952.1	+	428	496	**RNA**→ rplB (PRK09374)Ribosomal_L2 (pfam00181)→
env-255	CEVW01291667.1	-	97	29	**RNA**→
env-256	CAM_READ_0234711973	+	389	459	**RNA**→ ←hypo
env-257	JCVI_SCAF_1096626302357	-	446	378	**RNA**→ rplB (PRK09374)Ribosomal_L2 (pfam00181)→
env-258	CESA01208526.1	+	115	183	**RNA**→ RplB (COG0090)rplB (PRK09374)→
env-259	JGI24723J26617_10000490	-	34468	34404	**RNA**→ RplB (COG0090)rplB (PRK09374)→ rpsS (PRK00357)RpsS (COG0185)→<br>rplV (PRK00565)Ribosomal_L22 (pfam00237)→ RpsC (COG0092)rpsC (PRK00310)→<br>RplP (COG0197)rplP (PRK09203)→
Rsp-8-1	NZ_ABCR01000011.1	+	18945	19019	**RNA**→ RplB (COG0090)rplB (PRK09374)→ RplB (COG0090)rplB (PRK09374)→<br>rpsS (PRK00357)RpsS (COG0185)→ rpsS (PRK00357)RpsS (COG0185)→<br>rplV (PRK00565)Ribosomal_L22 (pfam00237)→ rplV (PRK00565)Ribosomal_L22 (pfam00237)→<br>RpsC (COG0092)rpsC (PRK00310)→ RpsC (COG0092)rpsC (PRK00310)→ RplP (COG0197)rplP (PRK09203)→<br>RplP (COG0197)rplP (PRK09203)→
env-260	CENP01298067.1	-	497	434	**RNA**→ rplB (PRK09374)Ribosomal_L2 (pfam00181)→
env-261	SI34jun09_10m_1176238	-	203	140	**RNA**→ RplB (COG0090)rplB (PRK09374)→
env-262	CESO01033516.1	-	891	828	**RNA**→ RplB (COG0090)rplB (PRK09374)→
env-263	2236876002_126045	-	189	126	**RNA**→ RplB (COG0090)rplB (PRK09374)→
env-264	JGI20151J14362_10250213	-	362	299	**RNA**→ rplB (PRK09374)Ribosomal_L2 (pfam00181)→
env-265	CENZ01260479.1	+	344	407	**RNA**→ RplB (COG0090)rplB (PRK09374)→
env-266	2236876002_106467	-	167	104	**RNA**→ RplB (COG0090)rplB (PRK09374)→
env-267	CENT01014152.1	+	496	559	**RNA**→ RplB (COG0090)rplB (PRK09374)→ rpsS (PRK00357)RpsS (COG0185)→<br>rplV (PRK00565)Ribosomal_L22 (pfam00237)→ RpsC (COG0092)rpsC (PRK00310)→<br>RplP (COG0197)rplP (PRK09203)→
env-268	CERM01014689.1	-	2838	2775	**RNA**→ RplB (COG0090)rplB (PRK09374)→ rpsS (PRK00357)RpsS (COG0185)→<br>rplV (PRK00565)Ribosomal_L22 (pfam00237)→ RpsC (COG0092)rpsC (PRK00310)→<br>RplP (COG0197)rplP (PRK09203)→
env-269	CENN01049021.1	+	1200	1263	**RNA**→ rplB (PRK09374)Ribosomal_L2 (pfam00181)→
env-270	CESS01003967.1	-	3048	2985	**RNA**→ RplB (COG0090)rplB (PRK09374)→ rpsS (PRK00357)RpsS (COG0185)→<br>rplV (PRK00565)Ribosomal_L22 (pfam00237)→ RpsC (COG0092)rpsC (PRK00310)→<br>RplP (COG0197)rplP (PRK09203)→
env-271	JGI20158J14315_10236147	-	342	279	**RNA**→ rplB (PRK09374)Ribosomal_L2 (pfam00181)→
env-272	DelMOWin2010_c10431137	+	54	117	**RNA**→ RplB (COG0090)rplB (PRK09374)→
env-273	JCVI_SCAF_1096628356459	-	1073	1010	**RNA**→ rpsS (PRK00357)RpsS (COG0185)→ rplV (PRK00565)Ribosomal_L22 (pfam00237)→
env-274	JCVI_READ_1103769101289	-	925	866	**RNA**→ RplB (COG0090)rplB (PRK09374)→
env-275	JCVI_SCAF_1096627271074	+	1225	1289	**RNA**→ RplB (COG0090)rplB (PRK09374)→ rpsS (PRK00357)RpsS (COG0185)→
env-276	JCVI_READ_1108799738541	-	650	586	**RNA**→ RplB (COG0090)rplB (PRK09374)→
env-277	JCVI_SCAF_1096627976978	+	67	130	**RNA**→ RplB (COG0090)rplB (PRK09374)→
env-278	CEWK01197169.1	-	120	57	**RNA**→
env-279	CESM01101386.1	-	277	213	**RNA**→ ←hypo
env-280	CEUH01138205.1	+	837	901	**RNA**→
env-281	CEWG01043024.1	+	1807	1871	**RNA**→ RplB (COG0090)rplB (PRK09374)→
env-282	CENF01097607.1	+	586	650	**RNA**→
env-283	CEUG01060018.1	-	123	59	**RNA**→
env-284	CEQW01039727.1	-	82	18	**RNA**→
env-285	JGI24820J26691_1324391	+	37	101	**RNA**→ ←hypo
env-286	CESM01340684.1	-	97	33	**RNA**→
env-287	JCVI_SCAF_1096627811062	-	972	908	**RNA**→ RplB (COG0090)rplB (PRK09374)→
env-288	JGI24820J26691_1087074	+	510	574	**RNA**→ hypo→

env-289	JCVI_SCAF_1096627081604	-	1775	1711	**RNA**→ RplB (COG0090)rplB (PRK09374)→ rpsS (PRK00357)RpsS (COG0185)→ rplV (PRK00565)Ribosomal_L22 (pfam00237)→ rpsC (PRK00310)30S_S3_KH (cd02412)→
env-290	JCVI_SCAF_1096627977260	+	343	407	**RNA**→ rplB (PRK09374)Ribosomal_L2 (pfam00181)→
env-291	CEPJ01112005.1	+	720	784	**RNA**→ rplB (PRK09374)Ribosomal_L2 (pfam00181)→
env-292	CAM_READ_0232770725	-	286	222	**RNA**→ RplB (COG0090)rplB (PRK09374)→
env-293	CAM_READ_0232288387	-	248	184	**RNA**→ hypo→
env-294	JCVI_READ_1108800192449	+	288	352	**RNA**→ rplB (PRK09374)Ribosomal_L2 (pfam00181)→
env-295	JCVI_SCAF_1096627092934	+	576	640	**RNA**→ RplB (COG0090)rplB (PRK09374)→
env-296	JCVI_SCAF_1096627099260	+	352	416	**RNA**→ RplB (COG0090)rplB (PRK09374)→ rpsS (PRK00357)RpsS (COG0185)→ rplV (PRK00565)Ribosomal_L22 (pfam00237)→ rpsC (PRK00310)30S_S3_KH (cd02412)→
env-297	JGI24820J26691_1028973	+	1055	1119	**RNA**→
env-298	CEUV01003215.1	+	768	832	**RNA**→
env-299	CEUQ01156669.1	-	85	21	**RNA**→
env-300	JCVI_SCAF_1101667251435	-	166	102	**RNA**→ hypo→
env-301	CENF01068770.1	-	88	24	**RNA**→
env-302	CEOM01237289.1	+	465	529	**RNA**→
env-303	CERW01142606.1	+	975	1039	**RNA**→
env-304	CEVS01036929.1	-	88	24	**RNA**→
env-305	CEVU01131676.1	+	1938	2002	**RNA**→
env-306	CEVX01017747.1	-	2836	2772	**RNA**→ RplB (COG0090)rplB (PRK09374)→ rpsS (PRK00357)RpsS (COG0185)→ rplV (PRK00565)Ribosomal_L22 (pfam00237)→ RpsC (COG0092)rpsC (PRK00310)→ RplP (COG0197)rplP (PRK09203)→
env-307	CEWO01083412.1	+	3433	3497	**RNA**→ RplB (COG0090)rplB (PRK09374)→
env-308	JCVI_SCAF_1096627863534	-	717	653	**RNA**→ RplB (COG0090)rplB (PRK09374)→
env-309	JCVI_SCAF_1101668429250	+	407	471	**RNA**→ RplB (COG0090)rplB (PRK09374)→
env-310	JCVI_SCAF_1101668671537	-	1477	1413	**RNA**→ rplB (PRK09374)Ribosomal_L2 (pfam00181)→ rplB (PRK09374)Ribosomal_L2_C (pfam03947)→ rpsS (PRK00357)RpsS (COG0185)→ rplV (PRK00565)Ribosomal_L22 (pfam00237)→
env-311	CETT01040907.1	-	3530	3466	**RNA**→ RplB (COG0090)rplB (PRK09374)→ rpsS (PRK00357)RpsS (COG0185)→ rplV (PRK00565)Ribosomal_L22 (pfam00237)→ RpsC (COG0092)rpsC (PRK00310)→ RplP (COG0197)rplP (PRK09203)→
env-312	JCVI_READ_1103242680220	-	329	265	**RNA**→ RplB (COG0090)rplB (PRK09374)→
env-313	JCVI_SCAF_1101668723498	+	490	554	**RNA**→ RplB (COG0090)rplB (PRK09374)→
env-314	JCVI_READ_1108800250675	-	813	749	**RNA**→ RplB (COG0090)rplB (PRK09374)→
env-315	JCVI_SCAF_1101667072466	-	232	168	**RNA**→
env-316	JCVI_READ_1104230004824	+	69	133	**RNA**→ RplB (COG0090)rplB (PRK09374)→
env-317	CEPU01102359.1	-	315	251	**RNA**→ RplB (COG0090)rplB (PRK09374)→
env-318	JCVI_READ_1104230151507	-	139	75	**RNA**→
env-319	JCVI_SCAF_1096626924486	-	857	793	**RNA**→ RplB (COG0090)rplB (PRK09374)→
env-320	CEQD01002240.1	+	207	271	**RNA**→ RplB (COG0090)rplB (PRK09374)→
env-321	JCVI_SCAF_1101668260309	-	1155	1091	**RNA**→ RplB (COG0090)rplB (PRK09374)→ rpsS (PRK00357)RpsS (COG0185)→
env-322	CEWK01132454.1	+	1102	1166	**RNA**→ RplB (COG0090)rplB (PRK09374)→ rpsS (PRK00357)RpsS (COG0185)→ rplV (PRK00565)Ribosomal_L22 (pfam00237)→ RpsC (COG0092)rpsC (PRK00310)→ RplP (COG0197)rplP (PRK09203)→
env-323	CETB01173647.1	+	105	169	**RNA**→ rplB (PRK09374)Ribosomal_L2 (pfam00181)→
env-324	CENY01053602.1	+	393	457	**RNA**→ rplB (PRK09374)Ribosomal_L2 (pfam00181)→
env-325	JCVI_READ_1108799747928	+	140	204	**RNA**→ RplB (COG0090)rplB (PRK09374)→
env-326	JCVI_SCAF_1096627704005	-	1325	1261	**RNA**→ RplB (COG0090)rplB (PRK09374)→ RplB (COG0090)rplB (PRK09374)→ rpsS (PRK00357)RpsS (COG0185)→
env-327	JCVI_SCAF_1101668170846	-	710	646	**RNA**→ RplB (COG0090)rplB (PRK09374)→
env-328	CEVY01136175.1	+	444	508	**RNA**→
env-329	CAM_READ_0072915149	+	365	429	**RNA**→
env-330	JCVI_READ_1103180852614	-	134	70	**RNA**→
env-331	CETS01117025.1	+	341	405	**RNA**→
env-332	CEUJ01061937.1	-	283	219	**RNA**→ RplB (COG0090)rplB (PRK09374)→

env-333	CEVE01027800.1	-	84	20	**RNA**→
env-334	CEWJ01004911.1	-	89	25	**RNA**→
env-335	CEWH01205319.1	+	544	608	**RNA**→
env-336	JCVI_SCAF_1096627835679	-	1455	1391	**RNA**→ RplB (COG0090)rplB (PRK09374)→ RplB (COG0090)rplB (PRK09374)→<br>rpsS (PRK00357)RpsS (COG0185)→ rplV (PRK00565)Ribosomal_L22 (pfam00237)→
env-337	JCVI_SCAF_1096627978139	-	713	649	**RNA**→ RplB (COG0090)rplB (PRK09374)→ rpsS (PRK00357)RpsS (COG0185)→
env-338	JCVI_SCAF_1096628267383	+	720	784	**RNA**→ rplV (PRK00565)Ribosomal_L22 (pfam00237)→ RpsC (COG0092)rpsC (PRK00310)→
env-339	JCVI_SCAF_1096627226171	-	356	292	**RNA**→ rplB (PRK09374)Ribosomal_L2 (pfam00181)→
env-340	CESB01055415.1	+	1248	1312	**RNA**→ RplB (COG0090)rplB (PRK09374)→
env-341	JCVI_SCAF_1101668333168	+	132	196	**RNA**→ RplB (COG0090)rplB (PRK09374)→ rpsS (PRK00357)RpsS (COG0185)→<br>rplV (PRK00565)Ribosomal_L22 (pfam00237)→
env-342	CEVZ01074731.1	+	606	670	**RNA**→
env-343	JCVI_READ_1108829670529	-	825	762	**RNA**→ RplB (COG0090)rplB (PRK09374)→
env-344	JCVI_SCAF_1101668663947	-	95	31	**RNA**→
env-345	JCVI_READ_1103242633429	+	739	803	**RNA**→
env-346	JCVI_SCAF_1101668342543	-	593	529	**RNA**→ RplB (COG0090)rplB (PRK09374)→
env-347	CENF01127799.1	+	676	740	**RNA**→
env-348	CEQO01040664.1	-	501	437	**RNA**→ rplB (PRK09374)Ribosomal_L2 (pfam00181)→
env-349	CEQW01046424.1	+	1679	1743	**RNA**→ ←hypo
env-350	CEQY01100769.1	-	387	323	**RNA**→ rplB (PRK09374)Ribosomal_L2 (pfam00181)→
env-351	CERR01085567.1	+	474	538	**RNA**→
env-352	CETI01093028.1	-	91	27	**RNA**→
env-353	CEVU01103598.1	-	85	21	**RNA**→
env-354	CEVX01107346.1	+	461	525	**RNA**→
env-355	CEWB01023124.1	+	120	184	**RNA**→ rplB (PRK09374)Ribosomal_L2 (pfam00181)→
env-356	JCVI_SCAF_1096627339121	-	1737	1673	**RNA**→ RplB (COG0090)rplB (PRK09374)→ rpsS (PRK00357)RpsS (COG0185)→<br>rplV (PRK00565)Ribosomal_L22 (pfam00237)→ rpsC (PRK00310)30S_S3_KH (cd02412)→
env-357	JCVI_SCAF_1101667362029	+	16	80	**RNA**→ rplB (PRK09374)Ribosomal_L2 (pfam00181)→
env-358	CETP01093824.1	+	77	141	**RNA**→ RplB (COG0090)rplB (PRK09374)→
env-359	CEUU01096110.1	-	95	31	**RNA**→
env-360	JCVI_SCAF_1101668661533	-	618	555	**RNA**→ RplB (COG0090)rplB (PRK09374)→
env-361	JCVI_SCAF_1101668691223	-	229	166	**RNA**→ RplB (COG0090)rplB (PRK09374)→
Psp-3-1	NZ_LDPY01000001.1	+	1628103	1628171	**RNA**→ RplB (COG0090)rplB (PRK09374)→ rpsS (PRK00357)RpsS (COG0185)→<br>rplV (PRK00565)Ribosomal_L22 (pfam00237)→ RpsC (COG0092)rpsC (PRK00310)→<br>RplP (COG0197)rplP (PRK09203)→
Hmi-1-1	NZ_JFGS01000025.1	-	34255	34184	**RNA**→ RplB (COG0090)rplB (PRK09374)→ rpsS (PRK00357)RpsS (COG0185)→<br>rplV (PRK00565)Ribosomal_L22 (pfam00237)→ RpsC (COG0092)rpsC (PRK00310)→<br>RplP (COG0197)rplP (PRK09203)→ MhpC (COG0596)Abhydrolase_6 (pfam12697)→<br>MhpC (COG0596)Abhydrolase_6 (pfam12697)→
Hma-1-1	NZ_JGYG01000018.1	-	57450	57378	**RNA**→ RplB (COG0090)rplB (PRK09374)→ rpsS (PRK00357)RpsS (COG0185)→<br>rplV (PRK00565)Ribosomal_L22 (pfam00237)→ RpsC (COG0092)rpsC (PRK00310)→<br>RplP (COG0197)rplP (PRK09203)→ MhpC (COG0596)Abhydrolase_6 (pfam12697)→
Laq-1-1	NZ_KI421504.1	+	1151633	1151741	**RNA**→ RplB (COG0090)rplB (PRK09374)→ rpsS (PRK00357)RpsS (COG0185)→<br>rplV (PRK00565)Ribosomal_L22 (pfam00237)→ RpsC (COG0092)rpsC (PRK00310)→<br>RplP (COG0197)rplP (PRK09203)→
env-362	JGI24723J26617_10003411	-	7982	7888	**RNA**→ RplB (COG0090)rplB (PRK09374)→ rpsS (PRK00357)RpsS (COG0185)→<br>rplV (PRK00565)Ribosomal_L22 (pfam00237)→ RpsC (COG0092)rpsC (PRK00310)→<br>RplP (COG0197)rplP (PRK09203)→
Rpo-1-1	NC_003911.12	+	487501	487565	**RNA**→ RplB (COG0090)rplB (PRK09374)→ rpsS (PRK00357)RpsS (COG0185)→<br>rplV (PRK00565)Ribosomal_L22 (pfam00237)→ RpsC (COG0092)rpsC (PRK00310)→<br>RplP (COG0197)rplP (PRK09203)→
env-363	CETY01001211.1	-	2221	2155	**RNA**→ RplB (COG0090)rplB (PRK09374)→ rpsS (PRK00357)RpsS (COG0185)→<br>rplV (PRK00565)Ribosomal_L22 (pfam00237)→ RpsC (COG0092)rpsC (PRK00310)→
env-364	JGI24023J19991_10525132	-	195	105	**RNA**→ RplB (COG0090)rplB (PRK09374)→

Sla-1-1	NZ_GG704596.1	+	1508433	1508499	**RNA**→ RplB (COG0090)rplB (PRK09374)→ RplB (COG0090)rplB (PRK09374)→ rpsS (PRK00357)RpsS (COG0185)→ rpsS (PRK00357)RpsS (COG0185)→ rplV (PRK00565)Ribosomal_L22 (pfam00237)→ rplV (PRK00565)Ribosomal_L22 (pfam00237)→ RpsC (COG0092)rpsC (PRK00310)→ RpsC (COG0092)rpsC (PRK00310)→ RplP (COG0197)rplP (PRK09203)→ RplP (COG0197)rplP (PRK09203)→
Rha-2-1	NZ_JQEZ01000002.1	-	890631	890567	**RNA**→ RplB (COG0090)rplB (PRK09374)→ rpsS (PRK00357)RpsS (COG0185)→ rplV (PRK00565)Ribosomal_L22 (pfam00237)→ RpsC (COG0092)rpsC (PRK00310)→ RplP (COG0197)rplP (PRK09203)→
Rsp-17-1	NZ_LGXZ01000018.1	-	15524	15460	**RNA**→ RplB (COG0090)rplB (PRK09374)→ rpsS (PRK00357)RpsS (COG0185)→ rplV (PRK00565)Ribosomal_L22 (pfam00237)→ RpsC (COG0092)rpsC (PRK00310)→ RplP (COG0197)rplP (PRK09203)→
Rsp-18-1	NZ_JWLJ01000003.1	+	9098	9163	**RNA**→ RplB (COG0090)rplB (PRK09374)→ rpsS (PRK00357)RpsS (COG0185)→ rplV (PRK00565)Ribosomal_L22 (pfam00237)→ RpsC (COG0092)rpsC (PRK00310)→ RplP (COG0197)rplP (PRK09203)→
Rsp-19-1	NZ_JWLK01000001.1	+	450619	450684	**RNA**→ RplB (COG0090)rplB (PRK09374)→ rpsS (PRK00357)RpsS (COG0185)→ rplV (PRK00565)Ribosomal_L22 (pfam00237)→ RpsC (COG0092)rpsC (PRK00310)→ RplP (COG0197)rplP (PRK09203)→
Rsp-22-1	NZ_AEYW01000001.1	+	6172	6237	**RNA**→ RplB (COG0090)rplB (PRK09374)→ RplB (COG0090)rplB (PRK09374)→ rpsS (PRK00357)RpsS (COG0185)→ rpsS (PRK00357)RpsS (COG0185)→ rplV (PRK00565)Ribosomal_L22 (pfam00237)→ rplV (PRK00565)Ribosomal_L22 (pfam00237)→ RpsC (COG0092)rpsC (PRK00310)→ RpsC (COG0092)rpsC (PRK00310)→ RplP (COG0197)rplP (PRK09203)→ RplP (COG0197)rplP (PRK09203)→
Rba-2-1	NZ_DS999531.1	+	702885	702950	**RNA**→ RplB (COG0090)rplB (PRK09374)→ RplB (COG0090)rplB (PRK09374)→ rpsS (PRK00357)RpsS (COG0185)→ rpsS (PRK00357)RpsS (COG0185)→ rplV (PRK00565)Ribosomal_L22 (pfam00237)→ rplV (PRK00565)Ribosomal_L22 (pfam00237)→ RpsC (COG0092)rpsC (PRK00310)→ RpsC (COG0092)rpsC (PRK00310)→ RplP (COG0197)rplP (PRK09203)→ RplP (COG0197)rplP (PRK09203)→
env-365	KGI_S1_ANT01_95m_c10129328	+	275	337	**RNA**→ rplB (PRK09374)Ribosomal_L2 (pfam00181)→
env-366	KGI_S1_ANT02_95m_c10007513	+	664	726	**RNA**→ RplB (COG0090)rplB (PRK09374)→ rpsS (PRK00357)RpsS (COG0185)→ rplV (PRK00565)Ribosomal_L22 (pfam00237)→ RpsC (COG0092)rpsC (PRK00310)→ RplP (COG0197)rplP (PRK09203)→ hypo→ M20_Acy1 (cd03886)amidohydrolases (TIGR01891)→
env-367	KGI_S2_ANT04_2345m_c1167548	+	316	378	**RNA**→ ←hypo
env-368	JGI24723J26617_10802083	+	118	180	**RNA**→ rplW (PRK05738)→
env-369	JGI12109J20283_10872926	-	175	77	**RNA**→ hypo→
env-370	JGI12109J20283_10006735	-	753	682	**RNA**→ RplB (COG0090)rplB (PRK09374)→
env-371	JGI12109J20283_10006736	+	78	149	**RNA**→ rplB (PRK09374)Ribosomal_L2 (pfam00181)→
env-372	HLSNC_1001521	-	9879	9807	**RNA**→ RplB (COG0090)rplB (PRK09374)→ rpsS (PRK00357)RpsS (COG0185)→ rplV (PRK00565)Ribosomal_L22 (pfam00237)→ RpsC (COG0092)rpsC (PRK00310)→ RplP (COG0197)rplP (PRK09203)→ QdoI (COG1917)DMSP_lyase (pfam16867)→ 2OG-FeII_Oxy_5 (pfam13759)→
env-373	KGI_S2_ANT06_2345m_c1009878	-	2821	2753	**RNA**→ RplB (COG0090)rplB (PRK09374)→ rpsS (PRK00357)RpsS (COG0185)→ rplV (PRK00565)Ribosomal_L22 (pfam00237)→ RpsC (COG0092)rpsC (PRK00310)→ RplP (COG0197)rplP (PRK09203)→
env-374	KGI_S2_ANT04_2345m_c1010869	-	2778	2711	**RNA**→ RplB (COG0090)rplB (PRK09374)→ rpsS (PRK00357)RpsS (COG0185)→ rplV (PRK00565)Ribosomal_L22 (pfam00237)→ RpsC (COG0092)rpsC (PRK00310)→ RplP (COG0197)rplP (PRK09203)→
env-375	JGI20153J14295_10082023	-	314	252	**RNA**→ RplB (COG0090)rplB (PRK09374)→
env-376	JGI20153J14318_10082023	-	314	252	**RNA**→ RplB (COG0090)rplB (PRK09374)→
env-377	JGI20159J14440_10153261	+	97	159	**RNA**→ RplB (COG0090)rplB (PRK09374)→
env-378	AMFJ01022780.1	-	134	67	**RNA**→ RplB (COG0090)rplB (PRK09374)→
env-379	ACD54_3	-	1770	1703	**RNA**→ RplB (COG0090)rplB (PRK09374)→ rpsS (PRK00357)RpsS (COG0185)→ rplV (PRK00565)Ribosomal_L22 (pfam00237)→ rpsC (PRK00310)30S_S3_KH (cd02412)→
env-380	CESJ01069642.1	-	121	57	**RNA**→
env-381	JGI12109J20283_10580390	-	247	150	**RNA**→ RplB (COG0090)rplB (PRK09374)→
Osp-2-1	NZ_JQMY01000001.1	+	973809	973875	**RNA**→ RplB (COG0090)rplB (PRK09374)→ rpsS (PRK00357)RpsS (COG0185)→ rplV (PRK00565)Ribosomal_L22 (pfam00237)→ RpsC (COG0092)rpsC (PRK00310)→ RplP (COG0197)rplP (PRK09203)→

env-382	JGI12396J12026_1059309	+	985	1055	**RNA**→ RplB (COG0090)rplB (PRK09374)→
env-383	JGI12396J12026_1059311	-	316	246	**RNA**→ RplB (COG0090)rplB (PRK09374)→
env-384	JGI12467J12023_1025468	+	360	430	**RNA**→ RplB (COG0090)rplB (PRK09374)→
env-385	JGI12273J12029_10120939	+	462	532	**RNA**→ RplB (COG0090)rplB (PRK09374)→
env-386	JGI12467J12023_1167671	+	157	226	**RNA**→ RplB (COG0090)rplB (PRK09374)→
env-387	JGI12273J12029_10075303	+	308	377	**RNA**→ RplB (COG0090)rplB (PRK09374)→
env-388	JGI12273J12029_10163269	+	383	452	**RNA**→
Rme-1-1	NZ_KK088521.1	-	243248	243181	**RNA**→ hypo→ hypo→ rpsS (PRK00357)RpsS (COG0185)→ rplV (PRK00565)Ribosomal_L22 (pfam00237)→<br>RpsC (COG0092)rpsC (PRK00310)→ RplP (COG0197)rplP (PRK09203)→<br>PRK00306 (PRK00306)Ribosomal_L29 (pfam00831)→ rpsQ (PRK05610)Ribosomal_S17 (pfam00366)→<br>rplN (PRK05483)Ribosomal_L14 (pfam00238)→ RplX (COG0198)rplX (PRK00004)→<br>rplE (PRK00010)RplE (COG0094)→ rpsN (PRK08881)Ribosomal_S14 (pfam00253)→<br>rpsH (PRK00136)Ribosomal_S8 (pfam00410)→ RplF (COG0097)rplF (PRK05498)→ hypo→<br>rplR (PRK05593)Ribosomal_L18p (pfam00861)→
env-389	JGI12467J12023_1134564	+	240	309	**RNA**→
env-390	JGI12273J12029_10074910	+	717	786	**RNA**→ RplB (COG0090)rplB (PRK09374)→
env-391	JGI12273J12029_10159171	+	74	143	**RNA**→ RplB (COG0090)rplB (PRK09374)→
env-392	CESO01257586.1	+	654	720	**RNA**→
env-393	CEVX01283897.1	-	753	687	**RNA**→ RplB (COG0090)rplB (PRK09374)→
env-394	CESM01223547.1	+	62	128	**RNA**→ RplB (COG0090)rplB (PRK09374)→ rpsS (PRK00357)Ribosomal_S19 (pfam00203)→
env-395	JGI26524J50256_1001720	+	3021	3111	**RNA**→ RplB (COG0090)rplB (PRK09374)→ rpsS (PRK00357)RpsS (COG0185)→<br>rplV (PRK00565)Ribosomal_L22 (pfam00237)→ RpsC (COG0092)rpsC (PRK00310)→<br>RplP (COG0197)rplP (PRK09203)→
env-396	3300001592_10003260	+	2614	2692	**RNA**→ RplB (COG0090)rplB (PRK09374)→ rpsS (PRK00357)RpsS (COG0185)→<br>rplV (PRK00565)Ribosomal_L22 (pfam00237)→ RpsC (COG0092)rpsC (PRK00310)→<br>RplP (COG0197)rplP (PRK09203)→
Pxi-1-1	NZ_AUBS01000014.1	+	30482	30560	**RNA**→ RplB (COG0090)rplB (PRK09374)→ rpsS (PRK00357)RpsS (COG0185)→<br>rplV (PRK00565)Ribosomal_L22 (pfam00237)→ RpsC (COG0092)rpsC (PRK00310)→<br>RplP (COG0197)rplP (PRK09203)→
Rmo-1-1	NZ_JXYG01000033.1	-	76643	76542	**RNA**→ RplB (COG0090)rplB (PRK09374)→ rpsS (PRK00357)RpsS (COG0185)→<br>rplV (PRK00565)Ribosomal_L22 (pfam00237)→ RpsC (COG0092)rpsC (PRK00310)→<br>RplP (COG0197)rplP (PRK09203)→
Rmo-2-1	NZ_AQCH01000135.1	-	22727	22626	**RNA**→ RplB (COG0090)rplB (PRK09374)→ rpsS (PRK00357)RpsS (COG0185)→<br>rplV (PRK00565)Ribosomal_L22 (pfam00237)→ RpsC (COG0092)rpsC (PRK00310)→<br>RplP (COG0197)rplP (PRK09203)→
Rsp-21-1	NC_008044.1	+	268831	268932	**RNA**→ RplB (COG0090)rplB (PRK09374)→ rpsS (PRK00357)RpsS (COG0185)→<br>rplV (PRK00565)Ribosomal_L22 (pfam00237)→ RpsC (COG0092)rpsC (PRK00310)→<br>RplP (COG0197)rplP (PRK09203)→
Ssp-1-1	NZ_GG703517.1	+	54267	54368	**RNA**→ RplB (COG0090)rplB (PRK09374)→ RplB (COG0090)rplB (PRK09374)→<br>rpsS (PRK00357)RpsS (COG0185)→ rpsS (PRK00357)RpsS (COG0185)→<br>rplV (PRK00565)Ribosomal_L22 (pfam00237)→ rplV (PRK00565)Ribosomal_L22 (pfam00237)→<br>RpsC (COG0092)rpsC (PRK00310)→ RpsC (COG0092)rpsC (PRK00310)→ RplP (COG0197)rplP (PRK09203)→<br>RplP (COG0197)rplP (PRK09203)→
Psp-1-1	NZ_JSWK01000016.1	-	260767	260659	**RNA**→ RplB (COG0090)rplB (PRK09374)→ rpsS (PRK00357)RpsS (COG0185)→<br>rplV (PRK00565)Ribosomal_L22 (pfam00237)→ RpsC (COG0092)rpsC (PRK00310)→<br>RplP (COG0197)rplP (PRK09203)→
Pga-1-1	NC_018286.1	-	3196082	3195974	**RNA**→ RplB (COG0090)rplB (PRK09374)→ rpsS (PRK00357)RpsS (COG0185)→<br>rplV (PRK00565)Ribosomal_L22 (pfam00237)→ RpsC (COG0092)rpsC (PRK00310)→<br>RplP (COG0197)rplP (PRK09203)→
Pin-1-1	NZ_KI421498.1	+	3089651	3089759	**RNA**→ RplB (COG0090)rplB (PRK09374)→ rpsS (PRK00357)RpsS (COG0185)→<br>rplV (PRK00565)Ribosomal_L22 (pfam00237)→ RpsC (COG0092)rpsC (PRK00310)→<br>RplP (COG0197)rplP (PRK09203)→
Psp-2-1	NZ_JSWJ01000014.1	-	97388	97280	**RNA**→ RplB (COG0090)rplB (PRK09374)→ rpsS (PRK00357)RpsS (COG0185)→<br>rplV (PRK00565)Ribosomal_L22 (pfam00237)→ RpsC (COG0092)rpsC (PRK00310)→<br>RplP (COG0197)rplP (PRK09203)→

Rsp-11-1	NZ_CH902583.1	+	2863415	2863523	**RNA**→ RplB (COG0090)rplB (PRK09374)→ rpsS (PRK00357)RpsS (COG0185)→ rplV (PRK00565)Ribosomal_L22 (pfam00237)→ RpsC (COG0092)rpsC (PRK00310)→ RplP (COG0197)rplP (PRK09203)→
Par-1-1	NZ_KI421507.1	+	92373	92482	**RNA**→ RplB (COG0090)rplB (PRK09374)→ rpsS (PRK00357)RpsS (COG0185)→ rplV (PRK00565)Ribosomal_L22 (pfam00237)→ RpsC (COG0092)rpsC (PRK00310)→ RplP (COG0197)rplP (PRK09203)→
env-397	CAM_READ_0074161329	-	292	213	**RNA**→ RplB (COG0090)rplB (PRK09374)→
env-398	JGI24025J20009_10459630	+	162	240	**RNA**→
Rsp-12-1	NZ_AAYC01000008.1	-	172899	172821	**RNA**→ RplB (COG0090)rplB (PRK09374)→ RplB (COG0090)rplB (PRK09374)→ rpsS (PRK00357)RpsS (COG0185)→ rpsS (PRK00357)RpsS (COG0185)→ rplV (PRK00565)Ribosomal_L22 (pfam00237)→ rplV (PRK00565)Ribosomal_L22 (pfam00237)→ RpsC (COG0092)rpsC (PRK00310)→ RpsC (COG0092)rpsC (PRK00310)→ RplP (COG0197)rplP (PRK09203)→ RplP (COG0197)rplP (PRK09203)→
Lsp-7-1	NZ_JWLH01000015.1	-	13431	13353	**RNA**→ RplB (COG0090)rplB (PRK09374)→ rpsS (PRK00357)RpsS (COG0185)→ rplV (PRK00565)Ribosomal_L22 (pfam00237)→ RpsC (COG0092)rpsC (PRK00310)→ RplP (COG0197)rplP (PRK09203)→
Lsp-1-1	NZ_JWLE01000039.1	-	14129	14051	**RNA**→ RplB (COG0090)rplB (PRK09374)→ rpsS (PRK00357)RpsS (COG0185)→ rplV (PRK00565)Ribosomal_L22 (pfam00237)→ RpsC (COG0092)rpsC (PRK00310)→ RplP (COG0197)rplP (PRK09203)→
Lsp-3-1	NZ_JWLG01000013.1	+	28657	28735	**RNA**→ RplB (COG0090)rplB (PRK09374)→ rpsS (PRK00357)RpsS (COG0185)→ rplV (PRK00565)Ribosomal_L22 (pfam00237)→ RpsC (COG0092)rpsC (PRK00310)→ RplP (COG0197)rplP (PRK09203)→
Lsp-4-1	NZ_JWLI01000007.1	-	341160	341082	**RNA**→ RplB (COG0090)rplB (PRK09374)→ rpsS (PRK00357)RpsS (COG0185)→ rplV (PRK00565)Ribosomal_L22 (pfam00237)→ RpsC (COG0092)rpsC (PRK00310)→ RplP (COG0197)rplP (PRK09203)→
Lsp-6-1	NZ_JWLF01000005.1	-	489868	489790	**RNA**→ RplB (COG0090)rplB (PRK09374)→ rpsS (PRK00357)RpsS (COG0185)→ rplV (PRK00565)Ribosomal_L22 (pfam00237)→ RpsC (COG0092)rpsC (PRK00310)→ RplP (COG0197)rplP (PRK09203)→
Lsp-9-1	NZ_AFCF02000006.1	+	28882	28960	**RNA**→ RplB (COG0090)rplB (PRK09374)→ rpsS (PRK00357)RpsS (COG0185)→ rplV (PRK00565)Ribosomal_L22 (pfam00237)→ RpsC (COG0092)rpsC (PRK00310)→ RplP (COG0197)rplP (PRK09203)→
Lsp-5-1	NZ_JWLM01000001.1	+	28726	28804	**RNA**→ RplB (COG0090)rplB (PRK09374)→ rpsS (PRK00357)RpsS (COG0185)→ rplV (PRK00565)Ribosomal_L22 (pfam00237)→ RpsC (COG0092)rpsC (PRK00310)→ RplP (COG0197)rplP (PRK09203)→
Pga-2-1	NC_018290.1	+	154778	154856	**RNA**→ RplB (COG0090)rplB (PRK09374)→ rpsS (PRK00357)RpsS (COG0185)→ rplV (PRK00565)Ribosomal_L22 (pfam00237)→ RpsC (COG0092)rpsC (PRK00310)→ RplP (COG0197)rplP (PRK09203)→
Nsp-1-1	NZ_LAUK01000016.1	-	42619	42541	**RNA**→ RplB (COG0090)rplB (PRK09374)→ rpsS (PRK00357)RpsS (COG0185)→ rplV (PRK00565)Ribosomal_L22 (pfam00237)→ RpsC (COG0092)rpsC (PRK00310)→ RplP (COG0197)rplP (PRK09203)→
Nit-1-1	NZ_CVRL01000015.1	-	39753	39675	**RNA**→ RplB (COG0090)rplB (PRK09374)→ rpsS (PRK00357)RpsS (COG0185)→ rplV (PRK00565)Ribosomal_L22 (pfam00237)→ RpsC (COG0092)rpsC (PRK00310)→ RplP (COG0197)rplP (PRK09203)→
Nit-1-2	NZ_CVRM01000022.1	+	261598	261676	**RNA**→ RplB (COG0090)rplB (PRK09374)→ rpsS (PRK00357)RpsS (COG0185)→ rplV (PRK00565)Ribosomal_L22 (pfam00237)→ RpsC (COG0092)rpsC (PRK00310)→ RplP (COG0197)rplP (PRK09203)→
Rsp-20-1	NZ_DS999054.1	+	3164594	3164672	**RNA**→ RplB (COG0090)rplB (PRK09374)→ RplB (COG0090)rplB (PRK09374)→ rpsS (PRK00357)RpsS (COG0185)→ rpsS (PRK00357)RpsS (COG0185)→ rplV (PRK00565)Ribosomal_L22 (pfam00237)→ rplV (PRK00565)Ribosomal_L22 (pfam00237)→ RpsC (COG0092)rpsC (PRK00310)→ RpsC (COG0092)rpsC (PRK00310)→ RplP (COG0197)rplP (PRK09203)→ RplP (COG0197)rplP (PRK09203)→
Lsp-2-1	NZ_JWLC01000007.1	-	265099	265021	**RNA**→ RplB (COG0090)rplB (PRK09374)→ rpsS (PRK00357)RpsS (COG0185)→ rplV (PRK00565)Ribosomal_L22 (pfam00237)→ RpsC (COG0092)rpsC (PRK00310)→ RplP (COG0197)rplP (PRK09203)→

Rba-7-1	NZ_DS995281.1	-	3127551	3127473	**RNA**→ RplB (COG0090)rplB (PRK09374)→ RplB (COG0090)rplB (PRK09374)→ rpsS (PRK00357)RpsS (COG0185)→ rpsS (PRK00357)RpsS (COG0185)→ rplV (PRK00565)Ribosomal_L22 (pfam00237)→ rplV (PRK00565)Ribosomal_L22 (pfam00237)→ RpsC (COG0092)rpsC (PRK00310)→ RpsC (COG0092)rpsC (PRK00310)→ RplP (COG0197)rplP (PRK09203)→ RplP (COG0197)rplP (PRK09203)→
Lca-1-1	NZ_KI421513.1	-	919197	919119	**RNA**→ RplB (COG0090)rplB (PRK09374)→ rpsS (PRK00357)RpsS (COG0185)→ rplV (PRK00565)Ribosomal_L22 (pfam00237)→ RpsC (COG0092)rpsC (PRK00310)→ RplP (COG0197)rplP (PRK09203)→
Lda-1-1	NZ_KI421500.1	-	2486523	2486445	**RNA**→ RplB (COG0090)rplB (PRK09374)→ rpsS (PRK00357)RpsS (COG0185)→ rplV (PRK00565)Ribosomal_L22 (pfam00237)→ RpsC (COG0092)rpsC (PRK00310)→ RplP (COG0197)rplP (PRK09203)→
Lsp-8-1	NZ_JWLD01000017.1	-	20237	20159	**RNA**→ RplB (COG0090)rplB (PRK09374)→ rpsS (PRK00357)RpsS (COG0185)→ rplV (PRK00565)Ribosomal_L22 (pfam00237)→ RpsC (COG0092)rpsC (PRK00310)→ RplP (COG0197)rplP (PRK09203)→
env-399	3300001605_10201626	+	440	500	**RNA**→ RplB (COG0090)rplB (PRK09374)→
env-400	3300001239_10025476	+	68	128	**RNA**→ rplB (PRK09374)Ribosomal_L2 (pfam00181)→
env-401	3300001605_10465888	+	68	128	**RNA**→ RplB (COG0090)rplB (PRK09374)→
env-402	3300001605_10229328	-	253	194	**RNA**→ RplB (COG0090)rplB (PRK09374)→
env-403	Antartic2_1036878	-	110	44	**RNA**→ ←hypo
env-404	CESI01066207.1	+	305	371	**RNA**→ rplB (PRK09374)Ribosomal_L2 (pfam00181)→
env-405	JCVI_SCAF_1096627291198	+	567	632	**RNA**→ RplB (COG0090)rplB (PRK09374)→
env-406	DelMOSum2010_c10000597	+	3357	3422	**RNA**→ RplB (COG0090)rplB (PRK09374)→ rpsS (PRK00357)RpsS (COG0185)→ rplV (PRK00565)Ribosomal_L22 (pfam00237)→ RpsC (COG0092)rpsC (PRK00310)→ RplP (COG0197)rplP (PRK09203)→
env-407	DelMOSum2011_c10059128	+	607	672	**RNA**→ RplB (COG0090)rplB (PRK09374)→
env-408	JGI20156J14371_10007799	-	4716	4651	**RNA**→ RplB (COG0090)rplB (PRK09374)→ rpsS (PRK00357)RpsS (COG0185)→ rplV (PRK00565)Ribosomal_L22 (pfam00237)→ RpsC (COG0092)rpsC (PRK00310)→ RplP (COG0197)rplP (PRK09203)→
env-409	JGI20154J14316_10019027	+	1070	1135	**RNA**→ RplB (COG0090)rplB (PRK09374)→ rpsS (PRK00357)RpsS (COG0185)→ rplV (PRK00565)Ribosomal_L22 (pfam00237)→ RpsC (COG0092)rpsC (PRK00310)→ RplP (COG0197)rplP (PRK09203)→
env-410	JGI20160J14292_10005213	+	2291	2356	**RNA**→ RplB (COG0090)rplB (PRK09374)→ rpsS (PRK00357)RpsS (COG0185)→ rplV (PRK00565)Ribosomal_L22 (pfam00237)→ RpsC (COG0092)rpsC (PRK00310)→ RplP (COG0197)rplP (PRK09203)→
env-411	JGI20157J14317_10008243	-	4807	4742	**RNA**→ RplB (COG0090)rplB (PRK09374)→ rpsS (PRK00357)RpsS (COG0185)→ rplV (PRK00565)Ribosomal_L22 (pfam00237)→ RpsC (COG0092)rpsC (PRK00310)→ RplP (COG0197)rplP (PRK09203)→
env-412	JGI20159J14440_10014743	-	561	496	**RNA**→ RplB (COG0090)rplB (PRK09374)→
env-413	JGI20155J14468_10115856	+	624	689	**RNA**→ RplB (COG0090)rplB (PRK09374)→
env-414	JGI20153J14295_10306322	-	286	219	**RNA**→ RplB (COG0090)rplB (PRK09374)→
env-415	JGI20153J14318_10306322	-	286	219	**RNA**→ RplB (COG0090)rplB (PRK09374)→
Rel-1-1	NZ_CP004372.1	+	1532613	1532674	**RNA**→ RplB (COG0090)rplB (PRK09374)→ rpsS (PRK00357)RpsS (COG0185)→ rplV (PRK00565)Ribosomal_L22 (pfam00237)→ RpsC (COG0092)rpsC (PRK00310)→ RplP (COG0197)rplP (PRK09203)→
env-416	JGI12210J13797_11047960	-	109	48	**RNA**→
env-417	SA_S1_NOR08_45m_c10022254	+	16	83	**RNA**→ RplB (COG0090)rplB (PRK09374)→ rpsS (PRK00357)RpsS (COG0185)→ rplV (PRK00565)Ribosomal_L22 (pfam00237)→ RpsC (COG0092)rpsC (PRK00310)→ RplP (COG0197)rplP (PRK09203)→
env-418	3300001605_10068995	-	547	488	**RNA**→ rplB (PRK09374)Ribosomal_L2 (pfam00181)→
env-419	JGI24768J34885_10007107	+	613	672	**RNA**→ RplB (COG0090)rplB (PRK09374)→ rpsS (PRK00357)RpsS (COG0185)→ rplV (PRK00565)Ribosomal_L22 (pfam00237)→ RpsC (COG0092)rpsC (PRK00310)→ RplP (COG0197)rplP (PRK09203)→
env-420	JGI25925J51416_10384036	+	89	148	**RNA**→ RplB (COG0090)rplB (PRK09374)→
env-421	3300001592_10004376	-	13045	12987	**RNA**→ RplB (COG0090)rplB (PRK09374)→ rpsS (PRK00357)Ribosomal_S19 (pfam00203)→ rplV (PRK00565)Ribosomal_L22 (pfam00237)→ RpsC (COG0092)rpsC (PRK00310)→ RplP (COG0197)rplP (PRK09203)→

Gne-1-1	NZ_KE386949.1	-	572600	572542	**RNA**→ RplB (COG0090)rplB (PRK09374)→ rpsS (PRK00357)RpsS (COG0185)→ rplV (PRK00565)Ribosomal_L22 (pfam00237)→ RpsC (COG0092)rpsC (PRK00310)→ RplP (COG0197)rplP (PRK09203)→
env-422	KVRMV2_101642803	+	262	356	**RNA**→ rplB (PRK09374)Ribosomal_L2 (pfam00181)→
env-423	JGI24724J26744_10668225	-	103	15	**RNA**→
env-424	JGI24723J26617_10025028	+	221	309	**RNA**→ RplB (COG0090)rplB (PRK09374)→ rpsS (PRK00357)RpsS (COG0185)→ rplV (PRK00565)Ribosomal_L22 (pfam00237)→ RpsC (COG0092)rpsC (PRK00310)→
env-425	JGI24723J26617_10031254	+	1462	1550	**RNA**→ RplB (COG0090)rplB (PRK09374)→
env-426	JGI24025J20009_10003199	+	1288	1376	**RNA**→ RplB (COG0090)rplB (PRK09374)→ rpsS (PRK00357)RpsS (COG0185)→ rplV (PRK00565)Ribosomal_L22 (pfam00237)→ RpsC (COG0092)rpsC (PRK00310)→ RplP (COG0197)rplP (PRK09203)→
Msp-1-1	NZ_JQEY01000002.1	+	278853	278919	**RNA**→ RplB (COG0090)rplB (PRK09374)→ rpsS (PRK00357)RpsS (COG0185)→ rplV (PRK00565)Ribosomal_L22 (pfam00237)→ RpsC (COG0092)rpsC (PRK00310)→ RplP (COG0197)rplP (PRK09203)→
Sno-1-1	NZ_JASC01000003.1	+	4659	4742	**RNA**→ RplB (COG0090)rplB (PRK09374)→ rpsS (PRK00357)RpsS (COG0185)→ rplV (PRK00565)Ribosomal_L22 (pfam00237)→ RpsC (COG0092)rpsC (PRK00310)→ RplP (COG0197)rplP (PRK09203)→
Sno-2-1	NZ_JASD01000008.1	-	392212	392129	**RNA**→ RplB (COG0090)rplB (PRK09374)→ rpsS (PRK00357)RpsS (COG0185)→ rplV (PRK00565)Ribosomal_L22 (pfam00237)→ RpsC (COG0092)rpsC (PRK00310)→ RplP (COG0197)rplP (PRK09203)→
env-427	CEWO01366965.1	+	1207	1273	**RNA**→ rplB (PRK09374)Ribosomal_L2 (pfam00181)→
env-428	wf2_AHAA5765_x1	-	329	263	**RNA**→
Tin-1-1	NZ_AUNB01000021.1	+	2941	3007	**RNA**→ RplB (COG0090)rplB (PRK09374)→ rpsS (PRK00357)RpsS (COG0185)→ rplV (PRK00565)Ribosomal_L22 (pfam00237)→ RpsC (COG0092)rpsC (PRK00310)→ RplP (COG0197)rplP (PRK09203)→
Pwa-1-1	NZ_LGHT01000042.1	-	148727	148661	**RNA**→ RplB (COG0090)rplB (PRK09374)→ rpsS (PRK00357)RpsS (COG0185)→ rplV (PRK00565)Ribosomal_L22 (pfam00237)→ RpsC (COG0092)rpsC (PRK00310)→ RplP (COG0197)rplP (PRK09203)→
env-429	3300001605_10795549	+	347	413	**RNA**→ ←hypo
env-430	F3OLC3J02I91T9	-	208	142	**RNA**→ hypo→
env-431	3300001605_10634355	-	145	79	**RNA**→
env-432	TB_FS06_10DRAFT_1000581	+	16462	16528	**RNA**→ RplB (COG0090)rplB (PRK09374)→ rpsS (PRK00357)RpsS (COG0185)→ rplV (PRK00565)Ribosomal_L22 (pfam00237)→ RpsC (COG0092)rpsC (PRK00310)→
env-433	3300000052_245369	+	133	199	**RNA**→
Osp-3-1	NZ_AFPM01000017.1	+	4012	4166	**RNA**→ ←hypo
env-434	JGI20160J14292_10238378	+	75	140	**RNA**→ hypo→ rplB (PRK09374)Ribosomal_L2 (pfam00181)→
env-435	GYQO9XB02IIOI9	-	83	18	**RNA**→
env-436	JGI24724J26744_10484319	-	357	266	**RNA**→ rplB (PRK09374)Ribosomal_L2 (pfam00181)→
env-437	3300001592_10003340	-	10494	10403	**RNA**→ RplB (COG0090)rplB (PRK09374)→ rpsS (PRK00357)RpsS (COG0185)→ rplV (PRK00565)Ribosomal_L22 (pfam00237)→ RpsC (COG0092)rpsC (PRK00310)→ RplP (COG0197)rplP (PRK09203)→
env-438	GSLSAS_GLMUJHB01D1RM5	-	167	75	**RNA**→ RplB (COG0090)rplB (PRK09374)→
env-439	JGI24025J20009_10404831	+	123	211	**RNA**→ ←hypo
Rsp-10-1	NZ_DS999213.1	+	3307378	3307453	**RNA**→ RplB (COG0090)rplB (PRK09374)→ RplB (COG0090)rplB (PRK09374)→ RplB (COG0090)rplB (PRK09374)→ RplB (COG0090)rplB (PRK09374)→ RplB (COG0090)rplB (PRK09374)→ rpsS (PRK00357)RpsS (COG0185)→ rpsS (PRK00357)RpsS (COG0185)→ rpsS (PRK00357)RpsS (COG0185)→ rplV (PRK00565)Ribosomal_L22 (pfam00237)→ rplV (PRK00565)Ribosomal_L22 (pfam00237)→ RpsC (COG0092)rpsC (PRK00310)→ RpsC (COG0092)rpsC (PRK00310)→ RplP (COG0197)rplP (PRK09203)→ RplP (COG0197)rplP (PRK09203)→
Tar-1-1	NZ_KE557314.1	-	63321	63252	**RNA**→ RplB (COG0090)rplB (PRK09374)→ RplB (COG0090)rplB (PRK09374)→ RplB (COG0090)rplB (PRK09374)→ rpsS (PRK00357)RpsS (COG0185)→ rplV (PRK00565)Ribosomal_L22 (pfam00237)→ RpsC (COG0092)rpsC (PRK00310)→ RplP (COG0197)rplP (PRK09203)→
env-440	JGI12109J20283_11967054	+	55	116	**RNA**→ hypo→

Sme-2-1	NZ_JASH01000010.1	-	261103	261034	**RNA**→ RplB (COG0090)rplB (PRK09374)→ rpsS (PRK00357)RpsS (COG0185)→ rplV (PRK00565)Ribosomal_L22 (pfam00237)→ RpsC (COG0092)rpsC (PRK00310)→ RplP (COG0197)rplP (PRK09203)→
Sme-1-1	NZ_JEMU01000021.1	+	4343	4412	**RNA**→ RplB (COG0090)rplB (PRK09374)→ rpsS (PRK00357)RpsS (COG0185)→ rplV (PRK00565)Ribosomal_L22 (pfam00237)→ RpsC (COG0092)rpsC (PRK00310)→ RplP (COG0197)rplP (PRK09203)→
Tsp-3-1	NZ_GG697169.2	+	2896550	2896617	**RNA**→ RplB (COG0090)rplB (PRK09374)→ RplB (COG0090)rplB (PRK09374)→ RplB (COG0090)rplB (PRK09374)→ rpsS (PRK00357)RpsS (COG0185)→ rpsS (PRK00357)RpsS (COG0185)→ rplV (PRK00565)Ribosomal_L22 (pfam00237)→ rplV (PRK00565)Ribosomal_L22 (pfam00237)→ RpsC (COG0092)rpsC (PRK00310)→ RpsC (COG0092)rpsC (PRK00310)→ RplP (COG0197)rplP (PRK09203)→ RplP (COG0197)rplP (PRK09203)→
env-441	P_1C_Liq_3_UnCty_1043905	-	143	47	**RNA**→
env-442	P_2C_Liq_1_UnCty_1002030	+	5317	5413	**RNA**→ RplB (COG0090)rplB (PRK09374)→ rpsS (PRK00357)RpsS (COG0185)→ rplV (PRK00565)Ribosomal_L22 (pfam00237)→ rpsC (PRK00310)30S_S3_KH (cd02412)→
env-443	P_2C_Liq_2_UnCty_1008969	+	248	344	**RNA**→ RplB (COG0090)rplB (PRK09374)→ rpsS (PRK00357)RpsS (COG0185)→ rplV (PRK00565)Ribosomal_L22 (pfam00237)→ rpsC (PRK00310)30S_S3_KH (cd02412)→
env-444	P_2C_Liq_2_UnCty_1056800	+	368	464	**RNA**→ RplB (COG0090)rplB (PRK09374)→
env-445	JGI12210J13797_10174589	-	302	232	**RNA**→ RplB (COG0090)rplB (PRK09374)→
env-446	JGI12210J13797_10174590	+	188	258	**RNA**→ RplB (COG0090)rplB (PRK09374)→
Rto-1-1	NZ_LGVV01000034.1	-	33402	33310	**RNA**→ RplB (COG0090)rplB (PRK09374)→ rpsS (PRK00357)RpsS (COG0185)→ RplV (COG0091)rplV (PRK00565)→ RpsC (COG0092)rpsC (PRK00310)→ RplP (COG0197)rplP (PRK09203)→
Ris-1-1	NZ_JAME01000033.1	-	54293	54185	**RNA**→ RplB (COG0090)rplB (PRK09374)→ rpsS (PRK00357)RpsS (COG0185)→ rplV (PRK00565)Ribosomal_L22 (pfam00237)→ RpsC (COG0092)rpsC (PRK00310)→ RplP (COG0197)rplP (PRK09203)→
env-447	KGI_S1_ANT02_95m_c10357205	+	47	140	**RNA**→ RplB (COG0090)rplB (PRK09374)→
env-448	LADL01003569.1	+	6160	6249	**RNA**→ RplB (COG0090)rplB (PRK09374)→ rpsS (PRK00357)RpsS (COG0185)→ RplV (COG0091)rplV (PRK00565)→ RpsC (COG0092)rpsC (PRK00310)→ RplP (COG0197)rplP (PRK09203)→
env-449	3300001239_10287636	-	266	177	**RNA**→ RplB (COG0090)rplB (PRK09374)→
env-450	3300001605_10004081	-	3779	3690	**RNA**→ RplB (COG0090)rplB (PRK09374)→ rpsS (PRK00357)RpsS (COG0185)→ RplV (COG0091)rplV (PRK00565)→ RpsC (COG0092)rpsC (PRK00310)→ RplP (COG0197)rplP (PRK09203)→
env-451	JGI25317J35169_1001357	-	3662	3573	**RNA**→ RplB (COG0090)rplB (PRK09374)→ rpsS (PRK00357)RpsS (COG0185)→ RplV (COG0091)rplV (PRK00565)→ RpsC (COG0092)rpsC (PRK00310)→ RplP (COG0197)rplP (PRK09203)→
env-452	JGI25319J35699_1000784	-	3894	3805	**RNA**→ RplB (COG0090)rplB (PRK09374)→ rpsS (PRK00357)RpsS (COG0185)→ RplV (COG0091)rplV (PRK00565)→ RpsC (COG0092)rpsC (PRK00310)→ RplP (COG0197)rplP (PRK09203)→
env-453	JGI25322J35698_1004507	-	4384	4295	**RNA**→ RplB (COG0090)rplB (PRK09374)→ rpsS (PRK00357)RpsS (COG0185)→ RplV (COG0091)rplV (PRK00565)→ RpsC (COG0092)rpsC (PRK00310)→ RplP (COG0197)rplP (PRK09203)→
env-454	JGI25318J35512_1000721	-	3894	3805	**RNA**→ RplB (COG0090)rplB (PRK09374)→ rpsS (PRK00357)RpsS (COG0185)→ RplV (COG0091)rplV (PRK00565)→ RpsC (COG0092)rpsC (PRK00310)→ RplP (COG0197)rplP (PRK09203)→
env-455	JGI25321J39296_1001405	+	10390	10479	**RNA**→ RplB (COG0090)rplB (PRK09374)→ rpsS (PRK00357)RpsS (COG0185)→ RplV (COG0091)rplV (PRK00565)→ RpsC (COG0092)rpsC (PRK00310)→ RplP (COG0197)rplP (PRK09203)→
env-456	JGI25320J50211_1000655	+	15085	15174	**RNA**→ RplB (COG0090)rplB (PRK09374)→ rpsS (PRK00357)RpsS (COG0185)→ RplV (COG0091)rplV (PRK00565)→ RpsC (COG0092)rpsC (PRK00310)→ RplP (COG0197)rplP (PRK09203)→
env-457	JGI25321J50212_10002409	+	10390	10479	**RNA**→ RplB (COG0090)rplB (PRK09374)→ rpsS (PRK00357)RpsS (COG0185)→ RplV (COG0091)rplV (PRK00565)→ RpsC (COG0092)rpsC (PRK00310)→ RplP (COG0197)rplP (PRK09203)→
Tpa-1-1	NZ_AUND01000002.1	+	2906	2974	**RNA**→ RplB (COG0090)rplB (PRK09374)→ rpsS (PRK00357)RpsS (COG0185)→ rplV (PRK00565)Ribosomal_L22 (pfam00237)→ RpsC (COG0092)rpsC (PRK00310)→ RplP (COG0197)rplP (PRK09203)→ hypo→
env-458	CEVN01086405.1	-	332	264	**RNA**→ rplB (PRK09374)Ribosomal_L2 (pfam00181)→
Tat-1-1	NZ_AQRC01000003.1	-	337439	337371	**RNA**→ RplB (COG0090)rplB (PRK09374)→ rpsS (PRK00357)RpsS (COG0185)→ rplV (PRK00565)Ribosomal_L22 (pfam00237)→ RpsC (COG0092)rpsC (PRK00310)→ RplP (COG0197)rplP (PRK09203)→
env-459	TFO_HY__Contig_1825	+	253	321	**RNA**→ RplB (COG0090)rplB (PRK09374)→ rpsS (PRK00357)RpsS (COG0185)→ rplV (PRK00565)Ribosomal_L22 (pfam00237)→ KH (smart00322)30S_S3_KH (cd02412)→
env-460	CESE01105678.1	+	551	617	**RNA**→
env-461	CESI01080217.1	-	93	27	**RNA**→
env-462	CESU01020394.1	-	93	27	**RNA**→
env-463	CESJ01170158.1	-	78	12	**RNA**→

env-464	CEST01002662.1	-	308	242	**RNA**→ RplB (COG0090)rplB (PRK09374)→
env-465	CESI01225209.1	-	91	25	**RNA**→
env-466	CESU01161643.1	-	91	25	**RNA**→
env-467	DelMOSum2010_c10465078	-	126	60	**RNA**→ RplB (COG0090)rplB (PRK09374)→
env-468	JGI20157J14317_10424860	+	203	269	**RNA**→ RplB (COG0090)rplB (PRK09374)→
env-469	JGI20152J14361_10060366	+	794	860	**RNA**→ RplB (COG0090)rplB (PRK09374)→
env-470	JGI20151J14362_10458567	+	170	236	**RNA**→ RplB (COG0090)rplB (PRK09374)→
env-471	JGI20157J14317_10486189	+	185	251	**RNA**→
env-472	CERM01031828.1	-	1160	1094	**RNA**→ RplB (COG0090)rplB (PRK09374)→ rpsS (PRK00357)Ribosomal_S19 (pfam00203)→
env-473	JGI20152J14361_10019476	-	938	873	**RNA**→ rplB (PRK09374)Ribosomal_L2 (pfam00181)→ rplB (PRK09374)Ribosomal_L2_C (pfam03947)→
env-474	JGI20157J14317_10015572	-	3473	3408	**RNA**→ RplB (COG0090)rplB (PRK09374)→ rpsS (PRK00357)RpsS (COG0185)→<br>rplV (PRK00565)Ribosomal_L22 (pfam00237)→ RpsC (COG0092)rpsC (PRK00310)→<br>RplP (COG0197)rplP (PRK09203)→
env-475	2236876002_042693	-	416	351	**RNA**→ rplB (PRK09374)Ribosomal_L2 (pfam00181)→
env-476	JGI20158J14315_10019288	+	625	691	**RNA**→ rplB (PRK09374)Ribosomal_L2 (pfam00181)→ rplB (PRK09374)Ribosomal_L2_C (pfam03947)→<br>rpsS (PRK00357)RpsS (COG0185)→ rplV (PRK00565)Ribosomal_L22 (pfam00237)→<br>RpsC (COG0092)rpsC (PRK00310)→ RplP (COG0197)rplP (PRK09203)→
env-477	P_2C_Liq_1_UnCty_1012554	+	141	207	**RNA**→ RplB (COG0090)rplB (PRK09374)→ rpsS (PRK00357)RpsS (COG0185)→<br>rplV (PRK00565)Ribosomal_L22 (pfam00237)→ rpsC (PRK00310)30S_S3_KH (cd02412)→
env-478	P_2C_Liq_2_UnCty_1004631	+	159	225	**RNA**→ RplB (COG0090)rplB (PRK09374)→ rpsS (PRK00357)RpsS (COG0185)→<br>rplV (PRK00565)Ribosomal_L22 (pfam00237)→ RpsC (COG0092)rpsC (PRK00310)→<br>RplP (COG0197)rplP (PRK09203)→
env-479	JGI20151J14362_10307003	+	248	314	**RNA**→ hypo→
env-480	JGI20154J14316_10012324	+	1879	1945	**RNA**→ RplB (COG0090)rplB (PRK09374)→ rpsS (PRK00357)RpsS (COG0185)→<br>rplV (PRK00565)Ribosomal_L22 (pfam00237)→ KH (smart00322)30S_S3_KH (cd02412)→<br>rpsC (PRK00310)Ribosomal_S3_C (pfam00189)→ RplP (COG0197)rplP (PRK09203)→
env-481	JGI20160J14292_10120340	-	142	76	**RNA**→
env-482	JCVI_SCAF_1101668062396	-	504	438	**RNA**→ rplB (PRK09374)Ribosomal_L2 (pfam00181)→
env-483	JGI20156J14371_10029263	-	2057	1991	**RNA**→ RplB (COG0090)rplB (PRK09374)→ rpsS (PRK00357)RpsS (COG0185)→<br>rplV (PRK00565)Ribosomal_L22 (pfam00237)→ RpsC (COG0092)rpsC (PRK00310)→
env-484	JCVI_SCAF_1101667021862	+	537	603	**RNA**→ rplB (PRK09374)Ribosomal_L2 (pfam00181)→
env-485	JCVI_SCAF_1096626884361	-	4210	4144	**RNA**→ RplB (COG0090)rplB (PRK09374)→ rpsS (PRK00357)RpsS (COG0185)→<br>rplV (PRK00565)Ribosomal_L22 (pfam00237)→ RpsC (COG0092)rpsC (PRK00310)→<br>RplP (COG0197)rplP (PRK09203)→
env-486	JCVI_SCAF_1096627292964	-	129	63	**RNA**→ RplB (COG0090)rplB (PRK09374)→
env-487	DelMOSum2010_c10287456	-	395	329	**RNA**→ rplB (PRK09374)Ribosomal_L2 (pfam00181)→
env-488	DelMOSum2011_c10012372	-	3519	3453	**RNA**→ RplB (COG0090)rplB (PRK09374)→ rpsS (PRK00357)RpsS (COG0185)→<br>rplV (PRK00565)Ribosomal_L22 (pfam00237)→ RpsC (COG0092)rpsC (PRK00310)→<br>RplP (COG0197)rplP (PRK09203)→
env-489	JGI20153J14295_10009487	-	3477	3411	**RNA**→ RplB (COG0090)rplB (PRK09374)→ rpsS (PRK00357)RpsS (COG0185)→<br>rplV (PRK00565)Ribosomal_L22 (pfam00237)→ RpsC (COG0092)rpsC (PRK00310)→<br>RplP (COG0197)rplP (PRK09203)→
env-490	JGI20153J14318_10009487	-	3477	3411	**RNA**→ RplB (COG0090)rplB (PRK09374)→ rpsS (PRK00357)RpsS (COG0185)→<br>rplV (PRK00565)Ribosomal_L22 (pfam00237)→ RpsC (COG0092)rpsC (PRK00310)→<br>RplP (COG0197)rplP (PRK09203)→
env-491	JGI20159J14440_10009403	+	2075	2141	**RNA**→ RplB (COG0090)rplB (PRK09374)→ rpsS (PRK00357)RpsS (COG0185)→<br>rplV (PRK00565)Ribosomal_L22 (pfam00237)→ RpsC (COG0092)rpsC (PRK00310)→<br>RplP (COG0197)rplP (PRK09203)→
env-492	JGI20155J14468_10011527	+	2004	2070	**RNA**→ RplB (COG0090)rplB (PRK09374)→ rpsS (PRK00357)RpsS (COG0185)→<br>rplV (PRK00565)Ribosomal_L22 (pfam00237)→ RpsC (COG0092)rpsC (PRK00310)→<br>RplP (COG0197)rplP (PRK09203)→
Pte-1-1	NZ_CP003984.1	+	2755361	2755427	**RNA**→ RplB (COG0090)rplB (PRK09374)→ rpsS (PRK00357)RpsS (COG0185)→<br>rplV (PRK00565)Ribosomal_L22 (pfam00237)→ RpsC (COG0092)rpsC (PRK00310)→<br>RplP (COG0197)rplP (PRK09203)→
env-493	JCVI_SCAF_1101668090558	+	847	913	**RNA**→ RplB (COG0090)rplB (PRK09374)→
env-494	DelMOSum2010_c10054733	-	257	191	**RNA**→ RplB (COG0090)rplB (PRK09374)→

env-495	JGI20151J14362_10072490	-	659	593	**RNA**→ RplB (COG0090)rplB (PRK09374)→
env-496	JGI20160J14292_10100804	-	278	212	**RNA**→ ←hypo
env-497	JGI24724J26744_10002738	-	11069	10947	**RNA**→ RplB (COG0090)rplB (PRK09374)→ rpsS (PRK00357)RpsS (COG0185)→
					rplV (PRK00565)Ribosomal_L22 (pfam00237)→ RpsC (COG0092)rpsC (PRK00310)→
					RplP (COG0197)rplP (PRK09203)→
env-498	JGI24723J26617_10030085	-	1778	1656	**RNA**→ RplB (COG0090)rplB (PRK09374)→ rpsS (PRK00357)RpsS (COG0185)→
					rplV (PRK00565)Ribosomal_L22 (pfam00237)→ rpsC (PRK00310)30S_S3_KH (cd02412)→
env-499	JGI24723J26617_10138499	+	494	616	**RNA**→
env-500	P_2C_Liq_1_UnCty_1025942	+	228	318	**RNA**→ RplB (COG0090)rplB (PRK09374)→
env-501	P_2C_Liq_2_UnCty_1020991	+	163	253	**RNA**→ RplB (COG0090)rplB (PRK09374)→
env-502	JCVI_SCAF_1096627363432	-	1000	917	**RNA**→ RplB (COG0090)rplB (PRK09374)→
env-503	P_1C_Liq_1_UnCty_1060565	+	89	180	**RNA**→ RplB (COG0090)rplB (PRK09374)→
env-504	P_2C_Liq_1_UnCty_1025617	+	238	329	**RNA**→ RplB (COG0090)rplB (PRK09374)→
env-505	P_2C_Liq_2_UnCty_1030521	+	237	327	**RNA**→ RplB (COG0090)rplB (PRK09374)→
Smu-1-1	NZ_KE557273.1	+	282336	282429	**RNA**→ RplB (COG0090)rplB (PRK09374)→ rpsS (PRK00357)RpsS (COG0185)→
					rplV (PRK00565)Ribosomal_L22 (pfam00237)→ RpsC (COG0092)rpsC (PRK00310)→
					RplP (COG0197)rplP (PRK09203)→
env-506	JGI20154J14316_10000278	-	10091	10028	**RNA**→ RplB (COG0090)rplB (PRK09374)→ rpsS (PRK00357)RpsS (COG0185)→
					rplV (PRK00565)Ribosomal_L22 (pfam00237)→ RpsC (COG0092)rpsC (PRK00310)→
					RplP (COG0197)rplP (PRK09203)→ PRK00306 (PRK00306)Ribosomal_L29 (pfam00831)→
					rpsQ (PRK05610)Ribosomal_S17 (pfam00366)→ rplN (PRK05483)Ribosomal_L14 (pfam00238)→
					RplX (COG0198)rplX (PRK00004)→ rplE (PRK00010)RplE (COG0094)→
					rpsN (PRK08881)Ribosomal_S14 (pfam00253)→ rpsH (PRK00136)Ribosomal_S8 (pfam00410)→
					RplF (COG0097)rplF (PRK05498)→ rplR (PRK05593)Ribosomal_L18p (pfam00861)→
					RpsE (COG0098)rpsE (PRK00550)→ Ribosomal_L30 (cd01658)rpmD (PRK05611)→
env-507	CESJ01079969.1	+	363	426	**RNA**→ RplB (COG0090)rplB (PRK09374)→
env-508	DelMOSum2011_c10084907	-	321	258	**RNA**→ rplB (PRK09374)Ribosomal_L2 (pfam00181)→
env-509	JGI20160J14292_10062109	+	335	398	**RNA**→ RplB (COG0090)rplB (PRK09374)→ rpsS (PRK00357)RpsS (COG0185)→
env-510	CESI01081701.1	-	1672	1609	**RNA**→ RplB (COG0090)rplB (PRK09374)→ rpsS (PRK00357)RpsS (COG0185)→
					rplV (PRK00565)Ribosomal_L22 (pfam00237)→ rpsC (PRK00310)30S_S3_KH (cd02412)→
env-511	CESU01035091.1	-	8481	8418	**RNA**→ RplB (COG0090)rplB (PRK09374)→ rpsS (PRK00357)RpsS (COG0185)→
					rplV (PRK00565)Ribosomal_L22 (pfam00237)→ RpsC (COG0092)rpsC (PRK00310)→
					RplP (COG0197)rplP (PRK09203)→ PRK00306 (PRK00306)Ribosomal_L29 (pfam00831)→
					rpsQ (PRK05610)Ribosomal_S17 (pfam00366)→ rplN (PRK05483)Ribosomal_L14 (pfam00238)→
					RplX (COG0198)rplX (PRK00004)→ rplE (PRK00010)RplE (COG0094)→
					rpsN (PRK08881)Ribosomal_S14 (pfam00253)→ rpsH (PRK00136)Ribosomal_S8 (pfam00410)→
					RplF (COG0097)rplF (PRK05498)→ rplR (PRK05593)Ribosomal_L18p (pfam00861)→
					RpsE (COG0098)rpsE (PRK00550)→ Ribosomal_L30 (cd01658)rpmD (PRK05611)→
env-512	JGI20151J14362_10229901	+	58	122	**RNA**→ rplB (PRK09374)Ribosomal_L2 (pfam00181)→
env-513	2236876002_225186	+	183	247	**RNA**→ RplB (COG0090)rplB (PRK09374)→
env-514	2236876010_0411961	+	127	191	**RNA**→
env-515	CERM01014667.1	+	479	543	**RNA**→ RplB (COG0090)rplB (PRK09374)→ rpsS (PRK00357)RpsS (COG0185)→
					rplV (PRK00565)Ribosomal_L22 (pfam00237)→ RpsC (COG0092)rpsC (PRK00310)→
					RplP (COG0197)rplP (PRK09203)→ PRK00306 (PRK00306)Ribosomal_L29 (pfam00831)→
					rpsQ (PRK05610)Ribosomal_S17 (pfam00366)→ rplN (PRK05483)Ribosomal_L14 (pfam00238)→
					RplX (COG0198)rplX (PRK00004)→ rplE (PRK00010)RplE (COG0094)→
					rpsN (PRK08881)Ribosomal_S14 (pfam00253)→ rpsH (PRK00136)Ribosomal_S8 (pfam00410)→
					RplF (COG0097)rplF (PRK05498)→ rplR (PRK05593)Ribosomal_L18p (pfam00861)→
					RpsE (COG0098)rpsE (PRK00550)→ Ribosomal_L30 (cd01658)rpmD (PRK05611)→
env-516	2236876009_129660	+	384	447	**RNA**→
env-517	JGI20160J14292_10014797	+	4611	4675	**RNA**→
env-518	JGI20157J14317_10244667	+	41	105	**RNA**→ rplB (PRK09374)Ribosomal_L2 (pfam00181)→
env-519	JCVI_READ_1105499001019	-	595	532	**RNA**→ rplB (PRK09374)Ribosomal_L2 (pfam00181)→
env-520	2236876010_0225648	+	55	119	**RNA**→ RplB (COG0090)rplB (PRK09374)→
env-521	2236876011_173305	+	341	406	**RNA**→

env-522	P_2C_Liq_2_UnCty_1069081	+	299	389	RNA→ RplB (COG0090)rplB (PRK09374)→
env-523	P_2C_Liq_1_UnCty_1043093	-	165	75	RNA→ RplB (COG0090)rplB (PRK09374)→
env-524	KGI_S2_ANT04_2345m_c1261754	+	93	130	RNA→ RplB (COG0090)rplB (PRK09374)→
env-525	JGI24723J26617_10273187	-	116	79	RNA→
env-526	CESU01002408.1	+	477	543	RNA→
Tda-1-1	NZ_JHEH01000004.1	+	3299	3366	RNA→ RplB (COG0090)rplB (PRK09374)→ rpsS (PRK00357)RpsS (COG0185)→ rplV (PRK00565)Ribosomal_L22 (pfam00237)→ RpsC (COG0092)rpsC (PRK00310)→ RplP (COG0197)rplP (PRK09203)→
Dsp-1-1	NZ_AYXI01000047.1	+	2274	2353	RNA→ RplB (COG0090)rplB (PRK09374)→ rpsS (PRK00357)RpsS (COG0185)→ rplV (PRK00565)Ribosomal_L22 (pfam00237)→ RpsC (COG0092)rpsC (PRK00310)→ RplP (COG0197)rplP (PRK09203)→
env-527	JGI24770J26754_10000998	-	16793	16732	RNA→ RplB (COG0090)rplB (PRK09374)→ rpsS (PRK00357)RpsS (COG0185)→ rplV (PRK00565)Ribosomal_L22 (pfam00237)→ RpsC (COG0092)rpsC (PRK00310)→ RplP (COG0197)rplP (PRK09203)→ 2OG-FeII_Oxy_5 (pfam13759)→
Jaq-1-1	NZ_JYFE01000080.1	-	61937	61870	RNA→ RplB (COG0090)rplB (PRK09374)→ rpsS (PRK00357)RpsS (COG0185)→ rplV (PRK00565)Ribosomal_L22 (pfam00237)→ RpsC (COG0092)rpsC (PRK00310)→ RplP (COG0197)rplP (PRK09203)→
Sge-1-1	NZ_JASE01000005.1	-	3545549	3545479	RNA→ RplB (COG0090)rplB (PRK09374)→ rpsS (PRK00357)RpsS (COG0185)→ rplV (PRK00565)Ribosomal_L22 (pfam00237)→ RpsC (COG0092)rpsC (PRK00310)→ RplP (COG0197)rplP (PRK09203)→
env-528	CESE01069575.1	-	1653	1587	RNA→ RplB (COG0090)rplB (PRK09374)→ rpsS (PRK00357)RpsS (COG0185)→ rplV (PRK00565)Ribosomal_L22 (pfam00237)→ rpsC (PRK00310)30S_S3_KH (cd02412)→
env-529	CEST01031231.1	-	1873	1807	RNA→ RplB (COG0090)rplB (PRK09374)→ rpsS (PRK00357)RpsS (COG0185)→ rplV (PRK00565)Ribosomal_L22 (pfam00237)→ KH (smart00322)30S_S3_KH (cd02412)→
env-530	2236876001_338482	-	82	18	RNA→ hypo→
env-531	2236876009_222696	-	140	75	RNA→ RplB (COG0090)rplB (PRK09374)→
env-532	JGI20152J14361_10053033	+	949	1014	RNA→
env-533	2236876001_438614	-	371	305	RNA→ rplB (PRK09374)Ribosomal_L2 (pfam00181)→
env-534	SI34jun09_10m_1003973	-	1624	1559	RNA→ RplB (COG0090)rplB (PRK09374)→ rpsS (PRK00357)RpsS (COG0185)→ rplV (PRK00565)Ribosomal_L22 (pfam00237)→
env-535	CESU01069477.1	-	82	17	RNA→
env-536	DelMOSum2010_c10041356	-	259	194	RNA→ RplB (COG0090)rplB (PRK09374)→
env-537	DelMOSum2011_c10032717	-	140	75	RNA→
env-538	SI60aug11_100m_c1018162	-	144	79	RNA→
env-539	SI47jul10_120m_c1099469	+	199	264	RNA→ RplB (COG0090)rplB (PRK09374)→
env-540	SI34jun09_135m_1040685	-	788	723	RNA→ RplB (COG0090)rplB (PRK09374)→
env-541	SI34jun09_100m_1002546	-	626	561	RNA→ RplB (COG0090)rplB (PRK09374)→
env-542	JGI20151J14362_10030512	+	2508	2573	RNA→
env-543	CESI01072935.1	+	1105	1170	RNA→
env-544	PML_READ_01030272	-	197	132	RNA→ RplB (COG0090)rplB (PRK09374)→
env-545	F10SI0310a_Sequence0000021673	-	388	323	RNA→ rplB (PRK09374)Ribosomal_L2 (pfam00181)→
env-546	2236876003_146267	+	345	409	RNA→ RplB (COG0090)rplB (PRK09374)→
env-547	2236876010_0560637	+	290	354	RNA→ ←hypo
env-548	2236876010_0169121	-	311	246	RNA→ RplB (COG0090)rplB (PRK09374)→
env-549	2236876009_267955	-	333	268	RNA→ rplB (PRK09374)Ribosomal_L2 (pfam00181)→
env-550	2236876009_052156	-	273	208	RNA→ RplB (COG0090)rplB (PRK09374)→
env-551	2236876010_0456997	-	181	116	RNA→ RplB (COG0090)rplB (PRK09374)→
env-552	DelMOSum2011_c10406745	-	105	40	RNA→ ←hypo
env-553	SI54feb11_100m_c1113376	-	189	124	RNA→ RplB (COG0090)rplB (PRK09374)→
env-554	SI48aug10_10m_c1000222	+	2332	2397	RNA→ RplB (COG0090)rplB (PRK09374)→ rpsS (PRK00357)RpsS (COG0185)→ rplV (PRK00565)Ribosomal_L22 (pfam00237)→ RpsC (COG0092)rpsC (PRK00310)→ RplP (COG0197)rplP (PRK09203)→
env-555	SI34jun09_120m_1016761	-	1571	1506	RNA→ RplB (COG0090)rplB (PRK09374)→ rpsS (PRK00357)RpsS (COG0185)→ rplV (PRK00565)Ribosomal_L22 (pfam00237)→
env-556	SI48aug10_100m_1019662	-	473	408	RNA→ rplB (PRK09374)Ribosomal_L2 (pfam00181)→

env-557	JGI20156J14371_10036454	-	128	63	**RNA**→
env-558	JGI20154J14316_10048488	+	1717	1782	**RNA**→
env-559	JGI20160J14292_10049921	-	944	879	**RNA**→ RplB (COG0090)rplB (PRK09374)→
env-560	JGI20153J14295_10106585	+	637	702	**RNA**→
env-561	JGI20153J14318_10106585	+	637	702	**RNA**→
env-562	JGI20157J14317_10081233	+	1167	1232	**RNA**→
env-563	JGI20158J14315_10394374	+	254	319	**RNA**→ rplW (PRK05738)Ribosomal_L23 (pfam00276)→
env-564	CERI01006995.1	-	80	15	**RNA**→
env-565	CESO01025210.1	-	5544	5479	**RNA**→ RplB (COG0090)rplB (PRK09374)→ rpsS (PRK00357)RpsS (COG0185)→ rplV (PRK00565)Ribosomal_L22 (pfam00237)→ RpsC (COG0092)rpsC (PRK00310)→ RplP (COG0197)rplP (PRK09203)→
env-566	F10SI03100a_Sequence0000087588	-	597	532	**RNA**→ RplB (COG0090)rplB (PRK09374)→
Rba-5-1	NZ_DS022282.1	-	1056719	1056654	**RNA**→ RplB (COG0090)rplB (PRK09374)→ rpsS (PRK00357)RpsS (COG0185)→ rplV (PRK00565)Ribosomal_L22 (pfam00237)→ RpsC (COG0092)rpsC (PRK00310)→ RplP (COG0197)rplP (PRK09203)→
env-567	JCVI_SCAF_1101667005364	+	568	633	**RNA**→ rplB (PRK09374)Ribosomal_L2 (pfam00181)→
env-568	DelMOSum2010_c10035518	+	2424	2489	**RNA**→ RplB (COG0090)rplB (PRK09374)→
env-569	DelMOSum2011_c10035518	+	2007	2072	**RNA**→
env-570	JGI20152J14361_10025542	+	1917	1982	**RNA**→
env-571	JGI20153J14295_10035283	-	131	66	**RNA**→
env-572	JGI20153J14318_10035283	-	131	66	**RNA**→
env-573	JGI20157J14317_10045573	+	1946	2011	**RNA**→
env-574	CESI01143925.1	+	1396	1461	**RNA**→
env-575	CESU01042376.1	-	78	13	**RNA**→
env-576	DelMOWin2010_c10019689	-	113	48	**RNA**→ ←hypo
env-577	JGI20152J14361_10025431	-	148	83	**RNA**→
env-578	JGI20160J14292_10037631	-	132	67	**RNA**→
env-579	JGI20151J14362_10009967	-	5544	5479	**RNA**→ RplB (COG0090)rplB (PRK09374)→ rpsS (PRK00357)RpsS (COG0185)→ rplV (PRK00565)Ribosomal_L22 (pfam00237)→ RpsC (COG0092)rpsC (PRK00310)→
env-580	JGI20157J14317_10012557	+	1945	2010	**RNA**→ RplB (COG0090)rplB (PRK09374)→ rpsS (PRK00357)RpsS (COG0185)→ rplV (PRK00565)Ribosomal_L22 (pfam00237)→ RpsC (COG0092)rpsC (PRK00310)→ RplP (COG0197)rplP (PRK09203)→
env-581	CERM01019450.1	+	628	693	**RNA**→
env-582	2236876001_009312	-	254	189	**RNA**→ RplB (COG0090)rplB (PRK09374)→
env-583	JGI20156J14371_10029870	+	2238	2303	**RNA**→ rplB (PRK09374)Ribosomal_L2 (pfam00181)→
env-584	JGI20154J14316_10019470	-	1818	1753	**RNA**→ RplB (COG0090)rplB (PRK09374)→ rpsS (PRK00357)RpsS (COG0185)→ rplV (PRK00565)Ribosomal_L22 (pfam00237)→ rpsC (PRK00310)30S_S3_KH (cd02412)→
env-585	JGI20153J14295_10035952	+	1907	1972	**RNA**→
env-586	JGI20153J14318_10035952	+	1907	1972	**RNA**→
env-587	JGI20159J14440_10007957	+	3434	3499	**RNA**→ RplB (COG0090)rplB (PRK09374)→ rpsS (PRK00357)RpsS (COG0185)→ rplV (PRK00565)Ribosomal_L22 (pfam00237)→ RpsC (COG0092)rpsC (PRK00310)→ rplP (PRK09203)Ribosomal_L16 (pfam00252)→
env-588	JGI20155J14468_10004488	+	2462	2527	**RNA**→ RplB (COG0090)rplB (PRK09374)→ rpsS (PRK00357)RpsS (COG0185)→ rplV (PRK00565)Ribosomal_L22 (pfam00237)→ RpsC (COG0092)rpsC (PRK00310)→ RplP (COG0197)rplP (PRK09203)→
env-589	JGI20158J14315_10007229	+	2960	3025	**RNA**→ RplB (COG0090)rplB (PRK09374)→ rpsS (PRK00357)RpsS (COG0185)→ rplV (PRK00565)Ribosomal_L22 (pfam00237)→ RpsC (COG0092)rpsC (PRK00310)→ RplP (COG0197)rplP (PRK09203)→
Sgu-1-1	NZ_JASG01000004.1	-	2771313	2771244	**RNA**→ RplB (COG0090)rplB (PRK09374)→ rpsS (PRK00357)RpsS (COG0185)→ rplV (PRK00565)Ribosomal_L22 (pfam00237)→ RpsC (COG0092)rpsC (PRK00310)→ RplP (COG0197)rplP (PRK09203)→
env-590	CENJ01071080.1	+	446	521	**RNA**→ RplB (COG0090)rplB (PRK09374)→ rpsS (PRK00357)RpsS (COG0185)→
env-591	CENR01032483.1	+	411	486	**RNA**→
env-592	JGI12120J13076_100262	+	774	849	**RNA**→ RplB (COG0090)rplB (PRK09374)→ rpsS (PRK00357)RpsS (COG0185)→ rplV (PRK00565)Ribosomal_L22 (pfam00237)→ rpsC (PRK00310)30S_S3_KH (cd02412)→

env-593	JGI11929J13085_109680	+	171	246	**RNA**→ RplB (COG0090)rplB (PRK09374)→
env-594	JGI12207J13218_1020216	+	89	164	**RNA**→ rplB (PRK09374)Ribosomal_L2 (pfam00181)→
env-595	CEOE01076289.1	-	558	483	**RNA**→ rplB (PRK09374)Ribosomal_L2 (pfam00181)→
env-596	LAZR01000132.1	-	92425	92350	**RNA**→ RplB (COG0090)rplB (PRK09374)→ rpsS (PRK00357)RpsS (COG0185)→<br>rplV (PRK00565)Ribosomal_L22 (pfam00237)→ RpsC (COG0092)rpsC (PRK00310)→<br>RplP (COG0197)rplP (PRK09203)→
env-597	CEWQ01009550.1	+	346	421	**RNA**→ RplB (COG0090)rplB (PRK09374)→
Ssp-3-1	NZ_JPOY01000011.1	-	2334383	2334308	**RNA**→ RplB (COG0090)rplB (PRK09374)→ rpsS (PRK00357)RpsS (COG0185)→<br>rplV (PRK00565)Ribosomal_L22 (pfam00237)→ RpsC (COG0092)rpsC (PRK00310)→<br>RplP (COG0197)rplP (PRK09203)→
Ssp-4-1	NZ_CH959310.1	-	345269	345194	**RNA**→ RplB (COG0090)rplB (PRK09374)→ rpsS (PRK00357)RpsS (COG0185)→<br>rplV (PRK00565)Ribosomal_L22 (pfam00237)→ RpsC (COG0092)rpsC (PRK00310)→<br>RplP (COG0197)rplP (PRK09203)→
env-598	CEQX01294317.1	-	107	32	**RNA**→
env-599	CESO01088717.1	+	87	162	**RNA**→ rplB (PRK09374)Ribosomal_L2 (pfam00181)→
Ssp-5-1	NZ_CH959312.1	-	929126	929051	**RNA**→ RplB (COG0090)rplB (PRK09374)→ rpsS (PRK00357)RpsS (COG0185)→<br>rplV (PRK00565)Ribosomal_L22 (pfam00237)→ RpsC (COG0092)rpsC (PRK00310)→<br>RplP (COG0197)rplP (PRK09203)→
Spo-1-1	NZ_AXZR01000005.1	+	3608	3683	**RNA**→ RplB (COG0090)rplB (PRK09374)→ rpsS (PRK00357)RpsS (COG0185)→<br>rplV (PRK00565)Ribosomal_L22 (pfam00237)→ RpsC (COG0092)rpsC (PRK00310)→<br>RplP (COG0197)rplP (PRK09203)→
env-600	CEQZ01007430.1	-	103	28	**RNA**→
env-601	CERD01103759.1	+	510	585	**RNA**→ RplB (COG0090)rplB (PRK09374)→ rpsS (PRK00357)RpsS (COG0185)→<br>rplV (PRK00565)Ribosomal_L22 (pfam00237)→ RpsC (COG0092)rpsC (PRK00310)→
env-602	CERE01033137.1	+	1304	1379	**RNA**→ RplB (COG0090)rplB (PRK09374)→ rpsS (PRK00357)RpsS (COG0185)→<br>rplV (PRK00565)Ribosomal_L22 (pfam00237)→ RpsC (COG0092)rpsC (PRK00310)→<br>RplP (COG0197)rplP (PRK09203)→
env-603	CERJ01019869.1	-	717	642	**RNA**→ RplB (COG0090)rplB (PRK09374)→
env-604	CESK01030715.1	-	2698	2623	**RNA**→ RplB (COG0090)rplB (PRK09374)→ rpsS (PRK00357)RpsS (COG0185)→<br>rplV (PRK00565)Ribosomal_L22 (pfam00237)→ RpsC (COG0092)rpsC (PRK00310)→<br>RplP (COG0197)rplP (PRK09203)→
env-605	CEOG01072571.1	-	582	507	**RNA**→ RplB (COG0090)rplB (PRK09374)→
env-606	JGI11755J13075_100039	+	3752	3827	**RNA**→ RplB (COG0090)rplB (PRK09374)→ rpsS (PRK00357)RpsS (COG0185)→<br>rplV (PRK00565)Ribosomal_L22 (pfam00237)→ RpsC (COG0092)rpsC (PRK00310)→<br>RplP (COG0197)rplP (PRK09203)→
env-607	JGI11949J13268_1000009	+	3765	3840	**RNA**→ RplB (COG0090)rplB (PRK09374)→ rpsS (PRK00357)RpsS (COG0185)→<br>rplV (PRK00565)Ribosomal_L22 (pfam00237)→ RpsC (COG0092)rpsC (PRK00310)→<br>RplP (COG0197)rplP (PRK09203)→
env-608	CEQG01048819.1	-	2905	2830	**RNA**→ RplB (COG0090)rplB (PRK09374)→ rpsS (PRK00357)RpsS (COG0185)→<br>rplV (PRK00565)Ribosomal_L22 (pfam00237)→ RpsC (COG0092)rpsC (PRK00310)→<br>RplP (COG0197)rplP (PRK09203)→
env-609	CEVJ01067098.1	+	321	396	**RNA**→ RplB (COG0090)rplB (PRK09374)→
env-610	CAM_READ_0079652197	+	1	76	**RNA**→
env-611	SL_1KL_011_SED_10748645	+	106	170	**RNA**→ RplB (COG0090)rplB (PRK09374)→
env-612	SL_9KL_010_SED_10060232	-	1582	1518	**RNA**→ RplB (COG0090)rplB (PRK09374)→ rpsS (PRK00357)RpsS (COG0185)→<br>rplV (PRK00565)Ribosomal_L22 (pfam00237)→
env-613	JGI24025J20009_10228543	-	185	112	**RNA**→ RplB (COG0090)rplB (PRK09374)→
env-614	JGI24724J26744_10018762	+	3389	3462	**RNA**→
Oin-1-1	NZ_ABID01000001.1	+	1417271	1417344	**RNA**→ RplB (COG0090)rplB (PRK09374)→ RplB (COG0090)rplB (PRK09374)→<br>rpsS (PRK00357)RpsS (COG0185)→ rpsS (PRK00357)RpsS (COG0185)→<br>rplV (PRK00565)Ribosomal_L22 (pfam00237)→ rplV (PRK00565)Ribosomal_L22 (pfam00237)→<br>RpsC (COG0092)rpsC (PRK00310)→ RpsC (COG0092)rpsC (PRK00310)→ RplP (COG0197)rplP (PRK09203)→<br>RplP (COG0197)rplP (PRK09203)→
env-615	LAZR01017474.1	+	457	530	**RNA**→ RplB (COG0090)rplB (PRK09374)→ rpsS (PRK00357)RpsS (COG0185)→<br>rplV (PRK00565)Ribosomal_L22 (pfam00237)→ RpsC (COG0092)rpsC (PRK00310)→
env-616	JGI24724J26744_10081951	+	450	522	**RNA**→ RplB (COG0090)rplB (PRK09374)→

env-617	ABEF01021384.1	-	622	549	**RNA**→ RplB (COG0090)rplB (PRK09374)→
env-618	CEQG01019720.1	+	5395	5468	**RNA**→ RplB (COG0090)rplB (PRK09374)→ rpsS (PRK00357)RpsS (COG0185)→ rplV (PRK00565)Ribosomal_L22 (pfam00237)→ RpsC (COG0092)rpsC (PRK00310)→ RplP (COG0197)rplP (PRK09203)→
env-619	CEQZ01013875.1	-	101	28	**RNA**→
env-620	CESC01001606.1	-	1753	1680	**RNA**→ RplB (COG0090)rplB (PRK09374)→ rpsS (PRK00357)RpsS (COG0185)→ rplV (PRK00565)Ribosomal_L22 (pfam00237)→ rpsC (PRK00310)30S_S3_KH (cd02412)→
env-621	CEVI01215114.1	-	178	105	**RNA**→ RplB (COG0090)rplB (PRK09374)→
env-622	CEWF01005413.1	+	2525	2598	**RNA**→ RplB (COG0090)rplB (PRK09374)→ rpsS (PRK00357)RpsS (COG0185)→ rplV (PRK00565)Ribosomal_L22 (pfam00237)→ rpsC (PRK00310)30S_S3_KH (cd02412)→
env-623	CERO01018958.1	-	2363	2290	**RNA**→ RplB (COG0090)rplB (PRK09374)→ rpsS (PRK00357)RpsS (COG0185)→ rplV (PRK00565)Ribosomal_L22 (pfam00237)→ RpsC (COG0092)rpsC (PRK00310)→
env-624	CEVA01074518.1	+	66	139	**RNA**→ rplB (PRK09374)Ribosomal_L2 (pfam00181)→
env-625	JGI24724J26744_10034861	+	2147	2220	**RNA**→
env-626	JGI24723J26617_10013494	+	1334	1407	**RNA**→ RplB (COG0090)rplB (PRK09374)→ rpsS (PRK00357)RpsS (COG0185)→ rplV (PRK00565)Ribosomal_L22 (pfam00237)→ RpsC (COG0092)rpsC (PRK00310)→ RplP (COG0197)rplP (PRK09203)→
env-627	LAZR01008751.1	+	1162	1237	**RNA**→ RplB (COG0090)rplB (PRK09374)→ rpsS (PRK00357)RpsS (COG0185)→ rplV (PRK00565)Ribosomal_L22 (pfam00237)→ RpsC (COG0092)rpsC (PRK00310)→ RplP (COG0197)rplP (PRK09203)→
env-628	JGI24724J26744_10022772	+	2527	2568	**RNA**→ RplB (COG0090)rplB (PRK09374)→
env-629	JGI24724J26744_10281221	+	457	498	**RNA**→ ←hypo
Sdo-1-1	NZ_JASF01000005.1	+	1880338	1880379	**RNA**→ RplB (COG0090)rplB (PRK09374)→ rpsS (PRK00357)RpsS (COG0185)→ rplV (PRK00565)Ribosomal_L22 (pfam00237)→ RpsC (COG0092)rpsC (PRK00310)→ RplP (COG0197)rplP (PRK09203)→
Sdo-1-2	NZ_JAMC01000005.1	+	6216	6257	**RNA**→ RplB (COG0090)rplB (PRK09374)→ rpsS (PRK00357)RpsS (COG0185)→ rplV (PRK00565)Ribosomal_L22 (pfam00237)→ RpsC (COG0092)rpsC (PRK00310)→ RplP (COG0197)rplP (PRK09203)→
env-630	KVRMV2_105402183	-	158	94	**RNA**→ RplB (COG0090)rplB (PRK09374)→
env-631	KVRMV2_100053128	+	1810	1873	**RNA**→ RplB (COG0090)rplB (PRK09374)→ rpsS (PRK00357)RpsS (COG0185)→ rplV (PRK00565)Ribosomal_L22 (pfam00237)→
env-632	KVRMV2_100511851	+	614	679	**RNA**→ RplB (COG0090)rplB (PRK09374)→ rpsS (PRK00357)RpsS (COG0185)→
env-633	3300001592_10006696	-	11253	11192	**RNA**→ RplB (COG0090)rplB (PRK09374)→ rpsS (PRK00357)RpsS (COG0185)→ rplV (PRK00565)Ribosomal_L22 (pfam00237)→ RpsC (COG0092)rpsC (PRK00310)→ RplP (COG0197)rplP (PRK09203)→
env-634	JGI24770J26754_10633113	-	235	176	**RNA**→ RplB (COG0090)rplB (PRK09374)→
env-635	JGI24770J26754_10084963	-	885	824	**RNA**→ RplB (COG0090)rplB (PRK09374)→
env-636	APMI01044789.1	+	283	344	**RNA**→ RplB (COG0090)rplB (PRK09374)→
env-637	JGI24770J26754_10316505	+	277	338	**RNA**→ RplB (COG0090)rplB (PRK09374)→
Lme-1-1	NC_023135.1	+	198601	198709	**RNA**→ RplB (COG0090)rplB (PRK09374)→ rpsS (PRK00357)RpsS (COG0185)→ rplV (PRK00565)Ribosomal_L22 (pfam00237)→ hypo→ RpsC (COG0092)rpsC (PRK00310)→ RplP (COG0197)rplP (PRK09203)→
env-638	CAM_READ_0234721741	-	220	152	**RNA**→ RplB (COG0090)rplB (PRK09374)→
env-639	84546017	-	387	322	**RNA**→ rplB (PRK09374)Ribosomal_L2 (pfam00181)→
Rba-1-1	NZ_AVDB01000002.1	+	2929	2994	**RNA**→ RplB (COG0090)rplB (PRK09374)→ rpsS (PRK00357)RpsS (COG0185)→ rplV (PRK00565)Ribosomal_L22 (pfam00237)→ RpsC (COG0092)rpsC (PRK00310)→ RplP (COG0197)rplP (PRK09203)→
env-640	84518656	-	774	709	**RNA**→ RplB (COG0090)rplB (PRK09374)→
env-641	CAM_READ_0234724723	+	190	256	**RNA**→ rplB (PRK09374)Ribosomal_L2 (pfam00181)→
env-642	CAM_READ_0235767883	+	190	256	**RNA**→ RplB (COG0090)rplB (PRK09374)→
env-643	JCVI_SCAF_1101667302592	-	664	599	**RNA**→ RplB (COG0090)rplB (PRK09374)→
env-644	CAM_READ_0236589595	-	346	280	**RNA**→ rplB (PRK09374)Ribosomal_L2 (pfam00181)→
env-645	84547844	+	884	948	**RNA**→
env-646	CAM_READ_0236055591	-	90	24	**RNA**→ hypo→
env-647	CAM_READ_0236222577	-	217	152	**RNA**→ RplB (COG0090)rplB (PRK09374)→

env-648	CAM_READ_0235239301	+	303	368	**RNA**→
env-649	JCVI_READ_1101854610991	+	341	406	**RNA**→ rplB (PRK09374)Ribosomal_L2 (pfam00181)→
env-650	CAM_READ_0235244455	+	234	302	**RNA**→
Wma-1-1	NZ_KB902287.1	-	74480	74410	**RNA**→ RplB (COG0090)rplB (PRK09374)→ rpsS (PRK00357)RpsS (COG0185)→ rplV (PRK00565)Ribosomal_L22 (pfam00237)→ RpsC (COG0092)rpsC (PRK00310)→ RplP (COG0197)rplP (PRK09203)→
env-651	JCVI_READ_1108839274073	+	792	857	**RNA**→
env-652	JGI11829J13087_100089	+	1021	1083	**RNA**→ RplB (COG0090)rplB (PRK09374)→ rpsS (PRK00357)RpsS (COG0185)→ rplV (PRK00565)Ribosomal_L22 (pfam00237)→ RpsC (COG0092)rpsC (PRK00310)→ RplP (COG0197)rplP (PRK09203)→ hypo→
Mal-2-1	NZ_CP010855.1	-	3005866	3005804	**RNA**→ RplB (COG0090)rplB (PRK09374)→ rpsS (PRK00357)RpsS (COG0185)→ rplV (PRK00565)Ribosomal_L22 (pfam00237)→ RpsC (COG0092)rpsC (PRK00310)→ RplP (COG0197)rplP (PRK09203)→
env-653	JGI12120J13076_116087	+	62	124	**RNA**→ RplB (COG0090)rplB (PRK09374)→
env-654	JGI11929J13085_103365	-	387	325	**RNA**→ rplB (PRK09374)Ribosomal_L2 (pfam00181)→
env-655	JGI11819J13099_125412	+	134	196	**RNA**→
env-656	JGI12210J13797_10226873	-	121	59	**RNA**→ hypo→
Pga-3-1	NC_023137.1	+	301203	301281	**RNA**→ RplB (COG0090)rplB (PRK09374)→ rpsS (PRK00357)RpsS (COG0185)→ rplV (PRK00565)Ribosomal_L22 (pfam00237)→ RpsC (COG0092)rpsC (PRK00310)→ RplP (COG0197)rplP (PRK09203)→

1.3 Conserved domains

Conserved domains found in protein-coding genes listed in Section 1.2 are shown below, with the first sentence in their description from the Conserved Domain Database (if any). Conserved domains associated with more than one L2-Alphaproteobacteria RNA are assigned a color, while others are shown in gray. The number in parentheses after the colored domain name is the number of occurrences in Section 1.2.

cd00719 (1) GIY-YIG nuclease domain superfamily.
cd01658 (4) Ribosomal protein L30, which is found in eukaryotes and prokaryotes but not in archaea, is one of the smallest ribosomal proteins with a molecular mass of about 7kDa.
cd02412 (29) K homology RNA-binding (KH) domain of the prokaryotic 30S small ribosomal subunit protein S3.
cd03886 (1) M20 Peptidase Aminoacylase 1 family.
COG0090 (567) Ribosomal protein L2 [Translation, ribosomal structure and biogenesis]
COG0091 (13) Ribosomal protein L22 [Translation, ribosomal structure and biogenesis]
COG0092 (310) Ribosomal protein S3 [Translation, ribosomal structure and biogenesis]
COG0094 (5) Ribosomal protein L5 [Translation, ribosomal structure and biogenesis]
COG0097 (5) Ribosomal protein L6P/L9E [Translation, ribosomal structure and biogenesis]
COG0098 (4) Ribosomal protein S5 [Translation, ribosomal structure and biogenesis]
COG0185 (365) Ribosomal protein S19 [Translation, ribosomal structure and biogenesis]
COG0186 (1) Ribosomal protein S17 [Translation, ribosomal structure and biogenesis]
COG0197 (289) Ribosomal protein L16/L10AE [Translation, ribosomal structure and biogenesis]
COG0198 (5) Ribosomal protein L24 [Translation, ribosomal structure and biogenesis]
COG0256 (1) Ribosomal protein L18 [Translation, ribosomal structure and biogenesis]
COG0596 (3) Pimeloyl-ACP methyl ester carboxylesterase [Coenzyme transport and metabolism, General function prediction only]
COG1917 (1) Cupin domain protein related to quercetin dioxygenase [General function prediction only]
pfam00181 (107) Ribosomal Proteins L2, RNA binding domain.
pfam00189 (3) Ribosomal protein S3, C-terminal domain.
pfam00203 (7) Ribosomal protein S19.
pfam00237 (339) Ribosomal protein L22p/L17e.
pfam00238 (5) Ribosomal protein L14p/L23e.
pfam00252 (8) Ribosomal protein L16p/L10e.
pfam00253 (5) Ribosomal protein S14p/S29e.
pfam00276 (1) Ribosomal protein L23.
pfam00366 (4) Ribosomal protein S17.
pfam00410 (5) Ribosomal protein S8.
pfam00831 (5) Ribosomal L29 protein.
pfam00861 (4) Ribosomal L18 of archaea, bacteria, mitoch.
pfam03947 (3) Ribosomal Proteins L2, C-terminal domain.
pfam06248 (1) Centromere/kinetochore Zw10.
pfam12697 (3) Alpha/beta hydrolase family.
pfam13759 (4) Putative 2OG-Fe(II) oxygenase.
pfam16867 (1) Dimethlysulfonioproprionate lyase.
PRK00004 (5) 50S ribosomal protein L24; Reviewed
PRK00010 (5) 50S ribosomal protein L5; Validated
PRK00136 (5) 30S ribosomal protein S8; Validated
PRK00306 (5) 50S ribosomal protein L29; Reviewed
PRK00310 (337) 30S ribosomal protein S3; Reviewed
PRK00357 (372) 30S ribosomal protein S19; Reviewed

PRK00550 (4) 30S ribosomal protein S5; Validated
PRK00565 (353) 50S ribosomal protein L22; Reviewed
PRK05483 (5) 50S ribosomal protein L14; Validated
PRK05498 (5) 50S ribosomal protein L6; Validated
PRK05593 (5) 50S ribosomal protein L18; Reviewed
PRK05610 (5) 30S ribosomal protein S17; Reviewed
PRK05611 (4) 50S ribosomal protein L30; Reviewed
PRK05738 (2) 50S ribosomal protein L23; Reviewed
PRK08881 (5) 30S ribosomal protein S14; Reviewed
PRK09203 (297) 50S ribosomal protein L16; Reviewed
PRK09374 (677) 50S ribosomal protein L2; Validated
smart00322 (5) K homology RNA-binding domain.
smart00465 (1) GIY-YIG type nucleases (URI domain).
TIGR01891 (1) amidohydrolase. [Protein fate, Degradation of proteins, peptides, and glycopeptides]
TIGR02466 (1) conserved hypothetical protein. [Hypothetical proteins, Conserved]

1.4 Multiple-sequence alignment

Each L2-Alphaproteobacteria RNA is denoted by an abbreviation (like "Eco-1-1") that refers to a taxonomy given in Section 1.1. The alignment may include sequences containing the RNA motif, as well as flanking sequence. The L2-Alphaproteobacteria RNA itself is denoted by the line underneath marked 5′ and 3′ on either end. Nucleotides in flanking sequences (i.e., not part of the motif) are written in gray letters. Stems of predicted rho-independent transcription terminators, if any, are shaded yellow. (Note: terminator predictions have not been analyzed manually, and many are likely to be false positives. Terminator predictions are those of the RNIE software.) Nucleotides predicted to function (as DNA) as transcription-factor binding sites, if any, are shaded in green. (But note: these predictions are manually annotated, so they might be under-predicted.) Annotated start codons, if any, are shaded green. (Note: start codons are frequently misannotated, especially in environmental samples.) Nucleotides proposed to basepair as part of the consensus structure are shaded in color when they comprise Watson-Crick or G-U pairs. Otherwise they are shaded gray. Conserved stems are also indicated at the bottom of the alignment by angle brackets, where matching $<$ and $>$ denote base-paired columns. Below these angle brackets, the symbol "2" denotes base pairs exhibiting covariation according to the statistically well-founded R-scape method. "1" denotes base pairs exhibiting covariation according to R2R's simplistic method. "0" denotes base pairs that are not observed to mutate and "?" denotes base pairs that have a significant frequency of non-canonical nucleotides for Watson-Crick or G-U pairs ($>$ 5%). Below these base pair annotation is the consensus sequence: "R" = "A" or "G", "Y" = "C" or "U", red nucleotides: nucleotide identity conserved more than 97% of the time, black nucleotides: 90%, gray nucleotides: 75%, red circle (•): nucleotide is present 97% of the time, black circle (•): 90%, gray circle (•): 75%, white circle (•): 50%. All percentages of sequences just described (e.g. 97% conserved) assume that sequences have been weighted by the GSC algorithm implemented by the Infernal software package.

Duplicate sequences: the following putative homologs are not shown in the alignment because their sequences are identical to a homolog already shown: Cba-1-1, Gne-1-1, Kvu-2-1, Lca-1-1, Lsp-5-1, Lsp-9-1, Nig-1-1, Nit-1-1, Nit-1-2, Oin-1-1, Pfe-2-1, Rba-5-1, Rca-1-1, Rca-2-1, Rca-3-1, Rca-5-1, Rmo-2-1, Rnu-1-1, Rsp-20-1, Rsp-3-1, Rsp-6-1, Sdo-1-2, Spo-1-1, Ssp-1-1, Ssp-3-1, Tsp-2-1, env-100, env-102, env-107, env-11, env-110, env-111, env-112, env-113, env-114, env-115, env-12, env-123, env-126, env-129, env-130, env-131, env-132, env-133, env-134, env-137, env-140, env-142, env-147, env-152, env-153, env-154, env-159, env-160, env-161, env-180, env-181, env-182, env-183, env-184, env-185, env-198, env-199, env-208, env-209, env-210, env-211, env-212, env-238, env-239, env-240, env-241, env-242, env-243, env-244, env-245, env-246, env-249, env-250, env-251, env-253, env-267, env-268, env-270, env-28, env-281, env-283, env-29, env-292, env-294, env-30, env-302, env-303, env-304, env-305, env-306, env-307, env-31, env-318, env-32, env-328, env-33, env-333, env-334, env-34, env-348, env-349, env-350, env-351, env-352, env-353, env-354, env-355, env-366, env-368, env-376, env-377, env-383, env-384, env-385, env-387, env-390, env-393, env-401, env-406, env-408, env-409, env-410, env-411, env-412, env-415, env-420, env-424, env-425, env-44, env-442, env-443, env-444, env-446, env-449, env-45, env-450, env-451, env-452, env-453, env-454, env-455, env-456, env-457, env-46, env-461, env-462, env-466, env-468, env-470, env-471, env-474, env-478, env-479, env-48, env-480, env-481, env-485, env-486, env-487, env-488, env-489, env-490, env-491, env-492, env-496, env-498, env-499, env-501, env-504, env-509, env-510, env-511, env-529, env-53, env-532, env-535, env-537, env-538, env-539, env-54, env-540, env-541, env-542, env-543, env-544, env-545, env-547, env-550, env-551, env-552, env-553, env-554, env-555, env-556, env-557, env-558, env-559, env-560, env-561, env-562, env-563, env-564, env-565, env-566, env-568, env-569, env-570, env-571, env-572, env-573, env-574, env-575, env-577, env-578, env-58, env-580, env-581, env-584, env-585, env-586, env-587, env-588, env-589, env-593, env-594, env-599, env-601, env-602, env-603, env-604, env-607, env-608, env-609, env-61, env-610, env-619, env-62, env-620, env-621, env-622, env-63, env-642, env-654, env-655, env-73, env-80, env-81, env-82, env-87, env-95

alignment positions 1 · · · 180

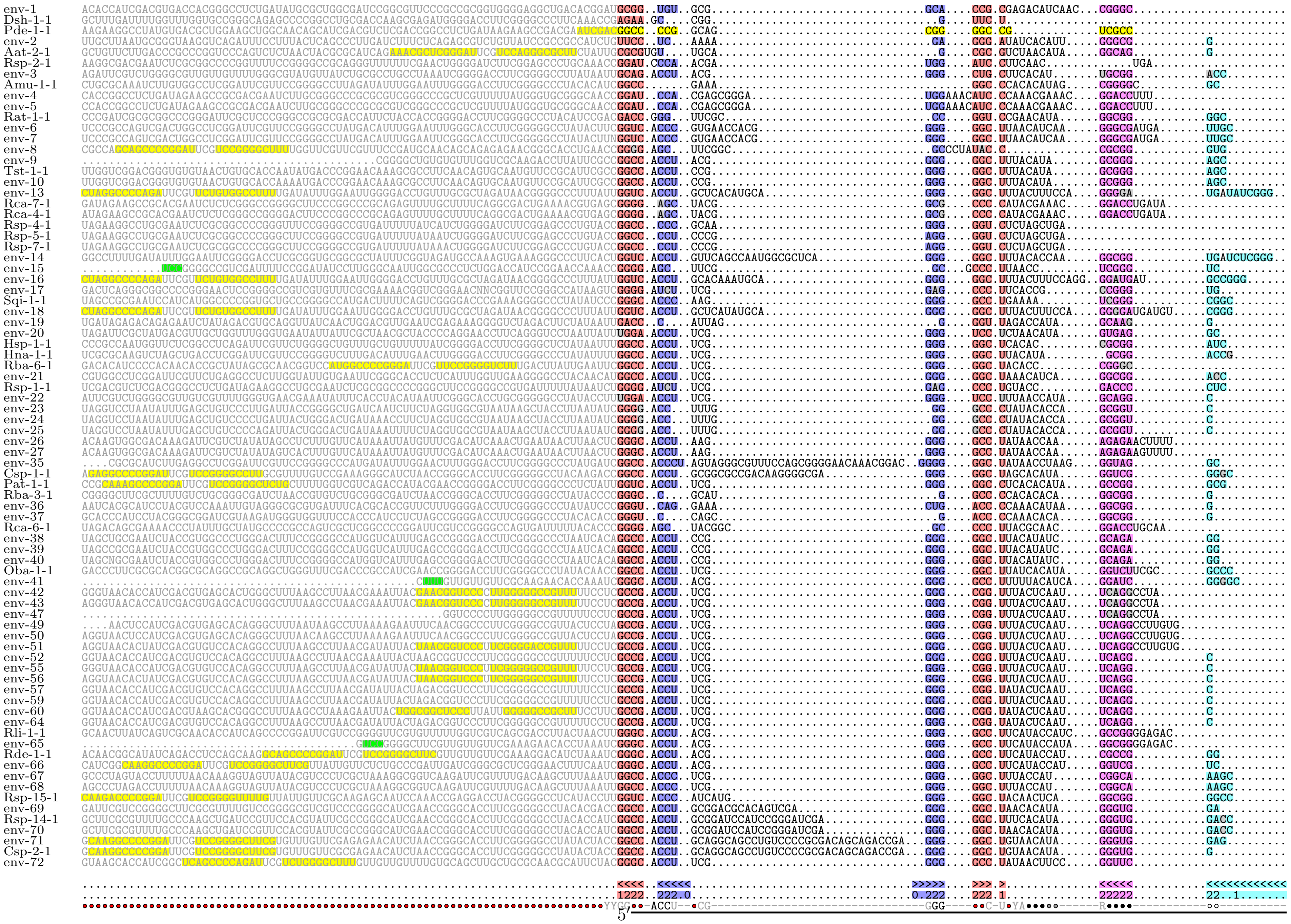

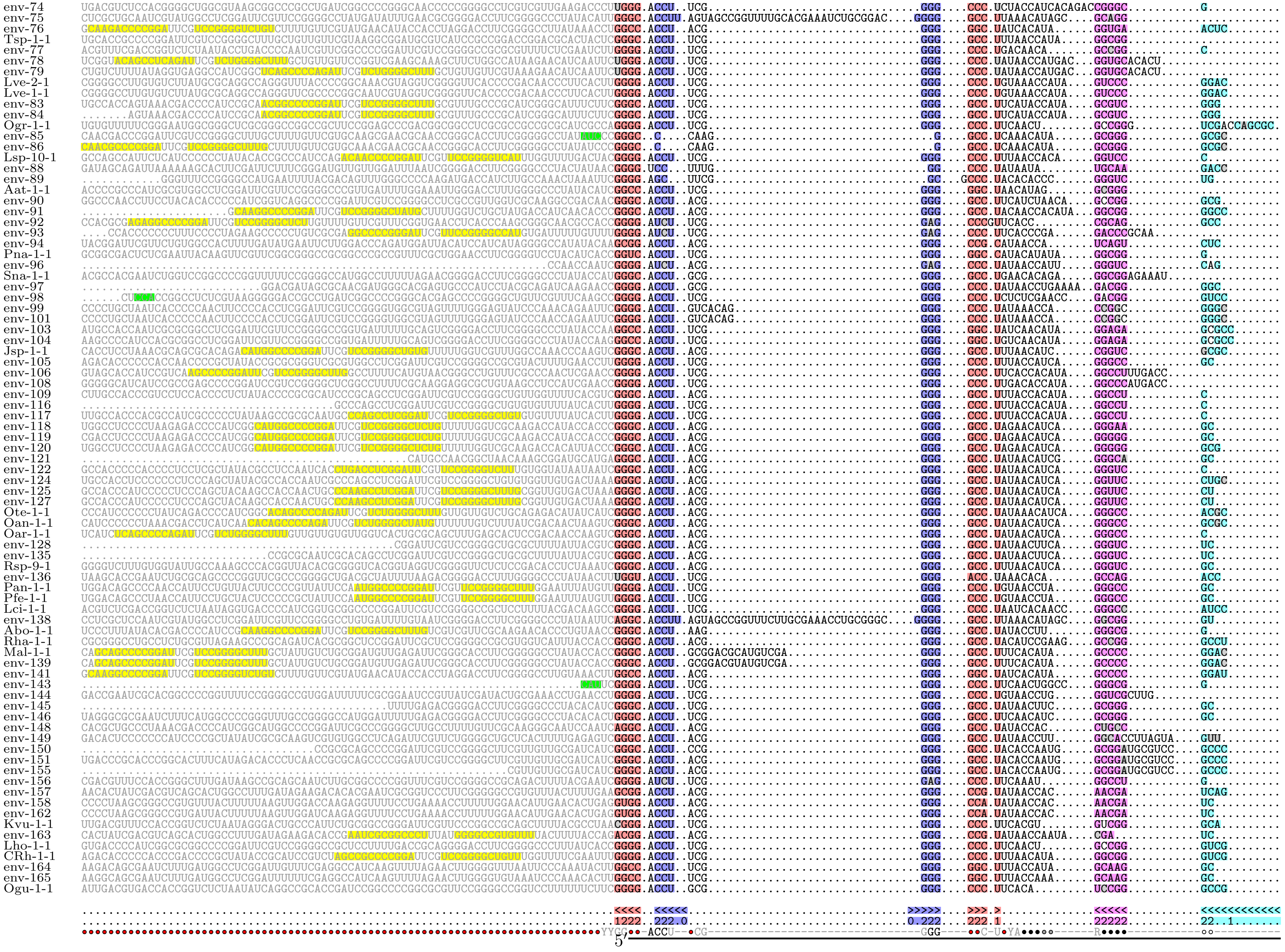
env-74
env-75
env-76
Tsp-1-1
env-77
env-78
env-79
Lve-2-1
Lve-1-1
env-83
env-84
Ogr-1-1
env-85
env-86
Lsp-10-1
env-88
env-89
Aat-1-1
env-90
env-91
env-92
env-93
env-94
Pna-1-1
env-96
Sna-1-1
env-97
env-98
env-99
env-101
env-103
env-104
Jsp-1-1
env-105
env-106
env-108
env-109
env-116
env-117
env-118
env-119
env-120
env-121
env-122
env-124
env-125
env-127
Ote-1-1
Oan-1-1
Oar-1-1
env-128
env-135
Rsp-9-1
env-136
Pan-1-1
Pfe-1-1
Lci-1-1
env-138
Abo-1-1
Rha-1-1
Mal-1-1
env-139
env-141
env-143
env-144
env-145
env-146
env-148
env-149
env-150
env-151
env-155
env-156
env-157
env-158
env-162
Kvu-1-1
env-163
Lho-1-1
CRh-1-1
env-164
env-165
Ogu-1-1
5′

```
env-166   UUGACGUCUCCACCGGGCUCGCCUAAGAGCCUCGCCUGACCGGCCCCGGGCAAACCCCGGGGCCUCGUCUUUUCCACUUGGGGG.ACCU..UCG................................GGG...CCC.UUCACAUUAA.....GCCGG...........GCC.........
Paq-1-1   GGCCUCGGGACUUCUCGGGGCCGUGGAUUUUUUCGCGAUGCAGGGUGUAGGCGACCGAUGCCUGACUUGCAAAGACCCCCGGGG.AUCU..ACG................................GAG...CCC.UAUAACCUU......GGCCA...........C...........
env-167   UCCCGCGCAUCUUGAGGCCCCGGAUUCGUUCCGGGGCCCAUGAUUUUUGGAAUUUGGGGACCUUUGGGGCCCUAUACAUCGGCC.ACCU..UCG................................GGG...GGC.UAACCAUA.......GGCAG...........G...........
env-168   AGAGCGCCAGUCCAAGGCCCCGGAUUCGUUCCGGGGCCUUGCGAUAUUGGAAUUCGGCCACCUUCGGGGGGCUACACACUGGGC.ACCU..GCGGACUUCGGUCGA....................GGG...GCC.UUACAACAUGA....GGCAG...........G...........
env-169   GCAAAGCCCCGGAUUCGUCCGGGGCUGUGCGUUUGUUCGCAAGAGCAUCUAACCGGGCACCUACGGGGGCCUAUAGACCAGGCC.ACCU..UCG................................GGG...GGC.UGUAAACAUA.....GGCAG...........G...........
env-170   .........................................AGAGCAUCUAACCGGGCACCUACGGGGGCCUAUAGACCAGGCC.ACCU..UCG................................GGG...GGC.UGUAAACAUA.....GGCAG...........G...........
env-171   ...........................AUAGAAGCCAGUCGAAUCACGGCCCCAAUCGGGGCCGUGUUUGACUUUUAGACGCGG.ACCU..ACG................................GGG...CCG.UUCACCAUU......GGCCCCU.......................
env-172   GUUUGGAAUAUUGGGCCAUCUGGCCCUUACACCGUGGACCUAGGGCGUUUUCAAAAACGCAUAAGGGGCCACAUAAAAUAGCGG.ACCU..ACG................................GGG...CCG.CGCACACUU......UCAGCC..........CUUGAA......
Rba-4-1   GUUUGGAAUAUUGGGCCAUCUGGCCCUUACACCGUGGACCUAGGGCGUUUUCAAAAACGCAUAAGGGGCCACUUAAAAUAGCGG.ACCU..ACG................................GGG...CCG.CGCACACUU......UCAGAC..........CUUGAA......
env-173   AAAGGGGUGGACAGUUUUGUUUUACCCCUUUAUAGGAACAAAACUUGAGCUUGUUCGGUAAGACCUAGCAAGAUUAAAUCGGCC.ACCU..CCG................................GGG...GGC.UUUACCAAA......GGCGGUUAAA.......................
Sps-1-1   GGCACCAGCCCCGGAUUCGUCCGGGGCUUUGCCUUUGUUCUUGGUCCAGACAUUAUUCUUGACCUCUGAGCACUCCAAACGGGG.ACCU..UCG................................GGG...CCC.UACAACCAC......GGCGA...........C...........
Ssp-2-1   GGCACCAGCCCCGGAUUCGUCCGGGGCUUUGCCUUUGUUCCUGGUCCAGACAUUAUUCUUGACCUCAGAACACUCCAAACGGGG.ACCU..UCG................................GGG...CCC.UACAACCAC......GGCGA...........C...........
env-174   CAAGGCCCCGGAUUCGUCCGGGGCUUCGCUGUUGUUCGCAAGAGCAACCCUAACCGGGGACCUUCGGGGCCCUAUAAACCGGCC.ACCUUUCAA..............................AAGGG...GGC.UUGAACAUA......GGCGGAGAU.......GCCC........
Osp-1-1   CUGAGCCCCGGAUUCGUCCGGGGCUUGCCUUUUGUUCGCAAGAACACACGAACCGGGCACCUGCGGGGGCUAUACCAUCGGCC.ACCU..ACG................................GGG...GGC.UAUCACAUA......GGCAG...........G...........
Sst-1-1   AACGGCCCCGGAUUCGUCCGGGGCUUCGCUUUUGCGCGGUGCGGACAGCGAAUCGGGCACCUUCGGGGGCUAUACCUCUGGCC.ACCU..UCG................................GGG...GGC.UAUUACAUA......AGCCG...........G...........
env-175   AAAGGGGUGGACAGUUUUGAUUUACCCCUUUAUACGGUCGAAAUCUUGGGAUUGUUCGAUUAAACCUCGCAGAUUAAAUUGGCC.ACCU..CUGG...............................GGG...GGC.UUUACCAAAC.....GGUGAAU........................
env-176   GCAAGGCCCCGGAUUCGUCCGGGGCUUCGCUAUUGUUCGCAAGAGCACUCCAACCGGGGACCUUCGGGGCCCUAAAUUCUGGCC.ACCU..UUG................................GGG...GGC.UACAACAUA......GGCGGGCAUGAU.....................
Rsp-16-1  CAAGGCCCCGGAUUCGUCCGGGGCUUCGCUGUUGUUCGCAAGGGCAACUCUAACCGGGGACCUACGGGGCCUCAUACCUUGGCC.ACCC..UCG................................GGG...GGC.UAUAACAUA......GGCGG...........GU..........
env-177   UAUCUCGCGCAGGUCGCGUGGCCUCGGAUUCGUUCCGGGGCCUGUGAUUUUGGGGUCGGGGUUACCUGAUUCCACUCUUGGGCC.ACCU..ACG................................GGG...GGC.UAUAACAGACA....GGCGG...........G...........
env-178   GACGGCCCGGGUCCACGGCCCCGGAUUCGCUCCGGGGCUGUCGAUUAUGGAAACGGGGUCCCCUCAGGGACCCGCGCUACGGCC.ACCU..UCG................................GGG...GGC.UACAACAGACA....GGCGG...........G...........
env-179   CAAGGCCCCGGAUUCGUCCGGGGCCUUGCAAUUGUUCGCAAGAGCAAUUCUAGCCGAGGACCUACGGGGCCUCAUACCUUGGCC.ACCC..UCG................................GGG...GGC.UACAACACA......GGCAG...........G...........
env-186   CAAGGCCCCGGAUUCGUCCGGGGCCUUGCAAUUGUUCGCAAGAGCAAUUCUAGCCGAGGACCUACGGGGCCUCAUACCUUGGCC.ACCC..UAG................................GGG...GGC.UACAACACA......GGCAG...........G...........
Rsp-13-1  CAAGGCCCCGGAUUCGUCCGGGGCCUUGCAAUUGUUCGCAAGAGCAAUUCUAGCCGAGGACCUACGGGGCCUCAUACCUUGGCC.ACCC..UCG................................GGG...GGC.UAUAACAUA......GGCGG...........G...........
env-187   .........................................GGGCAACUCUAACCGGGGACCUACGGGGCCUCAUACCUUGGCC.ACCC..UCG................................GGG...GGC.UAUAACAUA......GGCGG...........G...........
Rmu-1-1   CAAGGCCCCGGAUUCGUCCGGGGCUUCGCUGUUGUUCGCAAGGGCAACUCUAACCGGGGACCUACGGGGCCUCAUACCUUGGCC.ACCC..UCG................................GGG...GGC.UAUAACAUA......GGCGG...........G...........
env-188   GAAGCCGCGAAUCUUCGUGGCCCCGGGUGCGCCCGGGGCCACGGCUUUUGAACCGGGGACCUUCGGGGCCCUGUACACCUGGCC.ACCU..UCG................................GGG...GGC.UUACACAUA......GGCGGGCCCCU.....GGC.........
env-189   CCGUGCCCCGGAUUCGUCCGGGGCUCUGCUUUUGUUCGUAAGAACAGCAAGAACCGGGGACCUACGGGGCCUCAUACAUCGGCC.ACCU..UCG................................GGG...GGC.UAUUACAUA......GGCGG...........GGC.........
env-190   CCGUGCCCCGGAUUCGUCCGGGGCUCUGCUUUUGUUCGUAAGAACAGCAAUAACCGGGGACCUACGGGGCCUCAUAAACCGGCC.ACCU..UCG................................GGG...GGC.UAUUACAUA......GGCGG...........GGC.........
env-191   GUCGCGACUCAUCUUUGGCCCCGGCUUUGCCCGGGGCCACGGAUUUUGAACCGGGGGACCCACGGGGGCCUCAUACACUCGGCC.ACCU..UCG................................GGG...GGC.UAUUACAUA......GGCGG...........GGC.........
env-192   CUUGCCCCGGAUUCGUCCGGGGCUCUGCGUUUGUUCGAAAGAACACCAAAAACCGGGGACCUACGGGGCCUCAUACCAUCGGCC.ACCU..UCG................................GGG...GGC.UAUCACAUA......GGCAG...........GGC.........
env-193   AAGCCCUAGACCUUUUUGACAAAGGUAGUUAUACGUCCGUCGCUGAAGCGGUCAAGAUUCGUUUUGACAAGCUUUAAAUUGGCC.ACCU..UCG................................GGG...GGC.UUUACCAUC......GGCAA...........GG..........
env-194   AAGCCCUAGACCUUUGUAUCAAAGGCGGUUAUACGUCCGUCGCUAGAGCGGUCAAGAUUCUUUUUGACUGGCUUUUAAUCGGCC.ACCU..UCG................................GGG...GGC.UUUACCAUA......GGCAA...........AGC.........
env-195   ...........................................UAGAGUGGUCAGGAUUCUUUUUGACUGGCUUUAAAUCGGCC.ACCU..UCG................................GGG...GGC.UUUACCAUA......GGCAA...........AGC.........
env-196   AAGCCCUAGACCUUUGUAUCAAAGGUGGUUAUACGUCCGUCGCUAGAGCAGUCAGGAUUCUUUUUGACUGGCUUUAAAUUGGCC.ACCU..UCG................................GGG...GGC.UUUACCAUA......GGCAA...........AGC.........
env-197   AAGCCCUAGACCUUUGUAUCAAAGGCGGUUAUACGUCCGUCGCUAGAGCGGUCAGGAUUCUUUUUGACUGGCUUUAAAUUGGCC.ACCU..UCG................................GGG...GGC.UUUACCAUA......GGCAA...........AGC.........
env-200   AAGCCCUAGACCUUUGUAUCAAAGGUGGUUAUACGUCCGUCGCUAGAGCAGUCAGGAUUCUUUUUGACUGGCUUUAAAUCGGCC.ACCU..UCG................................GGG...GGC.UUUACCAUA......GGCAA...........AGC.........
env-201   AAGCCCUAGACCUUUUUUAUAAAGGUAGUUAUAUGUCCUUCGCUGAAGCGGUCAAGAUUCGUUUUGACAAGCUUUAAAUCGGCC.ACCU..UCG................................GGG...GGC.UUUACCAUC......GGCAA...........AGU.........
env-202   AAGCUCUAGACCUUUUUAAUAAAGGUCGUUAUAUGUCCGUCGCUGAAACGGUCAAGAUUCGUUUUGACAAGAUAUAAACUGGCC.ACCU..UCG................................GGG...GGC.UUUACCAUC......GGCAA...........GGU.........
env-203   AAGCUCUAGACCUUUUUAAUAAAGGUCGUUAUAUGUCCGUCGCUGAAGCGGUCAAGAUUCGUUUUGACAAGAUAUAAACUGGCC.ACCU..UCG................................GGG...GGC.UUUACCAUC......GGCAA...........GGU.........
env-204   AAGCCCUAGACCUUUUUAAUAAAGGUCGUUAUAUGUCCGUCGCUGAAGCGGUCAAGAUUCGUUCUGACAAGCUUUAAAUCGGCC.ACCU..CCG................................GGG...GGC.UUUACCAUA......GGCAA...........AGU.........
env-205   AAGCCCUAGACCUUUUUAGUAAAGGUAGUUAUACGUCCGUCGCUGGAGCGGUCAAGAUUCGUUUUGACAAGCUUUAAAUUGGCC.ACCU..UAG................................GGG...GGC.UUUACCAUA......GGCAA...........AGC.........
env-206   AAGCCCUAGACCUUUUUAACAAAGGUAGUUAUACGUCCGUCGCUGAAGCGGUCAAGAUUCGUUUUGACAAGCUUUAAAUCGGCC.ACCU..UCG................................GGG...GGC.UUUACCAUA......GGCAA...........AGC.........
env-207   AAGCCCUAGACCUUUUUAACAAAGGUAGUUAUACGUCCGUCGCUGAAGCGGUCAAGAUUCGUUUUGACAAGCUUUAAAUUGGCC.ACCU..UCG................................GGG...GGC.UUUACCAUA......GGCAA...........AGC.........
env-213   AAGCCCUAGACCUUUUUAACAAAGGUAGUUAUACGUCCUUCGCUGAAGCGGUCAAGAUUCGUUUUGACAGGCUUUAAAUCGGCC.ACCU..UCG................................GGG...GGC.UUUACCAUA......GGCAA...........AGC.........
env-214   AAGCCCUAGACCUUUUUAACAAAGGUAGUUAUACGUCCUUCGCUGAAGCGGUCAAGAUUCGUUUUGACAGGCUUUAAAUCGGCC.ACCU..UCG................................GGG...GGC.UUUACCAUC......GGCAA...........AGC.........
env-215   AAGCCCUAGACCUUUUUAAGAAAGGUAGUUAUACGUCCUUCGCUGAAGCGGUCAAGAUUCGUUUUGACAAGCUUUAAAUCGGCC.ACCU..UCG................................GGG...GGC.UUUACCAUC......GGCAA...........AGC.........
env-216   UAGCCCUAGACCUUUGUAACAAAGGUAGUUAUACGUCCUUCGCUAAAGCGGUCAAGAUUCGCUUUGACUAGUUUUAAAUCGGCC.ACCC..UCG................................GGG...GGC.UUUACCAUC......GGCAA...........AGC.........
env-217   AAGCCCUAGACCUUUUUAACAAAGGUAGUUAUACGUCCUUCGCUGAAGCGGUCAAGAUUCGUUUUGACUAGCUUUAAUUCGGCC.ACCC..UCG................................GGG...GGC.UUUACCACA......GGCAA...........AGC.........
env-218   AAGCCCUAGACCUUUUUAACAAAGGUGGUUAUACGUCCUACGCUGAAGCGGUCAAGAUUCGUUUUGACUAGCUUUAAAUUGGCC.ACCC..UCG................................GGG...GGC.UUUACCACA......GGCGG...........AGC.........
env-219   AAGCCCUAGACCUUUUUAACAAAGGUAGUUAUACGUCCUUCGCUGAAGCGGUCAAGAUUCGUUUUGACUAGCUUUAAUUCGGCC.ACCC..UCG................................GGG...GGC.UUUACCACC......GGCAG...........AGC.........
env-220   GAGCCCUAGACCUUUUUAACAAAGGUAGUUAUACGUCCUUCGCUGAAGCGGUCAAGAUUCGUUUUGACUAGCUUUAAUUCGGCC.ACCC..UCG................................GGG...GGC.UUUACCACC......GGCAG...........AGC.........
env-221   AAGCCCUAGACCUUUUUAACAAAGGUAGUUAUACGUCCUUCGCUGAGGCGGUCAAGAUUCGUUUUGACUAGCUUUAAAUCGGCC.ACCC..UCG................................GGG...GGC.UUUACCACC......GGCAA...........AGC.........
env-222   AAGCCCUAGACCUUUUUAACAAAGGUAGUUAUACGUCCGUCUCUGAAGCGGUCAAGAUUCGUUUUGGCUAGCUUUAAAUCGGCC.ACCC..UCG................................GGG...GGC.UUUACCACC......GGCAA...........AGC.........
env-223   AAGCCCUAGACCUUUUUAACAAAGGUAGUUAUACGUCCUUCGCUGAAGCGGUCAAGAUUCGUUUUGACUAGCUUUAAUUCGGCC.ACCC..UCG................................GGG...GGC.UUUACCACC......GGCAA...........AGC.........
env-224   AAGCCCUAGACCUUUUUAACAAAGGAAGUUAUACGUCCCUCGCUGAAGCGGUCAAGAUUCGUUUUGACUAGCUUUAAAUCGGCC.ACCC..UCG................................GGG...GGC.UUUACCACC......GGCAA...........AGC.........
env-225   GAGCCCUAGACCUUUUUAACAAAGGUAGUUAUACGUCCUUCGCUGAAGCGGUCAAGAUUCGUUUUGACUAGCUUUAAUUCGGCC.ACCC..UCG................................GGG...GGC.UUUACCACC......GGCAA...........AGC.........
env-226   GAGCCCUAGACCUUUUUAACAAAGGUAGUUAUACGUCCUUCGCUGAAGCGGUUAAGAUUCGUUUUGACUAGCUUUAAAUCGGCC.ACCC..UCG................................GGG...GGC.UUUACCACC......GGCAA...........AGC.........
env-227   AAGCCCUAGACCUUUUUAACAAAGGUAGUUAUACGUCCUUCGCUGAAGCGGUUAAGAUUCGUUUUGACUAGCUUUAAAUCGGCC.ACCC..UCG................................GGG...GGC.UUUACCUCC......GGCAA...........AGC.........
env-228   AAGCCCUAGACCUUUGUAUCAAAGGCGGUUAUACGUCCGUCGCUAGAGCGGUCAGGAUUCUUUUUGACUGGCUUUAAAUUGGCC.ACCUU.UCG...............................GGGG...GGC.UUUUACCAUA.....GGCAA...........AGC.........
env-229   AAGCCCUAGACCUUUUUUAUAAAGGUAGCUAUACGUCCUUCGCUAAAGCAAUCAAGAUUCGUUUUGAUUUGCAUCAAAUUGGCC.ACCU..CAG................................GGG...GGC.UUUACCAUU......GGCAG...........AGUA........
env-230   AAGCCCUAGACCUUUUUUAUAAAGGUAGCUAUACGUCCUUCGCUAAAGCAAUCAAGAUUCGUUUUGAUUUGCUUCAAAUUGGCC.ACC...UCAG................................GG...GGC.UUUACCAUU......GGCAG...........AGU.........
env-231   AAGCCCUAGACCUUUUUUAUAAAGAUAGCUAUACGUCCUUCGCUAAAGCAAUCAAGAUUCGUUUUGAUUUGCUUUAAAUCGGCC.ACCU..UAG................................GGG...GGC.UUUACCAUA......GGCAA...........AGU.........
env-232   ..GCCCUAGACCUUUUUUAUAAAGGUAGCUAUACGUCCUUCGCUAAAGCGAUCAAGAUUCGUUUUGAUUUGCUUAAAAUCGGCC.ACCU..UAG................................GGG...GGC.UUCACCAUA......GGCAG...........AGU.........
env-233   AAGCCCUAGACCUUUUUUAUAAAGGUGGCUAUAUGUCCUUCGCUACAGCAAUCAAGAUUCGUUUUGAGUUGCUUCAAAUUGGCC.ACCU..UCG................................GGG...GGC.UUCACCAUA......GGCAG...........AGU.........
env-234   ....................AAAGGUAGCUAUACGUCCUUCGCUAAAGCAAUCAAGAUUCGUUUUGAGUUGCUUAAAAUUGGCC.ACCU..UCG................................GGG...GGC.UUUACCAUA......GGCAG...........AGU.........
env-235   AAGCUCUAGACCUUUUUUAUAAAGGUAGCUAUACGUCCCUCGCUAAGGCAAUCAAGAUUCGUUUUGAUUUGCUUUAAAUCGGCC.ACCU..UCG................................GGG...GGC.UUUACCAUA......GGCAG...........AGU.........
env-236   AAGCCCUAGACCUUUUUAAUAAAGGUAGCUAUACGUCCUUCGCUAAAGCAAUCAAGAUUCGUUUUGAUCUGCUUUAAAUCGGCC.ACCU..UAG................................GGG...GGC.UUUACCAUA......GGCAG...........AGU.........
env-237   AAGCCCUAGACCUUUUUAAUAAAGGUAGCUAUACGUCCUUCGCUAAAGCAAUCAAGAUUCGUUUUGAUUUGCUUUAAAUCGGCC.ACCU..UAG................................GGG...GGC.UUUACCAUA......GGCAG...........AGU.........
env-247   AAGCCCUAGACCUUUUUUAUAAAGAUAGCUAUACGUCCUUCGCUAAAGCAAUCAAGAUUCGUUUUGAUUUGCUUUAAAUCGGCC.ACCU..UAG................................GGG...GGC.UUUACCAUA......GGCAG...........AGU.........
env-248   AAGCCCUAGACCUUUUUUAUAAAGGUAGCUAUACGUCCUUCGCUAAAGCAAUCAAGAUUCGUUUUGAUUUGCUUCAAAUUGGCC.ACCC..CAG................................GGG...GGC.UUUACCAUU......GGCAG...........AGU.........
env-252   AAGCCCUAGACCUUUUUUAUAAAGGUAGCUAUACGUCCUUCGCUAAAGCAAUCAAGAUUCGUUUUGAUUUGCUUCAAAUUGGCC.ACCU..CAG................................GGG...GGC.UUUACCAUA......GGCAG...........AGU.........
env-254   AAGCCCUAGACCUUUUUUAUAAAGGUAGCUAUACGUCCUUCGCUAAAGCAAUCAAGAUUCGUUUUGAUUUGCUUCAAAUUGGCC.ACCU..CAG................................GGG...GGC.UUUACCAUA......GGCAG...........AGU.........
env-255   AAGCCCUAGACCUUUUUUAUAAAGGUAGCUAUACGUCCUUCGCUAAAGCAAUCAAGAUUCGUUUUGAUUUGCUUCAAAUUGGCC.ACCU..CAG................................GGG...GGC.UUUACCAUA......GGCAG...........AGU.........
env-256   AGCCCUAGACCUUUUUUAUUAAAGGUAGCUAUACGUCCGUCGCUGAAGCAAUCAAGAUUCGUUUUGGUUUGCUUCAAAUUGGCC.ACCU..CAGG...............................GGG...GGCGUUUACCAUA......GGCAG...........AGU.........
env-257   AAGCCCUAGACCUUUUUUAUAAAGGUAGCUAUACGUCCCUCGCUACAGCAAUCAAGAUUCGUUUUGAUUUGCUUCAAAUUGGCC.ACCU..CAG................................GGG...GGC.UUUACCAUA......GGCAG...........AGU.........
env-258   AAGCCCUAGACCUUUUUUAUAAAGGUAGCUAUACGUCCUUCGCUAAAGCAAUCAAGAUUCGUUUUGAUUUGCUUCAAAUUGGCC.ACCU..CAG................................GGG...GGC.UUUACCAUA......GGCAG...........AGU.........
env-259   GCAAGGCCCCGGAUUCGUCCGGGGCUUCGCUAUUGUUCGCAAGAGCACUCCAACCGGGGACCUUCGGGGCCCUAAAUUCUGGCC.ACCU..UCG................................GGG...GGC.UACAACAUA......GGCGG...........G...........
Rsp-8-1   GAAGCCGCGACUCGCGUGGCCCCGGGCAGUUCCCGGGGCCACGGUUUUUGAACCGGGGACCUUCGGGGCCCUAUGAUACCGGCC.ACCU..UCG................................GGG...GGC.UUAACCAUA......GGCGGAUU........UGCC........
env-260   .AAGGCAACGAAUCGCAGCAGCUCCAAGCCUCGCUUGGGGCUGCGUCGUUUUAAGAACGGGAUCUUUUUGAUCCAAAUUUGGCC.ACCU..UCG................................GGG...GGC.UUCACCAUA......GGCAGAGUUU.......................
env-261   ........AGAAUCGUAGCAGCUCCAAGCCUCGCUUGGGGCUGCGUCGUUUUAAGAACGGGAUCUCUUUGGUCCAAAUUUGGCC.ACCU..UUG................................GGG...GGC.UUCACCAUA......GGCAGAGUAU.......................
env-262   GAAGGCAACGAAUCGUAGCAGCUCCAAGCCUCGCUUGGGGCUGCGUCGUUUUAAGAACGGGAUCUCUUUGGUCCAACUUUGGCC.ACCU..UUG................................GGG...GGC.UUCACCAUA......GGCAGAGUAU.......................
env-263   AAAGGCAACGAAUCGUAGCAGCUCCAAGCCUCGCUUGGGGCUGCGUCGUUUUAAGAACGGGAUCUCUUUGGUCCAACUUUGGCC.ACCU..UUG................................GGG...GGC.UUCACCAUA......GGCAGAGUAU.......................
env-264   AGGCAACGAAUCGUAGCAGCUCCAAGCCUCGCUUGGGGCUGCAUCGUUUUAAGAACGGGAUCUUUUUUUGGUCCAAAUUUGGCN.ACCU..UCG................................GGG...GGC.UUCACCAUA......GGCAGAGUAU.......................

          ................................................................................<<<<..<<<<<.................................>>>>>...>>>.>..............<<<<<...........<<<<<<<<<<<<
          ................................................................................1222..222.0.................................0.222...222.1..............22222...........22..1.......
          ●●●●●●●●●●●●●●●●●●●●●●●●●●●●●●●●●●●●●●●●●●●●●●●●●●●●●●●●●●●●●●●●●●●●●●●●●●●●YYGG●●-ACCU--●CG-------------------------------GGG---●●C-U●YA●●●●○○------R●●●●-----------○○---------
                                                                                  5′
```

```
env-265   GAAGGCAAUGAAUCGUAGCAGCUCCAAGCCUCGCUUGGGGCUGCGUCGUUUUAAGAACGGGAUCUUUUUGGUCCAAAUUUGGCC.ACCU..UCG................................GGG....GGC.UUCACCAUA......GGCAGAGUAU..................
env-266   AGGCAACGAAUCGUAGCAGCUCCAAGCCUCGCUUGGGGCUGCGUCGUUUUAAGAACGGGAUCUUUUUUUGGUCCAAAUUUGGCC.ACCU..UCG................................GGG....GGC.UUCACCAUA......GGCAGAGUAU..................
env-269   GAAGGCAACGAAUCGCAGCAGCUCCAAGCCUCGCUUGGGGCUGCGUCGUUUUAAGAACGGGAUCUUUUUGAUCCAAAUUUGGCC.ACCU..UCG................................GGG....GGC.UUCACCAUA......GGCAGAGUAU..................
env-271   GAAGGCAACGAAUCGUAGCAGCUCCAAGCCUCGCUUGGGGCUGCGUCGUUUUAAGAACGGGAUCUUUUUGGUCCAAAUUUGGCC.ACCU..UCG................................GGG....GGC.UUCACCAUA......GGCAGAGUAU..................
env-272   ...........................CUCGCUUGGGGCUGCCUCGUUUUAAGAACGGGAUCUUUUUUUGGUCCAAAUUUGGCC.ACCU..UCG................................GGG....GGC.UUCACCAUA......GGCAGAGUAU..................
env-273   AAAGGCAACGAAUCGCAGCAGCUCCAAGCCUCGCUUGGGGCUGCGUCGUUUUAAGAACGGGAUCUUUUUGAUCCAAAUUUGGCC.ACCU..UCG................................GGG....GGC.UUCACCAUA......GGCAGAGUUU..................
env-274   UGCACUACAAAAAUUAGGGGUGGACAGUUUGGUUACCCUUAUACGACGAAUCUGGGUGUAAAGAAUAUCGCCUAACAACCGGCC..CC...UUCG...............................GG....GGC.UUACCAAA.......GGCGGUAGU...................
env-275   AAGGGGUGGACAGUUGUGAUUUACCCCUUUAUAGGAACCAAAUCUUGCUGCUUCUCUAAAAAGUAUCGCAAGAUUAAAUUGGCC.ACCU..CCG................................GGG....GGC.UUUACCAAA......GGUGGUUAAA..................
env-276   UUAAGGGGUGGACACUUUCGCUUUACCCCUUUAUACGACCGAAAUCUUGGGUGGUAAAAGUUUUAUCGCCUAAACAAACCGGCC.ACCU..UCG................................GGG....GGC.UUUACCAAA......GGCGG...........UU..........
env-277   ..............UUCUGAUUUACCCCUUUAUACGAUCGAAAUCUUGAGAUCGUCGAUAAGAUCUCUCAAGAGUAAAUCGGCC.ACCU..UCG................................GGG....GGC.UUUACCAAA......GGCAGUAAUA..................
env-278   AAGGGGUGGACAGUUCUGAUUUACCCCUUUAUACGAUCGAAAUCUUGGGAUACCUCGAUAAGAGCUUCUAAGGUUAAAAUGGCC.ACCU..UCG................................GGG....GGC.UUUACCAAA......GGCGUUAAA...................
env-279   AAGGGGUGGACAGUUCUGAAUUACCCCUUUAUACGAUCGCAAUCUUAGGAACUUUGAAUAAAAGAUUGCAAGAUUAAAUUGGCC.ACCU..UCG................................GGG....GGC.UUUACCGAA......GGCGAUAAAA..................
env-280   AAGGGGUGGACAGUUCUGAAUUACCCCUUUAUACGAUCGCAAUCUUAGGAACUUUGAAUAAAAGAUUGCAAGAUUAAAUUGGCC.ACCU..UCG................................GGG....GGC.UUUACCGAA......GGCGAUAAAU..................
env-282   AAGGGGUGGACAGUUCUGAAUUACCCCUUUAUACGAUCGCAAUCUUAGGAACUUUGGAUAAAAGAUUGCAAGAUUAAAUUGGCC.ACCU..UCG................................GGG....GGC.UUUACCGAA......GGCGAUAAAA..................
env-284   AAGGGGUGGACAGUUCUGAAUUACCCCUUUAUACGAUCGCAAUCUUAGGAAAUUUGGAUAAAAGAUUGCAAGAUUAAAUUGGCC.ACCU..UCG................................GGG....GGC.UUUACCGAA......GGCGAUAAAA..................
env-285   ....................................UUAGGAACUUUGGAUAAAAGAUUUCAAGAUUAAAUUGGCC.ACCU..UCG................................GGG....GGC.UUUACCGAA......GGCGAUAAAA..................
env-286   AAGGGGUGGACAGUUUUAGUUUGCCCCAUUAUAUGAUCGCAAUCUUGAGUAGCUUUAGUAAUGGCUAACUAGAUUAACUUGGCC.ACCU..CCG................................GGG....GGC.UUUACCACA......GGCGCUAACA..................
env-287   AAAGGGGUGGACAGUUCUGAUUUACCCCUUUAUACGAUCGAAAUCUUGAGAACUUCGAUAAAAGUUCGAAAGAAUAAACUGGCC.ACCU..GUG................................GGG....GGC.UUUACCAUA......GGCGGUGAAU..................
env-288   AUGGGUGGACAGUUCUGAUUUACCACAUUAUACGAUCCAAAACCUGCAGAAUCUAGAAAUUUGGCUGCAAGUUUAAAAUGGCC.ACCU..UCG................................GGG....GGC.UUUACCGAA......GGCGGUAUUA..................
env-289   AAAGGGGUGGACAGUUCUGAUUUACCCCUUUAUAUGAUCGAAAUCUUGAGAACUUAGAUACACGUUCGCAAGAAUAAACUGGCC.ACCU..GCG................................GGG....GGC.UUUACCAUA......GGCAUUGAAA..................
env-290   AGGGGCUGGACAGUUUUGGUUUACACCUUUAUAUGAACGCAAUCUUGUGAUUGUUCGAUAGAACCUUGCAAAAUUAAAUUGGCC.ACCU..CUG................................GGG....GGC.UUUACCAAA......GGCGGUAAAU..................
env-291   AAGGGGCUGGACAGUUUUGAUUUACCCCUUUAUACGAAUGCAAUCUUGGAUAUGUUCGAUGGAACCUUCAAGAUUAAAUUGGCC.ACCU..CAG................................GGG....GGC.UUUACCAAA......GGCGGUAAAU..................
env-293   AAGGGGCUGGACAGUUUUGAUUUACCCCUUUAUACGAAUGCAAUCUUGGAUAUGUUCGAUGGAACCUUCAAGAUUAAAUUGGCC.ACCU..AAG................................GGG....GGC.UUUACCAAA......GGCGGUAAAU..................
env-295   AAGGGGGUGGACAGUUCUGAUUUACCCCUUUAUACGAUCGAAAUCUUGAGAACGUCGAUAAGAGUUCGCAAGAAUAAACUGGCC.ACCU..GCG................................GGG....GGC.UUUACCAUA......GGCGGUAAGA..................
env-296   AAGGGGGUGGACAGUUCUGAUUUACCCCUUUAUACGAUCGAAAUCUUGUGAGCUUGGAUAAAAGCGCGCAAGAAUAAACUGGCC.ACCU..GCG................................GGG....GGC.UUUACCAUA......GGCGGUAAGA..................
env-297   AUGGGGUGGACAGUUUUUAUUUACCACUUUAUAUGAUCCAAAUCUUGUAGAUUCUAGGUAAGAGUUUGCAAGUGUAAAAUGGCC.ACCU..UCG................................GGG....GGC.UUUACCAUA......GGCGGCUAAU..................
env-298   AUGGGGUGGACAGUUUUUAUUUACCACUUUAUAUGAUCCAAAUCUUGUAGAUUCUAGGUAAGAGUUUGCAAGUGUAAAAUGGCC.ACCU..UCG................................GGG....GGC.UUUACCAAA......GGCGGCAAAU..................
env-299   AUGGGGUGGACAGUUUUUAUUUACCACUUUAUAUGAUCCAAAUCUUGUAGAUUCUAGGUAAGAGUUCGCAAGUGUAAAUUGGCU.ACCU..UCG................................GGG....GGC.UUUACCAAA......GGCGGCAAAU..................
env-300   GGGGUGGACAGUUCUCUGAUUUACCCCCUUUAUACGGUCGAAAUCCUGAGAACUUGGAUAAAAGUUCGCAAGAAUAAACUGGCC.ACCU..GCG................................GGG....GGC.UUUACCAUA......GGCAGUAAAA..................
env-301   AAGGGGUGGACAGUUUUAAUUUACCCCUUUAUACGAUCGCAAUCUUGGGUUCUCUAAAAAAGAGAUCACAAGGUCAACCUGGCC.ACCU..UCG................................GGG....GGC.UUUACCAAA......AGCGGUAAAA..................
env-308   AAAGGGGUGGACAGUUCUGAUUUACCCCUUUAUACGAUCGAAAUCUUGAGAUCUUUGAUAAAAGGUCUCAAGAAGAAACCGGCC.ACCU..UCG................................GGG....GGC.UUUACCAUA......GGCGGUAAAA..................
env-309   AAAGGGGUGGACAGUUCUGAUUUACCCCUUUAUACGAUCGCAAUCUUAAGAACUUCGAUAAAAGUUCGCAAGAAUAAACUGGCC.ACCU..UCG................................GGG....GGC.UUUACCAUA......GGCGGUAAAA..................
env-310   ...............UUUGAUUUACCCCUUUAUACGAUCCAAAUCUCGAGAACUUCGAUAAAAGUUCGAAAGAAUAAACUGGCC.ACCU..UCG................................GGG....GGC.UUUACCAUA......GGCGGUAAAA..................
env-311   .......................................................CUUCGAUAAAAGUUCGCAAGAAUAAACCGGCC.ACCU..UCG................................GGG....GGC.UUUACCAUA......GGCGGUAAAA..................
env-312   AAAGGGGUGGACAGUUCUGAUUUACCCCUUUAUACGAUCGAAAUCUUGUGAACUCCGUUAAAAGUUCGCAAGAAUAAACCGGCC.ACCU..UCG................................GGG....GGC.UUUACCAUA......GGCGGUAAAA..................
env-313   AAAGGGGUGGACAGUUCUGAUUUACCCCUUUAUACGAUCCAAAUCUUGAGAUCUUCGAUAAAAGUUCGCAAGAAUAAACUGGCC.ACCU..UCG................................GGG....GGC.UUUACCAUA......GGCGGUAAAA..................
env-314   AAAUUUAAGGGGUGGACAGUUUGCUUUACCCUUUAUACGACGAAAUCUUGGGUGGUAAAUGGUUUAUCGCUAAACGCAUCGGCC.ACUU..UCG................................GGG....GGC.UUUACCAAA......GGCGGUUAGU..................
env-315   UUAAGGGGUGGACAGUUUUGCUUUACCCCUUUAUAUGACCGAAAUCUUGGGUGGUAAAAGUUUUAUCAGCCAAACAAACCGGCC.ACCC..UCG................................GGG....GGC.UUUACCAAA......GGCGGUUAGU..................
env-316   ............UAUAGGCGGAGAUACCCUUUAUACGACCAAAAUCUUGGGUGUUAAAAGAAUUAUCGCCUAAACAAACCGGCC.ACCU..UCG................................GGG....GGC.UUUACCAAA......GGCGGUUAGU..................
env-317   UUAAGGGGUGGACACUUUAGGUUUACCCCUUUAUACGACCGAAAUCUUGGGUGGUAAAAGUUUUAUCAGCCAAACAAACCGGCC.ACCU..UCG................................GGG....GGC.UUUACCAAA......GGCGGUUAGU..................
env-319   UUAAGGGGUGGACAGUUUUGCUUUACCCCUUUAUAUGACCGAAAUCUUGGGUGGUAAACGGUUUAUCGCCUAAACACAUCGGCC.ACCU..UCG................................GGG....GGC.UUUACCAAA......GGCGGUUAGU..................
env-320   UUAAGGGGUGGACAGUUUUGCUUUACCCCUUUAUACGACCGAAAUCUUGGGUGGUAAAAGAUUUAUCGCCUAAACAAUCUGGCC.ACCU..UCG................................GGG....GGC.UUUACCAAA......GGCGGUUAGU..................
env-321   UUAAGGGGUGGACAGUUUCGCUUUACCCCUUUAUACGACCGAAAUCUUGGGCGGUAAAAUAUUUAUCGCUUAAAUAAACCGGCC.ACCU..UCG................................GGG....GGC.UUUACCAAA......GGCGGUUAGU..................
env-322   UUAAGGGGUGGACAGUUUUGCUUUACCCCUUUAUACGACCGAGAUCUUGGGCGGUAAAAGAUUUAUCGCCUAAACAAACCGGCC.ACCU..UCG................................GGG....GGC.UUUACCAAA......GGCGGUUAGU..................
env-323   AAGGGGUGGACAGUUUUGAUUUACCCCUUUAUACGGUCGAAAUCUUGGGAUUGUUCGAUCAAACCUCGCAAGAUUAAAUUGGCC.ACCU..GCG................................GGG....GGC.UUUACCAAA......GGCGGUAAGU..................
env-324   UUAAGGGGUGGACAGUUUUGCUUUACCCCUUUAUACGACCGAAAUCUUGGGUGGUAAAAGAUUUAUCGCUUAAACAAUCUGGCC.ACCU..UCG................................GGG....GGC.UUUACCAAA......GGCGGUAAGU..................
env-325   UUAAGGGGUGGACAGUUUUGCUUUACCCCAUUAUACGACCGAAAUCUUGGGUGGUAAAAGAUUUAUCGCCUAAACAAUCCGGCC.ACCU..UCG................................GGG....GGC.UUUACCAAA......GGCGGUAAGU..................
env-326   UUAGGGGGUGGACAGUUUUGCUUUACCCCUUUAUAUGACCGAAAUCUUGGGUGGUAAAAGAUUUAUUGCCUAAAAAAUCUGGCC.ACCU..UCG................................GGG....GGC.UUUACCAAA......GGCGGUAAGU..................
env-327   UUAAGGGGUGGACAGUUUUGCUUUACCCCUUUAUACGACCGAAAUCUUGGGUGGUAAAAGAUUUAUCGCCUAAACAAUCUGGCC.ACCU..UCG................................GGG....GGC.UUUACCAAA......GGCGGUAAGU..................
env-329   UUAAGGGGUGGACAGUUUUGCUUUACCCCUUUAUACGACCAAAAUCUUGGGUGGUAAAAAAUUUAUCGCCUAAACAAACCGGCC.ACCU..UCG................................GGG....GGC.UUUACCAAA......GGCGGUAAGU..................
env-330   UUAAGGGGUGGACAGUUUUGCUUUACCCCUUUAUACGACCAAAAUCUUGGGUGGUAAAAAAUUUAUCGCCUAAAUAAACUGGCC.ACCU..UCG................................GGG....GGC.UUUACCAAA......GGCGGUAAGU..................
env-331   AUGGGGUGGACAGUUUUUAUUUACCACUUUAUAUGAUCCAAAACUUGUAGAUUCUAAUUAAGACUUUGCAAGUGUAAAAUGGCC.ACCU..UCG................................GGG....GGC.UUUACCAAA......GGCGGUAAAU..................
env-332   AUGGGGUGGACAGUUUUUAUUUACCACUUUAUAUGAUCCAAAUCUUGUAGAUUCUAGGUAAGAGUUUGCAAGUGUAAAAUGGCC.ACCU..UCG................................GGG....GGC.UUUACCAAA......GGCGGUAAAU..................
env-335   AUGGGGUGGACAGUUUUUAUUUACCACUUUAUAUGAUCCAAAUCUUGCAGAUUCUAGAUAAGAGUUUGCAGGUGUAAAAUGGCC.ACCU..UCG................................GGG....GGC.UUUACCAAA......GGCGGUAAAU..................
env-336   AAAGGGGUGGACAGUUCUGAUUUACCCCUUUAUACGAUCGCAAUCUUAAGAACUUCGAUAAAAGUUCGCAAGAAUAAACUGGCC.ACCU..UCG................................GGG....GGC.UUUACCAUA......GGCGGUAAAA..................
env-337   AAAGGGGUGGACAGUUCUGAUUUACCCCUUUAUACGAUCGAGAUCUUGAAGACUUUUAUCAAAGUUCGCAAGAGUAAACCGGCC.ACCU..UCG................................GGG....GGC.UUUACCAAA......GGCGGUAAAA..................
env-338   AAGGGGUGGACAGUUCUGGAUUACCCCUUUAUACGAGCGCAAUCUUGGGAAUUUUGGAAAAGAGUUCGAAAGAUAAAAACGGCC.ACCU..UCG................................GGG....GGC.UUUACCAAA......GGCGGUAAAA..................
env-339   AAGGGGUGGACAGUUCUGGAUUACCCCUCUAUAUGAGCGAAAUCUUGGGACUUUUGGAAAAGAGUUUAAAAGAUAAAAACGGCC.ACCU..UCG................................GGG....GGC.UUUACCAAA......GGCGGUAAAA..................
env-340   AAAGGGGUGGACAGUUCUGAUUUACCCCUUUAUACGAUCGAAAUCUUGAGAACUUCGAUAAAAGUUCGCAAGAAUAAACUGGCC.ACCU..UCG................................GGG....GGC.UUUACCAAA......GGCGGUAAAA..................
env-341   AAAGGGGUGGACAGUUCUGAUUUAGCCCUUUAUACGAUCGAAAUCUUGAAAGCUUUAAUCAAAGUUCGCAAGAGUAAACUGGCC.ACCU..UCG................................GGG....GGC.UUUACCAAA......GGCGGUAAAA..................
env-342   AGGGGGUGGACAGUUCUGAGUUACCCCUUUAUACGAUCGAAAUCUUGGGGACUCUCUAUAUGGGUUUGUAAGAUUAAACUGGCC.ACCU..UCG................................GGG....GGC.UUUACCAAA......GGCGGUAAAA..................
env-343   UUUAAGGGGUGGACAGUUUUGCUUUACCCCUUUAUACGACCGAAAUCUUGGGUGGUAAAAGAUUAUCGCCUAAACAACCUGGCC.ACU...UCGG...............................GG....GGC.UUUACCAAA......GGCGGUAAGU..................
env-344   UAAGGGGUGGACAGUCUUGCUUUACCCCUUUAUACGACCAAAAAACUUGGGUGGUAAAGAAUUUAUCGCCUAAACAAACCGGCC.ACCU..UCG................................GGG....GGC.UUUACCAAA......GGCGGCAAGU..................
env-345   AAGGGGUGGACAGUUUUGAUUUACCCCUUUAUAUGAUCAAAAUCUUAGGAUCGUUAGACAAGACAUCCAAAGAUUAAAUCGGCC.ACCU..CCG................................GGG....GGC.UUUACCAGA......GGCGGUAAAA..................
env-346   AAAGGGGUGGACAGUUCUGAUUUACCCCUUUAUACGAUCGAAAUCUUGAGAACUUCGAUAAAAGUUCGCAAGAAUAAACUGGCC.ACCU..GCG................................GGG....GGC.UUUACCAGA......GGCGGUAAAA..................
env-347   AAAGGGGUGGACAGUUCUGAUUUACCCCUUUAUACGAUCGAAAUCUUGAGAACUUCGAUAAAAGUUCGCAAGAAUAAACUGGCC.ACCU..GCG................................GGG....GGC.UUUACCAUA......GGCGGUAAAA..................
env-356   AAAGGGGUGGACAGUUCUGAUUUACCCCUUUAUAUGAUCGAAAUCUUGAGAACUUCGAUAAAAGUUCGCAAGAAUAAACUGGCC.ACCU..GCG................................GGG....GGC.UUUACCAUA......GGCGGUAAAA..................
env-357   ..........................................................UCGCCUAAACAACCUGGCC.ACCC..UCG................................GGG....GGC.UUUACCAAA......GGCGGAAAGU..................
env-358   ....GGUGGACAGUUCUGAUUUACCACUUUAUAUGAUCCCAACCUUGUAGAAUCUAUAACUUAGGCUGCAAGUUUAAAACGGCC.ACCU..UCG................................GGG....GGC.UUUACCAUA......GGCGGUUAUA..................
env-359   AUGGGGUGGACAGUUCUGAUUUACCACUUUAUAUGAUCCCAACCUUGUAGAAUCUAUAACUUAGGCUGCAAGUUUAAAACGGCC.ACCU..UCG................................GGG....GGC.UUUACCAUA......GGCGGUUAUA..................
env-360   AAAGGGGUGGACAGUUGUGAAUUACCCCUUUAUACGAGCGAAACCUUGAGAUCUUUUAUAAAAUUUCUCAAGAGUAAACUGGCC.ACCU..UCG................................GGG....GGC.UUUACCAAA......GGCGGUAAA...................
env-361   AAAGGGGUGGACAGUUCUGAUUUACCCCUUUAUACGAUCGAAAUCUUGAGAGCUUCGAUAAGAUUUCUCAAGAUUUAACUGGCC.ACCU..UCG................................GGG....GGC.UUUACCAGA......GGCGAUAAA...................
Psp-3-1   GGCUUGCCUUUUUUGUUCGGCGCGGUUUGCCCGCACUUCAGGACAGCGAAAAUCGGCACCUUCGGGGGCCUAUACUCAGGCC.ACCU..UCG................................GGG....GGC.UUUAACAUA......GGCGG...........GGGC........
Hmi-1-1   GAAUCUCGUGGCCCCGGACUCUUCCGGGGCCACGCGCUUUCAGCAGAAGCGUAGCUUUCAUUGCCGAAAGUGCACUCAGCGGGG.AUCU..UCG................................GAG....CCC.UAUAACCCCC.....GGGCGAC.........GC..........
Hma-1-1   GAAUCUCGUGGCCCCGGAUUCUUCCGGGGCCACGCGCUUUCAGCAGAGACGUUGCUUUCUUUGCCGGAAGUGCACUCAGCGGGG.AUCU..UCG................................GAG....CCC.UAUAACCUCC.....GGGCCAC.........GGC.........
Laq-1-1   UGCAGACCCCGGAUUCGUUCCGGGGUUGUUACGUUUGUCAGGAUGUUUGAACUUUGGGACCUUCGGGGCCCUAUAUCUCAGGCA.ACCCU.AGUAGGGCGUCUCAUCGUGGGACAACCGGGC...GGGG....GGC.UUACACAUA......GCGGGU..........CCAAAG......
env-362   CAUCCCCCCUAAACGAGCCUAUCUGUGUGGCCCCGGAUUCGUCCGGGGCUUCGCAUUCGUUCGUAAGAACACCCUAAGCCGGGC.ACCU..ACG................................GGG....GCC.UAUAAACAU.......GGUGG...........G...........
Rpo-1-1   ACGCCACCAAUCUUGCGCCCUGGGAUUCGUCCCGGGGCUCGUGAUUUUUGCAGCCGGGGACCUUCGGGGCCCUAUACCAUGGGC.ACCU..ACG................................GGG....GCC.UCAAUACAUA.....GGCGG...........G...........
env-363   ..............UUCUUUUAUUUACCCAUAUAUUUUGGGAUAAGUGUUAUUGGUAGCUAUUUAAGCUACUUUAAUUCAGGGG.ACCU..CUG................................GGG....CCC.UAUAAACCA.......GCGG...........UCU.........
env-364   AUGUCUCAACGGGACUCUGAUAGUUAGCCAGGACUCUGGUCUGGCCCUGAUAUCAAAUCAGGGCCUAGGCCGUUUAAGCUGGGG.ACCU..UUG................................GGG....CCC.UAAUUCAGA......AGCGAGCUAA......UCGUU.UCGC..
Sla-1-1   ACGCUCCCAAUCUUGCGGCCUCGGAUUCGUUCCGGGGCUUCUGAUAUUUGCAGCCGGGGACCUUCGGGGCCCUAUAACCUGGGC.ACCU..ACG................................GGG....GCC.UCUCACAUA......GCGGG...........GAUU........
Rha-2-1   AUGCACCCAAUCUUGCGGCCUCGGAUUCGUUCCGGGGCCUCUGAUAUUUGAAACCGGGGACCUUCGGGGCCCUAUACCUUUGGC.ACCU..ACG................................GGG....GCC.UAUCACAUA......GCGGG...........G...........
Rsp-17-1  AUGCACCCAAUCUUGCGGCCUCGGAUUCGUUCCGGGGCCUAAGAUAUUUGAAACCGGGGACCUUCGGGGCCCUAUACCUUUGGC.ACCU..ACG................................GGG....GCC.UAUCACAUA......GCGGG...........G...........

          ................................................................................<<<<..<<<<<.................................>>>>>....>>>.>..............<<<<<...........<<<<<<<<<<<<
          ................................................................................1222..222.0.................................0.222....222.1..............22222...........22..1.......
          ●●●●●●●●●●●●●●●●●●●●●●●●●●●●●●●●●●●●●●●●●●●●●●●●●●●●●●●●●●●●●●●●●●●●●●●●●●●●YYGG●●-ACCU--●CG--------------------------------GGG----●●C-U●YA●●●○○------R●●●●----------○○---------
                                                                                5′
```

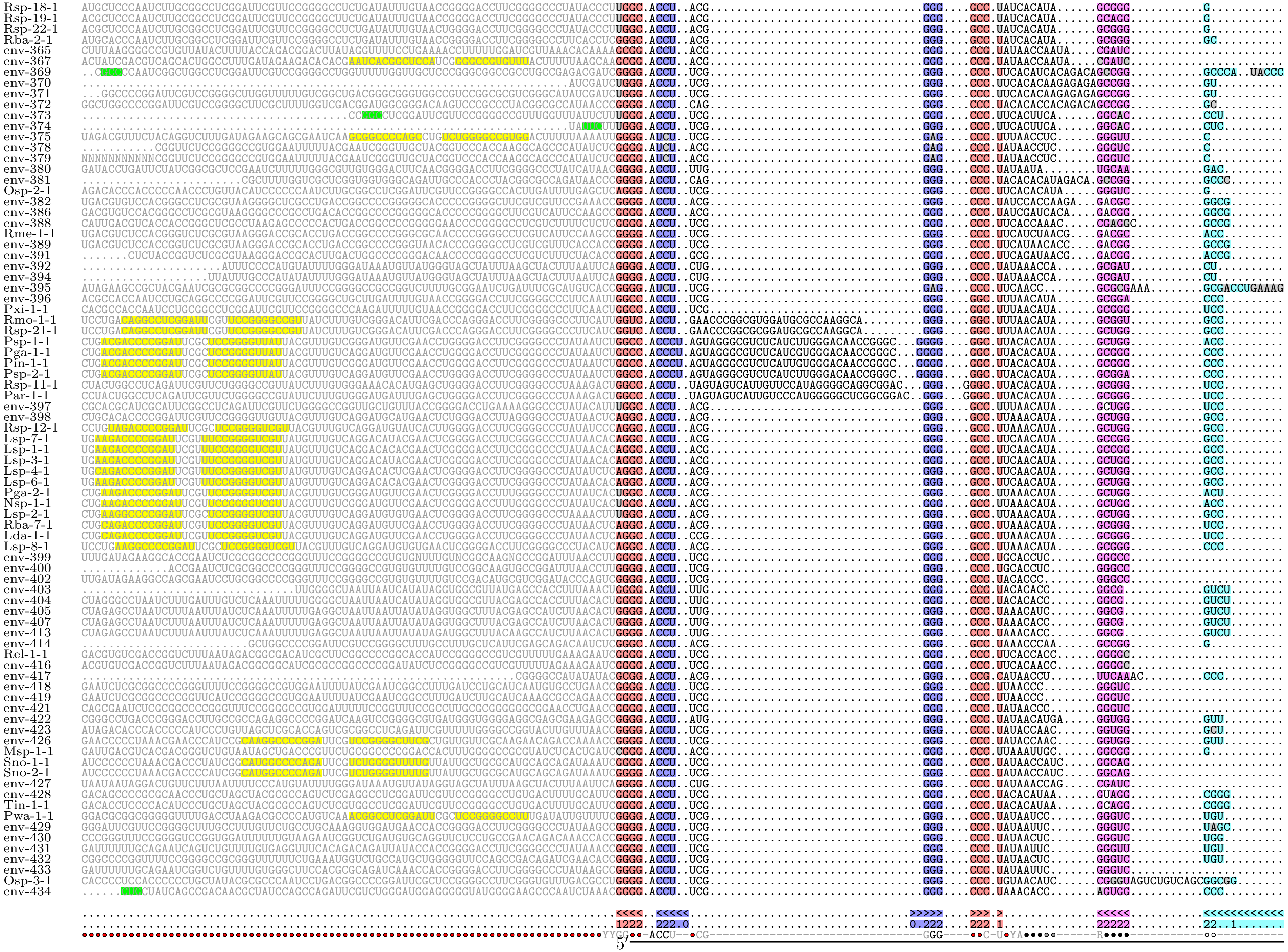
Rsp-18-1
Rsp-19-1
Rsp-22-1
Rba-2-1
env-365
env-367
env-369
env-370
env-371
env-372
env-373
env-374
env-375
env-378
env-379
env-380
env-381
Osp-2-1
env-382
env-386
env-388
Rme-1-1
env-389
env-391
env-392
env-394
env-395
env-396
Pxi-1-1
Rmo-1-1
Rsp-21-1
Psp-1-1
Pga-1-1
Pin-1-1
Psp-2-1
Rsp-11-1
Par-1-1
env-397
env-398
Rsp-12-1
Lsp-7-1
Lsp-1-1
Lsp-3-1
Lsp-4-1
Lsp-6-1
Pga-2-1
Nsp-1-1
Lsp-2-1
Rba-7-1
Lda-1-1
Lsp-8-1
env-399
env-400
env-402
env-403
env-404
env-405
env-407
env-413
env-414
Rel-1-1
env-416
env-417
env-418
env-419
env-421
env-422
env-423
env-426
Msp-1-1
Sno-1-1
Sno-2-1
env-427
env-428
Tin-1-1
Pwa-1-1
env-429
env-430
env-431
env-432
env-433
Osp-3-1
env-434
57

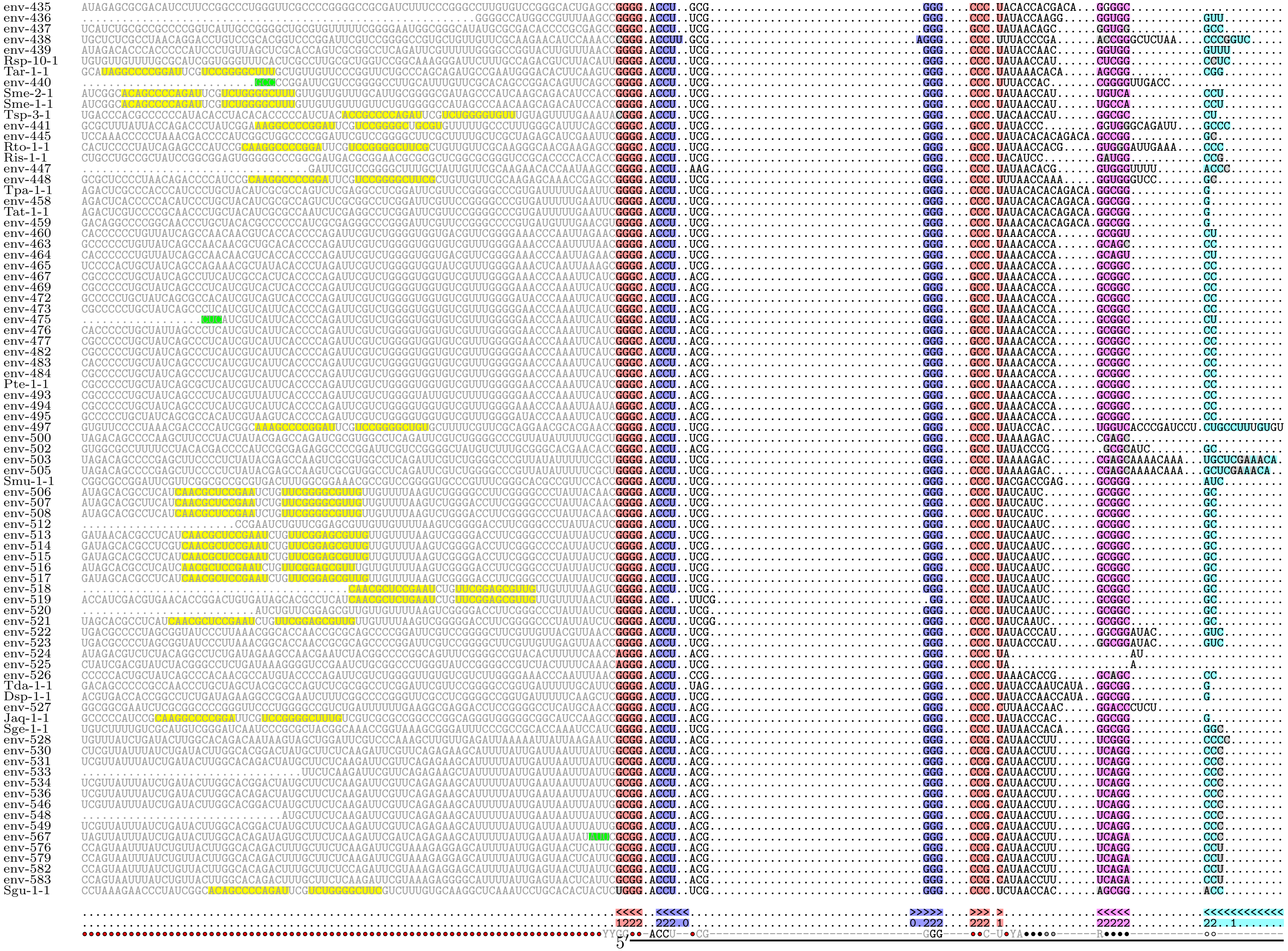
env-435
env-436
env-437
env-438
env-439
Rsp-10-1
Tar-1-1
env-440
Sme-2-1
Sme-1-1
Tsp-3-1
env-441
env-445
Rto-1-1
Ris-1-1
env-447
env-448
Tpa-1-1
env-458
Tat-1-1
env-459
env-460
env-463
env-464
env-465
env-467
env-469
env-472
env-473
env-475
env-476
env-477
env-482
env-483
env-484
Pte-1-1
env-493
env-494
env-495
env-497
env-500
env-502
env-503
env-505
Smu-1-1
env-506
env-507
env-508
env-512
env-513
env-514
env-515
env-516
env-517
env-518
env-519
env-520
env-521
env-522
env-523
env-524
env-525
env-526
Tda-1-1
Dsp-1-1
env-527
Jaq-1-1
Sge-1-1
env-528
env-530
env-531
env-533
env-534
env-536
env-546
env-548
env-549
env-567
env-576
env-579
env-582
env-583
Sgu-1-1
57

```
env-590   GCUGUGUCGUUUUGCGCAUUGGUGAGUUCUAGCCACGCGCAACGUCGGGCAAAGGGGUUCUUUGUCUGGCAUUUCAAAUUGGGG.ACCU..UCG............................GGG....CCC.UACAACCAU.....CUCGG...........CGCC........
env-591   GCUGUGUCGUUUUGCGCAUUGGUGAGUUCUAGCCACGCGCGAGGUUGGGCAAAGGGGUUCUUUGUCUGGCAUUUCAAAUUGGGG.ACCU..UCG............................GGG....CCC.UACAACCAU.....CUCGG...........CACC........
env-592   GCUGUGUCGUUUUGCGCAUUGGUGAGUUCUAGCCACGCGCGAGGUUGGGCAAAGGGGUUCUUUGUCUGGCAUUUCAAAUUGGGG.ACCU..UCG............................GGG....CCC.UACAACCAU.....CUCGG...........CACC........
env-595   ..................UUGGUGAGUUCUAGCCAAGCGCGAGGUUGGGCAAAGGGGUUCUUUGUCCGGCAUUUCAAAUUGGGG.ACCU..UCG............................GGG....CCC.UACAACCAU.....CUCGG...........CACC........
env-596   GCUGUGUCGUUUUGCGCGUUGGUAGGUUCUAGCCGCACGCAACGUCCGGCAAAGGGGUUCUUUGUCUGGCAUUUCAAAUUGGGG.ACCU..UCG............................GGG....CCC.UACAACCAU.....CUCGG...........CACC........
env-597   GCUGUGUCGUUUUGCGCAUAGGUGAGUUCUAGCCACGCGCGAGGUUGGGCAAAGGGGUUCUUUGUCUGGCAUUUCAAAUUGGGG.ACCU..UCG............................GGG....CCC.UACAACCAU.....CUCGG...........CACC........
Ssp-4-1   GCUGUGUCGUUUUGCGCAUGGGUGAGUUCUAGCCAAGCGCGAGGUUGGGCAAAGGGGUUCUUUGUCUGGCAUUUCAAAUUGGGG.ACCU..UCG............................GGG....CCC.UACAACCAU.....CUCGG...........CACC........
env-598   GCUGUGUCGUUCUGCGCAUUGGUGAGUUCUAGCCACGCGCGAGGUUGGGCAAAGGGGUUCUUUGUCUGGCAUUUCAAAUUGGGG.ACCU..UCG............................GGG....CCC.UACAACCAU.....CUCGG...........CACC........
Ssp-5-1   GCUGUGUCGUUUUGCGCAUUGGUGAGUUCUAGCCACGCGCGAGGUUGGGCAAAGGGGUUCUUUGUCUGGCAUUUCAAAUUGGGG.ACCU..UCG............................GGG....CCC.UACAACCAU.....CUCGG...........CACC........
env-600   GCUGUGUCGUUUUGCGCAUUGGUGAGUUCUAGCCAAGCGCGAGGUUGGGCAAAGGGGUUCUUUGUCUGGCAUUUCAAAUUGGGG.ACCU..UCG............................GGG....CCC.UACAACCAU.....CUCGG...........CACC........
env-605   GCUGUGUCGUUCUGCGCAUUGGUGAGUUCUAGCCACGCGCGAGGUUGGGCAAAGGGGUUCUUUGUCUGGCAUUUCAAAUUGGGG.ACCU..UCG............................GGG....CCC.UACAACCAU.....CUCGG...........CACC........
env-606   ................................................................................GGGG.ACCU..UCG............................GGG....CCC.UACAACCAU.....CUCGG...........CACC........
env-611   UAUCGACGUGACCACGGGCCUUUGAUAUAGGCAUCACGACUCAGCGGCCCCGACCCUCUCGGGGCCGCUGGUUAUUUGGCGCGG.ACCC..ACG............................GGG....CCG.CAUCACGACA....ACCGGACCUU.....................
env-612   ................................................CCCGACGCUCUCGGGGCCGCUGGUUAUUUGGCGCGG.ACCU..ACG............................GGG....CCG.CAUCACGACA....ACCGGACCUU.....................
env-613   GGGGUUUGGCUAUUUGAACGAUACCGGUGGAUCGGAUGCUUAGCUCGCAAGAGAUAAUCGCCCCAUCUGCCACUCCUACAGGGG.ACCU..UCG............................GGG....CCC.UAUAACCAU.....CGCGCCUACAUU....UU..........
env-614   GGGGUUUGGCUAUUUGAACGAUCCCGGUGGAUCGGAUGCUUACCUCGAGAGAGAUAAUCGCCCCAUUCGCCACUCCUACAGGGG.ACCU..UCG............................GGG....CCC.UACAACCAA.....CGCGCUUUCAU.....CUC.........
env-615   GGGGUUUGGCUAUUUGAACGAUCCCGGUGGAUCGGAUGCUUACCUCGAGAGAGAUAAUCGCCCCAUUCGCCACUCCUACAGGGG.ACCU..UCG............................GGG....CCC.UACAACCAA.....CGCGCUUUCAU.....CUC.........
env-616   CGGGGUUUGGCUAUUUGAACGAUCCCGGUGGUUGGGAUGCCUGCCUCGCAAGAGAUUUAAGCCCACCCACCACUCCUACAGGGG.ACCU..UCG............................GGG....CCC.UACAACCAA.....CGCGCUUUC.......AUCU........
env-617   CGGGGUUUGGCUAUUUGAACGAUCCCGGUGGUUGGGAUGCCUGCCUCGCAAGAGAUUUAAGCCCACCCACCACUCCUACAGGGG.ACCU..UCG............................GGG....CCC.UACAACCAA.....CGCGCUUUC.......AUCU........
env-618   CGGGGUUUGGCUAUUUGAACGAUCCCGGUGGUUGGGAUGCCUGCCUCGCAAGAGAUUUAAGCCCAUCCACCACUCCUACAGGGG.ACCU..UCG............................GGG....CCC.UACAACCAA.....CGCGCUUUC.......AUCA........
env-623   ................................................................ACCCACCACUCCUACAGGGG.ACCU..UCG............................GGG....CCC.UACAACCAA.....CGCGCUUUC.......AUCA........
env-624   ...............UGAACGAUCCCGGUGGUUGGGAUGCCUGCCUCGCAAGAGAUUUAAACCCAUCCACCACUCCCACAGGGG.ACCU..UCG............................GGG....CCC.UAUAACCAA.....CGCGCUUUCACC....UUG.........
env-625   GGGGUUUGGCUAUUUGAACGAUCCCGGUGGAUCGGAUGCUUAACUCGCAAGAGAUAAUCGCCCCAUCUGCCACUCCUACAGGGG.ACCU..UCG............................GGG....CCC.UAUAACCAA.....CGCGCCUUCGUC....UCG.........
env-626   GGGGUUUGGCUAUUUGAACGAUCCCGGUGGAUCGGAUGCUUAGCUCGCAAGAGAUAAUCGCCCCAUCUGCCACUCCUACAGGGG.ACCU..UCG............................GGG....CCC.UAUAACCAA.....CGCGCCUUCGUC....UCG.........
env-627   UUGUUUUGCGAUACGGUCAAUCACACUGACCCGACGCGCACUGAUGGGGCUGACGAUAUUCCGCCCUUAUCAUUAUAUUUUGGG.ACCU..UCG............................GGG....CCC.UAUAACCAU.....CUCGG...........CGUC........
env-628   AGAUUCGUCUGGGGCUUCGUCUUUGUGCACGGCUCAAACCUUGCACGCUACGUUGGGACCUUCGGGGCCCUCUAACCACAGCGG.ACCU..ACG............................GGG....CCG.CAUA.........................................
env-629   AUUCGUCUGGGGCUUCGUCUUUGUGCAUGGGUCAAACCCUGUGCGCGCUACUUUGGGACCUUCGGGGCCCUCUAACCACAGCGG.ACCU..ACG............................GGG....CCG.CAUA.........................................
Sdo-1-1   CACAGCCCCAGAUUCGUCUGGGGCUUUGUUGUUGUUCGCAAGAACAUAUCUACUGGGACCUUCGGGGCCCUCUAACCACAGCGG.ACCU..ACG............................GGG....CCG.CAUA.........................................
env-630   ...................GCUAUACCGCGCGGGUGUUUGGCCUCAGAUUCGUUCUGGGGCUGAAUUCCUUUUACCAAAUGCGG.ACCU..ACG............................GGG....CCG.UUCACCAUA.....GGGGC...........C...........
env-631   GCCACCCCCNAAACCCCCGCUAUACCGCGCGGGUGUUUGGCCUCAGANUCGUUCUGGGGCCGAAUUCCUUUUACCAAAUGCGG.ACCU..ACG............................GGG....ACG.UUCACCAUA.....GGGGC...........C...........
env-632   UUCGUUCUGGGGCCUGAUACGUUUUACCAUUAGNGGACCCAAGNGGUUUGAACAAACCUCCGCCGGAACCUAAACACCAUGCGG.ACCU..ACG............................GGG....CCG.UUCACAAUA.....GGGGC...........C...........
env-633   CACGAGUCUCGCGGCCCCGGGUAAUCGGGGCCGCGUGCCUUUUCGGGAAUCGUGCUUCGGCAUUUUCCCGAAUUCACGACGGGG.ACCU..GCG............................GGG....CCC.UAUAUCGAAAC....GGACCGAAA...................
env-634   CACCAGCAGCCCCGGAUUCGUCCGGGGCUCUUGGUUUGUUUGUCCCUGUGCAUUCAAGUAUCGGGCUGGACAGUCAAGCCGGGG.ACCU..UCG............................GGG....CCC.UUCAUCUAAC.....GGACCUAU....................
env-635   AACACGAUCGACGUUCAGUCGGGCCUGUGACGAUCUGGUCCCGCGGGCCGUGAAGGCACGCGGGAUCAGCUUUAGAAGACGGGG.ACCU..UCG............................GGG....CCC.UUCAUCCACCAC...GGACCAUG....................
env-636   AACACGAUCGACGUGCAGUCGGGCCUGUGACGAUCUGAUCCCGCGCGCCGUGAAGGCACGCGGGAUCAGCUUUAGAAGACGGGG.ACCU..UCG............................GGG....CCC.UUCACCAACCAC...GGACCAUG....................
env-637   GCGAAUCUCGCGGCCUCGGGAAUACCGGGGCCGUGAAUUUUUGGGUGGGGCGCAUCAGCAAGUCACUCAAACGACACGGCGGGG.ACCU..UCG............................GGG....CCC.UAUAAAUCCAUC...GGACCUAU....................
Lme-1-1   CUGAAGACCCCGGAUUCGUUCCGGGGUCGUUACGUUUGUCAGGAUGUUCGAACUUUGGGACCUUCGGGGCCCUAUAAAUCGGCC.ACCUU.AGUAGCUUGUUCCAAACGGAACCUGCGGGC....GGGG....GGC.UUUCAACAUA.....GCGGG...........UCC.........
env-638   .................................UUUCCGUUCGGUUUCGGGCCCUUCGUUGAAUUAUUUUAAUAAACGUCGGCCUACCG..UUCG...........................GGG....GGC.UUUACCAUG.....GGCGG...........G...........
env-639   CACCCACGCCAUUCACCACUAUACCGCAGCGAUCUUGUAUGGCCUUAGAUUCGUCUGGGGCCUCUUGAUGUUUAUAAACUGGCC.ACCU..UCG............................GGG....GGC.UUUACCAUG.....GGCGGGGAGAC.................
Rba-1-1   CACCCACGUCAUUCACCACUAUACCGCAGCGAUCUUGUAUGGCCUUAGAUUCGUCUGGGGCCUCUCGAUGUUUAUAAACUGGCC.ACCU..UCG............................GGG....GGC.UUUACCAUG.....GGCGGGGAGAC.................
env-640   CACCCAAGCCAUUCACCACUAUACCGCAGCAAUCUUGCAUGGCCCUAGAUUCGUCUUGGGCCUCUUGAUAUUUAUAAACUGGCC.ACCU..UCG............................GGG....GGC.UUUACCAUG.....GGCGG...........G...........
env-641   CACCCACGCCAUUCACCACUAUACCGCAACGAUCUUGUAUGGCCCUGGAUUCGUCUAGGGCCACUUGAUGUUUAUAAACUGGCC.ACCU..UCG............................GGG...GGGC.UUUACCAUG.....GGCGG...........G...........
env-643   CACCCACGCCAUUCACCACUAUACGACAGCAAUCUUGUAUGGCCCUAGAUUCGUCUAGGGCCUAGUGAUAUUUAUGAACUGGCC.ACCU..UCG............................GGG....GGC.UUUACCAUG.....GGCGG...........G...........
env-644   CACCCAAGCCAUUCACCACUAUACCGCAGCAAUCUUGCAUGGCCCUAGAUUCGUCUUGGGCCUCUUGAUAUUUAUAAACUGGCC.ACCU..UCGG...........................GGG....GGC.UUUACCAUG.....GGCGG...........G...........
env-645   CACCCAAGCCAUUCACCACUAUACCGCAGCAAUCUUGCAUGGCCCUAGAUUCGUCUUGGGCCUCUUGAUAUUUAUAAACUGGCC.ACCU..UCG............................GGG....GGC.UUUACCAUG.....GGCGG...........G...........
env-646   CAACCAAGCCAUUCACCACUAUACCGCAGCAAUCUUGCAUGGCCCUAGAUUCGUCUAGGGCCUCUUGAUGUUUAUAAACUGGCC.ACCU..UCGG...........................GGG....GGC.UUUACCAUG.....GGCGG...........G...........
env-647   CACCCACGCCAUUCACCACUAUACCGCAACGAUCUUGUAUGGCCCUGGAUUCGUCUAGGGCCACUUGAUGUUUAUAAACUGGCC.ACCU..UCG............................GGG....GGC.UUUACCAUG.....GGCGG...........G...........
env-648   CACCCAAGCCAUUCACCAUUAUACCGCAGCAAUCUUGUACGGCCCUAGAUUCGUCUAGGGCCUCUUGAUGUUUAUAAACUGGCC.ACCU..UCG............................GGG....GGC.UUUACCAUG.....GGCGG...........G...........
env-649   CAGCCAAGCCAUUCACCACUAUACCGCAGCAAUCUUGUAUGGCCCUAGAUUCGUCUAGGGCUUCUUGAUGUUUAUAAACUGGCC.ACCU..UCG............................GGG....GGC.UUUACCAUG.....GGCGG...........G...........
env-650   CACCCAAGCCAUUCACCACUAUACCGCAGCAAUCUUGCAUGGCCCUAGAUUCGUCUUGGGCCUCUUGAUAUUUAUAAACUGGCCAACCU..UCGG...........................GGG....GGC.UUUUACCAUG....GGCGG...........G...........
Wma-1-1   CCUCUAGUAGGGGGCUCCAUCCGAGCGGCCCCGGAGCGAUCCGGGGCUUCUCUUUACCCGGCGGGCCGAUUGCCCGUCACUGGG.ACCU..UCG............................GGG....CCC.UUCACUGACA....GCCGG...........GCGC........
env-651   AAAGCCCUAGACCUUUUUAAUAAAGAUAGCUAUACGUCCUUCGCUAAAGCAAUCAAGAUUCGUUUGAUUUGCUUUAAAUCGGCC.ACCU..UAU............................GGG....GGC.UUUACCAUA.....GCAGA...........GUA.........
env-652   GGGGGCCCCGGGGCAAGCCCGGGGCGCGAUGGGCGGGGCAGGUGGCGCACGAACCGGGGACCUUCGGGGCCCUAUACUCUGGCU..C....GCAA...........................G....AGC.CCAAACAUA.....GCGGG...........GC..........
Mal-2-1   AGGGGCCCCGGGGCAAGCCCGGGGCGCGAUGGGCGGGGCAGGUGGCGCACGAACCGGGGACCUUCGGGGCCCUAUACUCUGGCU..C....GCAA...........................G....AGC.CCAAACAUA.....GCGGG...........GC..........
env-653   ...................CGGGGCGCGAUGGGCGGGGCAGGUGGCGCACGAACCGGGGACCUUCGGGGCCCUAUACUCUGGCU..C....GCAA...........................G....AGC.CCAAACAUA.....GCGGG...........GC..........
env-656   UAACCCCCAACCGCGCAGCCCCGGAUUCGUCCGGGGCUUCGUUGUUGUUCUAACCGGGGACCUUCGGGGCCCUAUACCAAGGCU..C....CCAG...........................G....AGC.CCAAACAUA.....GCGGG...........UCG.........
Pga-3-1   CUGACGACCCCGGAUUCGCUCCGGGGUUAUUACGUUUGUCAGGAUGCACGAACUCGGGGACCUUUGGGGCCCUAUAUCACUGGC.ACCU..ACG............................GGG....GCC.UUAAACAUA.....GCUGG...........ACC.........
          ................................................................................<<<<..<<<<<..........................>>>>>....>>>.>.............<<<<<...........<<<<<<<<<<<<
          ................................................................................1222..222.0..........................0.222....222.1.............22222...........22..1.......
          ●●●●●●●●●●●●●●●●●●●●●●●●●●●●●●●●●●●●●●●●●●●●●●●●●●●●●●●●●●●●●●●●YYGG●●-ACCU--●CG--------------------------GGG----●●C-U●YA●●●●○○------R●●●●----------○○---------
                                                                                5′
```

alignment positions 181 · · · 343

```
env-1     ................CUGAG.............................................................................................G.CCCG......AAAGC.AGACGGA.AGACAGA.AAG.CAUG
Dsh-1-1   ................ACCCGGCACGCAAC.................................................................................................AACGGA.AGACAGA.AAG.CAUG
Pde-1-1   ................UUUUGACUGAAACGGACCACCUCA........................................................................G.GUCC......AAAGC..AACGGA.AGACAGA.AAG.CAUG
env-2     ................GGGA........................................................................C.......................C.GUCCG..U..U.UGC.UAACGGA.AGACAGA.AAG.CAUG
Aat-2-1   ................GCAA........................................................................C.......................C.UGCCG..U..U.UGCUAAACGGA.AGACAGA.AAG.CAUG
Rsp-2-1   ..................................................................................................................................................CGGA.AGACAGA.AAG.CAUG
env-3     ................UACG......................................................................GGG.......................C.CGCCA..CA.U.....ACACGGA.AGACAGU.AAG.AAUG
Amu-1-1   ................AAA........................................................................GC.......................C.CUCG...CA.C.AGCUAAACGGA.AGACAGA.AAG.CAUG
env-4     ..............................................................................................................G.GUCC......AAAGC..AACGGA.AGACAGA.GAG.CAUG
env-5     ..............................................................................................................G.GUCC......AAAGC..AACGGA.AGACAGA.GAG.CAUG
Rat-1-1   ................GCCCAUCCGGGGCA............................................................GCU.......................U.CGCCG.UC..U..GC.AAACGGA.AGACAGA.AAG.CAUG
env-6     ................GGGUAAACUCGCA............................................................GCAA.......................C.GCUC......A.AGCUAAACGGA.AGACAGA.AAG.CAUG
env-7     ................GGGUAAACUCGCA............................................................GCAA.......................C.GCUC......A.AGCUAAACGGA.AGACAGA.AAG.CAUG
env-8     ................GCAA......................................................................CAC.......................C.CGUG...C..A.AGC.AAACGGA.AGACAGA.GAA.CAUG
env-9     ................AACU......................................................................GCU.......................C.UCGC...C..C.AGA.CAACGGA.AGACAGU.AAA.CAUG
Tst-1-1   ................GCAG......................................................................GCU.......................C.UCGC...C..C.AGA.CAACGGA.AGACAGU.AAA.CAUG

          <<<<<<<<<<<<<<<<.....................................................>>>>>>>>>>>>>>>>>>>>>>>>>>>.......................>.>>>>.........................................
          ....1.000.00.000.....................................................000.00.000.1..........1..22.......................2.2222.........................................
          ----------------○○○○-----------------------------------------------------------------------○○----------------------●-●●●Y○--○--○-○GC-○AACGGA-AGACAGA-AAG-CAUG
                                                                                                                                                                     3′
```

```
env-10    ................GCAG...GCU...C.UCGC...C..C.AGA.CAACGGA.AGACAGU.AAA.CAUG
env-13    ................UAAA...CUCGGUG.CAU...C.ACCU.....G.AGCUAAACGGA.AGACAGA.AAG.CAUG
Rca-7-1   ...G.GUCCA....A.AGC..AACGGA.AGACAGA.GAG.CAUG
Rca-4-1   ...G.GUCCA....A.AGC..AACGGA.AGACAGA.GAG.CAUG
Rsp-4-1   ...CGGA.AGACAGG.AAG.CAUG
Rsp-5-1   ...CGGA.AGACAGG.AAG.CAUG
Rsp-7-1   ...CGGA.AGACAGG.AAG.CAUG
env-14    ................CGAA...CUCGGGG.CA...U.CGCU.....UAUGCUAAACGGA.AGACAGA.AAG.CAUG
env-15    ................GCAA...GA...C.CUGGA....U.AGC.AAGCGGA.AGACAGU.AAU.CAUG
env-16    ................UAAA...CUCGGUGCAU...C.ACCUG......AGCUAAACGGA.AGACAGA.AAG.CAUG
env-17    ................GCGA...CA...C.CCGUG..C...AAGC.AAACGGA.AGACAGG.AAA.CAUG
Sqi-1-1   ................ACG...GCCG...C.CUCA.....U.AGC.AAACGGA.AGACAGA.AAG.CAUG
env-18    ................AAAA...CUCGG...UGCAUCG..CCUG.AGCUAAACGGA.AGACAGA.AAG.CAUG
env-19    ................AUCAG...C...G.UUGCA..U..U.AGA.AAACGGA.AGACAGA.AAG.CAUG
env-20    ................GAU...GC...C.UCGCA..CU....GA.AAACGAA.AGACAGU.AAG.AAUG
Hsp-1-1   ................ACGCCAGUAGGGCAG...GGU...U.CGCU.....U.AGCUAAACGGA.AGACAGA.AAG.CAUG
Hna-1-1   ................UGACCAUUCGGGUU...GGGU...U.CGC.A..C....AGCUAAACGGA.AGACAGA.AAG.CAUG
Rba-6-1   ................ACCGCUCAGCGAGCAAAGCCCACACACAGACAGGCGGGGCACGCACCC...C.GCUG...C..AAAGC..AACGGA.AGACAGA.AAG.CAUG
env-21    ................AGUUACAG...GAU...C.CGCC...U..C.AGCUAGACGGA.AGACAGA.AAG.AAUG
Rsp-1-1   ................CC...GAG...G.GGUCU..C..U.AGC.UGACGGA.AGACAGG.AAG.CAUG
env-22    ................CAAUC...G...C.CUGCG..UUUAA....AAGCGGA.AGACAGU.AAG.AAUG
env-23    ................UUAAUC...G...G.CCGCG..CU.A.AGC.AAACGGA.AGACAGA.AAG.CAUG
env-24    ................UUAAUC...G...G.CCGCG..CU.A.AGC.AAACGGA.AGACAGA.AAG.CAUG
env-25    ................UUAAUC...G...G.CCGCG..CU.A.AGC.AAACGGA.AGACAGA.AAG.CAUG
env-26    ...U.CUCUU.AAUAU.AGC.AAACGGA.AGACAGC.AAU.CAUG
env-27    ...U.CUCUU.AAUAU.AGC.AAACGGA.AGACAGC.AAU.CAUG
env-35    ................AAUC...GC...C.UGCCU..CACA.....AAACGGA.AGACAGA..AG.CAUG
Csp-1-1   ...GACC...C.GACCG.UCU....GU.AAACGGA.AGACAGA.AAG.CAUG
Pat-1-1   ................CUCA...CGC...C.UGGCA..C..U..GCUAAACGGA.AGACAGA.AAG.CAUG
Rba-3-1   ................GCAA...C...C.CGCCG....GAAGC.UUACGGA.AGACAGA.AAG.CAUG
env-36    ................GUAA...C...C.CGUC.....UAAGCUAAACGGA.AGACAGA.AAG.CAUG
env-37    ................ACCA...C...C.CGCC...UUUU.AGC.UAACGGA.AGACAGA.AAG.CAUG
Rca-6-1   ...G.GUCCA......AAGC..AACGGA.AGACAGA.GAG.CAUG
env-38    ................CACA...CC...U.CUGCA....U.UGCUAAACGGA.AGACAGA.AAG.CAUG
env-39    ................CACA...CC...U.CUGCA....U.UGCUAAACGGA.AGACAGA.AAG.CAUG
env-40    ................CACA...CC...U.CUGCA....U.UGCUAAACGGA.AGACAGA.AAG.CAUG
Oba-1-1   ................GGAG...GGGCAGG...G.GAUCG..C..U.AGCUAAACGGA.AGACAGA.AAG.CAUG
env-41    ................CGC...GCGCCUUC...G.GUCCG..C..CAAGC.AAACGGA.AGA.AGC.UAA.CAUG
env-42    ...G.CCUGA.ACA.C......AACGGA.AGACAGA.UAA.AAUG
env-43    ...G.CCUGA.ACA.C......AACGGA.AGACAGA.UAA.AAUG
env-47    ...G.CCUGA.ACA.C......AACGGA.AGACAGA.UAA.AAUG
env-49    ...C.CUGA...CA...UAC..AACGGA.AGACAGU.UAA.CAUG
env-50    ...C.CUGA...CA...UAC..AACGGA.AGACAGU.UAA.CAUG
env-51    ...C.CUGA...CA...UAC..AACGGA.AGACAGU.AAA.CAUG
env-52    ................GCAA...G...U.CUGAA......AUAC..AACGGA.AGACAGA.UAA.AAUG
env-55    ................CUA...G...C.CUGAA......AUAC..AACGGA.AGACAGA.GAA.CAUG
env-56    ................CUA...G...C.CUGAA......AUAC..AACGGA.AGACAGA.GAA.CAUG
env-57    ................CUA...G...C.CUGAA......AUAC..AACGGA.AGACAGA.GAA.CAUG
env-59    ................CUA...G...C.CUGAA......AUAC..AACGGA.AGACAGA.UAA.AAUG
env-60    ................CUA...G...C.CUGAA......AUAC..AACGGA.AGACAGA.UAA.AAUG
env-64    ................CUA...G...C.CUGAA......AUAC..AACGGA.AGACAGA.UAA.AAUG
Rli-1-1   ...C.CGGCG..CA.U.AGCAAAACGGA.AGACAGA.CAA.CAUG
env-65    ...C.CGCUG..U..UAAGC.AAACGGA.AGACAGA.CAA.CAUG
Rde-1-1   ................GCAA...CC...C.GGCG...CA.U.AGCAAAACGGA.AGACAGA.CAA.CAUG
env-66    ................AUCGC...GA...C.GACCG.AC....AGCAAAACGGA.AGACAGA.CAA.CAUG
env-67    ................AUU...GCUU...U.GUCG...CU.UAAGC.AAACGGA.AGACAGA.AAG.CAUG
env-68    ................AUU...GCUU...U.GUCG...CU.UAAGC.AAACGGA.AGACAGA.AAG.CAUG
Rsp-15-1  ................AUUUCGGU...GGUU...CACGUCA.A...U.AGC.AAACGGA.AGACAGA.AAG.CAUG
env-69    ................UCC...UC...C.ACCCG..U..C.UGC.AAACGGA.AGACAGA..AG.CAUG
Rsp-14-1  ................C...GUUU...C.ACCCG..UUC..UGC.AAACGGA.AGACAGA..AG.CAUG
env-70    ................C...GUUU...C.ACCCG..UUC..UGC.AAACGGA.AGACAGA..AG.CAUG
env-71    ................U...CUC...C.ACCCG..UC...UGC.AAACGGA.AGACAGA..AG.CAUG
Csp-2-1   ................AUUCU...C...C.ACCCG..U..C.UGC.AAACGGA.AGACAGA..AG.CAUG
env-72    ................UGAUGGCGAAAGCCGAAU...G.AGCCG..C..AAAGC.AAAUGGA.AGACAGA.AAG.CAUG
env-74    ................GCAA...C...G.CCUGG..C..GAAGC.AGACGGA.AGACAGG.AAG.CAUG
env-75    ................AAAACCUGCGCGCGGCGGGGAGA...C.CCGC...CG.G.AGCUAAACGGA.AGACAGA.AAG.CAUG
env-76    ................AA...GGGU...U.CACCA..C..U..GCUAAACGGA.AGACAGA.AAG.CAUG
Tsp-1-1   ................GGGUGAUCGCACCCA...U.CGUCG..UU.U.AGCAAAACGGA.AGACAGU.AA..CAUG
env-77    ................UAACC...G...C.CCGC.....AAAGC..AACGGA.AGACAGA..AA.CAUG
env-78    ...G.CACC...CGAUAAGCAAAACGGA.AGACAUC..AA.CAUG
env-79    ...G.CACC...CGAUAAGCAAAACGGA.AGACAUC..AA.CAUG
Lve-2-1   ................CU...GUCC...G.GGACG..C..CAAGC.AAACGGA.AGA.AGC.UAA.CAUG
Lve-1-1   ................CU...GUCC...G.GGACG..C..CAAGC.AAACGGA.AGA.AGC.AAA.CAUG
env-83    ................AGGA...CCC...G.ACGUG..UU.U.AGCUAAACGGA.AGACAGC.AA..CAUG
env-84    ................AGGA...CCC...G.ACGUG..UU.U.AGCUAAACGGA.AGACAGC.AA..CAUG
Ogr-1-1   ................CAC...GCGCCG..CGAGAU...C.CG.C.....AAAGC..AACGGAAAGACAGU.AAA.CAUG
env-85    ................CCA...ACGC...C.UCGCA..C..U..GCUAAACGGA.AGACAGA.AAG.CAUG
env-86    ................CCA...ACGC...C.UCGCA..C..U..GCUAAACGGA.AGACAGA.AAG.CAUG
Lsp-10-1  ................AUCU...G...G.GACCG.ACA.G.AGC.AAACGGA.AGA.AGC.UAA.CAUG
env-88    ................CAAC...UGUU...U.UGCA...CAUA.....AAACGGA.AGACAGA.AAG.CAAG
env-89    ................AAACCU...CA...G.ACCC...C.....CC.UAACGGA.AGACAGA.GAG.CAUG
Aat-1-1   ................GAACCGCCCUGCGGGUCA...C.CCCCG..CAGU.UGCUAAACGGA.AGACAGA.AAG.CAUG
env-90    ................CGAAC...CGC...C.CG.C.....AAAGC..AACGGA.AGACAGA.AAG.CAUG

          <<<<<<<<<<<<<<<<...>>>>>>>>>>>>>>>>>>>>>>>>>>>>...>.>>>>...
          ....1.000.00.000...000.00.000.1..........1..22...2.2222...
          ----oooo----oo----●-●●●Yo--o--◦-◦GC-◦AACGGA-AGACAGA-AAG-CAUG
                                                                   3′
```

```
env-91    ................AAAGA...............................................................GGCC.......................UGCGUCG..UU...UGCUAAACGGA.AGACAGA.CAA.CAUG
env-92    ................GCAA.................................................................GGU.......................C.UGUG...C..A.AGC.AAACGGA.AGACAGA.GAA.CAUG
env-93    ..........................................................................................................G.GGUCG..C..GAAGC.AAACGGA.AGACAGA.GAA.CAUG
env-94    ................CACAA................................................................GAG.......................G.CUGAG..CU.A.....AAACGGA.AGACAGA.AAG.AAAA
Pna-1-1   ................CCGGCCUGGCCGCA.........................................................C.......................C.CGCCG..C..U.AGC.AAACGGA.AGACAGA.AAG.CAUG
env-96    ................ACCAU................................................................C.G.......................G.ACCCA..U..C.AGC.UGACGGA.AGACAGA.AAG.CAUG
Sna-1-1   ..........................................................................................................C.CGCCG....U.AGCUUAACGGA.AGACAGA.AAG.CAUG
env-97    ................GAAU.................................................................GCC.......................C.CG.C.....AAAGC.AAACGGA.AGACAGU.AAG.CAUG
env-98    ................CAA.................................................................GGAC.......................C.CG.CG....A.AGCACAACGGA.AGACAGG.AAG.CAUG
env-99    ................AAAU................................................................UCCC.........................GUCGG..C..CAAGC.AAACGGA.AGACAGA.AAG.CAUG
env-101   ................AAAU................................................................UCCC.........................GUCGG..C..CAAGC.AAACGGA.AGACAGA.AAG.CAUG
env-103   ................GCAAG..............................................................GGC.C.......................U.CUUCG..UU...UGCUAAACGGA.AGACAGA.AAG.CAUG
env-104   ................GCAAG..............................................................GGC.C.......................U.CUUCG..UU...UGCUAAACGGA.AGACAGA.AAG.CAUG
Jsp-1-1   ................GAAACAU.............................................................GCGG.......................G.GCCG...UC.A.AGC.AAACGGA.AGACAGU.GAA.CAUG
env-105   ................GCAG..................................................................GC.......................G.GUCCG..CA.CAAGC.AAACGGA.AGACAGU.AAA.CAUG
env-106   ..........................................................................................................A.GGCCG..C..AAAGC..AACGGAAAGACAGU.AAA.CAUG
env-108   ..........................................................................................................G.GGCCG..C..AAAGC..AACGGAAAGUCAGU.AAA.CAUG
env-109   ................GCAA...................................................................G.......................A.GGCCG..C..CAAGC.AAACGGA.AGA.AGC.UAA.CAUG
env-116   ................GCAA...................................................................G.......................A.GGCCG..C..CAAGC.AAACGGA.AGA..............
env-117   ................GCAA...................................................................G.......................A.GGCCG..C..CAAGC.AAACGGA.AGA..............
env-118   ................GAAA..................................................................GC.......................U.UCCCG..CA.CAAGC.AAACGGA.AGACAGU.UAA.CAUG
env-119   ................GCAG..................................................................GC.......................C.UCCCG..CA.CAAGC.AAACGGA.AGACAGU.UAA.CAUG
env-120   ................CA...................................................................CGC.......................C.UCCCG..CA.CAAGC.AAACGGA.AGACAGU.UAA.CAUG
env-121   ................GCAA..................................................................GC.......................C.GCCCA..CA.C.AGA.AAACGGA.AGACAGU.UAA.CAUG
env-122   ................GCAA...................................................................G.......................G.GCCCG..C..CAAGC.AAACGGA.AGA.AGC.UAA.CAUG
env-124   ................ACG.................................................................UCAG.......................G.GACCG..C..CAAGC.AAACGGA.AGA.AGCUAAU.CAUG
env-125   ................AUCU..................................................................AG.......................G.GACCG..C..CAAGC.AAACGGA.AGAAGCU.AAU.CAUG
env-127   ................AAAU..................................................................AG.......................G.GACCG..C..CAAGC.AAACGGA.AGAAGCU.AAU.CAUG
Ote-1-1   ....................................................................................GAGU.......................G.GUCCG..C..CAAGC.AAACGGA.AGU.AGC.AAA.CAUG
Oan-1-1   ....................................................................................GAGC.......................G.GUCCG..C..CAAGC.AAACGGA.AGU.AGC.AAA.CAUG
Oar-1-1   ................AUAU...................................................................G.......................G.GUCCG..C..CAAGC.AAACGGA.AGU.AGC.AAA.CAUG
env-128   ................CUCGC.................................................................GA.......................G.GCCCG..C..CAAGC.AAACGGA.AGA.AGC.AAA.CAUG
env-135   ................CUCGC.................................................................GA.......................G.GCCCG..C..CAAGC.AAACGGA.AGA.AGC.AAA.CAUG
Rsp-9-1   ................GCAA..................................................................GU.......................G.GCCCG..CC.A.AGC.AAACGGA.AGAAGCU.AA..CAUG
env-136   ................AAAU.................................................................GGU.......................U.UGGCA....U.AGCUAAACGGA.AGACAGA.AAG.CAUG
Pan-1-1   ................AAACU.................................................................GC.......................G.GUCCU..C..A.AGC.UAGCGGA.AGACAGA.GAG.CAUG
Pfe-1-1   ................AAACU.................................................................GC.......................G.GUCCU..C..A.AGC.UAGCGGA.AGACAGA.GAG.CAUG
Lci-1-1   ................AUUCG...............................................................GGAU.......................C.GCCCG..C..AAAGC..AACGGA.AGACACA..AG.CAUG
env-138   ................AAACCUGCCGCCAGUCGGGGAA................................................AC.......................C.CGAC...U..U.AGCUAAACGGA.AGACAGA.AAG.CAUG
Abo-1-1   ................CAUUGCCGUCCUCACAUAUUGGUUGAACU...........................................C.......................U.GUUCG.ACC.G.AGCUAAACGGA.AGACAGA.AAG.CAUG
Rha-1-1   ................CGCGCCGCGGCCGACCACAUAGGCGGACGCCCCGACCGGGGGC.........................GGGU.......................CGCUGC.......UGA.CAACGGA.AGACAGA.AAG.CAUG
Mal-1-1   ................ACU.................................................................UUCC.......................G.GGGCG..C..U.AGC.UAACGGA.AGACAGA.AAG.CAUG
env-139   ................CCU.................................................................UUCC.......................G.GGGCG..C..U.AGC.UAACGGA.AGACAGA.AAG.CAUG
env-141   ................CAU.................................................................GUCC.......................G.GGGCA..C..U..GCUAAACGGA.AGACAGA.AAG.CAUG
env-143   ................GACA...................................................................C.......................C.GUCCG..C..AAAGC..AACGGAAAGACAGAGAAA.CAUG
env-144   ..........................................................................................................C.GACCC.ACC.U.AGC.UGACGGA.AGACAGA.GAA.CAUG
env-145   ................UUU...................................................................GC.......................C.CUGCA.AC.....GCACAACGGA.AGACAGA.AAG.CAUG
env-146   ................UCU...................................................................GC.......................C.CUGC.....UACGCACAACGGA.AGACAGA.AAG.CAUG
env-148   ..................ACCUUCGGGGGGCUUACAUCGCGGGGGAUGGCCAUCGGGCCGAACUC.........................................GCACUG..C..U.AG..AAACGGA.AGACAGU.AAU.CAUG
env-149   ................GGCUUUGAGAUCAAAACCAGCGAGCAGGGGCUUCAUCUACGCAGAGAGCACGC.................UCU.........................UUGC......AUGC.ACACGGA.AGACAGA.AAG.CAUG
env-150   ................AAACAUAGCCCCGGGCAAAAUCCG............................................GGGC.......................A.CUGC.....U.....AACGGA.AGACAGA.AAG.CAUG
env-151   ................AAACAUAGCCCCGGGCAAAAUCCG............................................GGGC.......................A.CUGC.....U.....AACGGA.AGACAGA.AAG.CAUG
env-155   ................AAACAUAGCCCCGGGCAAAAUCCG............................................GGGC.......................A.CUGC.....U.....AACGGA.AGACAGA.AAG.CAUG
env-156   ................CAACGU..................................................................C.......................A.GGCC...CC.C.UAC..AACGGA.AGACAGA.AAG.CAUG
env-157   ....................................................................................CUGA.......................U.CGUU...UUUA....AAACGGA.AGACAGA.AAG.AAUG
env-158   ................AAUU..................................................................GA.......................U.CGUU...UA.A....AAACGGA.AGACAGA.AAG.AAUG
env-162   ................AAUU..................................................................GA.......................U.CGUU...UA.A....AAACGGA.AGACAGA.AAG.AAUG
Kvu-1-1   ................UGAC.................................................................UGC.......................C.CG.C.....AAAGC.AAACGGA.AGACAGA..AG.CAUG
env-163   ................AACU..................................................................GAU......................C.GUUUA....A....AAACGGA.AGACAGA.AAG.AAUG
Lho-1-1   ................ACA.................................................................CGAC.......................C.CG.C.....AAAGC..AACGGA.AGACACA.AAG.CAUG
CRh-1-1   ................GCUUUCUAACA.........................................................UGGC.......................C.CGCCG.UC..U.AGC.UAACGGA.AGAGAGC.AAG.CAUG
env-164   ................AUU...................................................................GC.......................C.UUGCG.AU...AGC.AAACGGA.AGACAGA.AAG.CAUG
env-165   ................AUU...................................................................GC.......................C.UUGCG.AU...AGC.AAACGGA.AGACAGA.AAG.CAUG
Ogu-1-1   ................ACC.................................................................UGGC.......................C.CGCA..C..A.AGC.AGACGGA.AGACAGA.AAG.CAUG
env-166   ................UCAGG...............................................................GUC.......................G.CGGCA..C..A.AGC.AGACGGA.AGACAGA.AAG.CAUG
Paq-1-1   ................AUUUU..................................................................G.......................U.GGCC...CU.UAAGC.UGACGGA.AGACAGA.GAG.CAUG
env-167   ................GUAA...................................................................C.......................C.UGCCG..C....AGC.UAACGGA.AGACAGA.AAG.CAUG
env-168   ................GCAA...................................................................C.......................C.UGCCG..C..AAAGC..AACGGA.AGACAGA.AAG.CAUG
env-169   ................CAAGA..................................................................C.......................C.UGCCG..U..U.AGC.UAACGGA.AGACAGA.AAG.CAUG
env-170   ................CAAGA..................................................................C.......................C.UGCCG..U..U.AGC.UAACGGA.AGACAGA.AAG.CAUG
env-171   ..........................................................................................................G.GGUCAAAC..A.AGC.AAACGGA.AGACAGC.AAG..AUG
env-172   ................UUA...............................................................UUCAAG.......................G.CUGAG..CA...UG..AAACGGA.AGACAGA.AAG.CAUG
Rba-4-1   ................AUA...............................................................UUCAAG.......................G.CUGAG..CA...UG..AAACGGA.AGACAGA.AAG.CAUG
env-173   ..........................................................................................................C.UGCCG...ACAUAGCAAACGGA.AGACAGA.AAG.CAUG
Sps-1-1   ................UUCG...................................................................G.......................U.CGCUG.AC..A.AGCAAAACGGA.AGACAGC.AAA.CAUG
Ssp-2-1   ................UUCG...................................................................G.......................U.CGCUG.AC..A.AGCAAAACGGA.AGACAGC.AAA.CAUG
env-174   ................GAA.................................................................GGGCCU.....................U.CGCCG..U..U.AGC.AAACGGA.AGACAGA.AAG.CAUG
Osp-1-1   ................AAUA...................................................................C.......................C.UGCCG..UU...UGC.UAACGGA.AGACAGA.AAG.CAUG
Sst-1-1   ................GCAA...................................................................C.......................C.GGCUG..C..U.AGC.UAACGGA.AGACAGA.AAG.CAUG
env-175   ..........................................................................................................C.CGCCC.AUA.U.AGC.AAACGG..AGACAGA..AG.CAUG
env-176   ..........................................................................................................C.CGCUG..C..A.AGC.AAACGGA.AGACAGA.AAG.CAUG
Rsp-16-1  ................UU....................................................................AC.......................C.UGCCG..U..A.AGC.AAACGGA.AGACAGA.AAG.CAUG
env-177   ................GUAA...................................................................C.......................C.CGCCG..C..AAAGC..AACGGA.AGACAGA.AAG.CAUG

          <<<<<<<<<<<<<<<<.....................................................>>>>>>>>>>>>>>>>>>>>>>>>>>>>.......................>.>>>>...................................
          ....1.000.00.000.....................................................000.00.000.1..........1..22.......................2.2222...................................
          ----------------oooo-------------------------------------------------------------------------oo----------------------•-•••Yo--o--o-oGC-oAACGGA-AGACAGA-AAG-CAUG
                                                                                                                                                                       3'
```

```
env-178    ................GCAA.............................................................................C.......................C.CGCCG..C..AAAGC..AACGGA.AGACAGA.AAG.CAUG
env-179    ................UUAU.............................................................................C.......................C.UGCCG....UAAGC.AAACGGA.AGACAGA.AAG.CAUG
env-186    ................UUAU.............................................................................C.......................C.UGCCG....UAAGC.AAACGGA.AGACAGA.AAG.CAUG
Rsp-13-1   ................GUAU.............................................................................C.......................C.CGCCG....UAAGC.AAACGGA.AGACAGA.AAG.CAUG
env-187    ................GCAU.............................................................................C.......................C.CGCCG....UAAGC.AAACGGA.AGACAGA.AAG.CAUG
Rmu-1-1    ................GCAU.............................................................................C.......................C.CGCCG....UAAGC.AAACGGA.AGACAGA.AAG.CAUG
env-188    ................AACCAG.........................................................................GCC.......................C.CGCCG..C..U.AGC.AAACGGA.AGACAGA.AAG.CAUG
env-189    ................AGG............................................................................GCC.......................C.UGCCG..U..U.AGC.AAACGGA.AGACAGA.AAG.CAUG
env-190    ................AGG............................................................................GCC.......................C.UGCCG..U..U.AGC.AAACGGA.AGACAGA.AAG.CAUG
env-191    ................AGG............................................................................GCC.......................C.UGCCG..C..U.AGC.AGACGGA.AGACAGA.AAG.CAUG
env-192    ................AGG............................................................................GCC.......................U.UGCCG....UAAGC.AAACGGA.AGACAGA.AAG.CAUG
env-193    ................GAUCG...........................................................................CU.......................U.UGCCG..CA.UAAGC.AAACGGA.AGACAGA.AAG.CAUG
env-194    ................UGU............................................................................GCU.......................U.UGCCG..CA.UAAGC.AAACGGA.AGACACA.AAG.CAUG
env-195    ................GAU............................................................................GCU.......................U.UGCCG..CA.UAAGC.AAACGGA.AGACACA.AAG.CAUG
env-196    ................GAU............................................................................GCU.......................U.UGCCG..CA.CAAGC.AAACGGA.AGACACA.AAG.CAUG
env-197    ................GAU............................................................................GCU.......................U.UGCCG..CA.CAAGC.AAACGGA.AGACACA.AAG.CAUG
env-200    ................GAU............................................................................GCU.......................U.UGCCG..CA.CAAGC.AAACGGA.AGACACA.AAG.CAUG
env-201    ................AUC............................................................................GCU.......................U.UGCCA..CA.UAAGC.AAACGGA.AGACAGA.AAG.CAUG
env-202    ................AUC............................................................................GCU.......................U.UGCCG..CA.UAAGC.AAACGGA.AGACAGA.AAG.CAUG
env-203    ................AUC............................................................................GCU.......................U.UGCCG..CA.UAAGC.AAACGGA.AGACAGA.AAG.CAUG
env-204    ................AUC............................................................................GCU.......................U.UGCCG..CA.UAAGC.AAACGGA.AGACAGA.AAG.CAUG
env-205    ................AUC............................................................................GCU.......................U.UGCCG..CU.UAAGC.AAACGGA.AGACAGA.AAG.CAUG
env-206    ................AUC............................................................................GCU.......................U.UGCCG..CU.UAAGC.AAACGGA.AGACAGA.AAG.CAUG
env-207    ................AUC............................................................................GCU.......................U.UGCCG..CU.UAAGC.AAACGGA.AGACAGA.AAG.CAUG
env-213    ................AUC............................................................................GCU.......................U.UGCCG..CU.UAAGC.AAACGGA.AGACAGA.AAG.CAUG
env-214    ................AUC............................................................................GCU.......................U.UGCCG..CU.UAAGC.AAACGGA.AGACAGA.AAG.CAUG
env-215    ................AUC............................................................................GCU.......................U.UGCCG..CU.UAAGC.AAACGGA.AGACAGA.AAG.CAUG
env-216    ................UUU............................................................................GCU.......................U.UGUCG..CU.UAAGC.AAACGGA.AGACAGA.AAG.CAUG
env-217    ................AUU............................................................................GCU.......................U.UGUCG..UU.UAAGC.AAACGGA.AGACAGA.AAG.CAUG
env-218    ................AUU............................................................................GCU.......................U.UGUCG..CU.UAAGC.AAACGGA.AGACAGA.AAG.CAUG
env-219    ................AUU............................................................................GCU.......................U.UGUCG..CU.UAAGC.AAACGGA.AGACAGA.AAG.CAUG
env-220    ................AUU............................................................................GCU.......................U.UGUCG..CU.UAAGC.AAACGGA.AGACAGA.AAG.CAUG
env-221    ................AUU............................................................................GCU.......................U.UGUCG..CU.UAAGC.AAACGGA.AGACAGA.AAG.CAUG
env-222    ................AUU............................................................................GCU.......................U.UGUCG..CU.UAAGC.AAACGGA.AGACAGA.AAG.CAUG
env-223    ................AUU............................................................................GCU.......................U.UGUCG..CU.UAAGC.AAACGGA.AGACAGA.AAG.CAUG
env-224    ................AUU............................................................................GCU.......................U.UGUCG..CU.UAAGC.AAACGGA.AGACAGA.AAG.CAUG
env-225    ................AUU............................................................................GCU.......................U.UGUCG..CU.UAAGC.AAACGGA.AGACAGA.AAG.CAUG
env-226    ................AUU............................................................................GCU.......................U.UGUCG..CU.UAAGC.AAACGGA.AGACAGA.AAG.CAUG
env-227    ................AUU............................................................................GCU.......................U.UGUCG..CU.UAAGC.AAACGGA.AGACAGA.AAG.CAUG
env-228    ................GUU............................................................................GCU.......................U.UGCCG..CA.UAAGC.AAACGGA.AGACACA.AAG.CAUG
env-229    ................U............................................................................UACU.......................U.UGCCG..UACA.AGC.AAACGGA.AGACAGA.AAG.CAUG
env-230    ................AUU............................................................................ACU.......................U.UGCCG..UA.CAAGC..AACGG..AGACAGA.AAG.CAUG
env-231    ................AAU............................................................................ACU.......................U.UGCCA..UA.CAAGC.AAACGGA.AGACAGA.AAG.CAUG
env-232    ................AAA............................................................................ACU.......................U.UGCCG..UA.CAAGC.AAACGGA.AGACAGA.AAG.CAUG
env-233    ................AAU............................................................................ACU.......................U.UGUCG..UA.CAAGC.AAACGGA.AGACAGA.AAG.CAUG
env-234    ................AAU............................................................................ACU.......................U.UGUCG..UA.CAAGC.AAACGGA.AGACAGA.AAG.CAUG
env-235    ................AAU............................................................................ACU.......................U.UGCCG..UA.CAAGC.AAACGGA.AGACAGA.AAG.CAUG
env-236    ................AAU............................................................................ACU.......................U.UGCCG..UA.CAAGC.AAACGGA.AGACAGA.AAG.CAUG
env-237    ................AAU............................................................................ACU.......................U.UGCCG..UA.CAAGC.AAACGGA.AGACAGA.AAG.CAUG
env-247    ................AAU............................................................................ACU.......................U.UGCCG..UA.CAAGC.AAACGGA.AGACAGA.AAG.CAUG
env-248    ................AUU............................................................................ACU.......................U.UGCCG..UA.CAAGC.AAACGGA.AGACAGA.AAG.CAUG
env-252    ................AUU............................................................................ACU.......................U.UGUC...UUACAAGC.AAACGGA.AGACAGA.AAG.CAUG
env-254    ................AUU............................................................................ACU.......................U.UGUCG..UA.AAAGC.AAACGGA.AGACAGA.AAG.CAUG
env-255    ................ACU............................................................................ACU.......................U.UGUCG..UA.CAAGC.AAACGGA.AGACAGA.AAG.CAUG
env-256    ................AUU............................................................................ACU.......................U.UGCCG..UA.CAAGC.AAACGGA.AGACAGA.AAG.CAUG
env-257    ................AUU............................................................................ACU.......................U.UGUCG..UA.CAAGC.AAACGGA.AGACAGA.AAG.CAUG
env-258    ................AUU............................................................................ACU.......................U.UGUCG..UA.CAAGC.AAACGGA.AGACAGA.AAG.CAUG
env-259    ................CAUGA............................................................................C.......................C.CGCUG..C..A.AGC.AAACGGA.AGACAGA.AAG.CAUG
Rsp-8-1    ................UCAACG........................................................................GGCG.......................C.CGCCG..U..U.AGC.AAACGGA.AGACAGA.AAG.CAUG
env-260    .........................................................................................................................C.UGCCG..C..UAAGC.AAACGGA.AGACAGA.AAG.CAUG
env-261    .........................................................................................................................C.UGCCG..C..UAAGC.AAACGGA.AGACAGA.AAG.CAUG
env-262    .........................................................................................................................C.UGCCG..C..UAAGC.AAACGGA.AGACAGA.AAG.CAUG
env-263    .........................................................................................................................C.UGCCG..C..UAAGC.AAACGGA.AGACAGA.AAG.CAUG
env-264    .........................................................................................................................C.UGCCG..C..UAAGC.AAACGGA.AGACAGA.AAG.CAUG
env-265    .........................................................................................................................C.UGCCG..C..UAAGC.AAACGGA.AGACAGA.AAG.CAUG
env-266    .........................................................................................................................C.UGCCG..C..UAAGC.AAACGGA.AGACAGA.AAG.CAUG
env-269    .........................................................................................................................C.UGCCG..C..UAAGC.AAACGGA.AGACAGA.AAG.CAUG
env-271    .........................................................................................................................C.UGCCG..C..UAAGC.AAACGGA.AGACAGA.AAG.CAUG
env-272    .........................................................................................................................C.UGCCG..C..UAAGC.AAACGGA.AGACAGA.AAG.CAUG
env-273    .........................................................................................................................C.UGCCG..CU.C.AGC.AAACGGA.AGACAGA.AAG.CAUG
env-274    .........................................................................................................................C.CGCCG.ACA.U.AGC.AAACGG..AGACAGA.AAG.CAUG
env-275    .........................................................................................................................C.CACCG.GCA.U.AGC.AAACGGA.AGACAGA.AAG.CAUG
env-276    ................A...............................................................................GA.......................C.CGCCG.ACA.U.AGC.AAACGGA.AGACAGA.AAG.CAUG
env-277    ..........................................................................................................................CGCCA.ACA.U.AGC.AGACGGA.AGACAGA.AAG.CAUG
env-278    .........................................................................................................................A.UGCCG.ACA.U.AGC.AAACGGA.AGACAGA.AAG.CAUG
env-279    .........................................................................................................................C.CGCCG.ACA.C.AGC.AAACGGA.AGACAGA.AAG.CAUG
env-280    .........................................................................................................................U.CGCCG.ACA.U.AGC.AAACGGA.AGACAGA.AAG.CAUG
env-282    .........................................................................................................................U.CGCCG.ACA.U.AGC.AAACGGA.AGACAGA.AAG.CAUG
env-284    .........................................................................................................................U.CGCCG.ACA.U.AGC.AAACGGA.AGACAGA.AAG.CAUG
env-285    .........................................................................................................................U.CGCCG.ACA.U.AGC.AAACGGA.AGACAGA.AAG.CAUG
env-286    .........................................................................................................................C.CGCCG.ACA.U.AGC.AAACGGA.AGACAGA.AAG.CAUG
env-287    .........................................................................................................................C.CGCCG.ACA.C.AGC.AAACGGA.AGACAGA.AAG.CAUG
env-288    .........................................................................................................................C.CGCCG.ACA.U.AGC.AAACGGA.AGACAGA.AAG.CAUG

           <<<<<<<<<<<<<<<<......................................................>>>>>>>>>>>>>>>>>>>>>>>>>>>>.......................>.>>>>....................................
           ....1.000.00.000......................................................000.00.000.1..........1..22.......................2.2222....................................
           ----------------oooo----------------------------------------------------------------------------oo-----------------------●-●●●Y○--○--●-●GC-●AACGGA-AGACAGA-AAG-CAUG
                                                                                                                                                                 3′
```

```
env-289    .........................................................................................................................C.UGCCG.AUA.U.AGC.AAACGGA.AGACAGA.AAG.CAUG
env-290    .........................................................................................................................C.CGCCG.ACA.U.AGC.AAACGGA.AGACAGA.AAG.CAUG
env-291    .........................................................................................................................C.CGCCG.ACA.U.AGC.AAACGGA.AGACAGA.AAG.CAUG
env-293    .........................................................................................................................C.CGCCG.ACA.U.AGC.AAACGGA.AGACAGA.AAG.CAUG
env-295    .........................................................................................................................C.CGCCG.ACA.U.AGC.AAACGGA.AGACAGA.AAG.CAUG
env-296    .........................................................................................................................C.CGCCG.ACA.U.AGC.AAACGGA.AGACAGA.AAG.CAUG
env-297    .........................................................................................................................C.CGUCG.ACA.U.AGC.AAACGGA.AGACAGA.AAG.CAUG
env-298    .........................................................................................................................C.CGUCG.ACA.U.AGC.AAACGGA.AGACAGA.AAG.CAUG
env-299    .........................................................................................................................C.CGCCG.ACA.U.AGC.AAACGGA.AGACAGA.AAG.CAUG
env-300    .........................................................................................................................C.UGCCG.ACA.U.AGC.AAACGGA.AGACAGA.AAG.CAUG
env-301    .........................................................................................................................C.CGCUG.ACA.U.AGC.AAACGGA.AGACAGA.AAG.CAUG
env-308    .........................................................................................................................C.UGCCG.ACG.U.AGC.AAACGGA.AGACAGA.AAG.CAUG
env-309    .........................................................................................................................C.UGCCG.ACA.U.AGC.AAACGGA.AGACAGA.AAG.CAUG
env-310    .........................................................................................................................C.UGCCG.ACA.U.AGC.AAACGGA.AGACAGA.AAG.CAUG
env-311    .........................................................................................................................C.UGCCG.ACA.U.AGC.AAACGGA.AGACAGA.AAG.CAUG
env-312    .........................................................................................................................C.UGCCG.ACA.U.AGC.AAACGGA.AGACAGA.AAG.CAUG
env-313    .........................................................................................................................C.UGCCG.ACA.U.AGC.AAACGGA.AGACAGA.AAG.CAUG
env-314    .........................................................................................................................C.CGCCG.ACA.U.AGC.AAACGGA.AGACAGA.AAG.CAUG
env-315    .........................................................................................................................C.CGCCG.ACA.U.AGC.AAACGGA.AGACAGA.AAG.CAUG
env-316    .........................................................................................................................C.CGCCG.ACA.U.AGC.AAACGGA.AGACAGA.AAG.CAUG
env-317    .........................................................................................................................C.CGCCG.ACA.U.AGC.AAACGGA.AGACAGA.AAG.CAUG
env-319    .........................................................................................................................C.CGCCG.ACA.U.AGC.AAACGGA.AGACAGA.AAG.CAUG
env-320    .........................................................................................................................C.CGCCG.ACA.U.AGC.AAACGGA.AGACAGA.AAG.CAUG
env-321    .........................................................................................................................C.CGCCG.ACA.U.AGC.AAACGGA.AGACAGA.AAG.CAUG
env-322    .........................................................................................................................C.CGCCG.ACA.U.AGC.AAACGGA.AGACAGA.AAG.CAUG
env-323    .........................................................................................................................C.CGCCG.ACA.U.AGC.AAACGGA.AGACAGA.AAG.CAUG
env-324    .........................................................................................................................C.CGCCG.ACA.U.AGC.AAACGGA.AGACAGA.AAG.CAUG
env-325    .........................................................................................................................C.CGCCG.ACA.U.AGC.AAACGGA.AGACAGA.AAG.CAUG
env-326    .........................................................................................................................C.CGCCG.ACA.U.AGC.AAACGGA.AGACAGA.AAG.CAUG
env-327    .........................................................................................................................C.CGCCG.ACA.U.AGC.AAACGGA.AGACAGA.AAG.CAUG
env-329    .........................................................................................................................C.CGCCG.ACA.U.AGC.AAACGGA.AGACAGA.AAG.CAUG
env-330    .........................................................................................................................C.CGCCG.ACA.U.AGC.AAACGGA.AGACAGA.AAG.CAUG
env-331    .........................................................................................................................C.CGCCG.ACA.U.AGC.AAACGGA.AGACAGA.AAG.CAUG
env-332    .........................................................................................................................C.CGCCG.ACA.U.AGC.AAACGGA.AGACAGA.AAG.CAUG
env-335    .........................................................................................................................C.CGCCG.ACA.U.AGC.AAACGGA.AGACAGA.AAG.CAUG
env-336    .........................................................................................................................C.CGCCG.ACA.U.AGC.AAACGGA.AGACAGA.AAG.CAUG
env-337    .........................................................................................................................C.CGCCG.ACA.U.AGC.AAACGGA.AGACAGA.AAG.CAUG
env-338    .........................................................................................................................C.CGCCG.ACA.U.AGC.AAACGGA.AGACAGA.AAG.CAUG
env-339    .........................................................................................................................C.CGCCG.ACA.U.AGC.AAACGGA.AGACAGA.AAG.CAUG
env-340    .........................................................................................................................C.CGCCG.ACA.U.AGC.AAACGGA.AGACAGA.AAG.CAUG
env-341    .........................................................................................................................C.CGCCG.ACA.U.AGC.AAACGGA.AGACAGA.AAG.CAUG
env-342    .........................................................................................................................C.CGCCG.ACA.U.AGC.AAACGGA.AGACAGA.AAG.CAUG
env-343    .........................................................................................................................C.CGCCG.ACA.U.AGC.AAACGGA.AGACAGA.AAG.CAUG
env-344    .........................................................................................................................C.CGCCG.ACA.C.AGC.AAACGGA.AGACAGA.AAG.CAUG
env-345    .........................................................................................................................C.CGCCG.ACA.U.AGC.AAACGGA.AGACAGA.AAG.CAUG
env-346    .........................................................................................................................C.CGCCG.ACA.C.AGC.AAACGGA.AGACAGA.AAG.CAUG
env-347    .........................................................................................................................C.CGCCG.ACA.C.AGC.AAACGGA.AGACAGA.AAG.CAUG
env-356    .........................................................................................................................C.CGCCG.ACA.C.AGC.AAACGGA.AGACAGA.AAG.CAUG
env-357    .........................................................................................................................C.CGCCG.ACA.U.AGC.AAACGGA.AGACAGA.AAG.CAUG
env-358    .........................................................................................................................C.CGCCA.ACA.U.AGC.AAACGGA.AGACAGA.AAG.CAUG
env-359    .........................................................................................................................C.CGCCA.ACA.U.AGC.AAACGGA.AGACAGA.AAG.CAUG
env-360    .........................................................................................................................A.CGCCA.AAA.U.AGC.AGACGGA.AGACAGA.AAG.CAUG
env-361    .........................................................................................................................A.CGCCA.ACA.U.AGC.AGACGGA.AGACAGA.AAG.CAUG
Psp-3-1    ................CUG...........................................................................UUCC.......................C.UGCCG.UC..U..GC.UAACGGA.AGACAGA.AAG.CAUG
Hmi-1-1    ................AUUCG...........................................................................GCCG.....................C.GCCCG..C..A.AGC.AGACGGA.AGACAGA.AAG.CAUG
Hma-1-1    ................AUUCG..........................................................................GCC.......................GCGCCCG..C...AAGC.AGACGGA.AGACAGA.AAG.CAUG
Laq-1-1    ................ACAAAAAC....................................................................CUAAGGG......................C.CUGCA.......CGCUAAACGGA.AGACAGA.AAG.CAUG
env-362    ................AUCAAAUCCCACCCUACAUAGGUGGGGCGAAAUC...............................................C.......................C.UGCCG..UU...UGCUAAACGGA.AGACAGA.AAG.CAUG
Rpo-1-1    ................GGAA.............................................................................C.......................C.CGCCG..U....UGCUAAACGGA.AGACAGA.AAG.CAUG
env-363    ................UAAU...........................................................................GGG.......................U.CGC.G..C..UAAGC.AAACGGA.AGACAGA.AAG.CAUG
env-364    ................AACAUA..................................................................GCGAUGGGGAAAC....................U.CAUC...C..U.UUCUAAACGGA.AGACAGA.AAG.CAUG
Sla-1-1    ..............................................................................................GAUC.......................C.UCGCG..CU...UGCUAAACGGA.AGACAGA.AAG.CAUG
Rha-2-1    ................AUGA.............................................................................C.......................C.CCGCA..CU...UGCUAAACGGA.AGACAGA.AAG.CAUG
Rsp-17-1   ................AAAU.............................................................................C.......................C.CCGCA..CU...UGCUAAACGGA.AGACAGA.AAG.CAUG
Rsp-18-1   ................AAAUC............................................................................C.......................C.CUGCA..CU...UGCUAAACGGA.AGACAGA.AAG.CAUG
Rsp-19-1   ................AAACC............................................................................C.......................C.CUGCA..CU...UGCUAAACGGA.AGACAGA.AAG.CAUG
Rsp-22-1   ................AAACC............................................................................C.......................C.CUGCA..CU...UGCUAAACGGA.AGACAGA.AAG.CAUG
Rba-2-1    ................AGC.............................................................................GC.......................C.UCGCA..CU...UGCUAAACGGA.AGACAGA.AAG.CAUG
env-365    ................AUUU.....................................................................................................G.AUCGU..UUAA.....AAACGGA.AGACAGA.AAG.AAUG
env-367    ................AAUUGAU..................................................................................................C.GUUU....A.A.....AAACGGA.AGACAGA.AAG.AAUG
env-369    GGCGC...........AAACCAGA.........................................................GAG.CGGGCCCUUGGGU.......................C.CGACG.........C.AAACGGA.AGACAGA.AAG.CAUG
env-370    ................CCAU............................................................................AC.......................C.CGGCG..C..AAACC..AACGGA.AGACAGA.AAG.CAUG
env-371    ................CCAU............................................................................AC.......................C.CGGCG..C..AAACC..AACGGA.AGACAGA.AAG.CAUG
env-372    ................CCAU............................................................................AC.......................C.CGGCG..CG.A.AGC.AAACGGA.AGACAGA.AAG.CAUG
env-373    ................GCAA...........................................................................GGG.......................G.CGCCG..C..CAAGC.AAACGGA.AGACAGA.AA..CAUG
env-374    ................GCAA...........................................................................GGG.......................G.CGCCG..C..CAAGC.AAACGGA.AGACAGA..AA.CAUG
env-375    ................UACG.....................................................................................................A.ACCC...C..U.AGA.UGACGGA.AGACAGA.AAG.CAUG
env-378    ................AAGUUAUC.........................................................................G.......................G.GCCCG..C..U.AGC.UGACGGA.AGACAGA.AAG.CAUG
env-379    ................AAGUUAUC.........................................................................G.......................G.GCCCG..C..U.AGC.UGACGGA.AGACAGA.AAG.CAUG
env-380    ................UUUUU..........................................................................GUC.......................U.UGCA...CG.U..AC..AACGGA.AGACAGA.AAG.CAUG
env-381    ................AUACCCGGCGCGACCCAAAGAGCGGACCUC................................................UGGU.......................C.CGACG..C..A.....AAACGGA.AGACAGA.AAG.CAUG
Osp-2-1    ................AAAG.............................................................................C.......................G.ACCCG..UA.U.AGCUAAACGGA.AGAGAGC.AAG.CAUG
env-382    ................GCAA..........................................................................CGCU.......................G.CG.CG.....A.AGC.AGACGGA.AGACAGA.AAG.CAUG

           <<<<<<<<<<<<<<<<......................................................>>>>>>>>>>>>>>>>>>>>>>>>>>>>.......................>.>>>>....................................
           ....1.000.00.000......................................................000.00.000.1...........1..22.......................2.2222....................................
           ----------------○○○○----------------------------------------------------------------------------○○-----------------------●-●●●Y○--○--●-●GC-●AACGGA-AGACAGA-AAG-CAUG
                                                                                                                                                                    3′
```

```
env-386   ................GCAA..........................................................................CGUC.......................C.CG.CG.....A.AGC.AGACGGA.AGACAGA.AAG.CAUG
env-388   ................GCAA..........................................................................CGGU.......................G.CGCG......A.AGC.AGACGGA.AGACAGA.AAG.CAUG
Rme-1-1   ................GUAC...........................................................................GGU.......................G.CG.CG.....A.AGC.AGACGGA.AGACAGA.AAG.CAUG
env-389   ................GCAA..........................................................................CGGU.......................C.CG.CG.....A.AGC.AGACGGA.AGACAGA.AAG.CAUG
env-391   ................GCAA..........................................................................CGGU.......................C.CG.CG.....A.AGC.AGACGGA.AGACAGA.AAG.CAUG
env-392   ................UAAU............................................................................GG.......................G.UCGCG..C..UAAGC.AAACGGA.AGACAGA.AAG.CAUG
env-394   ................UAAU............................................................................GG.......................G.UCGCG..C..UAAGC.AAACGGA.AGACAGA.AAG.CAUG
env-395   CC..............GCAA................................................................GGACCGAGGGGCGU.......................U.AUGCG..C..A.AGC.UAACGGA.AGACAGA.AAG.CAUG
env-396   ................GCAGGCAAGCGCCGAG...............................................................GGG.......................U.CCGUG.....U..GCUAGACGGA.AGACAGA.AAG.CAUG
Pxi-1-1   ................AAAGACAAGAACCUAA...............................................................GGA.......................C.CCGUG.....U..GCUAGACGGA.AGACAGA.AAG.CAUG
Rmo-1-1   ................AAAGACAAGAACCUAA...............................................................GGC.......................A.CUGCA.......CGCUAAACGGA.AGACAGA.AAG.CAUG
Rsp-21-1  ................AAAGAUAAUAACCUAA...............................................................GGC.......................U.CGGCA..C.....GCUAAACGGA.AGACAGA.AAG.CAUG
Psp-1-1   ................GAAGACAAAAACCUAA...............................................................GGU.......................C.UGGCA.......CGCUAAACGGA.AGACAGA.AAG.CAUG
Pga-1-1   ................AAAGACAAAAACCUAA...............................................................GGG.......................U.CUGCA.......CGCUAAACGGA.AGACAGA.AAG.CAUG
Pin-1-1   ................AAAGACAAAAACCUAA...............................................................GGG.......................U.CUGCA.......CGCUAAACGGA.AGACAGA.AAG.CAUG
Psp-2-1   ................AAAGACAAAAACCUAA...............................................................GGG.......................U.CUGCA.......CGCUAAACGGA.AGACAGA.AAG.CAUG
Rsp-11-1  ................AAAGACAAAAACCUAG...............................................................GGA.......................U.CCGCA.......CGCUAAACGGA.AGACAGA.AAG.CAUG
Par-1-1   ................AAAGACAAUAACCUAA...............................................................GGG.......................U.CCGCA.......CGCUAAACGGA.AGACAGA.AAG.CAUG
env-397   ................AAAGACAAGAACCUAA...............................................................GGA.......................C.CA.CG..C..A.CGCUAAACGGA.AGACAGA.AAG.CAUG
env-398   ................AAAGAAAAUAACCUAA...............................................................GGC.......................U.CGGC......A.AGCUAAACGGA.AGACAGA.AAG.CAUG
Rsp-12-1  ................AAAGACAACAACCUAA...............................................................GGC.......................U.CGGCA..C.....GCUAAACGGA.AGACAGA.AAG.CAUG
Lsp-7-1   ................AAAGACAAGAACCUAA...............................................................GGC.......................C.UGGCA.......CGCUAAACGGA.AGACAGA.AAG.CAUG
Lsp-1-1   ................AAAGACAAGAACCUAA...............................................................GGC.......................C.UGGCA.......CGCUAAACGGA.AGACAGA.AAG.CAUG
Lsp-3-1   ................AAAGACAAGAACCUAA...............................................................GGC.......................C.UGGCA.......CGCUAAACGGA.AGACAGA.AAG.CAUG
Lsp-4-1   ................AAAGACAAGAACCUAA...............................................................GGC.......................C.UGGCA.......CGCUAAACGGA.AGACAGA.AAG.CAUG
Lsp-6-1   ................AAAGACAAGAACCUAA...............................................................GGC.......................C.UGGCA.......CGCUAAACGGA.AGACAGA.AAG.CAUG
Pga-2-1   ................AAAGACAAAAACCUAA...............................................................GGU.......................C.UGGCA.......CGCUAAACGGA.AGACAGA.AAG.CAUG
Nsp-1-1   ................AAAGACAAAAACCUAA...............................................................GGU.......................C.UGGCA.......CGCUAAACGGA.AGACAGA.AAG.CAUG
Lsp-2-1   ................AAAGACAAUAACCUAA...............................................................GGC.......................C.UGGCA.......CGCUAAACGGA.AGACAGA.AAG.CAUG
Rba-7-1   ................AAAGACAAGAACCUAA...............................................................GGG.......................C.CUGCA.......CGCUAAACGGA.AGACAGA.AAG.CAUG
Lda-1-1   ................AAAGACAAGAACCUAA...............................................................GGG.......................C.CUGCA.......CGCUAAACGGA.AGACAGA.AAG.CAUG
Lsp-8-1   ................AAAGACAAUAACCUAA...............................................................GGG.......................U.CUGCA.......CGCUAAACGGA.AGACAGA.AAG.CAUG
env-399   ................CAAG.....................................................................................................G.GCCC...C..NAAAC.AGACGGA.AGACAGA.AAC.CAUG
env-400   ................CAAG.....................................................................................................G.GCCC...C..CAAAC.AGACGGA.AGACAGA.AAC.CAUG
env-402   ................UUUG.....................................................................................................G.GCCC...CU.G.AAC.AGACGGA.AGACAGA.AAC.CAUG
env-403   ................UAUUG.........................................................................AGAC.......................A.CGCU......A.AGC.AAACGGA.AGACAGA.AAG.CAUG
env-404   ................UAUUG.........................................................................AGAC.......................A.CGCU......A.AGC.AAACGGA.AGACAGA.AAG.CAUG
env-405   ................AUAA..........................................................................AGAU.......................A.CGCU......A.AGC.AAACGGA.AGACAGA.AAG.CAUG
env-407   ................AUAA..........................................................................AGAU.......................A.CGCU......A.AGC.AAACGGA.AGACAGA.AAG.CAUG
env-413   ................AUAA..........................................................................AGAC.......................A.CGCU......A.AGC.AAACGGA.AGACAGA.AAG.CAUG
env-414   ................GCAA.............................................................................C.......................C.CGGCAGACA.U.AGCAAAACGGA.AGACAGC.AAU.CAUG
Rel-1-1   ................UGAC.....................................................................................................C.CCCCG..C..AAAGC..AACGGA.AGACAGA.AAG.CAUG
env-416   ................UGAC.....................................................................................................C.CCCCG..C..AAAGC..UACGGA.AGACAGA.AAG.CAUG
env-417   ................ACAU...........................................................................GGGU......................U.UGAAG..CU.A.....AAACGGA.AGACAGA.AAGUAAUG
env-418   ................GCAA.....................................................................................................G.GCCCG.AU....AGC.UAACGGA.AGACAGA.GAA.CAUG
env-419   ................GUAA.....................................................................................................G.GCCCG.AU....AGC.UAACGGA.AGACAGA.GAA.CAUG
env-421   ................GCAA.....................................................................................................G.ACCCG..C....AGC.AGACGGA.AGACAGA..AA.CAUG
env-422   ................UCACCCACCCUACACAUAGGCGAAUUCAAG.................................................GAU.......................U.CGCCA..C..U..GCUAAACGGA.AGACAGA.AAG.CAUG
env-423   ................UCACCCACCCUACAUAAGACGGGGA......................................................GAC.......................C.CGUC.....UAAGCUAAACGGA.AGACAGA.AAG.CAUG
env-426   ................UCACCCACCCUACAUAAGACGGGGA......................................................GAC.......................C.CGUC.....UAAGCUAAACGGA.AGACAGA.AAG.CAUG
Msp-1-1   ................ACCGA............................................................................C.......................C.CGUCG..CCACAAUC..AACGGA.AGACAGA.AAG.CAUG
Sno-1-1   ................ACACCGGCCUUCAAAAGCCAUCU..................................................................................C.UGCCG..CA.C.AGCUAAACGGA.AGACAGA.CAA.CAUG
Sno-2-1   ................ACACCGGCCUUCAAAAGCCAUCU..................................................................................C.UGCCG..CA.C.AGCUAAACGGA.AGACAGA.CAA.CAUG
env-427   ................UUAAUG...................................................................................................G.GUCG...CACUAAGC.AAACGGA.AGACAGA.AAG.CAUG
env-428   ................GCAA..........................................................................CCCG.......................U.CU.C......A.AGC.UAACGGA.AGACAGA.AAG.CAUG
Tin-1-1   ................GCAA..........................................................................CCCG.......................U.CU.C......A.AGC.UAACGGA.AGACAGA.AAG.CAUG
Pwa-1-1   ................AUCA...........................................................................ACA.......................G.ACCC...C..CAAGC.UAACGGA.AGACAGA.GAG.CAUG
env-429   ................AC............................................................................GCCA.......................G.ACCCU..C..U.AGC.UGACGGA.AGACAGA.AAG.CAUG
env-430   ................AACU...........................................................................CCA.......................G.ACCCU..C..U.AGC.UGACGGA.AGACAGA.AAG.CAUG
env-431   ................AACA...........................................................................ACA.......................G.ACCCU..C..U.AGC.UGACGGA.AGACAGA.AAG.CAUG
env-432   ................AACA...........................................................................GCA.......................G.ACCCU..C..U.AGC.UGACGGA.AGACAGA.AAG.CAUG
env-433   ................UGGCACAUCA...............................................................................................G.ACCCU..C..U.AGC.UGACGGA.AGACAGA.AAG.CAUG
Osp-3-1   ................UACUUCCAACCGCCCGGCAAAUACCCAAUACAUAGCCGCCUCACGUGGACCC.........................CGGCCCGACUUUUCCGGACCAAACGAGGGCGGCG..C..U.UGCUAAACGGA.AGACAGA.AAG.CAUG
env-434   ................UUCG...........................................................................GGG.......................U.UGCCG.....A.AGC.AAACGGA.AGACAGA.AAG.CAUG
env-435   ................CCUCGC...................................................................................................G.CC.CA..C..AAAGC..AACGGA.AGACAGA.AAG.CAUG
env-436   ................CGACCCGCCCUACAUAACCGGCAGGGA....................................................AAC.......................C.UGUCG.....A.AGCUAAACGGA.AGACAGA.AAG.CAUG
env-437   ................UUACACCCACCCGAUAUAGCGGGCCCAAG..................................................GGC.......................G.CGCA...UU...UGC.AAACGGA.AGACAGA.AAG.CAUG
env-438   ................ACAACACA...................................................................GGCGGGGCGAC...................C.CGUCG.....CAAGC.UAACGGA.AGACAGA.AAG.CAUG
env-439   ................CACCCACCCUACAUAAGACGGGG.......................................................AAAC.......................C.CGUC.....UAAGCUAAACGGA.AGACAGA.AAG.CAUG
Rsp-10-1  ................AACAUGUU......................................................................GAAG.......................U.CGGG..CA.CAAGCAAAACGGA.AGACAGA.AAA.CAUG
Tar-1-1   ................GGCGAC.........................................................................CCGU......................U.UGCUG..C....AAC..AACGGA.AGACAGA.AAG.CAUG
env-440   .........................................................................................................................C.CCCG...C..AAAGC..AACGGA.AGACAGA.AAG.CAUG
Sme-2-1   ................UUCG...........................................................................GGG.......................U.GGCA...CUAC.AGCUAAACGGA.AGACAGA.CAA.CAUG
Sme-1-1   ................UUCG...........................................................................GGG.......................U.GGCA...CUAC.AGCUAAACGGA.AGACAGA.CAA.CAUG
Tsp-3-1   ................UACG............................................................................GG.......................G.CGUCG..C..CAAGC.AAACGGA.AGACAGAUAAU.CAUG
env-441   ................ACCCUACACAUAGCGGACCCCACAAA....................................................GGGU.......................A.CG.CG..UU...UGCUAAACGGA.AGACAGA.AAG.CAUG
env-445   ................CCAU............................................................................AC.......................C.CGGCG..CA.G.ACC..AACGGA.AGACAGA.AAG.CAUG
Rto-1-1   ................AUCUUACAUAGCGGGCCUUC...........................................................GGG.......................C.ACGCG..UU...UGCUAAACGGA.AGACAGA.AAG.CAUG
Ris-1-1   ................GAUCGCUCCGGCCGACACCAGGCGGACAGGCGGGCCCGAGGGCACGC................................AGG.......................C.CGCCA..U..C.UGA.AAACGGA.AGACAGG.AAG.CAUG
env-447   ................CACCGAUACACAUAGCGGAACCUU......................................................UGGU.......................U.CUGCA..C..U..GCUAAACGGA.AGACAGA.AAG.CAUG
env-448   ................CCAUCCGACAUACAGGCGGGGCA.........................................................AC.......................C.CGCCG..C..U.AGC.UAACGGA.AGACAGA.AAG.CAUG
Tpa-1-1   ................GUAA.............................................................................C.......................U.CGCCG..C..AAACC..AACGGA.AGACAGA.AAG.CAUG
env-458   ................GCAA.............................................................................C.......................U.CGCCG..C..AAACC..AACGGA.AGACAGA.AAG.CAUG

          <<<<<<<<<<<<<<<<......................................................>>>>>>>>>>>>>>>>>>>>>>>>>>>>.......................>.>>>>....................................
          ....1.000.00.000......................................................000.00.000.1...........1..22.......................2.2222....................................
          ----------------∘∘∘∘----------------------------------------------------------------------------∘∘-----------------------●-●●●Y∘--∘--∘-∘GC-∘AACGGA-AGACAGA-AAG-CAUG
                                                                                                                                                                         3′
```

```
Tat-1-1  ................GCAA.............................................................................C.......................U.CGCCG..C..A.AGC.UAACGGA.AGACAGA.AAG.CAUG
env-459  ................GCAA.............................................................................C.......................C.CGCCG..C..AAAGC..AACGGA.AGACAGA.AAG.CAUG
env-460  ................UUUG............................................................................GG.......................A.CUGCA..U..GAAGC.AAACGGA.AGACAGA.AAG.CAUG
env-463  ................UUUG............................................................................GG.......................A.CUGCA..U..GAAGC.AAACGGA.AGACAGA.AAG.CAUG
env-464  ................UUUG............................................................................GG.......................A.CUGCA..CG.A.AGC.AAACGGA.AGACAGA.AAG.CAUG
env-465  ................UUCG............................................................................GG.......................G.UUGCA..CG.A.AGC.AAACGGA.AGACAGA.AAG.CAUG
env-467  ................AUGC............................................................................GG.......................G.CUGCA..CG.A.AGC.AAACGGA.AGACAGA.AAG.CAUG
env-469  ................AUGC............................................................................GG.......................G.CUGCA..CG.A.AGC.AAACGGA.AGACAGA.AAG.CAUG
env-472  ................UCUU............................................................................GG.......................G.CUGCA..CG.A.AGC.AAACGGA.AGACAGA.AAG.CAUG
env-473  ................AAA.............................................................................GG.......................G.CUGCA..CG.A.AGC.AAACGGA.AGACAGA.AAG.CAUG
env-475  ................AUAA............................................................................GG.......................G.CUGCA..CG.A.AGC..AACGGA.AGACAGA.AAG.CAUG
env-476  ................ACAA............................................................................GG.......................G.CUGCA..CG.A.AGC.AAACGGA.AGACAGA.AAG.CAUG
env-477  ................ACAA............................................................................GG.......................G.CUGCA..CG.A.AGC.AAACGGA.AGACAGA.AAG.CAUG
env-482  ................AUAA............................................................................GG.......................G.CUGCG..CG.A.AGC.AAACGGA.AGACAGA.AAG.CAUG
env-483  ................AUAA............................................................................GG.......................G.CUGCA..CG.A.AGC.AAACGGA.AGACAGA.AAG.CAUG
env-484  ................AUAA............................................................................GG.......................G.CUGCA..CG.A.AGC.AAACGGA.AGACAGA.AAG.CAUG
Pte-1-1  ................AUAA............................................................................GG.......................G.CUGCA..CG.A.AGC.AAACGGA.AGACAGA.AAG.CAUG
env-493  ................AUAA............................................................................GG.......................G.CUGCA..CG.A.AGC.AAACGGA.AGACAGA.AAG.CAUG
env-494  ................UUUA............................................................................GG.......................G.CUGCA..CG.A.AGC.AAACGGA.AGACAGA.AAG.CAUG
env-495  ................UUUC............................................................................GG.......................G.CUGCA..CG.A.AGC.AAACGGA.AGACAGA.AAG.CAUG
env-497  GAUGGACGGGGGGCUGAACACA................................................UAGGCCGCCG.CCG....AC.AGGGCAGG......................G.GCCG...C....AGCUAAACGGA.AGACAGA.AAG.CAUG
env-500  ................AAAACAAAUGCUCGAAACAUACGGUCGGGGCA.........................................................................A.CCC.G.ACC.U.UGCUUAACGGA.AGACAGA.AAG.CAUG
env-502  ................GCCCAAACAUA.....................................................................GCCCCUCCAAC..............C.UGGGG..CA.G.AGC.UAACGGA.AGACAGA.AAG.CAUG
env-503  ................UAC....................................................................UGGUCGGGGCA.......................A.CCCGA..UC.U.UGCUUAACGGA.AGACAGA.AAG.CAUG
env-505  ................UAC.....................................................................UGGUCGGGGCA......................A.CCCGA..UC.U.UGCUUAACGGA.AGACAGA.AAG.CAUG
Smu-1-1  ................UCCCGCCGACCACAUAGCGGGCCCCACGG..................................................GGU.......................C.CCGUG..U..C.UGA.AAACGGA.AGACAGA.AAG.CAUG
env-506  ................GCAAA...........................................................................GU.......................G.CUGCA..UUUA.....UAACGGA.AGACAGA.AAG.CAUG
env-507  ................GCAAA...........................................................................GC.......................G.CUGCA..UUUA.....UAACGGA.AGACAGA.AAG.CAUG
env-508  ................GCAAA...........................................................................GC.......................G.CUGCA..UUUA.....UAACGGA.AGACAGA.AAG.CAUG
env-512  ................GUAAA...........................................................................GU.......................G.CUGCA.UCU.A.....UAACGGA.AGACAGA.AAG.CAUG
env-513  ................GUAAA...........................................................................GU.......................G.CUGCA.UCU.A.....UAACGGA.AGACAGA.AAG.CAUG
env-514  ................GUAAA...........................................................................GU.......................G.CUGCA.UCU.A.....UAACGGA.AGACAGA.AAG.CAUG
env-515  ................GUAAA...........................................................................GU.......................G.CUGCA.UCU.A.....UAACGGA.AGACAGA.AAG.CAUG
env-516  ................GUAAA...........................................................................GU.......................G.CUGCA.UCU.A.....UAACGG..AGACAGA.AAG.CAUG
env-517  ................GUAAA...........................................................................GU.......................G.CUGCA..UNUA.....UAACGGA.AGACAGA.AAG.CAUG
env-518  ................GUAAA...........................................................................GU.......................G.CUGCA..UUUA.....UAACGGA.AGACAGA.AAG.CAUG
env-519  ................GUAAA...........................................................................GU.......................G.CUGCA..UUUA.....UAACGGA.AGACAGA.AAG.CAUG
env-520  ................GUAAA...........................................................................GU.......................G.CUGCA..UUUA.....UAACGGA.AGACAGA.AAG.CAUG
env-521  ................GUAAA...........................................................................GU.......................G.CUGCA..UUUA.....UAACGGA.AGACAGA.AAG.CAUG
env-522  ................CGCCCAACAUAGCGGGUCGUCAAA.......................................................GAC.......................C.UG.CA..CU...UGC.UAACGGA.AGACAGA.AAG.CAUG
env-523  ................CGCCCAACAUAGCGGGUCAUCUAA.......................................................GAC.......................C.UGCA...CU...UGC.UAACGGA.AGACAGA.AAG.CAUG
env-524  ..................................................................................................................................C........UAACGGA.AGACAGA.AAG.CAUG
env-525  ..................................................................................................................................UU.C......AACGGA.AGACAGA.AAG.CAUG
env-526  ................UCCG............................................................................GG.......................G.CCGCA..C..GAAGC.AAACGGA.AGACAGA.AAG.CAUG
Tda-1-1  ................GCAA.............................................................................C.......................U.CGCCG..C..AAAGC..AACGGA.AGACAGA.AAG.CAUG
Dsp-1-1  ................CCGUUCCGAAACGGAC.................................................................C.......................C.CGCCG..C..A.AGC.AGACGGA.AGACAGG.AAG.CAUG
env-527  .........................................................................................................................G.GUCC......AAAGC.AAACGGA.AGACAGA.AAG.CAUG
Jaq-1-1  ................GCGA.............................................................................C.......................C.CGUCUGACA.U.AGC.AAACGGA.AGACAGCUGA..CAUG
Sge-1-1  ................AACA...........................................................................UCC.......................C.UGCCG..CA.C.AGCUAAACGGA.AGACAGA.CAA.CAUG
env-528  ................ACA...........................................................................UGGG.......................U.CUGAG..CU.A.....AAACGGA.AGACAGA.AAG.AAUG
env-530  ................AAAU...........................................................................UGG.......................U.CUGA.........GCUAAACGGA.AGACAGA.AAG.AAUG
env-531  ................AAAU...........................................................................UGG.......................U.CUGAG..CU.A.....AAACGGA.AGACAGA.AAG.AAUG
env-533  ................AAAU...........................................................................UGG.......................U.CUGAG.ACU.A.....AAACGGA.AGACAGA.AAG.AAUG
env-534  ................AAAU...........................................................................UGG.......................U.CUGAG..CU.A.....AAACGGA.AGACAGA.AAG.AAUG
env-536  ................AAAU...........................................................................UGG.......................U.CUGAG..CU.A.....AAACGGA.AGACAGA.AAG.AAUG
env-546  ................AAAU...........................................................................UGG.......................U.CUGAG..CU.A.....AAACGG..AGACAGA.AAG.AAUG
env-548  ................AAAU...........................................................................UGG.......................U.CUGAG..CU.A.....AAACGGA.AGACAGA.AAG.AAUG
env-549  ................AAAU...........................................................................UGG.......................U.CUGAG..CU.A.....AAACGGA.AGACAGA.AAG.AAUG
env-567  ................UAAU...........................................................................UGG.......................U.CUGAG..CU.A.....AAACGGA.AGACAGA.AAG.AAUG
env-576  ................UAAU...........................................................................UGG.......................U.CUGAG..CU.A.....AAACGGA.AGACAGA.AAG.AAUG
env-579  ................UAAU...........................................................................UGG.......................U.CUGAG..CU.A.....AAACGGA.AGACAGA.AAG.AAUG
env-582  ................UAAU...........................................................................UGG.......................U.CUGAG..CU.A.....AAACGGA.AGACAGA.AAG.AAUG
env-583  ................UAAU...........................................................................UGG.......................U.CUGAG..CU.A.....AAACGGA.AGACAGA.AAG.AAUG
Sgu-1-1  ................UACG...........................................................................GGG.......................C.CGCAG.AC..A.AGCAAAACGGA.AGACAGA.GAA.CAUG
env-590  ................AUCACCAC......................................................................GGGG.......................C.CGGGG.AC..A.AGCAAAACGGA.AGACAGA.GAA.CAUG
env-591  ................AUCACGAC......................................................................GGCG.......................U.CGGGG.AC..A.AGCAAAACGGA.AGACAGA.GAA.CAUG
env-592  ................AGCACUAC......................................................................GGGG.......................U.CGGGG.AC..A.AGCAAAACGGA.AGACAGA.GAA.CAUG
env-595  ................AUCACGAC......................................................................GGGG.......................C.CGGGG.AC..A.AGCAAAACGGA.AGACAGA.GAA.CAUG
env-596  ................AUCACGAC......................................................................GGGG.......................C.CGGGG.AC..A.AGCAAAACGGA.AGACAGA.GAA.CAUG
env-597  ................AUCACGAC......................................................................GGGG.......................C.CGGGG.AC..A.AGCAAAACGGA.AGACAGA.GAA.CAUG
Ssp-4-1  ................AUCACGAC......................................................................GGGG.......................C.CGGGG.AC..A.AGCAAAACGGA.AGACAGA.GAA.CAUG
env-598  ................AUCACGAC......................................................................GGGG.......................C.CGGGG.AC..A.AGCAAAACGGA.AGACAGA.GAA.CAUG
Ssp-5-1  ................AUCACGAC......................................................................GGGG.......................C.CGGGG.AC..A.AGCAAAACGGA.AGACAGA.GAA.CAUG
env-600  ................AUCACGAC......................................................................GGGG.......................C.CGGGG.AC..A.AGCAAAACGGA.AGACAGA.GAA.CAUG
env-605  ................AGCACGAC......................................................................GGGG.......................C.CGGGG.AC..A.AGCAAAACGGA.AGACAGA.GAA.CAUG
env-606  ................AGCACGAC......................................................................GGGG.......................C.CGGGG.AC..A.AGCAAAACGGA.AGACAGA.GAA.CAUG
env-611  .........................................................................................................................U.UGGUC..CA.G.AGC.ACACGGA.AGACAGA.AAG.CAUG
env-612  .........................................................................................................................U.UGGUC..CA.G.AGC.ACACGGA.AGACAGA.AAG.CAUG
env-613  ................GAUGU...........................................................................GA.......................G.CGUG...CC.U.AGC.AAACGGA.AGACAGA.GAA.CAUG
env-614  ................GAUG...........................................................................GAG.......................G.CGUGC..C..A.AGC.AAACGGA.AGACAGA.GAA.CAUG
env-615  ................GGUG...........................................................................GAG.......................G.CGUG...C..CAAGC.AAACGGA.AGACAGA.GAA.CAUG
env-616  ................GAU...........................................................................GGAU.......................G.CGUG...C..CAAGC.AAACGGA.AGACAGA.GAA.CAUG
env-617  ................CGAU..........................................................................GGAU.......................G.CGUG...CC.A.AGC.AAACGGA.AGACAGA.GAA.CAUG

         <<<<<<<<<<<<<<<<......................................................>>>>>>>>>>>>>>>>>>>>>>>>>>>>.......................>.>>>>....................................
         ....1.000.00.000......................................................000.00.000.1...........1..22.......................2.2222....................................
         ----------------∘∘∘∘----------------------------------------------------------------------------∘∘-----------------------•-•••Y∘--∘--∘-∘GC-∘AACGGA-AGACAGA-AAG-CAUG
                                                                                                                                                                     3'
```

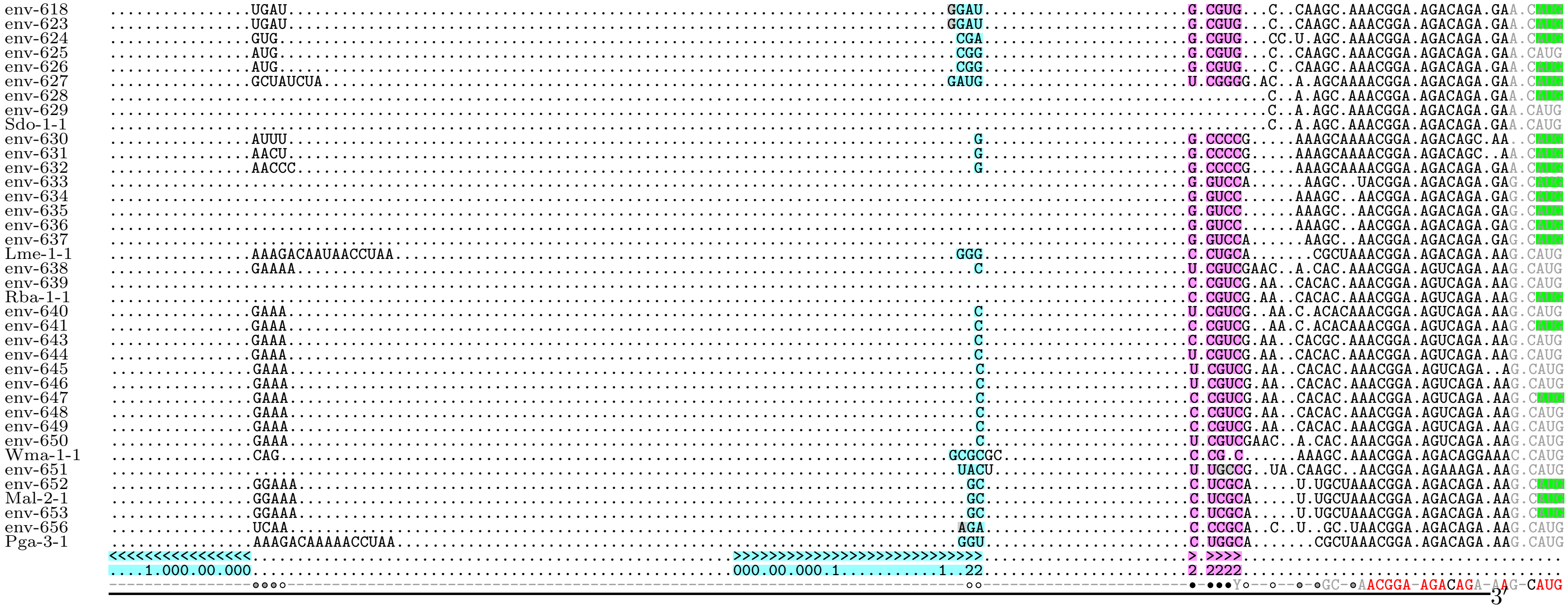

2 L4-Archaeoglobi

2.1 Taxa

The taxonomy of each organism containing a putative L4-Archaeoglobi RNA is listed, with abbreviations identifying each hit (e.g., “Eco-1-1” and “Eco-1-2” might hypothetically represent two distinct RNAs in *E. coli*). The abbreviations will be used to identify each individual L4-Archaeoglobi RNA in Sections 2.2 and 2.4:.

**abbrev. of hits**	**taxonomy of species**
har-1-1	halophilic archaeon DL31 Archaea
h3A-1-1	Archaea Euryarchaeota Halobacteria Halobacteriales haloarchaeon 3A1_DGR
Hsa-1-1	Archaea Euryarchaeota Halobacteria Halobacteriales Haloarculaceae *Halapricum salinum*
Hsp-1-1	Archaea Euryarchaeota Halobacteria Halobacteriales Haloarculaceae *Haloarcula sp.* CBA1115
CHa-1-1	Archaea Euryarchaeota Halobacteria Halobacteriales Halobacteriaceae *Candidatus* Halobonum tyrrellensis G22
Hci-1-1	Archaea Euryarchaeota Halobacteria Halobacteriales Halobacteriaceae *Haladaptatus cibarius* D43
Hpa-1-1 to Hpa-1-2	Archaea Euryarchaeota Halobacteria Halobacteriales Halobacteriaceae *Haladaptatus paucihalophilus* DX253
Hje-1-1	Archaea Euryarchaeota Halobacteria Halobacteriales Halobacteriaceae *Halalkalicoccus jeotgali* B3
Hsu-1-1	Archaea Euryarchaeota Halobacteria Halobacteriales Halobacteriaceae *Halanaeroarchaeum sulfurireducens*
Hac-1-1 to Hac-1-2	Archaea Euryarchaeota Halobacteria Halobacteriales Halobacteriaceae *Halarchaeum acidiphilum* MH1-52-1
Ham-1-1	Archaea Euryarchaeota Halobacteria Halobacteriales Halobacteriaceae *Haloarcula amylolytica* JCM 13557
Har-1-1	Archaea Euryarchaeota Halobacteria Halobacteriales Halobacteriaceae *Haloarcula argentinensis* DSM 12282
Hca-1-1	Archaea Euryarchaeota Halobacteria Halobacteriales Halobacteriaceae *Haloarcula californiae* ATCC 33799
Hhi-1-1	Archaea Euryarchaeota Halobacteria Halobacteriales Halobacteriaceae *Haloarcula hispanica* ATCC 33960
Hhi-2-1	Archaea Euryarchaeota Halobacteria Halobacteriales Halobacteriaceae *Haloarcula hispanica* N601
Hja-1-1	Archaea Euryarchaeota Halobacteria Halobacteriales Halobacteriaceae *Haloarcula japonica* DSM 6131

Hma-1-1	Archaea Euryarchaeota Halobacteria Halobacteriales Halobacteriaceae *Haloarcula marismortui* ATCC 43049
Hsa-2-1	Archaea Euryarchaeota Halobacteria Halobacteriales Halobacteriaceae *Haloarcula salaria*
Hsi-1-1	Archaea Euryarchaeota Halobacteria Halobacteriales Halobacteriaceae *Haloarcula sinaiiensis* ATCC 33800
Hva-1-1	Archaea Euryarchaeota Halobacteria Halobacteriales Halobacteriaceae *Haloarcula vallismortis* ATCC 29715
Hsa-3-1	Archaea Euryarchaeota Halobacteria Halobacteriales Halobacteriaceae *Halobacterium salinarum* R1
Hsp-2-1	Archaea Euryarchaeota Halobacteria Halobacteriales Halobacteriaceae *Halobacterium sp.* DL1
Hsp-3-1	Archaea Euryarchaeota Halobacteria Halobacteriales Halobacteriaceae *Halobacterium sp.* NRC-1
Hla-1-1 to Hla-1-2	Archaea Euryarchaeota Halobacteria Halobacteriales Halobacteriaceae *Halobiforma lacisalsi* AJ5
Hni-1-1	Archaea Euryarchaeota Halobacteria Halobacteriales Halobacteriaceae *Halobiforma nitratireducens* JCM 10879
Hha-1-1 to Hha-1-2	Archaea Euryarchaeota Halobacteria Halobacteriales Halobacteriaceae *Halococcus hamelinensis* 100A6
Hmo-1-1	Archaea Euryarchaeota Halobacteria Halobacteriales Halobacteriaceae *Halococcus morrhuae* DSM 1307
Hsa-4-1	Archaea Euryarchaeota Halobacteria Halobacteriales Halobacteriaceae *Halococcus saccharolyticus* DSM 5350
Hsa-5-1	Archaea Euryarchaeota Halobacteria Halobacteriales Halobacteriaceae *Halococcus salifodinae* DSM 8989
Hth-1-1	Archaea Euryarchaeota Halobacteria Halobacteriales Halobacteriaceae *Halococcus thailandensis* JCM 13552
Hal-1-1	Archaea Euryarchaeota Halobacteria Halobacteriales Halobacteriaceae *Haloferax alexandrinus* JCM 10717
Hde-1-1	Archaea Euryarchaeota Halobacteria Halobacteriales Halobacteriaceae *Haloferax denitrificans* ATCC 35960
Hel-1-1	Archaea Euryarchaeota Halobacteria Halobacteriales Halobacteriaceae *Haloferax elongans* ATCC BAA-1513
Hgi-1-1	Archaea Euryarchaeota Halobacteria Halobacteriales Halobacteriaceae *Haloferax gibbonsii* ATCC 33959
Hla-2-1	Archaea Euryarchaeota Halobacteria Halobacteriales Halobacteriaceae *Haloferax larsenii* JCM 13917
Hlu-1-1	Archaea Euryarchaeota Halobacteria Halobacteriales Halobacteriaceae *Haloferax lucentense* DSM 14919
Hme-1-1 to Hme-1-2	Archaea Euryarchaeota Halobacteria Halobacteriales Halobacteriaceae *Haloferax mediterranei* ATCC 33500
Hmu-1-1	Archaea Euryarchaeota Halobacteria Halobacteriales Halobacteriaceae *Haloferax mucosum* ATCC BAA-1512
Hpr-1-1	Archaea Euryarchaeota Halobacteria Halobacteriales Halobacteriaceae *Haloferax prahovense* DSM 18310
Hsp-4-1	Archaea Euryarchaeota Halobacteria Halobacteriales Halobacteriaceae *Haloferax sp.* ATCC BAA-644
Hsp-5-1	Archaea Euryarchaeota Halobacteria Halobacteriales Halobacteriaceae *Haloferax sp.* ATCC BAA-645
Hsp-6-1	Archaea Euryarchaeota Halobacteria Halobacteriales Halobacteriaceae *Haloferax sp.* ATCC BAA-646
Hsu-2-1	Archaea Euryarchaeota Halobacteria Halobacteriales Halobacteriaceae *Haloferax sulfurifontis* ATCC BAA-897
Hvo-1-1	Archaea Euryarchaeota Halobacteria Halobacteriales Halobacteriaceae *Haloferax volcanii* DS2
Hbo-1-1	Archaea Euryarchaeota Halobacteria Halobacteriales Halobacteriaceae *Halogeometricum borinquense* DSM 11551
Hsa-6-1	Archaea Euryarchaeota Halobacteria Halobacteriales Halobacteriaceae *Halogranum salarium* B-1
Hka-1-1	Archaea Euryarchaeota Halobacteria Halobacteriales Halobacteriaceae *Halomicrobium katesii* DSM 19301
Hmu-2-1	Archaea Euryarchaeota Halobacteria Halobacteriales Halobacteriaceae *Halomicrobium mukohataei* DSM 12286
Hxa-1-1	Archaea Euryarchaeota Halobacteria Halobacteriales Halobacteriaceae *Halopiger xanaduensis* SH-6
Hwa-1-1	Archaea Euryarchaeota Halobacteria Halobacteriales Halobacteriaceae *Haloquadratum walsbyi* C23
Hwa-2-1	Archaea Euryarchaeota Halobacteria Halobacteriales Halobacteriaceae *Haloquadratum walsbyi* DSM 16790
Hti-1-1	Archaea Euryarchaeota Halobacteria Halobacteriales Halobacteriaceae *Halorhabdus tiamatea* SARL4B
Hut-1-1	Archaea Euryarchaeota Halobacteria Halobacteriales Halobacteriaceae *Halorhabdus utahensis* DSM 12940
Hai-1-1	Archaea Euryarchaeota Halobacteria Halobacteriales Halobacteriaceae *Halorubrum aidingense* JCM 13560
Har-2-1	Archaea Euryarchaeota Halobacteria Halobacteriales Halobacteriaceae *Halorubrum arcis* JCM 13916
Hca-2-1	Archaea Euryarchaeota Halobacteria Halobacteriales Halobacteriaceae *Halorubrum californiensis* DSM 19288
Hco-1-1	Archaea Euryarchaeota Halobacteria Halobacteriales Halobacteriaceae *Halorubrum coriense* DSM 10284
Hdi-1-1	Archaea Euryarchaeota Halobacteria Halobacteriales Halobacteriaceae *Halorubrum distributum* JCM 10118
Hdi-2-1	Archaea Euryarchaeota Halobacteria Halobacteriales Halobacteriaceae *Halorubrum distributum* JCM 9100
Hho-1-1	Archaea Euryarchaeota Halobacteria Halobacteriales Halobacteriaceae *Halorubrum hochstenium* ATCC 700873
Hko-1-1	Archaea Euryarchaeota Halobacteria Halobacteriales Halobacteriaceae *Halorubrum kocurii* JCM 14978
Hla-3-1	Archaea Euryarchaeota Halobacteria Halobacteriales Halobacteriaceae *Halorubrum lacusprofundi* ATCC 49239
Hli-1-1	Archaea Euryarchaeota Halobacteria Halobacteriales Halobacteriaceae *Halorubrum lipolyticum* DSM 21995
Hli-2-1	Archaea Euryarchaeota Halobacteria Halobacteriales Halobacteriaceae *Halorubrum litoreum* JCM 13561
Hsa-7-1	Archaea Euryarchaeota Halobacteria Halobacteriales Halobacteriaceae *Halorubrum saccharovorum* DSM 1137
Hsp-7-1	Archaea Euryarchaeota Halobacteria Halobacteriales Halobacteriaceae *Halorubrum sp.* T3
Hte-1-1	Archaea Euryarchaeota Halobacteria Halobacteriales Halobacteriaceae *Halorubrum tebenquichense* DSM 14210
Hte-2-1	Archaea Euryarchaeota Halobacteria Halobacteriales Halobacteriaceae *Halorubrum terrestre* JCM 10247
Hpa-2-1	Archaea Euryarchaeota Halobacteria Halobacteriales Halobacteriaceae *Halosarcina pallida* JCM 14848
Hca-3-1	Archaea Euryarchaeota Halobacteria Halobacteriales Halobacteriaceae *Halosimplex carlsbadense* 2-9-1
Hli-3-1	Archaea Euryarchaeota Halobacteria Halobacteriales Halobacteriaceae *Haloterrigena limicola* JCM 13563
Hsa-8-1	Archaea Euryarchaeota Halobacteria Halobacteriales Halobacteriaceae *Haloterrigena salina* JCM 13891
Hth-2-1	Archaea Euryarchaeota Halobacteria Halobacteriales Halobacteriaceae *Haloterrigena thermotolerans* DSM 11522
Htu-1-1	Archaea Euryarchaeota Halobacteria Halobacteriales Halobacteriaceae *Haloterrigena turkmenica* DSM 5511
Has-1-1	Archaea Euryarchaeota Halobacteria Halobacteriales Halobacteriaceae *Halovivax asiaticus* JCM 14624
Nae-1-1	Archaea Euryarchaeota Halobacteria Halobacteriales Halobacteriaceae *Natrialba aegyptia* DSM 13077
Nas-1-1	Archaea Euryarchaeota Halobacteria Halobacteriales Halobacteriaceae *Natrialba asiatica* DSM 12278
Nch-1-1	Archaea Euryarchaeota Halobacteria Halobacteriales Halobacteriaceae *Natrialba chahannaoensis* JCM 10990
Nhu-1-1	Archaea Euryarchaeota Halobacteria Halobacteriales Halobacteriaceae *Natrialba hulunbeirensis* JCM 10989
Nma-1-1	Archaea Euryarchaeota Halobacteria Halobacteriales Halobacteriaceae *Natrialba magadii* ATCC 43099
Nta-1-1	Archaea Euryarchaeota Halobacteria Halobacteriales Halobacteriaceae *Natrialba taiwanensis* DSM 12281

Nal-1-1	Archaea Euryarchaeota Halobacteria Halobacteriales Halobacteriaceae *Natrinema altunense* JCM 12890
Nga-1-1	Archaea Euryarchaeota Halobacteria Halobacteriales Halobacteriaceae *Natrinema gari* JCM 14663
Npa-1-1	Archaea Euryarchaeota Halobacteria Halobacteriales Halobacteriaceae *Natrinema pallidum* DSM 3751
Npe-1-1	Archaea Euryarchaeota Halobacteria Halobacteriales Halobacteriaceae *Natrinema pellirubrum* DSM 15624
Nsp-1-1	Archaea Euryarchaeota Halobacteria Halobacteriales Halobacteriaceae *Natrinema sp.* J7-2
Nve-1-1	Archaea Euryarchaeota Halobacteria Halobacteriales Halobacteriaceae *Natrinema versiforme* JCM 10478
Ngr-1-1	Archaea Euryarchaeota Halobacteria Halobacteriales Halobacteriaceae *Natronobacterium gregoryi* SP2
Nam-1-1	Archaea Euryarchaeota Halobacteria Halobacteriales Halobacteriaceae *Natronococcus amylolyticus* DSM 10524
Nje-1-1	Archaea Euryarchaeota Halobacteria Halobacteriales Halobacteriaceae *Natronococcus jeotgali* DSM 18795
Noc-1-1	Archaea Euryarchaeota Halobacteria Halobacteriales Halobacteriaceae *Natronococcus occultus* SP4
Nin-1-1	Archaea Euryarchaeota Halobacteria Halobacteriales Halobacteriaceae *Natronolimnobius innermongolicus* JCM 12255
Nmo-1-1	Archaea Euryarchaeota Halobacteria Halobacteriales Halobacteriaceae *Natronomonas moolapensis* 8.8.11
Nph-1-1	Archaea Euryarchaeota Halobacteria Halobacteriales Halobacteriaceae *Natronomonas pharaonis* DSM 2160
Nba-1-1	Archaea Euryarchaeota Halobacteria Halobacteriales Halobacteriaceae *Natronorubrum bangense* JCM 10635
Nsu-1-1	Archaea Euryarchaeota Halobacteria Halobacteriales Halobacteriaceae *Natronorubrum sulfidifaciens* JCM 14089
Nti-1-1	Archaea Euryarchaeota Halobacteria Halobacteriales Halobacteriaceae *Natronorubrum tibetense* GA33
Ssp-1-1	Archaea Euryarchaeota Halobacteria Halobacteriales Halobacteriaceae *Salinarchaeum sp.* Harcht-Bsk1
Hag-1-1	Archaea Euryarchaeota Halobacteria Halobacteriales Halococcaceae *Halococcus agarilyticus*
Hse-1-1	Archaea Euryarchaeota Halobacteria Halobacteriales Halococcaceae *Halococcus sediminicola*
Hru-1-1	Archaea Euryarchaeota Halobacteria Haloferacales Haloferacaceae *Halobellus rufus*
Hal-2-1	Archaea Euryarchaeota Halobacteria Haloferacales Haloferacaceae *Haloferax alexandrinus*
Hgi-2-1	Archaea Euryarchaeota Halobacteria Haloferacales Haloferacaceae *Haloferax gibbonsii*
Hme-1-1 to Hme-1-2	Archaea Euryarchaeota Halobacteria Haloferacales Haloferacaceae *Haloferax mediterranei* ATCC 33500
Hsp-8-1	Archaea Euryarchaeota Halobacteria Haloferacales Haloferacaceae *Haloferax sp.* ATB1
Hna-1-1	Archaea Euryarchaeota Halobacteria Haloferacales Haloferacaceae *Haloplanus natans* DSM 17983
Hru-2-1	Archaea Euryarchaeota Halobacteria Haloferacales Halorubraceae *Halolamina rubra*
Hez-1-1	Archaea Euryarchaeota Halobacteria Haloferacales Halorubraceae *Halorubrum ezzemoulense* DSM 17463
Hha-2-1	Archaea Euryarchaeota Halobacteria Haloferacales Halorubraceae *Halorubrum halophilum*
Hsa-9-1	Archaea Euryarchaeota Halobacteria Haloferacales Halorubraceae *Halorubrum saccharovorum*
Hsp-9-1	Archaea Euryarchaeota Halobacteria Haloferacales Halorubraceae *Halorubrum sp.* AJ67
Hsp-10-1	Archaea Euryarchaeota Halobacteria Haloferacales Halorubraceae *Halorubrum sp.* BV1
Hdj-1-1	Archaea Euryarchaeota Halobacteria Natrialbales Natrialbaceae *Halopiger djelfimassiliensis*
Hgo-1-1	Archaea Euryarchaeota Halobacteria Natrialbales Natrialbaceae *Halopiger goleimassiliensis*
Hsa-10-1	Archaea Euryarchaeota Halobacteria Natrialbales Natrialbaceae *Halopiger salifodinae*
Hla-4-1	Archaea Euryarchaeota Halobacteria Natrialbales Natrialbaceae *Halostagnicola larsenii* XH-48
Hsp-11-1	Archaea Euryarchaeota Halobacteria Natrialbales Natrialbaceae *Halostagnicola sp.* A56
Hje-2-1	Archaea Euryarchaeota Halobacteria Natrialbales Natrialbaceae *Haloterrigena jeotgali* A29
Nal-2-1	Archaea Euryarchaeota Halobacteria Natrialbales Natrialbaceae *Natrinema altunense*
Nsp-2-1	Archaea Euryarchaeota Halobacteria Natrialbales Natrialbaceae *Natrinema sp.* J7-1
Mco-1-1	Archaea Euryarchaeota Methanomicrobia Methanocellales Methanocellaceae *Methanocella conradii* HZ254
CMe-1-1	Archaea Euryarchaeota Methanomicrobia Methanomicrobiales Generaincertae sedis *Candidatus* Methanoregula boonei 6A8
Mpa-1-1	Archaea Euryarchaeota Methanomicrobia Methanomicrobiales Generaincertae sedis *Methanosphaerula palustris* E1-9c
Mbo-1-1	Archaea Euryarchaeota Methanomicrobia Methanomicrobiales Methanomicrobiaceae *Methanoculleus bourgensis* MS2
Mma-1-1	Archaea Euryarchaeota Methanomicrobia Methanomicrobiales Methanomicrobiaceae *Methanoculleus marisnigri* JR1
Mse-1-1	Archaea Euryarchaeota Methanomicrobia Methanomicrobiales Methanomicrobiaceae *Methanoculleus sediminis*
Msp-1-1	Archaea Euryarchaeota Methanomicrobia Methanomicrobiales Methanomicrobiaceae *Methanoculleus sp.* MH98A
Mli-1-1	Archaea Euryarchaeota Methanomicrobia Methanomicrobiales Methanomicrobiaceae *Methanofollis liminatans* DSM 4140
Mpa-2-1	Archaea Euryarchaeota Methanomicrobia Methanomicrobiales Methanomicrobiaceae *Methanolacinia paynteri*
Mmo-1-1	Archaea Euryarchaeota Methanomicrobia Methanomicrobiales Methanomicrobiaceae *Methanomicrobium mobile* BP
Mli-2-1	Archaea Euryarchaeota Methanomicrobia Methanomicrobiales Methanomicrobiaceae *Methanoplanus limicola* DSM 2279
Mpe-1-1	Archaea Euryarchaeota Methanomicrobia Methanomicrobiales Methanomicrobiaceae *Methanoplanus petrolearius* DSM 11571
Mta-1-1	Archaea Euryarchaeota Methanomicrobia Methanomicrobiales Methanoregulaceae *Methanolinea tarda* NOBI-1
Mfo-1-1	Archaea Euryarchaeota Methanomicrobia Methanomicrobiales Methanoregulaceae *Methanoregula formicica* SMSP
Mhu-1-1	Archaea Euryarchaeota Methanomicrobia Methanomicrobiales Methanospirillaceae *Methanospirillum hungatei* JF-1
Mco-2-1	Archaea Euryarchaeota Methanomicrobia Methanosarcinales Methanosaetaceae *Methanosaeta concilii* GP6
Mha-1-1	Archaea Euryarchaeota Methanomicrobia Methanosarcinales Methanosaetaceae *Methanosaeta harundinacea* 6Ac
Msh-1-1	Archaea Euryarchaeota Methanomicrobia Methanosarcinales Methermicoccaceae *Methermicoccus shengliensis* DSM 18856
env-1 to env-513	environmental samples

2.2 Gene contexts

Each L4-Archaeoglobi RNA (indicated by "RNA→") is listed. For each hit, the downstream genes predicted to reside in a regulated operon are listed. If the nearest downstream gene is encoding in the opposite strand (and therefore presumed to not be a part of a regulated operon), then that gene is still depicted. Some environmental sequences and some RefSeq entries lack gene annotations, and so no genes are available for such sequences. The direction of each gene is indicated with an arrow (→), and each predicted conserved domain in the gene is named. Conserved domains associated with more than one L4-Archaeoglobi RNA are assigned a color; other domains are gray. Information about these conserved domains is given in Section 2.3. The accession of the sequence containing each L4-Archaeoglobi RNA is given in the column named "Seq. accession". Accessions beginning with "NC_", "NS_", "NW_" or "NZ_" are contained in RefSeq. Other accession refer to environmental samples. Nucleotide coordinates are given for the 5′ and 3′ boundaries of each L4-Archaeoglobi RNA. If the 5′ coordinate is greater than the 3′ coordinate, the RNA is present on the reverse-complement strand of the containing genomic DNA sequence. Each hit is denoted by an abbreviation (like "Eco-1-1") that refers to a taxonomy given in Section 2.1.

abbrev.	Seq. accession		5′ at	3′ at	genes
Mli-1-1	NZ_CM001555.1	+	465849	465921	**RNA**→ RplC (COG0087)rpl3p (PRK04231)→ RplC (COG0087)rpl3p (PRK04231)→<br>RplD (COG0088)rpl4lp (PRK04042)→ RplD (COG0088)rpl4lp (PRK04042)→<br>RplW (COG0089)PRK14548 (PRK14548)→ RplW (COG0089)PRK14548 (PRK14548)→<br>RplB (COG0090)rpl2p (PRK09612)→ RplB (COG0090)rpl2p (PRK09612)→<br>RpsS (COG0185)rps19p (PRK04038)→ RpsS (COG0185)rps19p (PRK04038)→<br>rpl22p (PRK04223)RplV (COG0091)→ rpl22p (PRK04223)RplV (COG0091)→<br>RpsC (COG0092)rps3p (PRK04191)→ RpsC (COG0092)rps3p (PRK04191)→<br>PRK14549 (PRK14549)Ribosomal_L29 (pfam00831)→ PRK14549 (PRK14549)Ribosomal_L29 (pfam00831)→<br>PRK03879 (PRK03879)UPF0086 (pfam01868)→ PRK03879 (PRK03879)UPF0086 (pfam01868)→<br>RpsQ (COG0186)rps17p (PRK08572)→ RpsQ (COG0186)rps17p (PRK08572)→<br>rpl14p (PRK08571)Ribosomal_L14 (pfam00238)→ rpl14p (PRK08571)Ribosomal_L14 (pfam00238)→<br>rpl24p (PRK01191)KOW_RPL26 (cd06089)→ rpl24p (PRK01191)KOW_RPL26 (cd06089)→<br>PRK04313 (PRK04313)S4 (cd00165)KOW_RPS4 (cd06087)Ribosomal_S4e (pfam00900)→<br>PRK04313 (PRK04313)S4 (cd00165)KOW_RPS4 (cd06087)Ribosomal_S4e (pfam00900)→<br>RplE (COG0094)rpl5p (PRK04219)→ RplE (COG0094)rpl5p (PRK04219)→<br>RpsN (COG0199)rps14P (PRK05766)→ RpsN (COG0199)rps14P (PRK05766)→<br>rps8p (PRK04034)RpsH (COG0096)→ rps8p (PRK04034)RpsH (COG0096)→<br>RplF (COG0097)rpl6p (PRK05518)→ RplF (COG0097)rpl6p (PRK05518)→<br>rpl32e (PRK08562)Ribosomal_L32e (pfam01655)→ rpl32e (PRK08562)Ribosomal_L32e (pfam01655)→<br>rpl19e (PRK08570)Ribosomal_L19e (pfam01280)→ rpl19e (PRK08570)Ribosomal_L19e (pfam01280)→<br>RplR (COG0256)rpl18p (PRK08569)→ RplR (COG0256)rpl18p (PRK08569)→<br>RpsE (COG0098)rps5p (PRK04044)→ RpsE (COG0098)rps5p (PRK04044)→
env-1	SakLake120mDRAFT_c0000096	+	22741	22827	**RNA**→ RplC (COG0087)rpl3p (PRK04231)→ RplD (COG0088)rpl4lp (PRK04042)→<br>uL23_arch (TIGR03636)Ribosomal_L23 (pfam00276)→ RplB (COG0090)rpl2p (PRK09612)→<br>RpsS (COG0185)rps19p (PRK04038)→ rpl22p (PRK04223)RplV (COG0091)→<br>RpsC (COG0092)rps3p (PRK04191)Spore_IV_A (pfam09547)→<br>PRK14549 (PRK14549)Ribosomal_L29 (pfam00831)→ PRK03879 (PRK03879)UPF0086 (pfam01868)→<br>RpsQ (COG0186)rps17p (PRK08572)→ rpl14p (PRK08571)Ribosomal_L14 (pfam00238)→<br>rpl24p (PRK01191)KOW_RPL26 (cd06089)→<br>PRK04313 (PRK04313)KOW_RPS4 (cd06087)Ribosomal_S4e (pfam00900)→ RplE (COG0094)rpl5p (PRK04219)→<br>RpsN (COG0199)rps14P (PRK05766)→ rps8p (PRK04034)RpsH (COG0096)→<br>RplF (COG0097)rpl6p (PRK05518)→ rpl32e (PRK08562)Ribosomal_L32e (pfam01655)→<br>rpl19e (PRK08570)Ribosomal_L19e (pfam01280)→ RplR (COG0256)rpl18p (PRK08569)→<br>RpsE (COG0098)rps5p (PRK04044)→
env-2	SL_8KL_010_SED_10004849	-	1682	1589	**RNA**→ Methyltrn_RNA_3 (pfam02598)→ RplC (COG0087)rpl3p (PRK04231)→<br>RplD (COG0088)rpl4lp (PRK04042)→
env-3	SL_8KL_010_SED_10000147	+	55887	55980	**RNA**→ MTH1 (COG2106)→ RplC (COG0087)rpl3p (PRK04231)→
env-4	JGI24732J26686_1113329	+	312	377	**RNA**→
env-5	JGI24731J21663_1001690	+	821	886	**RNA**→ hypo→
env-6	JGI24729J20445_1012742	-	868	807	**RNA**→ hypo→ rpl3p (PRK04231)Ribosomal_L3 (pfam00297)→
env-7	JGI24730J26740_1315709	+	120	181	**RNA**→ ←hypo

env-8	2014733311	-	1671	1608	**RNA**→ RplC (COG0087)rpl3p (PRK04231)→ RplD (COG0088)rpl4lp (PRK04042)→
env-9	ANASMECb_4085297_C1539	-	1671	1608	**RNA**→ RplC (COG0087)rpl3p (PRK04231)→ RplD (COG0088)rpl4lp (PRK04042)→
env-10	PCEOT_contig04337	+	574	637	**RNA**→ rpl3p (PRK04231)Ribosomal_L3 (pfam00297)→ RplC (COG0087)rpl3p (PRK04231)→<br>RplD (COG0088)rpl4lp (PRK04042)→ hypo→
env-11	JGI12104J13512_1001493	+	1210	1273	**RNA**→ RplC (COG0087)rpl3p (PRK04231)→ RplD (COG0088)rpl4lp (PRK04042)→<br>RplW (COG0089)PRK14548 (PRK14548)→ RplB (COG0090)rpl2p (PRK09612)→<br>RpsS (COG0185)rps19p (PRK04038)→ rpl22p (PRK04223)RplV (COG0091)→<br>RpsC (COG0092)rps3p (PRK04191)→ PRK14549 (PRK14549)Ribosomal_L29_HIP (cd00427)→<br>PRK03879 (PRK03879)UPF0086 (pfam01868)→ RpsQ (COG0186)rps17p (PRK08572)→<br>rpl14p (PRK08571)Ribosomal_L14 (pfam00238)→ rpl24p (PRK01191)KOW_RPL26 (cd06089)→<br>PRK04313 (PRK04313)KOW_RPS4 (cd06087)Ribosomal_S4e (pfam00900)→<br>rpl5p (PRK04219)Ribosomal_L5 (pfam00281)Ribosomal_L5_C (pfam00673)→<br>RpsN (COG0199)rps14P (PRK05766)→ rps8p (PRK04034)RpsH (COG0096)→<br>RplF (COG0097)rpl6p (PRK05518)→ rpl32e (PRK08562)Ribosomal_L32_L32e (cd00513)→<br>rpl19e (PRK08570)Ribosomal_L19e (pfam01280)→ RplR (COG0256)rpl18p (PRK08569)→<br>RpsE (COG0098)rps5p (PRK04044)→ Ribosomal_L7_archeal_euk (cd01657)rpl30p (PRK06049)→<br>rpl15p (PRK06419)Ribosomal_L27A (pfam00828)→ SecY (COG0201)PRK08568 (PRK08568)→<br>AdkA (COG2019)PRK04040 (PRK04040)→ COG1422 (COG1422)→ DUF106 (pfam01956)→<br>CmkB (COG1102)PRK04182 (PRK04182)→
env-12	JGI11944J13513_1000575	-	22491	22428	**RNA**→ RplC (COG0087)rpl3p (PRK04231)→ RplD (COG0088)rpl4lp (PRK04042)→<br>RplW (COG0089)PRK14548 (PRK14548)→ RplB (COG0090)rpl2p (PRK09612)→<br>RpsS (COG0185)rps19p (PRK04038)→ rpl22p (PRK04223)RplV (COG0091)→<br>RpsC (COG0092)rps3p (PRK04191)→ PRK14549 (PRK14549)Ribosomal_L29_HIP (cd00427)→<br>PRK03879 (PRK03879)UPF0086 (pfam01868)→ RpsQ (COG0186)rps17p (PRK08572)→<br>rpl14p (PRK08571)Ribosomal_L14 (pfam00238)→ rpl24p (PRK01191)KOW_RPL26 (cd06089)→<br>PRK04313 (PRK04313)KOW_RPS4 (cd06087)Ribosomal_S4e (pfam00900)→<br>rpl5p (PRK04219)Ribosomal_L5 (pfam00281)Ribosomal_L5_C (pfam00673)→<br>RpsN (COG0199)rps14P (PRK05766)→ rps8p (PRK04034)RpsH (COG0096)→<br>uL6_arch (TIGR03653)Ribosomal_L6 (pfam00347)→ rpl6p (PRK05518)Ribosomal_L6 (pfam00347)→<br>rpl32e (PRK08562)Ribosomal_L32_L32e (cd00513)→ rpl19e (PRK08570)Ribosomal_L19e (pfam01280)→<br>RplR (COG0256)rpl18p (PRK08569)→ RpsE (COG0098)rps5p (PRK04044)→
Mhu-1-1	NC_007796.1	-	2515622	2515559	**RNA**→ hypo→ RplC (COG0087)rpl3p (PRK04231)→ RplD (COG0088)rpl4lp (PRK04042)→<br>RplW (COG0089)PRK14548 (PRK14548)→ RplB (COG0090)rpl2p (PRK09612)→<br>RpsS (COG0185)rps19p (PRK04038)→ rpl22p (PRK04223)RplV (COG0091)→<br>RpsC (COG0092)rps3p (PRK04191)→ PRK14549 (PRK14549)Ribosomal_L29_HIP (cd00427)→<br>POP4 (COG1588)PRK03879 (PRK03879)→ RpsQ (COG0186)rps17p (PRK08572)→<br>rpl14p (PRK08571)Ribosomal_L14 (pfam00238)→ rpl24p (PRK01191)KOW_RPL26 (cd06089)→<br>PRK04313 (PRK04313)KOW_RPS4 (cd06087)Ribosomal_S4e (pfam00900)→<br>rpl5p (PRK04219)Ribosomal_L5 (pfam00281)Ribosomal_L5_C (pfam00673)→<br>RpsN (COG0199)rps14P (PRK05766)→
env-13	3300001567_10007289	+	4411	4498	**RNA**→ hypo→
env-14	BDMC2_contig11431	+	2927	3013	**RNA**→ rpl3p (PRK04231)Ribosomal_L3 (pfam00297)→ RplC (COG0087)rpl3p (PRK04231)→<br>RplD (COG0088)rpl4lp (PRK04042)→
env-15	APHM01006905.1	-	6665	6566	**RNA**→ Methyltrn_RNA_3 (pfam02598)→ RplC (COG0087)rpl3p (PRK04231)→<br>RplD (COG0088)rpl4lp (PRK04042)→ RplW (COG0089)PRK14548 (PRK14548)→<br>RplB (COG0090)rpl2p (PRK09612)→
env-16	LTJ07As871_J07ABscf098861	+	1109424	1109523	**RNA**→ Methyltrn_RNA_3 (pfam02598)→ RplC (COG0087)rpl3p (PRK04231)→<br>RplD (COG0088)rpl4lp (PRK04042)→ RplW (COG0089)PRK14548 (PRK14548)→<br>RplB (COG0090)rpl2p (PRK09612)→
Hwa-1-1	NC_017459.1	-	2240787	2240688	**RNA**→ Methyltrn_RNA_3 (pfam02598)→ RplC (COG0087)rpl3p (PRK04231)→<br>RplD (COG0088)rpl4lp (PRK04042)→ RplW (COG0089)PRK14548 (PRK14548)→<br>RplB (COG0090)rpl2p (PRK09612)→
env-17	77514049	-	684	585	**RNA**→ Methyltrn_RNA_3 (pfam02598)→
env-18	FLAS_READ_83861724	-	684	585	**RNA**→ ←hypo

env-19	P_A23_Liq_1_Fmt_1000094	+	5234	5333	**RNA**→ Methyltrn_RNA_3 (pfam02598)→ RplC (COG0087)rpl3p (PRK04231)→ RplD (COG0088)rpl4lp (PRK04042)→ RplW (COG0089)PRK14548 (PRK14548)→ RplB (COG0090)rpl2p (PRK09612)→
env-20	P_A23_Liq_3_Fmt_1000024	+	11155	11254	**RNA**→ Methyltrn_RNA_3 (pfam02598)→ RplC (COG0087)rpl3p (PRK04231)→ RplD (COG0088)rpl4lp (PRK04042)→ RplW (COG0089)PRK14548 (PRK14548)→ RplB (COG0090)rpl2p (PRK09612)→
env-21	P_A23_Liq_2_Fmt_1000003	-	57459	57360	**RNA**→ Methyltrn_RNA_3 (pfam02598)→ RplC (COG0087)rpl3p (PRK04231)→ RplD (COG0088)rpl4lp (PRK04042)→ RplW (COG0089)PRK14548 (PRK14548)→ RplB (COG0090)rpl2p (PRK09612)→
env-22	GSLNARP_contig28763	+	955	1054	**RNA**→ Methyltrn_RNA_3 (pfam02598)→ RplC (COG0087)rpl3p (PRK04231)→ rpl3p (PRK04231)Ribosomal_L3 (pfam00297)→ RplD (COG0088)rpl4lp (PRK04042)→ RplW (COG0089)PRK14548 (PRK14548)→ RplB (COG0090)rpl2p (PRK09612)→
Hwa-2-1	NC_008212.1	-	2141989	2141890	**RNA**→ Methyltrn_RNA_3 (pfam02598)→ RplC (COG0087)rpl3p (PRK04231)→ RplD (COG0088)rpl4lp (PRK04042)→ RplW (COG0089)PRK14548 (PRK14548)→ RplB (COG0090)rpl2p (PRK09612)→
env-23	GSLNARP_contig23680	+	166	268	**RNA**→ PRK07233 (PRK07233)Methyltrn_RNA_3 (pfam02598)→ rpl3p (PRK04231)Ribosomal_L3 (pfam00297)→
env-24	GSLNAS_F7I09SK02F1FOF	+	46	143	**RNA**→ MTH1 (COG2106)→
env-25	APHM01021632.1	-	3874	3775	**RNA**→ Methyltrn_RNA_3 (pfam02598)→ RplC (COG0087)rpl3p (PRK04231)→ RplD (COG0088)rpl4lp (PRK04042)→ RplW (COG0089)PRK14548 (PRK14548)→ RplB (COG0090)rpl2p (PRK09612)→ RpsS (COG0185)rps19p (PRK04038)→ rpl22p (PRK04223)RplV (COG0091)→
env-26	LTJ07As871_J07ABscf098869	-	558211	558112	**RNA**→ Methyltrn_RNA_3 (pfam02598)→ RplC (COG0087)rpl3p (PRK04231)→ RplD (COG0088)rpl4lp (PRK04042)→ RplW (COG0089)PRK14548 (PRK14548)→ RplB (COG0090)rpl2p (PRK09612)→ RpsS (COG0185)rps19p (PRK04038)→ rpl22p (PRK04223)RplV (COG0091)→ rpl22p (PRK04223)Ribosomal_L22 (pfam00237)→ RpsC (COG0092)rps3p (PRK04191)Astro_capsid_p (pfam12226)→ PRK14549 (PRK14549)Ribosomal_L29_HIP (cd00427)→ PRK03879 (PRK03879)UPF0086 (pfam01868)→ RpsQ (COG0186)rps17p (PRK08572)→ rpl14p (PRK08571)Ribosomal_L14 (pfam00238)→ RplX (COG0198)rpl24p (PRK01191)→ PRK04313 (PRK04313)KOW_RPS4 (cd06087)Ribosomal_S4e (pfam00900)→
env-27	PR_CR_10_Liq_1_inCR_1027519	-	654	552	**RNA**→ Methyltrn_RNA_3 (pfam02598)→ rpl3p (PRK04231)Ribosomal_L3 (pfam00297)→
env-28	SL_4KL_010_BRINE_10026912	-	325	226	**RNA**→ Methyltrn_RNA_3 (pfam02598)→
env-29	SL_5KL_010_BRINE_10001539	-	6337	6238	**RNA**→ Methyltrn_RNA_3 (pfam02598)→ RplC (COG0087)rpl3p (PRK04231)→ RplD (COG0088)rpl4lp (PRK04042)→ RplW (COG0089)PRK14548 (PRK14548)→ RplB (COG0090)rpl2p (PRK09612)→ RpsS (COG0185)rps19p (PRK04038)→ rpl22p (PRK04223)RplV (COG0091)→ RpsC (COG0092)rps3p (PRK04191)→ RpmC (COG0255)PRK14549 (PRK14549)→ PRK03879 (PRK03879)UPF0086 (pfam01868)→ RpsQ (COG0186)rps17p (PRK08572)→ rpl14p (PRK08571)→
env-30	SL_5KL_010_BRINE_10041088	-	903	804	**RNA**→ Methyltrn_RNA_3 (pfam02598)→ rpl3p (PRK04231)Ribosomal_L3 (pfam00297)→
env-31	P_1C_Sed_2_UnCty_1050957	-	389	290	**RNA**→ Methyltrn_RNA_3 (pfam02598)→ Methyltrn_RNA_3 (pfam02598)→
Hut-1-1	NC_013158.1	+	2340327	2340426	**RNA**→ Methyltrn_RNA_3 (pfam02598)→ rpl3p (PRK04231)Ribosomal_L3 (pfam00297)→ RplD (COG0088)rpl4lp (PRK04042)→ RplW (COG0089)PRK14548 (PRK14548)→ RplB (COG0090)rpl2p (PRK09612)→ RpsS (COG0185)rps19p (PRK04038)→ rpl22p (PRK04223)RplV (COG0091)→ RpsC (COG0092)rps3p (PRK04191)→ PRK14549 (PRK14549)Ribosomal_L29 (pfam00831)→ PRK03879 (PRK03879)UPF0086 (pfam01868)→ RpsQ (COG0186)rps17p (PRK08572)→ rpl14p (PRK08571)Ribosomal_L14 (pfam00238)→ RplX (COG0198)rpl24p (PRK01191)→ PRK04313 (PRK04313)KOW_RPS4 (cd06087)Ribosomal_S4e (pfam00900)→ RplE (COG0094)rpl5p (PRK04219)→ RpsN (COG0199)rps14P (PRK05766)→ rps8p (PRK04034)RpsH (COG0096)→ RplF (COG0097)rpl6p (PRK05518)→ NT_POLXc (cd00141)PRK12766 (PRK12766)Rpl32e (COG1717)COG2251 (COG2251)HHH_5 (pfam14520)→
env-32	GSLSAAI_contig12629	-	3841	3739	**RNA**→ Methyltrn_RNA_3 (pfam02598)→ RplC (COG0087)rpl3p (PRK04231)→ rpl4lp (PRK04042)Ribosomal_L4 (pfam00573)→ RplW (COG0089)PRK14548 (PRK14548)→ RplB (COG0090)rpl2p (PRK09612)→ RpsS (COG0185)rps19p (PRK04038)→ rpl22p (PRK04223)RplV (COG0091)→
env-33	GSLSAAI_contig12562	-	1374	1272	**RNA**→ Methyltrn_RNA_3 (pfam02598)→ RplC (COG0087)rpl3p (PRK04231)→

env-34	GSLSAAI_contig12554	-	3225	3123	**RNA**→ Methyltrn_RNA_3 (pfam02598)→ RplC (COG0087)rpl3p (PRK04231)→ RplD (COG0088)rpl4lp (PRK04042)→ RplW (COG0089)PRK14548 (PRK14548)→ RplB (COG0090)rpl2p (PRK09612)→
env-35	GSLSAAI_contig12558	-	4430	4328	**RNA**→ Methyltrn_RNA_3 (pfam02598)→ RplC (COG0087)rpl3p (PRK04231)→ RplD (COG0088)rpl4lp (PRK04042)→ RplW (COG0089)PRK14548 (PRK14548)→ RplB (COG0090)rpl2p (PRK09612)→ RpsS (COG0185)rps19p (PRK04038)→ rpl22p (PRK04223)RplV (COG0091)→ KH (smart00322)archeal_30S_S3_KH (cd02411)→
env-36	SL_6KL_011_BRINE_1000585	-	15210	15108	**RNA**→ Methyltrn_RNA_3 (pfam02598)→ RplC (COG0087)rpl3p (PRK04231)→ RplD (COG0088)rpl4lp (PRK04042)→ RplW (COG0089)uL23_arch (TIGR03636)→ RplB (COG0090)rpl2p (PRK09612)→ RpsS (COG0185)rps19p (PRK04038)→ rpl22p (PRK04223)RplV (COG0091)→ RpsC (COG0092)rps3p (PRK04191)→ PRK14549 (PRK14549)Ribosomal_L29 (pfam00831)→ POP4 (COG1588)PRK03879 (PRK03879)→ RpsQ (COG0186)rps17p (PRK08572)→ rpl14p (PRK08571)Ribosomal_L14 (pfam00238)→ rpl24p (PRK01191)KOW_RPL26 (cd06089)→ PRK04313 (PRK04313)KOW_RPS4 (cd06087)S4 (pfam01479)Ribosomal_S4e (pfam00900)→ RplE (COG0094)rpl5p (PRK04219)→ RpsN (COG0199)rps14P (PRK05766)→ rps8p (PRK04034)RpsH (COG0096)→ RplF (COG0097)rpl6p (PRK05518)→ Rpl32e (COG1717)rpl32e (PRK08562)HHH_5 (pfam14520)→
env-37	SL_5KL_010_BRINE_10000390	+	6046	6145	**RNA**→ Methyltrn_RNA_3 (pfam02598)→ rpl3p (PRK04231)Ribosomal_L3 (pfam00297)→ RplD (COG0088)rpl4lp (PRK04042)→ RplW (COG0089)PRK14548 (PRK14548)→ RplB (COG0090)rpl2p (PRK09612)→ RpsS (COG0185)rps19p (PRK04038)→ rpl22p (PRK04223)RplV (COG0091)→ RpsC (COG0092)rps3p (PRK04191)→ PRK14549 (PRK14549)Ribosomal_L29 (pfam00831)→ PRK03879 (PRK03879)UPF0086 (pfam01868)→ RpsQ (COG0186)rps17p (PRK08572)→ rpl14p (PRK08571)Ribosomal_L14 (pfam00238)→ RplX (COG0198)rpl24p (PRK01191)→ PRK04313 (PRK04313)KOW_RPS4 (cd06087)S4 (pfam01479)Ribosomal_S4e (pfam00900)→ RplE (COG0094)rpl5p (PRK04219)→ RpsN (COG0199)rps14P (PRK05766)→ rps8p (PRK04034)RpsH (COG0096)→ RplF (COG0097)rpl6p (PRK05518)→ ComEA (COG1555)Rpl32e (COG1717)rpl32e (PRK08562)HHH_5 (pfam14520)→ rpl19e (PRK08570)Ribosomal_L19e (pfam01280)→ RplR (COG0256)rpl18p (PRK08569)→ RpsE (COG0098)rps5p (PRK04044)→ Ribosomal_L7_archeal_euk (cd01657)rpl30p (PRK06049)→ rpl15p (PRK06419)Ribosomal_L27A (pfam00828)→ SecY (COG0201)PRK08568 (PRK08568)→
env-38	SL_4KL_010_BRINE_10108326	+	36	135	**RNA**→ Methyltrn_RNA_3 (pfam02598)→ rpl3p (PRK04231)Ribosomal_L3 (pfam00297)→
env-39	SL_8KL_010_SED_10227597	-	345	246	**RNA**→ Methyltrn_RNA_3 (pfam02598)→
env-40	SS_3KL_010_SOIL_10336605	-	246	147	**RNA**→ Methyltrn_RNA_3 (pfam02598)→
env-41	APHM01002125.1	+	421	520	**RNA**→ Methyltrn_RNA_3 (pfam02598)→ RplC (COG0087)rpl3p (PRK04231)→ RplD (COG0088)rpl4lp (PRK04042)→ RplW (COG0089)PRK14548 (PRK14548)→ rpl2p (PRK09612)Ribosomal_L2 (pfam00181)→
env-42	LTJ07As871_J07ABscf098859	-	2359772	2359673	**RNA**→ Methyltrn_RNA_3 (pfam02598)→ RplC (COG0087)rpl3p (PRK04231)→ RplD (COG0088)rpl4lp (PRK04042)→ RplW (COG0089)PRK14548 (PRK14548)→ rpl2p (PRK09612)Ribosomal_L2 (pfam00181)→ PRK12317 (PRK12317)Translation_factor_III (cd01513)GTPBP_II (cd03694)→
env-43	JXWU01069486.1	+	361	460	**RNA**→ Methyltrn_RNA_3 (pfam02598)→
env-44	SS_3KL_010_SOIL_10060740	-	118	19	**RNA**→ Methyltrn_RNA_3 (pfam02598)→
env-45	SL_5KL_010_BRINE_10001245	+	528	630	**RNA**→ Methyltrn_RNA_3 (pfam02598)→ rpl3p (PRK04231)Ribosomal_L3 (pfam00297)→ RplD (COG0088)rpl4lp (PRK04042)→ RplW (COG0089)PRK14548 (PRK14548)→ RplB (COG0090)rpl2p (PRK09612)→ RpsS (COG0185)rps19p (PRK04038)→ rpl22p (PRK04223)RplV (COG0091)→ RpsC (COG0092)rps3p (PRK04191)→ PRK14549 (PRK14549)Ribosomal_L29 (pfam00831)→ PRK03879 (PRK03879)UPF0086 (pfam01868)→ RpsQ (COG0186)rps17p (PRK08572)→ rpl14p (PRK08571)Ribosomal_L14 (pfam00238)→ rpl24p (PRK01191)KOW_RPL26 (cd06089)→ PRK04313 (PRK04313)KOW_RPS4 (cd06087)Ribosomal_S4e (pfam00900)→ RplE (COG0094)rpl5p (PRK04219)→ RpsN (COG0199)rps14P (PRK05766)→ rps8p (PRK04034)RpsH (COG0096)→ RplF (COG0097)rpl6p (PRK05518)→ PRK12766 (PRK12766)RecA (COG0468)Rpl32e (COG1717)→ rpl19e (PRK08570)Ribosomal_L19e (pfam01280)→ RplR (COG0256)rpl18p (PRK08569)→ RpsE (COG0098)rps5p (PRK04044)→ Ribosomal_L7_archeal_euk (cd01657)rpl30p (PRK06049)→

env-46	SL_6KL_011_BRINE_1001562	+	7391	7493	**RNA**→ Methyltrn_RNA_3 (pfam02598)→ rpl3p (PRK04231)Ribosomal_L3 (pfam00297)→<br>RplD (COG0088)rpl4lp (PRK04042)→ RplW (COG0089)PRK14548 (PRK14548)→<br>RplB (COG0090)rpl2p (PRK09612)→ RpsS (COG0185)rps19p (PRK04038)→<br>rpl22p (PRK04223)RplV (COG0091)→
env-47	SL_4KL_010_BRINE_10002586	+	550	652	**RNA**→ Methyltrn_RNA_3 (pfam02598)→ rpl3p (PRK04231)Ribosomal_L3 (pfam00297)→<br>RplD (COG0088)rpl4lp (PRK04042)→ RplW (COG0089)PRK14548 (PRK14548)→<br>RplB (COG0090)rpl2p (PRK09612)→ RpsS (COG0185)rps19p (PRK04038)→<br>rpl22p (PRK04223)RplV (COG0091)→ RpsC (COG0092)rps3p (PRK04191)→<br>PRK14549 (PRK14549)Ribosomal_L29 (pfam00831)→ PRK03879 (PRK03879)UPF0086 (pfam01868)→<br>RpsQ (COG0186)rps17p (PRK08572)→ rpl14p (PRK08571)Ribosomal_L14 (pfam00238)→<br>rpl24p (PRK01191)KOW_RPL26 (cd06089)→<br>PRK04313 (PRK04313)KOW_RPS4 (cd06087)Ribosomal_S4e (pfam00900)→ RplE (COG0094)rpl5p (PRK04219)→<br>RpsN (COG0199)rps14P (PRK05766)→ rps8p (PRK04034)RpsH (COG0096)→<br>RplF (COG0097)rpl6p (PRK05518)→ RecA (COG0468)Rpl32e (COG1717)HHH_5 (pfam14520)→
env-48	SL_7KL_010_BRINE_10000614	+	5277	5379	**RNA**→ Methyltrn_RNA_3 (pfam02598)→ rpl3p (PRK04231)Ribosomal_L3 (pfam00297)→<br>RplD (COG0088)rpl4lp (PRK04042)→ RplW (COG0089)PRK14548 (PRK14548)→<br>RplB (COG0090)rpl2p (PRK09612)→ RpsS (COG0185)rps19p (PRK04038)→<br>rpl22p (PRK04223)RplV (COG0091)→ RpsC (COG0092)rps3p (PRK04191)→<br>PRK14549 (PRK14549)Ribosomal_L29 (pfam00831)→ PRK03879 (PRK03879)UPF0086 (pfam01868)→<br>RpsQ (COG0186)rps17p (PRK08572)→ rpl14p (PRK08571)Ribosomal_L14 (pfam00238)→<br>rpl24p (PRK01191)KOW_RPL26 (cd06089)→<br>PRK04313 (PRK04313)KOW_RPS4 (cd06087)Ribosomal_S4e (pfam00900)→ RplE (COG0094)rpl5p (PRK04219)→<br>RpsN (COG0199)rps14P (PRK05766)→ rps8p (PRK04034)RpsH (COG0096)→<br>RplF (COG0097)rpl6p (PRK05518)→<br>RecA (COG0468)Rpl32e (COG1717)rpl32e (PRK08562)HHH_5 (pfam14520)→<br>rpl19e (PRK08570)Ribosomal_L19e (pfam01280)→ RplR (COG0256)rpl18p (PRK08569)→<br>RpsE (COG0098)rps5p (PRK04044)→
env-49	GSLNARP_contig28693	+	496	598	**RNA**→ Methyltrn_RNA_3 (pfam02598)→ RplC (COG0087)rpl3p (PRK04231)→<br>RplD (COG0088)rpl4lp (PRK04042)→ RplW (COG0089)PRK14548 (PRK14548)→<br>RplB (COG0090)rpl2p (PRK09612)→ RpsS (COG0185)rps19p (PRK04038)→<br>rpl22p (PRK04223)RplV (COG0091)→ RpsC (COG0092)rps3p (PRK04191)→<br>PRK14549 (PRK14549)Ribosomal_L29 (pfam00831)→ PRK03879 (PRK03879)UPF0086 (pfam01868)→<br>RpsQ (COG0186)rps17p (PRK08572)→ rpl14p (PRK08571)Ribosomal_L14 (pfam00238)→<br>rpl24p (PRK01191)KOW_RPL26 (cd06089)→
env-50	SL_5KL_010_BRINE_10001521	+	7795	7897	**RNA**→ Methyltrn_RNA_3 (pfam02598)→ RplC (COG0087)rpl3p (PRK04231)→<br>RplD (COG0088)rpl4lp (PRK04042)→ RplW (COG0089)PRK14548 (PRK14548)→<br>RplB (COG0090)rpl2p (PRK09612)→ rps19p (PRK04038)→
env-51	SL_6KL_011_BRINE_1000203	-	3778	3676	**RNA**→ Methyltrn_RNA_3 (pfam02598)→ RplC (COG0087)rpl3p (PRK04231)→<br>RplD (COG0088)rpl4lp (PRK04042)→ RplW (COG0089)PRK14548 (PRK14548)→<br>RplB (COG0090)rpl2p (PRK09612)→ RpsS (COG0185)rps19p (PRK04038)→
env-52	SL_5KL_010_SED_10001876	-	10224	10122	**RNA**→ Methyltrn_RNA_3 (pfam02598)→ RplC (COG0087)rpl3p (PRK04231)→<br>RplD (COG0088)rpl4lp (PRK04042)→ RplW (COG0089)PRK14548 (PRK14548)→<br>RplB (COG0090)rpl2p (PRK09612)→ RpsS (COG0185)rps19p (PRK04038)→<br>rpl22p (PRK04223)RplV (COG0091)→ RpsC (COG0092)rps3p (PRK04191)→<br>PRK14549 (PRK14549)Ribosomal_L29 (pfam00831)→ PRK03879 (PRK03879)UPF0086 (pfam01868)→<br>RpsQ (COG0186)rps17p (PRK08572)→ rpl14p (PRK08571)Ribosomal_L14 (pfam00238)→<br>rpl24p (PRK01191)KOW_RPL26 (cd06089)→<br>PRK04313 (PRK04313)S4 (cd00165)KOW_RPS4 (cd06087)Ribosomal_S4e (pfam00900)→<br>RplE (COG0094)rpl5p (PRK04219)→ RpsN (COG0199)rps14P (PRK05766)→<br>rps8p (PRK04034)RpsH (COG0096)→ RplF (COG0097)rpl6p (PRK05518)→<br>UvrC (COG0322)rpl32e (PRK08562)Ribosomal_L32e (pfam01655)HHH_5 (pfam14520)→<br>rpl19e (PRK08570)Ribosomal_L19e (pfam01280)→
env-53	SL_4KL_010_BRINE_10000708	+	14067	14169	**RNA**→ Methyltrn_RNA_3 (pfam02598)→ RplC (COG0087)rpl3p (PRK04231)→<br>RplD (COG0088)rpl4lp (PRK04042)→ RplW (COG0089)PRK14548 (PRK14548)→<br>RplB (COG0090)rpl2p (PRK09612)→ rps19p (PRK04038)→
env-54	P_1C_Liq_1_UnCty_1162688	-	122	20	**RNA**→ MTH1 (COG2106)→

env-55	SL_6KL_011_BRINE_1072209	-	402	300	**RNA**→ Methyltrn_RNA_3 (pfam02598)→
env-56	SL_5KL_010_BRINE_10188769	-	355	253	**RNA**→ Methyltrn_RNA_3 (pfam02598)→
env-57	SL_4KL_010_BRINE_10162798	+	316	418	**RNA**→ Methyltrn_RNA_3 (pfam02598)→
env-58	SL_6KL_011_BRINE_1009000	+	854	956	**RNA**→ Methyltrn_RNA_3 (pfam02598)→ RplC (COG0087)rpl3p (PRK04231)→ RplD (COG0088)rpl4lp (PRK04042)→ RplW (COG0089)PRK14548 (PRK14548)→ RplB (COG0090)rpl2p (PRK09612)→ rps19p (PRK04038)→
env-59	ADL20m3uS_contig12843	-	170	68	**RNA**→ Methyltrn_RNA_3 (pfam02598)→
env-60	2140918027-12578	+	373	475	**RNA**→
env-61	2140918027-03821	+	1792	1894	**RNA**→ Methyltrn_RNA_3 (pfam02598)→
env-62	ADL20m3uS_contig10848	-	468	366	**RNA**→ Methyltrn_RNA_3 (pfam02598)→
env-63	ADL5mRS1u_contig00538	+	967	1069	**RNA**→ Methyltrn_RNA_3 (pfam02598)→ rpl3p (PRK04231)Ribosomal_L3 (pfam00297)→ RplC (COG0087)rpl3p (PRK04231)→ RplD (COG0088)rpl4lp (PRK04042)→ RplW (COG0089)PRK14548 (PRK14548)→ RplB (COG0090)rpl2p (PRK09612)→ RpsS (COG0185)rps19p (PRK04038)→
env-64	GQKUMWE02JS9JS	-	377	275	**RNA**→ Methyltrn_RNA_3 (pfam02598)→
env-65	ADL20m3uS_contig10578	-	446	344	**RNA**→ Methyltrn_RNA_3 (pfam02598)→
env-66	2140918017-36	-	14923	14821	**RNA**→ Methyltrn_RNA_3 (pfam02598)→ rpl3p (PRK04231)Ribosomal_L3 (pfam00297)→ RplC (COG0087)rpl3p (PRK04231)→ RplD (COG0088)rpl4lp (PRK04042)→ RplW (COG0089)PRK14548 (PRK14548)→ RplB (COG0090)rpl2p (PRK09612)→ RpsS (COG0185)rps19p (PRK04038)→ rpl22p (PRK04223)RplV (COG0091)→ RpsC (COG0092)rps3p (PRK04191)→ PRK14549 (PRK14549)Ribosomal_L29 (pfam00831)→ PRK03879 (PRK03879)UPF0086 (pfam01868)→ RpsQ (COG0186)rps17p (PRK08572)→ rpl14p (PRK08571)Ribosomal_L14 (pfam00238)→ rpl24p (PRK01191)KOW_RPL26 (cd06089)→ PRK04313 (PRK04313)KOW_RPS4 (cd06087)Ribosomal_S4e (pfam00900)→ RplE (COG0094)rpl5p (PRK04219)→ RpsN (COG0199)rps14P (PRK05766)→ rps8p (PRK04034)RpsH (COG0096)→ RplF (COG0097)rpl6p (PRK05518)→ UvrC (COG0322)Rpl32e (COG1717)HHH_5 (pfam14520)→ rpl19e (PRK08570)Ribosomal_L19e (pfam01280)→
env-67	GRCP39E01CI3EF	-	306	204	**RNA**→ Methyltrn_RNA_3 (pfam02598)→
env-68	GRCP39E01CZCI9	+	86	188	**RNA**→ Methyltrn_RNA_3 (pfam02598)→
env-69	2140918027-00954	+	88	190	**RNA**→ Methyltrn_RNA_3 (pfam02598)→ rpl3p (PRK04231)Ribosomal_L3 (pfam00297)→ RplC (COG0087)rpl3p (PRK04231)→ RplD (COG0088)rpl4lp (PRK04042)→ RplW (COG0089)PRK14548 (PRK14548)→ RplB (COG0090)rpl2p (PRK09612)→ RpsS (COG0185)rps19p (PRK04038)→ rpl22p (PRK04223)RplV (COG0091)→ RpsC (COG0092)rps3p (PRK04191)→ PRK14549 (PRK14549)Ribosomal_L29 (pfam00831)→ PRK03879 (PRK03879)UPF0086 (pfam01868)→
env-70	GQKUMWE02F1GR6	+	61	163	**RNA**→ Methyltrn_RNA_3 (pfam02598)→
env-71	GQKUMWE02JMQQS	+	64	166	**RNA**→ Methyltrn_RNA_3 (pfam02598)→
env-72	SL_8KL_010_SED_10008943	+	2731	2830	**RNA**→ Methyltrn_RNA_3 (pfam02598)→ RplC (COG0087)rpl3p (PRK04231)→ RplD (COG0088)rpl4lp (PRK04042)→ RplW (COG0089)PRK14548 (PRK14548)→ RplB (COG0090)rpl2p (PRK09612)→ RpsS (COG0185)rps19p (PRK04038)→ rpl22p (PRK04223)RplV (COG0091)→ KH (smart00322)archeal_30S_S3_KH (cd02411)→
env-73	SL_4KL_010_BRINE_10005953	+	2732	2831	**RNA**→ Methyltrn_RNA_3 (pfam02598)→ RplC (COG0087)rpl3p (PRK04231)→ RplD (COG0088)rpl4lp (PRK04042)→ RplW (COG0089)PRK14548 (PRK14548)→ RplB (COG0090)rpl2p (PRK09612)→ RpsS (COG0185)rps19p (PRK04038)→ rpl22p (PRK04223)RplV (COG0091)→ KH (smart00322)archeal_30S_S3_KH (cd02411)→
env-74	SL_5KL_010_BRINE_10000601	+	12793	12892	**RNA**→ Methyltrn_RNA_3 (pfam02598)→ RplC (COG0087)rpl3p (PRK04231)→ rpl4lp (PRK04042)Ribosomal_L4 (pfam00573)→ rpl4lp (PRK04042)→ RplW (COG0089)PRK14548 (PRK14548)→ RplB (COG0090)rpl2p (PRK09612)→ RpsS (COG0185)rps19p (PRK04038)→ rpl22p (PRK04223)RplV (COG0091)→ KH (smart00322)archeal_30S_S3_KH (cd02411)→
env-75	SL_5KL_010_SED_10018610	-	1058	959	**RNA**→ Methyltrn_RNA_3 (pfam02598)→ rpl3p (PRK04231)Ribosomal_L3 (pfam00297)→
env-76	JXWU01116399.1	-	162	60	**RNA**→ Methyltrn_RNA_3 (pfam02598)→
env-77	GSLNARP_GFPJP1N02IUPJO	-	257	155	**RNA**→ Methyltrn_RNA_3 (pfam02598)→
env-78	GSLNARP_GFPJP1N02J3NU6	-	222	120	**RNA**→ Methyltrn_RNA_3 (pfam02598)→

Hsa-1-1	NZ_BBMO01000001.1	-	854034	853935	**RNA**→ Methyltrn_RNA_3 (pfam02598)→ RplC (COG0087)rpl3p (PRK04231)→ RplD (COG0088)rpl4lp (PRK04042)→ RplW (COG0089)PRK14548 (PRK14548)→ RplB (COG0090)rpl2p (PRK09612)→ RpsS (COG0185)rps19p (PRK04038)→ rpl22p (PRK04223)RplV (COG0091)→ RpsC (COG0092)rps3p (PRK04191)→ PRK14549 (PRK14549)Ribosomal_L29 (pfam00831)→ PRK03879 (PRK03879)UPF0086 (pfam01868)→ RpsQ (COG0186)rps17p (PRK08572)→ rpl14p (PRK08571)Ribosomal_L14 (pfam00238)→ rpl24p (PRK01191)KOW_RPL26 (cd06089)→ PRK04313 (PRK04313)KOW_RPS4 (cd06087)S4 (pfam01479)Ribosomal_S4e (pfam00900)→ RplE (COG0094)rpl5p (PRK04219)→ RpsN (COG0199)rps14P (PRK05766)→ rps8p (PRK04034)RpsH (COG0096)→ RplF (COG0097)rpl6p (PRK05518)→ PRK12766 (PRK12766)RecA (COG0468)Rpl32e (COG1717)→ rpl19e (PRK08570)Ribosomal_L19e (pfam01280)→
Hgi-1-1	NZ_AOLJ01000014.1	+	202046	202145	**RNA**→ Methyltrn_RNA_3 (pfam02598)→ rpl3p (PRK04231)Ribosomal_L3 (pfam00297)→ RplD (COG0088)rpl4lp (PRK04042)→ RplW (COG0089)PRK14548 (PRK14548)→ RplB (COG0090)rpl2p (PRK09612)→ RpsS (COG0185)rps19p (PRK04038)→ rpl22p (PRK04223)RplV (COG0091)→ RpsC (COG0092)rps3p (PRK04191)→ PRK14549 (PRK14549)Ribosomal_L29 (pfam00831)→ PRK03879 (PRK03879)UPF0086 (pfam01868)→ RpsQ (COG0186)rps17p (PRK08572)→ rpl14p (PRK08571)Ribosomal_L14 (pfam00238)→ RplX (COG0198)rpl24p (PRK01191)→ PRK04313 (PRK04313)KOW_RPS4 (cd06087)Ribosomal_S4e (pfam00900)→ RplE (COG0094)rpl5p (PRK04219)→ RpsN (COG0199)rps14P (PRK05766)→ rps8p (PRK04034)RpsH (COG0096)→ RplF (COG0097)rpl6p (PRK05518)→ PRK12766 (PRK12766)RecA (COG0468)Rpl32e (COG1717)→ rpl19e (PRK08570)Ribosomal_L19e (pfam01280)→
Hgi-2-1	NZ_CP011947.1	-	2522923	2522824	**RNA**→ Methyltrn_RNA_3 (pfam02598)→ rpl3p (PRK04231)Ribosomal_L3 (pfam00297)→ RplD (COG0088)rpl4lp (PRK04042)→ RplW (COG0089)PRK14548 (PRK14548)→ RplB (COG0090)rpl2p (PRK09612)→ RpsS (COG0185)rps19p (PRK04038)→ rpl22p (PRK04223)RplV (COG0091)→ RpsC (COG0092)rps3p (PRK04191)→ PRK14549 (PRK14549)Ribosomal_L29 (pfam00831)→ PRK03879 (PRK03879)UPF0086 (pfam01868)→ RpsQ (COG0186)rps17p (PRK08572)→ rpl14p (PRK08571)Ribosomal_L14 (pfam00238)→ RplX (COG0198)rpl24p (PRK01191)→ PRK04313 (PRK04313)KOW_RPS4 (cd06087)Ribosomal_S4e (pfam00900)→ RplE (COG0094)rpl5p (PRK04219)→ RpsN (COG0199)rps14P (PRK05766)→ rps8p (PRK04034)RpsH (COG0096)→ RplF (COG0097)rpl6p (PRK05518)→ PRK12766 (PRK12766)RecA (COG0468)Rpl32e (COG1717)→ rpl19e (PRK08570)Ribosomal_L19e (pfam01280)→
Hsu-2-1	NZ_AOLM01000020.1	+	180613	180712	**RNA**→ Methyltrn_RNA_3 (pfam02598)→ rpl3p (PRK04231)Ribosomal_L3 (pfam00297)→ RplD (COG0088)rpl4lp (PRK04042)→ RplW (COG0089)PRK14548 (PRK14548)→ RplB (COG0090)rpl2p (PRK09612)→ RpsS (COG0185)rps19p (PRK04038)→ rpl22p (PRK04223)RplV (COG0091)→ RpsC (COG0092)rps3p (PRK04191)→ PRK14549 (PRK14549)Ribosomal_L29 (pfam00831)→ PRK03879 (PRK03879)UPF0086 (pfam01868)→ RpsQ (COG0186)rps17p (PRK08572)→ rpl14p (PRK08571)Ribosomal_L14 (pfam00238)→ RplX (COG0198)rpl24p (PRK01191)→ PRK04313 (PRK04313)KOW_RPS4 (cd06087)Ribosomal_S4e (pfam00900)→ RplE (COG0094)rpl5p (PRK04219)→ RpsN (COG0199)rps14P (PRK05766)→ rps8p (PRK04034)RpsH (COG0096)→ RplF (COG0097)rpl6p (PRK05518)→ PRK12766 (PRK12766)RecA (COG0468)Rpl32e (COG1717)→ rpl19e (PRK08570)Ribosomal_L19e (pfam01280)→
Hpr-1-1	NZ_AOLG01000047.1	+	231631	231730	**RNA**→ Methyltrn_RNA_3 (pfam02598)→ rpl3p (PRK04231)Ribosomal_L3 (pfam00297)→ RplD (COG0088)rpl4lp (PRK04042)→ RplW (COG0089)PRK14548 (PRK14548)→ RplB (COG0090)rpl2p (PRK09612)→ RpsS (COG0185)rps19p (PRK04038)→ rpl22p (PRK04223)RplV (COG0091)→ RpsC (COG0092)rps3p (PRK04191)→ PRK14549 (PRK14549)Ribosomal_L29 (pfam00831)→ PRK03879 (PRK03879)UPF0086 (pfam01868)→ RpsQ (COG0186)rps17p (PRK08572)→ rpl14p (PRK08571)Ribosomal_L14 (pfam00238)→ RplX (COG0198)rpl24p (PRK01191)→ PRK04313 (PRK04313)KOW_RPS4 (cd06087)Ribosomal_S4e (pfam00900)→ RplE (COG0094)rpl5p (PRK04219)→ RpsN (COG0199)rps14P (PRK05766)→ rps8p (PRK04034)RpsH (COG0096)→ RplF (COG0097)rpl6p (PRK05518)→ PRK12766 (PRK12766)RecA (COG0468)Rpl32e (COG1717)→ rpl19e (PRK08570)Ribosomal_L19e (pfam01280)→

Hsp-8-1	NZ_JPES01000061.1	+	4380	4479	**RNA**→ Methyltrn_RNA_3 (pfam02598)→ hypo→ rpl3p (PRK04231)Ribosomal_L3 (pfam00297)→<br>RplD (COG0088)rpl4lp (PRK04042)→ RplW (COG0089)PRK14548 (PRK14548)→<br>RplB (COG0090)rpl2p (PRK09612)→ RpsS (COG0185)rps19p (PRK04038)→<br>rpl22p (PRK04223)RplV (COG0091)→ hypo→ RpsC (COG0092)rps3p (PRK04191)→<br>PRK14549 (PRK14549)Ribosomal_L29 (pfam00831)→ PRK03879 (PRK03879)UPF0086 (pfam01868)→<br>RpsQ (COG0186)rps17p (PRK08572)→ hypo→ rpl14p (PRK08571)Ribosomal_L14 (pfam00238)→<br>RplX (COG0198)rpl24p (PRK01191)→<br>PRK04313 (PRK04313)KOW_RPS4 (cd06087)Ribosomal_S4e (pfam00900)→ RplE (COG0094)rpl5p (PRK04219)→<br>RpsN (COG0199)rps14P (PRK05766)→ rps8p (PRK04034)RpsH (COG0096)→<br>RplF (COG0097)rpl6p (PRK05518)→ PRK12766 (PRK12766)RecA (COG0468)Rpl32e (COG1717)→<br>rpl19e (PRK08570)Ribosomal_L19e (pfam01280)→ RplR (COG0256)rpl18p (PRK08569)→ hypo→<br>RpsE (COG0098)rps5p (PRK04044)→ Ribosomal_L7_archeal_euk (cd01657)rpl30p (PRK06049)→<br>rpl15p (PRK06419)Ribosomal_L27A (pfam00828)→ SecY (COG0201)PRK08568 (PRK08568)→
Hde-1-1	NZ_AOLP01000011.1	+	331865	331964	**RNA**→ Methyltrn_RNA_3 (pfam02598)→ rpl3p (PRK04231)Ribosomal_L3 (pfam00297)→<br>RplD (COG0088)rpl4lp (PRK04042)→ RplW (COG0089)PRK14548 (PRK14548)→<br>RplB (COG0090)rpl2p (PRK09612)→ RpsS (COG0185)rps19p (PRK04038)→<br>rpl22p (PRK04223)RplV (COG0091)→ RpsC (COG0092)rps3p (PRK04191)→<br>PRK14549 (PRK14549)Ribosomal_L29 (pfam00831)→ PRK03879 (PRK03879)UPF0086 (pfam01868)→<br>RpsQ (COG0186)rps17p (PRK08572)→ rpl14p (PRK08571)Ribosomal_L14 (pfam00238)→<br>RplX (COG0198)rpl24p (PRK01191)→<br>PRK04313 (PRK04313)KOW_RPS4 (cd06087)Ribosomal_S4e (pfam00900)→ RplE (COG0094)rpl5p (PRK04219)→<br>RpsN (COG0199)rps14P (PRK05766)→ rps8p (PRK04034)RpsH (COG0096)→<br>RplF (COG0097)rpl6p (PRK05518)→ PRK12766 (PRK12766)RecA (COG0468)Rpl32e (COG1717)→<br>rpl19e (PRK08570)Ribosomal_L19e (pfam01280)→
Hlu-1-1	NZ_AOLH01000006.1	+	206888	206987	**RNA**→ Methyltrn_RNA_3 (pfam02598)→ rpl3p (PRK04231)Ribosomal_L3 (pfam00297)→<br>RplD (COG0088)rpl4lp (PRK04042)→ RplW (COG0089)PRK14548 (PRK14548)→<br>RplB (COG0090)rpl2p (PRK09612)→ RpsS (COG0185)rps19p (PRK04038)→<br>rpl22p (PRK04223)RplV (COG0091)→ RpsC (COG0092)rps3p (PRK04191)→<br>PRK14549 (PRK14549)Ribosomal_L29 (pfam00831)→ PRK03879 (PRK03879)UPF0086 (pfam01868)→<br>RpsQ (COG0186)rps17p (PRK08572)→ rpl14p (PRK08571)Ribosomal_L14 (pfam00238)→<br>RplX (COG0198)rpl24p (PRK01191)→<br>PRK04313 (PRK04313)KOW_RPS4 (cd06087)Ribosomal_S4e (pfam00900)→ RplE (COG0094)rpl5p (PRK04219)→<br>RpsN (COG0199)rps14P (PRK05766)→ rps8p (PRK04034)RpsH (COG0096)→<br>RplF (COG0097)rpl6p (PRK05518)→ PRK12766 (PRK12766)RecA (COG0468)Rpl32e (COG1717)→<br>rpl19e (PRK08570)Ribosomal_L19e (pfam01280)→
Hal-2-1	NZ_LK053000.1	-	908912	908813	**RNA**→ Methyltrn_RNA_3 (pfam02598)→ rpl3p (PRK04231)Ribosomal_L3 (pfam00297)→<br>RplD (COG0088)rpl4lp (PRK04042)→ RplW (COG0089)PRK14548 (PRK14548)→<br>RplB (COG0090)rpl2p (PRK09612)→ RpsS (COG0185)rps19p (PRK04038)→<br>rpl22p (PRK04223)RplV (COG0091)→ RpsC (COG0092)rps3p (PRK04191)→<br>PRK14549 (PRK14549)Ribosomal_L29 (pfam00831)→ PRK03879 (PRK03879)UPF0086 (pfam01868)→<br>RpsQ (COG0186)rps17p (PRK08572)→ rpl14p (PRK08571)Ribosomal_L14 (pfam00238)→<br>RplX (COG0198)rpl24p (PRK01191)→<br>PRK04313 (PRK04313)KOW_RPS4 (cd06087)Ribosomal_S4e (pfam00900)→ RplE (COG0094)rpl5p (PRK04219)→<br>RpsN (COG0199)rps14P (PRK05766)→ rps8p (PRK04034)RpsH (COG0096)→<br>RplF (COG0097)rpl6p (PRK05518)→ PRK12766 (PRK12766)RecA (COG0468)Rpl32e (COG1717)→<br>rpl19e (PRK08570)Ribosomal_L19e (pfam01280)→

Hsp-6-1	NZ_AOLD01000045.1	-	26639	26540	**RNA**→ Methyltrn_RNA_3 (pfam02598)→ rpl3p (PRK04231)Ribosomal_L3 (pfam00297)→<br>RplD (COG0088)rpl4lp (PRK04042)→ RplW (COG0089)PRK14548 (PRK14548)→<br>RplB (COG0090)rpl2p (PRK09612)→ RpsS (COG0185)rps19p (PRK04038)→<br>rpl22p (PRK04223)RplV (COG0091)→ RpsC (COG0092)rps3p (PRK04191)→<br>PRK14549 (PRK14549)Ribosomal_L29 (pfam00831)→ PRK03879 (PRK03879)UPF0086 (pfam01868)→<br>RpsQ (COG0186)rps17p (PRK08572)→ rpl14p (PRK08571)Ribosomal_L14 (pfam00238)→<br>RplX (COG0198)rpl24p (PRK01191)→<br>PRK04313 (PRK04313)KOW_RPS4 (cd06087)Ribosomal_S4e (pfam00900)→ RplE (COG0094)rpl5p (PRK04219)→<br>RpsN (COG0199)rps14P (PRK05766)→ rps8p (PRK04034)RpsH (COG0096)→<br>RplF (COG0097)rpl6p (PRK05518)→ PRK12766 (PRK12766)RecA (COG0468)Rpl32e (COG1717)→<br>rpl19e (PRK08570)Ribosomal_L19e (pfam01280)→
Hsp-4-1	NZ_AOLF01000024.1	-	14963	14864	**RNA**→ Methyltrn_RNA_3 (pfam02598)→ rpl3p (PRK04231)Ribosomal_L3 (pfam00297)→<br>RplD (COG0088)rpl4lp (PRK04042)→ RplW (COG0089)PRK14548 (PRK14548)→<br>RplB (COG0090)rpl2p (PRK09612)→ RpsS (COG0185)rps19p (PRK04038)→<br>rpl22p (PRK04223)RplV (COG0091)→ RpsC (COG0092)rps3p (PRK04191)→<br>PRK14549 (PRK14549)Ribosomal_L29 (pfam00831)→ PRK03879 (PRK03879)UPF0086 (pfam01868)→<br>RpsQ (COG0186)rps17p (PRK08572)→ rpl14p (PRK08571)Ribosomal_L14 (pfam00238)→<br>RplX (COG0198)rpl24p (PRK01191)→<br>PRK04313 (PRK04313)KOW_RPS4 (cd06087)Ribosomal_S4e (pfam00900)→ RplE (COG0094)rpl5p (PRK04219)→<br>RpsN (COG0199)rps14P (PRK05766)→ rps8p (PRK04034)RpsH (COG0096)→<br>RplF (COG0097)rpl6p (PRK05518)→ PRK12766 (PRK12766)RecA (COG0468)Rpl32e (COG1717)→<br>rpl19e (PRK08570)Ribosomal_L19e (pfam01280)→
Hsp-5-1	NZ_AOLE01000011.1	-	83691	83592	**RNA**→ Methyltrn_RNA_3 (pfam02598)→ rpl3p (PRK04231)Ribosomal_L3 (pfam00297)→<br>RplD (COG0088)rpl4lp (PRK04042)→ RplW (COG0089)PRK14548 (PRK14548)→<br>RplB (COG0090)rpl2p (PRK09612)→ RpsS (COG0185)rps19p (PRK04038)→<br>rpl22p (PRK04223)RplV (COG0091)→ RpsC (COG0092)rps3p (PRK04191)→<br>PRK14549 (PRK14549)Ribosomal_L29 (pfam00831)→ PRK03879 (PRK03879)UPF0086 (pfam01868)→<br>RpsQ (COG0186)rps17p (PRK08572)→ rpl14p (PRK08571)Ribosomal_L14 (pfam00238)→<br>RplX (COG0198)rpl24p (PRK01191)→<br>PRK04313 (PRK04313)KOW_RPS4 (cd06087)Ribosomal_S4e (pfam00900)→ RplE (COG0094)rpl5p (PRK04219)→<br>RpsN (COG0199)rps14P (PRK05766)→ rps8p (PRK04034)RpsH (COG0096)→<br>RplF (COG0097)rpl6p (PRK05518)→ PRK12766 (PRK12766)RecA (COG0468)Rpl32e (COG1717)→<br>rpl19e (PRK08570)Ribosomal_L19e (pfam01280)→
Hme-1-1	NZ_AOLO01000012.1	-	67999	67900	**RNA**→ Methyltrn_RNA_3 (pfam02598)→ rpl3p (PRK04231)Ribosomal_L3 (pfam00297)→<br>RplD (COG0088)rpl4lp (PRK04042)→ RplW (COG0089)PRK14548 (PRK14548)→<br>RplB (COG0090)rpl2p (PRK09612)→ RpsS (COG0185)rps19p (PRK04038)→<br>rpl22p (PRK04223)RplV (COG0091)→ RpsC (COG0092)rps3p (PRK04191)→<br>PRK14549 (PRK14549)Ribosomal_L29 (pfam00831)→ PRK03879 (PRK03879)UPF0086 (pfam01868)→<br>RpsQ (COG0186)rps17p (PRK08572)→ rpl14p (PRK08571)Ribosomal_L14 (pfam00238)→<br>RplX (COG0198)rpl24p (PRK01191)→<br>PRK04313 (PRK04313)KOW_RPS4 (cd06087)Ribosomal_S4e (pfam00900)→ RplE (COG0094)rpl5p (PRK04219)→<br>RpsN (COG0199)rps14P (PRK05766)→ rps8p (PRK04034)RpsH (COG0096)→<br>RplF (COG0097)rpl6p (PRK05518)→ PRK12766 (PRK12766)RecA (COG0468)Rpl32e (COG1717)→<br>rpl19e (PRK08570)Ribosomal_L19e (pfam01280)→
Hme-1-2	NC_017941.2	+	2068879	2068978	**RNA**→ Methyltrn_RNA_3 (pfam02598)→ rpl3p (PRK04231)Ribosomal_L3 (pfam00297)→<br>RplD (COG0088)rpl4lp (PRK04042)→ RplW (COG0089)PRK14548 (PRK14548)→<br>RplB (COG0090)rpl2p (PRK09612)→ RpsS (COG0185)rps19p (PRK04038)→<br>rpl22p (PRK04223)RplV (COG0091)→ RpsC (COG0092)rps3p (PRK04191)→<br>PRK14549 (PRK14549)Ribosomal_L29 (pfam00831)→ PRK03879 (PRK03879)UPF0086 (pfam01868)→<br>RpsQ (COG0186)rps17p (PRK08572)→ rpl14p (PRK08571)Ribosomal_L14 (pfam00238)→<br>RplX (COG0198)rpl24p (PRK01191)→<br>PRK04313 (PRK04313)KOW_RPS4 (cd06087)Ribosomal_S4e (pfam00900)→ RplE (COG0094)rpl5p (PRK04219)→<br>RpsN (COG0199)rps14P (PRK05766)→ rps8p (PRK04034)RpsH (COG0096)→<br>RplF (COG0097)rpl6p (PRK05518)→ PRK12766 (PRK12766)RecA (COG0468)Rpl32e (COG1717)→<br>rpl19e (PRK08570)Ribosomal_L19e (pfam01280)→

Hla-2-1	NZ_AOLI01000019.1	-	425010	424911	**RNA**→ Methyltrn_RNA_3 (pfam02598)→ rpl3p (PRK04231)Ribosomal_L3 (pfam00297)→<br>RplD (COG0088)rpl4lp (PRK04042)→ RplW (COG0089)PRK14548 (PRK14548)→<br>RplB (COG0090)rpl2p (PRK09612)→ RpsS (COG0185)rps19p (PRK04038)→<br>rpl22p (PRK04223)RplV (COG0091)→ RpsC (COG0092)rps3p (PRK04191)→<br>PRK14549 (PRK14549)Ribosomal_L29 (pfam00831)→ PRK03879 (PRK03879)UPF0086 (pfam01868)→<br>RpsQ (COG0186)rps17p (PRK08572)→ rpl14p (PRK08571)Ribosomal_L14 (pfam00238)→<br>RplX (COG0198)rpl24p (PRK01191)→<br>PRK04313 (PRK04313)KOW_RPS4 (cd06087)Ribosomal_S4e (pfam00900)→ RplE (COG0094)rpl5p (PRK04219)→<br>RpsN (COG0199)rps14P (PRK05766)→ rps8p (PRK04034)RpsH (COG0096)→<br>RplF (COG0097)rpl6p (PRK05518)→ PRK12766 (PRK12766)RecA (COG0468)Rpl32e (COG1717)→<br>rpl19e (PRK08570)Ribosomal_L19e (pfam01280)→
Hel-1-1	NZ_AOLK01000022.1	+	66470	66569	**RNA**→ Methyltrn_RNA_3 (pfam02598)→ RplC (COG0087)rpl3p (PRK04231)→<br>RplD (COG0088)rpl4lp (PRK04042)→ RplW (COG0089)PRK14548 (PRK14548)→<br>RplB (COG0090)rpl2p (PRK09612)→ RpsS (COG0185)rps19p (PRK04038)→<br>rpl22p (PRK04223)RplV (COG0091)→ RpsC (COG0092)rps3p (PRK04191)→<br>PRK14549 (PRK14549)Ribosomal_L29 (pfam00831)→ PRK03879 (PRK03879)UPF0086 (pfam01868)→<br>RpsQ (COG0186)rps17p (PRK08572)→ rpl14p (PRK08571)Ribosomal_L14 (pfam00238)→<br>RplX (COG0198)rpl24p (PRK01191)→<br>PRK04313 (PRK04313)KOW_RPS4 (cd06087)Ribosomal_S4e (pfam00900)→ RplE (COG0094)rpl5p (PRK04219)→<br>RpsN (COG0199)rps14P (PRK05766)→ rps8p (PRK04034)RpsH (COG0096)→<br>RplF (COG0097)rpl6p (PRK05518)→ PRK12766 (PRK12766)RecA (COG0468)Rpl32e (COG1717)→<br>rpl19e (PRK08570)Ribosomal_L19e (pfam01280)→
Hmu-1-1	NZ_AOLN01000013.1	-	37008	36909	**RNA**→ Methyltrn_RNA_3 (pfam02598)→ rpl3p (PRK04231)Ribosomal_L3 (pfam00297)→<br>RplD (COG0088)rpl4lp (PRK04042)→ RplW (COG0089)PRK14548 (PRK14548)→<br>RplB (COG0090)rpl2p (PRK09612)→ RpsS (COG0185)rps19p (PRK04038)→<br>rpl22p (PRK04223)RplV (COG0091)→ RpsC (COG0092)rps3p (PRK04191)→<br>PRK14549 (PRK14549)Ribosomal_L29 (pfam00831)→ PRK03879 (PRK03879)UPF0086 (pfam01868)→<br>RpsQ (COG0186)rps17p (PRK08572)→ rpl14p (PRK08571)Ribosomal_L14 (pfam00238)→<br>RplX (COG0198)rpl24p (PRK01191)→<br>PRK04313 (PRK04313)KOW_RPS4 (cd06087)Ribosomal_S4e (pfam00900)→ RplE (COG0094)rpl5p (PRK04219)→<br>RpsN (COG0199)rps14P (PRK05766)→ rps8p (PRK04034)RpsH (COG0096)→<br>RplF (COG0097)rpl6p (PRK05518)→ PRK12766 (PRK12766)RecA (COG0468)Rpl32e (COG1717)→<br>rpl19e (PRK08570)Ribosomal_L19e (pfam01280)→
env-79	SL_4KL_010_BRINE_10099275	-	413	314	**RNA**→ Methyltrn_RNA_3 (pfam02598)→
env-80	SL_4KL_010_BRINE_10236927	-	339	240	**RNA**→ Methyltrn_RNA_3 (pfam02598)→
env-81	SL_4KL_010_BRINE_10017268	+	119	218	**RNA**→ Methyltrn_RNA_3 (pfam02598)→ RplC (COG0087)rpl3p (PRK04231)→<br>RplD (COG0088)rpl4lp (PRK04042)→ RplW (COG0089)PRK14548 (PRK14548)→<br>RplB (COG0090)rpl2p (PRK09612)→ RpsS (COG0185)rps19p (PRK04038)→<br>rpl22p (PRK04223)RplV (COG0091)→
env-82	SL_4KL_010_BRINE_10000094	-	9413	9314	**RNA**→ Methyltrn_RNA_3 (pfam02598)→ RplC (COG0087)rpl3p (PRK04231)→<br>RplD (COG0088)rpl4lp (PRK04042)→ RplW (COG0089)PRK14548 (PRK14548)→<br>RplB (COG0090)rpl2p (PRK09612)→ RpsS (COG0185)rps19p (PRK04038)→<br>rpl22p (PRK04223)RplV (COG0091)→ RpsC (COG0092)rps3p (PRK04191)→<br>PRK14549 (PRK14549)Ribosomal_L29_HIP (cd00427)→ PRK03879 (PRK03879)UPF0086 (pfam01868)→<br>RpsQ (COG0186)rps17p (PRK08572)→ rpl14p (PRK08571)Ribosomal_L14 (pfam00238)→<br>RplX (COG0198)rpl24p (PRK01191)→<br>PRK04313 (PRK04313)S4 (cd00165)KOW_RPS4 (cd06087)Ribosomal_S4e (pfam00900)→<br>RplE (COG0094)rpl5p (PRK04219)→ RpsN (COG0199)rps14P (PRK05766)→<br>rps8p (PRK04034)RpsH (COG0096)→ RplF (COG0097)rpl6p (PRK05518)→<br>COG2251 (COG2251)HHH_5 (pfam14520)→
env-83	SL_4KL_010_BRINE_10065590	+	860	959	**RNA**→ Methyltrn_RNA_3 (pfam02598)→ rpl3p (PRK04231)Ribosomal_L3 (pfam00297)→

Hla-4-1	NZ_CP007055.1	+	1979362	1979461	**RNA**→ Methyltrn_RNA_3 (pfam02598)→ rpl3p (PRK04231)Ribosomal_L3 (pfam00297)→
					RplD (COG0088)rpl4lp (PRK04042)→ RplW (COG0089)PRK14548 (PRK14548)→
					RplB (COG0090)rpl2p (PRK09612)→ RpsS (COG0185)rps19p (PRK04038)→
					rpl22p (PRK04223)RplV (COG0091)→ RpsC (COG0092)rps3p (PRK04191)→
					PRK14549 (PRK14549)Ribosomal_L29 (pfam00831)→ PRK03879 (PRK03879)UPF0086 (pfam01868)→
					RpsQ (COG0186)rps17p (PRK08572)→ rpl14p (PRK08571)Ribosomal_L14 (pfam00238)→
					rpl24p (PRK01191)KOW_RPL26 (cd06089)→
					PRK04313 (PRK04313)KOW_RPS4 (cd06087)Ribosomal_S4e (pfam00900)→ RplE (COG0094)rpl5p (PRK04219)→
					RpsN (COG0199)rps14P (PRK05766)→ rps8p (PRK04034)RpsH (COG0096)→
					RplF (COG0097)rpl6p (PRK05518)→
					RecA (COG0468)Rpl32e (COG1717)rpl32e (PRK08562)HHH_5 (pfam14520)→
Ssp-1-1	NC_021313.1	+	2429676	2429775	**RNA**→ Methyltrn_RNA_3 (pfam02598)→ RplC (COG0087)rpl3p (PRK04231)→
					RplD (COG0088)rpl4lp (PRK04042)→ RplW (COG0089)PRK14548 (PRK14548)→
					RplB (COG0090)rpl2p (PRK09612)→ RpsS (COG0185)rps19p (PRK04038)→
					rpl22p (PRK04223)RplV (COG0091)→ RpsC (COG0092)rps3p (PRK04191)→
					PRK14549 (PRK14549)Ribosomal_L29 (pfam00831)→ PRK03879 (PRK03879)UPF0086 (pfam01868)→ hypo→
					RpsQ (COG0186)rps17p (PRK08572)→ rpl14p (PRK08571)Ribosomal_L14 (pfam00238)→
					RplX (COG0198)rpl24p (PRK01191)→
					PRK04313 (PRK04313)KOW_RPS4 (cd06087)S4 (pfam01479)Ribosomal_S4e (pfam00900)→
					RplE (COG0094)rpl5p (PRK04219)→ RpsN (COG0199)rps14P (PRK05766)→
					rps8p (PRK04034)RpsH (COG0096)→ RplF (COG0097)rpl6p (PRK05518)→
					UvrC (COG0322)Rpl32e (COG1717)rpl32e (PRK08562)HHH_5 (pfam14520)→
					Rpl32e (COG1717)rpl32e (PRK08562)HHH_5 (pfam14520)→
env-84	APHM01017236.1	-	204	105	**RNA**→ MTH1 (COG2106)→
env-85	LTJ07As871_J07ABscf096273	-	204	105	**RNA**→ MTH1 (COG2106)→
env-86	SL_4KL_010_BRINE_10002302	+	5048	5147	**RNA**→ Methyltrn_RNA_3 (pfam02598)→ rpl3p (PRK04231)Ribosomal_L3 (pfam00297)→
					RplD (COG0088)rpl4lp (PRK04042)→ RplW (COG0089)PRK14548 (PRK14548)→
					RplB (COG0090)rpl2p (PRK09612)→ RpsS (COG0185)rps19p (PRK04038)→
					rpl22p (PRK04223)RplV (COG0091)→ RpsC (COG0092)rps3p (PRK04191)→
					RpmC (COG0255)PRK14549 (PRK14549)→ PRK03879 (PRK03879)UPF0086 (pfam01868)→
Hsp-11-1	NZ_JMIP02000014.1	-	158997	158898	**RNA**→ Methyltrn_RNA_3 (pfam02598)→ RplC (COG0087)rpl3p (PRK04231)→ hypo→
					RplD (COG0088)rpl4lp (PRK04042)→ RplW (COG0089)PRK14548 (PRK14548)→ hypo→ hypo→
					RpsS (COG0185)rps19p (PRK04038)→ rpl22p (PRK04223)RplV (COG0091)→
					RpsC (COG0092)rps3p (PRK04191)→ PRK14549 (PRK14549)Ribosomal_L29 (pfam00831)→
					PRK03879 (PRK03879)UPF0086 (pfam01868)→ RpsQ (COG0186)rps17p (PRK08572)→
					rpl14p (PRK08571)Ribosomal_L14 (pfam00238)→ hypo→ rpl24p (PRK01191)KOW_RPL26 (cd06089)→
					PRK04313 (PRK04313)KOW_RPS4 (cd06087)Ribosomal_S4e (pfam00900)→ RplE (COG0094)rpl5p (PRK04219)→
					RpsN (COG0199)rps14P (PRK05766)→ rps8p (PRK04034)RpsH (COG0096)→
					RplF (COG0097)rpl6p (PRK05518)→ PRK12766 (PRK12766)UvrC (COG0322)Rpl32e (COG1717)→
env-87	GSLNARP_GFPJP1N02HFKWJ	-	439	343	**RNA**→ PRK12442 (PRK12442)Methyltrn_RNA_3 (pfam02598)→
env-88	SL_4KL_010_BRINE_10091194	+	755	857	**RNA**→ Methyltrn_RNA_3 (pfam02598)→
env-89	SL_4KL_010_BRINE_10095573	-	111	9	**RNA**→ MTH1 (COG2106)→
Hka-1-1	NZ_KB905379.1	+	273922	274024	**RNA**→ Methyltrn_RNA_3 (pfam02598)→ rpl3p (PRK04231)Ribosomal_L3 (pfam00297)→
					RplD (COG0088)rpl4lp (PRK04042)→ RplW (COG0089)PRK14548 (PRK14548)→
					RplB (COG0090)rpl2p (PRK09612)→ RpsS (COG0185)rps19p (PRK04038)→
					rpl22p (PRK04223)RplV (COG0091)→ RpsC (COG0092)rps3p (PRK04191)→
					PRK14549 (PRK14549)Ribosomal_L29 (pfam00831)→
Hmu-2-1	NC_013202.1	+	1747213	1747315	**RNA**→ Methyltrn_RNA_3 (pfam02598)→ rpl3p (PRK04231)Ribosomal_L3 (pfam00297)→
					RplD (COG0088)rpl4lp (PRK04042)→ RplW (COG0089)PRK14548 (PRK14548)→
					RplB (COG0090)rpl2p (PRK09612)→ RpsS (COG0185)rps19p (PRK04038)→
					rpl22p (PRK04223)RplV (COG0091)→ RpsC (COG0092)rps3p (PRK04191)→
					PRK14549 (PRK14549)Ribosomal_L29 (pfam00831)→

Hca-3-1	NZ_AOIU01000034.1	-	38153	38054	**RNA**→ Methyltrn_RNA_3 (pfam02598)→ rpl3p (PRK04231)Ribosomal_L3 (pfam00297)→<br>RplD (COG0088)rpl4lp (PRK04042)→ PRK14548 (PRK14548)Ribosomal_L23 (pfam00276)→<br>RplB (COG0090)rpl2p (PRK09612)→ RpsS (COG0185)rps19p (PRK04038)→<br>rpl22p (PRK04223)RplV (COG0091)→ RpsC (COG0092)rps3p (PRK04191)→<br>PRK14549 (PRK14549)Ribosomal_L29 (pfam00831)→ PRK03879 (PRK03879)UPF0086 (pfam01868)→<br>RpsQ (COG0186)rps17p (PRK08572)→ rpl14p (PRK08571)Ribosomal_L14 (pfam00238)→<br>RplX (COG0198)rpl24p (PRK01191)→<br>PRK04313 (PRK04313)KOW_RPS4 (cd06087)S4 (pfam01479)Ribosomal_S4e (pfam00900)→<br>RplE (COG0094)rpl5p (PRK04219)→ RpsN (COG0199)rps14P (PRK05766)→<br>rps8p (PRK04034)RpsH (COG0096)→ RplF (COG0097)rpl6p (PRK05518)→<br>Rpl32e (COG1717)rpl32e (PRK08562)HHH_5 (pfam14520)→ rpl19e (PRK08570)Ribosomal_L19e (pfam01280)→
Hsa-5-1	NZ_AOME01000079.1	-	8539	8440	**RNA**→ Methyltrn_RNA_3 (pfam02598)→ RplC (COG0087)rpl3p (PRK04231)→<br>RplD (COG0088)rpl4lp (PRK04042)→ RplW (COG0089)PRK14548 (PRK14548)→<br>RplB (COG0090)rpl2p (PRK09612)→ RpsS (COG0185)rps19p (PRK04038)→<br>rpl22p (PRK04223)RplV (COG0091)→ RpsC (COG0092)rps3p (PRK04191)HHH_5 (pfam14520)→<br>PRK14549 (PRK14549)Ribosomal_L29 (pfam00831)→ PRK03879 (PRK03879)UPF0086 (pfam01868)→<br>RpsQ (COG0186)rps17p (PRK08572)→ rpl14p (PRK08571)Ribosomal_L14 (pfam00238)→<br>RplX (COG0198)rpl24p (PRK01191)→<br>PRK04313 (PRK04313)S4 (cd00165)KOW_RPS4 (cd06087)Ribosomal_S4e (pfam00900)→<br>RplE (COG0094)rpl5p (PRK04219)→ hypo→ hypo→ RpsN (COG0199)rps14P (PRK05766)→
env-90	GQKUMWE01CQSAM	+	397	496	**RNA**→ MTH1 (COG2106)→
Hag-1-1	NZ_BAFM01000007.1	+	1659	1758	**RNA**→ Methyltrn_RNA_3 (pfam02598)→ RplC (COG0087)rpl3p (PRK04231)→<br>RplD (COG0088)rpl4lp (PRK04042)→ RplW (COG0089)PRK14548 (PRK14548)→<br>RplB (COG0090)rpl2p (PRK09612)→ RpsS (COG0185)rps19p (PRK04038)→<br>rpl22p (PRK04223)RplV (COG0091)→<br>RpsC (COG0092)UvrC (COG0322)rps3p (PRK04191)HHH_5 (pfam14520)→<br>PRK14549 (PRK14549)Ribosomal_L29 (pfam00831)→ PRK03879 (PRK03879)UPF0086 (pfam01868)→<br>RpsQ (COG0186)rps17p (PRK08572)→ rpl14p (PRK08571)Ribosomal_L14 (pfam00238)→<br>RplX (COG0198)rpl24p (PRK01191)→<br>PRK04313 (PRK04313)S4 (cd00165)KOW_RPS4 (cd06087)Ribosomal_S4e (pfam00900)→<br>RplE (COG0094)rpl5p (PRK04219)→ RpsN (COG0199)rps14P (PRK05766)→<br>rps8p (PRK04034)RpsH (COG0096)→ RplF (COG0097)rpl6p (PRK05518)→<br>RecA (COG0468)Rpl32e (COG1717)rpl32e (PRK08562)HHH_5 (pfam14520)→<br>rpl19e (PRK08570)Ribosomal_L19e (pfam01280)→ RplR (COG0256)rpl18p (PRK08569)→<br>RpsE (COG0098)rps5p (PRK04044)→ Ribosomal_L7_archeal_euk (cd01657)rpl30p (PRK06049)→<br>rpl15p (PRK06419)Ribosomal_L27A (pfam00828)→ SecY (COG0201)PRK08568 (PRK08568)→
Hsa-4-1	NZ_AOMD01000025.1	+	140368	140467	**RNA**→ Methyltrn_RNA_3 (pfam02598)→ RplC (COG0087)rpl3p (PRK04231)→<br>RplD (COG0088)rpl4lp (PRK04042)→ RplW (COG0089)PRK14548 (PRK14548)→<br>RplB (COG0090)rpl2p (PRK09612)→ RpsS (COG0185)rps19p (PRK04038)→<br>rpl22p (PRK04223)RplV (COG0091)→ RpsC (COG0092)rps3p (PRK04191)HHH_5 (pfam14520)→<br>PRK14549 (PRK14549)Ribosomal_L29 (pfam00831)→ PRK03879 (PRK03879)UPF0086 (pfam01868)→<br>RpsQ (COG0186)rps17p (PRK08572)→ rpl14p (PRK08571)Ribosomal_L14 (pfam00238)→<br>rpl24p (PRK01191)KOW_RPL26 (cd06089)→<br>PRK04313 (PRK04313)S4 (cd00165)KOW_RPS4 (cd06087)Ribosomal_S4e (pfam00900)→<br>RplE (COG0094)rpl5p (PRK04219)→ RpsN (COG0199)rps14P (PRK05766)→<br>rps8p (PRK04034)RpsH (COG0096)→ RplF (COG0097)rpl6p (PRK05518)→<br>RecA (COG0468)Rpl32e (COG1717)COG2251 (COG2251)rpl32e (PRK08562)HHH_5 (pfam14520)→
env-91	GSLNARP_contig29074	-	461	359	**RNA**→ Methyltrn_RNA_3 (pfam02598)→
env-92	P_1C_Liq_1_UnCty_1017446	+	565	667	**RNA**→ MTH1 (COG2106)→
env-93	P_1C_Liq_3_UnCty_1022117	-	224	122	**RNA**→ Methyltrn_RNA_3 (pfam02598)→
env-94	P_1C_Sed_2_UnCty_1128195	-	252	150	**RNA**→ Methyltrn_RNA_3 (pfam02598)→
env-95	P_1C_Liq_2_UnCty_1026371	-	311	209	**RNA**→ Methyltrn_RNA_3 (pfam02598)→
env-96	77515515	+	477	579	**RNA**→ MTH1 (COG2106)→
env-97	FLAS_READ_83860922	+	477	579	**RNA**→ MTH1 (COG2106)→
env-98	P_A23_Liq_3_Fmt_1016381	-	364	262	**RNA**→ MTH1 (COG2106)→

env-99	APHM01021628.1	+	4390	4492	**RNA**→ Methyltrn_RNA_3 (pfam02598)→ RplC (COG0087)rpl3p (PRK04231)→<br>RplD (COG0088)rpl4lp (PRK04042)→ RplW (COG0089)PRK14548 (PRK14548)→<br>RplB (COG0090)rpl2p (PRK09612)→ RpsS (COG0185)rps19p (PRK04038)→<br>rpl22p (PRK04223)RplV (COG0091)→ RpsC (COG0092)rps3p (PRK04191)→
env-100	LTJ07As871_J07ABscf098869	+	456526	456628	**RNA**→ Methyltrn_RNA_3 (pfam02598)→ RplC (COG0087)rpl3p (PRK04231)→<br>RplD (COG0088)rpl4lp (PRK04042)→ RplW (COG0089)PRK14548 (PRK14548)→<br>RplB (COG0090)rpl2p (PRK09612)→ RpsS (COG0185)rps19p (PRK04038)→<br>rpl22p (PRK04223)RplV (COG0091)→ RpsC (COG0092)rps3p (PRK04191)→
env-101	P_1C_Sed_2_UnCty_1030622	+	624	726	**RNA**→ MTH1 (COG2106)→
env-102	SL_5KL_010_BRINE_10180849	-	369	267	**RNA**→ PRK10575 (PRK10575)Methyltrn_RNA_3 (pfam02598)→
env-103	SL_6KL_011_BRINE_1082566	-	592	490	**RNA**→ PRK10575 (PRK10575)Methyltrn_RNA_3 (pfam02598)→
env-104	SL_8KL_010_SED_10233400	+	212	314	**RNA**→ Methyltrn_RNA_3 (pfam02598)→
env-105	SL_4KL_010_BRINE_10134291	+	227	329	**RNA**→ Methyltrn_RNA_3 (pfam02598)→
env-106	SL_5KL_010_BRINE_10114435	+	29	131	**RNA**→ Methyltrn_RNA_3 (pfam02598)→
env-107	P_1C_Sed_2_UnCty_1330058	+	56	155	**RNA**→ Methyltrn_RNA_3 (pfam02598)→
env-108	SL_4KL_010_BRINE_10687755	+	77	176	**RNA**→ Methyltrn_RNA_3 (pfam02598)→
env-109	JXWU01090489.1	+	605	701	**RNA**→ Methyltrn_RNA_3 (pfam02598)→
CHa-1-1	NZ_ASGZ01000021.1	+	7594	7693	**RNA**→ Methyltrn_RNA_3 (pfam02598)→ RplC (COG0087)rpl3p (PRK04231)→<br>RplD (COG0088)rpl4lp (PRK04042)→ RplW (COG0089)PRK14548 (PRK14548)→<br>RplB (COG0090)rpl2p (PRK09612)→ RpsS (COG0185)rps19p (PRK04038)→<br>rpl22p (PRK04223)RplV (COG0091)→ RpsC (COG0092)rps3p (PRK04191)→<br>PRK14549 (PRK14549)Ribosomal_L29 (pfam00831)→ PRK03879 (PRK03879)UPF0086 (pfam01868)→<br>RpsQ (COG0186)rps17p (PRK08572)→ rpl14p (PRK08571)Ribosomal_L14 (pfam00238)→<br>RplX (COG0198)rpl24p (PRK01191)→ PRK04313 (PRK04313)Ribosomal_S4e (pfam00900)→<br>RplE (COG0094)rpl5p (PRK04219)→ RpsN (COG0199)rps14P (PRK05766)→<br>rps8p (PRK04034)RpsH (COG0096)→ RplF (COG0097)rpl6p (PRK05518)→<br>PRK12766 (PRK12766)RecA (COG0468)Rpl32e (COG1717)→ rpl19e (PRK08570)Ribosomal_L19e (pfam01280)→<br>RplR (COG0256)rpl18p (PRK08569)→ RpsE (COG0098)rps5p (PRK04044)→<br>RpsE (COG0098)rps5p (PRK04044)→ Ribosomal_L7_archeal_euk (cd01657)rpl30p (PRK06049)→<br>rpl15p (PRK06419)Ribosomal_L27A (pfam00828)→
Hpa-2-1	NZ_AOIV01000008.1	-	240007	239908	**RNA**→ Methyltrn_RNA_3 (pfam02598)→ RplC (COG0087)rpl3p (PRK04231)→<br>RplD (COG0088)rpl4lp (PRK04042)→ RplW (COG0089)PRK14548 (PRK14548)→<br>RplB (COG0090)rpl2p (PRK09612)→ RpsS (COG0185)rps19p (PRK04038)→<br>rpl22p (PRK04223)RplV (COG0091)→ RpsC (COG0092)rps3p (PRK04191)→<br>PRK14549 (PRK14549)Ribosomal_L29 (pfam00831)→ PRK03879 (PRK03879)UPF0086 (pfam01868)→<br>RpsQ (COG0186)rps17p (PRK08572)→ rpl14p (PRK08571)Ribosomal_L14 (pfam00238)→<br>RplX (COG0198)rpl24p (PRK01191)→<br>PRK04313 (PRK04313)KOW_RPS4 (cd06087)Ribosomal_S4e (pfam00900)→ RplE (COG0094)rpl5p (PRK04219)→<br>RpsN (COG0199)rps14P (PRK05766)→ rps8p (PRK04034)RpsH (COG0096)→<br>RplF (COG0097)rpl6p (PRK05518)→ PRK12766 (PRK12766)RecA (COG0468)Rpl32e (COG1717)→<br>rpl19e (PRK08570)Ribosomal_L19e (pfam01280)→
env-110	SL_5KL_010_BRINE_10000579	-	6143	6044	**RNA**→ Methyltrn_RNA_3 (pfam02598)→ RplC (COG0087)rpl3p (PRK04231)→<br>RplD (COG0088)rpl4lp (PRK04042)→ RplW (COG0089)PRK14548 (PRK14548)→<br>RplB (COG0090)rpl2p (PRK09612)→ RpsS (COG0185)rps19p (PRK04038)→<br>rpl22p (PRK04223)RplV (COG0091)→ RpsC (COG0092)rps3p (PRK04191)→<br>RpmC (COG0255)PRK14549 (PRK14549)→ hypo→ PRK03879 (PRK03879)UPF0086 (pfam01868)→<br>RpsQ (COG0186)rps17p (PRK08572)→
env-111	SL_4KL_010_BRINE_10006116	-	6219	6120	**RNA**→ Methyltrn_RNA_3 (pfam02598)→ RplC (COG0087)rpl3p (PRK04231)→<br>RplD (COG0088)rpl4lp (PRK04042)→ RplW (COG0089)PRK14548 (PRK14548)→<br>RplB (COG0090)rpl2p (PRK09612)→ RpsS (COG0185)rps19p (PRK04038)→<br>rpl22p (PRK04223)RplV (COG0091)→ RpsC (COG0092)rps3p (PRK04191)→<br>RpmC (COG0255)PRK14549 (PRK14549)→ hypo→ PRK03879 (PRK03879)UPF0086 (pfam01868)→<br>RpsQ (COG0186)rps17p (PRK08572)→
env-112	SL_4KL_010_BRINE_10037372	+	1564	1663	**RNA**→ Methyltrn_RNA_3 (pfam02598)→ rpl3p (PRK04231)Ribosomal_L3 (pfam00297)→

Nhu-1-1	NZ_AOIM01000008.1	+	9157	9256	**RNA**→ Methyltrn_RNA_3 (pfam02598)→ rpl3p (PRK04231)Ribosomal_L3 (pfam00297)→ RplD (COG0088)rpl4lp (PRK04042)→ RplW (COG0089)PRK14548 (PRK14548)→ RplB (COG0090)rpl2p (PRK09612)→ RpsS (COG0185)rps19p (PRK04038)→ rpl22p (PRK04223)RplV (COG0091)→ RpsC (COG0092)rps3p (PRK04191)→ RpmC (COG0255)PRK14549 (PRK14549)→ PRK03879 (PRK03879)UPF0086 (pfam01868)→ RpsQ (COG0186)rps17p (PRK08572)→ rpl14p (PRK08571)Ribosomal_L14 (pfam00238)→ rpl24p (PRK01191)KOW_RPL26 (cd06089)→ PRK04313 (PRK04313)S4 (pfam01479)→ hypo→
env-113	P_A23_Liq_1_Fmt_1001862	-	2728	2626	**RNA**→ MTH1 (COG2106)→ RplC (COG0087)rpl3p (PRK04231)→ RplD (COG0088)rpl4lp (PRK04042)→ RplW (COG0089)PRK14548 (PRK14548)→ RplB (COG0090)rpl2p (PRK09612)→
env-114	P_A23_Liq_1_Fmt_1006312	-	589	487	**RNA**→ Methyltrn_RNA_3 (pfam02598)→
env-115	P_A23_Liq_2_Fmt_1001778	-	147	45	**RNA**→ MTH1 (COG2106)→
env-116	SL_5KL_010_BRINE_10182593	-	355	253	**RNA**→ PRK10575 (PRK10575)Methyltrn_RNA_3 (pfam02598)→
env-117	SL_6KL_011_BRINE_1122878	+	59	161	**RNA**→ Methyltrn_RNA_3 (pfam02598)→
env-118	SL_5KL_010_SED_10176812	-	343	241	**RNA**→ Methyltrn_RNA_3 (pfam02598)→
env-119	GSLSAAI_GFP0ZZ301BFHUD	+	260	362	**RNA**→ Methyltrn_RNA_3 (pfam02598)→
env-120	APHM01000442.1	+	2818	2917	**RNA**→ Methyltrn_RNA_3 (pfam02598)→ RplC (COG0087)rpl3p (PRK04231)→ RplD (COG0088)rpl4lp (PRK04042)→ RplW (COG0089)PRK14548 (PRK14548)→ RplB (COG0090)rpl2p (PRK09612)→ rps19p (PRK04038)→
env-121	LTJ07As871_J07ABscf098878	-	600442	600343	**RNA**→ Methyltrn_RNA_3 (pfam02598)→ RplC (COG0087)rpl3p (PRK04231)→ RplD (COG0088)rpl4lp (PRK04042)→ RplW (COG0089)PRK14548 (PRK14548)→ RplB (COG0090)rpl2p (PRK09612)→
env-122	JXWS01030537.1	+	838	937	**RNA**→ Methyltrn_RNA_3 (pfam02598)→
env-123	P_A23_Liq_1_Fmt_1000580	+	837	936	**RNA**→ Methyltrn_RNA_3 (pfam02598)→ RplC (COG0087)rpl3p (PRK04231)→ RplD (COG0088)rpl4lp (PRK04042)→ RplW (COG0089)PRK14548 (PRK14548)→ RplB (COG0090)rpl2p (PRK09612)→ RpsS (COG0185)rps19p (PRK04038)→ rpl22p (PRK04223)RplV (COG0091)→ RpsC (COG0092)rps3p (PRK04191)→ PRK14549 (PRK14549)Ribosomal_L29 (pfam00831)→ PRK03879 (PRK03879)UPF0086 (pfam01868)→
env-124	P_A23_Liq_3_Fmt_1000785	-	1067	968	**RNA**→ Methyltrn_RNA_3 (pfam02598)→ rpl3p (PRK04231)Ribosomal_L3 (pfam00297)→
env-125	P_A23_Liq_2_Fmt_1000789	-	1096	997	**RNA**→ Methyltrn_RNA_3 (pfam02598)→ rpl3p (PRK04231)Ribosomal_L3 (pfam00297)→
env-126	GSLNARP_GFPJP1N02GVYAL	+	71	170	**RNA**→ MTH1 (COG2106)→
env-127	P_1C_Liq_2_UnCty_1129324	+	141	240	**RNA**→ MTH1 (COG2106)→
env-128	JXWS01088076.1	+	222	321	**RNA**→ MTH1 (COG2106)→
env-129	SS_3KL_010_SOIL_10082725	-	438	339	**RNA**→ Methyltrn_RNA_3 (pfam02598)→
Hna-1-1	NZ_KE386573.1	-	1301517	1301418	**RNA**→ Methyltrn_RNA_3 (pfam02598)→ rpl3p (PRK04231)Ribosomal_L3 (pfam00297)→ RplD (COG0088)rpl4lp (PRK04042)→ RplW (COG0089)PRK14548 (PRK14548)→ RplB (COG0090)rpl2p (PRK09612)→ RpsS (COG0185)rps19p (PRK04038)→ rpl22p (PRK04223)RplV (COG0091)→ RpsC (COG0092)rps3p (PRK04191)→ PRK14549 (PRK14549)Ribosomal_L29 (pfam00831)→ PRK03879 (PRK03879)UPF0086 (pfam01868)→ RpsQ (COG0186)rps17p (PRK08572)→ rpl14p (PRK08571)Ribosomal_L14 (pfam00238)→ RplX (COG0198)rpl24p (PRK01191)→ PRK04313 (PRK04313)KOW_RPS4 (cd06087)Ribosomal_S4e (pfam00900)→ RplE (COG0094)rpl5p (PRK04219)→ RpsN (COG0199)rps14P (PRK05766)→ rps8p (PRK04034)RpsH (COG0096)→ RplF (COG0097)rpl6p (PRK05518)→ PRK12766 (PRK12766)RecA (COG0468)Rpl32e (COG1717)→ rpl19e (PRK08570)Ribosomal_L19e (pfam01280)→
env-130	P_1C_Sed_2_UnCty_1009263	-	1341	1242	**RNA**→ Methyltrn_RNA_3 (pfam02598)→ RplC (COG0087)rpl3p (PRK04231)→
env-131	ADL20m3uS_contig11573	-	6008	5909	**RNA**→ Methyltrn_RNA_3 (pfam02598)→ rpl3p (PRK04231)Ribosomal_L3 (pfam00297)→ RplC (COG0087)rpl3p (PRK04231)→ RplD (COG0088)rpl4lp (PRK04042)→ RplW (COG0089)PRK14548 (PRK14548)→ RplB (COG0090)rpl2p (PRK09612)→ RpsS (COG0185)rps19p (PRK04038)→ rpl22p (PRK04223)RplV (COG0091)→ RpsC (COG0092)rps3p (PRK04191)→ PRK14549 (PRK14549)Ribosomal_L29 (pfam00831)→ PRK03879 (PRK03879)UPF0086 (pfam01868)→

env-132	2140918017-125	-	28013	27914	**RNA**→ Methyltrn_RNA_3 (pfam02598)→ rpl3p (PRK04231)Ribosomal_L3 (pfam00297)→ RplC (COG0087)rpl3p (PRK04231)→ RplD (COG0088)rpl4lp (PRK04042)→ RplW (COG0089)PRK14548 (PRK14548)→ RplB (COG0090)rpl2p (PRK09612)→ RpsS (COG0185)rps19p (PRK04038)→ rpl22p (PRK04223)RplV (COG0091)→ RpsC (COG0092)rps3p (PRK04191)→ PRK14549 (PRK14549)Ribosomal_L29 (pfam00831)→ PRK03879 (PRK03879)UPF0086 (pfam01868)→ RpsQ (COG0186)rps17p (PRK08572)→ rpl14p (PRK08571)Ribosomal_L14 (pfam00238)→ rpl24p (PRK01191)KOW_RPL26 (cd06089)→ PRK04313 (PRK04313)KOW_RPS4 (cd06087)Ribosomal_S4e (pfam00900)→ rpl5p (PRK04219)Ribosomal_L5 (pfam00281)→ RpsN (COG0199)rps14P (PRK05766)→ rps8p (PRK04034)RpsH (COG0096)→ RplF (COG0097)rpl6p (PRK05518)→ PRK12766 (PRK12766)RecA (COG0468)Rpl32e (COG1717)→ rpl19e (PRK08570)Ribosomal_L19e (pfam01280)→
env-133	2140918027-00843	+	673	772	**RNA**→ Methyltrn_RNA_3 (pfam02598)→ rpl3p (PRK04231)Ribosomal_L3 (pfam00297)→ RplC (COG0087)rpl3p (PRK04231)→ RplD (COG0088)rpl4lp (PRK04042)→ RplW (COG0089)PRK14548 (PRK14548)→ RplB (COG0090)rpl2p (PRK09612)→ RpsS (COG0185)rps19p (PRK04038)→ rpl22p (PRK04223)RplV (COG0091)→ RpsC (COG0092)rps3p (PRK04191)→ PRK14549 (PRK14549)Ribosomal_L29 (pfam00831)→ PRK03879 (PRK03879)UPF0086 (pfam01868)→
har-1-1	NC_015954.1	-	1701293	1701194	**RNA**→ Methyltrn_RNA_3 (pfam02598)→ RplC (COG0087)rpl3p (PRK04231)→ RplD (COG0088)rpl4lp (PRK04042)→ RplW (COG0089)PRK14548 (PRK14548)→ RplB (COG0090)rpl2p (PRK09612)→ RpsS (COG0185)rps19p (PRK04038)→ rpl22p (PRK04223)RplV (COG0091)→ RpsC (COG0092)rps3p (PRK04191)→ PRK14549 (PRK14549)Ribosomal_L29 (pfam00831)→ PRK03879 (PRK03879)UPF0086 (pfam01868)→ RpsQ (COG0186)rps17p (PRK08572)→ rpl14p (PRK08571)Ribosomal_L14 (pfam00238)→ rpl24p (PRK01191)KOW_RPL26 (cd06089)→ PRK04313 (PRK04313)KOW_RPS4 (cd06087)Ribosomal_S4e (pfam00900)→ rpl5p (PRK04219)Ribosomal_L5 (pfam00281)→ RpsN (COG0199)rps14P (PRK05766)→ rps8p (PRK04034)RpsH (COG0096)→ RplF (COG0097)rpl6p (PRK05518)→ PRK12766 (PRK12766)RecA (COG0468)Rpl32e (COG1717)→ rpl19e (PRK08570)Ribosomal_L19e (pfam01280)→
env-134	SS_3KL_010_SOIL_10099808	-	212	113	**RNA**→ Methyltrn_RNA_3 (pfam02598)→
env-135	2140918017-82247	+	63	164	**RNA**→
Nph-1-1	NC_007426.1	+	2369313	2369412	**RNA**→ Methyltrn_RNA_3 (pfam02598)→ rpl3p (PRK04231)Ribosomal_L3 (pfam00297)→ RplD (COG0088)rpl4lp (PRK04042)→ RplW (COG0089)PRK14548 (PRK14548)→ RplB (COG0090)rpl2p (PRK09612)→ RpsS (COG0185)rps19p (PRK04038)→ rpl22p (PRK04223)RplV (COG0091)→ RpsC (COG0092)rps3p (PRK04191)→ RpmC (COG0255)PRK14549 (PRK14549)→ PRK03879 (PRK03879)UPF0086 (pfam01868)→ hypo→ RpsQ (COG0186)rps17p (PRK08572)→ rpl14p (PRK08571)Ribosomal_L14 (pfam00238)→ RplX (COG0198)rpl24p (PRK01191)→ PRK04313 (PRK04313)KOW_RPS4 (cd06087)Ribosomal_S4e (pfam00900)→ RplE (COG0094)rpl5p (PRK04219)→ RpsN (COG0199)rps14P (PRK05766)→ rps8p (PRK04034)RpsH (COG0096)→ RplF (COG0097)rpl6p (PRK05518)→ NT_POLXc (cd00141)PRK12766 (PRK12766)Rpl32e (COG1717)→ rpl19e (PRK08570)Ribosomal_L19e (pfam01280)→ RplR (COG0256)rpl18p (PRK08569)→
env-136	SL_4KL_010_BRINE_10016891	-	2655	2556	**RNA**→ Methyltrn_RNA_3 (pfam02598)→ RplC (COG0087)rpl3p (PRK04231)→ RplD (COG0088)rpl4lp (PRK04042)→ RplW (COG0089)PRK14548 (PRK14548)→ RplB (COG0090)rpl2p (PRK09612)→

env-137	SL_6KL_011_BRINE_1000523	+	4060	4159	**RNA**→ Methyltrn_RNA_3 (pfam02598)→ RplC (COG0087)rpl3p (PRK04231)→<br>RplD (COG0088)rpl4lp (PRK04042)→ RplW (COG0089)PRK14548 (PRK14548)→<br>RplB (COG0090)rpl2p (PRK09612)→ RpsS (COG0185)rps19p (PRK04038)→<br>rpl22p (PRK04223)RplV (COG0091)→ RpsC (COG0092)rps3p (PRK04191)→<br>RpmC (COG0255)PRK14549 (PRK14549)→ PRK03879 (PRK03879)UPF0086 (pfam01868)→<br>RpsQ (COG0186)rps17p (PRK08572)→ rpl14p (PRK08571)Ribosomal_L14 (pfam00238)→<br>RplX (COG0198)rpl24p (PRK01191)→<br>PRK04313 (PRK04313)KOW_RPS4 (cd06087)Ribosomal_S4e (pfam00900)→ RplE (COG0094)rpl5p (PRK04219)→<br>RpsN (COG0199)rps14P (PRK05766)→ rps8p (PRK04034)RpsH (COG0096)→<br>RplF (COG0097)rpl6p (PRK05518)→ PRK12766 (PRK12766)RecA (COG0468)Rpl32e (COG1717)→<br>rpl19e (PRK08570)Ribosomal_L19e (pfam01280)→ RplR (COG0256)rpl18p (PRK08569)→<br>RpsE (COG0098)rps5p (PRK04044)→ Ribosomal_L7_archeal_euk (cd01657)rpl30p (PRK06049)→<br>rpl15p (PRK06419)Ribosomal_L27A (pfam00828)→ SecY (COG0201)PRK08568 (PRK08568)→
env-138	P_1C_Liq_1_UnCty_1007975	+	1341	1443	**RNA**→ Methyltrn_RNA_3 (pfam02598)→
env-139	SL_6KL_011_BRINE_1092333	+	64	166	**RNA**→ Methyltrn_RNA_3 (pfam02598)→
env-140	SL_5KL_010_BRINE_10000145	-	14973	14871	**RNA**→ MTH1 (COG2106)→ rpl3p (PRK04231)Ribosomal_L3 (pfam00297)→<br>RplD (COG0088)rpl4lp (PRK04042)→ RplW (COG0089)PRK14548 (PRK14548)→<br>RplB (COG0090)rpl2p (PRK09612)→ RpsS (COG0185)rps19p (PRK04038)→<br>rpl22p (PRK04223)RplV (COG0091)→ RpsC (COG0092)rps3p (PRK04191)→<br>PRK14549 (PRK14549)Ribosomal_L29_HIP (cd00427)→ PRK03879 (PRK03879)UPF0086 (pfam01868)→<br>RpsQ (COG0186)rps17p (PRK08572)→ rpl14p (PRK08571)Ribosomal_L14 (pfam00238)→<br>rpl24p (PRK01191)KOW_RPL26 (cd06089)→<br>PRK04313 (PRK04313)KOW_RPS4 (cd06087)Ribosomal_S4e (pfam00900)→ RplE (COG0094)rpl5p (PRK04219)→<br>RpsN (COG0199)rps14P (PRK05766)→ rps8p (PRK04034)RpsH (COG0096)→<br>RplF (COG0097)rpl6p (PRK05518)→ PRK12766 (PRK12766)RecA (COG0468)Rpl32e (COG1717)→<br>rpl19e (PRK08570)Ribosomal_L19e (pfam01280)→
env-141	SL_6KL_011_BRINE_1010384	+	87	189	**RNA**→ Methyltrn_RNA_3 (pfam02598)→ rpl3p (PRK04231)Ribosomal_L3 (pfam00297)→<br>RplD (COG0088)rpl4lp (PRK04042)→ RplW (COG0089)PRK14548 (PRK14548)→<br>RplB (COG0090)rpl2p (PRK09612)→ RpsS (COG0185)rps19p (PRK04038)→<br>rpl22p (PRK04223)RplV (COG0091)→
env-142	SL_5KL_010_SED_10000993	-	17753	17651	**RNA**→ Methyltrn_RNA_3 (pfam02598)→ rpl3p (PRK04231)Ribosomal_L3 (pfam00297)→<br>RplD (COG0088)rpl4lp (PRK04042)→ RplW (COG0089)PRK14548 (PRK14548)→<br>RplB (COG0090)rpl2p (PRK09612)→ RpsS (COG0185)rps19p (PRK04038)→<br>rpl22p (PRK04223)RplV (COG0091)→ RpsC (COG0092)rps3p (PRK04191)→<br>PRK14549 (PRK14549)Ribosomal_L29_HIP (cd00427)→ PRK03879 (PRK03879)UPF0086 (pfam01868)→<br>RpsQ (COG0186)rps17p (PRK08572)→ rpl14p (PRK08571)Ribosomal_L14 (pfam00238)→<br>rpl24p (PRK01191)KOW_RPL26 (cd06089)→<br>PRK04313 (PRK04313)KOW_RPS4 (cd06087)Ribosomal_S4e (pfam00900)→ RplE (COG0094)rpl5p (PRK04219)→<br>RpsN (COG0199)rps14P (PRK05766)→ rps8p (PRK04034)RpsH (COG0096)→<br>RplF (COG0097)rpl6p (PRK05518)→ PRK12766 (PRK12766)RecA (COG0468)Rpl32e (COG1717)→<br>rpl19e (PRK08570)Ribosomal_L19e (pfam01280)→
env-143	SL_8KL_010_SED_10049127	-	1844	1742	**RNA**→ Methyltrn_RNA_3 (pfam02598)→ rpl3p (PRK04231)Ribosomal_L3 (pfam00297)→<br>RplD (COG0088)rpl4lp (PRK04042)→
env-144	SL_4KL_010_BRINE_10002885	-	1066	964	**RNA**→ MTH1 (COG2106)→ rpl3p (PRK04231)Ribosomal_L3 (pfam00297)→
env-145	SL_6KL_011_BRINE_1040731	-	290	188	**RNA**→ Methyltrn_RNA_3 (pfam02598)→
env-146	SL_6KL_011_BRINE_1090778	-	540	438	**RNA**→ Methyltrn_RNA_3 (pfam02598)→
env-147	SL_6KL_011_BRINE_1020969	+	1942	2044	**RNA**→ MTH1 (COG2106)→
env-148	SL_6KL_011_BRINE_1001075	-	545	443	**RNA**→ Methyltrn_RNA_3 (pfam02598)→
Hsp-9-1	NZ_CBVY010000043.1	+	1088	1190	**RNA**→ MTH1 (COG2106)→ RplC (COG0087)rpl3p (PRK04231)→

Hez-1-1	NZ_ATXS01000002.1	+	57125	57227	**RNA**→ MTH1 (COG2106)→ RplC (COG0087)rpl3p (PRK04231)→ RplD (COG0088)rpl4lp (PRK04042)→ RplW (COG0089)PRK14548 (PRK14548)→ RplB (COG0090)rpl2p (PRK09612)→ RpsS (COG0185)rps19p (PRK04038)→ rpl22p (PRK04223)RplV (COG0091)→ RpsC (COG0092)rps3p (PRK04191)→ RpmC (COG0255)PRK14549 (PRK14549)→ PRK03879 (PRK03879)UPF0086 (pfam01868)→ RpsQ (COG0186)rps17p (PRK08572)→ rpl14p (PRK08571)Ribosomal_L14 (pfam00238)→ rpl24p (PRK01191)KOW_RPL26 (cd06089)→ PRK04313 (PRK04313)KOW_RPS4 (cd06087)Ribosomal_S4e (pfam00900)→ RplE (COG0094)rpl5p (PRK04219)→ RpsN (COG0199)rps14P (PRK05766)→ rps8p (PRK04034)RpsH (COG0096)→ RplF (COG0097)rpl6p (PRK05518)→ PRK12766 (PRK12766)RecA (COG0468)Rpl32e (COG1717)→ rpl19e (PRK08570)Ribosomal_L19e (pfam01280)→
env-149	P_1C_Liq_1_UnCty_1110322	+	40	142	**RNA**→ Methyltrn_RNA_3 (pfam02598)→
env-150	P_1C_Liq_2_UnCty_1085242	-	257	155	**RNA**→ Methyltrn_RNA_3 (pfam02598)→
env-151	P_1C_Sed_2_UnCty_1018040	-	1354	1252	**RNA**→ Methyltrn_RNA_3 (pfam02598)→ RplC (COG0087)rpl3p (PRK04231)→
Hco-1-1	NZ_AOJL01000060.1	-	167010	166908	**RNA**→ MTH1 (COG2106)→ RplC (COG0087)rpl3p (PRK04231)→ RplD (COG0088)rpl4lp (PRK04042)→ RplW (COG0089)PRK14548 (PRK14548)→ RplB (COG0090)rpl2p (PRK09612)→ RplB (COG0090)rpl2p (PRK09612)→ RpsS (COG0185)rps19p (PRK04038)→ rpl22p (PRK04223)RplV (COG0091)→ RpsC (COG0092)rps3p (PRK04191)→ RpmC (COG0255)PRK14549 (PRK14549)→ PRK03879 (PRK03879)UPF0086 (pfam01868)→ RpsQ (COG0186)rps17p (PRK08572)→ rpl14p (PRK08571)Ribosomal_L14 (pfam00238)→ rpl24p (PRK01191)KOW_RPL26 (cd06089)→ PRK04313 (PRK04313)KOW_RPS4 (cd06087)Ribosomal_S4e (pfam00900)→ rpl5p (PRK04219)Ribosomal_L5 (pfam00281)Ribosomal_L5_C (pfam00673)→ hypo→ RpsN (COG0199)rps14P (PRK05766)→ rps8p (PRK04034)RpsH (COG0096)→ RplF (COG0097)rpl6p (PRK05518)→ PRK12766 (PRK12766)RecA (COG0468)Rpl32e (COG1717)→ rpl19e (PRK08570)Ribosomal_L19e (pfam01280)→
env-152	P_1C_Liq_3_UnCty_1007582	-	296	194	**RNA**→ MTH1 (COG2106)→
env-153	P_A23_Liq_1_Fmt_1009395	-	369	267	**RNA**→ MTH1 (COG2106)→
env-154	P_A23_Liq_2_Fmt_1002028	+	19	121	**RNA**→ Methyltrn_RNA_3 (pfam02598)→ RplC (COG0087)rpl3p (PRK04231)→ RplD (COG0088)rpl4lp (PRK04042)→ RplW (COG0089)PRK14548 (PRK14548)→ RplB (COG0090)rpl2p (PRK09612)→ RpsS (COG0185)rps19p (PRK04038)→
env-155	P_A23_Liq_2_Fmt_1023865	+	29	131	**RNA**→ Methyltrn_RNA_3 (pfam02598)→
env-156	P_A23_Liq_3_Fmt_1023393	+	38	140	**RNA**→ Methyltrn_RNA_3 (pfam02598)→
Hsp-10-1	NZ_JQKV01000002.1	-	421137	421035	**RNA**→ Methyltrn_RNA_3 (pfam02598)→ RplC (COG0087)rpl3p (PRK04231)→ RplD (COG0088)rpl4lp (PRK04042)→ RplW (COG0089)PRK14548 (PRK14548)→ RplB (COG0090)rpl2p (PRK09612)→ RpsS (COG0185)rps19p (PRK04038)→ rpl22p (PRK04223)RplV (COG0091)→ RpsC (COG0092)rps3p (PRK04191)→ PRK14549 (PRK14549)Ribosomal_L29_HIP (cd00427)→ PRK03879 (PRK03879)UPF0086 (pfam01868)→ RpsQ (COG0186)rps17p (PRK08572)→ rpl14p (PRK08571)Ribosomal_L14 (pfam00238)→ RplX (COG0198)rpl24p (PRK01191)→ PRK04313 (PRK04313)KOW_RPS4 (cd06087)Ribosomal_S4e (pfam00900)→ RplE (COG0094)rpl5p (PRK04219)→ RpsN (COG0199)rps14P (PRK05766)→ rps8p (PRK04034)RpsH (COG0096)→ RplF (COG0097)rpl6p (PRK05518)→ PRK12766 (PRK12766)RecA (COG0468)Rpl32e (COG1717)→ rpl19e (PRK08570)Ribosomal_L19e (pfam01280)→
Hli-1-1	NZ_AOJG01000040.1	-	82466	82364	**RNA**→ MTH1 (COG2106)→ rpl3p (PRK04231)Ribosomal_L3 (pfam00297)→ RplD (COG0088)rpl4lp (PRK04042)→ RplW (COG0089)PRK14548 (PRK14548)→ RplB (COG0090)rpl2p (PRK09612)→ RpsS (COG0185)rps19p (PRK04038)→ rpl22p (PRK04223)RplV (COG0091)→ RpsC (COG0092)rps3p (PRK04191)→ PRK14549 (PRK14549)Ribosomal_L29_HIP (cd00427)→ PRK03879 (PRK03879)UPF0086 (pfam01868)→ RpsQ (COG0186)rps17p (PRK08572)→ rpl14p (PRK08571)Ribosomal_L14 (pfam00238)→ rpl24p (PRK01191)KOW_RPL26 (cd06089)→ PRK04313 (PRK04313)Ribosomal_S4e (pfam00900)→ RplE (COG0094)rpl5p (PRK04219)→ RpsN (COG0199)rps14P (PRK05766)→ rps8p (PRK04034)RpsH (COG0096)→ RplF (COG0097)rpl6p (PRK05518)→ PRK12766 (PRK12766)RecA (COG0468)Rpl32e (COG1717)→ rpl19e (PRK08570)Ribosomal_L19e (pfam01280)→

Hte-1-1	NZ_AOJD01000064.1	+	2215	2317	**RNA**→ MTH1 (COG2106)→ RplC (COG0087)rpl3p (PRK04231)→ RplD (COG0088)rpl4lp (PRK04042)→ RplW (COG0089)PRK14548 (PRK14548)→ RplB (COG0090)rpl2p (PRK09612)→ RplB (COG0090)rpl2p (PRK09612)→ RpsS (COG0185)rps19p (PRK04038)→ rpl22p (PRK04223)RplV (COG0091)→ RpsC (COG0092)rps3p (PRK04191)→ PRK14549 (PRK14549)Ribosomal_L29_HIP (cd00427)→ PRK03879 (PRK03879)UPF0086 (pfam01868)→ RpsQ (COG0186)rps17p (PRK08572)→ rpl14p (PRK08571)Ribosomal_L14 (pfam00238)→ rpl24p (PRK01191)KOW_RPL26 (cd06089)→ PRK04313 (PRK04313)KOW_RPS4 (cd06087)Ribosomal_S4e (pfam00900)→ RplE (COG0094)rpl5p (PRK04219)→ RpsN (COG0199)rps14P (PRK05766)→ rps8p (PRK04034)RpsH (COG0096)→ RplF (COG0097)rpl6p (PRK05518)→ PRK12766 (PRK12766)RecA (COG0468)Rpl32e (COG1717)→ rpl19e (PRK08570)Ribosomal_L19e (pfam01280)→ RplR (COG0256)rpl18p (PRK08569)→ RpsE (COG0098)rps5p (PRK04044)→ Ribosomal_L7_archeal_euk (cd01657)rpl30p (PRK06049)→ rpl15p (PRK06419)Ribosomal_L27A (pfam00828)→ SecY (COG0201)PRK08568 (PRK08568)→
Hko-1-1	NZ_AOJH01000027.1	+	94307	94409	**RNA**→ Methyltrn_RNA_3 (pfam02598)→ rpl3p (PRK04231)Ribosomal_L3 (pfam00297)→ RplD (COG0088)rpl4lp (PRK04042)→ RplW (COG0089)PRK14548 (PRK14548)→ RplB (COG0090)rpl2p (PRK09612)→ RpsS (COG0185)rps19p (PRK04038)→ rpl22p (PRK04223)RplV (COG0091)→ RpsC (COG0092)rps3p (PRK04191)→ PRK14549 (PRK14549)Ribosomal_L29_HIP (cd00427)→ PRK03879 (PRK03879)UPF0086 (pfam01868)→ RpsQ (COG0186)rps17p (PRK08572)→ rpl14p (PRK08571)Ribosomal_L14 (pfam00238)→ rpl24p (PRK01191)KOW_RPL26 (cd06089)→ PRK04313 (PRK04313)Ribosomal_S4e (pfam00900)→ RplE (COG0094)rpl5p (PRK04219)→ RpsN (COG0199)rps14P (PRK05766)→ rps8p (PRK04034)RpsH (COG0096)→ RplF (COG0097)rpl6p (PRK05518)→ PRK12766 (PRK12766)RecA (COG0468)Rpl32e (COG1717)→ rpl19e (PRK08570)Ribosomal_L19e (pfam01280)→
Hli-2-1	NZ_AOJF01000042.1	+	2248	2350	**RNA**→ MTH1 (COG2106)→ RplC (COG0087)rpl3p (PRK04231)→ RplD (COG0088)rpl4lp (PRK04042)→ RplW (COG0089)PRK14548 (PRK14548)→ RplB (COG0090)rpl2p (PRK09612)→ RplB (COG0090)rpl2p (PRK09612)→ RpsS (COG0185)rps19p (PRK04038)→ rpl22p (PRK04223)RplV (COG0091)→ RpsC (COG0092)rps3p (PRK04191)→ RpmC (COG0255)PRK14549 (PRK14549)→ PRK03879 (PRK03879)UPF0086 (pfam01868)→ RpsQ (COG0186)rps17p (PRK08572)→ rpl14p (PRK08571)Ribosomal_L14 (pfam00238)→ rpl24p (PRK01191)KOW_RPL26 (cd06089)→ hypo→ PRK04313 (PRK04313)rpl5p (PRK04219)Ribosomal_L5 (pfam00281)Ribosomal_L5_C (pfam00673)Ribosomal_S4e (pfam0090[illegible] rpl5p (PRK04219)Ribosomal_L5 (pfam00281)Ribosomal_L5_C (pfam00673)→ RpsN (COG0199)rps14P (PRK05766)→ rps8p (PRK04034)RpsH (COG0096)→ RplF (COG0097)rpl6p (PRK05518)→ PRK12766 (PRK12766)RecA (COG0468)Rpl32e (COG1717)→ rpl19e (PRK08570)Ribosomal_L19e (pfam01280)→ RplR (COG0256)rpl18p (PRK08569)→ RpsE (COG0098)rps5p (PRK04044)→ Ribosomal_L7_archeal_euk (cd01657)rpl30p (PRK06049)→ rpl15p (PRK06419)Ribosomal_L27A (pfam00828)→ SecY (COG0201)PRK08568 (PRK08568)→
Hsa-7-1	NZ_AOJE01000020.1	-	44999	44897	**RNA**→ MTH1 (COG2106)→ rpl3p (PRK04231)Ribosomal_L3 (pfam00297)→ RplD (COG0088)rpl4lp (PRK04042)→ RplW (COG0089)PRK14548 (PRK14548)→ RplB (COG0090)rpl2p (PRK09612)→ RpsS (COG0185)rps19p (PRK04038)→ rpl22p (PRK04223)RplV (COG0091)→ RpsC (COG0092)rps3p (PRK04191)→ PRK14549 (PRK14549)Ribosomal_L29_HIP (cd00427)→ PRK03879 (PRK03879)UPF0086 (pfam01868)→ RpsQ (COG0186)rps17p (PRK08572)→ rpl14p (PRK08571)Ribosomal_L14 (pfam00238)→ rpl24p (PRK01191)KOW_RPL26 (cd06089)→ PRK04313 (PRK04313)Ribosomal_S4e (pfam00900)→ RplE (COG0094)rpl5p (PRK04219)→ RpsN (COG0199)rps14P (PRK05766)→ rps8p (PRK04034)RpsH (COG0096)→ RplF (COG0097)rpl6p (PRK05518)→ PRK12766 (PRK12766)RecA (COG0468)Rpl32e (COG1717)→ rpl19e (PRK08570)Ribosomal_L19e (pfam01280)→
Hca-2-1	NZ_AOJK01000017.1	+	53669	53771	**RNA**→ MTH1 (COG2106)→ RplC (COG0087)rpl3p (PRK04231)→ RplD (COG0088)rpl4lp (PRK04042)→ RplW (COG0089)PRK14548 (PRK14548)→ RplB (COG0090)rpl2p (PRK09612)→ RplB (COG0090)rpl2p (PRK09612)→ RpsS (COG0185)rps19p (PRK04038)→ rpl22p (PRK04223)RplV (COG0091)→ RpsC (COG0092)rps3p (PRK04191)→ RpmC (COG0255)PRK14549 (PRK14549)→ PRK03879 (PRK03879)UPF0086 (pfam01868)→ RpsQ (COG0186)rps17p (PRK08572)→ rpl14p (PRK08571)Ribosomal_L14 (pfam00238)→ rpl24p (PRK01191)KOW_RPL26 (cd06089)→ PRK04313 (PRK04313)Ribosomal_S4e (pfam00900)→

Hsp-7-1	NZ_JH815330.1	+	360566	360668	**RNA**→ MTH1 (COG2106)→ rpl3p (PRK04231)Ribosomal_L3 (pfam00297)→<br>RplC (COG0087)rpl3p (PRK04231)→ RplC (COG0087)rpl3p (PRK04231)→<br>RplD (COG0088)rpl4lp (PRK04042)→ RplW (COG0089)PRK14548 (PRK14548)→<br>RplB (COG0090)rpl2p (PRK09612)→ RplB (COG0090)rpl2p (PRK09612)→<br>RpsS (COG0185)rps19p (PRK04038)→ rpl22p (PRK04223)RplV (COG0091)→<br>RpsC (COG0092)rps3p (PRK04191)→ RpmC (COG0255)PRK14549 (PRK14549)→<br>PRK03879 (PRK03879)UPF0086 (pfam01868)→ RpsQ (COG0186)rps17p (PRK08572)→<br>rpl14p (PRK08571)Ribosomal_L14 (pfam00238)→ rpl24p (PRK01191)KOW_RPL26 (cd06089)→<br>PRK04313 (PRK04313)KOW_RPS4 (cd06087)Ribosomal_S4e (pfam00900)→ RplE (COG0094)rpl5p (PRK04219)→<br>RpsN (COG0199)rps14P (PRK05766)→ rps8p (PRK04034)RpsH (COG0096)→<br>RplF (COG0097)rpl6p (PRK05518)→ PRK12766 (PRK12766)RecA (COG0468)Rpl32e (COG1717)→<br>rpl19e (PRK08570)Ribosomal_L19e (pfam01280)→
Hha-2-1	NZ_BBJP01000034.1	+	23045	23147	**RNA**→ MTH1 (COG2106)→ rpl3p (PRK04231)Ribosomal_L3 (pfam00297)→<br>RplD (COG0088)rpl4lp (PRK04042)→ RplW (COG0089)PRK14548 (PRK14548)→<br>RplB (COG0090)rpl2p (PRK09612)→ RpsS (COG0185)rps19p (PRK04038)→<br>rpl22p (PRK04223)RplV (COG0091)→ RpsC (COG0092)rps3p (PRK04191)→<br>PRK14549 (PRK14549)Ribosomal_L29_HIP (cd00427)→ PRK03879 (PRK03879)UPF0086 (pfam01868)→<br>RpsQ (COG0186)rps17p (PRK08572)→ rpl14p (PRK08571)Ribosomal_L14 (pfam00238)→<br>rpl24p (PRK01191)KOW_RPL26 (cd06089)→ PRK04313 (PRK04313)Ribosomal_S4e (pfam00900)→<br>RplE (COG0094)rpl5p (PRK04219)→ RpsN (COG0199)rps14P (PRK05766)→<br>rps8p (PRK04034)RpsH (COG0096)→ RplF (COG0097)rpl6p (PRK05518)→<br>PRK12766 (PRK12766)RecA (COG0468)Rpl32e (COG1717)→ rpl19e (PRK08570)Ribosomal_L19e (pfam01280)→
Hdi-1-1	NZ_AOJN01000013.1	+	82475	82577	**RNA**→ MTH1 (COG2106)→ RplC (COG0087)rpl3p (PRK04231)→ RplD (COG0088)rpl4lp (PRK04042)→<br>RplW (COG0089)PRK14548 (PRK14548)→ RplB (COG0090)rpl2p (PRK09612)→<br>RplB (COG0090)rpl2p (PRK09612)→ RpsS (COG0185)rps19p (PRK04038)→<br>rpl22p (PRK04223)RplV (COG0091)→ RpsC (COG0092)rps3p (PRK04191)→<br>RpmC (COG0255)PRK14549 (PRK14549)→ PRK03879 (PRK03879)UPF0086 (pfam01868)→<br>RpsQ (COG0186)rps17p (PRK08572)→ rpl14p (PRK08571)Ribosomal_L14 (pfam00238)→<br>rpl24p (PRK01191)KOW_RPL26 (cd06089)→<br>PRK04313 (PRK04313)KOW_RPS4 (cd06087)Ribosomal_S4e (pfam00900)→ RplE (COG0094)rpl5p (PRK04219)→<br>RpsN (COG0199)rps14P (PRK05766)→ rps8p (PRK04034)RpsH (COG0096)→<br>RplF (COG0097)rpl6p (PRK05518)→ PRK12766 (PRK12766)RecA (COG0468)Rpl32e (COG1717)→<br>rpl19e (PRK08570)Ribosomal_L19e (pfam01280)→
Hdi-2-1	NZ_AOJM01000049.1	-	26024	25922	**RNA**→ MTH1 (COG2106)→ RplC (COG0087)rpl3p (PRK04231)→ RplD (COG0088)rpl4lp (PRK04042)→<br>RplW (COG0089)PRK14548 (PRK14548)→ RplB (COG0090)rpl2p (PRK09612)→<br>RplB (COG0090)rpl2p (PRK09612)→ RpsS (COG0185)rps19p (PRK04038)→<br>rpl22p (PRK04223)RplV (COG0091)→ RpsC (COG0092)rps3p (PRK04191)→<br>RpmC (COG0255)PRK14549 (PRK14549)→ PRK03879 (PRK03879)UPF0086 (pfam01868)→<br>RpsQ (COG0186)rps17p (PRK08572)→ rpl14p (PRK08571)Ribosomal_L14 (pfam00238)→<br>rpl24p (PRK01191)KOW_RPL26 (cd06089)→<br>PRK04313 (PRK04313)KOW_RPS4 (cd06087)Ribosomal_S4e (pfam00900)→<br>rpl5p (PRK04219)Ribosomal_L5 (pfam00281)Ribosomal_L5_C (pfam00673)→<br>RpsN (COG0199)rps14P (PRK05766)→ rps8p (PRK04034)RpsH (COG0096)→<br>RplF (COG0097)rpl6p (PRK05518)→ PRK12766 (PRK12766)RecA (COG0468)Rpl32e (COG1717)→<br>rpl19e (PRK08570)Ribosomal_L19e (pfam01280)→

Hsa-9-1	NZ_JNFH02000042.1	+	4162	4264	**RNA**→ hypo→ hypo→ rpl3p (PRK04231)Ribosomal_L3 (pfam00297)→ RplD (COG0088)rpl4lp (PRK04042)→<br>RplW (COG0089)PRK14548 (PRK14548)→ RplB (COG0090)rpl2p (PRK09612)→<br>RpsS (COG0185)rps19p (PRK04038)→ rpl22p (PRK04223)RplV (COG0091)→ hypo→<br>RpsC (COG0092)rps3p (PRK04191)→ PRK14549 (PRK14549)Ribosomal_L29_HIP (cd00427)→<br>PRK03879 (PRK03879)UPF0086 (pfam01868)→ RpsQ (COG0186)rps17p (PRK08572)→<br>rpl14p (PRK08571)Ribosomal_L14 (pfam00238)→ rpl24p (PRK01191)KOW_RPL26 (cd06089)→<br>PRK04313 (PRK04313)Ribosomal_S4e (pfam00900)→ RplE (COG0094)rpl5p (PRK04219)→<br>RpsN (COG0199)rps14P (PRK05766)→ rps8p (PRK04034)RpsH (COG0096)→<br>RplF (COG0097)rpl6p (PRK05518)→ PRK12766 (PRK12766)RecA (COG0468)Rpl32e (COG1717)→<br>rpl19e (PRK08570)Ribosomal_L19e (pfam01280)→ RplR (COG0256)rpl18p (PRK08569)→<br>RpsE (COG0098)rps5p (PRK04044)→ Ribosomal_L7_archeal_euk (cd01657)rpl30p (PRK06049)→<br>rpl15p (PRK06419)Ribosomal_L27A (pfam00828)→ SecY (COG0201)PRK08568 (PRK08568)→
Har-2-1	NZ_AOJJ01000075.1	+	39843	39945	**RNA**→ MTH1 (COG2106)→ RplC (COG0087)rpl3p (PRK04231)→ RplD (COG0088)rpl4lp (PRK04042)→<br>RplW (COG0089)PRK14548 (PRK14548)→ RplB (COG0090)rpl2p (PRK09612)→<br>RplB (COG0090)rpl2p (PRK09612)→ RpsS (COG0185)rps19p (PRK04038)→<br>rpl22p (PRK04223)RplV (COG0091)→ RpsC (COG0092)rps3p (PRK04191)→<br>RpmC (COG0255)PRK14549 (PRK14549)→ PRK03879 (PRK03879)UPF0086 (pfam01868)→<br>RpsQ (COG0186)rps17p (PRK08572)→ rpl14p (PRK08571)Ribosomal_L14 (pfam00238)→<br>rpl24p (PRK01191)KOW_RPL26 (cd06089)→<br>PRK04313 (PRK04313)KOW_RPS4 (cd06087)Ribosomal_S4e (pfam00900)→ RplE (COG0094)rpl5p (PRK04219)→<br>RpsN (COG0199)rps14P (PRK05766)→ rps8p (PRK04034)RpsH (COG0096)→<br>RplF (COG0097)rpl6p (PRK05518)→ PRK12766 (PRK12766)RecA (COG0468)Rpl32e (COG1717)→<br>rpl19e (PRK08570)Ribosomal_L19e (pfam01280)→
Hte-2-1	NZ_AOIW01000054.1	-	29402	29300	**RNA**→ MTH1 (COG2106)→ RplC (COG0087)rpl3p (PRK04231)→ RplD (COG0088)rpl4lp (PRK04042)→<br>RplW (COG0089)PRK14548 (PRK14548)→ RplB (COG0090)rpl2p (PRK09612)→<br>RplB (COG0090)rpl2p (PRK09612)→ RpsS (COG0185)rps19p (PRK04038)→<br>rpl22p (PRK04223)RplV (COG0091)→ RpsC (COG0092)rps3p (PRK04191)→<br>RpmC (COG0255)PRK14549 (PRK14549)→ PRK03879 (PRK03879)UPF0086 (pfam01868)→<br>RpsQ (COG0186)rps17p (PRK08572)→ rpl14p (PRK08571)Ribosomal_L14 (pfam00238)→<br>rpl24p (PRK01191)KOW_RPL26 (cd06089)→<br>PRK04313 (PRK04313)KOW_RPS4 (cd06087)Ribosomal_S4e (pfam00900)→ RplE (COG0094)rpl5p (PRK04219)→<br>RpsN (COG0199)rps14P (PRK05766)→ rps8p (PRK04034)RpsH (COG0096)→<br>RplF (COG0097)rpl6p (PRK05518)→ PRK12766 (PRK12766)RecA (COG0468)Rpl32e (COG1717)→<br>rpl19e (PRK08570)Ribosomal_L19e (pfam01280)→
env-157	P_1C_Liq_2_UnCty_1073497	-	265	163	**RNA**→ Methyltrn_RNA_3 (pfam02598)→
env-158	SL_6KL_011_BRINE_1032700	-	1548	1446	**RNA**→ Methyltrn_RNA_3 (pfam02598)→ RplC (COG0087)rpl3p (PRK04231)→
env-159	SL_4KL_010_BRINE_10001141	+	89	191	**RNA**→ MTH1 (COG2106)→ RplC (COG0087)rpl3p (PRK04231)→ RplD (COG0088)rpl4lp (PRK04042)→<br>RplW (COG0089)PRK14548 (PRK14548)→ RplB (COG0090)rpl2p (PRK09612)→<br>RpsS (COG0185)rps19p (PRK04038)→ rpl22p (PRK04223)RplV (COG0091)→<br>RpsC (COG0092)rps3p (PRK04191)→ RpmC (COG0255)PRK14549 (PRK14549)→<br>PRK03879 (PRK03879)UPF0086 (pfam01868)→ RpsQ (COG0186)rps17p (PRK08572)→<br>rpl14p (PRK08571)Ribosomal_L14 (pfam00238)→ rpl24p (PRK01191)KOW_RPL26 (cd06089)→<br>PRK04313 (PRK04313)KOW_RPS4 (cd06087)Ribosomal_S4e (pfam00900)→ RplE (COG0094)rpl5p (PRK04219)→<br>RpsN (COG0199)rps14P (PRK05766)→ rps8p (PRK04034)RpsH (COG0096)→<br>RplF (COG0097)rpl6p (PRK05518)→ PRK12766 (PRK12766)RecA (COG0468)Rpl32e (COG1717)→<br>rpl19e (PRK08570)Ribosomal_L19e (pfam01280)→ RplR (COG0256)rpl18p (PRK08569)→<br>RpsE (COG0098)rps5p (PRK04044)→ Ribosomal_L7_archeal_euk (cd01657)rpl30p (PRK06049)→<br>rpl15p (PRK06419)Ribosomal_L27A (pfam00828)→ SecY (COG0201)PRK08568 (PRK08568)→ hypo→
env-160	SL_6KL_011_BRINE_1001616	-	387	285	**RNA**→ MTH1 (COG2106)→

h3A-1-1	NZ_KK033115.1	+	666108	666210	**RNA**→ Methyltrn_RNA_3 (pfam02598)→ rpl3p (PRK04231)Ribosomal_L3 (pfam00297)→ RplD (COG0088)rpl4lp (PRK04042)→ RplW (COG0089)PRK14548 (PRK14548)→ RplB (COG0090)rpl2p (PRK09612)→ RpsS (COG0185)rps19p (PRK04038)→ rpl22p (PRK04223)RplV (COG0091)→ RpsC (COG0092)rps3p (PRK04191)→ PRK14549 (PRK14549)Ribosomal_L29_HIP (cd00427)→ PRK03879 (PRK03879)UPF0086 (pfam01868)→ RpsQ (COG0186)rps17p (PRK08572)→ rpl14p (PRK08571)Ribosomal_L14 (pfam00238)→ RplX (COG0198)rpl24p (PRK01191)→ PRK04313 (PRK04313)KOW_RPS4 (cd06087)Ribosomal_S4e (pfam00900)→ RplE (COG0094)rpl5p (PRK04219)→ RpsN (COG0199)rps14P (PRK05766)→ rps8p (PRK04034)RpsH (COG0096)→ RplF (COG0097)rpl6p (PRK05518)→ PRK12766 (PRK12766)RecA (COG0468)Rpl32e (COG1717)→ rpl19e (PRK08570)Ribosomal_L19e (pfam01280)→
Hai-1-1	NZ_AOJI01000026.1	-	211502	211400	**RNA**→ MTH1 (COG2106)→ RplC (COG0087)rpl3p (PRK04231)→ RplD (COG0088)rpl4lp (PRK04042)→ RplW (COG0089)PRK14548 (PRK14548)→ RplB (COG0090)rpl2p (PRK09612)→ RpsS (COG0185)rps19p (PRK04038)→ rpl22p (PRK04223)RplV (COG0091)→ RpsC (COG0092)rps3p (PRK04191)→ PRK14549 (PRK14549)Ribosomal_L29_HIP (cd00427)→ PRK03879 (PRK03879)UPF0086 (pfam01868)→ RpsQ (COG0186)rps17p (PRK08572)→ rpl14p (PRK08571)Ribosomal_L14 (pfam00238)→ RplX (COG0198)rpl24p (PRK01191)→ PRK04313 (PRK04313)KOW_RPS4 (cd06087)Ribosomal_S4e (pfam00900)→ RplE (COG0094)rpl5p (PRK04219)→ RpsN (COG0199)rps14P (PRK05766)→ rps8p (PRK04034)RpsH (COG0096)→ RplF (COG0097)rpl6p (PRK05518)→ PRK12766 (PRK12766)RecA (COG0468)Rpl32e (COG1717)→ rpl19e (PRK08570)Ribosomal_L19e (pfam01280)→
env-161	ADL20m3uS_contig08402	-	1754	1652	**RNA**→ Methyltrn_RNA_3 (pfam02598)→ rpl3p (PRK04231)Ribosomal_L3 (pfam00297)→ RplC (COG0087)rpl3p (PRK04231)→ RplD (COG0088)rpl4lp (PRK04042)→
env-162	2140918017-4520	-	13975	13873	**RNA**→ Methyltrn_RNA_3 (pfam02598)→ rpl3p (PRK04231)Ribosomal_L3 (pfam00297)→ RplC (COG0087)rpl3p (PRK04231)→ RplD (COG0088)rpl4lp (PRK04042)→ RplW (COG0089)PRK14548 (PRK14548)→
env-163	2140918027-00057	-	14028	13926	**RNA**→ Methyltrn_RNA_3 (pfam02598)→ rpl3p (PRK04231)Ribosomal_L3 (pfam00297)→ RplC (COG0087)rpl3p (PRK04231)→ RplD (COG0088)rpl4lp (PRK04042)→ RplW (COG0089)PRK14548 (PRK14548)→ RplB (COG0090)rpl2p (PRK09612)→ RpsS (COG0185)rps19p (PRK04038)→ rpl22p (PRK04223)RplV (COG0091)→ RpsC (COG0092)rps3p (PRK04191)→ PRK14549 (PRK14549)Ribosomal_L29_HIP (cd00427)→ PRK03879 (PRK03879)UPF0086 (pfam01868)→ RpsQ (COG0186)rps17p (PRK08572)→ rpl14p (PRK08571)Ribosomal_L14 (pfam00238)→ rpl24p (PRK01191)KOW_RPL26 (cd06089)→ PRK04313 (PRK04313)Ribosomal_S4e (pfam00900)→ RplE (COG0094)rpl5p (PRK04219)→ RpsN (COG0199)rps14P (PRK05766)→ rps8p (PRK04034)RpsH (COG0096)→ RplF (COG0097)rpl6p (PRK05518)→ PRK12766 (PRK12766)RecA (COG0468)Rpl32e (COG1717)→ rpl19e (PRK08570)Ribosomal_L19e (pfam01280)→
Hla-3-1	NC_012029.1	-	2428716	2428614	**RNA**→ Methyltrn_RNA_3 (pfam02598)→ rpl3p (PRK04231)Ribosomal_L3 (pfam00297)→ RplD (COG0088)rpl4lp (PRK04042)→ RplW (COG0089)PRK14548 (PRK14548)→ RplB (COG0090)rpl2p (PRK09612)→ RpsS (COG0185)rps19p (PRK04038)→ rpl22p (PRK04223)RplV (COG0091)→ RpsC (COG0092)rps3p (PRK04191)→ PRK14549 (PRK14549)Ribosomal_L29_HIP (cd00427)→ PRK03879 (PRK03879)UPF0086 (pfam01868)→ RpsQ (COG0186)rps17p (PRK08572)→ rpl14p (PRK08571)Ribosomal_L14 (pfam00238)→ rpl24p (PRK01191)KOW_RPL26 (cd06089)→ PRK04313 (PRK04313)Ribosomal_S4e (pfam00900)→ RplE (COG0094)rpl5p (PRK04219)→ RpsN (COG0199)rps14P (PRK05766)→ rps8p (PRK04034)RpsH (COG0096)→ RplF (COG0097)rpl6p (PRK05518)→ PRK12766 (PRK12766)RecA (COG0468)Rpl32e (COG1717)→ rpl19e (PRK08570)Ribosomal_L19e (pfam01280)→

Hho-1-1	NZ_AOJO01000045.1	-	129551	129449	RNA→ MTH1 (COG2106)→ RplC (COG0087)rpl3p (PRK04231)→ RplD (COG0088)rpl4lp (PRK04042)→ RplW (COG0089)PRK14548 (PRK14548)→ RplB (COG0090)rpl2p (PRK09612)→ RpsS (COG0185)rps19p (PRK04038)→ rpl22p (PRK04223)RplV (COG0091)→ RpsC (COG0092)rps3p (PRK04191)→ PRK14549 (PRK14549)Ribosomal_L29_HIP (cd00427)→ PRK03879 (PRK03879)UPF0086 (pfam01868)→ RpsQ (COG0186)rps17p (PRK08572)→ rpl14p (PRK08571)Ribosomal_L14 (pfam00238)→ rpl24p (PRK01191)KOW_RPL26 (cd06089)→ PRK04313 (PRK04313)KOW_RPS4 (cd06087)Ribosomal_S4e (pfam00900)→ RplE (COG0094)rpl5p (PRK04219)→ RpsN (COG0199)rps14P (PRK05766)→ rps8p (PRK04034)RpsH (COG0096)→ RplF (COG0097)rpl6p (PRK05518)→ PRK12766 (PRK12766)RecA (COG0468)Rpl32e (COG1717)→ rpl19e (PRK08570)Ribosomal_L19e (pfam01280)→
env-164	SL_6KL_011_BRINE_1124153	+	36	138	RNA→ PHA03252 (PHA03252)Methyltrn_RNA_3 (pfam02598)→
env-165	P_A23_Liq_2_Fmt_1045640	+	57	159	RNA→ Methyltrn_RNA_3 (pfam02598)→
env-166	P_A23_Liq_1_Fmt_1009104	+	715	817	RNA→ Methyltrn_RNA_3 (pfam02598)→
env-167	P_1C_Liq_1_UnCty_1002372	+	283	385	RNA→ Methyltrn_RNA_3 (pfam02598)→ RplC (COG0087)rpl3p (PRK04231)→ RplD (COG0088)rpl4lp (PRK04042)→ RplW (COG0089)PRK14548 (PRK14548)→ RplB (COG0090)rpl2p (PRK09612)→ rps19p (PRK04038)→
env-168	P_A23_Liq_3_Fmt_1018522	-	551	449	RNA→ Methyltrn_RNA_3 (pfam02598)→
env-169	P_2C_Liq_1_UnCty_1117457	-	344	242	RNA→ Methyltrn_RNA_3 (pfam02598)→
env-170	P_A23_Liq_1_Fmt_1046280	-	249	147	RNA→ Methyltrn_RNA_3 (pfam02598)→
env-171	P_A23_Liq_2_Fmt_1063179	-	245	143	RNA→ Methyltrn_RNA_3 (pfam02598)→
env-172	P_2C_Liq_1_UnCty_1157684	-	265	163	RNA→ Methyltrn_RNA_3 (pfam02598)→
env-173	SL_8KL_010_SED_10202168	-	335	236	RNA→ Methyltrn_RNA_3 (pfam02598)→
Hru-1-1	NZ_BBJO01000003.1	-	101302	101203	RNA→ Methyltrn_RNA_3 (pfam02598)→ RplC (COG0087)rpl3p (PRK04231)→ RplD (COG0088)rpl4lp (PRK04042)→ RplW (COG0089)PRK14548 (PRK14548)→ hypo→ hypo→ RpsS (COG0185)rps19p (PRK04038)→ rpl22p (PRK04223)RplV (COG0091)→ RpsC (COG0092)rps3p (PRK04191)→ PRK14549 (PRK14549)Ribosomal_L29 (pfam00831)→ PRK03879 (PRK03879)UPF0086 (pfam01868)→ RpsQ (COG0186)rps17p (PRK08572)→ rpl14p (PRK08571)Ribosomal_L14 (pfam00238)→ RplX (COG0198)rpl24p (PRK01191)→ PRK04313 (PRK04313)S4 (cd00165)KOW_RPS4 (cd06087)Ribosomal_S4e (pfam00900)→ RplE (COG0094)rpl5p (PRK04219)→ RpsN (COG0199)rps14P (PRK05766)→ rps8p (PRK04034)RpsH (COG0096)→ RplF (COG0097)rpl6p (PRK05518)→ PRK12766 (PRK12766)RecA (COG0468)Rpl32e (COG1717)→ rpl19e (PRK08570)Ribosomal_L19e (pfam01280)→
env-174	P_1C_Sed_2_UnCty_1300983	+	148	247	RNA→ Methyltrn_RNA_3 (pfam02598)→
Hru-2-1	NZ_BBJN01000060.1	+	9207	9306	RNA→ Methyltrn_RNA_3 (pfam02598)→ hypo→ rpl3p (PRK04231)Ribosomal_L3 (pfam00297)→
env-175	GSLNAS_F7I09SK02H8ZT2	+	136	236	RNA→ Methyltrn_RNA_3 (pfam02598)→
env-176	SL_6KL_011_BRINE_1005460	+	5299	5398	RNA→ Methyltrn_RNA_3 (pfam02598)→
env-177	SL_6KL_011_BRINE_1051712	-	689	590	RNA→ Methyltrn_RNA_3 (pfam02598)→ rpl3p (PRK04231)Ribosomal_L3 (pfam00297)→
env-178	SL_4KL_010_BRINE_10003656	-	660	561	RNA→ Methyltrn_RNA_3 (pfam02598)→ rpl3p (PRK04231)Ribosomal_L3 (pfam00297)→
env-179	JXWS01003911.1	-	291	192	RNA→ Methyltrn_RNA_3 (pfam02598)→
env-180	SL_6KL_011_BRINE_1016959	-	122	23	RNA→ Methyltrn_RNA_3 (pfam02598)→
Nmo-1-1	NC_020388.1	-	578139	578040	RNA→ Methyltrn_RNA_3 (pfam02598)→ rpl3p (PRK04231)Ribosomal_L3 (pfam00297)→ RplD (COG0088)rpl4lp (PRK04042)→ RplW (COG0089)PRK14548 (PRK14548)→ RplB (COG0090)rpl2p (PRK09612)→ RpsS (COG0185)rps19p (PRK04038)→ rpl22p (PRK04223)RplV (COG0091)→ RpsC (COG0092)rps3p (PRK04191)→ PRK14549 (PRK14549)Ribosomal_L29 (pfam00831)→ PRK03879 (PRK03879)UPF0086 (pfam01868)→ RpsQ (COG0186)rps17p (PRK08572)→ rpl14p (PRK08571)Ribosomal_L14 (pfam00238)→ RplX (COG0198)rpl24p (PRK01191)→ PRK04313 (PRK04313)KOW_RPS4 (cd06087)Ribosomal_S4e (pfam00900)→ RplE (COG0094)rpl5p (PRK04219)→ RpsN (COG0199)rps14P (PRK05766)→ rps8p (PRK04034)RpsH (COG0096)→ RplF (COG0097)rpl6p (PRK05518)→ PolY_Pol_IV_kappa (cd03586)PRK12766 (PRK12766)Rpl32e (COG1717)→ rpl19e (PRK08570)Ribosomal_L19e (pfam01280)→

Hha-1-1	NZ_AJRK01000444.1	-	10548	10449	**RNA**→ Methyltrn_RNA_3 (pfam02598)→ Methyltrn_RNA_3 (pfam02598)→ RplC (COG0087)rpl3p (PRK04231)→<br>RplC (COG0087)rpl3p (PRK04231)→ RplD (COG0088)rpl4lp (PRK04042)→<br>RplD (COG0088)rpl4lp (PRK04042)→ RplW (COG0089)PRK14548 (PRK14548)→<br>RplW (COG0089)PRK14548 (PRK14548)→ RplB (COG0090)rpl2p (PRK09612)→<br>RplB (COG0090)rpl2p (PRK09612)→ RpsS (COG0185)rps19p (PRK04038)→<br>RpsS (COG0185)rps19p (PRK04038)→ rpl22p (PRK04223)RplV (COG0091)→<br>rpl22p (PRK04223)RplV (COG0091)→<br>NT_POLXc (cd00141)RpsC (COG0092)rps3p (PRK04191)HHH_5 (pfam14520)→<br>NT_POLXc (cd00141)RpsC (COG0092)rps3p (PRK04191)HHH_5 (pfam14520)→<br>PRK14549 (PRK14549)Ribosomal_L29 (pfam00831)→ PRK14549 (PRK14549)Ribosomal_L29 (pfam00831)→<br>PRK03879 (PRK03879)UPF0086 (pfam01868)→ PRK03879 (PRK03879)UPF0086 (pfam01868)→<br>RpsQ (COG0186)rps17p (PRK08572)→ RpsQ (COG0186)rps17p (PRK08572)→<br>rpl14p (PRK08571)Ribosomal_L14 (pfam00238)→ rpl14p (PRK08571)Ribosomal_L14 (pfam00238)→<br>RplX (COG0198)rpl24p (PRK01191)→ RplX (COG0198)rpl24p (PRK01191)→<br>PRK04313 (PRK04313)KOW_RPS4 (cd06087)S4 (pfam01479)Ribosomal_S4e (pfam00900)→<br>PRK04313 (PRK04313)KOW_RPS4 (cd06087)S4 (pfam01479)Ribosomal_S4e (pfam00900)→<br>RplE (COG0094)rpl5p (PRK04219)→ RplE (COG0094)rpl5p (PRK04219)→<br>RpsN (COG0199)rps14P (PRK05766)→ RpsN (COG0199)rps14P (PRK05766)→<br>rps8p (PRK04034)RpsH (COG0096)→ rps8p (PRK04034)RpsH (COG0096)→<br>RplF (COG0097)rpl6p (PRK05518)→ RplF (COG0097)rpl6p (PRK05518)→<br>Lig (COG0272)Rpl32e (COG1717)rpl32e (PRK08562)HHH_5 (pfam14520)→<br>Lig (COG0272)Rpl32e (COG1717)rpl32e (PRK08562)HHH_5 (pfam14520)→
Hha-1-2	NZ_AOMB01000043.1	-	108188	108089	**RNA**→ Methyltrn_RNA_3 (pfam02598)→ RplC (COG0087)rpl3p (PRK04231)→<br>RplD (COG0088)rpl4lp (PRK04042)→ RplW (COG0089)PRK14548 (PRK14548)→<br>RplB (COG0090)rpl2p (PRK09612)→ RpsS (COG0185)rps19p (PRK04038)→<br>rpl22p (PRK04223)RplV (COG0091)→<br>NT_POLXc (cd00141)RpsC (COG0092)rps3p (PRK04191)HHH_5 (pfam14520)→<br>PRK14549 (PRK14549)Ribosomal_L29 (pfam00831)→ PRK03879 (PRK03879)UPF0086 (pfam01868)→<br>RpsQ (COG0186)rps17p (PRK08572)→ rpl14p (PRK08571)Ribosomal_L14 (pfam00238)→<br>RplX (COG0198)rpl24p (PRK01191)→<br>PRK04313 (PRK04313)KOW_RPS4 (cd06087)S4 (pfam01479)Ribosomal_S4e (pfam00900)→<br>RplE (COG0094)rpl5p (PRK04219)→ RpsN (COG0199)rps14P (PRK05766)→<br>rps8p (PRK04034)RpsH (COG0096)→ RplF (COG0097)rpl6p (PRK05518)→<br>Lig (COG0272)Rpl32e (COG1717)rpl32e (PRK08562)HHH_5 (pfam14520)→
env-181	SL_4KL_010_BRINE_10212872	-	267	168	**RNA**→ Methyltrn_RNA_3 (pfam02598)→

Hpa-1-1	NZ_AEMG01000030.1	-	41019	40920	**RNA**→ Methyltrn_RNA_3 (pfam02598)→ Methyltrn_RNA_3 (pfam02598)→<br>rpl3p (PRK04231)Ribosomal_L3 (pfam00297)→ rpl3p (PRK04231)Ribosomal_L3 (pfam00297)→<br>RplD (COG0088)rpl4lp (PRK04042)→ RplD (COG0088)rpl4lp (PRK04042)→<br>RplW (COG0089)PRK14548 (PRK14548)→ RplW (COG0089)PRK14548 (PRK14548)→<br>RplB (COG0090)rpl2p (PRK09612)→ RplB (COG0090)rpl2p (PRK09612)→<br>RpsS (COG0185)rps19p (PRK04038)→ RpsS (COG0185)rps19p (PRK04038)→<br>rpl22p (PRK04223)RplV (COG0091)→ rpl22p (PRK04223)RplV (COG0091)→<br>RpsC (COG0092)rps3p (PRK04191)→ RpsC (COG0092)rps3p (PRK04191)→<br>PRK14549 (PRK14549)Ribosomal_L29 (pfam00831)→ PRK14549 (PRK14549)Ribosomal_L29 (pfam00831)→<br>POP4 (COG1588)PRK03879 (PRK03879)→ POP4 (COG1588)PRK03879 (PRK03879)→<br>RpsQ (COG0186)rps17p (PRK08572)→ RpsQ (COG0186)rps17p (PRK08572)→<br>rpl14p (PRK08571)Ribosomal_L14 (pfam00238)→ rpl14p (PRK08571)Ribosomal_L14 (pfam00238)→<br>RplX (COG0198)rpl24p (PRK01191)→ RplX (COG0198)rpl24p (PRK01191)→<br>PRK04313 (PRK04313)KOW_RPS4 (cd06087)S4 (pfam01479)Ribosomal_S4e (pfam00900)→<br>PRK04313 (PRK04313)KOW_RPS4 (cd06087)S4 (pfam01479)Ribosomal_S4e (pfam00900)→<br>RplE (COG0094)rpl5p (PRK04219)→ RplE (COG0094)rpl5p (PRK04219)→<br>RpsN (COG0199)rps14P (PRK05766)→ RpsN (COG0199)rps14P (PRK05766)→<br>rps8p (PRK04034)RpsH (COG0096)→ rps8p (PRK04034)RpsH (COG0096)→<br>RplF (COG0097)rpl6p (PRK05518)→ RplF (COG0097)rpl6p (PRK05518)→<br>RecA (COG0468)rpl32e (PRK08562)Ribosomal_L32e (pfam01655)HHH_5 (pfam14520)→<br>RecA (COG0468)rpl32e (PRK08562)Ribosomal_L32e (pfam01655)HHH_5 (pfam14520)→<br>rpl19e (PRK08570)Ribosomal_L19e (pfam01280)→ rpl19e (PRK08570)Ribosomal_L19e (pfam01280)→
Hpa-1-2	NZ_AQXI01000001.1	-	1281236	1281137	**RNA**→ Methyltrn_RNA_3 (pfam02598)→ rpl3p (PRK04231)Ribosomal_L3 (pfam00297)→<br>RplC (COG0087)rpl3p (PRK04231)→ rpl3p (PRK04231)Ribosomal_L3 (pfam00297)→<br>RplD (COG0088)rpl4lp (PRK04042)→ RplW (COG0089)PRK14548 (PRK14548)→<br>RplB (COG0090)rpl2p (PRK09612)→ RpsS (COG0185)rps19p (PRK04038)→<br>rpl22p (PRK04223)RplV (COG0091)→ RpsC (COG0092)rps3p (PRK04191)→<br>PRK14549 (PRK14549)Ribosomal_L29 (pfam00831)→ POP4 (COG1588)PRK03879 (PRK03879)→<br>RpsQ (COG0186)rps17p (PRK08572)→ RpsQ (COG0186)rps17p (PRK08572)→<br>rpl14p (PRK08571)Ribosomal_L14 (pfam00238)→ RplX (COG0198)rpl24p (PRK01191)→<br>PRK04313 (PRK04313)KOW_RPS4 (cd06087)S4 (pfam01479)Ribosomal_S4e (pfam00900)→<br>RplE (COG0094)rpl5p (PRK04219)→ RpsN (COG0199)rps14P (PRK05766)→<br>rps8p (PRK04034)RpsH (COG0096)→ RplF (COG0097)rpl6p (PRK05518)→<br>RecA (COG0468)rpl32e (PRK08562)Ribosomal_L32e (pfam01655)HHH_5 (pfam14520)→<br>rpl19e (PRK08570)Ribosomal_L19e (pfam01280)→
Hci-1-1	NZ_JDTH01000001.1	+	807675	807774	**RNA**→ Methyltrn_RNA_3 (pfam02598)→ rpl3p (PRK04231)Ribosomal_L3 (pfam00297)→<br>RplD (COG0088)rpl4lp (PRK04042)→ RplW (COG0089)PRK14548 (PRK14548)→<br>RplB (COG0090)rpl2p (PRK09612)→ RpsS (COG0185)rps19p (PRK04038)→<br>rpl22p (PRK04223)RplV (COG0091)→ RpsC (COG0092)rps3p (PRK04191)→<br>PRK14549 (PRK14549)Ribosomal_L29 (pfam00831)→ POP4 (COG1588)PRK03879 (PRK03879)→<br>RpsQ (COG0186)rps17p (PRK08572)→ rpl14p (PRK08571)Ribosomal_L14 (pfam00238)→<br>rpl24p (PRK01191)KOW_RPL26 (cd06089)→<br>PRK04313 (PRK04313)KOW_RPS4 (cd06087)Ribosomal_S4e (pfam00900)→ RplE (COG0094)rpl5p (PRK04219)→<br>RpsN (COG0199)rps14P (PRK05766)→ rps8p (PRK04034)RpsH (COG0096)→<br>RplF (COG0097)rpl6p (PRK05518)→ UvrC (COG0322)Ribosomal_L32_L32e (cd00513)HHH_5 (pfam14520)→<br>rpl19e (PRK08570)Ribosomal_L19e (pfam01280)→
env-182	GRANPEH02G2JJD	+	188	287	**RNA**→ MTH1 (COG2106)→
env-183	GQKUMWE02FE1GX	-	166	68	**RNA**→ hypo→
env-184	GSLNARP_GFPJP1N02H7ZY7	-	182	83	**RNA**→ Methyltrn_RNA_3 (pfam02598)→
env-185	SL_5KL_010_BRINE_10021216	+	2341	2440	**RNA**→ Methyltrn_RNA_3 (pfam02598)→

Hdj-1-1	NZ_HG315688.1	-	94795	94696	**RNA**→ Methyltrn_RNA_3 (pfam02598)→ rpl3p (PRK04231)Ribosomal_L3 (pfam00297)→<br>RplD (COG0088)rpl4lp (PRK04042)→ RplW (COG0089)PRK14548 (PRK14548)→<br>RplB (COG0090)rpl2p (PRK09612)→ RpsS (COG0185)rps19p (PRK04038)→<br>rpl22p (PRK04223)RplV (COG0091)→ RpsC (COG0092)rps3p (PRK04191)→<br>RpmC (COG0255)PRK14549 (PRK14549)→ PRK03879 (PRK03879)UPF0086 (pfam01868)→<br>RpsQ (COG0186)rps17p (PRK08572)→ rpl14p (PRK08571)Ribosomal_L14 (pfam00238)→<br>rpl24p (PRK01191)KOW_RPL26 (cd06089)→<br>PRK04313 (PRK04313)KOW_RPS4 (cd06087)S4 (pfam01479)Ribosomal_S4e (pfam00900)→<br>RplE (COG0094)rpl5p (PRK04219)SDH (cd12188)→ RpsN (COG0199)rps14P (PRK05766)→<br>rps8p (PRK04034)RpsH (COG0096)→ RplF (COG0097)rpl6p (PRK05518)→<br>PRK12766 (PRK12766)RecA (COG0468)Rpl32e (COG1717)→ rpl19e (PRK08570)Ribosomal_L19e (pfam01280)→
Hsa-10-1	NZ_JROF01000007.1	-	139445	139346	**RNA**→ Methyltrn_RNA_3 (pfam02598)→ rpl3p (PRK04231)Ribosomal_L3 (pfam00297)→<br>RplD (COG0088)rpl4lp (PRK04042)→ RplW (COG0089)PRK14548 (PRK14548)→<br>RplB (COG0090)rpl2p (PRK09612)→ RpsS (COG0185)rps19p (PRK04038)→<br>rpl22p (PRK04223)RplV (COG0091)→ RpsC (COG0092)rps3p (PRK04191)→<br>PRK14549 (PRK14549)Ribosomal_L29 (pfam00831)→ PRK03879 (PRK03879)UPF0086 (pfam01868)→<br>RpsQ (COG0186)rps17p (PRK08572)→ rpl14p (PRK08571)Ribosomal_L14 (pfam00238)→<br>rpl24p (PRK01191)KOW_RPL26 (cd06089)→<br>PRK04313 (PRK04313)KOW_RPS4 (cd06087)Ribosomal_S4e (pfam00900)→ RplE (COG0094)rpl5p (PRK04219)→<br>RpsN (COG0199)rps14P (PRK05766)→ rps8p (PRK04034)RpsH (COG0096)→<br>RplF (COG0097)rpl6p (PRK05518)→ PRK12766 (PRK12766)RecA (COG0468)Rpl32e (COG1717)→<br>rpl19e (PRK08570)Ribosomal_L19e (pfam01280)→
Nsu-1-1	NZ_AOHX01000025.1	+	4846	4945	**RNA**→ Methyltrn_RNA_3 (pfam02598)→ rpl3p (PRK04231)Ribosomal_L3 (pfam00297)→<br>RplD (COG0088)rpl4lp (PRK04042)→ RplW (COG0089)PRK14548 (PRK14548)→<br>RplB (COG0090)rpl2p (PRK09612)→ RpsS (COG0185)rps19p (PRK04038)→<br>rpl22p (PRK04223)RplV (COG0091)→ RpsC (COG0092)rps3p (PRK04191)→<br>RpmC (COG0255)PRK14549 (PRK14549)→ PRK03879 (PRK03879)UPF0086 (pfam01868)→<br>RpsQ (COG0186)rps17p (PRK08572)→ rpl14p (PRK08571)Ribosomal_L14 (pfam00238)→<br>rpl24p (PRK01191)KOW_RPL26 (cd06089)→<br>PRK04313 (PRK04313)KOW_RPS4 (cd06087)Ribosomal_S4e (pfam00900)→ RplE (COG0094)rpl5p (PRK04219)→<br>RpsN (COG0199)rps14P (PRK05766)→ rps8p (PRK04034)RpsH (COG0096)→<br>RplF (COG0097)rpl6p (PRK05518)→<br>UvrC (COG0322)Rpl32e (COG1717)rpl32e (PRK08562)HHH_5 (pfam14520)→<br>rpl19e (PRK08570)Ribosomal_L19e (pfam01280)→ RplR (COG0256)rpl18p (PRK08569)→<br>RpsE (COG0098)rps5p (PRK04044)→ Ribosomal_L7_archeal_euk (cd01657)rpl30p (PRK06049)→<br>rpl15p (PRK06419)Ribosomal_L27A (pfam00828)→ SecY (COG0201)PRK08568 (PRK08568)→
Nba-1-1	NZ_AOHY01000055.1	+	4766	4865	**RNA**→ Methyltrn_RNA_3 (pfam02598)→ rpl3p (PRK04231)Ribosomal_L3 (pfam00297)→<br>RplD (COG0088)rpl4lp (PRK04042)→ RplW (COG0089)PRK14548 (PRK14548)→<br>RplB (COG0090)rpl2p (PRK09612)→ RpsS (COG0185)rps19p (PRK04038)→<br>rpl22p (PRK04223)RplV (COG0091)→ RpsC (COG0092)rps3p (PRK04191)→<br>RpmC (COG0255)PRK14549 (PRK14549)→ PRK03879 (PRK03879)UPF0086 (pfam01868)→<br>RpsQ (COG0186)rps17p (PRK08572)→ rpl14p (PRK08571)Ribosomal_L14 (pfam00238)→<br>rpl24p (PRK01191)KOW_RPL26 (cd06089)→<br>PRK04313 (PRK04313)KOW_RPS4 (cd06087)S4 (pfam01479)Ribosomal_S4e (pfam00900)→<br>RplE (COG0094)rpl5p (PRK04219)→ RpsN (COG0199)rps14P (PRK05766)→<br>rps8p (PRK04034)RpsH (COG0096)→ RplF (COG0097)rpl6p (PRK05518)→<br>RecA (COG0468)Rpl32e (COG1717)rpl32e (PRK08562)HHH_5 (pfam14520)→<br>rpl19e (PRK08570)Ribosomal_L19e (pfam01280)→ RplR (COG0256)rpl18p (PRK08569)→<br>RpsE (COG0098)rps5p (PRK04044)→ Ribosomal_L7_archeal_euk (cd01657)rpl30p (PRK06049)→<br>rpl15p (PRK06419)Ribosomal_L27A (pfam00828)→ SecY (COG0201)PRK08568 (PRK08568)→<br>SecY (COG0201)PRK08568 (PRK08568)→ SecY (COG0201)PRK08568 (PRK08568)→

Nin-1-1	NZ_AOHZ01000041.1	-	187314	187215	**RNA**→ Methyltrn_RNA_3 (pfam02598)→ rpl3p (PRK04231)Ribosomal_L3 (pfam00297)→
					RplD (COG0088)rpl4lp (PRK04042)→ RplW (COG0089)PRK14548 (PRK14548)→
					RplB (COG0090)rpl2p (PRK09612)→ RpsS (COG0185)rps19p (PRK04038)→
					rpl22p (PRK04223)RplV (COG0091)→ RpsC (COG0092)rps3p (PRK04191)→
					RpmC (COG0255)PRK14549 (PRK14549)→ PRK03879 (PRK03879)UPF0086 (pfam01868)→
					RpsQ (COG0186)rps17p (PRK08572)→ rpl14p (PRK08571)Ribosomal_L14 (pfam00238)→
					rpl24p (PRK01191)KOW_RPL26 (cd06089)→
					PRK04313 (PRK04313)S4 (cd00165)KOW_RPS4 (cd06087)Ribosomal_S4e (pfam00900)→
					RplE (COG0094)rpl5p (PRK04219)→ RpsN (COG0199)rps14P (PRK05766)→
					rps8p (PRK04034)RpsH (COG0096)→ RplF (COG0097)rpl6p (PRK05518)→
					RecA (COG0468)Rpl32e (COG1717)rpl32e (PRK08562)HHH_5 (pfam14520)→
					rpl19e (PRK08570)Ribosomal_L19e (pfam01280)→
env-186	SL_4KL_010_BRINE_10024882	-	1898	1799	**RNA**→ Methyltrn_RNA_3 (pfam02598)→ rpl3p (PRK04231)Ribosomal_L3 (pfam00297)→
					RplD (COG0088)rpl4lp (PRK04042)→
env-187	ADL20m3uS_GFI381O02JC3VL	+	316	416	**RNA**→
env-188	SL_6KL_011_BRINE_1000304	+	9714	9813	**RNA**→ Methyltrn_RNA_3 (pfam02598)→ rpl3p (PRK04231)Ribosomal_L3 (pfam00297)→
					RplD (COG0088)rpl4lp (PRK04042)→ RplW (COG0089)PRK14548 (PRK14548)→
					RplB (COG0090)rpl2p (PRK09612)→ RpsS (COG0185)rps19p (PRK04038)→
					rpl22p (PRK04223)RplV (COG0091)→ RpsC (COG0092)rps3p (PRK04191)→
					PRK14549 (PRK14549)Ribosomal_L29 (pfam00831)→ PRK03879 (PRK03879)UPF0086 (pfam01868)→
					RpsQ (COG0186)rps17p (PRK08572)→ rpl14p (PRK08571)Ribosomal_L14 (pfam00238)→
					rpl24p (PRK01191)KOW_RPL26 (cd06089)→
					PRK04313 (PRK04313)KOW_RPS4 (cd06087)S4 (pfam01479)Ribosomal_S4e (pfam00900)→
					RplE (COG0094)rpl5p (PRK04219)→ RpsN (COG0199)rps14P (PRK05766)→
					rps8p (PRK04034)RpsH (COG0096)→ RplF (COG0097)rpl6p (PRK05518)→
					UvrC (COG0322)Rpl32e (COG1717)rpl32e (PRK08562)HHH_5 (pfam14520)→
					rpl19e (PRK08570)Ribosomal_L19e (pfam01280)→
env-189	SL_8KL_010_SED_10375651	-	274	175	**RNA**→ Methyltrn_RNA_3 (pfam02598)→
env-190	SL_6KL_011_BRINE_1014318	-	155	56	**RNA**→ Methyltrn_RNA_3 (pfam02598)→
env-191	SL_8KL_010_SED_10000480	+	6111	6210	**RNA**→ Methyltrn_RNA_3 (pfam02598)→ rpl3p (PRK04231)Ribosomal_L3 (pfam00297)→
					RplD (COG0088)rpl4lp (PRK04042)→ RplW (COG0089)PRK14548 (PRK14548)→
					RplB (COG0090)rpl2p (PRK09612)→ RpsS (COG0185)rps19p (PRK04038)→
					rpl22p (PRK04223)RplV (COG0091)→ RpsC (COG0092)rps3p (PRK04191)→
					RpmC (COG0255)PRK14549 (PRK14549)→ PRK03879 (PRK03879)UPF0086 (pfam01868)→
					RpsQ (COG0186)rps17p (PRK08572)→ rpl14p (PRK08571)Ribosomal_L14 (pfam00238)→
					rpl24p (PRK01191)KOW_RPL26 (cd06089)→
					PRK04313 (PRK04313)KOW_RPS4 (cd06087)S4 (pfam01479)Ribosomal_S4e (pfam00900)→
					RplE (COG0094)rpl5p (PRK04219)→ RpsN (COG0199)rps14P (PRK05766)→
					rps8p (PRK04034)RpsH (COG0096)→ RplF (COG0097)rpl6p (PRK05518)→
					PRK12766 (PRK12766)UvrC (COG0322)Rpl32e (COG1717)→ rpl19e (PRK08570)Ribosomal_L19e (pfam01280)→
					RplR (COG0256)rpl18p (PRK08569)→ RpsE (COG0098)rps5p (PRK04044)→
					Ribosomal_L7_archeal_euk (cd01657)rpl30p (PRK06049)→ rpl15p (PRK06419)Ribosomal_L27A (pfam00828)→
					SecY (COG0201)PRK08568 (PRK08568)→
env-192	SL_4KL_010_BRINE_10005807	-	721	622	**RNA**→ Methyltrn_RNA_3 (pfam02598)→ rpl3p (PRK04231)Ribosomal_L3 (pfam00297)→

Hla-1-1	NZ_AGFZ01000103.1	-	104403	104304	**RNA**→ Methyltrn_RNA_3 (pfam02598)→ Methyltrn_RNA_3 (pfam02598)→ RplC (COG0087)rpl3p (PRK04231)→
					RplC (COG0087)rpl3p (PRK04231)→ RplD (COG0088)rpl4lp (PRK04042)→
					RplD (COG0088)rpl4lp (PRK04042)→ RplW (COG0089)PRK14548 (PRK14548)→
					RplW (COG0089)PRK14548 (PRK14548)→ RplB (COG0090)rpl2p (PRK09612)→
					RplB (COG0090)rpl2p (PRK09612)→ RpsS (COG0185)rps19p (PRK04038)→
					RpsS (COG0185)rps19p (PRK04038)→ rpl22p (PRK04223)RplV (COG0091)→
					rpl22p (PRK04223)RplV (COG0091)→ RpsC (COG0092)rps3p (PRK04191)→
					RpsC (COG0092)rps3p (PRK04191)→ PRK14549 (PRK14549)Ribosomal_L29 (pfam00831)→
					PRK14549 (PRK14549)Ribosomal_L29 (pfam00831)→ PRK03879 (PRK03879)UPF0086 (pfam01868)→
					PRK03879 (PRK03879)UPF0086 (pfam01868)→ RpsQ (COG0186)rps17p (PRK08572)→
					RpsQ (COG0186)rps17p (PRK08572)→ rpl14p (PRK08571)Ribosomal_L14 (pfam00238)→
					rpl14p (PRK08571)Ribosomal_L14 (pfam00238)→ rpl24p (PRK01191)KOW_RPL26 (cd06089)→
					rpl24p (PRK01191)KOW_RPL26 (cd06089)→
					PRK04313 (PRK04313)S4 (cd00165)KOW_RPS4 (cd06087)Ribosomal_S4e (pfam00900)→
					PRK04313 (PRK04313)S4 (cd00165)KOW_RPS4 (cd06087)Ribosomal_S4e (pfam00900)→
					RplE (COG0094)rpl5p (PRK04219)→ RplE (COG0094)rpl5p (PRK04219)→
					RpsN (COG0199)rps14P (PRK05766)→ RpsN (COG0199)rps14P (PRK05766)→
					rps8p (PRK04034)RpsH (COG0096)→ rps8p (PRK04034)RpsH (COG0096)→
					RplF (COG0097)rpl6p (PRK05518)→ RplF (COG0097)rpl6p (PRK05518)→
					RecA (COG0468)Rpl32e (COG1717)rpl32e (PRK08562)HHH_5 (pfam14520)→
					RecA (COG0468)Rpl32e (COG1717)rpl32e (PRK08562)HHH_5 (pfam14520)→
Hla-1-2	NZ_AOLZ01000040.1	-	62859	62760	**RNA**→ Methyltrn_RNA_3 (pfam02598)→ RplC (COG0087)rpl3p (PRK04231)→
					RplD (COG0088)rpl4lp (PRK04042)→ RplW (COG0089)PRK14548 (PRK14548)→
					RplB (COG0090)rpl2p (PRK09612)→ RpsS (COG0185)rps19p (PRK04038)→
					rpl22p (PRK04223)RplV (COG0091)→ RpsC (COG0092)rps3p (PRK04191)→
					PRK14549 (PRK14549)Ribosomal_L29 (pfam00831)→ PRK03879 (PRK03879)UPF0086 (pfam01868)→
					RpsQ (COG0186)rps17p (PRK08572)→ rpl14p (PRK08571)Ribosomal_L14 (pfam00238)→
					rpl24p (PRK01191)KOW_RPL26 (cd06089)→
					PRK04313 (PRK04313)S4 (cd00165)KOW_RPS4 (cd06087)Ribosomal_S4e (pfam00900)→
					RplE (COG0094)rpl5p (PRK04219)→ RpsN (COG0199)rps14P (PRK05766)→
					rps8p (PRK04034)RpsH (COG0096)→ RplF (COG0097)rpl6p (PRK05518)→
					RecA (COG0468)Rpl32e (COG1717)rpl32e (PRK08562)HHH_5 (pfam14520)→
env-193	SL_5KL_010_BRINE_10000580	-	14326	14227	**RNA**→ Methyltrn_RNA_3 (pfam02598)→ rpl3p (PRK04231)Ribosomal_L3 (pfam00297)→
					RplD (COG0088)rpl4lp (PRK04042)→ RplW (COG0089)PRK14548 (PRK14548)→
					RplB (COG0090)rpl2p (PRK09612)→ RpsS (COG0185)rps19p (PRK04038)→
					rpl22p (PRK04223)RplV (COG0091)→ RpsC (COG0092)rps3p (PRK04191)→
					RpmC (COG0255)PRK14549 (PRK14549)→ PRK03879 (PRK03879)UPF0086 (pfam01868)→
					RpsQ (COG0186)rps17p (PRK08572)→ rpl14p (PRK08571)Ribosomal_L14 (pfam00238)→
					RplX (COG0198)rpl24p (PRK01191)→
					PRK04313 (PRK04313)S4 (cd00165)KOW_RPS4 (cd06087)Ribosomal_S4e (pfam00900)→
					RplE (COG0094)rpl5p (PRK04219)→ RpsN (COG0199)rps14P (PRK05766)→
					rps8p (PRK04034)RpsH (COG0096)→ RplF (COG0097)rpl6p (PRK05518)→
					PRK12766 (PRK12766)UvrC (COG0322)Rpl32e (COG1717)→
env-194	SL_5KL_010_SED_10042598	+	887	986	**RNA**→ Methyltrn_RNA_3 (pfam02598)→
env-195	SL_4KL_010_BRINE_10006165	+	1728	1827	**RNA**→ Methyltrn_RNA_3 (pfam02598)→ rpl3p (PRK04231)Ribosomal_L3 (pfam00297)→
					RplD (COG0088)rpl4lp (PRK04042)→ RplW (COG0089)PRK14548 (PRK14548)→
					RplB (COG0090)rpl2p (PRK09612)→ RpsS (COG0185)rps19p (PRK04038)→
					rpl22p (PRK04223)RplV (COG0091)→ RpsC (COG0092)rps3p (PRK04191)→
					PRK14549 (PRK14549)Ribosomal_L29_HIP (cd00427)→
Hac-1-1	NZ_BATA01000095.1	+	7771	7870	**RNA**→ MTH1 (COG2106)→ hypo→ hypo→

Hac-1-2	NZ_BANO01000013.1	-	14331	14232	**RNA**→ Methyltrn_RNA_3 (pfam02598)→ rpl3p (PRK04231)Ribosomal_L3 (pfam00297)→<br>RplC (COG0087)rpl3p (PRK04231)→ RplC (COG0087)rpl3p (PRK04231)→<br>RplD (COG0088)rpl4lp (PRK04042)→ RplW (COG0089)PRK14548 (PRK14548)→<br>RplB (COG0090)rpl2p (PRK09612)→ RpsS (COG0185)rps19p (PRK04038)→<br>rpl22p (PRK04223)RplV (COG0091)→ RpsC (COG0092)rps3p (PRK04191)→<br>PRK14549 (PRK14549)Ribosomal_L29 (pfam00831)→ POP4 (COG1588)PRK03879 (PRK03879)→<br>RpsQ (COG0186)rps17p (PRK08572)→ rps17p (PRK08572)Ribosomal_S17 (pfam00366)→<br>rpl14p (PRK08571)Ribosomal_L14 (pfam00238)→ RplX (COG0198)rpl24p (PRK01191)→<br>PRK04313 (PRK04313)KOW_RPS4 (cd06087)Ribosomal_S4e (pfam00900)→ RplE (COG0094)rpl5p (PRK04219)→<br>RpsN (COG0199)rps14P (PRK05766)→ rps8p (PRK04034)RpsH (COG0096)→<br>RplF (COG0097)rpl6p (PRK05518)→
env-196	ADL20m3uS_GFI381O02IYW5A	-	121	22	**RNA**→ MTH1 (COG2106)→
env-197	2140918017-63356	-	193	94	**RNA**→ hypo→
env-198	GQKUMWE02GP8XW	-	475	376	**RNA**→ Methyltrn_RNA_3 (pfam02598)→
Hsp-2-1	NZ_CP007060.1	+	2200475	2200574	**RNA**→ Methyltrn_RNA_3 (pfam02598)→ rpl3p (PRK04231)Ribosomal_L3 (pfam00297)→<br>RplD (COG0088)rpl4lp (PRK04042)→ RplW (COG0089)PRK14548 (PRK14548)→<br>RplB (COG0090)rpl2p (PRK09612)→ RpsS (COG0185)rps19p (PRK04038)→<br>rpl22p (PRK04223)RplV (COG0091)→ RpsC (COG0092)rps3p (PRK04191)→<br>PRK14549 (PRK14549)Ribosomal_L29 (pfam00831)→ PRK03879 (PRK03879)UPF0086 (pfam01868)→<br>RpsQ (COG0186)rps17p (PRK08572)→ rpl14p (PRK08571)Ribosomal_L14 (pfam00238)→<br>RplX (COG0198)rpl24p (PRK01191)→<br>PRK04313 (PRK04313)KOW_RPS4 (cd06087)Ribosomal_S4e (pfam00900)→ RplE (COG0094)rpl5p (PRK04219)→<br>RpsN (COG0199)rps14P (PRK05766)→ rps8p (PRK04034)RpsH (COG0096)→<br>RplF (COG0097)rpl6p (PRK05518)→ PRK12766 (PRK12766)RecA (COG0468)Rpl32e (COG1717)→<br>rpl19e (PRK08570)Ribosomal_L19e (pfam01280)→
env-199	APHM01006302.1	-	1366	1273	**RNA**→ Methyltrn_RNA_3 (pfam02598)→ RplC (COG0087)rpl3p (PRK04231)→
env-200	LTJ07As871_J07ABscf092781	+	25242	25335	**RNA**→ Methyltrn_RNA_3 (pfam02598)→ rpl3p (PRK04231)Ribosomal_L3 (pfam00297)→ hypo→<br>PRK12766 (PRK12766)Rpl32e (COG1717)→ rpl19e (PRK08570)Ribosomal_L19e (pfam01280)→
Hsu-1-1	NZ_CP008874.1	-	1720180	1720081	**RNA**→ Methyltrn_RNA_3 (pfam02598)→ RplC (COG0087)rpl3p (PRK04231)→<br>RplD (COG0088)rpl4lp (PRK04042)→ RplW (COG0089)PRK14548 (PRK14548)→<br>RplB (COG0090)rpl2p (PRK09612)→ RpsS (COG0185)rps19p (PRK04038)→<br>rpl22p (PRK04223)RplV (COG0091)→ RpsC (COG0092)rps3p (PRK04191)→<br>PRK14549 (PRK14549)Ribosomal_L29 (pfam00831)→ PRK03879 (PRK03879)UPF0086 (pfam01868)→<br>RpsQ (COG0186)rps17p (PRK08572)→ rpl14p (PRK08571)Ribosomal_L14 (pfam00238)→<br>RplX (COG0198)rpl24p (PRK01191)→<br>PRK04313 (PRK04313)KOW_RPS4 (cd06087)Ribosomal_S4e (pfam00900)→ RplE (COG0094)rpl5p (PRK04219)→<br>RpsN (COG0199)rps14P (PRK05766)→ rps8p (PRK04034)RpsH (COG0096)→<br>RplF (COG0097)rpl6p (PRK05518)→<br>RecA (COG0468)Rpl32e (COG1717)rpl32e (PRK08562)HHH_5 (pfam14520)→<br>rpl19e (PRK08570)Ribosomal_L19e (pfam01280)→
env-201	P_A23_Liq_1_Fmt_1021786	-	270	171	**RNA**→ Methyltrn_RNA_3 (pfam02598)→
env-202	P_A23_Liq_2_Fmt_1024942	-	260	161	**RNA**→ Methyltrn_RNA_3 (pfam02598)→
env-203	P_A23_Liq_3_Fmt_1011772	+	655	754	**RNA**→ Methyltrn_RNA_3 (pfam02598)→
Hca-1-1	NZ_AOLS01000002.1	-	28213	28114	**RNA**→ Methyltrn_RNA_3 (pfam02598)→ rpl3p (PRK04231)Ribosomal_L3 (pfam00297)→<br>rpl4lp (PRK04042)Ribosomal_L4 (pfam00573)→ RplW (COG0089)PRK14548 (PRK14548)→<br>RplB (COG0090)rpl2p (PRK09612)→ RpsS (COG0185)rps19p (PRK04038)→<br>rpl22p (PRK04223)RplV (COG0091)→ RpsC (COG0092)rps3p (PRK04191)→<br>PRK14549 (PRK14549)Ribosomal_L29 (pfam00831)→ PRK03879 (PRK03879)UPF0086 (pfam01868)→<br>RpsQ (COG0186)rps17p (PRK08572)→ rpl14p (PRK08571)Ribosomal_L14 (pfam00238)→<br>rpl24p (PRK01191)KOW_RPL26 (cd06089)→<br>PRK04313 (PRK04313)KOW_RPS4 (cd06087)S4 (pfam01479)Ribosomal_S4e (pfam00900)→<br>RplE (COG0094)rpl5p (PRK04219)→ RpsN (COG0199)rps14P (PRK05766)→<br>rps8p (PRK04034)RpsH (COG0096)→ RplF (COG0097)rpl6p (PRK05518)→<br>PRK12766 (PRK12766)RecA (COG0468)Rpl32e (COG1717)→ rpl19e (PRK08570)Ribosomal_L19e (pfam01280)→<br>RplR (COG0256)rpl18p (PRK08569)→ RplR (COG0256)rpl18p (PRK08569)→

Hsi-1-1	NZ_AOLR01000002.1	-	251120	251021	**RNA**→ Methyltrn_RNA_3 (pfam02598)→ rpl3p (PRK04231)Ribosomal_L3 (pfam00297)→<br>rpl4lp (PRK04042)Ribosomal_L4 (pfam00573)→ RplW (COG0089)PRK14548 (PRK14548)→<br>RplB (COG0090)rpl2p (PRK09612)→ RpsS (COG0185)rps19p (PRK04038)→<br>rpl22p (PRK04223)RplV (COG0091)→ RpsC (COG0092)rps3p (PRK04191)→<br>PRK14549 (PRK14549)Ribosomal_L29 (pfam00831)→ POP4 (COG1588)PRK03879 (PRK03879)→<br>RpsQ (COG0186)rps17p (PRK08572)→ rpl14p (PRK08571)Ribosomal_L14 (pfam00238)→<br>rpl24p (PRK01191)KOW_RPL26 (cd06089)→<br>PRK04313 (PRK04313)KOW_RPS4 (cd06087)S4 (pfam01479)Ribosomal_S4e (pfam00900)→<br>RplE (COG0094)rpl5p (PRK04219)→ RpsN (COG0199)rps14P (PRK05766)→<br>rps8p (PRK04034)RpsH (COG0096)→ RplF (COG0097)rpl6p (PRK05518)→<br>PRK12766 (PRK12766)RecA (COG0468)Rpl32e (COG1717)→ rpl19e (PRK08570)Ribosomal_L19e (pfam01280)→<br>RplR (COG0256)rpl18p (PRK08569)→ RplR (COG0256)rpl18p (PRK08569)→
Hma-1-1	NC_006396.1	-	1443199	1443100	**RNA**→ Methyltrn_RNA_3 (pfam02598)→ rpl3p (PRK04231)Ribosomal_L3 (pfam00297)→<br>rpl4lp (PRK04042)Ribosomal_L4 (pfam00573)→ RplW (COG0089)PRK14548 (PRK14548)→<br>RplB (COG0090)rpl2p (PRK09612)→ RpsS (COG0185)rps19p (PRK04038)→<br>rpl22p (PRK04223)RplV (COG0091)→ RpsC (COG0092)rps3p (PRK04191)→<br>PRK14549 (PRK14549)Ribosomal_L29 (pfam00831)→ POP4 (COG1588)PRK03879 (PRK03879)→<br>POP4 (COG1588)PRK03879 (PRK03879)→ PRK03879 (PRK03879)UPF0086 (pfam01868)→<br>RpsQ (COG0186)rps17p (PRK08572)→ rpl14p (PRK08571)Ribosomal_L14 (pfam00238)→<br>rpl24p (PRK01191)KOW_RPL26 (cd06089)→<br>PRK04313 (PRK04313)KOW_RPS4 (cd06087)S4 (pfam01479)Ribosomal_S4e (pfam00900)→<br>RplE (COG0094)rpl5p (PRK04219)→ RpsN (COG0199)rps14P (PRK05766)→<br>rps8p (PRK04034)RpsH (COG0096)→ RplF (COG0097)rpl6p (PRK05518)→<br>PRK12766 (PRK12766)RecA (COG0468)Rpl32e (COG1717)→ rpl19e (PRK08570)Ribosomal_L19e (pfam01280)→<br>RplR (COG0256)rpl18p (PRK08569)→
Hsp-1-1	NZ_CP010529.1	-	696901	696802	**RNA**→ Methyltrn_RNA_3 (pfam02598)→ rpl3p (PRK04231)Ribosomal_L3 (pfam00297)→<br>rpl4lp (PRK04042)Ribosomal_L4 (pfam00573)→ RplW (COG0089)PRK14548 (PRK14548)→<br>RplB (COG0090)rpl2p (PRK09612)→ RpsS (COG0185)rps19p (PRK04038)→<br>rpl22p (PRK04223)RplV (COG0091)→ RpsC (COG0092)rps3p (PRK04191)→<br>PRK14549 (PRK14549)Ribosomal_L29 (pfam00831)→ PRK03879 (PRK03879)UPF0086 (pfam01868)→<br>RpsQ (COG0186)rps17p (PRK08572)→ rpl14p (PRK08571)Ribosomal_L14 (pfam00238)→<br>rpl24p (PRK01191)KOW_RPL26 (cd06089)→<br>PRK04313 (PRK04313)KOW_RPS4 (cd06087)S4 (pfam01479)Ribosomal_S4e (pfam00900)→<br>RplE (COG0094)rpl5p (PRK04219)→ RpsN (COG0199)rps14P (PRK05766)→<br>rps8p (PRK04034)RpsH (COG0096)→ RplF (COG0097)rpl6p (PRK05518)→<br>PRK12766 (PRK12766)RecA (COG0468)Rpl32e (COG1717)→ rpl19e (PRK08570)Ribosomal_L19e (pfam01280)→
Ham-1-1	NZ_AOLW01000047.1	+	239419	239518	**RNA**→ Methyltrn_RNA_3 (pfam02598)→ rpl3p (PRK04231)Ribosomal_L3 (pfam00297)→<br>rpl4lp (PRK04042)Ribosomal_L4 (pfam00573)→ RplW (COG0089)PRK14548 (PRK14548)→
Hhi-2-1	NC_023013.1	-	2093063	2092964	**RNA**→ Methyltrn_RNA_3 (pfam02598)→ rpl3p (PRK04231)Ribosomal_L3 (pfam00297)→<br>rpl4lp (PRK04042)Ribosomal_L4 (pfam00573)→ RplW (COG0089)PRK14548 (PRK14548)→<br>RplB (COG0090)rpl2p (PRK09612)→ RpsS (COG0185)rps19p (PRK04038)→<br>rpl22p (PRK04223)RplV (COG0091)→ RpsC (COG0092)rps3p (PRK04191)→<br>PRK14549 (PRK14549)Ribosomal_L29 (pfam00831)→ PRK03879 (PRK03879)UPF0086 (pfam01868)→<br>RpsQ (COG0186)rps17p (PRK08572)→ rpl14p (PRK08571)Ribosomal_L14 (pfam00238)→<br>rpl24p (PRK01191)KOW_RPL26 (cd06089)→<br>PRK04313 (PRK04313)KOW_RPS4 (cd06087)S4 (pfam01479)Ribosomal_S4e (pfam00900)→<br>RplE (COG0094)rpl5p (PRK04219)→ RpsN (COG0199)rps14P (PRK05766)→<br>rps8p (PRK04034)RpsH (COG0096)→ RplF (COG0097)rpl6p (PRK05518)→<br>PRK12766 (PRK12766)RecA (COG0468)Rpl32e (COG1717)→ rpl19e (PRK08570)Ribosomal_L19e (pfam01280)→

Hhi-1-1	NC_015948.1	-	2081630	2081531	**RNA**→ Methyltrn_RNA_3 (pfam02598)→ rpl3p (PRK04231)Ribosomal_L3 (pfam00297)→ rpl4lp (PRK04042)Ribosomal_L4 (pfam00573)→ RplW (COG0089)PRK14548 (PRK14548)→ RplB (COG0090)rpl2p (PRK09612)→ RpsS (COG0185)rps19p (PRK04038)→ rpl22p (PRK04223)RplV (COG0091)→ RpsC (COG0092)rps3p (PRK04191)→ PRK14549 (PRK14549)Ribosomal_L29 (pfam00831)→ PRK03879 (PRK03879)UPF0086 (pfam01868)→ RpsQ (COG0186)rps17p (PRK08572)→ rpl14p (PRK08571)Ribosomal_L14 (pfam00238)→ rpl24p (PRK01191)KOW_RPL26 (cd06089)→ PRK04313 (PRK04313)KOW_RPS4 (cd06087)S4 (pfam01479)Ribosomal_S4e (pfam00900)→ PRK04313 (PRK04313)KOW_RPS4 (cd06087)S4 (pfam01479)Ribosomal_S4e (pfam00900)→ RplE (COG0094)rpl5p (PRK04219)→ RpsN (COG0199)rps14P (PRK05766)→ rps8p (PRK04034)RpsH (COG0096)→ RplF (COG0097)rpl6p (PRK05518)→ PRK12766 (PRK12766)RecA (COG0468)Rpl32e (COG1717)→ rpl19e (PRK08570)Ribosomal_L19e (pfam01280)→
Hsa-2-1	NZ_JXAO01000052.1	+	4509	4608	**RNA**→ hypo→ rpl3p (PRK04231)Ribosomal_L3 (pfam00297)→ rpl4lp (PRK04042)Ribosomal_L4 (pfam00573)→ RplW (COG0089)PRK14548 (PRK14548)→ hypo→ RpsS (COG0185)rps19p (PRK04038)→ rpl22p (PRK04223)RplV (COG0091)→ RpsC (COG0092)rps3p (PRK04191)→ PRK14549 (PRK14549)Ribosomal_L29 (pfam00831)→ POP4 (COG1588)PRK03879 (PRK03879)→ RpsQ (COG0186)rps17p (PRK08572)→ rpl14p (PRK08571)Ribosomal_L14 (pfam00238)→ rpl24p (PRK01191)KOW_RPL26 (cd06089)→ hypo→ RplE (COG0094)rpl5p (PRK04219)→ RpsN (COG0199)rps14P (PRK05766)→ rps8p (PRK04034)RpsH (COG0096)→ RplF (COG0097)rpl6p (PRK05518)→ hypo→ rpl19e (PRK08570)Ribosomal_L19e (pfam01280)→
Har-1-1	NZ_AOLX01000026.1	+	231236	231335	**RNA**→ Methyltrn_RNA_3 (pfam02598)→ rpl3p (PRK04231)Ribosomal_L3 (pfam00297)→ rpl4lp (PRK04042)Ribosomal_L4 (pfam00573)→ RplW (COG0089)PRK14548 (PRK14548)→
Hva-1-1	NZ_AOLQ01000042.1	-	26191	26092	**RNA**→ Methyltrn_RNA_3 (pfam02598)→ rpl3p (PRK04231)Ribosomal_L3 (pfam00297)→ rpl4lp (PRK04042)Ribosomal_L4 (pfam00573)→ RplW (COG0089)PRK14548 (PRK14548)→ RplB (COG0090)rpl2p (PRK09612)→ RpsS (COG0185)rps19p (PRK04038)→ rpl22p (PRK04223)RplV (COG0091)→ RpsC (COG0092)rps3p (PRK04191)→ PRK14549 (PRK14549)Ribosomal_L29 (pfam00831)→ POP4 (COG1588)PRK03879 (PRK03879)→ RpsQ (COG0186)rps17p (PRK08572)→ rpl14p (PRK08571)Ribosomal_L14 (pfam00238)→ rpl24p (PRK01191)KOW_RPL26 (cd06089)→ PRK04313 (PRK04313)KOW_RPS4 (cd06087)S4 (pfam01479)Ribosomal_S4e (pfam00900)→ RplE (COG0094)rpl5p (PRK04219)→ RpsN (COG0199)rps14P (PRK05766)→ rps8p (PRK04034)RpsH (COG0096)→ RplF (COG0097)rpl6p (PRK05518)→ PRK12766 (PRK12766)RecA (COG0468)Rpl32e (COG1717)→ rpl19e (PRK08570)Ribosomal_L19e (pfam01280)→
Hja-1-1	NZ_AOLY01000009.1	+	92110	92209	**RNA**→ Methyltrn_RNA_3 (pfam02598)→ rpl3p (PRK04231)Ribosomal_L3 (pfam00297)→ rpl4lp (PRK04042)Ribosomal_L4 (pfam00573)→ RplW (COG0089)PRK14548 (PRK14548)→ RplB (COG0090)rpl2p (PRK09612)→ RpsS (COG0185)rps19p (PRK04038)→ rpl22p (PRK04223)RplV (COG0091)→ RpsC (COG0092)rps3p (PRK04191)→ PRK14549 (PRK14549)Ribosomal_L29 (pfam00831)→ PRK03879 (PRK03879)UPF0086 (pfam01868)→ RpsQ (COG0186)rps17p (PRK08572)→ rpl14p (PRK08571)Ribosomal_L14 (pfam00238)→ rpl24p (PRK01191)KOW_RPL26 (cd06089)→ PRK04313 (PRK04313)KOW_RPS4 (cd06087)S4 (pfam01479)Ribosomal_S4e (pfam00900)→ RplE (COG0094)rpl5p (PRK04219)→ RpsN (COG0199)rps14P (PRK05766)→ rps8p (PRK04034)RpsH (COG0096)→ RplF (COG0097)rpl6p (PRK05518)→ PRK12766 (PRK12766)RecA (COG0468)Rpl32e (COG1717)→ rpl19e (PRK08570)Ribosomal_L19e (pfam01280)→
env-204	APHM01021165.1	+	2893	2995	**RNA**→ Methyltrn_RNA_3 (pfam02598)→ RplC (COG0087)rpl3p (PRK04231)→ RplD (COG0088)rpl4lp (PRK04042)→ rpl4lp (PRK04042)→ RplW (COG0089)PRK14548 (PRK14548)→ RplB (COG0090)rpl2p (PRK09612)→ RpsS (COG0185)rps19p (PRK04038)→
env-205	LTJ07As871_J07ABscf098843	+	48637	48739	**RNA**→ Methyltrn_RNA_3 (pfam02598)→ RplC (COG0087)rpl3p (PRK04231)→ RplD (COG0088)rpl4lp (PRK04042)→ rpl4lp (PRK04042)→ RplW (COG0089)PRK14548 (PRK14548)→ RplB (COG0090)rpl2p (PRK09612)→ RpsS (COG0185)rps19p (PRK04038)→

Hse-1-1	NZ_BBMP01000022.1	-	550285	550186	**RNA**→ Methyltrn_RNA_3 (pfam02598)→ RplC (COG0087)rpl3p (PRK04231)→<br>RplD (COG0088)rpl4lp (PRK04042)→ RplW (COG0089)PRK14548 (PRK14548)→<br>RplB (COG0090)rpl2p (PRK09612)→ RpsS (COG0185)rps19p (PRK04038)→<br>rpl22p (PRK04223)RplV (COG0091)→<br>RpsC (COG0092)MDN1 (COG5271)rps3p (PRK04191)HHH_5 (pfam14520)→<br>PRK14549 (PRK14549)Ribosomal_L29 (pfam00831)→ PRK03879 (PRK03879)UPF0086 (pfam01868)→<br>RpsQ (COG0186)rps17p (PRK08572)→ rpl14p (PRK08571)Ribosomal_L14 (pfam00238)→<br>RplX (COG0198)rpl24p (PRK01191)→<br>PRK04313 (PRK04313)KOW_RPS4 (cd06087)S4 (pfam01479)Ribosomal_S4e (pfam00900)→<br>RplE (COG0094)rpl5p (PRK04219)→ RpsN (COG0199)rps14P (PRK05766)→<br>rps8p (PRK04034)RpsH (COG0096)→ RplF (COG0097)rpl6p (PRK05518)→<br>RecA (COG0468)Rpl32e (COG1717)COG2251 (COG2251)rpl32e (PRK08562)HHH_5 (pfam14520)→<br>RecA (COG0468)rpl32e (PRK08562)Ribosomal_L32_L32e (cd00513)HHH_5 (pfam14520)→
Hth-1-1	NZ_AOMF01000153.1	+	44875	44974	**RNA**→ Methyltrn_RNA_3 (pfam02598)→ RplC (COG0087)rpl3p (PRK04231)→<br>RplD (COG0088)rpl4lp (PRK04042)→ RplW (COG0089)PRK14548 (PRK14548)→<br>RplB (COG0090)rpl2p (PRK09612)→ RpsS (COG0185)rps19p (PRK04038)→<br>rpl22p (PRK04223)RplV (COG0091)→<br>RpsC (COG0092)Lig (COG0272)rps3p (PRK04191)Nop14 (pfam04147)HHH_5 (pfam14520)→<br>PRK14549 (PRK14549)Ribosomal_L29 (pfam00831)→ PRK03879 (PRK03879)UPF0086 (pfam01868)→<br>RpsQ (COG0186)rps17p (PRK08572)→ rpl14p (PRK08571)Ribosomal_L14 (pfam00238)→<br>RplX (COG0198)rpl24p (PRK01191)→<br>PRK04313 (PRK04313)KOW_RPS4 (cd06087)Ribosomal_S4e (pfam00900)→ RplE (COG0094)rpl5p (PRK04219)→<br>RpsN (COG0199)rps14P (PRK05766)→ rps8p (PRK04034)RpsH (COG0096)→<br>RplF (COG0097)rpl6p (PRK05518)→<br>NT_POLXc (cd00141)Rpl32e (COG1717)rpl32e (PRK08562)HHH_5 (pfam14520)→<br>RecA (COG0468)Rpl32e (COG1717)rpl32e (PRK08562)HHH_5 (pfam14520)→
Hmo-1-1	NZ_AOMC01000109.1	-	103550	103451	**RNA**→ Methyltrn_RNA_3 (pfam02598)→ RplC (COG0087)rpl3p (PRK04231)→<br>RplD (COG0088)rpl4lp (PRK04042)→ RplW (COG0089)PRK14548 (PRK14548)→<br>RplB (COG0090)rpl2p (PRK09612)→ RpsS (COG0185)rps19p (PRK04038)→<br>rpl22p (PRK04223)RplV (COG0091)→<br>RpsC (COG0092)UvrC (COG0322)rps3p (PRK04191)HHH_5 (pfam14520)→<br>PRK14549 (PRK14549)Ribosomal_L29 (pfam00831)→ PRK03879 (PRK03879)UPF0086 (pfam01868)→<br>RpsQ (COG0186)rps17p (PRK08572)→ rpl14p (PRK08571)Ribosomal_L14 (pfam00238)→<br>rpl24p (PRK01191)KOW_RPL26 (cd06089)→<br>PRK04313 (PRK04313)KOW_RPS4 (cd06087)Ribosomal_S4e (pfam00900)→ RplE (COG0094)rpl5p (PRK04219)→<br>RpsN (COG0199)rps14P (PRK05766)→ rps8p (PRK04034)RpsH (COG0096)→<br>rpl6p (PRK05518)Ribosomal_L6 (pfam00347)→
env-206	P_1C_Sed_2_UnCty_1102543	+	116	215	**RNA**→ Methyltrn_RNA_3 (pfam02598)→
env-207	APHM01019633.1	+	691	784	**RNA**→ Methyltrn_RNA_3 (pfam02598)→ rpl3p (PRK04231)Ribosomal_L3 (pfam00297)→
env-208	LTJ07As871_J07ABscf090118	-	8900	8807	**RNA**→ Methyltrn_RNA_3 (pfam02598)→ rpl3p (PRK04231)Ribosomal_L3 (pfam00297)→
env-209	P_1C_Sed_2_UnCty_1164547	+	86	185	**RNA**→ Methyltrn_RNA_3 (pfam02598)→
Nta-1-1	NZ_AOIL01000042.1	+	111800	111899	**RNA**→ Methyltrn_RNA_3 (pfam02598)→ rpl3p (PRK04231)Ribosomal_L3 (pfam00297)→<br>RplD (COG0088)rpl4lp (PRK04042)→ RplW (COG0089)PRK14548 (PRK14548)→<br>RplB (COG0090)rpl2p (PRK09612)→ RpsS (COG0185)rps19p (PRK04038)→<br>rpl22p (PRK04223)RplV (COG0091)→ RpsC (COG0092)rps3p (PRK04191)→<br>RpmC (COG0255)PRK14549 (PRK14549)→ PRK03879 (PRK03879)UPF0086 (pfam01868)→<br>RpsQ (COG0186)rps17p (PRK08572)→ rpl14p (PRK08571)Ribosomal_L14 (pfam00238)→<br>rpl24p (PRK01191)KOW_RPL26 (cd06089)→<br>PRK04313 (PRK04313)KOW_RPS4 (cd06087)Ribosomal_S4e (pfam00900)→ RplE (COG0094)rpl5p (PRK04219)→<br>RpsN (COG0199)rps14P (PRK05766)→ rps8p (PRK04034)RpsH (COG0096)→<br>RplF (COG0097)rpl6p (PRK05518)→ PRK12766 (PRK12766)RecA (COG0468)Rpl32e (COG1717)→

Nas-1-1	NZ_AOIO01000023.1	-	59520	59421	**RNA**→ Methyltrn_RNA_3 (pfam02598)→ rpl3p (PRK04231)Ribosomal_L3 (pfam00297)→<br>RplD (COG0088)rpl4lp (PRK04042)→ RplW (COG0089)PRK14548 (PRK14548)→<br>RplB (COG0090)rpl2p (PRK09612)→ RpsS (COG0185)rps19p (PRK04038)→<br>rpl22p (PRK04223)RplV (COG0091)→ RpsC (COG0092)rps3p (PRK04191)→<br>RpmC (COG0255)PRK14549 (PRK14549)→ PRK03879 (PRK03879)UPF0086 (pfam01868)→<br>RpsQ (COG0186)rps17p (PRK08572)→ rpl14p (PRK08571)Ribosomal_L14 (pfam00238)→<br>rpl24p (PRK01191)KOW_RPL26 (cd06089)→<br>PRK04313 (PRK04313)KOW_RPS4 (cd06087)Ribosomal_S4e (pfam00900)→ RplE (COG0094)rpl5p (PRK04219)→<br>RpsN (COG0199)rps14P (PRK05766)→ rps8p (PRK04034)RpsH (COG0096)→<br>RplF (COG0097)rpl6p (PRK05518)→ PRK12766 (PRK12766)RecA (COG0468)Rpl32e (COG1717)→
Noc-1-1	NC_019974.1	-	3406135	3406036	**RNA**→ Methyltrn_RNA_3 (pfam02598)→ rpl3p (PRK04231)Ribosomal_L3 (pfam00297)→<br>RplD (COG0088)rpl4lp (PRK04042)→ RplW (COG0089)PRK14548 (PRK14548)→<br>RplB (COG0090)rpl2p (PRK09612)→ RpsS (COG0185)rps19p (PRK04038)→<br>rpl22p (PRK04223)RplV (COG0091)→ RpsC (COG0092)rps3p (PRK04191)→<br>PRK14549 (PRK14549)Ribosomal_L29 (pfam00831)→ PRK03879 (PRK03879)UPF0086 (pfam01868)→<br>RpsQ (COG0186)rps17p (PRK08572)→ rpl14p (PRK08571)Ribosomal_L14 (pfam00238)→<br>rpl24p (PRK01191)KOW_RPL26 (cd06089)→<br>PRK04313 (PRK04313)KOW_RPS4 (cd06087)S4 (pfam01479)Ribosomal_S4e (pfam00900)→<br>RplE (COG0094)rpl5p (PRK04219)→ RpsN (COG0199)rps14P (PRK05766)→<br>rps8p (PRK04034)RpsH (COG0096)→ RplF (COG0097)rpl6p (PRK05518)→<br>UvrC (COG0322)Rpl32e (COG1717)rpl32e (PRK08562)HHH_5 (pfam14520)→
Nje-1-1	NZ_AOIA01000019.1	+	36830	36929	**RNA**→ Methyltrn_RNA_3 (pfam02598)→ rpl3p (PRK04231)Ribosomal_L3 (pfam00297)→<br>RplD (COG0088)rpl4lp (PRK04042)→ RplW (COG0089)PRK14548 (PRK14548)→<br>RplB (COG0090)rpl2p (PRK09612)→ RpsS (COG0185)rps19p (PRK04038)→<br>rpl22p (PRK04223)RplV (COG0091)→ RpsC (COG0092)rps3p (PRK04191)→<br>PRK14549 (PRK14549)Ribosomal_L29 (pfam00831)→ PRK03879 (PRK03879)UPF0086 (pfam01868)→<br>RpsQ (COG0186)rps17p (PRK08572)→ rpl14p (PRK08571)Ribosomal_L14 (pfam00238)→<br>rpl24p (PRK01191)KOW_RPL26 (cd06089)→<br>PRK04313 (PRK04313)KOW_RPS4 (cd06087)S4 (pfam01479)Ribosomal_S4e (pfam00900)→<br>RplE (COG0094)rpl5p (PRK04219)→ RpsN (COG0199)rps14P (PRK05766)→<br>rps8p (PRK04034)RpsH (COG0096)→ RplF (COG0097)rpl6p (PRK05518)→<br>RecA (COG0468)Rpl32e (COG1717)HHH_5 (pfam14520)→
Nam-1-1	NZ_AOIB01000037.1	-	63552	63453	**RNA**→ Methyltrn_RNA_3 (pfam02598)→ rpl3p (PRK04231)Ribosomal_L3 (pfam00297)→<br>RplD (COG0088)rpl4lp (PRK04042)→ RplW (COG0089)PRK14548 (PRK14548)→<br>RplB (COG0090)rpl2p (PRK09612)→ RpsS (COG0185)rps19p (PRK04038)→<br>rpl22p (PRK04223)RplV (COG0091)→ RpsC (COG0092)rps3p (PRK04191)→<br>PRK14549 (PRK14549)Ribosomal_L29 (pfam00831)→ PRK03879 (PRK03879)UPF0086 (pfam01868)→<br>RpsQ (COG0186)rps17p (PRK08572)→ rpl14p (PRK08571)Ribosomal_L14 (pfam00238)→<br>rpl24p (PRK01191)KOW_RPL26 (cd06089)→<br>PRK04313 (PRK04313)KOW_RPS4 (cd06087)S4 (pfam01479)Ribosomal_S4e (pfam00900)→<br>RplE (COG0094)rpl5p (PRK04219)→ RpsN (COG0199)rps14P (PRK05766)→<br>rps8p (PRK04034)RpsH (COG0096)→ RplF (COG0097)rpl6p (PRK05518)→<br>UvrC (COG0322)Rpl32e (COG1717)rpl32e (PRK08562)HHH_5 (pfam14520)→<br>rpl19e (PRK08570)Ribosomal_L19e (pfam01280)→
env-210	SL_5KL_010_BRINE_10002219	+	679	778	**RNA**→ Methyltrn_RNA_3 (pfam02598)→ rpl3p (PRK04231)Ribosomal_L3 (pfam00297)→<br>RplD (COG0088)rpl4lp (PRK04042)→ RplW (COG0089)PRK14548 (PRK14548)→<br>RplB (COG0090)rpl2p (PRK09612)→ RpsS (COG0185)rps19p (PRK04038)→<br>rpl22p (PRK04223)RplV (COG0091)→ RpsC (COG0092)rps3p (PRK04191)→<br>PRK14549 (PRK14549)Ribosomal_L29 (pfam00831)→ PRK03879 (PRK03879)UPF0086 (pfam01868)→<br>RpsQ (COG0186)rps17p (PRK08572)→ rpl14p (PRK08571)Ribosomal_L14 (pfam00238)→<br>rpl24p (PRK01191)KOW_RPL26 (cd06089)→<br>PRK04313 (PRK04313)S4 (cd00165)KOW_RPS4 (cd06087)Ribosomal_S4e (pfam00900)→<br>RplE (COG0094)rpl5p (PRK04219)SDH (cd12188)→ RpsN (COG0199)rps14P (PRK05766)→ rps8p (PRK04034)→
env-211	SL_5KL_010_BRINE_10004382	+	5582	5681	**RNA**→ Methyltrn_RNA_3 (pfam02598)→ rpl3p (PRK04231)Ribosomal_L3 (pfam00297)→

env-212	SL_6KL_011_BRINE_1000450	+	5568	5667	**RNA**→ Methyltrn_RNA_3 (pfam02598)→ rpl3p (PRK04231)Ribosomal_L3 (pfam00297)→ RplD (COG0088)rpl4lp (PRK04042)→ RplW (COG0089)PRK14548 (PRK14548)→ RplB (COG0090)rpl2p (PRK09612)→ RpsS (COG0185)rps19p (PRK04038)→ rpl22p (PRK04223)RplV (COG0091)→ RpsC (COG0092)rps3p (PRK04191)→ PRK14549 (PRK14549)Ribosomal_L29 (pfam00831)→ PRK03879 (PRK03879)UPF0086 (pfam01868)→ RpsQ (COG0186)rps17p (PRK08572)→ rpl14p (PRK08571)Ribosomal_L14 (pfam00238)→ rpl24p (PRK01191)KOW_RPL26 (cd06089)→ PRK04313 (PRK04313)S4 (cd00165)KOW_RPS4 (cd06087)Ribosomal_S4e (pfam00900)→ RplE (COG0094)rpl5p (PRK04219)SDH (cd12188)→ RpsN (COG0199)rps14P (PRK05766)→ rps8p (PRK04034)RpsH (COG0096)→ RplF (COG0097)rpl6p (PRK05518)→ RecA (COG0468)Rpl32e (COG1717)rpl32e (PRK08562)HHH_5 (pfam14520)→ rpl19e (PRK08570)Ribosomal_L19e (pfam01280)→ RplR (COG0256)rpl18p (PRK08569)→ RpsE (COG0098)rps5p (PRK04044)→ Ribosomal_L7_archeal_euk (cd01657)rpl30p (PRK06049)→ rpl15p (PRK06419)Ribosomal_L27A (pfam00828)→ Plug_translocon (pfam10559)→
env-213	SL_4KL_010_BRINE_10000109	+	5553	5652	**RNA**→ Methyltrn_RNA_3 (pfam02598)→ rpl3p (PRK04231)Ribosomal_L3 (pfam00297)→ RplD (COG0088)rpl4lp (PRK04042)→ RplW (COG0089)PRK14548 (PRK14548)→ RplB (COG0090)rpl2p (PRK09612)→ RpsS (COG0185)rps19p (PRK04038)→ rpl22p (PRK04223)RplV (COG0091)→ RpsC (COG0092)rps3p (PRK04191)→ PRK14549 (PRK14549)Ribosomal_L29 (pfam00831)→ PRK03879 (PRK03879)UPF0086 (pfam01868)→ RpsQ (COG0186)rps17p (PRK08572)→ rpl14p (PRK08571)Ribosomal_L14 (pfam00238)→ rpl24p (PRK01191)KOW_RPL26 (cd06089)→ PRK04313 (PRK04313)S4 (cd00165)KOW_RPS4 (cd06087)Ribosomal_S4e (pfam00900)→ RplE (COG0094)rpl5p (PRK04219)SDH (cd12188)→ RpsN (COG0199)rps14P (PRK05766)→ rps8p (PRK04034)RpsH (COG0096)→ RplF (COG0097)rpl6p (PRK05518)→ RecA (COG0468)Rpl32e (COG1717)rpl32e (PRK08562)HHH_5 (pfam14520)→ rpl19e (PRK08570)Ribosomal_L19e (pfam01280)→ RplR (COG0256)rpl18p (PRK08569)→ RpsE (COG0098)rps5p (PRK04044)→ Ribosomal_L7_archeal_euk (cd01657)rpl30p (PRK06049)→ rpl15p (PRK06419)Ribosomal_L27A (pfam00828)→ SecY (COG0201)PRK08568 (PRK08568)→
Hsp-3-1	NC_002607.1	+	1258523	1258622	**RNA**→ MTH1 (COG2106)→ rpl3p (PRK04231)Ribosomal_L3 (pfam00297)→ RplD (COG0088)rpl4lp (PRK04042)→ RplW (COG0089)PRK14548 (PRK14548)→ RplB (COG0090)rpl2p (PRK09612)→ RpsS (COG0185)rps19p (PRK04038)→ rpl22p (PRK04223)RplV (COG0091)→ RpsC (COG0092)rps3p (PRK04191)→ RpmC (COG0255)PRK14549 (PRK14549)→ POP4 (COG1588)PRK03879 (PRK03879)→ RpsQ (COG0186)rps17p (PRK08572)→ rpl14p (PRK08571)Ribosomal_L14 (pfam00238)→ RplX (COG0198)rpl24p (PRK01191)→ PRK04313 (PRK04313)KOW_RPS4 (cd06087)Ribosomal_S4e (pfam00900)→ RplE (COG0094)rpl5p (PRK04219)→ RpsN (COG0199)rps14P (PRK05766)→ rps8p (PRK04034)RpsH (COG0096)→ rps8p (PRK04034)RpsH (COG0096)→ RplF (COG0097)rpl6p (PRK05518)→ PRK12766 (PRK12766)RecA (COG0468)Rpl32e (COG1717)→ rpl19e (PRK08570)Ribosomal_L19e (pfam01280)→
Hsa-3-1	NC_010364.1	+	1247763	1247862	**RNA**→ MTH1 (COG2106)→ rpl3p (PRK04231)Ribosomal_L3 (pfam00297)→ RplD (COG0088)rpl4lp (PRK04042)→ RplW (COG0089)PRK14548 (PRK14548)→ RplB (COG0090)rpl2p (PRK09612)→ RpsS (COG0185)rps19p (PRK04038)→ rpl22p (PRK04223)RplV (COG0091)→ RpsC (COG0092)rps3p (PRK04191)→ RpmC (COG0255)PRK14549 (PRK14549)→ POP4 (COG1588)PRK03879 (PRK03879)→ RpsQ (COG0186)rps17p (PRK08572)→ rpl14p (PRK08571)Ribosomal_L14 (pfam00238)→ RplX (COG0198)rpl24p (PRK01191)→ PRK04313 (PRK04313)KOW_RPS4 (cd06087)Ribosomal_S4e (pfam00900)→ RplE (COG0094)rpl5p (PRK04219)→ RpsN (COG0199)rps14P (PRK05766)→ rps8p (PRK04034)RpsH (COG0096)→ RplF (COG0097)rpl6p (PRK05518)→ PRK12766 (PRK12766)RecA (COG0468)Rpl32e (COG1717)→ rpl19e (PRK08570)Ribosomal_L19e (pfam01280)→

Nch-1-1	NZ_AOIN01000067.1	+	25511	25610	**RNA**→ Methyltrn_RNA_3 (pfam02598)→ rpl3p (PRK04231)Ribosomal_L3 (pfam00297)→<br>RplD (COG0088)rpl4lp (PRK04042)→ RplW (COG0089)PRK14548 (PRK14548)→<br>RplB (COG0090)rpl2p (PRK09612)→ RpsS (COG0185)rps19p (PRK04038)→<br>rpl22p (PRK04223)RplV (COG0091)→ RpsC (COG0092)rps3p (PRK04191)→<br>PRK14549 (PRK14549)Ribosomal_L29 (pfam00831)→ PRK03879 (PRK03879)UPF0086 (pfam01868)→<br>RpsQ (COG0186)rps17p (PRK08572)→ rpl14p (PRK08571)Ribosomal_L14 (pfam00238)→<br>rpl24p (PRK01191)KOW_RPL26 (cd06089)→<br>PRK04313 (PRK04313)KOW_RPS4 (cd06087)S4 (pfam01479)Ribosomal_S4e (pfam00900)→<br>RplE (COG0094)rpl5p (PRK04219)→ RpsN (COG0199)rps14P (PRK05766)→<br>rps8p (PRK04034)RpsH (COG0096)→ RplF (COG0097)rpl6p (PRK05518)→<br>PRK12766 (PRK12766)RecA (COG0468)Rpl32e (COG1717)→
Nae-1-1	NZ_AOIP01000033.1	-	55488	55389	**RNA**→ PRK13493 (PRK13493)Methyltrn_RNA_3 (pfam02598)→ rpl3p (PRK04231)Ribosomal_L3 (pfam00297)→<br>RplD (COG0088)rpl4lp (PRK04042)→ RplW (COG0089)PRK14548 (PRK14548)→<br>RplB (COG0090)rpl2p (PRK09612)→ RpsS (COG0185)rps19p (PRK04038)→<br>rpl22p (PRK04223)RplV (COG0091)→ RpsC (COG0092)rps3p (PRK04191)→<br>RpmC (COG0255)PRK14549 (PRK14549)→ PRK03879 (PRK03879)UPF0086 (pfam01868)→<br>RpsQ (COG0186)rps17p (PRK08572)→ rpl14p (PRK08571)Ribosomal_L14 (pfam00238)→<br>rpl24p (PRK01191)KOW_RPL26 (cd06089)→<br>PRK04313 (PRK04313)KOW_RPS4 (cd06087)Ribosomal_S4e (pfam00900)→ RplE (COG0094)rpl5p (PRK04219)→<br>RpsN (COG0199)rps14P (PRK05766)→ rps8p (PRK04034)RpsH (COG0096)→<br>RplF (COG0097)rpl6p (PRK05518)→ PRK12766 (PRK12766)RecA (COG0468)Rpl32e (COG1717)→
env-214	SS_3KL_010_SOIL_10059833	-	123	24	**RNA**→ Methyltrn_RNA_3 (pfam02598)→
env-215	SL_3KL_010_SED_10538221	+	53	152	**RNA**→ MTH1 (COG2106)→
env-216	P_1C_Sed_2_UnCty_1120614	-	293	194	**RNA**→ Methyltrn_RNA_3 (pfam02598)→
Hni-1-1	NZ_AOMA01000004.1	+	593	692	**RNA**→ Methyltrn_RNA_3 (pfam02598)→ rpl3p (PRK04231)Ribosomal_L3 (pfam00297)→<br>RplD (COG0088)rpl4lp (PRK04042)→ RplW (COG0089)PRK14548 (PRK14548)→<br>RplB (COG0090)rpl2p (PRK09612)→ RpsS (COG0185)rps19p (PRK04038)→<br>rpl22p (PRK04223)RplV (COG0091)→ RpsC (COG0092)rps3p (PRK04191)→<br>RpmC (COG0255)PRK14549 (PRK14549)→ PRK03879 (PRK03879)UPF0086 (pfam01868)→<br>RpsQ (COG0186)rps17p (PRK08572)→ rpl14p (PRK08571)Ribosomal_L14 (pfam00238)→<br>rpl24p (PRK01191)KOW_RPL26 (cd06089)→<br>PRK04313 (PRK04313)KOW_RPS4 (cd06087)Ribosomal_S4e (pfam00900)→ RplE (COG0094)rpl5p (PRK04219)→<br>RpsN (COG0199)rps14P (PRK05766)→ rps8p (PRK04034)RpsH (COG0096)→<br>RplF (COG0097)rpl6p (PRK05518)→ UvrC (COG0322)HHH_5 (pfam14520)→ hypo→
Has-1-1	NZ_AOIQ01000021.1	+	411355	411454	**RNA**→ Methyltrn_RNA_3 (pfam02598)→ RplC (COG0087)rpl3p (PRK04231)→<br>RplD (COG0088)rpl4lp (PRK04042)→ RplW (COG0089)PRK14548 (PRK14548)→<br>RplB (COG0090)rpl2p (PRK09612)→ RpsS (COG0185)rps19p (PRK04038)→<br>rpl22p (PRK04223)RplV (COG0091)→ RpsC (COG0092)rps3p (PRK04191)→<br>RpmC (COG0255)PRK14549 (PRK14549)→ PRK03879 (PRK03879)UPF0086 (pfam01868)→<br>RpsQ (COG0186)rps17p (PRK08572)→ rpl14p (PRK08571)Ribosomal_L14 (pfam00238)→<br>rpl24p (PRK01191)KOW_RPL26 (cd06089)→<br>PRK04313 (PRK04313)KOW_RPS4 (cd06087)Ribosomal_S4e (pfam00900)→ RplE (COG0094)rpl5p (PRK04219)→<br>RpsN (COG0199)rps14P (PRK05766)→ rps8p (PRK04034)RpsH (COG0096)→<br>RplF (COG0097)rpl6p (PRK05518)→<br>UvrC (COG0322)Rpl32e (COG1717)rpl32e (PRK08562)HHH_5 (pfam14520)→<br>rpl19e (PRK08570)Ribosomal_L19e (pfam01280)→
env-217	2140918027-11734	-	146	47	**RNA**→ Methyltrn_RNA_3 (pfam02598)→

Nal-2-1	NZ_JNCS01000001.1	-	130682	130583	**RNA**→ Methyltrn_RNA_3 (pfam02598)→ rpl3p (PRK04231)Ribosomal_L3 (pfam00297)→<br>RplD (COG0088)rpl4lp (PRK04042)→ RplW (COG0089)PRK14548 (PRK14548)→<br>RplB (COG0090)rpl2p (PRK09612)→ RpsS (COG0185)rps19p (PRK04038)→<br>rpl22p (PRK04223)RplV (COG0091)→ RpsC (COG0092)rps3p (PRK04191)→<br>RpmC (COG0255)PRK14549 (PRK14549)→ PRK03879 (PRK03879)UPF0086 (pfam01868)→<br>RpsQ (COG0186)rps17p (PRK08572)→ rpl14p (PRK08571)Ribosomal_L14 (pfam00238)→<br>rpl24p (PRK01191)KOW_RPL26 (cd06089)→<br>PRK04313 (PRK04313)KOW_RPS4 (cd06087)Ribosomal_S4e (pfam00900)→ RplE (COG0094)rpl5p (PRK04219)→<br>RpsN (COG0199)rps14P (PRK05766)→ rps8p (PRK04034)RpsH (COG0096)→<br>RplF (COG0097)rpl6p (PRK05518)→ PRK12766 (PRK12766)UvrC (COG0322)Rpl32e (COG1717)→
Nal-1-1	NZ_AOIK01000025.1	+	71137	71236	**RNA**→ Methyltrn_RNA_3 (pfam02598)→ rpl3p (PRK04231)Ribosomal_L3 (pfam00297)→<br>RplD (COG0088)rpl4lp (PRK04042)→ RplW (COG0089)PRK14548 (PRK14548)→<br>RplB (COG0090)rpl2p (PRK09612)→ RpsS (COG0185)rps19p (PRK04038)→<br>rpl22p (PRK04223)RplV (COG0091)→ RpsC (COG0092)rps3p (PRK04191)→<br>RpmC (COG0255)PRK14549 (PRK14549)→ PRK03879 (PRK03879)UPF0086 (pfam01868)→<br>RpsQ (COG0186)rps17p (PRK08572)→ rpl14p (PRK08571)Ribosomal_L14 (pfam00238)→<br>rpl24p (PRK01191)KOW_RPL26 (cd06089)→<br>PRK04313 (PRK04313)KOW_RPS4 (cd06087)Ribosomal_S4e (pfam00900)→ RplE (COG0094)rpl5p (PRK04219)→<br>RpsN (COG0199)rps14P (PRK05766)→ rps8p (PRK04034)RpsH (COG0096)→<br>RplF (COG0097)rpl6p (PRK05518)→ PRK12766 (PRK12766)UvrC (COG0322)Rpl32e (COG1717)→
Npa-1-1	NZ_AOII01000067.1	-	29396	29297	**RNA**→ Methyltrn_RNA_3 (pfam02598)→ rpl3p (PRK04231)Ribosomal_L3 (pfam00297)→<br>RplD (COG0088)rpl4lp (PRK04042)→ RplW (COG0089)PRK14548 (PRK14548)→<br>RplB (COG0090)rpl2p (PRK09612)→ RpsS (COG0185)rps19p (PRK04038)→<br>rpl22p (PRK04223)RplV (COG0091)→ RpsC (COG0092)rps3p (PRK04191)→<br>RpmC (COG0255)PRK14549 (PRK14549)→ PRK03879 (PRK03879)UPF0086 (pfam01868)→<br>RpsQ (COG0186)rps17p (PRK08572)→ rpl14p (PRK08571)Ribosomal_L14 (pfam00238)→<br>rpl24p (PRK01191)KOW_RPL26 (cd06089)→<br>PRK04313 (PRK04313)KOW_RPS4 (cd06087)Ribosomal_S4e (pfam00900)→ RplE (COG0094)rpl5p (PRK04219)→<br>RpsN (COG0199)rps14P (PRK05766)→ rps8p (PRK04034)RpsH (COG0096)→<br>RplF (COG0097)rpl6p (PRK05518)→<br>UvrC (COG0322)Rpl32e (COG1717)rpl32e (PRK08562)HHH_5 (pfam14520)→
Nga-1-1	NZ_AOIJ01000042.1	-	42266	42167	**RNA**→ Methyltrn_RNA_3 (pfam02598)→ rpl3p (PRK04231)Ribosomal_L3 (pfam00297)→<br>RplD (COG0088)rpl4lp (PRK04042)→ RplW (COG0089)PRK14548 (PRK14548)→<br>RplB (COG0090)rpl2p (PRK09612)→ RpsS (COG0185)rps19p (PRK04038)→<br>rpl22p (PRK04223)RplV (COG0091)→ RpsC (COG0092)rps3p (PRK04191)→<br>RpmC (COG0255)PRK14549 (PRK14549)→ PRK03879 (PRK03879)UPF0086 (pfam01868)→<br>RpsQ (COG0186)rps17p (PRK08572)→ rpl14p (PRK08571)Ribosomal_L14 (pfam00238)→<br>rpl24p (PRK01191)KOW_RPL26 (cd06089)→<br>PRK04313 (PRK04313)KOW_RPS4 (cd06087)Ribosomal_S4e (pfam00900)→ RplE (COG0094)rpl5p (PRK04219)→<br>RpsN (COG0199)rps14P (PRK05766)→ rps8p (PRK04034)RpsH (COG0096)→<br>RplF (COG0097)rpl6p (PRK05518)→<br>RecA (COG0468)Rpl32e (COG1717)rpl32e (PRK08562)HHH_5 (pfam14520)→<br>rpl19e (PRK08570)Ribosomal_L19e (pfam01280)→
Nsp-2-1	NZ_AJVG01000002.1	-	354632	354533	**RNA**→ Methyltrn_RNA_3 (pfam02598)→ rpl3p (PRK04231)Ribosomal_L3 (pfam00297)→<br>RplD (COG0088)rpl4lp (PRK04042)→ RplW (COG0089)PRK14548 (PRK14548)→<br>RplB (COG0090)rpl2p (PRK09612)→ RpsS (COG0185)rps19p (PRK04038)→<br>rpl22p (PRK04223)RplV (COG0091)→ RpsC (COG0092)rps3p (PRK04191)→<br>RpmC (COG0255)PRK14549 (PRK14549)→ PRK03879 (PRK03879)UPF0086 (pfam01868)→<br>RpsQ (COG0186)rps17p (PRK08572)→ rpl14p (PRK08571)Ribosomal_L14 (pfam00238)→<br>rpl24p (PRK01191)KOW_RPL26 (cd06089)→<br>PRK04313 (PRK04313)KOW_RPS4 (cd06087)Ribosomal_S4e (pfam00900)→ RplE (COG0094)rpl5p (PRK04219)→<br>RpsN (COG0199)rps14P (PRK05766)→ rps8p (PRK04034)RpsH (COG0096)→<br>RplF (COG0097)rpl6p (PRK05518)→<br>RecA (COG0468)Rpl32e (COG1717)rpl32e (PRK08562)HHH_5 (pfam14520)→<br>rpl19e (PRK08570)Ribosomal_L19e (pfam01280)→

Nsp-1-1	NC_018224.1	-	1817666	1817567	**RNA**→ Methyltrn_RNA_3 (pfam02598)→ rpl3p (PRK04231)Ribosomal_L3 (pfam00297)→<br>RplD (COG0088)rpl4lp (PRK04042)→ RplW (COG0089)PRK14548 (PRK14548)→<br>RplB (COG0090)rpl2p (PRK09612)→ RpsS (COG0185)rps19p (PRK04038)→<br>rpl22p (PRK04223)RplV (COG0091)→ RpsC (COG0092)rps3p (PRK04191)→<br>RpmC (COG0255)PRK14549 (PRK14549)→ PRK03879 (PRK03879)UPF0086 (pfam01868)→<br>RpsQ (COG0186)rps17p (PRK08572)→ rpl14p (PRK08571)Ribosomal_L14 (pfam00238)→<br>rpl24p (PRK01191)KOW_RPL26 (cd06089)→<br>PRK04313 (PRK04313)KOW_RPS4 (cd06087)Ribosomal_S4e (pfam00900)→ RplE (COG0094)rpl5p (PRK04219)→<br>RpsN (COG0199)rps14P (PRK05766)→ rps8p (PRK04034)RpsH (COG0096)→<br>RplF (COG0097)rpl6p (PRK05518)→<br>RecA (COG0468)Rpl32e (COG1717)rpl32e (PRK08562)HHH_5 (pfam14520)→<br>rpl19e (PRK08570)Ribosomal_L19e (pfam01280)→
Htu-1-1	NC_013743.1	+	2295636	2295735	**RNA**→ Methyltrn_RNA_3 (pfam02598)→ rpl3p (PRK04231)Ribosomal_L3 (pfam00297)→<br>RplD (COG0088)rpl4lp (PRK04042)→ RplW (COG0089)PRK14548 (PRK14548)→<br>RplB (COG0090)rpl2p (PRK09612)→ RpsS (COG0185)rps19p (PRK04038)→<br>rpl22p (PRK04223)RplV (COG0091)→ RpsC (COG0092)rps3p (PRK04191)→<br>RpmC (COG0255)PRK14549 (PRK14549)→ PRK03879 (PRK03879)UPF0086 (pfam01868)→<br>RpsQ (COG0186)rps17p (PRK08572)→ rpl14p (PRK08571)Ribosomal_L14 (pfam00238)→<br>rpl24p (PRK01191)KOW_RPL26 (cd06089)→<br>PRK04313 (PRK04313)S4 (cd00165)KOW_RPS4 (cd06087)Ribosomal_S4e (pfam00900)→<br>RplE (COG0094)rpl5p (PRK04219)→ RpsN (COG0199)rps14P (PRK05766)→<br>rps8p (PRK04034)RpsH (COG0096)→ RplF (COG0097)rpl6p (PRK05518)→<br>PRK12766 (PRK12766)UvrC (COG0322)Rpl32e (COG1717)→ rpl19e (PRK08570)Ribosomal_L19e (pfam01280)→
Hsa-8-1	NZ_AOIS01000045.1	-	15715	15616	**RNA**→ Methyltrn_RNA_3 (pfam02598)→ rpl3p (PRK04231)Ribosomal_L3 (pfam00297)→<br>RplD (COG0088)rpl4lp (PRK04042)→ RplW (COG0089)PRK14548 (PRK14548)→<br>RplB (COG0090)rpl2p (PRK09612)→ RpsS (COG0185)rps19p (PRK04038)→<br>rpl22p (PRK04223)RplV (COG0091)→ RpsC (COG0092)rps3p (PRK04191)→<br>PRK14549 (PRK14549)Ribosomal_L29 (pfam00831)→ PRK03879 (PRK03879)UPF0086 (pfam01868)→<br>RpsQ (COG0186)rps17p (PRK08572)→ rpl14p (PRK08571)Ribosomal_L14 (pfam00238)→<br>rpl24p (PRK01191)KOW_RPL26 (cd06089)→<br>PRK04313 (PRK04313)S4 (cd00165)KOW_RPS4 (cd06087)Ribosomal_S4e (pfam00900)→<br>RplE (COG0094)rpl5p (PRK04219)→ RpsN (COG0199)rps14P (PRK05766)→<br>rps8p (PRK04034)RpsH (COG0096)→ RplF (COG0097)rpl6p (PRK05518)→<br>PRK12766 (PRK12766)UvrC (COG0322)Rpl32e (COG1717)→ rpl19e (PRK08570)Ribosomal_L19e (pfam01280)→
Hth-2-1	NZ_AOIR01000063.1	+	173689	173788	**RNA**→ Methyltrn_RNA_3 (pfam02598)→ RplC (COG0087)rpl3p (PRK04231)→<br>RplD (COG0088)rpl4lp (PRK04042)→ RplW (COG0089)PRK14548 (PRK14548)→<br>RplB (COG0090)rpl2p (PRK09612)→ RpsS (COG0185)rps19p (PRK04038)→<br>rpl22p (PRK04223)RplV (COG0091)→ RpsC (COG0092)rps3p (PRK04191)→<br>RpmC (COG0255)PRK14549 (PRK14549)→ PRK03879 (PRK03879)UPF0086 (pfam01868)→<br>RpsQ (COG0186)rps17p (PRK08572)→ rpl14p (PRK08571)Ribosomal_L14 (pfam00238)→<br>rpl24p (PRK01191)KOW_RPL26 (cd06089)→<br>PRK04313 (PRK04313)KOW_RPS4 (cd06087)Ribosomal_S4e (pfam00900)→ RplE (COG0094)rpl5p (PRK04219)→<br>RpsN (COG0199)rps14P (PRK05766)→ rps8p (PRK04034)RpsH (COG0096)→<br>RplF (COG0097)rpl6p (PRK05518)→<br>UvrC (COG0322)Rpl32e (COG1717)rpl32e (PRK08562)HHH_5 (pfam14520)→<br>rpl19e (PRK08570)Ribosomal_L19e (pfam01280)→

Hje-2-1	NZ_JDTG01000014.1	+	597395	597494	**RNA**→ Methyltrn_RNA_3 (pfam02598)→ RplC (COG0087)rpl3p (PRK04231)→<br>RplD (COG0088)rpl4lp (PRK04042)→ RplW (COG0089)PRK14548 (PRK14548)→<br>RplB (COG0090)rpl2p (PRK09612)→ RpsS (COG0185)rps19p (PRK04038)→<br>rpl22p (PRK04223)RplV (COG0091)→ RpsC (COG0092)rps3p (PRK04191)→<br>RpmC (COG0255)PRK14549 (PRK14549)→ PRK03879 (PRK03879)UPF0086 (pfam01868)→<br>RpsQ (COG0186)rps17p (PRK08572)→ rpl14p (PRK08571)Ribosomal_L14 (pfam00238)→<br>rpl24p (PRK01191)KOW_RPL26 (cd06089)→<br>PRK04313 (PRK04313)KOW_RPS4 (cd06087)Ribosomal_S4e (pfam00900)→ RplE (COG0094)rpl5p (PRK04219)→<br>RpsN (COG0199)rps14P (PRK05766)→ rps8p (PRK04034)RpsH (COG0096)→<br>RplF (COG0097)rpl6p (PRK05518)→<br>UvrC (COG0322)Rpl32e (COG1717)rpl32e (PRK08562)HHH_5 (pfam14520)→<br>rpl19e (PRK08570)Ribosomal_L19e (pfam01280)→
Nve-1-1	NZ_AOID01000027.1	+	70079	70178	**RNA**→ Methyltrn_RNA_3 (pfam02598)→ rpl3p (PRK04231)Ribosomal_L3 (pfam00297)→<br>RplD (COG0088)rpl4lp (PRK04042)→ RplW (COG0089)PRK14548 (PRK14548)→<br>RplB (COG0090)rpl2p (PRK09612)→ RpsS (COG0185)rps19p (PRK04038)→<br>rpl22p (PRK04223)RplV (COG0091)→ RpsC (COG0092)rps3p (PRK04191)→<br>PRK14549 (PRK14549)Ribosomal_L29 (pfam00831)→ PRK03879 (PRK03879)UPF0086 (pfam01868)→<br>RpsQ (COG0186)rps17p (PRK08572)→ rpl14p (PRK08571)Ribosomal_L14 (pfam00238)→<br>rpl24p (PRK01191)KOW_RPL26 (cd06089)→<br>PRK04313 (PRK04313)KOW_RPS4 (cd06087)Ribosomal_S4e (pfam00900)→ RplE (COG0094)rpl5p (PRK04219)→<br>RpsN (COG0199)rps14P (PRK05766)→ rps8p (PRK04034)RpsH (COG0096)→<br>RplF (COG0097)rpl6p (PRK05518)→ PRK12766 (PRK12766)UvrC (COG0322)Rpl32e (COG1717)→
Hli-3-1	NZ_AOIT01000030.1	+	41883	41982	**RNA**→ Methyltrn_RNA_3 (pfam02598)→ rpl3p (PRK04231)Ribosomal_L3 (pfam00297)→<br>RplD (COG0088)rpl4lp (PRK04042)→ RplW (COG0089)PRK14548 (PRK14548)→<br>RplB (COG0090)rpl2p (PRK09612)→ RpsS (COG0185)rps19p (PRK04038)→<br>rpl22p (PRK04223)RplV (COG0091)→ RpsC (COG0092)rps3p (PRK04191)→<br>RpmC (COG0255)PRK14549 (PRK14549)→ PRK03879 (PRK03879)UPF0086 (pfam01868)→<br>RpsQ (COG0186)rps17p (PRK08572)→ rpl14p (PRK08571)Ribosomal_L14 (pfam00238)→<br>rpl24p (PRK01191)KOW_RPL26 (cd06089)→<br>PRK04313 (PRK04313)S4 (cd00165)KOW_RPS4 (cd06087)Ribosomal_S4e (pfam00900)→<br>RplE (COG0094)rpl5p (PRK04219)→ RpsN (COG0199)rps14P (PRK05766)→<br>rps8p (PRK04034)RpsH (COG0096)→ RplF (COG0097)rpl6p (PRK05518)→<br>RecA (COG0468)rpl32e (PRK08562)Ribosomal_L32e (pfam01655)HHH_5 (pfam14520)→<br>rpl19e (PRK08570)Ribosomal_L19e (pfam01280)→
Hxa-1-1	NC_015666.1	-	345106	345007	**RNA**→ Methyltrn_RNA_3 (pfam02598)→ rpl3p (PRK04231)Ribosomal_L3 (pfam00297)→<br>RplD (COG0088)rpl4lp (PRK04042)→ RplW (COG0089)PRK14548 (PRK14548)→<br>RplB (COG0090)rpl2p (PRK09612)→ RpsS (COG0185)rps19p (PRK04038)→<br>rpl22p (PRK04223)RplV (COG0091)→ RpsC (COG0092)rps3p (PRK04191)→<br>RpmC (COG0255)PRK14549 (PRK14549)→ PRK03879 (PRK03879)UPF0086 (pfam01868)→<br>RpsQ (COG0186)rps17p (PRK08572)→ rpl14p (PRK08571)Ribosomal_L14 (pfam00238)→<br>rpl24p (PRK01191)KOW_RPL26 (cd06089)→<br>PRK04313 (PRK04313)S4 (cd00165)KOW_RPS4 (cd06087)Ribosomal_S4e (pfam00900)→<br>RplE (COG0094)rpl5p (PRK04219)→ RpsN (COG0199)rps14P (PRK05766)→<br>rps8p (PRK04034)RpsH (COG0096)→ RplF (COG0097)rpl6p (PRK05518)→<br>RecA (COG0468)Rpl32e (COG1717)rpl32e (PRK08562)HHH_5 (pfam14520)→<br>rpl19e (PRK08570)Ribosomal_L19e (pfam01280)→

Hgo-1-1	NZ_HG315690.1	+	2029000	2029099	**RNA**→ Methyltrn_RNA_3 (pfam02598)→ rpl3p (PRK04231)Ribosomal_L3 (pfam00297)→
					RplD (COG0088)rpl4lp (PRK04042)→ RplW (COG0089)PRK14548 (PRK14548)→
					RplB (COG0090)rpl2p (PRK09612)→ RpsS (COG0185)rps19p (PRK04038)→
					rpl22p (PRK04223)RplV (COG0091)→ RpsC (COG0092)rps3p (PRK04191)→
					RpmC (COG0255)PRK14549 (PRK14549)→ PRK03879 (PRK03879)UPF0086 (pfam01868)→
					RpsQ (COG0186)rps17p (PRK08572)→ rpl14p (PRK08571)Ribosomal_L14 (pfam00238)→
					rpl24p (PRK01191)KOW_RPL26 (cd06089)→
					PRK04313 (PRK04313)KOW_RPS4 (cd06087)Ribosomal_S4e (pfam00900)→ RplE (COG0094)rpl5p (PRK04219)→
					RpsN (COG0199)rps14P (PRK05766)→ rps8p (PRK04034)RpsH (COG0096)→
					RplF (COG0097)rpl6p (PRK05518)→
					UvrC (COG0322)Rpl32e (COG1717)rpl32e (PRK08562)HHH_5 (pfam14520)→
env-218	SL_5KL_010_BRINE_10298960	+	132	231	**RNA**→ Methyltrn_RNA_3 (pfam02598)→
env-219	SL_4KL_010_BRINE_10234182	+	69	168	**RNA**→ MTH1 (COG2106)→
env-220	GSLNARP_GFPJP1N02GH0H6	+	114	213	**RNA**→ MTH1 (COG2106)→
env-221	SL_5KL_010_BRINE_10027796	+	1534	1633	**RNA**→ Methyltrn_RNA_3 (pfam02598)→
Hsa-6-1	NZ_ALJD01000008.1	+	211183	211282	**RNA**→ Methyltrn_RNA_3 (pfam02598)→ Methyltrn_RNA_3 (pfam02598)→ RplC (COG0087)rpl3p (PRK04231)→
					RplC (COG0087)rpl3p (PRK04231)→ RplD (COG0088)rpl4lp (PRK04042)→
					RplD (COG0088)rpl4lp (PRK04042)→ RplW (COG0089)PRK14548 (PRK14548)→
					RplW (COG0089)PRK14548 (PRK14548)→ RplB (COG0090)rpl2p (PRK09612)→
					RplB (COG0090)rpl2p (PRK09612)→ RpsS (COG0185)rps19p (PRK04038)→
					RpsS (COG0185)rps19p (PRK04038)→ rpl22p (PRK04223)RplV (COG0091)→
					rpl22p (PRK04223)RplV (COG0091)→ RpsC (COG0092)rps3p (PRK04191)→
					RpsC (COG0092)rps3p (PRK04191)→ PRK14549 (PRK14549)Ribosomal_L29 (pfam00831)→
					PRK14549 (PRK14549)Ribosomal_L29 (pfam00831)→ PRK03879 (PRK03879)UPF0086 (pfam01868)→
					PRK03879 (PRK03879)UPF0086 (pfam01868)→ RpsQ (COG0186)rps17p (PRK08572)→
					rps17p (PRK08572)Ribosomal_S17 (pfam00366)→ rps17p (PRK08572)Ribosomal_S17 (pfam00366)→
					rpl14p (PRK08571)Ribosomal_L14 (pfam00238)→ rpl14p (PRK08571)Ribosomal_L14 (pfam00238)→
					rpl24p (PRK01191)KOW_RPL26 (cd06089)→ rpl24p (PRK01191)KOW_RPL26 (cd06089)→
					PRK04313 (PRK04313)KOW_RPS4 (cd06087)Ribosomal_S4e (pfam00900)→
					PRK04313 (PRK04313)KOW_RPS4 (cd06087)Ribosomal_S4e (pfam00900)→ RplE (COG0094)rpl5p (PRK04219)→
					RplE (COG0094)rpl5p (PRK04219)→ RpsN (COG0199)rps14P (PRK05766)→
					RpsN (COG0199)rps14P (PRK05766)→ rps8p (PRK04034)RpsH (COG0096)→
					rps8p (PRK04034)RpsH (COG0096)→ RplF (COG0097)rpl6p (PRK05518)→
					RplF (COG0097)rpl6p (PRK05518)→ PRK12766 (PRK12766)RecA (COG0468)Rpl32e (COG1717)→
					PRK12766 (PRK12766)RecA (COG0468)Rpl32e (COG1717)→ rpl19e (PRK08570)Ribosomal_L19e (pfam01280)→
					rpl19e (PRK08570)Ribosomal_L19e (pfam01280)→
env-222	SL_4KL_010_BRINE_10138442	+	547	646	**RNA**→ Methyltrn_RNA_3 (pfam02598)→
env-223	GSLNARP_GFPJP1N02IH3RV	+	123	222	**RNA**→ MTH1 (COG2106)→
env-224	GSLNARP_GFPJP1N02G85LW	-	148	49	**RNA**→
env-225	CEQN01112068.1	-	3026	2927	**RNA**→ Methyltrn_RNA_3 (pfam02598)→ RplC (COG0087)rpl3p (PRK04231)→
					RplD (COG0088)rpl4lp (PRK04042)→ RplW (COG0089)PRK14548 (PRK14548)→
					rpl2p (PRK09612)Ribosomal_L2 (pfam00181)Ribosomal_L2_C (pfam03947)→
env-226	CEUZ01492716.1	+	1180	1279	**RNA**→ MTH1 (COG2106)→
env-227	CEWR01279639.1	-	119	20	**RNA**→ Methyltrn_RNA_3 (pfam02598)→
env-228	APHM01021436.1	-	16571	16472	**RNA**→ Methyltrn_RNA_3 (pfam02598)→ RplC (COG0087)rpl3p (PRK04231)→
					RplD (COG0088)rpl4lp (PRK04042)→ RplW (COG0089)PRK14548 (PRK14548)→
					RplB (COG0090)rpl2p (PRK09612)→
env-229	LTJ07As871_J07ABscf098859	-	1081824	1081725	**RNA**→ Methyltrn_RNA_3 (pfam02598)→ RplC (COG0087)rpl3p (PRK04231)→
					RplD (COG0088)rpl4lp (PRK04042)→ RplW (COG0089)PRK14548 (PRK14548)→
					RplB (COG0090)rpl2p (PRK09612)→
env-230	JXWS01149363.1	-	957	858	**RNA**→ Methyltrn_RNA_3 (pfam02598)→ rpl3p (PRK04231)Ribosomal_L3 (pfam00297)→
env-231	3300000032_0007378	-	20967	20877	**RNA**→ RplC (COG0087)rpl3p (PRK04231)→ rpl4lp (PRK04042)Ribosomal_L4 (pfam00573)→
env-232	3300000568_10935138	+	219	307	**RNA**→ AF6_RA_repeat2 (cd01781)→
env-233	2232068851	-	371	281	**RNA**→ ←hypo

env-234	3300000558_10378615	+	2773	2863	**RNA**→ RplC (COG0087)rpl3p (PRK04231)→ RplD (COG0088)rpl4lp (PRK04042)→<br>RplW (COG0089)PRK14548 (PRK14548)→ rpl2p (PRK09612)Ribosomal_L2 (pfam00181)→
env-235	3300000568_10054768	+	16095	16185	**RNA**→ RplC (COG0087)rpl3p (PRK04231)→ RplD (COG0088)rpl4lp (PRK04042)→<br>RplW (COG0089)PRK14548 (PRK14548)→ RplB (COG0090)rpl2p (PRK09612)→<br>RpsS (COG0185)rps19p (PRK04038)→ rpl22p (PRK04223)RplV (COG0091)→<br>RpsC (COG0092)rps3p (PRK04191)→ PRK14549 (PRK14549)Ribosomal_L29 (pfam00831)→<br>PRK03879 (PRK03879)UPF0086 (pfam01868)→ RpsQ (COG0186)rps17p (PRK08572)→<br>rpl14p (PRK08571)Ribosomal_L14 (pfam00238)→ rpl24p (PRK01191)KOW_RPL26 (cd06089)→<br>PRK04313 (PRK04313)KOW_RPS4 (cd06087)Ribosomal_S4e (pfam00900)→ RplE (COG0094)rpl5p (PRK04219)→<br>RpsN (COG0199)rps14P (PRK05766)→ rps8p (PRK04034)RpsH (COG0096)→<br>RplF (COG0097)rpl6p (PRK05518)→ rpl32e (PRK08562)Ribosomal_L32e (pfam01655)→<br>rpl19e (PRK08570)Ribosomal_L19e (pfam01280)→ RplR (COG0256)rpl18p (PRK08569)→<br>RpsE (COG0098)rps5p (PRK04044)→
env-236	3300001580_10000682	+	16095	16185	**RNA**→ RplC (COG0087)rpl3p (PRK04231)→ RplD (COG0088)rpl4lp (PRK04042)→<br>RplW (COG0089)PRK14548 (PRK14548)→ RplB (COG0090)rpl2p (PRK09612)→<br>RpsS (COG0185)rps19p (PRK04038)→ rpl22p (PRK04223)RplV (COG0091)→<br>RpsC (COG0092)rps3p (PRK04191)→ PRK14549 (PRK14549)Ribosomal_L29 (pfam00831)→<br>PRK03879 (PRK03879)UPF0086 (pfam01868)→ RpsQ (COG0186)rps17p (PRK08572)→<br>rpl14p (PRK08571)Ribosomal_L14 (pfam00238)→ rpl24p (PRK01191)KOW_RPL26 (cd06089)→<br>PRK04313 (PRK04313)KOW_RPS4 (cd06087)Ribosomal_S4e (pfam00900)→ RplE (COG0094)rpl5p (PRK04219)→<br>RpsN (COG0199)rps14P (PRK05766)→ rps8p (PRK04034)RpsH (COG0096)→<br>RplF (COG0097)rpl6p (PRK05518)→ rpl32e (PRK08562)Ribosomal_L32e (pfam01655)→<br>rpl19e (PRK08570)Ribosomal_L19e (pfam01280)→ RplR (COG0256)rpl18p (PRK08569)→<br>RpsE (COG0098)rps5p (PRK04044)→
env-237	WSSedB2T_c028865	-	329	244	**RNA**→ ←hypo
env-238	JGI24020J35080_1001304	-	6969	6882	**RNA**→ PRK05883 (PRK05883)Methyltrn_RNA_3 (pfam02598)→ RplC (COG0087)rpl3p (PRK04231)→<br>RplD (COG0088)rpl4lp (PRK04042)→ RplW (COG0089)PRK14548 (PRK14548)→<br>RplB (COG0090)rpl2p (PRK09612)→ RpsS (COG0185)rps19p (PRK04038)→<br>rpl22p (PRK04223)RplV (COG0091)→ RpsC (COG0092)rps3p (PRK04191)→<br>PRK14549 (PRK14549)Ribosomal_L29 (pfam00831)→ POP4 (COG1588)PRK03879 (PRK03879)→<br>RpsQ (COG0186)rps17p (PRK08572)→ rpl14p (PRK08571)Ribosomal_L14 (pfam00238)→<br>rpl24p (PRK01191)KOW_RPL26 (cd06089)→<br>PRK04313 (PRK04313)S4 (cd00165)KOW_RPS4 (cd06087)Ribosomal_S4e (pfam00900)→
env-239	JGI24019J35510_1000070	-	95685	95598	**RNA**→ PRK05883 (PRK05883)Methyltrn_RNA_3 (pfam02598)→ RplC (COG0087)rpl3p (PRK04231)→<br>RplD (COG0088)rpl4lp (PRK04042)→ RplW (COG0089)PRK14548 (PRK14548)→<br>RplB (COG0090)rpl2p (PRK09612)→ RpsS (COG0185)rps19p (PRK04038)→<br>rpl22p (PRK04223)RplV (COG0091)→ RpsC (COG0092)rps3p (PRK04191)→<br>PRK14549 (PRK14549)Ribosomal_L29 (pfam00831)→ POP4 (COG1588)PRK03879 (PRK03879)→<br>RpsQ (COG0186)rps17p (PRK08572)→ rpl14p (PRK08571)Ribosomal_L14 (pfam00238)→<br>rpl24p (PRK01191)KOW_RPL26 (cd06089)→<br>PRK04313 (PRK04313)S4 (cd00165)KOW_RPS4 (cd06087)Ribosomal_S4e (pfam00900)→<br>RplE (COG0094)rpl5p (PRK04219)→ RpsN (COG0199)rps14P (PRK05766)→<br>rps8p (PRK04034)RpsH (COG0096)→ RplF (COG0097)rpl6p (PRK05518)→<br>Rpl32e (COG1717)rpl32e (PRK08562)HHH (pfam00633)HHH_5 (pfam14520)→<br>rpl19e (PRK08570)Ribosomal_L19e (pfam01280)→ RplR (COG0256)rpl18p (PRK08569)→<br>RpsE (COG0098)rps5p (PRK04044)→
env-240	PBDCA2_contig02310	+	406	502	**RNA**→ Methyltrn_RNA_3 (pfam02598)→
env-241	LGVC01039323.1	+	218	314	**RNA**→ lepA (TIGR01393)Methyltrn_RNA_3 (pfam02598)→

env-242	JGI24730J26740_1000024	-	31005	30928	**RNA**→ RplC (COG0087)rpl3p (PRK04231)→ RplD (COG0088)rpl4lp (PRK04042)→ RplW (COG0089)PRK14548 (PRK14548)→ RplB (COG0090)rpl2p (PRK09612)→ rps19p (PRK04038)Ribosomal_S19 (pfam00203)→ rpl22p (PRK04223)RplV (COG0091)→ RpsC (COG0092)rps3p (PRK04191)→ PRK14549 (PRK14549)Ribosomal_L29_HIP (cd00427)→ POP4 (COG1588)PRK03879 (PRK03879)→ RpsQ (COG0186)rps17p (PRK08572)→ rpl14p (PRK08571)Ribosomal_L14 (pfam00238)→ rpl24p (PRK01191)KOW_RPL26 (cd06089)→ PRK04313 (PRK04313)S4 (cd00165)KOW_RPS4 (cd06087)Ribosomal_S4e (pfam00900)→ rpl5p (PRK04219)Ribosomal_L5 (pfam00281)Ribosomal_L5_C (pfam00673)→ RpsN (COG0199)rps14P (PRK05766)→ rps8p (PRK04034)RpsH (COG0096)→ RplF (COG0097)rpl6p (PRK05518)→ rpl32e (PRK08562)Ribosomal_L32e (pfam01655)→ rpl19e (PRK08570)Ribosomal_L19e (pfam01280)→ RplR (COG0256)rpl18p (PRK08569)→ RpsE (COG0098)rps5p (PRK04044)→
env-243	JGI24729J20445_1009152	+	2242	2319	**RNA**→
env-244	3300001580_10004676	+	12012	12089	**RNA**→ RplC (COG0087)rpl3p (PRK04231)→
env-245	3300001592_10154490	-	853	774	**RNA**→ ←hypo
env-246	3300000032_0003629	-	1709	1630	**RNA**→ RplC (COG0087)rpl3p (PRK04231)→ RplD (COG0088)rpl4lp (PRK04042)→
env-247	3300000568_10010498	-	796	717	**RNA**→ hypo→ rpl3p (PRK04231)Ribosomal_L3 (pfam00297)→
env-248	3300000558_10003870	-	1201	1122	**RNA**→ rpl3p (PRK04231)Ribosomal_L3 (pfam00297)→ rpl3p (PRK04231)Ribosomal_L3 (pfam00297)→
env-249	3300001592_10087609	+	1266	1344	**RNA**→
env-250	3300001567_10002847	+	2463	2541	**RNA**→ RplC (COG0087)rpl3p (PRK04231)→ RplD (COG0088)rpl4lp (PRK04042)→ RplW (COG0089)PRK14548 (PRK14548)→ RplB (COG0090)rpl2p (PRK09612)→ RpsS (COG0185)rps19p (PRK04038)→ rpl22p (PRK04223)RplV (COG0091)→ RpsC (COG0092)rps3p (PRK04191)→ PRK14549 (PRK14549)Ribosomal_L29 (pfam00831)→ PRK03879 (PRK03879)UPF0086 (pfam01868)→ RpsQ (COG0186)rps17p (PRK08572)→ rpl14p (PRK08571)Ribosomal_L14 (pfam00238)→ rpl24p (PRK01191)KOW_RPL26 (cd06089)→ PRK04313 (PRK04313)S4 (cd00165)KOW_RPS4 (cd06087)Ribosomal_S4e (pfam00900)→ RplE (COG0094)rpl5p (PRK04219)→
env-251	SL_3KL_010_SED_10010201	-	3562	3491	**RNA**→ RplC (COG0087)rpl3p (PRK04231)→ RplD (COG0088)rpl4lp (PRK04042)→ RplW (COG0089)PRK14548 (PRK14548)→ RplB (COG0090)rpl2p (PRK09612)→ RpsS (COG0185)rps19p (PRK04038)→ hypo→
env-252	SL_3KL_010_SED_10013818	-	1092	1021	**RNA**→ rpl3p (PRK04231)Ribosomal_L3 (pfam00297)→
env-253	SL_3KL_010_SED_10042920	+	1511	1582	**RNA**→ RplC (COG0087)rpl3p (PRK04231)→ RplD (COG0088)rpl4lp (PRK04042)→
env-254	BDMC2_FXAG6RB01ERNKC	-	187	100	**RNA**→ hypo→
env-255	JGI24730J26740_1038966	-	597	511	**RNA**→ ←hypo
env-256	3300000568_10019878	+	535	613	**RNA**→ hypo→
env-257	3300001592_10003410	-	16086	16008	**RNA**→ RplC (COG0087)rpl3p (PRK04231)→ RplD (COG0088)rpl4lp (PRK04042)→ RplW (COG0089)PRK14548 (PRK14548)→ rpl2p (PRK09612)Ribosomal_L2_C (pfam03947)→ RpsS (COG0185)rps19p (PRK04038)→ rpl22p (PRK04223)RplV (COG0091)→ RpsC (COG0092)rps3p (PRK04191)→ PRK14549 (PRK14549)Ribosomal_L29 (pfam00831)→ PRK03879 (PRK03879)UPF0086 (pfam01868)→ RpsQ (COG0186)rps17p (PRK08572)→ rpl14p (PRK08571)Ribosomal_L14 (pfam00238)→ RplX (COG0198)rpl24p (PRK01191)→ PRK04313 (PRK04313)KOW_RPS4 (cd06087)Ribosomal_S4e (pfam00900)→ RplE (COG0094)rpl5p (PRK04219)→ RpsN (COG0199)rps14P (PRK05766)→ rps8p (PRK04034)RpsH (COG0096)→ RplF (COG0097)rpl6p (PRK05518)→ rpl32e (PRK08562)Ribosomal_L32e (pfam01655)→ rpl19e (PRK08570)Ribosomal_L19e (pfam01280)→ RplR (COG0256)rpl18p (PRK08569)→ RpsE (COG0098)rps5p (PRK04044)→

env-258	3300001567_10001101	+	23848	23926	**RNA**→ RplC (COG0087)rpl3p (PRK04231)→ RplD (COG0088)rpl4lp (PRK04042)→<br>RplW (COG0089)PRK14548 (PRK14548)→ rpl2p (PRK09612)Ribosomal_L2_C (pfam03947)→<br>RpsS (COG0185)rps19p (PRK04038)→ rpl22p (PRK04223)RplV (COG0091)→<br>RpsC (COG0092)rps3p (PRK04191)→ PRK14549 (PRK14549)Ribosomal_L29 (pfam00831)→<br>PRK03879 (PRK03879)UPF0086 (pfam01868)→ RpsQ (COG0186)rps17p (PRK08572)→<br>rpl14p (PRK08571)Ribosomal_L14 (pfam00238)→ RplX (COG0198)rpl24p (PRK01191)→<br>PRK04313 (PRK04313)KOW_RPS4 (cd06087)Ribosomal_S4e (pfam00900)→ RplE (COG0094)rpl5p (PRK04219)→<br>RpsN (COG0199)rps14P (PRK05766)→ rps8p (PRK04034)RpsH (COG0096)→<br>RplF (COG0097)rpl6p (PRK05518)→ rpl32e (PRK08562)Ribosomal_L32e (pfam01655)→<br>rpl19e (PRK08570)Ribosomal_L19e (pfam01280)→ RplR (COG0256)rpl18p (PRK08569)→<br>RpsE (COG0098)rps5p (PRK04044)→
env-259	3300000558_10195618	+	1540	1618	**RNA**→ hypo→
env-260	3300000032_0041450	-	12420	12342	**RNA**→ hypo→
env-261	JGI24713J26584_10001748	-	5737	5659	**RNA**→ RplC (COG0087)rpl3p (PRK04231)→ RplD (COG0088)rpl4lp (PRK04042)→<br>RplW (COG0089)PRK14548 (PRK14548)→ rpl2p (PRK09612)Ribosomal_L2_C (pfam03947)→<br>RpsS (COG0185)rps19p (PRK04038)→ rpl22p (PRK04223)RplV (COG0091)→<br>RpsC (COG0092)rps3p (PRK04191)→ PRK14549 (PRK14549)Ribosomal_L29 (pfam00831)→<br>PRK03879 (PRK03879)UPF0086 (pfam01868)→ RpsQ (COG0186)rps17p (PRK08572)→<br>rpl14p (PRK08571)Ribosomal_L14 (pfam00238)→
env-262	JGI24714J26587_10004747	+	2188	2266	**RNA**→ RplC (COG0087)rpl3p (PRK04231)→ RplD (COG0088)rpl4lp (PRK04042)→<br>RplW (COG0089)PRK14548 (PRK14548)→ rpl2p (PRK09612)Ribosomal_L2_C (pfam03947)→<br>RpsS (COG0185)rps19p (PRK04038)→ rpl22p (PRK04223)RplV (COG0091)→<br>RpsC (COG0092)rps3p (PRK04191)→ PRK14549 (PRK14549)Ribosomal_L29 (pfam00831)→<br>PRK03879 (PRK03879)UPF0086 (pfam01868)→ RpsQ (COG0186)rps17p (PRK08572)→<br>rpl14p (PRK08571)Ribosomal_L14 (pfam00238)→
env-263	JGI24504J29685_1004785	+	209	287	**RNA**→ RplC (COG0087)rpl3p (PRK04231)→ RplD (COG0088)rpl4lp (PRK04042)→<br>RplW (COG0089)PRK14548 (PRK14548)→ rpl2p (PRK09612)Ribosomal_L2_C (pfam03947)→<br>RpsS (COG0185)rps19p (PRK04038)→ rpl22p (PRK04223)RplV (COG0091)→<br>RpsC (COG0092)rps3p (PRK04191)→ PRK14549 (PRK14549)Ribosomal_L29 (pfam00831)→<br>PRK03879 (PRK03879)UPF0086 (pfam01868)→ RpsQ (COG0186)rps17p (PRK08572)→<br>rpl14p (PRK08571)Ribosomal_L14 (pfam00238)→ RplX (COG0198)rpl24p (PRK01191)→<br>PRK04313 (PRK04313)KOW_RPS4 (cd06087)Ribosomal_S4e (pfam00900)→<br>rpl5p (PRK04219)Ribosomal_L5 (pfam00281)→
env-264	JGI24505J29691_1002356	+	209	287	**RNA**→ RplC (COG0087)rpl3p (PRK04231)→ RplD (COG0088)rpl4lp (PRK04042)→<br>RplW (COG0089)PRK14548 (PRK14548)→ rpl2p (PRK09612)Ribosomal_L2_C (pfam03947)→<br>RpsS (COG0185)rps19p (PRK04038)→ rpl22p (PRK04223)RplV (COG0091)→<br>RpsC (COG0092)rps3p (PRK04191)→ PRK14549 (PRK14549)Ribosomal_L29 (pfam00831)→<br>PRK03879 (PRK03879)UPF0086 (pfam01868)→ RpsQ (COG0186)rps17p (PRK08572)→<br>rpl14p (PRK08571)Ribosomal_L14 (pfam00238)→ RplX (COG0198)rpl24p (PRK01191)→<br>PRK04313 (PRK04313)KOW_RPS4 (cd06087)Ribosomal_S4e (pfam00900)→ RplE (COG0094)rpl5p (PRK04219)→<br>RpsN (COG0199)rps14P (PRK05766)→ rps8p (PRK04034)RpsH (COG0096)→<br>RplF (COG0097)rpl6p (PRK05518)→ rpl32e (PRK08562)Ribosomal_L32e (pfam01655)→<br>rpl19e (PRK08570)Ribosomal_L19e (pfam01280)→ RplR (COG0256)rpl18p (PRK08569)→<br>RpsE (COG0098)rps5p (PRK04044)→ Ribosomal_L7_archeal_euk (cd01657)rpl30p (PRK06049)→<br>rpl15p (PRK06419)Ribosomal_L27A (pfam00828)→
env-265	3300000507_100004	-	18302	18224	**RNA**→ hypo→ RplC (COG0087)rpl3p (PRK04231)→ RplD (COG0088)rpl4lp (PRK04042)→<br>RplW (COG0089)PRK14548 (PRK14548)→ RplB (COG0090)rpl2p (PRK09612)→<br>RpsS (COG0185)rps19p (PRK04038)→ rpl22p (PRK04223)RplV (COG0091)→<br>RpsC (COG0092)rps3p (PRK04191)→ PRK14549 (PRK14549)Ribosomal_L29 (pfam00831)→<br>PRK03879 (PRK03879)UPF0086 (pfam01868)→ RpsQ (COG0186)rps17p (PRK08572)→<br>rpl14p (PRK08571)Ribosomal_L14 (pfam00238)→ RplX (COG0198)rpl24p (PRK01191)→<br>PRK04313 (PRK04313)KOW_RPS4 (cd06087)Ribosomal_S4e (pfam00900)→ RplE (COG0094)rpl5p (PRK04219)→<br>RpsN (COG0199)rps14P (PRK05766)→ rps8p (PRK04034)RpsH (COG0096)→<br>RplF (COG0097)rpl6p (PRK05518)→ rpl32e (PRK08562)Ribosomal_L32e (pfam01655)→<br>rpl19e (PRK08570)Ribosomal_L19e (pfam01280)→ RplR (COG0256)rpl18p (PRK08569)→<br>RpsE (COG0098)rps5p (PRK04044)→

env-266	3300000558_10023046	-	1376	1297	**RNA**→ RplC (COG0087)rpl3p (PRK04231)→
env-267	3300001592_10042930	-	999	920	**RNA**→ ←hypo
env-268	3300000568_10983650	+	160	239	**RNA**→ hypo→
env-269	3300000568_10052832	+	512	591	**RNA**→ hypo→
env-270	3300000032_0016008	+	347	426	**RNA**→ hypo→
env-271	JGI24711J26586_10020110	-	584	506	**RNA**→ rpl3p (PRK04231)Ribosomal_L3 (pfam00297)→
env-272	JGI24708J26588_10012893	+	2107	2185	**RNA**→ RplC (COG0087)rpl3p (PRK04231)→ RplD (COG0088)rpl4lp (PRK04042)→ RplW (COG0089)PRK14548 (PRK14548)→
env-273	JGI24710J26742_10008143	+	5280	5358	**RNA**→ RplC (COG0087)rpl3p (PRK04231)→ RplD (COG0088)rpl4lp (PRK04042)→ RplW (COG0089)PRK14548 (PRK14548)→
env-274	JGI24707J26582_10001803	+	2105	2183	**RNA**→ RplC (COG0087)rpl3p (PRK04231)→ RplD (COG0088)rpl4lp (PRK04042)→ RplW (COG0089)PRK14548 (PRK14548)→ RplB (COG0090)rpl2p (PRK09612)→
env-275	JGI24712J26585_10006974	-	5713	5635	**RNA**→ RplC (COG0087)rpl3p (PRK04231)→ RplD (COG0088)rpl4lp (PRK04042)→ RplW (COG0089)PRK14548 (PRK14548)→ RplB (COG0090)rpl2p (PRK09612)→ RpsS (COG0185)rps19p (PRK04038)→ rpl22p (PRK04223)RplV (COG0091)→ RpsC (COG0092)rps3p (PRK04191)→ PRK14549 (PRK14549)Ribosomal_L29 (pfam00831)→ PRK03879 (PRK03879)UPF0086 (pfam01868)→ RpsQ (COG0186)rps17p (PRK08572)→ rpl14p (PRK08571)Ribosomal_L14 (pfam00238)→
env-276	JGI24709J26583_10619039	+	43	121	**RNA**→
env-277	JGI24499J29688_1000702	+	823	901	**RNA**→ RplC (COG0087)rpl3p (PRK04231)→ RplD (COG0088)rpl4lp (PRK04042)→ RplW (COG0089)PRK14548 (PRK14548)→ RplB (COG0090)rpl2p (PRK09612)→ RpsS (COG0185)rps19p (PRK04038)→ rpl22p (PRK04223)RplV (COG0091)→ RpsC (COG0092)rps3p (PRK04191)→ PRK14549 (PRK14549)Ribosomal_L29 (pfam00831)→ PRK03879 (PRK03879)UPF0086 (pfam01868)→ RpsQ (COG0186)rps17p (PRK08572)→ rpl14p (PRK08571)Ribosomal_L14 (pfam00238)→ rpl24p (PRK01191)KOW_RPL26 (cd06089)→ PRK04313 (PRK04313)KOW_RPS4 (cd06087)Ribosomal_S4e (pfam00900)→ RplE (COG0094)rpl5p (PRK04219)→ RpsN (COG0199)rps14P (PRK05766)→ rps8p (PRK04034)RpsH (COG0096)→ RplF (COG0097)rpl6p (PRK05518)→ rpl32e (PRK08562)Ribosomal_L32e (pfam01655)→ rpl19e (PRK08570)Ribosomal_L19e (pfam01280)→ RplR (COG0256)rpl18p (PRK08569)→ RpsE (COG0098)rps5p (PRK04044)→ Ribosomal_L7_archeal_euk (cd01657)rpl30p (PRK06049)→ rpl15p (PRK06419)Ribosomal_L27A (pfam00828)→ SecY (COG0201)PRK08568 (PRK08568)→
env-278	JGI24707J26582_10000621	+	14235	14314	**RNA**→ ←hypo
env-279	JGI24708J26588_10001024	+	13186	13265	**RNA**→ ←hypo
env-280	JGI24713J26584_10000622	+	13651	13730	**RNA**→ ←hypo
env-281	JGI24714J26587_10000570	+	13186	13265	**RNA**→ ←hypo
env-282	JGI24712J26585_10002902	+	13100	13179	**RNA**→ ←hypo
env-283	JGI24711J26586_10000985	-	9166	9087	**RNA**→ RplC (COG0087)rpl3p (PRK04231)→ RplD (COG0088)rpl4lp (PRK04042)→ RplW (COG0089)PRK14548 (PRK14548)→ RplB (COG0090)rpl2p (PRK09612)→ RpsS (COG0185)rps19p (PRK04038)→ rpl22p (PRK04223)RplV (COG0091)→ RpsC (COG0092)rps3p (PRK04191)→ PRK14549 (PRK14549)Ribosomal_L29 (pfam00831)→ PRK03879 (PRK03879)UPF0086 (pfam01868)→ RpsQ (COG0186)rps17p (PRK08572)→ rpl14p (PRK08571)Ribosomal_L14 (pfam00238)→ RplX (COG0198)rpl24p (PRK01191)→ PRK04313 (PRK04313)KOW_RPS4 (cd06087)Ribosomal_S4e (pfam00900)→ RplE (COG0094)rpl5p (PRK04219)→ RpsN (COG0199)rps14P (PRK05766)→ rps8p (PRK04034)RpsH (COG0096)→ RplF (COG0097)rpl6p (PRK05518)→ rpl32e (PRK08562)Ribosomal_L32e (pfam01655)→ rpl19e (PRK08570)Ribosomal_L19e (pfam01280)→

env-284	JGI24709J26583_10001164	-	9166	9087	**RNA**→ RplC (COG0087)rpl3p (PRK04231)→ RplD (COG0088)rpl4lp (PRK04042)→<br>RplW (COG0089)PRK14548 (PRK14548)→ RplB (COG0090)rpl2p (PRK09612)→<br>RpsS (COG0185)rps19p (PRK04038)→ rpl22p (PRK04223)RplV (COG0091)→<br>RpsC (COG0092)rps3p (PRK04191)→ PRK14549 (PRK14549)Ribosomal_L29 (pfam00831)→<br>PRK03879 (PRK03879)UPF0086 (pfam01868)→ RpsQ (COG0186)rps17p (PRK08572)→<br>rpl14p (PRK08571)Ribosomal_L14 (pfam00238)→ RplX (COG0198)rpl24p (PRK01191)→<br>PRK04313 (PRK04313)KOW_RPS4 (cd06087)Ribosomal_S4e (pfam00900)→ RplE (COG0094)rpl5p (PRK04219)→<br>RpsN (COG0199)rps14P (PRK05766)→ rps8p (PRK04034)RpsH (COG0096)→<br>RplF (COG0097)rpl6p (PRK05518)→ rpl32e (PRK08562)Ribosomal_L32e (pfam01655)→<br>rpl19e (PRK08570)Ribosomal_L19e (pfam01280)→
env-285	JGI24710J26742_10000974	+	13183	13262	**RNA**→ ←hypo
env-286	JGI24505J29691_1015564	+	211	290	**RNA**→ ←hypo
env-287	JGI24500J29687_10001710	+	1224	1303	**RNA**→ RplC (COG0087)rpl3p (PRK04231)→ RplD (COG0088)rpl4lp (PRK04042)→<br>RplW (COG0089)PRK14548 (PRK14548)→ RplB (COG0090)rpl2p (PRK09612)→<br>RpsS (COG0185)rps19p (PRK04038)→ rpl22p (PRK04223)RplV (COG0091)→<br>RpsC (COG0092)rps3p (PRK04191)→ PRK14549 (PRK14549)Ribosomal_L29 (pfam00831)→<br>PRK03879 (PRK03879)UPF0086 (pfam01868)→ RpsQ (COG0186)rps17p (PRK08572)→<br>rpl14p (PRK08571)Ribosomal_L14 (pfam00238)→ RplX (COG0198)rpl24p (PRK01191)→<br>PRK04313 (PRK04313)KOW_RPS4 (cd06087)Ribosomal_S4e (pfam00900)→ RplE (COG0094)rpl5p (PRK04219)→<br>RpsN (COG0199)rps14P (PRK05766)→ rps8p (PRK04034)RpsH (COG0096)→<br>RplF (COG0097)rpl6p (PRK05518)→ rpl32e (PRK08562)Ribosomal_L32e (pfam01655)→<br>rpl19e (PRK08570)Ribosomal_L19e (pfam01280)→ RplR (COG0256)rpl18p (PRK08569)→<br>RpsE (COG0098)rps5p (PRK04044)→ Ribosomal_L7_archeal_euk (cd01657)rpl30p (PRK06049)→<br>rpl15p (PRK06419)Ribosomal_L27A (pfam00828)→ SecY (COG0201)PRK08568 (PRK08568)→
env-288	JGI24502J29692_10012380	+	876	955	**RNA**→ RplC (COG0087)rpl3p (PRK04231)→ RplD (COG0088)rpl4lp (PRK04042)→<br>RplW (COG0089)PRK14548 (PRK14548)→ RplB (COG0090)rpl2p (PRK09612)→<br>RpsS (COG0185)rps19p (PRK04038)→ rpl22p (PRK04223)RplV (COG0091)→<br>RpsC (COG0092)rps3p (PRK04191)→ PRK14549 (PRK14549)Ribosomal_L29 (pfam00831)→<br>PRK03879 (PRK03879)UPF0086 (pfam01868)→ RpsQ (COG0186)rps17p (PRK08572)→<br>rpl14p (PRK08571)Ribosomal_L14 (pfam00238)→ RplX (COG0198)rpl24p (PRK01191)→<br>PRK04313 (PRK04313)KOW_RPS4 (cd06087)Ribosomal_S4e (pfam00900)→ RplE (COG0094)rpl5p (PRK04219)→<br>RpsN (COG0199)rps14P (PRK05766)→ rps8p (PRK04034)RpsH (COG0096)→<br>RplF (COG0097)rpl6p (PRK05518)→ rpl32e (PRK08562)Ribosomal_L32e (pfam01655)→<br>rpl19e (PRK08570)Ribosomal_L19e (pfam01280)→ RplR (COG0256)rpl18p (PRK08569)→<br>RpsE (COG0098)rps5p (PRK04044)→ Ribosomal_L7_archeal_euk (cd01657)rpl30p (PRK06049)→<br>rpl15p (PRK06419)Ribosomal_L27A (pfam00828)→ SecY (COG0201)PRK08568 (PRK08568)→
env-289	JGI24501J29690_1003097	+	211	290	**RNA**→ RplC (COG0087)rpl3p (PRK04231)→ RplD (COG0088)rpl4lp (PRK04042)→<br>RplW (COG0089)PRK14548 (PRK14548)→ RplB (COG0090)rpl2p (PRK09612)→<br>RpsS (COG0185)rps19p (PRK04038)→ rpl22p (PRK04223)RplV (COG0091)→<br>RpsC (COG0092)rps3p (PRK04191)→ PRK14549 (PRK14549)Ribosomal_L29 (pfam00831)→<br>PRK03879 (PRK03879)UPF0086 (pfam01868)→ RpsQ (COG0186)rps17p (PRK08572)→<br>rpl14p (PRK08571)Ribosomal_L14 (pfam00238)→ RplX (COG0198)rpl24p (PRK01191)→<br>PRK04313 (PRK04313)KOW_RPS4 (cd06087)Ribosomal_S4e (pfam00900)→ RplE (COG0094)rpl5p (PRK04219)→<br>RpsN (COG0199)rps14P (PRK05766)→ rps8p (PRK04034)RpsH (COG0096)→<br>RplF (COG0097)rpl6p (PRK05518)→ rpl32e (PRK08562)Ribosomal_L32e (pfam01655)→<br>rpl19e (PRK08570)Ribosomal_L19e (pfam01280)→ RplR (COG0256)rpl18p (PRK08569)→<br>RpsE (COG0098)rps5p (PRK04044)→ Ribosomal_L7_archeal_euk (cd01657)rpl30p (PRK06049)→<br>rpl15p (PRK06419)Ribosomal_L27A (pfam00828)→ SecY (COG0201)PRK08568 (PRK08568)→

env-290	JGI24503J29689_10009320	+	876	955	**RNA**→ RplC (COG0087)rpl3p (PRK04231)→ RplD (COG0088)rpl4lp (PRK04042)→<br>RplW (COG0089)PRK14548 (PRK14548)→ RplB (COG0090)rpl2p (PRK09612)→<br>RpsS (COG0185)rps19p (PRK04038)→ rpl22p (PRK04223)RplV (COG0091)→<br>RpsC (COG0092)rps3p (PRK04191)→ PRK14549 (PRK14549)Ribosomal_L29 (pfam00831)→<br>PRK03879 (PRK03879)UPF0086 (pfam01868)→ RpsQ (COG0186)rps17p (PRK08572)→<br>rpl14p (PRK08571)Ribosomal_L14 (pfam00238)→ RplX (COG0198)rpl24p (PRK01191)→<br>PRK04313 (PRK04313)KOW_RPS4 (cd06087)Ribosomal_S4e (pfam00900)→ RplE (COG0094)rpl5p (PRK04219)→<br>RpsN (COG0199)rps14P (PRK05766)→ rps8p (PRK04034)RpsH (COG0096)→<br>RplF (COG0097)rpl6p (PRK05518)→ rpl32e (PRK08562)Ribosomal_L32e (pfam01655)→<br>rpl19e (PRK08570)Ribosomal_L19e (pfam01280)→ RplR (COG0256)rpl18p (PRK08569)→<br>RpsE (COG0098)rps5p (PRK04044)→ Ribosomal_L7_archeal_euk (cd01657)rpl30p (PRK06049)→<br>rpl15p (PRK06419)Ribosomal_L27A (pfam00828)→ SecY (COG0201)PRK08568 (PRK08568)→
env-291	JGI24499J29688_1000521	+	876	955	**RNA**→ RplC (COG0087)rpl3p (PRK04231)→ RplD (COG0088)rpl4lp (PRK04042)→<br>RplW (COG0089)PRK14548 (PRK14548)→ RplB (COG0090)rpl2p (PRK09612)→<br>RpsS (COG0185)rps19p (PRK04038)→ rpl22p (PRK04223)RplV (COG0091)→<br>RpsC (COG0092)rps3p (PRK04191)→ PRK14549 (PRK14549)Ribosomal_L29 (pfam00831)→<br>PRK03879 (PRK03879)UPF0086 (pfam01868)→ RpsQ (COG0186)rps17p (PRK08572)→<br>rpl14p (PRK08571)Ribosomal_L14 (pfam00238)→ RplX (COG0198)rpl24p (PRK01191)→<br>PRK04313 (PRK04313)KOW_RPS4 (cd06087)Ribosomal_S4e (pfam00900)→ RplE (COG0094)rpl5p (PRK04219)→<br>RpsN (COG0199)rps14P (PRK05766)→ rps8p (PRK04034)RpsH (COG0096)→<br>RplF (COG0097)rpl6p (PRK05518)→ rpl32e (PRK08562)Ribosomal_L32e (pfam01655)→<br>rpl19e (PRK08570)Ribosomal_L19e (pfam01280)→ RplR (COG0256)rpl18p (PRK08569)→<br>RpsE (COG0098)rps5p (PRK04044)→ Ribosomal_L7_archeal_euk (cd01657)rpl30p (PRK06049)→<br>rpl15p (PRK06419)Ribosomal_L27A (pfam00828)→ SecY (COG0201)PRK08568 (PRK08568)→
env-292	JGI24712J26585_10029187	+	2386	2464	**RNA**→ hypo→
env-293	JGI24711J26586_10244524	-	365	287	**RNA**→ ←hypo
env-294	JGI24710J26742_10309618	-	365	287	**RNA**→ ←hypo
env-295	JGI24500J29687_10002376	+	168	246	**RNA**→ ←hypo
env-296	JGI24503J29689_10006492	+	104	182	**RNA**→ rpl3p (PRK04231)Ribosomal_L3 (pfam00297)→
Mbo-1-1	NC_018227.2	+	1712758	1712836	**RNA**→ RplC (COG0087)rpl3p (PRK04231)→ RplD (COG0088)rpl4lp (PRK04042)→<br>RplW (COG0089)PRK14548 (PRK14548)→ RplB (COG0090)rpl2p (PRK09612)→<br>RpsS (COG0185)rps19p (PRK04038)→ rpl22p (PRK04223)RplV (COG0091)→<br>RpsC (COG0092)rps3p (PRK04191)→ PRK14549 (PRK14549)Ribosomal_L29 (pfam00831)→<br>PRK03879 (PRK03879)UPF0086 (pfam01868)→ RpsQ (COG0186)rps17p (PRK08572)→<br>rpl14p (PRK08571)Ribosomal_L14 (pfam00238)→ RplX (COG0198)rpl24p (PRK01191)→<br>PRK04313 (PRK04313)KOW_RPS4 (cd06087)Ribosomal_S4e (pfam00900)→ RplE (COG0094)rpl5p (PRK04219)→<br>RpsN (COG0199)rps14P (PRK05766)→ rps8p (PRK04034)RpsH (COG0096)→<br>RplF (COG0097)rpl6p (PRK05518)→ rpl32e (PRK08562)Ribosomal_L32e (pfam01655)→<br>rpl19e (PRK08570)Ribosomal_L19e (pfam01280)→ RplR (COG0256)rpl18p (PRK08569)→<br>RpsE (COG0098)rps5p (PRK04044)→
env-297	JGI24501J29690_1006097	+	749	827	**RNA**→ rpl3p (PRK04231)Ribosomal_L3 (pfam00297)→
env-298	JGI24707J26582_10054179	+	722	800	**RNA**→ rpl3p (PRK04231)Ribosomal_L3 (pfam00297)→
env-299	JGI24708J26588_10030031	+	1058	1136	**RNA**→ ←hypo
env-300	JGI24709J26583_10416662	-	277	199	**RNA**→ hypo→
env-301	JGI24502J29692_10003709	+	657	735	**RNA**→ rpl3p (PRK04231)Ribosomal_L3 (pfam00297)→
env-302	JGI24503J29689_10003031	+	41	119	**RNA**→ ←hypo
env-303	JGI24710J26742_10068176	+	372	450	**RNA**→ rpl3p (PRK04231)Ribosomal_L3 (pfam00297)→
env-304	JGI24709J26583_10014213	+	553	631	**RNA**→ hypo→ RplC (COG0087)rpl3p (PRK04231)→ RplD (COG0088)rpl4lp (PRK04042)→<br>RplW (COG0089)PRK14548 (PRK14548)→
env-305	JGI24502J29692_10002344	+	84	165	**RNA**→ RplC (COG0087)rpl3p (PRK04231)→ RplD (COG0088)rpl4lp (PRK04042)→
env-306	JGI24712J26585_10639760	+	51	132	**RNA**→ ←hypo
env-307	JGI24711J26586_10552659	+	51	132	**RNA**→ ←hypo
env-308	JGI24709J26583_10614246	+	51	132	**RNA**→ ←hypo
env-309	JGI24710J26742_10664497	+	51	132	**RNA**→ ←hypo

Mma-1-1	NC_009051.1	+	549081	549159	**RNA**→ RplC (COG0087)rpl3p (PRK04231)→ RplD (COG0088)rpl4lp (PRK04042)→ RplW (COG0089)PRK14548 (PRK14548)→ RplB (COG0090)rpl2p (PRK09612)→ RpsS (COG0185)rps19p (PRK04038)→ rpl22p (PRK04223)RplV (COG0091)→ RpsC (COG0092)rps3p (PRK04191)→ PRK14549 (PRK14549)Ribosomal_L29 (pfam00831)→ PRK03879 (PRK03879)UPF0086 (pfam01868)→ RpsQ (COG0186)rps17p (PRK08572)→ rpl14p (PRK08571)Ribosomal_L14 (pfam00238)→ RplX (COG0198)rpl24p (PRK01191)→ PRK04313 (PRK04313)KOW_RPS4 (cd06087)Ribosomal_S4e (pfam00900)→ RplE (COG0094)rpl5p (PRK04219)→ RpsN (COG0199)rps14P (PRK05766)→ rps8p (PRK04034)RpsH (COG0096)→ RplF (COG0097)rpl6p (PRK05518)→ rpl32e (PRK08562)Ribosomal_L32_L32e (cd00513)→ rpl19e (PRK08570)Ribosomal_L19e (pfam01280)→ RplR (COG0256)rpl18p (PRK08569)→ RpsE (COG0098)rps5p (PRK04044)→
Mse-1-1	NZ_JXOJ01000001.1	+	373962	374040	**RNA**→ RplC (COG0087)rpl3p (PRK04231)→ RplD (COG0088)rpl4lp (PRK04042)→ RplW (COG0089)PRK14548 (PRK14548)→ rpl2p (PRK09612)Ribosomal_L2_C (pfam03947)→ RpsS (COG0185)rps19p (PRK04038)→ rpl22p (PRK04223)RplV (COG0091)→ RpsC (COG0092)rps3p (PRK04191)→ PRK14549 (PRK14549)Ribosomal_L29 (pfam00831)→ PRK03879 (PRK03879)UPF0086 (pfam01868)→ RpsQ (COG0186)rps17p (PRK08572)→ rpl14p (PRK08571)Ribosomal_L14 (pfam00238)→ RplX (COG0198)rpl24p (PRK01191)→ PRK04313 (PRK04313)KOW_RPS4 (cd06087)Ribosomal_S4e (pfam00900)→ RplE (COG0094)rpl5p (PRK04219)→ RpsN (COG0199)rps14P (PRK05766)→ rps8p (PRK04034)RpsH (COG0096)→ RplF (COG0097)rpl6p (PRK05518)→ rpl32e (PRK08562)Ribosomal_L32e (pfam01655)→ rpl19e (PRK08570)Ribosomal_L19e (pfam01280)→ RplR (COG0256)rpl18p (PRK08569)→ RpsE (COG0098)rps5p (PRK04044)→
Msp-1-1	NZ_JMIO01000016.1	+	890	968	**RNA**→ RplC (COG0087)rpl3p (PRK04231)→ RplD (COG0088)rpl4lp (PRK04042)→ RplW (COG0089)PRK14548 (PRK14548)→ RplB (COG0090)rpl2p (PRK09612)→ RpsS (COG0185)rps19p (PRK04038)→ rpl22p (PRK04223)RplV (COG0091)→ RpsC (COG0092)rps3p (PRK04191)→ PRK14549 (PRK14549)Ribosomal_L29 (pfam00831)→ PRK03879 (PRK03879)UPF0086 (pfam01868)→ RpsQ (COG0186)rps17p (PRK08572)→ rpl14p (PRK08571)Ribosomal_L14 (pfam00238)→ RplX (COG0198)rpl24p (PRK01191)→ PRK04313 (PRK04313)S4 (cd00165)KOW_RPS4 (cd06087)Ribosomal_S4e (pfam00900)→ RplE (COG0094)rpl5p (PRK04219)→ RpsN (COG0199)rps14P (PRK05766)→ rps8p (PRK04034)RpsH (COG0096)→ RplF (COG0097)rpl6p (PRK05518)→ rpl32e (PRK08562)Ribosomal_L32e (pfam01655)→ rpl19e (PRK08570)Ribosomal_L19e (pfam01280)→ RplR (COG0256)rpl18p (PRK08569)→ RpsE (COG0098)rps5p (PRK04044)→ Ribosomal_L7_archeal_euk (cd01657)rpl30p (PRK06049)→ rpl15p (PRK06419)Ribosomal_L27A (pfam00828)→ SecY (COG0201)PRK08568 (PRK08568)→ hypo→
env-310	JGI24712J26585_10057261	-	366	297	**RNA**→ ←hypo
env-311	JGI24710J26742_10004413	-	10050	9981	**RNA**→ RplC (COG0087)rpl3p (PRK04231)→ RplD (COG0088)rpl4lp (PRK04042)→ RplW (COG0089)PRK14548 (PRK14548)→ RplB (COG0090)rpl2p (PRK09612)→ RpsS (COG0185)rps19p (PRK04038)→ rpl22p (PRK04223)RplV (COG0091)→ RpsC (COG0092)rps3p (PRK04191)→ PRK14549 (PRK14549)Ribosomal_L29 (pfam00831)→ PRK03879 (PRK03879)UPF0086 (pfam01868)→ RpsQ (COG0186)rps17p (PRK08572)→ rpl14p (PRK08571)Ribosomal_L14 (pfam00238)→ rpl24p (PRK01191)KOW_RPL26 (cd06089)→ PRK04313 (PRK04313)KOW_RPS4 (cd06087)Ribosomal_S4e (pfam00900)→ RplE (COG0094)rpl5p (PRK04219)→ RpsN (COG0199)rps14P (PRK05766)→ rps8p (PRK04034)RpsH (COG0096)→ RplF (COG0097)rpl6p (PRK05518)→ rpl32e (PRK08562)Ribosomal_L32e (pfam01655)→ rpl19e (PRK08570)Ribosomal_L19e (pfam01280)→ RplR (COG0256)rpl18p (PRK08569)→
env-312	JGI24500J29687_10026414	+	210	279	**RNA**→ RplC (COG0087)rpl3p (PRK04231)→ RplD (COG0088)rpl4lp (PRK04042)→ RplW (COG0089)PRK14548 (PRK14548)→ RplB (COG0090)rpl2p (PRK09612)→ RpsS (COG0185)rps19p (PRK04038)→ rpl22p (PRK04223)RplV (COG0091)→ RpsC (COG0092)rps3p (PRK04191)→ PRK14549 (PRK14549)Ribosomal_L29 (pfam00831)→ PRK03879 (PRK03879)UPF0086 (pfam01868)→ RpsQ (COG0186)rps17p (PRK08572)→ rpl14p (PRK08571)Ribosomal_L14 (pfam00238)→ rpl24p (PRK01191)KOW_RPL26 (cd06089)→ PRK04313 (PRK04313)KOW_RPS4 (cd06087)Ribosomal_S4e (pfam00900)→ RplE (COG0094)rpl5p (PRK04219)→ RpsN (COG0199)rps14P (PRK05766)→
env-313	JGI24501J29690_1062315	+	210	279	**RNA**→ ←hypo

env-314	JGI24499J29688_1102173	+	190	259	**RNA**→ ←hypo
env-315	JGI24709J26583_10005356	-	7831	7762	**RNA**→ RplC (COG0087)rpl3p (PRK04231)→ RplD (COG0088)rpl4lp (PRK04042)→ RplW (COG0089)PRK14548 (PRK14548)→ RplB (COG0090)rpl2p (PRK09612)→ RpsS (COG0185)rps19p (PRK04038)→ rpl22p (PRK04223)RplV (COG0091)→ RpsC (COG0092)rps3p (PRK04191)→ PRK14549 (PRK14549)Ribosomal_L29 (pfam00831)→ PRK03879 (PRK03879)UPF0086 (pfam01868)→ RpsQ (COG0186)rps17p (PRK08572)→ rpl14p (PRK08571)Ribosomal_L14 (pfam00238)→ rpl24p (PRK01191)KOW_RPL26 (cd06089)→ PRK04313 (PRK04313)KOW_RPS4 (cd06087)Ribosomal_S4e (pfam00900)→ RplE (COG0094)rpl5p (PRK04219)→ RpsN (COG0199)rps14P (PRK05766)→ rps8p (PRK04034)Ribosomal_S8 (pfam00410)→
env-316	JGI24711J26586_10160586	+	77	146	**RNA**→ ←hypo
env-317	JGI24500J29687_10008301	+	42	123	**RNA**→ RplC (COG0087)rpl3p (PRK04231)→ RplD (COG0088)rpl4lp (PRK04042)→ RplW (COG0089)PRK14548 (PRK14548)→ RplB (COG0090)rpl2p (PRK09612)→ RpsS (COG0185)rps19p (PRK04038)→ rpl22p (PRK04223)RplV (COG0091)→ RpsC (COG0092)rps3p (PRK04191)→ PRK14549 (PRK14549)Ribosomal_L29 (pfam00831)→ PRK03879 (PRK03879)UPF0086 (pfam01868)→ RpsQ (COG0186)rps17p (PRK08572)→ rpl14p (PRK08571)Ribosomal_L14 (pfam00238)→ rpl24p (PRK01191)KOW_RPL26 (cd06089)→ PRK04313 (PRK04313)KOW_RPS4 (cd06087)Ribosomal_S4e (pfam00900)→ RplE (COG0094)rpl5p (PRK04219)→ RpsN (COG0199)rps14P (PRK05766)→ rps8p (PRK04034)RpsH (COG0096)→ RplF (COG0097)rpl6p (PRK05518)→ rpl32e (PRK08562)Ribosomal_L32e (pfam01655)→ rpl19e (PRK08570)Ribosomal_L19e (pfam01280)→ RplR (COG0256)rpl18p (PRK08569)→
env-318	JGI24502J29692_10009873	+	777	846	**RNA**→ rpl3p (PRK04231)Ribosomal_L3 (pfam00297)→
env-319	JGI24503J29689_10009607	+	112	181	**RNA**→ rpl3p (PRK04231)Ribosomal_L3 (pfam00297)→
env-320	JGI24712J26585_10076884	-	494	425	**RNA**→ rpl3p (PRK04231)Ribosomal_L3 (pfam00297)→
env-321	JGI24500J29687_10085663	+	72	141	**RNA**→ rpl3p (PRK04231)Ribosomal_L3 (pfam00297)→
env-322	JGI24711J26586_10010708	-	494	425	**RNA**→ rpl3p (PRK04231)Ribosomal_L3 (pfam00297)→
Mpa-1-1	NC_011832.1	+	459047	459117	**RNA**→ RplC (COG0087)rpl3p (PRK04231)→ RplD (COG0088)rpl4lp (PRK04042)→ RplW (COG0089)PRK14548 (PRK14548)→ rpl2p (PRK09612)Ribosomal_L2_C (pfam03947)→ RpsS (COG0185)rps19p (PRK04038)→ rpl22p (PRK04223)RplV (COG0091)→ RpsC (COG0092)rps3p (PRK04191)→ PRK14549 (PRK14549)Ribosomal_L29 (pfam00831)→ PRK03879 (PRK03879)UPF0086 (pfam01868)→ RpsQ (COG0186)rps17p (PRK08572)→ rpl14p (PRK08571)Ribosomal_L14 (pfam00238)→ rpl24p (PRK01191)KOW_RPL26 (cd06089)→ PRK04313 (PRK04313)S4 (cd00165)KOW_RPS4 (cd06087)Ribosomal_S4e (pfam00900)→ RplE (COG0094)rpl5p (PRK04219)→ RpsN (COG0199)rps14P (PRK05766)→ rps8p (PRK04034)RpsH (COG0096)→ RplF (COG0097)rpl6p (PRK05518)→ rpl32e (PRK08562)Ribosomal_L32e (pfam01655)→ rpl19e (PRK08570)Ribosomal_L19e (pfam01280)→ RplR (COG0256)rpl18p (PRK08569)→ RpsE (COG0098)rps5p (PRK04044)→
env-323	DHTCA2_GJDNVXK01BZYAY	-	259	189	**RNA**→ hypo→
env-324	3300001580_10004417	+	2215	2285	**RNA**→ hypo→ RplC (COG0087)rpl3p (PRK04231)→ RplD (COG0088)rpl4lp (PRK04042)→ RplW (COG0089)PRK14548 (PRK14548)→ RplB (COG0090)rpl2p (PRK09612)→ rps19p (PRK04038)Ribosomal_S19 (pfam00203)→ rpl22p (PRK04223)RplV (COG0091)→ RpsC (COG0092)rps3p (PRK04191)→ PRK14549 (PRK14549)Ribosomal_L29 (pfam00831)→ PRK03879 (PRK03879)UPF0086 (pfam01868)→ RpsQ (COG0186)rps17p (PRK08572)→ rpl14p (PRK08571)Ribosomal_L14 (pfam00238)→ rpl24p (PRK01191)KOW_RPL26 (cd06089)→ PRK04313 (PRK04313)S4 (cd00165)KOW_RPS4 (cd06087)Ribosomal_S4e (pfam00900)→ RplE (COG0094)rpl5p (PRK04219)→ RpsN (COG0199)rps14P (PRK05766)→ rps8p (PRK04034)RpsH (COG0096)→ RplF (COG0097)rpl6p (PRK05518)→ rpl32e (PRK08562)Ribosomal_L32e (pfam01655)→ rpl19e (PRK08570)Ribosomal_L19e (pfam01280)→ RplR (COG0256)rpl18p (PRK08569)→ RpsE (COG0098)rps5p (PRK04044)→ Ribosomal_L7_archeal_euk (cd01657)rpl30p (PRK06049)→
env-325	3300001580_10080578	-	953	870	**RNA**→ ←hypo
env-326	2232135516	+	86	169	**RNA**→
env-327	3300000558_11733700	-	1392	1309	**RNA**→ ←hypo
env-328	3300000558_11835925	-	418	335	**RNA**→

env-329	3300000568_10049962	-	21413	21330	**RNA**→ hypo→ RplC (COG0087)rpl3p (PRK04231)→ RplD (COG0088)rpl4lp (PRK04042)→ PRK14548 (PRK14548)Ribosomal_L23 (pfam00276)→ RplB (COG0090)rpl2p (PRK09612)→ rps19p (PRK04038)Ribosomal_S19 (pfam00203)→ rpl22p (PRK04223)RplV (COG0091)→ RpsC (COG0092)rps3p (PRK04191)→ PRK14549 (PRK14549)Ribosomal_L29 (pfam00831)→ PRK03879 (PRK03879)UPF0086 (pfam01868)→ RpsQ (COG0186)rps17p (PRK08572)→ rpl14p (PRK08571)Ribosomal_L14 (pfam00238)→ rpl24p (PRK01191)KOW_RPL26 (cd06089)→ PRK04313 (PRK04313)KOW_RPS4 (cd06087)Ribosomal_S4e (pfam00900)→ rpl5p (PRK04219)Ribosomal_L5 (pfam00281)Ribosomal_L5_C (pfam00673)→ RpsN (COG0199)rps14P (PRK05766)→ rps8p (PRK04034)RpsH (COG0096)→ RplF (COG0097)rpl6p (PRK05518)→ rpl32e (PRK08562)Ribosomal_L32e (pfam01655)→ rpl19e (PRK08570)Ribosomal_L19e (pfam01280)→ RplR (COG0256)rpl18p (PRK08569)→
env-330	3300001605_10004100	-	1325	1242	**RNA**→ ←hypo
env-331	3300000032_0004919	+	16062	16145	**RNA**→ RplC (COG0087)rpl3p (PRK04231)→ RplD (COG0088)rpl4lp (PRK04042)→ PRK14548 (PRK14548)Ribosomal_L23 (pfam00276)→ RplB (COG0090)rpl2p (PRK09612)→ rps19p (PRK04038)Ribosomal_S19 (pfam00203)→ rpl22p (PRK04223)RplV (COG0091)→ RpsC (COG0092)rps3p (PRK04191)→ PRK14549 (PRK14549)Ribosomal_L29 (pfam00831)→ PRK03879 (PRK03879)UPF0086 (pfam01868)→ RpsQ (COG0186)rps17p (PRK08572)→ rpl14p (PRK08571)Ribosomal_L14 (pfam00238)→ rpl24p (PRK01191)KOW_RPL26 (cd06089)→ PRK04313 (PRK04313)RPS4A (COG1471)→ KOW (smart00739)KOW_RPS4 (cd06087)→ rpl5p (PRK04219)Ribosomal_L5 (pfam00281)Ribosomal_L5_C (pfam00673)→ RpsN (COG0199)rps14P (PRK05766)→ rps8p (PRK04034)RpsH (COG0096)→ RplF (COG0097)rpl6p (PRK05518)→ rpl32e (PRK08562)Ribosomal_L32e (pfam01655)→ rpl19e (PRK08570)Ribosomal_L19e (pfam01280)→ RplR (COG0256)rpl18p (PRK08569)→
env-332	3300000185_104355	-	418	335	**RNA**→
Mfo-1-1	NC_019943.1	-	2583107	2583023	**RNA**→ RplC (COG0087)rpl3p (PRK04231)→ RplD (COG0088)rpl4lp (PRK04042)→ RplW (COG0089)PRK14548 (PRK14548)→ RplB (COG0090)rpl2p (PRK09612)→ rps19p (PRK04038)Ribosomal_S19 (pfam00203)→ rpl22p (PRK04223)RplV (COG0091)→ RpsC (COG0092)rps3p (PRK04191)→ PRK14549 (PRK14549)Ribosomal_L29 (pfam00831)→ PRK03879 (PRK03879)UPF0086 (pfam01868)→ RpsQ (COG0186)rps17p (PRK08572)→ rpl14p (PRK08571)Ribosomal_L14 (pfam00238)→ rpl24p (PRK01191)KOW_RPL26 (cd06089)→ PRK04313 (PRK04313)S4 (cd00165)KOW_RPS4 (cd06087)Ribosomal_S4e (pfam00900)→ rpl5p (PRK04219)Ribosomal_L5 (pfam00281)Ribosomal_L5_C (pfam00673)→ RpsN (COG0199)rps14P (PRK05766)→ rps8p (PRK04034)RpsH (COG0096)→ RplF (COG0097)rpl6p (PRK05518)→ rpl32e (PRK08562)Ribosomal_L32e (pfam01655)→ rpl19e (PRK08570)Ribosomal_L19e (pfam01280)→ RplR (COG0256)rpl18p (PRK08569)→
env-333	JGI20225J20221_1000316	-	54770	54691	**RNA**→ RplC (COG0087)rpl3p (PRK04231)→ RplD (COG0088)rpl4lp (PRK04042)→ RplW (COG0089)PRK14548 (PRK14548)→ RplB (COG0090)rpl2p (PRK09612)→ rps19p (PRK04038)Ribosomal_S19 (pfam00203)→ rpl22p (PRK04223)RplV (COG0091)→ RpsC (COG0092)rps3p (PRK04191)→ PRK14549 (PRK14549)Ribosomal_L29 (pfam00831)→ PRK03879 (PRK03879)UPF0086 (pfam01868)→ RpsQ (COG0186)rps17p (PRK08572)→ rpl14p (PRK08571)Ribosomal_L14 (pfam00238)→ rpl24p (PRK01191)KOW_RPL26 (cd06089)→ PRK04313 (PRK04313)S4 (cd00165)KOW_RPS4 (cd06087)Ribosomal_S4e (pfam00900)→ RplE (COG0094)rpl5p (PRK04219)→ RpsN (COG0199)rps14P (PRK05766)→ rps8p (PRK04034)RpsH (COG0096)→ RplF (COG0097)rpl6p (PRK05518)→ rpl32e (PRK08562)Ribosomal_L32e (pfam01655)→ rpl19e (PRK08570)Ribosomal_L19e (pfam01280)→ RplR (COG0256)rpl18p (PRK08569)→
env-334	BMHB3a_c145790	-	1545	1466	**RNA**→ RplC (COG0087)rpl3p (PRK04231)→ RplD (COG0088)rpl4lp (PRK04042)→
env-335	JGI24730J26740_1000194	-	6399	6320	**RNA**→ RplC (COG0087)rpl3p (PRK04231)→ RplD (COG0088)rpl4lp (PRK04042)→ RplW (COG0089)PRK14548 (PRK14548)→ RplB (COG0090)rpl2p (PRK09612)→ rps19p (PRK04038)Ribosomal_S19 (pfam00203)→ rpl22p (PRK04223)RplV (COG0091)→ RpsC (COG0092)rps3p (PRK04191)→ PRK14549 (PRK14549)Ribosomal_L29 (pfam00831)→ PRK03879 (PRK03879)UPF0086 (pfam01868)→ RpsQ (COG0186)rps17p (PRK08572)→ rpl14p (PRK08571)Ribosomal_L14 (pfam00238)→ rpl24p (PRK01191)KOW_RPL26 (cd06089)→ PRK04313 (PRK04313)RPS4A (COG1471)→

env-336	JGI24730J26740_1000273	-	21541	21462	RNA→ RplC (COG0087)rpl3p (PRK04231)→ RplD (COG0088)rpl4lp (PRK04042)→ RplW (COG0089)PRK14548 (PRK14548)→ RplB (COG0090)rpl2p (PRK09612)→ rps19p (PRK04038)Ribosomal_S19 (pfam00203)→ rpl22p (PRK04223)RplV (COG0091)→ RpsC (COG0092)rps3p (PRK04191)→ PRK14549 (PRK14549)Ribosomal_L29 (pfam00831)→ PRK03879 (PRK03879)UPF0086 (pfam01868)→ RpsQ (COG0186)rps17p (PRK08572)→ rpl14p (PRK08571)Ribosomal_L14 (pfam00238)→ rpl24p (PRK01191)KOW_RPL26 (cd06089)→ PRK04313 (PRK04313)S4 (cd00165)KOW_RPS4 (cd06087)Ribosomal_S4e (pfam00900)→ RplE (COG0094)rpl5p (PRK04219)→ RpsN (COG0199)rps14P (PRK05766)→ rps8p (PRK04034)RpsH (COG0096)→ RplF (COG0097)rpl6p (PRK05518)→ rpl32e (PRK08562)Ribosomal_L32e (pfam01655)→ rpl19e (PRK08570)Ribosomal_L19e (pfam01280)→ RplR (COG0256)rpl18p (PRK08569)→
env-337	2013874846	+	4629	4707	RNA→ RplC (COG0087)rpl3p (PRK04231)→ RplD (COG0088)rpl4lp (PRK04042)→ RplW (COG0089)PRK14548 (PRK14548)→
env-338	PRSSGFe2_Sequence0000003268	+	541	620	RNA→ hypo→
env-339	WSSedL1Cam_c0014	-	25929	25851	RNA→ RplC (COG0087)rpl3p (PRK04231)→ RplD (COG0088)rpl4lp (PRK04042)→ RplW (COG0089)PRK14548 (PRK14548)→ RplB (COG0090)rpl2p (PRK09612)→ rps19p (PRK04038)Ribosomal_S19 (pfam00203)→ rpl22p (PRK04223)RplV (COG0091)→ RpsC (COG0092)rps3p (PRK04191)→ PRK14549 (PRK14549)Ribosomal_L29 (pfam00831)→ PRK03879 (PRK03879)UPF0086 (pfam01868)→ RpsQ (COG0186)rps17p (PRK08572)→ rpl14p (PRK08571)Ribosomal_L14 (pfam00238)→ rpl24p (PRK01191)KOW_RPL26 (cd06089)→ PRK04313 (PRK04313)S4 (cd00165)KOW_RPS4 (cd06087)Ribosomal_S4e (pfam00900)→ RplE (COG0094)rpl5p (PRK04219)→ RpsN (COG0199)rps14P (PRK05766)→ rps8p (PRK04034)RpsH (COG0096)→ RplF (COG0097)rpl6p (PRK05518)→ rpl32e (PRK08562)Ribosomal_L32_L32e (cd00513)→ rpl19e (PRK08570)Ribosomal_L19e (pfam01280)→ RplR (COG0256)rpl18p (PRK08569)→
CMe-1-1	NC_009712.1	+	517727	517802	RNA→ RplC (COG0087)rpl3p (PRK04231)→ RplD (COG0088)rpl4lp (PRK04042)→ RplW (COG0089)PRK14548 (PRK14548)→ RplB (COG0090)rpl2p (PRK09612)→ rps19p (PRK04038)Ribosomal_S19 (pfam00203)→ rpl22p (PRK04223)RplV (COG0091)→ RpsC (COG0092)rps3p (PRK04191)→ PRK14549 (PRK14549)Ribosomal_L29 (pfam00831)→ PRK03879 (PRK03879)UPF0086 (pfam01868)→ RpsQ (COG0186)rps17p (PRK08572)→ rpl14p (PRK08571)Ribosomal_L14 (pfam00238)→ rpl24p (PRK01191)KOW_RPL26 (cd06089)→ PRK04313 (PRK04313)S4 (cd00165)KOW_RPS4 (cd06087)Ribosomal_S4e (pfam00900)→ RplE (COG0094)rpl5p (PRK04219)→ RpsN (COG0199)rps14P (PRK05766)→ rps8p (PRK04034)RpsH (COG0096)→ RplF (COG0097)rpl6p (PRK05518)→ rpl32e (PRK08562)Ribosomal_L32_L32e (cd00513)→ rpl19e (PRK08570)Ribosomal_L19e (pfam01280)→ RplR (COG0256)rpl18p (PRK08569)→
env-340	3300001567_10001587	+	11347	11431	RNA→ rpl3p (PRK04231)Ribosomal_L3 (pfam00297)→
env-341	WSSedA2C_c003915	-	773	689	RNA→ rpl3p (PRK04231)Ribosomal_L3 (pfam00297)→
env-342	WSSedA1TJGI_c001789	-	1725	1641	RNA→ RplC (COG0087)rpl3p (PRK04231)→ RplD (COG0088)rpl4lp (PRK04042)→
env-343	WSSedB1B2_c003929	-	407	323	RNA→
env-344	WSSedA1Ba_1001899	-	3122	3038	RNA→ ←hypo
env-345	WSSedA1Ba2_1001418	-	2992	2908	RNA→ ←hypo
env-346	WSSedA1Ba3_1004880	-	2426	2342	RNA→ ←hypo
env-347	WSSedA2Ca3_1003915	-	773	689	RNA→ ←hypo
env-348	JGI20214J14112_1009109	+	1012	1096	RNA→ rpl3p (PRK04231)Ribosomal_L3 (pfam00297)→
env-349	JGI20213J14113_1038457	-	635	551	RNA→ ←hypo
env-350	B3_all_c_Cons72656	+	183	267	RNA→ RplC (COG0087)rpl3p (PRK04231)→ RplD (COG0088)rpl4lp (PRK04042)→
env-351	3300000568_10104459	-	1600	1514	RNA→ RplC (COG0087)rpl3p (PRK04231)→ RplD (COG0088)rpl4lp (PRK04042)→
env-352	SakLake120mDRAFT_c0023947	-	758	684	RNA→ rpl3p (PRK04231)Ribosomal_L3 (pfam00297)→
env-353	3300000032_0022845	-	6964	6878	RNA→ RplC (COG0087)rpl3p (PRK04231)→ RplD (COG0088)rpl4lp (PRK04042)→
env-354	3300000568_10039005	-	399	313	RNA→ ←hypo
env-355	3300001567_10000694	+	33701	33787	RNA→ ←hypo
env-356	3300000558_11950893	-	214	128	RNA→ ←hypo
env-357	JGI26437J51864_10016855	+	1418	1504	RNA→

env-358	WSSedA1Ba_1000820	-	2896	2810	RNA→ RplC (COG0087)rpl3p (PRK04231)→ RplD (COG0088)rpl4lp (PRK04042)→ RplW (COG0089)PRK14548 (PRK14548)→ RplB (COG0090)rpl2p (PRK09612)→
env-359	JGI20214J14112_1000181	-	5712	5626	RNA→ RplC (COG0087)rpl3p (PRK04231)→ RplD (COG0088)rpl4lp (PRK04042)→ RplW (COG0089)PRK14548 (PRK14548)→ RplB (COG0090)rpl2p (PRK09612)→ rps19p (PRK04038)Ribosomal_S19 (pfam00203)→ rpl22p (PRK04223)RplV (COG0091)→ RpsC (COG0092)rps3p (PRK04191)→ PRK14549 (PRK14549)Ribosomal_L29 (pfam00831)→ PRK03879 (PRK03879)UPF0086 (pfam01868)→ RpsQ (COG0186)rps17p (PRK08572)→ rpl14p (PRK08571)Ribosomal_L14 (pfam00238)→
env-360	WSSedB2Ca_1001724	-	887	801	RNA→ rpl3p (PRK04231)Ribosomal_L3 (pfam00297)→
env-361	WSSedA2C_c001213	+	2849	2935	RNA→ rpl3p (PRK04231)Ribosomal_L3 (pfam00297)→
env-362	WSSedA1TJGI_c002576	+	889	975	RNA→ RplC (COG0087)rpl3p (PRK04231)→ RplD (COG0088)rpl4lp (PRK04042)→ RplW (COG0089)PRK14548 (PRK14548)→ RplB (COG0090)rpl2p (PRK09612)→
env-363	WSSedB1B2_c000733	-	1648	1562	RNA→ rpl3p (PRK04231)Ribosomal_L3 (pfam00297)→
env-364	WSSedB2T_c003532	+	434	520	RNA→ rpl3p (PRK04231)Ribosomal_L3 (pfam00297)→
env-365	WSSedA1Ba2_1000822	-	2885	2799	RNA→ RplC (COG0087)rpl3p (PRK04231)→ RplD (COG0088)rpl4lp (PRK04042)→ RplW (COG0089)PRK14548 (PRK14548)→ RplB (COG0090)rpl2p (PRK09612)→
env-366	WSSedA1Ba3_1000489	+	1543	1629	RNA→ RplC (COG0087)rpl3p (PRK04231)→ RplD (COG0088)rpl4lp (PRK04042)→ RplW (COG0089)PRK14548 (PRK14548)→ RplB (COG0090)rpl2p (PRK09612)→ rps19p (PRK04038)Ribosomal_S19 (pfam00203)→ rpl22p (PRK04223)RplV (COG0091)→ RpsC (COG0092)rps3p (PRK04191)→ PRK14549 (PRK14549)Ribosomal_L29 (pfam00831)→ PRK03879 (PRK03879)UPF0086 (pfam01868)→ RpsQ (COG0186)rps17p (PRK08572)→ rpl14p (PRK08571)Ribosomal_L14 (pfam00238)→ rpl24p (PRK01191)KOW_RPL26 (cd06089)→ PRK04313 (PRK04313)KOW_RPS4 (cd06087)Ribosomal_S4e (pfam00900)→ rpl5p (PRK04219)Ribosomal_L5 (pfam00281)Ribosomal_L5_C (pfam00673)→ RpsN (COG0199)rps14P (PRK05766)→ rps8p (PRK04034)RpsH (COG0096)→ RplF (COG0097)rpl6p (PRK05518)→ rpl32e (PRK08562)Ribosomal_L32e (pfam01655)→ rpl19e (PRK08570)Ribosomal_L19e (pfam01280)→ rpl18p (PRK08569)→
env-367	WSSedB2Ba_1001051	-	1502	1416	RNA→ RplC (COG0087)rpl3p (PRK04231)→ RplD (COG0088)rpl4lp (PRK04042)→
env-368	WSSedA2Ca3_1001213	+	2849	2935	RNA→ rpl3p (PRK04231)Ribosomal_L3 (pfam00297)→
env-369	JGI20213J14113_1000441	-	1013	927	RNA→ rpl3p (PRK04231)Ribosomal_L3 (pfam00297)→
env-370	JGI26437J51864_10613048	+	135	220	RNA→ hypo→
env-371	3300000568_10163768	+	37	123	RNA→ hypo→
env-372	JGI20213J14113_1352303	+	18	105	RNA→ ←hypo
env-373	WSSedB1B2_c001061	-	813	728	RNA→ hypo→
env-374	JGI26437J51864_10024878	+	905	990	RNA→
env-375	LWFCAn_GLQU6AQ01CDIKX	-	257	171	RNA→ hypo→
env-376	JGI24732J26686_1167705	-	325	240	RNA→ hypo→
env-377	JGI24732J26686_1058151	-	388	303	RNA→ hypo→
env-378	GYO3UBL02HBW2K	-	172	86	RNA→ hypo→
env-379	LWFCAn_GLO1YSU01C0WMX	+	42	125	RNA→ ←hypo
env-380	3300000558_10014112	+	936	1015	RNA→ RplC (COG0087)rpl3p (PRK04231)→ RplD (COG0088)rpl4lp (PRK04042)→ RplW (COG0089)PRK14548 (PRK14548)→ RplB (COG0090)rpl2p (PRK09612)→ rps19p (PRK04038)Ribosomal_S19 (pfam00203)→ rpl22p (PRK04223)RplV (COG0091)→ RpsC (COG0092)rps3p (PRK04191)→ PRK14549 (PRK14549)Ribosomal_L29 (pfam00831)→ PRK03879 (PRK03879)UPF0086 (pfam01868)→ RpsQ (COG0186)rps17p (PRK08572)→ rpl14p (PRK08571)Ribosomal_L14 (pfam00238)→ rpl24p (PRK01191)KOW_RPL26 (cd06089)→ PRK04313 (PRK04313)KOW_RPS4 (cd06087)Ribosomal_S4e (pfam00900)→ rpl5p (PRK04219)Ribosomal_L5 (pfam00281)Ribosomal_L5_C (pfam00673)→ RpsN (COG0199)rps14P (PRK05766)→ rps8p (PRK04034)RpsH (COG0096)→ rpl6p (PRK05518)Ribosomal_L6 (pfam00347)→
env-381	3300001580_10840377	-	216	137	RNA→ ←hypo
env-382	JGI20214J14112_1015362	+	514	593	RNA→ rpl3p (PRK04231)Ribosomal_L3 (pfam00297)→
env-383	3300001605_10335424	-	366	287	RNA→
env-384	JGI26437J51864_10189827	+	91	170	RNA→ ←hypo
env-385	JGI26437J51864_10656079	-	220	141	RNA→ ←hypo

env-386	3300001580_10004519	-	9169	9090	**RNA**→ RplC (COG0087)rpl3p (PRK04231)→ RplD (COG0088)rpl4lp (PRK04042)→<br>RplW (COG0089)PRK14548 (PRK14548)→ RplB (COG0090)rpl2p (PRK09612)→<br>rps19p (PRK04038)Ribosomal_S19 (pfam00203)→ rpl22p (PRK04223)RplV (COG0091)→<br>RpsC (COG0092)rps3p (PRK04191)→ PRK14549 (PRK14549)Ribosomal_L29 (pfam00831)→<br>PRK03879 (PRK03879)UPF0086 (pfam01868)→ RpsQ (COG0186)rps17p (PRK08572)→<br>rpl14p (PRK08571)Ribosomal_L14 (pfam00238)→ rpl24p (PRK01191)KOW_RPL26 (cd06089)→<br>PRK04313 (PRK04313)KOW_RPS4 (cd06087)Ribosomal_S4e (pfam00900)→<br>rpl5p (PRK04219)Ribosomal_L5 (pfam00281)Ribosomal_L5_C (pfam00673)→<br>RpsN (COG0199)rps14P (PRK05766)→ rps8p (PRK04034)RpsH (COG0096)→<br>RplF (COG0097)rpl6p (PRK05518)→ rpl32e (PRK08562)Ribosomal_L32e (pfam01655)→
env-387	3300000032_0388072	-	284	205	**RNA**→
env-388	3300001605_10952026	+	300	379	**RNA**→ ←hypo
env-389	B3_all_c_Cons98067	+	267	345	**RNA**→ rpl3p (PRK04231)Ribosomal_L3 (pfam00297)→
env-390	LWAnNiSIP_F624WLL02IC2DN	-	276	191	**RNA**→ hypo→
env-391	LWAnN_F624WLL02IC2DN	-	276	191	**RNA**→ hypo→
env-392	JGI24728J21555_1000024	+	55297	55372	**RNA**→ RplC (COG0087)rpl3p (PRK04231)→ RplD (COG0088)rpl4lp (PRK04042)→<br>RplW (COG0089)PRK14548 (PRK14548)→ RplB (COG0090)rpl2p (PRK09612)→<br>rps19p (PRK04038)Ribosomal_S19 (pfam00203)→ rpl22p (PRK04223)RplV (COG0091)→<br>RpsC (COG0092)rps3p (PRK04191)→ PRK14549 (PRK14549)Ribosomal_L29 (pfam00831)→<br>PRK03879 (PRK03879)UPF0086 (pfam01868)→ RpsQ (COG0186)rps17p (PRK08572)→<br>rpl14p (PRK08571)Ribosomal_L14 (pfam00238)→ rpl24p (PRK01191)KOW_RPL26 (cd06089)→<br>PRK04313 (PRK04313)KOW_RPS4 (cd06087)Ribosomal_S4e (pfam00900)→<br>rpl5p (PRK04219)Ribosomal_L5 (pfam00281)Ribosomal_L5_C (pfam00673)→<br>RpsN (COG0199)rps14P (PRK05766)→ rps8p (PRK04034)RpsH (COG0096)→<br>RplF (COG0097)rpl6p (PRK05518)→ rpl32e (PRK08562)Ribosomal_L32_L32e (cd00513)→ hypo→ hypo→<br>rpl19e (PRK08570)Ribosomal_L19e (pfam01280)→ RplR (COG0256)rpl18p (PRK08569)→
env-393	JGI24731J21663_1041451	+	763	838	**RNA**→
env-394	JGI24732J26686_1000141	+	22138	22213	**RNA**→ RplC (COG0087)rpl3p (PRK04231)→ RplD (COG0088)rpl4lp (PRK04042)→<br>RplW (COG0089)PRK14548 (PRK14548)→ RplB (COG0090)rpl2p (PRK09612)→<br>rps19p (PRK04038)Ribosomal_S19 (pfam00203)→ rpl22p (PRK04223)RplV (COG0091)→<br>RpsC (COG0092)rps3p (PRK04191)→ PRK14549 (PRK14549)Ribosomal_L29 (pfam00831)→<br>PRK03879 (PRK03879)UPF0086 (pfam01868)→ RpsQ (COG0186)rps17p (PRK08572)→<br>rpl14p (PRK08571)Ribosomal_L14 (pfam00238)→ rpl24p (PRK01191)KOW_RPL26 (cd06089)→<br>PRK04313 (PRK04313)KOW_RPS4 (cd06087)Ribosomal_S4e (pfam00900)→<br>rpl5p (PRK04219)Ribosomal_L5 (pfam00281)Ribosomal_L5_C (pfam00673)→<br>RpsN (COG0199)rps14P (PRK05766)→ rps8p (PRK04034)RpsH (COG0096)→<br>RplF (COG0097)rpl6p (PRK05518)→ rpl32e (PRK08562)Ribosomal_L32_L32e (cd00513)→ hypo→ hypo→<br>rpl19e (PRK08570)Ribosomal_L19e (pfam01280)→ RplR (COG0256)rpl18p (PRK08569)→
env-395	JGI24729J20445_1000265	-	14288	14211	**RNA**→ RplC (COG0087)rpl3p (PRK04231)→ RplD (COG0088)rpl4lp (PRK04042)→<br>RplW (COG0089)PRK14548 (PRK14548)→ RplB (COG0090)rpl2p (PRK09612)→<br>rps19p (PRK04038)Ribosomal_S19 (pfam00203)→ rpl22p (PRK04223)RplV (COG0091)→<br>RpsC (COG0092)rps3p (PRK04191)→ PRK14549 (PRK14549)Ribosomal_L29 (pfam00831)→<br>PRK03879 (PRK03879)UPF0086 (pfam01868)→ RpsQ (COG0186)rps17p (PRK08572)→<br>rpl14p (PRK08571)Ribosomal_L14 (pfam00238)→ rpl24p (PRK01191)KOW_RPL26 (cd06089)→<br>PRK04313 (PRK04313)S4 (cd00165)KOW_RPS4 (cd06087)Ribosomal_S4e (pfam00900)→<br>RplE (COG0094)rpl5p (PRK04219)→ RpsN (COG0199)rps14P (PRK05766)→<br>rps8p (PRK04034)RpsH (COG0096)→ RplF (COG0097)rpl6p (PRK05518)→<br>rpl32e (PRK08562)Ribosomal_L32e (pfam01655)→ rpl19e (PRK08570)Ribosomal_L19e (pfam01280)→<br>RplR (COG0256)rpl18p (PRK08569)→

env-396	JGI24729J20445_1000376	-	22541	22464	**RNA**→ RplC (COG0087)rpl3p (PRK04231)→ RplD (COG0088)rpl4lp (PRK04042)→ RplW (COG0089)PRK14548 (PRK14548)→ RplB (COG0090)rpl2p (PRK09612)→ rps19p (PRK04038)Ribosomal_S19 (pfam00203)→ rpl22p (PRK04223)RplV (COG0091)→ RpsC (COG0092)rps3p (PRK04191)→ PRK14549 (PRK14549)Ribosomal_L29 (pfam00831)→ PRK03879 (PRK03879)UPF0086 (pfam01868)→ RpsQ (COG0186)rps17p (PRK08572)→ rpl14p (PRK08571)Ribosomal_L14 (pfam00238)→ rpl24p (PRK01191)KOW_RPL26 (cd06089)→ PRK04313 (PRK04313)S4 (cd00165)KOW_RPS4 (cd06087)Ribosomal_S4e (pfam00900)→ RplE (COG0094)rpl5p (PRK04219)→ RpsN (COG0199)rps14P (PRK05766)→ rps8p (PRK04034)RpsH (COG0096)→ RplF (COG0097)rpl6p (PRK05518)→ rpl32e (PRK08562)Ribosomal_L32e (pfam01655)→ rpl19e (PRK08570)Ribosomal_L19e (pfam01280)→ RplR (COG0256)rpl18p (PRK08569)→
env-397	JGI24732J26686_1000166	-	22911	22834	**RNA**→ RplC (COG0087)rpl3p (PRK04231)→ RplD (COG0088)rpl4lp (PRK04042)→ RplW (COG0089)PRK14548 (PRK14548)→ RplB (COG0090)rpl2p (PRK09612)→ rps19p (PRK04038)Ribosomal_S19 (pfam00203)→ rpl22p (PRK04223)RplV (COG0091)→ RpsC (COG0092)rps3p (PRK04191)→ PRK14549 (PRK14549)Ribosomal_L29 (pfam00831)→ PRK03879 (PRK03879)UPF0086 (pfam01868)→ RpsQ (COG0186)rps17p (PRK08572)→ rpl14p (PRK08571)Ribosomal_L14 (pfam00238)→ rpl24p (PRK01191)KOW_RPL26 (cd06089)→ PRK04313 (PRK04313)S4 (cd00165)KOW_RPS4 (cd06087)Ribosomal_S4e (pfam00900)→ RplE (COG0094)rpl5p (PRK04219)→ RpsN (COG0199)rps14P (PRK05766)→ rps8p (PRK04034)RpsH (COG0096)→ RplF (COG0097)rpl6p (PRK05518)→ rpl32e (PRK08562)Ribosomal_L32e (pfam01655)→ rpl19e (PRK08570)Ribosomal_L19e (pfam01280)→ RplR (COG0256)rpl18p (PRK08569)→
env-398	JGI24730J26740_1278979	+	62	139	**RNA**→ ←hypo
env-399	JGI20214J14112_1005972	+	608	694	**RNA**→ hypo→ RplC (COG0087)rpl3p (PRK04231)→
env-400	3300000558_10001908	-	3593	3508	**RNA**→ hypo→
env-401	3300001605_12127170	+	26	111	**RNA**→ hypo→
env-402	JGI24732J26686_1168257	-	324	239	**RNA**→ hypo→
env-403	JGI24729J20445_1001367	-	9125	9040	**RNA**→ RplC (COG0087)rpl3p (PRK04231)→ RplD (COG0088)rpl4lp (PRK04042)→ RplW (COG0089)PRK14548 (PRK14548)→ RplB (COG0090)rpl2p (PRK09612)→ rps19p (PRK04038)Ribosomal_S19 (pfam00203)→ rpl22p (PRK04223)RplV (COG0091)→ RpsC (COG0092)rps3p (PRK04191)→ PRK14549 (PRK14549)Ribosomal_L29 (pfam00831)→ PRK03879 (PRK03879)UPF0086 (pfam01868)→ RpsQ (COG0186)rps17p (PRK08572)→ rpl14p (PRK08571)Ribosomal_L14 (pfam00238)→ rpl24p (PRK01191)KOW_RPL26 (cd06089)→ PRK04313 (PRK04313)S4 (cd00165)KOW_RPS4 (cd06087)Ribosomal_S4e (pfam00900)→ rpl5p (PRK04219)Ribosomal_L5 (pfam00281)Ribosomal_L5_C (pfam00673)→ RpsN (COG0199)rps14P (PRK05766)→ rps8p (PRK04034)RpsH (COG0096)→ RplF (COG0097)rpl6p (PRK05518)→ rpl32e (PRK08562)Ribosomal_L32e (pfam01655)→
env-404	JGI24730J26740_1168855	+	233	318	**RNA**→ hypo→
env-405	JGI24728J21555_1218516	-	187	102	**RNA**→ ←hypo
env-406	JGI24729J20445_1004575	+	4026	4111	**RNA**→
env-407	WSSedA1Ba3_1333478	-	172	89	**RNA**→ hypo→

Mli-2-1	NZ_CM001436.1	-	2884644	2884571	**RNA**→ RplC (COG0087)rpl3p (PRK04231)→ RplC (COG0087)rpl3p (PRK04231)→ RplD (COG0088)rpl4lp (PRK04042)→ RplD (COG0088)rpl4lp (PRK04042)→ RplW (COG0089)PRK14548 (PRK14548)→ RplW (COG0089)PRK14548 (PRK14548)→ RplB (COG0090)rpl2p (PRK09612)→ RplB (COG0090)rpl2p (PRK09612)→ rps19p (PRK04038)Ribosomal_S19 (pfam00203)→ rps19p (PRK04038)Ribosomal_S19 (pfam00203)→ rpl22p (PRK04223)RplV (COG0091)→ rpl22p (PRK04223)RplV (COG0091)→ RpsC (COG0092)rps3p (PRK04191)→ RpsC (COG0092)rps3p (PRK04191)→ PRK14549 (PRK14549)Ribosomal_L29 (pfam00831)→ PRK14549 (PRK14549)Ribosomal_L29 (pfam00831)→ PRK03879 (PRK03879)UPF0086 (pfam01868)→ PRK03879 (PRK03879)UPF0086 (pfam01868)→ RpsQ (COG0186)rps17p (PRK08572)→ RpsQ (COG0186)rps17p (PRK08572)→ rpl14p (PRK08571)Ribosomal_L14 (pfam00238)→ rpl14p (PRK08571)Ribosomal_L14 (pfam00238)→ rpl24p (PRK01191)KOW_RPL26 (cd06089)→ rpl24p (PRK01191)KOW_RPL26 (cd06089)→ PRK04313 (PRK04313)KOW_RPS4 (cd06087)Ribosomal_S4e (pfam00900)→ PRK04313 (PRK04313)KOW_RPS4 (cd06087)Ribosomal_S4e (pfam00900)→ RplE (COG0094)rpl5p (PRK04219)→ RplE (COG0094)rpl5p (PRK04219)→ RpsN (COG0199)rps14P (PRK05766)→ RpsN (COG0199)rps14P (PRK05766)→ rps8p (PRK04034)RpsH (COG0096)→ rps8p (PRK04034)RpsH (COG0096)→ RplF (COG0097)uL6_arch (TIGR03653)→ RplF (COG0097)uL6_arch (TIGR03653)→ rpl32e (PRK08562)Ribosomal_L32e (pfam01655)→ rpl32e (PRK08562)Ribosomal_L32e (pfam01655)→ rpl19e (PRK08570)Ribosomal_L19e (pfam01280)→ rpl19e (PRK08570)Ribosomal_L19e (pfam01280)→ RplR (COG0256)rpl18p (PRK08569)→ RplR (COG0256)rpl18p (PRK08569)→ RpsE (COG0098)rps5p (PRK04044)→ RpsE (COG0098)rps5p (PRK04044)→
Mpe-1-1	NC_014507.1	+	2206565	2206637	**RNA**→ RplC (COG0087)rpl3p (PRK04231)→ RplD (COG0088)rpl4lp (PRK04042)→ RplW (COG0089)PRK14548 (PRK14548)→ RplB (COG0090)rpl2p (PRK09612)→ RpsS (COG0185)rps19p (PRK04038)→ rpl22p (PRK04223)RplV (COG0091)→ RpsC (COG0092)rps3p (PRK04191)→ PRK14549 (PRK14549)Ribosomal_L29 (pfam00831)→ PRK03879 (PRK03879)UPF0086 (pfam01868)→ RpsQ (COG0186)rps17p (PRK08572)→ rpl14p (PRK08571)Ribosomal_L14 (pfam00238)→ rpl24p (PRK01191)KOW_RPL26 (cd06089)→ PRK04313 (PRK04313)KOW_RPS4 (cd06087)Ribosomal_S4e (pfam00900)→ RplE (COG0094)rpl5p (PRK04219)→ RpsN (COG0199)rps14P (PRK05766)→ rps8p (PRK04034)RpsH (COG0096)→ RplF (COG0097)rpl6p (PRK05518)→ hypo→ rpl32e (PRK08562)Ribosomal_L32e (pfam01655)→ rpl19e (PRK08570)Ribosomal_L19e (pfam01280)→ RplR (COG0256)rpl18p (PRK08569)→ RpsE (COG0098)rps5p (PRK04044)→
Mpa-2-1	NZ_KN360930.1	-	82807	82734	**RNA**→ ←hypo
env-408	M940CN_1020281	+	3372	3442	**RNA**→ rpl3p (PRK04231)Ribosomal_L3 (pfam00297)→
Mmo-1-1	NZ_JOMF01000007.1	+	9542	9636	**RNA**→ RplC (COG0087)rpl3p (PRK04231)→ RplD (COG0088)rpl4lp (PRK04042)→ RplW (COG0089)PRK14548 (PRK14548)→ RplB (COG0090)rpl2p (PRK09612)→ RpsS (COG0185)rps19p (PRK04038)→ rpl22p (PRK04223)RplV (COG0091)→ RpsC (COG0092)rps3p (PRK04191)→ PRK14549 (PRK14549)Ribosomal_L29 (pfam00831)→ POP4 (COG1588)PRK03879 (PRK03879)→ RpsQ (COG0186)rps17p (PRK08572)→ rpl14p (PRK08571)Ribosomal_L14 (pfam00238)→ RplX (COG0198)rpl24p (PRK01191)→ PRK04313 (PRK04313)KOW_RPS4 (cd06087)Ribosomal_S4e (pfam00900)→ RplE (COG0094)rpl5p (PRK04219)→ RpsN (COG0199)rps14P (PRK05766)→ rps8p (PRK04034)RpsH (COG0096)→ RplF (COG0097)rpl6p (PRK05518)→ rpl32e (PRK08562)Ribosomal_L32e (pfam01655)→ rpl19e (PRK08570)Ribosomal_L19e (pfam01280)→ RplR (COG0256)rpl18p (PRK08569)→ RpsE (COG0098)rps5p (PRK04044)→
env-409	RUMENNODE_4185760_38501	-	73103	73009	**RNA**→ RplC (COG0087)rpl3p (PRK04231)→ RplD (COG0088)rpl4lp (PRK04042)→ RplW (COG0089)PRK14548 (PRK14548)→ RplB (COG0090)rpl2p (PRK09612)→ rpl2p (PRK09612)→ RpsS (COG0185)rps19p (PRK04038)→ rpl22p (PRK04223)RplV (COG0091)→ KH (smart00322)archeal_30S_S3_KH (cd02411)→ rps3p (PRK04191)Ribosomal_S3_C (pfam00189)→ PRK14549 (PRK14549)Ribosomal_L29 (pfam00831)→ POP4 (COG1588)PRK03879 (PRK03879)→ RpsQ (COG0186)rps17p (PRK08572)→ rpl14p (PRK08571)Ribosomal_L14 (pfam00238)→ RplX (COG0198)rpl24p (PRK01191)→ PRK04313 (PRK04313)Ribosomal_S4e (pfam00900)→ PRK04313 (PRK04313)KOW_RPS4 (cd06087)→ RplE (COG0094)rpl5p (PRK04219)→ RpsN (COG0199)rps14P (PRK05766)→ rps8p (PRK04034)RpsH (COG0096)→ RplF (COG0097)rpl6p (PRK05518)→

env-410	SL_5KL_010_SED_10008304	+	2725	2796	**RNA**→ RplC (COG0087)rpl3p (PRK04231)→ RplD (COG0088)rpl4lp (PRK04042)→
env-411	LCWY01003686.1	+	978	1049	**RNA**→ hypo→
env-412	LCWZ01000078.1	+	773	844	**RNA**→ RplC (COG0087)rpl3p (PRK04231)→ RplD (COG0088)rpl4lp (PRK04042)→<br>RplW (COG0089)PRK14548 (PRK14548)→ RplB (COG0090)rpl2p (PRK09612)→<br>RpsS (COG0185)rps19p (PRK04038)→ rpl22p (PRK04223)RplV (COG0091)→<br>RpsC (COG0092)rps3p (PRK04191)→ PRK14549 (PRK14549)Ribosomal_L29 (pfam00831)→<br>PRK03879 (PRK03879)UPF0086 (pfam01868)→ RpsQ (COG0186)rps17p (PRK08572)→<br>rpl14p (PRK08571)Ribosomal_L14 (pfam00238)→ rpl24p (PRK01191)KOW_RPL26 (cd06089)→<br>PRK04313 (PRK04313)KOW_RPS4 (cd06087)S4 (pfam01479)Ribosomal_S4e (pfam00900)→<br>rpl5p (PRK04219)Ribosomal_L5 (pfam00281)Ribosomal_L5_C (pfam00673)→<br>RpsN (COG0199)rps14P (PRK05766)→ rps8p (PRK04034)→ rps8p (PRK04034)Ribosomal_S8 (pfam00410)→<br>RplF (COG0097)rpl6p (PRK05518)→ hypo→ Rpl32e (COG1717)rpl32e (PRK08562)→<br>rpl19e (PRK08570)Ribosomal_L19e (pfam01280)→ RplR (COG0256)rpl18p (PRK08569)→<br>RpsE (COG0098)rps5p (PRK04044)→ Ribosomal_L30_like (cd00355)rpl30p (PRK06049)→<br>Ribosomal_L7_archeal_euk (cd01657)rpl30p (PRK06049)→ RplO (COG0200)rpl15p (PRK06419)→
Mco-1-1	NC_017034.1	+	1276328	1276418	**RNA**→ MTH1 (COG2106)→
env-413	JGI24024J18818_10045249	+	1393	1489	**RNA**→ Methyltrn_RNA_3 (pfam02598)→
env-414	LGVD01025758.1	-	1753	1657	**RNA**→ Methyltrn_RNA_3 (pfam02598)→
env-415	ASHM260b_GM9OZ8V02GZRNU	+	167	263	**RNA**→ hypo→
env-416	LGVE01111852.1	-	441	345	**RNA**→ Methyltrn_RNA_3 (pfam02598)→
env-417	LGVF01232254.1	-	138	42	**RNA**→ Methyltrn_RNA_3 (pfam02598)→
env-418	LGOV01013195.1	+	3349	3445	**RNA**→ Methyltrn_RNA_3 (pfam02598)→ RplC (COG0087)rpl3p (PRK04231)→<br>RplD (COG0088)rpl4lp (PRK04042)→ RplW (COG0089)PRK14548 (PRK14548)→<br>RplB (COG0090)rpl2p (PRK09612)→ RpsS (COG0185)rps19p (PRK04038)→<br>rpl22p (PRK04223)Ribosomal_L22 (pfam00237)→ rps3p (PRK04191)Ribosomal_S3_C (pfam00189)→<br>PRK14549 (PRK14549)Ribosomal_L29 (pfam00831)→ PRK03879 (PRK03879)UPF0086 (pfam01868)→<br>RpsQ (COG0186)rps17p (PRK08572)→ rpl14p (PRK08571)Ribosomal_L14 (pfam00238)→<br>rpl24p (PRK01191)KOW_RPL26 (cd06089)→<br>PRK04313 (PRK04313)KOW_RPS4 (cd06087)Ribosomal_S4e (pfam00900)→<br>rpl5p (PRK04219)Ribosomal_L5_C (pfam00673)→ RpsN (COG0199)rps14P (PRK05766)→<br>rps8p (PRK04034)RpsH (COG0096)→ RplF (COG0097)rpl6p (PRK05518)→<br>Rpl32e (COG1717)rpl32e (PRK08562)→
env-419	LGVC01002321.1	+	1612	1708	**RNA**→ Methyltrn_RNA_3 (pfam02598)→ RplC (COG0087)rpl3p (PRK04231)→<br>RplD (COG0088)rpl4lp (PRK04042)→ RplW (COG0089)PRK14548 (PRK14548)→<br>RplB (COG0090)rpl2p (PRK09612)→ RpsS (COG0185)rps19p (PRK04038)→<br>rpl22p (PRK04223)RplV (COG0091)→ RpsC (COG0092)rps3p (PRK04191)→<br>PRK14549 (PRK14549)Ribosomal_L29 (pfam00831)→ PRK03879 (PRK03879)UPF0086 (pfam01868)→<br>RpsQ (COG0186)rps17p (PRK08572)→ rpl14p (PRK08571)Ribosomal_L14 (pfam00238)→<br>rpl24p (PRK01191)KOW_RPL26 (cd06089)→<br>PRK04313 (PRK04313)KOW_RPS4 (cd06087)Ribosomal_S4e (pfam00900)→ RplE (COG0094)rpl5p (PRK04219)→<br>RpsN (COG0199)rps14P (PRK05766)→ rps8p (PRK04034)RpsH (COG0096)→<br>RplF (COG0097)rpl6p (PRK05518)→ rpl32e (PRK08562)Ribosomal_L32_L32e (cd00513)→<br>rpl19e (PRK08570)Ribosomal_L19e (pfam01280)→ RplR (COG0256)rpl18p (PRK08569)→<br>RpsE (COG0098)rps5p (PRK04044)→ Ribosomal_L7_archeal_euk (cd01657)rpl30p (PRK06049)→<br>RplO (COG0200)rpl15p (PRK06419)→ SecY (COG0201)PRK08568 (PRK08568)→<br>adk (PRK00279)ADK (cd01428)→ COG1422 (COG1422)spore_lon_C (TIGR02903)→<br>CmkB (COG1102)PRK04182 (PRK04182)→<br>PRK04270 (PRK04270)PseudoU_synth_hDyskerin (cd02572)PUA (pfam01472)→ hypo→ hypo→ hypo→

env-420	LGVD01004949.1	+	1392	1488	RNA→ Methyltrn_RNA_3 (pfam02598)→ RplC (COG0087)rpl3p (PRK04231)→ rpl4lp (PRK04042)Ribosomal_L4 (pfam00573)→ RplW (COG0089)PRK14548 (PRK14548)→ rpl2p (PRK09612)Ribosomal_L2_C (pfam03947)→ RpsS (COG0185)rps19p (PRK04038)→ rpl22p (PRK04223)RplV (COG0091)→ rps3p (PRK04191)Ribosomal_S3_C (pfam00189)→ PRK14549 (PRK14549)Ribosomal_L29 (pfam00831)→ PRK03879 (PRK03879)UPF0086 (pfam01868)→ RpsQ (COG0186)rps17p (PRK08572)→ rpl14p (PRK08571)Ribosomal_L14 (pfam00238)→ rpl24p (PRK01191)→ PRK04313 (PRK04313)KOW_RPS4 (cd06087)Ribosomal_S4e (pfam00900)→ RplE (COG0094)rpl5p (PRK04219)→ RpsN (COG0199)rps14P (PRK05766)→ rps8p (PRK04034)RpsH (COG0096)→ RplF (COG0097)rpl6p (PRK05518)→ rpl32e (PRK08562)Ribosomal_L32_L32e (cd00513)→ rpl19e (PRK08570)Ribosomal_L19e (pfam01280)→ RplR (COG0256)rpl18p (PRK08569)→ RpsE (COG0098)rps5p (PRK04044)→ RpmD (COG1841)rpl30p (PRK06049)→
env-421	LGVE01002492.1	-	11696	11600	RNA→ Methyltrn_RNA_3 (pfam02598)→ RplC (COG0087)rpl3p (PRK04231)→ RplD (COG0088)rpl4lp (PRK04042)→ RplW (COG0089)PRK14548 (PRK14548)→ RplB (COG0090)rpl2p (PRK09612)→ rpl2p (PRK09612)Ribosomal_L2_C (pfam03947)→ RpsS (COG0185)rps19p (PRK04038)→ rpl22p (PRK04223)RplV (COG0091)→ rps3p (PRK04191)archeal_30S_S3_KH (cd02411)→ RpsC (COG0092)rps3p (PRK04191)→ RpmC (COG0255)PRK14549 (PRK14549)→ PRK03879 (PRK03879)UPF0086 (pfam01868)→ rps17p (PRK08572)→ rpl14p (PRK08571)Ribosomal_L14 (pfam00238)→ rpl24p (PRK01191)KOW_RPL26 (cd06089)→ PRK04313 (PRK04313)rpl5p (PRK04219)KOW_RPS4 (cd06087)Ribosomal_L5_C (pfam00673)Ribosomal_S4e (pfam00900)→ rps8p (PRK04034)RpsH (COG0096)→ RplF (COG0097)rpl6p (PRK05518)→ RplF (COG0097)rpl6p (PRK05518)→ Rpl32e (COG1717)rpl32e (PRK08562)→
env-422	LGVF01002618.1	+	131	227	RNA→ Methyltrn_RNA_3 (pfam02598)→ rpl3p (PRK04231)Ribosomal_L3 (pfam00297)→ RplC (COG0087)rpl3p (PRK04231)→
env-423	LGVE01240744.1	-	496	400	RNA→ Methyltrn_RNA_3 (pfam02598)→
env-424	LGVF01231129.1	-	567	471	RNA→ Methyltrn_RNA_3 (pfam02598)→
env-425	LGOV01087460.1	+	959	1055	RNA→ Methyltrn_RNA_3 (pfam02598)→
env-426	LGVE01063670.1	+	1228	1324	RNA→ Methyltrn_RNA_3 (pfam02598)→ RplC (COG0087)rpl3p (PRK04231)→
env-427	LGVF01061963.1	+	239	335	RNA→ Methyltrn_RNA_3 (pfam02598)→ rpl3p (PRK04231)Ribosomal_L3 (pfam00297)→
env-428	WSSedA1Ba3_1023920	+	778	871	RNA→ Methyltrn_RNA_3 (pfam02598)→
env-429	WSSedA1Ba_1038552	+	407	500	RNA→ MTH1 (COG2106)→
Msh-1-1	NZ_JONQ01000008.1	-	165643	165550	RNA→ Methyltrn_RNA_3 (pfam02598)→ RplC (COG0087)rpl3p (PRK04231)→ RplD (COG0088)rpl4lp (PRK04042)→ RplW (COG0089)PRK14548 (PRK14548)→ RplB (COG0090)rpl2p (PRK09612)→ RpsS (COG0185)rps19p (PRK04038)→ rpl22p (PRK04223)RplV (COG0091)→ RpsC (COG0092)rps3p (PRK04191)→ PRK14549 (PRK14549)Ribosomal_L29_HIP (cd00427)→ PRK03879 (PRK03879)UPF0086 (pfam01868)→ RpsQ (COG0186)rps17p (PRK08572)→ rpl14p (PRK08571)Ribosomal_L14 (pfam00238)→ RplX (COG0198)rpl24p (PRK01191)→ PRK04313 (PRK04313)S4 (cd00165)Ribosomal_S4e (pfam00900)→ RplE (COG0094)rpl5p (PRK04219)→ RpsN (COG0199)rps14P (PRK05766)→ rps8p (PRK04034)RpsH (COG0096)→ RplF (COG0097)rpl6p (PRK05518)→ rpl32e (PRK08562)Ribosomal_L32e (pfam01655)→ rpl19e (PRK08570)Ribosomal_L19e (pfam01280)→ RplR (COG0256)rpl18p (PRK08569)→
env-430	taComm3_C1244	-	4081	3988	RNA→ Methyltrn_RNA_3 (pfam02598)→ RplC (COG0087)rpl3p (PRK04231)→ RplD (COG0088)rpl4lp (PRK04042)→ RplW (COG0089)PRK14548 (PRK14548)→ RplB (COG0090)rpl2p (PRK09612)→ RpsS (COG0185)rps19p (PRK04038)→ rpl22p (PRK04223)RplV (COG0091)→
env-431	JGI12104J13512_1002612	-	3904	3811	RNA→ Methyltrn_RNA_3 (pfam02598)→ RplC (COG0087)rpl3p (PRK04231)→ RplD (COG0088)rpl4lp (PRK04042)→ RplW (COG0089)PRK14548 (PRK14548)→ RplB (COG0090)rpl2p (PRK09612)→ RpsS (COG0185)rps19p (PRK04038)→ rpl22p (PRK04223)Ribosomal_L22 (pfam00237)→

env-432	JGI11944J13513_1000082	-	41802	41709	**RNA**→ Methyltrn_RNA_3 (pfam02598)→ RplC (COG0087)rpl3p (PRK04231)→ RplD (COG0088)rpl4lp (PRK04042)→ RplW (COG0089)PRK14548 (PRK14548)→ RplB (COG0090)rpl2p (PRK09612)→ RpsS (COG0185)rps19p (PRK04038)→ rpl22p (PRK04223)RplV (COG0091)→ RpsC (COG0092)rps3p (PRK04191)→ PRK14549 (PRK14549)Ribosomal_L29 (pfam00831)→ PRK03879 (PRK03879)UPF0086 (pfam01868)→ RpsQ (COG0186)rps17p (PRK08572)→ rpl14p (PRK08571)Ribosomal_L14 (pfam00238)→ rpl24p (PRK01191)KOW_RPL26 (cd06089)→ PRK04313 (PRK04313)KOW_RPS4 (cd06087)S4 (pfam01479)Ribosomal_S4e (pfam00900)→ rpl5p (PRK04219)Ribosomal_L5 (pfam00281)Ribosomal_L5_C (pfam00673)→ RpsN (COG0199)rps14P (PRK05766)→ rps8p (PRK04034)RpsH (COG0096)→ RplF (COG0097)rpl6p (PRK05518)→ rpl32e (PRK08562)Ribosomal_L32e (pfam01655)→ rpl19e (PRK08570)Ribosomal_L19e (pfam01280)→ RplR (COG0256)rpl18p (PRK08569)→
env-433	JGI20179J14886_1000343	-	1356	1263	**RNA**→ Methyltrn_RNA_3 (pfam02598)→ RplC (COG0087)rpl3p (PRK04231)→
env-434	3300001580_10791145	-	337	244	**RNA**→ MTH1 (COG2106)→
env-435	3300000568_10023551	-	5356	5263	**RNA**→ Methyltrn_RNA_3 (pfam02598)→ RplC (COG0087)rpl3p (PRK04231)→ RplD (COG0088)rpl4lp (PRK04042)→ RplW (COG0089)PRK14548 (PRK14548)→ RplB (COG0090)rpl2p (PRK09612)→ RpsS (COG0185)rps19p (PRK04038)→ rpl22p (PRK04223)RplV (COG0091)→ RpsC (COG0092)rps3p (PRK04191)→ PRK14549 (PRK14549)Ribosomal_L29 (pfam00831)→ PRK03879 (PRK03879)UPF0086 (pfam01868)→
env-436	3300001592_10171977	+	123	216	**RNA**→ MTH1 (COG2106)PRK10611 (PRK10611)→ rpl3p (PRK04231)Ribosomal_L3 (pfam00297)→
env-437	3300000032_0536775	-	94	1	**RNA**→
env-438	3300001605_11573450	-	95	2	**RNA**→ MTH1 (COG2106)→
env-439	3300001567_10002499	-	7097	7004	**RNA**→ Methyltrn_RNA_3 (pfam02598)→ RplC (COG0087)rpl3p (PRK04231)→ RplD (COG0088)rpl4lp (PRK04042)→ RplW (COG0089)PRK14548 (PRK14548)→ RplB (COG0090)rpl2p (PRK09612)→ RpsS (COG0185)rps19p (PRK04038)→ rpl22p (PRK04223)RplV (COG0091)→ RpsC (COG0092)rps3p (PRK04191)→ PRK14549 (PRK14549)Ribosomal_L29 (pfam00831)→ PRK03879 (PRK03879)UPF0086 (pfam01868)→ RpsQ (COG0186)rps17p (PRK08572)→ rpl14p (PRK08571)Ribosomal_L14 (pfam00238)→ KOW (smart00739)KOW_RPL26 (cd06089)→ PRK04313 (PRK04313)Ribosomal_S4e (pfam00900)→
env-440	3300000558_10031507	-	264	171	**RNA**→ MTH1 (COG2106)→
env-441	PCEOT_contig04628	+	1388	1481	**RNA**→ Methyltrn_RNA_3 (pfam02598)→ RplC (COG0087)rpl3p (PRK04231)→ RplD (COG0088)rpl4lp (PRK04042)→ RplW (COG0089)PRK14548 (PRK14548)→ RplB (COG0090)rpl2p (PRK09612)→ RpsS (COG0185)rps19p (PRK04038)→ rpl22p (PRK04223)RplV (COG0091)→ RpsC (COG0092)rps3p (PRK04191)→ PRK14549 (PRK14549)Ribosomal_L29 (pfam00831)→ PRK03879 (PRK03879)UPF0086 (pfam01868)→
env-442	JGI12104J13512_1100715	+	77	170	**RNA**→ MTH1 (COG2106)→
Mco-2-1	NC_015416.1	-	2306426	2306333	**RNA**→ Methyltrn_RNA_3 (pfam02598)→ RplC (COG0087)rpl3p (PRK04231)→ RplD (COG0088)rpl4lp (PRK04042)→ RplW (COG0089)PRK14548 (PRK14548)→ RplB (COG0090)rpl2p (PRK09612)→ RpsS (COG0185)rps19p (PRK04038)→ rpl22p (PRK04223)RplV (COG0091)→ RpsC (COG0092)rps3p (PRK04191)→ PRK14549 (PRK14549)Ribosomal_L29 (pfam00831)→ PRK03879 (PRK03879)UPF0086 (pfam01868)→ RpsQ (COG0186)rps17p (PRK08572)→ rpl14p (PRK08571)Ribosomal_L14 (pfam00238)→ rpl24p (PRK01191)KOW_RPL26 (cd06089)→ rpl24p (PRK01191)KOW_RPL26 (cd06089)→ PRK04313 (PRK04313)KOW_RPS4 (cd06087)Ribosomal_S4e (pfam00900)→ RplE (COG0094)rpl5p (PRK04219)→ RpsN (COG0199)rps14P (PRK05766)→ rps8p (PRK04034)RpsH (COG0096)→ RplF (COG0097)rpl6p (PRK05518)→ rpl32e (PRK08562)Ribosomal_L32e (pfam01655)→ rpl19e (PRK08570)Ribosomal_L19e (pfam01280)→ RplR (COG0256)rpl18p (PRK08569)→
env-443	JGI24730J26740_1002326	-	3281	3188	**RNA**→ Methyltrn_RNA_3 (pfam02598)→ RplC (COG0087)rpl3p (PRK04231)→ RplD (COG0088)rpl4lp (PRK04042)→ RplW (COG0089)PRK14548 (PRK14548)→ RplB (COG0090)rpl2p (PRK09612)→

env-444	JGI24732J26686_1000856	+	2537	2630	**RNA**→ Methyltrn_RNA_3 (pfam02598)→ rpl3p (PRK04231)Ribosomal_L3 (pfam00297)→ rpl3p (PRK04231)Ribosomal_L3 (pfam00297)→ RplD (COG0088)rpl4lp (PRK04042)→ RplW (COG0089)PRK14548 (PRK14548)→ RplB (COG0090)rpl2p (PRK09612)→ RpsS (COG0185)rps19p (PRK04038)→ rpl22p (PRK04223)RplV (COG0091)→ RpsC (COG0092)rps3p (PRK04191)→ RpmC (COG0255)PRK14549 (PRK14549)→ POP4 (COG1588)PRK03879 (PRK03879)→ RpsQ (COG0186)rps17p (PRK08572)→ rpl14p (PRK08571)Ribosomal_L14 (pfam00238)→ rpl24p (PRK01191)KOW_RPL26 (cd06089)→ PRK04313 (PRK04313)KOW_RPS4 (cd06087)S4 (pfam01479)Ribosomal_S4e (pfam00900)→ rpl5p (PRK04219)Ribosomal_L5 (pfam00281)Ribosomal_L5_C (pfam00673)→ RpsN (COG0199)rps14P (PRK05766)→ rps8p (PRK04034)RpsH (COG0096)→ RplF (COG0097)rpl6p (PRK05518)→ rpl32e (PRK08562)Ribosomal_L32_L32e (cd00513)→ rpl19e (PRK08570)Ribosomal_L19e (pfam01280)→ RplR (COG0256)rpl18p (PRK08569)→ RpsE (COG0098)rps5p (PRK04044)→ Ribosomal_L7_archeal_euk (cd01657)rpl30p (PRK06049)→ rpl15p (PRK06419)→
env-445	JGI24730J26740_1077952	-	136	43	**RNA**→ MTH1 (COG2106)→
env-446	JGI24729J20445_1000277	+	15893	15986	**RNA**→ Methyltrn_RNA_3 (pfam02598)→ RplC (COG0087)rpl3p (PRK04231)→ RplD (COG0088)rpl4lp (PRK04042)→ RplW (COG0089)PRK14548 (PRK14548)→ RplB (COG0090)rpl2p (PRK09612)→ RpsS (COG0185)rps19p (PRK04038)→ rpl22p (PRK04223)RplV (COG0091)→ RpsC (COG0092)rps3p (PRK04191)→ PRK14549 (PRK14549)Ribosomal_L29 (pfam00831)→ PRK03879 (PRK03879)UPF0086 (pfam01868)→ RpsQ (COG0186)rps17p (PRK08572)→ rpl14p (PRK08571)Ribosomal_L14 (pfam00238)→ rpl24p (PRK01191)KOW_RPL26 (cd06089)→ PRK04313 (PRK04313)S4 (cd00165)KOW_RPS4 (cd06087)→ RplE (COG0094)rpl5p (PRK04219)→ RpsN (COG0199)rps14P (PRK05766)→ rps8p (PRK04034)RpsH (COG0096)→ RplF (COG0097)rpl6p (PRK05518)→ rpl32e (PRK08562)Ribosomal_L32e (pfam01655)→ rpl19e (PRK08570)Ribosomal_L19e (pfam01280)→ RplR (COG0256)rpl18p (PRK08569)→
env-447	JGI24732J26686_1234070	+	153	246	**RNA**→ MTH1 (COG2106)→
env-448	SakLake120mDRAFT_c0020500	-	1276	1183	**RNA**→ Methyltrn_RNA_3 (pfam02598)→
env-449	JGI24730J26740_1097811	-	201	108	**RNA**→ Methyltrn_RNA_3 (pfam02598)→
env-450	JGI20198J14950_1052355	+	64	157	**RNA**→ MTH1 (COG2106)→
env-451	JGI24729J20445_1001989	-	4749	4656	**RNA**→ Methyltrn_RNA_3 (pfam02598)→ RplC (COG0087)rpl3p (PRK04231)→ RplD (COG0088)rpl4lp (PRK04042)→ RplW (COG0089)PRK14548 (PRK14548)→ RplB (COG0090)rpl2p (PRK09612)→ RpsS (COG0185)rps19p (PRK04038)→ rpl22p (PRK04223)RplV (COG0091)→ RpsC (COG0092)rps3p (PRK04191)→
env-452	JGI20214J14112_1145042	+	137	230	**RNA**→ MTH1 (COG2106)→
env-453	JGI24732J26686_1001287	-	3712	3619	**RNA**→ Methyltrn_RNA_3 (pfam02598)→ RplC (COG0087)rpl3p (PRK04231)→ RplD (COG0088)rpl4lp (PRK04042)→ RplW (COG0089)PRK14548 (PRK14548)→ rpl2p (PRK09612)Ribosomal_L2 (pfam00181)→ rpl2p (PRK09612)Ribosomal_L2_C (pfam03947)→ RpsS (COG0185)rps19p (PRK04038)→
env-454	JGI24730J26740_1003612	-	4510	4417	**RNA**→ Methyltrn_RNA_3 (pfam02598)→ RplC (COG0087)rpl3p (PRK04231)→ RplD (COG0088)rpl4lp (PRK04042)→ RplW (COG0089)PRK14548 (PRK14548)→ RplB (COG0090)rpl2p (PRK09612)→ RpsS (COG0185)rps19p (PRK04038)→ rpl22p (PRK04223)RplV (COG0091)→ KH (smart00322)archeal_30S_S3_KH (cd02411)→
env-455	JGI24732J26686_1002014	+	4931	5024	**RNA**→ Methyltrn_RNA_3 (pfam02598)→ RplC (COG0087)rpl3p (PRK04231)→ RplD (COG0088)rpl4lp (PRK04042)→ RplW (COG0089)PRK14548 (PRK14548)→
env-456	taComm3_C551	-	847	754	**RNA**→ Methyltrn_RNA_3 (pfam02598)→ rpl3p (PRK04231)Ribosomal_L3 (pfam00297)→

env-457	JGI12104J13512_1000496	-	35195	35102	**RNA**→ MTH1 (COG2106)→ RplC (COG0087)rpl3p (PRK04231)→ RplD (COG0088)rpl4lp (PRK04042)→<br>RplW (COG0089)PRK14548 (PRK14548)→ RplB (COG0090)rpl2p (PRK09612)→<br>RpsS (COG0185)rps19p (PRK04038)→ rpl22p (PRK04223)RplV (COG0091)→<br>RpsC (COG0092)rps3p (PRK04191)→ PRK14549 (PRK14549)Ribosomal_L29 (pfam00831)→<br>PRK03879 (PRK03879)UPF0086 (pfam01868)→ RpsQ (COG0186)rps17p (PRK08572)→<br>rpl14p (PRK08571)Ribosomal_L14 (pfam00238)→ rpl24p (PRK01191)KOW_RPL26 (cd06089)→<br>PRK04313 (PRK04313)KOW_RPS4 (cd06087)S4 (pfam01479)→ RplE (COG0094)rpl5p (PRK04219)→<br>RpsN (COG0199)rps14P (PRK05766)→ rps8p (PRK04034)RpsH (COG0096)→<br>RplF (COG0097)rpl6p (PRK05518)→ rpl32e (PRK08562)Ribosomal_L32e (pfam01655)→<br>rpl19e (PRK08570)Ribosomal_L19e (pfam01280)→ RplR (COG0256)rpl18p (PRK08569)→<br>RpsE (COG0098)rps5p (PRK04044)→
env-458	JGI12104J13512_1006964	-	3599	3506	**RNA**→ Methyltrn_RNA_3 (pfam02598)→ RplC (COG0087)rpl3p (PRK04231)→<br>RplD (COG0088)rpl4lp (PRK04042)→ RplW (COG0089)PRK14548 (PRK14548)→<br>RplB (COG0090)rpl2p (PRK09612)→ rps19p (PRK04038)Ribosomal_S19 (pfam00203)→
env-459	JGI11944J13513_1000282	+	18417	18510	**RNA**→ Methyltrn_RNA_3 (pfam02598)→ RplC (COG0087)rpl3p (PRK04231)→<br>RplD (COG0088)rpl4lp (PRK04042)→ RplW (COG0089)PRK14548 (PRK14548)→<br>RplB (COG0090)rpl2p (PRK09612)→ RpsS (COG0185)rps19p (PRK04038)→<br>rpl22p (PRK04223)RplV (COG0091)→ RpsC (COG0092)rps3p (PRK04191)→<br>PRK14549 (PRK14549)Ribosomal_L29 (pfam00831)→ PRK03879 (PRK03879)UPF0086 (pfam01868)→<br>RpsQ (COG0186)rps17p (PRK08572)→ rpl14p (PRK08571)Ribosomal_L14 (pfam00238)→<br>rpl24p (PRK01191)KOW_RPL26 (cd06089)→ PRK04313 (PRK04313)KOW_RPS4 (cd06087)S4 (pfam01479)→<br>RplE (COG0094)rpl5p (PRK04219)→ RpsN (COG0199)rps14P (PRK05766)→<br>rps8p (PRK04034)RpsH (COG0096)→ RplF (COG0097)rpl6p (PRK05518)→<br>rpl32e (PRK08562)Ribosomal_L32e (pfam01655)→ rpl19e (PRK08570)Ribosomal_L19e (pfam01280)→<br>RplR (COG0256)rpl18p (PRK08569)→ RpsE (COG0098)rps5p (PRK04044)→
env-460	WSSedA2C_c029104	+	89	182	**RNA**→ MTH1 (COG2106)→ rpl3p (PRK04231)Ribosomal_L3 (pfam00297)→
env-461	WSSedA2Ca3_1029104	+	89	182	**RNA**→ MTH1 (COG2106)→ rpl3p (PRK04231)Ribosomal_L3 (pfam00297)→
env-462	WSSedA1TJGI_c020031	-	764	671	**RNA**→ MTH1 (COG2106)→ rpl3p (PRK04231)Ribosomal_L3 (pfam00297)→
env-463	WSSedA1Ba3_1007453	+	836	929	**RNA**→ Methyltrn_RNA_3 (pfam02598)→ RplC (COG0087)rpl3p (PRK04231)→<br>rpl4lp (PRK04042)Ribosomal_L4 (pfam00573)→
env-464	JGI20213J14113_1028243	-	729	636	**RNA**→ MTH1 (COG2106)→ rpl3p (PRK04231)Ribosomal_L3 (pfam00297)→
env-465	JGI20214J14112_1082845	-	335	242	**RNA**→ MTH1 (COG2106)→
env-466	JGI20214J14112_1065024	-	197	104	**RNA**→ MTH1 (COG2106)→
env-467	3300000558_11706232	-	175	82	**RNA**→ Methyltrn_RNA_3 (pfam02598)→
env-468	3300001605_10001574	+	12383	12476	**RNA**→ Methyltrn_RNA_3 (pfam02598)→ rpl3p (PRK04231)Ribosomal_L3 (pfam00297)→<br>rpl3p (PRK04231)Ribosomal_L3 (pfam00297)→ RplD (COG0088)rpl4lp (PRK04042)→<br>RplW (COG0089)PRK14548 (PRK14548)→ RplB (COG0090)rpl2p (PRK09612)→<br>RpsS (COG0185)rps19p (PRK04038)→ rpl22p (PRK04223)RplV (COG0091)→<br>RpsC (COG0092)rps3p (PRK04191)→ PRK14549 (PRK14549)Ribosomal_L29 (pfam00831)→<br>PRK03879 (PRK03879)UPF0086 (pfam01868)→ RpsQ (COG0186)rps17p (PRK08572)→<br>rpl14p (PRK08571)Ribosomal_L14 (pfam00238)→ rpl24p (PRK01191)KOW_RPL26 (cd06089)→<br>PRK04313 (PRK04313)KOW_RPS4 (cd06087)S4 (pfam01479)Ribosomal_S4e (pfam00900)→<br>rpl5p (PRK04219)Ribosomal_L5 (pfam00281)Ribosomal_L5_C (pfam00673)→<br>RpsN (COG0199)rps14P (PRK05766)→ rps8p (PRK04034)RpsH (COG0096)→<br>RplF (COG0097)rpl6p (PRK05518)→ rpl32e (PRK08562)Ribosomal_L32e (pfam01655)→<br>rpl19e (PRK08570)Ribosomal_L19e (pfam01280)→ RplR (COG0256)rpl18p (PRK08569)→
env-469	2232004817	+	121	214	**RNA**→ MTH1 (COG2106)→
env-470	3300000558_10046922	-	175	82	**RNA**→ Methyltrn_RNA_3 (pfam02598)→
env-471	3300000032_0025728	-	2226	2133	**RNA**→ Methyltrn_RNA_3 (pfam02598)→ RplC (COG0087)rpl3p (PRK04231)→<br>RplD (COG0088)rpl4lp (PRK04042)→
env-472	3300000568_10127450	-	4713	4620	**RNA**→ Methyltrn_RNA_3 (pfam02598)→ RplC (COG0087)rpl3p (PRK04231)→<br>RplD (COG0088)rpl4lp (PRK04042)→ RplW (COG0089)PRK14548 (PRK14548)→<br>RplB (COG0090)rpl2p (PRK09612)→ RpsS (COG0185)rps19p (PRK04038)→<br>rpl22p (PRK04223)RplV (COG0091)→ RpsC (COG0092)rps3p (PRK04191)→

env-473	3300001580_10000904	+	13878	13971	**RNA**→ Methyltrn_RNA_3 (pfam02598)→ RplC (COG0087)rpl3p (PRK04231)→<br>RplD (COG0088)rpl4lp (PRK04042)→ RplW (COG0089)PRK14548 (PRK14548)→<br>RplB (COG0090)rpl2p (PRK09612)→ RpsS (COG0185)rps19p (PRK04038)→<br>rpl22p (PRK04223)RplV (COG0091)→ RpsC (COG0092)rps3p (PRK04191)→<br>PRK14549 (PRK14549)Ribosomal_L29 (pfam00831)→ PRK03879 (PRK03879)UPF0086 (pfam01868)→<br>RpsQ (COG0186)rps17p (PRK08572)→ rpl14p (PRK08571)Ribosomal_L14 (pfam00238)→<br>rpl24p (PRK01191)KOW_RPL26 (cd06089)→<br>PRK04313 (PRK04313)KOW_RPS4 (cd06087)S4 (pfam01479)Ribosomal_S4e (pfam00900)→<br>rpl5p (PRK04219)Ribosomal_L5 (pfam00281)Ribosomal_L5_C (pfam00673)→<br>RpsN (COG0199)rps14P (PRK05766)→ rps8p (PRK04034)RpsH (COG0096)→<br>RplF (COG0097)rpl6p (PRK05518)→ rpl32e (PRK08562)Ribosomal_L32e (pfam01655)→<br>rpl19e (PRK08570)Ribosomal_L19e (pfam01280)→ RplR (COG0256)rpl18p (PRK08569)→
env-474	3300001592_10239960	+	303	396	**RNA**→ MTH1 (COG2106)→
env-475	3300001580_10151383	-	980	887	**RNA**→ MTH1 (COG2106)→
Mha-1-1	NC_017527.1	-	484979	484886	**RNA**→ Methyltrn_RNA_3 (pfam02598)→
env-476	JGI12104J13512_1010054	+	871	964	**RNA**→ Methyltrn_RNA_3 (pfam02598)→ RplC (COG0087)rpl3p (PRK04231)→<br>RplD (COG0088)rpl4lp (PRK04042)→
env-477	SakLake120mDRAFT_c0004292	+	2569	2662	**RNA**→ Methyltrn_RNA_3 (pfam02598)→ RplC (COG0087)rpl3p (PRK04231)→<br>RplD (COG0088)rpl4lp (PRK04042)→ PRK14548 (PRK14548)→ RplB (COG0090)rpl2p (PRK09612)→<br>RpsS (COG0185)rps19p (PRK04038)→ rpl22p (PRK04223)RplV (COG0091)→<br>RpsC (COG0092)rps3p (PRK04191)→
env-478	JXWW01104647.1	-	233	140	**RNA**→ Methyltrn_RNA_3 (pfam02598)→
env-479	JGI24025J20009_10001050	-	20258	20165	**RNA**→ Methyltrn_RNA_3 (pfam02598)→ RplC (COG0087)rpl3p (PRK04231)→<br>RplD (COG0088)rpl4lp (PRK04042)→ RplW (COG0089)PRK14548 (PRK14548)→<br>RplB (COG0090)rpl2p (PRK09612)→ RpsS (COG0185)rps19p (PRK04038)→<br>rpl22p (PRK04223)RplV (COG0091)→ RpsC (COG0092)rps3p (PRK04191)rne (PRK10811)→<br>PRK14549 (PRK14549)Ribosomal_L29 (pfam00831)→ PRK03879 (PRK03879)UPF0086 (pfam01868)→<br>RpsQ (COG0186)rps17p (PRK08572)RING-CH-C4HC3_FANCL (cd16490)→<br>rpl14p (PRK08571)Ribosomal_L14 (pfam00238)→ RplX (COG0198)rpl24p (PRK01191)→<br>PRK04313 (PRK04313)KOW_RPS4 (cd06087)S4 (pfam01479)Ribosomal_S4e (pfam00900)→<br>RplE (COG0094)rpl5p (PRK04219)→
env-480	ASA129_contig07056	-	867	774	**RNA**→ MTH1 (COG2106)→
env-481	JGI24020J35080_1000033	+	3315	3408	**RNA**→ MTH1 (COG2106)→ RplC (COG0087)rpl3p (PRK04231)→ RplD (COG0088)rpl4lp (PRK04042)→<br>RplW (COG0089)PRK14548 (PRK14548)→ RplB (COG0090)rpl2p (PRK09612)→<br>RpsS (COG0185)rps19p (PRK04038)→ rpl22p (PRK04223)RplV (COG0091)→<br>RpsC (COG0092)rps3p (PRK04191)→ PRK14549 (PRK14549)Ribosomal_L29 (pfam00831)→<br>POP4 (COG1588)PRK03879 (PRK03879)→ RpsQ (COG0186)rps17p (PRK08572)→<br>rpl14p (PRK08571)Ribosomal_L14 (pfam00238)→ rpl24p (PRK01191)KOW_RPL26 (cd06089)→<br>PRK04313 (PRK04313)KOW_RPS4 (cd06087)S4 (pfam01479)Ribosomal_S4e (pfam00900)→<br>RplE (COG0094)rpl5p (PRK04219)→ hypo→ rps8p (PRK04034)RpsH (COG0096)→<br>RplF (COG0097)rpl6p (PRK05518)→ rpl32e (PRK08562)Ribosomal_L32e (pfam01655)→<br>rpl19e (PRK08570)Ribosomal_L19e (pfam01280)→ RplR (COG0256)rpl18p (PRK08569)→<br>RpsE (COG0098)rps5p (PRK04044)→ Ribosomal_L7_archeal_euk (cd01657)rpl30p (PRK06049)→<br>rpl15p (PRK06419)Ribosomal_L27A (pfam00828)→ SecY (COG0201)PRK08568 (PRK08568)→<br>adk (PRK00279)ADK (cd01428)→ COG1422 (COG1422)PRK02201 (PRK02201)→<br>CmkB (COG1102)PRK04182 (PRK04182)→<br>PRK04270 (PRK04270)PseudoU_synth_hDyskerin (cd02572)PUA (pfam01472)→ hypo→ hypo→<br>AAA (smart00382)CooC (cd02034)GTPase_EngA (TIGR03594)→

env-482	JGI24020J35080_1000258	+	14429	14513	**RNA**→ MTH1 (COG2106)Raftlin (pfam15250)→ RplC (COG0087)rpl3p (PRK04231)→ RplD (COG0088)rpl4lp (PRK04042)→ RplW (COG0089)PRK14548 (PRK14548)→ RplB (COG0090)rpl2p (PRK09612)→ RpsS (COG0185)rps19p (PRK04038)→ rpl22p (PRK04223)RplV (COG0091)→ RpsC (COG0092)rps3p (PRK04191)→ PRK14549 (PRK14549)Ribosomal_L29 (pfam00831)→ POP4 (COG1588)PRK03879 (PRK03879)→ RpsQ (COG0186)rps17p (PRK08572)→ rpl14p (PRK08571)Ribosomal_L14 (pfam00238)→ rpl24p (PRK01191)KOW_RPL26 (cd06089)→ PRK04313 (PRK04313)KOW_RPS4 (cd06087)S4 (pfam01479)Ribosomal_S4e (pfam00900)→ rpl5p (PRK04219)Ribosomal_L5 (pfam00281)Ribosomal_L5_C (pfam00673)→ RpsN (COG0199)rps14P (PRK05766)→ rps8p (PRK04034)RpsH (COG0096)→ RplF (COG0097)rpl6p (PRK05518)→ Rpl32e (COG1717)rpl32e (PRK08562)HHH (pfam00633)HHH_5 (pfam14520)→ rpl19e (PRK08570)Ribosomal_L19e (pfam01280)→ RplR (COG0256)rpl18p (PRK08569)→
env-483	JGI20177J14857_1074829	-	299	212	**RNA**→ MTH1 (COG2106)→
env-484	JGI20193J14888_1132815	-	101	14	**RNA**→ Methyltrn_RNA_3 (pfam02598)→
env-485	JGI20191J14862_1083948	+	194	281	**RNA**→ MTH1 (COG2106)→
env-486	JGI20195J14853_1011539	-	972	885	**RNA**→ MTH1 (COG2106)→ rpl3p (PRK04231)Ribosomal_L3 (pfam00297)→
env-487	JGI20179J14886_1000011	-	26349	26262	**RNA**→ MTH1 (COG2106)→ RplC (COG0087)rpl3p (PRK04231)→ RplD (COG0088)rpl4lp (PRK04042)→ RplW (COG0089)PRK14548 (PRK14548)→ RplB (COG0090)rpl2p (PRK09612)→ RpsS (COG0185)rps19p (PRK04038)→ rpl22p (PRK04223)RplV (COG0091)→ RpsC (COG0092)rps3p (PRK04191)→ RpmC (COG0255)PRK14549 (PRK14549)→ POP4 (COG1588)PRK03879 (PRK03879)→ RpsQ (COG0186)rps17p (PRK08572)→ rpl14p (PRK08571)Ribosomal_L14 (pfam00238)→ rpl24p (PRK01191)KOW_RPL26 (cd06089)→ PRK04313 (PRK04313)KOW_RPS4 (cd06087)S4 (pfam01479)Ribosomal_S4e (pfam00900)→ rpl5p (PRK04219)Ribosomal_L5 (pfam00281)Ribosomal_L5_C (pfam00673)→
env-488	JGI20199J14953_1000018	+	6725	6812	**RNA**→ Methyltrn_RNA_3 (pfam02598)→ RplC (COG0087)rpl3p (PRK04231)→ RplD (COG0088)rpl4lp (PRK04042)→ RplW (COG0089)PRK14548 (PRK14548)→ RplB (COG0090)rpl2p (PRK09612)→ RpsS (COG0185)rps19p (PRK04038)→ rpl22p (PRK04223)RplV (COG0091)→ RpsC (COG0092)rps3p (PRK04191)→ RpmC (COG0255)PRK14549 (PRK14549)→ POP4 (COG1588)PRK03879 (PRK03879)→ RpsQ (COG0186)rps17p (PRK08572)→
env-489	JGI20201J14949_1002905	-	1578	1491	**RNA**→ MTH1 (COG2106)→ RplC (COG0087)rpl3p (PRK04231)→ rpl4lp (PRK04042)→
env-490	JGI20197J15001_1000199	-	8178	8091	**RNA**→ MTH1 (COG2106)→ RplC (COG0087)rpl3p (PRK04231)→ RplD (COG0088)rpl4lp (PRK04042)→ RplW (COG0089)PRK14548 (PRK14548)→ RplB (COG0090)rpl2p (PRK09612)→ RpsS (COG0185)rps19p (PRK04038)→ rpl22p (PRK04223)RplV (COG0091)→ RpsC (COG0092)rps3p (PRK04191)→ RpmC (COG0255)PRK14549 (PRK14549)→ POP4 (COG1588)PRK03879 (PRK03879)→ RpsQ (COG0186)rps17p (PRK08572)→ rpl14p (PRK08571)Ribosomal_L14 (pfam00238)→ rpl24p (PRK01191)KOW_RPL26 (cd06089)→ PRK04313 (PRK04313)KOW_RPS4 (cd06087)S4 (pfam01479)Ribosomal_S4e (pfam00900)→ rpl5p (PRK04219)Ribosomal_L5 (pfam00281)Ribosomal_L5_C (pfam00673)→
env-491	JGI20197J15136_1000199	-	8178	8091	**RNA**→ MTH1 (COG2106)→ RplC (COG0087)rpl3p (PRK04231)→ RplD (COG0088)rpl4lp (PRK04042)→ RplW (COG0089)PRK14548 (PRK14548)→ RplB (COG0090)rpl2p (PRK09612)→ RpsS (COG0185)rps19p (PRK04038)→ rpl22p (PRK04223)RplV (COG0091)→ RpsC (COG0092)rps3p (PRK04191)→ RpmC (COG0255)PRK14549 (PRK14549)→ POP4 (COG1588)PRK03879 (PRK03879)→ RpsQ (COG0186)rps17p (PRK08572)→ rpl14p (PRK08571)Ribosomal_L14 (pfam00238)→ rpl24p (PRK01191)KOW_RPL26 (cd06089)→ PRK04313 (PRK04313)KOW_RPS4 (cd06087)S4 (pfam01479)Ribosomal_S4e (pfam00900)→ rpl5p (PRK04219)Ribosomal_L5 (pfam00281)Ribosomal_L5_C (pfam00673)→
env-492	AGCH01000237.1	-	413	326	**RNA**→ MTH1 (COG2106)→
env-493	PermFrostAlaska_NODE_9622	-	413	326	**RNA**→ MTH1 (COG2106)→

env-494	B3_all_c_Cons150562	-	21306	21219	**RNA**→ MTH1 (COG2106)→ rpl3p (PRK04231)Ribosomal_L3 (pfam00297)→ RplC (COG0087)rpl3p (PRK04231)→ RplD (COG0088)rpl4lp (PRK04042)→ RplW (COG0089)PRK14548 (PRK14548)→ RplB (COG0090)rpl2p (PRK09612)→ RpsS (COG0185)rps19p (PRK04038)→ rpl22p (PRK04223)RplV (COG0091)→ RpsC (COG0092)rps3p (PRK04191)→ RpmC (COG0255)PRK14549 (PRK14549)→ POP4 (COG1588)PRK03879 (PRK03879)→ RpsQ (COG0186)rps17p (PRK08572)→ rpl14p (PRK08571)Ribosomal_L14 (pfam00238)→ rpl24p (PRK01191)KOW_RPL26 (cd06089)→ PRK04313 (PRK04313)KOW_RPS4 (cd06087)S4 (pfam01479)Ribosomal_S4e (pfam00900)→ rpl5p (PRK04219)Ribosomal_L5 (pfam00281)Ribosomal_L5_C (pfam00673)→
env-495	JGI20199J14953_1000162	+	190	277	**RNA**→ MTH1 (COG2106)→ RplC (COG0087)rpl3p (PRK04231)→ RplD (COG0088)rpl4lp (PRK04042)→ RplW (COG0089)PRK14548 (PRK14548)→ RplB (COG0090)rpl2p (PRK09612)→ RpsS (COG0185)rps19p (PRK04038)→ rpl22p (PRK04223)RplV (COG0091)→ RpsC (COG0092)rps3p (PRK04191)→ RpmC (COG0255)PRK14549 (PRK14549)→ POP4 (COG1588)PRK03879 (PRK03879)→ RpsQ (COG0186)rps17p (PRK08572)→ rpl14p (PRK08571)Ribosomal_L14 (pfam00238)→ rpl24p (PRK01191)→
env-496	JGI20198J14950_1000001	+	50351	50438	**RNA**→ Methyltrn_RNA_3 (pfam02598)→ RplC (COG0087)rpl3p (PRK04231)→ RplD (COG0088)rpl4lp (PRK04042)→ RplW (COG0089)PRK14548 (PRK14548)→ RplB (COG0090)rpl2p (PRK09612)→ RpsS (COG0185)rps19p (PRK04038)→ rpl22p (PRK04223)RplV (COG0091)→ RpsC (COG0092)rps3p (PRK04191)→ RpmC (COG0255)PRK14549 (PRK14549)→ POP4 (COG1588)PRK03879 (PRK03879)→ RpsQ (COG0186)rps17p (PRK08572)→ rpl14p (PRK08571)Ribosomal_L14 (pfam00238)→ RplX (COG0198)rpl24p (PRK01191)→ PRK04313 (PRK04313)KOW_RPS4 (cd06087)Ribosomal_S4e (pfam00900)→ rpl5p (PRK04219)Ribosomal_L5 (pfam00281)Ribosomal_L5_C (pfam00673)→
env-497	JGI20200J14955_1011738	+	613	700	**RNA**→ MTH1 (COG2106)→
env-498	JGI20201J14949_1007869	+	187	274	**RNA**→ MTH1 (COG2106)→ rpl3p (PRK04231)Ribosomal_L3 (pfam00297)→
env-499	JGI20197J15001_1000002	-	33762	33675	**RNA**→ MTH1 (COG2106)→ RplC (COG0087)rpl3p (PRK04231)→ RplD (COG0088)rpl4lp (PRK04042)→ RplW (COG0089)PRK14548 (PRK14548)→ RplB (COG0090)rpl2p (PRK09612)→ RpsS (COG0185)rps19p (PRK04038)→ rpl22p (PRK04223)RplV (COG0091)→ RpsC (COG0092)rps3p (PRK04191)→ RpmC (COG0255)PRK14549 (PRK14549)→ POP4 (COG1588)PRK03879 (PRK03879)→ RpsQ (COG0186)rps17p (PRK08572)→ rpl14p (PRK08571)Ribosomal_L14 (pfam00238)→ RplX (COG0198)rpl24p (PRK01191)→ PRK04313 (PRK04313)KOW_RPS4 (cd06087)Ribosomal_S4e (pfam00900)→ rpl5p (PRK04219)Ribosomal_L5 (pfam00281)Ribosomal_L5_C (pfam00673)→
env-500	JGI20197J15136_1000002	-	33762	33675	**RNA**→ MTH1 (COG2106)→ RplC (COG0087)rpl3p (PRK04231)→ RplD (COG0088)rpl4lp (PRK04042)→ RplW (COG0089)PRK14548 (PRK14548)→ RplB (COG0090)rpl2p (PRK09612)→ RpsS (COG0185)rps19p (PRK04038)→ rpl22p (PRK04223)RplV (COG0091)→ RpsC (COG0092)rps3p (PRK04191)→ RpmC (COG0255)PRK14549 (PRK14549)→ POP4 (COG1588)PRK03879 (PRK03879)→ RpsQ (COG0186)rps17p (PRK08572)→ rpl14p (PRK08571)Ribosomal_L14 (pfam00238)→ RplX (COG0198)rpl24p (PRK01191)→ PRK04313 (PRK04313)KOW_RPS4 (cd06087)Ribosomal_S4e (pfam00900)→ rpl5p (PRK04219)Ribosomal_L5 (pfam00281)Ribosomal_L5_C (pfam00673)→
env-501	AGCH01000005.1	-	11078	10991	**RNA**→ Methyltrn_RNA_3 (pfam02598)→ rpl3p (PRK04231)Ribosomal_L3 (pfam00297)→ RplC (COG0087)rpl3p (PRK04231)→ RplD (COG0088)rpl4lp (PRK04042)→ RplW (COG0089)PRK14548 (PRK14548)→ RplB (COG0090)rpl2p (PRK09612)→ RpsS (COG0185)rps19p (PRK04038)→ rpl22p (PRK04223)RplV (COG0091)→ RpsC (COG0092)rps3p (PRK04191)→ RpmC (COG0255)PRK14549 (PRK14549)→ POP4 (COG1588)PRK03879 (PRK03879)→ RpsQ (COG0186)rps17p (PRK08572)→ rpl14p (PRK08571)Ribosomal_L14 (pfam00238)→ rpl24p (PRK01191)KOW_RPL26 (cd06089)→ PRK04313 (PRK04313)KOW_RPS4 (cd06087)S4 (pfam01479)Ribosomal_S4e (pfam00900)→

env-502	PermFrostAlaska_NODE_6124	-	23466	23379	**RNA**→ Methyltrn_RNA_3 (pfam02598)→ rpl3p (PRK04231)Ribosomal_L3 (pfam00297)→ RplC (COG0087)rpl3p (PRK04231)→ RplD (COG0088)rpl4lp (PRK04042)→ RplW (COG0089)PRK14548 (PRK14548)→ RplB (COG0090)rpl2p (PRK09612)→ RpsS (COG0185)rps19p (PRK04038)→ rpl22p (PRK04223)RplV (COG0091)→ RpsC (COG0092)rps3p (PRK04191)→ RpmC (COG0255)PRK14549 (PRK14549)→ POP4 (COG1588)PRK03879 (PRK03879)→ RpsQ (COG0186)rps17p (PRK08572)→ rpl14p (PRK08571)Ribosomal_L14 (pfam00238)→ rpl24p (PRK01191)KOW_RPL26 (cd06089)→ PRK04313 (PRK04313)KOW_RPS4 (cd06087)S4 (pfam01479)Ribosomal_S4e (pfam00900)→
env-503	B3_all_c_Cons68376	-	6993	6906	**RNA**→ Methyltrn_RNA_3 (pfam02598)→ RplC (COG0087)rpl3p (PRK04231)→ RplD (COG0088)rpl4lp (PRK04042)→ RplW (COG0089)PRK14548 (PRK14548)→ RplB (COG0090)rpl2p (PRK09612)→ RpsS (COG0185)rps19p (PRK04038)→ rpl22p (PRK04223)RplV (COG0091)→ RpsC (COG0092)rps3p (PRK04191)→ RpmC (COG0255)PRK14549 (PRK14549)→ POP4 (COG1588)PRK03879 (PRK03879)→
env-504	JGI20179J14886_1002439	-	264	177	**RNA**→ Methyltrn_RNA_3 (pfam02598)→
env-505	JGI24728J21555_1000539	+	10047	10116	**RNA**→ RplC (COG0087)rpl3p (PRK04231)→ RplD (COG0088)rpl4lp (PRK04042)→ RplW (COG0089)PRK14548 (PRK14548)→ RplB (COG0090)rpl2p (PRK09612)→ RpsS (COG0185)rps19p (PRK04038)→ rpl22p (PRK04223)RplV (COG0091)→ RpsC (COG0092)rps3p (PRK04191)→ PRK14549 (PRK14549)Ribosomal_L29 (pfam00831)→ PRK03879 (PRK03879)UPF0086 (pfam01868)→ RpsQ (COG0186)rps17p (PRK08572)→ rpl14p (PRK08571)Ribosomal_L14 (pfam00238)→ rpl24p (PRK01191)KOW_RPL26 (cd06089)→ PRK04313 (PRK04313)KOW_RPS4 (cd06087)Ribosomal_S4e (pfam00900)→ rpl5p (PRK04219)Ribosomal_L5 (pfam00281)Ribosomal_L5_C (pfam00673)→ RpsN (COG0199)rps14P (PRK05766)→ rps8p (PRK04034)RpsH (COG0096)→ RplF (COG0097)rpl6p (PRK05518)→ rpl32e (PRK08562)Ribosomal_L32e (pfam01655)→
env-506	JGI24728J21555_1000541	-	9030	8961	**RNA**→ RplC (COG0087)rpl3p (PRK04231)→ RplD (COG0088)rpl4lp (PRK04042)→ RplW (COG0089)PRK14548 (PRK14548)→ RplB (COG0090)rpl2p (PRK09612)→ RpsS (COG0185)rps19p (PRK04038)→ rpl22p (PRK04223)RplV (COG0091)→ RpsC (COG0092)rps3p (PRK04191)→ PRK14549 (PRK14549)Ribosomal_L29 (pfam00831)→ PRK03879 (PRK03879)UPF0086 (pfam01868)→ RpsQ (COG0186)rps17p (PRK08572)→ rpl14p (PRK08571)Ribosomal_L14 (pfam00238)→ rpl24p (PRK01191)KOW_RPL26 (cd06089)→ PRK04313 (PRK04313)KOW_RPS4 (cd06087)Ribosomal_S4e (pfam00900)→ rpl5p (PRK04219)Ribosomal_L5 (pfam00281)Ribosomal_L5_C (pfam00673)→ RpsN (COG0199)rps14P (PRK05766)→ rps8p (PRK04034)RpsH (COG0096)→ RplF (COG0097)rpl6p (PRK05518)→ rpl32e (PRK08562)Ribosomal_L32e (pfam01655)→
env-507	JGI24728J21555_1000542	-	9049	8980	**RNA**→ RplC (COG0087)rpl3p (PRK04231)→ RplD (COG0088)rpl4lp (PRK04042)→ RplW (COG0089)PRK14548 (PRK14548)→ RplB (COG0090)rpl2p (PRK09612)→ RpsS (COG0185)rps19p (PRK04038)→ rpl22p (PRK04223)RplV (COG0091)→ RpsC (COG0092)rps3p (PRK04191)→ PRK14549 (PRK14549)Ribosomal_L29 (pfam00831)→ PRK03879 (PRK03879)UPF0086 (pfam01868)→ RpsQ (COG0186)rps17p (PRK08572)→ rpl14p (PRK08571)Ribosomal_L14 (pfam00238)→ rpl24p (PRK01191)KOW_RPL26 (cd06089)→ PRK04313 (PRK04313)KOW_RPS4 (cd06087)Ribosomal_S4e (pfam00900)→ rpl5p (PRK04219)Ribosomal_L5 (pfam00281)Ribosomal_L5_C (pfam00673)→ RpsN (COG0199)rps14P (PRK05766)→ rps8p (PRK04034)RpsH (COG0096)→ RplF (COG0097)rpl6p (PRK05518)→ rpl32e (PRK08562)Ribosomal_L32e (pfam01655)→
env-508	taComm3_C12988	+	619	703	**RNA**→ rpl3p (PRK04231)Ribosomal_L3 (pfam00297)→

env-509	JGI12104J13512_1000732	+	3737	3822	**RNA**→ RplC (COG0087)rpl3p (PRK04231)→ RplD (COG0088)rpl4lp (PRK04042)→<br>RplW (COG0089)PRK14548 (PRK14548)→ RplB (COG0090)rpl2p (PRK09612)→<br>RpsS (COG0185)rps19p (PRK04038)→ rpl22p (PRK04223)RplV (COG0091)→<br>RpsC (COG0092)rps3p (PRK04191)→ PRK14549 (PRK14549)Ribosomal_L29 (pfam00831)→<br>PRK03879 (PRK03879)UPF0086 (pfam01868)→ RpsQ (COG0186)rps17p (PRK08572)→<br>rpl14p (PRK08571)Ribosomal_L14 (pfam00238)→ rpl24p (PRK01191)KOW_RPL26 (cd06089)→<br>PRK04313 (PRK04313)S4 (cd00165)KOW_RPS4 (cd06087)Ribosomal_S4e (pfam00900)→<br>RplE (COG0094)rpl5p (PRK04219)→ RpsN (COG0199)rps14P (PRK05766)→<br>rps8p (PRK04034)RpsH (COG0096)→ RplF (COG0097)rpl6p (PRK05518)→<br>rpl32e (PRK08562)Ribosomal_L32e (pfam01655)→ rpl19e (PRK08570)Ribosomal_L19e (pfam01280)→<br>RplR (COG0256)rpl18p (PRK08569)→ RpsE (COG0098)rps5p (PRK04044)→<br>Ribosomal_L7_archeal_euk (cd01657)rpl30p (PRK06049)→ rpl15p (PRK06419)Ribosomal_L27A (pfam00828)→<br>SecY (COG0201)PRK08568 (PRK08568)→ AdkA (COG2019)PRK04040 (PRK04040)→ DUF106 (pfam01956)→<br>CmkB (COG1102)PRK04182 (PRK04182)→ Lactamase_B (smart00849)DHPS-like_MBL-fold (cd07713)→
env-510	JGI11944J13513_1000175	+	47191	47276	**RNA**→ RplC (COG0087)rpl3p (PRK04231)→ RplD (COG0088)rpl4lp (PRK04042)→<br>RplW (COG0089)PRK14548 (PRK14548)→ RplB (COG0090)rpl2p (PRK09612)→<br>RpsS (COG0185)rps19p (PRK04038)→ rpl22p (PRK04223)RplV (COG0091)→<br>RpsC (COG0092)rps3p (PRK04191)→ PRK14549 (PRK14549)Ribosomal_L29 (pfam00831)→<br>PRK03879 (PRK03879)UPF0086 (pfam01868)→ RpsQ (COG0186)rps17p (PRK08572)→<br>rpl14p (PRK08571)Ribosomal_L14 (pfam00238)→ rpl24p (PRK01191)KOW_RPL26 (cd06089)→<br>PRK04313 (PRK04313)S4 (cd00165)KOW_RPS4 (cd06087)Ribosomal_S4e (pfam00900)→<br>RplE (COG0094)rpl5p (PRK04219)→ RpsN (COG0199)rps14P (PRK05766)→<br>rps8p (PRK04034)RpsH (COG0096)→ RplF (COG0097)rpl6p (PRK05518)→<br>rpl32e (PRK08562)Ribosomal_L32e (pfam01655)→ rpl19e (PRK08570)Ribosomal_L19e (pfam01280)→<br>RplR (COG0256)rpl18p (PRK08569)→ RpsE (COG0098)rps5p (PRK04044)→
Mta-1-1	NZ_AGIY02000001.1	-	1752945	1752858	**RNA**→ hypo→ RplC (COG0087)rpl3p (PRK04231)→ RplD (COG0088)rpl4lp (PRK04042)→<br>RplW (COG0089)PRK14548 (PRK14548)→ RplB (COG0090)rpl2p (PRK09612)→<br>RpsS (COG0185)rps19p (PRK04038)→ rpl22p (PRK04223)RplV (COG0091)→<br>RpsC (COG0092)rps3p (PRK04191)→ PRK14549 (PRK14549)Ribosomal_L29 (pfam00831)→<br>PRK03879 (PRK03879)UPF0086 (pfam01868)→ RpsQ (COG0186)rps17p (PRK08572)→<br>rpl14p (PRK08571)Ribosomal_L14 (pfam00238)→ rpl24p (PRK01191)KOW_RPL26 (cd06089)→<br>PRK04313 (PRK04313)S4 (cd00165)KOW_RPS4 (cd06087)Ribosomal_S4e (pfam00900)→<br>RplE (COG0094)rpl5p (PRK04219)→ RpsN (COG0199)rps14P (PRK05766)→<br>rps8p (PRK04034)RpsH (COG0096)→ RplF (COG0097)rpl6p (PRK05518)→<br>rpl32e (PRK08562)Ribosomal_L32e (pfam01655)→ rpl19e (PRK08570)Ribosomal_L19e (pfam01280)→<br>RplR (COG0256)rpl18p (PRK08569)→ RpsE (COG0098)rps5p (PRK04044)→
env-511	taComm3_C1003	-	71292	71205	**RNA**→ hypo→ RplC (COG0087)rpl3p (PRK04231)→ RplD (COG0088)rpl4lp (PRK04042)→<br>RplW (COG0089)PRK14548 (PRK14548)→ RplB (COG0090)rpl2p (PRK09612)→<br>RpsS (COG0185)rps19p (PRK04038)→ rpl22p (PRK04223)RplV (COG0091)→<br>RpsC (COG0092)rps3p (PRK04191)→ PRK14549 (PRK14549)Ribosomal_L29 (pfam00831)→<br>PRK03879 (PRK03879)UPF0086 (pfam01868)→ RpsQ (COG0186)rps17p (PRK08572)→<br>rpl14p (PRK08571)Ribosomal_L14 (pfam00238)→ rpl24p (PRK01191)KOW_RPL26 (cd06089)→<br>PRK04313 (PRK04313)S4 (cd00165)KOW_RPS4 (cd06087)Ribosomal_S4e (pfam00900)→<br>RplE (COG0094)rpl5p (PRK04219)→ RpsN (COG0199)rps14P (PRK05766)→<br>rps8p (PRK04034)RpsH (COG0096)→ RplF (COG0097)rpl6p (PRK05518)→<br>rpl32e (PRK08562)Ribosomal_L32e (pfam01655)→ rpl19e (PRK08570)Ribosomal_L19e (pfam01280)→<br>RplR (COG0256)rpl18p (PRK08569)→ RpsE (COG0098)rps5p (PRK04044)→

env-512	JGI12104J13512_1000711	-	24857	24770	**RNA**→ hypo→ RplC (COG0087)rpl3p (PRK04231)→ RplD (COG0088)rpl4lp (PRK04042)→<br>RplW (COG0089)PRK14548 (PRK14548)→ RplB (COG0090)rpl2p (PRK09612)→<br>RpsS (COG0185)rps19p (PRK04038)→ rpl22p (PRK04223)RplV (COG0091)→<br>RpsC (COG0092)rps3p (PRK04191)→ PRK14549 (PRK14549)Ribosomal_L29 (pfam00831)→<br>PRK03879 (PRK03879)UPF0086 (pfam01868)→ RpsQ (COG0186)rps17p (PRK08572)→<br>rpl14p (PRK08571)Ribosomal_L14 (pfam00238)→ rpl24p (PRK01191)KOW_RPL26 (cd06089)→<br>PRK04313 (PRK04313)S4 (cd00165)KOW_RPS4 (cd06087)Ribosomal_S4e (pfam00900)→<br>RplE (COG0094)rpl5p (PRK04219)→ RpsN (COG0199)rps14P (PRK05766)→<br>rps8p (PRK04034)RpsH (COG0096)→ RplF (COG0097)rpl6p (PRK05518)→<br>rpl32e (PRK08562)Ribosomal_L32e (pfam01655)→ rpl19e (PRK08570)Ribosomal_L19e (pfam01280)→<br>RplR (COG0256)rpl18p (PRK08569)→ RpsE (COG0098)rps5p (PRK04044)→
env-513	JGI11944J13513_1000170	-	24861	24774	**RNA**→ hypo→ RplC (COG0087)rpl3p (PRK04231)→ RplD (COG0088)rpl4lp (PRK04042)→<br>RplW (COG0089)PRK14548 (PRK14548)→ RplB (COG0090)rpl2p (PRK09612)→<br>RpsS (COG0185)rps19p (PRK04038)→ rpl22p (PRK04223)RplV (COG0091)→<br>RpsC (COG0092)rps3p (PRK04191)→ PRK14549 (PRK14549)Ribosomal_L29 (pfam00831)→<br>PRK03879 (PRK03879)UPF0086 (pfam01868)→ RpsQ (COG0186)rps17p (PRK08572)→<br>rpl14p (PRK08571)Ribosomal_L14 (pfam00238)→ rpl24p (PRK01191)KOW_RPL26 (cd06089)→<br>PRK04313 (PRK04313)S4 (cd00165)KOW_RPS4 (cd06087)Ribosomal_S4e (pfam00900)→<br>RplE (COG0094)rpl5p (PRK04219)→ RpsN (COG0199)rps14P (PRK05766)→<br>rps8p (PRK04034)RpsH (COG0096)→ RplF (COG0097)rpl6p (PRK05518)→<br>rpl32e (PRK08562)Ribosomal_L32e (pfam01655)→ rpl19e (PRK08570)Ribosomal_L19e (pfam01280)→<br>RplR (COG0256)rpl18p (PRK08569)→ RpsE (COG0098)rps5p (PRK04044)→
Hbo-1-1	NC_014729.1	+	955608	955707	**RNA**→ Methyltrn_RNA_3 (pfam02598)→ rpl3p (PRK04231)Ribosomal_L3 (pfam00297)→<br>RplD (COG0088)rpl4lp (PRK04042)→ RplW (COG0089)PRK14548 (PRK14548)→<br>RplB (COG0090)rpl2p (PRK09612)→ RpsS (COG0185)rps19p (PRK04038)→<br>rpl22p (PRK04223)RplV (COG0091)→ RpsC (COG0092)rps3p (PRK04191)→<br>PRK14549 (PRK14549)Ribosomal_L29 (pfam00831)→ PRK03879 (PRK03879)UPF0086 (pfam01868)→<br>RpsQ (COG0186)rps17p (PRK08572)→ rpl14p (PRK08571)Ribosomal_L14 (pfam00238)→<br>RplX (COG0198)rpl24p (PRK01191)→<br>PRK04313 (PRK04313)KOW_RPS4 (cd06087)Ribosomal_S4e (pfam00900)→ RplE (COG0094)rpl5p (PRK04219)→<br>RpsN (COG0199)rps14P (PRK05766)→ rps8p (PRK04034)RpsH (COG0096)→<br>RplF (COG0097)rpl6p (PRK05518)→ PRK12766 (PRK12766)RecA (COG0468)Rpl32e (COG1717)→<br>rpl19e (PRK08570)Ribosomal_L19e (pfam01280)→
Ngr-1-1	NC_019792.1	-	316138	316039	**RNA**→ Methyltrn_RNA_3 (pfam02598)→ rpl3p (PRK04231)Ribosomal_L3 (pfam00297)→<br>RplD (COG0088)rpl4lp (PRK04042)→ RplW (COG0089)PRK14548 (PRK14548)→<br>RplB (COG0090)rpl2p (PRK09612)→ RpsS (COG0185)rps19p (PRK04038)→<br>rpl22p (PRK04223)RplV (COG0091)→ RpsC (COG0092)rps3p (PRK04191)→<br>RpmC (COG0255)PRK14549 (PRK14549)→ PRK03879 (PRK03879)UPF0086 (pfam01868)→<br>RpsQ (COG0186)rps17p (PRK08572)→ rpl14p (PRK08571)Ribosomal_L14 (pfam00238)→<br>rpl24p (PRK01191)KOW_RPL26 (cd06089)→<br>PRK04313 (PRK04313)S4 (cd00165)KOW_RPS4 (cd06087)Ribosomal_S4e (pfam00900)→<br>RplE (COG0094)rpl5p (PRK04219)→ RpsN (COG0199)rps14P (PRK05766)→<br>rps8p (PRK04034)RpsH (COG0096)→ RplF (COG0097)rpl6p (PRK05518)→<br>PRK12766 (PRK12766)RecA (COG0468)Rpl32e (COG1717)→ rpl19e (PRK08570)Ribosomal_L19e (pfam01280)→
Hje-1-1	NC_014297.1	+	971638	971737	**RNA**→ Methyltrn_RNA_3 (pfam02598)→ RplC (COG0087)rpl3p (PRK04231)→<br>RplD (COG0088)rpl4lp (PRK04042)→ RplW (COG0089)PRK14548 (PRK14548)→<br>RplB (COG0090)rpl2p (PRK09612)→ RpsS (COG0185)rps19p (PRK04038)→<br>rpl22p (PRK04223)RplV (COG0091)→ RpsC (COG0092)rps3p (PRK04191)→<br>PRK14549 (PRK14549)Ribosomal_L29 (pfam00831)→ PRK03879 (PRK03879)UPF0086 (pfam01868)→<br>RpsQ (COG0186)rps17p (PRK08572)→ rpl14p (PRK08571)Ribosomal_L14 (pfam00238)→<br>rpl24p (PRK01191)KOW_RPL26 (cd06089)→<br>PRK04313 (PRK04313)KOW_RPS4 (cd06087)Ribosomal_S4e (pfam00900)→ RplE (COG0094)rpl5p (PRK04219)→<br>RpsN (COG0199)rps14P (PRK05766)→ rps8p (PRK04034)RpsH (COG0096)→<br>RplF (COG0097)rpl6p (PRK05518)→ PRK12766 (PRK12766)RecA (COG0468)Rpl32e (COG1717)→<br>rpl19e (PRK08570)Ribosomal_L19e (pfam01280)→

Nma-1-1	NC_013922.1	+	222997	223096	**RNA**→ Methyltrn_RNA_3 (pfam02598)→ rpl3p (PRK04231)Ribosomal_L3 (pfam00297)→<br>RplD (COG0088)rpl4lp (PRK04042)→ RplW (COG0089)PRK14548 (PRK14548)→<br>RplB (COG0090)rpl2p (PRK09612)→ RpsS (COG0185)rps19p (PRK04038)→<br>rpl22p (PRK04223)RplV (COG0091)→ RpsC (COG0092)rps3p (PRK04191)→<br>RpmC (COG0255)PRK14549 (PRK14549)→ PRK03879 (PRK03879)UPF0086 (pfam01868)→<br>RpsQ (COG0186)rps17p (PRK08572)→ rpl14p (PRK08571)Ribosomal_L14 (pfam00238)→<br>rpl24p (PRK01191)KOW_RPL26 (cd06089)→<br>PRK04313 (PRK04313)S4 (cd00165)KOW_RPS4 (cd06087)Ribosomal_S4e (pfam00900)→<br>RplE (COG0094)rpl5p (PRK04219)→ RpsN (COG0199)rps14P (PRK05766)→<br>rps8p (PRK04034)RpsH (COG0096)→ RplF (COG0097)rpl6p (PRK05518)→<br>PRK12766 (PRK12766)RecA (COG0468)Rpl32e (COG1717)→
Hal-1-1	NZ_AOLL01000031.1	-	14961	14862	**RNA**→ Methyltrn_RNA_3 (pfam02598)→ rpl3p (PRK04231)Ribosomal_L3 (pfam00297)→<br>RplD (COG0088)rpl4lp (PRK04042)→ RplW (COG0089)PRK14548 (PRK14548)→<br>RplB (COG0090)rpl2p (PRK09612)→ RpsS (COG0185)rps19p (PRK04038)→<br>rpl22p (PRK04223)RplV (COG0091)→ RpsC (COG0092)rps3p (PRK04191)→<br>PRK14549 (PRK14549)Ribosomal_L29 (pfam00831)→ PRK03879 (PRK03879)UPF0086 (pfam01868)→<br>RpsQ (COG0186)rps17p (PRK08572)→ rpl14p (PRK08571)Ribosomal_L14 (pfam00238)→<br>RplX (COG0198)rpl24p (PRK01191)→<br>PRK04313 (PRK04313)KOW_RPS4 (cd06087)Ribosomal_S4e (pfam00900)→ RplE (COG0094)rpl5p (PRK04219)→<br>RpsN (COG0199)rps14P (PRK05766)→ rps8p (PRK04034)RpsH (COG0096)→<br>RplF (COG0097)rpl6p (PRK05518)→ PRK12766 (PRK12766)RecA (COG0468)Rpl32e (COG1717)→<br>rpl19e (PRK08570)Ribosomal_L19e (pfam01280)→
Hvo-1-1	NC_013967.1	-	2419949	2419850	**RNA**→ Methyltrn_RNA_3 (pfam02598)→ rpl3p (PRK04231)Ribosomal_L3 (pfam00297)→<br>RplD (COG0088)rpl4lp (PRK04042)→ RplW (COG0089)PRK14548 (PRK14548)→<br>RplB (COG0090)rpl2p (PRK09612)→ RpsS (COG0185)rps19p (PRK04038)→<br>rpl22p (PRK04223)RplV (COG0091)→ RpsC (COG0092)rps3p (PRK04191)→<br>PRK14549 (PRK14549)Ribosomal_L29 (pfam00831)→ PRK03879 (PRK03879)UPF0086 (pfam01868)→<br>RpsQ (COG0186)rps17p (PRK08572)→ rpl14p (PRK08571)Ribosomal_L14 (pfam00238)→<br>RplX (COG0198)rpl24p (PRK01191)→<br>PRK04313 (PRK04313)KOW_RPS4 (cd06087)Ribosomal_S4e (pfam00900)→ RplE (COG0094)rpl5p (PRK04219)→<br>RpsN (COG0199)rps14P (PRK05766)→ rps8p (PRK04034)RpsH (COG0096)→<br>RplF (COG0097)rpl6p (PRK05518)→ PRK12766 (PRK12766)RecA (COG0468)Rpl32e (COG1717)→<br>rpl19e (PRK08570)Ribosomal_L19e (pfam01280)→
Nti-1-1	NZ_KB913017.1	-	3677377	3677278	**RNA**→ Methyltrn_RNA_3 (pfam02598)→ rpl3p (PRK04231)Ribosomal_L3 (pfam00297)→<br>RplC (COG0087)rpl3p (PRK04231)→ rpl3p (PRK04231)Ribosomal_L3 (pfam00297)→<br>RplD (COG0088)rpl4lp (PRK04042)→ RplW (COG0089)PRK14548 (PRK14548)→<br>RplB (COG0090)rpl2p (PRK09612)→ RpsS (COG0185)rps19p (PRK04038)→<br>rpl22p (PRK04223)RplV (COG0091)→ RpsC (COG0092)rps3p (PRK04191)→<br>RpmC (COG0255)PRK14549 (PRK14549)→ PRK03879 (PRK03879)UPF0086 (pfam01868)→<br>RpsQ (COG0186)rps17p (PRK08572)→ rpl14p (PRK08571)Ribosomal_L14 (pfam00238)→<br>rpl24p (PRK01191)KOW_RPL26 (cd06089)→<br>PRK04313 (PRK04313)KOW_RPS4 (cd06087)S4 (pfam01479)Ribosomal_S4e (pfam00900)→<br>RplE (COG0094)rpl5p (PRK04219)→ RpsN (COG0199)rps14P (PRK05766)→<br>rps8p (PRK04034)RpsH (COG0096)→ RplF (COG0097)rpl6p (PRK05518)→<br>PRK12766 (PRK12766)RecA (COG0468)Rpl32e (COG1717)→ rpl19e (PRK08570)Ribosomal_L19e (pfam01280)→

Npe-1-1	NC_019962.1	+	854999	855098	**RNA**→ Methyltrn_RNA_3 (pfam02598)→ RplC (COG0087)rpl3p (PRK04231)→ RplD (COG0088)rpl4lp (PRK04042)→ RplW (COG0089)PRK14548 (PRK14548)→ RplB (COG0090)rpl2p (PRK09612)→ RpsS (COG0185)rps19p (PRK04038)→ rpl22p (PRK04223)RplV (COG0091)→ RpsC (COG0092)rps3p (PRK04191)→ RpmC (COG0255)PRK14549 (PRK14549)→ PRK03879 (PRK03879)UPF0086 (pfam01868)→ RpsQ (COG0186)rps17p (PRK08572)→ rpl14p (PRK08571)Ribosomal_L14 (pfam00238)→ rpl24p (PRK01191)KOW_RPL26 (cd06089)→ PRK04313 (PRK04313)KOW_RPS4 (cd06087)Ribosomal_S4e (pfam00900)→ RplE (COG0094)rpl5p (PRK04219)→ RpsN (COG0199)rps14P (PRK05766)→ rps8p (PRK04034)RpsH (COG0096)→ RplF (COG0097)rpl6p (PRK05518)→ UvrC (COG0322)Rpl32e (COG1717)rpl32e (PRK08562)HHH_5 (pfam14520)→ rpl19e (PRK08570)Ribosomal_L19e (pfam01280)→
Hti-1-1	NC_021921.1	-	1979794	1979695	**RNA**→ Methyltrn_RNA_3 (pfam02598)→ RplC (COG0087)rpl3p (PRK04231)→ RplD (COG0088)rpl4lp (PRK04042)→ RplW (COG0089)PRK14548 (PRK14548)→ RplB (COG0090)rpl2p (PRK09612)→ RpsS (COG0185)rps19p (PRK04038)→ rpl22p (PRK04223)RplV (COG0091)→ RpsC (COG0092)rps3p (PRK04191)→ PRK14549 (PRK14549)Ribosomal_L29 (pfam00831)→ PRK03879 (PRK03879)UPF0086 (pfam01868)→ RpsQ (COG0186)rps17p (PRK08572)→ rpl14p (PRK08571)Ribosomal_L14 (pfam00238)→ RplX (COG0198)rpl24p (PRK01191)→ PRK04313 (PRK04313)KOW_RPS4 (cd06087)Ribosomal_S4e (pfam00900)→ RplE (COG0094)rpl5p (PRK04219)→ RpsN (COG0199)rps14P (PRK05766)→ rps8p (PRK04034)RpsH (COG0096)→ RplF (COG0097)rpl6p (PRK05518)→

2.3 Conserved domains

Conserved domains found in protein-coding genes listed in Section 2.2 are shown below, with the first sentence in their description from the Conserved Domain Database (if any). Conserved domains associated with more than one L4-Archaeoglobi RNA are assigned a color, while others are shown in gray. The number in parentheses after the colored domain name is the number of occurrences in Section 2.2.

cd00141 (6) Nucleotidyltransferase (NT) domain of family X DNA Polymerases. [D/E]
cd00165 (46) S4/Hsp/ tRNA synthetase RNA-binding domain; The domain surface is populated by conserved, charged residues that define a likely RNA-binding site; Found in stress proteins, ribosomal proteins and tRNA synthetases; This may imply a hitherto unrecognized functional similarity between these three protein classes.
cd00355 (1) Ribosomal protein L30, which is found in eukaryotes and prokaryotes but not in archaea, is one of the smallest ribosomal proteins with a molecular mass of about 7kDa.
cd00427 (22) Ribosomal L29 protein/HIP.
cd00513 (12) Ribosomal_L32_L32e: L32 is a protein from the large subunit that contains a surface-exposed globular domain and a finger-like projection that extends into the RNA core to stabilize the tertiary structure.
cd01428 (2) Adenylate kinase (ADK) catalyzes the reversible phosphoryl transfer from adenosine triphosphates (ATP) to adenosine monophosphates (AMP) and to yield adenosine diphosphates (ADP).
cd01513 (1) Domain III of Elongation factor (EF) Tu (EF-TU) and related proteins.
cd01657 (30) Ribosomal protein L7, which is found in archaea and eukaryotes but not in prokaryotes, binds domain II of the 23S rRNA as well as the 5S rRNA and is one of five ribosomal proteins that mediate the interactions 5S rRNA makes with the ribosome.
cd01781 (1) Ubiquitin domain of AT-6, second repeat.
cd02034 (1) The accessory protein CooC, which contains a nucleotide-binding domain (P-loop) near the N-terminus, participates in the maturation of the nickel center of carbon monoxide dehydrogenase (CODH).
cd02411 (7) K homology RNA-binding domain (KH) of the archaeal 30S small ribosomal subunit S3 protein.
cd02572 (2) Pseudouridine synthase, human dyskerin like.
cd03586 (1) DNA Polymerase IV/Kappa.
cd03694 (1) Domain II of the GTPBP family of GTP binding proteins.
cd06087 (219) KOW motif of Ribosomal Protein S4 (RPS4).
cd06089 (159) KOW motif of Ribosomal Protein L26.
cd07713 (1) Methanocaldococcus jannaschii dihydropteroate synthase, Thermoanaerobacter tengcongensis Tflp, and related proteins; MBL-fold metallo hydrolase domain.
cd12188 (4) Saccharopine Dehydrogenase NAD-binding and catalytic domains.
cd16490 (1) RING-CH finger, H2 subclass (C4HC3-type), found in Fanconi anemia group L protein (FANCL) and similar proteins.
COG0087 (255) Ribosomal protein L3 [Translation, ribosomal structure and biogenesis]
COG0088 (321) Ribosomal protein L4 [Translation, ribosomal structure and biogenesis]
COG0089 (310) Ribosomal protein L23 [Translation, ribosomal structure and biogenesis]
COG0090 (299) Ribosomal protein L2 [Translation, ribosomal structure and biogenesis]
COG0091 (269) Ribosomal protein L22 [Translation, ribosomal structure and biogenesis]
COG0092 (256) Ribosomal protein S3 [Translation, ribosomal structure and biogenesis]
COG0094 (188) Ribosomal protein L5 [Translation, ribosomal structure and biogenesis]

COG0096 (210) Ribosomal protein S8 [Translation, ribosomal structure and biogenesis]
COG0097 (208) Ribosomal protein L6P/L9E [Translation, ribosomal structure and biogenesis]
COG0098 (59) Ribosomal protein S5 [Translation, ribosomal structure and biogenesis]
COG0185 (256) Ribosomal protein S19 [Translation, ribosomal structure and biogenesis]
COG0186 (242) Ribosomal protein S17 [Translation, ribosomal structure and biogenesis]
COG0198 (75) Ribosomal protein L24 [Translation, ribosomal structure and biogenesis]
COG0199 (213) Ribosomal protein S14 [Translation, ribosomal structure and biogenesis]
COG0200 (2) Ribosomal protein L15 [Translation, ribosomal structure and biogenesis]
COG0201 (25) Preprotein translocase subunit SecY [Intracellular trafficking, secretion, and vesicular transport]
COG0255 (60) Ribosomal protein L29 [Translation, ribosomal structure and biogenesis]
COG0256 (85) Ribosomal protein L18 [Translation, ribosomal structure and biogenesis]
COG0272 (4) NAD-dependent DNA ligase [Replication, recombination and repair]
COG0322 (25) Excinuclease UvrABC, nuclease subunit [Replication, recombination and repair]
COG0468 (99) RecA/RadA recombinase [Replication, recombination and repair]
COG1102 (4) Cytidylate kinase [Nucleotide transport and metabolism]
COG1422 (3) Uncharacterized archaeal membrane protein, DUF106 family, distantly related to YidC/Oxa1 [Function unknown]
COG1471 (2) Ribosomal protein S4E [Translation, ribosomal structure and biogenesis]
COG1555 (1) DNA uptake protein ComE and related DNA-binding proteins [Replication, recombination and repair]
COG1588 (34) RNase P/RNase MRP subunit p29 [Translation, ribosomal structure and biogenesis]
COG1717 (131) Ribosomal protein L32E [Translation, ribosomal structure and biogenesis]
COG1841 (1) Ribosomal protein L30/L7E [Translation, ribosomal structure and biogenesis]
COG2019 (2) Archaeal adenylate kinase [Nucleotide transport and metabolism]
COG2106 (89) Predicted RNA methylase MTH1, SPOUT superfamily [General function prediction only]
COG2251 (4) Predicted nuclease, RecB family [General function prediction only]
COG5271 (1) Midasin, AAA ATPase with vWA domain, involved in ribosome maturation [Translation, ribosomal structure and biogenesis]
pfam00181 (5) Ribosomal Proteins L2, RNA binding domain.
pfam00189 (3) Ribosomal protein S3, C-terminal domain.
pfam00203 (23) Ribosomal protein S19.
pfam00237 (3) Ribosomal protein L22p/L17e.
pfam00238 (239) Ribosomal protein L14p/L23e.
pfam00276 (4) Ribosomal protein L23.
pfam00281 (36) Ribosomal protein L5.
pfam00297 (180) Ribosomal protein L3.
pfam00347 (4) Ribosomal protein L6.
pfam00366 (3) Ribosomal protein S17.
pfam00410 (2) Ribosomal protein S8.
pfam00573 (16) Ribosomal protein L4/L1 family.
pfam00633 (2) Helix-hairpin-helix motif.
pfam00673 (35) ribosomal L5P family C-terminus.
pfam00828 (25) Ribosomal proteins 50S-L15, 50S-L18e, 60S-L27A.
pfam00831 (172) Ribosomal L29 protein.
pfam00900 (227) Ribosomal family S4e.
pfam01280 (169) Ribosomal protein L19e.
pfam01472 (2) PUA domain.
pfam01479 (47) S4 domain.
pfam01655 (63) Ribosomal protein L32.
pfam01868 (219) Domain of unknown function UPF0086.
pfam01956 (2) Integral membrane protein DUF106.
pfam02598 (347) Putative RNA methyltransferase.
pfam03947 (12) Ribosomal Proteins L2, C-terminal domain.
pfam04147 (1) Nop14-like family.
pfam09547 (1) Stage IV sporulation protein A (spore_IV_A).
pfam10559 (1) Plug domain of Sec61p.
pfam12226 (1) Turkey astrovirus capsid protein.
pfam14520 (61) Helix-hairpin-helix domain.
pfam15250 (1) Raftlin.
PHA03252 (1) DNA packaging tegument protein UL25; Provisional
PRK00279 (2) adenylate kinase; Reviewed
PRK01191 (235) 50S ribosomal protein L24P; Validated
PRK02201 (1) putative inner membrane protein translocase component YidC; Provisional
PRK03879 (253) ribonuclease P protein component 1; Validated
PRK04034 (214) 30S ribosomal protein S8P; Reviewed
PRK04038 (284) 30S ribosomal protein S19P; Provisional
PRK04040 (2) adenylate kinase; Provisional
PRK04042 (341) 50S ribosomal protein L4P; Provisional
PRK04044 (59) 30S ribosomal protein S5P; Reviewed
PRK04182 (4) cytidylate kinase; Provisional
PRK04191 (260) 30S ribosomal protein S3P; Reviewed
PRK04219 (226) 50S ribosomal protein L5P; Reviewed
PRK04223 (272) 50S ribosomal protein L22P; Reviewed
PRK04231 (435) 50S ribosomal protein L3P; Reviewed
PRK04270 (2) H/ACA RNA-protein complex component Cbf5p; Reviewed
PRK04313 (234) 30S ribosomal protein S4e; Validated
PRK05518 (209) 50S ribosomal protein L6P; Reviewed
PRK05766 (213) 30S ribosomal protein S14P; Reviewed
PRK05883 (2) acyl carrier protein; Validated
PRK06049 (32) 50S ribosomal protein L30P; Reviewed
PRK06419 (28) 50S ribosomal protein L15P; Reviewed
PRK07233 (1) hypothetical protein; Provisional
PRK08562 (116) 50S ribosomal protein L32e; Validated
PRK08568 (25) preprotein translocase subunit SecY; Reviewed
PRK08569 (86) 50S ribosomal protein L18P; Reviewed
PRK08570 (169) 50S ribosomal protein L19e; Reviewed
PRK08571 (240) 50S ribosomal protein L14P; Reviewed
PRK08572 (246) 30S ribosomal protein S17P; Reviewed
PRK09612 (316) 50S ribosomal protein L2P; Validated
PRK10575 (3) iron-hydroxamate transporter ATP-binding subunit; Provisional
PRK10611 (1) chemotaxis methyltransferase CheR; Provisional
PRK10811 (1) ribonuclease E; Reviewed
PRK12317 (1) elongation factor 1-alpha; Reviewed
PRK12442 (1) translation initiation factor IF-1; Reviewed
PRK12766 (86) 50S ribosomal protein L32e; Provisional
PRK13493 (1) chemoreceptor glutamine deamidase CheD; Provisional
PRK14548 (313) 50S ribosomal protein L23P; Provisional
PRK14549 (254) 50S ribosomal protein L29P; Provisional
smart00322 (6) K homology RNA-binding domain.
smart00382 (1) ATPases associated with a variety of cellular activities.

smart00739 (2) KOW (Kyprides, Ouzounis, Woese) motif.
smart00849 (1) Metallo-beta-lactamase superfamily.
TIGR01393 (1) elongation factor 4. [Unknown function, General]
TIGR02903 (1) ATP-dependent protease, Lon family. [Protein fate, Degradation of proteins, peptides, and glycopeptides, Cellular processes, Sporulation and germination]
TIGR03594 (1) ribosome-associated GTPase EngA. [Protein synthesis, Other]
TIGR03636 (2) ribosomal protein uL23, archaeal form. [Protein synthesis, Ribosomal proteins: synthesis and modification]
TIGR03653 (3) ribosomal protein uL6, archaeal form. [Protein synthesis, Ribosomal proteins: synthesis and modification]

2.4 Multiple-sequence alignment

Each L4-Archaeoglobi RNA is denoted by an abbreviation (like "Eco-1-1") that refers to a taxonomy given in Section 2.1. The alignment may include sequences containing the RNA motif, as well as flanking sequence. The L4-Archaeoglobi RNA itself is denoted by the line underneath marked 5′ and 3′ on either end. Nucleotides in flanking sequences (i.e., not part of the motif) are written in gray letters. Stems of predicted rho-independent transcription terminators, if any, are shaded yellow. (Note: terminator predictions have not been analyzed manually, and many are likely to be false positives. Terminator predictions are those of the RNIE software.) Nucleotides predicted to function (as DNA) as transcription-factor binding sites, if any, are shaded in green. (But note: these predictions are manually annotated, so they might be under-predicted.) Annotated start codons, if any, are shaded green. (Note: start codons are frequently misannotated, especially in environmental samples.) Nucleotides proposed to basepair as part of the consensus structure are shaded in color when they comprise Watson-Crick or G-U pairs. Otherwise they are shaded gray. Conserved stems are also indicated at the bottom of the alignment by angle brackets, where matching < and > denote base-paired columns. Below these angle brackets, the symbol "2" denotes base pairs exhibiting covariation according to the statistically well-founded R-scape method. "1" denotes base pairs exhibiting covariation according to R2R's simplistic method. "0" denotes base pairs that are not observed to mutate and "?" denotes base pairs that have a significant frequency of non-canonical nucleotides for Watson-Crick or G-U pairs (> 5%). Below these base pair annotation is the consensus sequence: "R" = "A" or "G", "Y" = "C" or "U", red nucleotides: nucleotide identity conserved more than 97% of the time, black nucleotides: 90%, gray nucleotides: 75%, red circle (•): nucleotide is present 97% of the time, black circle (•): 90%, gray circle (•): 75%, white circle (∘): 50%. All percentages of sequences just described (e.g. 97% conserved) assume that sequences have been weighted by the GSC algorithm implemented by the Infernal software package.

Duplicate sequences: the following putative homologs are not shown in the alignment because their sequences are identical to a homolog already shown: Hdi-2-1, Hgi-2-1, Hha-1-2, Hhi-1-1, Hje-2-1, Hla-1-2, Hla-3-1, Hma-1-1, Hme-1-2, Hpa-1-2, Hsa-3-1, Hsi-1-1, Hsp-1-1, Hsp-4-1, Hsp-5-1, Hte-2-1, Hvo-1-1, Hwa-2-1, Mco-2-1, Mhu-1-1, Nal-1-1, Nsp-1-1, env-100, env-111, env-112, env-12, env-121, env-124, env-125, env-132, env-133, env-141, env-142, env-143, env-144, env-16, env-162, env-163, env-168, env-171, env-177, env-18, env-19, env-192, env-194, env-195, env-20, env-200, env-202, env-205, env-208, env-21, env-211, env-212, env-213, env-22, env-229, env-230, env-234, env-235, env-236, env-239, env-26, env-262, env-263, env-264, env-267, env-273, env-276, env-279, env-280, env-281, env-282, env-283, env-284, env-285, env-286, env-287, env-288, env-289, env-290, env-291, env-294, env-299, env-300, env-301, env-302, env-307, env-308, env-309, env-311, env-312, env-313, env-314, env-319, env-327, env-328, env-329, env-330, env-336, env-342, env-343, env-344, env-345, env-346, env-347, env-348, env-362, env-363, env-364, env-365, env-366, env-367, env-368, env-369, env-388, env-391, env-393, env-394, env-396, env-397, env-398, env-401, env-404, env-411, env-412, env-417, env-419, env-42, env-420, env-421, env-422, env-424, env-426, env-427, env-431, env-432, env-438, env-447, env-453, env-454, env-457, env-458, env-459, env-46, env-461, env-463, env-47, env-470, env-473, env-48, env-487, env-488, env-489, env-490, env-491, env-493, env-494, env-496, env-497, env-498, env-499, env-500, env-501, env-502, env-503, env-506, env-507, env-51, env-510, env-513, env-52, env-53, env-60, env-61, env-66, env-67, env-68, env-69, env-7, env-73, env-75, env-85, env-9, env-97, har-1-1

alignment positions 1 · · · 79

```
Mli-1-1  GUGAUGCCCCGGCUGCCCCUUAUGGGGGCACAACCGAGAAGGACUCUAAUCCAGUCCCAAGGAGCUGACAGACGCUGGA
env-1    UUCCCAGAUCCGUUCUCAUUCGUAAUACGAAAUGAGACCAGAAAGGAGUUGUACAAACAACAGGGCUGAAUUAGUGGGA
env-2    CAAUGUCACGAAGGAUAUAUCGGUUGGAGAAUAUAGAGAGGGAUUAGUACUCGAAAGGUCUAGCGAAGAGAAAGUAGGU
env-3    UAAUCUCGAUAAAACAACGGAAGUCGGUGAAAUAAGAGAGGGGUUUGUGUUAGAGAAGUUUGGUGAUGAGAGAGUAGGU
env-4    AUCGGGGACGGAUCGCCGUGCCCGGUCAUGUUAUGGGACGAAUGCCGUGCUUCUCGCACGGUGGCUGAUAGACGCUGGG
env-5    AUCGGGGGCGGAUCGCCGUGCCCGGUCAUGUUAUGGGACGAAUGCCGUGCUUCUCGCACGGUGGCUGAUAGACGCUGGG
env-6    GAAUGUUCCAUUUAGAGGCCAUUUAGGCUUUGAAAUGAGCAAUAAUUGCAUAAUAUAGCAAUGGGCUGAAAGAGUGGGA
env-8    UAAUUGAACCUAAUCUGACUUUUUUCGGUCAAGACGCGGUCAUAAAUUGCUUUAAUUGCAAUGGGCUGAAAGAGUGGGA

         ...............................................................................
         ...............................................................................
         •••••••••••••••••••••••••••••••••••••RR•••RRR•••••••••••••RRR•••GY•R••RRRGY•GGA
```

env-10	UGAAUGUACUGAUUCUGGCUUCUUUUAGCUAUGAAGCGGUCAAAAAUUGCUUUAAUUGCAAUGGGCUGAUAGAGUGGGA
env-11	UGAAUGUACUGAUUCUGGCUUCUUUUAGCUAUGAAGCGGUCAAAAAUUGCUUUAAUUGCAAUGGGCUGAUAGAGUGGGA
env-13	CUGAGGUCACGGCCCUCUCAUCUGACAGGGCCGUGGUUAUGGGAGUUGUGAGGAGAUCACAACCGGCUGACAUGGUGGA
env-14	GAAUGGUGUGAAGAUCCCGAUCCCGGGAUCAUAAACCGAUAGGAGUUGUGCCAGGGCACAACGGGCUGACAGAGCUGGA
env-15	CCUCCGCGUGUCGUCUACGACCGGCUCUGAGUCAACUGAUUCAGGGUCGUUAACACAGGGAAUCGUGACUGAGGUCGGA
Hwa-1-1	CCUCCGCGUGUCAUCUACGACCGGCUCUGAGUCAACUGAUUCAGGGUCGUUAACACAGGGAAUCGUGACCGAGGUCGGA
env-17	CCUCCGCGUGUCAUCUACGACCGGCUCUGAGUCAACUGAUUCAGGGUCGUUAACACAGGGAAUCGUGACCGAGGUCGGA
env-23	ACUCCGCGUCCCGUCGCAGACCGGCUCGACUCCAGACGGAGCCGAGUCGUCAACACAGGGAAUCGUGACCAAGGUAGGA
env-24	...................................AGUCCGAACCCGAGUACAGAGAAGGCGUGGUGACGAAAGUCGGA
env-25	GCUCCGCACGCGUUCACAGACCGGCUCCGGAUCAGAUGGUUCGGGGUCGUUAAGACAGGGAAUCGUGACCGAGGUCGGA
env-27	GAUGCUGCCGACGGCCGAGGCCGGCGCCGGAUCGGAGGGUCCGGGGUCGUUAAUAGACGGAAUCGUGACCGAGGUCGGA
env-28	GCUUCGCGCCACGACACAGACCGGCUCCGAAUCAGACGGUUCGGGGUCGUCAAAACACGGAAUCGUGACCGAGGUCGGA
env-29	GCUUCGCGCCACGACACAGACCGGCUCCGAAUCAGACGGUUCGGGGUCGUCAAAACACGGAAUCGUGACCGAGGUCGGA
env-30	GCUUCGCGCCACGACACAGACCGGCUCCGAAUCAGACGGUUCGGGGUCGUCAAAACACGGAAUCGUGACCGAGGUCGGA
env-31	GCUCCGCGUGCGUUCACAGACCGGCUCCGGAUCCGAGGGUUCGGGGUCGUUAAGACAGGGAAUCGUGACCGAGGUCGGA
Hut-1-1	GCUCCGCGUUGCGACCCAGACCGGCUCCGGAUCGGACGAUUCGGGGUCGUUAAGACAGGGAAUCGUGACCGAGGUCGGA
env-32	GAUGCAGCCGUCGGCCGAGGCCGGCUCCGGAUCGCAGGGUCCGGGGUCGUUAAUAGACGGAAUCGUGACCGAGGUCGGA
env-33	GAUGCAGCCGUCGGCCGAGGCCGGCUCCGGAUCGCAGGGUCCGGGGUCGUUAAUAGACGGAAUCGUGACCGAGGUCGGA
env-34	GAUGCAGCCAUCGGCCGAGGCCGGCUCCGGAUCGCAGGGUCCGGGGUCGUUAAUAGACGGAAUCGUGACCGAGGUCGGA
env-35	GAUGCAGCCGUCGGCCGAGGCCGGCUCCGGAUCGCAGGGUCCGGGGUCGUUAAUAGACGGAAUCGUGACCGAGGUCGGA
env-36	GCUCCGCGUGGUGACACAGACCGGCUCCGGAUCCGAGGGUUCGGGGUCGUUAAGACAGGGAAUCGUGACCGAGGUCGGA
env-37	GCUCCGCGUCACAACACGGACCGGCUCCGGAUCGGAGGGUUCGGGGUCGUUAAGACAGGGAAUCGUGACCGAUGUCGGU
env-38	....GUCGUUAAGACAGGGAAUCGUGACCGAUGUCGGU
env-39	GCUCCGCGUCACAACACGGACCGGCUCCGGAUCGGAGGGUUCGGGGUCGUUAAGACAGGGAAUCGUGACCGAUGUCGGU
env-40	GCUUCGGCUGCCGACAUGGACCGGCUCCGAAUCAAAGGAUUCGGGGUCGAUAAGAGAAGGAAUCGUGACCGAGGUCGGA
env-41	CCUCCGUAUCCCGUCUAUGACCGGCUCUGAAUCAGAUGAUUCAGAGUCGUUAACACAAGGAAUCGUGACCGAGGUCGGA
env-43	GCUCCGCGUGCGUUCACAGACCGGCUCCGGAUCCGAGGGUUCGGGGUCGUUAAGACAGGGAAUCGUGACCGAGGUCGGC
env-44	GCUCCGCGCCACGCCACAGACCGGCUCCGGAUCGAACGGUCCGGGGUCGUCAAGACGAGGGAUCGUGACCGAGGUCGGA
env-45	ACCGCUCCGCGCAUCAUCACGGACAUCGACCGACCCAGACGGGCCGGUCGAACAAACGGGAAUCGUGACCGAGGUCGGA
env-49	CCCGUUGCGGUGCUCGUCGCAGACCACAGCCGAUUCGUCCGAGUCGGCUGUCAACCAGGGAAUCGUGACCGAGGUCGGA
env-50	GCCGCUCCGGGUUUCGUCACAGACCGUAGCCGACCCGAACGGGCCGGCUACACACCAAGGAAUCGUGACCGAGGUCGGA
env-54	CCCGUUGGGCGUCUCGUCGCAGACCACAGCCGAUUCGUCCGAGUCGGCUGUCAACCACGGAAUCGUGACCGAGGUCGGA
env-55	UCCGCUCCGCGUUUCGUCACGGACGGUAACCGAUCCAAACGGAUCGGUUACGCACCAAGGAAUCGUGACCAAGGUCGGA
env-56	....CCGGCUACACACCAAGGAAUCGUGACCAAGGUCGGA
env-57	CCGGGUUUCGUCACAGACCGUAGCCGGUCCAGACGACGAUGGACCGGCUACACACCAAGGAAUCGUGACCAAGGUCGGA
env-58	ACCGCUCCGCGUUUCGUCACAGACCGUAGCCAACCCGGAUGGGCCGGCUACACACCAGGGAAUCGUGACCAAGGUCGGA
env-59	UCCGCUCCGGGUUUCGUCACAGACCGUAGCCGAUCCAGCCGGAUCGGUUACACAUCAAGGAAUCGUGACCAAGGUCGGA
env-62	UCCGCUCCGGGUUUCGUCACAGACCGUAGCCGAUCCAGCCGGAUCGGUUACACAUCAAGGAAUCGUGACCAAGGUCGGA
env-63	UCCGCUCCGGGUUUCGUCACAGACCGUAGCCGAUCCAGCCGGAUCGGUUACACAUCAAGGAAUCGUGACCAAGGUCGGA
env-64	...................ACAGACCGUAGCCGAUCCAGCCGGAUCGGUUACACAUCAAGGAAUCGUGACCAGGUCGGA
env-65	UCCGCUCCGGGUUUCGUCACAGACCGUAGCCGAUCCAGCCGGAUCGGUUACACAUCAAGGAAUCGUGACCAAGGUCGGA
env-70	....................AGACCGUAGCCGAUCCAGCCGGAUCGGUUACACAUCAAGGAAUCGUGACCAAGGUCGGA
env-71	.................CACAGACCGUAGCCGAUCCAGCCGGAUCGGUUACACAUCAAGGAAUCGUGACCAAGGUCGGA
env-72	GCUCCGUGCCACGUCACAGGCCGGCUCCGGAUCGAACGGUCCGGGGUCGAGAAGACAAGGGAUCGUGACCGAGGUCGGA
env-74	GCUCCGUGCCACGUCACAGACCGGCUCCGGAUCGAACGGUCCGGGGUCGAGAAGACAAGGGAUCGUGACCGAGGUCGGA
env-76	GCUCCACCUGGGUGCACAGACCGGCUCCGGAUCGAACGGUUCGGGGUCGUCAAGACAGGGCAUCGUGACCGAGGUCGGA
env-77	GCUCCACCUGGGUGCACGGACCGGCUCCGGAUCCGACGGUUCGGGGUCGUCAAGACAGGGCAUCGUGACCGAGGUCGGA
env-78	CGCUCCACCUGGGUGCACGGACCGGCUCCGGAUCCGACGUUCGGGGUCGUCAAGACAGGGCAUCGUGACCGAGGUCGGA
Hsa-1-1	GCUCCACGUGACACCACGGACCGGCUCCGGAUCGAACGAUUCGGGGUCGUUGAGACAGGGACUCGUGACCGAGGUCGGA
Hgi-1-1	CGUUCUCGUGGCGUCUACGACCGCGGACGACUCUGACGAGUCGCCCGCGUUGCGAGAAGGAAUCGUGACCGAGGUCGGA
Hsu-2-1	CGUUCUCGUCGCGUCUACGACCGCGGACGACUCCGACGAGUCGCCCGCGUUGCGAGAAGGAAUCGUGACCGAGGUCGGA
Hpr-1-1	CGUUCUCGUGGCGUCUACGACCGCGGACGACUCUGACGAGUCGCCCGCGUUGCGAGAAGGAAUCGUGACCGAGGUCGGA
Hsp-8-1	CGUUCUCGUCGCGUCUACGACCGCGGACGACUCCGACGAGUCGCCCGCGUUGCGAGAAGGAAUCGUGACCGAGGUCGGA
Hde-1-1	CGUUCUCGUCGCGUCUACGACCGCGGACGACUCCGACGAGUCGCCCGCGUUGCGAGAAGGAAUCGUGACCGAGGUCGGA
Hlu-1-1	CGUUCUCGUCGCGUCUACGACCGCGGACGACUCCGACGAGUCGCCCGCGUUGCGAGAAGGAAUCGUGACCGAGGUCGGA
Hal-2-1	CGUUCUCGUGGCGUCUACGACCGCGGACGGCUCUGACGAGUCGCCCGCGUUGCGAGAAGGAAUCGUGACCGAGGUCGGA
Hsp-6-1	CGUUCUCGUGGCGUCUACGACCGCGGACGACUCCGACGAGUCGCCCGCGUUGCGAGAAGGAAUCGUGACCGAGGUCGGA
Hme-1-1	GGUCCUGGUAUCGUCUACGACCGCGGGCGACUCUGACGAGACGCCCGCGUUGCGAGAAGGAAUCGUGACCGAGGUCGGA
Hla-2-1	CGUUCUCGUCUCGUCUACGACCGCGGACGACUCUGACGAGUUGCCCGCGUUGCGAGAAGGAAUCGUGACCGAGGUCGGA
Hel-1-1	CGUUCUCGUCUCGUCUACGACCGCGGACGACUCUGACGAGUUGCCCGCGUUGCGAGAAGGAAUCGUGACCGAGGUCGGA
Hmu-1-1	GGUCCUGGUAUCGUCUACGACCGCGGGCGACUCUGACGAGACGCCCGCGUUGCGAGAAGGAAUCGUGACCGAGGUCGGA
env-79	GCUCCGCGUCACAACACGGACCGGCUCCGGAUCGGAGGGUUCGGGGUCGUUAAGACAGGGAAUCGUGACCGAUGUCGGU
env-80	GCUCCGCGCGCGUUCACAGACCGGCUCCGGAUCGGAGGGUUCGGGGUCGUUAAGACAGGGAAUCGUGACGAAGGCCGGA
env-81	GCUCCGCGCGCGUUCACAGACCGGCUCCGGAUCGGAGGGUUCGGGGUCGUUAAGACAGGGAAUCGUGACGAAGGCCGGA
env-82	GCUCCGCGCGUUGUCACAGACCGGCUCCGGAUCAGACGGUUCGGGGUCGUUAAGACAGGGAAUCGUGACGAAGGCCGGA
env-83	GCUCCGCGCGUGUUCACAGACCGGCUCCGGAUCGGACGGUUCGGGGUCGUUAAGACAGGGAAUCGUGACGAAGGCCGGA
Hla-4-1	GCUCCGUGCCACGUCACAGACCGGCUCCGAAUCCGACGAUUCGGGGUCGAAAAGACAAGGAAUCGUGACCGAGGUCGGA
Ssp-1-1	GCUCCACGCCUCUUCACGGACCGGCUCCGGAUCGAGCGGUCCGGGGUCGUUGAGACAGGGUAUCGUGACCGAGGUCGGA
env-84	GCUCCGUCUCUCGGCAUGGACCGGCUCCGAAUCGAGCGGUUCGGGGUCGAUACGACAGGGAGUAGUGACCCAGGUCGGA
env-86	GCUCCGUGCCACCUCACAGACCGGCUCCGAAUCGGAGGGUUCGGGGUCGUCAAGACAAGGGAUCGUGACCGAGGUCGGA
Hsp-11-1	GCUCCGCGCACCGUCACAGACCGGCUCCGAAUCCGACGAUUCGGGGUCGAAAAGAUACGGAAUCGUGACCGAGGUCGGA
env-87	....UUCGGGGCGUUAACACAGGGAAUCGUGACCAAGGUCGGA
env-88	ACCGCUCCGCGUUUCGUCACAGACCGUAGCCAACCCGGAUGGGCCGGCUACACACCAGGGAAUCGUGACCAAGGUCGGA
env-89	GCUCCGCAUCGCAACACAGACCGGCUCCGGAUCGGAGGGUUCGGGGUCGUUAAGACAGGGAAUCGUGACCGAGGUCGGA
Hka-1-1	GCUCCGCGUCCGUUCACAGACCGGCUCCGGAUCUGAGGGUUCGGGGUCGUUAAGACAGGGAAUCGUGACCGAGGUCGGA
Hmu-2-1	GCUCCGCGUCCGUUCACAGACCGGCUCCGGAUCUGAGGGUUCGGGGUCGUUAAGACAGGGAAUCGUGACCGAGGUCGGA
Hca-3-1	GCUCCGGGUCCGCUCAUGGACCGGCUCCGGUUCGGACGAAUCGGGGUCGUUAAGACAGGGAAUCGUGACCGAGGUCGGU
Hsa-5-1	GCUUCGCGCCGUCUCACGGACCGGCUCCGAAUCGCAAGGUUCGGGGUCGUCAAGACAGGGAAUCGUGACCGAGGUCGGC
env-90	GCUUCGCACCGUUUCACGGACCGGCUCCGGAUCACAGAAUCCGGGGUCGUUAAGACAGGGAAUCGUGACCGAGGUCGGC
Hag-1-1	GCUUCGCGCCGUCUCACGGACCGGCUCCGAAUCCCAAGGUUCGGGGUCGUCAAGACAGGGAAUCGUGACCGAGGUCGGC
Hsa-4-1	GCUUCGCGCCGUCUCACGGACCGGCUCCGAAUCCCAAGGUUCGGGGUCGUCAAGACAGGGAAUCGUGACCGAGGUCGGC
env-91	GCUCCGCGUGCGUUCACAGACCGGCUCCGGACCGAAGCGUUCGGGGUCGUCAAGACAGGGAAUCGUGACCGAGGUCGGA
env-92	CCCGUUGCGGCUCUCGUCGCAGACCACAGCCGAUUCGUCCGAGUCGGCUGUCAACCAGGGAAUCGUGACCGAGGUCGGA
env-93	CCCGUUGCGGCUCUCGUCGCAGACCACAGCCGAUUCGUCCGAGUCGGCUGUCAACCAGGGAAUCGUGACCGAGGUCGGA
	....
	....
	•••••••••••••••••••••••••••••••••••••RR•••RRR•••••••••••••RRR•••GY•R••RRRGY•GGA

```
env-94     CCCGUUGCGGCUCUCGUCGCAGACCACAGCCGAUUCGUCCGAGUCGGCUGUCAACCAGGGAAUCGUGACCGAGGUCGGA
env-95     CCCGUUGCGGCUCUCGUCGCAGACCACAGCCGAUUCGUCCGAGUCGGCUGUCAACCAGGGAAUCGUGACCGAGGUCGGA
env-96     GCCCUUGCGGCUCUCGUCGCAGACCACAGCCGAUUCGUCCGAGUCGGCUGUCAACCAGGGAAUCGUGACCGAGGUCGGA
env-98     GCCCUUGCGGCUCUCGUCGCAGACCACAGCCGAUUCGUCCGAGUCGGCUGUCAACCAGGGAAUCGUGACCGAGGUCGGA
env-99     CCCAUUGCGGUGCUCGUCGCAGACCACAGCCGAUUCGUCCGAGUCGGCUGUCAACCAGGGAAUCGUGACCGAGGUCGGA
env-101    GCCGCUCCGCGUUUCGUCACAGACGGUAGCCGACCCAGACGGGACGGCUACACACCAAGGAAUCGUGACCAAGGUCGGA
env-102    ...........................................CCGGCUACACACCAAGGAAUCGUGACCAAGGUCGGA
env-103    .............................................GGUUACGCACCAAGGAAUCGUGACCAAGGUCGGA
env-104    GCUCCGGGUUUCGUCACAGACCGUAGCCGAUCCGGACGACGGACCGGCUACACACCAAGGAAUCGUGACCAAGGUCGGA
env-105    CCGCGUCUCGUCGCAGACCGUAGCCGAUCCAGACGACGACGGACCGGCUACACACCAAGGAAUCGUGACCAAGGUCGGA
env-106    ....................................................CACCAAGGAAUCGUGACCAAGGUCGGA
env-107    .........................UCCGGACCUGAGCGUCCGGGGUCGUUACAAGAGGGAAUCGUGACCGAGGUCGGA
env-108    ....AUGCCGCCGCUGCUCCCGUCGCGGACGGGUUCCGAGUCAUCGGAACUGCGAGAAGGAAUCGUGACCGAGGUCGGA
env-109    GCUCCGCGUCUCGUCGCAGACCGGCUCCGGAUCGGACGGUUCGGGGUCGUAAAGACAGGGAAUCGUGACCGAGGUCGGA
CHa-1-1    CGUGCUCCCGCCGAUGCGGCCCGACUCAUCCGGCCCCGACGGGUCGGAGCGACGAGCCGGAAUCGUGACCGAGGUCGGA
Hpa-2-1    CCUCCUCGUCUCGUCUACGACCGGCUCCGAAUCGAACGAUUCGGGGUCGUUACAACAGGGAAUCGUGACCGAGGUCGGA
env-110    GCUUCGCGCCGCGUCACAGACCGGCUCCGAAUCGAACGGUUCGGGGUCGUCAAGACAAGGGAUCGUGACCGAGGUCGGA
Nhu-1-1    GCUCCGCGCCACGUCACAGACCGGCUCCGAAUCAGACGGUUCGGGGUCGUUAAGACAAGGGAUCGUGACCGAGGUCGGA
env-113    GCUCCGCGUGCCGUGGCGGACCGGCUCGACCCCUAGCGGGGAAGAGUCGAAAACACAGGGACUCGUGACCGAGGUCGGA
env-114    CCCGUUGCGGUGCUCGUCGCAGACCACAGCCGAUUCGUCCGAGUCGGCUGUCAACCAGGGAAUCGUGACCGAGGUCGGA
env-115    CCCGUUGCGGUGCUCGUCGCAGACCACAGCCGAUUCGUCCGAGUCGGCUGUUAACCAGGGAAUCGUGACCGAGGUCGGA
env-116    ................................CGGACGACGGACCGGCUACACACCAAGGAAUCGUGACCAAGGUCGGA
env-117    ......................GCCGAUCCAGGCGAUGACGGACCGGCUACACACCAAGGAAUCGUGACCAAGGUCGGA
env-118    .....................................GACGGACCGGCUACACACCAAGGAAUCGUGACCAAGGUCGGA
env-119    GCCGCUCCGCGUUUCGUCACAGACCGUAGCCGACCCAGACGGGGCGGCUACACACCAAGGAAUCGUGACCAAGGUCGGA
env-120    UGUGCUCCCGCCGUUGUUGCCAGACACCUCCGGCCCAGACGGGGCGGAGCUACGAGACGGAAUCGUGACCGAGGUCGGA
env-122    CGUGCUCCCGCCGCUGUUGCCAGACACCUCCGGCCCAGACGGGGCGGAGCUGCGAGACGGAAUCGUGACCGAGGUCGGA
env-123    CGUGCUCCCGCCGUUGUUGCCAGACACCUCCGGCCCAGACGGGGCGGAGCUCCGAGACGGAAUCGUGACCGAGGUCGGA
env-126    ..........CCGGCUACGACCGGCUCCGAAUCCAACGGUUCGGGGUCGUUAACACAGGGAAUCGUGACCGAGGUCGGA
env-127    CCUCCGCGUCCCGUCUACGACCGGCUCCGAAUCCAACGGUUCGGGGUCGUUAACACAGGGAAUCGUGACCGAGGUCGGA
env-128    CCUCCGCGUCCCGUCUACGACCGGCUCCGAAUCCAACGGUUCGGGGUCGUUAACACAGGGAAUCGUGACCGAGGUCGGA
env-129    GCUUCGCGCGCUCUCACGGACCGGCUCCGGAUCCACCGAUCCGGGGUCGUUAAGACAGGGAAUCGUGACCGAGGUCGGA
Hna-1-1    CCUUCGCGUCUCGUCACGGACCGGCUCCGGACCGGAGCGUCCGGGGUCGUUACAAGAGGGAAUCGUGACCGAGGUCGGA
env-130    CCUUCGCGUCUCGUCACGGACCGGCUCCGGACCGGAGCGUCCGGGGUCGUUACAAGAGGGAAUCGUGACCGAGGUCGGA
env-131    CCCGCUCCGCGUCUCCCCACGGACCGGCUCGGCGUCUGAACGGCCCGAGUCGAGAGAAGGAAUCGUGACCGAGGUCGGA
env-134    GCUCCGCGUCCGUUCACAGACCGGCUCCGGAUCGGACGGUUCGGGGUCGUUAAGACAGGGAAUCGUGACCGAGGUCGGA
env-135    ..................GACCGGCUCCGAAUCUACCGGUUCGGGGUCGUCAAGACAAGGAAUCGUGACCGAGGUCGGA
Nph-1-1    GCUCCGCGUGCGUUCACAGACCGGCUCCGGAUCGGACGGUUCGGGGUCGUUAAGACAGGGAAUCGUGACCGAGGUCGGA
env-136    GCUCCUCGUCCGUUCGCAGACCGGCUCCGGAUCGGACGGUUCGGGGUCGUUAAGACAGGGAAUCGUGACCGAGGUCGGA
env-137    GCUCCGCGUUCGUUCACAGACCGGCUCCGGAUCGGACGGUUCGGGGUCGUUAAGACAGGGAAUCGUGACCGAGGUCGGA
env-138    GCUCCGCGUACCAUGGCGGACCGGCUCGCCCCCAGACGGGAGCGAGUCGACAACACAGGGACUCGUGACCGAGGUCGGA
env-139    .................GGACCGGCUCGCCCCCGACGGGGAGGAGUCGACAACACAGGGACUCGUGACCGAGGUCGGA
env-140    GCUUCGCGUGUCUUGGCGGACCGGCUCGCCCCCCGACGGGGGCGAGUCGACAACACAGGGACUCGUGACCGAGGUCGGA
env-145    GCUCCGCGUGUCGUGGCGGACCGGCUCCCCCCCCGACGGGGCGGAGUCGAAAACACAGGGACUCGUGACCGAGGUCGGA
env-146    .................................................AAAACACAGGGACUCGUGACCGAGGUCGGA
env-147    GCUCCGCGUGUCGUGGCGGACCGGCUCGUCCCUCGACGGGGAGGAGUCGACAACACAGGGACUCGUGACCGAGGUCGGA
env-148    GCUCCGCGUGCCGUGGCGGACCGGCUCGUCCCCCGACGGGGAGGAGUCGAUAACACAGGGACUCGUGACCGAGGUCGGA
Hsp-9-1    GCUCCGCGUGCCGUGGCGGACCGGCUCGACCCCUAGCGGGGGAGAGUCGAAAACACAGGGACUCGUGACCGAGGUCGGA
Hez-1-1    GCUCCGCGUGCCGUGGCGGACCGGCUCGACCCCUAGCGGGGAAGAGUCGAAAACACAGGGACUCGUGACCGAGGUCGGA
env-149    .........................................AAGAGUCGAAAACACAGGGACUCGUGACCGAGGUCGGA
env-150    .........................................AACAGUCGAAAACACAGGGACUCGUGACCGAGGUCGGA
env-151    .....................................................CACAGGGACUCGUGACCGAGGUCGGA
Hco-1-1    GCUCCGCGUGCCGUGGCGGACCGGCUCGACCCCUAGCGGGGAAGAGUCGAAAACACAGGGACUCGUGACCGAGGUCGGA
env-152    GCUCCGCGUGCCGUGGCGGACCGGCUCGACCCCUAGCGGGGAAGAGUCGAAAACACAGGGACUCGUGACCGAGGUCGGA
env-153    GCUCCGCGUGCCGUGGCAGACCGGCUCGGCCCCCGACGGGGCGGAGUCGAAAACACAGGGACUCGUGACCGAGGUCGGA
env-154    ..............................................................UCGUGACCGAGGUCGGA
env-155    ....................................................ACACAGGGACUCGUGACCGAGGUCGGA
env-156    ...........................................GAGUCGAAAACACAGGGACUCGUGACCGAGGUCGGA
Hsp-10-1   GCUCCGCGUGCCGUGGCGGACCGGCUCGACCCCGAGCGGGGAAGAGUCGAAAACACAGGGACUCGUGACCGAGGUCGGA
Hli-1-1    GCUCCGCGUGCCGUGGCGGACCGGCUCGACCCCUAGCGGGGAAGAGUCGAAAACACAGGGACUCGUGACCGAGGUCGGA
Hte-1-1    GCUCCGCGUGCCGUGGCGGACCGGCUCGACCCCUAGCGGGGGAGAGUCGAAAACACAGGGACUCGUGACCGAGGUCGGA
Hko-1-1    GCUCCGCGUGCCGUGGCGGACCGGCUCGACCCCUAGCGGGGGAGAGUCGAAAACACAGGGACUCGUGACCGAGGUCGGA
Hli-2-1    GCUCCGCGUGCCGUGGCGGACCGGCUCGACCCCUAGCGGGGGAGAGUCGAAAACACAGGGACUCGUGACCGAGGUCGGA
Hsa-7-1    GCUCCGCGUGCCGUGGCGGACCGGCUCGACCCCUAGCGGGGAAGAGUCGAAAACACAGGGACUCGUGACCGAGGUCGGA
Hca-2-1    GCUCCGCGUGCCGUGGCGGACCGGCUCGACCCCUAACGGGGAAGAGUCGAAAACACAGGGACUCGUGACCGAGGUCGGA
Hsp-7-1    GCUCCGCGUGCCGUGGCGGACCGGCUCGACCCCUAGCGGGGGAGAGUCGAAAACACAGGGACUCGUGACCGAGGUCGGA
Hha-2-1    GCUCCGCGUGCCGUGGCGGACCGGCUCGACCCCUAGCGGGGAAGAGUCGAAAACACAGGGACUCGUGACCGAGGUCGGA
Hdi-1-1    GCUCCGCGUGCCGUGGCGGACCGGCUCGACCCCUAGCGGGGGAGAGUCGAAAACACAGGGACUCGUGACCGAGGUCGGA
Hsa-9-1    CGCUCCGCGUGCCGUGGCGGACCGGCUCGACCCUAGCGGGGAAGAGUCGAAAACACAGGGACUCGUGACCGAGGUCGGA
Har-2-1    GCUCCGCGUGCCGUGGCGGACCGGCUCGACCCCUAGCGGGGGAGAGUCGAAAACACAGGGACUCGUGACCGAGGUCGGA
env-157    .............................CCCCUAGCGGGGNAGAGUCGAAAACACAGGGACUCGUGACCGAGGUCGGA
env-158    .............................................................CUCGUGACCGAGGUCGGA
env-159    GCUCCGCGUGCCGUGGCGGACCGGCUCGACUCCUAGCGGAGAAGAGUCGAAAACACAGGGACUCGUGACCGAGGUCGGA
env-160    GCUCCGCGUGUCAUGGCGGACCGGCUCGUCCCCCGACGGGGAGGAGUCGACAACACAGGGACUCGUGACCGAGGUCGGA
h3A-1-1    GCUUCGUGUCCCGUCACGGACCGGCUCGCCCCCCGACGGGGACGAGUCGUCAACACAGGGACUCGUGACCGAGGUCGGA
Hai-1-1    GCUCCGCGUGCCGUGGCGGACCGGCUCGACCCCUAGCGGGGAAGAGUCGAAAACACAGGGACUCGUGACCGAGGUCGGA
env-161    GCUCCGCGUGCCGUGGCGGACCGGCUCGACCCCUAGCGGGGAAGAGUCGAAAACACAGGGACUCGUGACCGAGGUCGGA
Hho-1-1    GCUCCGCGUGCUGUGGCGGACCGGCUCGACCCCUAGCGGGGGAGAGUCGAAAACACAGGGACUCGUGACCGAGGUCGGA
env-164    .............................................GUCGAAAACACAGGGACUCGUGACCGAGGUCGGA
env-165    .........................CUCGCCCCAGACGGGGGCGAGUCGACAACACAGGGACUCGUGACCGAGGUCGGA
env-166    GCUCCGCGUACCAUGGCGGACCGGCUCGCCCCCAGACGGGAGCGAGUCGACAACACAGGGACUCGUGACCGAGGUCGGA
env-167    ..............GGCGGACCGGCUCGCCCCCAGACGGGAGCGAGUCGACAACACAGGGACUCGUGACCGAGGUCGGA
env-169    .........GCCGUGGCGGACCGGCUCGACCCCUAGCGGGGAAGAGUCGAAAACACAGGGACUCGUGACCGAGGUCGGA
env-170    ................................................................GUGACCGAGGUCGGA

           ...............................................................................
           ...............................................................................
           •••••••••••••••••••••••••••••••••••••RR•••RRR•••••••••••••RRR•••GY•R••RRRGY•GGA
```

```
env-172    GCUCCGCGUACCAUGGCGGACCGGCUCGCCCCCAGACGGGAGCGAGUCGACAACACAGGGACUCGUGACCGAGGUCGGA
env-173    GCUUCACGCCUUGUCGCAGACCGGCUCUGGAUCAAGUGAUCCGGGGUCGUUAAGACAGGGAAUCGUGACCGAGGUCGGA
Hru-1-1    CCUCCGCGUCCCGUCUAUGACCGGCUCCGAAUCAGACGGUUCGGGGUCGUUAACACAGGGAAUCGUGACCGAGGUCGGA
env-174    CCUCCGCGUCCCGUCUAUGACCGGCUCCGAAUCAGACGGUUCGGGGUCGUUAACACAGGGAAUCGUGACCGAGGUCGGA
Hru-2-1    CCCCAUGCGUGUCUCCCCACGGACCGGCUCCGAGGGUGAAACCCCGGAGUCGAGAGAAGGAAUCGUGACCGAGGUCGGA
env-175    GCUUCGUGUCCGUGCACAGACCGGCUCCGGAUCAGAUGGUUCGGGGUCGUUAAGACAGGGAAUCGUGACCGAGGUCGGA
env-176    GCUCCGGGUUCGUUCACAGACCGGCUCCGGAUCGGACGGUUCGGGGUCGUUAAGACAGGGAAUCGUGACCGAGGUCGGA
env-178    GCUCCGAGUUCGUUCACAGACCGGCUCCGGAUCGGACGGUUCGGGGUCGUUAAGACAGGGAAUCGUGACCGAGGUCGGA
env-179    GCUUCGUGUCCGUGCACAGACCGGCUCCGGAUCGGACGGUUCGGGGUCGUUAAGACAGGGAAUCGUGACCGAGGUCGGA
env-180    GCUCCGCGUCCGUACACAGACCGGCUCCGGAUCGGAUGGUUCGGGGUCGUUAAGACAGGGAAUCGUGACCGAGGUCGGA
Nmo-1-1    GCUUCGUGUCCGUACACAGACCGGCUCCGGAUCAGACGGUUCGGGGUCGUUAAGACAGGGAAUCGUGACCGAGGUCGGA
Hha-1-1    GCUUCGCCCCGUCCCACGGACCGGCUCCGGACCACAGAGUUCGGGGUCGUUAAGACAGGGAAUCGUGACCGAGGUCGGC
env-181    GCUCCGUGCCACGUCACAGACCGGCUCCGAAUCUGACGGUUCGGGGUCGUUAAAACAAGGGAUCGUGACCGAGGUCGGA
Hpa-1-1    GCUCCGCGCCACAUCACAGACCGGCUCCGAAUCGGAGGGUUCGGGGUCGUUAAGACAGGGAAUCGUGACCGAGGUCGGA
Hci-1-1    GCUCCGCGCACCAUCGCAGACCGGCUCCGAAUCCGACGAUUCGGGGUCGUUAAGACAGGGAAUCGUGACCGAGGUCGGA
env-182    GCUCCGCGCACCGUCGCAGACCGGCUCCGAAUCUGACGAUUCGGGGUCGACAAGACAGGGAAUCGUGACCGAGGUCGGA
env-183    CGCUUCGCGCGGCCGCUCGGACCGGCUCCGAAUCCACAGUUCGGGGUCGUCAAGACAGGGAAUCGUGACCGAGGUCGGC
env-184    GCUUCGGCAGACGCCACAGACCGGCUCCGGAUCCAGCGAUUCGGGGUCGUCAAGACAGGGAAUCGUGACCGAGGUCGGA
env-185    GCUUCGCGUCCCAUCACAGACCGGCUCCGAAUCGAACGGUUCGGGGUCGAUAAGACGAGGAAUCGUGACCGAGGUCGGA
Hdj-1-1    GCUCCGCGCUGUGUCACGGACCGGCUCCGAAUCCGACGGUUCGGGGUCGUUAAGACAAGGGAUCGUGACCGAGGUCGGA
Hsa-10-1   GCUCCGCGCCAUGUCACAGACCGGCUCCGAAUCUACCGGUUCGGGGUCGUCAAGACAAGGGAUCGUGACCGAGGUCGGA
Nsu-1-1    GCUCCGCGCCAUGUCACAGACCGGCUCCGAAUCUACCGGUUCGGGGUCGUCAAGACAAGGAAUCGUGACCGAGGUCGGA
Nba-1-1    GCUCCGCGCCAUGUCACAGACCGGCUCCGAAUCUACCGGUUCGGGGUCGUCAAGACAAGGAAUCGUGACCGAGGUCGGA
Nin-1-1    GCUCCGCGCGGUGUCACAGACCGGCUCCGAAUCGAACGGUUCGGGGUCGUUAAGACAAGGGAUCGUGACCGAGGUCGGA
env-186    GCUCCGCGCACCGUCACAGACCGGCUCCGAAUCAGACGAUUCGGGGUCGACAAGACAGGGAAUCGUGACCGAGGUCGGA
env-187    GCUCCGCGCACCGUCGCAGACCGGCUCCGAAUCUGACGAUUCGGGGUCGACAAGACAGGGAAUCGUGACCGAGGUCGGA
env-188    GCUCCGCGCACCGUCACAGACCGGCUCCGAAUCGAACGAUUCGGGGUCGUCAAGACAGGGAAUCGUGACCGAGGUCGGA
env-189    ...CCGCGCACCGUCACAGACCGGCUCCGAAUCGAACGGUUCGGGGUCGACAAGACAGGGAAUCGUGACCGAGGUCGGA
env-190    GCUCCGUGCCCCGUCACAGACCGGCUCCGAAUCGAACGGUUCGGGGUCGUCAAAACAAGGAAUCGUGACCGAGGUCGGA
env-191    GCUCCGUGCCCCGUCACAGACCGGCUCCGAAUCGAACGGUUCGGGGUCGUCAAAACAAGGAAUCGUGACCGAGGUCGGA
Hla-1-1    GCUCCGCGCCGUGUCACGGACCGGCUCCGAAUCGAACGAUUCGGGGUCGUUAAGACAAGGGAUCGUGACCGAGGUCGGA
env-193    GCUCCGUGCCCCGUCACAGACCGGCUCCGAAUCGGACGGUUCGGGGUCGUCAAGACAAGGAAUCGUGACCGAGGUCGGA
Hac-1-1    GCUCCGCCUCUCGUCAUGGACCGGCUCCGAAUCGAGCGGUUCGGGGUCGCAUCGAGAAGGGAUCGUGACCCAGGUCGGA
Hac-1-2    GCUCCGCCUCUCGUCAUGGACCGGCUCCGAAUCGAGCGGUUCGGGGUCGCAUCGAGAAGGGAUCGUGACCCAGGUCGGA
env-196    GCUCCGUCUCUCGUCAUGGACCGGCUCAGACUCGAGCGGGUCUGGGUCGUUACGACAGGGAAUCGUGACCCAGGUCGGA
env-197    ...............................................................CGUGACCCAGGUCGGA
env-198    .................................................................UGACCCAGGUCGGA
Hsp-2-1    GCUCCGUCUCUCGUCAUGGACCGGCUCAGACUCGAGCGGGUCUGGGUCGUUACGACAGGGAAUCGUGACCCAGGUCGGA
env-199    GCUGCGCGCCGUGUCACGGACCGGCUCCGGACCACAGAGUUCGGGGUCGUUGAGACAGGGAAUCGUGACCGAGGUCGGC
Hsu-1-1    GCUUCGCGUUUCAGCAUGGACCGGCUCCGAAUCGAGCGGUUCGGGGUCGUUACGACAGGGAAUAGUGACCCAGGUCGGA
env-201    GCUCCGCGUGCGUUCACAGACCGGCUCCGGAUCUGAGGGUUCGGGGUCGUUAAGACAGGGAAUCGUGACCGAGGUCGGA
env-203    GCUCCGCGUGCGUUCACAGACCGGCUCCGGAUCUGAGGGUUCGGGGUCGUUAAGACAGGGAAUCGUGACCGAGGUCGGA
Hca-1-1    GCUCCGCGUGCGUUCACAGACCGGCUCCGGAUCUGAGGGUUCGGGGUCGUUAAGACAGGGAAUCGUGACCGAGGUCGGA
Ham-1-1    GCUCCGCGUGCGUUCACAGACCGGCUCCGGAUCUGAGGGUUCGGGGUCGUUAAGACAGGGAAUCGUGACCGAGGUCGGA
Hhi-2-1    GCUCCGCGUGCGUUCACAGACCGGCUCCGGAUCUGAGGGUUCGGGGUCGUUAAGACAGGGAAUCGUGACCGAGGUCGGA
Hsa-2-1    GCUCCGCGUGCGUUCACAGACCGGCUCCGGAUCUGAGGGUUCGGGGUCGUUAAGACAGGGAAUCGUGACCGAGGUCGGA
Har-1-1    GCUCCGCGUGCGUUCACAGACCGGCUCCGGAUCUGAGGGUUCGGGGUCGUUAAGACAGGGAAUCGUGACCGAGGUCGGA
Hva-1-1    GCUCCGCGUGCGUUCACAGACCGGCUCCGGAUCUGAGGGUUCGGGGUCGUUAAGACAGGGAAUCGUGACCGAGGUCGGA
Hja-1-1    GCUCCGCGUGCGUUCACAGACCGGCUCCGGAUCUGAGGGUUCGGGGUCGUUAAGACAGGGAAUCGUGACCGAGGUCGGA
env-204    CCCAUUGGGCGUCUCGUCGCAGACCACGGCCGGUUCGUCCGAAUCGGCCGUCCACCACGGAAUCGUGACCGAGGUCGGA
Hse-1-1    GCUUCGCGCGGCCGCUCGGACCGGCUCCGGAUCGAACGGUUCGGGGUCGUCAAGACAGGGAAUCGUGACCGAGGUCGGC
Hth-1-1    GCUUCGCGCGGCCGCUCGGACCGGCUCCGAAUCGAACGGUUCGGGGUCGUUAAGACAGGGAAUCGUGACCGAGGUCGGC
Hmo-1-1    GCUUCGCGCGGCCGCUCGGACCGGCUCCGAAUCGAACGGUUCGGGGUCGUCAAGACAGGGAAUCGUGACCGAGGUCGGC
env-206    GCUGCGUGCGACGUCAUGGACCGGCUCCGGAUCCAACGGUUCGGGGUCGUUAAGACAGGGAAUCGUGACCGAGGUCGGA
env-207    CGUGCUCCCGCCCGUGCUUCCGGAUCGGUCCGGCCCGGAUGGGUCGGAACUCCGAGACGGAAUCGUGACCGAGGUCGGA
env-209    CCUCCGAAUCGUGUCUACGACCGGCUCCGACUCGGACGAGUCGGGGUCGUUAACACAGGGAAUCGUGACUGAGGUCGGA
Nta-1-1    GCUCCGCGCCAUGUCACAGACCGGCUCCGAAUCUACCGGUUCGGGGUCGUUAAGACAAGGGAUCGUGACCGAGGUCGGA
Nas-1-1    GCUCCGCGCCGUGUCACAGACCGGCUCCGAAUCUACCGGUUCGGGGUCGUUAAGACAAGGGAUCGUGACCGAGGUCGGA
Noc-1-1    GCUCCGUGCCGUGUCACGGACCGGCUCCGAAUCGAACGAUUCGGGGUCGUCAAGACAAGGAAUCGUGACCGAGGUCGGA
Nje-1-1    GCUCCGCGCCGUGUCACAGACCGGCUCCGAAUCGGACGAUUCGGGGUCGUCAAGACAAGGAAUCGUGACCGAGGUCGGA
Nam-1-1    GCUCCGCGCCGUGUCACAGACCGGCUCCGAAUCGAACGGUUCGGGGUCGUCAAGACAAGGAAUCGUGACCGAGGUCGGA
env-210    GCUCCGCGCCAUGUCACAGACCGGCUCCGAAUCAGACGGUUCGGGGUCGUCAAGACAAGGAAUCGUGACCGAGGUCGGA
Hsp-3-1    GCUCCGUCUCUCGUCAUGGACCGGCUCAGACUCGAGCGGGUCUGGGUCGUUACGACAGGGAAUCGUGACCCAGGUCGGA
Nch-1-1    GCUCCGCGCCACGUCACAGACCGGCUCCGAAUCAGACGGUUCGGGGUCGUUAAGACAAGGGAUCGUGACCGAGGUCGGA
Nae-1-1    GCUCCGCGCCAUGUCACAGACCGGCUCCGAAUCUACCGGUUCGGGGUCGUUAAGACAAGGGAUCGUGACCGAGGUCGGA
env-214    GCUCCGCGCCGUGUCACAGACCGGCUCCGAAUCGAACGGUUCGGGGUCGUCAAGACAAGGAAUCGUGACCGAGGUCGGA
env-215    ............................GAAUCGAACGGUUCGGGGUCGUCAAGACAAGGAAUCGUGACCGAGGUCGGA
env-216    GCUGCUCCCCGCGUCACGGACCGGCUCCGAAUCGAACGAUUCGGGGUCGUUAAGACAGGGAAUCGUGACCGAGGUCGGA
Hni-1-1    GCUGCGUGCGGCGUCACGGACCGGCUCCGAAUCGAACGAUUCGGGGUCGUUAAGACAAGGGAUCGUGACCGAGGUCGGA
Has-1-1    GCUCCGCGCUUCAUCACGGACCGGCUCCGAAUCGAACGGUUCGGGGUCGUUAAGACGAGGGAUCGUGACCGAGGUCGGA
env-217    GCUCCGCGCCAUGUCACAGACCGGCUCCGAAUCUACCGGUUCGGGGUCGUCAAGACAAGGAAUCGUGACCGAGGUCGGA
Nal-2-1    GCUCCGCGCCAUGUCACAGACCGGCUCCGAAUCUACCGGUUCGGGGUCGUCAAGACAAGGAAUCGUGACCGAGGUCGGA
Npa-1-1    GCUCCGCGCCAUGUCACAGACCGGCUCCGAAUCUACCGGUUCGGGGUCGUCAAGACAAGGAAUCGUGACCGAGGUCGGA
Nga-1-1    GCUCCGCGCCAUGUCACAGACCGGCUCCGAAUCUACCGGUUCGGGGUCGUCAAGACAAGGAAUCGUGACCGAGGUCGGA
Nsp-2-1    GCUCCGCGCCAUGUCACAGACCGGCUCCGAAUCUACCGGUUCGGGGUCGUCAAGACAAGGAAUCGUGACCGAGGUCGGA
Htu-1-1    GCUCCGCGCCAUGUCACAGACCGGCUCCGAAUCUACCGGUUCGGGGUCGUCAAGACAAGGGAUCGUGACCGAGGUCGGA
Hsa-8-1    GCUCCGCGCCAUGUCACAGACCGGCUCCGAAUCUACCGGUUCGGGGUCGUCAAGACAAGGGAUCGUGACCGAGGUCGGA
Hth-2-1    GCUCCGCGCGGUGUCACAGACCGGCUCCGAAUCGAACGGUUCGGGGUCGUCAAGACAAGGGAUCGUGACCGAGGUCGGA
Nve-1-1    GCUCCGCGCCAUGUCACAGACCGGCUCCGAAUCUACCGGUUCGGGGUCGUCAAGACAAGGAAUCGUGACCGAGGUCGGA
Hli-3-1    GCUCCGCGCGGUGUCACAGACCGGCUCCGAAUCGAACGGUUCGGGGUCGUCAAGACAAGGGAUCGUGACCGAGGUCGGA
Hxa-1-1    GCUGCGCGCGGUGUCACAGACCGGCUCCGAAUCGAACGAUUCGGGGUCGUUAAGACAAGGGAUCGUGACCGAGGUCGGA
Hgo-1-1    GCUCCGCGCCGUGUCACGGACCGGCUCCGAAUCGAACGAUUCGGGGUCGUUAAGACAAGGGAUCGUGACCGAGGUCGGA
env-218    GCUCCGCGCCACGUCACAGACCGGCUCCGAAUCGAACGGUUCGGGGUCGUCAAGACAAGGGAUCGUGACCGAGGUCGGA
env-219    ............GUCACAGACCGGCUCCGAAUCUGACGGUUCGGGGUCGUUAAGACAAGGAUCGUGACCGAGGUCGGA

           ...............................................................................
           ...............................................................................
           •••••••••••••••••••••••••••••••••••••RR•••RRR•••••••••••••RRR•••GY•R••RRRGY•GGA
```

```
env-220   GCUUCGGCAGACGCCACAGACCGGCUCCGGAUCCAGCGAUUCGGGGUCGUCAACACAGGGAAUCGUGACCGAGGUCGGA
env-221   GCUCCGCGCCAUGUCACAGACCGGCUCCGAAUCUGACGGUUCGGGGUCGUCAAGACAAGGGAUCGUGACCGAGGUCGGA
Hsa-6-1   CCUGCGCGUCUCCCCACAGACCGGCUCCGAAUCAGACGGUUCGGGGUCGUUAACACAGGGAAUCGUGACCGAGGUCGGA
env-222   GCUCCUCGUCCGUGCACAGACCGGCUCCGGAUCGAACGGUUCGGGGUCGUUAAGACAGGGAAUCGUGACCGAGGUCGGA
env-223   GCUCCGCGUGCGUUCACAGACCGGCUCCGGAUCGAACGGUUCGGGGUCGUUAAGACAGGGAAUCGUGACCGAGGUCGGA
env-224   ..............................................UCGUUAAGACAGGGAAUCGUGACCGAGGUCGGA
env-225   UCUUAGAAUCUUACCACAGGCCGGCUCUGGAUCAGAAGAUUCAGAGUCGUUAAGACAAGGAAUAGUGACCCAGGUCGGA
env-226   UCUUAGAAUUUUACCACAGGCCGGCUCUGGAUCAGAAGAUUCAGAGUCGUUAAGACAAGGAAUAGUGACCCAGGUCGGA
env-227   UCUUAGAAUUUUACCACAGGCCGGCUCUGGAUCAGAAGAUUCCGAGUCGUUAAGACAAGGAAUAGUGACCCAGGUCGGA
env-228   CCUCCGCGUGUCGUCUACGACCGGCUCUGAGUCAACUGAUUCGGGGUCGUUAACACAGGGAAUCGUGACCGAGGUCGGA
env-231   GUCGGGGGUAUGGACAUGAAGCGUUCAUACCUUUGCAUGAGUGCGUGGUGAGCAAUCAUCACCAGCUGACGUGGUUGGA
env-232   UCGGGGGUAUGGACAUGAAGCGUUCAUACCUUUGCAUGAGUGUCGUGGUGAGCAAUCAUCACCAGCUGACGUGGUUGGA
env-233   GUCGGGGGUAUGGACAUGAAGCGUUCAUACCUUUGCAUGAGUGCGUGGUGAGCAAUCAUCACCAGCUGACGUGGUUGGA
env-237   CUCAUAGGUGAGUGACUCCCGGAGGGGAGUUACCGAGAAGGAGUUGUGGACUAAUUCACAACGGGCUGACAUAGCUGGA
env-238   CCCUCCACUCAGAACUUCUCAUCAUCCGCUGAAAAGCGAGUGGAGGGAGUUUCGAGAGGGAGUAGUCGUAAAGGUCGCA
env-240   CAUCCCACCGUUGCGCAUACCCUCUCAUACAGUUUCGAAAGAAUCGGAAUACCGCGAAGGUAUUGUGACCAAUGUCGGG
env-241   AUCAUCUGACCUCACUGAUGGAGAAUUCCGCGUUGGGGUCGUGGUCGCAAGACCAAAGAAGAGCGAGACAAAAGUCGGA
env-242   CAUGCCUGGUGUUUCCUGCAGCCUGCAGGGAAAACUCACAUGGAGUUGUGAUCCGUCACAACGGGAAGACAGAGUUGGA
env-243   CAUGCCUGGUGUUUCCUGCAGCCUGCAGGGAAAACUCACAUGGAGUUGUGAUCCGUCACAACGGGAAGACAGAGUUGGA
env-244   AUGGGUGGGCGUGCGCCCCCGUGUACGGGGCGCAGCCGAGAAGGAGUUGUGAUCAUUCACAACGGGCUGACACGGUGGA
env-245   AUAUGUGGCGGGUGCCGUCCAUGCGGGCGGCACAGCCGAGAAGGAGUUGUGUUCAUUCACAACGGGCUGACACGGUGGA
env-246   AUAUGUGGCGGGUGCCGUCCAUGCGGGCGGCACAGCCGAGAAGGAGUUGUGUUCAUUCACAACGGGCUGACACGGUGGA
env-247   AUAUGUGGCGGGUGCCGUCCAUGCGGGCGGCACAGCCGAGAAGGAGUUGUGUUCAUUCACAACGGGCUGACACGGUGGA
env-248   AUAUGUGGCGGGUGCCGUCCAUGCGGGCGGCACAGCCGAGAAGGAGUUGUGUUCAUUCACAACGGGCUGACACGGUGGA
env-249   CAGGUAGUGUCAUCCGGAGAUCCCGGGUGGCGUAACCGAGACGGAGUUGUGAUCACUCACAACGGGCUGACUUAGUAGA
env-250   CAGGUAGCGCCAUCCGGAGAUUCCGGGUGGCUUCACCGAGACGGAGUUGUGAUCAUUCACAACGGGCUGACACAGUAGA
env-251   AAGAGAGUAUCAUUCUGCUGCCACGUGUGGCGCAGGCUGAUGAGUUCUGCGAUAUCGCAGACGAGAUGACCAUUGCAGG
env-252   AAGAGAGUAUCACCCUGCUACCGAUUGUGGUGCAGGCUGGUGAGUUCUGCGAUAUCGCAGACGAGAUGACCAUUGCAGG
env-253   AAGAUAGCAUCAACCUGUAACCAAUCGCGGUGCAGGAUGGUGAGUUCUGCGAUAACGCAGACGAGAUGACCAUUGCAGG
env-254   CAUCAUAGGGUCAUACCCUCAGGAGGGAAAACAAGAAGGAGUUGUGGUCAGACACCACAACGGGCUGACAGAGGUAGGU
env-255   UUGGUGGAUCCCUGCUCAUCAUGAGCGGGGGUAUCCCGAGAAGGAGUUGUGAUCCGUCACAACAGGCUGACAGAGUGGG
env-256   GAAGGUCGAACCCUCCACAUGGAGGGCCGAGUGUCGAUAUGGAGUUGUGCUCGUCACGAACGGGAUGACAGGAGCUGGA
env-257   GCGAAGGUCGAACCCUCCACAUGGAGGGCCGAUGUCGAUAUGGAGUUGUGCUCGUCACAACGGGAUGACAGGAGCUGGA
env-258   GCGAAGGUCGAACCCUCCACAUGGAGGGCCGAUGUCGAUAUGGAGUUGUGCUCGUCACAACGGGAUGACAGGAGCUGGA
env-259   GCGAAGGUCGAACCCUCCACAUGGAGGGCCGAUGUCGAUAUGGAGUUGUGCUCGUCACAACGGGAUGACAGGAGCUGGA
env-260   GCGAAGGUCGAACCCUCCACAUGGAGGGCCGAUGUCGAUAUGGAGUUGUGCUCGUCACAACGGGAUGACAGGAGCUGGA
env-261   GCGAAGGUCGAACCCUCCUCCCGGAGGGCCGAUGUCGAUACGGAGUUGUGCUCGUCACAACGGGAUGACAGGAGCUGGA
env-265   CACAAGGUCGAACCCUCCUCACGGAGGGCCGAUGUCGAUAUGGAGUUGUGCUCGUCACAACGGGAUGACAGGAGCUGGA
env-266   GUGGAGAUUGAACCCUCCUUACGGAGGGCAGGUGUCGAUAUGGAGUUGUGCUUGGCACAACGGGACGACAGGAGCUGGA
env-268   UGGAGAUUGAACCCUCCUUACGGAGGGCAGGUAGUCGAUAUGGAGUUGUGCUUGGCACAACGGGACGACAGGAGCUGGA
env-269   GUGGAGAUUGAACCCUCCUUACGGAGGGCAGGUGUCGAUAUGGAGUUGUGCUUGGCACAACGGGACGACAGGAGCUGGA
env-270   UGGAGAUUGAACCCUCCUUACGGAGGGACAGGUGUCGAUAUGGAGUUGUGCUUGGCACAACGGGACGACAGGAGCUGGA
env-271   UCGAAGGUCGAACCCUCCAGAUGGAGGGCGAUAUCGAUUGAGGAGUUGUGCUCGUCACAACGGGAUGACAGGAGCUGGA
env-272   UCGAAGGUCGAACCCUCCAGAUGGAGGGCGAUAUCGAUUGAGGAGUUGUGCUCGUCACAACGGGAUGACAGGAGCUGGA
env-274   UCGAAGGUCGAACCCUCCAGAUGGAGGGCGAUAUCGAUUGAGGAGUUGUGCUCGUCACAACGGGAUGACAGGAGCUGGA
env-275   ......................................UGAGGAGUUGUGCUCGUCACAACGGGAUGACAGGAGCUGGA
env-277   UCGAAGGUCGAACCCUCCAGAUGGAGGGCGAUAUCGAUUGAGGAGUUGUGCUCGUCACAACGGGAUGACAGGAGCUGGA
env-278   GCGAAGGUUGAACCCUCCAGAUGGAGGGCGAUGUCGAUUAAGGAGUUGUGCUCGUCACAACGGGAUGACAGGAGCUGGA
env-292   CAAAGGUCGAACCCUCCACAUGGAGGGCCGAUGUCGAUUGAGGAGUUGUGCUCGUCACAACGGGAUGACAGGAGCUGGA
env-293   ...............................GAUGUCGAUAGGAGUUGUGCUCGUCACAACGGGAUGACAGGAGCUGGA
env-295   CACAAAGGUCGAACCCUCCACAUGGAGGGCCGAUGUCGAUAGGAGUUGUGCUCGUCACAACGGGAUGACAGGAGCUGGA
env-296   CACAAAGGUCGAACCCUCCACAUGGAGGGCCGAUGUCGAUAGGAGUUGUGCUCGUCACAACGGGAUGACAGGAGCUGGA
Mbo-1-1   CGCAAAGGUCGAACCCUCCACAUGGAGGGCCGAUGUCGAUAGGAGUUGUGCUCGUCACAACGGGAUGACAGGAGCUGGA
env-297   CACAAAGGUCGAACCCUCCACAUGGAGGGCCGAUGUCGAUAGGAGUUGUGCUCGUCACAACGGGAUGACAGGAGCUGGA
env-298   ........................................AGGAGUUGUGCUCGUCACAACGGGAUGACAGGAGCUGGA
env-303   CAAAGGUCGAACCCUCCACAUGGAGGGCCGAUGUCGAUUGAGGAGUUGUGCUCGUCACAACGGGAUGACAGGAGCUGGA
env-304   CGCAAAGGUCGAACCCUCCACAUGGAGGGCCGAUGUCGAUAGGAGUUGUGCUCGUCACAACGGGAUGACAGGAGCUGGA
env-305   UCGAAGGUCGAACCCUCCAGAUGGAGGGCGAUAUCGAUUGAGGAGUUGUGCUCGUCACAACGGGAUGACAGGAGCUGGA
env-306   ..............................AUAUCGAUUGAGGAGUUGUGCUCGUCACAACGGGAUGACAGGAGCUGGA
Mma-1-1   GACAAGGUCGAACCCUCCUCACGGAGGGCCGAUGUCGAUAUGGAGUUGUGCUCGUCACAACGGGAUGACAGGAGCUGGA
Mse-1-1   CACAAGGUCGAACCCUCCUCACGGAGGGCCGAUGUCGAUAUGGAGUUGUGCUCGUCACAACGGGAUGACAGGAGCUGGA
Msp-1-1   GACAAGGUCGAACCCUCCUCACGGAGGGCCGAUGUCGAUAUGGAGUUGUGCUCGUCACAACGGGAUGACAGGAGCUGGA
env-310   AGCAGGUCGAACCCUCCUUGCGGAGGGGCGACGUCGAUUGAGGAGUUGUGCUCGUCACAACGGGAUGACAGGAGCUGGA
env-315   AGCAGGUCGAACCCUCCUUGCGGAGGGGCGACGUCGAUUGAGGAGUUGUGCUCGUCACAACGGGAUGACAGGAGCUGGA
env-316   ....GGUCGAACCCUCCUUGCGGAGGGGCGACGUCGAUUGAGGAGUUGUGCUCGUCACAACGGGAUGACAGGAGCUGGA
env-317   .......................................GAGGAGUUGUGCUCGUCACAACGGGAUGACAGGAGCUGGA
env-318   AGCAGGUCGAACCCUCCUUGCGGAGGGGCGACGUCGAUUGAGGAGUUGUGCUCGUCACAACGGGAUGACAGGAGCUGGA
env-320   AGCAGGUCGAACCCUCCUUGCGGAGGGGCGACGUCGAUUGAGGAGUUGUGCUCGUCACAACGGGAUGACAGGAGCUGGA
env-321   .........AACCCUCCUUGCGGAGGGGCGACGUCGAUUGAGGAGUUGUGCUCGUCACAACGGGAUGACAGGAGCUGGA
env-322   AGCAGGUCGAACCCUCCUUGCGGAGGGGCGACGUCGAUUGAGGAGUUGUGCUCGUCACAACGGGAUGACAGGAGCUGGA
Mpa-1-1   AUACGGUCGCACCCGACCCUGAUGGUCGGGAAUGCCGAGAGGAGUUGUGAGCGAUCACAACGGGCUGACAGAAGCUGGA
env-323   ..............................AAUGCCGAGAGGAGUUGUGAGCGAUCACAACGGGCUGACAGAAGCUGGA
env-324   UUGAUUGGUGAACUCAUCAGUAACCUGAUGAGAUCCCGAGAAGGAGUUGUGAUUCGUCACAACAGGCUGACAGAGUGGG
env-325   CUCGAUUGGUGAACCCUCCUUCAGGAGGGGUAUACCGAGAAGGAGUUGUGAUCCGUCACAACAGGCUGACAGAGUAGGA
env-326   CUCGAUUGGUGAACCCUCCUUCAGGAGGGGUAUACCGAGAAGGAGUUGUGAUCCGUCACAACAGGCUGACAGAGUAGGA
env-331   CUCGAUUGGUGAACCCUCCUUCAGGAGGGGUAUACCGAGAAGGAGUUGUGAUCCGUCACAACAGGCUGACAGAGUAGGA
env-332   CUCGAUUGGUGAACCCUCCUUCAGGAGGGGUAUACCGAGAAGGAGUUGUGAUCCGUCACAACAGGCUGACAGAGUAGGA
Mfo-1-1   CGAUUGGUGGAUCCCCGUAUAACAACGGGGGUAUCCCGAGAAGGAGUUGUGAUUCGUCACAACAGGCUGACAGAGUGGG
env-333   CGAUUGGUGACCCCCGCAGACAGUGCGGGGGAUCCCGAGAAGGAGUUGUGAUCCGUCACAACGGGCUGACAGAGUGGGA
env-334   GAUUGGUGACCCCUGCAGAAAUAUGCGGGGGAUCCCGAGAAGGAGUUGUGAUCCGUCACAACGGGCUGACAAAGUGGGA
env-335   GAUUGGUGACCCUCGCAGGAAUCUGCGGGGGAUCCCGAGAAGGAGUUGUGAUCCGUCACAACGGGCUGACAAAGUGGGA
env-337   GAUUGGUGACCCCCGCAGAAAUAUGCGGGGGAUCCCGAGAAGGAGUUGUGAUCCGUCACAACGGGCUGACAAAGUGGGA
env-338   CGAUUGGUGACCCCCGCAGACAUUGCGGGGGAUCCCGAGAAGGAGUUGUGAUCCGUCACAACGGGCUGACAGAGUGGGA
env-339   CGAUUGGUGACCCCCGCACACAAUGCGGGGGAUCCCGAGAAGGAGUUGUGAUCCGUCACAACGGGCUGACAGAGUGGGA

          ...............................................................................
          ...............................................................................
          •••••••••••••••••••••••••••••••••••••RR•••RRR•••••••••••••RRR•••GY•R••RRRGY•GGA
```

CMe-1-1	CGAUUGGUGACCUCCUCAUUUCGUGAGGAGGAUCCCGAGAAGGAGUUGUGAUCCGUCACAACGGGCUGACAGAGUUGGA
env-340	GAUUGGUGAACCUCGCAGAAAUGCGGGGGGCAACCCGAGAAGGAGUUGUGAUUCGUCACAACAGGCUGACAGAGUAGGA
env-341	GAUUGGUGAACCCCGCAUACAGGUGGGGGUCAACCCGAGAAGGAGUUGUGUAUCGUCACAACAGGCUGACUGAGUGGGA
env-349	....GAGUGGGA
env-350	CGAUUGGUGAACUCCGCGUUAUUGCGGGGGAAUCCCGAGUAGGAGUUGUGAUUAAUCACAACAGGCUGACUGAGUAGGA
env-351	GAUUGGUGAACCUCGCAGCAAUGCGGGGGGCAACCCGAGAAGGAGUUGUGAUUCGUCACAACAGGCUGACAGAGUAGGA
env-352	CCUUGAUUGGUGAACUCAUCCUAACGGAUGGGAUCCCGAGAAGGAGUUGUGAUUCGUCACAACAGGCUGACAGAGUGGG
env-353	GAUUGGUGAACUCCGCUUAAUUGCGGGGGGAAACCCGAGAAGGAGUUGUGACUCGUCACAACAGGUUGACUGAGUGGGA
env-354	GAUUGGUGAACUCCGCUUAAUUGCGGGGGGAAACCCGAGAAGGAGUUGUGACUCGUCACAACAGGUUGACUGAGUGGGA
env-355	GAUUGGUGAACUCCGUUUAAUUGCGGGGGGAAACCCGAGAAGGAGUUGUGACUCGUCACAACAGGUUGACUGAGUGGGA
env-356	CGAUUGGUGGACUCCGCUUAAUUGCGGGGGAAACCCGAGAAGGAGUUGUGACUCGUCACAACAGGUUGACUGAGUGGGA
env-357	GAUUGGUGAACUCCGCUUCAUNGCGGGNGGAAACCCGAGAAGGAGUUGUGACUCGUCACAACAGGCUGACUAAGUGGGA
env-358	CGAUUGGUGAACUCCGCAUCAGAGCGGAGGAAACCCGAGAAGGAGUUGUGACUCGUCACAACAGGCUGACUAAGUGGGA
env-359	CGAUUGGUGAACUCCGCAUCAGAGCGGAGGAAACCCGAGAAGGAGUUGUGACUCGUCACAACAGGCUGACUAAGUGGGA
env-360	CGAUUGGUGAACUCCGCAUCAGAGCGGAGGAAACCCGAGAAGGAGUUGUGACUCGUCACAACAGGCUGACUAAGUGGGA
env-361	CGAUUGGUGAACUCCGCAUCAGAGCGGAGGAAACCCGAGAAGGAGUUGUGACUCGUCACAACAGGCUGACUAAGUGGGA
env-370	GAUUGGUGAACUCCGCUUCAUUGCGGGGGGAAACCCGAGAAGGAGUUGUGACUCGUCACAACAGGCUGACUAAGUGGGA
env-371	....GUUGUGACUCGUCACAACAGGCUGACUAAGUGGGA
env-372	....GGCUGACUGAGUGGGA
env-373	GAUUGGUGAACCCCGCAUACAAGCGGGGGUCAACCCGAGAAGGAGUUGUGUAUCGUCACAACAGGCUGACUGAGUGGGA
env-374	CGAUUGGUGAACCCCGCCUCAUUGCGGGGGAAACCCGAGAAGGAGUUGUGAUUCGUCACAACAGGCUGACUACGUGGGA
env-375	....UCAAUUGGUGCCCUCUCCGAGAAGGUGAGGACCGAGAAGGAGUUGUGAUUCGUCACAACAGGCUGACAGAGUGGG
env-376	....UCACAACGGGCUGACAGAGUGGG
env-377	CCUCAAUUGGUGCCCUCUCCGCAAGGUGAGGUAUACCGAGAAGGAGUUGUGAUUCGUCACAACAGGCUGACAGAGUGGG
env-378	UCGAUUGGUGCCCCUCUUAAAAAUGGGAGGGUAAACCGAGAAGGAGUUGUGAUUCGUCACAACAGGCUGACAGAGUGGG
env-379	....GAAGGAGUUGUGAUUCGUCACAACAGGCUGACUGAGUGGG
env-380	CUCGAUUGGUGACCCCUCCUUAUGGAGGGCUGAACCGAGAAGGAGUUGUGAUUCGUCACAACAGGCUGACAGAGUAGGA
env-381	CUCGAUUGGUGACCCCUCCUUAUGGAGGGCUGAACCGAGAAGGAGUUGUGAUUCGUCACAACAGGCUGACAGAGUAGGA
env-382	CUCGAUUGGUGACCCCUCCUUAUGGAGGGCUGAACCGAGAAGGAGUUGUGAUUCGUCACAACAGGCUGACAGAGUAGGA
env-383	CUCGAUUGGUGACCCCUCCUUAUGGGGGGCUGAACCGAGAAGGAGUUGUGAUNCGUCACAACAGGCUGACAGAGUAGGA
env-384	CUCGAUUGGUGACCCCUCCUUAUGGAGGGCUGAACCGAGAAGGAGUUGUGAUUCGUCACAACAGGCUGACAGAGUAGGA
env-385	..................CUUACGGAGGGCUAAACCGAGAAGGAGUUGUGAUCCGUCACAACGGGCUGACAGAGUAGGA
env-386	CUCGAUUGGUGAACCCUCCUUACGGAGGGCUAAACCGAGAAGGAGUUGUGAUCCGUCACAACGGGCUGACAGAGUAGGA
env-387	CUCGAUUGGUGAACCCUCCUUACGGAGGGCUAAACCGAGAAGGAGUUGUGAUCCGUCACAACGGGCUGACAGAGUAGGA
env-389	CUCGAUUGGUGACCCCUCCUUACGGAGGGUUAAACCGAGAAGGAGUUGUGAUUCGUCACAACAGGCUGACAGAGUAGGA
env-390	CGAUUGGUGAACCCCGUGUAAACACGGGGUCAACCCGAGAAGGAGUUGUGAUUCGUCACAACAGGCUGACUGAGUGGGA
env-392	CCCGAUUGGUGGAUCCCCCGUAUGGGGGGGUAUCCCGAGAAGGAGUUGUGAUUCGUCACAACAGGCUGACAGAGUGGG
env-395	...................AAAAAGGAUGGGUAUCCCGAGAAGGAGUUGUGAUUCGUCACAACAGGCUGACCGAGUGGG
env-399	CUCGAUUGGUGAACCUUCCUUUAGGAAGGGUAUACCGAGAAAGGAGUUGUGAUUCGUCACAACGGGCUGACAGAGUGGG
env-400	....UCACAACAGGCUGACAGAGUAGGA
env-402	....UCACAACGGGCUGACAGAGUGGG
env-403	CUCGAUUGGUGCCUCUCCCCACAAGGAGGGGUAAACCGAGAAGGAGUUGUGAUUCGUCACAACGGGCUGACAGAGUGGG
env-405	..............CCCGCCUCACCGCGGGUAUACCGAGAAGGAGUUGUGCAUCGUCACAACAGGCUGACAGAGUGGGA
env-406	CUUCGAUUGGUGAACCCGCCUCACCGCGGGUAUACCGAGAAGGAGUUGUGCAUCGUCACAACAGGCUGACAGAGUGGGA
env-407	GAUUGGUGAACUCCGCAGGAAUACGGAGGGCAACCCGAGAAGGAGUUGUGAUUCGUCACAACAGGCUGACAGAGUUGGA
Mli-2-1	AUGUGGUUGCCCCGGGUAUUUAUGCCGGGAUUUGCCGAGAUUAAUGUCCGGUUACGCCGGACGGGCUGACGAUGUAGAA
Mpe-1-1	CAACGUAAUCGCUGUCCGGAGAUUGCCGAGAAAGGACAAAAAGCCUAUGAAGGCUUUCUGUUCGGCUGACAAAGUAGGA
Mpa-2-1	CAACGUAAUCGCUGUCCGGAGAUUGCCGAGAAAGGACAAAAAGCCUAUGAAGGCUUUCUGUUCGGCUGACAAAGUAGGA
env-408	ACAACAUUUUUGCUGUCCGGAGAUUGCCGAGAAUGGACUGAAAGCCGCAGAGGCUGCAAGUCCGGCUGACAAAGUAGGA
Mmo-1-1	UUUCCGGUGAUUGCCGAGAUAGUAGGUCACACAAAAUUCCGACGGGAAUUUACAAGUGUGACGGGCUGACAACGUAGGA
env-409	UUUCCGGUGAUUGCCGAGAUAGUAGGUCACACAAAAUUCCGACGGGAAUUUACAAGUGUGACGGGCUGACAACGUAGGA
env-410	GAUCAACCGGCUCUGCUACGUAAGGUAGUAUAGCGGGUGAAUGCUUGCGACAAUUCGCAGGCCGACGACCACUAGCAGG
Mco-1-1	CGGAGUCAUCCCGCCACUGAGGAUACCAUCUCAUACUAGCGAAGAGGAAUACCGAGAAGGUAUUGUGACCAAAGUCGGC
env-413	GAACCUCAACGAAGGAGAAUUCCGGGUUGGGGUAGUGGUCCCCGGACCGCAAAAAAAGAAACGCGAGAAUAAAGUCGGA
env-414	GAACCUCAACGAAGGAGAAUUCCGGGUUGGGGUAGUGGUCCCGGGACCGCAAAAAAAGAAAAGCGAGAAAAAAGUCGGA
env-415	GAACCUCAACGAAGGAGAAUUCCGGGUUGGGGUAGUGGUCCCAGGACCGCAAAAAAAGAAAAGCGAGAAAAAAGUCGGA
env-416	GAACCUCAACGAAGGAGAAUUCCGGGUUGGGGUAGUGGUCCCGGGACCACACAAAAAGAAAAGCGAGAAAAAAGUCGGA
env-418	ACACAUCAACGUAGGAGAAUACCGCGUUGGAGUAGUGGUUUCACAACCAAAAAAAAAAAUUAGCGAGAAAAAAGUAGGA
env-423	ACACAUCAACGUUGGAGAAUACCGCGUUGGAGUAGUGGUUCCACAACCAAAAAGAAAAAACAGCGAGAAAGAAGUAGGA
env-425	ACACAUCAACGUAGGAGAAUACCGCGUUGGAGUAGUGGUUAGACAACCAGGAAAAAAAAACAGCGAGAAAAAAGUAGGA
env-428	UCAUCAUCCCAUAAACAAAAUGACAUCAGCGCUCAAGAUCGGCGAGUACCGAGUCGGAGCAGUCGUCGAAAGCGUCGGA
env-429	UCAUCAUCCCAUAAACAAAAUGACAUCAGCGCUCAAGAUCGGCGAGUACCGAGUCGGAGCAGUCGUCGAAAGCGUCGGA
Msh-1-1	GCCACACCAUCCGACGACAUCGGACACAUCGAAAACGAACAUCGGAGACAUCCGAACAGGUGUAGUGACCAAGGUAGGA
env-430	UCAUCAUCCCAUCAACUCAAAAUCAGAAUCGCUCAAGAUUGGCGAGUUUCGCGUCGGAGCAGUCGUUGAAAGCGUCGGA
env-433	ACAUCAUCCCAUAAACUCAAAAUCAGAAUCGCUCAAGAUUGGCGAGUUUCGCGUCGGAGCAGUCGUUGAAAGCGUCGGA
env-434	....AGCAGUCGUUGAAAGCGUCGGA
env-435	UCAUCAUCCCAUCAACUCAAAAUCAGAAUCGCUCAAGAUUGGCGAGUUUCGCGUCGGAGCAGUCGUUGAAAGCGUCGGA
env-436	CAUCAUCCCAUCAACUCAAAAAUCAGAAUCGCUCAAGAUUGGCGAGUUUCGCGUCGGAGCAGUCGUUGAAAGCGUCGGA
env-437	UCAUCAUCCCAUCAACUCAAAAUCAGAAUCGCUCAAGAUUGGCGAGUUUCGCGUCGGAGCAGUCGUUGAAAGCGUCGGA
env-439	UCAUCAUCCCAUCAACUCAAAAUCAGAAUCGCUCAAGAUUGGCGAGUUUCGCGUCGGAGCAGUCGUUGAAAGCGUCGGA
env-440	NCAUCAUCCCAUNAACUCAAAAUCAGAAUCGCUCAAGAUUGGCGAGUUUCGCGUCGGAGCAGUCGUUGAAAGCGUCGGA
env-441	UCAUCAUCCCAUCAACUCAAAAUCAGAAUCGCUCAAGAUUGGCGAGUUUCGCGUCGGAGCAGUCGUUGAAAGCGUCGGA
env-442	....CAUCCCAUCAACUCAAAAUCAGAAUCGCUCAAGAUUGGCGAGUUUCGCGUCGGAGCAGUCGUUGAAAGCGUCGGA
env-443	UCAUCAUCCCACAAACUCAAAAUCAGAAUCGCUCAAGAUUGGCGAGUUUCGCGUCGGAGUUGUCGUUGAAAGCGUCGGA
env-444	UAAUCAUCCGUUGAACUCAAAAACAUCAGCGCUCAAAAUUGGCGAGUACCGAGUCGGAGCAGUCGUCGAAAGCGUCGGA
env-445	UCAUCAUCCCAUAAACUCAAGAUCAGAAUCGCUCAAGAUUGGCGAGUUUCGCGUCGGAGCAGUCGUUGAAAGCGUCGGA
env-446	UCAUCAUCCAUUAAACUCAAGAUCAGAAUCGCUCAAGAUUGGCGAGUUUCGCGUCGGAGCAGUCGUUGAAAGCGUCGGA
env-448	CCAUCAUCCUGUGAACUCAGAGAGCACAACGCUCAGAGUUGGCGAGUUCAGAGUCGGAGCAGUCGUCGAAAGCGUCGGA
env-449	UCAUCAUCCAAUCAACUCAAAGACGUCAGCGCUCAAGAUCGGCGAGUACCGAGUCGGAGCAGUCGUCGAAAGCGUCGGA
env-450	.................CAAAAUCAGAAUCGCUCAAGAUUGGCGAGUUUCGCGUCGGAGCAGUCGUUGAAAGCGUCGGA
env-451	UCAUCAUCCAAUAAACUCAAAAUCGGAAUCGCUCAAGAUUGGCGAGUUUCGCGUCGGAGCAGUCGUUGAAAGCGUCGGA
env-452	UCAUCAUCCAAUAAACUCAAAAUCGGAAUCGCUCAAGAUUGGCGAGUUUCGCGUCGGAGCAGUCGUUGAAAGCGUCGGA
env-455	UCAUCAUCCCAUAAACUCAAAAUCGGAAUCGCUCAAGAUUGGCGAGUUUCGCGUCGGAGCAGUCGUUGAAAGCGUCGGA
env-456	...............CUCAAAAUCAGAAUCGCUCAAGAUUGGCGAGUUUCGCGUCGGAGCAGUCGUUGAAAGCGUCGGA
	....
	....
	•••••••••••••••••••••••••••••••••••••RR•••RRR•••••••••••••RRR•••GY•R••RRRGY•GGA

```
env-460  UCAUCAUCCCAUCAACUCAAAAUCAGAAGCUCUCAAGAUCGGCGAGUUUCGCGUCGGAGCAGUCGUUGAAAGCGUCGGA
env-462  UCAUCAUCCCAUCAACUCAAAAUCAGAAGCUCUCAAGAUCGGCGAGUUUCGCGUCGGAGCAGUCGUUGAAAGCGUCGGA
env-464  ................................................UCGCGUCGGAGCAGUCGUUGAAAGCGUCGGA
env-465  UCAUCAUCCCAUAAACUCAAAAGCAGAAGCUCUCAAGAUCGGCGAGUUUCGCGUCGGAGCAGUCGUUGAAAGCGUCGGA
env-466  UCAUCAUCCAUUAAACUCAAAAUCAGAAUCGCUCAAGAUUGGCGAGUUUCGCGUCGGAGCAGUCGUUGAAAGCGUCGGA
env-467  UCAUCAUCCCACAAGCUCAAAGGUUUCAACGCUCAAGGUCGGCGAGUUUCGAGUAGGUUUGGUAGUCGAUGAAGUGGGA
env-468  UCAUCAUCCCACAAGCUCAAAGGUUUCAACGCUCAAGGUCGGCGAGUUUCGAGUAGGUUUGGUAGUCGACGAAGUGGGA
env-469  UCAUCAUCCCACAAGCUCAAAGGUUUCAACGCUCAAGGUCGGCGAGUUUCGAGUAGGUUUGGUAGUCGACGAAGUGGGA
env-471  UCAUCAUCCCACAAGCUCAAAGGUUUCAACGCUCAAGGUCGGCGAGUUUCGAGUAGGUUUGGUAGUCGACGAAGUGGGA
env-472  UCAUCAUCCCACAAGCUCAAAGGUUUCAACGCUCAAGGUCGGCGAGUUUCGAGUAGGUUUGGUAGUCGACGAAGUGGGA
env-474  UCAUCAUCCCACAAGCUCAAAGGUUUCAACGCUCAAGGUCGGCGAGUAUCGAGUAGGUUUAGUAGUCGAUGAAGUGGGA
env-475  UCAUCAUCCCAAAAGCUCAAAGGUUUCAACGCUCAAGGUCGGCGAGUAUCGAGUAGGUUUAGUAGUCGACGAAGUGGGA
Mha-1-1  UCAUCAUCCCAAAAGCUCAAAGGUUUCAACGCUCAAGGUCGGCGAGUAUCGAGUAGGUUUAGUAGUCGACGAAGUGGGA
env-476  UCAUCAUCCCACAAGCUCAAAGGUUUCAACGCUCAAGGUCGGCGAGUAUCGAGUAGGUUUAGUAGUCGACGAAGUGGGA
env-477  UCAUCAUCCCACAAGCUCAAAGGUUUCAACGCUCAAGGUCGGCGAGUUCAGAGUAGGUUUAGUAGUCGAUGAAGUGGGA
env-478  UCAUCAUCCGCUGAACUCAAAGUCGUCAGCGCUCAAGGUCGGCGAGUUCAGAGUCGGAGCUGUAGUCGAAAGCGUCGGA
env-479  ACGCACAGCACAUCAUCCCAAGGUUUCAACGCUCGAAGUCGGCGAGUUCAGAGUAGGAUUGGUUGUUGACGACGUCGGA
env-480  ...................GAGGUCACAACGCUCGAAGUCGGCGAGUUCAGAGUAGGAUUGGUUGUUGACGACGUCGGA
env-481  CCCCUUACGAACGCCACAUCAUCCAACAAGCUCAUCCGGGGUCGGAGAAAUCCGUCCCGGCGUGGUGACAAAAGUGGAG
env-482  CCCUCACCAUCCGGUAGGUUCCAGUGUAGAAAGCCUUUCCACUGGUGAAAUCAGGGAAGGCGUGGUAAGAAAGGUAGGG
env-483  ........................................CAAGAUAACGUCCGUGAUGGAAUUGUGAUAGACGUCGGA
env-484  GGUUGUCCCCCCUUUAAAAACCCGUCAUCAUGUGCACGAGCAAGAUAACGUCCGUGAUGGAAUUGUGAUAGACGUCGGA
env-485  GGUUGUCCCCCCUUUAAAAACCCGUCAUCAUGUGCACGAGCAAGAUAACGUCCGUGAUGGAAUUGUGAUAGACGUCGGA
env-486  GGUUGUCCCCCCUUUAAAAACCCGUCAUCAUGUGCACGAGCAAGAUAACGUCCGUGAUGGAAUUGUGAUAGACGUCGGA
env-492  GGUUGUCCCCCCUUUAAAAACCCGUCAUCAUGUGCACGAGCAAGAUAACGUCCGUGAUGGAAUUGUGAUAGACGUCGGA
env-495  GGUUGUCCCCCCGUUGAAGACCCGCCAUCAUGUGCACGAGUAUGAUAACGUUCGUGAUGGAAUUGUGAUAGACGUCGGA
env-504  GGUGGUUCCCCCGUUGAAGACCCCUCAUCACGCGCGGCCGCGUGAUGACAUUCGAGAAGGAAUUGUGAUAGACGUCGGA
env-505  UGACGGUCAAAUCGCAGAAAAAUAAAAUUUCUGCGGAGUGACAGGAUUGCGUUCAUCGCAAUGGGCUGAUAUAGUGGGA
env-508  GUCGGGGGCACGGGUCCCGGAUGGCCCGUGCCUUUGUAUGAGUAUGUGGUGAGCAAUCACCACCGGACGACACGGUGGA
env-509  GUCAGGGACAUGCGUUCCAGGCAACGCGUGUCUUUGCGUGAGAAUGUGGUGCAUGGUCACCACCGGCUGACACGGUGUA
Mta-1-1  GGUCAGGGACACGGGUUCCAGGAACGCGUGUCUUUGCGUGAGAAUGUGGUGAAUGGUCACCACCGGCUGACAGGGUGUC
env-511  GGUCAGGGACACGGGUUCCAGGAACGCGUGUCUUUGCGUGAGAAUGUGGUGAAUGGUCACCACCGGCUGACAGGGUGUC
env-512  GGUCAGGGACACGGGUUCCAGGAACGCGUGUCUUUGCGUGAGAAUGUGGUGAAUGGUCACCACCGGCUGACAGGGUGUC
Hbo-1-1  CCUGCUCGUUUCGUCUACGACCGGCUCCGAAUCAGACGAUUCGGGGUCGUUACAACAGGGAAUCGUGACCGAGGUCGGA
Ngr-1-1  GCUCCGCGCCACGUCACAGACCGGCUCCGAAUCGAACGGUUCGGGGUCGACAAGACAAGGGAUCGUGACCGAGGUCGGA
Hje-1-1  GCUUCGCGCCGUGUCACGGACCGGCUCCGGAUCGAACGGUUCGGGGUCGUUAAGACAGGGACUCGUGACCGAGGUCGGA
Nma-1-1  ACUCCGCGCCACGUCACAGACCGGCUCCGAAUCAGACGGUUCGGGGUCGUUAAGACAAGGGAUCGUGACCGAGGUCGGA
Hal-1-1  CGUUCUCGUCGCGUCUACGACCGCGGACGACUCCGACGAGUCGCCCGCGUUGCGAGAAGGAAUCGUGACCGAGGUCGGA
Nti-1-1  GCUCCGCGCCAUGUCACAGACCGGCUCCGAAUCUGACGGUUCGGGGUCGUCAAGACAAGGAAUCGUGACCGAGGUCGGA
Npe-1-1  GCUCCGCGCGGUGUCACAGACCGGCUCCGAAUCGAACGGUUCGGGGUCGUCAAGACAAGGGAUCGUGACCGAGGUCGGA
Hti-1-1  GCUCCGCGUUGCGACCCAGACCGGCUCCGGAUCGGACGAUUCGGGGUCGUUAAGACAGGGAAUCGUGACCGAGGUCGGA
         ...............................................................................
         ...............................................................................
         ●●●●●●●●●●●●●●●●●●●●●●●●●●●●●●●●●●●●●RR●●●RRR●●●●●●●●●●●●●RRR●●●GY●R●●RRRGY●GGA
```

alignment positions 80 · · · 259

```
Mli-1-1  UCUGAU...CCGUGU.AAU..GGGCCUCC...GCC......CGUGCA.........GGC.GAGCCCGG....CCGGC........................CCCGU........................................G.CCGGCAU...GCACAACG.......AUCAGGA
env-1    UGUGAU...GAUAGC.AA...GGGUUGAA..AGCCA.....UCUG..........UGGCUUUGCCCGUG...UCUGCA....UGAAGG.............AAAUA......................CCU.CU............G.UAGACCU...GCUGAAAC.......GUCACGC
env-2    UCUGAC.UACUGUGCUA....GGGUGGAU...AUUG.....GCCU..........UGAU.GAGCCAGC....CCUCC.....UCGUUGAAAAU........GAAGAA................GUU...AGCGA............G.GAAGAAC...GCAUAACU.......GUCAGAA
env-3    UCUGAU.UACUGCGC.AA...GGGUGUAUAUAGGUC.....UUGACC........GACC.A.GCCCUU....CUCGC.....UGAUU..............GUGAAG......................GAUUA...........AG.CGAGAAACA.GCGCAUAACU.....GUCAGGA
env-4    GCUGAG.GCAGCAGC.A....GGGUGGAC...GUC......AUG............GAC.G.GCCCU.....GCCCG........................CACGA........................................C.GGGCGCU...GCUGCA.........UUCAGGA
env-5    GCUGAG.GCAGCAGC.A....GGGUGGAC...GUC......AUG............GAC.G.GCCCU.....GCCCG........................CACGA........................................C.GGGCGCU...GCUGCA.........UUCAGGA
env-6    UGUGAU..GACGGC..AA...GGGUGGAC...G........AUG..............C.G.GCCCGA....AUCC.........................UUAACC.........................................GGAUAA.....GCCGGAGA......AUCACGC
env-8    UGUGAU...GUCGGC.AA...GGGUGGAC...G........AUG..............C.G.GCCCGA....GUCCU........................GGUAAC.......................................A.GGAUAA....GCCGAAAG.......AUCACGC
env-10   UGUGAU...GUCGGC.AA...GGGUGGAC...G........AUG..............C.G.GCCCGG....AUCCU.....G..................GUUU............................C............A.GGAUAA....GCCGAAGU.......AUCACGC
env-11   UGUGAU...GUCGGC.AA...GGGUGGAC...G........AUG..............C.G.GCCCGG....AUCCU.....G..................GUUU............................C............A.GGAUAA....GCCGAAGU.......AUCACGC
env-13   UCUGAA.AACGGCAC.AAC..GGGUGGAC...GUAAUCU..UUGA.......AGA.UAC.GAGCCCUC....AUCCGG....CGGA...............UCAA.........................UCCG...........GC.GGAUAU...GUGCCCGG.......UUCAGGA
env-14   UCUGAC.GAAUCCGC.G....GGGUGGAC...GCACG....AAC..........CGUGC.GAGCCCAG....UUCUG.....GUUAU..............AUCGG.......................AUA.C............C.GGAA......GCGGAGAUAGUAACCGUCAGGA
env-15   UCUGAU.GACCGCGUCC....GGGUCAAU...UGUG.....AAC...........UGCA.ACACCCAA....UUUCU.....CUCCAUGUACCAGAC....UCGAU.............GUCUG.UGC...GAG...........AA.GGGAC.....ACGCGUUGCC.....AUCAGAA
Hwa-1-1  CCUGAU.GACCGCGUUC....GGGUCAAU...UGUG.....AAU...........UGCA.ACACCCGG....UCUCU.....CUCC.AU..GUGCC.....AGAAUC.............GGUAUCUGUACGAG...........AG.GGGAC.....ACGCGUUGCU.....AUCAGGA
env-17   CCUGAU.GACCGCGUUC....GGGUCAAU...UGUG.....AAU...........UGCA.ACACCCGG....UCUCU.....CUC...CAUGUGCC.....AGAAUC.............GGUAUCUGUACGAG...........AG.GGGAC.....ACGCGUUGCU.....AUCAGGA
env-23   CCUGAU.GAUCGCGUCC....GGGUCAAU...UGCGC....CCUC.........GCGCA.ACACCCGA....UCUCU.....UUGCUCGUGCCCGACG...GCC..............UGUCGG.....UCCGA.........AAGG.GAGA......GCGCGUCACA.....CUCAGGG
env-24   ACUGAC.GGACGCGUCU....GGGUCAAU...UGCG.....GAA...........CGCA.ACACCCGG....UCGCCC....UUCGC..GUCCCC......GAUGAG..............GGGGAUACGGGAG............G.GGGA......ACGCGUCACC.....CUCAGAG
env-25   GCUGAU.GGGCGCGUCC....GGGUCAAU...UGCG.....GUC...........UGCA.ACACCCAA....UCUCG.....CUA.CCGG.UGCCGG....AUGGA.............CUGGCAGCCGGCGAG............C.GUGA......GCGCGUCACC.....GUCAGGG
env-27   UCUGAA.GGCCGCGUCU....GGGUCAAU...UGCG.....ACA...........UGCA.ACACCCAC....UCGCC.....CUCAUC......GCCCCACCUGAG.........GU.GGGCACACUGGAUGAG............G.GCGA......CCGCGUCACC.....AUCAGGA
env-28   CCUGAC.AACCGCGUCC....GGGUCAAU...UGCG.....GCU...........UGCA.ACACCCAA....UCUCG.....CUCCCCGUUGGACCA....GAAA..............UGG.CCAUAAAGGAG............G.GAGA......GCGCGUAACA.....GUCAGGA
env-29   CCUGAC.AACCGCGUCC....GGGUCAAU...UGCG.....GCU...........UGCA.ACACCCAC....UCUCG.....CUCCCCGUUGGGCCA....GAAA..............UGGCCC.UCACAGAG............G.GAGA......ACGCGUGACA.....GUCAGGA
env-30   CCUGAC.AACCGCGUCC....GGGUCAAU...UGCG.....GCU...........UGCA.ACACCCAA....UCUCG.....CUCCCCGUUGGACCA....GAAA..............UGGCCC.UCGAGGAG............G.GAGA......GCGCGUAACA.....GUCAGGA
env-31   GCUGAU.GGGCGCGUCC....GGGUCAAU...UGCG.....GUA...........UGCA.ACACCCGA....UCUCU.....CU...CCACGUUCCAGA..CAG.............UCUGGA.CGUGGCUCAG............G.GAGA......GCGCGUGACC.....GUCAGGG
Hut-1-1  UCUGAU.GGGCGCGUCC....GGGUCAAU...UGCG.....GCA...........UGCA.ACACCCGAU....CUCC.....CUC....GCCGUUCCGG..GCGA............CCGGA.CGAUAAAGGAG............G.GGGA......ACGCGUCACC.....GUCAGGA
env-32   UCUGAA.GGCCGCGUCU....GGGUCAAU...UGCG.....AAC...........UGCA.ACACCCAC....UCGCC.....CUCAUC..GC...CCCACCCGA...........GGUGGGACCGCUGGAUGAG............G.GCGA......CCGCGUCACC.....AUCAGGA
env-33   UCUGAA.GGCCGCGUCU....GGGUCAAU...UGCG.....AUC...........UGCA.ACACCCAC....UCGCC.....CUCAUC.GC....UCCACCCGA...........GGUGGGACCGGUGGAUGAG............G.GUGA......CCGCGUCACC.....AUCAGGA
env-34   UCUGAA.GGCCGCGUCU....GGGUCAAU...UGCG.....AGC...........UGCA.ACACCCAC....UCGCC.....CUCAUC..GC...CCCACCUGA...........GGUGGGACCGCUGGAUGAG............G.GCGA......CCGCGUCACC.....AUCAGGA
env-35   UCUGAA.GGCCGCGUCU....GGGUCAAU...UGCG.....AUC...........UGCA.ACACCCAC....UCGCC.....CUCAUC.GC....CCCACCCGA...........GGUGGGACCGGUGGAUGAG............G.GUGA......CCGCGUCACC.....AUCAGGA
env-36   GCUGAU.GGCCGCGUAC....GGGUCAAU...UGCG.....GAC...........UGCA.ACACCCGG....UCUCC.....CU..GCCGGUCCCGUCCG.GAA............UGGACG.AACCGGUCGAG............G.GGGA......ACGCGUCUCC.....GUCAGGG
env-37   GCUGAU.GGGCGCGUCU....GGGUCAAU...UGCG.....GAC...........UGCA.ACACCCAC....UCUCU.....CUCCC.......CGUCCC.GAAC...........GGGAUGGAACUCACGGAG............G.GAGA......CCGCGUUUCC.....GUCAGGG
env-38   GCUGAU.GGGCGCGUCU....GGGUCAAU...UGCG.....GAC...........UGCA.ACACCCAC....UCUCU.....CUCCC.......CGUCCC.GGAU...........GGGAUGGAACUCACGGAG............G.GAGA......ACGCGUUUCC.....GUCAGGG
env-39   GCUGAU.GGGCGCGUCU....GGGUCAAU...UGCG.....GAC...........UGCA.ACACCCAC....UCUCU.....CUCCC.......CGUCCC.AGAU...........GGGAUGGAACUCACGGAG............G.GGGA......ACGCGUUUCC.....GUCAGGG
env-40   GCUGAU.GGGCGCGUCC....GGGUCAAU...UGCG.....GCU...........UGCA.ACACCCGA....UCUCC.....CUU..GUGGUGG.......CGGAUGGCGAU..........CCGCCGGCAAAG............G.GAGA......ACGCGUGACC.....GUCAGAA

         .<<<<<....<<<<<<.....<<<<...<...<<<<<<<<<.........>>>>>>>>>.>.>>>>......<<<<<.....<<<<<<<<<<<<<<<<<<<..............>>>>>>>>>>>>>>>>>>>............>.>>>>.....>>>>>>..........>>>>>..
         .20.......22222......001...2...22221110...........01112222.2.100.......22222.....222222..2.2.1111110..............0111111.2.2..222222............2.2222......22222...........02..
         YCUGAY-○R●●RCGY○Y----GGGU●RAY---●●○○-----●●●○----------○R●●-R○RCCCR●----UYYC●-----●●○○○--------------●●●○-----------------------○○○○○●------------R-GRRR------GCGY●●●○○------RUCAGGR
         5′                                                                                                                                                                                 3′
```

```
env-41    UCUGAA.GGCCGCGUUC....GGGUCAAU...UGUG.....AAC...........UACA.ACACCCAA....UCUCG......CUCG.UGCAAC..CGUCA.ACAA..........UGACG....CUCACUGAG............G.GAGA......ACGCGUUGCC.....AUCAGAA
env-43    ACUGAU.GGACGCGUUC....GGGUCAAU...UGCG.....GAU...........UGCA.ACACCCGA....UCUCC......CUCC.CC.GUUCCCG....AUGGC............UGGGAACUGGCCGAG............G.GGGA......GCGCGUCUCC.....GUCAGGG
env-44    CCUGAA.GGCCGCGUCC....GGGUCAAU...UGCG.....GAC...........UGCA.ACACCCGA....UCUCC......CUCCCCGUACCGGCA....GAAA.............UGUCGGU.CGAGGAG............G.GAGA......GCGCGUGACC.....GUCAGGA
env-45    CCUGAA.GGCCGCGUCC....GGGUCAAU...UGCG.....GAC...........UGCA.ACACCCGA....UCUCC......CU.GCUUG.UGCCACCA..ACGA...........UGGAGGAUCCAAGUAAG............G.GAGA......ACGCGUCACC.....GUCAGGA
env-49    UCUGAA.GGCCGCGUCC....GGGUCACU...UGCG.....GAC...........UGCA.ACACCCGA....UCUCC......CUCGC..CGUGCCGCCA..UCGA...........UGGCGACACCGGCCGAG............G.GGGA......GCGCGUCACC.....GUCAGGA
env-50    UCUGAA.GAGCGCGUUC....GGGUCACU...UGCG.....GAC...........UGCA.ACACCCGA....UCUCU......CUCCU..GGUGCCGCCG..UCGA...........UGGCGACACCCGCGGAG............G.GAGA......GCGCGUCACC.....AUCAGGA
env-54    CCUGAA.GGCCGCGUUC....GGGUCACU...UGCG.....GAC...........UGCA.ACACCCGA....UCUCC......CUCAU..GGUGCCGCCG..GCAA...........UGGCGACACCGACCGAG............G.GGGA......GCGCGUCACC.....GUCAGGA
env-55    UCUGAA.GGCCGCGUUC....GGGUCACU...UGCG.....GAC...........UGCA.ACACCCGA....UCUCC......CUCGG..CGUGCCGCCG..GAGA...........UGGCGACACCCACUGAG............G.GAGA......GCGCGUCACC.....GUCAGGA
env-56    UCUGAA.GGCCGCGUUC....GGGUCACU...UGCG.....GAC...........UGCA.ACACCCGA....UCUCC......CUCAU..GGUGCCGCCG..GAUA...........UGGCGACACCCACUGAG............G.GAGA......GCGCGUCACC.....GUCAGGA
env-57    UCUGAA.GGCCGCGUUC....GGGUCACU...UGCG.....GAC...........UGCA.ACACCCGA....UCUCC......CUUAU..GGUGCCGCCG..GAUA...........UGGCGACACCCACUGAG............G.GGGA......GCGCGUCACC.....GUCAGGA
env-58    UCUGAA.GGCCGCGUCU....GGGUCAAU...UGCG.....GAC...........UGCA.ACACCCAA....UCUCG......CUCAU..GGUGCCGCCG..AAUA...........UGGCGACACCGCAGGAG............G.GAGA......GCGCGUCACC.....GUCAGGA
env-59    UCUGAA.GGCCGCGUUC....GGGUCAAU...UGCG.....GAC...........UGCA.ACACCCGA....UCUCG......CUCAU..GGUGCCGCCG..AAUA...........UGGCGACACCCGCUGAG............G.GGGA......GCGCGUCACC.....GUCAGGA
env-62    UCUGAA.GGCCGCGUUC....GGGUCAAU...UGCG.....GAC...........UGCA.ACACCCGA....UCUCG......CUCAU..GGUGCCGCCG..AAUA...........UGGCGACACCCGCUGAG............G.GGGA......GCGCGUCACC.....GUCAGGA
env-63    UCUGAA.GGCCGCGUUC....GGGUCAAU...UGCG.....GAC...........UGCA.ACACCCGA....UCUCG......CUCAU..GGUGCCGCCG..AAUA...........UGGCGACACCCGCUGAG............G.GGGA......GCGCGUCACC.....GUCAGGA
env-64    UCUGAA.GGCCGCGUUC....GGGUCAAU...UGCG.....GAC...........UGCA.ACACCCGA....UCUCG......CUCAU..GGUGCCGCCG..AAUA...........UGGCGACACCCGCUGAG............G.GGGA......GCGCGUCACC.....GUCAGGA
env-65    UCUGAA.GGCCGCGUUC....GGGUCAAU...UGCG.....GAC...........UGCA.ACACCCGA....UCUCG......CUCAU..GGUGCCGCCG..AAUA...........UGGCGACACCCGCUGAG............G.GGGA......GCGCGUCACC.....GUCAGGA
env-70    UCUGAA.GGCCGCGUUC....GGGUCAAU...UGCG.....GAC...........UGCA.ACACCCGA....UCUCG......CUCAU..GGUGCCGCCG..AAUA...........UGGCGACACCCGCUGAG............G.GGGA......GCGCGUCACC.....GUCAGGA
env-71    UCUGAA.GGCCGCGUUC....GGGUCAAU...UGCG.....GAC...........UGCA.ACACCCGA....UCUCG......CUCAU..GGUGCCGCCG..AAUA...........UGGCGACACCCGCUGAG............G.GGGA......GCGCGUCACC.....GUCAGGA
env-72    CCUGAU.CGGCGCGUCC....GGGUCAAU...UGCG.....GAC...........UGCA.ACACCCGA....UCUCC......CUCGCCGUUCCUCCC....GACA.............GGGAGGUACG.CGAG............G.GAGA......GCGCGUGACA.....AUCAGGA
env-74    CCUGAU.CGGCGCGUCC....GGGUCAAU...UGCG.....GAC...........UGCA.ACACCCGA....UCUCC......CUCGCCGUCCCUCCC....GACA.............GGGAGGUCA.GCGAG............G.GAGA......GCGCGUGACA.....AUCAGGA
env-76    CCUGAG.GAGCGCGUCC....GGGUCAAU...UGCG.....GCC...........UGCA.ACACCCGA....UCUCC......CUGC..CGGUGCCUCCA..GGAA...........UGGAGGUGCCGCGCGAG............G.GGGA......ACGCGUGACU.....AUCAGGG
env-77    CCUGAG.GAGCGCGUCC....GGGUCAAU...UGCG.....GCC...........UGCA.ACACCCGA....UCUCC......CUCC..CGGUGCCGCCA..GGAA...........UGGCGGCCCCGAGCGAG............G.GGGA......ACGCGUAACC.....GUCAGGG
env-78    CCUGAG.GAGCGCGUCC....GGGUCAAU...UGCG.....GCC...........UGCA.ACACCCGA....UCUCC......CUCC..CGGUGCCGCCA..GGAA...........UGGCGGCCCCGAGCGAG............G.GGGA......ACGCGUAACC.....GUCAGGG
Hsa-1-1   UCUGAU.GGGCGCGUUC....GGGUCAAU...UGCG.....GCA...........UGCA.ACACCCGA....UCUCU......CUCGGUGUGCCUCC.....UUCCAU............GGAGGU.CGCCGAG............G.GGGA......GCGCGUUGCC.....GUCAGGA
Hgi-1-1   CCUGAC.GACCGCGUUC....GGGUCAAU...UGCG.....GAA...........UGCA.ACACCCGA....UCUCCC.....UGUUCGUCCC.........UCCC..................GGGAUGGACC....UCACAGAGG.GAGA......GCGCGUCGCU.....GUCAGGA
Hsu-2-1   CCUGAC.GACCGCGUUC....GGGUCAAU...UGCG.....GAA...........UGCA.ACACCCGA....UCUCCC.....UGUUCGUCCC.........UCCC..................GGGAUGGACC....UCACAGAGG.GAGA......GCGCGUCGCU.....GUCAGGA
Hpr-1-1   CCUGAC.GACCGCGUUC....GGGUCAAU...UGCG.....GAA...........UGCA.ACACCCGA....UCUCCC.....UGUUCGUCCC.........UCCC..................GGGAUGGACC....UCACAGAGG.GAGA......GCGCGUCGCU.....GUCAGGA
Hsp-8-1   CCUGAC.GACCGCGUUC....GGGUCAAU...UGCG.....GAA...........UGCA.ACACCCGA....UCUCCC.....UGUUCGUCCC.........UCCC..................GGGAUGGACC....UCACAGAGG.GAGA......GCGCGUCGCU.....GUCAGGA
Hde-1-1   CCUGAC.GACCGCGUUC....GGGUCAAU...UGCG.....GAA...........UGCA.ACACCCGA....UCUCCC.....UGUUCGUCCC.........UCCC..................GGGAUGGACC....UCACAGAGG.GAGA......GCGCGUCGCU.....GUCAGGA
Hlu-1-1   CCUGAC.GACCGCGUUC....GGGUCAAU...UGCG.....GAA...........UGCA.ACACCCGA....UCUCCC.....UGUUCGUCCC.........UCCC..................GGGAUGGACC....UCACAGAGG.GAGA......GCGCGUCGCU.....GUCAGGA
Hal-2-1   CCUGAC.GACCGCGUUC....GGGUCAAU...UGCG.....GAA...........UGCA.ACACCCGA....UCUCCC.....UGUUCGUCCC.........UCCC..................GGGAUGGACC....UCACAGAGG.GAGA......GCGCGUCGCU.....GUCAGGA
Hsp-6-1   CCUGAC.GACCGCGUUC....GGGUCAAU...UGCG.....GAA...........UGCA.ACACCCGA....UCUCCC.....UGUUCGUCCC.........UCCC..................GGGAUGGACC....UCACAGAGG.GAGA......GCGCGUCGCU.....GUCAGGA
Hme-1-1   CCUGAC.GACCGCGUUC....GGGUCAAU...UGCG.....GAA...........UGCA.ACACCCGA....UCUCCC.....UGUUCAUCCC.........UCCG..................GGGAUGGAUA....UUGCAAAGG.GGGA......GCGCGUCGCU.....GUCAGGA
Hla-2-1   CCUGAC.GACCGCGUUC....GGGUCAAU...UGCG.....GAA...........UGCA.ACACCCGA....UCUCCC.....UGUUCGUCCC.........UCCC..................GGGAUGGACG.....CCACAGAGGGAGA......GCGCGUCGCU.....GUCAGGA
Hel-1-1   CCUGAC.GACCGCGUUC....GGGUCAAU...UGCG.....GAA...........UGCA.ACACCCGA....UCUCCC.....UGUUCGUCCC.........UCCC..................GGGAUGGACG.....CCACAGAGGGAGA......GCGCGUCGCU.....GUCAGGA
Hmu-1-1   CCUGAC.GACCGCGUUC....GGGUCAAU...UGCG.....GAA...........UGCA.ACACCCGG....UCUCC......CUGUUCACCCC........UCCG.................GGGAUGAAC.G....UUUCAAAGG.GGGA......GCGCGUCGCU.....GUCAGGA
env-79    GCUGAU.GGGCGCGUCU....GGGUCAAU...UGCG.....GAC...........UGCA.ACACCCACU....CUCU......CUCC........CCGUCCCGAAC.........GGGAUGG.AACUCACGGAG............G.GGGG......CCGCGUUUCC.....GUCAGGG
env-80    ACUGAU.GGGCGCGUCC....GGGUCAAU...UGCG.....GAA...........UGCA.ACACCCGA....UCUCCC.....UUC.........CCGUCCCGAAU.........GGGAUGG.AUAUCACAGAG............G.GGGA......GCGUGUCACC.....GUCAGAG
env-81    ACUGAU.GGGCGCGUCC....GGGUCAAU...UGCG.....GAA...........UGCA.ACACCCGA....UCUCC......CUUCC.......CGUCCC.CACU..........GGGAUGCAAGUACGGGAA............G.GAGA......GCGUGUCGCC.....GUCAGAG
env-82    ACUGAU.GGGCACGUCC....GGGUCAAU...UGCG.....GAC...........UGCA.ACACCCGA....UCUCC......CUUCC.......CGUCCC.GGAU..........GGGAUGGAUCUCACAGAG............G.GGGA......GCGUGUCACC.....GUCAGAG
env-83    ACUGAU.GGGCACGUCC....GGGUCAAU...UGCG.....GAC...........UGCA.ACACCCGA....UCUCC......CUUCC.......CGUCCC.GGAU..........GGGAUAGAUCUCACAGAG............G.GGGA......GCGUGUCACC.....GUCAGAG
Hla-4-1   CCUGAU.CAACGCGUCC....GGGUCAAU...UGCG.....GAC...........UGCA.ACACCCGA....UCUCU......CUCAACG..UGCCAUC...GGA.............GAUGGCACUCGCGGAG............G.GAGA......GCGCGUGACA.....GUCAGGA
Ssp-1-1   CCUGAU.GGGCGCGUAC....GGGUCAAU...UGCG.....GCG...........UGCA.ACACCCGA....UCUCC......CU.GCCGGUUCCCC.....ACGAAC............GGGACAUCGGCGAG............G.GGGA......GCGUGUGACC.....GUCAGGG
env-84    UCUGAC.GGGCGCGUUC....GGGUCAAU...UGCG.....GAA...........UGCA.ACACCCGA....UCUCC......CUCC.ACACG.CCAC....CCGAC............GUGGCCGUGUCGGAG............G.GGGA......GCGCGUGACC.....AUCAGGG
env-86    CCUGAA.GGGCGCGUCC....GGGUCAAU...UGCG.....GCU...........UGCA.ACACCCGA....UCUCU......CUCAA..UGUGCCG.....CCAUCGA...........UGGCACUCGAAGAG............G.GAGA......GCGCGUGACC.....GUCAGGA
Hsp-11-1  CCUGAU.CAACGCGUCC....GGGUCAAU...UGCG.....GAC...........UGCA.ACACCCGA....UCUCU......CUGAA..CGUGCCGUC...GGA.............GAUGGCACUCGAGCAG............G.GAGA......GCGCGUGACA.....GUCAGGA
env-87    CCUGAG.GACCGCGUUC....GGGUCACU...UGCG.....GAA...........CGCA.ACACCCGA....UCUCC......CUUC..UCGCGCCC.....GGAC..............GGGCGCUGACGGAG............G.GGGA......GCGCGUCACC.....AUCAGGG
env-88    UCUGAA.GGCCGCGUCU....GGGUCAAU...UGCG.....GAC...........UGCA.ACACCCAA....UCUCG......CUCAU..GGUGCCGCCG..AAUA...........UGGCGACACCCCAAGAG............G.GAGA......GCGCGUCACC.....GUCAGGA
env-89    GCUGAU.GGGCGCGCUC....GGGUCAAU...UGCG.....GUC...........UGCA.ACACCCAA....UCUCC......CU..GCCGGUCCCGUCCG.GAA............UGGAUGGGCUGGUUGAG............G.GGGA......ACGCGUCUCC.....GUCAGGG
Hka-1-1   GCUGAU.GGGCGCGUUC....GGGUCAAU...UGCG.....GAC...........UGCA.ACACCCGA....UCUCU......CUC.CCGGUACCUUCCG..CGA............UGGAAGUGCCCGGCGAG............G.GGGA......GCGCGUCACC.....GUCAGGG
Hmu-2-1   GCUGAU.GGGCGCGUUC....GGGUCAAU...UGCG.....GAC...........UGCA.ACACCCGA....UCUCU......CUC.CCGGUACCUUCCG..CGA............UGGAAGUGCCCGGCGAG............G.GGGA......GCGCGUCACC.....GUCAGGG
Hca-3-1   GCUGAU.GAACGCGUAC....GGGUCAAU...UGCG.....GCC...........UGCA.ACACCCAG....UCUCG......CUCCCCGUGCCCUCCG...GAA.............UGGAGGUCGGGG..AG............G.GGGA......GCGCGUCUCC.....GUCAGGG
Hsa-5-1   CCUGAU.GGACGCGUUC....GGGUCAAU...UGCG.....GAC...........UGCA.ACACCCGA....UCUCA......CUGGUC.GAUCCCAC....CGAC.............GUGGGACUCGACGAG............G.GAGA......GCGUGUGACC.....GUCAGGA
env-90    CCUGAU.GGACGCGUUC....GGGUCAAU...UGCG.....GCU...........UGCA.ACACCCGA....UCUCG......CUGGUC.GAUCCCAC....CGAC.............GUGGGACUCGACGAG............G.GAGA......GCGUGUGACC.....GUCAGGA
Hag-1-1   CCUGAU.GGACGCGUUC....GGGUCAAU...UGCG.....GAC...........UGCA.ACACCCGA....UCUCG......CUGGUC.GAUCCCAC....CGAC.............GUGGGACUCGACGAG............G.GAGA......GCGUGUGACC.....GUCAGGA
Hsa-4-1   CCUGAU.GGACGCGUUC....GGGUCAAU...UGCG.....GAC...........UGCA.ACACCCGA....UCUCG......CUGGUC.GAUCCCAC....CGAC.............GUGGGACUCGACGAG............G.GGGA......GCGUGUGACC.....GUCAGGA
env-91    GCUGAU.GGCCGCGUUC....GGGUCAAU...UGCG.....GAC...........UGCA.ACACCCGG....UCUCA......CUCC..CGGUGUCGCC...CGCGAU..........GGCGACGCCGUCGGAG............G.GGGA......GCGUGUCACC.....GUCAGCA
env-92    UCUGAA.GGCCGCGUCC....GGGUCACU...UGCG.....GAC...........UGCA.ACACCCGA....UCUCU......CUCA..CGGUGCCGCCG..UCGA...........UGGCGACACCGGCUGAG............G.GGGA......GCGCGUCACC.....GUCAGGA
env-93    UCUGAA.GGCCGCGUCC....GGGUCACU...UGCG.....GAC...........UGCA.ACACCCGA....UCUCU......CUCA..CGGUGCCGCCG..UCGA...........UGGCGACACCGGCUGAG............G.GGGA......GCGCGUCACC.....GUCAGGA
env-94    UCUGAA.GGCCGCGUCC....GGGUCACU...UGCG.....GAC...........UGCA.ACACCCGA....UCUCU......CUCA..CGGUGCCGCCG..UCGA...........UGGCGACACCGGCUGAG............G.GGGA......GCGCGUCACC.....GUCAGGA
env-95    UCUGAA.GGCCGCGUCC....GGGUCACU...UGCG.....GAC...........UGCA.ACACCCGA....UCUCU......CUCA..CGGUGCCGCCG..UCGA...........UGGCGACACCGGCUGAG............G.GGGA......GCGCGUCACC.....GUCAGGA
env-96    UCUGAA.GGCCGCGUCC....GGGUCACU...UGCG.....GAC...........UGCA.ACACCCGA....UCUCU......CUCA..CGGUGCCGCCG..UCGA...........UGGCGACACCGGCUGAG............G.GGGA......GCGCGUCACC.....GUCAGGA
env-98    UCUGAA.GGCCGCGUCC....GGGUCACU...UGCG.....GAC...........UGCA.ACACCCGA....UCUCC......CUCA..CGGUGCCGCCG..UCGA...........UGGCGACACCGGCCGAG............G.GGGA......GCGCGUCACC.....GUCAGGA
env-99    UCUGAA.GGCCGCGUCC....GGGUCACU...UGCG.....GAC...........UGCA.ACACCCGA....UCUCU......CUCA..CGGUGCCGCCG..UCGA...........UGGCGACACCGGCCGAG............G.GGGA......GCGCGUCACC.....GUCAGAA
env-101   UCUGAA.GGCCGCGUUC....GGGUCACU...UGCG.....GAC...........UGCA.ACACCCGA....UCUCC......CUCA..UGGUGCCGCCG..GAGA...........UGGCAGCGCCGAGCGAG............G.GGGA......GCGCGUCACC.....GUCAGGA
env-102   UCUGAA.GGCCGCGUUC....GGGUCACU...UGCG.....GAC...........UGCA.ACACCCGA....UCUCC......CUCA..UGGUGCCGCCG..AAUA...........UGGCGACGCCAGCCGAG............G.GGGA......GCGCGUCACC.....GUCAGGA
env-103   UCUGAA.GGCCGCGUUC....GGGUCACU...UGCG.....GAC...........UGCA.ACACCCGA....UCUCC......CUCA..UGGUGCCGCCG..AAUA...........UGGCGACGCCAGCCGAG............G.GGGA......GCGCGUCACC.....GUCAGGA
env-104   UCUGAA.GGCCGCGUUC....GGGUCACU...UGCG.....GAC...........UGCA.ACACCCGA....UCUCC......CUCA..UGGUGCCGCCG..GAUA...........UGGCGACGCCAGCCGAG............G.GGGA......GCGCGUCACC.....GUCAGGA
env-105   UCUGAA.GGCCGCGUUC....GGGUCACU...UGCG.....GAC...........UGCA.ACACCCGA....UCUCC......CUCA..UGGUGCCGCCG..GAUA...........UGGCGACGCCAGCCGAG............G.GGGA......GCGCGUCACC.....GUCAGGA
env-106   UCUGAA.GGCCGCGUUC....GGGUCACU...UGCG.....GAC...........UGCA.ACACCCGA....UCUCC......CUCA..UGGUGCCGCCG..GAUA...........UGGCGACGCCAGCCGAG............G.GGGA......GCGCGUCACC.....GUCAGGA
env-107   UCUGAC.GGCCGCGUCC....GGGUCAAU...UGCG.....GAC...........UGCA.ACACCCGA....UCUCC......CUCCUCCUCCCC.......GAC.................GGGAUGGAGGUG......UCCGAAG.GAGA......GCGCGUCGCC.....AUCAGGA
env-108   UCUGAG.GGACGCGUUC....GGGUCAAU...UGCG.....GAC...........UGCA.ACACCCGA....UCUCU......CUCCACACGCCUCCC....GGAC.............GGGAGCUGU.CGGAG............G.GGGA......GCGCGCAACC.....AUCAGGA
env-109   UCUGAC.GGGCGCGUUC....GGGUCAAU...UGCG.....GCA...........UGCA.ACACCCGA....UCUCCCU....CCUC..GCUCCC.......GACC................GGGAGUUCGAGG..........UCG.GAGA......GCGCGUUACC.....GUCAGGG
CHa-1-1   CCUGAA.GGCCGCGUUC....GGGUCAAU...UGCG.....GAC...........UGCA.ACACCCGA....UCUCCCUC...CACCUG..CCUCCC.....GGAC..............GGGAGGCGCAGGAG............G.GGGA......GCGCGUCACC.....GUCAGGG
Hpa-2-1   CCUGAA.GGUCGCGUUC....GGGUCAAU...UGCG.....GAC...........UGCA.ACACCCGG....UCUCC......CUCUA..CGCUCCGU....CCGGU............ACGGAGUUGGAGGAG............G.GAGA......GCGCGUCGCC.....AUCAGGA
env-110   CCUGAU.CGGCGCGUCC....GGGUCAAU...UGCG.....GCU...........UGCA.ACACCCGA....UCUCG......CUCAACGUGCCUCCA....CGAA.............UGGAGGUU.ACGGAG............G.GAGA......GCGCGUGACA.....GUCAGGA
Nhu-1-1   CCUGAU.CAACGCGUGC....GGGUCAAU...UGCG.....GCU...........UGCA.ACACCCGA....UCUCC......CUCAACG..UACCGCCG..AAAA...........UGGCGGUCUCCG...AG............G.GAGA......GCGCGUGACU.....AUCAGGA
env-113   CCUGAA.GGCCGCGUCC....GGGUCAAU...UCCC.....CGCUGC........GGGA.ACACCCGA....UCUCC......CUGCUCGUCCCCUCCG...GAA.............UGGAGGCCGAGC.CGG..............GGGA......ACGCGUCACC.....AUCAGGG
env-114   UCUGAA.GGCCGCGUCC....GGGUCACU...UGCG.....GAC...........UGCA.ACACCCGA....UCUCC......CUCGCCGUGC..CGCCG..UCGA...........UGGCGACACCGGCCGAG............G.GGGA......GCGCGUCACC.....GUCAGGA
env-115   UCUGAA.GGCCGCGUCC....GGGUCACU...UGCG.....GAC...........UGCA.ACACCCGA....UCUCC......CUCGCCGUGC..CGCCG..UCGA...........UGGCGACACCGGCCGAG............G.GGGA......GCGCGUCACC.....GUCAGGA
env-116   UCUGAA.GGCCGCGUUC....GGGUCACU...UGCG.....GAC...........UGCA.ACACCCGA....UCUCC......CUUGUGGUGC..CGCCG..GAGA...........UGGCGACACCCACUGAG............G.GGGA......GCGCGUCACC.....GUCAGGA
env-117   UCUGAA.GGCCGCGUUC....GGGUCACU...UGCG.....GAC...........UGCA.ACACCCGA....UCUCC......CUCAUGGUGC..CGCCG..GAUA...........UGGCGACGCCCACUGAG............G.GGGA......GCGCGUCACC.....GUCAGGA
env-118   UCUGAA.GGCCGCGUUC....GGGUCACU...UGCG.....GAC...........UGCA.ACACCCGA....UCUCU......CUCAUGGUGC..CGCCG..GAUA...........UGGCGACACCCACUGAG............G.GAGA......GCGCGUCACC.....GUCAGGA

          .<<<<<....<<<<<<.....<<<<...<...<<<<<<<<<.........>>>>>>>>>.>.>>>>......<<<<<......<<<<<<<<<<<<<<<<<<<.............>>>>>>>>>>>>>>>>>>>.............>.>>>>.....>>>>>>..........>>>>>..
          .20.......22222......001...2...22221110..........01112222.2.100.......22222......222222..2.2.1111110.............0111111.2.2..222222............2.2222......22222..........02..
          YCUGAY-○R●●RCGY○Y----GGGU●RAY---●●○○-----●●●○----------○R●●-R○RCCCR●----UYYC●------●●○○○--------------●●●○-------------------○○○○○●-----------R-GRRR-----GCGY●●●●○○----RUCAGGR
          5'                                                                                                                                                                                3'
```

```
env-119   UCUGAA.GGCCGCGUUC....GGGUCACU...UGCG.....GAC...........UGCA.ACACCCGA....UCUCC......CUUGUGGUGC..CGCCG..AAUA...........UGGCGACACCCGCUGCG............G.GAGA......GCGCGUCACC.....GUCAGGA
env-120   CCUGAG.GGCCGCGUUC....GGGUCAAU...UGCG.....GAC...........UGCA.ACACCCGA....UCUCCCU....CCGGGCGCCCC........CAGAC................GGGGUGCCCGA........CGAGG.GGGA......GCGUGUCACC.....GUCAGGG
env-122   CCUGAA.GGCCGCGUUC....GGGUCAAU...UGCG.....GAC...........UGCA.ACACCCGA....UCUCCCU....CCGGGCACCCC........CAGAC................GAGGUGCCCGC........CGAGG.GGGA......GCGCGUCACC.....GUCAGGG
env-123   CCUGAA.GGCCGCGUCC....GGGUCAAU...UGCG.....GAC...........UGCA.ACACCCGA....UCUCCCU....CCGGGCACCCC........CAGAC................GGGGUGCCCGC........CGAGG.GGGA......GCGCGUCACC.....GUCAGGG
env-126   CCUGAC.GGCCGCGUCC....GGGUCAAU...UGCG.....AAC...........UGCA.ACACCCAACC..GCCCU......CCGUGCGCCUCCG......UCGA...............UGGAGG..UGCGG..........GAG.GGGCA.....ACGCGUCGCC.....AUCAGGA
env-127   CCUGAC.GGCCGCGUCC....GGGUCAAU...UGCG.....AAC...........UGCA.ACACCCAACCA.CCCUU......CGUGCGCCUCCG.......UCGA................UGGAGGUGCGGG............A.GGGGCA....ACGCGUCGCC.....AUCAGGA
env-128   CCUGAC.GGCCGCGUCC....GGGUCAAU...UGCG.....AAC...........UGCA.ACACCCAACCA.CCCUU......CGUGCGCCUCCG.......UCGA................UGGAGGUGCGGG............A.GGGGCA....ACGCGUCGCC.....AUCAGGA
env-129   CCUGAU.GACCGCGUUC....GGGUCACU...UGCG.....GAC...........UGCA.ACACCCGA....UCUCA......CUCGG.CGUG.CCG.....AAAGAAA...........UGGAGGUCGCCGAG............G.GGGA......GCGCGUCACC.....GUCAGGA
Hna-1-1   CCUGAC.GGCCGCGUUC....GGGUCAAU...UGCG.....GAC...........UGCA.ACACCCGA....UCUCC......CUCCU.CAC.UCCGG....ACGGA............CUGGAAGUCAAGGAG............G.GGGA......GCGCGUCGCC.....AUCAGGA
env-130   CCUGAC.GGCCGCGUCC....GGGUCAAU...UGCG.....GAC...........UGCA.ACACCCAA....UCUCC......CUCCU...CCUCC......CGGAUGGGAU.........GGAGGUGUCGGAC............G.GAGA......GCGCGUCGCC.....AUCAGGA
env-131   CCUGAC.GACCGCGUCC....GGGUCAAU...UGCG.....GCU...........UGCA.ACACCCGAUC..UCCCU......CCACGUCCCGUCG......UCGA...............UGACGGU.CGUGG..........AAG.GGGA......GCGCGUCACU.....GUCAGGG
env-134   CCUGAC.GGGCGCGUCC....GGGUCAAU...UGCG.....GAC...........UGCA.ACACCCGA....UCUCG......CUCCCCGU..GCCUCC...GUC.............GGAGGAGCUCGAGGAG............G.GAGA......ACGCGUCACC.....AUCAGGG
env-135   CCUGAA.GGGCGCGUCC....GGGUCAAU...UGCG.....GAC...........UGCA.ACACCCCGA...UCUCC......CUCAACAUACCGCCG....AACA.............UGGCGG..UCGAGGG...........AG.GGGGA.....GCGCGUGACC.....GUCAGGA
Nph-1-1   CCUGAC.GGGCGCGUCC....GGGUCAAU...UGCG.....GAA...........UGCA.ACACCCGA....UCUCU......CUCCCCGUCCCUCC.....CUCGAU............GGAGG.UCGGGAAG............G.GAGA......GCGCGUCACC.....AUCAGGG
env-136   UCUGAC.GAGCGCGUCC....GGGUCAAU...UGCG.....GAC...........UGCA.ACACCCGA....UCUCC......CUCCCCGUUCCUCCA....UCGA.............UGGA.GGUCGAGGAA............G.GGGA......ACGCGUCACC.....AUCAGGG
env-137   UCUGAC.AAGCGCGUCC....GGGUCAAU...UGCG.....GAC...........UGCA.ACACCCGA....UCUCC......CUCCCCGUUCCUCCA....UCGA.............UGGA.GGUCGAGGAA............G.GGGA......ACGCGUCACC.....AUCAGGG
env-138   CCUGAA.GGCCGCGUCC....GGGUCAAU...UCCC.....CGCUGC........GGGA.ACACCCGA....UCUCCCU....GCUCGUCCCCUCCG.....AGA...............UGGAGGUG.CGAGC...........GG.GAGA......GCGCGUCACC.....AUCAGGG
env-139   CCUGAA.GGCCGCGUCC....GGGUCAAU...UCCC.....CGCUGC........GGGA.ACACCCGA....UCUCCCU....GCUCGUUCCCUCCG.....AGA...............UGGGGGU..CGAGU..........CGG.GGGA......GCGCGUUACC.....AUCAGGG
env-140   CCUGAA.GGCCGCGUCC....GGGUCAAU...UCCC.....CGCUGC........GGGA.ACACCCGA....UCUCCCUGC..UCGUCC..CCGACG.....AUA...............UGUCGGUCGAGCCG............G.GGGA......GCGCGUCACC.....AUCAGGG
env-145   CCUGAA.GGCCGCGUUC....GGGUCAAU...UCCCC....GCUG.........GGGGA.ACACCCGA....UCUCCCUGC..UCGUCC..CCUCCG.....AAA...............UGGAGGUCGAGCGG............G.GGGA......ACGCGUCACC.....AUCAGGG
env-146   CCUGAA.GGCCGCGUCC....GGGUCAAU...UCCC.....CGCUGC........GGGA.ACACCCGA....UCUCCCUGC..UCGUCC..CCUCCG.....GAA...............UGGAGGUUGAGCAA............G.GAGA......GCGCGUCACC.....AUCAGGG
env-147   CCUGAA.GGCCGCGUUC....GGGUCAAU...UCCC.....CGCUGC........GGGA.ACACCCGA....UCUCCCUGC..UCGUCC..CCUCCG.....GAA...............UGGAGGUCGAGCAG............G.GGGA......GCGCGUCACC.....AUCAGGG
env-148   CCUGAA.GGCCGCGUUC....GGGUCAAU...UCCCC....GCUG.........GGGGA.ACACCCGA....UCUCCCUGC..UCGUUC..CCUCCG.....GAC...............UGGAGGUCGAGCAG............G.GGGA......GCGCGUCACC.....AUCAGGG
Hsp-9-1   CCUGAA.GGCCGCGUCC....GGGUCAAU...UCCC.....CGCUGC........GGGA.ACACCCGA....UCUCCCUGC..UCGUUC..CCUCCG.....ACA...............UGGAGGUCGAGCAG............G.GGGA......GCGCGUCACC.....AUCAGGG
Hez-1-1   CCUGAA.GGCCGCGUCC....GGGUCAAU...UCCC.....CGCUGC........GGGA.ACACCCGA....UCUCCCUGC..UCGUCC..CCUCCG.....AAA...............UGGAGGUCGAGCGG............G.GGGA......GCGCGUCACC.....AUCAGGG
env-149   CCUGAA.GGCCGCGUCC....GGGUCAAU...UCCC.....CGCUGC........GGGA.ACACCCGA....UCUCCCUGC..UCGUGC..CCUCCG.....GAA...............UGGAGGUCGAGCGG............G.GGGA......GCGCGUCACC.....AUCAGGG
env-150   CCUGAA.GGCCGCGUCC....GGGUCAAU...UCCC.....CGCUGC........GGGA.ACACCCGA....UCUCCCUGC..UCGUGC..CCUCCG.....GAA...............UGGAGGUCGAGCGG............G.GGGA......GCGCGUCACC.....AUCAGGG
env-151   CCUGAA.GGCCGCGUCC....GGGUCAAU...UCCC.....CGCUGC........GGGA.ACACCCGA....UCUCCCUGC..UCGUCC..CCUCCG.....GAA...............UGGAGGUCGAGCGG............G.GGGA......ACGCGUCACC.....AUCAGGG
Hco-1-1   CCUGAA.GGCCGCGUCC....GGGUCAAU...UCCC.....CGCUGC........GGGA.ACACCCGA....UCUCCCUGC..UCGUCC..CCUCCG.....GAA...............UGGAGGUCGUGCGG............G.GGGA......GCGCGUCACC.....AUCAGGG
env-152   CCUGAA.GGCCGCGUCC....GGGUCAAU...UCCC.....CGCUGC........GGGA.ACACCCGA....UCUCCCUGC..UCGUCC..CCUCCG.....GAA...............UGGAGGUCGAGCGG............G.GGGA......GCGCGUCACC.....AUCAGGG
env-153   CCUGAA.GGCCGCGUCC....GGGUCAAU...UCCC.....CGCUGC........GGGA.ACACCCGA....UCUCCCUGC..UCGUCC..CCUCCG.....GAA...............UGGAGGUCGAGCGG............G.GGGA......GCGCGUCACC.....AUCAGGG
env-154   CCUGAA.GGCCGCGUCC....GGGUCAAU...UCCC.....CGCUGC........GGGA.ACACCCGA....UCUCCCUGC..UCGUCC..CCUCCG.....GAA...............UGGAGGUCGAGCGG............G.GGGA......GCGCGUCACC.....AUCAGGG
env-155   CCUGAA.GGCCGCGUCC....GGGUCAAU...UCCC.....CGCUGC........GGGA.ACACCCGA....UCUCCCUGC..UCGUCC..CCUCCG.....GAA...............UGGAGGUCGAGCGG............G.GGGA......GCGCGUCACC.....AUCAGGG
env-156   CCUGAA.GGCCGCGUCC....GGGUCAAU...UCCC.....CGCUGC........GGGA.ACACCCGA....UCUCCCUGC..UCGUCC..CCUCCG.....GAA...............UGGAGGUCGAGCGG............G.GGGA......GCGCGUCACC.....AUCAGGG
Hsp-10-1  CCUGAA.GGCCGCGUCC....GGGUCAAU...UCCC.....CGCUGC........GGGA.ACACCCGA....UCUCCCUGC..UCGUCC..CCUCCG.....GAA...............UGGAGGUCGAGCGG............G.GGGA......GCGCGUCACC.....AUCAGGG
Hli-1-1   CCUGAA.GGCCGCGUCC....GGGUCAAU...UCCC.....CGCUGC........GGGA.ACACCCGA....UCUCCCUGC..UCGUUC..CCUCCG.....GAA...............UGGAGGUCGAGCAA............G.GGGA......GCGCGUCACC.....AUCAGGG
Hte-1-1   CCUGAA.GGCCGCGUCC....GGGUCAAU...UCCCC....GCUG.........GGGGA.ACACCCGA....UCUCCCUGC..UCGUUC..CCUCCG.....GAA...............UGGAGGUCGAGCAG............G.GGGA......GCGCGUCACC.....AUCAGGG
Hko-1-1   CCUGAA.GGCCGCGUCC....GGGUCAAU...UCCC.....CGCUGC........GGGA.ACACCCGA....UCUCCCUGC..UCGUUC..CCUCCG.....GAA...............UGGAGGUCGAGCAG............G.GGGA......GCGCGUCACC.....AUCAGGG
Hli-2-1   CCUGAA.GGCCGCGUCC....GGGUCAAU...UCCC.....CGCUGC........GGGA.ACACCCGA....UCUCCCUGC..UCGUUC..CCUCCG.....GAA...............UGGAGGUCGAGCAG............G.GGGA......GCGCGUCACC.....AUCAGGG
Hsa-7-1   CCUGAA.GGCCGCGUCC....GGGUCAAU...UCCC.....CGCUGC........GGGA.ACACCCGA....UCUCCCUGC..UCGUUC..CCUCCG.....GAA...............UGGAGGUCGAGCAG............G.GGGA......GCGCGUCACC.....AUCAGGG
Hca-2-1   CCUGAA.GGCCGCGUCC....GGGUCAAU...UCCC.....CGCUGC........GGGA.ACACCCGA....UCUCCCUGC..UCGUUC..CCUCCG.....GAA...............UGGAGGUCGAGCAG............G.GGGA......GCGCGUCACC.....AUCAGGG
Hsp-7-1   CCUGAA.GGCCGCGUCC....GGGUCAAU...UCCC.....CGCUGC........GGGA.ACACCCGA....UCUCCCUGC..UCGUUC..CCUCCG.....GAA...............UGGAGGUCGAGCAG............G.GGGA......GCGCGUCACC.....AUCAGGG
Hha-2-1   CCUGAA.GGCCGCGUCC....GGGUCAAU...UCCC.....CGCUGC........GGGA.ACACCCGA....UCUCCCUGC..UCGUUC..CCUCCG.....GAA...............UGGAGGUCGAGCAG............G.GGGA......GCGCGUCACC.....AUCAGGG
Hdi-1-1   CCUGAA.GGCCGCGUCC....GGGUCAAU...UCCC.....CGCUGC........GGGA.ACACCCGA....UCUCCCUGC..UCGUUC..CCUCCG.....GAA...............UGGAGGUCGAGCAG............G.GGGA......GCGCGUCACC.....AUCAGGG
Hsa-9-1   CCUGAA.GGCCGCGUCC....GGGUCAAU...UCCC.....CGCUGC........GGGA.ACACCCGA....UCUCCCUGC..UCGUUC..CCUCCG.....GAA...............UGGAGGUCGAGCAG............G.GGGA......GCGCGUCACC.....AUCAGGG
Har-2-1   CCUGAA.GGCCGCGUCC....GGGUCAAU...UCCC.....CGCUGC........GGGA.ACACCCGA....UCUCCCUGC..UCGUUC..CCUCCG.....GAA...............UGGAGGUCGAGCAG............G.GGGA......GCGCGUCACC.....AUCAGGG
env-157   CCUGAA.GGCCGCGUCC....GGGUCAAU...UCCC.....CGCUGC........GGGA.ACACCCGA....UCUCCCUGC..UCGUUC..CCUCCG.....GAA...............UGGAGGUCGAGCAG............G.GGGA......GCGCGUCACC.....AUCAGGG
env-158   CCUGAA.GGCCGCGUCC....GGGUCAAU...UCCC.....CGCUGC........GGGA.ACACCCGA....UCUCCCUGC..UCGUUC..CCUCCG.....GAA...............UGGAGGUCGAGCGU............G.GAGA......GCGCGUCACC.....AUCAGGG
env-159   CCUGAA.GGCCGCGUCC....GGGUCAAU...UCCC.....CGCUGC........GGGA.ACACCCGA....UCUCCCUGC..UCGUUC..CCUCCG.....GAA...............UGGAGGUCGAGCGU............G.GAGA......GCGCGUCACC.....AUCAGGG
env-160   CCUGAA.GGCCGCGUUC....GGGUCAAU...UCCC.....CGCUGC........GGGA.ACACCCAA....UCUCCCUGC..UCGUCC..CCUCCG.....GAA...............UGGAGGUUGAGCGG............G.GGGA......GCGCGUCACC.....AUCAGGG
h3A-1-1   CCUGAC.GGCCGCGUUC....GGGUCAAU...UCCCC....GCUG.........GGGGA.ACACCCGA....UCUCCCUGC..UCGUUC..CCGACG.....GAC...............UGUCGGUCGAGCGG............G.GGGA......GCGCGUCACC.....AUCAGGA
Hai-1-1   CCUGAA.GGCCGCGUCC....GGGUCAAU...UCCC.....CGCUGC........GGGA.ACACCCGA....UCUCC......CUGCUCGUGCCCUCCG...GAA.............UGGAGG..UCGAGCAG............G.GGGA......GCGCGUCACC.....AUCAGGG
env-161   CCUGAA.GGCCGCGUCC....GGGUCAAU...UCCC.....CGCUGC........GGGA.ACACCCGA....UCUCC......CUGCUCGUCCCCUCCG...GAA.............UGGAGG..UCGAGCAG............G.GGGA......GCGCGUCACC.....AUCAGGG
Hho-1-1   CCUGAA.GGCCGCGUCC....GGGUCAAU...UCCC.....CGCUGC........GGGA.ACACCCGA....UCUCC......CUGCUCGUCCCCUCCG...ACA.............UGGAGG..UCGAGCAG............G.GGGA......GCGCGUCACC.....AUCAGGG
env-164   CCUGAA.GGCCGCGUCC....GGGUCAAU...UCCC.....CGCUGC........GGGA.ACACCCGA....UCUCCCUG...CUCGUC.CCCGCCG.....ACA...............UGGCGGUCGAGCCG............G.GGGA......GCGCGUCACC.....AUCAGGG
env-165   CCUGAA.GGCCGCGUCC....GGGUCAAU...UCCC.....CGCUGC........GGGA.ACACCCAA....UCUCC......CUGCUCGUCCCCUCCG...AGG.............UGGAGG.UGCGGGC.G............G.GAGA......GCGCGUCACC.....AUCAGGG
env-166   CCUGAA.GGCCGCGUCC....GGGUCAAU...UCCC.....CGCUGC........GGGA.ACACCCAA....UCUCC......CUGCUCGUCCCCUCCG...AGG.............UGGAGG.UGCGGGC.G............G.GAGA......GCGCGUCACC.....AUCAGGG
env-167   CCUGAA.GGCCGCGUCC....GGGUCAAU...UCCC.....CGCUGC........GGGA.ACACCCGA....UCUCCCUGCU.CGUCC...CCUCCA.....ACA...............UGGAGGUGCGGGCG............G.GAGA......GCGCGUCACC.....AUCAGGG
env-169   CCUGAA.GGCCGCGUCC....GGGUCAAU...UCCC.....CGCUGC........GGGA.ACACCCGA....UCUCCCUGCU.CGUCC...CCUCCA.....ACA...............UGGAGGUGCGGGCG............G.GAGA......GCGCGUCACC.....AUCAGGG
env-170   CCUGAA.GGCCGCGUCC....GGGUCAAU...UCCC.....CGCUGC........GGGA.ACACCCGA....UCUCCCUGCU.CGUCC...CCUCCA.....ACA...............UGGAGGUGCGGGCG............G.GAGA......GCGCGUCACC.....AUCAGGG
env-172   CCUGAA.GGCCGCGUCC....GGGUCAAU...UCCC.....CGCUGC........GGGA.ACACCCGA....UCUCCCUGCU.CGUCC...CCUCCA.....ACA...............UGGAGGUGCGGGCG............G.GAGA......GCGCGUCACC.....AUCAGGG
env-173   CCUGAU.GGGCGCGUUC....GGGUCAAU...UGCG.....GCC...........UGCA.ACACCCGA....UCUCGCUC...CCCGUGCCGUCA.......AAAA................UGUCGGUACAGG..........AGG.GAGA......GCGCGUAACC.....GUCAGGG
Hru-1-1   CCUGAC.GGCCGCGUUC....GGGUCAAU...UGCG.....GAC...........UGCA.ACACCCGG....UCUCU......CUGCUCGCGCCUCCG....UCGG.............UGGAGGUC.CAGCAG............G.GGGA......GCGCGUCGCC.....AUCAGGA
env-174   CCUGAC.GGCCGCGUUC....GGGUCAAU...UGCG.....AAC...........UGCA.ACACCCGG....UCUC.GCUG..CUCGU..GCCUCCG.....UCGA..............UGGAGGUCGAGGAG..............GGGAC.....GCGCGUCGCC.....AUCAGGA
Hru-2-1   CCUGAC.GACCGCGUCC....GGGUCAAU...UGCG.....GAC...........UGCA.ACACCCGAUC..UCCCUG.....CUCGU..CCCGCCG.....UCGA..............UGGCGGUCGAGGAG............G.GGGA......GCGCGUCACA.....GUCAGGG
env-175   UCUGAC.GGGCGCGUCC....GGGUCGAA...UUGCG....GAC..........UGCAA..CACCCGAUC..UCCCUG.....CCGAUACCGCCA.......UCGA................UGGCGGUAUCGG..........CAG.GGGA......ACGCGUCACC.....AUCAGGG
env-176   UCUGAC.GGGCGCGUCC....GGGUCAAU...UGCG.....GAC...........UGCA.ACACCCGAUC..UCCCUA.....CCGGUGCCGC.........AGACGUUG..............GCGGUGUCGG..........AAG.GGGA......ACGCGUCACC.....AUCAGGG
env-178   UCUGAC.GGGCGCGUCC....GGGUCAAU...UGCG.....GAC...........UGCA.ACACCCGAUC..UCCCUG.....CCGGUACCGCCG.......ACGA................UGGCGGUGUCGG..........AAG.GGGA......ACGCGUCACC.....AUCAGGG
env-179   UCUGAC.GGGCGCGUCC....GGGUCAAU...UGCG.....GAC...........UGCA.ACACCCGAUC..UCCCUG.....CCGGUACCGCCG.......UCGA................UGGCGGUCUCGG..........CCG.GGGA......ACGCGUCACC.....AUCAGGG
env-180   UCUGAC.GGACGCGUCC....GGGUCAAU...UGCG.....GAC...........UGCA.ACACCCGAUC..UCCCU......UCCGGUGCCGCCG......UCGA...............UGGCGGUUUCGGA...........GG.GGGA......GCGCGUCACC.....AUCAGGG
Nmo-1-1   UCUGAC.GGGCGCGUCC....GGGUCAAU...UGCG.....GAC...........UGCA.ACACCCGAUC..UCCCU......GCCGGUGCCGCCG......UCGA...............UGGCGGUCUCGGC...........CG.GGGA......ACGCGUCACC.....AUCAGGG
Hha-1-1   CCUGAU.GGGCGCGUUC....GGGUCAAU...UGCG.....GAC...........UGCA.ACACCCGG....UCUCG......CUCGUCGUGCCUCC.....CGGAGU............GGAGGUC.GGCGAG............G.GGGA......ACGCGUGACC.....GUCAGGA
env-181   CCUGAU.CGGCGCGUCC....GGGUCAAU...UGCG.....GCG...........UGCA.ACACCCGA....UCUCCCUCAA.CU..G..UCCGUCG.....GAAA..............UGACGGUCACCGAG............G.GGGA......GCGCGUGACG.....AUCAGGA
Hpa-1-1   CCUGAA.GGGCGCGUUC....GGGUCAAU...UGCG.....GAC...........UGCA.ACACCCGA....UCUCACUCG..UCGU..UCCGUCG......CAAA...............UGACGGUCGGCGA...........GG.GAGA......ACGCGUUACC.....GUCAGGA
Hci-1-1   CCUGAA.GGGCGCGUCC....GGGUCAAU...UGCG.....GAU...........UGCA.ACACCCGAUU..UCGCUC.....GUCGU..CCCGUCG.....CAAA..............UGACGGUCGGCGAG............G.GGGA......GCGCGUUACC.....GUCAGGA
env-182   CCUGAC.GGGCGCGUAC....GGGUCAAU...UG.CG....GCU..........UGACA.ACACCCGA....UCUCGCUUG..UCGUC.CCUCCG.......AAAA................UGGAGUCGACGA...........GG.GGGA......GCGCGUAACA.....GUCAGGA
env-183   CCUGAU.GGGCGCGUUC....GGGUCAAU...UGCG.....GCU...........UGCA.ACACCCGA....UCUCGCUCG..UCGU.GCCCUCCG......ACA................UGGAGGUCGACGA............G.GGGA......GCGCGUGACC.....GUCAGGA
env-184   UCUGAC.GGGCGUGUCC....GGGUCAAU...UGCG.....GAC...........UGCA.ACACCCGA....UCUCCCUCGU.CGUU..CCCUCCG......GAA................UGGAGGUCGAG.G..........AGG.GGGA......GCGCGUGACC.....GUCAGUA
env-185   CCUGAU.CAACGCGUGC....GGGUCAAU...UGCG.....GCU...........UGCA.ACACCCGG....UCUCCCUCAA.CACG....CCUCC......CGGUAU.............GGAGGUCGCCGAG............G.GGGA......ACGCGUGACC.....GUCAGGA
Hdj-1-1   CCUGAA.GGGCGCGUCC....GGGUCAAU...UGCG.....GAC...........UGCA.ACACCCGA....UCUCCCUC...AACGUC..CCUCCG.....AAAA..............UGGAGGUCGACGAG............G.GGGA......GCGCGUGACC.....GUCAGGA
Hsa-10-1  CCUGAA.GGGCGCGUCC....GGGUCAAU...UGCG.....GAC...........UGCA.ACACCCGA....UCUCCCUC...AAUGUC..CCUCCG.....AAAA..............UGGAGGUCGACGAG............G.GGGA......GCGCGUGACC.....GUCAGGA
Nsu-1-1   CCUGAA.GGGCGCGUCC....GGGUCAAU...UGCG.....GCU...........UGCA.ACACCCGA....UCUCCCUC...AACGUC..CCUCCG.....AAAA..............UGGAGGUCGACGAG............G.GGGA......GCGCGUGACC.....GUCAGGA
Nba-1-1   CCUGAA.GGGCGCGUCC....GGGUCAAU...UGCG.....GCU...........UGCA.ACACCCGA....UCUCCCUCA..ACGUU..CCUCCG......AAAA...............UGGAGGUCGACGA...........GG.GGGA......GCGCGUGACC.....GUCAGGA

          .<<<<<....<<<<<<.....<<<<...<...<<<<<<<<<.........>>>>>>>>>.>.>>>>......<<<<<......<<<<<<<<<<<<<<<<<<<.............>>>>>>>>>>>>>>>>>>>............>.>>>>.....>>>>>>..........>>>>>..
          .20.......22222......001...2...22221110..........01112222.2.100.......22222......222222..2.2.1111110.............0111111.2.2..222222............2.2222.....22222...........02..
          YCUGAY-∘R••RCGY∘Y----GGGU•RAY---••∘○-----•••○----------○R••-R∘RCCCR•----UYYC•------••∘∘○-------------•••∘---------------------○○∘∘○•-----------R-GRRR-----GCGY••••○○----RUCAGGR
           5'                                                                                                                                                                             3'
```

```
Nin-1-1   CCUGAA.GGGCGCGUCC....GGGUCAAU...UGCG.....GCU..........UGCA.ACACCCGA....UCUCCCUCAA.CACG....CCUCCG.....GAUA..............UGGAGGUCUCCGAG............G.GGGA.....GCGCGUGACC.....GUCAGGA
env-186   CCUGAC.GGGCGCGUAC....GGGUCAAU...UGCG.....GCU..........UGCA.ACACCCGA....UCUCGCUC...GUCGUC..CCUCCG.....CAAA..............UGGAGGUCGACGAG............G.GGGA.....GCGCGUAACA.....GUCAGGA
env-187   CCUGAC.GGGCGCGUAC....GGGUCAAU...UGCG.....GCU..........UGCA.ACACCCGA....UCUCGCUU...GUCGUC..CCUCCG.....AAAAA.............UGGAGGUCGACGAG............G.GGGA.....GCGCGUAACA.....GUCAGGA
env-188   CCUGAC.GGGCGCGUAC....GGGUCAAU...UGCG.....GCU..........UGCA.ACACCCGA....UCUCGCUG...GUCGUC..CCUCCG.....AAAA..............UGGAGGUCGACGAG............G.GAGA.....GCGCGUAACA.....GUCAGGA
env-189   CCUGAC.GGGCGCGUAC....GGGUCAAU...UGCG.....GCU..........UGCA.ACACCCGA....UCUCGCUG...GUCGUC..CCUCCG.....AAAA..............UGGAGGUCGACGAG............G.GAGA.....GCGCGUAACA.....GUCAGGA
env-190   CCUGAA.GGGCGCGUCC....GGGUCAAU...UGCG.....GAC..........UGCA.ACACCCGA....UCUCCCU....CAACA.CGCCUCC......CGGAAU.............GGAGGUCGACGAG............G.GAGA.....GCGCGUGACC.....GUCAGGA
env-191   CCUGAA.GGGCGCGUCC....GGGUCAAU...UGCG.....GAC..........UGCA.ACACCCGA....UCUCCCU....CAACA.CGCCUCC......CGGAAU.............GGAGGUCGACGAG............G.GAGA.....GCGCGUGACC.....GUCAGGA
Hla-1-1   CCUGAA.GGGCGCGUCC....GGGUCAAU...UGCG.....GAA..........UGCA.ACACCCGA....UCUCCCU....CAACAC.CCCUCC......UGACAU.............GGAGGUCGAAGAG............G.GGGA.....GCGCGUGACC.....GUCAGGA
env-193   CCUGAA.GGGCGCGUCC....GGGUCAAU...UGCG.....GCU..........UGCA.ACACCCAA....UCGCUCUCAA.CACA....CCUCC......CGGAAU.............GGAGGUCGCUGAG............G.GCGA.....GCGCGUGACC.....GUCAGGA
Hac-1-1   UCUGAA.GGGCGCGUUC....GGGUCAAU...UGCG.....GCA..........UGCA.ACACCCGA....UCUCC......CUCC....ACCUCC.....CCGGCGAUAU.........GGAGGUUUCGGAG............G.GGGA.....GCGCGUGACC.....AUCAGGG
Hac-1-2   UCUGAA.GGGCGCGUUC....GGGUCAAU...UGCG.....GCA..........UGCA.ACACCCGA....UCUCC......CUCC....ACCUCC.....CCGGCGAUAU.........GGAGGUUUCGGAG............G.GGGA.....GCGCGUGACC.....AUCAGGG
env-196   UCUGAA.GGGCGCGUUC....GGGUCAAU...UGCG.....GCA..........UGCA.ACACCCGA....UCUCCCUC...CACCA..GCCUCC......CGGUAU.............GGAGGUUGUGGAG............G.GGGA.....GCGCGUGACC.....AUCAGGG
env-197   UCUGAA.GGGCGCGUUC....GGGUCAAU...UGCG.....GCA..........UGCA.ACACCCGA....UCUCCCUC...CACCA..GCCUCC......CGGUAU.............GGAGGUUGUGGAG............G.GGGA.....GCGCGUGACC.....AUCAGGG
env-198   UCUGAA.GGGCGCGUUC....GGGUCAAU...UGCG.....GCA..........UGCA.ACACCCGA....UCUCCCUC...CACCA..GCCUCC......CGGUAU.............GGAGGUUGUGGAG............G.GGGA.....GCGCGUGACC.....AUCAGGG
Hsp-2-1   UCUGAA.GGGCGCGUUC....GGGUCAAU...UGCG.....GCA..........UGCA.ACACCCGA....UCUCCCUC...CACCA..GCCUCC......CGGUAU.............GGAGGUUGUGGAG............G.GGGA.....GCGCGUGACC.....AUCAGGG
env-199   CCUGAU.GGGCGCGUUC....GGGUCAAU...UGCG.....GCC..........UGCA.ACACCCGA....UCUCA......CUCGUGACCACC.......GACGA...............GGUGG..ACGAG............G.GGGA.....GCGCGUGACC.....GUCAGGA
Hsu-1-1   UCUGAC.GGGCGCGUUC....GGGUCAAU...UGCG.....GAU..........UGCA.ACACCCAA....UCUCCCUC...CAC.GUC.CCGCC......CUCGAU.............GGCGGUCGCGGAG............G.GGGA.....ACGCGUGACC.....AUCAGGG
env-201   GCUGAU.GGGCGCGUCC....GGGUCAAU...UGCG.....GUA..........UGCA.ACACCCGA....UCUCC......CU..UCC.CGUCC......CAGCGGAUAU.........GGACGUGGACCAG............G.GGGA.....GCGCGUGACC.....GUCAGGG
env-203   GCUGAU.GGGCGCGUCC....GGGUCAAU...UGCG.....GUA..........UGCA.ACACCCGA....UCUCC......CU..UCC.CGUCC......CAGCGGAUAU.........GGACGUGGACCAG............G.GGGA.....GCGCGUGACC.....GUCAGGG
Hca-1-1   GCUGAU.GGGCGCGUCC....GGGUCAAU...UGCG.....GCA..........UGCA.ACACCCGA....UCUCC......CU..UCC.CGUCC......CAGCGGAUAU.........GGACGUGGAACAG............G.GAGA.....GCGCGUGACC.....GUCAGGG
Ham-1-1   GCUGAU.GGGCGCGUCC....GGGUCAAU...UGCG.....GCA..........UGCA.ACACCCGA....UCUCC......CU..UCC.CGUCC......CAGCGGAUAU.........GGAUGUGGAACAG............G.GAGA.....GCGCGUGACC.....GUCAGGG
Hhi-2-1   GCUGAU.GGGCGCGUCC....GGGUCAAU...UGCG.....GCA..........UGCA.ACACCCGA....UCUCU......CU..UCC.CGUCC......CAGCGGAUAU.........GGACGUGGAACAG............G.GGGA.....GCGCGUGACC.....GUCAGGG
Hsa-2-1   GCUGAU.GGGCGCGUCC....GGGUCAAU...UGCG.....GCA..........UGCA.ACACCCGA....UCUCC......CU..UCC.CGUCC......CAGCGGAUAU.........GGACGUGGAACAG............G.GGGA.....GCGCGUGACC.....GUCAGGG
Har-1-1   GCUGAU.GGGCGCGUCC....GGGUCAAU...UGCG.....GCA..........UGCA.ACACCCGA....UCUCC......CU..UCC.CGUCC......CAGCGGAUAU.........GGACGUGGAACAG............G.GGGA.....GCGCGUGACC.....GUCAGGG
Hva-1-1   GCUGAU.GGGCGCGUCC....GGGUCAAU...UGCG.....GCA..........UGCA.ACACCCGA....UCUCC......CU..UCC.CGUCC......CAGCGGAUAU.........GGACGUGGAACAG............G.GGGA.....GCGCGUGACC.....GUCAGGG
Hja-1-1   GCUGAU.GGGCGCGUCC....GGGUCAAU...UGCG.....GCA..........UGCA.ACACCCGA....UCUCC......CU..UCC.CGUCC......CAGCGGCUAU.........GGACGUGGAACAG............G.GGGA.....GCGCGUGACC.....GUCAGGG
env-204   CCUGAA.GGCCGCGUUC....GGGUCACU...UGCG.....GAC..........UGCA.ACACCCGA....UCUCC......CUCGGCGU..GCCGCCG..GAGA...........UGGCGACACCCGCCGAG............G.GGGA.....GCGCGUCACC.....GUCAGGA
Hse-1-1   CCUGAU.GGGCGCGUUC....GGGUCAAU...UGCG.....GAC..........UGCA.ACACCCGA....UCUCG......CUCGUCGUGCCUCC.....CGAGAU............GGAGGU.CGACGAG............G.GGGA.....GCGCGUGACC.....GUCAGGA
Hth-1-1   CCUGAU.GGGCGCGUUC....GGGUCAAU...UGCG.....GAC..........UGCA.ACACCCGA....UCUCG......CUCGUCGCACCGCCG....GAGA.............UGGCGGU.CGACGAG............G.GGGA.....GCGCGUGACC.....GUCAGGA
Hmo-1-1   CCUGAU.GGGCGCGUUC....GGGUCAAU...UGCG.....GAC..........UGCA.ACACCCGA....UCUCG......CUCGUCGCACCGCCG....GAGA.............UGGCGGU.CGACGAG............G.GGGA.....GCGCGUGACC.....GUCAGGA
env-206   CCUGAC.CGGCGCGUCC....GGGUCAAU...UGCG.....GCA..........UGCA.ACACCCGAUC..UCCCU......CGUCGCCCCGCC.......CGAAAC.............GGCGAU.CGACG...........AGG.GGGA.....GCGCGUGACA.....GUCAGGA
env-207   CCUGAC.GGCCGCGUUC....GGGUCACU...UGCG.....GAC..........UGCA.ACACCCGA....UCUCG......CUCCGUACCACG.......CGCGA...............CGUG..GUGGAG............G.GGGA.....GCGCGUCACC.....GUCAGUG
env-209   CCUGAA.GGCCGCGUUC....GGGUCACU...UGCG.....GAC..........UGCA.ACACCCGA....UCUCG......CUCCUCGUCCCCCCCG...AGA.............UGGCGGUC..GAGGAG............G.GAGA.....GCGCGUCACC.....AUCAGGA
Nta-1-1   CCUGAU.CAACGCGUGC....GGGUCAAU...UGCG.....GCU..........UGCA.ACACCCGA....UCUCC......CUCAACGUGCCGCCG....AAAA.............UGGCG.CUCGACGAG............G.GCGA.....GCGCGUGACU.....AUCAGGA
Nas-1-1   CCUGAU.CAACGCGUGC....GGGUCAAU...UGCG.....GCU..........UGCA.ACACCCGA....UCUCC......CUCAACGUGCCGCCG....AAAA.............UGGCG.CUCGACGAG............G.GCGA.....GCGCGUGACU.....AUCAGGA
Noc-1-1   CCUGAU.CAACGCGUGC....GGGUCAAU...UGCG.....GCU..........UGCA.ACACCCGA....UCUCC......CUCAACGUGCCUCC.....CUCGAU............GGAG.CUCGAAGAG............G.GGGA.....GCGCGUGACC.....GUCAGGA
Nje-1-1   CCUGAU.CAACGCGUGC....GGGUCAAU...UGCG.....GCU..........UGCA.ACACCCGA....UCUCC......CUCAACGUGCCUCC.....CGGUAU............GGAG.CUCGAGGAG............G.GGGA.....GCGCGUAACC.....GUCAGGG
Nam-1-1   CCUGAU.CAACGCGUGC....GGGUCAAU...UGCG.....GCU..........UGCA.ACACCCGA....UCUCC......CUCAACGUGCCUCC.....CGGUAU............GGAG.CUCGAGGAG............G.GGGA.....GCGCGUGACC.....GUCAGGA
env-210   CCUGAU.CAACGCGUCC....GGGUCAAU...UGCG.....GCU..........UGCA.ACACCCGA....UCUCC......CUCAACGUUCCGCCA....GGAA.............UGGCG.GUCGACGAG............G.GAGA.....GCGCGUGACC.....GUCAGGA
Hsp-3-1   UCUGAA.GGGCGCGUUC....GGGUCAAU...UGCG.....GCA..........UGCA.ACACCCGA....UCUCC......CUCCACGAACCACCA....GGAA.............UGGCGG.UCUCGGAA............G.GAGA.....GCGCGUGACC.....AUCAGGG
Nch-1-1   CCUGAU.CAACGCGUGC....GGGUCAAU...UGCG.....GCU..........UGCA.ACACCCGA....UCUCC......CUCAACGUACCGCCG....AAAA.............UGGCGGU.CUCCGUG............G.GAGA.....GCGCGUGACU.....AUCAGGA
Nae-1-1   CCUGAU.CAACGCGUGC....GGGUCAAU...UGCG.....GCU..........UGCA.ACACCCGA....UCUCC......CUCAACGUGCCGCCG....AAAA.............UGGCGGU.CGACGAG............G.GCGA.....GCGCGUGACU.....AUCAGGA
env-214   CCUGAU.CAACGCGUGC....GGGUCAAU...UGCG.....GCU..........UGCA.ACACCCGA....UCUCC......CUCAACGUGCCUCC.....CGGUAU............GGAGGU.CGACGAG............G.GGGA.....GCGCGUGACU.....GUCAGGA
env-215   CCUGAU.CAACGCGUGC....GGGUCAAU...UGCG.....GCU..........UGCA.ACACCCGA....UCUCC......CUCAACGUGCCUCC.....CGGUAU............GGAGGU.CGACGAG............G.GGGA.....GCGCGUGACU.....GUCAGGA
env-216   CCUGAA.GGGCGCGUCC....GGGUCAAU...UGCG.....GAC..........UGCA.ACACCCGA....UCUCC......CUCAAUACGCCUCC.....CGGAAU............GGAGGU.GGCUGAG............G.GAGA.....GCGCGUGACC.....GUCAGGG
Hni-1-1   CCUGAA.GGGCGCGUCC....GGGUCAAU...UGCG.....GCA..........UGCA.ACACCCGA....UCUCC......CUCAAUACACCUCC.....CGACAU............GGAGGU.CUCGGAG............G.GGGA.....GCGCGUGACC.....GUCAGGA
Has-1-1   CCUGAA.GGCCGCGUCC....GGGUCAAU...UGCG.....GAC..........UGCA.ACACCCGA....UCUCC......CUCAACGUGCCACCG....AAAA.............UGGUGGU.CGAUGAG............G.GGGA.....GCGCGUGACC.....GUCAGGA
env-217   CCUGAA.GGGCGCGUCC....GGGUCAAU...UGCG.....GAC..........UGCA.ACACCCGA....UCUCC......CUCAACAUACCGCCG....AACA.............UGGCGGU.CGAAGAG............G.GGGA.....GCGCGUGACC.....GUCAGGA
Nal-2-1   CCUGAA.GGGCGCGUCC....GGGUCAAU...UGCG.....GAC..........UGCA.ACACCCGA....UCUCC......CUCAACGUACCGCCG....AAAA.............UGGCGGU.CGAAGAG............G.GGGA.....GCGCGUGACC.....GUCAGGA
Npa-1-1   CCUGAA.GGGCGCGUCC....GGGUCAAU...UGCG.....GAC..........UGCA.ACACCCGA....UCUCC......CUCAACGUACCGCCG....AAAA.............UGGCGGU.CGAAGAG............G.GGGA.....GCGCGUGACC.....GUCAGGA
Nga-1-1   CCUGAA.GGGCGCGUCC....GGGUCAAU...UGCG.....GAC..........UGCA.ACACCCGA....UCUCC......CUCAACGUACCGCCG....AAAA.............UGGCGGU.CGAAGAG............G.GGGA.....GCGCGUGACC.....GUCAGGA
Nsp-2-1   CCUGAA.GGGCGCGUCC....GGGUCAAU...UGCG.....GAC..........UGCA.ACACCCGA....UCUCC......CUCAACGUACCGCCG....AAAA.............UGGCGGU.CGAAGAG............G.GGGA.....GCGCGUGACC.....GUCAGGA
Htu-1-1   CCUGAA.GGGCGCGUCC....GGGUCAAU...UGCG.....GAU..........UGCA.ACACCCGA....UCUCC......CUCAACGUACCGCCG....AAAA.............UGGCGGU.CGAGGAG............G.GGGA.....GCGCGUGACC.....GUCAGGA
Hsa-8-1   CCUGAA.GGGCGCGUCC....GGGUCAAU...UGCG.....GAC..........UGCA.ACACCCGA....UCUCC......CUCAACGUACCGCCG....AAAA.............UGGCGGU.CGACGAG............G.GGGA.....GCGCGUGACC.....GUCAGGA
Hth-2-1   CCUGAA.GGGCGCGUCC....GGGUCAAU...UGCG.....GAC..........UGCA.ACACCCGA....UCUCC......CUCAACGUACCGCCG....AAAA.............UGGCGGU.CGAGGAG............G.GGGA.....GCGCGUGACC.....GUCAGGA
Nve-1-1   CCUGAA.GGGCGCGUCC....GGGUCAAU...UGCG.....GAC..........UGCA.ACACCCGA....UCUCC......CUCAACGUACCGCCG....AAAA.............UGGCGGU.CGAGGAG............G.GGGA.....GCGCGUGACC.....GUCAGGA
Hli-3-1   CCUGAA.GGGCGCGUCC....GGGUCAAU...UGCG.....GAC..........UGCA.ACACCCGA....UCUCC......CUCAACACACCUCC.....UGACAU............GGAGGU.CGACGAG............G.GAGA.....GCGCGUGACC.....GUCAGGA
Hxa-1-1   CCUGAA.GGGCGCGUCC....GGGUCAAU...UGCG.....GAC..........UGCA.ACACCCGA....UCUCC......CUCAACACGCCUCCG....GAUA.............UGGAGGU.CGACGAG............G.GGGA.....GCGCGUGACC.....GUCAGGA
Hgo-1-1   CCUGAU.CAUCGCGUCC....GGGUCAAU...UGCG.....GCU..........UGCA.ACACCCGA....UCUCG......CUCAACGUACCGCC.....CGAAAU............GGCGGU.CGCUGAG............G.GAGA.....ACGCGUGACA.....GUCAGGA
env-218   CCUGAU.CAUCGCGUCC....GGGUCAAU...UGCG.....GCU..........UGCA.ACACCCGA....UCUCC......CUCAACGUACCACCA....GAAA.............UGGCGGU.CGAGGAG............G.GAGA.....ACGCGUGACA.....GUCAGGA
env-219   CCUGAU.CGGCGCGUCC....GGGUCAAU...UGCG.....GAU..........UGCA.ACACCCGA....UCUCC......CUCACUGUGCCGUCA....GCAC.............UGACGGU.CGCCGAG............G.GUGA.....GCGCGUGACG.....AUCAGGA
env-220   UCUGAC.GGGCGUGUCC....GGGUCAAU...UGCG.....GAC..........UGCA.ACACCCGA....UCUCC......CUCGUUGUUCCCUCCG...GAA.............UGGAGGU.....CGAG.........GCGG.GAGA.....ACGCGUGACC.....GUCAGUA
env-221   CCUGAA.GGGCGCGUCC....GGGUCAAU...UGCG.....GCU..........UGCA.ACACCCGA....UCUCC......CUCAACGUUCCGCCG....GGAA.............UGGCGGUCG.AAGAG............G.GGGA.....GCGCGUGACC.....GUCAGGA
Hsa-6-1   CCUGAA.GGCCGCGUCC....GGGUCAAU...UGCG.....GAA..........UGCA.ACACCCGA....UCUCCCUCA..UCACCC.CCGACG......GAA................UGUCGGUGGGCGA...........GG.GAGA.....GCGCGUCACC.....GUCAGGA
env-222   UCUGAC.CGACGCGUCC....GGGUCAAU...UGCG.....GAC..........UGCA.ACACCCUAU...CUCCC......UUCCCGUCCCGCCG.....UCGA..............UGGU..GGUCGAGA...........AG.GGCGA....ACGCGUCACU.....GUCAGGG
env-223   UCUGAU.GGGCGCGUCC....GGGUCAAU...UGCG.....GCU..........UGCA.ACACCCGA....UCUUGC.....UGCCGGU.GCCA.......GACGACA.............UGGCCGCCGGCA..........AGC.GGGA.....GCGCGUGACC.....GUCAGGG
env-224   GCUGAC.GGGCGCGUCC....GGGUCAAU...UGCG.....GCC..........UGCA.ACACCCAA....UCUCGC.....UGCCGG.UGCCGG......AUGGA..............UUGGCAGUCGGCG..........AGC.GCGA.....GCGCGUCACC.....GUCAGGG
env-225   CCUGAU.AAUCGCGUUU....GGGUAAUU...UGCG.....GAC..........UGCA.AAACCCAAUU..UCCCU......AACAAUCCCCA........CUAAUA...............UGGAUAUUGUA...........GA.GGGACA...ACGCGUUUCU.....GUCAGAA
env-226   CCUGAU.AAUCGCGUUU....GGGUAAUU...UGUG.....GAC..........UGCA.AAACCCAAUU..UCCCU......AACAAUUCCCA........CUAAUA...............UGGAUAUUGUA...........GA.GGGACA...ACGCGUUUCU.....GUCAGAA
env-227   CCUGAU.AAUCGCGUUU....GGGUAAUU...UGUG.....GAC..........UACA.AAGCCCAA....UUUCUC.....UC.ACAAUACCUCC.....UAACAU............GGA..UGUUGUCGA...........UG.GACA.....ACGCGUUUCU.....GUCAGAG
env-228   CCUGAU.AACCGCGUUC....GGGUCAAU...UGUG.....AAU..........UGCA.ACACCCAGUCUCCCUUCAUGU..GCCAGA.............AUCAAUA...................UCUGUC.........CGAG.AAGGAAC..ACGCGUUGCU.....AUCAGGA
env-231   UCUGAA.CGUGGCGUAAA...GGGUGGAC...GUAAACCC.AGAU.....GGG.UUAU.GUGCCCAU....UCCCU......GCCAU..............CCCA.......................AUGGC..........CAG.GUGA.....ACGCCCAC.......AUCAGGA
env-232   UCUGAA.CGUGGCGUAAA...GGGU.GAC...GUAAACCC.AGAU.....GGG.UUAU.GUGCCCAU....UCCCU......GCCAU..............CCCA.......................AUGGC..........CAG.GUGA.....ACGCCCAC.......AUCAGGA
env-233   UCUGAA.CGUGGCGUAAA...GGGUGGAC...GUAAACCC.CAGAU....GGG.UUAU.GUGCCCAU....UCCCU......GCCAU..............CCCA.......................AUGGC..........CAG.GUGA.....ACGCCCAC.......AUCAGGA
env-237   UCUGAU.GAGUGUGUCAU...GGGUGGAC...G........AUACCAAAA.......C.G.GCCCG.....CUCUG......AAAAG..............ACAAUUGC...................UCUUU...........AC.AGGG....GACACAUAAGU.....AUCAGGA
env-238   UCUGAC.GACCGCGUAU....GGGUCGAU...GUCG.....GUCU.........CGAC.A.GCCCAU....CACUC......GCAAAAG............GUAAAG...................CAAAGGU............G.GGUGAAC..GCGUGACA.......GUCAGGU
env-240   UCUGAC.CGGAGCGUUC....GGGUCGAU...GUCG.....GUAU.........CGAC.A.GCCCAAU...CUCCCUGG...AGACGCCU...........GAAA....................GGGCG.CU.......GAAGAG.GGAGA....GCGCAUAUCA.....GUCAGAA
env-241   UCUGAC.ACAGUCACAU....GGGUCGAU...AUU......GGUGUA........GAU.A.GCCCGG....UUCCCCUCAA.ACCUUCC............UCAA.....................GGAAGGU......GCAAAAG.GGCGAGC..GUGUCAAU.......GUCAGGA
env-242   ACUGAU.GUGUGCGCUCAU.GGGCCAAC...GUU......GAAG..........AAC.GUGCCCGG....UUCAC......CGU................GAAA.........................ACG...........GG.AGAA....AGCGCCCCA.......GUCAGAA
env-243   ACUGAU.GUGUGCGCUCAU.GGGCCAAC...GUU......GAAG..........AAC.GUGCCCGG....UUCAC......CGU................GAAA.........................ACG...........GG.AGAA....AGCGCCCCA.......GUCAGAA
env-244   UCUGAU...AAGCGCAC....GGGUGGAC...GUG......GUCUCUA.......CAC.GAGCCCGC....AUGUG......CCGC...............AGG.........................GCGG............U.ACAUAU...GCGCGAGA.......CUCAGUA
env-245   ACUGAU...CAGCGC.AG...GGGUGGAC...GUGC.....UCU..........GCAC.GAGCCCGC....GUGUA......UCAGCA.............UCA.......................UACUGG............U.ACAUCC...GCGCGAUA.......GUCAGGU
env-246   ACUGAU...CAGCGC.AG...GGGUGGAC...GUGC.....UCU..........GCAC.GAGCCCGC....GUGUA......UCAGCA.............UCA.......................UACUGG............U.ACAUCC...GCGCGAUA.......GUCAGGU

          .<<<<<....<<<<<<.....<<<<...<...<<<<<<<<<.........>>>>>>>>>.>.>>>>......<<<<<.....<<<<<<<<<<<<<<<<<<<<............>>>>>>>>>>>>>>>>>>...........>.>>>>.....>>>>>>..........>>>>>..
          .20......22222......001...2...22221110..........01112222.2.100......22222.....222222..2.2.1111110............0111111.2.2..222222...........2.2222.....22222..........02..
          YCUGAY-○R●●RCGY○Y----GGGU●RAY---●●○○------●●●○-----------○R●●-R○RCCCR●----UYYC●------●●○○○--------------●●●○---------------------○○○○○●------------R-GRRR------GCGY●●●●○○----RUCAGGR
          5′                                                                                                                                                                                  3′
```

```
env-247   ACUGAU...CAGCGC.AG...GGGUGGAC...GUGC.....UCU...........GCAC.GAGCCCGC....GUGUA......UCAGCA.............UCA.......................UACUGG...........U.ACAUCC....GCGCGAUA.......GUCAGGU
env-248   ACUGAU...CAGCGC.AG...GGGUGGAC...GUGC.....UCU...........GCAC.GAGCCCGC....GUGUA......UCAGCA.............UCA.......................UACUGG...........U.ACAUCC....GCGCGAUA.......GUCAGGU
env-249   UCUGAU..AUCACGCAAA...GGGUGGAC...GUCUU....CCC..........GGAAC.GAGCCCGU....UUCUG......CCA................UGC..........................UGG...........C.AGAAAAC...GCGUGAAA.......GUCAGGA
env-250   UCUGAU..AUCACGCAAA...GGGUGGAC...GUCUU....CCC..........GGAAC.GAGCCCGU....UUUUG......CC.................AUACA.........................GG...........C.AGAAAAC...GCGUGAAC.......AUCAGGA
env-251   UCUGAUUGAACGCGC.C....GGGCGAAC...GU.......GAAA............AC.GAGCCCAC....UUUCU......GC.................AUA...........................GC..........CG.GAAA......GCGCGUCCGG.....GUCAGGA
env-252   UCUGAUUGAACGCGC.C....GGGCGAAC...GU.......GAAA............AC.GAGCCCAC....UUUCU......G..................AAUACA.........................C...........A.GAAA......GCGCGUCCGG.....GUCAGGA
env-253   UCUGAUUGAACGCGC.C....GGGCGAAC...GU.......GAAA............AC.GAGCCCAC....UUUCU......GA.................ACUA..........................UC...........A.GAAA......GCGCGUCAGG.....GUCAGGA
env-254   ACUGAU.CAAGCCGCCU....GGGUCGAC...GUUG.....GUG...........CAAC.GAGCCCUG....UUCCU......AUGGCCG............AUGUAA...................UGCCCAU...........A.GGAAG.....GUGGAUCACG.....GUCAGAA
env-255   UCUGAU..CAGGCGUAUU...GGGCGAAC...GUU......CGUGC..........AAC.GAGCCCUU....CUUCA......UACAGC.............CAUUAAU...................GAUGUG..........AU.GAAGAU....GCGCCAUU.......GUCAGGA
env-256   UCUGAU.CGAGACGA.U....GGGCCAAC...GC.......CAGUGCA.........GC.GCGCCCGG....UUCCG......GGAA...............CACUC.......................UUUC...........C.GGAA......ACGUCGGAGA.....AUCAGGA
env-257   UCUGAU.CGAGACGA.U....GGGCCAAC...GC.......CAGUGCA.........GC.GCGCCCGG....UUCCG......GGAA...............CACUC.......................UUUC...........C.GGAA......ACGUCGGAGA.....AUCAGGA
env-258   UCUGAU.CGAGACGA.U....GGGCCAAC...GC.......CAGUGCA.........GC.GCGCCCGG....UUCCG......GGAA...............CACUC.......................UUUC...........C.GGAA......ACGUCGGAGA.....AUCAGGA
env-259   UCUGAU.CGAGACGA.U....GGGCCAAC...GC.......CAGUGCA.........GC.GCGCCCGG....UUCCG......GGAA...............CACUC.......................UUUC...........C.GGAA......ACGUCGGAGA.....AUCAGGA
env-260   UCUGAU.CGAGACGA.U....GGGCCAAC...GC.......CAGUGCA.........GC.GCGCCCGG....UUCCG......GGAA...............CACUC.......................UUUC...........C.GGAA......ACGUCGGAGA.....AUCAGGA
env-261   UCUGAU.CGAGACGC.U....GGGCCAAC...GC.......CUGUGCA.........GC.GUGCCCGA....UUCCA......GGAGA..............ACG........................UUGCC...........U.GGGA......GCGUCGAAGA.....AUCAGGA
env-265   UCUGAU.CGAGACGC.U....GGGCCAAC...GC.......CAGUGCA.........GC.GCGCCCGG....UUCC.......GGGGA..............GAAUG......................UCCCC..........   GGAA......GCGUCGAAGU.....GUCAGGA
env-266   UCUGAU.CGAGACGC.U....GGGCCAAC...GC.......CAGUGCA.........GC.GCGCCCGG....UUUC.......CGGGA..............UAAAUC.....................UUCCC..........   GGGA......GCGUCAGAGA.....GUCAGGA
env-268   UCUGAU.CGAGACGC.U....GGGCCAAC...GC.......CAGUGCA.........GC.GCGCCCGG....UUUC.......CGGGA..............UAAAUC.....................UUCCC..........   GGGA......GCGUCAGAGA.....GUCAGGA
env-269   UCUGAU.CGAGACGC.U....GGGCCAAC...GC.......CAGUGCA.........GC.GCGCCCGG....UUUC.......CGGGA..............UAAAUC.....................UUCCC..........   GGGA......GCGUCAGAGA.....GUCAGGA
env-270   UCUGAU.CGAGACGC.U....GGGCCAAC...GC.......CAGUGCA.........GC.GCGCCCGG....UUUC.......CGGGA..............UAAAUC.....................UUCCC..........   GGGA......GCGUCAGAGA.....GUCAGGA
env-271   UCUGAU.CGAGACGU.U....GGGCCAAC...GC.......CAGUGCA.........GC.GCGCCCGG....UUCC.......AGAGA..............ACA........................UCUCU.........CC. GGAA......ACGUCAAAGA.....GUCAGGA
env-272   UCUGAU.CGAGACGU.U....GGGCCAAC...GC.......CAGUGCA.........GC.GCGCCCGG....UUCC.......AGAGA..............ACA........................UCUCU.........CC. GGAA......ACGUCAAAGA.....GUCAGGA
env-274   UCUGAU.CGAGACGU.U....GGGCCAAC...GU.......CAGUGCA.........GC.GCGCCCGG....UUCC.......AGAGA..............ACA........................UCUCU.........CC. GGAA......ACGUCAAAGA.....GUCAGGA
env-275   UCUGAU.CGAGACGU.U....GGGCCAAC...GC.......CAGUGCA.........GC.GCGCCCGG....UUCC.......AGAGA..............ACA........................UCUCU.........CC. GGAA......ACGCCAAAGA.....GUCAGGA
env-277   UCUGAU.CGAGACGU.U....GGGCCAAC...GC.......CAGUGCA.........GC.GCGCCCGG....UUCC.......AGAGA..............ACA........................UCUCU.........CC. GGAA......ACGCCAAAGA.....GUCAGGA
env-278   UCUGAU.CGAGACGU.U....GGGCCAAC...GC.......CGGUGCA.........GC.GCGCCCGG....UUCC.......GGGAA..............CAGGCU.....................UUCCC..........   GGAA......ACGUUGAAGA.....AUCAGGA
env-292   UCUGAU.CGAGACGU.U....GGGCCAAC...GC.......CGGUGCA.........GC.GAGCCCGU....UUCCG......GGAA...............CAAGU.......................UUCC...........C.GAAA......GCGUCGAAGA.....GUCAGGA
env-293   UCUGAU.CGAGACGU.U....GGGCCAAC...GC.......CGGUGCA.........GC.GAGCCCGU....UUCCG......GGAA...............CAAGU.......................UUCC...........C.GAAA......GCGUCGAAGA.....GUCAGGA
env-295   UCUGAU.CGAGACGU.U....GGGCCAAC...GC.......CGGUGCA.........GC.GAGCCCGU....UUCCG......GGAA...............CAAGU.......................UUCC...........C.GAAA......GCGUCGAAGA.....GUCAGGA
env-296   UCUGAU.CGAGACGU.U....GGGCCAAC...GC.......CGGUGCA.........GC.GAGCCCGU....UUCCG......GGAA...............CAAGU.......................UUCC...........C.GAAA......GCGUCGAAGA.....GUCAGGA
Mbo-1-1   UCUGAU.CGAGACGU.U....GGGCCAAC...GC.......CGGUGCA.........GC.GAGCCCGU....UUCCG......GGAA...............CAAGU.......................UUCC...........C.GAAA......GCGUCGAAGA.....GUCAGGA
env-297   UCUGAU.CGAGACGU.U....GGGCCAAC...GC.......CGGUGCA.........GC.GAGCCCGU....UUCC.......GGGAA..............CAGGU......................UUCCC..........   GAAA......GCGUCGAAGA.....GUCAGGA
env-298   UCUGAU.CGAGACGU.U....GGGCCAAC...GC.......CGGUGCA.........GC.GAGCCCGU....UUCC.......GGGAA..............CAGGU......................UUCCC..........   GAAA......GCGUCGAAGA.....GUCAGGA
env-303   UCUGAU.CGAGACGU.U....GGGCCAAC...GC.......CGGUGCA.........GC.GAGCCCGU....UUCC.......GGGAA..............CAGGU......................UUCCC..........   GAAA......GCGUCGAAGA.....GUCAGGA
env-304   UCUGAU.CGAGACGU.U....GGGCCAAC...GC.......CGGUGCA.........GC.GAGCCCGU....UUCC.......GGGAA..............CANGU......................UUCCC..........   GAAA......GCGUCGAAGA.....GUCAGGA
env-305   UCUGAU.CGAGACGU.U....GGGCCAAC...GC.......CAGUGCA.........GC.GCGCCCGG....UUCCA......GAGA...............AGAAUAUC....................UCUC...........C.GGAA......ACGUCGAAGA.....GUCAGGA
env-306   UCUGAU.CGAGACGU.U....GGGCCAAC...GC.......CAGUGCA.........GC.GCGCCCGG....UUCCA......GAGA...............AGAAUAUC....................UCUC...........C.GGAA......ACGUCGAAGA.....GUCAGGA
Mma-1-1   UCUGAU.CGAGACGU.U....GGGCCAAC...GC.......CAGUGCA.........GC.GCGCCCGG....UUCCG......GGAA...............GAAUG.......................UUCC...........C.GGGA......GCGUCGAAGA.....GUCAGGA
Mse-1-1   UCUGAU.CGAGACGC.U....GGGCCAAC...GC.......CAGUGCA.........GC.GCGCCCGG....UUCCG......GGAA...............GAGUG.......................UUCC...........C.GGGA......GCGUCGAAGA.....GUCAGGA
Msp-1-1   UCUGAU.CGAGACGC.U....GGGCCAAC...GC.......CAGUGCA.........GC.GCGCCCGG....UUCCG......GGAA...............GAGUG.......................UUCC...........C.GGGA......GCGUCGAAGA.....GUCAGGA
env-310   UCUGAU.CGAGACGU.C....GGGCCAAC...GC.......CAGUGCA.........GC.GAGCCCGU....UUCCU.........................GAG........................................A.GGAGA.....GCGUCGAAGA.....GUCAGGA
env-315   UCUGAU.CGAGACGU.C....GGGCCAAC...GC.......CAGUGCA.........GC.GAGCCCGU....UUCCU.........................GAG........................................A.GGAGA.....GCGUCGAAGA.....GUCAGGA
env-316   UCUGAU.CGAGACGU.C....GGGCCAAC...GC.......CAGUGCA.........GC.GAGCCCGU....UUCCU.........................GAG........................................A.GGAGA.....GCGUCGAAGA.....GUCAGGA
env-317   UCUGAU.CGAGACGU.U....GGGCCAAC...GC.......CAGUGCA.........GC.GCGCCCGG....UUCC.......AGAGA..............ACAAUA.....................UCUCU..........CC.GGAA......ACGUCAAAGA.....GUCAGGA
env-318   UCUGAU.CGAGGCGC.A....GGGCCAAC...GC.......CAGUGCA.........GC.GAGCCCGU....UUUCU.........................GAAA.......................................G.GAGA......GCGUCGAAGA.....GUCAGGA
env-320   UCUGAU.CGAGGCGC.A....GGGCCAAC...GC.......CAGUGCA.........GC.GAGCCCGU....UUUCU.........................GAAA.......................................G.GAGA......GCGUCGAAGA.....GUCAGGA
env-321   UCUGAU.CGAGGCGC.A....GGGCCAAC...GC.......CAGUGCA.........GC.GAGCCCGU....UUUCU.........................GAAA.......................................G.GAGA......GCGUCGAAGA.....GUCAGGA
env-322   UCUGAU.CGAGGCGC.A....GGGCCAAC...GC.......CAGUGCA.........GC.GAGCCCGU....UUUCU.........................GAAA.......................................G.GAGA......GCGUCGAAGA.....GUCAGGA
Mpa-1-1   UCUGAA....GGACAGU....GGGUGGAC...GUU......CGUGC..........AAC.GAGCCCGU....UCCUG......CA.................CAC...........................UG...........C.AGGAGAU...UGUCCUG........UUCAGGA
env-323   UCUGAA...GGACAG.U....GGGUGGAC...GUU......CGUGC..........AAC.GAGCCCGU....UCCUG......CA.................CUC...........................UG...........C.AGGAGA....UUGUCCUG.......UUCAGGA
env-324   UCUGAU....ACCGCGCAU..GGGUGGAC...GUU......CGUGC..........AAC.GAGCCCGU....UCCUG......A..................ACAU...........................U...........C.AGGAU.....GCGUUCAU.......UUCAGGA
env-325   UCUGAC.CAGGACGCAU....GGGUCGAC...GUU......CGUGC..........AAC.GAGCCCGU....UCCUUCU....GCUGCA.............UCAG......................UGUAGC...........A.GGGAU.....GCGCCAUU.......GUCAGGA
env-326   UCUGAC.CAGGACGCAU....GGGUCGAC...GUU......CGUGC..........AAC.GAGCCCGU....UCCUUCU....GCUGCA.............UCAG......................UGUAGC...........A.GGGAU.....GCGCCAUU.......GUCAGGA
env-331   UCUGAC.CAGGACGCAU....GGGUCGAC...GUU......CGUGC..........AAC.GAGCCCGU....UCCUUCU....GCUGCA.............UCAG......................UGUAGC...........A.GGGAU.....GCGCCAUU.......GUCAGGA
env-332   UCUGAC.CAGGACGCAU....GGGUCGAC...GUU......CGUGC..........AAC.GAGCCCGU....UCCUUCU....GCUGCA.............UCAG......................UGUAGC...........A.GGGAU.....GCGCCAUU.......GUCAGGA
Mfo-1-1   UCUGAU..CAGGCGCAU....GGGCGAAC...GUU......CGUGC..........AAC.GAGCCCCU....UCUUCGU....GCGGAC.............UAAU......................GUCCGC.........GUG.AAGAU.....GCGCCAUU.......GUCAGGA
env-333   UCUGAC.CGUGACGCAU....GGGUGGAC...GUUU.....CCAUAAA.......AAAC.GAGCCUGU....UCCCC......GG.................AAUUAC........................CC...........G.GGGAU.....GCGUCAAU.......GUCAGGG
env-334   UCUGAC.CGUGACGCAU....GGGUGGAC...GUUUU....CAUAAA.......AAAAC.GAGCCUGU....UCCCC......GG.................AAUAC.........................CC...........G.GGGAU.....GCGUCAAU.......GUCAGGG
env-335   UCUGAC.CGUGACGCAU....GGGUGGAC...GUUUU....CAUAAA.......AAAAC.GAGCCUGU....UCCCC......GG.................AAUAC.........................CC...........G.GGGAU.....GCGUCAAU.......GUCAGGG
env-337   UCUGAC.CGUGACGCAU....GGGUGGAC...GUUUU....CAUAA........AAAAC.GAGCCUGU....UCCCC......GG.................AAUAC.........................CC...........G.GGGAU.....GCGUCAAU.......GUCAGGG
env-338   UCUGAC.CGUGACGCAC....GGGUGGAC...GUUUC....CAAGA........GAAAC.GAGCCUGU....UCCCC......GG.................AAUUAC........................CC...........G.GGGAU.....GCGUCAAU.......GUCAGGG
env-339   UCUGAC.CGUGACGCAC....GGGUGGAC...GUUUC....CAAGA........GAAAC.GAGCCUGU....UCCCU......GG.................AAUAC.........................CC...........G.GGGAU.....GCGUCAAU.......GUCAGGG
CMe-1-1   UCUGAC.CGCGACGCAU....GGGUGGAC...GUUU.....AAA...........AAAC.GAGCCUGG....UUCCC......UGA................AUCA.........................UCA...........G.GGAAU.....GCGUCAGU.......GUCAGGG
env-340   ACUGAC.AGGUACGCAU....GGGUCGAC...GUU......CGUGC..........AAC.GAGCCCGU....UCCC.GC....UGCUU..............CAAUAAU....................GGGCA.........GA..AGGAU.....GCGUUAUU.......GUCAGGA
env-341   UCUGAC.CGGUACGCAU....GGGUCGAC...GUUU.....GAAC..........AAAC.GAGCCCAU....UCCACA.....UGCUCU.............AAAA......................AGUGCA..........CG.UGGAU.....GCGUUAUU.......GUCAGGA
env-349   UCUGAC.CGGUACGCAU....GGGUCGAC...GUUU.....GAAC..........AAAC.GAGCCCAU....UCCACA.....UGCUCU.............AAAA......................AGUGCA..........CG.UGGAU.....GCGUUAUU.......GUCAGGA
env-350   UCUGAC.CGAUACGCA.....GGGUCGAC...GUU......CGUGC..........AAC.GAGCCCGC....UCCCG......CUGCUU.............CCCUCAAA..................GAGUAG...........A.AGGAU.....GCGUUAUU.......GUCAGGA
env-351   UCUGAC.CGGUACGCAU....GGGUCGAC...GUU......CGUGC..........AAC.GAGCCCGU....UCCCG......CUGUUU.............CAAUAAUUU.................GGGCAG...........A.AGGAU.....GCGUUAUU.......GUCAGGG
env-352   UCUGAU.UACCGCGCAU....GGGUCGAC...GUU......CGUGC..........AAC.GAGCCCGU....UCCCC......C..................UGAAUAC........................G...........G.GGGAU.....GCGUCAUU.......AUCAGGA
env-353   UCUGAU.AAGCACGCAU....GGGUCGAC...GUUU.....GAAC..........AAAC.GAGCCCGU....UCCAA......UUGCUC.............AAAUCAUA..................GGGUGA...........A.UGGAU.....GCGUUAUU.......GUCAGGA
env-354   UCUGAU.AAGCACGCAU....GGGUCGAC...GUUU.....GAAC..........AAAC.GAGCCCGU....UCCAA......UUGCUC.............AAAUUAUA..................GGGCGG...........A.UGGAU.....GCGUUAUU.......GUCAGGA
env-355   UCUGAU.AAGCACGCAU....GGGUCGAC...GUUU.....GAAC..........AAAC.GAGCCCGU....UCCAA......UUGCUC.............AAAUCAUA..................GGGCGG...........A.UGGAU.....GCGUUAUU.......GUCAGGA
env-356   UCUGAU.AAGCACGCAU....GGGUCGAC...GUUU.....GAAC..........AAAC.GAGCCCGU....UCCAA......UUGCUC.............AAAUCAUA..................GGGCGG...........A.UGGAU.....GCGUUAUU.......GUCAGGA
env-357   UCUGAC.CAGCACGCAU....GGGUCGAC...GUUU.....AAAC..........AAAC.GAGCCCGU....UCCAC......CUGUUCU............CAUCAU...................GGAGCAG...........A.UGGAU.....GCGUUAUU.......GUCAGGA
env-358   UCUGAC.CAGCACGCAU....GGGUCGAC...GUUU.....GAAC..........AAAC.GAGCCCGU....UCCAC......CUGCUCU............CCUCAU...................GGAGAAG...........A.UGGAU.....GCGUUAUU.......GUCAGGA
env-359   UCUGAC.CAGCACGCAU....GGGUCGAC...GUUU.....GAAC..........AAAC.GAGCCCGU....UCCAC......CUGCUCU............CCUCAU...................GGAGAAG...........A.UGGAU.....GCGUUAUU.......GUCAGGA
env-360   UCUGAC.CAGCACGCAU....GGGUCGAC...GUUU.....GAAC..........AAAC.GAGCCCGU....UCCAC......CUGCUCU............CCUCAU...................GGAGAAG...........A.UGGAU.....GCGUUAUU.......GUCAGGA
env-361   UCUGAC.CAGCACGCAU....GGGUCGAC...GUUU.....GAAC..........AAAC.GAGCCCGU....UCCAC......CUGCUCU............CCUCAU...................GGAGAAG...........A.UGGAU.....GCGUUAUU.......GUCAGGA
env-370   UCUGAC.CAGCACGCAU....GGGUCGAC...GUUU.....GAAC..........AAAC.GAGCCCGU....UCCAA......UUGCCCCA...........UCA.....................UGGGGCAG...........A.UGGAU.....GCGUUAUU.......GUCAGGA
env-371   UCUGAC.CAGCACGCAU....GGGUCGAC...GUUU.....GAAC..........AAAC.GAGCCCGU....UCCAA......UUGCUCUG...........AUCA....................CGGAGCAG...........A.UGGAU.....GCGUUAUU.......GUCAGGA
env-372   UCUGAC.CGGUACGCAU....GGGUCGAC...GUUU.....GAAC..........AAAC.GAGCCCGU....UCCAA......AUGCUC.............UAAUAAAAU.................GAGCCU...........C.UGGAU.....GCGUUAUU.......GUCAGGA
env-373   UCUGAC.CGGUACGCAU....GGGUCGAC...GUUU.....GAAU..........AGAC.GAGCCCGU....UCCAA......AUGCUCUA...........AAA.....................UAGAGCAU...........C.UGGAU.....GCGUUAUU.......GUCAGGA
env-374   UCUGAC.CAGCACGUAU....GGGUCGAC...GUU......AAAAA..........AAC.GAGCCCGU....UCCAU......CCGCUC.............UAAAAAAU..................GUGCAG...........A.UGGAU.....GCGUUAUU.......GUCAGGA

          .<<<<<....<<<<<<.....<<<<...<...<<<<<<<<<.........>>>>>>>>>.>.>>>>......<<<<<......<<<<<<<<<<<<<<<<<<<............>>>>>>>>>>>>>>>>>>>............>.>>>>.....>>>>>>.........>>>>>..
          .20.......22222......001...2...22221110..........01112222.2.100.......22222......222222..2.2.1111110............0111111.2.2..222222............2.2222.....22222..........02..
          YCUGAY-○R●●RCGY○Y----GGGU●RAY---●●○○-----●●●○----------○R●●-R○RCCCR●----UYYC●-------●●○○○--------------●●●○----------------------○○○○○●-------------R-GRRR------GCGY●●●●○○-----RUCAGGR
          5′                                                                                                                                                                                 3′
```

```
env-375  UCUGAU.UAGGACGCAU...GGGUCGAC...GUU.....CGUGC.........AAC.GAGCCCGU....UCCCU.....CUGCUGU...........AAUAAUA.................GCAGCAG...........G.AGGAU....GCACCAAU.......AUCAGGA
env-376  UCUGAU.UAGGACGCAU...GGGUCGAC...GUU.....CGUGC.........AAC.GAGCCCUU....UCCCU.....AUGCUGC...........AACAGA..................GCAGCAG...........G.UGGAU....GCGCCAAU.......AUCAGGA
env-377  UCUGAU.UAGGACGCAU...GGGUCGAC...GUU.....CGUGC.........AAC.GAGCCCGU....UCCCU.....AUGCUGC...........AACAAA..................GCAGCAG...........G.UGGAU....GCGCCAAU.......AUCAGGA
env-378  UCUGAC.UAAGACGCAU...GGGUCGAC...GUU.....CGUGC.........AAC.GAGCCCUU....UCCCU.....CUGCUGC...........AACAUGA.................GCAGCAG...........G.UGGAU....GCGCCAUU.......GUCAGGA
env-379  UCUGAC.CAGGACGCAU...GGGUCGAC...GUU.....CGUGC.........AAC.GAGCCCUU....UCCCU.....CUGCUGC...........AAAA....................GCAGCAG...........G.CGGAU....GCGCCAUU.......GUCAGGA
env-380  UCUGAC.CAGGACGCAU...GGGUCGAC...GUU.....CGUGC.........AAC.GAGCCCCU....UCCUC.....CUGC..............UGAU.......................GCAG...........G.UGGAU....GCGACCAAAA.....GUCAGGA
env-381  UCUGAC.CAGGACGCAU...GGGUCGAC...GUU.....CGUGC.........AAC.GAGCCCCU....UCCUC.....CUGC..............UGAU.......................GCAG...........G.UGGAU....GCGACCAAAA.....GUCAGGA
env-382  UCUGAC.CGGGACGCAU...GGGUCGAC...GUU.....CGUGC.........AAC.GAGCCCCU....UCUGC.....CUGC..............UAAC.......................GCAG...........G.UGGAU....GCGACCAAAA.....GUCAGGA
env-383  UCUGAC.CAGGACGCAU...GGGUCGAC...GUU.....CGUGC.........AAC.GAGCCCCU....UCCGC.....CUGC..............UUAU.......................GCAG...........G.UGGAU....GCGACCAAAA.....GUCAGGA
env-384  UCUGAC.CAGGACGCAU...GGGUCGAC...GUU.....CGUGC.........AAC.GAGCCCCC....UUCAC.....CUGC..............UGAC.......................GCAG...........G.AGGAU....GCGACCAGAA.....GUCAGGA
env-385  UCUGAC.CAGGACGCAA...GGGUCGAC...GUU.....CGUGC.........AAC.GAGCCCAU....UCCAC.....CUGC..............AUUAA......................GCAG...........G.UGGAU....GCCCCAAAA......GUCAGGA
env-386  UCUGAC.CAGGACGCAU...GGGUCGAC...GUU.....CGUGC.........AAC.GAGCCCAU....UCCAC.....CUGCU.............AUA.......................AGCAG...........G.UGGAU....GCCCCAAAA......GUCAGGA
env-387  UCUGAC.CAGGACGCAU...GGGUCGAC...GUU.....CGUGC.........AAC.GAGCCCAU....UCCAC.....CUGCU.............AUA.......................AGCAG...........G.UGGAU....GCCCCAAAA......GUCAGGA
env-389  UCUGAC.CAGGAUGCAU...GGGUCGAC...GUU.....CGUGC.........AAC.GAGCCCUC....UCCGC.....CUG...............CUAAGC......................CAG...........G.CGGAU....GCGCCAAAA......GUCAGGA
env-390  UCUGAC.CAGCACGCAU...GGGUCGAC...GUU.....CAUGC.........AAC.GAGCCCGU....UUCAU.....CUGCUUCA..........AACA...................UGAGGCAG...........A.UGGAU....GCGUUAUU.......GUCAGGA
env-392  UCUGAU..CAGGCGCAU...GGGCCAAC...GUU.....CGUGC.........AAC.GAGCCCCC....UUCAC.....GUG...............AAAAC.......................CAC...........G.UGAAU....GCGCCAUU.......AUCAGGA
env-395  UCUGAU.UACGACGCAU...GGGCCGAC...GUU.....CGUGC.........AAC.GAGCCCUC....UCCAC.....CUGC..............UAAC.......................GCAG...........G.UGGAU....GCGCCAUU.......AUCAGGA
env-399  UCUGAU.CUGGCCGCAU...GGGUCGAC...GUU.....CGUGC.........AAC.GAGCCCGU....UCCGCGAU..GUUAC.............CUUUGA....................GUAAC.........AAA.CGGAU....GCGCCAAU.......GUCAGGA
env-400  UCUGAC.CAGGACGCAU...GGGUCGAC...GUU.....CGUGC.........AAC.GAGCCCC.....UCUCCU....UACAACC...........UGAC.....................GGUAGUA..........AG.GAGAU....GCGACCAAAA.....GUCAGGA
env-402  UCUGAU.UAGGACGCAU...GGGUCGAC...GUU.....CGUGC.........AAC.GAGCCCUU....UCCCUAU...GCUGC.............AACAGA....................GCAGC.........AGG.UGGAU....GCGCUAAU.......AUCAGGA
env-403  UCUGAU.UAGGACGCAU...GGGUCGAC...GUU.....CGUGC.........AAC.GAGCCCUU....UCCCUAU...GCUGC.............AACAGA....................GCAGC.........AGG.UGGAU....GCGCUAAU.......AUCAGGA
env-405  UCUGAU.UAGGACGCAU...GGGUCGAC...GUU.....CGUGC.........AAC.GAGCCCUU....UCCCCCG...UCACA.............GGAUA.....................UGUGA........GUUG.GGAAU....GCGCCAUU.......AUCAGGA
env-406  UCUGAU.UAGGACGCAU...GGGUCGAC...GUU.....CGUGC.........AAC.GAGCCCUU....UCCCCCG...UCACA.............GGAUA.....................UGUGA........GUUG.GGAAU....GCGCCAUU.......AUCAGGA
env-407  UCUGAU.UGGGACGCAU...GGGUCGAC...GUU.....CGUGC.........AAC.GAGCCCGU....UCCGC.....UGCC..............UCAUUGAAU...................GGCA...........GAAGGAU....GCGUUAUU.......GUCAGGA
Mli-2-1  UUUGAU.GUAUGCGCAUAU.GGGUGGAC...GAA.....AUUU..........UUC.G.GCCCUU....ACUC.......................UUAAAU..............................GAGUAGU.GCAUAUUAAG....GUCAGAA
Mpe-1-1  UCUGAU.AAAUGCGCUCAA.GGGUGGAC...GAA.....AUU...........UUC.G.GCCCUU....UAUCC......................GACG...................................G.GAUA.....GCGCACAAAUAG...GUCAGAA
Mpa-2-1  UCUGAU.AAAUGCGCUUUUGAGGGUGGAC...GAA.....AUU...........UUC.G.GCCCUU....UAUCC......................GACG...................................G.GAUA.....GCGCACAAAUAG...GUCAGAA
env-408  UCUGAU.UAAUGCGCUCA..GGGUGGAC...GAA.....AUAU..........UUC.G.GCCCUU....UAUCC......................GAAG...................................G.GAUA.....GCGCAUAAUAG....GUCAGAA
Mmo-1-1  UCUGAU.UAAUGCGCCAA..GGGUGGAC..AGAUGCG..GAUG.......UGCAUC.GGGCCCUU....UCUU.A.....AAGGCA............CAAG....................UGUCUU.........AA..AAGA.....GCGCAAAUUACUG..GUCAGAA
env-409  UCUGAU.UAAUGCGCCAA..GGGUGGAC..AGAUGCG..GAUG.......UGCAUC.GGGCCCUU....UCUU.A.....AAGGCA............CAAA....................UGUCUU.........AA..AAGA.....GCGCAAAUUACUG..GUCAGAA
env-410  UCUGAU.UGACGCGG.C...GGGCCAAC...G.......CACCGU..........C.GACCCCAU....UUCCC.....GG................CAGU.......................CC............G.GGAA.....GCGCGCUUAGG....GUCAGGA
Mco-1-1  ACUGAC.GGAAACGCAU...GGGUCGAU...GUCG....GAAG.........CGAC.A.GCCCGGCAAUGCUCCC.....CGAC..............GCAAAA....................GUCG........AGAAG.GGGCAGC...GCGUGACA.......GUCAGGA
env-413  UCUGAC.AAAAAUGCAU...GGGUCGAU...AUCG....GAAU.........CGAU.A.GCCCGGUC..CGCCUCGAA..GCACCUU...........CAAGA..................AAAGUGC.......GAACU.GGCGAGC...GCGUUAAU.......GUCAGAA
env-414  UCUGAC.AAAAGCGCAU...GGGUCGAU...AUC.....GGAAUA........GAU.A.GCCCGGUC..CGCC.UCGAA..GCACCUUC..........AAG.....................GAAAGUGC.......GAACU.GGCGAGC...GCGUUAAU.......GUCAGAA
env-415  UCUGAC.AAAAGCGCAU...GGGUCGAU...AUCG....GAAU.........CGAU.A.GCCCGGUC..CGCCUCGAA..GCACCUUC..........AAG.....................GAAAGUGC.......GAACU.GGCGA.....GCGCGUUAAN.....GUCAGAA
env-416  UCUGAC.ACAGGCGCAU...GGGUUGAU...AUCG....GAAU.........CGAU.A.GCCCGGUC..CGCCUCGAA..GCACCUUC..........AAG.....................GAAAGUGC........GAAAUGGCGA.....GCGCGUUAAU.....GUCAGAA
env-418  UCUGAU.AAAUAUGCAU...GGGUCGAU...AUC.....GGAAUA........GAU.A.GCCCGG....UUCCUCUUAAAGUACUUC...........AGCAG....................GAAGUAU.......CAAUA.GGAGA.....GCGCGUUAAU.....GUCAGAA
env-423  UCUGAC.AAAUUCGUAU...GGGUCGAU...AUC.....GGAAUA........GAU.A.GCCCGGU....UCGCCUUGAAGUACUUUC..........GAA.....................GGAAGUAU.....CAAUAGG.AGAAC....GCGUUAAU.......GUCAGGA
env-425  UCUGAC.AAAUUCGUAU...GGGUCGAU...AUC.....GGAAUA........GAU.A.GCCCGGUU..CGCCU.....UGGAGUACUUC........CGAAA...............GAAGUAU..CA.........AUA.GGAGAAC...GCGUUAAU.......GUCAGGA
env-428  UCUGAU.GGCGGCGCAU...GGGUGGAA...AUCG....GUGU.........CGAU..CGCCCAA....UUCCC.....CUUCGAAC..........UGAUGAGAU..............GUUCGAAG..........CU.GGAAA....ACGCCUAAAU.....GUCAGAA
env-429  UCUGAU.GGCGGCGCAU...GGGUGGAA...AUCG....GUGU.........CGAU..CGCCCAA....UUCCC.....CUUCGAAC..........UGAUGAGAU..............GUUCGAAG..........CU.GGAAA....ACGCCUAAAU.....GUCAGAA
Msh-1-1  UCUGAC.GGCAGCGCAU...GGGUCGA...ACUCG....GACU.........CGAG..AGCCCA.....GCUCC.....ACU..CAGAAACCC.....GAAA............GG...UGUGAAGGU...........G.GGGCA....GCGCAUAGAC.....GUCAGAA
env-430  UCUGAU.GGCGGCGUCU...GGGUGGAA...AUCG....GAAU.........CAA..UCGCCCAAUUC.CCCUUCGCAC.CGGAGAA............AAG.......................UUC.CCG..........GUAGGGCA....GCGCCUAAGU.....GUCAGAA
env-433  UCUGAU.GGCGGCGUCU...GGGUGGAA...AUCG....GAAU.........CAA..UCGCCCUAU...UCCCCUUCG..CACCGG............AGAAAAGUAC................UCGGUG............G.GGCA....GCGCCUAAGU.....GUCAGAA
env-434  UCUGAU.GGCGGCGUAU...GGGUGGAA...AUCG....GAAU.........CAAU..CGCCCAAU...UCCCCUUCGC.ACCGGA............GAAAAUUA..................UCCGGU...........AG.GGCA....ACGCCUAAAC.....GUCAGAA
env-435  UCUGAU.GGCGGCGUAU...GGGUGGAA...AUCG....GAAU.........CAAU..CGCCCAAU...UCCCCUUCGC.ACCGGA............GAAAAGUA..................UCCGGU...........AG.GGCA....ACGCCUAAAC.....GUCAGA.
env-436  UCUGAU.GGCGGCGUAU...GGGUGGAA...AUCG....GAAU.........CAAU..CGCCCAAU...UCCCCUUCGC.ACCGGA............GAAAAGUA..................UCCGGU...........AG.GGCA....ACGCCUAAAC.....GUCAGAA
env-437  UCUGAU.GGCGGCGUAU...GGGUGGAA...AUCG....GAAU.........CAAU..CGCCCAAU...UCCCCUUCGC.ACCGGA............GAAAAGUA..................UCCGGU...........AG.GGCA....ACGCCUAAAC.....GUCAG..
env-439  UCUGAU.GGCGGCGUAU...GGGUGGAA...AUCG....GAAU.........CAAU..CGCCCAAU...UCCCCUUCGC.ACCGGA............GAAAAGUA..................UCCGGU...........AG.GGCA....ACGCCUAAAC.....GUCAGAA
env-440  UCUGAU.GGCGGCGUAU...GGGUGGAA...AUCG....GAAU.........CAAU..CGCCCAAU...UCCCCUUCGC.ACCGGA............GAAAAGUA..................UCCGGU...........AG.GGCA....ACGCCUAAAC.....GUCAGAA
env-441  UCUGAU.GGCGGCGUAU...GGGUGGAA...AUCG....GAAU.........CAAU..CGCCCAAU...UCCCCUUCGC.ACCGGA............GAAAAGUA..................UCCGGU...........AG.GGCA....ACGCCUAAAC.....GUCAGAA
env-442  UCUGAU.GGCGGCGUAU...GGGUGGAA...AUCG....GAAU.........CAAU..CGCCCAAU...UCCCCUUCGC.ACCGGA............GAAAAGUA..................UCCGGU...........AG.GGCA....ACGCCUAAAC.....GUCAGAA
env-443  UCUGAU.AGCGGCGUCU...GGGUGGAA...AUCG....GAAU.........CAA..UCGCCCAAU...UCCCCUUCGC.ACCGA.............AAGUAGGUAC.................UCGGU...........AG.GGGA....GCGCCUNAAU.....GUCAGAA
env-444  UCUGAU.AAAGGCGUCU...GGGUGGAA...AUCG....GAGU.........UGA..UCGCCCAAUA..CCCCUA....GUGUC.............GAAAGA....................GAUAU.....ACAAGCC.GGGGCA...ACGCCUAAAU.....GUCAGAA
env-445  UCUGAU.GGCGGCGUUU...GGGUGGAA...AUCG....GAAU.........CGAU..CGCCCAAUU..CCCCU.....CCG..CACC..........GGAGGCA...............GGUACUCGG...........U..GGGGCA...GCGCCUUAAC.....GUCAGAA
env-446  UCUGAU.GGCGGCGUUU...GGGUGGAA...AUCG....GAAU.........CGAU..CGCCCAAUU..CCCCU.....CCG..CACC..........GGAGGCA...............GGUACUCGG...........U..GGGGCA...GCGCCUUAAC.....GUCAGAA
env-448  UCUGAU.GGCGGUGCAU...GGGUGGAA...AUCG....GUAU.........CGAU..CGCCCAGU...GCCUCUG...CGAACC............GAGAAUGA..................GGUUCG........ACCG.GGGCA....GCGCCUCAAC.....GUCAGAA
env-449  UCUGAU.GGUGGCGCAU...GGGUGGAA...AUCG....GUGU.........CGAU..CGCCCAAUAC.CCUC......CGCA..............CUGAGGAGAAAUA...............UGCG..........GUAGGGAG....ACGCCUGAAC.....GUCAGAA
env-450  UCUGAU.GGCGGCGUCU...GGGUGGAA...AUCG....GAAU.........CAAU..CGCCCUAUU..CCCCU.....UCG..UA...CC.......GGU...............GGUAAAUACUCGG...........U..GGGGCA...GCGCCUAAAU.....GUCAGAA
env-451  UCUGAU.GGCGGCGUAU...GGGUGGAA...AUCG....GAAU.........CGAU..CGCCCAAUU..CCCCU.....CCGC..ACC..........GUGGGUA...............GGUACUCGG...........U..GGGGCA...GCGCCUAAGU.....GUCAGAA
env-452  UCUGAU.GGCGGCGUAU...GGGUGGAA...AUCG....GAAU.........CGAU..CGCCCAAUU..CCCCU.....CCGC..ACC..........GUGGGUA...............GGUACUCGG...........U..GGGGCA...GCGCCUAAGU.....GUCAGAA
env-455  UCUGAU.GGCGGCGUCU...GGGUGGAA...AUCG....GAAU.........CGAU..CGCCCAAUU..CCCCU.....CCGC..ACC..........GUGGGUA...............GGUACUCGG...........U..GGGGCA...GCGCCUAAAU.....GUCAGAA
env-456  UCUGAU.GGCGGCGUCU...GGGUGGAA...AUCG....GAAU.........CGAU..CGCCCAAUU..CCCCU.....CCGC..ACC..........GCGGGUA...............GGUACUCGG...........U..GGGGCA...ACGCCUAAAU.....GUCAGAA
env-460  UCUGAU.GACGGCGUAU...GGGUGGAA...AUCG....GAAU.........CGAU..CGCCCAAUU..CCCCUUC...GCACC.............GAUAA.....................GGUGU....ACCAGUUG.GGGGA....GCGCCUUGAC.....GUCAGAA
env-462  UCUGAU.GACGGCGUAU...GGGUGGAA...AUCG....GAAU.........CGAU..CGCCCAAUU..CCCCUCC...GCACC.............GAUAA.....................GGUGU....ACCAGUUG.GGGGA....GCGCCUUGAC.....GUCAGAA
env-464  UCUGAU.GACGGCGUAU...GGGUGGAA...AUCG....GAAU.........CGAU..CGCCCAA...UUCCCCUCC..GCACC.............GAUAA.....................GGUGU....ACCAGGCG.GGGGA....GCGCCUUGAC.....GUCAGAA
env-465  UCUGAU.GACGGCGUAU...GGGUGGAA...AUCG....GAAU.........CGAU..CGCCCAAUU..CCCCUC....CGCACC............GAUAA....................GGUGUG.....CCAGGUG.GGGGA....GCGCCUUGAC.....GUCAGAA
env-466  UCUGAU.GGCGGCGUCU...GGGUGGAA...AUCG....GAGU.........CGAU..CGCCCAAUU..CCCCUCC...GCACC.............AAUGG.....................GGUGU.....UCGAGUAGGGGCA...GCGCCUAAGU.....GUCAGAA
env-467  UCUGAC.GACGGCGCAU...GGGUGGAG...AUAG....GUGU.........UGAG.C.GCCCAU....UGCCCCUCAG.GACCGAAA..........AGCGG..................UUUCGGUU...........GG.GGCA....GCGCCUGAAC.....GUCAGAG
env-468  UCUGAC.GACGGCGCAU...GGGUGGAG...AUAG....GUGU.........UGAG.C.GCCCAU....UGCCCCUCAG.GACCGAAA..........AGCGG..................UUUCGGUU...........GG.GGCA....GCGCCUGAAC.....GUCAGAG
env-469  UCUGAC.GACGGCGCAU...GGGUGGAG...AUAG....GUGU.........UGAG.C.GCCCAU....UGCCCCUCAG.GACCGAAA..........AGCGG..................UUUCGGUU...........GG.GGCA....GCGCCUGAAC.....GUCAGAA
env-471  UCUGAC.GACGGCGCAU...GGGUGGAG...AUAG....GUGU.........UGAG.C.GCCCAU....UGCCCCUCAG.GACCGAAA..........AGCGG..................UUUCGGUU...........GG.GGCA....GCGCCUGAAC.....GUCAGAN
env-472  UCUGAC.GACGGCGCAU...GGGUGGAG...AUAG....GUGU.........UGAG.C.GCCCAU....UGCCCCUCAG.GACCGAAA..........AGCGG..................UUUCGGUU...........GG.GGCA....GCGCCUGAAC.....GUCAGAA
env-474  UCUGAC.AACGGCGCAU...GGGUGGAG...AUAG....GUGU.........UGAG.C.GCCCAU....UGCCC.....CUCAAGACCGC.......AUC.................GCGGUACAGAG..........CAG.GGCA....GCGCCUGAAC.....GUCAGAG
env-475  UCUGAC.GACGGCGCAU...GGGUGGAG...AUAG....GUGU.........UGAG.C.GCCCUU....UGCCC.....CUCAGAACCGC.......GAA.................GCGGUUUUCUG..........CUG.GGCA....GCGUCUGAAC.....GUCAGAG
Mha-1-1  UCUGAC.GACGGCGCAU...GGGUGGAG...AUAG....GUGU.........UGAG.C.GCCCUU....UGCCC.....CUCAGAACCGC.......GAA.................GCGGUUUUCUG..........CUG.GGCA....GCGUCUGAAC.....GUCAGAG
env-476  UCUGAC.GACGGCGCAU...GGGUGGAG...AUAG....GUGU.........UGAG.C.GCCCUU....UGCCC.....CUGAGAAC..........CGCUCUGC.................GGUUUCAG........ACUG.GGCA....GCGUCUGAAC.....GUCAGAA
env-477  UCUGAC.AACGGCGCAU...GGGUGGAC...AUAG....GUG..........UUGA.GUGCCCAU....UGCCCCUCAAGACCGC..............UCG.......................GCGGU...ACGAAGUGG.GGCA....GCGCCUGAAC.....GUCAGAG
env-478  UCUGAU.AAAGGCGUCU...GGGUGGAA...AUCG....GAGU.........CGAU..CGCCCUG....UGCCC.....CUCGUC............ACA.......................GACGAGAAAUACAAGUUGG.GGCA....GCGCCUGAAC.....GUCAGGA
env-479  UCUGAU.GGUGGCGCAA...GGGUGGAA...CUCG....GAGU.........CGAG.U.GCCCGG....UGCCCCUCC..CUCACC............AAGAAAAC..................GGUGAG.......ACAG.GGCA....GCGCCUGAAC.....GUCAGAA
env-480  UCUGAU.GGUGGCGCAA...GGGUAGAA...CUCG....GAGU.........UGAG.U.GCCCGG....UGCCCCUCCA.CACC..............AAGAGAAC....................GGUG.....AGACGAG.GGCA....GCGCCUGAAC.....GUCAGAA
env-481  CCUGAC.GGAAGCGCAU...GGGUAGAG...CUCG....GGUU.........UGAG.C.GCCCAGCGUACUUC.....CUGAGCCU..........AAAG..................GGGCAAAG...........G.AAGGAGA..GCGCGUGAAC.....GUCAGGA
env-482  CCUGAC.GGUCGCGUUU...GGGUAGAU...AUAG....GUG..........UUGG.ACGCCUUG....CCCUC.....CUCAA.............AAAUGG.....................UAAAG..........CUG.GGCA....ACGUGUGACC.....GUCAGGG

         .<<<<....<<<<<.....<<<<...<...<<<<<<<<<.........>>>>>>>>>.>.>>>>......<<<<......<<<<<<<<<<<<<<<<<<............>>>>>>>>>>>>>>>>>>...........>.>>>>.....>>>>>...........>>>>>..
         .20.....22222......001...2...22221110..........01112222.2.100.......22222......222222..2.2.1111110.............0111111.2.2..222222...........2.2222.....22222............02..
         YCUGAY-∘R••RCGY∘Y---GGGU•RAY---•••∘-----•••∘---------∘R••-R∘RCCCR•----UYYC•------••∘∘∘-------------•••∘---------------------∘∘∘∘∘•-----------R-GRRR-----GCGY••••∘∘-----RUCAGGR
         5'                                                                                                                                                                             3'
```

```
env-483    ACUGGC....UGCGCUU....GGGUGGAA...AUC......GGUUUG.........GAU.U.GCCCAG....CCUUU......UUGUGCA.CG.........AAUA.................CGCUGCGCAA............G.GGGGA.....GCGCGUGACA.....GUUAAGA
env-484    ACUGGC....UGCGCUU....GGGUGGAA...AUC......GGUUUG.........GAU.U.GCCCAG....CCUUU......UUGUGCA.CG.........AAUA.................CGCUGCGCAA............G.GGGGA.....GCGCGUGACA.....GUUAAGA
env-485    ACUGGC....UGCGCUU....GGGUGGAA...AUC......GGUUUG.........GAU.U.GCCCAG....CCUUU......UUGUGCA.CG.........AAUA.................CGCUGCGCAA............G.GGGGA.....GCGCGUGACA.....GUUAAGA
env-486    ACUGGC....UGCGCUU....GGGUGGAA...AUC......GGUUUG.........GAU.U.GCCCAG....CCUUU......UUGUGCA.CG.........AAUA.................CGCUGCGCAA............G.GGGGA.....GCGCGUGACA.....GUUAAGA
env-492    ACUGGC....UGCGCUU....GGGUGGAA...AUC......GGUUUG.........GAU.U.GCCCAG....CCUUU......UUGUGCA.CG.........AAUA.................CGCUGCGCAA............G.GGGGA.....GCGCGUGACA.....GUUAAGA
env-495    ACUGAC....UGCGCUU....GGGUUGAA...AUC......GGUUUG.........GAU.U.GCCCAG....CCUUU......UUGUGCA.CG.........AAGA.................CGCUGCGCAA............A.GGGGA.....GCGCGUGACA.....GUCAAGA
env-504    ACUGAC....UGCGCUU....GGGUUGAA...AUC......GGUUUG.........GAU.U.GCCCGG....CCCUU......UUGCGAACCG........CGA..................CGUUGCGCAA............A.GGGGA.....GCGCGUGACA.....GUUAAGA
env-505    UGUGAU.UUCGGCAA......GGGUGGAC...G........AUG..............C.G.GCCCAU....UACCU......GUGA..............UUGA.......................UCAC............G.GGUU.....AAGCCUGAAU.....UUCACGC
env-508    UCUGAA.AGCGGCGAGAA...GGGCGGAC...GUAUCU...UCUG........GGAUAU.GAGCCCGG....UACC.......AGCCA.............GCAGG.....................UGGCA.............GGUAC.....ACGCCCGU.......UUCAGGA
env-509    ACUGAAAACAGGCAUGCG...GGGUGGAC...GUAUCC...CCUG........GGAUAU.GAGCCCAU....AAUCU......GCCUU.............GAA.......................ACGGC............A.GGUUC.....AUGCUGGA.......UUCAGGA
Mta-1-1    GCUGAA.ACAGGCAUGCA...GGGUGGAC...GAAUCC...CCUG........GGAUAC.GGGCCCGU....CAUCU......GCC...............CAUAAAAAGA..................GGC............A.GGUGC.....AUGCCGGA.......UUCAGGA
env-511    GCUGAA.ACAGGCAUGCA...GGGUGGAC...GAAUCC...CCUG........GGAUAC.GGGCCCGU....CAUCU......GCC...............CAUAAAAAGA..................GGC............A.GGUGC.....AUGCCGGA.......UUCAGGA
env-512    GCUGAA.ACAGGCAUGCA...GGGUGGAC...GAAUCC...CCUG........GGAUAC.GGGCCCGU....CAUCU......GCC...............CAUAAAAAGA..................GGC............A.GGUGC.....AUGCCGGA.......UUCAGGA
Hbo-1-1    CCUGAC.GGCCGCGUUC....GGGUCAAU...UGCG.....GAA...........UGCA.ACACCCGA....UCUCC......CUCUACACGCCAUCG...GAUA............UGAUGGUGAA.AGAG............G.GGGA......GCGCGUCGCC.....AUCAGGA
Ngr-1-1    CCUGAA.GGGCGCGUCC....GGGUCAAU...UGCG.....GCU...........UGCA.ACACCCGA....UCUCC......CUCAACACACCUCC....CGACGU............GGAGGU.CGCCGAG............G.GGGA......GCGCGUGACC.....AUCAGGA
Hje-1-1    CCUGAA.GGGCGCGUAC....GGGUCAAU...UGCG.....GAC...........UGCA.ACACCCAC....UCUCC......CUCAUAACGCCUCC....CGGAAU............GGAGGU.CCAGCAG............G.GGGA......ACGCGUGACC.....GUCAGGA
Nma-1-1    CCUGAU.CAACGCGUGC....GGGUCAAU...UGCG.....GCU...........UGCA.ACACCCGA....UCUCC......CUCAAUGUACCGCCG...AAAA............UGGCGGU.CUCCGUG............G.GAGA......GCGCGUGACU.....AUCAGGA
Hal-1-1    CCUGAC.GACCGCGUUC....GGGUCAAU...UGCG.....GAA...........UGCA.ACACCCGA....UCUCCC.....UGUUCGUCCC........UCCC.................GGGAUGGACC....UCACAGAGG.GAGA......GCGCGUCGCU.....GUCAGGA
Nti-1-1    CCUGAA.GGGCGCGUCC....GGGUCAAU...UGCG.....GCU...........UGCA.ACACCCGA....UCUCC......CUCAACGUUCCUCCG...AAAA............UGGAGGU.CGAAGAG............G.GGGA......GCGCGUGACC.....GUCAGGA
Npe-1-1    CCUGAA.GGGCGCGUCC....GGGUCAAU...UGCG.....GAC...........UGCA.ACACCCGA....UCUCC......CUCAACGUACCGCCG...AAAA............UGGCGGU.CGAGGAG............G.GAGA......GCGCGUGACC.....GUCAGGA
Hti-1-1    UCUGAU.GGGCGCGUCC....GGGUCAAU...UGCG.....GUA...........UGCA.ACACCCGA....UCUCC......CUC....GCCGUUCCGG..GCGA..........CCGGA.CGAUACGUGAG............G.GGGA......ACGCGUCACC.....GUCAGGA
           .<<<<<....<<<<<<.....<<<<...<...<<<<<<<<<.........>>>>>>>>>.>.>>>>.....<<<<<......<<<<<<<<<<<<<<<<<<<<.............>>>>>>>>>>>>>>>>>>>>............>.>>>>....>>>>>>.........>>>>>..
           .20......22222.......001...2...22221110.........01112222.2.100.......22222......222222..2.2.1111110.............0111111.2.2..222222............2.2222.....22222..........02..
           YCUGAY-∘R••RCGY∘Y----GGGU•RAY---••∘∘-----•••∘----------∘R••-R∘RCCCR•----UYYC•------••∘∘∘---------------••••∘---------------------∘∘∘∘∘•-----------R-GRRR-----GCGY••••∘∘-----RUCAGGR
           5'                                                                                                                                                                                        3'
```

alignment positions 260 · · · 439

```
Mli-1-1    UCUCUUCGCGAGACGAGGUAUUCCGGUGCACACCGGAAUGAUCUCCAUGACAUGGAGACCGCGACCCACGGCACAUCACUCCCGACACCACCGCAGCAGGACUCCAGAUGAUCUGCUGCGCGGAAGAAGCGUGCCCGUUCGAUCUGUCCGGACCCCGGUAACGGGGCGAGGAGUGGGCCG
env-1      AUCUCUUGCUUGAGACCGGUCGUUAUAUGAGAAUUGCAGAGAAGCAUUCUAACCGCAUCACGGGGAAAGGAUUUCUGGCAGGAUAUUCCUGCAUGAAGACCAUUGCGGUGCCAGUGUGAUGUUUUUGGAGAAUGCUUCGUAGUCACUCUCAUCUCUAUCAUACCUGCCUGAUCAGCAGAU
env-2      CUAUUUCCUGCGAGAGCCCUGUUAAGGUACGUAAGGUUAACAAAUCUGAUGUUCCCUAUUACUGGGGAUACGAUACUACCGUUUGCAAAAAUCUUGAGAAAAAGAUUUCAGAUCUUCGGGAUGAGGGAUGGAGCAUAAUUUCCACCUCCCGAUAUGGUGAAAAAGUUAGUACAGAAAAUG
env-3      CUAUUUCCUGCGAUGACAAAAUUGUGGUUGAAAAGGUCCCGCCCAAUGAUGUACCCUGGUAUUGGGGAUUUGACGUAGGUAUCUGUGACCUAGAUACUGAGCUUAAGAACCUAAAACAAGGGGAUUGGAGUGUAAUAUCCACUUCUAGAAAAGGAGAGGUGGUUUCAAAUAGCUCCAUUG
env-4      UCUCUUUGCGAGACAGGUUGGAGUCGGCGAUUCACGCCGACAGGUGCUCCUCCAU.............................................................................................................................
env-5      UCUCUUUGCGAGACAGGUUGGAGUCGGCGAUUCACGCCGACAGGUGCUCCUUCAUACCGGUCACGGAGUCUGCAGACGGGAUGCUGCAGAACGGGGCCGGGCAUGCGGAGAGUUACAACUGGCACUCUCUUUGACGAUCCGCGGCCUUUUCAGUCGACGGCAUUCUCCUGCCGGGUCAGC
env-6      AUCUUACGAGAGACCGGUAGUCGGACAUUCCGGCUAAAGAUCUGUUUACACGCGAGAGAAUUUGUUCUGGC.............................................................................................................
env-8      AUCUUACGAGAGACCGGUAGCCGCAAGAUAAAGGCAAGAUCUGUCUACACGCGAGAGUGUUCAUUCUAGCAGGAUUCCCUGCAUAUCCGAAAAGGGACUCCGUGUUGUAGGGAGAGAUCUGGUCAUGUCUUCUGGCAAGUAGCAGUUAUGGCGAAAGCGCAUAUACAGCUCAUGUCCCGU
env-10     AUCUUACGAGAGACCGGUAGCUGGACGAUACAAAUCCGAUCUGUCUACACAUGUGAGGAACAUUCUGGCAGUUUAUUCUGCACAUCCGAAACGGAACUCCAUGAGGUAGGAAGAGAUCUGAUCGUAGGUCCGGCAUGCAGCAGUUAUGGCGAAAGCGCAUAUACAGCUCAUGUCCCGUGU
env-11     AUCUUACGAGAGACCGGUCGCUGGAUGAUACAAAUCCGAUCUGUCUACACAUGUGAGGAUCAUUCUGGCAGUAUUCUGCACAUCCGAAACGGAACUCCAUGAGGUAGGAAGAGAUCUGAUCGUAUGUCCGGCAUGCAGCAGUUAUGGCGAAAGCGCAUAUACAGCUCAUGUCCCGUGUCU
env-13     UCUAUUCGCGAGACUGGUUCAUCCGGAUGACGGAGCGGACAUGACCGACUCCACCGGAGAGAUUCCGAAAGAGGGAAGGAUCCUCUGCAGGUAUCGGAACGCCACAGUACCCCCGCGGUAAUGGACGAAGAGAUUUCGUUCUCGUCCUGUACCGCAUGAUCAGUGUCGGAAGCGUCACUC
env-14     UCUCUUCGCGAAACGAAUGCUCCCGAGGGAAGAACCGGACAUCGCCGGAUCAUCCUUUUAGGAAUAUCGGGCGAGGGAGAAGAACGCUUUGAGUGAGCGGUAUUCUCCUUCUCCACGAUCAGCACACGGCAGCACAGCCAUUUCCUGCGGCACGAACACUCGAAUAGAUGUAAGUGAGUU
env-15     UUUCUUCGCGAGAACCGGUCCGAGCACGGAUCGUCGAUACUCCACUUUCGGGGUUUGAUGUAUCCUGCUUGGAUCUUGUAGAUGCACUCGACCACGCCACUGCAGGGAUCACCGUUGCGACAUCCAGACAUGGUGAGAUGAUAACCACCGGGCGAUUGCCAGAACUUACCGCCCGUAUCG
Hwa-1-1    UCUCUUCGCGAGAACCGGUCCGGGCACGGAUCGUCGAUACUCCACUUUCGGGGUUUGAUGUCUCCUGCCUGGACCUUGUGGAUGCACUUGAGCACGCUGCUGCAGGUAUCACCGUUGCGACGUCCAGACACGGUGACACAGUAACCACAGGGCGAUUGCCAGAACUUGCCGCACGCGUCA
env-17     UCUCUUCGCGAGAACCGGUCCGGGCACGGAUCGUCGAUACUCCACUUUCGGGGUUUGAUGUCUCCUGCCUGGACCUUGUGGAUGCACUUGAGCACGCUGCUGCAGGUAUCACCGUUGCGACGUCCAGACACGGUGACACAGUAACCACAGGGCGAUUGCCAGAACUUGCCGCACGCGUCA
env-23     UAUCUUCGCGAGAUCCACUCCGCGCCCGCAUCACCGGCUAUCCCGAGGCUGGAUUCCAGGUGACCGGCGCGGAGCUCACGGAAGUCAUUGCCGAGGCUGGACUGACAAUUGCCGCCUCACGACACGGACAGGAGUUAUCCGUGUCCCGGCUAGGCGAGGUAGUCCAGACGGUGUCGGAGG
env-24     UCUCUUCGAGAGAGCCUUUGCGUGCCGAGAUAGCCGAGGACGCAGAUUCGCCCUACGGCGGAUACGAGGUCGAACGCGCACCGCUCGACGACUGGCUCGACUCCUACGAAGGGGUCGCCGUCUGUACGUCGAGACGGGGCAAGGAGGUCGAACUGUCUGACCUCCGUGUCGACGACGAAA
env-25     UCUCUUCGCGGGAGCCGGUCCGGGCAAAACUUGUCGACCAGUCUCCACCGGGAUAUGUCGUCGAGCGCGCGGACCUCGACACAGUGCUGGCGCGGGCGGACGGCCUAACCGUCGCAGCAUCGCGACACGGCCGGCCGCUCACGCCCGAUCGGUAACCCGGUUGGGCGAGCGCGCCGACG
env-27     UCUCUUCGAGAGACCCACUCCAAGCACGGAUCGUGCAGCGCGCUCCGGCGACGUUCGCCGUCGAGACGGCGACGCUCCCCGAGCUGCUCCAGCGACCCGACGCCGGGGUAUGCAUCGCGACGUCCCGCCCACGGGACGCCGCUGACGCAGGCACACCUGGCGACGCUCACCACGGCGAUCG
env-28     UCUCUUCGCGACGACCGGUCCGGGCAAAGAUCGUCGACACGGACCCUUCGGGGCUCGUUGUCGACCGCACGGACCUUCAGGCAGCACUCGGCCCGUGAGGACGCUGGCGUUCGGAUCGCGUCGUCCCGCUACGGUGAACAUCUCACCGUGGGGCGACUCGAGACGCUGGCCGGAGAUGCG
env-29     UCUCUUCGCGACGACCGGUCCGGGCAAAAAUCGUCGACACGGACCCUUCGGGGCUCGUUGUCGACCGCACGGGCCUUCAGGCAGCACUCAGCCCGUGAGGAUGCUGGCGUUUGCAUCGCGUCAUCCCGCUACGGUGAACAUCUCACUGUCGGACGACUCGAGACGCUGGCCGGACAAGUCG
env-30     UCUCUUCGCGACGACCGGUCCGGGCAAAGAUCGUCGACACGGACCCUUCGGGGCUCGUUGUCGACCGCACGGACCUUCAGGCAGCACUCGGCCCGUGAGGACGCUGGCGUUCGGAUCGCGUCGUCCCGCUACGGUGAACAUCUCACCGUGGGGCGACUCGAGACGCUGGCCGGAGAUGCG
env-31     UCUCUUCGCGACGGCCGGUUCGGGCGAAACUCGUCGACGCCCCACUCUCGGGGUUCGACGUCGUUUCGCGCGGGCGAGGGGGAGCGCUUUACCGUCAGGGUCUUCUCGCGACGGCCGGUCCGGGCGAAACUCGUCGACGCCCCAUAUCGGGGUUCGACGUCGUUUCGCGCGGACCUCGA
Hut-1-1    UCUCUUCGCGAGACGACCGGUUCGCGCGAAGAUCGUCGAUCAGCCCCCACCGGGCUAUCAGGUCGACCGUGCGAACCUUGACGCUGCGCUCUCGCGGCCCGACGCCGGCGUACGGAUCGCCGCGUCCCGACACGGCCAGUCGCUCACGACCGAGCGACUCGGGCGACUCGCCGGGACGAUCG
env-32     UCUCUUCGAGAGACCCACUCCAAGCACGGAUCGUCGAGCGCGCUCCGGCGACGUUCGCCGUCGAGGCGGCGGCGCUGGAUGCGCUGCUCGACCGACCCGAUGCCGGGGUGCCCAUCGCGACGUCCCGCCCACGGGACGCCGCUGACGCAGGCACACCUGGCUGCGCUCACCACGGCGAUCG
env-33     UCUCUUCGAGAGACCCACUCCAAGCACGGAUCGUGGAGCGCGCACCCGCGACGUUCGCCGUCGAGACGGCGGCGCUGCCCGAGCUGCUCGGGCGGCCCGACGCGGGGUGUCCAUCGCGACGUCCCGCCCAUGGGACGCCGCUGACACAGGCACACCUGGCUGCGCUCACUGCCGCGAUCG
env-34     UCUCUUCGAGAGACCCACUCCAAGCACGGAUCGUCGAGCGCGCACCCGCGACGUUCGCCGUCGAGACGGCGACGUUGGCGGAGCUGCUCGGGCGGCCCGACGCCGGGGUGUGCAUCGCGACGUCCCGACACGGGACGGCGCUGACGCAGGCACACCUGGCUGCGCUCACCGCGGCGAUCG
env-35     UCUCUUCGAGAGACCCACUCCAAGCCCGGAUCGUCGAGCACGCACCCGGCGACGUUCGCCGUCGAGACGGCGACGCUCACCGAGCAGCUCCAGCGACCCGACGCCGGGGUGCGCAUCGCGACGUCCCGCCACGGGACGCCGCUGACGCAGGCACACCUGGCGACGCUCACCACGGCGAUCG
env-36     UCUCUUCGCGAGAGCCGGUUCGGGCGAAACUCGUCGACGAGCCACAGCCUGGCUACGUCGUCGACACCGCGACACUCGACAGCGCACUCGCCCGUGCAGACGCCGGGUUCACUAUCGCCUCGUCGAGAUACGGCGAGCGCCUGCGCACAGGCCGUCUCGACGAACUUGCCGCGAGAACCA
env-37     UCUCUUCGCGAGAGCCGGUUCGGGCGAAACUAACUGAUGAGCCACUUCCGGCUACGCAGUGGACGCCGCGAACCUCGAUUCGGCAGUUAUACACGCGACGCUGGGUUCACUAUCGCAACUUCGCGAUUUGGUGAGCCAGUGACCGCGUCGACUCUCCACGCUCGUCCCCAGCACAC
env-38     UCUCUUCGCGAGAGCCGGUUCGGGCGAAACUGACCGAAGAGCCAGUCGCUGGUUACGUCGGUAGACGCCGCGAACCUCGAUUCGGCAGUUAGCCGCGACGACGCUGGGUUCACUAUCGCAACUUCGCGAUUUGGUGAGCCAGUUACCACGUCGGCGCUCGAAACGCUCGUUCCCAGUACGG
env-39     UCUCUUCGCGAGAGCCGGUUCGGGCGAAACUGACCGAAGAGCCAGUCGCUGGUUACGUCGGUAGACGCCGCGAACCUCGAUUCGGCAGUUAGCCGCGACGACGCUGGGUUCACUAUCGCAACUUCGCGAUUUGGUGAGCCAGUUACCACGUCGGCGCUCGAAACGCUCGUUCCCAGUACGG
env-40     UCUCUUCGCGAGAACCGGUUCGGGCAAAACUCUCGAUGAGGAGCCACCCGGACCACGUGUCUCCGAACGAUCGCUAUCGUCGGCGCUCAACGCCGAUGAUGCAGGGCUCCGAAUCGCCACCUCACGGCUGGGCCACCGCUCA..................................
env-41     UCUCUUCGCGAGAACCAGUCCGGGCACGGCUCAUUGAUGAGCCACUACCGGGCUUCCAGGUCUCUCGUAUGGACCUGUCAGACGCACUCAAUCGAGAUGAAGCAGGGGUUCGAAUCGCAACCUCUCGACAUGGCACUGCUAUUACCCCUUCACUGAUGUCCACGCUGUCUGCACGGAUAG
env-43     UCUCUUCGCGACGGCCGGUUCGGGCGAAACUCGUCGACGAGCCCACUCCAGGGUUCGCCGUCGACGCUUCGAACCUGCAGACGGCACUCGCGCGUGACGACGUCGGACUCGCCAUCGCCGCUUCGCGGCUCGCGGCGAGGAUCUGACC..................................
env-44     UCUCUUCGCGACGACC....................................................................................................................................................................
env-45     UCUCUUCGCGAGAACCGGUCCGUGCUCGCCUUGUCAACGAGGCCCCCCGGGCUUCGAUGUGAAGGCCAUGGACCUGUCGGAAGCACUCACCACCGAAUCCGGAUUACGGAUUGCCACGUCUCGAUAUGGAGAACCACUCUCCAUUUCGCGGCUUGGAGAACUCGCCCCGCAGACGCGG
env-49     UCUCUUCGAGAGAACCGGUUCGCGCGCGCCUCGUCGAUAAGCCCACACCGGGCUUCGACGUGGCGACUGUGAACCUGUCGGCCGCCCUUGCCGACGCCGCCGGACGACGGAUCGCCACCUCCCGUCAUGGACAGGAGUUGUCCAUCGGGCGACUGGCGGAGUUCCGCCCCGACUACCGCG
env-50     UCUCUUCGAGAGAACCGGUUCGUGCCCGCCUCGUCGACGAGGCCCCCCGGGCUUCGUCGUCGACCGCAUGGACCUGUCGGAAGCGCUUGCCGACGAACCGGGGCUGACGAUCGCCACAUCCGCGUAUGGACAGACGCUGUCGCAUGCGCGACUCGGCACCCUCCUCGCCGACGUUCGCG
env-54     UCUCUUCGAGAGACCCG...................................................................................................................................................................
env-55     UCUCUUCGAGAGAACCGGUCCGUGCACGACUCGUCGACGAGCCCAUAUCGGGGCUUGUUGUCGAAUGUGCGGACCUCUCGGCUGCCCUCGACGACGAAUCAGGCGUAACGGUCGCCGCCUCCGAGUAUGGGGAACUACUCUCCCAUAUCUCGACUCGGCGACCUCCUUCCGGAUCUCCGG
env-56     UCUCUUCGAGAGAACCGGUCCGUGCACGACUCGUCGACGAGCCCAUCUCGGGGCUCGUUGUCGAGUGUAUGGACCUCUCGGCUGCCCUCCAGCGACGAAUCAGGCGGCGGAUCGCUGCCUCCGAGUACGGGGAGGAACUCUCCCACACUCGGCUCGGCGAACUCCUUCCGGAUCUCCGG
env-57     UCUCUUCGAGAGAACCGGUCCGUGCACGACUCGUCGACGAACCCAUAUCGGGGCUCGUUGUCGAAUGUAUGGACCUCUCGGCUGCCCUCAGCGACGAAUCGGGCCUAACGGUUGCCGCCUCCGAGUACGGGGAAACACUCUCCCAUAUCUCGACUCGGCGAACUCCUUCCGGAACUCCGG
env-58     UCUCUUCGCGAGAACCGGUCCGUGCCCGCCUCGUCGACGAACCCGCGCCGGGCUUCGUCGUCGACAGCAUGGACCUGUCGGCAGCCCUCAACGGAGAAUCGGGGCUACGGAUCGCCACGUCCGCUUAUGGACAGCCACUGUCCAUCGAGCGGCUUGGCAAACUCCGUCCGCAACUGGCGG
env-59     UCUC................................................................................................................................................................................
env-62     UCUCUUCGAGAGAACCGGUCCGUGCACGGCUCGUCGACGAGGCCCCACCGGGCUUCGACGUCGAAAGCACGGACCUCUCGGCAGCCCUCGACGACGAAUCGGGGCUACGGAUCGCCACGUCAGCUAUGGGGAAGAACUGUCCCAUGGUCGACUCGGUGAUCUCCUUCCGCAGAUCGCGG
env-63     UCUCUUCGAGAGAACCGGUCCGUGCACGGCUCGUCGACGAGGCCCCACCGGGCUUCGACGUCGAAUGCACGGACCUCUCGGACGCCCUCGCUGAAGAAUCGGGGCUACGGAUCGCCACGUCAGCUAUGGAGAAGAACUCUCCCAUGGUCGACUCGGUGAUCUCCUUCCGCAGCUCGCGG
env-64     UCUCUUCGAGAGAACCGGUCCGUGCACGGCUCGUCGACGAGGCCCCACCGGGCUUCGACGUCGAAUGCACGGACCUCUCGGACGCCCUCGCUGAAGAAUCGGGGCUACGGAUCGCCACGUCAGCUAUGGAGAAGAACUCUCCCAUGGUCGACUCGGUGAUCUCCUUCCGCAGCUCGCGG
env-65     UCUCUUCGAGAGAACCGGUCCGUGCACGGCUCGUCGACGAGGCCCCACCGGGCUUCGACGUCGAAUGCACGGACCUCUCGGACGCCCUCGCUGAAGAAUCGGGGCUACGGAUCGCCACGUCAGCUAUGGAGAAGAACUCUCCCAUGGUCGACUCGGUGAUCUCCUUCCGCAGCUCGCGG
env-70     UCUCUUCGAGAGAACCGGUCCGUGCACGGCUCGUCGACGAGGCCCCACCGGGCUUCGACGUCGAAUGCACGGACCUCUCGGACGCCCUGCUGAAGAAUCGGGCUACGGAUCGCCACGUCAGCUAUGGAGAAGAACUCUCCCAUAGGUCGACUCGGUGAUCUCCUUCCGCAGCUCGC...
env-71     UCUCUUCGAGAGAACCGGUCCGUGCACGGCUCGUCGACGAGGCCCCACCGGGCUUCGACGUCGAAUGCACGGACCUCUCGGACGCCCUCGCUGAAGAAUCGGGGCUACGGAUCGCCACGUCAGCUAUGGAGAAGAACUCUCCCAUGGUCGACUCGGUGAUCUCCUUCCGCAGCUCGCGG
env-72     UCUCUUCGCGACGACCGGUCCGGGCGAAGCUCGUCGACGACCCCCUCCCGGGGCUCGCCGUCGAGUGGACGGACCUAUCGGCAGCGCUCGGCCGUGAGGACGCCGGCGUUCGGAUCGCGGCUUCCAGACACGGUGAACCGCUCACCGUCGGAGGCCUCGAGACGCUGGUCGGGACGCGUCG
env-74     UCUCUUCGCGACGACCGGUCCGGGCGAAGCUCGUCGACGACCCCCUCCCGGGGCUCGCCGUCGAGUGGACGGACCUCUCGGCAGCACUCGGCCGUGAGGACGCCGGCGUUCGGAUCGCGGCUUCCAGACACGGUGAACCGCUCACCGUCGGAGGCCUCGAGACGCUGGUCGGGACGCGUCG

           ....................................................................................................................................................................................
           ....................................................................................................................................................................................
           UCUYUUYR•GRRRR•YR•••••••Y•••R•••RY•RR•R•••••••••••••••••••••••••••••••••••••••••••R••••••••••••••••••••••••••••••••••••••••••••••••••••••••••••••••••••••••••••••••••••••••••••••••••
```

env-76	UUGCCUCGCGCGAACCGGUCCGCGCCAGGCUCGUCGACGAACCCCAGCCGGGGUUCG..
env-77	UUUCAUCGCGCGAACCGGUCCGCGCCAAGCUCGUCGACGAACCCCAGCCGGGGUUCGACGUCGAACGCGCGGGCCUCGCUGCAACGCUCGCCCGAGCCGACGCCGGCGUCCGCAUCGCCGCGUCCCGGUUCGGGGCCGAGCUCUCCGUCGCC...........................
env-78	UUUCAUCGCGCGAACCGGUCCGCGCCAAGCUCGUCGACGAACCCCAGCCGGGGUUCGACGUCGAACGCGCGGGCCUCGCUGCAACGCUCGCCCGAGCCGACGCCGGCGUCCGCAUCG...
Hsa-1-1	UCUAUUCGCGAAGACCGGUCCGCGCGAAGCUCGUCGACGAACCCCUUCCGGGGUACGCCGUCGAUCGGGCGGACCUGGACGAAGCGCUCUCGCGUGCCGACGCUGGCGUUCGGAUCGCCUCCUCGCGACACGGUGUCGAAGCGACGACUGGCCGUCUCGGCCAGUUGAUCGAGACGACAC
Hgi-1-1	UCUCUUCGCGAGAACCGGUCCGUGCGCGGAUCGUCGACGAGCCCCUCCCGGGCUUCGACGUAUCCCGCAUGGACCUCACGGAAGCACUCGGCAGGCCAGAUGCGGGCGUGCGAAUCGCCACGUCUCGCUAUGGCGAACCGCUGUCGGUCCCCACAUUGGGCGACUGACGGCCCGCAUCA
Hsu-2-1	UCUCUUCGCGAGAACCGGUCCGUGCGCGGAUCGUCGACGAGCCCCGUCCGGGCUUCGACGUAUCCCGCAUGGACCUCACGGAAGCACUCGGCAGGCCGGAUGCGGGCGUGCGAAUCGCCACGUCUCGCUAUGGCGAACCGCUGUCGGUCCCCACAUUGGGUGACUGACGGCCCGCAUCA
Hpr-1-1	UCUCUUCGCGAGAACCGGUCCGUGCGCGGAUCGUCGACGAGCCCCUCCCGGGCUUCGACGUAUCCCGCAUGGACCUCACGGAAGCACUCGGCAGGCCAGAUGCGGGCGUGCGAAUCGCCACGUCUCGCUAUGGCGAACCGCUGUCGGUCCCCACAUUGGGUGACUGACGGCCCGCAUCA
Hsp-8-1	UCUCUUCGCGAGAACCGGUCCGUGCGCGGAUCGUCGACGAGCCCCUCCCGGGCUUCGACGUAUCCCGCAUGGACCUCACGGAAGCACUCGGCAGGCCGGAUGCGGGCGUGCGAAUCGCCACGUCUCGCUAUGGCGAACCGCUGUCGGUCCCCAGAUUGGGCGACCUGACGGCCCGCAUCA
Hde-1-1	UCUCUUCGCGAGAACCGGUCCGUGCGCGGAUCGUCGACGAGCCCCUUCCGGGCUUCGACGUAUCCCGCAUGGACCUCACGGAAGCACUCGGCAGGCCGGAUGCGGGCGUGCGAAUCGCCACGUCUCGCUAUGGCGAACCGCUGUCGGUCCCCACAUUGGGUGACCUGACGGCCCGCAUCA
Hlu-1-1	UCUCUUCGCGAGAACCGGUCCGUGCGCGGAUCGUCGACGAGCCCCUCCCGGGCUUCGACGUAUCCCGCAUGGACCUCACGGAAGCACUCGGCAGGCCGGAUGCGGGCGUGCGAAUCGCCACGUCUCGCUAUGGCGAACCGCUGUCGGUCCCCACAUUGGGCGACUGACGGCCCGCAUCA
Hal-2-1	UCUCUUCGCGAGAACCGGUCCGUGCGCGGAUCGUCGACGAGCCCCUCCCGGGCUUCGACGUAUCCCGCAUGGACCUCACGGAAGCACUCGGCAGGCCAGAUGCGGGCGUGCGAAUCGCCACGUCUCGCUAUGGCGAACCGCUGUCGGUCCCCACAUUGGGUGACUGACGGCCCGCAUCA
Hsp-6-1	UCUCUUCGCGAGAACCGGUCCGUGCGCGGAUCGUCGACGAGCCCCUCCCGGGCUUCGACGUAUCCCGCAUGGACCUCACGGAAGCACUCGGCAGGCCGGAUGCGGGCGUGCGAAUCGCCACGUCUCGCUAUGGCGAACCGCUGUCGGUCCCCACAUUGGGCGACUGACGGCCCGCAUCA
Hme-1-1	UCUCUUCGCGAGAACCGGUCCGUGCGAGGAUCGUCGACGAGCCCCUUCCGGGCUUCGACGUAUCCCGCAUGGACCUCACGGAAGCACUCGGCAGGCCAGAUGCGGGCGUGCGAAUCGCCACGUCUCGCUAUGGCGAGUCGCUGUCGGUCCCCCGACUGGGUGACCUGACGGCCCGCAUCA
Hla-2-1	UCUCUUCGCGAGAACCGGUCCGUGCGAGGAUCGUCGACGAGCCCCUUCCGGGGUUCGACGUAUCCCGCAUGGACCUCACGGAAGCUCUCGGCAGACCGGAUGCGGGCGUGCGAAUCGCCACGUCUCGCUAUGGUGAGCCGCUUUCGGUCCCCCGACUGGGGGAACUGACAGCCCGUAUCG
Hel-1-1	UCUCUUCGCGAGAACCGGUCCGUGCGAGGAUCGUCGACGAGCCCCUUCCGGGGUUCGACGUAUCCCGCAUGGACCUCACGGAAGCUCUCGGCAGACCGGAUGCGGGCGUGCGAAUCGCCACGUCUCGCUAUGGUGAGCCGCUUUCGGUCCCCCGACUGGGGGAACUGACAGCCCGUAUCG
Hmu-1-1	UCUCUUCGCGAGAACCGGUCCGUGCGAGGAUCGUCGAUGAACCCCUUCCGGGGUUCGACGUAUCCCGCAUGGACCUCACGGAAGCACUCGGCAGGCCAGAUGCGGGCGUGCGAAUCGCCACGUCUCGCUAUGGCGAGUCGCUGUCGGUCCCCCGACUGGGCGACCUGACGGCCCGCAUCA
env-79	UCUCUUCGCGAGAGCCGGUUCGCGGCGAAACUGACCGAGGAGCCAGUCGCUGGGUUACUCGGUAGACGCCGCGAGCCUCGAUUCGGCAGUUAGCCGCGACGACGCUGGUUUCACCAUCGCUACUUCGCGAUUUGGUGAGCCAGUUACCACGUCGACACUCAACACACUCGUCCCAGCACGG
env-80	UCUCUUCGCGAGAGCCGGUCCGAGCAAAGUUAGUCGACGUCGACCCAUCUGGGUUCGUUGUCGACCGCUCGGACCUCGAUACGGCAGUGUCGCGCGCGGAUGCAGGUCUCACUAUCGCCGCUUCGCGGUACGGUGAGCAACUCACGCCGCAGCGCCUCGACGAGAUCGCCCCCAGUGUC
env-81	UCUCUUCGCGAGAGCCAAUCCGAGCAAAACUCGUCGACACCUCACUCACGGGGUAUCACGUCGACCGCUCGGAGAUCGAUACGGCAGUCGACCGCUCGAAUGCAGGACUCACUAUUGCCGCUUCGCGGUAUGGUAAGUCGCUUGCGCCGGGUCGCCUCGACGACAUCGCACGCCAGGUC
env-82	UCUCUUCGCGAGAGCCGGUCCGAGCAAAGCUCGUCGACGUCGACCCAUCUGGGUUCGUCGUCGACCGCUCGGACCUCGAUACGGCAGUGUCGCGCUCGGAUGCAGGACUCGCUAUCGCCGCUUCGCGGUACGGCGAGGAACUCACACCGAAGCGCCUCGACGAGAUCGCCCCACAGUGUC
env-83	UCUCUUCGCGAGAGCCGGUCCGAGCAAAGCUCGUCGACGUCGACCCAUCUGGGUUCGUCGUCGACCGCUCGGACCUCGAUACGGCAGUGUCGCGCUCGGAUGCAGGACUCGCUAUCGCCGCUUCGCGGUACGGCGAGGAACUCACACCGAAGCGCCUCGACGAGAUCGCCCCACAGUGUC
Hla-4-1	UCUCUUCGCGACGACCGGUCCGUGCGAAAAUCGUCGACAAGCCCACACAGGGGCUAUCGAUCGAGCAAACGGACCUUUCGGCGGCUCUCAGCCGUGAGGACGCCGGCGUCCGAAUCGCAGCUUCCCGGUUCGGUGAACCGCUCACCGUGGGGAAACUCGAGACGCUGGCCGGACGCACUCG
Ssp-1-1	UCUCUUCGCGACGACCGGUCCGCGCCAAGAUCGUCGACGAGCCCCUCCCGGGCUUCGAGGUUGAUCGCGCGGACCUGCCGACGGCCAUCGAGGACUCCCGGAACACGUGUCGCAUCGCCGCGUCCCGCCACGGGACGCCGCUGACCGCGACCCGACUGACGGACCUCGUCGGACAUCUCC
env-84	UCUCUUCGCGACGACCGGUCGCGCGCGAAACUCGUCGAUGAGCCCCGUCCGGGCUUUAUCGUCGAACGCGUGGACCUCCGGGACGCUCUCGACCGGCCGACG..
env-86	UCUCUUCGCGACGACCGGUCCGUGCCAAGCUCGUCGACGAGCCCCCUCGGGGCUGUCGGUCGAGCGCGCGGACCUGCGGGCAGCUCUCGGCCGUGAGGAUGCUGGCGUCUGCAUCGCGUCGUCCCGGUUCGGUGAGGAACUCACCGUCGGGCGACUCGGGACGCUGGCCGGACGCAUCG
Hsp-11-1	UCUCUUCGCGACGACCGGUCCGUGCGAAAAUCGUCGGCAAGCCCACACAGGGGUUGUCGAUCGAGCAAACGGACCUUUCGGCGGCUCUCAGCCGUGAGGAUGCCGGCGUUCGAAUCGCAGCUUCCCGGUUCGGUGAAGCGCUCACCGUGGGGAAACUCGAGACGCUGGCCGGACGAAUCG
env-87	UCUCUUCGUAGAACCGGUCCGCGCCCGCAUCGUCGACGAGCCCCUCCGGGGCCGACGGUGCUGGAACAGGAUCUGUCGACAACGCUCGGAGCGGCGGACGCGGGGUUGUAUCGCGGCCGCGCCACGGCUCGCGCUCACGGCGGCCGUCUGGCCGACCUGCCGGGCUCCGGCG
env-88	UCUCUUCGCGAGAACCGGUCCGUGCCCGCCUCGUCGACGAACCCCGCGCCGGGCUUCGUCGUCGACAGCAUGGACCUGUCGGCAGCCCUUUCGGUCGAAUCGGGGCUACGGAUCGCCACAUCCGCUUAUGGACAGCCACUGUCCAUCGAGCGGCUUGGCCAACUCCGUCCGCAACUGGCGG
env-89	UCUCUU..
Hka-1-1	UCUCUUCGCGACGGCCAGUCCGCGCAAAGCUCGUCGACGAGCCCCUCACCGGGUACGUCGUCGACACCGCGGACGUGGAUACGGCACUCGAACGGUCCGACGCCGGGUUCGCCAUCGCCGCAUCGCGGUAUGGUGAGGAACUCGGCGUCGACCGGCUCGGCCAACUGGUCGAGCGCCGGG
Hmu-2-1	UCUCUUCGCGACGGCCAGUCCGCGCAAAGCUCGUCGACGAGCCCCUCACCGGGUACGUCGUCGACACCGCGGACGUGGAUACGGCACUCGAACGGUCCGACGCCGGGUUCGCCAUCGCCGCAUCGCGGUAUGGUGAGGAACUCGGCGUCGACCGGCUCGGCCAACUGGUCGAGCGCCGGG
Hca-3-1	UCUCUUCGCGACGCGGUACGGGCGAAGCUCGUCGACGAGCCCCGCGGCAGGGUUCGUCGUCGACUCGACCGAUAUCGACGACGCACUCGGCGCGUCCGGACGCCGGGCUGACCAUCGCGGCUCUCGCUACGGCGAGGAGCUCACGUCACGAAGCUCGACCAGCUGGUCGAACGGCUUC
Hsa-5-1	UCUCUUCGCGAGAGCCGGUCCGCGCGCGGAUCGUCGACGAGCCCCGCACCCGGGUUCGCCGUCGAGCGCGCGGACCUCUCGGCAGCGCUCGGCCGUGAGGACGCCGGGCUCCGGAUCGCCACGUCGCGCCACGGUGAGGCGCUCACGACGGAUCGACUCGGAGUGCUGGCCGGACGGUCG
env-90	UCUCUUCGCGAGAGCCGGUUCGCGCACGGAUCGUCGACGAG...
Hag-1-1	UCUCUUCGCGAGAGCCGGUUCGCGCGCGGAUCGUCGACGAGCCCCGCACCCGGGUUCGCCGUCGAGCGUGCGGACCUCUCGGCAGCGCUCGGCCGUGAGGACGCCGGGCUCCGGAUCGCCACGUCGCGCCACGGUGAGGCGCUCACGACGGAGCGACUCGGGGUGCUGGCCGGACGGUCG
Hsa-4-1	UCUCUUCGCGAGAGCCGGUCCGCGCGCGGAUCGUCGACGAGCCCCUUCCGGGGUUCGUCGUCGAGCGCGCGGACCUCUCGGCAGCGCUCGGCCGUGAGGACGCCGGGCUCCGGAUCGCCACGUCGCGCCACGGUGAGGCGCUCACGACGGAGCGACUCGGAGUGCUGGCCGGACGGUCG
env-91	UCUCUUCGCGAGAGCCGGUCCGGGCGAAGCUUAUCGACCAGCCCCCGUCGGGCUACGCUGUCGACGCCGCGGACCUCGAUACAGCGCUCGCGCGCGAGGACGCAGGACUGAGAAUCGCGUCUCGCGGCACGGCGAGCGCCUGCGCCGGGUCGACUCGACAACCUCGCGGCGGCGUGCC
env-92	UCUCUUCGAGAGAACCGGUUCGCGCGCGCCUCGUCGANGAGCCCACACCGGGCUUCGACGUGGCGACUGUGAACCUGUCGGCCGCCCUUGCCGACGCCGCCGGACGACGGAUCGCCACCUCCCGUCAUGGACAGGAGCUGUCCAUCACGCGACUGGCGGAGUUCCGUCCCGACUACCGG
env-93	UCUCUUCGAGAGAACCGGUUCGCGCGCGCCUCGUCGANGANCCCACACCGGGCUUCGACGUGGCGACUGUGAACCUGUCGGCCGCCCUUGCCGACGCCGCCGGACGACGGAUCGCCACC..
env-94	UCUCUUCGAGAGAACCGGUUCGCGCGCGCCUCGUCGACGAGCCCACACCGGGCUUCGACGUGGCGACUGUGAACCUGUCGGCCGCCCUUGCCGACGCCGCCGGACGACGGAUCGCCACCUCCCGUCAUGGACAGGAGCUGUCCAUCA...................................
env-95	UCUCUUCGAGAGAACCGGUUCGCGCGCGCCUCGUCGACGAGCCCACACCGGGCUUCGACGUGGCGACUGUGAACCUGUCGGCCGCCCUUGCCGACGCCGCCGGACGACGGAUCGCCACCUCCCGUCAUGGACAGGAGCUGUCAUCNCGCGACUGGCGGAGUUCCGUCCCGACUACCGG
env-96	UCUCUUCGAGAGAACCGGUUCGCGCGCGCCUCGUCGACGAGCCCACACCGGGCUUCGACGUGGCGACUGUGAACCUGUCGGCCGCCCUUGCCGACGCCGCCGGACGACGGAUCGCCACGUCCCGUCAUGGACAGAUGCUGUCCAUCGCGCGACUGGCUGAGUCCCGCCCGACUAACCGG
env-98	UCUCUUCGAGAGAACCGGUUCGCGCGCGCCUCGUCGACGAGCCCACACCGGGCUUCGACGUGGCGACUGUGAACCUGUCGGCCGCCCUUGCCGACGCCGCCGGACGACGGAUCGCCACGUCCCGUCAUGGACAGCAGCUGUCCAUCGCGCGACUGGCGGAGUUCCGCCCCGACUACUGCG
env-99	UCUCUUCGAGAGAACCGGUUCGCGCGCGCCUCGUCGACAAGCCCACACCGGGCUUCGAUGUGGCGACUGUGAACCUGUCGGCCGCCCUUGCCGACGCCGCCGGACGACGGAUCGCCACCUCCCGUCAUGGACAGCAGCUGUCCAUCGCGGGCGACUGGCGGAGUUCCGCCCCGCACGGGACG
env-101	UCUCUUCGAGAGAACCGGUCCGUGCACGACUCGUCGACGAGCCCAUAUCGGGGUUCGACGUCGAAUGCAUGGGCCUCUCGGCUGCCCUCAGCGACGAAUCGGGGCUCCGGAUCGCCGCCUCCGAGUACGGGAAAGAGCUGUCCCACACUCGGCUCGGCGACCUCCUGCCGCAGUUGGCAG
env-102	UCUCUUCGAGAGAACCGGUUCGUGCACGACUCGUCGACGAGCCCACAACGGGGUUCGACGUCGAAUGUACGGGCCUCUCGGCUGCCCUCAGCGACGAAUCGGGCCUCACAAUCGCCACCUCGGCCUACGGAGACAAACUCUCUUCUGGCCGACUUGGCGAACUCCUUUCGGAACUCCGGG
env-103	UCUCUUCGAGAGAACCGGUUCGUGCACGACUCGUCGACGAACCCACAACGGGGUUCGACGUCGAAUGUACGGGCCUCUCGGCUGCCCUCAGCGACGAAUCGGGCCUCACAAUCGCCACCUCGGCCUACGGAGACAAACUCUCUUCUGGCCGGCUUGGCGAACUCCUUUCGGAACUCCGGG
env-104	UCUCUUCGAGAGAACCGGUCCGUGCACGCCUCGUCGACGAACCCAUAUCGGGGCUCGUUGUCGAACGUGCGGACCUCUCGGCUGCCCUCAGCGACGAAUCGGGCGCAACGAUCGCCACCUCGGCCUACGGGGAGAGCGUCUCCCACGGUCGACUCGGCGAACUCCUUC............
env-105	UCUCUUCGAGAGAACCGGUCCGUGCACGACUCGUCGACGAACCCAUAUCGGGGUUCGACGUCGAAUGUACGGGCCUCUCGGCUGCCCUCAGCGACGAAUCGGGCCUCACAAUCGCCACCUCGGCCUACGGAGACAAACUCUCUUCUGGCCGGCUUGGCGAACUCCUUUCGGAACUCCGGG
env-106	UCUCUUCGAGAGAACCGGUCCGUGCACGACUCGUCGACGAGCCCAUAUCGGGGCUCGUUGUCGAAUGUAUGGGCCUCUCGGCUGCCCUCAGCGACGAAUCAGGCCUCACGGUUGCCGCCUCCGAGUACGGGGAGAAACUCACCCAUACUCGACUCGGCGGACUCCUUCCGGAACUCCGGG
env-107	UCUCUUCGAGAGAGCCGGUCCGCGCACGGAUCGUCGACGAACCCUUGCCUGGGUUCGAGAUCCAGCGUGCGGACCUGCCGGAAGUGCUGGCCCGGCCCGACGCCGGGGCUC...
env-108	UCUCUUCGCGAGAGCCGGUCCGAGCACGGAUCGUGGACGAGCCCGCACCGGGCUACACGAUCCUGAC...
env-109	UCUCUUCGCGACGGCCGGUCCGCGCCAAGAUCGUCGACG..
CHa-1-1	UCUCUUCGAGAGAACCCGUCCGCGCCCGCAUCGUGGACGACCCCCACCGGGGUUCGCGUCGCGGGCGAGGACGUCGCGGACGCCGCCGCCGCCGCCGAACUGGCGGUCGCCACCUCGCGGCACGGACGCCCGCUGUCCGUGUCGGGACUGAGCGAUCUCCACGCGCGACUGGACGACA
Hpa-2-1	UCUCUUCGCGAGAACCGGUCCGUGCGCGGAUCGUGGACGAGCCCGUUCCGGGGUUCUCCAUCGCCCGCACGGACCUCGAGGAAGCACUCGGCCGCUCCGACGCGGGCGUCACUAUCGCGACGUCCCGCCACGGAGAGUCGCUGACGGUGCCGAAGCUAGCGGAACUCUCCCCGCGAAUCG
env-110	UCUCUUCGCGACGACCGGUCCGAGCGAAAUCUCGAAGACAGCCCCCUCCCGGGGCUUGUAUCGAGCGGACGGACCUACAGGCAGCACUCAGCCGUGAGGACGCCGGCUCUGCAUCGCGUCUCGCUACGGUGAACCGCUCACCGUCGGGCGACUCGAGACGCUGGCCGGACGGAUG
Nhu-1-1	UCUCUUCGCGACGACCGGUCCGGGCGAAACUCGUCGACGAGCCCCUCCCGGGGCUUACCGUCGAGCGGACGGACCUUCAGGCGGCUCUCAGCCGUGAGGACGCCGGCGUUCGAAUUGCGGCUUCCCGAUUCGGUGCAGAACUCACCGUCGGGCGGCUCGGAACGCUGGCCGGACACACAG
env-113	UCUCUUCGAGAGAACCGGUCCGCGCCCGUAUCACCGGGAAGCCGAGGCCGGCUUCCAGGUCGCGGCCGCGGACCUGUCGGAAGCGCUCGCCGGCGACGGACUGGCCGUCGCAACGUCGCGACACGGACGGCACUCUCCGUCUCGCGGCUCGGCGACCUCGUUUCGGACGCGCGGGAUG
env-114	UCUCUUCGAGAGAACCGGUUCGCGCGCGCCUCGUCGAUAAGCCCACACCGGGCUUCGACGUGGCGACUGUGAACCUGUCGGCCGCCCUUGCCGACGCCGCCGGACGACGGAUCGCCACCUCCCGUCAUGGACAGGAGUUGUCCAUCGGGCGGCUGGCGGAGUUCCGCCCCGACUACCGCG
env-115	UCUCUUCGAGAGAACCGGUUCGCGCGCGCCUCGUCGAUAAGC..
env-116	UCUCUUCGAGAGAACCGGUCCGUGCACGACUCGUCGACGAGCCCACAACGGGGUUCGACGUCGAAUGUACGGGCCUCUCGGCUGCCCUCGACGAUGAAUCAGGCAUAACGGUCGCCGCCUCCGAGUAUGGCGAGAUACUCUCGCAUGCGCGGCUCGGCGAACUCCUUCCGGAUCUCCGGG
env-117	UCUCUUCGAGAGAACCGGUCCGUGCACGNCUCGUCGACGAACCCAUAUCGGGGCUCGUUGUCGAACGUGCGGACCUCUCGGCUGCCCUCGACGACGAAUCGGGCGUAACGGUUGCCGCCUCCGAGUAUGGGGAGAAACUCUCCCACACUCGACUCGGCGACCUCCUUCCGGGUCUCCGGG
env-118	UCUCUUCGAGAGAACCGGUCCGUGCACGACUCGUCGACGAACCCAUAUCGGGGCUCGUUGUCGAACGUGCGGACCUCUCGGCUGCCCUCAGCGAUGAAUCAGGCAUAACGGUCGCCGCCUCCGAGUAUGGCGAGAUACUCUCGCAUGCGCGGCUCGGCGAACUCCUUCCGGAUCUCCGGG
env-119	UCUCUUCGAGAGAACCGGUCCGUGCACGACUCGUCGACGAACCC..
env-120	UCUCUUCGCGAGAGCCCGUUCGCGCCAAGCUCGUCGACGACACGGUGUUGUCGUCGAGCGCGCGGACGUGGGGAGCAUCACGGCCGACGCCGACGUGUCGAUCGCCGCCUCCCGCCACGGGGAACGUCUCUCCGUGCGCGGUUGGAGACGGUGACGGCCGCACUACGCGACGCAGACC
env-122	UCUCUUCGCGAGAGCCCGUUCGCGCCAAGCUCGUCGACGACACCGCGUUGUCGGUCGAGCGCGCGGACGUGGGGAGCGUCACGGCCGACGCCGACGUGUCGAUCGCCGCCUCCCGCCACGGGGACACACUCUCCGUGCCGGCGCUGGAGACGGUGACGACGCAGUUGGCGACGGCCGACC
env-123	UCUCUUCGCGAGAGCCGGUUCGCGCCAAACUCGUCGACGACACCGUGUUGUCGGUCGAGCGCGCGGACCUGGGGAGCGUCACAGCCGACGCCGACGUGUCGAUCGCCGCCUCCCGCCACGGGGACACACUCUCCGUGCCGGCGUUGGAGGUGGUGACGACGGAGUUGGUGGCGGCAGACC
env-126	UCUCUUCGCGAGAACCGGUCCGUGCGCGGAUCGUCGAUCGGCCCCUUCCGGGGCUUCGACGUCUCCCGUAUGGACCUCGAGGAAGCACUCGGCCGCGCCGACGCGGGGGUCGUAAUCGCGACGUCCCGCCACGGCGAGGCGCUGACCACCACGCGGCUGCCGGAUCUCGCCGCCCGUAUCG
env-127	UCUCU...
env-128	UCUCUUCGCGAGAACCGGUCCGUGCGCGGAUCGUCAAUCGGCCCCUUCCGGGCUUCGACGUGUCCCGUAUGGACCUCGAGGAAGCACUCGGCCGCGCCGACACGGGGUGUCGCUAUCGCGACAUCCCGCCACGGCGAGGCGCUGACCACCACGCGGCUGCCGGAUCUCGCCGCCCGCAUCG
env-129	UCUCUUCGCGACGACCGGUCCGCGCACGGCUGGUGAACGGGCCAACGGGGCUCACAGUCGAGCGUGUGGACCUAUCGGCGGCGCUCGGCCGUGAGGACGCCGGCGUCCGUAUCGCGACGUCGAGGCUCGGUGAACCGCUCACCGUCGCUCGACUGCGGACGCUGGCCGGACGGGUCGAGG
Hna-1-1	UCUCUUCGAGAGAGCCGGUCCGCGCACGGAUCGUCGACGAACCCCUGCCGGGGUUCGAGGUCCAACGCGCGGACCUGCCGGACGUGCUGGCCCGCCCCGACGCCGGGGUUCGGAUCGCCACCUCCCGUCCACGGGACGGAACUGUCGGUCGGUCGCCUGACGGAACUCGUCGGGCGGAUCG
env-130	UCUCUUCGAGAGAGCCGGUCCGCGCACGGAUCGUCGACGACCCCCUACCGGGGUUCGAGGUCCAACGCGCGGACCUGCCGGAAGUGCUGGCCCGCCCCGAUGCGGAGNUCGGAUCGCCACGUCCGUCCACGGGACGGAACUGUCGGUCGGUCGCCUGACCGGGUUGGUCGGGCGCAGG
env-131	UCUCUUCGCGAGAACCCGUCCGCGCCCGCCUCGUCGACGAGCCCCACCGCGGGCUACGUUGUCGAGGCCGCGGACAUCCGGAAACCCUCGCCCGUCAGAACGCGGGCGUCCGGAUCGGCACCUCACGCCACGGUGAUGACCUCACCGUGUCGCGACUCGCCGAACUGGGCUCGCAGUGUC
env-134	UCUCUUCGCGACGGCCGGUUCGCGCGCGGAUCGUCGACGAACCCCACCGGGGUUCGCCGUCGAGCGCGCGACACUCGAUGACGCCCUCGCCAGGGAGGACGCAGGCGUG...
env-135	UCUCUUCGCGACGACCGGUCCGGGCGAAGCUCGUCGAU...
Nph-1-1	UCUCUUCGCGAGAGCCGGUUCGCGCAAAGCUCGUCGACGAAUCCCCACCGGGAUUCGCCGUCGACUGUGCGGACCAUCGAAGCCAGUCUCGCCCGCCCGGACGCCGGCGUCCGUAUCGCCGCGUCCCGGCAUGGGGAGACGCUUUCGGUCGACCGUCUCGACGGUAUCGUCGGUCGGACGA
env-136	UCUCUUCGCGAGAGCCGGUUCGCGCAAAGCUCGUGGCCGAGCCCCCACCGGGGUUCGCCGUCGAGCGCGCGACCAUCGCAGCAGAUGCCUCACCCGUCCGGACGCUGGCGUCCGUAUCGCCGCGUCCAGACACGGCGAGGUGUUGACGGUCGAUCUGCUCGGCGACAUCGUCGAGCGAUCGA
env-137	UCUCUUCGCGAGAGCCGGUUCGCGCAAAGCUCGUGGCCGAGCCCCCACCGGGGUUCGCCGUCGAGCGCGCGACCAUCGCAGACUGCCUCACCCGUCGGACGCUGGCGUCCGUAUCGCCGCGUCCAGACACGGCGAGGUGUUGACGGUCGAUCUGCUCGGCGACAUCGUCGAGCGAUCGA
env-138	UCUCUUCGAGAGAACCGGUCCGCGCCCGGAUCACCGGGAAGCCGUCGUCGGCUUCCAGGUGGUGGGUCGGACGGACCUGUCGGAAGCGCUCGUCGAUGGCGGACUCGCCGUCGCAACGUCGCGACACGGAGCGAACCUCUCCGUCUCGCGGCUCGGCGAACUCGUCGCCGACGCGUCGAAGG
env-139	UCUCUUCGAGAGAACCGAUCCGCGCCCGUAUCACUGGAAAGCCGGCGGUCGGCUUCCAGGUCGUGGACGCGGACCUGUCGGAAGCACUCGACGCCGACGGACUCGCCGUCGCCACGUCGCGACACGGGGAGCAGCUCUCCGUUUCGCGGCUCGGUGAGAUCGUCACGGACGCACGGACGG
env-140	UCUCUUCGAGAGAACCGGUCCGCGCCCGCAUCACCGGAAAGCCGGCGGACGGCUUUCAGGUCGUUAGCGCGGACCUGUCGGCAGCACUCGAUGACGGACUCGCCAUCGCAACGUCGCGACACGGGGAAUCGCUCUCCGUCUCGCGGCUCGGCGAGAUCGUCGCGGACGCACGGGAGGCUG
env-145	UCUCUUCGAGAGAACCGGUCCGCGCCCGCAUCACCGGAAAGCCGGAGGACGGCUUUCAGGUCGUGGCCGCGGACCUGUCGGAAGCGCUCGCCGGCGACGAACUCGCCAUCGCGACGUCGCGGCACGGGGAACCGCUCUCCGUCCCGCGGCUCGGCGAACUCGUCGCCGGCGCACGGAGG
env-146	UCUCUUCGAGAGAACCAGUCCGCGCCCGCAUCACCGGGAAGCCGGAGGUCGGUUUCCAGGUCGUGGGCGCGAGGCUGUCGGAAGCGCUCGCCGGCGACGGAGUGGCCGUCGCGACGUCGCGACACGGAGAAGAACUCUCCGUCUCGCGGCUCGGCGACCUCGUUUCGGACGCGCGGAAGG
	....
	....
	UCUYUUYR•GRRRR•YR••••••Y•••R•••RY•RR•R••R•••

```
env-147   UCUCUUCGAGAGAACCGGUCCGCGCCCGCAUCACCGGAACGCCGGCGGACGGCUUUCAGGUCGUGGGAGCGGACCUUUCGGAAGCGCUCGGCGGCGACGGACUCGCCGUCGCGACAUCGCGGUACGGGGAGGAGCUCUCCGUCCCGCGACUGGGCGAUAUCGUCUCGGACGCACGCGAAG
env-148   UCUCUUCGAGAGAACCGGUCCGCGCCCGCAUCACCGGGAAGCCGGAGGACGGUUUCCAGGUCGUGGGUGCGGACCUUUCGGAAGCGCUCGACGGCGACGGACUCGCCGUCGCAACGUCACGACACGGGGAAGAGCUCUCCGUCUCGCGGCUGGGCGACAUCGUCUCGGACGCACGGGAGG
Hsp-9-1   UCUCUUCGAGAGAACCGGUCCGCGCCCGCAUCGCCGGGAAGCCCGAGGAGGGUUUCCAGGUCGUGGCCGCGGACCUGUCGGAAGCGCUCGCCGGCGACGGACUGGCCGUCGCGACGUCGCGACACGGAGCAGAACUCUCCGUCUCGCGGCUCGGCGACCUCGUCUCGGACGCGCGGAAGG
Hez-1-1   UCUCUUCGAGAGAACCGGUCCGCGCCCGCAUCACCGGGAAGCCGGAGGUCGGCUUCCAGGUCGCGGCCGCGGACCUGUCGGAAGCGCUCGCCGGCGACGGACUGGCCGUCGCGACGUCGCGACACGGAGCGGAACUCUCCGUCUCGCGGCUCGGCGACCUCGUCUCGGACGCCCGCGAGG
env-149   UCUCUUCGAGAGAACCGGUCCGCGCCCGCAUCACCGGGAAGCCGGAGGACGGUUUCCAGGUCGUGGACGCGGACCUGUCGGAAGCGCUCGCCGGCGACGGACUGGCCGUCGCGACGUCGCGACACGGGGAGGAACUCUCCGUCUCGCGGCUCGGCG.......................
env-150   UCUCUUCGAGAGAACCGGUCCGCGCCCGCAUCACCGGGAAGCCGGAGGACGGUUUCCAGGUCGUGGACGCGGACCUGUCGGAAGCGCUCGCCGGCGACGGCUUGGCCGUCGCGACGUCGCGACACGGGGAGGAACUCUCCGUCUCGCGGCUC...........................
env-151   UCUCUUCGAGAGAACCGGUCCGCGCCCGCAUCACCGGGAAGCCGGAGGUCGGUUUCCAGGUCGCGGCCGCGGACCUGUCGGAAGCGCUCGCCGGCGACGGACUGGCCGUCGCGACGUCGCGACACGGAGCGGAACUCUCCGUCUCGCGGCUCGGCGACCUCGUCUCGGACGCCCGCGAGG
Hco-1-1   UCUCUUCGAGAGAACCGGUCCGCGCCCGCAUCACCGGGAAGCCGGAGGUCGGUUUCCAGGUCGCGGCCGCGGACCUGUCGGAAGCGCUCGCCGGCGACGGACUGGCCGUCGCGACGUCGCGACACGGAGGGGAACUCUCCGUCUCGCGGCUCGGCGACCUCGUCUCGGACGCCCGCGAGG
env-152   UCUCUUCGAGAGAACCGGUCCGCGCCCGCAUCACCGGGAAGCCGGAGGACGGUUUCCAGGUCGUGGACGCGGACCUGUCGGAAGCGCUCGCCGGCGACGGACUGGCCGUCGCGACGUCGCGACACGGGGAGGAACUCUCCGUCUCGCGGCUCGGCGGCAUCGUCUCGGACGCGCGGAAGG
env-153   UCUCUUCGAGAGAACCGGUCCGCGCCCGCAUCACCGGGAAGCCGGAGGUCGGCUUCCAGGUCGCGGCCGCGGACCUGUCGGAAGCGCUCGCCGGCGACGGACUGGCCGUCGCGACGUCGCGACACGGAGCGGAACUCUCCGUCUCGCGGCUCGGCGACCUCGUCUCGGACGCCCGCGAGG
env-154   UCUCUUCGAGAGAACCGGUCCGCGCCCGCAUCACCGGGAAGCCGGAGGUCGGCUUCCAGGUCGCGGCCGCGGACCUGUCGGAAGCGCUCGCCGGCGACGGACUGGCCGUCGCGACGUCGCGACACGGAGCGGAACUCUCCGUCUCGCGGCUCGGCGACCUCGUUUCGGACGCCCGCGAGG
env-155   UCUCUUCGAGAGAACCGGUCCGCGCCCGCAUCACCGGGAAGCCGGAGGCCGGCUUCCAGGUCGCGGCCGCGGACCUGUCGGAAGCGCUCGCCGGCGACGGACUGGCCGUCGCAACGUCGCGACACGGAGCGGCACUCUCCGUCUCGCGGCUCGGCGACCUCGUUUCGGACGCGCGGGAGG
env-156   UCUCUUCGAGAGAACCGGUCCGCGCCCGCAUCACCGGGAAGCCGGAGGUCGGCUUCCAGGUCGCGGCCGCGGACCUGUCGGAAGCGCUCGCCGGCGACGGACUGGCCGUCGCGACGUCGCGACACGGAGCGGAACUCUCCGUCUCGCGGCUCGGCGACCUCGUCUCGGACGCCCGCGAGG
Hsp-10-1  UCUCUUCGAGAGAACCGGUCCGCGCCCGCAUCACCGGGAAGCCGGAGGACGGUUUCCAGGUCGUGGCCGCGGACCUAUCGGAAGCGCUCGCCGGCGACGGACUGACCGUCGCCACGUCGCGACACGGGGAGGAACUCUCCGUCUCGCGGCUCGGCGACGUCGUCUCGGACGCGCGGAAGG
Hli-1-1   UCUCUUCGAGAGAACCGGUCCGCGCCCGCAUCACCGGGAAGCCCGAGGAGGGGUUCCAGGUCGUGGGAGCGGACCUGUCGGAAGCGCUCGCCGGCGACGGCUUGGCCGUCGCGACGUCGCGACACGGGGAGGAACUCUCCGUCUCGCGGCUCGGCGGCAUCGUCUCGGACGCGCGGGAGG
Hte-1-1   UCUCUUCGAGAGAACCGGUCCGCGCCCGCAUCACCGGGAAGCCCGAGGCGGGCUUCCAGGUCGUGGCCGCGGACCUGUCGGAAGCGCUCGCCGGCGACGGACUGGCCGUCGCGACGUCGCGACACGGGGAGGAACUCUCCGUCUCGCGGCUCGGCGACCUCGUCUCGGACGCCCGCGAGG
Hko-1-1   UCUCUUCGAGAGAACCGGUCCGCGCCCGCAUCACCGGGAAGCCGGAGGACGGCUUCCAGGUCGUGGAAGCGGACCUGUCGGAAGCGCUCGCCGGCGACGGUUUGGCCGUCGCGACGUCGCGACACGGGGAGGAACUCUCCGUCUCGCGGCUCGGCACCAUCGUCUCGGACGCGCGGGAGG
Hli-2-1   UCUCUUCGAGAGAACCGGUCCGCGCCCGCAUCGCCGGGAAGCCCGAGGAGGGGUUCCAGGUCGUGGCCGCGGACCUGUCGGAAGCGCUCGCCGGCGACGGAGUGGCCGUCGCGACGUCGCGACACGGAGAAUCGCUCUCCGUCUCGCGGCUCGGCGACCUCGUCUCGGACGCGCGGAAGG
Hsa-7-1   UCUCUUCGAGAGAACCGGUCCGCGCCCGCAUCACCGGGAAGCCGGAGGACGGUUUCCAGGUCGUGGAAGCGGACCUGUCGGAAGCGCUCGCCGGCGACGGCUUGGCCGUCGCGACGUCGCGACACGGGGAGGAACUCUCCGUCUCGCGGCUUGGCACCAUCGUCUCGGACGCGCGGGAGG
Hca-2-1   UCUCUUCGAGAGAACCGGUCCGCGCCCGCAUCGCCGGGAAGCCCGAGGAGGGGUUCCAGGUCGUGGCCGCGGACCUGUCGGAAGCGCUCGCCGGCGACGGACUGGCCGUCGCGACGUCGCGACACGGAGAAUCGCUCUCCGUCUCGCGGCUCGGCGACCUCGUCUCGGACGCCCGCGAGG
Hsp-7-1   UCUCUUCGAGAGAACCGGUCCGCGCCCGCAUCGCCGGGAAGCCCGAGGAGGGCUUCCAGGUCGUGGCCGCGGACCUGUCGGAAGCGCUCGCCGGCGACGGAGUGGCCGUCGCGACGUCGCGACACGGAGAAGCACUCUCCGUCUCGCGGCUCGGCGACCUCGUCUCGGACGCCCGCGAGG
Hha-2-1   UCUCUUCGAGAGAACCGGUCCGCGCCCGCAUCACCGGGAAGCCCGAGGAGGGGUUCCAAGUCGUGGGGGCGGACCUGUCGGAAGCGCUCGCCGGCGACGGCUUGGCCGUCGCGACGUCGCGACACGGAGAAGAGCUCUCCGUCUCGCGGCUCGGCACCAUCGUCUCGGACGCGCGGAAGG
Hdi-1-1   UCUCUUCGAGAGAACCGGUCCGCGCCCGCAUCGCCGGGAAGCCCGAGGAGGGGUUCCAGGUCGUGGCCGCGGACCUGUCGGAAGCGCUCGCCGGCGACGGAGUGGCCGUCGCGACGUCGCGACACGGAGAAUCGCUCUCCGUCUCGCGGCUCGGCGACCUCGUCUCGGACGCGCGGAAGG
Hsa-9-1   UCUCUUCGAGAGAACCGGUCCGCGCCCGCAUCACCGGGAAGCCCGAGGAGGGCUUCCAGGUCGUGGACGCGGACCUGUCGGAAGCGCUCGCCGGCGACGGCUUGGCCGUCGCGACGUCGCGACACGGGGAGGAACUCUCCGUCUCGCGGCUCGGCACCAUCGUCUCGGACGCGCGGAAGG
Har-2-1   UCUCUUCGAGAGAACCGGUCCGCGCCCGCAUCGCCGGGAAGCCCGAGGAGGGGUUCCAGGUCGUGGCCGCGGACCUGUCGGAAGCGCUCGCCGGCGACGGAGUGGCCGUCGCGACGUCGCGACACGGAGAAUCGCUCUCCGUCUCGCGGCUCGGCGACCUCGUCUCGGACGCGCGGAAGG
env-157   UCUCUUCGAGAGAACCGGUCCGCGCCCGCAUCACCGGGAAGCCGGAGGUCGGCUUCCAGGUCGUGGCCGCGGACCUGUCGGAAGCGCUCGCCGGCGACGGACUGGCCGUCGCGACGUCGCGACACGGAGCGGAACUCUCCGUCUCGCGGCUCGGCGACCU...................
env-158   UCUCUUCGAGAGAACCGGUCCGUGCCCGCAUCACCGGGAAGCCGGAGGUCGGUUUCCAGGUCGUGGGCGCGGAUCUGUCGGAAGCGCUUGCCGGCGACGGAGUGGCCGUCGCGACGUCGCGACACGGAGAAGCGCUCUCCGUGUCGCGUCUCGGCGACCUCGUUUCGGACGCGCGAGAGG
env-159   UCUCUUCGAGAGAACCGGUCCGUGCCCGCAUCACCGGGAAGCCGGAGGUCGGUUUCCAGGUCGUGGGCGCGGAUCUGUCGGAAGCGCUUGCCGGCGACGGAGUGGCCGUCGCGACGUCGCGACACGGAGAAGCGCUCUCCGUGUCGCGUCUCGGCGACCUCGUUUCGGACGCGCGAGAGG
env-160   UCUCUUCGAGAGAACCGGUCCGCGCCCGCAUCACCGGAAAACCGGCGAACGGUUUUCAGGUCGUGGGAGCGGACCUUUCGGAAGCGCUCGGCGGCGACGGACUCGCCGUCGCGACGUCGCGGUACGGGGAGGAGCUCUCCGUCUCGCGACUGGGCGAUAUCGUCUCGGACGCGCGCGAAG
h3A-1-1   UCUCUUCGAGAGAACCGGUCCGCGCCCGCAUCACCGGGAAGCCGGAGGACGGUUUCCAGGUCGUUGGCGCGGACCUUCCGGAGGUGCUCGCCGACGGCGGGCUCGCCAUCGCGACGUCACGACACGGGGAGCCGCUCUCCGUGUCGCGGCUCGCCGAACUCGCCCCGCGGGUGCAGGAGG
Hai-1-1   UCUCUUCGAGAGAACCGGUCCGCGCCCGCAUCACCGGGAAGCCCGAGGAGGGUUUCCAGGUCGUGGGUGCGGACCUGUCGGAAGCGCUCGCCGGCGACGGCUUGGCCGUCGCGACGUCGCGGCACGGAGAAGAGCUCUCCGUCUCGCGGCUCGGCGACCUCGUCUCGGACGCACGGAAGG
env-161   UCUCUUCGAGAGAACCGGUCCGCGCCCGCAUCACCGGGAAGCCCGAGGAGGGUUUCCAGGUCGUGGAAGCGGACCUGUCGGAAGCGCUCGCCGGCGACGGCUUGGCCGUCGCGACGUCGCGACACGGAGAGGAACUCUCCGUCUCGCGGCUCGGCGACAUCGUCUCGGACGCGCGGAAGG
Hho-1-1   UCUCUUCGAGAGAACCGGUCCGCGCCCGCAUCGCCGGGAAGCCCGAGGCAGGCUUCCAGGUCGUGGCCGCGGACCUGUCGGAAGCGCUCGCCGGCGACGGACUGGCCGUCGCGACGUCGCGACACGGGGAGGAACUCUCCGUCUCGCGGCUCGGCGACCUCGUCUCGGACGCCCGCGAGG
env-164   UCUCUUCGAGAGAACCGGUCCGCGCCCGCAUCACCGGGAAGCCGUCGGAGGGCUUCCAGGUCGUGACCGCGGACCUGUCGGCAGCGCUCGACGACGACGGACUCGCCGUCGCCACGUCACGGUUCGGAGAGGAGCUCUCCGUGCCACGACUCGGCGGGAUCGUCACGGACGCACGGGGGG
env-165   UCUCUUCGAGAGAACCGGUCCGCGCCCGGAUCACCGGGAAGCCGUCUGCUGGCUUCCAAGUGGUGGGUGCGGACCUGUCGGAAGCGCUCGCCGAUGGCGGGCUCGCCGUCGCAACGUCGCGACACGGAGAAUCGCUCUCCGUCUCGCGGCUCGGCGAACU..................
env-166   UCUCUUCGAGAGAACCGGUCCGCGCCCGGAUCACCGGGAAGCCGUCUGCUGGCUUCCAAGUGGUGGGUGCGGACCUGUCGGAAGCGCUCGCCGAUGGCGGGCUCGCCGUCGCAACGUCGCGACACGGAGAAUCGCUCUCC......................................
env-167   UCUCUUCGAGAGAACCGGUCCGCGCCCGGAUCACCGGGAAGCCAUCCGCUGGCUUCCAGGUGGUGGGUGCGGACCUGUCGGAAGCGCUCGCCGAUGGCGGGCUCGCCGUCGCAACGUCGCGACACGGAGAAUCGCUCUCCGUCUCGCGGCUCGGCGAACUCGUCGCCGACGCGGCAAAGG
env-169   UCUCUUCGAGAGAACCGGUCCGCGCCCGGAUCACCGGGAAGCCAUCCGCUGGCUUCCAGGUGGUGGGUGCGGACCUGUCGGAAGCGCUCGCCGAUGGCGGGCUCGCCGUCGCAACGUCGCGACACGGAGAAUCGCUCUCCGUCUCGCGGCUCGGCGAACUCGUCGCCGACGCGGCAAAGG
env-170   UCUCUUCGAGAGAACCGGUCCGCGCCCGGAUCACCGGGAAGCCAUCCGCUGGCUUCCAGGUGGUGGGUGCGGACCUGUCGGAAGCGCUCGCCGAUGGCGGGCUCGCCGUCGCAACGUCGCGACACGGAGAAUCGCUCUCC......................................
env-172   UCUCUUCGAGAGAACCGGUCCGCGCCCGGAUCACCGGGAAGCCAUCCGCUGGCUUCCAGGUGGUGGGUGCGGACCUGUCGGAAGCGCUCGCCGAUGGCGGGCUCGCCGUCGCAACGUCGCGACACGGAGAAUCGCUCUCCGUCUCGCGGCUCGGCGAGCU..................
env-173   UCUCUUUGCGACGACCCGUCCGCGCGAAACUUGCUGACGAGCCCGUUUCGGGCUUUCAAGUUUAUCGUGCGGACCUCCCGGAAGCUCUCAGCUGGUCCGAUACGGGACUCUGCAUUGCAGCGUCUCGGUAUGGCCAGGAUUUGACCGUUGAUACUCUCGACGAGUUAACGUCGGAGAUCA
Hru-1-1   UCUCUUCGCGAGAACCGGUCCGUGCGCGGAUCGUCGACGAGCCCGUUCCGGGCUUCGACGUGUCCCGUAUGGACCUCGAGGAAGCACUCGGCCACGCCGACGCGGGCGUCGCGAUUGCGACGUCCCGCUUCGGCGAGGCGCUGACCACGAGUCGGCUGCCGCAGCUCGCCUCGCGCGUCG
env-174   UCUCUUCGCGAGAACCGGUCCGUGCGCGGAUCG.......................................................................................................................................................
Hru-2-1   UCUCUUCGCGAGAACCCGUCCGCGCACGGCUGGUCGACGAGGACCCACCCGGGUUUACCGUCGACGCCUGCGGGGACAUCCAGGAAGCCCUGGCCCGCGACGACGCGGGCCUCUGCAUCGGCACGUCGCGCCACGGGGACGAACUCACCCGUUCGCGGCUGACCGAACUCGCCCCGCGCU
env-175   UCUCUUCGCGAGAACCGGUUCGCGCGAAAAUCGUGGCCGAACCCCCACCGGGGUUCGCCGUUGAGCGCGCGGCCAUCGGCAACUGCCUUGCCCGGCCGGACGCUGGCAUUCGAAUUGCCGCGUCCAGGCACGGGGA..........................................
env-176   UCUCUUCGCGAGAGCCGGUUCGCGCGAAACUCGUGGCGGACGCCCCACCGGGCUUCGCCGUCGAGCGCGCGACCGUCGACGAGUGCCUCUCCCGGUCGGACGCCGGCGUCCGUAUCGCCGCGUCCAGACACGGGGAGGUGCUGACGGUCGCCCGACUCGGGGAACUCUCCGAGCGGACGA
env-178   UCUCUUCGCGAGAGCCGGUUCGCGCGAAACUCGUGGCGGACGCCCCACCGGGCUUCGCCGUCGAGCGCGCGACCGUCGACGAGUGCCUCUCCCGGUCGGACGCCGGCGUCCGUAUCGCCGCGUCCAGACACGGGGAGGUGCUGACGGUCGCUCGGCUCGGAGAACUCUCCGAGCGGACGA
env-179   UCUCUUCGCGAGAGCCGGUUCGCGCGAAACUCGUGGCCGAGCCCCCACCGGGGUUCGCCGUCGAGCGCGCGGCCAUCGACGACUGCCUCGCCCGUCCGGACGCCGGCGUCCGAAUCGCCGCGUCCAGACACGGGGAGGUACUGACGGUCGAUCGGCUCGGGGAACUCUCCGGGCGGACGA
env-180   UCUCUUCGCGAGAGCCGGUC....................................................................................................................................................................
Nmo-1-1   UCUCUUCGCGAGAGCCGGUUCGCGCGAAACUCGUGGCGGACCCCUCACCGGGGUUCGCCGUCGAGCGUGCGACCAUCGACGACUGCCUCGCCCGUCCGGACGCCGGCGUCCGAAUCGCCGCGUCCAGACACGGGGAGGUACUGACGGUCGAUCGGCUCGGGGAACUCUCCGGGCGGACGG
Hha-1-1   UAUCUUCGCGAGAACCGGUCCGCGCACGGAUCGUCGACGAACCCGCACCCGGGUUCGCCGUCGACCGCACGGACCUCCCGGCGGCGCUCGGCCGUGAGGAUGCCGGGCUCCGGGUCGCCACGUCGCGCCACGGCGAGGCGCUGACGGCGGACGGACUCGGAGCACUGGCCGGCGGGCCCG
env-181   UCUCUUCGCGACGACCGGUCCGGGCGAAACUCGUCGACGACCCCUCCCGGGGCUCGCCGUCGAGCGGACGGACCUCUCCGCAGCACUCGGCCGUGAGGACGCCGGCGUCACCAUUGCUGCCUCUCGAUUCGGUGAGAUUCUCACCGUGGGGCGGCUGGACACGC..............
Hpa-1-1   UCUCUUCGAGAAGACCGGUCCGUGCGAAGCUCGUGGACGCCCCGUUCCGGGGCUCACAGUGACGCGCAUGGACCUCUCGGCGGCCCUCGACCGCGACGAUGCUGGCGUCAGAAUCGCCACAUCGCGUUACGGAGAACCGCUCUCCGUCGGACGGCUUGAAGAAGUCGUCACGCGAACGA
Hci-1-1   UCUCUUCGAGAAGACCGGUUCGUGCGAAGCUCGUGGACGCCCCGCAGUCGGGCUACACAGUGAUGCGCAUGAACCUCUCGGCGGCCCUCGACCGCGACGAUGCUGGCGUUCGAAUCGCCACGUCUCGCUUCGGAGAAUCGCUCUCCGUGGCGCGACUCGACGAAAUCGUUGCGCAAACCG
env-182   UCUCUUCGCG..............................................................................................................................................................................
env-183   UAUCUUCGAGAGAACCGGUCCGCGCGCGAAUCACGGACGAAGCCCACCGGGUUUCGCCGUCGAAC.......................................................................................................................
env-184   UCUCUUCGCGAGAUCCGGUCCGGGCCCGACUCACCGAGGAGCCCCGCCGGGCUACUCGGUCGGGCCGUCGGACCUUUCG.........................................................................................................
env-185   UCUCUUCGCGACGACCGGUCCGGGCGAAGCUCGUAGACGAGCCCCUCCCGGGGCUCGCCGUCGAGUCGACGGACCUAUCGGCGGCUCUC...............................................................................................
Hdj-1-1   UCUCUUCGCGACGACCGGUCCGGGCGAAGCUCGUCGGCCGGCCCCUCCCGGGGCUGUCGGUCGAGCGGACGGACCUUUCGGCAGCACUCGGCCGUGAGGACGCCGGCGUCUGCAUCGCCGCCUCCCGCUACGGUGAACGACUCACCGUCGGGCGGCUCGAGACGCUGGCCGGGACGAUCG
Hsa-10-1  UCUCUUCGCGACGACCGGUCCGGGCGAAGCUCGUCGAUCGGCCCCUUCCGGGGCUAUCGAUUGAGCAGACGGACCUUUCGGCAGCACUCGGCCGUGAGGACGCCGGCGUCCGUAUCGCCGCCUCCCGUUACGGUGAAGAACUCACCGUGGGGCGGCUCGAGACGCUGGCCGGGCGCGUCG
Nsu-1-1   UCUCUUCGCGACGACCGGUCCGGGCGAAACUCGAGAACAAGCCCCUCCCGGGGCUUGCAGUCGAGCGGACGGACCUUUCCGCAGCUCUCGGCCGUGAGGACGCUGGCGUCUGCAUCGCUGCCUCCCGAUUCGGUGAAGAACUCACCGUCGGGCGGCUCGAGACGCUGGCCGGACGCAUCG
Nba-1-1   UCUCUUCGCGACGACCGGUCCGAGCGAAACUCGAGGACAAACCCCUCCCGGGGCUGUCAGUCGAGCGGACGGACCUUUCCGCAGCACUCGGCCGUGAGGACGCUGGCGUCUGCAUCGCCGCUUCCCGAUUCGGUGAAGAGCUCACCGUCGGGCGGCUUGGGACGCUGGCCGGACGCAUCG
Nin-1-1   UCUCUUCGCGACGACCGGUCCGGGCGAAACUCGUCGACGAACCCCUUCCGGGGCUCGCAGUCGAGCGGACGGACCUUUCGACAGCUCUCGGCCGUGAGGACGCCGGCGUCUGUAUUGCGUCGUCUCGCUACGGUAUCGAACUUACCGUCGGACGACUCGAGACGCUGGCCGGACGCGUCG
env-186   UCUCUUCGCGAGAGCCGGUCCGUGCGAGGCUGGUCGACGAGCCCGUUUCGGGGCUCUCGAUCCAUCGCAUGGACCUAUCGGAAGCGCUCGGCCGUGAGGACGCCGGUGUUCGGAUCGCGACGUCGAGGUUCGGCGAGAACCUUAGCGUCUCGCGACUCGGAACGCUGGCCGGACGGAUCG
env-187   UCUCUUCGCGAGAGCCGGU.....................................................................................................................................................................
env-188   UCUCUUCGCGAGAGCCGGUCCGUGCGAAGCUGGUCGACGAGCCCAUUUCGGGGCUCUCGAUCCAUCGCAUGGACCUAUCGGAAGCGCUCGGCCGUGAGGACGCCGGUGUCCGAAUCGCGACGUCGAGGUUCGGCGAGAACCUUAGCGUCUCGCGACUCGGGACACUGGCCGGACGAAUCG
env-189   UCUCUUCGCGAGAGCCGGUCCGUGCGAAGCUGGUCGACGAGCCCAUUUCGGGGCUCUCGAUCCAUCGCAUGGACCUAUCGGAAGCGCUCGGCCGUGAGGACGCCGGUGUCCGAAUCGCGACGUCGAGGUUCGGCGAGAACCUUAGCGUCUCGCGACUCGGGACACUGGCCGG........
env-190   UCUCUUCGCGACGACCGGUCCGGGCGAAACUCGUCGACGAAUCCCCGCCGGGG...................................................................................................................................
env-191   UCUCUUCGCGACGACCGGUCCGGGCGAAACUCGUCGACGAAUCCCCACCGGGAUUCGCCGUCGAGCGGACGGACCUCCCGGCAGCACUCGGCCGUGAGGACGCCGGCGUUCGUAUCGCCGCCUCCCGAUUCGGUGAACCGCUCACCAUCGGACGGCUCGAGACGCUGGCCGGGGACAUCG
Hla-1-1   UCUCUUCGCGACGACCGGUCCGGGCGAAGCUCGUCGAUCGCCCCCUUCCGGGGCUUUCAGUCGAGCGGACGGACCUAUCGGCAGCACUCGGCCGUGAGGACGCCGGCGUCCGCAUCGCCGCCUCCCGACACGGUGAGGAACUCACCGUGGGGCGGCUCGAGACGCUGGCCGGACGCAUCG
env-193   UCUCUUCGCGACGACCGGUCCGGGCGAAACUCGUCGACGAAUCCCCACCGGGAUUUACCGUCGAGCGGACGGACCUCUCGGCAGCACUCGGCCGUGAGGACGCUGGCGUUCGUAUCGCCGCUUCUCGCUACGGUGAGCAACUCACCGUGGAACGGCUC......................
Hac-1-1   UAUCUUCGAGACGACCGGUCCGGGCGAAGAUCAUCGAGGAGGCCCCACCGGGCUUUCAG.............................................................................................................................
Hac-1-2   UAUCUUCGAGACGACCGGUCCGGGCGAAGAUCAUCGAGGAGGCCCCACCGGGCUUUCAGGUCUUCACCCGGCCCAUCGGGGACGAACUCGACCGUCCGGACGCGGGGUUCCGGGUCGCCACAUCACGCUUCGGGGUGCCAGUCGGCGCCGCGAACGUGGGCGACCUCACCCGCCGCGUCC
env-196   UCUCUUCGCGACGACCGGU.....................................................................................................................................................................
env-197   UCUCUUCGCGACGACCGGUCCGUGCGAAACUGGUCGACGAACCCCUCCCGGGGUUCUCGGUCGAACGCACGGCCCUCGGUGACGCUCUCGA.............................................................................................
env-198   UCUCUUCGCGACGACCGGUCCGUGCGAAACUGGUCGACGAACCCCUCCCGGGGUUCUCGGUCGAACGCACGACCAUCGGGGACGCCCUCGACCGCCCCGACGCGGGCGUUCGCAUCGCUACCUCCCGCCACGGGACGCCGCUUUCGGCGGCGUCCCUCGGGGGGUACGUCGAACGCAUCG
Hsp-2-1   UCUCUUCGCGACGACCGGUCCGUGCGAAACUGGUCGACGAACCCCUCCCGGGGUUCUCGGUCGAACGCACGACCAUCGGGGACGCCCUCGACCGCCCCGACGCGGGCGUUCGCAUCGCUACCUCCCGCCACGGGACGCCGCUUUCGGCGGCGUCCCUCGGGGGGUACGUCGAACGCAUCG
env-199   UCUCUUCGAGAGACCCGGUCCGGGCGAAGAUCGUCGACCAGUCGAUCGGUUGGUCGGUCGACCGUGCGGACCUGCCGGCGGCACUCGGCCGUGAGGACGCCGGACUCCGGAUCGCCGCCUCACGCCACGGCGACGUGGUGAGAGCGGGUGACAUCGGGAGUCUGGCCGGUCGCGUCGAGG
Hsu-1-1   UCUCUUCGCGACGACCGGUUCGUGCGAAGCUGGUCGACGAGCCCCUUCCGGGGUUCGUCGUCACUCGGACGAACCUCCGGGAUGCCCUCGAUCGCCCCGACGCAGGCUAUCGCGUUGCCACCUCCCGACACGGGACACCGGUUACGGUGUCCGGACUGUCGGGAAUCAAAGAACGCGUCG
env-201   UCUCUUCGCGACGGCCGGUCCGGGCGAAACUCGUCGACGCCCCCACAACGGGAUUCGACGUUGUCGCCGCGGACCUCGAUGCGGCACUCUCGCGCGACGAUGCCGGGCUCACUAUCGCGUCCUCGCGAUACGGUGAGCCGGUAACGUCGACCCGUCUC......................
env-203   UCUCUUCGCGACGGCCGGUCCGGGCGAAACUCGUCGACGCCCCCACAACGGGAUUCGACGUUGUCGCCGCGGACCUCGAUGCGGCACUCUCGCGCGACGAUGCCGGGCUCACUAUCGCGUCCUCGCGAUACGGUGAGCCGGUAACGUCGACCCGUCUCGGCCAGCUGGUC..........
Hca-1-1   UCUCUUCGCGACGGCCGGUCCGGGCGAAACUCGUCGACGCCCCCACAACGGGAUUCGACGUUGUCGCCGCGGACCUCGAUGCGGCACUCUCGCGCGACGAUGCCGGGCUCACUAUCGCGUCCUCGCGAUACGGCGAGCCGGUAACGUCGACCCGUCUCGGCCAGUUGGCCGAGCGGCGCG
Ham-1-1   UCUCUUCGCGACGGCCGGUCCGGGCGAAACUCGUCGACGCCCCCACAACGGGAUUCGACGUUGUCGCCGCGGACCUCGAUGCGGCACUCUCGCGCGACGAUGCCGGGCUCACUAUCGCGUCCUCGCGAUACGGCGAGCCGGUAACGUCGACCCGUCUCGGCCAGUUGGCCGAGCGGCGCG
Hhi-2-1   UCUCUUCGCGACGGCCGGUCCGGGCGAAACUCGUCGACGCCCCCACAACGGGAUUCGACGUUGUCGCCGCGGACCUCGAUGCGGCACUCUCGCGCGACGAUGCCGGGCUCACUAUCGCGUCCUCGCGAUACGGCGAGCCGGUAACGUCGACCCGUCUCGGCCAGUUGGCCGAGCGGCGCG

          ............................................................................................................................................................................................................................
          ............................................................................................................................................................................................................................
          UCUYUUYR•GRRRR•YR•••••••Y•••R•••RY•RR•R•••••••••••••••••••••••••••••••••••••••••••••••••••••••••••R•••••••••••••••••••••••••••••••••••••••••••••••••••••••••••••••••••••••••••••••••••••••••••••••••••••••••••••••••••••••••••••
```

```
Hsa-2-1   UCUCUUCGCGACGGCCGGUCCGGGCGAAACUCGUCGACGCCCCCACAACGGGAUUCGACGUUGUCGCCGCGGACCUCGAUGCGGCACUCUCGCGCGACGAUGCCGGGCUCACUAUCGCGUCCUCGCGAUACGGCGAGCCGGUAACGUCGACCCGUCUCGGCCAGUUGGCCGAGCGGCGCG
Har-1-1   UCUCUUCGCGACGGCCGGUCCGGGCGAAACUCGUCGACGCCCCCUACAACGGGAUUCGACGUUGUCGCCGCGGACCUCGAUGCGGCACUCUCGCGCGACGAUGCCGGGCUCACUAUCGCGUCCUCGCGAUACGGCGAGCCGGUAACGUCGACCCGUCUCGGCCAGCUGGCCGAGCGGCGCG
Hva-1-1   UCUCUUCGCGACGGCCGGUCCGGGCGAAACUCGUCGACGCCCCCACAACGGGAUUCGACGUUGUCGCCGCGGACCUCGAUGCGGCACUCUCGCGCGACGAUGCCGGGCUCACUAUCGCGUCCUCGCGAUACGGCGAGCCGGUAACGUCGACCCGUCUCGGCCAGCUGGCCGAGCGGCGCG
Hja-1-1   UCUCUUCGCGACGGCCGGUCCGGGCGAAACUCGUCGACGCCCCCACAACGGGAUUCGACGUUGUCGCCGCGGACCUCGAUGCGGCACUCUCGCGCGACGAUGCCGGGCUCACUAUCGCGUCCUCGCGAUACGGCGAGCCGGUAACGUCGACCCGUCUCGGCCAGCUGGCCGAGCGGCGCG
env-204   UCUCUUCGAGAGAGCCGGUCCGUGCCCGCCUCGUCGACGAGCCCACACCGGGCUUCGACGUGACGGUCACGGACCGUCGCGGCUGCCCUCGACGCCGCCGGACGACGGAUCGCCACCGCGGCACGGACGGCUGCGUCCGCUGCGAAACUCGGCGAGAUCCGAUCCUGAAUUUCAGG
Hse-1-1   UAUCUUCGAGAGAACCGGUCCGCGCGCGCAUCACGGACGAAGCCCCACCGGGGUUCGCCGUCGAGCGCGCGGGCCUCUCGGCAGCGCUCGGCCGUGAGGACGCCGGGCUUCGGGUCGCCACGUCGCGCCACGGUGAGGCGCUCUCGACGGGGCGACUCGGGGCGCUGGCCGGUCGUGCCG
Hth-1-1   UCUCUUCGCGAGAGCCGGUCCGCGCGCGCAUCACGGACGAAUCCCCACCGGGGUUCGCCGUCGAGCGCGCGGACCUCUCGGCAGCGCUCGGCCGUGAGGACGCCGGGUUCCGGGUCGCAACGUCACGCCACGGUGAGGCGCUGACGACGGAGCGACUCGGAACGCUGGCCGGUCGUGCCG
Hmo-1-1   UCUCUUCGCGAGAGCCGGUCCGCGCGCGAAUCACGGACGAAUCCCCACCGGGGUUCGCCGUCGAGCGCGCGGACCUCUCGGCAGCGCUCGGCCGUGAGGACGCCGGGUUCCGGGUCGCAACGUCACGCCACGGUGAGGUGCUGACGACGGAGCGACUCGGAACGCUGGCCGGUCGUGCCG
env-206   UCUCUUCGCGACGUCGGGUCCGGUGCCAAGAUCGUCGACGAACCCCUCCCGGGGUUCGCGGUCGACCGCGCGGACCUUUCGGCAGCGCUCGGCCGUGAGGACGCGGGCGUUCGGAUCGCAGCUCUCGCGACACGGUGCGGAUCUCACCGUCUCGCGGCUCCGAACGCUCGCCGGACGCGUGG
env-207   UCUCUUCGAGAGAACCGAUCCGCGCCAAACUCGUGGACGCCACGGUCGGUGUGCGCGUGGAGCGCGCGGACUGACGGACACCCUCGCGGACGCGGACGCCGCACUCGCCGCCUCGCGGUACGGGACGGAGCUGACCGUCUCGGGGUGGACGAGUUGCCGGCCACGCUGGGCGACCGG
env-209   UCUCUUCGAGAGAGCCGGUCCGUGCGCGGAUCGUCGACGCCCCACAGCCGGGGUACGACGUCGCCCGUAUGGACCUCGCGGAAGCGUUGGCCCGCCCCGACGCCGGGAUUCGCGUGGCGACAUCCCGCUUCGGGCGGCCGUUGUCCGCCGACGGUGUGGCGGACCUCUCCCGACAGGUCG
Nta-1-1   UCUCUUCGCGACGACCGGUCCGGGCAAAGCUCGUCGACGACCCCCUUCCGGGGCUCUCGUCGAGCGGACGGACCUACAGGCAGCACUCAGCCGUGAGGACGCCGGCGUUCGAAUCGCCGCUCCCGAUACGGUACCGAACUUACCGUCGGGCGGCUCGACACGCUGGCCGGACGCGUCG
Nas-1-1   UCUCUUCGCGACGACCGGUCCGGGCAAAGCUCGUCGACGAACCCCUUCCGGGGCUCUCCGUCGAGCGGACGGACCUACAGGCAGCACUCAGCCGUGAGGACGCCGGCGUUCGAAUCGCCGCUCCCGAUACGGUACCGAACUUACCGUCGGGCGGCUCGACACGCUGGCCGGACACGUCG
Noc-1-1   UCUCUUCGCGACGACCGGUCCGGGCGAAGCUCGUCGACGACUCCCUCCCGGGGAUCGAUGUCGAGUGGACGGACCUCGAGGCAGCGCUCGGCCGUGAGGACGCCGGCGUCCGCAUUGCGGCCUCCCGAUUCGGUGCCGAACUCACCGUCGGGCGGCUGGGGACGCUGGCCGGACGCCGCG
Nje-1-1   UCUCUUCGCGACGACCGGUCCGGGCGAAACUCGUCGACGGGCCCCUUCCGGGGCUCUCGAUCGAGCGGACGGACCUCGAGGCAGCGCUCGGCCGUGAGGACGCCGGCGUCCGCAUCGCGGCUCCCGAUUCGGUGCCGAACUCACCGUCGGGCGGCUGGGGACGCUGGCCGGACGCCGCG
Nam-1-1   UCUCUUCGCGACGACCGGUCCGGGCGAAACUCGAGGACGAGCCCCUUCCGGGGCUCGACGUCGAGCGGACGGACCUCCAGGCAGCACUCGGCCGUGAGGACGCCGGCGUCCGCAUCGCAGCUCCCGAUUCGGUGCAGAACUCACCGUCGGGCGGCUGGGGACGCUGGCCGGACGCCGCG
env-210   UCUCUUCGCGACGACCGGUCCGGGCGAAGCUCGUCGAUCGCCCCUCCCUUUCGGGGCUGUCGGUCGAGCGGACGGACCUGCAGGCAGCUCUCGGCCGUGAGGACGCCGGCGUUCGCAUCGCCGCUCCCGAUUCGGUGAAGAACUCACCGUCGGGCGGCUCGCAACGCUGGCCGGACGCAUCA
Hsp-3-1   UCUCUUCGCGACGACCGGUCCGGUGCGAAACUGGUCGACGACCCCCUCCCGGGGUUUUCGGUCGAACGCACGGGUCUCGGUGACGCUUUAGACCGUUCCGACGCCGGCGUUCGCAUUGCGACCUCCCGGCACGGGGAGCCGCUUUCGGUGGCUUCCCUCGGUGGGUACCGCGAACGCAUCG
Nch-1-1   UCUCUUCGCGACGACCGGUCCGGGCGAAACUCGUCGACGAGCCCCUCCCGGGGCUUACCGUCGAGCGGACGGACCUUCAGGCGGCUCUCAGCCGUGAGGACGCCGGCGUUCGAAUUGCGGCUUCCCGAUUCGGUGCAGAACUCACCGUCGGGCGGCUCGGAACGCUGGCCGGACACACAG
Nae-1-1   UCUCUUCGCGACGACCGGUCCGGGCAAAGCUCGUCGACGACCCCCUUCCGGGGCUCUCCGUCGAGCGGACGGACCUACAGGCAGCACUCAGCCGUGAGGACGCCGGCGUUCGAAUCGCCGCUCCCGAUACGGUACCGAACUUACCGUCGGGCGGCUCGGAACGCUGGCCGGACGCGUCG
env-214   UCUCUUCGCGACGACCGGUCC..........................................................................................................................................
env-215   UCUCUUCGCGACGACCGGUCCGGGCGAAACUCGAGGACGAGCCCCUUCCGGGGAUCGAAGUCGAGCGGACGGACCUCCAGGCAGCGCUCGGCCGUGAGGACGCCGGCGUCCGCAUCGCAGCUCCCGAUUCGGUGCAGAACUCACCGUCGGGCGGCUGGGGACGCUGGCCGGACGCCGCG
env-216   UCUCUUCGAGACGACCGGUUCGUGCGCGACUCGUCGACGAACCCCUUCCGGGGCUCGACGUCUCGCAAUCGACCAUACAGGCAGCACUCGGCCGUGAGGACGCAGGCGUGACCAUCGCCGCUCCUCGCGACACGGGGACGAACUCUCCGUGUCCAGGCUGGACACGCUCGCCGGGCGCAUCC
Hni-1-1   UCUCUUCGCGACGACCGGUCCGGGCGAAGCUCGUCGAUCGCCCCUCCCUCCCGGGGCUUUCAGUCGAGCGGACGGACCUAUCGGCAGCACUCGGCCGUGAGGACGCUGGCGUUUGCAUCGCCGCUCCCGUUACGGUGAGCGCUCACCGUCGGGCGGCUCGAGACGCUGGCCGGACGCGUCG
Has-1-1   UCUCUUCGCGACGACCGGUCCGGGCGAAGCUCGUCGACGAGCCCCUUGCGGGGCUGUCGAUCGAGCGGACGGACCUCUCCGCAGCACUCGGCCGUGAGGACGCCGGUGUUCGCAUCGCGUCCUCCCGGUACGGUGAGGAACUCACCGCCGGACGUCUCGAGACACUGGCCGGACGCGUCG
env-217   UCUCUUCGCGACGACCGGUCCGGGCGAAGCUCGUCGAUGAACCC...................................................................................................................
Nal-2-1   UCUCUUCGCGACGACCGGUCCGGGCGAAGCUCGUCGACGAGCCCCUCCCGGGGCUUUCGAUCGAGCAGACGGACCUGCAGGCAGCACUCGGCCGUGAGGAUGCCGGCGUCCGUAUCGCAGCUCCCGAUUCGGUGAUGAACUCACCGUCGGGCGGCUCGAGACGCUGGCCGGACGCGUCC
Npa-1-1   UCUCUUCGCGACGACCGGUCCGAGCGAAGCUCGUCGACGAGCCCCUCCCGGGGCUUUCGAUCGAGCAAACGGACCUGCAGGCAGCACUCGGCCGUGAGGAUGCCGGCGUCCGCAUUGCAGCUCCCGAUUCGGUGAUGAACUCACCGUCGGACGGCUCGAGACGCUGGCCGGACGCGUCC
Nga-1-1   UCUCUUCGCGACGACCGGUCCGGGCGAAGCUCGUCGACGAGCCCCUCCCGGGGCUUUCGAUCGAGCAGACGGACCUGCAGGCAGCACUCGGCCGUGAGGAUGCUGGCGUCCGCAUCGCAGCUCCCGAUUCGGUGAUGCACUCACCGUCGGGCGACUCGAGACGCUGGCCGGACGCGUCC
Nsp-2-1   UCUCUUCGCGACGACCGGUCCGGGCGAAGCUCGUCGACGAGCCCCUCCCGGGGCUUUCGAUCGAGCAGACGGACCUGCAGGCAGCACUCGGCCGUGAGGAUGCCGGCGUCCGCAUCGCAGCUCCCGAUUCGGUGAUGCACUCACCGUCGGGCGACUCGAGACGCUGGCCGGACGCGUCC
Htu-1-1   UCUCUUCGCGACGACCGGUCCGGGCGAAACUCGUCGACGAGCCCCUCCCGGGGCUGUCGGUCGAGCGGACGGACCUGCAGACAGCACUCGGCCGUGAGGACGCCGGCGUCCGCAUCGCGUCGUCCCGAUUCGGUGAAGAACUCACCGUCGGGCGACUCGAGACGCUGGCCGGACGCAUCG
Hsa-8-1   UCUCUUCGCGACGACCGGUCCGGGCGAAGCUCGUCGACGAGCCCCUUCCGGGGCUUUCGGUCGAGCGGACGGACCUGCAGACAGCACUCGGCCGUGAGGACGCCGGCGUCCGCAUCGCGGCUCCCGAUUCGGUGAAGAGCUCACCGUCGGGCGGCUCGAGACGCUGGCCGGACGUAUCG
Hth-2-1   UCUCUUCGCGACGACCGGUCCGGGCGAAACUCGAAGACGUUCCCCUUCCGGGGCUCGCCGUCGAGCGGACGGACCUUUCGACAGCACUCGGCCGUGAGGACGCCGGCGUCCGUAUCGCCGCUCCCGAUUCGGUGAAGAACUCACCGUCGGGCGGCUCGGGACGCUGGCCGGACGUAUCG
Nve-1-1   UCUCUUCGCGACGACCGGUCCGGGCGAAGCUCGUCGAUGAACCCCUUCCGGGGCUUUCGAUCGAGCAGACGGACCUCGGCAGCACUCGGCCGUGAGGACGCCGGCGUUCGCAUCGCGGCUCCCGAUUCGGUGAACAUCUCACCGUCGGGCGGCUCGAGACGCUGGCCGGACGCACCA
Hli-3-1   UCUCUUCGCGACGACCGGUCCGGGCGAAGCUCGUCGACGAGCCCCUUCCGGGGCUCUCAGUCGAGCAGACGGACCUUUCGGCAGCACUCGGCCGUGAGGACGCCGGCGUCUGUAUCGCAGCUCCCGAUUCGGUGAAGAACUCACCGUGGGACGGCUCGAGACGCUGGCCGGACGCGUCG
Hxa-1-1   UCUCUUCGCGACGACCGGUCCGGGCGAAGCUCGUCGACGAACCCCUUCCGGGGCUUUCCGUCGAGCAAACGGACCUAUCGGCAGCACUCAGCCGUGAGGACGCUGGCGUCCGCAUCGCCGCUCUCGACACGGUGAACGGCUCACCGUCGGGCGGCUCGAGACGCUGGCCGGACGCAUCG
Hgo-1-1   UCUCUUCGCGACGACCGGUCCGGGCGAAGCUCGUCGACCGGCCCCUUCCGGGGCUGUCGGUCGAGCGGACGGACCUUUCGGCAGCACUCGGCCGUGAGGACGCCGGCGUCCGCAUCGCCGCUCCCGCUACGGUGAACGGCUCACCGUCGGGCGGCUCGAGACGCUGGCCGGACGCGUCG
env-218   UCUCUUCGCGACGACCGGUCCGGGCGAAGCUCGUCGACGAGCCCCUUC...............................................................................................................
env-219   UCUCUUCGCGACGACCGGUCCGGGCGAAACUCGAGGUGGGCCCCUUCCGGGGCUUACUGUCGAGCGGACGGACCUGCAGGCAGCACUCGGCCGUGAGGACGCUGGCGUCUGUAUUGCCGCUCCCGUCACGGUGAAGAACUCACCGUCGGGCGGCUUGGGACGCUUGCCGGGCAACUCA
env-220   UCUCUUCGCGAGAUCCGGUUCGUGCCCGACUCACCGAGGAACCCCUACCAGGCUUCGGGUCGGGUCGUCGACCUUUCGGCCGCCCUCGACUCCGAGGACGGGUUGGCCAUCGCCGCCUCCCGGUACGGUGACCCCCUCACGGNGGUCGCCUGCAGGGACUCCUCGGGCGCACGACAG
env-221   UCUCUUCGCGACGACCGGUCCGGGCGAAGCUCGUCGAUGACCCCCUCCCGGGGCUCUCGAUCGAGCGGACGGACCUGCAGGCAGCUCUCGGCCGUGAGGACGCCGGCGUUCGCAUCGCAGCUCCCGAUUCGGUGACGAACUCACCGUCGGGCGGCUCGAGACGCUGGCCGGACGUGUCG
Hsa-6-1   UCUCUUCGCGAGAACCGGUCCGUGCGCGGAUCGUCGACGAAGCCCCGUCGGGGUACGUCGUCGAACGCAUGGACCUCGCCGAAGCGCUCACCCGGGACGACGCCGGCGUACGCAUCGCGACGUCGCGCUACGGAGAGAAGCUCUCCGUCUCGCGUCUGUCGUCGCUCGUCGGGCGUCUCG
env-222   UCUCUUCGCGACGGCCGGUUCGCGCGAAACUCGUCGACGAACCCCCACCGGGGUUCGCCGUCGAGCGUGCGACCAUCGAGGACGUACUCACCCGUCCGGACGCCGGCGUGCGUAUCGCCGCGUCCAGACACGGGGAGGAGUUGAC....................................
env-223   UCUCUUCGCGAGAGCCGGUCCGGGCGAAACUCGUCGACGAGCCCCUCACCGGGGCUUUGUCGUCGACCGCGCGGAUCUGGAUGCGCGCUGGCGCGGGCGGACGGCCGUCGACGUCGCGACGUCGCGAC.................................................
env-224   UCUCUUCGCGGGAGCCAGUCCGGCGAAACUCGUCGACCAGUCCCCA.................................................................................................................
env-225   UAUCUUCCGAAAAACCAGUCCGGGCUCGACUACUGACCGAUUCUAUACCAGGAAUCUUAGUCGAGAACACCGAUCUAGAAUCUGCAUUAGAACGUAAUGAUGCGGGAGUUAGAAUCGCUGCAUCCCGGUAUGGGAAUCCACUCGAAAAUAAGAACCUCGAUGAUCUUAUUACUCGAGUAA
env-226   UAUC...........................................................................................................................................................
env-227   UCUCCUCCGAAAAACCG..............................................................................................................................................
env-228   UCUCUUCGCGAGAACCGGUUCGGGCACGGAUUGUCGAUACUCCCCUUUCGGGAUUUGAUGUUUCUGCCUGGACCUUGUGGAUGCACUGACGCCGCUGCAGGUAUCACCAUUGCGACAUCCAGACAUGGUGAAGCCGUAACCACAGGGCGAUUGCCAGAACUCACCGCGCGUAUUG
env-231   UCUCUUUGCGAGACAGGUCACAGUGAAGGCAGCACGAAGUGUGGAAAUACGGGGUGCUCCUUUCAGAAAACUGCGCCUGAAGAAGGAUUAAUCCCUCUUCAUGCUGCAAGGGAGGGCCAGCAUGAACCCGGGGGAACCCGGGAAUUGUGCCAUGCCCUUACACGCAAAAAACCCGAGGAG
env-232   UCUCUUUGCGAGACAGGUCACAGUGAAGGCAGCACGAAGUG......................................................................................................................
env-233   UCUCUUUGCGAGACAGGUCACAGUGAAGGCAGCACGAAGUGUGGAAAUACGGGGUGCUCCUUUCAGAAAACUGCGCCUGAAGAAGGAUUAAUCCCUCUUCAUGCUGCAAGGGAGGGCCAGCAUGAACCCGGGGGAACCCGGGAAUUGUGCCAUGCCCUUACACGCAAAAAACCCGAGGAG
env-237   UCUAUUCGCGAGACCGGUAGUCUGGUAAUACCAGCUUGGGUCCAUUGGAAGGACCAGUAAAAGGACAAAAUCCCAGGUGGUUUCCCGGAGAACGGGAGUCUGUCAGAAGGGACUACAUUGUCUGUAAUACGAACUAUCGCGGGUUAUCUCAGUACUGAGCGUGGAGCAUCACAUCAGUCUC
env-238   UAAAAGAAGAAGGAAAUCUGGAAGGAGAGAUAAUUAGGGAGGUUCCAGUUUAUCGAGGUUACCGUGUGAGAAUUGUUGAGGGUCUUGAUUCUGCUUUGAAGUUCUCUCAACCACCUUUCAUAGGAACUUCGAGGUAUGGGGAAGUGGUAACAUUGGAAAAGCUUAAAAAGUGGAAUGUGG
env-240   UCUGUUCGCGAAGUAAAAACGCAGAGCUCGUGAAUAAGAAGGACAUCCCCCUGUAUUGGGGCUAUGAAGUAAAGACUGCAAAGUCGCUUCACGAAGCGCUGGCGGAAAUCGAUGGCUUAAGGAUAGCCACUUCGGUCAAGGGGCAACCCCUGGACGUAACGUUGCUGUCCGAGAUAGGCG
env-241   UUUUUUCGCGACGACCGAUACAAGUCGAACUUGAUAGAAACGGACAAUAUCCCUAUAUACUGGGGAUACCGGACAGCAAUUGAAGACAGCCUUGCAGGGGCACGUGAUAAAAUAUCCGGACAGGAUGCCCUGUAUUGGCCACCUCACGGGAAGGCAGGGUACUUGAUACCCGGUUCCUUG
env-242   UCUGUUUGCGAGACUGGUAUUCCGGUGCCGUGCCGGACCUCUGAUAGCAGGGAUCCGGCACGCAGGUAUACGGAAAAACAAAGGAUGGCUUCAGCAUCACUGGAGACCCUGCCAUGUUUUUCCUGGUUCCGGUACCGAUAUCGGCAUCACCCCUAAUCGGACACAAUCCGCCGAAUGCCG
env-243   UCUGUUUGCGAGACUGGUAUUCCGGUGCCGUGCCGGACCUCUGAUAGCAGGGAUCCGGCACGCAGGUAUACGGAAAAACAAAGGAUGGCUUCAGCAUCACUGGAGACCCUGCCAUGUUUUUCCUGGUUCCGGUACCGAUAUCGGCAUCACCCCUAAUCGGAC.................
env-244   UCUGUUGACGAAGACCGGUUGGAUCUGAUGGUCGGCGGAGAAAAGAGAUUCGCAGGACCAGACAACGGGAUCAUUCACAAAUUCGGGUUCGCACCGCUGGUGGAAUGCCGGCGGACCAGCUCAAGGAUUCAGAGCAUCACGCUUUCUCGGAGCACACCGGACCAUUCCCGGCUUCCCCCG
env-245   UCUCUUUGCGACGACAGGUAGGAACGAGGGGGGGUGAAGGCAGGAUCCCGGAUCCGGCUGGUUCACCCGUACUCCUCUCUCACCGUCAUAAAUUCGCACCGCAGGCAGGAGCUUUCAUGGCUCCGGUUUCCGGAAAGGUCGCGUACCCGGAGCGUCACGCUCUCUCGGAGCAGGUACAUG
env-246   UCUCUUUGCGACGACAGGUAGGAACGAGGGGGUGUGAAGGCAGGAUCCCGGAUCCGGCUGGUUCACCCGUACUCCUCUCUCACCGUCAUAAAUUCGCACCGAAGGCAGGAGCUUUCAUGGCUCCGGUUUCCGGAAAGGUCGCGUACCCGGAGCGUCACGCUCUCUCGGAGCAGGUACAUG
env-247   UCUCUUUGCGACGACAGGUAGGAACGAGGGGGGGUGAAGGCAGGAUCCCGGAUCCGGCUGGUUCACCCGUACUCCUCUCUCACCGUCAUAAAUUCGCACCGCAGGCAGGAGCUUUCAUGGCUCCGGUUUCCGGAAAGGUCGCGUACCCGGAGCGUCACGCUCUCUCGGAGCAGGUACAUGC
env-248   UCUCUUUGCGACGACAGGUAGGAACGAGGGGGGGUGAAGGCAGGAUCCCGGAUCCGGCUGGUUCACCCGUACUCCUCUCUCACCGUCAUAAAUUCGCACCGCAGGCAGGAGCUUUCAUGGCUCCGGUUUCCGGAAAGGUCGCGUACCCGGAGCGUCACGCUCUCUCGGAGCAGGUACAU
env-249   UCUCUUCGCGAGACUUUGGUUGACCGGAUUUAUCCGGGCAGGUUCAACGGGAUGAACCAUAUCAGUGCAGAACAUCCGGGCGCCGUGUGCAUAUCAUGCCAGUCGUAUGGUAUGGGCACGAAGAAGGAUAGCUGCCACGUUCGAUCGCGGGUCGCUUUAAAAAGAUUCGAACAUCACUCA
env-250   UCUCUUCGCGAGACUUUGGUUGACCGGAUUUAUCCGGGCAGGUUCAACGGGAUGAACCGUAUCAGUGCAGAACAUCCGGCGUCGUGAGCAUAUCAUGCCAGACGGUAUGGUAUUAGCAGGAAGAAGGAUAUCUGCACGUUCGAUCGCGGGUCGCUUUAAAAAGAUAGGAACAUCACUCAAU
env-251   ACUCUUACACGAGACUGGUACGCCCGAUUCGGGUGCAGGAGAGAUCGGAAGAAGUGGAGUUAUUUCAUCUCCGAAUCGGAGAUGUGCAUCUUCAGCAGAUACCGGUAUCAACCGCAUACUACCUCAUACAUUGGUGUUCCGACCACAGUAUCCCGUAUAAUCUUCCAAAAGUUGAUUAGA
env-252   ACUCUUAUAUGAGACUGGUAAGCCCGAUUUGGGUGCAGGAGAGAUCGGAAGACGAGGGGGUAUGCCAUCUCUGAUCGGAGAUGUGCAUCUUCAGCAGAUACCGGUAUCUUUUGCAGACUGCCUCAGAGAAUUGGUGUUCCGACCACAGUAUCCCGUAUAAUCUUCCGUAUAGGUCGAUUA
env-253   ACUCUUAUACGAGACUGGUAAGCCCGAUUUGGGUGCAUGAGCGAUCGGAAGAAGUGGAGGUAUGCCUCUCUCAUCAUCGGAGGUGUGCAUCUUCAGCAGACACCGGUAUCGUUUGCAUACUGCCUCAAAGAAUUGGUGUUCCGACCACAGUAUCCCGUGUAAUCUCCCUUUAUGGUCGA
env-254   UCUAUUCGCGAGACGGGAUAUUCUGGUGCACGCCAGAGCAAUCUCCGGCAAGGGAUAUCGAACGGAAUACCACCCUAUUCUCGCCCAACUGCACGCU.............................................................
env-255   UCUUCUUUCGCGAGACAGGAAUGCCGGUGCACACUGGCCAGCGUUGGGAUCGAAUCUCAUACGCAAAUCCAAUAACGGGUGAAUUUGUCCGCGGUCAGCAGUGCAACGUAUCCACAAUGGAUCGUAUGCACUGUCUGCCAUCGCCGGUCGAUCAUCCCAGUCGCGUGUUGGCAUCUGGAA
env-256   UCUAUUAGCGACGAUUGGAAUGCACCCGGUGCACACCGGGACGGCGGCAAAGAGAGCGA....................................................................................................
env-257   UCUAUUAGCGACGAUUGGAAUGCACCCGGUGCACACCGGGACGGCGGCAAAGAGAGCGACGAAACGCAGAGAGACGGAUGUCUCUCUGAUCCUCUCACGUUGCCGCCUCCAUCAACUGUUUCCUGACCCCCGGGCGUUUUGGCCGGGUGUUCGAUAUCUGCCCAGAUGGGUUCGCUAGA
env-258   UCUAUUAGCGACGAUUGGAAUGCACCCGGUGCACACCGGGACGGCGGCAAAGAGAGCGACGAAACGCAGAGAGACGGAUGUCUCUCUGAUCCUCUCACGUUGCCGCCUCCAUCAACUGUUUCCUGACCCCCGGGCGUUUUGGCCGGGUGUUCGAUAUCUGCCCAGAUGGGUUCGCUAG
env-259   UCUAUUAGCGACGAUUGGAAUGCACCCGGUGCACACCGGGACGGCGGCAAAGAGAGCGACGAAACGCAGAGAGACGGAUGUCUCUCUGAUCCUCUCACGUUGCCGCCUCCAUCAACUGUUUCCUGACCCCCGGGCGUUUUGGCCGGGUGUUCGAUAUCUGCCCAGAUGGGUUCGCUAGA
env-260   UCUAUUAGCGACGAUUGGAAUGCACCCGGUGCACACCGGGACGGCGGCAAAGAGAGCGACGAAACGCAGAGAGACGGAUGUCUCUCUGAUCCUCUCACGUUGCCGCCUCCAUCAACUGUUUCCUGACCCCCGGGCGUUUUGGCCGGGUGUUCGAUAUCUGCCCAGAUGGGUUCGCUAGA
env-261   UCUAUUGGCGACGACUGGAGUGUGCCCGGUGCACACCGGGCCGGCAGUGGAGUGGGCGACAGGAUUGUGCGGAGACGGAUGUCUCUUGUACUCUCAUAUCUACUGCCUCCAUCAACUGUUUCCCUUCACGCCCGGGUAGACGUACCGGGGUGUUCGAUGUCUUGCCCCGAGUUACGGGUU
env-265   UCUGUUGGCGACGACUGGAAAGCACCCGGUGCACACCGGGACGGCAGCAGAGAGCGGUUUGGACACGCAGAGAAGGAUAUAUCUGUGUGUCCCGCGCUCCGCUGCCUCCAUCAACUGUUUCCCUUGCGCCACGGCAAAAUGGCGUGACGCUCGAUGUCCCGCCCCGUACGGGUUCGCCG
env-266   UCUAUUAGCGACGACUGGAAUGGACCCGGUGCACACCGGGGCUGUAGCAGAGAACGGCAGGAUAUUCGGAGAUUGUUCUCUUUGUAUUCUCCAUGCUCUGCUACCUUCCGUCAACUGUUUCCCUUCCACCCCACAGUUUCCGGGUUGGCCGGAAGGGUUGCCAUGCCGGCACGUCCGGCA
env-268   UCUAUUAGCGACGACUGGAAUGGACCCGGUGCACACCGGGGCUGUAGCAGAGAACGGCAGGA.................................................................................................
env-269   UCUAUUAGCGACGACUGGAAUGGACCCGGUGCACACCGGGGCUGUAGCAGAGAGCGGCAGGAUAUUCGGAGAUUGUUCUCUUUGUAUUCUCCAUGCUCUGCUACCUUCCGUCAACUGUUUCCCUUCCACCCCACAGUUUCCGGGUUGGCCGGAAGGGUUGCCAUGCCGGCACGUCCGGCA
env-270   UCUAUUAGCGACGACUGGAAUGGACCCGGUGCACUACCGGGGCUGUAGCAGAGANCGGCAGGAUAUUCGGAGAUUGUUCUCUUUGUAUUCUCCAUGCUCUGCUACCUUCCGUCAACUGUUUCCCUUCCACCCCACAGUUUCCGGGUUGGCCGGAAGGGUUGCCAUGCCGGCACGUCCGGC
env-271   UCUAUUAGCGACGACUGGACACGGACCCGGUGCACGCCGGGGCGGUGGCGGGAGCGAUAGGAGAGGAUCGUCUCUCCCCCGUCGCCUCCAUCCGACUGUUUCCCUUCACGCCGUGCCAUCAUGGCACGACGUUCGAUGUCAUGCCCAAGUGGGUUCACCACAAUUUGCCCGAUCUGCGACC
env-272   UCUAUUAGCGACGACUGGACACGGACCCGGUGCACGCCGGGGCGGUGGCGGGAGCGAUAGGAGAGGAUCGUCUCUCCCCCGUCGCCUCCAUCCGACUGUUUCCCUUCACGCCGUGCCAUGAUGGCACGACGUUCGAUGUCAUGCCCAAGUGGGUUCACCACAAUUUGCCCGAUCUGCGAC
env-274   UCUAUUAGCGACGACUGGACACGGACCCGGUGCACGCCGGGGCGGUGGCGGGAGCGAUAGGAGAGGAUCGUCUCUCCCCCGUCGCCUCCAUCCGACUGUUUCCCUUCACGCCGUGCCAUGAUGGCACGACGUUCGAUGUCAUGCCCAAGUGGGUUCACCACAAUUUGCCCGAUCUGCGAC

          ...............................................................................................................................................................
          ...............................................................................................................................................................
          UCUYUUYR•GRRRR•YR••••••Y•••R•••RY•RR•R••••••••••••••••••••••••••••••••••••••••••••••R••••••••••••••••••••••••••••••••••••••••••••••••••••••••••••••••••••••••••••••
```

```
env-275   UCUAUUAGCGACGACUGGACACGGACCCGGUGCACGCCGGGGCGGUGGCGGGAGCGAUAGGAGAGGAUCGUCUCUCCCCCGUCGCCUC.........................................................................................................
env-277   UCUAUUAGCGACGACUGGACACGGACCCGGUGCACGCCGGGGCGGUGGCGGGAGCGAUAGGAGAGGAUCGUCUCUCCCCCGUCGCCUCCAUCCGACUGUUUCCCUUCACGCCGUGCCAUGAUGGCACGACGUUCGAUGUCAUGCCCAAGUGGGUUCACCACAAUUUGCCCGAUCUGCGAC
env-278   UCUAUUAGCGACGACUGGACGUACCCGGCGCACACCGGGCCGGUGGCGGGGAAGAUAGGAGGAGAUGGGCGUCUCCCCGCCGCCUCCAUCCAACUGUAUCCCUUCACGCCGCGGCAAAACGGCGUGGCGUUCGAUGUCAUGCCCGAUACGGGUUCGCCGUAAUGCCCGACUGCGGCGG
env-292   UCUAUUAGCGACGACUGGACGUACCCGGUGCACACCGGGCCGGUGGCGGGAGCGGUAGGAGACGAUCGUCUCCCCCGUGCCUGCCGCCUCCAUCCAACUGUUUCCCUUCACGCCGCGCCAUUCUGGCGCGUCGUUCGAUGUCAUGCCCGAUACGGGUUCGCCGUAAUGCCCGACCGUGCC
env-293   UCUAUUAGCGACGACUGGACGUACCCGGUGCACACCGGGCCGGUGGCGGGAGCGGUAGGAGACGAUCGUCUCCCCCGUGCCUGCCGCCUCCAUCCAACUGUUUCCCUUCACGCCGCGCCAUUCUGGCGCGUCGUUCGAUGUCAUGCCCGAUACGGGUUCGCCGUAAUGCCCGACCGUGCC
env-295   UCUAUUAGCGACGACUGGACGUACCCGGUGCACACCGGGCCGGUGGCGGGAGCGGUAGGAGACGAUCGUCUCCCCCGUGCCUGCCGCCUCCAUCCAACUGUUUCCCUUCACGCCGCGCCAUUCUGGCGCGUCGUUCGAUGUCAUGCCCGAUACGGGUUCGCCGUAAUGCCCGACCGUGCC
env-296   UCUAUUAGCGACGACUGGACGUACCCGGUGCACACCGGGCCGGUGGCGGGAGCGGUAGGAGACGAUCGUCUCCCCCGUGCCUGCCGCCUCCAUCCAACUGUUUCCCUUCACGCCGCGCCAGUUCUGGCGCGUCGUUCGAUGUCAUGCCCGAUACGGGUUCGCCGUAAUGCCCGACCGUGC
Mbo-1-1   UCUAUUAGCGACGACUGGACGUACCCGGUGCACACCGGGCUGGUGGCGGGAGCGAUAGGAGACGAUCGUCUCCCCCGUGCCUGCCGCCUCCAUCCAACUGUUUCCCUUCACGCCGCGCCGUUCUGGCGCGUCGUUCGAUGUCAUGCCCGAUACGGGUUCGCCGUAAUGCCCGACCGUGCC
env-297   UCUAUUAGCGACGACUGGACGUACCCGGUGCACACCGGGCCGGUGGCGGGAGCGAUAGGAGACGAGUGUCUCCCCGACCCCGCGGCCUCCAUCCAACUGUUUCCCUUCACGACGCGCUAGAACUGCGCGCCGUUCGAUGUCAUGCCCGAUACGGGUUCGCCGUAAUGCCCGACCGUGC
env-298   UCUAUUAGCGACGACUGGACGUACCCGGUGCACACCGGGCCGGUGGCGGGAGCGAUAGGAGACGAGUGUCUCCCCGACCCCGCGGCCUCCAUCCAACUGUUUCCCUUCACGACGCGCUAGAACUGCGCGCCGUUCGAUGUCAUGCCCGAUACGGGUUCGCCGUAAUGCCCGACCGUGCCG
env-303   UCUAUUAGCGACGACUGGACGUACCCGGUGCACACCGGGCCGGUGGCGGGAGCGAUAGGAGACGAGUGUCUCCCCGACCCCGCGGCCUCCAUCCAACUGUUUCCCUUCACGACGCGCUAGAACUGCGCGCCGUUCGAUGUCAUGCCCGAUACGGGUUCGCCGUAAUGCCCGACCGUGCCG
env-304   UCUAUUAGCGACGACUGGACGUACCCGGUGCACACCGGGCCGGUGGCGGGAGCGNUAGGAGACGAUCGUCUCCCCCGUGCCUGCCGCCUCCAUCCAACUGUUUCCCUUCACGCCGCGCCAUUCUGGCGCGNCGUUCGAUGUCAUGCCCGAUACGGGUUCGCCGUAAUGCCCGACCGUGCC
env-305   UCUAUUAGCGACGACUGGACACGGACCCGGUGCACGCCGGGGCGGUGGCGGGAGCGAUAGGAGAGGAUCGUCUCUCCCCCGUCGCCUCCAUCCGACUGUUUCCCUUCACGCCGUGCCAUGAUGGCACGACGUUCGAUGUCAUGCCCAAGUGGGUUCACCACAAUUUGCCCGAUCUGCGAC
env-306   UCUAUUAGCGACGACUGGACACGGACCCGGUGCACGCCGGGGCGGUGGCGGGAGCGAUAGGAGAGGAUCGUCUCUCCCCCGUCGCCUCCA................................................................................................
Mma-1-1   UCUAUUGGCGACGACUGGAAUGCACCCGGUGCACACCGGGACGGCAGCAGAGAGAACGGUCGAGGCGCACGGAGACGAACGUCUCUGUGACUUCGCGUUCCGCUGCCUCCAUCAACUGUUUCCCUUACGCUACGGCAAAAUGGCGUGGCGUUCGAUAUCCCGCCCCAUCCGGGUUCGCCA
Mse-1-1   UCUAUUGGCGACGACUGGAAUGCACCCGGUGCACACCGGGACGGCAGCAGAGAGAGCGGUCGAGGCGCACGGAGACGAAUGUCUUUGUGACUUCACGUUCCGCUGCCUCCAUCAACUGUUUCCCUUACGCUACGGCAAAACGGCGUGGCGUUCGAUAUCCCGCCCCAUCCGGGUUCGCCA
Msp-1-1   UCUAUUGGCGACGACUGGAAUGCACCCGGUGCACACCGGGACGGCAGCAGAGAGAGCGGUCGAGGCGCACAGAGACGAAUGUCUCUGUGACUUCACGUUCCGCUGCCUCCAUCAACUGUAUCCCUUACGCUACGGCAACAUGGCGUGGCGUUCGAUGUCCCGCCCCAUCCGGGUUCGCCA
env-310   UCUAUUAGCGAC..........................................................................................................................................................................
env-315   UCUAUUAGCGACGACUGGACGCACCCGGUGCACGCCGGGCCGGUGGCGGGAGCGAUCAGGAGACAGGCGUCUCUCUGACCCCCGCUGCCACCAUCCAACUGUUUCCCAUCACGUCGCGUCAGAACGGCGCGUCGUUCGAUGUCAUGCCCGAUACGGGUUCGCCGUAAUGUGCCUGAUCUG
env-316   UCUAUUAGCGACGACUGGACGCACCCGGUGCACGCCGGGCCGGUGGCGGGAGCGAUCAGGAGACAGGCGUCUCUCUGACCCCGCUGCCACCAUCCAACUGUUUCCCAUCACGUCGCGUCAGAACGGCGCGUCGUUCGAUGUCAUGCCCGAUACGGGUUCGCCGUAAUGUGCCUGAUCUG
env-317   UCUAUUAGCGACGACUGGACACGGACCCGGUGCACGCCGGGGCGGUGGCGGGAGCGAUAGGAGAGGAUCGUCUCUCCCCCGUCGCCUCCAUCCGACUGUUUCCCUUCACGCCGUGCCAUGAUGGCACGACGUUCGAUGUCAUGCCCAAGUGGGUUCACCACAAUUUGCCCGAUCUGCGAC
env-318   UCUAUUAGCGACGACUGGGCAUACCCGGUGCACGCCGGGCCGGUGGCGGGAGCGAUUCAGGAGACAGGCGUCUCCCUGACCCCCCGCUAUCACCAUCCAACUGUUUCCCAUCACGCCGCGCCGGAACGGCACGUCGUUCGAUGUCACACCCGAUACGGGUUCGCCGCAAUGUGCCUGACC
env-320   UCUAUUAGCGACGACUGGGCAUACCCGGUGCACACCGGGCCGGUGGCGGGAGCGAUGCAGGAGACAGGCGUCUCCCUGACCCCCCGCUAUCACCAUCCAACUGUUUCCCAUCACGCCGCGCCGGAACGGCGCGUCGUUCGAUGUCACACCCGAUACGGGUUCGCCGCAAUGUGCCUGACC
env-321   UCUAUUAGCGACGACUGGGCAUACCCGGUGCACGCCGGGCCGGUGGCGGGAGCGAUUCAGGAGACAGACGUCUCCCUGACCCCCCGCUAUCACCAUCCAACUGUUUCCCAUCACGCCGCGCCGGAACGGCACGUCGUUCGAUGUCACACCCGAUACGGGUUCGCCGCAAUGUGCCUGACC
env-322   UCUAUUAGCGACGACUGGGCAUACCCGGUGCACNCCGGGCCGGUGGCGGGAGCGAUNCAGGAGACAGGCGUCUCCCUGACCCCCCGCUAUCACCAUCCAACUGUUUCCCAUCACGCCGCGCCGGAACGGCNCGUCGUUCGAUGUCACACCCGAUACGGGUUCGCCGCAAUGUGCCUGACC
Mpa-1-1   UCUAUUCGCGAGACAGGUACAUUCUGGCGCACUCCAGAAGAUGCUCACCAGGAAGGAACGAUUCCGCUCUCUGUGAAAGGGGGGCUGAUCGCUCUGGACUGGCAUAAUCCUCAAGCGAAGUGCACUGCUCAAUCUCGGGUUUACGAUGCGGCAUCGUCCCGUCGAUAAACAUGAACAAAC
env-323   UCUAUUCGCGAGACAGGUACAUUCUGGCGCACUCCAGAAGAUGCUCACCAUGAAAGGACGGUCUGCCCCUGCAGAAGGUGGCAGAUCGCCCUGGACUGGCAUAAUCCUCAAGCGAAGUGCACUGCUCAAUCUCGGGUUUACGAUGGGGCAUCGUCCCGUCGGUAAGCAUCAACAAACGC
env-324   UCUUCUUUCGCGAGACAGGAAAUACCCGGUGCACACCGGAUCAUGCCGAAGAGACAAUAUCUUCUGCAUCCUCGUAACCGGACGAACCGACAGAGCAGGGAUAACCAGGAUUAUCUGCCACGAACUGGCGGAUAUCCCAAAAAAUUCCCCGAUCUUCGGCGUGCCGCGUGUUGGCUUAAG
env-325   ACUUCUUUCGCGAGACAGGAAUGCUGGUGCACACCAGUAGGGCAAUAACACAAGCUCAUUCGUGCAUCUCCAGAAACGGGUGAAUGGUAACCAUGACCCGCUGCCUUGAAAAACCACGUGGUUUUUCAUGGCAUGCCGUGGUCGGGCAUAAAAACCACCGUUCCCGCGUGUUGGCUUAAC
env-326   ACUUCUUUCGCGAGACAGGAAUGCUGGUGCACACCAGUAGGGCAAUAACACAAGCUCAUUCGUGCAUCUCCAGAAACGGGUGAAUGGUAACCAUGACCCGCUGCCUUGAAAAACCACGUGGUUUUUCAUGGCAUGCCGUGGUCGGGCAUAAAAACCACCGUUCCCGCGUGUUGGCUUAAC
env-331   ACUUCUUUCGCGAGACAGGAAUGCUGGUGCACACCAGUAGGGCAAUAACACAAGCUCAUUCGUGCAUCUCCAGAAACGGGUGAAUGGUAACCAUGACCCGCUGCCUUAGAAAAACCACGUGGUUUUUCAUGGCAUGCCGUGGUCGGGCAUAAAAACCACCGUUCCCGCGUGUUGGCUUAA
env-332   ACUUCUUUCGCGAGACAGGAAUGCUGGUGCACACCAGUAGGGCAAUAACACAAGCUCAUUCGUGCAUCUCCAGAAACGGGUGAAUGGUAACCAUGACCCGCUGCCUUGAAAAACCACGUGGUUUUUCAUGGCAUGCCGUGGUCGGGCAUAAAAACCACCGUUCCCGCGUGUUGGCUUAAC
Mfo-1-1   UCUUCUUUCGCGAGACAGGAAUACCGGUGCACACCGGUCCAGCGGUGGGAGUAAUCUCAUACGCACAUCCCCGAAACGAUGAACCAGUCUGCGGUCGGCAGUGUAAUGUAUCCCUACCGGAUCCUCUGCACUGCCUGUCGCGACUGCCUUUCAUCUCCGUCGCGUGUUGGCAUCUGUAAC
env-333   UCUUCUUUCGCGAACAAGAAAAAAACACCAAAACCGGGGAGCCGGGCAGUAAUAUGCCCCGGCCUCCCGGGAUAACGGUGUGCCGGCAAAGGGAAAACAACCGGUUUCAGGAGGUUUUCCCGCAUUUUGCCGGUCGCGUGUUGGCUUACCAGACAGGCACGUGAUAUCACUUCAGUCUCG
env-334   UCUUCUUUCGCGAAAAAAGCAAUACCGAAAAUAUCCGGGAACGAGGCACACAAAUGUCUCGUCCCAGAUAUAACGGUGUACCGACAAAGGGACAAUUACCGGUUCCGGUAGUUUUCCCUCAUGGCGGUCGCGUGUUGGCUUACCAAGACAGGCACGUGAUAUCACUUCAGUCUCGGAAUC
env-335   UCUUCUUUCGCGAAAAAGGAAACACUGAAUGUAUCAGGGAACGGGACACCCCGUGUCCCCUUCCUGAUAUAACGGUGUGCCGGCAAAAGGAAAAUUACCGGUUCCGGUGGUUUUCCGGUAUGCUGGUCGCGUGUUGGCUUACCAAGACAGGCACGUGAUAUCACUUCAGUCUCGGAAUCA
env-337   UCUUCUUUCGCGAAAAAAGCAAUACCGAAAAUAUCCGGGAACGAGGCACACAAAUGUCUCGUCCCAGAUAUAACGGUGUACCGACAAAGGGACAAUUACCGGUUCCCGGUAGUUUUCCCUCAUGGCGGUCGCGUGUUGGCUUACCAAGACAGGCACGUGAUAUCACUUCAGUCUCGGAAU
env-338   UCUUCUUUCGCGAAAAAGAAAAGCACCCGAACCGGGGAUACGGGCAGGAAUGUGCCCGGCUUUCCGGUUAACGGUGUGCCGGCAAAGGAAAAACAACCGGUUUUCCGGUGGUUUUUCCCGUAUCUUGCCGGUCGCGUGUUGGCUUACCAGACAGGCACGUGAUAUCACUUCAGUCUCGGA
env-339   UCUUCUUUCGCGAAAACGAAAAAAAACACCCGAACCGGGAAUCCGGGCAGUAAUGUGCCCGGCUUUUCGGUGAAGGUGUGCCGGCAAAGGAAAAACCACCGGUUUCCGGUGGUUUUUUUAUAUUUUGCCGGUCGCGUGUUGGCUUACCAGACAGGCACGUGAUAUCACUUCAGUCUCGGA
CMe-1-1   UCUUCUUUCGCGAGGAAAAAAAGCGGAGAUCCCUUGAGGGUGGGAGAAAGCUCUCUCCCUCUGCAGGUCUCCAGGCUAACCGGUUCACGGGCAGGGUGAGUAUGCUCCCGCGCAUCACGUGCGGGAAAAAUCGUCCUGCAAGAUGACACCGGAUCGCGUGUUGGCUUACCGGACAGGCAAG
env-340   UCUUCUUCGCGAGACUAGGAAUGCUGGUGCACAUCAGCUCAUGGAUGAAACAUCAUCCACAUGUCUGUAAGCUACAGAAAACAGGGAUAUUCAGUACUACAUGAAAUCCGCUUGCCGCGGAUCCUCGUUGUACUUGAGUUACCUCGUUUUUUCGAUGGCGUCGCGUGUUGGCUCAUUAAA
env-341   UCUUCUUUCGCGAGACAGGAAUGCUGGUGAAUACCAGCUCAUGGAUGAGGAAAUUAUCAUCCAUAUCUCCUUAAGCAGAGGAAAACCGGACGUUCAAUAAUACAAAAAAUCCGAAUAUAUGGAUCAUUGUAUUUUUUUGAAUUCGCCGGUUCUCUUCUCUGGCGUCGCGUGUUGGCAUCA
env-349   UCUUCUUUCGCGAGACAGGAAUGCUGGUGAAUACCAGCUCAUGGAUGAGGAAAUUAUCAUCCAUAUCUCCUUAAGCAGAGGAAAACCGGACGUUCAAUAAUACAAAAAAUCCGAAUAUAUGGAUCAUUGUAUGUAUUUUUGNAUUCNCCGUUUUUCUUCUCUGGCGUCGCGUGUUGGCAA
env-350   UCUUGUUUCGCGAGACUUAGGCAAGCUGGUGCACAUCAGCUCAUGGAUGAGGAAAAAACAUCCCUAUUGUCCGUACCGGAAAAGAAUGUCAAGAUCUUCAAUACAACCUUCAAAUCCGUGUUAUCGGAUCGGUGUAUAAUUGAACGAUCCUGGUUCACCGGCAUCGCGUGUUGGCUUCAG
env-351   UCUUCUUUCGCGAGACAGGAAUGCUGGUGCACAUCAGCUCAUGGGUGAAAAAUCUCAUCCAAAUGUCCGUAACCCAAUGAAAAACCGGAUGUUCGAUACUCCAUGAAAUCCGCUUAACGCGGAUCAAGGAAGUCCCGGAGACAUCCUGUUAUUCAGAUGGCGUCGCGUGUUGGCUAUAUC
env-352   UCUUCUUUCGCGAGACAGGAAUGCCGGUGCACAUCCAGCGGCGCAGGCGUUUGAGUUCAUCUUCUUCGCAUCUCCGUAACCGGGCAAACUGACAGAACGAGCGCAUACGGAUAAGGGCCCACAUAAAGGGAUCCCUCGUAUAGCUCACUAUUGUCGGCAUGUCCGUGUCGCGUGUUGGCUUUAU
env-353   UCUUCUUUCGCGAGACACAGAAUGCUGGUGCAUACCAGCUCAUGGAUUGGGACAGUCUCAUCCAUAUGUCCGUAGCCGGAUGAAAAACCGGAGUUCGAUAAUGCAUAAAAAAUCCGCAACCGCGGAUUAGUGUAUUACCCGAGGUAUCCGGUUAACCGACUGGCGUCGCGUGUUGGCAAC
env-354   UCUUCUUUCGCGAGACACAGAAUGCUGGUGCAUACCAGCUCAUGGAUUGGGACAGUCUCAUCCAUAUGUCCGUAGCCGGAUGAAAAAACCGGAGUUCGAUAAUGCAUAAAAAAUCCGCAACCGCGGAUUAGUGUAUUACCCGAGGUAUCCGGUUAACCGACUGGCGUCGCGUGUUGGCAA
env-355   UCUUCUUUCGCGAGACACAGAAUGCUGGUGCAGACCAGCUCAUGGAUUGGGACAGUCUCAUCCAUAUGUCCGUAGCCGGAUGAAAAAACCGGAGUUCGAUAAUACAUAAAAAAUCCGCAACCGCGGAUUAGUGUAUUACCCGAGGUAUCCGGUUAACCGACUGGCGUCGCGUGUUGGCAAC
env-356   UCUUCUUUCGCGAGACACAGAAUGCUGGUGCAUACCAGCUCAUGGAUUGGGACAGUCUCAUCCAUAUGUCCGUAGCCGGAUGAAAAAACCGGAGUUCGAUAAUGCAUAAAAAAUCCGCAACCGCGG......................................................
env-357   UCUUCUUUCGCGAGACAGGAAAGCUGGUGUAUAUCAGCUCGUGGAUGAGAAAAAUAUCUCCCUAUGUCCGUUGG..........................................................................................................
env-358   UCUUCUUUCGCGAGACAGAAAAGCUGGUGUAAUUCAGCUUUUGGAUGAGAAAAAUAUCAUCCGUAUGUCCGUAGCCACACGAAAAAUCCGGACGUUCAUGAAUACCGAAAAAUCCCGCAACAGUGGGAUAGCGUAUUACCUGAGUUGUCCGGAUUUCUCACCAGGCUUCGCGUGUUGGCA
env-359   UCUUCUUUCGCGAGACAGAAAAGCUGGNGUAAUUCAGCUUUUGGAUGAGAAAAAUAUCAUCNGUAUGUCCGUAGCCACANGAANAAUCCGGACGUUCAUGAAUACCCGAAAAAUCCCGCAACNGUGGGAUAGCGUAUUACCUGAGUUGUCCGNAUUUCUCACCAGGCUUCGCGUGUUGGC
env-360   UCUUCUUUCGCGAGACAGAAAAGCUGGUGUAAUUCAGCUUUUGGAUGAGAAAAAUAUCAUCCGUAUGUCCGUAGCCACACGAAAAAUCCGGACGUUCAUGAAUACCCGAAAAAUCCCGCAACAGUGGGAUAGCGUAUUACCUGAGUUGUCCGGAUUUCUCACCAGGCUUCGCGUGUUGGC
env-361   UCUUCUUUCGCGAGACAGAAAAGCUGGUGUAAUUCAGCUUUUGGAUGAGAAAAAUAUCAUCUGUAUGUCCGUAGCCACAUGAAAAAUCCGGACGUUCAUGAAUACCCGAAAAAUCCCGCAACAGUGGGAUAGCGUAUUACCUGAGUUGUCCGGAUUUCUCACCAGGCUUCGCGUGUUGGC
env-370   UCUUCUUUCGCGAGACAGGAAAGCUGGUAUACAUCAGCUCUCGGAUGAGCAAAAUAUCAUCCCUAUGUC...............................................................................................................
env-371   UCUUCUUUCGCGAGACAGGAUUGCUGAUCAAGAAUCAGCUCAUGGAUGAGUAUAGUAUCAUCCAUAUGUCCAUUGCCCGUUGCAUAACCGGAUGUUCAAUCAUAUAUAAAAUCCACAAAAGUGGAUUCGUGUAUAACCUGAAUUCUCCGGUUCUCUCAACUGGCGUCGCGUGUUGGCAUC
env-372   UCUUCUUUCGCGAGACAGGAACGCUGGUGCAUACCAGCUCAUGGAUGAGGAAAAUUUCAUCCAUAUCUCCUUAGGCAGAGGAAAAGCGGUCGUUCAAUAAUACAAAAAAAAUCCGUAUAUGCGGAUUAUUGUAUUUUUGUAUU................................
env-373   UCUUCUUUCGCGAGACAGGAAUGCUGGUGCAUACCAGCUCAUGGAUGAUGAAAUUAUCAUCCAUAUCUCCUUAAGCAGAGGAAAAGCGGACGUUCAAUAAUACAAAAAAAUAAUACAAAAAAUCCGAAUAUGCGGAUCAUUGUAUUUUUUGUAUUCGCCGUUUUUCUUCUCUGGAGUCGC
env-374   UCUUCUUUCGCGAGACAGGAUUGCUGAUAAAAAAUCAGCGUAAGGAUGAGGCAUUAUCAUCCUAAUGUCUAUACCCCGAUAAGAAAACCGGAUAUCCGAUAAUACCUGAAAAACCCAUAAUCUGGGAACGUGUAUUACCUGGACUUUCCGGUUUCCUAAACCGGCGUCGCGUGUUGGCAUC
env-375   UCUUCUUUCGCGAGACAGGAAUGCCGGUGCACACUGGCACAGUGAUGCAACUUCUGUUGUAUCCCCUAUAAUUACGGACGAAAAUACCAGUAUCAGUUAUCAGAGCCAGUUAUCCGUUACUACGGAAUGACCGCGCUGAUGGUGGUGGUAAGUUGCCCGCUA...........
env-376   UCUUCUUUCGCGAGACAGGAAUACCGGUGCACACUGGUACAGCGAUGCAACUUCUGUUGCAUCCCCUUAAACUACGGACGAAAAUACCAAUAUCGUCACAGGCUGUAAUCCGUUUAACGGAUGAAUUCCCCGACAAUCUUGGUGUGUUAUUGUCCGUGUCGCGUGUUAGUUUCUGAAACAAU
env-377   UCUUCUUUCGCGAGACAGGAAUGCCGGUGCACACUGGCACAGCGAUGCAACUUCUGUUGCAUCCCCUUAAUUACGGACGAAAACCAGCAUCAGUCAUAAGAGCCGGACAUCCAUAACUUUGGAUUGACUUCGCUGAUGGCAGGUGUCGGUGUUAUUGUCCGUGUCGCGUGUUGGCUUCUGA
env-378   UCUUCUUUCGCGAGACAGGAAUGCCGGUGCACACUGGCAAAGCGAUGCAACUCUCGUUGCAUCCCCUAACUAUGGACGAAUAU.................................................................................................
env-379   UCUUCUUUCGCGAGACAGGAAUGCCGGUGCACACUGGCAUAAGCGAUGCAAUUAUCCAUUGCAUCCCCUUACUACGGACAAAUAUUUUCUCAUCGUCAUCAGAGCCGGUAAUCCAUAAAUAAGGAUUUGUGCCCUGAUGGCUGCUGAUAUACAAGUCCGUGGCCGCGUGUUAGUUUCUGG
env-380   ACUUCUUUCGCGAGACAGGUAAGCUGGUGCACACCAGUAGGGCAAUAAAAUAACGCUCAAUCGUGCAUCCCCAGAAACGGGUGGAUAUCGAUCAUGAUCCGCUACCCUCAAACCCCCCGGGUUAAAAGGGCAGACUGUCAUCCCCGGUACACACCACCCGUUCCCGCGUGUUGGCUUAAC
env-381   ACUUCUUUCGCGAGACAGGUAAGCUGGUGCACACCAGUAGGGCAAUACAAUAACGCUCAAUCGUGCAUCUCCAGAAACGGGUGGAUAUCGAUCAUGAUCCGCUACCCUCAAACCCUUCGGGUUAAAAGGGCAGA........................................
env-382   ACUUCUUUCGCGAGACAGGAAAGCUGGUGCACACCAGUAGGGCAAUAAAAUAACGCUCAAUCGUGCAUCUCCAGAAACGGGUGGAUAUCGAUCAUGAUCCGCUACCCCCAAACCCCCCGGGGUUUUCAAGGGGCAGACGGUCAUAAUAAACCAUCCGUUCCCGCGUGUUGGCUU
env-383   ACUUCUUUCGCGAGACAGGAAAGCUGGUGCACACCAGUAGGGCAAUAAAAUAUCGCUCAAUCGUGCAUCCCCAGAAACGGGUGGAUAUCGAUCAUGAUCUGCUACCCUCAAACCCCCCGGGGUUAAAAGGGUCGACUGUCAUCAAAGGUACACACCACCCGUUCCCGCGUGUUGGCUUAA
env-384   ACUUCUUUCGCGAGACAGGAAUGCUGGUGCACACCAGUAGGGCAAAAAAGCUCAAUUGUGCAUCUCCAGAAACGGGUGGAUAUUUACGAUGAUCCACUGCUGAUAACCCCCGCGGGGGUUUUCCAGGGCAGAACAUGAUCAUGGAUAUACACCACCCGUUCCCGCGUGUUGGCUUACCA
env-385   ACUUCUUUCGCGAGACAGGAAUGCUGGUGCACACCAGUAGGGCAAUACAUAACGCUCAAUCGUGCAUCUCCAGAAACGGGUGGAUAUCUGCCACGAUCCACUGCCAAAAUACUCUUGGCAGACCGUGAACAGAGGAGU....................................
env-386   ACUUCUUUCGCGAGACAGGAAUGCUGGUGCACACCAGUAGGGCAAUACGUAACGCUCAAUCGUGCAUCUCCAGAAACGGGUGGAUAUCUGCCAUGACGCUGCCAACGAACUCUUGGCAGGCCGUGAACAGAGGAGUUAACCCCCCGUUCCCGCGUGUUGGCUUAACCGGAAGCGAAGCUA
env-387   ACUUCUUUCGCGAG............................................................................................................................................................................
env-389   ACUUCUUUCGCGAGACAGGAAUGCUGGUGCACACCAGUAGAGCGAUAUAAAAAAAGCUCAAUCGUGCAUCUCCAGAAACGGGUGAAUAUCAAAAACGAUCCGCUGCAAGAAAAUCCCCCCGGGGUUUUUCCGGACAGACCGUGAUCACAGGUAUAAACCAUCCGUUCCCGCGUGUUGGC
env-390   UCUUCUUUCGCGAGACAGGAAUGCUGGUAAAUACCAGACCCUGGAUGAGAAAAAUAUCAUCCAUAUGUCCGUAGCCAAAAAAAAUCGGAUAUCUCAAUAAUACGAAAAAAUCUGCAACUGCGGACUAGUGUAUUAUUUGUACUCUCCGAUUUCUCCCUCUGGCGUCCGCGUGUUGGCAUC
env-392   UCUUCUUUCGCGAGACAGGAACGCCGGUGCACACUGGCAUCGCCAUGAGAUCACAUCUAUGUGCAGC...............................................................................................................
env-395   UCUUCUUUCGCGAGACAGGUAUGCCGGUGCACAACCGGCUCAAGCGACGAGUAUUCCUUGUCGCAUCUCCGUAACCGGGCGAACAAUCCCCAGAGCGCAUACGGGUAAUCCACCCUGACGUGGUUUCCCGUG........................................
env-399   UCUUCUUUCGCGAGACAGGAAUACUGGUGCACACCGGUACAGCGAUGCAAUAACCAUUGCAUCCCCUCCAUCGGAUGAAUACAUCAGCUUCAGUCACCGGUCCGAAGGCAACCAAUACUGGUUUCAUGUCCCGAGAUAUGUCUGAUCUGUUGUCCGUCCCCGCGAGUUGGCUUCUGGAAUUA
env-400   ACUUCUUUCGCGAGACAGGAAUGCUGGUGCACACCAGUAGGGCAAUAGAAUAACGCUCAAUUGUGCAUCUCCAGAAACGGCUGGAUAUCGAUCAUGAUCCGCUGCC.....................................
env-402   UCUUCUUUCGCGAGACAGGAAUACCGGUGCACACUGGUACAGCGAUGCAACUUCUGUUGCAUCCCCUUAAACUACGGACGAAAAUACCAAUAUCGUUACAGGCUGUAAUCCGUUUAACGGAUGAAUUCGCCGAUGGUCUUGGUGUUAUUGUCCGUGUCGCGUGUUAGUUUCUGAAACAAUA
env-403   UCUUCUUUCGCGAGACAGGAAUACCGGUGCACACUGGUACAG.........................................................................................................................................
env-405   UCUUCUUUCGCGAGACAGGCUUGCCGGUGCACUCCGGCAAUACGAUGAGUAUAUUCUCAUCCGAAUCACCAUAACCGGACGAAAUGUGUUCCAAUAUCG....................................................................
env-406   UCUUCUUUCGCGAGACAGGCUU.........................................................................................................................................................
env-407   UCUUCUUUCGCGAGACAGGAAAAGGCUGAUGCACAUCAGCUCAUGGAUGAAAAAUAUCAUCCCCAUGUCCGUAACCUCAUGAAAAC....................................................................................

          ..........................................................................................................................................................................................
          ..........................................................................................................................................................................................
          UCUYUUYR•GRRRR•YR••••••Y•••R•••RY•RR•R••••••••••••••••••••••••••••••••••••••••••••••R•••••••••••••••••••••••••••••••••••••••••••••••••••••••••••••••••••••••••••••••••••••••••••
```

Mli-2-1	UCUGUUUGAGAGUAGUAAGUUGCCCUUUAUAACGUAUAGGUGCACUGUCGGAUGUCUGUGAAGAUGCAGUUUAUCCCCGGCCGGGUUUAAAGCCUGACUGCUGUACAUUCGUUUCUGUUCCGAUUCAGUCGUUUACUGUCCUUUGCAGGAUUUAUUUCUGCUUCUCGGAAGAAUAUUGUG
Mpe-1-1	UCUUUAGAGAGAGAUUUCCGAUAACAGGAAAGACAAGGCAGAGGAAAAACGUCCUGUAUAACAGGCAGGUUCCCGGCCGAAUGCAGACAGCAUUACAGUCUGCCGCAGUUAUGGUUAUUCUCAGAUUCAGCAGACCGAAAAUCCUUUUUGGUCCGGCCGAAAUCCGUAAACCCGGAUUUC
Mpa-2-1	UCUUUAGAGAGAGAUUUCCGAUAACAGGAAAGACAAGGCAGAGGAAAAACGUCCUGUAACAACAGGCAGGUUCCCGGCCGAAUGCAGACAGCAUUACAGUCUGCCGCAGUUAUGGUUAUUCUCAGAUUCAACAGACCGAAAAUCCUUUUUGGUCCGGCCGAAAUCCGUAAACACGGAUUU
env-408	UCUUUAGAGAGAGAUUUCCGAUAACAGGAAAGACAAGGCAUGGGAAGAGCACCAUAUUUAAGGCAUGCUUCCGGCCGAAUGCAGACAGCAUUACCGUCCACCGCAGUUAUGGUUAUUCUCAGAUUCAGCAGACCGAAAUCCUUUUUGGUCCGGCCGAAAUCCGUUAAAACGGAUUUCUCG
Mmo-1-1	UCUGUUUGAGAGACGUGAUACAAAGAUCGGUCUGCAAAGUUAACAUGCAGACGGGUCUUGGGAAUAUUAAUUCCAAGGCAAUGCGAGGCAAAGUCAGUUUGGUCUGAAUACCAAGUAUUGUGCCCUGAAAUUACACGGAUAUUUCAGGUUCGAAAAAAGACCGACGAAGACCGUCUGUUA
env-409	UCUGUUUGAGAGACGUGAUACAAAGAUCGGUCUGCAAAGUUAACAUGCAGACGGGUCUUGGGAAUAUUAAUUCCAAGGCAAUGCGAGGCAAAAUCAGUUUGGUCUGAAUACCAAGUAUUGUGCCCUGAAAUUACACGGAUAUUUCAGGUUCGAAAAAAGACCGACGAAGACCGUCUGUUA
env-410	ACUCUUUGAGAGACCGGGAAACCCGUUAUCGGGAAAGAAAUAUCAAUUCAUGAAAGGAGACGCUUCAGGCAUCCUGAUGUAUCAGGCAGAGCCCUUUCGUCCAGCAUUCAUGAGCCAGAACGAUUUCACAGGUCCUCUAUUGCUCCGGCCAACGACUCCCGCCUACCAGCCGUUGCGGCU
Mco-1-1	UCUAUUCGCGAAGGCCGCUAACGGUCGAGCUCGUGAAGAGGAGCGACGUGCCCCUGUACUGGGGCUACGAAGUGCGUAUCGCCGAUACGCUCCACGAUGCGCUGGAAACCGAUGGCUUAAGGAUAGCGACGUCGAGGCUGGGGCAUCCCCUGGCCUGCGAGAUGCUAUCCGAGAUAAAAA
env-413	UCUUUUCGCGAAGACCAAUACAAGCAAAGCUGGUAACAAAGGACGAGAUCCCCAUGUACUGGGGAUACCGUACAGCAGUAGAAGACAGCCUGUCAGGGGCGCUGGAAAAGAUUUCAGAAGAAGGUGCCCUGAUAAUCGCCACAUCCCGCCAAGG..........................
env-414	UCUUUUCGCGAAGACCAAUACAAGCAAAGCUGGUAACAAAGGACGAGAUCCCCAUGUACUGGGGAUACCGUACAGCAGUAGAAGACAGCCUGUCAGGGGCGCUGGAAAAGAUUUCAGAAGAAGGUGCCCUGAUAAUCGCCACAUCCCGCCAGGGUAAGUUGCUCGACAACACACUCCUGG
env-415	UCUUUUCGCGAAGACCAAUACAAGUAAAGCUGGUAACAAGGACGAGAUCCCCAUGUACUGGGGAUACCGUACAGCAGUAGAAGACAGCCUGUCAGGGGCGCUGGAAAAGAUUUCAGAAGAAGGUGCCCUGAUAAU..
env-416	UCUUUUCGCGAAGACCAAUACAAGCAAAGCUGGUAACAA..
env-418	UCUCUUUAAGACGACCAUUACAAGUAAAACUUGUAGAAAAAGAUGAGAUUCCACUAUAUUGGGGAUACUCAAUUAUAAUAGCAAAUAGCCUUUCUGAAGCUCUCAAAGAGCUUUCAAAACAAGGUGCACUGAUCAUAGUAACCGGACGACAGGGCCAGGUAUUAAAUACAAGUGUUCUAA
env-423	UCUCUUUAAGACGACCAAUACAAGCAGAACUUGUAGAAAAAAAUGAGAUUCCACUAUAUUGGGGAUACUCUACUAUAAUGGCAGAUAGCCUUUCUAAAGCUCUUAAAAAGCUUUCAAGGCAAAAUGCACUGAUCAUAGUAACUGCACGUCAGGGCCAGGUAUUAAAUACCAGCGUUCUAA
env-425	UCUCUUUAAGACGACCAAUACAAGCAAAACUUGUAGAAAAAGAUGAGAUUCCACUAUAUUGGGGAUACUCUACUAUAAUAGCAGAUAGUCUUUCUAAAGCUCUUAAAAAGCUUUCAAGGCAAAAUGCACAGAUCAUAGUAACUGCACGUCAGGGCCAGGUACUAGAUACCAGCGUUCUAA
env-428	UCUUUUCGCUAGACCCUCUCGCCGCUGAGCCUGUCGGUCGAAAAGAGAUCCCUCAAUACUGGGGGUAUGAAACAGAAGUAGUAGGUAGCCUGGAGAAAUGCCUAGAGGAUUAUCUGGGCUCAACUGAGGCUUUAGUGCUUCUAUCCUCCCGGAAGGGAAGAACUCUCACGACCCAACUCC
env-429	UCUUUUCGCUAGACCCCCUCGCCGCUGAGCCUGUCGGUCGAAAAGAGAUCCCUCAAUACUGGGGGUACGAGACAGAAGUAGUAGGUAGCCUGGAGAAAUACCUAGAGGAUUAUCUGGGCUCAAUUGAGGCUUUAGUGCUUCUGACCUCCAGGAAGGGCAGACCUCUUACGGCCGAACUCC
Msh-1-1	UCUUUUCGCGAAGACGCCACUUACAGUCGAGUAUAGCGAGGGAGCACAUACCCACAUACUGGGGCUACAGAACCCAUGUGUGCGGACCCCUCCACUCCACGCUCAGCGCCCUGCGCUCUCGGGGCUGGUGGGUGCUGGGCACAUCGAGGGAGGGAAGACCGCUGGACGCCAAUGCCCUCA
env-430	UCUUUUCGCAGGAACCGCUCGCCGCUGAGCCUGUGGACCAGAAGGAUAUACCCCAUUACUGGGGCUACGAGACCGAGAUAGUGGAUAGCCUGGAGAGCUAUCUGGCGUCAUUAGGAGAUGCCCGAAUCGUUCUCGCCUCCAGAAUGGCCGGGCAGUCACGCCCCGGGUAUCUCAGGAGA
env-433	UCUUUUCGCAUGAACCGCUCGCCGCUGAGCCUGUGGACCCGAAGGAGAUCCCUCAAUACUGGGGGUUCGAGACCGAGAUAGUGGAGAGCCUGGAAGGCUACUUAGCUGCAAAGCAUGAGCAGGGUAGCCAUGUGGUUCUCACCUCCCGGAAUGGAAAGGCCUAACGCCUGAACUCUCU
env-434	UCUUUUCGCAGGAACCGCUCGCCGCUGAGCCUGUGGACCAGAAGGACAUACCCCAUUACUGGGGGUACGAGACCGAGGUAGUGGAUAGCCUGGAGGGCUAUCUGGAAUCAAUGAGAGAUACCCGGAUCGUUCUCGCCUCCAGAAGCGGUCGGGCUAUAACGCCCCAGGUACUCAGGAGU
env-435	....
env-436	UCUUUUCGCAGGAACCGCUCGCCGCUGAGCCUGUGGACCAGAAGGAUAUACCCCAUUACUGGGGGUACGAGACCGAGGUAGUGGAUAGCCUGGAGAGCUAUCUGGAAUCAAUGAGAGAUACCCGGAUCGUUCUUGCCUCCAGAAGCGGUCGGGCUAUAACGCCCCANAUACUCCAGGAGU
env-437	....
env-439	UCUUUUCGCAGGAACCGCUCGCCGCUGAGCCUGUUGACCAAAAGGAUAUACCCCAUUACUGGGGGUACGAGACCGAGGUAGUGGAUAGCCUGGAGNGCUAUCUGGAAUCAAUGAGAGAUACCCGGAUCGUUCUUGCCUCCAGAAGCGGUCGGGCUAUAACGCCCCAGGCACUUCUGGAGU
env-440	UCUUUUCGCAGGAACCGCUCGCCGCUGAGCCUGUNGACCANAAGGAUAUACCCCAUUACUGGGGGUACGAGACCGAGGUAGUGGAUAGCCUGGAGAGCUAUCUGGAAUCAAUGAGAGAUACCCGGAUCGUUCUUGCCUCCAGAAGCGGUCGGGCUAUAACGCCCCAGG............
env-441	UCUUUUCGCAGGAACCGCUCGCCGCUGAGCCUGUUGACCAAAAGGAUAUACCCCAUUACUGGGGGUACGAGACCGAGGUAGUGGAUAGCCUGGAGAGCUAUCUGGAAUCAAUGAGAGAUACCCGGAUCGUUCUUGCCUCCAGAAGCGGUCGGGCUAUAACGCCCCAGGCACUUCUGGAGU
env-442	UCUUUUCGCAGGAACCGCUCGCCGCUGAGCCUGUUGACCAAAAGGAUAUACCCCAUUACUGGGGGUACGAGACCGAGGUAGUGGAUAGCCUGGAGAGCUAUCUGGAAUCAAUGAGAGAUACCCGGAUCGUUCUUGCCUCCAGAAGCGGUCGGGCUAUAACGCCCCAGGCACUUCUGGAGU
env-443	UCUUUUCGCAGCAGCGCUCGCCGCUGAGCCUGUGGACCAGAAGGAUAUCCCCCAUUACUGGGGGUACGAGACCGUUGUAGUGGAGAGCUUAGAGAGCUACCUUGCUGCAAAGCAGGGUGGCUACUGGGUUCUUGCCUCCGCGGUACGGAAAAGGCAUAACGCCUGAAAUCCUCU
env-444	UCUUUUCGCUGAACCGCUCGCCGCUGAGCCUGUGGAUAUGUUGGAGAUCCCCAGAGUACUGGGGGUAUGAGACCGAGGUAGUCGGCAGCCUGGAGAAAUAUCUCUGGCCAAGGAAGCCUUUGUCAUACUUACUUCCAAGAAGGGCAGGGUCAUAGAUCCAAAACUCCUUCAGAGCAUAG
env-445	UCUUUUCGCAGGAACCGCUCGCCGCUGAGCCUGUGGACCA..
env-446	UCUUUUCGCAGGAACCGCUCGCCGCUGAGCCUGUGGACCA..
env-448	UCUUUUCGCUGAGCCGCUCGCCACUGAGCAAGUGGAUCGGAGUAAGAUUCCCGGCUAUUGGGGCUACCAGACGGUAUCGGUGGAGAGCGCUGAAGAGUACCUGCGCUCCUGGAACCCACGGGUGGUGGCUACAUCCCGAAAGGGACGGGUGGUGACUCCGGAUCUGCUGGAGCAGAUAG
env-449	UCUUUUCGCGAAACCGCUCGCCGCUGAGCCUGUGGACCAGGAAGGAAAUUCCCCAGUACUGGGGGUAUGAGACAGAGGUAGUAGAGAGCCUUGAGAAAUAUCUGG...
env-450	UCUUUUCGCAUGAACCGCUCGCCGCUGAGCCUGUGGACCCGAAGGAUAUCCCUCAUUACUGGGGAUUCGAGACCGAGAUAGUGGAGAGCCUGGAAGGCUACUUA..
env-451	UCUUUUCGCAUAACCCGCUCGCCGCUGAGCCAGUGGACCAGAAGGAGAUCCCUCAUUACUGGGGGUUUGAAACCGAAGUAGUGGAUAGCCUUGAAGGCUACCUGGCGUCGAAAGAUGCCGCCUCCGUGCUUCUCACCUCCAUAAAAGGAAAGGCGAUAACGCCAGAAAUCCUCCAGCAAA
env-452	UCUUUUCGCAUAACCCGCUCGCCGCUGAGCCAGUGGACCAGAAGGAGAUCCCUCAUUACUGGGGGUUUGAAACCGAAGUAGUGGAUAGCCUUGAAGGCUACCUGGCGUCGAAAGAUGCCGCCUCCGUGCUUCUCACCUCCAUAAAAGGAAAGGCGAUAACGCCAGAAAU...........
env-455	UCUUUUCGCAUAACCCGCUCGCCGCUGAGCCAGUGGACCAGAAGGAGAUCCCUCAUUACUGGGGGUACGAGACCGAAGUAGUGGACAGCCUUGAAGGCUACCUGGCAUCGAAAGAUGCCGCCUCCGUGCUUCUUACCUCCAUAAAAGGAAAGGCGAUAACGCCAGAAAUCCUCCAGCAAA
env-456	UCUUUUCGCGAAAACCGCUCGCCGCUGAGCCAGUAGACCGAAAGGAGAUCCCUCACUACUGGGGGUAUGAGAUAGAAGUAGUCGAUAGCCUUGAAGGCUACCUGGCGUCGAAGGACGACGCCCUUUGUGCUUCUCACCUCCAGAAAAGGAAGGCCAUAACGCCAGAAACCCUCCAGAAGA
env-460	UCUUUUCGCAUAACCCGCUCGCCGCUGAGCCUGUGGACCAAAAGGAGAUCCCUCAUUACUGGGGUUACGGGACCGAAGUAGUGGAAAGCCUUGAAGGCUACCUGGCAUCAAGAGACGAUGCCCAAGUGGUUCUAGCCUCCAGGAAUGGAAAGGCUAUCACGCCCGAAAUCCUCCAGAAGU
env-462	UCUUUUCGCAUAACCCGCUCGCCGCUGAGCCUGUGGACCAAAAGGAGAUCCCUCAUUACUGGGGUUACGGGACCGAAGUAGUGGAAAGCCUUGAAGGCUACCUGGCAUCAAGAGACGAUGCCCAAGUGGUUCUAGCCUCCAGGAAUGGAAAGGCUAUCACGCCCGAAAUCCUCCAGAAGU
env-464	UCUUUUCGCAUAACCCGCUCGCCGCUGAGCCUGUGGACCAAAAGGAGAUCCCUCAUUACUGGGGUUACGGGACCGAAGUAGUGGAAAGCCUUGAAGGCUACCUGGCAUCAAGAGACGAUGCCCAAGUGGUUCUAGCCUCCAGGAAUGGAAAGGCUAUCACGCCCGAAAUCCUCCAGAAGU
env-465	UCUUUUCGCAUAACCCGCUCGCCGCUGAGCCUGUGGACCAAAAGGAGAUCCCUCAUUACUGGGGUUACGGGACCGAAGUAGUGGAAAGCCUUGAAGGCUACCUGGCAUCAAGAGACGAUGCCCAAGUGGUUCUAGCCUCCAGGAAUGGAAAGGCUAUCACGCCCGAAAUCCUCCAAAGCU
env-466	UCUUUUCGCAUGAACCGCUCGCCGCUGAGCCUGUGGACCAAAAGGACAUACCCCAUCUACUGGGGGUAUGAGAUAGAGGCAGCACAGAGCCUGGAGGCCGCA...
env-467	UCUUUUCGCUGAAACCCCUCGCCGCGGAGCCGGUAGACCGAUCGGAGAUACCUCACUACUGGGGCUACGAGACAGAGGU...
env-468	UCUUUUCGCUGAAACCCCUCGCCGCGGAGCCGGUAGACCGAUCGGAGAUACCUCACUACUGGGGCUACGAGACAGAGGUCAUCCCGAACCUGGACGACCACGUCCGCUCGACAGAGAGGCUAGUCGUGGCCACGUCCAGGAGGGGCGCGCCAGCCACCCCAGAACUCCUGAGCCAAAUGG
env-469	UCUUUUCGCUGAAACCCCUCGCCGCGGAGCCGGUAGACCGAUCGGAGAUACCUCACUACUGGGGCUACGAGACAGAGGU...
env-471	UCUUUUCGCUGAAACCCCUCGCCGCGGAGCCGGUAGACCGAUCGGAGAUACCUCACUACUGGGGCUACGAGACAGAGGUCAUCCCGAACCUGGACGACCACGUCCGCUCGACAGAGAGGCUAGUCGUGGCCACGUCCAGGAGGGGCGCNCCAGCCACCCCAGAACUCCUGAGCCAAAUGG
env-472	UCUUUUCGCUGAAACCCCUCGCCGCGGAGCCGGUAGACCGAUCGGAGAUACCUCACUACUGGGGCUACGAGACAGAGGUCAUCCCGAACCUGGACGACCACGUCCGCUCGACAGAGAGGCUAGUCGUGGCCACGUCCAGGAGGGGCGCGCCAGCCACCCCAGAACUCCUGAGCCAAAUGG
env-474	UCUUUUCGCUGAAACCCCUCGCCGCGGAGCCGGUAGACCGAUCGGAGAUACCUCACUACUGGGGCUACGAGACAGAGGUCAUCCCGAACCUGGACGAACACGUCCGCUCUAGCGACGGGCUAGUGGUGGUCACGUCCAGGCGUGGUAAGCCAGCAACCCCUAGACCUUCUGGCCCAGACGG
env-475	UCUUUUCGCUGAAACCCCUCGCCGCGGAGCCGGUAGACCGAUCGGAGAUACCCCAUUACUGGGGCUACGAGACCGAGGUCAUCCCAGACCUGGACGGGCACGUCCGCUCGAGCGACGGCUGGUGGUGGCCACGUCCAGAUUGGGGACGCCAGCAACCCCAGACCUCCUCGCGCGGGUGG
Mha-1-1	UCUUUUCGCUGAAACCCCUCGCCGCGGAGCCUGUAGACCGAUCGGAGAUACCCCAUUACUGGGGCUACGAGACCGAGGUCAUCCCGGACCUGGACGAGCACGUCCGCUCGAGCGGGCGGCUGGUGGUGGCCACGUCCAGAUUGGGGACGCCAGCAACCCCAGACCUCCUCGCGCGGGUGG
env-476	UCUUUUCGCUGAAACCCCUCGCCGCGGAGCCGGUAGACCGAUCGGAGAUACCCCACUACUGGGGCUACGAGACCGAGGUCAUCCCGAACCUGGACGAGCACGUCCGCUCGAGCGAGCGGCUGGUCGUGGCCACGUCCAGAUUGGGGACGUCAGCAACCCCAGACCUCCUCGCGCGGGUGG
env-477	UCUUUUCGCUGAAACCCCUCGCCGCGGAGCCGGUAGACCGAUCGGAGAUACCUCACUACUGGGGCUACGAAACAGAGGUCAUCCCGAACCUGGAAGAACACGUUCGCUCUAGCGAUAGGCUAGUCGUGGCCACGUCCAGGCAGGGCACGCUGGCGACCCCAGACCUCCUGAGGCAGACGG
env-478	UCUUUUCGCUAGAACCCCUCGCCGCUGAGCCCGUGGAUAAAAGAACAUCCCCCUGUACUGGGGGUACGAUACGAAGGUAGUGGGAAGCCUAGAGGAUUAUCUCAGGGCGACCAAGGCCUUCGUAGUGCUCACCUCU..
env-479	UCUCUCGCUGGACCCGCUCGCCGCGGAGCCCGUAGAUCAAAACGAGAUACCCCACUACUGGGGCUACGAGACUAUAGUCGUGGACGACCUGGAGGAGUACCUCCUGUCCACCGACGCCCGCCUCGUAGUCGCAUCGAGAAAAGGCAGACCGGCGACCCCAGAUCUCCUGGAGAAUAUGG
env-480	UCUUCUCGCUGGACCCGCUCGCCGCGGAGCCCGUAGACCAAAACGAGAUACCCCACUACUGGGGUUACGAGACGAUAGUCGUGGAUGACCUGGAGGAAUACCUCCAGUCUAUCGACGCCCUGCUCGUGGUCGCAUCGAGAAAAGGCAGACCGGCGACCCCUAGAUCUCCUUAGAGAAUAUGG
env-481	UCUUUUCGUUGACGCCCUUGGUGGUAGAGCGUGUCCAAAAAGACGAAAUCCCCAAAUAUUGGGGAUACACCGUAAGGAUAGAGGGAAACCUUACCUCUACACUCAAAAAAUGAGGAAAGAAGGUAUGUACAUAAUAGGUACUUCAAAGUAUGGAGAUAGUGUGAGUAUGGAAAAACUUG
env-482	UCUGUUCGCGAAGAAACCGUUGGAAGUGGAACCGGUUGAAAAACCGGAAGUGUGGGGAUACCGGGUAAAAGAGGUCAAGCUGAGAGAGCUUGUCCAGAAGGAUCUGCUGAUAACCUCCAGAAAAUGUCCCUAUGCGGAUCCGCAGGGGAUAAAGAGGUAUAUCCAGUCGAGAGAUGAGAUAU
env-483	UCUUUUCGCGAGAUCCGCUCGAAGUUGAAGCGAUUCAAAAGCCACCUGAAUAUUGGGGAUACUCGGUUCGGGUGACAGCUAGCAUCAAAGAGGCACUGGAAGGGUUUGUGGUGACCGCCGCGAAGCGCGGACAGGUGAUAAACCCUGAAAUGUGCGAUGAAAUUAAAAGGAGAGGCACCG
env-484	UCUUUUCGCGA...
env-485	UCUUUUCGCGAGAUCCGCUCGAAGUUGAAGCGAUUCAAAAGCCACCUGAAUAUUGGGGAUACUCGGUUCGGGUGACAGCUAGCAUCA..
env-486	UCUUUUCGCGAGAUCCGCUCGAAGUUGAAGCGAUUCAAAAGCCACCUGAAUAUUGGGGAUACUCGGUUCGGGUGACAGCUAGCAUCAAAGAGGCACUGGAAGGGUUUGUGGUGACCGCCGCGAAGCGCGGACAGGUGAUAAACCCUGAAAUGUGCGAUGAAAUUAAAAGGAGAGGCACCG
env-492	UCUUUUCGCGAGAUCCGCUCGAAGUUGAAGCGAUUCAAAAGCCACCUGAAUAUUGGGGAUACUCGGUUCGGGUGACCGCUAGCAUCAAAGAGGCACUGGAAGGGUUUGUGGUGACCGCCGCGAAGCGCGGACAGGUGAUAAACCCUGAAAUGUGCGAUGAAAUUAAAAGGAGAGGCACCG
env-495	UCUUUUCGCGAGAUCCGCUCGAAGUUGAAGCGAUUCAAAAGCCGCCUGGAUAUUGGGGAUAUUCGGUUCGGGUGGUAGCUAGCAUCAAAGAAGCACUGGAGGGGCUGGUGGUGACCACCGCGAAGCGCGGACAGGUAAUAAACCCUAUAAUGCGAGAUGACAUUAAAAGGAGAGGCACCG
env-504	UCUUUUCGCGAGAUCCGCUUGAAGUUGGACUGACUCAGAAGCCGCCGCGUUAUUGGGGGUAUUCGGUGCGGGUGGUCGAUAGCAUCGCAGACGCGUUGGAGGGGUUGGUCAUAGCCACGUCGAGGCACGGACAGCCAAUAAGUCCCGCUAUACACAAUGAAAUAAGAAGGAGAA......
env-505	AUCUUACGAGAGACCGGUAUCUGAACGCAUUACAUCUGACUGCCUGACUACACGUCAAGGAUUUCGUUCUGGCACCCUGAGUAAUAAAGGGCGCAUAAGACUUCUGGUACCUUGACGCUGUGGGGAGAGGCAGGUCUGAACGUUCGUUCAGCUGAAGUUACGCUUCAUGCAACUUCUGCU
env-508	UCUCUUCGCGAGACAGGUCACAGGGGAGACGGCCAGGAGCCAGAGGAAAGGCCGCCUUUCCGGGGUACUGCACCAGAAGGGGGAGUCUCCCUCUUCGCACCGCUGCGGCGAUGCGCCGGGCGCCUGGCUAGAGUCCGGCGUCCUGCAGGCAUAUGCGUGCCAGCGGCCCGGAAACAUC
env-509	UCUCUUUGCGAGACAGGUCACAGGGAGGCAGUCGCUGCCAGGAGAAAGACUGCCUUUCCGGGGUACUGCACCGGGAGCGGGUAUCUUCCCCUCCAACCCUGCGGCGAAGAAUGGAUCGGGUGCCUGUGCAUGCAAUGCGGGGCAUCCUCCUAUCUUCCGCACGUCAGGAGCCCGGACACA
Mta-1-1	UCUCUUUGCGAGACAGGUCACAGGGAGCGGUCACUAACCAGGAACGAAAUCCCCAAAUAUUGGGGAUACACCGUAAGGAUAGAGGGAAACCCACUCCAUACUGCUGACGGAGGGAUAGACGGGUGCCUGGACAUCUAUCCAUCGCCCUUUCCCCCUCCGCGCGACAGGAGCCCGGACACGUCA
env-511	UCUCUUUGCGAGACAGGUCACAGGGAGACGGUCACUAACCAGGAACAGGGCCGUCUUUUUGGAGUACUGCACCUGGAGGGGGAAAACCCACUCCAUACUGCUGACGGAGGGAUAGACGGGUGCCUGGACAUCUAUCCAUCGCCCUUUCCCCCUCCGCGCGACAGGAGCCCGGACACGUCA
env-512	UCUCUUUGCGAGACAGGUCACAGGGAGACGGUCACUAACCAGGAACAGGGCCGUCUUUUUGGAGUACUGCACCUGGAGGGGGAAAACCCACUCCAUACUGCUGACGGAGGGAUAGACGGGUGCCUGGACAUCUAUCCAUCGCCCUUUCCCCCUCCGCGCGACAGGAGCCCGGACACGUCA
Hbo-1-1	UCUCUUCGCGAGAACCGGUCGUGCGCGGAUUGGUGACGAGCCCGUUCCAGGUUCGACGUAUCCCGCAUGGACCUCGAGGAAGCACUCCGCGCCGCCGAUGCGGGCAUCACCAUCGCUACGUCCCGCUUCGGUGAUGCGCUGACGGUGCCGAAACUGGCGGAUCUCCCGCGAAUUG
Ngr-1-1	UCUCUUCGCGACGACCGGUCCGGGCGAAACUCGUCGAUUGCCCCCUUCCGGGGCUUUCGAUCGAGCGGACGGACCUAUCGGCAGCACUCGGCCGUGAGGAUGCUGGCGUCCGUAUUGCCGCCUCUCGAUUCGGUGAGCACCUCACCGUCGGGCGGCUCGAGACGCUGGCCGGACGCAUCG
Hje-1-1	UAUCUUCGCGACGACCGGUCCGUGCAAAGCUCACCGAGGAGCCUGCGGGGCUCGAAGUCGAGCGCACGGACCUUCCGGCAGCACUCGGCCGCCCGAGCGCGGGCGUCCGGAUCGCGACUUCUCGCUUCGGCGAGGAGCUCACGACCGGACGUCUGCCGGGGCUUUCGGCGCGCAUCGAAG
Nma-1-1	UCUCUUCGCGACGACCGGUCCGGGCGAAACUCGUCGACGAGCCCCUCCCGGGGCUUACCGUCGAGCGGACGGACCUUCAGGCGGCUCUCAGCCGUGAGGACGCCGGCGUUCGAAUUGCGGCUUCCCGAUUCGGUGCAGAACUCACCGUCGGGCGGCUCGGAACGCUGGCCGGACACACAG
Hal-1-1	UCUCUUCGCGAGAACCGGUCCGUGCGCGGAUCGUCGACGAGCCCCUUCCGGGCUUCGACGUAUCCCGCAUGGACCUCACGGAAGCACUCGGCAGGCCGGAUGCGGGCGUGCGAAUCGCCACGUCUCGCUAUGGCGAACCGCUGUCGGUCCCCACAUUGGGCGACUUGACGGCCCGCAUCA
Nti-1-1	UCUCUUCGCGACGACCGGUCCGGGCGAAACUCGUCGACGAUCCCCUUCCGGGAUUGUCGGUCGAGCGGACGGACCUCUCCGCAGCACUCGGCCGUGAGGACGCCGGCGUCCGUAUCGCAGCUUCCCGGUUCGGUGAGAAUCUCACCGUCGGGCGGCUCGAGACGUGGCCGGACGCGUCG
Npe-1-1	UCUCUUCGCGACGACCGGUCCGGGCGAAACUCGAAGACGUUCCCCUUCCGGGGCUCGCCGUCGAGCGGACGGACCUUUCGACAGCACUCGGCCGUGAGGACGCCGGCGUCGUCAUCGCCGCCUCCCGAUUCGGUGAAGAACUCACCGUCGGGCGGCUCGAGACGCUGGCCGGACGCAUCG
Hti-1-1	UCUCUUCGCGAGACGACCGGUUCGCGCGAAGAUCGUCGAUCAGCCCCCACCGGGCUAUCAGGUCGACCGUGCGAACCUUGACGCUGCGCUCUCGCGGCCCGACGCCGGCGUGCGGAUCGCCGCGUCCCGACACGGCCAGUCGCUCACGACCGAGCGACUCGGUCGACUCGUCGGAACGAUCG
	....
	....
	UCUYUUYR•GRRRR•YR•••••••Y•••R•••RY•RR•R••R••

alignment positions 440 · · · 557

```
Mli-1-1   GUCCCCGUUCGCAAGUGUUUGCGCAAUCCUGAGCAGGAAACCUGGGAUUCUCGGAGAGAACCAAAGCAGAGGAUUGAUAGGAACAUGGCGAAUAUACACAGGCCACGCAGGGGUUCCC
env-1     AUCAUAUCAUAAACUAUGAUAUGCCGGUUAGAAAAUAUCCAGCGGCAUCUUUCAUGAGGAUCAUGAACUAUGCCGGUUUAAGGAUUGUAAUAAUUAGGACUUACAUACAAUGCCACGA
env-2     UCGAUACAUAUCUUUCAGAUAAGGUUGCCUUGAUAUUUGGAUCCCCAGAUAAAGGAGUUGAUGCUUUCAUUGAUCAAGAUCAGCUUGUCGAUGAUUCUAUUAACACAGUUGUCAAGCA
env-3     ACCAGUAUCUUUCAGAUCGAACCGCAGUUAUAUUUGGAUCUCCAGACAGGGGAGUCAGCCAGAUAACUGGUAGGAUGGACCUUGUGGACCAGGAAAUAAACACAAUUCCAAACCAAGG
env-4     ......................................................................................................................
env-5     ACGACCACGCCGUCUCCCGUGCGGCAUCACAACCAGAUAGGACUGAUCGGACAAUGCCAAAAAUUAACAGGCCGCGGCGGGGAUCGCUUGCGUACAGCCCGAGAAAGCGGGCAAAGAG
env-6     ......................................................................................................................
env-8     GUCUCUUUCCUGCAUCGAAGCACUACCGGAAAUCGGGGUGUUCCCAGAUACAGUAAAGAAGGACUGAUUGAUACAUGCCAACUAUUCACAGACCUCGCAUGGGUUCCCUGGCAUAUAG
env-10    CUCUUUCCUGCAUCGAAGCAUACCGGAAUUCCGGUGUGUCCCCAGAUACAGAAAAGAAGGACUGAUUGAUACAUGCCAACAAUUCACAGACCUCGCAUGGGUUCUCUGGCAUACAGCC
env-11    CUUUCCUGCAUCGAAGCAUACCGGAAUUCUGGUGUGUCCCCAGAUACAGAAAAGAAGGACUGAUUGAUACAUGCCAACAAUUCACAGACCUCGCAUGGGUUCUCUGGCAUACAGCCCG
env-13    AUCCCCGGCUCAUAUGGCCGAUAUGGGCGAUUUACAUUUCCGGGAAACCGGAUUUGCGGUCGAAUAAGACCCGAAGAUUGAGUCAUGUAGAGGAUUGAUUGAACAAUGCCCAAGAUCA
env-14    GAACAGACCAUGCCGAAGAUCAACAGGCCACGCAGGGGUUCCCUUGCCUACAGUCCGCGAAAACGGGCCAAGAGUCCGAUACCACGGUACCAUUCCUGGGCGGACACUCGGGGAGAAC
env-15    ACCGGGCGGGAGCGACAAUCGUCUUUGGUGCGCCUGGUCGUGGGCUCCUAGCCAUCCUUGGCGAUGACAACCCACCAAAUACUCAAACGCAGAUGGGUGAGCACGCGAGUUCACACAU
Hwa-1-1   AUCGCGCGGGAGCGACAAUCGCUUUUGGUGCGCCUGGUCGUGGGCUUCCAGUCAUCCUUGGCGAUGAUAAUGCGCCGGAUAGCCAAACACAAACGGAUGAGAAAACGGUCUCACCCAC
env-17    AUCGCGCGGGAGCGACAAUCGCUUUUGGUGCGCCUGGUCGUGGGCUUCCAGUCAUCCUUGGCGAUGAUAAUGCGCCGGAUAGCCAAACACAAACGGAUGAGAAAACGGUCUCACCCGC
env-23    CCGACGGAACGGCUGUCGUCUUCGGUGCGCCUCAGCGGGGACUUCCGGCGAUGUUGGGGGUUGCGCCCGAGCAGGUGACCGGUGAGCGGGCAUCUGCCGGGCGUGACCAGUCCCAGUC
env-24    CAGACCUCGCCUUAGUCUUCGGCUCUCCCGAGCGCGGCGUUCACGAGAUUCUGAGGGAGCGUGACGCUUCCGUCGAUUUCGAUUACGUACUAAACACCGUGCCCGACCAGGGCGUAGA
env-25    GUCGGUUCAUGAUUGCGUUUGGCGCCCCCGAGCGUGGCCUCCCGGCGAUACUUGGGAUUGCUCCGGACGCGGUCCGCGGAGACGACCAGCGCGAGACUGGCCGGUUUGAUCUCUGGCU
env-27    CCGAGGAUGGGAUGACGGUCGUCUUCGGCGCCCCCGGACGGGGGCUGCUGGCGAUGGCCGACGCAGAUCCAGAUGCACUCGCUGCGGGGAUGUCCCCGCUGGGGGUGGAUCACUGGCU
env-28    AAGACGGCAUGACAGUCGCCUUCGGCUCGCCCGAGAGAGGGUU...........................................................................
env-29    AAGACGGCAUGACCGUCGCCUUCGGCUCGCCCGAGAGAGGGUUGCCCGAGAUACUCGACAUCGACGCGACCGCCGUUGAGGCGUCCACCGGGGACGACGGAGACAUCGAACCUGCCGA
env-30    AAGACGGCAUGACAGUCGCCUUCGGCUCGCCCGAGAGAGGGUUGCCCGAGAUACUCGGAAUCGACGCGACCGCCGUUGAGUCGUCCAGCAGGGACGACGGAGACGUCGAACCCGCGGC
env-31    UACGGCACUCUCGCGUGACGACGCCGGACUCACCAUCGCGUCAUCGCGAUACGGUGAGCUGGCGACGACUCGUCGACUCGGCCAACUGGCCGACCGACGAGACCGCG...........
Hut-1-1   CGUCGGCUGGCGACAUGACAGUCGCCUUUGGCGCGCCGGAGCGCGGCUUGCCGGCUAUCCUCGGCGUCGAACCAGGUGCUGUCGACCCGUCCACUGUGGACGAGUUGGCGGCAGCCGU
env-32    CCGAGGAUGGGAUGACGGUCGUCUUCGGCGCCCCCGGACGGGGGCUGCUGGCGAUGGCCGACGCAGAUCCGGAUGCACUCGCGGCGGGGAUGUCCCCGCUGGGGGUGGAUCGCUGGCU
env-33    CCGAGGAUGGGAUGACGGUCGUCUUCGGCGCCCCCGGACGGGGGCUGCUGGCGAUGGCCGACGCAGAUCCGGAUGCACUGUCGGCGGGGAUGUCCCCGCUGGGGGUGGAUCACUGGCU
env-34    CCGAGGAUGGGAUGACGGUCGUCUUCGGCGCCCCCGGACGGGGGCUGCUGGCGAUGGCCAACGCAGAUCCGGAUGCGCUGUCGGCGGGGAUGUCCCCGCUGGCGGUGGAUCACUGGCU
env-35    CCGAGGAUGGGAUGACGGUCGUCUUCGGCGCCCCCGGACGGGGGCUGCUGGCGAUGGCCGACGCAGAUCCAGAUGCACUCACCGCGGGGAUGUCCCCGCUGGGGGUGGAUCACUGGCU
env-36    GCACGGAUGCUGGCGAUGGCGAGGAUGGCGAACGGACCUACACAGUCGCGUUCGGUGCACCCGAGCGGGGCAUUCCGGCGGUUCUGGGCAUCGAGCCUGGAGCGGUUGGCGAAGACGA
env-37    ACGCUGGCAGCGAGGGUUCCGGUGUCGACUGUACCGUCGCCUUUGGCGCGCCUGAGCGCGGUCUGCCGGAGAUAUUGGGCAUUUCGCCCGAGUCGAUACGCGAAGCGGACUCCACCGA
env-38    AUGCUGGCAGCGAGGGUGUGAGUGUCGACCUGACCGUCGCCUUUGGCGCACCCGAGCGCGGCCUGCCGGAGAUUCUGGGCAUUUCGCCCGAGUCGAUACGCGAAGCUGACUCCACCGA
env-39    AUGCUGGCAGCGAGGGUGCGGGUGUCGACCUGACCGUCGCCUUUGGCGCACCCGAGCGCGGCC.......................................................
env-40    ......................................................................................................................
env-41    CGAAUCAAGGGGCAACAGUGGUGUUUGGUUCGCCUGGCCGAGGGCUUCCAGCUAUCCUUGGGGUCGACGUAGCAAGCGACUCUGAGACCACUGUCGAGCCCUCCGACGGUGUCGGGUU
env-43    ......................................................................................................................
env-44    ......................................................................................................................
env-45    CAGGACUUACCGUUGUCUUUGGAUCACCCGGUCGAGGGCUUCCAGAAAUCCUUGGGGUUACCCCCGAGGCCAUUACGGCCGCCGAAGGCGGUCCAGUCGAACCCGAUCCGGGGUUCGA
env-49    AGGGGCUGACCGUCGCGUUCGGCUCGCCGGGACGAGGGCUGCCGGACAUACUCGGGAUCCCACCCGAGGCCAUCUCGGCUGAGCAAUCGGCCGGGGCGUCCGAGCCAGACGGCUUGGA
env-50    AGGGGAGUAUGACCGUCGCCUUCGGUUCGCCCGGUAGGGGCCUUCCGCAAAUGCUCGGGAUCUCACCCGAGGCGGUUUCGGCCGCCAUCGCGGCCGACACUCACGUCGACACCGAUCC
env-54    ......................................................................................................................
env-55    AGGGGAUGACCGUCGCCUUUGGUUCGCCCGGACGAGGGUUGCCGGAAAUACUCGGGAUCGAACCCGAGGCAGUCUCGGCCGCAGUCGCGGCUGGAGACCAUGUUGAAUCCGACCCCG.
env-56    AGGGGAUGACCGUCGCCUUCGGUUCGCCCGGACGAGGGUUGCCGGAAAUACUCGGGAUCGAACCCGAGGC................................................
env-57    AGGGCGCAACUGUUGCGUUCGGCUCGCCUGGCAGAGGGUUGCCGGAAAUACUCGGGAUCGAACCCGAGGCAGU.............................................
env-58    AUGGAAUGACCGUCGCCUUCGGCUCGCCGGGACGGGGACUGCCGGAAAUACUCGGGAUCACUCCCGAGGCAGUCUCGGCCGCAAUCGCGGCUGGAGACCAUGUUGAAUCCGAUCCGGG
env-59    ......................................................................................................................
env-62    AUGGAGCAACCGUCGCCUUCGGCUCGCCCGGCAGAGGGCUUCCGGAAAUACUCGGGAUUGAACCCGAGGCAGUCUCGGCCGCAGUCGCGGCUGGAGACCAUGUCGACUCCGAUCCGGG
env-63    AUGGAGCAACCGUCGCCUUCGGUUCGCCCGGCAGAGGGCUUCCGGAAAUACUCGGGAUUGAACCCGAGGCAGUCUCGGCCGCAGUCGCGGCUGGAGACCACGUUGAAUCCGUUCCUGG
env-64    AUGGAGCAACCGUCGCCUUCGGUUCGCCCGGCAGAGGGCUUCCGGAAAUACUCGGGAUUGAACCCGAGGCAGUCUCGGCCGCAGUCGCGGCU.........................
env-65    AUGGAGCAACCGUCGCCUUCG.................................................................................................
env-70    ......................................................................................................................
env-71    GAUGGAGCAACCGUCGCCUUCGGUUCNCCCGGCAGAGGGCUUCCGGAAAUACUCGGGAUUGAACCCGAGGCAGUCUCGGCCGCAGUCGCGGCUGGAGACCACGUUGAAUC........
env-72    AGCGCGACGGGAUGACCGUCGCGUUCGGUGCGCCCGGGAGAGGGCUGCCGGCCAUCCUCGGCAUCGAGGAAUCUGCCGUCCCGUCGUCGACGAUAGACGACUGGGCCGGCCGCGACGA
env-74    AACGCGACGGUUAUACCGUCGCGUUCGGGGCGCCCGGGAGAGGGCUGCCGGCCAUCCUCGGCAUCGAGGAAUCGGCCGUCCCGUCGUCGUCGGUAGACGACUGGGCCGGCCGCGACGA
env-76    ......................................................................................................................
env-77    ......................................................................................................................
env-78    ......................................................................................................................
Hsa-1-1   AGUCGCGUGGCGAUCUGACCGUCGCGUUCGGUUCACCCGAGCGUGGGUUGCCCGCGAUCAUGGGGAUCGACCCCGAUGACGUCGGGCCGACUGAAAUCGAAGCGGUCACCGGAUCCGA
Hgi-1-1   CCGAUGCCGGCGGCAUGACCGUCGUCUUCGGGUCGCCGGGCCGUGGGCUUCCGGACAUGCUCGGGAUGUCUCCCGAGGAUGUUGCAGACGUCGAACCUUCCGUUGGUCCGGGGUUCGA
Hsu-2-1   CCGAUGCCGGCGGCAUGACCGUCGUCUUCGGGUCGCCGGGCCGUGGGCUUCCGGACAUGCUCGGGAUGCCUCCCGAGGAUGUUGCAGACGUCGAACCUUCCGUUGGUCCGGGGUUCGA
Hpr-1-1   CCGAUGUCGGCGGCAUGACCGUCGUCUUCGGGUCGCCGGGCCGUGGGCUUCCGGACAUGCUCGGGAUGUCUCCCGAGGAUGUUGCAGACGUCGAACCUUCCGUUGGUCCGGGGUUCGA
Hsp-8-1   CCGAUGCCGGCGGCAUGACCGUCGUCUUCGGGUCGCCGGGCCGUGGGCUUCCGGACAUGCUCGGGAUGCCUCCCGAGGAUGUUGCAGACGUCGAACCUUCCGUUGGUCCGGGGUUCGA
Hde-1-1   CCGACGCCGGCGGUAUGACCGUCGUCUUCGGGUCGCCGGGCCGUGGGCUUCCGGACAUGCUCGGGAUGUCUCCCGAGGAUGUUGCAGACGUCGAACCUUCCGUUGGUCCGGGGUUCGA
Hlu-1-1   CCGAUGCCGGCGGCAUGACCGUCGUCUUCGGGUCGCCGGGCCGUGGGCUUCCGGACAUGCUCGGGAUGCCUCCCGAGGAUGUUGCAGACGUCGAACCUUCCGUUGGUCCGGGGUUCGA
Hal-2-1   CCGAUGUCGGCGGCAUGACCGUCGUCUUCGGGUCGCCGGGCCGUGGGCUUCCGGACAUGCUCGGGAUGUCUCCCGAGGAUGUUGCAGACGUCGAACCUUCCGUUGGUCCGGGGUUCGA
Hsp-6-1   CCGAGGCCGGCGGCAUGACCGUCGUCUUCGGGUCGCCGGGCCGUGGGCUUCCGGACAUGCUCGGGAUGUCUCCCGAGGAUGUUGCAGACGUCGAACCUUCCGUUGGUCCGGGGUUCGA
Hme-1-1   CCGAUGCCGGCGGUAUGACCGUCGCCUUCGGUUCACCAGGCCGUGGGCUUCCGGACAUGCUUGGGAUGUCUCCCGAGGAUGUUGCAGACGUCGAACCUUCCGUUGGUCCGGGGUUCGA
Hla-2-1   CCGACGCCGACGGUAUGACCGUCGUGUUCGGUUCGCCGGGCCGUGGGCUUCCGGACAUGCUGGGGAUGACUCCCGAGGAUGUCGCAGACGUCGAACCCUCCGUUGGUCCGGGGUUCGA
Hel-1-1   CCGACGCCGACGGCAUGACCGUCGUGUUCGGUUCGCCGGGCCGUGGGCUUCCGGACAUGCUGGGGAUGACUCCCGAGGAUGUCGCAGACGUCGAACCCUCCGUUGGUCCGGGGUUCGA
Hmu-1-1   CCGAUGCCGGCGGUAUGACCGUCGUCUUCGGUUCUCCCGGCCGUGGGCUUCCGGACAUGCUCGGGAUGUCUCCCGAGGAUGUUGCAGACGUCGAACCUUCCGUUGGUCCGGGGUUCGA
env-79    AUGCUGGCAGCGAGGGUUCCGGUGCCGACCUGACCGUCGCCUUUGGCGCACCCGAGCGCGGCCUGCCGGAGAUUUUGGGCAUUUCGCCCGAGUCGAUACGCGAAGCUGACUCCACCGA
env-80    GCCGAGAGUCGACGACAGGCGGUGAUCUGGCCGAUUCCGCGUUCACAGUCGCCUUUG.............................................................
env-81    ACACAGCGGGUGUGAACACAGUCGAUGGCGAGGCUGACUCGGAGACCGAGCACGCCUAUACAGUCGCGUUCGGUGCACCCGAGCGUGGAUUGCCAGAAAUCCUGGGCACAACGCCCGA
env-82    GCCGUGAGUCGGCGACAGGCGGUGAUCUAGCCGAUUCCGCGUUCACAGUCGCCUUUGGCGCACCAGAGCGCGGUUUGCCGGAGAUUCUCGGAUUCACACCCGAGAAGAUUCGCGAAGA
env-83    GCCGUGAGUCGGCGACAGGCGGUGAUCUAGCCGAUUCCGCGUUCACAGUCGCCUUUGGCGCACCAGAGCGCGGUUUGCCGGAGAUUCUCGGAUUCACACCCGAGAAGAUUCGCGAAGA
Hla-4-1   AGCGGGACGGUCUCACCGUCGCGUUCGGUGCGCCCGAGAGAGGGCUGCCGGCAAUCCUCGGCAUCGAUGCCGACGCCGUACAGAAAGACGGCGUCGCGCGACUCGAUGCCGCGGACGA
Ssp-1-1   AAACCGAGGGGCUCACCCUCGCGCUGGGGGCGCCCGAGCGGGGGCUUCCGGAGAUCCUCGACGUCGACGCCGAUUCGGUCGACGACUCACCGAGUGCGCUUCGUCGCGCGAUCGAGUC
env-84    ......................................................................................................................
env-86    ACGACGACGGGAUGACCGUCGCCUUCGGUGCGCCCGAGAGAGGGCUGCCGGCGAUCCUCGAUAUCGAGGAUGCUGGCGAGGCCCUAUCCGGGUCUCGUUUCGGCGAAGCGGGCGACGG
Hsp-11-1  AGCGGGACGGUCUCACCGUCGCGUUCGGCGCGCCCGAGAGAGGGCUGCCGGCAAUCCUCGGCAUCGACGCCGACACCGUGCGAACGGAUGGUGUCGCGCGACUCGAUGCCGCGGACGA
env-87    GCGACCUCACGGUCGCCUUCGGCGCGCCCGAACGAGGGCUGCCGGCGAUCCUCGGCCUCGCGGUCGAGGACGUGAGCGCGGGCGAGUCCACGGCUGGAUUCGACCGCUGGCUCAACAC
env-88    AUGGAAUGACCGUUGCCUUCGGCUCGCCGGGACGGGGACUGCCGGAAAUACUCGGGAUCACUCCCGAGGCAGUCUCGGCCGCAACCGCGGCUGGAGACCAUGUUGAAUCCGAUCCGGG

          ......................................................................................................................
          ......................................................................................................................
          ••••••••••••••••••••••••••••••••••••••••••••••••••••••••••••••••••••••••••••••••••••••••••••••••••••••••••••••••••••••
```

```
env-89     ......................................................................................................................
Hka-1-1    ACGACGCGGGCCACAUGACAGUGGCCUUCGGCGCUCCGGAGCGCGGGUUGCCAGCGAUAUUCGGAUUGGACCCGGACGACGCCGUCCCAGACGGCACAGGUGACGACGACGCCGGGUU
Hmu-2-1    ACGACGCGGGCCACAUGACAGUGGCCUUCGGCGCUCCGGAGCGCGGGUUGCCAGCGAUAUUCGGAUUGGACCCGGACGACGCCGUCCCAGACGGCACAGGUGACGACGACGCCGGGUU
Hca-3-1    GUGGCGGAGACGAUGGGAACGCAUCCGGUGUCGAGACGGGCGGAUCCGACGAAUCUGACGCAUCCGACGACGAGGCGGCGCCCCUGGGUGACCUGACAGUCGCCUUCGGUGCGCCCGA
Hsa-5-1    AAGACGGCAUGACCGUCGCCUUCGGCGCGCCCGAGAGAGGGCUGCCGGCGAUGCUUGGCAUCGACACCGGGUCGGUGGCGAGUGGCAGCGAGACCGACGAGCCUGCGGGGUUCGAUCG
env-90     ......................................................................................................................
Hag-1-1    AAGACGGCAUGACCGUCGCCUUCGGCGCGCCCGAGCGAGGGCUGCCGGCGAUGCUCGGCAUCGACGAGGCGUCGGUGGCGAGUGCGGACGAGACCGGGUCGGACCCUGCGGGGUUCGA
Hsa-4-1    AAGACGGCAUGACUGUCGCCUUCGGCGCGCCCGAGCGAGGGCUGCCGGCGAUGCUCGGCAUCGACACGGCGUCGGUGGCGAGUGCGGACGAGACCGGGUCGGACCCUGGAGGGUUCGA
env-91     GCGAGACUGUCGACAGCGACGGCCGACCCCCGUACACGGUCGCGUUCGGAGCGCCCGAACGUGGCCUGCCGGCGAUCCGCGGACUCGACCCGGAGGCCGUCCGGGCGGGCUCGCGCGG
env-92     AUGGGCUGACCGUUGCUUUCGGCUCGCCGGGACGAGGGCUGCCGGAGAUACUCGGGGUUGACCCCGAGACAA..............................................
env-93     ......................................................................................................................
env-94     ......................................................................................................................
env-95     AUGGGCUGACCGUUGCUUUCGGCUCG............................................................................................
env-96     AUGGGCUGACGUCGCUUCGGCGCGCGGGCCGCGGGCUGCCGGAGAUACUCGGGAUCCCACCCGAAGGCUAUCUCGGCUGCUGACGCCG..............................
env-98     AGGGGCUGACCGUCGCCUUCGGCGCGCCGGGCCGCGGGCUGCCGGAGAUACUCGGGAUCCCACCCGAGGCAAUCUCGGC.......................................
env-99     GGCUGACCGUCGCCUUCGGCGCGCCGGAACGAGGGCUGCCGGAGAUACUCGGGAUUCCACCCGAGGCUAUCUCGGCUGCUGACGCAGCCGGGGCGUCCAAGCCGUAUGGAUUGGACGA
env-101    GGGGAUGUCCGUCGCCUUCGGUUCGCCCGGCAGAGGGCUGCCGGAAAUACUCGGGAUCGAACCCGAGGCAGUCU............................................
env-102    AGGGCGCAACCGUUGCGUUCGGCUCGCCAGGCAGAGGGUUGCCGGAAAUACUCGGGAUCAAACCCGAGGCAGUCUCAGCCGCAC..................................
env-103    AGGGCGCAACCGUUGCGUUCGGCUCGCCAGGCAGAGGGUUGCCGGAAAUACUCGGGAUCAAACCCGAGGCAGUCUCAGCCGCACUCGCGGCUGGAGACCAUGUUGAAUCCGACCCCGG
env-104    ......................................................................................................................
env-105    AGGGCGCAACCGUUGCGUUCGGCUCGCCAGGCAGAGGGUUGCCGGAAAUACUCGGGAUCAAACCCGAGGCAGUCUCAGCCGCACUCGCGGCUGGAGACCAUGUUGAAUCCGACCCCGG
env-106    AGGGGAUGACCGUUGGCUUUGGUUCGCCCGGACGAGGGUUGCCGGAAAUACUCGGGAUCGAACCCGAGGCAGUCUCGGCCGCAGUCGCGGCUGGGCAGCACGUUGAAUCCACGCCGGG
env-107    ......................................................................................................................
env-108    ......................................................................................................................
env-109    ......................................................................................................................
CHa-1-1    CGCACGUCGCCGUCGCGUUCGGUUCGCCGGGGCGGGGGCUUCCGGCCAUCCUCGGGAUGUCCCCCGAGGAGGUGCCGGUCGAACCCGGAGACGCAGACGACUCGGGGUUCGACCUCUG
Hpa-2-1    ACCGGGAGGGAGCCACCGUCGUCUUCGGUUCGCCAGGCCGCGGGCUUCCGGACAUUCUCGGCAUCAACGCCGAGGAAGUCACAGUCGAACCCUCCGACGGUCCGGGGUUCGACCUCUG
env-110    AACGUGACGGAAUGACCGUCGCCUUCGGCUCGCCCGAAAGAGGGCUGCCAGAUAUCCUCGGUAUCGAGGUGUCGGCCAUCGAGGCGUCGAACGAAGACGCCGGUGGCUCGCCUGCGAC
Nhu-1-1    AACGCGAUACUACCCGCGACGGAUACACAAUCGCGUUCGGUGCGCCCGAGAGAGGGCUGCCUGCCAUCCUCGAACUCGAGGAUACCACCGUCGAGUCGUUGUCGGCAUGGCCAUCGUG
env-113    CCGGCGGCUACACCGUCGCCUUCGGCGCGCCCGAACGGGGGCUUCCGGAGAUGCUCGGGCUCUCGCCCGACGACGUUCGGGCGGCCGUCGCCGACGGUCGCCCGGUCGAACCCGAUCC
env-114    AGGGGCUGACCGUCGCGUUCGGCUCGCCGGGACGAGGGCUGCCGGACAUACUCGGGAUCGCCCCCGAGACAAUCUCGGCUGAGCAAUCGGCCGGGGCGUCCGAGCCAGACGGCUUGGA
env-115    ......................................................................................................................
env-116    AGGGGAUGACCGUCGCCUUCGGUUCGCCCGGACGAGGGUUGCCGGAAAUACUCGGGAUCGAACCCGAGGC................................................
env-117    AGGGGAUGACCGUCGCCUUUGGUUCACCCGGACGAGGGUUGCCGGAAAUACUCGGGAUCGAACCCGAGGCAGUCUCGGCCGCAGUCGCG.............................
env-118    AGGGGAUGACCGUCGCCUUCGGUUCGCCCGGACGAGGGUUGCCGGAAAUACUCGGGAU............................................................
env-119    ......................................................................................................................
env-120    GCGUCGCAGUCGCGUUCGGCGCCCCGGCGCGUGGGCUCCCCGCCAUCUUCGGGAUCGAGGGUGGACCGGUCGACGCCGAUACGCGGUUCGACCGGUGGCUGAACACGGUUCCAGACCA
env-122    GCGUCGCCGUCGCGUUCGGCGCGCCGGCGCGGGGGCUCCCCGCCAUCCUCGGGUACGAGGAAGGCCCGGUCGACCCCGCCGACACGCGGUUCGACCGGUGGCUGAACACGGUUCCGGA
env-123    GCGUCGCCGUCGCGUUCGGCGCGCCGGCGCGGGGGCUCCCCGCCAUCCUCGGGUACGAGGACGGGCCGGUCGACCCCGCGGACACGCGGUUCGACCGGUGGCUGAACACGGUUCCGGA
env-126    AGCGGGACGGCGCGACAGUCGUGUUCGGGUCGCCCGGCCGCGGGCUUCCGGGCCAUUCUCGGCGACGGCGUCGAUGUCGACGCCCGAUCCGAGGUGGUGGU.................
env-127    ......................................................................................................................
env-128    AGCGGGAUGGCGCGACGGUCGCGUUCGGGUCGCC....................................................................................
env-129    GCGACGGCACCACCGUCGUCUUCGGCUCGCCCGCGAGAGGGCUGCCGGAGAUCCUCGGGGUGGACCCCGAGGUGGUCGAGCGGUCGGGAGACGACGUCGAACCCGCUGGGGGGUUCGA
Hna-1-1    GCGACGGUGAUCUGACGGUCGCCUUCGGGGCGCCCGGGCGCGGCCUUCCGGCCAUCCUCGACGUGGCUGUCGAGGAAGUAGCCGCCGGCGGCGGCGAGGGCGUCGAACCCGAUCCGGG
env-130    ACGACGCGGGCGAUCUGACGGUCGCCUUCGGGGCGCCCGGGCGCGGCCUUCCGGCCAUCCUCGACGUGGCUGUCGAGGACGUAGCCGCACGCGGCGGCGAGGGCGUCGAACCCGGUGA
env-131    GGGACGAUGGGGCGACCAUCGUUUUCGGCUCGCCGGGGCGAGGGAUUCCGGAGAUUCUCGGGGUGUCCCCCGAUGAUCUCCCCGUCGAACCCGACUCACCCGGGUUCGACCUCUGGCU
env-134    ......................................................................................................................
env-135    ......................................................................................................................
Nph-1-1    CCGCCAACGGAUUCACGGUCGCCUUCGGCGCCCCCGAACGGGGGCUGCCGGAGAUACUCGGCUGUGAGCCUCGCGAGGAGGCUAUCGGCGAUGAGCCCGAAGCGCGGUUCGACCUUUG
env-136    CCGACGACGGACUCACGGUCGUCUUCGGAGCCCCCGAACGGGGACUGCCGGAGAUACUCGACUGUGAGCCUCGCGCGGAGACUAUCGGCGACGAACCGAAUGCGCGGUUCGACCUUUG
env-137    CCGACGACGGACUCACGGUCGUCUUCGGAGCCCCCGAACGGGGACUGCCGGAGAUACUCGACUGUGAGCCUCGCGCGGAGACUAUCGGCGACGAACCGAAUGCGCGGUUCGACCUUUG
env-138    CUGGCGGCUACACCGUUGCCUUC...............................................................................................
env-139    CCGGCGGCUACGUCGUCGCCGUCGGUGCGCCCGAACGAGGGCUUCCGGACGUUCUCGGGCUCACACCCGAGGACAUCCGCGCGGCCGUCGCCGACGGUCGCACGGUCGAACCCGAUCC
env-140    GCGGAUACACCGUCGCCUUCGGUGCGCCCGGUCGAGGGCUGCCGGAAAUGCUCGGGCUGUCGCCCGAGGAGCUCCGCGCGGCCGUCGCCGACGGUCGCCCGGUCGAACCCGAUCCGGG
env-145    CCGGC.................................................................................................................
env-146    CCGGCGGCUACACCGUCGCUUUCGGCUCACCCGAACGCGGGCUUCCAGAAAUGCUCGGGCUAUCGCCCGACGACAUUCGAGCGGCCGUCGCCGACGGUCGCUCGGUCGAACCCGAUCC
env-147    CCGGCGGGUACACCGUCGCCUUCGGCGCGCCCGAACGAGGGCUUCCGGAGAUGCUCGGGGUAUCACCCGAGACAGUCCAGGCGGCCGUCGCCGACGGCCGCCGU..............
env-148    CCGGCGGAUACACCGUCGCCUUCGGUGCGCCCGAACGAGGGCUUCCGGAGAUGCUCGGGCUGUCGCCCGAGGACAUCCGCACGGCCGUCUCAGACGGCCGCCGAGUCGAACCCGAUCC
Hsp-9-1    CCGGCGGGUACACCGUCGCCUUCGGCGCGCCCGAGCGGGGGCUUCCGGAGAUGCUCGGGCUUUCGCCCGACCGCAUUCGGGCGGCCGUCGCCGACGGCCGCUCGGUCGAACCCGAUCC
Hez-1-1    CCGGCGGUUACACCGUCGCCUUCGGCGCGCCCGAGCGGGGGCUUCCGGAGAUGCUCGGGCUUCCGCCCGACCGCAUUCGGGCGGCCGUCGCCGACGGCCGCUCGGUCGAACCCGAUCC
env-149    ......................................................................................................................
env-150    ......................................................................................................................
env-151    CCGGCGGUUACACCGUCGCCUUCGGCGCACCCGAGCGGGGGCUUCCGGAGAUGCUCGGGCUUUCGCCCGACCGCAUUCGGGCGGCCGUCGCCGACGGCCGCUCGGUCGAACCCGAUCC
Hco-1-1    CCGGCGGUUACACCGUCGCCUUCGGCGCGCCCGAGCGGGGGCUUCCGGAGAUGCUCGGGCUUUCGCCCGACCGCAUUCGGUCGGCCGUCGCCGACGGCCGUCCGGUCGAACCCGAUCC
env-152    CCGGCGGCUAC...........................................................................................................
env-153    CCGGCGGUUACACCGUCGCCUUCGGCGCGCCCGAGCGGGGGCUUCCGGAGAUGCUCGGGCUUCCGCCCGACCGCAUUCGGGCGG..................................
env-154    CCGGCGGUUACACCGUCGCCUUCGGCGCGCCCGAGCGGGGGCUUCCGGAGAUGCUCGGGCUUUCGCCCGACCGCAUUCGGGCGGCCGUCGCCGACGGCCGCUCGGUCGAACCCGAUCC
env-155    CCGGCGGCUACACCGUCGCCUUCGGCGCGCCCGAACGGGGGCUUCCGGAGAUGCUCGGGCUCUCGCCCGACCGCAUUCGGGCGGCCGUCGCCGACGGCCGCCCGGUCGAACCCGAUCC
env-156    CCGGCGGUUACACCGUCGCCUUCGGCGCGCCCGAGCGGGGGCUUCCGGAGAUGCUCGGGCUUCCGCCCGACCGCAUUCGGGCGGCCGUCGCCGACGGCCGCUCGGUCGAACCCGAUCC
Hsp-10-1   CCGGCGGCUACACCGUCGCCUUCGGCGCGCCCGAGCGGGGGCUUCCGGAGAUGCUCGGGCUGUCGCCCGACGACAUUCGGACGGCCGUUGCCGACGGUCGCCCGGUCGAACCCGAUCC
Hli-1-1    CCGGCGGGUACACCGUCGCCUUCGGCUCGCCCGAACGAGGGCUUCCGGAGAUGCUCGGGCUUUCGCCCGACGGCAUUCGGGCGGCCGUUGCCGACGGCCGCUCGGUCGAACCCGAUCC
Hte-1-1    CAGGCGGUUACACCGUCGCCUUCGGCGCGCCCGAGCGGGGGCUUCCGGAGAUGCUCGGGCUCUCGCCCGACCGCAUUCGAGCGGCCGUCGCCGACGGCCGCUCGGUCGAACCCGAUCC
Hko-1-1    CCGGCGGGUACACCGUCGCCUUCGGCUCGCCCGAGCGAGGGCUUCCGGAGAUGCUCGGGCUUUCGCCCGACGGCAUUCGGGCGGCCGUUGCCGACGGCCGCUCGGUCGAACCCGAUCC
Hli-2-1    CCGGCAGUUACACCGUCGCCUUCGGCGCGCCCGAGCGGGGGCUUCCGGAGAUGCUCGGGCUUUCGCCCGACCGCAUUCGGGCGGCCGUCGCCGACGGCCGCUCGGUCGAACCCGAUCC
Hsa-7-1    CCGGCGGGUACACCGUCGCCUUCGGCUCGCCCGAGCGAGGGCUUCCGGAGAUGCUCGGGCUCUCGCCCGACGGCAUUCGGGCGGCUGUCGCCGACGGCCGCUCGGUCGAACCCGAUCC
Hca-2-1    CCGGCGGUUACACCGUCGCCUUCGGGGCGCCCGAGCGGGGGCUUCCGGAGAUGCUCGGGCUUUCGCCCGACCGCAUUCGGGCGGCCGUCGCCGACGGCCGCUCGGUCGAACCCGAUCC
Hsp-7-1    CCGGCGGUUACACCGUCGCCUUCGGGGCGCCCGAGCGGGGGCUUCCGGAGAUGCUCGGGCUUUCGCCCGACCGCAUUCGGGCGGCCGUCGCCGACGGCCGCUCGGUCGAACCCGAUCC
Hha-2-1    CCGGCGGCUACACCGUCGCCUUCGGCUCGCCCGAGCGAGGGCUUCCGGAGAUGCUCGGGCUUUCGCCCGACAGCAUUCGGGCGGCCGCCGCCGACGGCCGCUCGGUCGAACCCGAUCC
Hdi-1-1    CCGGCGGUUACACCGUCGUCUUCGGCGCGCCCGAGCGGGGGCUUCCGGAGAUGCUCGGGCUUUCGCCCGACCGCAUUCGGGCGGCCGUCGCCGACGGCCGCUCGGUCGAACCCGAUCC
Hsa-9-1    CCGGCGGCUACACCGUCGCCUUCGGCUCGCCCGAGCGAGGGCUUCCGGAGAUGCUCGGGCUUUCGCCCGACGGCAUUCGGGCGGCCGUCGCCGACGGCCGCUCGGUCGAACCCGAUCC
Har-2-1    CCGGCGGUUACACCGUCGCCUUCGGCGCGCCCGAGCGGGGGCUUCCGGAGAUGCUCGGGCUUUCGCCCGACCGCAUUCGGGCGGCCGUCGCCGACGGCCGCUCGGUCGAACCCGAUCC
env-157    ......................................................................................................................
env-158    CGGGCGGUUACACCGUCGCCUUUGGCUCGCCCGAACGAGGGCUUCCGGAGAUGCUCGGGCUGUCGCCCGACAGCAUUCGGGCGGCCGUCGCCGACGGCCGCCCGGUCGAACCCGAUCC
env-159    CGGGCGGUUACACCGUCGCCUUUGGCUCGCCCGAACGAGGGCUUCCGGAGAUGCUCGGGCUGUCGCCCGACAGCAUUCGGGCGGCCGUCGCCGACGGCCGCCCGGUCGAACCCGAUCC

           ......................................................................................................................
           ......................................................................................................................
           ......................................................................................................................
```

```
env-160    CCGGCGGGUACACCGUCGCUUUCGGCGCGCCCGAACGGGGGCUUCCGGAGAUGCUCGGGGUAUCACCCGAGACAGUCCAGGCGGCCGUCGCCGACGGCCGCC................
h3A-1-1    CGGACGAUCCGGUCGUCGCCUUCGGCGCGCCCGGCAGAGGGCUUCCGGAGAUCCUCGGGGUCCGCCCCGAGGACGUCACGGCCGCAGCCGGCGGCCCCGUCGAACCCGAUCCGGGGUU
Hai-1-1    CCGGUGGCUACGUCAUCGCCUUCGGUGCGCCCGAACGAGGGCUUCCGGAGAUGCUCGGGCUUUCGCCCGACGGCAUUCGGGCGGCCGUCGCCGACGGCCGUCCGGUCGAACCCGAUCC
env-161    CCGGCGGCUACACCGUCGCCUUCGGCUCGCCCGAACGAGGGCUUCCGGAGAUGCUCGGGCUUUCGCCCGACGGCAUUCGGGCGGCCGUUGCCGACGGCCGCUCGGUCGAACCCGAUCC
Hho-1-1    CCGGCGGUUACACCGUCGCCUUCGGCGCGCCCGAGCGGGGGCUUCCGGAGAUGCUCGGGCUCUCGCCCGACCGCAUUCGGGCGGCCGUCGCCGACGGCCGCUCGGUCGAACCCGAUCC
env-164    CCGGCGGCUACGUCGUCGCCUUCGGUGCGCCCGGUCGAGGGCUGCCGGACAUGCUCGGGCUGUCGCCCGAGGAUCUCCGCGCGGCCGUCGCCGACGGCCGCCCGGUCG..........
env-165    ........................................................................................................................
env-166    ........................................................................................................................
env-167    CUGGCGGCUACACCGUCGCCUUUGGCGCGCCGGAGCGGGGGCUUCCGGAGAUGCUCGGGUGUCAACCCGAGGACGUUCACACGGCCGUCGAAGACGGUCGUCGAGUCGAACCCGAUCC
env-169    CUGGCGGCUACACCGUCGCCUUUGGCGCGCCGGAGCGGGGGCUUCCGGAGAUGCUCGGG.............................................................
env-170    ........................................................................................................................
env-172    ........................................................................................................................
env-173    GCCAACGAGGUCUCACAAUCGCGUUUGGCGCUCCACAGCGGGGACUUCCGGAC...................................................................
Hru-1-1    AGCGCGAGGGCGCGACAGUCGUCUUCGGGUCGCCCGGUCGUGGGCUUCCGGCCAUCCUCGGCGUCGACGUCGAGGAUGCGGUCGAACCCGCAGACGGUCCGGGGUUCGAUCUCUGGCU
env-174    ........................................................................................................................
Hru-2-1    ACCGGGACGGCGCCACCGUCGUCUUCGGCUCGCCGGGCCGCGGGCUUCCGGAGAUCUUGGGGGUAUCCCCCGACGAUCUCCCCGUCGAACCCGACUCGCCCGGGUUCGACCUCUGGCU
env-175    ........................................................................................................................
env-176    CAGACGAUGGGUUCACGGUCGCCUUCGGCGCCCCCGAACGGGGGUUGCCGGAGAUACUCGACUGCGAACCGAGCGCGGAGACCAUCGGCGACGAACCGAACGUGCGGUUCGACCUUUG
env-178    CCGACGACGGGUUCACGGUCGCUUUCGGCGCCCCCGAACGGGGGUUGCCGGAGAUACUCGACUGUGAGCCGGGCGCGGAGGCCAUCGGCGACGAACCGAACGUGCGGUUCGACCUUUG
env-179    CCGAGGACG...............................................................................................................
env-180    ........................................................................................................................
Nmo-1-1    CCGACGGACUCACGGUCGCCUUCGGCGCCCCCGAACGGGGGUUGCCGGAGAUACUCGACUGUGAGCCACGCGCGGAGGCCAUCGGCGACGAACCGAACGCGCGGUUCGACCUUUGGCU
Hha-1-1    GCGGCGACCUGACAGUCGCCUUCGGGGCCCCCGAACGAGGGCUGCCGGCGAUGCUCGGCAUCGACGUCGAGUCGGUGACGAGCGCCGAACCGAACGGCGGGUUCGACCGCUGGCUCAA
env-181    ........................................................................................................................
Hpa-1-1    ACCGCGACGGAAUGACCGUCGCGUUCGGUUCGCCGGGUCGAGGACUGCCGGAGAUAUUCGGGGUCCCCCCCGAAGGUAUCUCCGCGGUCGAAUCCGGCGUACCAGCCCGGUUCGACCU
Hci-1-1    AACGCGACGGCAUGACCGUCGUGUUCGGCUCGCCCGGUCGGGGACUGCCGGAGAUACUCGGGGUUGAACCCGAGCGUAUCUCCGCGGUCGAAUCCGGCGUACCAGCCCGGUUCGACCU
env-182    ........................................................................................................................
env-183    ........................................................................................................................
env-184    ........................................................................................................................
env-185    ........................................................................................................................
Hdj-1-1    AACGGGACGGGAUGACCGUCGCGUUCGGUGCACCCGAGCGAGGGUUGCCGGAUAUCCUCGGAAUCGAGGCGUCAGCCGUCCCCUCGUCCGACGAGGGUGACGCUGACGACGGAGUCGA
Hsa-10-1   AGCGUGACGGUAUGACCGUCGCGUUCGGGGCGCCCGAGAGAGGGCUGCCGGACAUCCUCGGAAUCGAGGCAUCGGCCGUCACCGCGUCGGACGACUCCGACGACGGGGUGGACGAUGA
Nsu-1-1    AUCGUGACGGGCUGACCGUCGCAUUUGGUGCGCCCGAGAGAGGGCUGCCGGAUAUCCUCGGAAUCGAGGCAUCGGCUGUCAACGCGUCGUCGGGACAAGGCGACGCGGCCGAAUCGUC
Nba-1-1    AUCGUGACGGGCUGACUGUCGCGUUCGGUGCGCCCGAGAGAGGGCUGCCGGAUAUCCUCGGAAUCGAGGCAUCAGCUGUCAACGCGUCGUCGGGACAAGGCGACGCGGCCGAAUCGUC
Nin-1-1    GCGACGGACUGACCGUCGCCUUUGGUGCGCCCGAGAGAGGGCUGCCGGAUAUCCUCGGAAUCGAGGCAUCCGCCAUCGACGCUGACCCAUCGGGGCAGCUGGACACCGCGGCGGAUGA
env-186    AAGCCGACGGGAUGACCGUCGCCUUCGGCUCGCCCGAGAGAGGGCUUCCGGAGAUGCUCGACGGGGUAACUGUCGAGCAACGAGCGGACGGGGACCGAGUCCCCGGGAACGGAGACGA
env-187    ........................................................................................................................
env-188    AAGCCGACGGGAUGACCGUCGCCUUCGGCUCGCCCGAGAGAGGGCUUCCGGAGAUGCUCGACGGGGUAACUGUCGAGCAACGAGCGGACGGGGACGCAGUCCCCGGGAACGGAGACGA
env-189    ........................................................................................................................
env-190    ........................................................................................................................
env-191    AACGGGACGGGCUCACCGUCGCCUUCGGUGCCCCCGAACGAGGGUUGCCGGACAUCCUCGACAUCGACGAGGCGGCUGUCGAAGCCGCCGUCGAUGGCGAGGGCGACGCAAACGUCGA
Hla-1-1    AGGACGACGGUCUGACCGUCGCCUUCGGCGCGCCCGAGAGAGGGCUGCCGGAUAUCCUCGGAAUCGAGGCGUCCGCCGUCGCCGUCGAUCCGGAUCCAUCGGAUCGAGGGGAUUCCGA
env-193    ........................................................................................................................
Hac-1-1    ........................................................................................................................
Hac-1-2    ACGAGGACGGACUGACCGUGGCGUUCGGCUCGCCGGAACGCGGGCUGCCGCCGAUGCUCGGCCUCACCGCCGAGGCGGUCAGGGAGUUCGACUCCGCCCAGUCCACCCGCGCACCCGG
env-196    ........................................................................................................................
env-197    ........................................................................................................................
env-198    CCCGGGACGGACUGACAGUCGCCUUCGGUGCACCCGAACGUGGGCUUCCGCCCAUGCUCGGACUCACCGAGGACGAGGUCAGCGAGGCCGUCGCCGACCCCAACUCCGACGCACCCGC
Hsp-2-1    CCCGGGACGGACUGACAGUCGCCUUCGGUGCACCCGAACGUGGGCUUCCGCCCAUGCUCGGACUCACCGAGGACGAGGUCAGCGAGGCCGUCGCCGACCCCAACUCCGACGCACCCGC
env-199    ACGGCGUCACCGUUGCCUUCGGCGCGCCCGAGCGAGGGCUGCCGGCGAUACUCGGUAUCGACCCAGCGCGGGUCGGUGCCGACUCCCCGCCAGGGGACUUCGACCGCUGGCUGAACAC
Hsu-1-1    CCGCCGACGGACUGACCGUCGCCUUCGGAGCGCCGGGUCGUGGCCUCCCCGACAUCUUGGACGUCGACGUCCAGGAAAUCGGGGCCGCGACCGGCUCCACGACGGUCGGCGAGGGUGC
env-201    ........................................................................................................................
env-203    ........................................................................................................................
Hca-1-1    ACGCCGAGGGCGGCAUGACCGUCGCCUUCGGGGCACCCGAGCGCGGGUUACCGUCGAUACUCGACGUUGCCCCGGACGCCGUCGGGGGAGACCAGACGAGCGACGAACCAGAAGGGUU
Ham-1-1    ACGCCGAGGGCGGCAUGACCGUCGCCUUCGGGGCACCCGAGCGCGGGUUACCGUCGAUACUCGACGUUGCCCCGGACGCCGUCGGGGGAGACCAGACGAGCGACGAACCAGAAGGGUU
Hhi-2-1    ACGCCGAGGGCGGCAUGACCGUCGCCUUCGGGGCACCCGAGCGCGGGUUACCGUCGAUACUCGACGUUGCCCCGGACGCCGUCGGGGGAGACCAGACGAGCGACGAACCAGAAGGGUU
Hsa-2-1    ACGCCGAGGGCGGCAUGACCGUCGCCUUCGGGGCACCCGAGCGCGGGUUACCGUCGAUACUCGACGUUGCCCGGACGCGUCGGGGGAGACCAGACGAGCGACGAACCAGAAGGGUUCG
Har-1-1    ACGCCGAGGGCGGCAUGACAGUCGCCUUCGGGGCACCCGAGCGCGGGUUACCGUCGAUACUCGACGUUGCCCCGGACGCCGUCGGGGGAGACCAGACGAGCGACGAACCAGAAGGGUU
Hva-1-1    ACGCCGAGGGCGGCAUGACCGUCGCCUUCGGGGCACCCGAGCGCGGGUUACCGUCGAUACUCGACGUUGCCCCGGACGCCGUCGGGGGAGACCAGACGAGCGACGAACCAGAAGGGUU
Hja-1-1    ACGCCGAGGGCGGCAUGACUGUCGCCUUCGGGGCACCCGAGCGCGGGUUACCGUCGAUACUCGACGUUGCCCCGGACGCCGUCGGGGGAGACCAGACGAGCGACGAACCAGAAGGGUU
env-204    ACGGACUCACCGUCGCCUUCGGCGCGCCACAACGGGGGCUGCCGGCGAUUCUCGGGAUCGAACCCGAGGUCAUUUCGGCCGAACCAUCGGCCGACACGUCCGAGCCAGCCGGUCGGA
Hse-1-1    ACGACGGACUGACCGUCGCCUUCGGCGCGCCCGAGCGAGGGCUGCCGGCCAUGCUCGGCAUCGAGCACGAAUCGGUGUCGAGCGCCGAAUCGAACCACGCGGGGUUCGACUGCUGGCU
Hth-1-1    ACGACGGACUGACCGUCGCCUUCGGCGCGCCCGAGCGAGGGCUGCCGGCGAUGCUCGACAUCGACGCGGCGUCGGUGGCGAGCGACUCACCCCACGAGGGGUUCGACUGCUGGCUCAA
Hmo-1-1    ACGACGGGCUGACCGUCGCCUUCGGCGCGCCCGAGCGAGGGCUGCCGGCGAUGCUCGACAUCGACGCGGCGUCGGUGGCGAGCGAUACACCCCACGCGGGGUUCGACUGCUGGCUCAA
env-206    AAGCCGACGGGAUGACCGUCGCCUUCGGCGCGCCCGAGAGAGGGCUGCCGGAGAUCCUCGAUCUGGCGAUCGAGGCUGUCGGCG....................................
env-207    AACGAGUCGCCGUGGCUUUCGGCGCGCCGGCGCGAGGCUUGCCCGCCAUCCUCGACGGGGUGGCGGACGACGAGCGGAUCGACGCGGCGGAGACGCCGUUCGACCGCUGGUUGAACAC
env-209    CAGGAGGCG...............................................................................................................
Nta-1-1    ACCGCGACGGGAUGACCGUCGCCUUCGGCGCGCCCGAGAGAGGGCUGCCGGACAUCCUCGAGUUCGAAUCCGAGGACGCCGUCGACACGUUGUCGUCCUGGCCGCCGCGGUCAGACGA
Nas-1-1    ACCGCGACGGGAUGACCGUCGCCUUCGGCGCGCCCGAGAGAGGGCUGCCGGACAUCCUCGAGUUCGAAUCCGAGGACGCCGUCGACACACUGUCGUCCUGGCCACCGCGGCCGGACGA
Noc-1-1    AGCGCGACGGGAUGACCGUCGCCUUCGGGGCACCCGAGAGAGGGCUGCCGGCCAUCCUCGGAAUGGACGAGGAGACCGUCGACGCGCUGUCCCGCUGGGACGGCGUCGACGGUGGCGG
Nje-1-1    AGCGCGACGGGAUGACCGUCGCCUUCGGGGCACCCGAGAGAGGGCUGCCGGCAAUCCUCGGAAUGGACGAGGAGGCCGUCGACGCGCUGUCCCGCCGGGACGGCGUCGACGGCGACGG
Nam-1-1    AGCGCGACGGGAUGACCGUCGUCUUCGGGGCACCCGAGAGAGGGCUGCCGGACAUCCUCGGAAUGGACGAGGAGGCCGUCGACGCGCUGUCCAACUGGGACGGCGUCGACGGUGACGG
env-210    AAGACGACGGGAUGACCGUCGCGUUCGGUGCGCCCGAGAGAGGGCUGCCGGCUAUCCUCGGAAUCGAGGCAUCGGCUAUCGAGCCGUCCGGCGAGGACGAGCCGGCCGAUGACGGAGU
Hsp-3-1    CCAGGGACGGCGUGACCGUUGCGUUCGGAGCACCGGAACGUGGGCUGCCGCCCAUGCUUGGUGUUUCGGCGGACGCGGUCAACGAGUCGGUCACCGACUCAUCCGCAGACGCCCCCGC
Nch-1-1    AACGCGAUACUACCCGCGACGGAUACACAAUCGCGUUCGGUGCGCCCGAAAGAGGGCUGCCUGCCAUCCUCGAACUCGAGGAUACCACCGUCGAGUCGUUGUCGGCAUGGCCAUCGUG
Nae-1-1    AGCGCGACGGGAUGACCGUCGCCUUCGGCGCGCCCGAGAGAGGGCUGCCGGACAUCCUCGAGUUCGAAUCCGAGGACGCCGUCGACACGUUGUCGUCCUGGCCGCCGCGGUCAGACGA
env-214    ........................................................................................................................
env-215    AGCGCGACGGGAUGACCGUCGCCUUCGGGGCACCCGAGAGAGGGCUGCCGGACAUCCUCGGAAUGGCCGAGGAGACC...........................................
env-216    AGGACGAGGGA.............................................................................................................
Hni-1-1    AAGCCGACGGGAUGACCGUCGCUUUCGGUGCGCCCGAGAGAGGGCUGCCGGACAUCCUCGGAAUCGAGGCAUCCGACGUUGCGUCGGCCCACGCAGGCGACGGGCCGGAGAGCGCGGA
Has-1-1    AGCGCGACGGCAUGACCGUUGCGUUCGGUGCGCCCGAGAGAGGGCUGCCGGACAUCCUCGGAAUCGAGGAGUCACGUGCCGGCGCGUCGUCCAGCGGGGACGGCGGGGCGGCAGACGG
env-217    ........................................................................................................................
Nal-2-1    AGCGCGACGGGAUGACCGUCGCCUUCGGCGCGCCCGAGAGAGGGCUGCCGGCUAUCCUCGGAAUCGAGGAAUCGGCCAUCGAACCGUCGUCCGGACAAGACGACGCGAUCGAACCGUC
Npa-1-1    AGCGCGACGGGAUGACCGUCGCCUUCGGCGCGCCCGAGAGAGGGCUGCCGGCUAUCCUCGGAAUCGAGGAAUCGGCCAUCGAAGCGUCGUCCGGACAAGACGACGCGAUCGAACCGUC

           ........................................................................................................................
           ........................................................................................................................
           ●●●●●●●●●●●●●●●●●●●●●●●●●●●●●●●●●●●●●●●●●●●●●●●●●●●●●●●●●●●●●●●●●●●●●●●●●●●●●●●●●●●●●●●●●●●●●●●●●●●●●●●●●●●●●●●●●●●●
```

Nga-1-1	AGCGCGACGGGAUGACCGUCGCCUUCGGCGCGCCCGAGAGAGGGCUGCCGGCUAUCCUCGGAAUCGAGGAAUCGGCCAUCGACGCGUCGUCCGGACGAGACGACGCGGUCGACCCGUC
Nsp-2-1	AGCGCGACGGGAUGACCGUCGCCUUCGGCGCGCCCGAGAGAGGGCUGCCGGCUAUCCUCGGAAUCGAGGAAUCGGCCAUCGACGCGUCGUCCGGACGAGACGACGCGGUCGACCCGUC
Htu-1-1	AGGGCGACGGGAUGACCGUCGCCUUCGGCGCGCCCGAGAGAGGGCUGCCUGCUAUCCUCGGAAUCGAGGCAUCGGCCAUCGAAGCGUCGUCGGGACAAGGCGACGCGAUCGAAUCGUC
Hsa-8-1	AGGGUGACGGGAUGACCGUCGCCUUCGGCGCGCCCGAGAGAGGGCUGCCUGCUAUCCUCGGAAUCGAGGCAUCGGCCAUCGAAGCGUCGUCGGGACAAGGCGACGUGAUCGAACCGUC
Hth-2-1	AGGGCGAUGGGAUGACCGUCGCGUUCGGUGCGCCCGAACGAGGGCUGCCGGAUAUCCUCGAAAUCGAGGCAUCCGCCGUCGGGGCCCCGAAGGACGCGGCCGCCGGCGAGGAUGACGG
Nve-1-1	AGCGCGACGGGAUGACCGUUGCCUUCGGCGCGCCCGAGAGAGGGCUGCCGGCUAUCCUCGGAAUCGAGGAAUCGGCCAUCGAAGCGUCGUCCGGACAAGGCGACGCGGCCGAUAACGG
Hli-3-1	AGGACGACGGGAUGACCGUCGCCUUUGGCGCGCCCGAGCGAGGGUUGCCGGAUAUCCUCGGAAUCGAGGCAUCCGCCGUCGGGGCUGUCCAGGAGACAGCCACGGACGGGGAUGACGG
Hxa-1-1	AUCGCGACGGUUUGACCGUCGCCUUCGGAGCGCCCGAGAGAGGGCUGCCGGCCAUCCUCGGAAUCGAGGCGUCCGACGUCGCUGCCGCAUACGGCGGCGACGCGGACGACGGCAACGU
Hgo-1-1	AGGCCGACGGGAUGACCGUCGCCUUCGGCGCGCCCGAGAGAGGGCUGCCGAGCAUCCUCGGAAUCGAGGCGUCGGCCGUCGAGUCGUCGUCCACCGGGGACGACGCCGGACGAUCGUC
env-218	....
env-219	AGCAGGACGGAUACACCGUCACGUUCGGAGCGCCCGAUAGAGGGCUGCCGGAUAUCCUCGAUAUCGAGGCGUCGGCCGUCGCGUCGUCAGCGGACACCAGCGGCGGUGGCGACGACGU
env-220	AGGGACUCACAGUCGCCUUUGGCGCGCCCGAACGCGGUCUACCGGCCAUUCUUGGCGUCGAGGCCGGCGAUCCCGAAGCGGGAUUCGACCUGUGGCUGAACACGGUUCCGAACCAGGG
env-221	AACGAGACGGGAUGACCGUCGCGUUCGGUGCGCCCGAGAGAGGGCUGCCGGCUAUCCUCGGAAUCGAGGAAUCGGCUGUCGAGUCGUCCAGUGAGGACGGGGCAGCCGUCGACGGAGU
Hsa-6-1	ACGACGGAAUGACCGUCGUCUUCGGAUCCCCCGGACGAGGGCUUCCGGACAUCCUCGACGUCGCCGUCGAGGACCUCCCCACCAGUACCGGUGGGGCGAGCGUCGGACCCGACAGCGG
env-222	....
env-223	....
env-224	....
env-225	AUCGGGAGGGAAUGACAGUUGCAUUUGGGGCCCCCGGCAGGGGUUUGCCAACGAUUCUUGGGUUAGACCCCAUAGAAAUACUUGAGCCAGAUAUCCCUGGCCCCAAGUUCGAUUGCUG
env-226	....
env-227	....
env-228	AUCGGGCGGGAGCGACAAUUGCCUUCGGUGCGCCUGGUCGUGGGCUCCCAGUCAUCCUUGGCGAUGACAAUGCGCCGGAUAGCCGAACGCAAACGGGGAACCAAACAGCCUCACCCGU
env-231	CCUCACUCAUCUCCGGUGUGUAUCGGCCGAUAUAGGCGAAUUCAACCGGUAUACCGGUAUGUCGUGGUCAAAUACCACAAAUCCAGGAAAUUAGUGCAGGAUUGAUAGAACCAUGCCA
env-232	....
env-233	CCUCACUCAUCUCCGGUGUGUAUCGGCCGAUAUAGGCGAAUUCAACCGGUAUACCGGUAUGUCGUGGUCAAAUACCACAAAUCCAGGAAAUUAGUGCA....................
env-237	GGACCCGGUGGAAAUCCCCGGAAAAUCCAGAAGAAAGGACACCUGUUACCAGGUAGUUCC..
env-238	AGAAUGGAUACACUCUAAUCUUUGGCUCACCUCUGGGUGGAAUUCCCAGAGAUCACUGGGAGAAAUUUCACAUGGUUGUGAAUUUCAUUCCAGAUCAGGAGACCGAAACUGUGAGGAC
env-240	CAAAUGCGAAGCAAAGGGAUAAGGUUUCCGUGGCGUUCGGCAGCCCCUCGAAAGGGCUGGAUGCUAUCUUACUCGAUGAAGGGCACAAGCUUGAGGAUCAUUCCAGUUACGUGGUGAA
env-241	AGGAAUUGGGCAAGCAAGCGAGGUUAAAGUCAUAUACUGUUGUGGUAUUCGGUUCACCCAAACAGGGUGUGGAGUCCAUAUUAGCAAGAGAGGGUACAGAACUCUCAAAAUAUCAGUG
env-242	GAAAUUCCGGCAUGCACGAUGGAACGAAGGAAUGAUUGAAAAAUGCCGAAAAUAAACAGGCCACGCAGAGGAUCUCUUGCAUUCAGCCCCCGGAAGAGGGCAAAGAGCCCGGUGCCCA
env-243	....
env-244	GUAUGGUGAAGCCGCGGUCGGAAUACUGCACGGAAAGUUCAGAUCUCAUACGAGGAUUGAUAAAAAAUGCCGAAGAUCAACACACCGCGAAAGGGAUCCCUCGCAUUCAGCCCCAG
env-245	CCGUACACANAUUACCCCGGUUUUCCGCGGGAGAGGUUGCGGCAGGUGCAGCACUGAAUUGUCAGAUUAGAGAAGGAUUGAUGAAAAAAUGCCGAAGAUCAACACACCGCGAAAAGGA
env-246	CCGUACACACAUUACCCCGGUUUUCCGCGGGAGAGGUUGCGGCAGGUGCAGCACUGAAUUGUCAGAUUAGAGAAGGAUUGAUGAAAAAAUGCCGAAGAUCAACACACCGCGAAAAGGA
env-247	CGUACACAAAUUACCCCGGUUUUCCGCGGGAGAGGUUGCGGCAGGUGCAGCACUGAAUUGUCAGAUUAGAGAAGGAUUGAUGAAAAAAUGCCGAAGAUCAACACACCGCGAAAAGGAU
env-248	GCCGUACACAACUUACCCCGGUUUUCCGCGGGAGAGGUUGCGGCAGGUGCAGCACUGAAUUGUCAGAUUAGAGAAGGAUUGAUGAAAAAAUGCCGAAGAUCAACACACCGCGAAAAGG
env-249	AUCUCGGAUCCCGGACCG..
env-250	CUCGGAUCCAAAGACCGGUGGAUUAUUUUACCGGUAAAUGACAGAGCCUGGAGGUUCCCCGGUAAAAAUUACCGGGCCCUCCGCAUGUACGGGCACAAUUGUCCCUGCAUCAAAAGAC
env-251	CGGUGCAGAGCGGGCAUACGCCCUUGAUAGAAAGAUUGAAGGAUUGAAGAAUACAUGCCGAAGAUUAACAGGCCACGUUAUGGUUCACUCGCAUUCAGCCCACGGAAACGUGCACGAA
env-252	CACGGUGCAGAAUGGGUAUACGCCCUUGAUAGAAAGACUUGAAGGAUUGAAGAAUACAUGCCGAAGAUUCACAGGCCACGUUCAGGUUCACUCGCAUUCAGCCCGCGGACACGUGCUC
env-253	UUACCCGGUGCAGAAUGGGCGUACGCCCUUGUUAGAAAGACUUGAAGGAUUGAAGAAUACAUGCCGAAGAUUCACAGGCCACGUUCAGGUUCACUCGCAUUCAGCCCACGGAAGCGUG
env-254	....
env-255	CCUGCAACAGGUCUCACAGAGUCUCGGAUGAAUCUCUCAAACGUGUCGCAAUGAGAAUAACCUACACGCAAAGGUUAGGAUGGAUUAACACAUGCCAAAAAUCAACAGACCACGCCGU
env-256	....
env-257	AGGCCGAUCGUGCCGGCAGAUCCGGCGCGAGGUCCGGCGCACGCAUCGCUCUGCAGUAUACCUGCAGUCGAUGCACCACGCUACAAUGCACGAAGGAUUGAUUGAAACAUGCCGAAGA
env-258	AAGGCCGAUCGUGCCGGCAGAUCCGGCGCGAGGUCCGGCGCACGCAUCGCUCUGCAGUAUACCUGCAGUCGAUGCACCACGCUACAAUGCACGAAGGAUUGAUUGAAACAUGCCGAAG
env-259	AGGCCGAUCGUGCCGGCAGAUCCGGCGCGAGGUCCGGCGCACGCAUCGCUCUGCAGUAUACCUGCAGUCGAUGCACCACGCUACAAUGCACGAAGGAUUGAUUGAAACAU........
env-260	AGACCGAUCGUGCCGGCAGAUCCGGCGCGAGGUCCGGCGCACGCAUCGCUCUGCAGUAUACCUGCAGUCGAUGCACCACGCUACAAUGCACGAAGGAUUGAUUGAAACAUGCCGAAGA
env-261	CACCAGAAGAAUCCAUCGUGUCGGGUACCCCGGCACGGGGUUCGGCGCAGUCAUCUCACUACAACGCACGAAGGGUUGAUUGAAACAUGCCGAAUAUACACAGACCACGCAGAGGAUC
env-265	AAAGGGCUUUCGUGCCGGAACAUCCGGCACGGAGCCCGGCGAGUGCAUCGCUCUGCAGCAGGCCUGCGGCCGAUGCAUCACGCUACAACGCACGAAGGGUUGAUUGAAACAUGCCGAA
env-266	CGGAAGCCCGGCGCAGGCAUCGCUCUGCAACAGAGUGGUGCGUCACGCUAUAACCACGAAGGGUUGAUUGAAACAUGCCCAAGAUUAACAGACCACGCAGAGGAUCCCUCGCGUAUAG
env-268	....
env-269	CGGAAGCCCGGCGCAGGCAUCGCUCUGCAACAGAGUGGUGCGUCACGCUAUAACCACGAAGGGUUGAUUGAAACAUGCCCAAGAUUAACAGACCACGCAGAGG...............
env-270	ACGGAAGCCCGGCGCAGGCAUCGCUCUGCAACAGAGUGGUGCGUCACGCUAUAACCACGAAGGGUUGAUUGAAACAUGCCCAAGAUUAACAGACCACGCAGAGGNUCCCUCGCGUAUA
env-271	UGCCUCUCCGGCAGGGAUCAUGCAGUCGGUGCAUCAGACUAGUUGCACGAAGGGUUGAUUGAAACAUGCCGAAGUAUAACAGGCCACGCAGAGGAUCCCUUGCAUACAGUCCAAGGAA
env-272	CUGCCUCUCCGGCAGGGAUCAUGCAGUCGGUGCAUCAGACUAGUUGCACGAAGGGUUGAUUGAAACAUGCCGAAGUAUAACAGGCCACGCAGAGGAUCCCUUGCAUACAGUCCAAGGA
env-274	CUGCCUCUCCGGCAGGGAUCAUGCAGUCGGUGCAUCAGACUAGUUGCACGAAGGGUUGAUUGAAACAUGCCGAAGUAUAACAGGCCACGCAGAGGAUCCCUUGCAUACAGUCCAAGGA
env-275	....
env-277	CUGCCUCUCCGGCGGGGAUCAUGCAGUCGGUGCAUCAGACUAGUUGCACGAAGGGUUGAUUGAAACAUGCCGAAGUAUAACAGGCCACGCAGAGGAUCCCUUGCAUACAGUCCAAGGA
env-278	CCUGUCCGGCAGCGAUUUGGUGCGUUCUGCAGUCCAUGCAGCAGUACGAAUCGCUACGCAGUCGGUGCAUCUCAGACUACAGUUGCACGAAGGGUUGAUUAAAAACAUGCCGAAGUAU
env-292	GGCCCGUCCGGCAGAGGAUUCGGUGCAUCACACUACAGUUGCACGAAGGGUUGAUUGAAACAUGCCGAAGUAUAACAGGCCACGCAGGGGAUCCCUCGCAUACA.............
env-293	GGCCCGUCCGGCAGAGGAUUCGGUGCAUCACACUACAGUUGCACGAAGGGUUGAUUGAAACAUGCCGAAGUAUAACAGGCCACGCAGGGGAUCCCUCGCAUACA.............
env-295	GGCCCGUCCGGCAGAGGAUUCGGUGCAUCACACUACAGUUGCACGAAGGGUUGAUUGAAACAUGCCGAAGUAUAACAGGCCACGCAGGGGAUCCCUCGCAUACAGCCCGAGAAAACGG
env-296	CGGCCCGUCCGGCAGAGGAUUCGGUGCAUCACACUACAGUUGCACGAAGGGUUGAUUGAAACAUGCCGAAGUAUAACAGGCCACGCAGGGGAUCCCUCGCAUACAGCCCGAGAAAACG
Mbo-1-1	GGCCCGUCCGGCAGAGGAUUCGGUGCAUCACACUACAGUUGCACGAAGGGUUGAUUGAAACAUGCCGAAGUAUAACAGGCCACGCAGGGGAUCCCUCGCAUACAGCCCGAGAAAACGG
env-297	CGGUCCGUCCGGCAGAGGAUUCGGUGCAUCACACUACAGUUGCACGAAGGGUUGAUUGAAACAUGCCGAAGUAUAACAGGCCACGCAGGGGAUCCCUCGCAUACAGCCCGAGAAAACG
env-298	GUCCGUCCGGCAGAGG..
env-303	GUCCGUCCGGCAGAGGAUUCGGUGCAUCACACUACAGUUGCACGAAGGGUUGAUUGAAACAUGCCGAAGUAUAACAGGCCACGCAGGGGAUCCCUCGCAUACAGCCCGAGAAAACGGG
env-304	GGNCCGUCCGGCAGAGGAUUCGGUGCAUCACACUACAGUUGCACGAAGGGUUGAUUGAAACAUGCCGAAGUAUAACAGGCCACGCAGGGGAUCCCUCGCAUACAGCCCGAGAAAACGG
env-305	CUGCCUCUCCGGCAGGGAUCAUGCAGUCGGUGCAUCAGACUAGUUGCACGAAGGGUUGAUUGAAACAUGCCGAAGUAUAACAGGCCACGCAGAGGAUCCCUUGCAUACAGUCCAAGGA
env-306	....
Mma-1-1	GAGGGCUUACCGUGCCGGUGCGUCCGGUACGGGGCCCGGCGCACGCAUCGCGCUGCAGCAAGCCUGCAGUCGAUGCCUCACGCUACAACGCACGAAGGGUUGAUUGAAAAAUGCCGAA
Mse-1-1	GAGGGCUUACCAUGCCGGUGCGUCCGGCACGGGGCCCGGCGCACGCAUCGCACUGCAGCGAGCCUGCAGUCGAUGCCUCACGCUAUAACGCACGAAGGGUUGAUUGAAAAAUGCCGAA
Msp-1-1	GAGGCUUAUCGUGCCGGUGCGUCCGGCACGGAGCCCGGCGCACGCAUCGCACUGCAGCGAGCCUGCAGUCGAUGCCUCACGCUACAACGCACGAAGGGUUGAUUGAAAAAUGCCGAAG
env-310	....
env-315	GCCGGCUCGCCCGGCAGAGGAUCUGGCAUAUCAACACUAGUUGCACGAAGGGUUGAUUGAAACAUGCCGAAAUAUAACAGGCCACGCAGAGGAUCCCUCGCGUACAGCCCGAGGAAAC
env-316	GCCGGCUCGCCCGGCAGAGGAUCUGGCAUAUCAACACUAGUUGCACGAAGGGUUGAUUGAAACAUGCCGAAAUAUAACAGGCCACGCAGAGGAUCCCUCGCGUACAGCCCGAGGAAAC
env-317	CUGCCUCUCCGGCAGGGAUCAUGCAGUCGGUGCAUCAGACUAGUUGCACGAAGGGUUGAUUGAAACAUGCCGAAGUAUAACAGGCCACGCAGAGGAUCCCUUGCAUACAGUCCAAGGA
env-318	UAGCCGGCCCGUCCGGCAGAGGAUAUGGCACAUCAACACUAGUUGCACGAAGGGUUGAUUGAAACAUGCCGAAAUAUAACAGGCCACGCAGAGGAUCCCUCGCGUACAGCCCGAGGAA
env-320	UGGCCGGCCCGUCCGGCAGAGGAUCUGGCACAUCAACACUAGUUGCACGAAGGGUUGAUUGAAACAUGCCGAAAUAUAACAGGCCACGCAGAGGAUCCCUCGCGUACAGCCCGAGGAA
env-321	UAGCCGGCCCGUCCGGCAGAGGAUAUGGCACAUCAACACUAGUUGCACGAAGGGUUGAUUGAAACAUGCCGAAAUAUAACAGGCCACGCAGAGGAUCCCUCGCGUACAGCCCGAGGAA
env-322	UNGCCGGCCCGUCCGGCAGAGGAUNUGGCACAUCAACACUAGUUGCACGAAGGGUUGAUUGAAACAUGCCGAAAUAUAACAGGCCACGCAGAGGAUCCCUCGCGUACAGCCCGAGGAA
Mpa-1-1	GCAGAGAAGGAUUGAUUAAGACAUGGCAAAUAUACACAGGCCACGUAGAGGAUCACUUGCAUACAGUCCUCGCAAGCGUGCAAAGAGCCAGGUUCCAAAGUAUCACUCUUGGCCUGCC
env-323	AGAGAA..
env-324	AGCGAAAAAUAUCACACAGUCUCGGAUUUCAUAAAAAAUUCGUGUCGCAUUUGAGAAUAACCUACACGCAAAAAGGAUUGAUUACCACAUGCCAAAAAUCAACAGACCACGCAGAGGC
	....
	....
	....

```
env-325   CGGAAGCGAAGCUAUCACACAAUCUCGGAUUCAUCUCACAAUCGUGUCGCAAUGAGAAUAACCUACACGCAAAAAGGAUUGAUUCACACAUGCCAAAAAUCAACAGACCACGCCGCGG
env-326   CGGAAGCGAAGCUAUCACACAAUCUCGGAUUCAUCUCACAAUCGUGUCGCAAUGAGAAUAACCUA.......................................................
env-331   CCGGAAGCGAAGCUAUCACACAAUCUCGGAUUCAUCUCACAAUCGUGUCGCAAUGAGAAUAACCUACACGCAAAAAGGAUUGAUUCACACAUGCCAAAAAUCAACAGACCACGUCGCG
env-332   CGGAAGCGAAGCUAUCACACAAUCUCGGAUUCAUCUCACAAUCGUGUCGCAAUGAGAAUAACCUACACGCAAAAAGGAUUGAUUCACACAUGCCAAAAAUCAACAGACCACGUCGCGG
Mfo-1-1   CUGCAAAGGUUUCAGAAGAGUCUCGGAUGAAUCUCUCAAACGUGUCGCAAUGAGAACAACCUACACGCAAAAGUCAGGAUGGAUUAAUACAUGCCAAAAAUUAACAGACCACGCCGUG
env-333   GAAUCAUCUUUGAAAAGUUCGUGUCACAAAAAAACAAAAAAACACGAAUGAAAAGGAUUGAUUGACACAUGCCAAAGAUAAACAGACCACGUCGAGGCUCGCUGGCAUUCAGCCCACG
env-334   AUCUUUGAAAAGUUCGUGUCACGCAAAAUUUCGAAAAAAAACACGAAUGAAAAGGAUUGAUUGACACAUGCCAAAGAUAAACAGACCACGCCGAGGCUCGCUGGCAUUCAGCCCACGG
env-335   UCUUUGAAAAGUUCGUGUCACACAAAAUUUACAAAAAAACACGAAUGAAAAGGAUUGAUUGACACAUGCCAAAGAUAAACAGACCACGCCGAGGCUCGCUGGCAUUCAGCCCACGGAA
env-337   CAUCUUUGAAAAGUUCGUGUCACGCAAAAUUUCGAAAAAAAACACGAAUGAAAAGGAUUGAUUGACACAUGCCAAAGAUAAACAGACCACGCCGAGGCUCGCUGGCAUUCAGCCCACG
env-338   AUCAUCUUUGAAAAGUUCGUGUCAUAAAAAAAAAUCAAAAAAAACACGAAUGAAAAGGAUUGAUUGACACAUGCCAAAGAUAAACAGACCACGUCGAGGCUCGCUGGCAUUCAGCCCAC
env-339   AUCAUCUUUGAAAAAUUCGUGUCACCAAAAAAAAAUCAAAAAAACACGAAUGAAAAGGAUUGAUUGACACAUGCCAAAGAUAAACAGACCACGUCGAGGCUCGCUGGCAUUCAGCCCAC
CMe-1-1   UGAGAGCACUUCCGUCUCGGAUUCAUCUCUCUAGUUCGUGUCAAAAAGAUCCAAAACACGAAUGUACAGGAAUGAUUGACACAUGCCAAAGAUAAACAGACCACGCCGAGGCUCGCUG
env-340   ACUAACAGGUAUCACACGGUCUCGGAUUAAUCGUAAUUCGUGUCGAAUUGGGAAUAACCUACACGCAGAAAAAGGAUUGAUUAACACAUGCCAAAAAUAAACAGACCACGCCGUGGUU
env-341   CAAACACAGUUAUCACGCUGUCUCGGAUUAAUCGUAAUUCGUGUCACAAUGGGAAUCACCCACACGCACAAAAAGGAUUGAUUAACACAUGCCAAAAAUAAACAGACCACGCCGUGGU
env-349   CAAAAACACAGUUAUCACACUGUCUCGGAUUAAUCGUAAUUCGUGUCACAAUGGGAAUCACCCACACGCACAAAAAGGAUUGAUUAACACAUGCCAAAAAUAAACAGACCACGCCGUG
env-350   CAACAAACAACUGAUUAUAAAUCUCGGAUUAAUCUAAAAUUCGUGUCGCAAUGGGAACAACCUACACGCAGAAAAAGGAUGGAUUAACACAUGCCAAAAAUAAACCGACCACGCCGUG
env-351   AACAGACGAAGGCAUCGUCUCGGAUUAAUCGUAAUUCGUGUCAAAAUGGGAAUAACCUACACGCAGAAAAAGGAUUGAUUAACACAUGCCAAAAAUAAACAGACCACGCCGUGGUUCU
env-352   CACGAAGUUAUCACACAUCUCGGAUUUAAAAAAAAAAUUCGUGUCGCACUUGAGAAUAACCUACACGCAAAAAGGAUUGAUAAACACAUGCCAAAAAUCAACAGACCACGCCGCGGCU
env-353   UUAACCAGACAAAGUUAUCACACAGUCUCGGAUUAAUCAAAAAUUCGUGUCGCAUUAGGAAUAACCUACACGCAGAAAAAGGAUUGAUUAACACAUGCCAAAAAUUAACAGACCACGC
env-354   CUUAACCAGACAAAGUUAUCACACAGUCUCGGAUUAAUCAAAAAUUCGUGUCGCAUUAGGAAUAACCUACACGCAGAAAAAGGAUUGAUUAACACAUGCCAAAAAUAAACAGACCACG
env-355   UUAACCAGACAAAGUUAUCACACAGUCUCGGAUUAAUCAAAAAUUCGUGUCGCAUAAGGAAUAACCUACACGCAGAAAAAGGAUUGAUUAACACAUGCCAAAAAUUAACAGACCACGC
env-356   ........................................................................................................................
env-357   ........................................................................................................................
env-358   UCUUAACCAGACAACGUUAUCACACGGUCUCGGAUUAAUCAAAAAUUCGUGUCGCAAUGGGAAUAACCUACACGCAGAAAAAGGAUUGAUUAACACAUGCCAAAAAUAAACAGACCAC
env-359   AUCUUAACCAGACAACGUUAUCACACGGUCUCGGAUUAAUCAAAAAUUCGUGUCGCAAUGGGAAUAACCUACACGCAGAAAAAGGAUUGAUUAACACAUGCCAAAAAUAAACAGACCA
env-360   AUCUUAACCAGACAACGUUAUCACACGGUCUCGGAUUAAUCAAAAAUUCGUGUCGCAAUGGGAAUAACCUACACGCAGAAAAAGGAUUGAUUAACACAUGCCAAAAAUAAACAGACCA
env-361   AUCUUAACCAGACAACGUUAUCACACGGUCUCGGAUUAAUCAAAAAUUCGUGUCGCAAUGGGAAUAACCUACACGCAGAAAAAGGAUUGAUUAACACAUGCCAAAAAUAAACAGACCA
env-370   ........................................................................................................................
env-371   UUAACCAGAUACGGUUAUCACACCGUCUCGGAUUAAUCGUAAUUCGUGUCGCAAUGGGAAUAACCUACACGCAGAAAAAGGAUUGAUUAACACAUGCCAAAAAUAAACAGACCAC...
env-372   ........................................................................................................................
env-373   GUGUUGGCAUCAUAAACACAGUUAUCACACUGUCUCGGAUUAAUCGUAAUUCGUGUCAUAAUGGGAAUCACCCACACGCACAAAAAGGAUUGAUUAACACAUGCCAAAAAUAAACAGA
env-374   UUAACCAGACAAAGUUAUCACACGGUCUCGGAUUAAUCGUAUUUCGUGUCGAAAUGGGAAUAACCUACACGCAGAAAAAGGAUUGAUUAACACAUGCCAAAAAUAAACA.........
env-375   ........................................................................................................................
env-376   AUUUUGUUUCAAAAGCUCGGAUUAAUCUCAAAUUCGUGUCGCAAUCAGAAUAACCUA...............................................................
env-377   AACAAUAUUUUGUUUCAAAAGCUCGGAUUAAUCUCAAAUUCGUGUCGCAAUCAGAAUAACCUACACGCAAAUAAAGGAUUGAUUAACACAUGCCAAAAAUUAACAGACCUCGCCGUGG
env-378   ........................................................................................................................
env-379   AACAAUACGUUUCAAAAGCUCGGAUUAAUCUUAAAAAUCGUGUCGCAAUCAGAAUAACCUACACGCUAAAAAAGGAUUGAUUAACACAUGCCAAAAAUUAACAGACCCCGCCGUGGUU
env-380   CGAAAGCGAAGCUAUCACACAAUCUCGGAUUAAUCUCACAAACGUGUCGCAAUGAGAAUAACCUACACGCAAAAAGGAUUGAUUCACACAUGCCAAAAAUCAACAGACCACGCCGCGG
env-381   ........................................................................................................................
env-382   AACCGGAAGCGAAGCUAUCACACAAUCUCGGAUUAAUCUCACAAAACGUGUCGCAAUGAGAAUAACCUACACGCAAAAAGGAUUGAUUCACACAUGCCAAAAAUUAACAGACCACGCC
env-383   CCGAAAGCGAAGCUAUCACACAAUCUCGGAUUAAUCUCACAAACGUGUCGCAAUGAGAAUAACCUACACGCAAAAAGGAUUGAUUCACACAUGCCAAAAAUCAA..............
env-384   GAAGCGAAGCUAUCACACAAUCUCGGAUUAAUCUCAAAAAUCGUGUCGCAAUGAGAAUAACCUACACGCAAAAAGGAUUGAUUCACACAUGCCAAAAAUUAACAGACCACGCCGUGGC
env-385   ........................................................................................................................
env-386   UCACACAAUCUCGGAUUCAUCUCACAAAACGUGUCGCAAUGAGAAUAACCUACACGCAAAAAGGAUUGAUUCACACAUGCCAAAAAUCAACAGACCACGCCGCGGCUCUCUUGCAUUC
env-387   ........................................................................................................................
env-389   UUAACGGAAGCGAAGCUAUCACACAUCUCGGAUUAAUCUUAUCAAAACGUGUCGCAAUGAGAAUAACCUACACGCAAAAAGGAUUGAUUCACACAUGCCAAAAAUCAACAGACCACGC
env-390   AUAAACAA................................................................................................................
env-392   ........................................................................................................................
env-395   ........................................................................................................................
env-399   UCAUUGUUCCAAAAGUCUCGGAUUAAUCUCACAUACGUGUCGCUAUCAGAAUAACCUACACGCAGAAGAGGAUUGAUUUACACAUGCCAAAAAUUAACAGACCACGCCGUGGAUCUCU
env-400   ........................................................................................................................
env-402   UUUUGUUUCAAAAGCUCGGAUUAAUCUCAAAUUCGUGUCGCAAUCAGAAUAACCUA................................................................
env-403   ........................................................................................................................
env-405   ........................................................................................................................
env-406   ........................................................................................................................
env-407   ........................................................................................................................
Mli-2-1   AAAAUUUAGUUUGUAGGACUGAUCGAAAAAUGCCCCAAACAACCAGACCACGCAGGGGAUCCCUUGCAUACAGCCCCCGCAAGCGUGCAAAAAGCCAGGUACCAAAGUACCAGUCAUG
Mpe-1-1   UCGGAAAAAAGUUCAAAGAAUAAACAGGACUGAUUGAUAAAUGCCUGGAACAACCAGACCACGCAGAGGAUCACUUGCAUACAGCCCGCGCAAAAGGGCGAAGAGUCAGGUACCGAAG
Mpa-2-1   CUCGGAAAAAAGUUCAAAGAAUAAACAGGACUGAUUGAUAAAUGCCUGGAACAACCAGACCACGCAGAGGAUCACUUGCAUACAGCCCGCGCAAAAGGGCGAAGAGUCAGGUACCGAA
env-408   GGAAAAAAGUUCAAAGAACAAAUAGGACUGAUUGAUAAAUGCCUGGAAAAACCAGACCACGCAGAGGAUCACUUGCAUACAGCCCGCGCAAAAGGGCAAAGAGUCAGGUACCUAAGUA
Mmo-1-1   CACCCAAAAAUGUCCGAAUUAUCUUUCGAUACCGAAAUUCGCAUAAAUACGGCAUGACGCCCUCUGUGAUGCGAAAAACAACGGAAAAACGAAACAGGAUAUUCGGAAUCUCGGAAAA
env-409   CACCCAAAAAUGUCCGAAGUAUCUUUCGAUACCGAAAUUCGCAUAAAUACGGCAUGACGCCCUCUGUGAUGCGAAAAACAACGGAAAAACGAAACAGGAUAUUCGGAAUCUCGGAAAA
env-410   GGGUGCAGCGC.............................................................................................................
Mco-1-1   GCAAAAUCCGGGAUAAGGUAUCCGUCGCAUUUGGAAGCCCCUCGAAGGGGCUGGAGCAGCUCCUGCACGAUGAAGGCCACAAGCUUGAGGACCAUUCCGAUUGCGUGGUGAACUCCAU
env-413   ........................................................................................................................
env-414   CACAGAUUGGUGAGAGGACAAAGCAGUCCAAUCAUACUGCUGUGGUAUUUGGCUCACCCAGGCAAGGUUUAGAGACCAUACUGGACAAUGAGGGUUUCAAGCUCUCAGACUUCCAUUG
env-415   ........................................................................................................................
env-416   ........................................................................................................................
env-418   CAAACAUCGGUAGUAUGAUCAGACAAUCAUCAUUAACUGCUAUUGUAUUCGGAUCACCCAAACAAGGUGUGGAAUCGAUAUUGGCAAAUGAGGGAACACAACUCUCACAUAUCCAGUG
env-423   AACACGUCAGUGGUUUGAUCAGACAUUCAUCAUCAACUGCUAUUGUAUUCGGGUCACCCAAACAAGGUGUGGAAUCGAUAUUGGCAAAUGAGGGCACACAACUCUCACAAAUUCAGUG
env-425   AACAUGUCAGUGGUUUGAUCAGACAUUCAUCAUUAACUGCUAUUGUAUUCGGGUCACCCAAACAAGGUGUGGAAUCGAUAUUGGCAAAUGAGGGUACUCAACUCUCACAAAUUCAGUG
env-428   UGCAAAAGAUAGGAAAUGCAGGCCAAAGCGACCUUGCGGUGGCGUUCGGCUCCCCUGCACGAGGCGUUGAUGCAUUCUUGAGCCAGAGGCUCAUGGAAAGAUGCUGCAUGAUAAAUAC
env-429   UGCAAAGAUUAGGAAAAGCAGGCCAAAGCGACCUUGCGGUGGCAUUCGGCUCCCCUGCACGAGGCGUUGAUGCAUU............................................
Msh-1-1   UCGCUCUCAAAGGGCACAUGAGCGGCAGGGUCUGUGUGGUGUUCGGCUCCCAGAAGAGAGGCAUCAGAGAGAUGCUAAGUGCCCAUGUGGGCGAUGGAUGGAGGCAGCACUUCGAUGA
env-430   UGGGCCAAUCAGGCAAAAAUCGCGACCUUGCGGUGGCCUUCGGCUCACCCGCAAGGGGUCUGGAUGCGUUCUUGAGUGAAGAAUCAAUGAAGAGGUAUGAAAUGAUCAACACCAUUCC
env-433   CAAAAUUGGGCCACUCAGUUCAGAAGCGCGAUCUUGCGGUGGCCUUCGGCUCACCCGCACGAGGCGUGGAUGCCUUCUUGAGCAAAGAAUCGAUGGACAGGUAUGAAAUGAUCAACAC
env-434   UGGUCCAAUCGGGCUGCAAAUGCGACCUUGCGGUGGCCUUCGGCUCACCCGCACGAGGGCU...........................................................
env-435   ........................................................................................................................
env-436   UGGUCCAAUCGGGCUGCAAAUGCGACCUUGCGGUGGCCUUUGGCUCACCCGCACGAGGGCUAGAUGCGUUCUUGAGUAAAGAAUCAAUGAAGAGGUAUGAAAUGAUCAACACCAUUCC
env-437   ........................................................................................................................
env-439   UGGUCCAAUCGGGCUGCAAAUGCGACCUUGCGGUGGCCUUUGGCUCACCCGCACGAGGGCUAGAUGCGUUCUUGAGUAAAGAAUCAAUGAAGAGGUAUGAAAUGAUCAACACCAUUCC
env-440   ........................................................................................................................
env-441   UGGUCCAAUCGGGCAACAAACGCGACCUUGCGGUGGCCUUUGGCUCACCCGCACGAGGGCUAGAUGCGUUCUUGAGUAAAGAAUCAAUGAAGAGGUAUGAAAUGAUCAACACCAUUCC
env-442   UGGUCCAAUCGGGCAACAAA....................................................................................................

          ........................................................................................................................
          ........................................................................................................................
          ........................................................................................................................
```

```
env-443   CCAACAUGGGACACACCGGCUAUCAGCGCGAUCUUGCGGUCGUCUUCGGCUCUCCCUCACGGGGCGUAGAUGCAUUCUUGAGCAAAGAGUCAAUGGAAAGAUAUGAAAUGAUCAACAC
env-444   GCAGAUCCAGUCAGAAGAGCGAUCUUGCAGUGGUAUUCGGCUCCCCUUCGCAAGGCGUUGAUGCGUUCUUGAGCGAUGAAGUCAAGGACAGGUAUUGCAUGAUUAACACCAUUCCGCA
env-445   .........................................................................................................................
env-446   .........................................................................................................................
env-448   AGCGAUCCAGCAAGCGAGGCCUUGCGGUUGUCUUUGGCUCCCCAUCAAGAGGCGUUGAUGCGUUCUUGAGUCGAGAUAUGUUGGAGAGAUGUUGUAUGAUCAACACCAUUCCGCAUCA
env-449   .........................................................................................................................
env-450   .........................................................................................................................
env-451   UGGGUCAAUUGGGCCAGAAGCGCGAUCUUGUGGUGGUAUUCGGCUCACCCGCACGAGGCGUAGAUGCGUUCUUGAGUUGUGAAGCAAUUGGCAGAUAUGACAUGAUUAACACCAUUCC
env-452   .........................................................................................................................
env-455   UGGGUCAAUUGGACCAGAAGCGCGAUCUUGCGGUGGUAUUCGGCUCACCAGCACGAGGCGUAGAUGCGUUCUUGAGUAAGGAAGCUAUUGGCAGGUAUGACAUGAUUAACACCAUUCC
env-456   UAGGUCGAUUGGGCCAGAAGCGCGAUCUUGCGGUGGUAUUCGGCUCACCCGCACGAGGCGUAGAUGCGUUCUUGAGUUGUGAAGCAAUGAACAGGUACGACAUGAUCAACACCAUUCC
env-460   UGGGUCAACUAGGCCAGAAGCGUGAUCUUGCGGUGGUAUUCGGCUCACCCUCACGAGGCGUAGAUGCGUUCUUGAGCAAGGAAACAAUGGACAGGUAUGACAUGAUUAACACCAUUCC
env-462   UGGGUCAACUAGGCCAGAAGCGUGAUCUUGCGGUGGUAUUCGGCUCACCCUCACGAGGCGUAGAUGCGUUCUUGAGCAAGGAAACAAUGGACAGGUAUGACAUGAUUAACACCAUUCC
env-464   UGGGUCAACUAGGCCAGAAGCGUGAUCUUGCGGUGGUAUUCGGCUCACCCUCACGAGGCGUAGAUGCGUUCUUGAGCAAGGAAACAAUGGACAGGUAUGACAUGAUUAACACCAUUCC
env-465   UAGGUCAAUUAGGCCAGAAGCGUGAUCUUGCGGUGGUAUUCGGCUCACCCUCACGAGGC..............................................................
env-466   .........................................................................................................................
env-467   .........................................................................................................................
env-468   UCCAUGAGGGCCGAAGGAGAAGGGGUCUUGAGGUGGUCUUCGGCUCGCCCGCACGCGGCGUUGAUGCGUUCUUGAGCAGCGAAACGAUGGAAGGAUGUUGCAUGAUUAACACCAUACC
env-469   .........................................................................................................................
env-471   UCCAUGAGGGCCGANGGAGANGGGGUCUUGAGGUGGUCUUCGGCUCGCCCGCACGCGGCGUUGAUGCGUUCUUGAGCAGCGAAACGAUGGAAGGAUGUUGCAUGAUUAACACCAUACC
env-472   UCCAUGAGGGCCGAAGGAGAAGGGGUCUUGAGGUGGUCUUCGGCUCGCCCGCACGCGGCGUUGAUGCGUUCUUGAGCAGCGAAACGAUGGAAGGAUGUUGCAUGAUUAACACCAUACC
env-474   UGCGUCAGGGCCAACGGCGUGGUCUGGAGGUGGUCUUCGGCUCGCCCGCACGCGGCGUUGAUGCGUUCUUGAGCAGCGAAACGAUGGAAGGAUGUUGCGUGAUUAACAC.........
env-475   UCCGCCAGGGCCAAGGGCGAGGUCUGGAACUGGUCUACGGAUCGCCCGCUCGCGGCGUUGAUGCGUUCUUGAGCAGCGAAACGAUGGAAGGAUGUUGCGUGAUUAACACCAUACCGCA
Mha-1-1   UCCGCCAGGGCCAAGGGCGAGGUCUGGAACUGGUCUACGGAUCGCCCGCUCGCGGCGUUGAUGCGUUCUUGAGCAGCGAAACGAUGGAAGGAUGUUGCGUGAUUAACACCAUACCGCA
env-476   UCCGCCAGGGCCAAAGGAGAGGUCUGGAACUGGUCUACGGCUCGCCCGCUCGCGGCGUUGAUGCGUUCUUGAGCAGCGAAACGAUGGAAGGAUGUUGCGUGAUUAACACCAUUCCGCA
env-477   UACGUCAAGGCCGAAAGCAUGGUCUGGAGGUGGUCUUCGGCUCGCCCGCACGCGGCGUUGAUGCAUUCUUGAGCAGCGAAACGAUGGAAGGAUGUUGCGUGAUUAACACCAUACCGCA
env-478   .........................................................................................................................
env-479   CCGUACACUGCAACCUGGUGGCGAUAUUCGGUUCACCAGCUCGGGGCGUCGACCGGUUCUUGAGCGACGAAAUGAUGGAUAGAUGCUGCGUAAUCAACACAAUUCCGCAUCAGGGAAC
env-480   CCGUACACCGCAACCUUGUGGCGAUAUUCGGUUCACCAGCUCGGGGCGUCGACCGGUUCUUGAGCGACGAAAUGAUGGAUAGAUGCUGCGUAAUCAACACAAUUCCGCAUCAGGGAAC
env-481   UGUCCCUUAAGGACAGGGGAGACCUGUGUAUAGUGUUCGGUUCACCAAGGAAAGGACUACUGGAAAUGGUGGAGAGAAAUAUGUUCGACGCAGUGCUGAACACAAUACCAAAUCAAGG
env-482   GUGUGAUAUUUGGCUCUGCCAGAAAAGGAGUAUUUGAUAUGGCAAAGCAGGAAAAAGUCGAACUGAAUGGAACAUGUUGGAAUUUCGUUCCUGAACAGGGGACAGAAAGUGUGAGAAC
env-483   GCUUGACAACCUUCGUCUUUGGCGCUCCA............................................................................................
env-484   .........................................................................................................................
env-485   .........................................................................................................................
env-486   GCUUGACAACCUUCGUCUUUGGCGCUCCAAACCGCGGAGUAGAAGCGCUUUUAAACGAAGCCGGGCUAUCGAUCGAGGAAGCGUCAGACUUUGUCGUUAAUACGAUUCCUGACCAAGG
env-492   GCUUGACAACCUUUGUCUUUGGCGCUCCAAACCGCGGAGUAGAAGCGCUUUUAAACGAAGCCGGGCUAUCGAUCGAGGAAGCGUCAGACUUUGUCGUUAAUACGAUUCCUGACCAAGG
env-495   GCUUGA...................................................................................................................
env-504   .........................................................................................................................
env-505   AUAAGGUAUACUUCGUGUCUCUGAUCCUGCAUCGACUAUCAUUCUGGCAGAACUGCCUGAAUGAUCUGAUACAGACAGAAGGACUGAUUGAAAAAUGCCAAACGUACACAGACCCCGC
env-508   ACUCAUAUCCGGUGUAACAAUCGGCCGAUAUUAGGCGAGUUCAACCGAUUAGUUUCGGUUCCGGCCAAAAAAAUGGCCAACGUUCCAGUCAUUAGUGCAGGAUUGAUUGAACCAUGCC
env-509   UCACUAUCUCCGGUAUACUCUGGCCAAAGAGUAGGCGAAUUGACCCGGAACUCCGGGAAAGGCCACCGCAGGUGGCUCCAGUGAACCGCACGUGAAGGAUUGAUUGAAACAUGCCCAA
Mta-1-1   CACAUCUCCGGUAUGCUCUGGCCGAAGAUAGGCGAAAAAACCGGAAAUACCGGUAAAGGCCACAAGGAGUGGCCUGCGACACAAUGCAAGUGCAGGAUUGAUUGAAACAUGCCCAAGA
env-511   CACAUCUCCGGUAUGCUCUGGCCGAAGAUAGGCGAAAAAACCGGAAAUACCGGUAAAGGCCACAAGGAGUGGCCUGCGACACAAUGCAAGUGCAGGAUUGAUUGAAACAUGCCCAAGA
env-512   CACAUCUCCGGCAUGCUCUGGCCGAAGAUAGGCGAAAAAACCGGAAAUACCGGUAAAGGCCACAAGGAGUGGCCUGCGACACAAUGCAAGUGCAGGAUUGAUUGAAACAUGCCCAAGA
Hbo-1-1   AGCGGGAAGGGGCGACCGUCGUCUUCGGUUCACCCGGUCGUGGGCUUCCAGACAUCCUCGGCAUCGACGCCGAGGAAGUCACAGUCGAACCCUCCGACGGUCCGGGGUUCGACCUCUG
Ngr-1-1   AGGGCGACGGGAUGACCGUCGCCUUCGGCGCGCCCGAGAGAGGGCUGCCGGACAUCCUCGGAAUCGAGGCGUCUGCCAUCUCCUCGAGCGACGAGGGGGGCGACGACGGAGUCGAACC
Hje-1-1   CCGACGGAACGACCGUCGCCUUCGGUUCGCCGGGCCGCGGCCUGCCGGACAUACUGGACGUGAACGCCGAGGCCGUCGGCGACCAGCCGGCGGACGGUGUCGAACCCGGUUCGGACGG
Nma-1-1   AACGCGAUACUACCCGCGACGGAUUCACAAUCGCGUUCGGUGCGCCCGAGAGAGGGCUGCCUGCCAUCCUCGAACUCGAGGAUACCACCGUCGAGUCGUUGUCGGCAUGGCCAUCGUG
Hal-1-1   CCGAUGCCGGCGGCAUGACCGUCGUCUUCGGGUCGCCGGGCCGUGGGCUUCCGGACAUGCUCGGGAUGCCUCCCGAGGAUGUUGCAGACGUCGAACCUUCCGUUGGUCCGGGGUUCGA
Nti-1-1   AACGGGACGGGAUGACCGUCGCGUUCGGGGCGCCCGAACGAGGGCUGCCGGACAUCCUCGACAUCGAGGCUUCCGCUGUCGCCCCCGCGGAUGGAACGGGCGACGCGGACGACGGAGU
Npe-1-1   AGGGCGAUGGGAUGACCGUCGCGUUCGGUGCGCCCGAACGAGGGCUGCCGGAUAUCCUCGAAAUCGAGGCAUCCGCCGUCGGGGCCCCGAAGGACGCGGCCGCCGGCGAGGAUGACGG
Hti-1-1   CGUCGGCUGGCGACAUGACAGUCGCCUUUGGCGCGCCGGAGCGCGGCUUGCCGGCUAUCCUCGGCGUCGAACCGGGUGCUGUCGACCCGUGCACCGUGGACGAGUUGGCGGCAGCCGU
          .........................................................................................................................
          .........................................................................................................................
          .........................................................................................................................
```

3 L13-Bacteroidia

3.1 Taxa

The taxonomy of each organism containing a putative L13-Bacteroidia RNA is listed, with abbreviations identifying each hit (e.g., “Eco-1-1” and “Eco-1-2” might hypothetically represent two distinct RNAs in *E. coli*). The abbreviations will be used to identify each individual L13-Bacteroidia RNA in Sections 3.2 and 3.4:.

**abbrev. of hits**	**taxonomy of species**
Bsp-1-1	Bacteria Bacteroidetes Bacteroidia Bacteroidales Bacteroidaceae *Bacteroides sp.* Ga6A1
Bsp-2-1	Bacteria Bacteroidetes Bacteroidia Bacteroidales Bacteroidaceae *Bacteroides sp.* Ga6A2
Psp-1-1	Bacteria Bacteroidetes Bacteroidia Bacteroidales Prevotellaceae Alloprevotella *Prevotella sp. oral taxon* 473 str. F0040
Pcl-1-1	Bacteria Bacteroidetes Bacteroidia Bacteroidales Prevotellaceae *Paraprevotella clara* YIT 11840
Pxy-1-1	Bacteria Bacteroidetes Bacteroidia Bacteroidales Prevotellaceae *Paraprevotella xylaniphila* YIT 11841
Hse-1-1	Bacteria Bacteroidetes Bacteroidia Bacteroidales Prevotellaceae Prevotella *Hallella seregens* ATCC 51272

Pal-1-1 to Pal-1-2	Bacteria Bacteroidetes Bacteroidia Bacteroidales Prevotellaceae *Prevotella albensis* DSM 11370 = JCM 12258
Pam-1-1	Bacteria Bacteroidetes Bacteroidia Bacteroidales Prevotellaceae *Prevotella amnii* CRIS 21A-A
Pam-2-1	Bacteria Bacteroidetes Bacteroidia Bacteroidales Prevotellaceae *Prevotella amnii* DNF00058
Pam-3-1	Bacteria Bacteroidetes Bacteroidia Bacteroidales Prevotellaceae *Prevotella amnii* DSM 23384
Pam-4-1	Bacteria Bacteroidetes Bacteroidia Bacteroidales Prevotellaceae *Prevotella amnii* DSM 23384 = JCM 14753
Pau-1-1	Bacteria Bacteroidetes Bacteroidia Bacteroidales Prevotellaceae *Prevotella aurantiaca* JCM 15754
Pba-1-1 to Pba-1-2	Bacteria Bacteroidetes Bacteroidia Bacteroidales Prevotellaceae *Prevotella baroniae* DSM 16972 = JCM 13447
Pba-2-1	Bacteria Bacteroidetes Bacteroidia Bacteroidales Prevotellaceae *Prevotella baroniae* F0067
Pbe-1-1	Bacteria Bacteroidetes Bacteroidia Bacteroidales Prevotellaceae *Prevotella bergensis* DSM 17361
Pbi-1-1	Bacteria Bacteroidetes Bacteroidia Bacteroidales Prevotellaceae *Prevotella bivia*
Pbi-2-1	Bacteria Bacteroidetes Bacteroidia Bacteroidales Prevotellaceae *Prevotella bivia* DNF00188
Pbi-3-1	Bacteria Bacteroidetes Bacteroidia Bacteroidales Prevotellaceae *Prevotella bivia* DNF00320
Pbi-4-1	Bacteria Bacteroidetes Bacteroidia Bacteroidales Prevotellaceae *Prevotella bivia* DNF00650
Pbi-5-1	Bacteria Bacteroidetes Bacteroidia Bacteroidales Prevotellaceae *Prevotella bivia* DSM 20514
Pbi-6-1	Bacteria Bacteroidetes Bacteroidia Bacteroidales Prevotellaceae *Prevotella bivia* JCVIHMP010
Pbr-1-1	Bacteria Bacteroidetes Bacteroidia Bacteroidales Prevotellaceae *Prevotella brevis* ATCC 19188
Pbr-2-1	Bacteria Bacteroidetes Bacteroidia Bacteroidales Prevotellaceae *Prevotella brevis* P6B11
Pbr-3-1	Bacteria Bacteroidetes Bacteroidia Bacteroidales Prevotellaceae *Prevotella bryantii* B14
Pbr-4-1	Bacteria Bacteroidetes Bacteroidia Bacteroidales Prevotellaceae *Prevotella bryantii* C21a
Pbu-1-1	Bacteria Bacteroidetes Bacteroidia Bacteroidales Prevotellaceae *Prevotella buccae*
Pbu-2-1	Bacteria Bacteroidetes Bacteroidia Bacteroidales Prevotellaceae *Prevotella buccae* ATCC 33574
Pbu-3-1	Bacteria Bacteroidetes Bacteroidia Bacteroidales Prevotellaceae *Prevotella buccae* D17
Pbu-4-1	Bacteria Bacteroidetes Bacteroidia Bacteroidales Prevotellaceae *Prevotella buccalis* ATCC 35310
Pbu-5-1	Bacteria Bacteroidetes Bacteroidia Bacteroidales Prevotellaceae *Prevotella buccalis* DNF00853
Pbu-6-1	Bacteria Bacteroidetes Bacteroidia Bacteroidales Prevotellaceae *Prevotella buccalis* DNF00985
Pco-1-1	Bacteria Bacteroidetes Bacteroidia Bacteroidales Prevotellaceae *Prevotella conceptionensis* 9403948
Pco-2-1	Bacteria Bacteroidetes Bacteroidia Bacteroidales Prevotellaceae *Prevotella copri* DSM 18205
Pco-3-1 to Pco-3-2	Bacteria Bacteroidetes Bacteroidia Bacteroidales Prevotellaceae *Prevotella corporis* DSM 18810 = JCM 8529
Pde-1-1	Bacteria Bacteroidetes Bacteroidia Bacteroidales Prevotellaceae *Prevotella dentalis* DSM 3688
Pde-2-1	Bacteria Bacteroidetes Bacteroidia Bacteroidales Prevotellaceae *Prevotella dentasini* JCM 15908
Pde-3-1	Bacteria Bacteroidetes Bacteroidia Bacteroidales Prevotellaceae *Prevotella denticola* CRIS 18C-A
Pde-4-1	Bacteria Bacteroidetes Bacteroidia Bacteroidales Prevotellaceae *Prevotella denticola* DNF00960
Pde-5-1 to Pde-5-2	Bacteria Bacteroidetes Bacteroidia Bacteroidales Prevotellaceae *Prevotella denticola* DSM 20614 = JCM 13449
Pde-6-1	Bacteria Bacteroidetes Bacteroidia Bacteroidales Prevotellaceae *Prevotella denticola* F0289
Pdi-1-1	Bacteria Bacteroidetes Bacteroidia Bacteroidales Prevotellaceae *Prevotella disiens* DNF00882
Pdi-2-1	Bacteria Bacteroidetes Bacteroidia Bacteroidales Prevotellaceae *Prevotella disiens* FB035-09AN
Pdi-3-1	Bacteria Bacteroidetes Bacteroidia Bacteroidales Prevotellaceae *Prevotella disiens* JCM 6334 = ATCC 29426
Pen-1-1	Bacteria Bacteroidetes Bacteroidia Bacteroidales Prevotellaceae *Prevotella enoeca* JCM 12259
Pfa-1-1	Bacteria Bacteroidetes Bacteroidia Bacteroidales Prevotellaceae *Prevotella falsenii* DSM 22864 = JCM 15124
Pfu-1-1	Bacteria Bacteroidetes Bacteroidia Bacteroidales Prevotellaceae *Prevotella fusca* JCM 17724
Phi-1-1	Bacteria Bacteroidetes Bacteroidia Bacteroidales Prevotellaceae *Prevotella histicola* F0411
Phi-2-1 to Phi-2-2	Bacteria Bacteroidetes Bacteroidia Bacteroidales Prevotellaceae *Prevotella histicola* JCM 15637 = DNF00424
Pin-1-1	Bacteria Bacteroidetes Bacteroidia Bacteroidales Prevotellaceae *Prevotella intermedia* 17
Pin-2-1	Bacteria Bacteroidetes Bacteroidia Bacteroidales Prevotellaceae *Prevotella intermedia* ATCC 25611 = DSM 20706
Pin-3-1	Bacteria Bacteroidetes Bacteroidia Bacteroidales Prevotellaceae *Prevotella intermedia* ZT
Plo-1-1	Bacteria Bacteroidetes Bacteroidia Bacteroidales Prevotellaceae *Prevotella loescheii* DSM 19665 = JCM 12249 = ATCC 15930
Pma-1-1	Bacteria Bacteroidetes Bacteroidia Bacteroidales Prevotellaceae *Prevotella maculosa* DSM 19339
Pma-2-1	Bacteria Bacteroidetes Bacteroidia Bacteroidales Prevotellaceae *Prevotella maculosa* DSM 19339 = JCM 15638
Pma-3-1	Bacteria Bacteroidetes Bacteroidia Bacteroidales Prevotellaceae *Prevotella maculosa* OT 289
Pma-4-1	Bacteria Bacteroidetes Bacteroidia Bacteroidales Prevotellaceae *Prevotella marshii* DSM 16973
Pma-5-1	Bacteria Bacteroidetes Bacteroidia Bacteroidales Prevotellaceae *Prevotella marshii* DSM 16973 = JCM 13450
Pme-1-1	Bacteria Bacteroidetes Bacteroidia Bacteroidales Prevotellaceae *Prevotella melaninogenica* ATCC 25845
Pme-2-1	Bacteria Bacteroidetes Bacteroidia Bacteroidales Prevotellaceae *Prevotella melaninogenica* D18
Pme-3-1	Bacteria Bacteroidetes Bacteroidia Bacteroidales Prevotellaceae *Prevotella melaninogenica* DNF00666
Pmi-1-1	Bacteria Bacteroidetes Bacteroidia Bacteroidales Prevotellaceae *Prevotella micans* DSM 21469
Pmi-2-1	Bacteria Bacteroidetes Bacteroidia Bacteroidales Prevotellaceae *Prevotella micans* DSM 21469 = JCM 16134
Pmi-3-1	Bacteria Bacteroidetes Bacteroidia Bacteroidales Prevotellaceae *Prevotella micans* F0438
Pmu-1-1	Bacteria Bacteroidetes Bacteroidia Bacteroidales Prevotellaceae *Prevotella multiformis* DSM 16608
Pmu-2-1	Bacteria Bacteroidetes Bacteroidia Bacteroidales Prevotellaceae *Prevotella multisaccharivorax* DSM 17128
Pna-1-1	Bacteria Bacteroidetes Bacteroidia Bacteroidales Prevotellaceae *Prevotella nanceiensis* DSM 19126
Pna-2-1	Bacteria Bacteroidetes Bacteroidia Bacteroidales Prevotellaceae *Prevotella nanceiensis* DSM 19126 = JCM 15639
Pni-1-1	Bacteria Bacteroidetes Bacteroidia Bacteroidales Prevotellaceae *Prevotella nigrescens* ATCC 33563
Pni-2-1	Bacteria Bacteroidetes Bacteroidia Bacteroidales Prevotellaceae *Prevotella nigrescens* CC14M
Pni-3-1	Bacteria Bacteroidetes Bacteroidia Bacteroidales Prevotellaceae *Prevotella nigrescens* F0103
Por-1-1	Bacteria Bacteroidetes Bacteroidia Bacteroidales Prevotellaceae *Prevotella oralis* ATCC 33269

Por-2-1	Bacteria Bacteroidetes Bacteroidia Bacteroidales Prevotellaceae *Prevotella oralis* CC98A
Por-3-1	Bacteria Bacteroidetes Bacteroidia Bacteroidales Prevotellaceae *Prevotella oralis* HGA0225
Por-4-1	Bacteria Bacteroidetes Bacteroidia Bacteroidales Prevotellaceae *Prevotella oris* C735
Por-5-1	Bacteria Bacteroidetes Bacteroidia Bacteroidales Prevotellaceae *Prevotella oris* DSM 18711
Por-6-1	Bacteria Bacteroidetes Bacteroidia Bacteroidales Prevotellaceae *Prevotella oris* DSM 18711 = JCM 12252
Por-7-1	Bacteria Bacteroidetes Bacteroidia Bacteroidales Prevotellaceae *Prevotella oris* F0302
Por-8-1	Bacteria Bacteroidetes Bacteroidia Bacteroidales Prevotellaceae *Prevotella oryzae* DSM 17970
Pou-1-1	Bacteria Bacteroidetes Bacteroidia Bacteroidales Prevotellaceae *Prevotella oulorum* F0390
Pou-2-1	Bacteria Bacteroidetes Bacteroidia Bacteroidales Prevotellaceae *Prevotella oulorum* JCM 14966
Ppa-1-1	Bacteria Bacteroidetes Bacteroidia Bacteroidales Prevotellaceae *Prevotella pallens* ATCC 700821
Ppa-2-1	Bacteria Bacteroidetes Bacteroidia Bacteroidales Prevotellaceae *Prevotella paludivivens* DSM 17968
Ppa-3-1	Bacteria Bacteroidetes Bacteroidia Bacteroidales Prevotellaceae *Prevotella paludivivens* DSM 17968 = JCM 13650
Ppl-1-1	Bacteria Bacteroidetes Bacteroidia Bacteroidales Prevotellaceae *Prevotella pleuritidis* F0068
Ppl-2-1	Bacteria Bacteroidetes Bacteroidia Bacteroidales Prevotellaceae *Prevotella pleuritidis* JCM 14110
Pru-1-1	Bacteria Bacteroidetes Bacteroidia Bacteroidales Prevotellaceae *Prevotella ruminicola* 23
Pru-2-1	Bacteria Bacteroidetes Bacteroidia Bacteroidales Prevotellaceae *Prevotella ruminicola* Ga6B6
Psa-1-1	Bacteria Bacteroidetes Bacteroidia Bacteroidales Prevotellaceae *Prevotella saccharolytica* F0055
Psa-2-1	Bacteria Bacteroidetes Bacteroidia Bacteroidales Prevotellaceae *Prevotella salivae* DSM 15606
Psa-3-1	Bacteria Bacteroidetes Bacteroidia Bacteroidales Prevotellaceae *Prevotella salivae* F0493
Psc-1-1	Bacteria Bacteroidetes Bacteroidia Bacteroidales Prevotellaceae *Prevotella scopos* JCM 17725
Psh-1-1	Bacteria Bacteroidetes Bacteroidia Bacteroidales Prevotellaceae *Prevotella shahii* DSM 15611 = JCM 12083
Psp-2-1	Bacteria Bacteroidetes Bacteroidia Bacteroidales Prevotellaceae *Prevotella sp.* 109
Psp-3-1	Bacteria Bacteroidetes Bacteroidia Bacteroidales Prevotellaceae *Prevotella sp.* AGR2160
Psp-4-1	Bacteria Bacteroidetes Bacteroidia Bacteroidales Prevotellaceae *Prevotella sp.* BV3P1
Psp-5-1	Bacteria Bacteroidetes Bacteroidia Bacteroidales Prevotellaceae *Prevotella sp.* C561
Psp-6-1	Bacteria Bacteroidetes Bacteroidia Bacteroidales Prevotellaceae *Prevotella sp.* F0091
Psp-7-1	Bacteria Bacteroidetes Bacteroidia Bacteroidales Prevotellaceae *Prevotella sp.* FD3004
Psp-8-1	Bacteria Bacteroidetes Bacteroidia Bacteroidales Prevotellaceae *Prevotella sp.* HJM029
Psp-9-1	Bacteria Bacteroidetes Bacteroidia Bacteroidales Prevotellaceae *Prevotella sp.* HUN102
Psp-10-1	Bacteria Bacteroidetes Bacteroidia Bacteroidales Prevotellaceae *Prevotella sp.* ICM33
Psp-11-1	Bacteria Bacteroidetes Bacteroidia Bacteroidales Prevotellaceae *Prevotella sp.* MA2016
Psp-12-1	Bacteria Bacteroidetes Bacteroidia Bacteroidales Prevotellaceae *Prevotella sp.* MSX73
Psp-23-1	Bacteria Bacteroidetes Bacteroidia Bacteroidales Prevotellaceae *Prevotella sp. oral taxon* 299 str. F0039
Psp-24-1	Bacteria Bacteroidetes Bacteroidia Bacteroidales Prevotellaceae *Prevotella sp. oral taxon* 306 str. F0472
Psp-25-1	Bacteria Bacteroidetes Bacteroidia Bacteroidales Prevotellaceae *Prevotella sp. oral taxon* 317 str. F0108
Psp-26-1	Bacteria Bacteroidetes Bacteroidia Bacteroidales Prevotellaceae *Prevotella sp. oral taxon* 472 str. F0295
Psp-13-1	Bacteria Bacteroidetes Bacteroidia Bacteroidales Prevotellaceae *Prevotella sp.* P4-65
Psp-14-1	Bacteria Bacteroidetes Bacteroidia Bacteroidales Prevotellaceae *Prevotella sp.* P4-76
Psp-15-1	Bacteria Bacteroidetes Bacteroidia Bacteroidales Prevotellaceae *Prevotella sp.* P5-119
Psp-16-1	Bacteria Bacteroidetes Bacteroidia Bacteroidales Prevotellaceae *Prevotella sp.* P5-125
Psp-17-1	Bacteria Bacteroidetes Bacteroidia Bacteroidales Prevotellaceae *Prevotella sp.* P5-60
Psp-18-1	Bacteria Bacteroidetes Bacteroidia Bacteroidales Prevotellaceae *Prevotella sp.* P6B1
Psp-19-1	Bacteria Bacteroidetes Bacteroidia Bacteroidales Prevotellaceae *Prevotella sp.* P6B4
Psp-20-1	Bacteria Bacteroidetes Bacteroidia Bacteroidales Prevotellaceae *Prevotella sp.* RM4
Psp-21-1	Bacteria Bacteroidetes Bacteroidia Bacteroidales Prevotellaceae *Prevotella sp.* S7 MS 2
Psp-22-1	Bacteria Bacteroidetes Bacteroidia Bacteroidales Prevotellaceae *Prevotella sp.* S7-1-8
Pst-1-1	Bacteria Bacteroidetes Bacteroidia Bacteroidales Prevotellaceae *Prevotella stercorea* DSM 18206
Pti-1-1	Bacteria Bacteroidetes Bacteroidia Bacteroidales Prevotellaceae *Prevotella timonensis* 4401737 = DSM 22865 = JCM 15640
Pti-2-1	Bacteria Bacteroidetes Bacteroidia Bacteroidales Prevotellaceae *Prevotella timonensis* CRIS 5C-B1
Pti-3-1	Bacteria Bacteroidetes Bacteroidia Bacteroidales Prevotellaceae *Prevotella timonensis* S9-PR14
Pve-1-1	Bacteria Bacteroidetes Bacteroidia Bacteroidales Prevotellaceae *Prevotella veroralis* DSM 19559
Pve-2-1	Bacteria Bacteroidetes Bacteroidia Bacteroidales Prevotellaceae *Prevotella veroralis* DSM 19559 = JCM 6290
Pve-3-1	Bacteria Bacteroidetes Bacteroidia Bacteroidales Prevotellaceae *Prevotella veroralis* F0319
env-1 to env-1259	environmental samples

3.2 Gene contexts

Each L13-Bacteroidia RNA (indicated by "RNA→") is listed. For each hit, the downstream genes predicted to reside in a regulated operon are listed. If the nearest downstream gene is encoding in the opposite strand (and therefore presumed to not be a part of a regulated operon), then that gene is still depicted. Some environmental sequences and some RefSeq entries lack gene annotations, and so no genes are available for such sequences. The direction of each gene is

indicated with an arrow (→), and each predicted conserved domain in the gene is named. Conserved domains associated with more than one L13-Bacteroidia RNA are assigned a color; other domains are gray. Information about these conserved domains is given in Section 3.3. The accession of the sequence containing each L13-Bacteroidia RNA is given in the column named "Seq. accession". Accessions beginning with "NC_", "NS_", "NW_" or "NZ_" are contained in RefSeq. Other accession refer to environmental samples. Nucleotide coordinates are given for the 5′ and 3′ boundaries of each L13-Bacteroidia RNA. If the 5′ coordinate is greater than the 3′ coordinate, the RNA is present on the reverse-complement strand of the containing genomic DNA sequence. Each hit is denoted by an abbreviation (like "Eco-1-1") that refers to a taxonomy given in Section 3.1.

abbrev.	Seq. accession		5′ at	3′ at	genes
env-1	AMCI01008330.1	-	1066	991	RNA→ rplM (PRK09216)Ribosomal_L13 (pfam00572)→ rpsI (PRK00132)Ribosomal_S9 (pfam00380)→
env-2	AUXO012860029.1	-	1571	1495	RNA→ rplM (PRK09216)Ribosomal_L13 (pfam00572)→ rpsI (PRK00132)Ribosomal_S9 (pfam00380)→
env-3	SRS011529_WUGC_scaffold_27015	-	1315	1235	RNA→ rplM (PRK09216)Ribosomal_L13 (pfam00572)→ rpsI (PRK00132)Ribosomal_S9 (pfam00380)→
env-4	RUMENNODE_4362630_1	+	14588	14659	RNA→ rplM (PRK09216)Ribosomal_L13 (pfam00572)→ rplM (PRK09216)Ribosomal_L13 (pfam00572)→ rpsI (PRK00132)Ribosomal_S9 (pfam00380)→
env-5	scaffold15734_5_MH0046	+	243	321	RNA→ rplM (PRK09216)Ribosomal_L13 (pfam00572)→ rpsI (PRK00132)Ribosomal_S9 (pfam00380)→
env-6	SRMUA_GNVGR9Q01D7VOE	-	237	168	RNA→ rplM (PRK09216)RplM (COG0102)→
env-7	AUXO010978888.1	+	545	616	RNA→ rplM (PRK09216)Ribosomal_L13 (pfam00572)→ rpsI (PRK00132)Ribosomal_S9 (pfam00380)→
env-8	SRS048164_C2255498	+	534	617	RNA→
env-9	scaffold6072_39_MH0053	+	450	533	RNA→
env-10	SRS019910_WUGC_scaffold_74502	+	208	291	RNA→ rplM (PRK09216)Ribosomal_L13 (pfam00572)→ rpsI (PRK00132)Ribosomal_S9 (pfam00380)→
env-11	scaffold204458_1_MH0006	-	1372	1304	RNA→ rplM (PRK09216)Ribosomal_L13 (pfam00572)→ rpsI (PRK00132)Ribosomal_S9 (pfam00380)→
env-12	UnmappedStool_Broad_scaffold_711238	-	140	72	RNA→ hypo→
env-13	4491686.3.479386	+	127	205	RNA→
env-14	scaffold15346_2_O2.UC-24	+	302	371	RNA→ rplM (PRK09216)Ribosomal_L13 (pfam00572)→
env-15	HCF12C_103092	-	189	116	RNA→
env-16	AUXO015828536.1	+	340	412	RNA→ rplM (PRK09216)Ribosomal_L13 (pfam00572)→
env-17	DOM022_scaffold34730_2	-	1753	1684	RNA→ rplM (PRK09216)Ribosomal_L13 (pfam00572)→ rpsI (PRK00132)Ribosomal_S9 (pfam00380)→
env-18	scaffold19504_1_MH0036	-	69802	69732	RNA→ rplM (PRK09216)Ribosomal_L13 (pfam00572)→ rpsI (PRK00132)Ribosomal_S9 (pfam00380)→
env-19	scaffold4731_12_MH0057	-	597	527	RNA→ rplM (PRK09216)Ribosomal_L13 (pfam00572)→
env-20	DLM014_scaffold3130_5	-	5881	5811	RNA→ rplM (PRK09216)Ribosomal_L13 (pfam00572)→ rpsI (PRK00132)Ribosomal_S9 (pfam00380)→
Pxy-1-1	NZ_GL883827.1	-	169388	169318	RNA→ rplM (PRK09216)Ribosomal_L13 (pfam00572)→ rpsI (PRK00132)Ribosomal_S9 (pfam00380)→
env-21	SRS048164_WUGC_scaffold_29212	+	2023	2093	RNA→ rplM (PRK09216)Ribosomal_L13 (pfam00572)→
env-22	scaffold5875_2_MH0020	+	21461	21531	RNA→ rplM (PRK09216)Ribosomal_L13 (pfam00572)→ rpsI (PRK00132)Ribosomal_S9 (pfam00380)→
env-23	scaffold18015_8_MH0044	+	3289	3359	RNA→ rplM (PRK09216)Ribosomal_L13 (pfam00572)→ rpsI (PRK00132)Ribosomal_S9 (pfam00380)→
env-24	scaffold39182_1_MH0047	-	395	325	RNA→ rplM (PRK09216)Ribosomal_L13 (pfam00572)→
env-25	scaffold33768_3_MH0073	+	2463	2533	RNA→ rplM (PRK09216)Ribosomal_L13 (pfam00572)→
env-26	scaffold6160_1_V1.UC-19	+	273	343	RNA→ rplM (PRK09216)Ribosomal_L13 (pfam00572)→
env-27	scaffold2340_6_MH0035	+	477	547	RNA→ rplM (PRK09216)Ribosomal_L13 (pfam00572)→
env-28	scaffold54231_2_MH0050	+	1169	1239	RNA→ rplM (PRK09216)Ribosomal_L13 (pfam00572)→
env-29	scaffold58113_4_MH0056	-	1209	1139	RNA→ rplM (PRK09216)Ribosomal_L13 (pfam00572)→ rpsI (PRK00132)Ribosomal_S9 (pfam00380)→
env-30	scaffold2091_1_MH0058	-	156	86	RNA→
env-31	scaffold16985_10_MH0059	-	522	452	RNA→ rplM (PRK09216)Ribosomal_L13 (pfam00572)→
env-32	scaffold41222_2_MH0070	-	522	452	RNA→ rplM (PRK09216)Ribosomal_L13 (pfam00572)→
env-33	scaffold1773_9_MH0082	+	1494	1564	RNA→ rplM (PRK09216)Ribosomal_L13 (pfam00572)→ rpsI (PRK00132)Ribosomal_S9 (pfam00380)→ rpsB (PRK05299)Ribosomal_S2 (pfam00318)→
env-34	scaffold14401_2_O2.UC-12	+	160	230	RNA→ rplM (PRK09216)Ribosomal_L13 (pfam00572)→ rpsI (PRK00132)Ribosomal_S9 (pfam00380)→
env-35	4491401.3_NODE_125	+	6382	6452	RNA→ rplM (PRK09216)Ribosomal_L13 (pfam00572)→ rpsI (PRK00132)Ribosomal_S9 (pfam00380)→
env-36	4491414.3_NODE_4995	+	3654	3724	RNA→ rplM (PRK09216)Ribosomal_L13 (pfam00572)→ rpsI (PRK00132)Ribosomal_S9 (pfam00380)→
env-37	4491415.3_NODE_16304	-	35739	35669	RNA→ rplM (PRK09216)Ribosomal_L13 (pfam00572)→ rpsI (PRK00132)Ribosomal_S9 (pfam00380)→
env-38	4491417.3_NODE_72813	+	28708	28778	RNA→ rplM (PRK09216)Ribosomal_L13 (pfam00572)→ rpsI (PRK00132)Ribosomal_S9 (pfam00380)→
env-39	4491419.3_NODE_42377	-	198572	198502	RNA→ rplM (PRK09216)Ribosomal_L13 (pfam00572)→ rpsI (PRK00132)Ribosomal_S9 (pfam00380)→
env-40	4491477.3_NODE_70256	-	8117	8047	RNA→ rplM (PRK09216)Ribosomal_L13 (pfam00572)→

env-41	DLM006_scaffold552_72	-	772	702	**RNA**→ rplM (PRK09216)Ribosomal_L13 (pfam00572)→ rpsI (PRK00132)Ribosomal_S9 (pfam00380)→
env-42	DLM028_scaffold52693_6	+	193	263	**RNA**→ rplM (PRK09216)Ribosomal_L13 (pfam00572)→ rpsI (PRK00132)Ribosomal_S9 (pfam00380)→
env-43	DOM010_scaffold9237_5	+	12401	12471	**RNA**→ rplM (PRK09216)Ribosomal_L13 (pfam00572)→ rpsI (PRK00132)Ribosomal_S9 (pfam00380)→
env-44	NLM008_scaffold4989_13	+	788	858	**RNA**→ rplM (PRK09216)Ribosomal_L13 (pfam00572)→
env-45	SRS011529_WUGC_scaffold_21913	+	1369	1439	**RNA**→ rplM (PRK09216)Ribosomal_L13 (pfam00572)→ rpsI (PRK00132)Ribosomal_S9 (pfam00380)→
env-46	SRS013521_LANL_scaffold_6003	-	8813	8743	**RNA**→ rplM (PRK09216)Ribosomal_L13 (pfam00572)→ rpsI (PRK00132)Ribosomal_S9 (pfam00380)→
env-47	SRS013687_C2637478	-	685	615	**RNA**→ rplM (PRK09216)Ribosomal_L13 (pfam00572)→
env-48	SRS013800_Baylor_scaffold_15627	+	1409	1479	**RNA**→ rplM (PRK09216)Ribosomal_L13 (pfam00572)→
env-49	SRS015133_WUGC_scaffold_36455	-	685	615	**RNA**→ rplM (PRK09216)Ribosomal_L13 (pfam00572)→
env-50	SRS015782_WUGC_scaffold_38346	+	1363	1433	**RNA**→ rplM (PRK09216)Ribosomal_L13 (pfam00572)→
env-51	SRS017307_Baylor_scaffold_15330	-	1112	1042	**RNA**→ rplM (PRK09216)Ribosomal_L13 (pfam00572)→ rpsI (PRK00132)Ribosomal_S9 (pfam00380)→
env-52	SRS017433_Baylor_scaffold_19937	-	547	477	**RNA**→ rplM (PRK09216)Ribosomal_L13 (pfam00572)→
env-53	SRS017701_Baylor_scaffold_13140	-	970	900	**RNA**→ rplM (PRK09216)Ribosomal_L13 (pfam00572)→ rpsI (PRK00132)Ribosomal_S9 (pfam00380)→
env-54	SRS023914_C2116633	-	685	615	**RNA**→ rplM (PRK09216)Ribosomal_L13 (pfam00572)→
env-55	SRS024435_LANL_scaffold_1171	-	1412	1342	**RNA**→ rplM (PRK09216)Ribosomal_L13 (pfam00572)→ rpsI (PRK00132)Ribosomal_S9 (pfam00380)→
env-56	SRS045004_LANL_scaffold_14319	+	1359	1429	**RNA**→ rplM (PRK09216)Ribosomal_L13 (pfam00572)→ rpsI (PRK00132)Ribosomal_S9 (pfam00380)→
env-57	SRS045645_C2019336	+	871	941	**RNA**→
env-58	SRS047044_C1551280	-	547	477	**RNA**→ rplM (PRK09216)Ribosomal_L13 (pfam00572)→
env-59	SRS049712_C15572285	+	896	966	**RNA**→ rplM (PRK09216)Ribosomal_L13 (pfam00572)→
env-60	SRS049995_LANL_scaffold_2946	-	33940	33870	**RNA**→ rplM (PRK09216)Ribosomal_L13 (pfam00572)→ rpsI (PRK00132)Ribosomal_S9 (pfam00380)→
env-61	SRS063985_LANL_scaffold_39061	-	685	615	**RNA**→ rplM (PRK09216)Ribosomal_L13 (pfam00572)→
env-62	SRS078176_C2299272	-	333	263	**RNA**→ rplM (PRK09216)Ribosomal_L13 (pfam00572)→
Pcl-1-1	NZ_JH376625.1	+	66716	66786	**RNA**→ rplM (PRK09216)Ribosomal_L13 (pfam00572)→ rpsI (PRK00132)Ribosomal_S9 (pfam00380)→
env-63	scaffold5721_2_MH0077	-	792	722	**RNA**→ rplM (PRK09216)Ribosomal_L13 (pfam00572)→ rpsI (PRK00132)Ribosomal_S9 (pfam00380)→
env-64	scaffold32997_1_MH0002	-	109	39	**RNA**→
env-65	scaffold77706_1_MH0069	-	138	68	**RNA**→
env-66	NLM006_scaffold34944_6	+	1503	1573	**RNA**→ rplM (PRK09216)Ribosomal_L13 (pfam00572)→ rpsI (PRK00132)Ribosomal_S9 (pfam00380)→
env-67	NOF008_scaffold67812_2	-	140	70	**RNA**→
env-68	NOF010_scaffold32157_6	+	458	528	**RNA**→ rplM (PRK09216)Ribosomal_L13 (pfam00572)→ rpsI (PRK00132)Ribosomal_S9 (pfam00380)→
env-69	SRS017821_Baylor_scaffold_12585	+	5448	5518	**RNA**→ rplM (PRK09216)Ribosomal_L13 (pfam00572)→ rpsI (PRK00132)Ribosomal_S9 (pfam00380)→
env-70	AUXO011504091.1	-	895	820	**RNA**→ rplM (PRK09216)Ribosomal_L13 (pfam00572)→
env-71	AUXO012576065.1	+	558	627	**RNA**→ rplM (PRK09216)Ribosomal_L13 (pfam00572)→ rpsI (PRK00132)Ribosomal_S9 (pfam00380)→
env-72	AUXO017805936.1	+	175	245	**RNA**→ rplM (PRK09216)Ribosomal_L13 (pfam00572)→
env-73	AUXO015702136.1	-	1049	974	**RNA**→ rplM (PRK09216)Ribosomal_L13 (pfam00572)→ rpsI (PRK00132)Ribosomal_S9 (pfam00380)→
env-74	AUXO010123457.1	+	71	143	**RNA**→ rplM (PRK09216)Ribosomal_L13 (pfam00572)→ rpsI (PRK00132)Ribosomal_S9 (pfam00380)→
env-75	CAM_READ_0099979911	+	161	230	**RNA**→ rplM (PRK09216)Ribosomal_L13 (pfam00572)→
env-76	NOM008_scaffold29131_6	-	3871	3801	**RNA**→ rplM (PRK09216)Ribosomal_L13 (pfam00572)→ rpsI (PRK00132)Ribosomal_S9 (pfam00380)→
env-77	AUXO013980254.1	-	161	92	**RNA**→ hypo→
env-78	AUXO014577836.1	-	989	916	**RNA**→ rplM (PRK09216)Ribosomal_L13 (pfam00572)→ rpsI (PRK00132)Ribosomal_S9 (pfam00380)→
env-79	2013414608	-	600	529	**RNA**→ rplM (PRK09216)Ribosomal_L13 (pfam00572)→ rplM (PRK09216)Ribosomal_L13 (pfam00572)→
env-80	pre-rum113-01766	+	19308	19380	**RNA**→ rplM (PRK09216)Ribosomal_L13 (pfam00572)→ rpsI (PRK00132)Ribosomal_S9 (pfam00380)→
Pbr-3-1	NZ_ADWO01000013.1	-	1784	1707	**RNA**→ rplM (PRK09216)Ribosomal_L13 (pfam00572)→ rplM (PRK09216)Ribosomal_L13 (pfam00572)→ rpsI (PRK00132)Ribosomal_S9 (pfam00380)→ rpsI (PRK00132)Ribosomal_S9 (pfam00380)→
Pbr-4-1	NZ_AUKF01000007.1	-	102040	101963	**RNA**→ rplM (PRK09216)Ribosomal_L13 (pfam00572)→ rpsI (PRK00132)Ribosomal_S9 (pfam00380)→
env-81	AUXO013781889.1	+	110	187	**RNA**→ rplM (PRK09216)Ribosomal_L13 (pfam00572)→ rpsI (PRK00132)Ribosomal_S9 (pfam00380)→
env-82	AUXO016535493.1	+	268	342	**RNA**→ rplM (PRK09216)Ribosomal_L13 (pfam00572)→ rpsI (PRK00132)Ribosomal_S9 (pfam00380)→
env-83	AUXO012398056.1	+	1683	1756	**RNA**→ rplM (PRK09216)Ribosomal_L13 (pfam00572)→ rpsI (PRK00132)Ribosomal_S9 (pfam00380)→
env-84	scaffold11907_5_MH0040	-	2845	2774	**RNA**→ rplM (PRK09216)Ribosomal_L13 (pfam00572)→ rpsI (PRK00132)Ribosomal_S9 (pfam00380)→
env-85	4491405.3_NODE_26186	+	3884	3955	**RNA**→ rplM (PRK09216)Ribosomal_L13 (pfam00572)→ rpsI (PRK00132)Ribosomal_S9 (pfam00380)→
env-86	scaffold25515_12_MH0059	+	284	355	**RNA**→ rplM (PRK09216)Ribosomal_L13 (pfam00572)→ rpsI (PRK00132)Ribosomal_S9 (pfam00380)→
env-87	AUXO015466288.1	+	1512	1584	**RNA**→ rplM (PRK09216)Ribosomal_L13 (pfam00572)→ rpsI (PRK00132)Ribosomal_S9 (pfam00380)→
Pal-1-1	NZ_AUFP01000001.1	-	13559	13481	**RNA**→ rplM (PRK09216)Ribosomal_L13 (pfam00572)→ rpsI (PRK00132)Ribosomal_S9 (pfam00380)→

Pal-1-2	NZ_BAJD01000011.1	+	60623	60701	**RNA**→ rplM (PRK09216)Ribosomal_L13 (pfam00572)→ rpsI (PRK00132)Ribosomal_S9 (pfam00380)→
env-88	SRS062878_C3036997	-	256	183	**RNA**→ hypo→ rplM (PRK09216)RplM (COG0102)→
env-89	Tongue_dor_WUGI_scaffold_10412	+	328	401	**RNA**→ rplM (PRK09216)Ribosomal_L13 (pfam00572)→ rpsI (PRK00132)Ribosomal_S9 (pfam00380)→
env-90	SRS014470_WUGC_scaffold_46671	-	1515	1442	**RNA**→ rplM (PRK09216)Ribosomal_L13 (pfam00572)→ rpsI (PRK00132)Ribosomal_S9 (pfam00380)→
env-91	SRS018439_C2372430	+	460	533	**RNA**→ rplM (PRK09216)Ribosomal_L13 (pfam00572)→
env-92	SRS019607_WUGC_scaffold_53821	-	2535	2462	**RNA**→ rplM (PRK09216)Ribosomal_L13 (pfam00572)→ rpsI (PRK00132)Ribosomal_S9 (pfam00380)→
env-93	SRS019894_C3844330	-	310	237	**RNA**→ rplM (PRK09216)Ribosomal_L13 (cd00392)→
env-94	SRS047219_C2319273	+	489	562	**RNA**→ rplM (PRK09216)Ribosomal_L13 (pfam00572)→ rpsI (PRK00132)Ribosomal_S9 (pfam00380)→
env-95	SRS057539_LANL_scaffold_61402	-	1835	1762	**RNA**→ rplM (PRK09216)Ribosomal_L13 (pfam00572)→ rpsI (PRK00132)Ribosomal_S9 (pfam00380)→
env-96	SRS057791_C3471145	+	135	208	**RNA**→
env-97	SRS062544_LANL_scaffold_41679	-	1006	933	**RNA**→ rplM (PRK09216)Ribosomal_L13 (pfam00572)→ rpsI (PRK00132)Ribosomal_S9 (pfam00380)→
env-98	Throat_LANL_C5730482	+	257	330	**RNA**→
env-99	SRS011306_Baylor_scaffold_83447	+	6688	6761	**RNA**→ rplM (PRK09216)Ribosomal_L13 (pfam00572)→ rpsI (PRK00132)Ribosomal_S9 (pfam00380)→
env-100	SRS015038_C1882965	-	403	330	**RNA**→ rplM (PRK09216)Ribosomal_L13 (pfam00572)→
env-101	SRS015762_C3563104	-	158	85	**RNA**→
env-102	SRS015893_WUGC_scaffold_41335	-	2217	2144	**RNA**→ rplM (PRK09216)Ribosomal_L13 (pfam00572)→ rpsI (PRK00132)Ribosomal_S9 (pfam00380)→
env-103	SRS015941_C3475421	+	479	552	**RNA**→ rplM (PRK09216)→
env-104	SRS016002_WUGC_scaffold_14080	-	451	378	**RNA**→ rplM (PRK09216)Ribosomal_L13 (pfam00572)→
env-105	SRS018357_Baylor_scaffold_26234	+	880	953	**RNA**→ hypo→
env-106	SRS018591_WUGC_scaffold_33810	-	202	129	**RNA**→ hypo→
env-107	SRS018739_C3556239	+	325	398	**RNA**→
env-108	SRS019022_C2654036	-	1005	932	**RNA**→ rplM (PRK09216)Ribosomal_L13 (pfam00572)→ rpsI (PRK00132)Ribosomal_S9 (pfam00380)→
env-109	SRS019026_C1997533	-	401	328	**RNA**→ rplM (PRK09216)Ribosomal_L13 (pfam00572)→
env-110	SRS019027_WUGC_scaffold_18825	+	340	413	**RNA**→ rplM (PRK09216)Ribosomal_L13 (pfam00572)→
env-111	SRS019045_WUGC_scaffold_59681	-	2127	2054	**RNA**→ rplM (PRK09216)Ribosomal_L13 (pfam00572)→ rpsI (PRK00132)Ribosomal_S9 (pfam00380)→
env-112	SRS019974_Baylor_scaffold_58098	+	480	553	**RNA**→ rplM (PRK09216)Ribosomal_L13 (pfam00572)→ rpsI (PRK00132)Ribosomal_S9 (pfam00380)→
env-113	SRS020856_C2589008	+	484	557	**RNA**→
env-114	SRS022621_C4094963	-	158	85	**RNA**→ hypo→
env-115	SRS023352_C4653467	-	740	667	**RNA**→ rplM (PRK09216)Ribosomal_L13 (pfam00572)→ rpsI (PRK00132)Ribosomal_S9 (pfam00380)→
env-116	SRS024138_C2697359	+	474	547	**RNA**→
env-117	SRS024277_LANL_scaffold_45644	+	325	398	**RNA**→ rplM (PRK09216)Ribosomal_L13 (pfam00572)→ rpsI (PRK00132)Ribosomal_S9 (pfam00380)→
env-118	SRS024580_C3091241	-	952	879	**RNA**→ rplM (PRK09216)Ribosomal_L13 (pfam00572)→ rpsI (PRK00132)Ribosomal_S9 (pfam00380)→
env-119	SRS047824_WUGC_scaffold_40341	-	1220	1147	**RNA**→ rplM (PRK09216)Ribosomal_L13 (pfam00572)→ rpsI (PRK00132)Ribosomal_S9 (pfam00380)→
env-120	SRS048411_C6958491	-	407	334	**RNA**→ rplM (PRK09216)Ribosomal_L13 (pfam00572)→
env-121	SRS048791_C3141610	+	191	264	**RNA**→ rplM (PRK09216)RplM (COG0102)→
env-122	SRS053603_LANL_scaffold_55573	+	1162	1235	**RNA**→ rplM (PRK09216)Ribosomal_L13 (pfam00572)→ rpsI (PRK00132)Ribosomal_S9 (pfam00380)→
env-123	SRS058336_C2151893	+	935	1008	**RNA**→
env-124	SRS064423_LANL_scaffold_69482	-	3254	3181	**RNA**→ rplM (PRK09216)Ribosomal_L13 (pfam00572)→ rpsI (PRK00132)Ribosomal_S9 (pfam00380)→
env-125	SRS065278_C2565433	-	3238	3165	**RNA**→ rplM (PRK09216)Ribosomal_L13 (pfam00572)→ rpsI (PRK00132)Ribosomal_S9 (pfam00380)→
env-126	SRS016740_C2737901	-	1286	1213	**RNA**→ hypo→ rplM (PRK09216)Ribosomal_L13 (pfam00572)→ rpsI (PRK00132)Ribosomal_S9 (pfam00380)→
env-127	SRS047210_C3305333	-	1003	930	**RNA**→ rplM (PRK09216)Ribosomal_L13 (pfam00572)→ rpsI (PRK00132)Ribosomal_S9 (pfam00380)→
env-128	SRS064774_C2715190	+	51	124	**RNA**→ hypo→ rplM (PRK09216)Ribosomal_L13 (pfam00572)→
env-129	SRS014692_C1518495	-	385	312	**RNA**→ rplM (PRK09216)Ribosomal_L13 (pfam00572)→
env-130	SRS014684_WUGC_scaffold_43074	+	2486	2559	**RNA**→ rplM (PRK09216)Ribosomal_L13 (pfam00572)→ rpsI (PRK00132)Ribosomal_S9 (pfam00380)→
env-131	SRS014689_WUGC_scaffold_12190	+	2627	2700	**RNA**→ rplM (PRK09216)Ribosomal_L13 (pfam00572)→ rpsI (PRK00132)Ribosomal_S9 (pfam00380)→
env-132	Supragingival_plaque_LANL_scaffold_252697	-	158	85	**RNA**→
env-133	SRS013705_Baylor_scaffold_2470	-	1400	1327	**RNA**→ rplM (PRK09216)Ribosomal_L13 (pfam00572)→ rpsI (PRK00132)Ribosomal_S9 (pfam00380)→
env-134	SRS014271_C2964309	+	4142	4215	**RNA**→
env-135	SRS015215_WUGC_scaffold_38570	+	157	230	**RNA**→ rplM (PRK09216)Ribosomal_L13 (pfam00572)→
env-136	SRS016342_C2855557	-	402	329	**RNA**→ rplM (PRK09216)Ribosomal_L13 (pfam00572)→
env-137	SRS017808_Baylor_scaffold_55041	-	1325	1252	**RNA**→ rplM (PRK09216)Ribosomal_L13 (pfam00572)→ rpsI (PRK00132)Ribosomal_S9 (pfam00380)→
env-138	SRS018791_C2310859	+	489	562	**RNA**→ rplM (PRK09216)Ribosomal_L13 (pfam00572)→ rpsI (PRK00132)Ribosomal_S9 (pfam00380)→

env-139	SRS020220_Baylor_scaffold_52325	-	173	100	**RNA**→
env-140	SRS021954_Baylor_scaffold_48638	+	2555	2628	**RNA**→ rplM (PRK09216)Ribosomal_L13 (pfam00572)→ rpsI (PRK00132)Ribosomal_S9 (pfam00380)→
env-141	SRS023617_Baylor_scaffold_62383	+	2343	2416	**RNA**→ rplM (PRK09216)Ribosomal_L13 (pfam00572)→ rpsI (PRK00132)Ribosomal_S9 (pfam00380)→
env-142	SRS023926_Baylor_scaffold_35364	+	6413	6486	**RNA**→ rplM (PRK09216)Ribosomal_L13 (pfam00572)→ rpsI (PRK00132)Ribosomal_S9 (pfam00380)→
env-143	SRS024081_C2596496	-	158	85	**RNA**→
env-144	SRS044373_C2896605	+	144	217	**RNA**→ rplM (PRK09216)Ribosomal_L13 (pfam00572)→ rpsI (PRK00132)Ribosomal_S9 (pfam00380)→
env-145	SRS044486_C2263445	-	551	478	**RNA**→ rplM (PRK09216)Ribosomal_L13 (pfam00572)→
env-146	SRS050244_C4466451	-	293	220	**RNA**→ rplM (PRK09216)Ribosomal_L13 (pfam00572)→
env-147	SRS056892_C1498053	-	740	667	**RNA**→ rplM (PRK09216)Ribosomal_L13 (pfam00572)→ rpsI (PRK00132)Ribosomal_S9 (pfam00380)→
env-148	SRS015797_C2641570	+	936	1009	**RNA**→
env-149	SRS019219_C2933673	+	150	223	**RNA**→ rplM (PRK09216)Ribosomal_L13 (pfam00572)→ rpsI (PRK00132)Ribosomal_S9 (pfam00380)→
env-150	SRS024318_LANL_scaffold_22081	-	1688	1615	**RNA**→ rplM (PRK09216)Ribosomal_L13 (pfam00572)→ rpsI (PRK00132)Ribosomal_S9 (pfam00380)→
env-151	SRS042643_WUGC_scaffold_65245	-	159	86	**RNA**→
env-152	SRS043663_LANL_scaffold_40074	-	6366	6293	**RNA**→ rplM (PRK09216)Ribosomal_L13 (pfam00572)→ rpsI (PRK00132)Ribosomal_S9 (pfam00380)→
env-153	SRS053854_LANL_scaffold_53869	+	18601	18674	**RNA**→ rplM (PRK09216)Ribosomal_L13 (pfam00572)→ rpsI (PRK00132)Ribosomal_S9 (pfam00380)→
env-154	SRS062761_LANL_scaffold_7973	+	6020	6093	**RNA**→ rplM (PRK09216)Ribosomal_L13 (pfam00572)→ rpsI (PRK00132)Ribosomal_S9 (pfam00380)→
env-155	SRS075404_C3410168	+	150	223	**RNA**→
env-156	SRS023835_Baylor_scaffold_45883	-	172	99	**RNA**→
env-157	SRS049147_LANL_scaffold_19223	-	1397	1324	**RNA**→ rplM (PRK09216)Ribosomal_L13 (pfam00572)→ rpsI (PRK00132)Ribosomal_S9 (pfam00380)→
env-158	SRS020856_Baylor_scaffold_43228	+	1090	1165	**RNA**→
env-159	SRS023557_C1740022	+	539	614	**RNA**→
env-160	SRS023930_Baylor_scaffold_1355	+	765	840	**RNA**→ rplM (PRK09216)Ribosomal_L13 (pfam00572)→ rpsI (PRK00132)Ribosomal_S9 (pfam00380)→
env-161	SRS013946_WUGC_scaffold_1088	+	79348	79423	**RNA**→ rplM (PRK09216)Ribosomal_L13 (pfam00572)→ rpsI (PRK00132)Ribosomal_S9 (pfam00380)→
env-162	SRS019124_C1404027	-	844	769	**RNA**→ rplM (PRK09216)Ribosomal_L13 (pfam00572)→ rpsI (PRK00132)Ribosomal_S9 (pfam00380)→
Psp-23-1	NC_022111.1	-	1426745	1426670	**RNA**→ rplM (PRK09216)Ribosomal_L13 (pfam00572)→ rpsI (PRK00132)Ribosomal_S9 (pfam00380)→
env-163	Keratinized_gingiva_LANL_scaffold_26598	-	469	394	**RNA**→ rplM (PRK09216)Ribosomal_L13 (pfam00572)→
env-164	SRS045127_LANL_scaffold_9552	-	310	235	**RNA**→ rplM (PRK09216)Ribosomal_L13 (pfam00572)→
env-165	SRS057539_LANL_scaffold_47289	+	1537	1612	**RNA**→ rplM (PRK09216)Ribosomal_L13 (pfam00572)→ rpsI (PRK00132)Ribosomal_S9 (pfam00380)→
env-166	SRS064774_LANL_scaffold_66778	-	205	130	**RNA**→ rplM (PRK09216)→
env-167	SRS024375_LANL_scaffold_43091	-	2175	2100	**RNA**→ rplM (PRK09216)Ribosomal_L13 (pfam00572)→ rpsI (PRK00132)Ribosomal_S9 (pfam00380)→
env-168	SRS045715_LANL_scaffold_76474	+	674	749	**RNA**→ rplM (PRK09216)Ribosomal_L13 (pfam00572)→ rpsI (PRK00132)Ribosomal_S9 (pfam00380)→
env-169	SRS013818_Baylor_scaffold_58539	-	3474	3399	**RNA**→ rplM (PRK09216)Ribosomal_L13 (pfam00572)→ rpsI (PRK00132)Ribosomal_S9 (pfam00380)→
env-170	SRS018439_Baylor_scaffold_27830	+	14827	14902	**RNA**→ rplM (PRK09216)Ribosomal_L13 (pfam00572)→ rpsI (PRK00132)Ribosomal_S9 (pfam00380)→
env-171	Palatine_Tonsils_LANL_C6199093	-	206	131	**RNA**→ rplM (PRK09216)→
env-172	SRS016740_Baylor_scaffold_45058	-	3466	3391	**RNA**→ rplM (PRK09216)Ribosomal_L13 (pfam00572)→ rpsI (PRK00132)Ribosomal_S9 (pfam00380)→
env-173	SRS011306_Baylor_scaffold_30322	-	1178	1103	**RNA**→ rplM (PRK09216)Ribosomal_L13 (pfam00572)→ rpsI (PRK00132)Ribosomal_S9 (pfam00380)→
env-174	SRS013164_Baylor_scaffold_48360	-	1235	1160	**RNA**→ rplM (PRK09216)Ribosomal_L13 (pfam00572)→ rpsI (PRK00132)Ribosomal_S9 (pfam00380)→
env-175	SRS013942_C1226381	+	56	131	**RNA**→ rplM (PRK09216)Ribosomal_L13 (pfam00572)→
env-176	SRS013947_WUGC_scaffold_26275	+	3554	3629	**RNA**→ rplM (PRK09216)Ribosomal_L13 (pfam00572)→ rpsI (PRK00132)Ribosomal_S9 (pfam00380)→
env-177	SRS013948_C1540062	-	1341	1266	**RNA**→ rplM (PRK09216)Ribosomal_L13 (pfam00572)→ rpsI (PRK00132)Ribosomal_S9 (pfam00380)→
env-178	SRS014888_C2952726	-	278	203	**RNA**→ rplM (PRK09216)RplM (COG0102)→
env-179	SRS015762_WUGC_scaffold_24890	-	155	80	**RNA**→
env-180	SRS016002_WUGC_scaffold_61027	+	308	383	**RNA**→ rplM (PRK09216)Ribosomal_L13 (pfam00572)→ rpsI (PRK00132)Ribosomal_S9 (pfam00380)→
env-181	SRS016086_WUGC_scaffold_26845	-	1575	1500	**RNA**→ rplM (PRK09216)Ribosomal_L13 (pfam00572)→ rpsI (PRK00132)Ribosomal_S9 (pfam00380)→
env-182	SRS016225_C2845363	+	174	249	**RNA**→
env-183	SRS016569_Baylor_scaffold_56398	-	14475	14400	**RNA**→ rplM (PRK09216)Ribosomal_L13 (pfam00572)→ rpsI (PRK00132)Ribosomal_S9 (pfam00380)→
env-184	SRS017808_C3912739	+	987	1062	**RNA**→ rplM (PRK09216)Ribosomal_L13 (pfam00572)→ rpsI (PRK00132)Ribosomal_S9 (pfam00380)→
env-185	SRS018145_Baylor_scaffold_43542	-	203	128	**RNA**→ rplM (PRK09216)→
env-186	SRS019022_WUGC_scaffold_45455	+	194	269	**RNA**→ rplM (PRK09216)Ribosomal_L13 (pfam00572)→ rpsI (PRK00132)Ribosomal_S9 (pfam00380)→
env-187	SRS019045_C2719629	+	63	138	**RNA**→ rplM (PRK09216)Ribosomal_L13 (pfam00572)→ rpsI (PRK00132)Ribosomal_S9 (pfam00380)→
env-188	SRS019126_WUGC_scaffold_13188	+	379	454	**RNA**→ rplM (PRK09216)Ribosomal_L13 (pfam00572)→ rpsI (PRK00132)Ribosomal_S9 (pfam00380)→
env-189	SRS019894_C3913439	-	664	589	**RNA**→ rplM (PRK09216)Ribosomal_L13 (pfam00572)→ rpsI (PRK00132)rps9 (CHL00079)→

env-190	SRS019974_Baylor_scaffold_2117	+	182	257	**RNA**→ rplM (PRK09216)Ribosomal_L13 (pfam00572)→ rpsI (PRK00132)Ribosomal_S9 (pfam00380)→
env-191	SRS021954_C3044878	+	70	145	**RNA**→ rplM (PRK09216)Ribosomal_L13 (pfam00572)→ rpsI (PRK00132)Ribosomal_S9 (pfam00380)→
env-192	SRS023958_C1974288	+	58	133	**RNA**→ rplM (PRK09216)Ribosomal_L13 (pfam00572)→
env-193	SRS024015_Baylor_scaffold_54415	+	4601	4676	**RNA**→ rplM (PRK09216)Ribosomal_L13 (pfam00572)→ rpsI (PRK00132)Ribosomal_S9 (pfam00380)→
env-194	SRS024277_C2835398	+	56	131	**RNA**→ rplM (PRK09216)Ribosomal_L13 (pfam00572)→ rpsI (PRK00132)Ribosomal_S9 (pfam00380)→
env-195	SRS048411_C6973885	+	102	177	**RNA**→ rplM (PRK09216)Ribosomal_L13 (pfam00572)→ rpsI (PRK00132)Ribosomal_S9 (pfam00380)→
env-196	SRS048791_C3265861	-	1162	1087	**RNA**→ rplM (PRK09216)Ribosomal_L13 (pfam00572)→ rpsI (PRK00132)Ribosomal_S9 (pfam00380)→
env-197	SRS052227_C2916121	-	1045	970	**RNA**→ rplM (PRK09216)Ribosomal_L13 (pfam00572)→ rpsI (PRK00132)Ribosomal_S9 (pfam00380)→
env-198	SRS053603_LANL_scaffold_51146	-	1182	1107	**RNA**→ rplM (PRK09216)Ribosomal_L13 (pfam00572)→ rpsI (PRK00132)Ribosomal_S9 (pfam00380)→
env-199	SRS054687_C3493368	+	1536	1611	**RNA**→ rplM (PRK09216)Ribosomal_L13 (pfam00572)→
env-200	SRS056323_C2562669	-	459	384	**RNA**→ rplM (PRK09216)Ribosomal_L13 (pfam00572)→
env-201	SRS057355_C2358048	-	1102	1027	**RNA**→ rplM (PRK09216)Ribosomal_L13 (pfam00572)→ rpsI (PRK00132)Ribosomal_S9 (pfam00380)→
env-202	SRS063193_LANL_scaffold_66856	-	3099	3024	**RNA**→ rplM (PRK09216)Ribosomal_L13 (pfam00572)→ rpsI (PRK00132)Ribosomal_S9 (pfam00380)→
env-203	SRS064423_LANL_scaffold_47969	-	3499	3424	**RNA**→ rplM (PRK09216)Ribosomal_L13 (pfam00572)→ rpsI (PRK00132)Ribosomal_S9 (pfam00380)→
env-204	SRS077736_LANL_scaffold_38236	+	42461	42536	**RNA**→ rplM (PRK09216)Ribosomal_L13 (pfam00572)→ rpsI (PRK00132)Ribosomal_S9 (pfam00380)→
env-205	Tongue_dor_WUGI_scaffold_8501	+	269	344	**RNA**→ rplM (PRK09216)Ribosomal_L13 (pfam00572)→ rpsI (PRK00132)Ribosomal_S9 (pfam00380)→
env-206	SRS014470_C3713445	+	547	622	**RNA**→ rplM (PRK09216)RplM (COG0102)→
env-207	SRS018300_C3341863	-	280	205	**RNA**→ rplM (PRK09216)RplM (COG0102)→
env-208	SRS024441_LANL_scaffold_48252	+	856	931	**RNA**→
env-209	SRS075404_C3567588	+	297	372	**RNA**→ rplM (PRK09216)RplM (COG0102)→
env-210	SRS011086_WUGC_scaffold_15953	-	4147	4072	**RNA**→ rplM (PRK09216)Ribosomal_L13 (pfam00572)→ rpsI (PRK00132)Ribosomal_S9 (pfam00380)→
env-211	SRS013879_WUGC_scaffold_7871	+	156	231	**RNA**→ rplM (PRK09216)Ribosomal_L13 (pfam00572)→ rpsI (PRK00132)Ribosomal_S9 (pfam00380)→
env-212	SRS011243_Baylor_scaffold_40702	-	1370	1295	**RNA**→ rplM (PRK09216)Ribosomal_L13 (pfam00572)→ rpsI (PRK00132)Ribosomal_S9 (pfam00380)→
env-213	SRS014573_WUGC_scaffold_49775	-	908	833	**RNA**→ rplM (PRK09216)Ribosomal_L13 (pfam00572)→ rpsI (PRK00132)Ribosomal_S9 (pfam00380)→
env-214	SRS015797_WUGC_scaffold_1453	-	1656	1581	**RNA**→ rplM (PRK09216)Ribosomal_L13 (pfam00572)→ rpsI (PRK00132)Ribosomal_S9 (pfam00380)→
env-215	SRS016037_WUGC_scaffold_35398	+	102	177	**RNA**→ rplM (PRK09216)Ribosomal_L13 (pfam00572)→
env-216	SRS021496_Baylor_scaffold_24398	-	5055	4980	**RNA**→ rplM (PRK09216)Ribosomal_L13 (pfam00572)→ rpsI (PRK00132)Ribosomal_S9 (pfam00380)→
env-217	SRS024138_Baylor_scaffold_896	+	1270	1345	**RNA**→ rplM (PRK09216)Ribosomal_L13 (pfam00572)→ rpsI (PRK00132)Ribosomal_S9 (pfam00380)→
env-218	SRS042643_WUGC_scaffold_46473	+	11248	11323	**RNA**→ rplM (PRK09216)Ribosomal_L13 (pfam00572)→ rpsI (PRK00132)Ribosomal_S9 (pfam00380)→
env-219	SRS047219_C2146198	+	105	180	**RNA**→ rplM (PRK09216)RplM (COG0102)→
env-220	SRS049147_LANL_scaffold_18610	+	946	1021	**RNA**→ rplM (PRK09216)Ribosomal_L13 (pfam00572)→ rpsI (PRK00132)Ribosomal_S9 (pfam00380)→
env-221	SRS055426_LANL_scaffold_19831	-	3585	3510	**RNA**→ rplM (PRK09216)Ribosomal_L13 (pfam00572)→ rpsI (PRK00132)Ribosomal_S9 (pfam00380)→ hypo→
env-222	Buccal_mucosa_LANL_scaffold_78460	-	760	685	**RNA**→ rplM (PRK09216)Ribosomal_L13 (pfam00572)→ rpsI (PRK00132)Ribosomal_S9 (pfam00380)→
env-223	SRS014271_WUGC_scaffold_629	+	43	118	**RNA**→ rplM (PRK09216)Ribosomal_L13 (pfam00572)→ rpsI (PRK00132)Ribosomal_S9 (pfam00380)→
env-224	SRS062544_LANL_scaffold_4602	-	1246	1171	**RNA**→ rplM (PRK09216)Ribosomal_L13 (pfam00572)→ rpsI (PRK00132)Ribosomal_S9 (pfam00380)→
env-225	SRS065278_LANL_scaffold_63970	-	3725	3650	**RNA**→ rplM (PRK09216)Ribosomal_L13 (pfam00572)→ rpsI (PRK00132)Ribosomal_S9 (pfam00380)→
env-226	SRS024081_LANL_scaffold_32043	+	406	481	**RNA**→ rplM (PRK09216)Ribosomal_L13 (pfam00572)→ rpsI (PRK00132)Ribosomal_S9 (pfam00380)→
env-227	SRS022719_LANL_scaffold_53966	+	5202	5277	**RNA**→ rplM (PRK09216)Ribosomal_L13 (pfam00572)→ rpsI (PRK00132)Ribosomal_S9 (pfam00380)→
env-228	SRS044662_LANL_scaffold_14148	-	15885	15810	**RNA**→ rplM (PRK09216)Ribosomal_L13 (pfam00572)→ rpsI (PRK00132)Ribosomal_S9 (pfam00380)→
env-229	SRS050669_LANL_scaffold_25595	+	601	676	**RNA**→ rplM (PRK09216)Ribosomal_L13 (pfam00572)→ rpsI (PRK00132)Ribosomal_S9 (pfam00380)→
env-230	SRS058336_LANL_scaffold_14781	-	102	27	**RNA**→ hypo→
env-231	SRS016342_Baylor_scaffold_48981	+	82	157	**RNA**→ rplM (PRK09216)Ribosomal_L13 (pfam00572)→ rpsI (PRK00132)Ribosomal_S9 (pfam00380)→
env-232	SRS018739_WUGC_scaffold_60144	-	1366	1291	**RNA**→ rplM (PRK09216)Ribosomal_L13 (pfam00572)→ rpsI (PRK00132)Ribosomal_S9 (pfam00380)→
env-233	SRS019126_C2043960	+	254	329	**RNA**→
Pna-2-1	NZ_BAKD01000002.1	-	398642	398567	**RNA**→ rplM (PRK09216)Ribosomal_L13 (pfam00572)→ rpsI (PRK00132)Ribosomal_S9 (pfam00380)→
Pna-1-1	NZ_KB904328.1	+	132132	132207	**RNA**→ rplM (PRK09216)Ribosomal_L13 (pfam00572)→ rpsI (PRK00132)Ribosomal_S9 (pfam00380)→
env-234	SRS011140_Baylor_scaffold_53572	-	2158	2083	**RNA**→ rplM (PRK09216)Ribosomal_L13 (pfam00572)→ rpsI (PRK00132)Ribosomal_S9 (pfam00380)→
env-235	SRS015434_C5898829	+	238	313	**RNA**→ rplM (PRK09216)Ribosomal_L13 (pfam00572)→
env-236	SRS015644_WUGC_scaffold_48566	+	1536	1611	**RNA**→ rplM (PRK09216)Ribosomal_L13 (pfam00572)→ rpsI (PRK00132)Ribosomal_S9 (pfam00380)→
env-237	SRS019077_WUGC_scaffold_47444	-	372	297	**RNA**→ rplM (PRK09216)Ribosomal_L13 (pfam00572)→
env-238	SRS019127_C1659470	+	56	131	**RNA**→ rplM (PRK09216)Ribosomal_L13 (pfam00572)→ rpsI (PRK00132)Ribosomal_S9 (pfam00380)→
env-239	SRS020856_Baylor_scaffold_12760	-	1113	1038	**RNA**→ rplM (PRK09216)Ribosomal_L13 (pfam00572)→ rpsI (PRK00132)Ribosomal_S9 (pfam00380)→

env-240	SRS022077_Baylor_scaffold_47573	+	56	131	**RNA**→ rplM (PRK09216)Ribosomal_L13 (pfam00572)→ rpsI (PRK00132)Ribosomal_S9 (pfam00380)→
env-241	SRS022621_Baylor_scaffold_3806	-	26534	26459	**RNA**→ rplM (PRK09216)Ribosomal_L13 (pfam00572)→ rpsI (PRK00132)Ribosomal_S9 (pfam00380)→
env-242	SRS023617_Baylor_scaffold_52938	-	36529	36454	**RNA**→ rplM (PRK09216)Ribosomal_L13 (pfam00572)→ rpsI (PRK00132)Ribosomal_S9 (pfam00380)→
env-243	SRS024580_C3074526	-	620	545	**RNA**→ rplM (PRK09216)Ribosomal_L13 (pfam00572)→
env-244	SRS042131_WUGC_scaffold_8821	-	37335	37260	**RNA**→ rplM (PRK09216)Ribosomal_L13 (pfam00572)→ rpsI (PRK00132)Ribosomal_S9 (pfam00380)→
env-245	SRS050244_LANL_scaffold_95985	+	1714	1789	**RNA**→ rplM (PRK09216)Ribosomal_L13 (pfam00572)→ rpsI (PRK00132)Ribosomal_S9 (pfam00380)→
env-246	SRS019607_C2757825	-	1575	1500	**RNA**→ rplM (PRK09216)Ribosomal_L13 (pfam00572)→ rpsI (PRK00132)Ribosomal_S9 (pfam00380)→
env-247	SRS023835_Baylor_scaffold_30646	-	382	307	**RNA**→ rplM (PRK09216)Ribosomal_L13 (pfam00572)→
env-248	SRS057205_LANL_scaffold_32691	+	1114	1189	**RNA**→
env-249	SRS012279_C2758674	+	3094	3169	**RNA**→ rplM (PRK09216)Ribosomal_L13 (pfam00572)→ rpsI (PRK00132)Ribosomal_S9 (pfam00380)→
env-250	SRS014692_C1492733	+	63	138	**RNA**→ rplM (PRK09216)Ribosomal_L13 (pfam00572)→
env-251	SRS019026_C1981803	+	132	207	**RNA**→ rplM (PRK09216)Ribosomal_L13 (pfam00572)→ rpsI (PRK00132)Ribosomal_S9 (pfam00380)→
env-252	SRS019027_C1968276	+	63	138	**RNA**→ rplM (PRK09216)Ribosomal_L13 (pfam00572)→ rpsI (PRK00132)Ribosomal_S9 (pfam00380)→
env-253	SRS019327_WUGC_scaffold_24766	-	2945	2870	**RNA**→ rplM (PRK09216)Ribosomal_L13 (pfam00572)→ rpsI (PRK00132)Ribosomal_S9 (pfam00380)→
env-254	SRS019389_C2252888	+	2434	2509	**RNA**→
env-255	SRS023352_C4687326	-	1370	1295	**RNA**→ rplM (PRK09216)Ribosomal_L13 (pfam00572)→ rpsI (PRK00132)Ribosomal_S9 (pfam00380)→
env-256	SRS047210_C3287465	-	670	595	**RNA**→ rplM (PRK09216)Ribosomal_L13 (pfam00572)→ rpsI (PRK00132)rps9 (CHL00079)→
env-257	SRS057791_LANL_scaffold_82241	+	477	552	**RNA**→ rplM (PRK09216)Ribosomal_L13 (pfam00572)→ rpsI (PRK00132)Ribosomal_S9 (pfam00380)→
env-258	SRS014684_WUGC_scaffold_44011	+	3280	3355	**RNA**→ rplM (PRK09216)Ribosomal_L13 (pfam00572)→ rpsI (PRK00132)Ribosomal_S9 (pfam00380)→
env-259	SRS017533_Baylor_scaffold_27038	-	80	5	**RNA**→
env-260	SRS043663_LANL_scaffold_17693	+	210	285	**RNA**→ rplM (PRK09216)Ribosomal_L13 (pfam00572)→
env-261	SRS062761_C4207862	-	374	299	**RNA**→ rplM (PRK09216)Ribosomal_L13 (pfam00572)→
env-262	SRS013946_WUGC_scaffold_14040	+	788	863	**RNA**→ rplM (PRK09216)Ribosomal_L13 (cd00392)→
env-263	SRS019125_WUGC_scaffold_9864	-	274	199	**RNA**→ rplM (PRK09216)RplM (COG0102)→
env-264	SRS013506_Baylor_scaffold_8126	-	301	226	**RNA**→ rplM (PRK09216)Ribosomal_L13 (cd00392)→
env-265	SRS014473_C1126965	+	422	497	**RNA**→ rplM (PRK09216)Ribosomal_L13 (pfam00572)→ rpsI (PRK00132)Ribosomal_S9 (pfam00380)→
env-266	AUXO014368639.1	-	674	600	**RNA**→ rplM (PRK09216)Ribosomal_L13 (pfam00572)→
env-267	AUXO015562770.1	+	235	303	**RNA**→ rplM (PRK09216)Ribosomal_L13 (pfam00572)→ rpsI (PRK00132)Ribosomal_S9 (pfam00380)→
Bsp-2-1	NZ_JNKX01000045.1	-	3997	3929	**RNA**→ rplM (PRK09216)Ribosomal_L13 (pfam00572)→ rpsI (PRK00132)Ribosomal_S9 (pfam00380)→
Bsp-1-1	NZ_JNLA01000048.1	+	27849	27917	**RNA**→ rplM (PRK09216)Ribosomal_L13 (pfam00572)→ rpsI (PRK00132)Ribosomal_S9 (pfam00380)→
env-268	SRS023352_C4496944	-	123	46	**RNA**→ hypo→
env-269	SRS062544_C2920745	+	463	540	**RNA**→
env-270	SRS019219_WUGC_scaffold_426	-	214	137	**RNA**→ hypo→
env-271	SRS051791_LANL_scaffold_54765	-	217	140	**RNA**→
env-272	SRS015057_WUGC_scaffold_61382	+	542	619	**RNA**→
env-273	SRS048411_C6924690	+	505	582	**RNA**→
env-274	SRS057791_C3531782	+	268	345	**RNA**→
env-275	SRS045715_LANL_scaffold_76924	+	360	437	**RNA**→
env-276	SRS015272_C3500034	-	214	137	**RNA**→
env-277	SRS018791_C2278485	+	773	850	**RNA**→
env-278	SRS011255_C2362793	-	117	40	**RNA**→
env-279	SRS013950_WUGC_scaffold_2138	-	220	143	**RNA**→
env-280	SRS013836_C4630228	+	244	321	**RNA**→
env-281	SRS023841_C1489733	-	221	144	**RNA**→ hypo→
env-282	Supragingival_plaque_LANL_scaffold_194841	-	474	397	**RNA**→ rplM (PRK09216)Ribosomal_L13 (pfam00572)→
env-283	SRS017691_C5410429	+	2077	2154	**RNA**→
env-284	SRS024561_C2727733	+	380	457	**RNA**→
env-285	SRS024277_C2727216	-	173	96	**RNA**→ hypo→
env-286	SRS016319_C3957492	-	123	46	**RNA**→ hypo→
env-287	SRS042131_C2217398	+	340	417	**RNA**→
env-288	SRS043663_C4080713	+	808	885	**RNA**→
env-289	SRS062761_C4201374	+	277	354	**RNA**→

env-290	SRS015797_C2619071	-	219	142	**RNA**→
env-291	SRS019607_C2703769	-	184	107	**RNA**→ ←hypo
env-292	SRS021496_Baylor_scaffold_55246	+	49	126	**RNA**→ rplM (PRK09216)Ribosomal_L13 (pfam00572)→
env-293	SRS024081_C2601354	+	658	735	**RNA**→
env-294	SRS049389_C4506064	+	176	253	**RNA**→
env-295	SRS075404_C3622013	+	648	725	**RNA**→
env-296	SRS018739_C3603893	+	489	566	**RNA**→
env-297	SRS020220_C3576851	-	115	38	**RNA**→ hypo→
env-298	SRS011306_C3387646	+	94	171	**RNA**→
env-299	SRS019071_C5065527	-	138	61	**RNA**→ hypo→
env-300	SRS019327_WUGC_scaffold_20822	+	376	453	**RNA**→
env-301	Supragingival_plaque_LANL_C182575899	-	117	40	**RNA**→ hypo→
env-302	SRS016002_C2496321	-	179	102	**RNA**→ hypo→
env-303	SRS019126_C2138744	+	361	438	**RNA**→
env-304	SRS019127_C1661984	+	1415	1492	**RNA**→
env-305	SRS017209_Baylor_scaffold_64433	-	214	137	**RNA**→
env-306	SRS024318_LANL_scaffold_131	-	214	137	**RNA**→
env-307	SRS057205_LANL_scaffold_46232	-	410	333	**RNA**→ rplM (PRK09216)Ribosomal_L13 (pfam00572)→
env-308	SRS018357_C3783001	-	179	102	**RNA**→ hypo→
env-309	SRS020856_C2544654	+	239	316	**RNA**→
env-310	SRS044373_WUGC_scaffold_63614	-	123	46	**RNA**→
env-311	SRS014271_C2955858	+	2330	2407	**RNA**→
env-312	SRS050244_C4442009	+	418	495	**RNA**→
env-313	SRS062544_C3030084	+	2332	2409	**RNA**→
env-314	SRS013164_C2312325	-	119	42	**RNA**→ hypo→
env-315	SRS016225_C2929058	+	310	388	**RNA**→
env-316	4491686.3.521687	+	140	220	**RNA**→ ←hypo
env-317	scaffold7222_1_MH0052	-	220	140	**RNA**→
env-318	4491686.3.475604	-	103	23	**RNA**→ ←hypo
env-319	2013397958	-	676	600	**RNA**→ rplM (PRK09216)Ribosomal_L13 (pfam00572)→ rplM (PRK09216)Ribosomal_L13 (pfam00572)→
Psp-3-1	NZ_AUJK01000003.1	+	63931	64008	**RNA**→ rplM (PRK09216)Ribosomal_L13 (pfam00572)→ rpsI (PRK00132)Ribosomal_S9 (pfam00380)→
env-320	scaffold26454_4_MH0084	+	5212	5288	**RNA**→ rplM (PRK09216)Ribosomal_L13 (pfam00572)→ rpsI (PRK00132)Ribosomal_S9 (pfam00380)→
env-321	scaffold66021_2_MH0081	+	3270	3346	**RNA**→ rplM (PRK09216)Ribosomal_L13 (pfam00572)→ rpsI (PRK00132)Ribosomal_S9 (pfam00380)→
env-322	scaffold25402_2_MH0054	+	2416	2492	**RNA**→ rplM (PRK09216)Ribosomal_L13 (pfam00572)→ rpsI (PRK00132)Ribosomal_S9 (pfam00380)→
env-323	scaffold8724_1_O2.UC-16	-	106	30	**RNA**→
env-324	SRS015782_WUGC_scaffold_20008	-	24364	24288	**RNA**→ rplM (PRK09216)Ribosomal_L13 (pfam00572)→ rpsI (PRK00132)Ribosomal_S9 (pfam00380)→
env-325	SRS047044_WUGC_scaffold_15995	-	1654	1578	**RNA**→ rplM (PRK09216)Ribosomal_L13 (pfam00572)→ rpsI (PRK00132)Ribosomal_S9 (pfam00380)→
env-326	scaffold34413_1_V1.CD-13	+	111	187	**RNA**→ rplM (PRK09216)Ribosomal_L13 (pfam00572)→ rpsI (PRK00132)Ribosomal_S9 (pfam00380)→
env-327	scaffold69522_1_MH0044	+	6237	6313	**RNA**→ rplM (PRK09216)Ribosomal_L13 (pfam00572)→ rpsI (PRK00132)Ribosomal_S9 (pfam00380)→
env-328	scaffold59624_1_MH0052	+	3663	3739	**RNA**→ ←hypo
env-329	scaffold15726_3_MH0032	-	12257	12181	**RNA**→ rplM (PRK09216)Ribosomal_L13 (pfam00572)→ rpsI (PRK00132)Ribosomal_S9 (pfam00380)→
env-330	scaffold58986_1_MH0033	-	3162	3086	**RNA**→ rplM (PRK09216)Ribosomal_L13 (pfam00572)→ rpsI (PRK00132)Ribosomal_S9 (pfam00380)→
env-331	scaffold11675_1_MH0026	+	12207	12283	**RNA**→ rplM (PRK09216)Ribosomal_L13 (pfam00572)→ rpsI (PRK00132)Ribosomal_S9 (pfam00380)→
env-332	NLM025_scaffold6375_35	-	965	889	**RNA**→ rplM (PRK09216)Ribosomal_L13 (pfam00572)→ rpsI (PRK00132)Ribosomal_S9 (pfam00380)→
env-333	SRS013951_WUGC_scaffold_10163	-	35684	35608	**RNA**→ rplM (PRK09216)Ribosomal_L13 (pfam00572)→ rpsI (PRK00132)Ribosomal_S9 (pfam00380)→
env-334	SRS019161_WUGC_scaffold_63747	+	921	997	**RNA**→ rplM (PRK09216)Ribosomal_L13 (pfam00572)→ rpsI (PRK00132)Ribosomal_S9 (pfam00380)→
env-335	SRS050422_LANL_scaffold_39679	-	16737	16661	**RNA**→ rplM (PRK09216)Ribosomal_L13 (pfam00572)→ rpsI (PRK00132)Ribosomal_S9 (pfam00380)→
env-336	scaffold3922_2_MH0001	-	532	456	**RNA**→ rplM (PRK09216)Ribosomal_L13 (pfam00572)→
env-337	scaffold12670_1_MH0041	+	6023	6099	**RNA**→ rplM (PRK09216)Ribosomal_L13 (pfam00572)→ rpsI (PRK00132)Ribosomal_S9 (pfam00380)→
env-338	4491401.3_NODE_59631	+	440	516	**RNA**→ rplM (PRK09216)Ribosomal_L13 (pfam00572)→
env-339	scaffold24499_3_MH0018	-	199	123	**RNA**→
env-340	SRS048164_WUGC_scaffold_7139	-	11028	10952	**RNA**→ rplM (PRK09216)Ribosomal_L13 (pfam00572)→ rpsI (PRK00132)Ribosomal_S9 (pfam00380)→

env-341	scaffold13081_6_MH0009	-	3032	2956	**RNA**→ rplM (PRK09216)Ribosomal_L13 (pfam00572)→ rpsI (PRK00132)Ribosomal_S9 (pfam00380)→
env-342	scaffold29722_3_MH0034	+	2377	2453	**RNA**→ rplM (PRK09216)Ribosomal_L13 (pfam00572)→ rpsI (PRK00132)Ribosomal_S9 (pfam00380)→
env-343	scaffold19923_2_O2.UC-12	-	430	354	**RNA**→ rplM (PRK09216)Ribosomal_L13 (pfam00572)→
env-344	SRS015794_Baylor_scaffold_29113	-	896	820	**RNA**→ rplM (PRK09216)Ribosomal_L13 (pfam00572)→ rpsI (PRK00132)Ribosomal_S9 (pfam00380)→
env-345	scaffold988_4_MH0046	-	10774	10698	**RNA**→ rplM (PRK09216)Ribosomal_L13 (pfam00572)→ rpsI (PRK00132)Ribosomal_S9 (pfam00380)→
env-346	scaffold60646_3_MH0076	-	3034	2958	**RNA**→ rplM (PRK09216)Ribosomal_L13 (pfam00572)→ rpsI (PRK00132)Ribosomal_S9 (pfam00380)→
env-347	scaffold60041_3_MH0056	-	523	447	**RNA**→ rplM (PRK09216)Ribosomal_L13 (pfam00572)→
env-348	scaffold62900_7_O2.UC-19	+	1505	1581	**RNA**→ rplM (PRK09216)Ribosomal_L13 (pfam00572)→ rpsI (PRK00132)Ribosomal_S9 (pfam00380)→
env-349	Subgingival_plaque_LANL_C11191810	+	401	479	**RNA**→
Pma-3-1	NZ_JH594503.1	-	275251	275173	**RNA**→ rplM (PRK09216)Ribosomal_L13 (pfam00572)→ rpsI (PRK00132)Ribosomal_S9 (pfam00380)→
env-350	SRS024087_C3572528	-	219	141	**RNA**→
env-351	SRS024289_LANL_scaffold_69854	-	837	759	**RNA**→ rplM (PRK09216)Ribosomal_L13 (pfam00572)→ rpsI (PRK00132)Ribosomal_S9 (pfam00380)→
env-352	SRS047634_LANL_scaffold_34142	-	1677	1599	**RNA**→ rplM (PRK09216)Ribosomal_L13 (pfam00572)→ rpsI (PRK00132)Ribosomal_S9 (pfam00380)→
Pma-1-1	NZ_KB908319.1	+	316856	316934	**RNA**→ rplM (PRK09216)Ribosomal_L13 (pfam00572)→ rpsI (PRK00132)Ribosomal_S9 (pfam00380)→
Pma-2-1	NZ_BAJZ01000003.1	+	9704	9782	**RNA**→ rplM (PRK09216)Ribosomal_L13 (pfam00572)→ rpsI (PRK00132)Ribosomal_S9 (pfam00380)→
env-353	SRS018443_Baylor_scaffold_42450	+	2749	2827	**RNA**→
env-354	SRS019980_C3029122	+	888	966	**RNA**→
env-355	SRS017227_Baylor_scaffold_86068	-	121	43	**RNA**→
env-356	SRS049268_LANL_scaffold_27761	+	405	483	**RNA**→
env-357	SRS017120_Baylor_scaffold_16174	+	172	250	**RNA**→ rplM (PRK09216)Ribosomal_L13 (pfam00572)→ rpsI (PRK00132)Ribosomal_S9 (pfam00380)→
env-358	SRS024318_LANL_scaffold_13466	-	227	149	**RNA**→ hypo→
env-359	SRS018791_WUGC_scaffold_24827	+	398	476	**RNA**→ rplM (PRK09216)Ribosomal_L13 (pfam00572)→ rpsI (PRK00132)Ribosomal_S9 (pfam00380)→
env-360	SRS075404_C3518551	+	334	412	**RNA**→
env-361	SRS015395_C2316301	+	133	211	**RNA**→
env-362	SRS019122_C5835162	+	454	532	**RNA**→
env-363	SRS011086_WUGC_scaffold_41294	+	17651	17729	**RNA**→ rplM (PRK09216)Ribosomal_L13 (pfam00572)→ rpsI (PRK00132)Ribosomal_S9 (pfam00380)→
env-364	SRS020334_Baylor_scaffold_26466	-	656	578	**RNA**→ rplM (PRK09216)Ribosomal_L13 (pfam00572)→
env-365	SRS023617_Baylor_scaffold_72410	+	1232	1310	**RNA**→
env-366	SRS047210_C3259279	-	125	47	**RNA**→
env-367	SRS050244_LANL_scaffold_47339	-	253	175	**RNA**→
env-368	SRS014689_C1337541	-	253	175	**RNA**→
env-369	SRS016225_C2877068	+	184	262	**RNA**→
env-370	SRS018591_WUGC_scaffold_20233	+	10037	10115	**RNA**→ rplM (PRK09216)Ribosomal_L13 (pfam00572)→ rpsI (PRK00132)Ribosomal_S9 (pfam00380)→
env-371	SRS013705_C3658225	+	116	194	**RNA**→
env-372	SRS019607_WUGC_scaffold_9276	-	440	362	**RNA**→ rplM (PRK09216)Ribosomal_L13 (pfam00572)→
env-373	SRS014684_C3202863	+	288	366	**RNA**→
env-374	SRS047824_WUGC_scaffold_43430	+	1021	1099	**RNA**→ rplM (PRK09216)Ribosomal_L13 (pfam00572)→ rpsI (PRK00132)Ribosomal_S9 (pfam00380)→
env-375	SRS014573_WUGC_scaffold_51555	-	550	472	**RNA**→ rplM (PRK09216)Ribosomal_L13 (pfam00572)→
env-376	SRS015209_WUGC_scaffold_7145	-	1222	1144	**RNA**→ rplM (PRK09216)Ribosomal_L13 (pfam00572)→ rpsI (PRK00132)Ribosomal_S9 (pfam00380)→
env-377	SRS021496_Baylor_scaffold_21245	+	106	184	**RNA**→ rplM (PRK09216)Ribosomal_L13 (pfam00572)→ rpsI (PRK00132)Ribosomal_S9 (pfam00380)→
env-378	SRS023835_Baylor_scaffold_21120	-	440	362	**RNA**→ rplM (PRK09216)Ribosomal_L13 (pfam00572)→
env-379	SRS047219_WUGC_scaffold_3870	+	3309	3387	**RNA**→ rplM (PRK09216)Ribosomal_L13 (pfam00572)→ rpsI (PRK00132)Ribosomal_S9 (pfam00380)→
env-380	SRS048791_LANL_scaffold_13616	+	156	234	**RNA**→
env-381	SRS051791_LANL_scaffold_6037	+	5220	5298	**RNA**→ rplM (PRK09216)Ribosomal_L13 (pfam00572)→ rpsI (PRK00132)Ribosomal_S9 (pfam00380)→
env-382	SRS062544_LANL_scaffold_33552	+	2985	3063	**RNA**→ rplM (PRK09216)Ribosomal_L13 (pfam00572)→ rpsI (PRK00132)Ribosomal_S9 (pfam00380)→
env-383	SRS063193_LANL_scaffold_17717	-	550	472	**RNA**→ rplM (PRK09216)Ribosomal_L13 (pfam00572)→
env-384	SRS023926_Baylor_scaffold_61302	-	446	368	**RNA**→ rplM (PRK09216)Ribosomal_L13 (pfam00572)→
env-385	SRS019126_C2050939	+	106	184	**RNA**→
env-386	SRS014692_C1429530	+	228	306	**RNA**→
env-387	SRS023352_LANL_scaffold_12203	+	574	652	**RNA**→ rplM (PRK09216)Ribosomal_L13 (pfam00572)→ rpsI (PRK00132)Ribosomal_S9 (pfam00380)→
env-388	SRS053603_LANL_scaffold_56678	-	125	47	**RNA**→ hypo→
env-389	SRS075404_LANL_scaffold_32190	+	334	412	**RNA**→ rplM (PRK09216)Ribosomal_L13 (pfam00572)→

env-390	SRS019026_C1988327	+	1161	1239	**RNA**→
env-391	SRS042131_WUGC_scaffold_38955	-	396	318	**RNA**→ rplM (PRK09216)Ribosomal_L13 (pfam00572)→
env-392	SRS015038_WUGC_scaffold_15341	-	253	175	**RNA**→
env-393	SRS016002_WUGC_scaffold_10716	+	1413	1491	**RNA**→ rplM (PRK09216)Ribosomal_L13 (pfam00572)→ rplM (PRK09216)Ribosomal_L13 (pfam00572)→ rpsI (PRK00132)Ribosomal_S9 (pfam00380)→
env-394	SRS056323_C2617078	-	253	175	**RNA**→
env-395	SRS065278_C2566571	-	253	175	**RNA**→
env-396	SRS019974_C3363040	+	862	940	**RNA**→
Psa-2-1	NZ_GL629647.1	+	2253698	2253776	**RNA**→ rplM (PRK09216)Ribosomal_L13 (pfam00572)→ rpsI (PRK00132)Ribosomal_S9 (pfam00380)→
env-397	SRS016086_C2389321	-	101	23	**RNA**→ hypo→
env-398	SRS020856_Baylor_scaffold_13408	+	234	312	**RNA**→ rplM (PRK09216)Ribosomal_L13 (pfam00572)→ rpsI (PRK00132)Ribosomal_S9 (pfam00380)→
env-399	SRS014271_WUGC_scaffold_18759	+	466	544	**RNA**→ rplM (PRK09216)Ribosomal_L13 (pfam00572)→
env-400	SRS015057_WUGC_scaffold_34905	+	1097	1175	**RNA**→ rplM (PRK09216)Ribosomal_L13 (pfam00572)→
Psa-3-1	NZ_AWGW01000017.1	+	92405	92483	**RNA**→ rplM (PRK09216)Ribosomal_L13 (pfam00572)→ rpsI (PRK00132)Ribosomal_S9 (pfam00380)→
env-401	SRS012279_C2722110	+	914	992	**RNA**→
env-402	SRS024015_Baylor_scaffold_12438	+	2443	2521	**RNA**→ rplM (PRK09216)Ribosomal_L13 (pfam00572)→ rpsI (PRK00132)Ribosomal_S9 (pfam00380)→
env-403	SRS013234_C2993348	-	253	175	**RNA**→
env-404	SRS024081_LANL_scaffold_75049	+	9042	9120	**RNA**→
env-405	SRS057205_C3268011	+	63	141	**RNA**→
env-406	Tongue_dor_WUGI_scaffold_48438	-	373	295	**RNA**→ rplM (PRK09216)Ribosomal_L13 (pfam00572)→
env-407	SRS022143_WUGC_scaffold_32861	+	47	125	**RNA**→ rplM (PRK09216)Ribosomal_L13 (pfam00572)→
env-408	SRS016541_Baylor_scaffold_9219	-	350	274	**RNA**→ rplM (PRK09216)Ribosomal_L13 (pfam00572)→
env-409	SRS016575_C3620180	+	304	380	**RNA**→
env-410	SRS017691_Baylor_scaffold_1218	-	223	147	**RNA**→
env-411	SRS049318_C6476930	+	709	785	**RNA**→
env-412	SRS016569_Baylor_scaffold_25639	-	252	175	**RNA**→
Por-6-1	NZ_BAJC01000029.1	+	9652	9728	**RNA**→ hypo→ rplM (PRK09216)Ribosomal_L13 (pfam00572)→ rpsI (PRK00132)Ribosomal_S9 (pfam00380)→
Por-5-1	NZ_KB898581.1	-	166045	165968	**RNA**→ rplM (PRK09216)Ribosomal_L13 (pfam00572)→ rpsI (PRK00132)Ribosomal_S9 (pfam00380)→
env-413	SRS013723_C3447116	-	123	46	**RNA**→
env-414	SRS013170_Baylor_scaffold_49654	-	224	147	**RNA**→ hypo→
env-415	SRS015055_C1639257	-	224	147	**RNA**→ hypo→
env-416	SRS015062_C160810	-	234	157	**RNA**→ hypo→
env-417	SRS023964_C2594004	-	224	147	**RNA**→
Por-4-1	NZ_GL349571.1	+	7135	7212	**RNA**→ rplM (PRK09216)Ribosomal_L13 (pfam00572)→ rplM (PRK09216)Ribosomal_L13 (pfam00572)→ rpsI (PRK00132)Ribosomal_S9 (pfam00380)→ rpsI (PRK00132)Ribosomal_S9 (pfam00380)→
Por-7-1	NZ_GG703883.1	+	239161	239238	**RNA**→ rplM (PRK09216)Ribosomal_L13 (pfam00572)→ rpsI (PRK00132)Ribosomal_S9 (pfam00380)→
env-418	Buccal_mucosa_LANL_C65077335	+	342	419	**RNA**→ hypo→
env-419	SRS019980_C3064054	-	118	41	**RNA**→ hypo→
env-420	SRS019029_C3217778	+	42	119	**RNA**→ hypo→ rplM (PRK09216)Ribosomal_L13 (pfam00572)→
env-421	SRS024087_C3239609	+	108	185	**RNA**→
env-422	SRS014476_C3433381	+	868	945	**RNA**→
env-423	Subgingival_plaque_LANL_C11098963	-	118	41	**RNA**→ hypo→
env-424	SRS024649_LANL_scaffold_18754	+	580	657	**RNA**→
Psp-8-1	NZ_AZHT01000177.1	+	6805	6883	**RNA**→ rplM (PRK09216)Ribosomal_L13 (pfam00572)→ rpsI (PRK00132)Ribosomal_S9 (pfam00380)→
env-425	SRS019219_WUGC_scaffold_64631	-	18808	18730	**RNA**→ rplM (PRK09216)Ribosomal_L13 (pfam00572)→ rpsI (PRK00132)Ribosomal_S9 (pfam00380)→
env-426	SRS014271_C2786805	-	121	43	**RNA**→ hypo→
env-427	SRS024318_LANL_scaffold_76469	+	3580	3658	**RNA**→
env-428	SRS062544_C2827308	-	121	43	**RNA**→ hypo→
env-429	SRS017120_Baylor_scaffold_66317	-	655	577	**RNA**→ rplM (PRK09216)Ribosomal_L13 (pfam00572)→
env-430	SRS042643_WUGC_scaffold_1740	-	1427	1349	**RNA**→ rplM (PRK09216)Ribosomal_L13 (pfam00572)→ rpsI (PRK00132)Ribosomal_S9 (pfam00380)→
env-431	SRS018739_WUGC_scaffold_55643	+	229	307	**RNA**→ rplM (PRK09216)Ribosomal_L13 (pfam00572)→
env-432	SRS050244_LANL_scaffold_95751	+	228	306	**RNA**→ rplM (PRK09216)Ribosomal_L13 (pfam00572)→ rpsI (PRK00132)Ribosomal_S9 (pfam00380)→
env-433	SRS019026_C1859658	+	229	307	**RNA**→

env-434	SRS044373_C2740995	-	121	43	**RNA**→ hypo→
env-435	SRS021496_Baylor_scaffold_66287	+	228	306	**RNA**→ rplM (PRK09216)Ribosomal_L13 (pfam00572)→ rpsI (PRK00132)Ribosomal_S9 (pfam00380)→
env-436	SRS023352_LANL_scaffold_79269	-	463	385	**RNA**→ rplM (PRK09216)Ribosomal_L13 (pfam00572)→
env-437	SRS022143_WUGC_scaffold_73957	+	77	155	**RNA**→ rplM (PRK09216)Ribosomal_L13 (pfam00572)→ rpsI (PRK00132)Ribosomal_S9 (pfam00380)→
env-438	SRS015893_C2357989	-	655	577	**RNA**→ rplM (PRK09216)Ribosomal_L13 (pfam00572)→
env-439	SRS015057_WUGC_scaffold_71704	+	230	308	**RNA**→ rplM (PRK09216)Ribosomal_L13 (pfam00572)→
env-440	SRS024355_LANL_scaffold_106705	-	2853	2775	**RNA**→ rplM (PRK09216)Ribosomal_L13 (pfam00572)→ rpsI (PRK00132)Ribosomal_S9 (pfam00380)→
env-441	SRS058053_C5276896	-	119	41	**RNA**→
env-442	SRS023595_C6791388	-	141	63	**RNA**→ hypo→
env-443	SRS047219_C2204013	+	361	439	**RNA**→
Pou-1-1	NZ_JH114217.1	+	161819	161897	**RNA**→ rplM (PRK09216)Ribosomal_L13 (pfam00572)→ rpsI (PRK00132)Ribosomal_S9 (pfam00380)→
env-444	SRS019980_C2940732	+	377	455	**RNA**→
env-445	SRS022536_LANL_scaffold_97402	-	1401	1323	**RNA**→ rplM (PRK09216)Ribosomal_L13 (pfam00572)→ rpsI (PRK00132)Ribosomal_S9 (pfam00380)→
env-446	SRS018591_C1244473	-	270	192	**RNA**→ rplM (PRK09216)→
env-447	SRS063999_LANL_scaffold_89325	+	324	402	**RNA**→ rplM (PRK09216)Ribosomal_L13 (pfam00572)→ rpsI (PRK00132)Ribosomal_S9 (pfam00380)→
Pou-2-1	NZ_BAJQ01000079.1	-	3607	3529	**RNA**→ rplM (PRK09216)Ribosomal_L13 (pfam00572)→ rpsI (PRK00132)Ribosomal_S9 (pfam00380)→
env-448	AUXO010375897.1	+	341	415	**RNA**→ rplM (PRK09216)Ribosomal_L13 (pfam00572)→ rpsI (PRK00132)Ribosomal_S9 (pfam00380)→
env-449	SRS063603_C4098342	+	290	366	**RNA**→
Pba-2-1	NZ_AWEY01000020.1	-	92342	92266	**RNA**→ rplM (PRK09216)Ribosomal_L13 (pfam00572)→ rpsI (PRK00132)Ribosomal_S9 (pfam00380)→
Pba-1-1	NZ_AUFQ01000002.1	-	90788	90712	**RNA**→ rplM (PRK09216)Ribosomal_L13 (pfam00572)→ rpsI (PRK00132)Ribosomal_S9 (pfam00380)→
Pba-1-2	NZ_BAJF01000002.1	+	155272	155348	**RNA**→ rplM (PRK09216)Ribosomal_L13 (pfam00572)→ rpsI (PRK00132)Ribosomal_S9 (pfam00380)→
env-450	SRS049389_C4455900	+	102	178	**RNA**→
Pde-2-1	NZ_BAKG01000016.1	+	37012	37087	**RNA**→ rplM (PRK09216)Ribosomal_L13 (pfam00572)→ rpsI (PRK00132)Ribosomal_S9 (pfam00380)→
Pbe-1-1	NZ_GG704782.1	+	371273	371346	**RNA**→ rplM (PRK09216)Ribosomal_L13 (pfam00572)→ rpsI (PRK00132)Ribosomal_S9 (pfam00380)→
env-451	AUXO016035411.1	-	430	357	**RNA**→ rplM (PRK09216)Ribosomal_L13 (pfam00572)→
env-452	AUXO017177372.1	+	741	814	**RNA**→ rplM (PRK09216)Ribosomal_L13 (pfam00572)→ rpsI (PRK00132)Ribosomal_S9 (pfam00380)→
env-453	AUXO011185619.1	+	157	231	**RNA**→ rplM (PRK09216)Ribosomal_L13 (pfam00572)→ rpsI (PRK00132)Ribosomal_S9 (pfam00380)→
env-454	SRS022725_LANL_scaffold_110518	-	1243	1167	**RNA**→ rplM (PRK09216)Ribosomal_L13 (pfam00572)→ rpsI (PRK00132)Ribosomal_S9 (pfam00380)→
env-455	SRS047634_LANL_scaffold_138961	-	948	872	**RNA**→ rplM (PRK09216)Ribosomal_L13 (pfam00572)→ rpsI (PRK00132)Ribosomal_S9 (pfam00380)→
env-456	SRS063603_LANL_scaffold_20726	+	2347	2423	**RNA**→ rplM (PRK09216)Ribosomal_L13 (pfam00572)→ rpsI (PRK00132)Ribosomal_S9 (pfam00380)→
env-457	SRS058808_LANL_scaffold_70845	-	2078	2005	**RNA**→ rplM (PRK09216)Ribosomal_L13 (pfam00572)→ rpsI (PRK00132)Ribosomal_S9 (pfam00380)→
env-458	SRS022602_Baylor_scaffold_3137	+	97	171	**RNA**→ rplM (PRK09216)Ribosomal_L13 (pfam00572)→
env-459	SRS013252_Baylor_scaffold_54444	-	7007	6933	**RNA**→ rplM (PRK09216)Ribosomal_L13 (pfam00572)→ rpsI (PRK00132)Ribosomal_S9 (pfam00380)→
env-460	SRS017511_C3369784	-	87	13	**RNA**→ hypo→
env-461	SRS063603_LANL_scaffold_102327	+	763	837	**RNA**→
Psa-1-1	NZ_KB290984.1	-	62870	62796	**RNA**→ rplM (PRK09216)Ribosomal_L13 (pfam00572)→ rpsI (PRK00132)Ribosomal_S9 (pfam00380)→
env-462	SRS049268_C4307889	-	590	516	**RNA**→ rplM (PRK09216)Ribosomal_L13 (pfam00572)→
env-463	SRS017227_C5254572	-	533	459	**RNA**→ rplM (PRK09216)Ribosomal_L13 (pfam00572)→
env-464	SRS018665_C3785311	+	95	169	**RNA**→ rplM (PRK09216)Ribosomal_L13 (pfam00572)→ rpsI (PRK00132)Ribosomal_S9 (pfam00380)→
env-465	SRS051941_C3920117	-	311	237	**RNA**→ rplM (PRK09216)Ribosomal_L13 (pfam00572)→
env-466	HCF12C_28379	-	230	155	**RNA**→ hypo→
env-467	08_07_GOS_DNA_C8001741_1	+	143	218	**RNA**→ rplM (PRK09216)Ribosomal_L13 (pfam00572)→
env-468	AUXO013741021.1	+	250	324	**RNA**→ hypo→
env-469	AUXO016321942.1	+	109	183	**RNA**→ rplM (PRK09216)Ribosomal_L13 (pfam00572)→ rpsI (PRK00132)Ribosomal_S9 (pfam00380)→
env-470	AUXO016352636.1	+	21	94	**RNA**→ rplM (PRK09216)Ribosomal_L13 (pfam00572)→ rpsI (PRK00132)Ribosomal_S9 (pfam00380)→
Ppa-3-1	NZ_BAJH01000014.1	+	85099	85176	**RNA**→ rplM (PRK09216)Ribosomal_L13 (pfam00572)→ rpsI (PRK00132)Ribosomal_S9 (pfam00380)→
Ppa-2-1	NZ_KB890632.1	+	85379	85456	**RNA**→ rplM (PRK09216)Ribosomal_L13 (pfam00572)→ rpsI (PRK00132)Ribosomal_S9 (pfam00380)→
env-471	2205195008	+	38	115	**RNA**→
env-472	2157459302	+	38	115	**RNA**→
env-473	JGI20225J20221_1004075	+	804	881	**RNA**→ rplM (PRK09216)Ribosomal_L13 (pfam00572)→ rpsI (PRK00132)Ribosomal_S9 (pfam00380)→
env-474	BMHB3a_c149604	-	2595	2518	**RNA**→ rplM (PRK09216)Ribosomal_L13 (pfam00572)→ rpsI (PRK00132)Ribosomal_S9 (pfam00380)→
env-475	JGI20225J20221_1000003	-	388531	388454	**RNA**→ rplM (PRK09216)Ribosomal_L13 (pfam00572)→ rpsI (PRK00132)Ribosomal_S9 (pfam00380)→

env-476	BMHBC_68715	+	149	226	RNA→ rplM (PRK09216)Ribosomal_L13 (pfam00572)→ rpsI (PRK00132)Ribosomal_S9 (pfam00380)→
env-477	BMHBC_2_1802482	+	57	134	RNA→
env-478	BMHBC_2_3001399	+	57	134	RNA→
env-479	BMHBC_2_2370598	+	57	134	RNA→
env-480	BMHBC_2_1178038	+	57	134	RNA→
env-481	BMHBC_2_612469	+	57	134	RNA→
env-482	BMHBC_2_3578336	+	57	134	RNA→
env-483	HCE12Call500_c0056283	+	177	251	RNA→ rplM (PRK09216)Ribosomal_L13 (pfam00572)→ rpsI (PRK00132)Ribosomal_S9 (pfam00380)→
env-484	HCF12C_221933	-	28484	28410	RNA→ rplM (PRK09216)Ribosomal_L13 (pfam00572)→ rpsI (PRK00132)Ribosomal_S9 (pfam00380)→
env-485	HCF14C_11080	-	2194	2120	RNA→ rplM (PRK09216)Ribosomal_L13 (pfam00572)→ rpsI (PRK00132)Ribosomal_S9 (pfam00380)→
env-486	AUXO012434654.1	-	366	290	RNA→ rplM (PRK09216)RplM (COG0102)Ribosomal_L13 (cd00392)→
env-487	AUXO011866134.1	+	7	83	RNA→ ←hypo
env-488	AUXO017757733.1	-	1325	1250	RNA→ rplM (PRK09216)Ribosomal_L13 (pfam00572)→ rpsI (PRK00132)Ribosomal_S9 (pfam00380)→
env-489	AUXO011774349.1	-	771	700	RNA→ rplM (PRK09216)Ribosomal_L13 (pfam00572)→ rpsI (PRK00132)Ribosomal_S9 (pfam00380)→
env-490	SRS013542_LANL_scaffold_1766	-	69128	69055	RNA→ rplM (PRK09216)Ribosomal_L13 (pfam00572)→ rpsI (PRK00132)Ribosomal_S9 (pfam00380)→
env-491	SRS023604_Baylor_scaffold_50	+	320	393	RNA→ rplM (PRK09216)Ribosomal_L13 (pfam00572)→ rpsI (PRK00132)Ribosomal_S9 (pfam00380)→
env-492	SRS017497_Baylor_scaffold_193	-	91994	91921	RNA→ rplM (PRK09216)Ribosomal_L13 (pfam00572)→ rpsI (PRK00132)Ribosomal_S9 (pfam00380)→
env-493	SRS023468_LANL_scaffold_8569	+	176	249	RNA→ rplM (PRK09216)Ribosomal_L13 (pfam00572)→ rpsI (PRK00132)Ribosomal_S9 (pfam00380)→
Pam-1-1	NZ_ADFQ01000027.1	+	26891	26964	RNA→ rplM (PRK09216)Ribosomal_L13 (pfam00572)→ rplM (PRK09216)Ribosomal_L13 (pfam00572)→ rpsI (PRK00132)Ribosomal_S9 (pfam00380)→ rpsI (PRK00132)Ribosomal_S9 (pfam00380)→
Pam-2-1	NZ_JRNU01000014.1	-	45509	45436	RNA→ rplM (PRK09216)Ribosomal_L13 (pfam00572)→ rpsI (PRK00132)Ribosomal_S9 (pfam00380)→
Pam-3-1	NZ_KB905270.1	+	27167	27240	RNA→ rplM (PRK09216)Ribosomal_L13 (pfam00572)→ rpsI (PRK00132)Ribosomal_S9 (pfam00380)→
Pam-4-1	NZ_BAJP01000004.1	-	90359	90286	RNA→ rplM (PRK09216)Ribosomal_L13 (pfam00572)→ rpsI (PRK00132)Ribosomal_S9 (pfam00380)→
Pbi-1-1	NZ_JUWN01000307.1	+	2136	2209	RNA→ rplM (PRK09216)Ribosomal_L13 (pfam00572)→ rpsI (PRK00132)Ribosomal_S9 (pfam00380)→
Pbi-6-1	NZ_ADFO01000036.1	-	4358	4285	RNA→ rplM (PRK09216)Ribosomal_L13 (pfam00572)→ rplM (PRK09216)Ribosomal_L13 (pfam00572)→ rpsI (PRK00132)Ribosomal_S9 (pfam00380)→ rpsI (PRK00132)Ribosomal_S9 (pfam00380)→
Pbi-5-1	NZ_JH660660.1	+	893597	893670	RNA→ rplM (PRK09216)Ribosomal_L13 (pfam00572)→ rpsI (PRK00132)Ribosomal_S9 (pfam00380)→
Pbi-2-1	NZ_JRNF01000004.1	+	30225	30298	RNA→ rplM (PRK09216)Ribosomal_L13 (pfam00572)→ rpsI (PRK00132)Ribosomal_S9 (pfam00380)→
Pbi-4-1	NZ_JRNM01000029.1	+	14173	14246	RNA→ rplM (PRK09216)Ribosomal_L13 (pfam00572)→ rpsI (PRK00132)Ribosomal_S9 (pfam00380)→
Pbi-3-1	NZ_JRNQ01000001.1	-	38223	38150	RNA→ rplM (PRK09216)Ribosomal_L13 (pfam00572)→ rpsI (PRK00132)Ribosomal_S9 (pfam00380)→
Pve-1-1	NZ_KB898330.1	+	50120	50193	RNA→ rplM (PRK09216)Ribosomal_L13 (pfam00572)→ rpsI (PRK00132)Ribosomal_S9 (pfam00380)→
Pve-2-1	NZ_BAIP01000005.1	+	49830	49903	RNA→ rplM (PRK09216)Ribosomal_L13 (pfam00572)→ rpsI (PRK00132)Ribosomal_S9 (pfam00380)→
Pve-3-1	NZ_GG698713.1	-	155542	155469	RNA→ rplM (PRK09216)Ribosomal_L13 (pfam00572)→ rpsI (PRK00132)Ribosomal_S9 (pfam00380)→
env-494	SRS013836_C4770317	-	112	39	RNA→ hypo→
env-495	SRS014689_WUGC_scaffold_10524	-	158	84	RNA→ hypo→
env-496	SRS019045_WUGC_scaffold_55373	+	7079	7153	RNA→
env-497	SRS064329_LANL_scaffold_12626	+	135	209	RNA→ rplM (PRK09216)Ribosomal_L13 (pfam00572)→
Psc-1-1	NZ_BAKP01000004.1	+	58256	58330	RNA→ rplM (PRK09216)Ribosomal_L13 (pfam00572)→ rpsI (PRK00132)Ribosomal_S9 (pfam00380)→
env-498	SRS017808_Baylor_scaffold_59118	+	835	909	RNA→
env-499	SRS044373_WUGC_scaffold_55808	+	931	1005	RNA→
env-500	SRS053603_LANL_scaffold_86116	+	1113	1187	RNA→
Psp-6-1	NZ_KI259601.1	+	30175	30249	RNA→ rplM (PRK09216)Ribosomal_L13 (pfam00572)→ rpsI (PRK00132)Ribosomal_S9 (pfam00380)→
Pme-3-1	NZ_JRNS01000294.1	+	180	255	RNA→ rplM (PRK09216)Ribosomal_L13 (pfam00572)→ rpsI (PRK00132)Ribosomal_S9 (pfam00380)→
env-501	SRS024318_LANL_scaffold_11533	-	620	547	RNA→ rplM (PRK09216)Ribosomal_L13 (pfam00572)→ rpsI (PRK00132)Ribosomal_S9 (pfam00380)→
env-502	SRS017439_Baylor_scaffold_50412	-	108	36	RNA→
env-503	SRS011086_WUGC_scaffold_40282	-	108	36	RNA→ hypo→
env-504	SRS014124_WUGC_scaffold_14407	-	802	730	RNA→ rplM (PRK09216)Ribosomal_L13 (pfam00572)→ rplM (PRK09216)Ribosomal_L13 (pfam00572)→ rpsI (PRK00132)Ribosomal_S9 (pfam00380)→
env-505	SRS017533_Baylor_scaffold_7923	+	285	357	RNA→ rplM (PRK09216)Ribosomal_L13 (pfam00572)→
env-506	SRS049147_LANL_scaffold_62005	+	1217	1289	RNA→
Phi-1-1	NZ_JH376764.1	+	326001	326073	RNA→ hypo→ rplM (PRK09216)Ribosomal_L13 (pfam00572)→ rpsI (PRK00132)Ribosomal_S9 (pfam00380)→
env-507	SRS017076_C2635852	+	287	359	RNA→ hypo→
Phi-2-1	NZ_BAJX01000005.1	-	144658	144586	RNA→ rplM (PRK09216)Ribosomal_L13 (pfam00572)→ rpsI (PRK00132)Ribosomal_S9 (pfam00380)→

env-508	SRS013234_Baylor_scaffold_28796	-	148	76	**RNA**→
env-509	SRS024637_LANL_scaffold_306	+	443	515	**RNA**→ rplM (PRK09216)Ribosomal_L13 (pfam00572)→ rpsI (PRK00132)Ribosomal_S9 (pfam00380)→
Phi-2-2	NZ_JRNJ01000036.1	-	45722	45650	**RNA**→ rplM (PRK09216)Ribosomal_L13 (pfam00572)→ rpsI (PRK00132)Ribosomal_S9 (pfam00380)→
env-510	SRS014573_WUGC_scaffold_34708	-	1134	1060	**RNA**→ rplM (PRK09216)Ribosomal_L13 (pfam00572)→ rpsI (PRK00132)Ribosomal_S9 (pfam00380)→
env-511	SRS021496_Baylor_scaffold_22917	+	96	170	**RNA**→ rplM (PRK09216)Ribosomal_L13 (pfam00572)→
env-512	SRS015644_WUGC_scaffold_31522	-	876	802	**RNA**→ rplM (PRK09216)Ribosomal_L13 (pfam00572)→ rpsI (PRK00132)Ribosomal_S9 (pfam00380)→
env-513	SRS042131_C2183549	+	266	340	**RNA**→
env-514	SRS022719_LANL_scaffold_14486	+	176535	176609	**RNA**→ rplM (PRK09216)Ribosomal_L13 (pfam00572)→ rpsI (PRK00132)Ribosomal_S9 (pfam00380)→
env-515	SRS044662_LANL_scaffold_9271	+	199524	199598	**RNA**→ rplM (PRK09216)Ribosomal_L13 (pfam00572)→ rpsI (PRK00132)Ribosomal_S9 (pfam00380)→
env-516	SRS013879_C2814589	-	110	36	**RNA**→
env-517	SRS018145_Baylor_scaffold_16176	-	11602	11528	**RNA**→ rplM (PRK09216)Ribosomal_L13 (pfam00572)→ rpsI (PRK00132)Ribosomal_S9 (pfam00380)→
env-518	SRS057692_LANL_scaffold_51493	+	229	303	**RNA**→
env-519	SRS016002_WUGC_scaffold_30040	+	6147	6221	**RNA**→
Psp-5-1	NZ_JH114141.1	+	226443	226517	**RNA**→ rplM (PRK09216)Ribosomal_L13 (pfam00572)→ rpsI (PRK00132)Ribosomal_S9 (pfam00380)→
env-520	SRS017713_Baylor_scaffold_12488	-	158	84	**RNA**→ hypo→
env-521	SRS056622_LANL_scaffold_17923	-	158	84	**RNA**→ hypo→
env-522	SRS022625_Baylor_scaffold_4609	-	158	84	**RNA**→ hypo→
env-523	SRS016037_WUGC_scaffold_34392	+	1842	1916	**RNA**→ rplM (PRK09216)Ribosomal_L13 (pfam00572)→ rpsI (PRK00132)Ribosomal_S9 (pfam00380)→
env-524	SRS054687_LANL_scaffold_40466	-	149	75	**RNA**→ hypo→
env-525	SRS016569_Baylor_scaffold_12365	+	198	272	**RNA**→ rplM (PRK09216)Ribosomal_L13 (pfam00572)→ rplM (PRK09216)Ribosomal_L13 (pfam00572)→ rpsI (PRK00132)Ribosomal_S9 (pfam00380)→
env-526	SRS043663_LANL_scaffold_8456	+	196	270	**RNA**→ rplM (PRK09216)Ribosomal_L13 (pfam00572)→
env-527	SRS011086_WUGC_scaffold_9083	-	2222	2148	**RNA**→ rplM (PRK09216)Ribosomal_L13 (pfam00572)→ rpsI (PRK00132)Ribosomal_S9 (pfam00380)→
env-528	SRS024441_LANL_scaffold_8252	-	965	891	**RNA**→ rplM (PRK09216)Ribosomal_L13 (pfam00572)→ rpsI (PRK00132)Ribosomal_S9 (pfam00380)→
env-529	SRS045127_LANL_scaffold_25864	+	4	78	**RNA**→ rplM (PRK09216)Ribosomal_L13 (pfam00572)→
env-530	SRS052227_C2913848	-	999	925	**RNA**→ rplM (PRK09216)Ribosomal_L13 (pfam00572)→ rpsI (PRK00132)Ribosomal_S9 (pfam00380)→
Psp-10-1	NZ_JACD01000007.1	+	195948	196022	**RNA**→ rplM (PRK09216)Ribosomal_L13 (pfam00572)→ rpsI (PRK00132)Ribosomal_S9 (pfam00380)→
env-531	SRS018591_WUGC_scaffold_24424	+	1589	1663	**RNA**→
env-532	SRS013947_C1601091	-	178	104	**RNA**→ hypo→
env-533	SRS019120_C1333688	+	439	513	**RNA**→
env-534	SRS017209_Baylor_scaffold_33086	+	1225	1299	**RNA**→
env-535	SRS018394_C4052969	+	130	204	**RNA**→ rplM (PRK09216)Ribosomal_L13 (pfam00572)→ rpsI (PRK00132)Ribosomal_S9 (pfam00380)→
env-536	SRS055378_LANL_scaffold_77236	-	397	323	**RNA**→ rplM (PRK09216)Ribosomal_L13 (pfam00572)→
env-537	SRS013164_Baylor_scaffold_24536	-	1111	1037	**RNA**→ rplM (PRK09216)Ribosomal_L13 (pfam00572)→ rpsI (PRK00132)Ribosomal_S9 (pfam00380)→
env-538	SRS011243_Baylor_scaffold_14041	+	7051	7125	**RNA**→ rplM (PRK09216)Ribosomal_L13 (pfam00572)→ rpsI (PRK00132)Ribosomal_S9 (pfam00380)→
env-539	SRS011247_C1360241	+	440	514	**RNA**→ rplM (PRK09216)Ribosomal_L13 (pfam00572)→
env-540	SRS013502_Baylor_scaffold_7651	-	3320	3246	**RNA**→ rplM (PRK09216)Ribosomal_L13 (pfam00572)→ rpsI (PRK00132)Ribosomal_S9 (pfam00380)→
env-541	SRS013879_WUGC_scaffold_9024	-	172	98	**RNA**→
env-542	SRS014124_WUGC_scaffold_11710	-	948	874	**RNA**→ rplM (PRK09216)Ribosomal_L13 (pfam00572)→ rplM (PRK09216)Ribosomal_L13 (pfam00572)→ rpsI (PRK00132)Ribosomal_S9 (pfam00380)→ rpsI (PRK00132)Ribosomal_S9 (pfam00380)→
env-543	SRS014689_WUGC_scaffold_10523	-	158	84	**RNA**→
env-544	SRS016501_C1543222	+	266	340	**RNA**→ rplM (PRK09216)RplM (COG0102)→
env-545	SRS019022_WUGC_scaffold_10171	-	190	116	**RNA**→
env-546	SRS019045_WUGC_scaffold_18044	+	6417	6491	**RNA**→
env-547	SRS023557_Baylor_scaffold_343	-	20477	20403	**RNA**→ rplM (PRK09216)Ribosomal_L13 (pfam00572)→ rpsI (PRK00132)Ribosomal_S9 (pfam00380)→
env-548	SRS024580_LANL_scaffold_5027	+	1876	1950	**RNA**→ rplM (PRK09216)Ribosomal_L13 (pfam00572)→ rpsI (PRK00132)Ribosomal_S9 (pfam00380)→
env-549	SRS057355_LANL_scaffold_16577	-	735	661	**RNA**→ rplM (PRK09216)Ribosomal_L13 (pfam00572)→ rpsI (PRK00132)Ribosomal_S9 (pfam00380)→
env-550	SRS063288_LANL_scaffold_61854	+	267	341	**RNA**→ rplM (PRK09216)Ribosomal_L13 (pfam00572)→ rpsI (PRK00132)Ribosomal_S9 (pfam00380)→
env-551	SRS077736_LANL_scaffold_11170	-	5468	5394	**RNA**→ rplM (PRK09216)Ribosomal_L13 (pfam00572)→ rpsI (PRK00132)Ribosomal_S9 (pfam00380)→
Pme-2-1	NZ_GG740010.1	+	211066	211140	**RNA**→ rplM (PRK09216)Ribosomal_L13 (pfam00572)→ rplM (PRK09216)Ribosomal_L13 (pfam00572)→ rpsI (PRK00132)Ribosomal_S9 (pfam00380)→ rpsI (PRK00132)Ribosomal_S9 (pfam00380)→
Pme-1-1	NC_014370.1	-	173052	172978	**RNA**→ rplM (PRK09216)Ribosomal_L13 (pfam00572)→ rpsI (PRK00132)Ribosomal_S9 (pfam00380)→
env-552	SRS024447_LANL_scaffold_5458	-	189	115	**RNA**→ hypo→

env-553	SRS018739_WUGC_scaffold_1229	-	110	36	**RNA**→ hypo→
env-554	SRS021954_Baylor_scaffold_773	+	402	476	**RNA**→ rplM (PRK09216)Ribosomal_L13 (pfam00572)→
env-555	SRS020858_C742461	+	146	220	**RNA**→
env-556	SRS055426_LANL_scaffold_45579	-	373	299	**RNA**→ rplM (PRK09216)Ribosomal_L13 (pfam00572)→
env-557	SRS013818_Baylor_scaffold_15170	+	1030	1104	**RNA**→ rplM (PRK09216)Ribosomal_L13 (pfam00572)→
env-558	SRS022530_LANL_scaffold_8517	-	2577	2503	**RNA**→ rplM (PRK09216)Ribosomal_L13 (pfam00572)→ rpsI (PRK00132)Ribosomal_S9 (pfam00380)→
env-559	SRS018969_WUGC_scaffold_48352	-	1834	1760	**RNA**→ rplM (PRK09216)Ribosomal_L13 (pfam00572)→ rpsI (PRK00132)Ribosomal_S9 (pfam00380)→
env-560	SRS019029_C3094948	+	207	281	**RNA**→
env-561	SRS057539_LANL_scaffold_11258	+	1634	1708	**RNA**→ rplM (PRK09216)Ribosomal_L13 (pfam00572)→
Pfu-1-1	NZ_CP012074.1	-	335584	335511	**RNA**→ rplM (PRK09216)Ribosomal_L13 (pfam00572)→ rpsI (PRK00132)Ribosomal_S9 (pfam00380)→
Psp-24-1	NZ_AJIN01000058.1	+	75456	75530	**RNA**→ rplM (PRK09216)Ribosomal_L13 (pfam00572)→ rplM (PRK09216)Ribosomal_L13 (pfam00572)→ rpsI (PRK00132)Ribosomal_S9 (pfam00380)→ rpsI (PRK00132)Ribosomal_S9 (pfam00380)→
env-562	SRS018357_Baylor_scaffold_53049	+	659	733	**RNA**→
env-563	SRS044486_WUGC_scaffold_29347	+	207	281	**RNA**→
env-564	SRS042643_WUGC_scaffold_65829	+	2043	2117	**RNA**→
Pde-3-1	NZ_AEXO01000095.1	+	287046	287120	**RNA**→ rplM (PRK09216)Ribosomal_L13 (pfam00572)→ rplM (PRK09216)Ribosomal_L13 (pfam00572)→ rpsI (PRK00132)Ribosomal_S9 (pfam00380)→ rpsI (PRK00132)Ribosomal_S9 (pfam00380)→
env-565	SRS017691_C5408377	+	1907	1981	**RNA**→
Pde-4-1	NZ_JRNO01000001.1	-	3358	3284	**RNA**→ rplM (PRK09216)Ribosomal_L13 (pfam00572)→ rpsI (PRK00132)Ribosomal_S9 (pfam00380)→
Pde-5-1	NZ_ATWK01000001.1	+	151366	151440	**RNA**→ rplM (PRK09216)Ribosomal_L13 (pfam00572)→ rpsI (PRK00132)Ribosomal_S9 (pfam00380)→
Pde-5-2	NZ_BAJG01000001.1	+	303181	303255	**RNA**→ rplM (PRK09216)Ribosomal_L13 (pfam00572)→ rpsI (PRK00132)Ribosomal_S9 (pfam00380)→
env-566	SRS013836_Baylor_scaffold_11239	+	579	653	**RNA**→
env-567	SRS011255_Baylor_scaffold_46592	+	1416	1490	**RNA**→
Pde-6-1	NC_015311.1	+	832827	832901	**RNA**→ rplM (PRK09216)Ribosomal_L13 (pfam00572)→ rpsI (PRK00132)Ribosomal_S9 (pfam00380)→
env-568	SRS013836_C4811161	-	112	38	**RNA**→
env-569	SRS019029_WUGC_scaffold_26302	+	226	300	**RNA**→
Pmu-1-1	NZ_GL872283.1	+	37707	37781	**RNA**→ rplM (PRK09216)Ribosomal_L13 (pfam00572)→ rpsI (PRK00132)Ribosomal_S9 (pfam00380)→
env-570	AUXO010614209.1	+	217	289	**RNA**→
env-571	Supragingival_plaque_LANL_C181911197	-	124	49	**RNA**→ hypo→
env-572	SRS018739_C3456198	-	221	146	**RNA**→ hypo→
env-573	Supragingival_plaque_LANL_C182733701	+	533	608	**RNA**→
env-574	MA40A_contig28992	-	33539	33466	**RNA**→ rplM (PRK09216)Ribosomal_L13 (pfam00572)→ rpsI (PRK00132)Ribosomal_S9 (pfam00380)→
env-575	SRS023604_C759437	+	77	149	**RNA**→ rplM (PRK09216)Ribosomal_L13 (pfam00572)→
env-576	SRS023468_LANL_scaffold_9905	+	61199	61271	**RNA**→ rplM (PRK09216)Ribosomal_L13 (pfam00572)→ rpsI (PRK00132)Ribosomal_S9 (pfam00380)→
Psp-22-1	NZ_JRNC01000011.1	+	61278	61350	**RNA**→ rplM (PRK09216)Ribosomal_L13 (pfam00572)→ rpsI (PRK00132)Ribosomal_S9 (pfam00380)→
env-577	RUMENNODE_143372_1	-	3480	3407	**RNA**→ rplM (PRK09216)Ribosomal_L13 (pfam00572)→ rpsI (PRK00132)Ribosomal_S9 (pfam00380)→
env-578	SRS049318_LANL_scaffold_15105	-	3354	3278	**RNA**→ rplM (PRK09216)Ribosomal_L13 (pfam00572)→ rpsI (PRK00132)Ribosomal_S9 (pfam00380)→
env-579	SRS017691_C5027809	-	118	42	**RNA**→ hypo→
Pmi-3-1	NZ_JH594521.1	-	42001	41925	**RNA**→ rplM (PRK09216)Ribosomal_L13 (pfam00572)→ rpsI (PRK00132)Ribosomal_S9 (pfam00380)→
Pmi-1-1	NZ_KB892256.1	-	41862	41786	**RNA**→ rplM (PRK09216)Ribosomal_L13 (pfam00572)→ rpsI (PRK00132)Ribosomal_S9 (pfam00380)→
Pmi-2-1	NZ_BAKH01000001.1	+	67606	67682	**RNA**→ rplM (PRK09216)Ribosomal_L13 (pfam00572)→ rpsI (PRK00132)Ribosomal_S9 (pfam00380)→
env-580	SRS047634_LANL_scaffold_139898	+	902	978	**RNA**→ rplM (PRK09216)Ribosomal_L13 (pfam00572)→ rpsI (PRK00132)Ribosomal_S9 (pfam00380)→
env-581	SRS055378_C3881430	-	175	99	**RNA**→ hypo→
env-582	SRS022602_Baylor_scaffold_67890	-	3320	3244	**RNA**→ rplM (PRK09216)Ribosomal_L13 (pfam00572)→ rpsI (PRK00132)Ribosomal_S9 (pfam00380)→
Pin-1-1	NC_017861.1	-	192856	192781	**RNA**→ rplM (PRK09216)Ribosomal_L13 (pfam00572)→ rpsI (PRK00132)Ribosomal_S9 (pfam00380)→
env-583	SRS014692_WUGC_scaffold_15999	+	4242	4316	**RNA**→ rplM (PRK09216)Ribosomal_L13 (pfam00572)→ rpsI (PRK00132)Ribosomal_S9 (pfam00380)→
env-584	SRS014684_WUGC_scaffold_44181	+	3215	3289	**RNA**→
env-585	SRS014689_WUGC_scaffold_31060	+	16578	16652	**RNA**→
env-586	SRS019026_WUGC_scaffold_25915	-	1526	1452	**RNA**→ rplM (PRK09216)Ribosomal_L13 (pfam00572)→ rpsI (PRK00132)Ribosomal_S9 (pfam00380)→
env-587	SRS019027_C1975919	+	1325	1399	**RNA**→
env-588	SRS022530_LANL_scaffold_21668	-	23768	23694	**RNA**→ rplM (PRK09216)Ribosomal_L13 (pfam00572)→ rpsI (PRK00132)Ribosomal_S9 (pfam00380)→
env-589	SRS043663_C4103193	+	3216	3290	**RNA**→
env-590	SRS044373_WUGC_scaffold_63271	+	16814	16888	**RNA**→ rplM (PRK09216)Ribosomal_L13 (pfam00572)→ rpsI (PRK00132)Ribosomal_S9 (pfam00380)→

env-591	SRS048791_LANL_scaffold_39630	-	1512	1438	**RNA**→ rplM (PRK09216)Ribosomal_L13 (pfam00572)→ rpsI (PRK00132)Ribosomal_S9 (pfam00380)→
env-592	SRS047824_WUGC_scaffold_53533	-	614	540	**RNA**→ rplM (PRK09216)Ribosomal_L13 (pfam00572)→
env-593	SRS057205_LANL_scaffold_58665	+	670	744	**RNA**→ rplM (PRK09216)RplM (COG0102)→
env-594	SRS012279_Baylor_scaffold_56590	-	3134	3060	**RNA**→ rplM (PRK09216)Ribosomal_L13 (pfam00572)→ rpsI (PRK00132)Ribosomal_S9 (pfam00380)→
env-595	SRS011306_Baylor_scaffold_89003	-	10148	10074	**RNA**→ rplM (PRK09216)Ribosomal_L13 (pfam00572)→ rpsI (PRK00132)Ribosomal_S9 (pfam00380)→
env-596	SRS075404_LANL_scaffold_64891	+	238	312	**RNA**→
env-597	SRS023352_LANL_scaffold_62464	-	47439	47365	**RNA**→ rplM (PRK09216)Ribosomal_L13 (pfam00572)→ rpsI (PRK00132)Ribosomal_S9 (pfam00380)→
env-598	SRS063603_C4102126	+	332	406	**RNA**→
env-599	SRS064329_C2725335	+	3215	3289	**RNA**→
env-600	SRS062878_LANL_scaffold_70775	-	8062	7988	**RNA**→ rplM (PRK09216)Ribosomal_L13 (pfam00572)→ rpsI (PRK00132)Ribosomal_S9 (pfam00380)→
env-601	Subgingival_plaque_LANL_C11028051	+	219	293	**RNA**→
env-602	SRS014691_C2755366	+	2883	2957	**RNA**→
env-603	SRS015272_C3490354	+	339	413	**RNA**→
env-604	SRS015644_WUGC_scaffold_48472	+	933	1007	**RNA**→ rplM (PRK09216)Ribosomal_L13 (pfam00572)→ rpsI (PRK00132)Ribosomal_S9 (pfam00380)→
env-605	SRS018357_Baylor_scaffold_17708	-	24023	23949	**RNA**→ rplM (PRK09216)Ribosomal_L13 (pfam00572)→ rpsI (PRK00132)Ribosomal_S9 (pfam00380)→
env-606	SRS019974_Baylor_scaffold_54950	+	3215	3289	**RNA**→ rplM (PRK09216)Ribosomal_L13 (pfam00572)→ rpsI (PRK00132)Ribosomal_S9 (pfam00380)→
env-607	SRS023617_Baylor_scaffold_30918	+	5850	5924	**RNA**→ rplM (PRK09216)Ribosomal_L13 (pfam00572)→ rpsI (PRK00132)Ribosomal_S9 (pfam00380)→
env-608	SRS024375_C2325854	+	138	212	**RNA**→
env-609	SRS042131_WUGC_scaffold_50435	+	3411	3485	**RNA**→
env-610	SRS047210_WUGC_scaffold_57327	+	9864	9938	**RNA**→ rplM (PRK09216)Ribosomal_L13 (pfam00572)→ rpsI (PRK00132)Ribosomal_S9 (pfam00380)→
env-611	SRS050244_LANL_scaffold_37949	-	1334	1260	**RNA**→ rplM (PRK09216)Ribosomal_L13 (pfam00572)→ rpsI (PRK00132)Ribosomal_S9 (pfam00380)→
env-612	SRS053603_LANL_scaffold_49200	+	16206	16280	**RNA**→ rplM (PRK09216)Ribosomal_L13 (pfam00572)→ rpsI (PRK00132)Ribosomal_S9 (pfam00380)→
env-613	SRS017076_C2680416	+	454	528	**RNA**→
env-614	SRS013946_C747973	-	1186	1112	**RNA**→ rplM (PRK09216)Ribosomal_L13 (pfam00572)→ rpsI (PRK00132)Ribosomal_S9 (pfam00380)→
env-615	SRS014470_WUGC_scaffold_48816	-	9526	9452	**RNA**→ rplM (PRK09216)Ribosomal_L13 (pfam00572)→ rpsI (PRK00132)Ribosomal_S9 (pfam00380)→
env-616	SRS014573_C3755401	+	2926	3000	**RNA**→
env-617	SRS015038_WUGC_scaffold_38040	+	3215	3289	**RNA**→
env-618	SRS015057_WUGC_scaffold_8828	+	2155	2229	**RNA**→ rplM (PRK09216)Ribosomal_L13 (pfam00572)→ rpsI (PRK00132)Ribosomal_S9 (pfam00380)→
env-619	SRS015209_WUGC_scaffold_49105	+	45106	45180	**RNA**→ rplM (PRK09216)Ribosomal_L13 (pfam00572)→ rpsI (PRK00132)Ribosomal_S9 (pfam00380)→
env-620	SRS015893_WUGC_scaffold_32763	+	3119	3193	**RNA**→ rplM (PRK09216)Ribosomal_L13 (pfam00572)→ rpsI (PRK00132)Ribosomal_S9 (pfam00380)→
env-621	SRS016002_WUGC_scaffold_17242	+	35903	35977	**RNA**→ rplM (PRK09216)Ribosomal_L13 (pfam00572)→ rpsI (PRK00132)Ribosomal_S9 (pfam00380)→
env-622	SRS017120_Baylor_scaffold_72849	+	96	170	**RNA**→ rplM (PRK09216)Ribosomal_L13 (pfam00572)→ rpsI (PRK00132)Ribosomal_S9 (pfam00380)→
env-623	SRS017439_Baylor_scaffold_52553	-	1177	1103	**RNA**→ rplM (PRK09216)Ribosomal_L13 (pfam00572)→ rpsI (PRK00132)Ribosomal_S9 (pfam00380)→
env-624	SRS018300_Baylor_scaffold_51866	+	3216	3290	**RNA**→ rplM (PRK09216)Ribosomal_L13 (pfam00572)→ rpsI (PRK00132)Ribosomal_S9 (pfam00380)→
env-625	SRS018439_Baylor_scaffold_51883	-	3196	3122	**RNA**→ rplM (PRK09216)Ribosomal_L13 (pfam00572)→ rpsI (PRK00132)Ribosomal_S9 (pfam00380)→
env-626	SRS018739_WUGC_scaffold_60459	-	1177	1103	**RNA**→ rplM (PRK09216)Ribosomal_L13 (pfam00572)→ rpsI (PRK00132)Ribosomal_S9 (pfam00380)→
env-627	SRS019126_WUGC_scaffold_41627	-	1177	1103	**RNA**→ rplM (PRK09216)Ribosomal_L13 (pfam00572)→ rpsI (PRK00132)Ribosomal_S9 (pfam00380)→
env-628	SRS019127_C1673699	+	3216	3290	**RNA**→
env-629	SRS019219_WUGC_scaffold_22098	-	3099	3025	**RNA**→ rplM (PRK09216)Ribosomal_L13 (pfam00572)→ rpsI (PRK00132)Ribosomal_S9 (pfam00380)→
env-630	SRS020220_Baylor_scaffold_52465	-	1526	1452	**RNA**→ rplM (PRK09216)Ribosomal_L13 (pfam00572)→ rpsI (PRK00132)Ribosomal_S9 (pfam00380)→
env-631	SRS020856_Baylor_scaffold_35690	-	3199	3125	**RNA**→ rplM (PRK09216)Ribosomal_L13 (pfam00572)→ rpsI (PRK00132)Ribosomal_S9 (pfam00380)→
env-632	SRS022143_WUGC_scaffold_32909	-	9534	9460	**RNA**→ rplM (PRK09216)Ribosomal_L13 (pfam00572)→ rpsI (PRK00132)Ribosomal_S9 (pfam00380)→
env-633	SRS022621_C4119557	+	2940	3014	**RNA**→
env-634	SRS024318_LANL_scaffold_74074	-	11441	11367	**RNA**→ rplM (PRK09216)Ribosomal_L13 (pfam00572)→ rpsI (PRK00132)Ribosomal_S9 (pfam00380)→
env-635	SRS024580_C3038766	-	171	97	**RNA**→
env-636	SRS024637_LANL_scaffold_30251	+	233	307	**RNA**→
env-637	SRS042910_C5625461	+	3216	3290	**RNA**→
env-638	SRS045715_LANL_scaffold_89135	+	3431	3505	**RNA**→ rplM (PRK09216)Ribosomal_L13 (pfam00572)→
env-639	SRS049147_LANL_scaffold_58601	+	95	169	**RNA**→ rplM (PRK09216)Ribosomal_L13 (pfam00572)→ rpsI (PRK00132)Ribosomal_S9 (pfam00380)→
env-640	SRS053854_C1915023	+	140	214	**RNA**→
env-641	SRS054687_C3415499	-	171	97	**RNA**→ hypo→
env-642	SRS055426_LANL_scaffold_56878	+	12351	12425	**RNA**→ rplM (PRK09216)Ribosomal_L13 (pfam00572)→

env-643	SRS056622_LANL_scaffold_38514	-	47437	47363	**RNA**→ rplM (PRK09216)Ribosomal_L13 (pfam00572)→ rpsI (PRK00132)Ribosomal_S9 (pfam00380)→
env-644	SRS058336_C2178143	+	3215	3289	**RNA**→
env-645	SRS063193_LANL_scaffold_53070	+	2926	3000	**RNA**→ rplM (PRK09216)Ribosomal_L13 (pfam00572)→ rpsI (PRK00132)Ribosomal_S9 (pfam00380)→
env-646	SRS064423_LANL_scaffold_66525	-	11227	11153	**RNA**→ rplM (PRK09216)Ribosomal_L13 (pfam00572)→ rpsI (PRK00132)Ribosomal_S9 (pfam00380)→
env-647	SRS065278_C2564619	+	3215	3289	**RNA**→
Ppa-1-1	NZ_GL982513.1	+	723490	723564	**RNA**→ rplM (PRK09216)Ribosomal_L13 (pfam00572)→ rpsI (PRK00132)Ribosomal_S9 (pfam00380)→
env-648	SRS018791_WUGC_scaffold_38475	-	1334	1260	**RNA**→ rplM (PRK09216)Ribosomal_L13 (pfam00572)→ rpsI (PRK00132)Ribosomal_S9 (pfam00380)→
env-649	SRS022077_Baylor_scaffold_52460	-	1526	1452	**RNA**→ rplM (PRK09216)Ribosomal_L13 (pfam00572)→ rpsI (PRK00132)Ribosomal_S9 (pfam00380)→
env-650	SRS024015_Baylor_scaffold_54468	-	5401	5327	**RNA**→ rplM (PRK09216)Ribosomal_L13 (pfam00572)→ rpsI (PRK00132)Ribosomal_S9 (pfam00380)→
env-651	SRS016319_WUGC_scaffold_54116	+	3215	3289	**RNA**→ rplM (PRK09216)Ribosomal_L13 (pfam00572)→ rpsI (PRK00132)Ribosomal_S9 (pfam00380)→
env-652	SRS047219_WUGC_scaffold_57943	+	16790	16864	**RNA**→ rplM (PRK09216)Ribosomal_L13 (pfam00572)→ rpsI (PRK00132)Ribosomal_S9 (pfam00380)→
env-653	Buccal_mucosa_LANL_C64807355	-	110	36	**RNA**→ hypo→
env-654	Contig7238817.1	-	110	36	**RNA**→
env-655	SRS015434_C5938921	-	110	36	**RNA**→
env-656	SRS017808_Baylor_scaffold_37180	+	3883	3957	**RNA**→
env-657	SRS016740_C2751815	-	117	43	**RNA**→
env-658	SRS023930_C2173199	-	90	16	**RNA**→
env-659	SRS013705_Baylor_scaffold_102904	+	23297	23371	**RNA**→
env-660	SRS013879_C2856030	+	1392	1466	**RNA**→
env-661	SRS015762_WUGC_scaffold_41259	+	774	848	**RNA**→ rplM (PRK09216)Ribosomal_L13 (pfam00572)→ rpsI (PRK00132)Ribosomal_S9 (pfam00380)→
env-662	SRS015797_WUGC_scaffold_34883	-	23901	23827	**RNA**→ rplM (PRK09216)Ribosomal_L13 (pfam00572)→ rpsI (PRK00132)Ribosomal_S9 (pfam00380)→
env-663	SRS015941_WUGC_scaffold_78227	+	3216	3290	**RNA**→ rplM (PRK09216)Ribosomal_L13 (pfam00572)→ rpsI (PRK00132)Ribosomal_S9 (pfam00380)→
env-664	SRS016086_WUGC_scaffold_26855	-	171	97	**RNA**→
env-665	SRS016225_C2803421	-	171	97	**RNA**→ hypo→
env-666	SRS016569_Baylor_scaffold_56485	+	16533	16607	**RNA**→ rplM (PRK09216)Ribosomal_L13 (pfam00572)→ rpsI (PRK00132)Ribosomal_S9 (pfam00380)→
env-667	SRS021496_Baylor_scaffold_85386	-	12316	12242	**RNA**→ rplM (PRK09216)Ribosomal_L13 (pfam00572)→ rpsI (PRK00132)Ribosomal_S9 (pfam00380)→
env-668	SRS021954_C2960878	+	284	358	**RNA**→
env-669	SRS023926_C2818602	+	2890	2964	**RNA**→
env-670	SRS024081_LANL_scaffold_39378	+	40034	40108	**RNA**→ rplM (PRK09216)Ribosomal_L13 (pfam00572)→ rpsI (PRK00132)Ribosomal_S9 (pfam00380)→
env-671	SRS042643_WUGC_scaffold_26426	-	1334	1260	**RNA**→ rplM (PRK09216)Ribosomal_L13 (pfam00572)→ rpsI (PRK00132)Ribosomal_S9 (pfam00380)→
env-672	SRS051791_LANL_scaffold_12138	-	47738	47664	**RNA**→ rplM (PRK09216)Ribosomal_L13 (pfam00572)→ rpsI (PRK00132)Ribosomal_S9 (pfam00380)→
env-673	SRS057539_LANL_scaffold_35057	+	49731	49805	**RNA**→ rplM (PRK09216)Ribosomal_L13 (pfam00572)→ rpsI (PRK00132)Ribosomal_S9 (pfam00380)→
env-674	SRS063288_C2542184	+	84	158	**RNA**→ rplM (PRK09216)Ribosomal_L13 (pfam00572)→
env-675	SRS064774_LANL_scaffold_17002	-	16294	16220	**RNA**→ rplM (PRK09216)Ribosomal_L13 (pfam00572)→ rpsI (PRK00132)Ribosomal_S9 (pfam00380)→
env-676	UnmappedStool_Broad_C252508257	-	155	79	**RNA**→ hypo→
Pco-3-1	NZ_AUME01000007.1	-	36087	36011	**RNA**→ rplM (PRK09216)Ribosomal_L13 (pfam00572)→ rpsI (PRK00132)Ribosomal_S9 (pfam00380)→
Pco-3-2	NZ_BAIT01000003.1	+	74722	74798	**RNA**→ rplM (PRK09216)Ribosomal_L13 (pfam00572)→ rpsI (PRK00132)Ribosomal_S9 (pfam00380)→
Pni-1-1	NZ_GL982464.1	+	2020144	2020219	**RNA**→ rplM (PRK09216)Ribosomal_L13 (pfam00572)→ rplM (PRK09216)Ribosomal_L13 (pfam00572)→ rpsI (PRK00132)Ribosomal_S9 (pfam00380)→
env-677	SRS012285_Baylor_scaffold_36585	+	18214	18289	**RNA**→ rplM (PRK09216)Ribosomal_L13 (pfam00572)→ rpsI (PRK00132)Ribosomal_S9 (pfam00380)→
env-678	SRS022083_C1814115	-	190	115	**RNA**→ hypo→
env-679	SRS024144_C1709965	-	224	149	**RNA**→ rplM (PRK09216)RplM (COG0102)Ribosomal_L13 (cd00392)Ribosomal_L13 (pfam00572)→
env-680	SRS063932_LANL_scaffold_105725	+	15855	15930	**RNA**→
env-681	Subgingival_plaque_LANL_C10982129	-	147	72	**RNA**→ hypo→
env-682	SRS022725_LANL_scaffold_18998	-	20108	20033	**RNA**→ rplM (PRK09216)Ribosomal_L13 (pfam00572)→ rpsI (PRK00132)Ribosomal_S9 (pfam00380)→
env-683	SRS063215_C2206217	+	314	389	**RNA**→ hypo→
env-684	SRS045313_LANL_scaffold_12927	+	11427	11502	**RNA**→
env-685	SRS058053_LANL_scaffold_46648	+	2496	2571	**RNA**→ rplM (PRK09216)Ribosomal_L13 (pfam00572)→ rpsI (PRK00132)Ribosomal_S9 (pfam00380)→
env-686	SRS015215_WUGC_scaffold_60845	+	31443	31518	**RNA**→
env-687	SRS016575_C3771021	-	171	96	**RNA**→ hypo→
env-688	SRS017227_Baylor_scaffold_128981	-	4583	4508	**RNA**→ rplM (PRK09216)Ribosomal_L13 (pfam00572)→ rpsI (PRK00132)Ribosomal_S9 (pfam00380)→
env-689	SRS018573_C2468474	-	171	96	**RNA**→ hypo→

env-690	SRS045197_C3607732	+	2688	2763	**RNA**→
env-691	SRS047113_C5438044	+	4885	4960	**RNA**→
env-692	SRS049318_LANL_scaffold_180129	+	334	409	**RNA**→ rplM (PRK09216)Ribosomal_L13 (pfam00572)→
env-693	SRS051930_C3291029	+	3901	3976	**RNA**→
env-694	SRS057022_LANL_scaffold_21753	+	985	1060	**RNA**→ rplM (PRK09216)Ribosomal_L13 (pfam00572)→
env-695	SRS064449_C4104329	+	731	806	**RNA**→
env-696	SRS014691_C2755332	-	159	84	**RNA**→ hypo→
env-697	SRS019029_WUGC_scaffold_30607	-	318	243	**RNA**→ rplM (PRK09216)Ribosomal_L13 (pfam00572)→
env-698	SRS011255_Baylor_scaffold_75811	-	105	30	**RNA**→
env-699	SRS023938_C2346941	-	137	62	**RNA**→
Pni-3-1	NZ_ADGJ01000034.1	+	4034	4109	**RNA**→ rplM (PRK09216)Ribosomal_L13 (pfam00572)→ rpsI (PRK00132)Ribosomal_S9 (pfam00380)→
env-700	SRS013533_PGA_scaffold_55845	+	31911	31986	**RNA**→ ←hypo
env-701	SRS013950_WUGC_scaffold_19463	+	6777	6852	**RNA**→ rplM (PRK09216)Ribosomal_L13 (pfam00572)→ rpsI (PRK00132)Ribosomal_S9 (pfam00380)→
env-702	SRS015574_C3429056	+	765	840	**RNA**→
env-703	SRS019980_Baylor_scaffold_52314	-	426	351	**RNA**→ rplM (PRK09216)Ribosomal_L13 (pfam00572)→
env-704	SRS022536_LANL_scaffold_126332	+	32859	32934	**RNA**→ rplM (PRK09216)Ribosomal_L13 (pfam00572)→ rpsI (PRK00132)Ribosomal_S9 (pfam00380)→
env-705	SRS023595_C7191239	-	113	38	**RNA**→
env-706	SRS024087_LANL_scaffold_6797	-	49193	49118	**RNA**→ rplM (PRK09216)Ribosomal_L13 (pfam00572)→ rpsI (PRK00132)Ribosomal_S9 (pfam00380)→
env-707	SRS058808_C2875695	-	162	87	**RNA**→
env-708	SRS065099_LANL_scaffold_67594	-	171	96	**RNA**→ hypo→
Pni-2-1	NZ_KI669452.1	-	66638	66563	**RNA**→ rplM (PRK09216)Ribosomal_L13 (pfam00572)→ rpsI (PRK00132)Ribosomal_S9 (pfam00380)→
env-709	SRS057355_C2357028	+	1923	1998	**RNA**→
env-710	SRS014573_C3689837	+	580	655	**RNA**→
env-711	SRS013164_Baylor_scaffold_6044	+	24960	25035	**RNA**→ rplM (PRK09216)Ribosomal_L13 (pfam00572)→ rpsI (PRK00132)Ribosomal_S9 (pfam00380)→
env-712	SRS015434_C5939871	+	1383	1458	**RNA**→ hypo→
env-713	SRS016037_WUGC_scaffold_40495	-	1517	1442	**RNA**→ rplM (PRK09216)Ribosomal_L13 (pfam00572)→ rpsI (PRK00132)Ribosomal_S9 (pfam00380)→
env-714	SRS017209_C2612132	-	171	96	**RNA**→ hypo→
env-715	SRS018145_Baylor_scaffold_45351	+	15741	15816	**RNA**→ rplM (PRK09216)Ribosomal_L13 (pfam00572)→ rpsI (PRK00132)Ribosomal_S9 (pfam00380)→
env-716	SRS019071_WUGC_scaffold_1653	-	393	318	**RNA**→ rplM (PRK09216)Ribosomal_L13 (pfam00572)→
env-717	SRS019327_WUGC_scaffold_7031	-	9945	9870	**RNA**→ rplM (PRK09216)Ribosomal_L13 (pfam00572)→ rpsI (PRK00132)Ribosomal_S9 (pfam00380)→
env-718	SRS022719_LANL_scaffold_29884	-	15205	15130	**RNA**→ rplM (PRK09216)Ribosomal_L13 (pfam00572)→ rpsI (PRK00132)Ribosomal_S9 (pfam00380)→
env-719	SRS023958_Baylor_scaffold_35671	+	24960	25035	**RNA**→ rplM (PRK09216)Ribosomal_L13 (pfam00572)→ rpsI (PRK00132)Ribosomal_S9 (pfam00380)→
env-720	SRS044662_LANL_scaffold_26917	-	15127	15052	**RNA**→ rplM (PRK09216)Ribosomal_L13 (pfam00572)→ rpsI (PRK00132)Ribosomal_S9 (pfam00380)→
env-721	SRS049389_C4741864	+	3211	3286	**RNA**→ hypo→
env-722	SRS050669_C2732573	-	171	96	**RNA**→ hypo→
env-723	SRS057692_LANL_scaffold_59063	+	15741	15816	**RNA**→ rplM (PRK09216)Ribosomal_L13 (pfam00572)→ rpsI (PRK00132)Ribosomal_S9 (pfam00380)→
env-724	SRS043422_C2606710	-	418	343	**RNA**→ rplM (PRK09216)Ribosomal_L13 (pfam00572)→
Pau-1-1	NZ_BAKF01000067.1	-	163	88	**RNA**→
Psp-9-1	NZ_JIAF01000004.1	-	988952	988877	**RNA**→ rplM (PRK09216)Ribosomal_L13 (pfam00572)→ rpsI (PRK00132)Ribosomal_S9 (pfam00380)→
env-725	SRS055401_C2262988	-	171	96	**RNA**→ hypo→
env-726	SRS016746_C3112314	-	171	96	**RNA**→ hypo→
env-727	Contig7208859.1	-	113	38	**RNA**→
env-728	SRS048411_C6994262	+	2390	2465	**RNA**→
env-729	SRS013723_C3473767	+	936	1011	**RNA**→ hypo→
env-730	SRS020226_C3504182	+	3062	3137	**RNA**→ hypo→
env-731	SRS022149_C2844760	-	185	110	**RNA**→ hypo→
env-732	SRS023595_Baylor_scaffold_12538	-	315	240	**RNA**→ rplM (PRK09216)Ribosomal_L13 (pfam00572)→
env-733	SRS017511_Baylor_scaffold_37568	-	3386	3311	**RNA**→ rplM (PRK09216)Ribosomal_L13 (pfam00572)→ rpsI (PRK00132)Ribosomal_S9 (pfam00380)→
env-734	SRS062761_C4185168	-	157	82	**RNA**→ hypo→
env-735	SRS063603_LANL_scaffold_16033	+	3063	3138	**RNA**→ rplM (PRK09216)Ribosomal_L13 (pfam00572)→ rpsI (PRK00132)Ribosomal_S9 (pfam00380)→
env-736	SRS049268_C4330408	+	785	860	**RNA**→ hypo→
Pin-3-1	NZ_ATMK01000012.1	-	3560	3485	**RNA**→ rplM (PRK09216)Ribosomal_L13 (pfam00572)→ rpsI (PRK00132)Ribosomal_S9 (pfam00380)→

Pfa-1-1	NZ_BAJY01000023.1	+	18148	18223	**RNA**→ rplM (PRK09216)Ribosomal_L13 (pfam00572)→ rpsI (PRK00132)Ribosomal_S9 (pfam00380)→
env-737	SRS049318_LANL_scaffold_111644	+	3483	3558	**RNA**→ rplM (PRK09216)Ribosomal_L13 (pfam00572)→ rpsI (PRK00132)Ribosomal_S9 (pfam00380)→
Pin-2-1	NZ_KE392224.1	+	52909	52984	**RNA**→ rplM (PRK09216)Ribosomal_L13 (pfam00572)→ rpsI (PRK00132)Ribosomal_S9 (pfam00380)→
env-738	SRS014474_C725551	+	364	439	**RNA**→
env-739	SRS014477_C1641189	+	1209	1284	**RNA**→
env-740	SRS015061_C521155	-	3425	3350	**RNA**→ rplM (PRK09216)Ribosomal_L13 (pfam00572)→ rpsI (PRK00132)Ribosomal_S9 (pfam00380)→
env-741	SRS015062_WUGC_scaffold_566	-	3425	3350	**RNA**→ rplM (PRK09216)Ribosomal_L13 (pfam00572)→ rpsI (PRK00132)Ribosomal_S9 (pfam00380)→
env-742	SRS047634_C4505245	+	2722	2797	**RNA**→
Pdi-3-1	NZ_KI259320.1	-	18576	18501	**RNA**→ rplM (PRK09216)Ribosomal_L13 (pfam00572)→ rpsI (PRK00132)Ribosomal_S9 (pfam00380)→
env-743	SRS017521_Baylor_scaffold_47871	-	90	15	**RNA**→
Pdi-2-1	NZ_AEDO01000037.1	+	25023	25098	**RNA**→ rplM (PRK09216)Ribosomal_L13 (pfam00572)→ rplM (PRK09216)Ribosomal_L13 (pfam00572)→ rpsI (PRK00132)Ribosomal_S9 (pfam00380)→ rpsI (PRK00132)Ribosomal_S9 (pfam00380)→
Pdi-1-1	NZ_JRNR01000099.1	-	3529	3454	**RNA**→ rplM (PRK09216)Ribosomal_L13 (pfam00572)→ rpsI (PRK00132)Ribosomal_S9 (pfam00380)→
env-744	NOM017_scaffold152_1	+	2821	2896	**RNA**→
env-745	4448812.3_Contig1872	+	367	442	**RNA**→ rplM (PRK09216)Ribosomal_L13 (pfam00572)→ rpsI (PRK00132)Ribosomal_S9 (pfam00380)→
env-746	DLM003_scaffold159_2	+	2840	2915	**RNA**→ rplM (PRK09216)Ribosomal_L13 (pfam00572)→ rpsI (PRK00132)Ribosomal_S9 (pfam00380)→
env-747	NOM022_scaffold34272_1	+	2699	2774	**RNA**→ rplM (PRK09216)Ribosomal_L13 (pfam00572)→ rpsI (PRK00132)Ribosomal_S9 (pfam00380)→
env-748	contig104641	+	10740	10815	**RNA**→ rplM (PRK09216)Ribosomal_L13 (pfam00572)→
env-749	DLM001_scaffold46771_1	-	555	480	**RNA**→ rplM (PRK09216)Ribosomal_L13 (pfam00572)→
env-750	DOM003_scaffold20629_2	+	164	239	**RNA**→ rplM (PRK09216)Ribosomal_L13 (pfam00572)→
env-751	NLM001_scaffold44488_1	+	2699	2774	**RNA**→ rplM (PRK09216)Ribosomal_L13 (pfam00572)→ rpsI (PRK00132)Ribosomal_S9 (pfam00380)→
env-752	SRS011271_WUGC_scaffold_12531	+	603	678	**RNA**→
env-753	4448818.3_Contig13931	-	222	147	**RNA**→ rplM (PRK09216)→
env-754	NLM006_scaffold2689_1	-	559	484	**RNA**→ rplM (PRK09216)Ribosomal_L13 (pfam00572)→
env-755	SRS015794_Baylor_scaffold_8969	+	743	818	**RNA**→ rplM (PRK09216)Ribosomal_L13 (pfam00572)→
env-756	DOF011_scaffold4575_1	+	2870	2945	**RNA**→ rplM (PRK09216)Ribosomal_L13 (pfam00572)→ rpsI (PRK00132)Ribosomal_S9 (pfam00380)→
env-757	NLM004_scaffold39342_1	-	326	250	**RNA**→ rplM (PRK09216)Ribosomal_L13 (pfam00572)→
env-758	DLF003_scaffold48930_1	+	25126	25201	**RNA**→ rplM (PRK09216)Ribosomal_L13 (pfam00572)→
env-759	NLM004_scaffold4959_1	-	319	244	**RNA**→ rplM (PRK09216)Ribosomal_L13 (pfam00572)→
env-760	NOM012_scaffold33926_2	-	272	197	**RNA**→ rplM (PRK09216)Ribosomal_L13 (pfam00572)→
env-761	scaffold3211_3_V1.CD-2	+	538	613	**RNA**→
env-762	SRS019910_WUGC_scaffold_54703	+	204	279	**RNA**→
env-763	DLM001_scaffold46772_1	-	551	476	**RNA**→ rplM (PRK09216)Ribosomal_L13 (pfam00572)→
env-764	DOM016_scaffold20994_2	+	58303	58378	**RNA**→ rplM (PRK09216)Ribosomal_L13 (pfam00572)→ rpsI (PRK00132)Ribosomal_S9 (pfam00380)→
env-765	SRS013521_LANL_scaffold_8254	-	121	46	**RNA**→
env-766	NLM016_scaffold16524_1	-	542	467	**RNA**→ rplM (PRK09216)Ribosomal_L13 (pfam00572)→
env-767	4491403.3_NODE_17602	-	322	247	**RNA**→ rplM (PRK09216)Ribosomal_L13 (pfam00572)→
env-768	SRS049959_WUGC_scaffold_5722	-	414	338	**RNA**→ rplM (PRK09216)Ribosomal_L13 (pfam00572)→
env-769	scaffold28598_1_V1.UC-17	-	126	50	**RNA**→
env-770	scaffold2660_2_MH0049	+	139	215	**RNA**→ rplM (PRK09216)Ribosomal_L13 (pfam00572)→
env-771	4491403.3_NODE_60160	-	980	904	**RNA**→ rplM (PRK09216)Ribosomal_L13 (pfam00572)→ rpsI (PRK00132)Ribosomal_S9 (pfam00380)→
env-772	SRS013521_LANL_scaffold_15414	-	158	82	**RNA**→ hypo→
env-773	SRS049995_LANL_scaffold_36980	+	752	828	**RNA**→
env-774	BAAX01002455.1	+	95	171	**RNA**→ rplM (PRK09216)Ribosomal_L13 (pfam00572)→ rpsI (PRK00132)Ribosomal_S9 (pfam00380)→ rpsI (PRK00132)Ribosomal_S9 (pfam00380)→
env-775	SRS015794_C2240237	+	338	414	**RNA**→
env-776	DLM006_scaffold20724_1	+	4613	4689	**RNA**→ rplM (PRK09216)Ribosomal_L13 (pfam00572)→ rpsI (PRK00132)Ribosomal_S9 (pfam00380)→
env-777	NLF006_scaffold9001_1	-	11920	11844	**RNA**→ rplM (PRK09216)Ribosomal_L13 (pfam00572)→ rpsI (PRK00132)Ribosomal_S9 (pfam00380)→
env-778	NLM002_scaffold28362_1	+	827	903	**RNA**→ rplM (PRK09216)Ribosomal_L13 (pfam00572)→ rpsI (PRK00132)Ribosomal_S9 (pfam00380)→
env-779	NLM004_scaffold34280_1	+	818	894	**RNA**→ rplM (PRK09216)Ribosomal_L13 (pfam00572)→
env-780	NLM006_scaffold7359_1	+	197	273	**RNA**→ rplM (PRK09216)Ribosomal_L13 (pfam00572)→
env-781	DLM002_scaffold24298_1	+	12011	12086	**RNA**→ rplM (PRK09216)Ribosomal_L13 (pfam00572)→ rpsI (PRK00132)Ribosomal_S9 (pfam00380)→
env-782	NOF010_scaffold54121_1	+	59215	59290	**RNA**→ rplM (PRK09216)Ribosomal_L13 (pfam00572)→ rpsI (PRK00132)Ribosomal_S9 (pfam00380)→

env-783	NOM009_scaffold53764_1	+	59215	59290	**RNA**→ rplM (PRK09216)Ribosomal_L13 (pfam00572)→ rpsI (PRK00132)Ribosomal_S9 (pfam00380)→
env-784	NOM017_scaffold40285_1	+	1439	1514	**RNA**→ rplM (PRK09216)Ribosomal_L13 (pfam00572)→
env-785	NLM004_scaffold29467_1	+	19562	19637	**RNA**→ rplM (PRK09216)Ribosomal_L13 (pfam00572)→
env-786	SRS022609_Baylor_scaffold_13243	+	21067	21142	**RNA**→ rplM (PRK09216)Ribosomal_L13 (pfam00572)→ rpsI (PRK00132)Ribosomal_S9 (pfam00380)→
env-787	4491403.3_NODE_11716	+	384	459	**RNA**→
env-788	DLM027_scaffold23115_4	+	11864	11939	**RNA**→ rplM (PRK09216)Ribosomal_L13 (pfam00572)→ rpsI (PRK00132)Ribosomal_S9 (pfam00380)→
env-789	4448819.3_Contig631	-	7314	7239	**RNA**→ rplM (PRK09216)Ribosomal_L13 (pfam00572)→ rpsI (PRK00132)Ribosomal_S9 (pfam00380)→
env-790	4448812.3_Contig639	+	2967	3042	**RNA**→ rplM (PRK09216)Ribosomal_L13 (pfam00572)→ rpsI (PRK00132)Ribosomal_S9 (pfam00380)→
env-791	scaffold18566_1_MH0009	-	109	34	**RNA**→
env-792	NLM016_scaffold16376_1	-	143	68	**RNA**→
env-793	scaffold58558_1_MH0065	+	213	288	**RNA**→ rplM (PRK09216)Ribosomal_L13 (pfam00572)→ rpsI (PRK00132)Ribosomal_S9 (pfam00380)→
env-794	4491477.3_NODE_2169	-	8743	8668	**RNA**→ rplM (PRK09216)Ribosomal_L13 (pfam00572)→ rpsI (PRK00132)Ribosomal_S9 (pfam00380)→
env-795	SRS015782_WUGC_scaffold_26114	-	11843	11768	**RNA**→ rplM (PRK09216)Ribosomal_L13 (pfam00572)→ rpsI (PRK00132)Ribosomal_S9 (pfam00380)→
env-796	SRS024132_Baylor_scaffold_32360	-	11900	11825	**RNA**→ rplM (PRK09216)Ribosomal_L13 (pfam00572)→ rpsI (PRK00132)Ribosomal_S9 (pfam00380)→
env-797	scaffold43386_1_MH0050	+	966	1041	**RNA**→
env-798	4448813.3_Contig493	-	5921	5846	**RNA**→ rplM (PRK09216)Ribosomal_L13 (pfam00572)→ rpsI (PRK00132)Ribosomal_S9 (pfam00380)→
env-799	NOF001_scaffold377_4	-	5305	5230	**RNA**→ rplM (PRK09216)Ribosomal_L13 (pfam00572)→ rpsI (PRK00132)Ribosomal_S9 (pfam00380)→
env-800	CAM_READ_0099651871	-	384	309	**RNA**→ rplM (PRK09216)Ribosomal_L13 (pfam00572)→
env-801	SRS047044_WUGC_scaffold_16328	+	257	332	**RNA**→ rplM (PRK09216)Ribosomal_L13 (pfam00572)→
env-802	4444165.3.02693	+	265	340	**RNA**→ rplM (PRK09216)Ribosomal_L13 (pfam00572)→
env-803	4448807.3_Contig1253	-	1893	1817	**RNA**→ rplM (PRK09216)Ribosomal_L13 (pfam00572)→ rpsI (PRK00132)Ribosomal_S9 (pfam00380)→
env-804	NLM008_scaffold29501_1	-	12251	12176	**RNA**→ rplM (PRK09216)Ribosomal_L13 (pfam00572)→ rpsI (PRK00132)Ribosomal_S9 (pfam00380)→
env-805	NLM024_scaffold18343_2	+	16425	16500	**RNA**→ rplM (PRK09216)Ribosomal_L13 (pfam00572)→ rpsI (PRK00132)Ribosomal_S9 (pfam00380)→
env-806	NOM013_scaffold55258_1	-	10498	10423	**RNA**→ rplM (PRK09216)Ribosomal_L13 (pfam00572)→ rpsI (PRK00132)Ribosomal_S9 (pfam00380)→
env-807	SRS017849_C4402944	-	468	393	**RNA**→ rplM (PRK09216)Ribosomal_L13 (pfam00572)→
env-808	C4444188_1_MH0077	+	224	299	**RNA**→ rplM (PRK09216)Ribosomal_L13 (pfam00572)→
env-809	4448817.3_Contig838	-	764	689	**RNA**→ rplM (PRK09216)Ribosomal_L13 (pfam00572)→ rpsI (PRK00132)Ribosomal_S9 (pfam00380)→
env-810	4491404.3_NODE_16218	+	95	170	**RNA**→ rplM (PRK09216)Ribosomal_L13 (pfam00572)→ rpsI (PRK00132)Ribosomal_S9 (pfam00380)→
env-811	4491414.3_NODE_1809	+	43317	43392	**RNA**→ rplM (PRK09216)Ribosomal_L13 (pfam00572)→ rpsI (PRK00132)Ribosomal_S9 (pfam00380)→
env-812	4491421.3_NODE_2987	+	40037	40112	**RNA**→ rplM (PRK09216)Ribosomal_L13 (pfam00572)→ rpsI (PRK00132)Ribosomal_S9 (pfam00380)→
env-813	DLF006_scaffold45333_1	+	259	334	**RNA**→ rplM (PRK09216)Ribosomal_L13 (pfam00572)→
env-814	DLM005_scaffold13338_1	+	240	315	**RNA**→ rplM (PRK09216)Ribosomal_L13 (pfam00572)→ rpsI (PRK00132)Ribosomal_S9 (pfam00380)→
env-815	DLM017_scaffold38149_2	-	11964	11889	**RNA**→ rplM (PRK09216)Ribosomal_L13 (pfam00572)→ rpsI (PRK00132)Ribosomal_S9 (pfam00380)→
env-816	DOM001_scaffold19626_3	+	109863	109938	**RNA**→ rplM (PRK09216)Ribosomal_L13 (pfam00572)→ rpsI (PRK00132)Ribosomal_S9 (pfam00380)→
env-817	DOM003_scaffold20232_1	+	1899	1974	**RNA**→ rplM (PRK09216)Ribosomal_L13 (pfam00572)→
env-818	NOM019_scaffold53163_1	+	23837	23912	**RNA**→ rplM (PRK09216)Ribosomal_L13 (pfam00572)→ rpsI (PRK00132)Ribosomal_S9 (pfam00380)→
env-819	SRS019910_WUGC_scaffold_10772	+	132	207	**RNA**→ rplM (PRK09216)Ribosomal_L13 (pfam00572)→
env-820	SRS053398_LANL_scaffold_37336	+	287	362	**RNA**→ rplM (PRK09216)Ribosomal_L13 (pfam00572)→ rpsI (PRK00132)Ribosomal_S9 (pfam00380)→
env-821	4448820.3_Contig_1858	-	2732	2657	**RNA**→ rplM (PRK09216)Ribosomal_L13 (pfam00572)→ rpsI (PRK00132)Ribosomal_S9 (pfam00380)→
env-822	DLF003_scaffold47495_1	+	445	520	**RNA**→ rplM (PRK09216)Ribosomal_L13 (pfam00572)→
env-823	DOM023_scaffold26204_1	-	12481	12406	**RNA**→ rplM (PRK09216)Ribosomal_L13 (pfam00572)→ rpsI (PRK00132)Ribosomal_S9 (pfam00380)→
env-824	4448818.3_Contig459	-	19229	19154	**RNA**→ rplM (PRK09216)Ribosomal_L13 (pfam00572)→ rpsI (PRK00132)Ribosomal_S9 (pfam00380)→
env-825	4448811.3_Contig642	-	2634	2559	**RNA**→ rplM (PRK09216)RplM (COG0102)→ rplM (PRK09216)Ribosomal_L13 (pfam00572)→ rpsI (PRK00132)Ribosomal_S9 (pfam00380)→
env-826	4448814.3_Contig2119	+	771	846	**RNA**→ rplM (PRK09216)Ribosomal_L13 (pfam00572)→
env-827	scaffold12317_1_MH0026	-	272	197	**RNA**→ rplM (PRK09216)Ribosomal_L13 (pfam00572)→
env-828	scaffold30210_1_MH0049	-	536	461	**RNA**→ rplM (PRK09216)Ribosomal_L13 (pfam00572)→
env-829	4448810.3.47000	-	13461	13386	**RNA**→ rplM (PRK09216)Ribosomal_L13 (pfam00572)→ rpsI (PRK00132)Ribosomal_S9 (pfam00380)→
env-830	4448816.3_Contig1564	-	700	625	**RNA**→ rplM (PRK09216)Ribosomal_L13 (pfam00572)→ rplM (PRK09216)Ribosomal_L13 (pfam00572)→ rpsI (PRK00132)Ribosomal_S9 (pfam00380)→
env-831	NOM001_scaffold36808_1	-	12342	12267	**RNA**→ rplM (PRK09216)Ribosomal_L13 (pfam00572)→ rpsI (PRK00132)Ribosomal_S9 (pfam00380)→
env-832	CAM_READ_0099565819	+	27	102	**RNA**→ rplM (PRK09216)Ribosomal_L13 (pfam00572)→
env-833	4448808.3_Contig33004	-	288	214	**RNA**→ rplM (PRK09216)RplM (COG0102)→

env-834	NLM016_scaffold13879_1	-	134	59	**RNA**→ hypo→
env-835	scaffold9468_1_O2.UC-12	+	3921	3996	**RNA**→ rplM (PRK09216)Ribosomal_L13 (pfam00572)→
env-836	NLM006_scaffold9242_1	+	439	514	**RNA**→ rplM (PRK09216)RplM (COG0102)→
env-837	NLM009_scaffold10030_1	+	15733	15808	**RNA**→ rplM (PRK09216)Ribosomal_L13 (pfam00572)→ rpsI (PRK00132)Ribosomal_S9 (pfam00380)→
Pco-2-1	NZ_GG703855.1	-	96347	96272	**RNA**→ rplM (PRK09216)Ribosomal_L13 (pfam00572)→ rpsI (PRK00132)Ribosomal_S9 (pfam00380)→
env-838	contig13883	-	85	10	**RNA**→ hypo→
env-839	DLF004_scaffold32113_1	-	12265	12190	**RNA**→ rplM (PRK09216)Ribosomal_L13 (pfam00572)→ rpsI (PRK00132)Ribosomal_S9 (pfam00380)→
env-840	SRS013687_Baylor_scaffold_46077	-	545	470	**RNA**→ rplM (PRK09216)Ribosomal_L13 (pfam00572)→
env-841	SRS023914_Baylor_scaffold_16462	+	15313	15388	**RNA**→ rplM (PRK09216)Ribosomal_L13 (pfam00572)→ rpsI (PRK00132)Ribosomal_S9 (pfam00380)→
env-842	SRS049959_WUGC_scaffold_10211	-	449	374	**RNA**→ rplM (PRK09216)Ribosomal_L13 (pfam00572)→
env-843	scaffold2008_3_V1.UC-21	+	224	299	**RNA**→ rplM (PRK09216)Ribosomal_L13 (pfam00572)→ rpsI (PRK00132)Ribosomal_S9 (pfam00380)→
env-844	NOM020_scaffold12283_2	-	11931	11856	**RNA**→ rplM (PRK09216)Ribosomal_L13 (pfam00572)→ rpsI (PRK00132)Ribosomal_S9 (pfam00380)→
Por-8-1	NZ_KK073873.1	+	3030252	3030326	**RNA**→ rplM (PRK09216)Ribosomal_L13 (pfam00572)→ rpsI (PRK00132)Ribosomal_S9 (pfam00380)→
Psp-21-1	NZ_JRPT01000012.1	-	33492	33419	**RNA**→ rplM (PRK09216)Ribosomal_L13 (pfam00572)→ rpsI (PRK00132)Ribosomal_S9 (pfam00380)→
env-845	RUMENNODE_2697187_1	-	3085	3011	**RNA**→ rplM (PRK09216)Ribosomal_L13 (pfam00572)→ rpsI (PRK00132)Ribosomal_S9 (pfam00380)→
env-846	AUXO015280558.1	-	453	379	**RNA**→ rplM (PRK09216)Ribosomal_L13 (pfam00572)→
env-847	AUXO011297008.1	-	1734	1660	**RNA**→ rplM (PRK09216)Ribosomal_L13 (pfam00572)→ rpsI (PRK00132)Ribosomal_S9 (pfam00380)→
env-848	SRS013723_Baylor_scaffold_19570	+	4339	4413	**RNA**→ rplM (PRK09216)Ribosomal_L13 (pfam00572)→ rpsI (PRK00132)Ribosomal_S9 (pfam00380)→
env-849	SRS012285_C2809459	-	149	75	**RNA**→ hypo→
env-850	SRS019980_C2790887	-	135	61	**RNA**→ hypo→
env-851	SRS016043_WUGC_scaffold_31124	+	1754	1828	**RNA**→ rplM (PRK09216)Ribosomal_L13 (pfam00572)→
env-852	SRS011152_Baylor_scaffold_57690	-	154	80	**RNA**→
env-853	SRS013836_C4801934	+	838	912	**RNA**→
env-854	SRS045313_C1296217	-	154	80	**RNA**→ hypo→
env-855	SRS022725_LANL_scaffold_20670	-	412	338	**RNA**→ rplM (PRK09216)Ribosomal_L13 (pfam00572)→
env-856	SRS022149_C2846288	+	4393	4467	**RNA**→
env-857	SRS051930_C3273277	+	1215	1289	**RNA**→
env-858	SRS042984_LANL_scaffold_16758	+	9998	10072	**RNA**→ rplM (PRK09216)Ribosomal_L13 (pfam00572)→ rpsI (PRK00132)Ribosomal_S9 (pfam00380)→
env-859	SRS052876_LANL_scaffold_54535	+	954	1028	**RNA**→
Pco-1-1	NZ_HE999443.1	-	281229	281155	**RNA**→ rplM (PRK09216)Ribosomal_L13 (pfam00572)→ rpsI (PRK00132)Ribosomal_S9 (pfam00380)→
env-860	SRS014578_WUGC_scaffold_18370	-	7328	7254	**RNA**→ rplM (PRK09216)Ribosomal_L13 (pfam00572)→ rpsI (PRK00132)Ribosomal_S9 (pfam00380)→
env-861	SRS013170_Baylor_scaffold_112597	+	2727	2801	**RNA**→
env-862	SRS043239_C1976103	-	186	112	**RNA**→ hypo→
env-863	SRS063999_LANL_scaffold_2422	-	7865	7791	**RNA**→ rplM (PRK09216)Ribosomal_L13 (pfam00572)→ rpsI (PRK00132)Ribosomal_S9 (pfam00380)→
env-864	SRS016360_Baylor_scaffold_11199	-	720	646	**RNA**→ rplM (PRK09216)Ribosomal_L13 (pfam00572)→ rpsI (PRK00132)Ribosomal_S9 (pfam00380)→
env-865	SRS019128_C4357049	-	303	229	**RNA**→ rplM (PRK09216)Ribosomal_L13 (pfam00572)→
env-866	SRS024087_LANL_scaffold_47659	-	135	61	**RNA**→
env-867	SRS063932_LANL_scaffold_92613	+	4336	4410	**RNA**→
env-868	SRS024289_LANL_scaffold_70016	-	135	61	**RNA**→
env-869	SRS018443_Baylor_scaffold_65820	+	8016	8090	**RNA**→ rplM (PRK09216)Ribosomal_L13 (pfam00572)→ rpsI (PRK00132)Ribosomal_S9 (pfam00380)→
env-870	SRS018337_C2365684	+	512	586	**RNA**→
env-871	SRS063215_LANL_scaffold_21409	-	945	871	**RNA**→ rplM (PRK09216)Ribosomal_L13 (pfam00572)→ rpsI (PRK00132)Ribosomal_S9 (pfam00380)→
env-872	SRS014107_WUGC_scaffold_50706	-	134	60	**RNA**→
env-873	SRS015044_WUGC_scaffold_67840	-	135	61	**RNA**→
env-874	SRS016331_C4131247	-	156	82	**RNA**→
env-875	SRS017139_C3804675	-	135	61	**RNA**→
env-876	SRS024355_LANL_scaffold_42842	-	1441	1367	**RNA**→ rplM (PRK09216)Ribosomal_L13 (pfam00572)→ rpsI (PRK00132)Ribosomal_S9 (pfam00380)→
Psp-25-1	NZ_GG740073.1	-	461425	461351	**RNA**→ rplM (PRK09216)Ribosomal_L13 (pfam00572)→ rplM (PRK09216)Ribosomal_L13 (pfam00572)→ rpsI (PRK00132)Ribosomal_S9 (pfam00380)→ rpsI (PRK00132)Ribosomal_S9 (pfam00380)→
env-877	SRS011343_C2242542	+	1051	1125	**RNA**→
env-878	SRS014691_C2503925	-	145	71	**RNA**→ hypo→
env-879	SRS019028_WUGC_scaffold_45064	-	145	71	**RNA**→ hypo→

env-880	SRS015574_WUGC_scaffold_63161	+	871	945	**RNA**→ rplM (PRK09216)Ribosomal_L13 (pfam00572)→
env-881	SRS016746_Baylor_scaffold_73191	+	1882	1956	**RNA**→
env-882	SRS017511_Baylor_scaffold_92834	-	135	61	**RNA**→
env-883	SRS023358_C3462583	+	482	556	**RNA**→
env-884	SRS049318_LANL_scaffold_32648	+	9208	9282	**RNA**→
env-885	SRS050628_LANL_scaffold_20687	-	978	904	**RNA**→ rplM (PRK09216)Ribosomal_L13 (pfam00572)→ rpsI (PRK00132)Ribosomal_S9 (pfam00380)→
env-886	SRS022719_LANL_scaffold_45285	-	804	729	**RNA**→ rplM (PRK09216)Ribosomal_L13 (pfam00572)→ rpsI (PRK00132)Ribosomal_S9 (pfam00380)→
env-887	SRS019129_WUGC_scaffold_43257	-	338	263	**RNA**→ rplM (PRK09216)Ribosomal_L13 (pfam00572)→
env-888	SRS016043_C2115867	-	218	143	**RNA**→ rplM (PRK09216)RplM (COG0102)→
env-889	SRS013533_PGA_scaffold_117699	+	3103	3178	**RNA**→
env-890	SRS017511_Baylor_scaffold_92627	+	951	1026	**RNA**→
env-891	SRS021477_C2588523	-	323	248	**RNA**→ rplM (PRK09216)Ribosomal_L13 (pfam00572)→
env-892	SRS013170_Baylor_scaffold_67347	-	307	232	**RNA**→ rplM (PRK09216)Ribosomal_L13 (pfam00572)→
env-893	SRS064449_C4126558	-	118	43	**RNA**→
env-894	SRS053917_LANL_scaffold_88433	+	607	682	**RNA**→ rplM (PRK09216)Ribosomal_L13 (pfam00572)→
env-895	SRS013252_C3525919	-	153	78	**RNA**→ hypo→
env-896	SRS015803_C2675896	+	3433	3508	**RNA**→ rplM (PRK09216)Ribosomal_L13 (pfam00572)→ rpsI (PRK00132)Ribosomal_S9 (pfam00380)→
env-897	SRS018394_C4057209	-	982	907	**RNA**→ rplM (PRK09216)Ribosomal_L13 (pfam00572)→ rpsI (PRK00132)Ribosomal_S9 (pfam00380)→
env-898	SRS024649_LANL_scaffold_51754	-	635	560	**RNA**→ rplM (PRK09216)Ribosomal_L13 (pfam00572)→
env-899	SRS054430_LANL_scaffold_20956	-	988	913	**RNA**→ rplM (PRK09216)Ribosomal_L13 (pfam00572)→ rpsI (PRK00132)Ribosomal_S9 (pfam00380)→
Plo-1-1	NZ_KB899213.1	+	249357	249432	**RNA**→ rplM (PRK09216)Ribosomal_L13 (pfam00572)→ rpsI (PRK00132)Ribosomal_S9 (pfam00380)→
env-900	SRS017445_Baylor_scaffold_83464	+	1528	1603	**RNA**→ rplM (PRK09216)Ribosomal_L13 (pfam00572)→
env-901	SRS018573_C2480980	-	139	64	**RNA**→
env-902	SRS045197_WUGC_scaffold_65903	+	7998	8073	**RNA**→ rplM (PRK09216)Ribosomal_L13 (pfam00572)→ rpsI (PRK00132)Ribosomal_S9 (pfam00380)→
Psp-26-1	NZ_GG704825.1	-	516881	516806	**RNA**→ rplM (PRK09216)Ribosomal_L13 (pfam00572)→ rpsI (PRK00132)Ribosomal_S9 (pfam00380)→ hypo→ rpsB (PRK05299)Ribosomal_S2 (pfam00318)→ tsf (PRK09377)UBA_EF-Ts (cd14275)EF_TS (pfam00889)→
env-903	SRS023841_Baylor_scaffold_21872	-	947	872	**RNA**→ rplM (PRK09216)Ribosomal_L13 (pfam00572)→ rpsI (PRK00132)Ribosomal_S9 (pfam00380)→
env-904	SRS011098_C1871947	-	560	485	**RNA**→ rplM (PRK09216)Ribosomal_L13 (pfam00572)→
env-905	SRS011255_C2493253	-	151	76	**RNA**→
env-906	SRS016746_C2995254	+	311	386	**RNA**→
env-907	SRS017227_C5088817	+	278	353	**RNA**→
env-908	SRS018157_Baylor_scaffold_33504	+	7670	7745	**RNA**→ rplM (PRK09216)Ribosomal_L13 (pfam00572)→ rpsI (PRK00132)Ribosomal_S9 (pfam00380)→
env-909	SRS018665_C3707955	+	500	575	**RNA**→
env-910	SRS019906_C2276695	+	7839	7914	**RNA**→
env-911	SRS023595_C6887425	-	139	64	**RNA**→ hypo→
env-912	SRS023938_C2241954	-	119	44	**RNA**→ hypo→
env-913	SRS024087_LANL_scaffold_42583	-	139	64	**RNA**→
env-914	SRS051378_C1873917	-	1295	1220	**RNA**→ rplM (PRK09216)Ribosomal_L13 (pfam00572)→ rpsI (PRK00132)Ribosomal_S9 (pfam00380)→
env-915	SRS051941_LANL_scaffold_77484	-	153	78	**RNA**→
env-916	SRS052604_LANL_scaffold_13013	+	539	614	**RNA**→ rplM (PRK09216)Ribosomal_L13 (pfam00572)→ rpsI (PRK00132)Ribosomal_S9 (pfam00380)→
env-917	SRS055401_C2209469	+	195	270	**RNA**→
env-918	SRS058053_LANL_scaffold_60299	+	304	379	**RNA**→
env-919	SRS063603_C4249750	-	118	43	**RNA**→
env-920	SRS063932_LANL_scaffold_98774	-	139	64	**RNA**→
env-921	SRS017808_C3874880	-	146	71	**RNA**→
env-922	SRS017439_Baylor_scaffold_51404	-	632	557	**RNA**→ rplM (PRK09216)Ribosomal_L13 (pfam00572)→
env-923	SRS075404_LANL_scaffold_60611	+	3391	3466	**RNA**→
env-924	SRS012279_Baylor_scaffold_46820	+	3560	3635	**RNA**→ rplM (PRK09216)Ribosomal_L13 (pfam00572)→ rpsI (PRK00132)Ribosomal_S9 (pfam00380)→
env-925	SRS013705_Baylor_scaffold_63054	+	3114	3189	**RNA**→ rplM (PRK09216)Ribosomal_L13 (pfam00572)→ rpsI (PRK00132)Ribosomal_S9 (pfam00380)→
env-926	SRS013879_C2765042	+	277	352	**RNA**→
env-927	SRS017209_Baylor_scaffold_40939	+	72651	72726	**RNA**→ rplM (PRK09216)Ribosomal_L13 (pfam00572)→ rpsI (PRK00132)Ribosomal_S9 (pfam00380)→
env-928	SRS018357_Baylor_scaffold_46794	+	98	173	**RNA**→ rplM (PRK09216)Ribosomal_L13 (pfam00572)→

env-929	SRS022077_Baylor_scaffold_41018	+	3560	3635	**RNA**→ rplM (PRK09216)Ribosomal_L13 (pfam00572)→ rpsI (PRK00132)Ribosomal_S9 (pfam00380)→
env-930	SRS023926_Baylor_scaffold_55027	+	3644	3719	**RNA**→ rplM (PRK09216)Ribosomal_L13 (pfam00572)→ rpsI (PRK00132)Ribosomal_S9 (pfam00380)→
env-931	SRS050669_LANL_scaffold_50571	+	3562	3637	**RNA**→ rplM (PRK09216)Ribosomal_L13 (pfam00572)→ rpsI (PRK00132)Ribosomal_S9 (pfam00380)→
env-932	SRS064329_LANL_scaffold_50907	-	985	910	**RNA**→ rplM (PRK09216)Ribosomal_L13 (pfam00572)→ rpsI (PRK00132)Ribosomal_S9 (pfam00380)→
env-933	Tongue_dor_WUGI_scaffold_12918	+	1043	1118	**RNA**→
env-934	SRS011306_Baylor_scaffold_62841	-	35842	35767	**RNA**→ rplM (PRK09216)Ribosomal_L13 (pfam00572)→ rpsI (PRK00132)Ribosomal_S9 (pfam00380)→
env-935	SRS014573_WUGC_scaffold_21848	+	3270	3345	**RNA**→ rplM (PRK09216)Ribosomal_L13 (pfam00572)→
env-936	SRS015762_C3664821	+	2929	3004	**RNA**→
env-937	SRS016002_C2493893	-	135	60	**RNA**→ hypo→
env-938	SRS018300_Baylor_scaffold_10118	+	3538	3613	**RNA**→ rplM (PRK09216)Ribosomal_L13 (pfam00572)→ rpsI (PRK00132)Ribosomal_S9 (pfam00380)→
env-939	SRS018739_WUGC_scaffold_27638	+	3156	3231	**RNA**→ rplM (PRK09216)Ribosomal_L13 (pfam00572)→ rpsI (PRK00132)Ribosomal_S9 (pfam00380)→
env-940	SRS019389_C2124919	-	146	71	**RNA**→ hypo→
env-941	SRS024081_LANL_scaffold_17499	+	2653	2728	**RNA**→ rplM (PRK09216)Ribosomal_L13 (pfam00572)→ rpsI (PRK00132)Ribosomal_S9 (pfam00380)→
env-942	SRS024441_LANL_scaffold_33890	+	6153	6228	**RNA**→ rplM (PRK09216)Ribosomal_L13 (pfam00572)→ rpsI (PRK00132)Ribosomal_S9 (pfam00380)→
env-943	SRS049389_WUGC_scaffold_44708	-	146	71	**RNA**→
env-944	SRS053603_LANL_scaffold_56769	+	3538	3613	**RNA**→
env-945	SRS057205_LANL_scaffold_36133	-	1438	1363	**RNA**→ rplM (PRK09216)Ribosomal_L13 (pfam00572)→ rpsI (PRK00132)Ribosomal_S9 (pfam00380)→
env-946	SRS063193_LANL_scaffold_48783	+	3570	3645	**RNA**→ rplM (PRK09216)Ribosomal_L13 (pfam00572)→
env-947	SRS064774_C2783916	-	146	71	**RNA**→ hypo→
Psh-1-1	NZ_BAIZ01000030.1	-	35483	35408	**RNA**→ rplM (PRK09216)Ribosomal_L13 (pfam00572)→ rpsI (PRK00132)Ribosomal_S9 (pfam00380)→
env-948	SRS022143_WUGC_scaffold_91746	-	632	557	**RNA**→ rplM (PRK09216)Ribosomal_L13 (pfam00572)→
env-949	SRS019327_WUGC_scaffold_70870	-	10558	10483	**RNA**→ rplM (PRK09216)Ribosomal_L13 (pfam00572)→ rpsI (PRK00132)Ribosomal_S9 (pfam00380)→
env-950	SRS015434_C5949372	-	146	71	**RNA**→
env-951	SRS018439_Baylor_scaffold_22146	-	17449	17374	**RNA**→ rplM (PRK09216)Ribosomal_L13 (pfam00572)→ rpsI (PRK00132)Ribosomal_S9 (pfam00380)→
env-952	SRS019071_C5217890	+	1981	2056	**RNA**→
env-953	SRS013164_Baylor_scaffold_59130	-	979	904	**RNA**→ rplM (PRK09216)Ribosomal_L13 (pfam00572)→ rpsI (PRK00132)Ribosomal_S9 (pfam00380)→
env-954	SRS014888_C3104655	-	146	71	**RNA**→
env-955	SRS023958_Baylor_scaffold_31813	+	3560	3635	**RNA**→ rplM (PRK09216)Ribosomal_L13 (pfam00572)→
env-956	SRS057355_C2359830	-	146	71	**RNA**→
env-957	SRS021496_C2846806	-	146	71	**RNA**→
env-958	SRS022530_LANL_scaffold_60444	-	7595	7520	**RNA**→ rplM (PRK09216)Ribosomal_L13 (pfam00572)→ rpsI (PRK00132)Ribosomal_S9 (pfam00380)→
env-959	SRS063288_C2558656	+	371	446	**RNA**→
env-960	scaffold15263_2_MH0055	-	943	868	**RNA**→ rplM (PRK09216)Ribosomal_L13 (pfam00572)→ rpsI (PRK00132)Ribosomal_S9 (pfam00380)→
env-961	scaffold18695_5_MH0014	+	2193	2268	**RNA**→ rplM (PRK09216)Ribosomal_L13 (pfam00572)→ rpsI (PRK00132)Ribosomal_S9 (pfam00380)→
env-962	scaffold58703_4_MH0042	+	509	584	**RNA**→ rplM (PRK09216)Ribosomal_L13 (pfam00572)→
env-963	scaffold410_3_MH0063	-	5281	5206	**RNA**→ rplM (PRK09216)Ribosomal_L13 (pfam00572)→ rpsI (PRK00132)Ribosomal_S9 (pfam00380)→
env-964	scaffold1879_1_O2.UC-24	+	1299	1374	**RNA**→ rplM (PRK09216)Ribosomal_L13 (pfam00572)→ rpsI (PRK00132)Ribosomal_S9 (pfam00380)→
env-965	scaffold6667_6_V1.UC-14	-	958	883	**RNA**→ rplM (PRK09216)Ribosomal_L13 (pfam00572)→ rpsI (PRK00132)Ribosomal_S9 (pfam00380)→
env-966	4491482.3_NODE_74973	-	6204	6129	**RNA**→ rplM (PRK09216)Ribosomal_L13 (pfam00572)→ rpsI (PRK00132)Ribosomal_S9 (pfam00380)→
env-967	BAAX01036178.1	-	165	88	**RNA**→
env-968	4491403.3_NODE_60304	-	979	902	**RNA**→ rplM (PRK09216)Ribosomal_L13 (pfam00572)→ rplM (PRK09216)Ribosomal_L13 (pfam00572)→ rpsI (PRK00132)Ribosomal_S9 (pfam00380)→ rpsI (PRK00132)Ribosomal_S9 (pfam00380)→
env-969	DLF003_scaffold24849_4	-	8212	8135	**RNA**→ rplM (PRK09216)Ribosomal_L13 (pfam00572)→ rpsI (PRK00132)Ribosomal_S9 (pfam00380)→
env-970	NLM016_scaffold8572_3	-	1028	951	**RNA**→ rplM (PRK09216)Ribosomal_L13 (pfam00572)→ rpsI (PRK00132)Ribosomal_S9 (pfam00380)→
env-971	4491404.3_NODE_3637	+	6552	6629	**RNA**→ rplM (PRK09216)Ribosomal_L13 (pfam00572)→
env-972	DLM006_scaffold334_9	+	10545	10622	**RNA**→ rplM (PRK09216)Ribosomal_L13 (pfam00572)→ rpsI (PRK00132)Ribosomal_S9 (pfam00380)→
env-973	scaffold78050_1_O2.UC-19	+	162	239	**RNA**→ rplM (PRK09216)Ribosomal_L13 (pfam00572)→ rpsI (PRK00132)Ribosomal_S9 (pfam00380)→
env-974	DOM016_scaffold1189_4	+	9750	9827	**RNA**→ rplM (PRK09216)Ribosomal_L13 (pfam00572)→ rpsI (PRK00132)Ribosomal_S9 (pfam00380)→
env-975	DOM022_scaffold19179_2	+	11637	11714	**RNA**→ rplM (PRK09216)Ribosomal_L13 (pfam00572)→ rpsI (PRK00132)Ribosomal_S9 (pfam00380)→
env-976	SRS011529_WUGC_scaffold_9481	+	35	112	**RNA**→ rplM (PRK09216)Ribosomal_L13 (pfam00572)→ rpsI (PRK00132)Ribosomal_S9 (pfam00380)→
Pst-1-1	NZ_JH379462.1	-	10330	10253	**RNA**→ rplM (PRK09216)Ribosomal_L13 (pfam00572)→ rpsI (PRK00132)Ribosomal_S9 (pfam00380)→
env-977	NLF006_scaffold10643_1	-	1262	1185	**RNA**→ rplM (PRK09216)Ribosomal_L13 (pfam00572)→ rpsI (PRK00132)Ribosomal_S9 (pfam00380)→

env-978	NLM029_scaffold46472_1	-	9492	9415	**RNA**→ rplM (PRK09216)Ribosomal_L13 (pfam00572)→ rpsI (PRK00132)Ribosomal_S9 (pfam00380)→
env-979	NLF005_scaffold222_5	+	9773	9850	**RNA**→ rplM (PRK09216)Ribosomal_L13 (pfam00572)→ rpsI (PRK00132)Ribosomal_S9 (pfam00380)→
env-980	NOM007_scaffold112_4	+	15977	16054	**RNA**→ rplM (PRK09216)Ribosomal_L13 (pfam00572)→ rpsI (PRK00132)Ribosomal_S9 (pfam00380)→
env-981	NLM025_scaffold10432_10	-	8972	8895	**RNA**→ rplM (PRK09216)Ribosomal_L13 (pfam00572)→ rpsI (PRK00132)Ribosomal_S9 (pfam00380)→
env-982	DLM002_scaffold10936_3	+	5488	5565	**RNA**→ rplM (PRK09216)Ribosomal_L13 (pfam00572)→ rpsI (PRK00132)Ribosomal_S9 (pfam00380)→
env-983	NLM009_scaffold4804_2	+	26579	26656	**RNA**→ rplM (PRK09216)Ribosomal_L13 (pfam00572)→ rpsI (PRK00132)Ribosomal_S9 (pfam00380)→
env-984	NLM002_scaffold27297_2	-	9435	9358	**RNA**→ rplM (PRK09216)Ribosomal_L13 (pfam00572)→ rpsI (PRK00132)Ribosomal_S9 (pfam00380)→
env-985	UnmappedStool_Broad_scaffold_727222	-	162	82	**RNA**→ hypo→
env-986	4491404.3_NODE_8492	+	545	625	**RNA**→ rplM (PRK09216)Ribosomal_L13 (pfam00572)→
env-987	SRS016541_C1524265	+	279	359	**RNA**→
env-988	SRS013687_Baylor_scaffold_57705	+	279	359	**RNA**→ rplM (PRK09216)Ribosomal_L13 (pfam00572)→
env-989	SRS023914_Baylor_scaffold_14686	+	279	359	**RNA**→ rplM (PRK09216)Ribosomal_L13 (pfam00572)→ rpsI (PRK00132)Ribosomal_S9 (pfam00380)→
env-990	BAAX01006449.1	-	273	193	**RNA**→ rplM (PRK09216)Ribosomal_L13 (pfam00572)→
env-991	NLF006_scaffold165_1	-	402	322	**RNA**→ rplM (PRK09216)Ribosomal_L13 (pfam00572)→
env-992	4491403.3_NODE_60304	-	780	700	**RNA**→ rplM (PRK09216)Ribosomal_L13 (pfam00572)→ rplM (PRK09216)Ribosomal_L13 (pfam00572)→ rpsI (PRK00132)Ribosomal_S9 (pfam00380)→ rpsI (PRK00132)Ribosomal_S9 (pfam00380)→
env-993	SRS049959_C3758114	-	124	44	**RNA**→ hypo→
env-994	scaffold29644_4_O2.UC-13	-	6421	6346	**RNA**→ rplM (PRK09216)Ribosomal_L13 (pfam00572)→ rpsI (PRK00132)Ribosomal_S9 (pfam00380)→
Pbu-3-1	NZ_GG739928.1	-	179846	179770	**RNA**→ rplM (PRK09216)Ribosomal_L13 (pfam00572)→ rplM (PRK09216)Ribosomal_L13 (pfam00572)→ rpsI (PRK00132)Ribosomal_S9 (pfam00380)→ rpsI (PRK00132)Ribosomal_S9 (pfam00380)→
Pbu-1-1	NZ_JVYX01000008.1	-	5815	5739	**RNA**→ rplM (PRK09216)Ribosomal_L13 (pfam00572)→ rpsI (PRK00132)Ribosomal_S9 (pfam00380)→
Pbu-2-1	NZ_GL586311.1	+	1240091	1240167	**RNA**→ rplM (PRK09216)Ribosomal_L13 (pfam00572)→ rpsI (PRK00132)Ribosomal_S9 (pfam00380)→
Psp-12-1	NZ_ALJQ01000010.1	-	159165	159089	**RNA**→ rplM (PRK09216)Ribosomal_L13 (pfam00572)→ rplM (PRK09216)Ribosomal_L13 (pfam00572)→ rpsI (PRK00132)Ribosomal_S9 (pfam00380)→ rpsI (PRK00132)Ribosomal_S9 (pfam00380)→
env-995	AUXO015968780.1	+	216	290	**RNA**→
env-996	AUXO017534585.1	-	968	894	**RNA**→ rplM (PRK09216)Ribosomal_L13 (pfam00572)→ rpsI (PRK00132)Ribosomal_S9 (pfam00380)→
env-997	pre-rum113-00662	-	3481	3406	**RNA**→ rplM (PRK09216)Ribosomal_L13 (pfam00572)→ rpsI (PRK00132)Ribosomal_S9 (pfam00380)→
env-998	pre-rum114-03875	+	2977	3052	**RNA**→ rplM (PRK09216)Ribosomal_L13 (pfam00572)→ rpsI (PRK00132)Ribosomal_S9 (pfam00380)→
env-999	HCE12Call500_c0260910	+	4902	4978	**RNA**→ rplM (PRK09216)Ribosomal_L13 (pfam00572)→
env-1000	HCF12C_329458	-	179	103	**RNA**→ ←hypo
Por-1-1	NZ_GL833119.1	-	125193	125119	**RNA**→ rplM (PRK09216)Ribosomal_L13 (pfam00572)→ rpsI (PRK00132)Ribosomal_S9 (pfam00380)→
Por-3-1	NZ_KE340302.1	-	130474	130400	**RNA**→ rplM (PRK09216)Ribosomal_L13 (pfam00572)→ rpsI (PRK00132)Ribosomal_S9 (pfam00380)→
env-1001	SRS013836_C4829201	-	117	43	**RNA**→
Por-2-1	NZ_KI669542.1	-	130434	130360	**RNA**→ rplM (PRK09216)Ribosomal_L13 (pfam00572)→ rpsI (PRK00132)Ribosomal_S9 (pfam00380)→
env-1002	SRS023604_Baylor_scaffold_3034	+	34418	34492	**RNA**→ rplM (PRK09216)Ribosomal_L13 (pfam00572)→ rpsI (PRK00132)Ribosomal_S9 (pfam00380)→
env-1003	SRS013542_C165903	-	485	411	**RNA**→ rplM (PRK09216)Ribosomal_L13 (pfam00572)→
Pti-3-1	NZ_JRPQ01000080.1	+	19409	19483	**RNA**→ rplM (PRK09216)Ribosomal_L13 (pfam00572)→ rpsI (PRK00132)Ribosomal_S9 (pfam00380)→
env-1004	SRS011111_Baylor_scaffold_4265	+	5684	5758	**RNA**→ rplM (PRK09216)Ribosomal_L13 (pfam00572)→
Pti-1-1	NZ_HG417093.1	-	162971	162897	**RNA**→ rplM (PRK09216)Ribosomal_L13 (pfam00572)→ rpsI (PRK00132)Ribosomal_S9 (pfam00380)→
Pti-2-1	NZ_ADEF01000048.1	+	153828	153902	**RNA**→ rplM (PRK09216)Ribosomal_L13 (pfam00572)→ rplM (PRK09216)Ribosomal_L13 (pfam00572)→ rpsI (PRK00132)Ribosomal_S9 (pfam00380)→ rpsI (PRK00132)Ribosomal_S9 (pfam00380)→
env-1005	SRS023468_LANL_scaffold_1144	-	3752	3678	**RNA**→ rplM (PRK09216)Ribosomal_L13 (pfam00572)→ rpsI (PRK00132)Ribosomal_S9 (pfam00380)→
env-1006	SRS023604_Baylor_scaffold_2736	-	116	42	**RNA**→ hypo→
Psp-4-1	NZ_AWXC01000037.1	-	65697	65623	**RNA**→ rplM (PRK09216)Ribosomal_L13 (pfam00572)→ rpsI (PRK00132)Ribosomal_S9 (pfam00380)→
Pbu-6-1	NZ_JRNP01000028.1	+	52030	52104	**RNA**→ rplM (PRK09216)Ribosomal_L13 (pfam00572)→ rpsI (PRK00132)Ribosomal_S9 (pfam00380)→
env-1007	SRS011111_Baylor_scaffold_12781	-	1177	1103	**RNA**→ rplM (PRK09216)Ribosomal_L13 (pfam00572)→ rpsI (PRK00132)Ribosomal_S9 (pfam00380)→
Pbu-5-1	NZ_JRNN01000034.1	+	33581	33655	**RNA**→ rplM (PRK09216)Ribosomal_L13 (pfam00572)→ rpsI (PRK00132)Ribosomal_S9 (pfam00380)→
Pbu-4-1	NZ_ADEG01000023.1	-	35564	35490	**RNA**→ rplM (PRK09216)Ribosomal_L13 (pfam00572)→ rplM (PRK09216)Ribosomal_L13 (pfam00572)→ rpsI (PRK00132)Ribosomal_S9 (pfam00380)→ rpsI (PRK00132)Ribosomal_S9 (pfam00380)→
env-1008	SRS017511_Baylor_scaffold_48340	-	333	258	**RNA**→ hypo→ rplM (PRK09216)Ribosomal_L13 (pfam00572)→
Pen-1-1	NZ_BAIX01000001.1	+	113639	113714	**RNA**→ rplM (PRK09216)Ribosomal_L13 (pfam00572)→ rpsI (PRK00132)Ribosomal_S9 (pfam00380)→
env-1009	SRS013950_C2181444	-	1427	1352	**RNA**→ rplM (PRK09216)Ribosomal_L13 (pfam00572)→ rpsI (PRK00132)Ribosomal_S9 (pfam00380)→
env-1010	SRS014107_WUGC_scaffold_54517	-	223	148	**RNA**→ rplM (PRK09216)RplM (COG0102)→

env-1011	SRS014476_C3414245	+	514	589	RNA→ rplM (PRK09216)RplM (COG0102)→
env-1012	SRS014691_C2727318	+	851	926	RNA→
env-1013	SRS016746_Baylor_scaffold_78063	+	1074	1149	RNA→ rplM (PRK09216)Ribosomal_L13 (pfam00572)→
env-1014	SRS017227_Baylor_scaffold_162432	-	14350	14275	RNA→ rplM (PRK09216)Ribosomal_L13 (pfam00572)→ rpsI (PRK00132)Ribosomal_S9 (pfam00380)→
env-1015	SRS019029_WUGC_scaffold_53097	+	1214	1289	RNA→ rplM (PRK09216)Ribosomal_L13 (pfam00572)→
env-1016	SRS047634_LANL_scaffold_137848	+	8745	8820	RNA→
Ppl-1-1	NZ_AWET01000036.1	+	123358	123433	RNA→ rplM (PRK09216)Ribosomal_L13 (pfam00572)→ rpsI (PRK00132)Ribosomal_S9 (pfam00380)→
env-1017	Buccal_mucosa_LANL_C64865474	+	28	103	RNA→ rplM (PRK09216)Ribosomal_L13 (pfam00572)→
env-1018	SRS011255_C2371241	-	446	371	RNA→ rplM (PRK09216)Ribosomal_L13 (pfam00572)→
env-1019	SRS047113_LANL_scaffold_81006	+	28	103	RNA→ hypo→ rplM (PRK09216)Ribosomal_L13 (pfam00572)→ rpsI (PRK00132)Ribosomal_S9 (pfam00380)→
env-1020	SRS058808_C2744514	+	90	165	RNA→ hypo→ rplM (PRK09216)Ribosomal_L13 (pfam00572)→
Ppl-2-1	NZ_BAJN01000002.1	-	253813	253738	RNA→ rplM (PRK09216)Ribosomal_L13 (pfam00572)→ rpsI (PRK00132)Ribosomal_S9 (pfam00380)→
env-1021	scaffold29515_4_MH0046	-	478	404	RNA→ rplM (PRK09216)Ribosomal_L13 (pfam00572)→
env-1022	scaffold6118_1_MH0049	+	1778	1852	RNA→ rplM (PRK09216)Ribosomal_L13 (pfam00572)→ rpsI (PRK00132)Ribosomal_S9 (pfam00380)→
env-1023	scaffold121376_1_MH0056	-	1807	1733	RNA→ rplM (PRK09216)Ribosomal_L13 (pfam00572)→ rpsI (PRK00132)Ribosomal_S9 (pfam00380)→
env-1024	4448812.3_Contig33336	+	223	300	RNA→
env-1025	DLF003_scaffold46021_1	-	4697	4623	RNA→ rplM (PRK09216)Ribosomal_L13 (pfam00572)→ rpsI (PRK00132)Ribosomal_S9 (pfam00380)→
env-1026	NOF001_scaffold14758_1	-	4682	4608	RNA→ rplM (PRK09216)Ribosomal_L13 (pfam00572)→ rpsI (PRK00132)Ribosomal_S9 (pfam00380)→
env-1027	SRS017227_C4992648	-	155	81	RNA→ hypo→
Pma-4-1	NZ_GL397214.1	-	1714297	1714223	RNA→ rplM (PRK09216)Ribosomal_L13 (pfam00572)→ rpsI (PRK00132)Ribosomal_S9 (pfam00380)→
env-1028	SRS023595_Baylor_scaffold_66299	+	21	95	RNA→ rplM (PRK09216)Ribosomal_L13 (pfam00572)→ rpsI (PRK00132)Ribosomal_S9 (pfam00380)→
Pma-5-1	NZ_BAJI01000002.1	-	152336	152262	RNA→ rplM (PRK09216)Ribosomal_L13 (pfam00572)→ hypo→ rpsI (PRK00132)Ribosomal_S9 (pfam00380)→
env-1029	AUXO014575475.1	+	800	874	RNA→ rplM (PRK09216)Ribosomal_L13 (pfam00572)→ rpsI (PRK00132)Ribosomal_S9 (pfam00380)→
env-1030	RUMENNODE_3847963_1	+	42999	43073	RNA→ rplM (PRK09216)Ribosomal_L13 (pfam00572)→ rplM (PRK09216)Ribosomal_L13 (pfam00572)→
env-1031	AUXO014586155.1	+	7411	7485	RNA→ rplM (PRK09216)Ribosomal_L13 (pfam00572)→ rpsI (PRK00132)Ribosomal_S9 (pfam00380)→
env-1032	HCF12C_125474	-	35705	35631	RNA→ rplM (PRK09216)Ribosomal_L13 (pfam00572)→ rpsI (PRK00132)Ribosomal_S9 (pfam00380)→
env-1033	HCE12Call500_c0173985	+	472	546	RNA→ rplM (PRK09216)Ribosomal_L13 (pfam00572)→ rpsI (PRK00132)Ribosomal_S9 (pfam00380)→
env-1034	NLF006_scaffold31027_2	-	1626	1552	RNA→ rplM (PRK09216)Ribosomal_L13 (pfam00572)→ rpsI (PRK00132)Ribosomal_S9 (pfam00380)→
env-1035	AUXO013075926.1	+	551	625	RNA→ rplM (PRK09216)Ribosomal_L13 (pfam00572)→ rpsI (PRK00132)Ribosomal_S9 (pfam00380)→
env-1036	RUMENNODE_4167426_1	+	15701	15775	RNA→ rplM (PRK09216)Ribosomal_L13 (pfam00572)→ rpsI (PRK00132)Ribosomal_S9 (pfam00380)→
env-1037	AUXO014456099.1	-	1496	1422	RNA→ rplM (PRK09216)Ribosomal_L13 (pfam00572)→ rpsI (PRK00132)Ribosomal_S9 (pfam00380)→
env-1038	scaffold2367_2_V1.UC-14	-	1330	1257	RNA→ rplM (PRK09216)Ribosomal_L13 (pfam00572)→ rpsI (PRK00132)Ribosomal_S9 (pfam00380)→
env-1039	4491414.3_NODE_1526	-	5046	4973	RNA→ rplM (PRK09216)Ribosomal_L13 (pfam00572)→ rpsI (PRK00132)Ribosomal_S9 (pfam00380)→
env-1040	DLF003_scaffold16038_8	-	1088	1015	RNA→ rplM (PRK09216)Ribosomal_L13 (pfam00572)→ rpsI (PRK00132)Ribosomal_S9 (pfam00380)→
env-1041	DLF005_scaffold3173_4	-	4285	4212	RNA→ rplM (PRK09216)Ribosomal_L13 (pfam00572)→ rpsI (PRK00132)Ribosomal_S9 (pfam00380)→
env-1042	NOM012_scaffold23711_5	+	2538	2611	RNA→ rplM (PRK09216)Ribosomal_L13 (pfam00572)→ rpsI (PRK00132)Ribosomal_S9 (pfam00380)→
env-1043	scaffold1280_4_V1.UC-13	-	11294	11221	RNA→ rplM (PRK09216)Ribosomal_L13 (pfam00572)→ rpsI (PRK00132)Ribosomal_S9 (pfam00380)→
env-1044	SRS015663_WUGC_scaffold_11548	+	5547	5620	RNA→ rplM (PRK09216)Ribosomal_L13 (pfam00572)→ rpsI (PRK00132)Ribosomal_S9 (pfam00380)→
env-1045	SRS051031_LANL_scaffold_24636	-	16175	16102	RNA→ rplM (PRK09216)Ribosomal_L13 (pfam00572)→ rpsI (PRK00132)Ribosomal_S9 (pfam00380)→
env-1046	scaffold8699_1_MH0021	+	371	444	RNA→ rplM (PRK09216)Ribosomal_L13 (pfam00572)→
env-1047	C1729250_1_MH0022	-	754	681	RNA→ rplM (PRK09216)Ribosomal_L13 (pfam00572)→ rpsI (PRK00132)Ribosomal_S9 (pfam00380)→
env-1048	scaffold49_1_MH0057	+	5529	5602	RNA→ rplM (PRK09216)Ribosomal_L13 (pfam00572)→ rpsI (PRK00132)Ribosomal_S9 (pfam00380)→
env-1049	scaffold98913_1_O2.UC-19	-	593	520	RNA→ rplM (PRK09216)Ribosomal_L13 (pfam00572)→
env-1050	scaffold80421_1_O2.UC-1	+	180	253	RNA→ rplM (PRK09216)Ribosomal_L13 (pfam00572)→
env-1051	DLM018_scaffold38383_1	+	191	264	RNA→ rplM (PRK09216)Ribosomal_L13 (pfam00572)→ rpsI (PRK00132)Ribosomal_S9 (pfam00380)→
env-1052	4491401.3_NODE_2070	-	1171	1098	RNA→ rplM (PRK09216)Ribosomal_L13 (pfam00572)→ rpsI (PRK00132)Ribosomal_S9 (pfam00380)→
env-1053	4491477.3_NODE_12413	+	2389	2462	RNA→ rplM (PRK09216)Ribosomal_L13 (pfam00572)→ rpsI (PRK00132)Ribosomal_S9 (pfam00380)→
Psp-2-1	NZ_LFQU01000025.1	+	6048	6121	RNA→ rplM (PRK09216)Ribosomal_L13 (pfam00572)→ rpsI (PRK00132)Ribosomal_S9 (pfam00380)→
env-1054	4491405.3_NODE_34345	+	86865	86938	RNA→ rplM (PRK09216)Ribosomal_L13 (pfam00572)→ rpsI (PRK00132)Ribosomal_S9 (pfam00380)→
env-1055	scaffold4912_4_MH0034	-	2299	2226	RNA→ rplM (PRK09216)Ribosomal_L13 (pfam00572)→ rpsI (PRK00132)Ribosomal_S9 (pfam00380)→
env-1056	C1986582_1_MH0079	+	198	271	RNA→ rplM (PRK09216)Ribosomal_L13 (pfam00572)→
env-1057	scaffold47598_1_O2.UC-11	+	2719	2792	RNA→ rplM (PRK09216)Ribosomal_L13 (pfam00572)→ rpsI (PRK00132)Ribosomal_S9 (pfam00380)→

env-1058	AUXO010723907.1	-	1439	1366	**RNA**→ rplM (PRK09216)Ribosomal_L13 (pfam00572)→ rpsI (PRK00132)Ribosomal_S9 (pfam00380)→
env-1059	SRM_contig03116	+	29	103	**RNA**→ rplM (PRK09216)Ribosomal_L13 (pfam00572)→
env-1060	AUXO013680712.1	+	1792	1865	**RNA**→ rplM (PRK09216)Ribosomal_L13 (pfam00572)→ rpsI (PRK00132)Ribosomal_S9 (pfam00380)→
env-1061	SRS049959_WUGC_scaffold_56943	-	909	836	**RNA**→ rplM (PRK09216)Ribosomal_L13 (pfam00572)→ rpsI (PRK00132)Ribosomal_S9 (pfam00380)→
Psp-13-1	NZ_JXQH01000086.1	+	71385	71458	**RNA**→ rplM (PRK09216)Ribosomal_L13 (pfam00572)→ rpsI (PRK00132)Ribosomal_S9 (pfam00380)→
Psp-14-1	NZ_JXQI01000060.1	+	39882	39955	**RNA**→ rplM (PRK09216)Ribosomal_L13 (pfam00572)→ rpsI (PRK00132)Ribosomal_S9 (pfam00380)→
env-1062	scaffold253617_1_MH0006	+	404	477	**RNA**→
Psp-17-1	NZ_JXQJ01000163.1	+	35397	35470	**RNA**→ rplM (PRK09216)Ribosomal_L13 (pfam00572)→ rpsI (PRK00132)Ribosomal_S9 (pfam00380)→
env-1063	NOM022_scaffold35257_3	+	8385	8458	**RNA**→ rplM (PRK09216)Ribosomal_L13 (pfam00572)→
Psp-15-1	NZ_JXQK01000088.1	+	79922	79995	**RNA**→ rplM (PRK09216)Ribosomal_L13 (pfam00572)→ rpsI (PRK00132)Ribosomal_S9 (pfam00380)→
Psp-16-1	NZ_JXQL01000086.1	-	112978	112905	**RNA**→ rplM (PRK09216)Ribosomal_L13 (pfam00572)→ rpsI (PRK00132)Ribosomal_S9 (pfam00380)→
env-1064	BAAX01026741.1	-	768	695	**RNA**→ rplM (PRK09216)Ribosomal_L13 (pfam00572)→
env-1065	scaffold15697_4_MH0030	-	142	69	**RNA**→
env-1066	NOM022_scaffold26603_2	-	448	375	**RNA**→ rplM (PRK09216)Ribosomal_L13 (pfam00572)→
env-1067	AUXO015787294.1	-	1451	1378	**RNA**→ rplM (PRK09216)Ribosomal_L13 (pfam00572)→ rpsI (PRK00132)Ribosomal_S9 (pfam00380)→
Hse-1-1	NZ_JADU01000007.1	+	92405	92478	**RNA**→ rplM (PRK09216)Ribosomal_L13 (pfam00572)→ rpsI (PRK00132)Ribosomal_S9 (pfam00380)→
env-1068	scaffold4605_11_MH0050	-	3079	3003	**RNA**→ rplM (PRK09216)Ribosomal_L13 (pfam00572)→ rpsI (PRK00132)Ribosomal_S9 (pfam00380)→
env-1069	HCE12Call500_c0256376	-	434	359	**RNA**→ rplM (PRK09216)Ribosomal_L13 (pfam00572)→
env-1070	HCF14C_2_1_1_newblercontig23395	+	452	527	**RNA**→ rplM (PRK09216)Ribosomal_L13 (pfam00572)→
env-1071	AUXO017008710.1	+	176	252	**RNA**→ hypo→
env-1072	HCF14C_3223	-	3241	3167	**RNA**→ rplM (PRK09216)Ribosomal_L13 (pfam00572)→ rpsI (PRK00132)Ribosomal_S9 (pfam00380)→
env-1073	HCF12C_138759	-	454	380	**RNA**→ rplM (PRK09216)Ribosomal_L13 (pfam00572)→
env-1074	HCF14C_3378	+	16846	16920	**RNA**→
env-1075	HCF12C_432612	+	209	283	**RNA**→ rplM (PRK09216)Ribosomal_L13 (pfam00572)→ rpsI (PRK00132)Ribosomal_S9 (pfam00380)→
env-1076	AUXO011672201.1	-	118	44	**RNA**→
env-1077	AUXO010615907.1	-	963	889	**RNA**→ rplM (PRK09216)Ribosomal_L13 (pfam00572)→ rpsI (PRK00132)Ribosomal_S9 (pfam00380)→
env-1078	RUMENNODE_3874741_74000	+	4185	4261	**RNA**→ rplM (PRK09216)Ribosomal_L13 (pfam00572)→
env-1079	AUXO013969103.1	+	2307	2382	**RNA**→
env-1080	AUXO015357845.1	+	1768	1843	**RNA**→ rplM (PRK09216)Ribosomal_L13 (pfam00572)→ rpsI (PRK00132)Ribosomal_S9 (pfam00380)→
env-1081	SRM_contig10604	+	931	1006	**RNA**→
env-1082	SRMUA_GNVGR9Q01BHOFT	-	77	2	**RNA**→
env-1083	SRMUA_GNVGR9Q01BGO0O	-	154	79	**RNA**→
env-1084	AUXO010497420.1	+	284	358	**RNA**→ rplM (PRK09216)Ribosomal_L13 (pfam00572)→ rpsI (PRK00132)Ribosomal_S9 (pfam00380)→
env-1085	AUXO018287067.1	-	792	718	**RNA**→ rplM (PRK09216)Ribosomal_L13 (pfam00572)→ rpsI (PRK00132)Ribosomal_S9 (pfam00380)→
env-1086	AUXO015587525.1	+	974	1048	**RNA**→
env-1087	AUXO011220652.1	+	3323	3399	**RNA**→
env-1088	AUXO012321124.1	-	85	11	**RNA**→
env-1089	AUXO014609123.1	-	80	6	**RNA**→
env-1090	AUXO018327041.1	+	2387	2463	**RNA**→
env-1091	AUXO018475375.1	-	90	14	**RNA**→ ←hypo
env-1092	SRMUA_GMSDS1U03GX2P3	+	122	197	**RNA**→ rplM (PRK09216)Ribosomal_L13 (pfam00572)→
env-1093	AUXO015617850.1	+	121	196	**RNA**→ rplM (PRK09216)Ribosomal_L13 (pfam00572)→ rpsI (PRK00132)Ribosomal_S9 (pfam00380)→
env-1094	AUXO010064957.1	-	160	84	**RNA**→ hypo→
env-1095	AUXO012890930.1	-	277	202	**RNA**→
env-1096	RUMENNODE_1622158_1	+	23986	24061	**RNA**→ rplM (PRK09216)Ribosomal_L13 (pfam00572)→ rpsI (PRK00132)Ribosomal_S9 (pfam00380)→
env-1097	AUXO012504799.1	+	349	424	**RNA**→
env-1098	RUMENNODE_4144373_1	-	20587	20512	**RNA**→ rplM (PRK09216)Ribosomal_L13 (pfam00572)→ rplM (PRK09216)Ribosomal_L13 (pfam00572)→ rpsI (PRK00132)Ribosomal_S9 (pfam00380)→
Psp-7-1	NZ_KN050820.1	-	266447	266372	**RNA**→ rplM (PRK09216)Ribosomal_L13 (pfam00572)→ rpsI (PRK00132)Ribosomal_S9 (pfam00380)→
env-1099	4491686.3.305921	-	489	414	**RNA**→ rplM (PRK09216)Ribosomal_L13 (pfam00572)→
env-1100	4491686.3.312252	+	219	294	**RNA**→ rplM (PRK09216)Ribosomal_L13 (pfam00572)→
Psp-11-1	NZ_JHUW01000006.1	+	275505	275580	**RNA**→ rplM (PRK09216)Ribosomal_L13 (pfam00572)→ rpsI (PRK00132)Ribosomal_S9 (pfam00380)→

Psp-19-1	NZ_JHVE01000005.1	+	85620	85695	**RNA**→ rplM (PRK09216)Ribosomal_L13 (pfam00572)→ rpsI (PRK00132)Ribosomal_S9 (pfam00380)→
Psp-18-1	NZ_JNKF01000010.1	+	588125	588200	**RNA**→ rplM (PRK09216)Ribosomal_L13 (pfam00572)→ rpsI (PRK00132)Ribosomal_S9 (pfam00380)→
env-1101	4491686.3.93290	-	116	41	**RNA**→
Pru-1-1	NC_014033.1	+	3193901	3193976	**RNA**→ rplM (PRK09216)Ribosomal_L13 (pfam00572)→ rpsI (PRK00132)Ribosomal_S9 (pfam00380)→
Pru-2-1	NZ_JHXD01000002.1	+	95378	95453	**RNA**→ rplM (PRK09216)Ribosomal_L13 (pfam00572)→ rpsI (PRK00132)Ribosomal_S9 (pfam00380)→
Psp-20-1	NZ_JNKG01000008.1	+	135579	135654	**RNA**→ rplM (PRK09216)Ribosomal_L13 (pfam00572)→ rpsI (PRK00132)Ribosomal_S9 (pfam00380)→
env-1102	AUXO018400480.1	-	139	64	**RNA**→
env-1103	AUXO015757529.1	-	664	588	**RNA**→ rplM (PRK09216)Ribosomal_L13 (pfam00572)→
env-1104	AUXO015472756.1	-	170	95	**RNA**→
Pbr-1-1	NZ_JHXM01000009.1	-	128416	128341	**RNA**→ rplM (PRK09216)Ribosomal_L13 (pfam00572)→ rpsI (PRK00132)Ribosomal_S9 (pfam00380)→
Pbr-2-1	NZ_KK211353.1	-	323686	323611	**RNA**→ rplM (PRK09216)Ribosomal_L13 (pfam00572)→ rpsI (PRK00132)Ribosomal_S9 (pfam00380)→
env-1105	AUXO010774619.1	-	135	60	**RNA**→
env-1106	AUXO016205324.1	+	977	1053	**RNA**→
env-1107	AUXO018699643.1	-	84	9	**RNA**→
env-1108	AUXO010272107.1	+	282	357	**RNA**→ ←hypo
env-1109	AUXO014911040.1	+	6911	6987	**RNA**→ rplM (PRK09216)Ribosomal_L13 (pfam00572)→ rpsI (PRK00132)Ribosomal_S9 (pfam00380)→
env-1110	AUXO018573799.1	-	2207	2131	**RNA**→ rplM (PRK09216)Ribosomal_L13 (pfam00572)→ rpsI (PRK00132)Ribosomal_S9 (pfam00380)→
env-1111	AUXO016034740.1	-	861	785	**RNA**→ rplM (PRK09216)Ribosomal_L13 (pfam00572)→ rpsI (PRK00132)Ribosomal_S9 (pfam00380)→
env-1112	AUXO013985143.1	+	1048	1123	**RNA**→ rplM (PRK09216)Ribosomal_L13 (pfam00572)→
env-1113	AUXO012106665.1	+	1076	1151	**RNA**→ rplM (PRK09216)Ribosomal_L13 (pfam00572)→ rpsI (PRK00132)Ribosomal_S9 (pfam00380)→
env-1114	AUXO010611192.1	+	2306	2381	**RNA**→ rplM (PRK09216)Ribosomal_L13 (pfam00572)→ rpsI (PRK00132)Ribosomal_S9 (pfam00380)→
env-1115	AUXO017780793.1	+	2412	2486	**RNA**→ rplM (PRK09216)Ribosomal_L13 (pfam00572)→
env-1116	AUXO016964339.1	-	2517	2443	**RNA**→ ←hypo
env-1117	DLM014_scaffold29163_3	-	749	677	**RNA**→ rplM (PRK09216)Ribosomal_L13 (pfam00572)→ rpsI (PRK00132)Ribosomal_S9 (pfam00380)→
env-1118	AUXO012501382.1	+	1300	1372	**RNA**→ rplM (PRK09216)Ribosomal_L13 (pfam00572)→
env-1119	RUMENNODE_4186824_1	-	20452	20380	**RNA**→ rplM (PRK09216)Ribosomal_L13 (pfam00572)→ rpsI (PRK00132)Ribosomal_S9 (pfam00380)→
env-1120	HCE12Call500_c0163880	-	1019	945	**RNA**→ rplM (PRK09216)Ribosomal_L13 (pfam00572)→ rpsI (PRK00132)Ribosomal_S9 (pfam00380)→
env-1121	HCF14C_7730	+	2022	2096	**RNA**→ rplM (PRK09216)Ribosomal_L13 (pfam00572)→ rpsI (PRK00132)Ribosomal_S9 (pfam00380)→
env-1122	HCF12C_211171	+	370	445	**RNA**→ rplM (PRK09216)Ribosomal_L13 (pfam00572)→
env-1123	4491686.3.18377	-	3185	3110	**RNA**→ rplM (PRK09216)Ribosomal_L13 (pfam00572)→ rpsI (PRK00132)Ribosomal_S9 (pfam00380)→
env-1124	4491686.3.998042	+	30719	30794	**RNA**→ rplM (PRK09216)Ribosomal_L13 (pfam00572)→ rpsI (PRK00132)Ribosomal_S9 (pfam00380)→
env-1125	HCF12C_268453	+	254	328	**RNA**→
env-1126	HCF12C_148665	+	852	926	**RNA**→ hypo→
env-1127	HCE12Call500_c0204626	-	679	605	**RNA**→ rplM (PRK09216)Ribosomal_L13 (pfam00572)→ rpsI (PRK00132)rps9 (CHL00079)→
env-1128	HCF12C_185472	+	268	342	**RNA**→ rplM (PRK09216)RplM (COG0102)→
env-1129	4491421.3_NODE_9264	+	154	230	**RNA**→ rplM (PRK09216)Ribosomal_L13 (pfam00572)→
env-1130	AUXO015981657.1	-	1275	1203	**RNA**→ rplM (PRK09216)Ribosomal_L13 (pfam00572)→ rpsI (PRK00132)Ribosomal_S9 (pfam00380)→
env-1131	AUXO010201767.1	+	271	345	**RNA**→ rplM (PRK09216)Ribosomal_L13 (pfam00572)→
env-1132	AUXO014390899.1	-	563	489	**RNA**→ rplM (PRK09216)Ribosomal_L13 (pfam00572)→
env-1133	AUXO015967901.1	+	641	712	**RNA**→ rplM (PRK09216)Ribosomal_L13 (pfam00572)→ rpsI (PRK00132)Ribosomal_S9 (pfam00380)→
env-1134	AUXO015633410.1	+	206	279	**RNA**→ rplM (PRK09216)Ribosomal_L13 (pfam00572)→ rpsI (PRK00132)Ribosomal_S9 (pfam00380)→
env-1135	AUXO017839357.1	+	144	211	**RNA**→ rplM (PRK09216)Ribosomal_L13 (pfam00572)→
env-1136	AUXO016134445.1	+	192	259	**RNA**→ rplM (PRK09216)Ribosomal_L13 (pfam00572)→ rpsI (PRK00132)Ribosomal_S9 (pfam00380)→
env-1137	SRS064449_C3977517	+	160	232	**RNA**→ rplM (PRK09216)RplM (COG0102)→
env-1138	SRS013170_C4245439	-	179	107	**RNA**→
env-1139	SRS019071_C4979592	+	177	249	**RNA**→
env-1140	SRS063999_C3760459	+	1331	1403	**RNA**→ rplM (PRK09216)Ribosomal_L13 (pfam00572)→ rpsI (PRK00132)Ribosomal_S9 (pfam00380)→
env-1141	SRS053917_LANL_scaffold_97956	+	384	456	**RNA**→ rplM (PRK09216)Ribosomal_L13 (pfam00572)→
env-1142	SRS049318_LANL_scaffold_147506	+	27	99	**RNA**→ rplM (PRK09216)Ribosomal_L13 (pfam00572)→
env-1143	SRS047634_C4471531	-	1001	929	**RNA**→ rplM (PRK09216)Ribosomal_L13 (pfam00572)→ rpsI (PRK00132)Ribosomal_S9 (pfam00380)→
env-1144	SRS022602_Baylor_scaffold_89900	-	465	393	**RNA**→ rplM (PRK09216)Ribosomal_L13 (pfam00572)→
env-1145	SRS019028_C2913254	-	170	98	**RNA**→

env-1146	SRS063603_LANL_scaffold_35578	+	6849	6921	**RNA**→ rplM (PRK09216)Ribosomal_L13 (pfam00572)→ rpsI (PRK00132)Ribosomal_S9 (pfam00380)→
env-1147	SRS016225_WUGC_scaffold_29763	+	2155	2226	**RNA**→
env-1148	SRS022621_Baylor_scaffold_78299	+	1192	1263	**RNA**→ rplM (PRK09216)Ribosomal_L13 (pfam00572)→
env-1149	SRS022719_LANL_scaffold_24264	-	1070	999	**RNA**→ rplM (PRK09216)Ribosomal_L13 (pfam00572)→ rpsI (PRK00132)Ribosomal_S9 (pfam00380)→ rpsI (PRK00132)Ribosomal_S9 (pfam00380)→
env-1150	SRS023926_C2746934	+	199	270	**RNA**→ rplM (PRK09216)Ribosomal_L13 (pfam00572)→
env-1151	SRS014888_C3094079	-	1005	933	**RNA**→ rplM (PRK09216)Ribosomal_L13 (pfam00572)→ rpsI (PRK00132)Ribosomal_S9 (pfam00380)→
env-1152	SRS057355_LANL_scaffold_38534	+	74	146	**RNA**→ rplM (PRK09216)Ribosomal_L13 (pfam00572)→
env-1153	SRS018739_C3681903	-	158	86	**RNA**→
env-1154	SRS013164_Baylor_scaffold_53891	-	1272	1200	**RNA**→ rplM (PRK09216)Ribosomal_L13 (pfam00572)→ rpsI (PRK00132)Ribosomal_S9 (pfam00380)→
env-1155	SRS015797_WUGC_scaffold_34484	-	665	593	**RNA**→ rplM (PRK09216)Ribosomal_L13 (pfam00572)→ rpsI (PRK00132)Ribosomal_S9 (pfam00380)→
env-1156	SRS042131_WUGC_scaffold_59323	-	801	729	**RNA**→ rplM (PRK09216)Ribosomal_L13 (pfam00572)→ rpsI (PRK00132)Ribosomal_S9 (pfam00380)→
env-1157	SRS024441_LANL_scaffold_55878	-	1004	932	**RNA**→ rplM (PRK09216)Ribosomal_L13 (pfam00572)→ rpsI (PRK00132)Ribosomal_S9 (pfam00380)→
env-1158	SRS016037_WUGC_scaffold_44051	-	27077	27005	**RNA**→ rplM (PRK09216)Ribosomal_L13 (pfam00572)→ rpsI (PRK00132)Ribosomal_S9 (pfam00380)→
env-1159	SRS018357_Baylor_scaffold_49795	+	563	635	**RNA**→
env-1160	SRS044662_LANL_scaffold_9532	+	25254	25326	**RNA**→ rplM (PRK09216)Ribosomal_L13 (pfam00572)→ rpsI (PRK00132)Ribosomal_S9 (pfam00380)→
env-1161	SRS045127_LANL_scaffold_15711	-	3179	3107	**RNA**→ rplM (PRK09216)Ribosomal_L13 (pfam00572)→ rpsI (PRK00132)Ribosomal_S9 (pfam00380)→
env-1162	SRS049147_C4127564	+	678	750	**RNA**→
env-1163	Palatine_Tonsils_LANL_C6209475	+	331	403	**RNA**→ rplM (PRK09216)Ribosomal_L13 (pfam00572)→
env-1164	SRS015434_C5847974	-	383	311	**RNA**→ rplM (PRK09216)Ribosomal_L13 (pfam00572)→
env-1165	SRS017533_Baylor_scaffold_51088	+	1318	1390	**RNA**→
env-1166	SRS019327_C2789739	+	1129	1201	**RNA**→ rplM (PRK09216)Ribosomal_L13 (pfam00572)→
env-1167	Buccal_mucosa_LANL_scaffold_123568	-	308	236	**RNA**→ rplM (PRK09216)Ribosomal_L13 (cd00392)→
env-1168	SRS015985_C1563967	+	138	210	**RNA**→ rplM (PRK09216)Ribosomal_L13 (pfam00572)→
env-1169	SRS062878_LANL_scaffold_81556	+	2146	2218	**RNA**→ rplM (PRK09216)RplM (COG0102)→
env-1170	SRS075404_C3564214	-	158	86	**RNA**→ hypo→
env-1171	Throat_LANL_C5768501	+	142	214	**RNA**→ rplM (PRK09216)Ribosomal_L13 (pfam00572)→
env-1172	SRS011306_Baylor_scaffold_57099	+	475	547	**RNA**→ rplM (PRK09216)RplM (COG0102)→
env-1173	SRS013879_WUGC_scaffold_12081	+	2721	2793	**RNA**→ rplM (PRK09216)Ribosomal_L13 (pfam00572)→ rpsI (PRK00132)Ribosomal_S9 (pfam00380)→
env-1174	SRS013947_C1628089	-	350	278	**RNA**→ rplM (PRK09216)Ribosomal_L13 (pfam00572)→
env-1175	SRS013948_WUGC_scaffold_13434	+	25402	25474	**RNA**→ rplM (PRK09216)Ribosomal_L13 (pfam00572)→
env-1176	SRS014271_C2902322	-	158	86	**RNA**→
env-1177	SRS015762_WUGC_scaffold_30677	-	1005	933	**RNA**→ rplM (PRK09216)Ribosomal_L13 (pfam00572)→ rpsI (PRK00132)Ribosomal_S9 (pfam00380)→
env-1178	SRS016002_C2530304	+	27	99	**RNA**→ rplM (PRK09216)Ribosomal_L13 (pfam00572)→
env-1179	SRS016569_C2383578	+	27	99	**RNA**→ rplM (PRK09216)Ribosomal_L13 (pfam00572)→
env-1180	SRS017209_Baylor_scaffold_3818	+	25398	25470	**RNA**→ rplM (PRK09216)Ribosomal_L13 (pfam00572)→ rpsI (PRK00132)Ribosomal_S9 (pfam00380)→
env-1181	SRS018145_Baylor_scaffold_64	+	25195	25267	**RNA**→ rplM (PRK09216)Ribosomal_L13 (pfam00572)→ rpsI (PRK00132)Ribosomal_S9 (pfam00380)→
env-1182	SRS018439_Baylor_scaffold_41344	-	879	807	**RNA**→ rplM (PRK09216)Ribosomal_L13 (pfam00572)→ rpsI (PRK00132)Ribosomal_S9 (pfam00380)→
env-1183	SRS019071_C4950970	-	163	91	**RNA**→ hypo→
env-1184	SRS019120_C1301558	+	33	105	**RNA**→ rplM (PRK09216)Ribosomal_L13 (pfam00572)→
env-1185	SRS019126_WUGC_scaffold_35353	+	161	233	**RNA**→ rplM (PRK09216)Ribosomal_L13 (pfam00572)→ rpsI (PRK00132)Ribosomal_S9 (pfam00380)→
env-1186	SRS023958_Baylor_scaffold_34017	+	4806	4878	**RNA**→ rplM (PRK09216)Ribosomal_L13 (pfam00572)→ rpsI (PRK00132)Ribosomal_S9 (pfam00380)→
env-1187	SRS024081_C2561546	+	437	509	**RNA**→
env-1188	SRS050669_LANL_scaffold_65278	-	28287	28215	**RNA**→ rplM (PRK09216)Ribosomal_L13 (pfam00572)→ rpsI (PRK00132)Ribosomal_S9 (pfam00380)→
env-1189	SRS057205_LANL_scaffold_2683	-	1005	933	**RNA**→ rplM (PRK09216)Ribosomal_L13 (pfam00572)→ rpsI (PRK00132)Ribosomal_S9 (pfam00380)→
env-1190	SRS057692_C2560902	+	134	206	**RNA**→
env-1191	SRS058336_LANL_scaffold_19337	-	5666	5594	**RNA**→ rplM (PRK09216)Ribosomal_L13 (pfam00572)→ rpsI (PRK00132)Ribosomal_S9 (pfam00380)→
env-1192	SRS015057_WUGC_scaffold_40957	-	277	205	**RNA**→ rplM (PRK09216)Ribosomal_L13 (pfam00572)→
env-1193	SRS063603_LANL_scaffold_3918	-	1001	931	**RNA**→ rplM (PRK09216)Ribosomal_L13 (pfam00572)→ rpsI (PRK00132)Ribosomal_S9 (pfam00380)→
env-1194	SRS016746_Baylor_scaffold_3854	+	350	421	**RNA**→
Psp-1-1	NZ_KB290717.1	-	518225	518153	**RNA**→ rplM (PRK09216)Ribosomal_L13 (pfam00572)→ rpsI (PRK00132)Ribosomal_S9 (pfam00380)→
env-1195	SRS013949_C3355882	-	134	64	**RNA**→ hypo→

env-1196	SRS024087_C3225267	-	91	21	RNA→ hypo→
env-1197	SRS013950_C2065648	-	258	188	RNA→ rplM (PRK09216)RplM (COG0102)→
env-1198	SRS018157_C4805273	-	576	506	RNA→ rplM (PRK09216)Ribosomal_L13 (pfam00572)→
env-1199	SRS019128_C4141267	+	154	224	RNA→
env-1200	SRS047634_LANL_scaffold_18390	+	479	549	RNA→ rplM (PRK09216)Ribosomal_L13 (pfam00572)→ rpsI (PRK00132)Ribosomal_S9 (pfam00380)→
env-1201	SRS055401_C2292367	-	364	294	RNA→ rplM (PRK09216)Ribosomal_L13 (pfam00572)→
env-1202	SRS063932_LANL_scaffold_79042	+	232	302	RNA→ rplM (PRK09216)Ribosomal_L13 (pfam00572)→ rpsI (PRK00132)Ribosomal_S9 (pfam00380)→
env-1203	SRS015803_WUGC_scaffold_2190	+	350	420	RNA→ rplM (PRK09216)Ribosomal_L13 (pfam00572)→ rpsI (PRK00132)Ribosomal_S9 (pfam00380)→
env-1204	SRS024289_LANL_scaffold_56240	+	232	302	RNA→ rplM (PRK09216)Ribosomal_L13 (pfam00572)→ rpsI (PRK00132)Ribosomal_S9 (pfam00380)→
env-1205	SRS023938_C2238754	-	301	231	RNA→ rplM (PRK09216)Ribosomal_L13 (pfam00572)→
env-1206	SRS012285_C2904644	-	382	310	RNA→ rplM (PRK09216)Ribosomal_L13 (pfam00572)→
env-1207	SRS017227_Baylor_scaffold_121114	+	26	98	RNA→ rplM (PRK09216)Ribosomal_L13 (pfam00572)→ rpsI (PRK00132)Ribosomal_S9 (pfam00380)→
env-1208	SRS023595_Baylor_scaffold_24496	+	230	302	RNA→ rplM (PRK09216)Ribosomal_L13 (pfam00572)→ rpsI (PRK00132)Ribosomal_S9 (pfam00380)→
env-1209	SRS047113_C5260445	-	272	200	RNA→ rplM (PRK09216)RplM (COG0102)→
env-1210	SRS050628_C1880365	+	114	186	RNA→ rplM (PRK09216)Ribosomal_L13 (pfam00572)→
env-1211	SRS019028_C3034176	-	899	827	RNA→ rplM (PRK09216)Ribosomal_L13 (pfam00572)→ rpsI (PRK00132)Ribosomal_S9 (pfam00380)→
env-1212	SRS019029_C3106614	+	230	302	RNA→
env-1213	SRS016037_C2055413	-	3265	3192	RNA→ rplM (PRK09216)Ribosomal_L13 (pfam00572)→ rpsI (PRK00132)Ribosomal_S9 (pfam00380)→
env-1214	SRS022719_LANL_scaffold_71174	-	5214	5141	RNA→ rplM (PRK09216)Ribosomal_L13 (pfam00572)→ rpsI (PRK00132)Ribosomal_S9 (pfam00380)→
env-1215	SRS044662_LANL_scaffold_46735	-	2254	2181	RNA→ rplM (PRK09216)Ribosomal_L13 (pfam00572)→ rpsI (PRK00132)Ribosomal_S9 (pfam00380)→
env-1216	SRS013164_Baylor_scaffold_32078	+	335	407	RNA→ rplM (PRK09216)Ribosomal_L13 (pfam00572)→ rpsI (PRK00132)Ribosomal_S9 (pfam00380)→
env-1217	SRS013879_WUGC_scaffold_46467	-	1318	1246	RNA→ rplM (PRK09216)Ribosomal_L13 (pfam00572)→ rpsI (PRK00132)Ribosomal_S9 (pfam00380)→
env-1218	SRS015434_C5929714	-	1009	937	RNA→ rplM (PRK09216)Ribosomal_L13 (pfam00572)→ rpsI (PRK00132)Ribosomal_S9 (pfam00380)→
env-1219	SRS019071_C5025454	-	172	100	RNA→ hypo→
env-1220	SRS023958_Baylor_scaffold_23657	+	335	407	RNA→ rplM (PRK09216)Ribosomal_L13 (pfam00572)→ rpsI (PRK00132)Ribosomal_S9 (pfam00380)→
env-1221	SRS049389_WUGC_scaffold_42978	-	343	271	RNA→ rplM (PRK09216)Ribosomal_L13 (pfam00572)→
env-1222	SRS052227_LANL_scaffold_7065	-	172	100	RNA→
env-1223	SRS011140_C5077184	+	62	134	RNA→ rplM (PRK09216)Ribosomal_L13 (pfam00572)→
env-1224	SRS016225_WUGC_scaffold_16679	-	161	89	RNA→
env-1225	SRS017439_C3816727	+	159	231	RNA→ rplM (PRK09216)Ribosomal_L13 (pfam00572)→ rpsI (PRK00132)Ribosomal_S9 (pfam00380)→
env-1226	SRS018357_Baylor_scaffold_50190	-	1318	1246	RNA→ rplM (PRK09216)Ribosomal_L13 (pfam00572)→ rpsI (PRK00132)Ribosomal_S9 (pfam00380)→
env-1227	SRS019327_WUGC_scaffold_31	+	300	372	RNA→ rplM (PRK09216)Ribosomal_L13 (pfam00572)→ rpsI (PRK00132)Ribosomal_S9 (pfam00380)→
env-1228	SRS013239_Baylor_scaffold_8550	+	223	295	RNA→ rplM (PRK09216)Ribosomal_L13 (pfam00572)→ rpsI (PRK00132)Ribosomal_S9 (pfam00380)→
env-1229	SRS013252_C3407129	+	146	218	RNA→
env-1230	SRS019329_C822464	-	1070	998	RNA→ rplM (PRK09216)Ribosomal_L13 (pfam00572)→ rpsI (PRK00132)Ribosomal_S9 (pfam00380)→
env-1231	SRS045049_C1487558	-	103	31	RNA→
env-1232	SRS046686_C923094	+	169	241	RNA→ rplM (PRK09216)Ribosomal_L13 (pfam00572)→ rpsI (PRK00132)Ribosomal_S9 (pfam00380)→
env-1233	SRS012279_C2693900	-	583	511	RNA→ rplM (PRK09216)Ribosomal_L13 (pfam00572)→
env-1234	SRS014573_WUGC_scaffold_5954	+	353	425	RNA→ rplM (PRK09216)Ribosomal_L13 (pfam00572)→ rpsI (PRK00132)Ribosomal_S9 (pfam00380)→
env-1235	SRS014888_C3088705	-	1002	930	RNA→ rplM (PRK09216)Ribosomal_L13 (pfam00572)→ rpsI (PRK00132)Ribosomal_S9 (pfam00380)→
env-1236	SRS018145_Baylor_scaffold_49105	-	1318	1246	RNA→ rplM (PRK09216)Ribosomal_L13 (pfam00572)→ rpsI (PRK00132)Ribosomal_S9 (pfam00380)→
env-1237	SRS019122_C5893890	+	223	295	RNA→ rplM (PRK09216)Ribosomal_L13 (pfam00572)→ rpsI (PRK00132)Ribosomal_S9 (pfam00380)→
env-1238	SRS019126_C2101879	-	343	271	RNA→ rplM (PRK09216)Ribosomal_L13 (pfam00572)→
env-1239	SRS021496_Baylor_scaffold_82682	+	222	294	RNA→ rplM (PRK09216)Ribosomal_L13 (pfam00572)→ rpsI (PRK00132)Ribosomal_S9 (pfam00380)→
env-1240	SRS054569_C1464969	-	199	127	RNA→ hypo→
env-1241	SRS057205_LANL_scaffold_57883	-	1002	930	RNA→ rplM (PRK09216)Ribosomal_L13 (pfam00572)→ rpsI (PRK00132)Ribosomal_S9 (pfam00380)→
env-1242	SRS057692_LANL_scaffold_10341	+	335	407	RNA→ rplM (PRK09216)Ribosomal_L13 (pfam00572)→ rpsI (PRK00132)Ribosomal_S9 (pfam00380)→
env-1243	SRS018739_C3498499	-	172	100	RNA→ hypo→
env-1244	SRS013946_WUGC_scaffold_9812	+	797	869	RNA→
env-1245	SRS017127_Baylor_scaffold_10764	-	3240	3168	RNA→ rplM (PRK09216)Ribosomal_L13 (pfam00572)→ rpsI (PRK00132)Ribosomal_S9 (pfam00380)→
env-1246	SRS019120_C1331431	+	223	295	RNA→ rplM (PRK09216)Ribosomal_L13 (pfam00572)→
env-1247	SRS019124_WUGC_scaffold_15768	+	336	408	RNA→ rplM (PRK09216)Ribosomal_L13 (pfam00572)→ rpsI (PRK00132)Ribosomal_S9 (pfam00380)→

env-1248	SRS019125_WUGC_scaffold_5306	+	336	408	RNA→ rplM (PRK09216)Ribosomal_L13 (pfam00572)→ rpsI (PRK00132)Ribosomal_S9 (pfam00380)→
env-1249	SRS019128_C4205376	+	199	271	RNA→
env-1250	SRS047113_C5255867	-	272	200	RNA→ rplM (PRK09216)RplM (COG0102)→
env-1251	SRS014473_WUGC_scaffold_265	-	32420	32348	RNA→ rplM (PRK09216)Ribosomal_L13 (pfam00572)→ rpsI (PRK00132)Ribosomal_S9 (pfam00380)→
env-1252	SRS015060_WUGC_scaffold_459	+	456	528	RNA→ rplM (PRK09216)Ribosomal_L13 (pfam00572)→ rpsI (PRK00132)Ribosomal_S9 (pfam00380)→
env-1253	SRS063272_LANL_scaffold_10757	-	7611	7539	RNA→ rplM (PRK09216)Ribosomal_L13 (pfam00572)→ rpsI (PRK00132)Ribosomal_S9 (pfam00380)→
env-1254	SRS017215_C2622978	+	37	109	RNA→ rplM (PRK09216)Ribosomal_L13 (pfam00572)→ rpsI (PRK00132)Ribosomal_S9 (pfam00380)→
env-1255	SRS049318_C6258073	-	173	101	RNA→ hypo→
env-1256	SRS016225_WUGC_scaffold_16862	-	163	90	RNA→ hypo→
env-1257	SRS018300_C3300755	-	410	337	RNA→ rplM (PRK09216)Ribosomal_L13 (pfam00572)→
env-1258	SRS013506_Baylor_scaffold_6896	+	328	400	RNA→ rplM (PRK09216)Ribosomal_L13 (pfam00572)→ rpsI (PRK00132)Ribosomal_S9 (pfam00380)→
env-1259	SRS049147_C4035837	-	173	101	RNA→ hypo→
Pmu-2-1	NZ_GL945017.1	+	791406	791482	RNA→ rplM (PRK09216)Ribosomal_L13 (pfam00572)→ rplM (PRK09216)Ribosomal_L13 (pfam00572)→ rpsI (PRK00132)Ribosomal_S9 (pfam00380)→ rpsI (PRK00132)Ribosomal_S9 (pfam00380)→
Pde-1-1	NC_019960.1	-	1444430	1444357	RNA→ rplM (PRK09216)Ribosomal_L13 (pfam00572)→ rpsI (PRK00132)Ribosomal_S9 (pfam00380)→

3.3 Conserved domains

Conserved domains found in protein-coding genes listed in Section 3.2 are shown below, with the first sentence in their description from the Conserved Domain Database (if any). Conserved domains associated with more than one L13-Bacteroidia RNA are assigned a color, while others are shown in gray. The number in parentheses after the colored domain name is the number of occurrences in Section 3.2.

cd00392 (6) Ribosomal protein L13.
cd14275 (1) UBA domain found in elongation factor Ts (EF-Ts) from bacteria, chloroplasts and mitochondria of eukaryotes.
CHL00079 (3) ribosomal protein S9
COG0102 (26) Ribosomal protein L13 [Translation, ribosomal structure and biogenesis]
pfam00318 (2) Ribosomal protein S2.
pfam00380 (712) Ribosomal protein S9/S16.
pfam00572 (955) Ribosomal protein L13.
pfam00889 (1) Elongation factor TS.
PRK00132 (715) 30S ribosomal protein S9; Reviewed
PRK05299 (2) 30S ribosomal protein S2; Provisional
PRK09216 (990) 50S ribosomal protein L13; Reviewed
PRK09377 (1) elongation factor Ts; Provisional

3.4 Multiple-sequence alignment

Each L13-Bacteroidia RNA is denoted by an abbreviation (like "Eco-1-1") that refers to a taxonomy given in Section 3.1. The alignment may include sequences containing the RNA motif, as well as flanking sequence. The L13-Bacteroidia RNA itself is denoted by the line underneath marked 5′ and 3′ on either end. Nucleotides in flanking sequences (i.e., not part of the motif) are written in gray letters. Stems of predicted rho-independent transcription terminators, if any, are shaded yellow. (Note: terminator predictions have not been analyzed manually, and many are likely to be false positives. Terminator predictions are those of the RNIE software.) Nucleotides predicted to function (as DNA) as transcription-factor binding sites, if any, are shaded in green. (But note: these predictions are manually annotated, so they might be under-predicted.) Annotated start codons, if any, are shaded green. (Note: start codons are frequently misannotated, especially in environmental samples.) Nucleotides proposed to basepair as part of the consensus structure are shaded in color when they comprise Watson-Crick or G-U pairs. Otherwise they are shaded gray. Conserved stems are also indicated at the bottom of the alignment by angle brackets, where matching $<$ and $>$ denote base-paired columns. Below these angle brackets, the symbol "2" denotes base pairs exhibiting covariation according to the statistically well-founded R-scape method. "1" denotes base pairs exhibiting covariation according to R2R's simplistic method. "0" denotes base pairs that are not observed to mutate and "?" denotes base pairs that have a significant frequency of non-canonical nucleotides for Watson-Crick or G-U pairs ($> 5\%$). Below these base pair annotation is the consensus sequence: "R" = "A" or "G", "Y" = "C" or "U", red nucleotides: nucleotide identity conserved more than 97% of the time, black nucleotides: 90%, gray nucleotides: 75%, red circle (•): nucleotide is present 97% of the time, black circle (•): 90%, gray circle (•): 75%, white circle (◦): 50%. All percentages of sequences just described (e.g. 97% conserved) assume that sequences have been weighted by the GSC algorithm implemented by the Infernal software package.

Duplicate sequences: the following putative homologs are not shown in the alignment because their sequences are identical to a homolog already shown: Bsp-1-1, Pal-1-2, Pam-2-1, Pam-4-1, Pba-1-2, Pbi-2-1, Pbi-3-1, Pbi-4-1, Pbi-5-1, Pbi-6-1, Pbr-4-1, Pbu-6-1, Pcl-1-1, Pco-1-1, Pco-3-2, Pde-5-2, Phi-1-1, Pin-2-1, Plo-1-1, Pma-2-1, Pma-5-1, Pme-1-1, Pme-2-1, Pmi-1-1, Pmi-2-1, Pmi-3-1, Pna-1-1, Pna-2-1, Pni-2-1, Por-3-1, Por-4-1, Por-7-1, Ppa-1-1, Ppa-2-1, Ppl-1-1, Pru-2-1, Psa-1-1, Psa-2-1, Psa-3-1, Psh-1-1, Psp-10-1, Psp-14-1, Psp-18-1, Psp-20-1, Psp-22-1, Psp-23-1, Pst-1-1, Pve-2-1, Pxy-1-1, env-100, env-1000, env-101, env-1010, env-1011, env-1012, env-1013, env-1014, env-1015, env-1016, env-1018, env-1019, env-102, env-1026, env-103, env-1030, env-1036, env-104, env-1041, env-1042, env-1045, env-1047, env-1048, env-1049, env-105, env-1050, env-1051, env-1057, env-106, env-107, env-1070, env-1073, env-108, env-1082, env-109, env-110, env-111, env-112, env-1121, env-1124, env-1128, env-113, env-114, env-1142, env-1146, env-115, env-1150, env-1152, env-116, env-1160, env-1161, env-1162, env-1168, env-1169, env-117, env-1170, env-1171, env-1172, env-1173, env-1174, env-1175, env-1176, env-1177, env-1178, env-1179, env-118, env-1180, env-1181, env-1182, env-1183, env-1184, env-1185, env-1186, env-1187, env-1188, env-1189, env-119, env-1190, env-1191, env-1196, env-1198, env-1199, env-120, env-1200, env-1201, env-1202, env-1204, env-1207, env-1208, env-1209, env-121, env-1210, env-1212, env-1214, env-1215, env-1217, env-1218, env-1219, env-122, env-1220, env-1221, env-1222, env-1225, env-1226, env-1227, env-1229, env-123, env-1230, env-1231, env-1232, env-1234, env-1235, env-1236, env-1237, env-1238, env-1239, env-124, env-1240, env-1241, env-1242, env-1246, env-1247, env-1248, env-1249, env-125, env-1252, env-1253, env-1255, env-1257, env-127, env-128, env-130, env-131, env-133, env-134, env-135, env-136, env-137, env-138, env-139, env-140, env-141, env-142, env-143, env-144, env-145, env-146, env-147, env-149, env-150, env-151, env-152, env-153, env-154, env-156, env-161, env-162, env-172, env-173, env-174, env-175, env-176, env-177, env-178, env-179, env-180, env-181, env-182, env-183, env-184, env-185, env-186, env-187, env-188, env-189, env-19, env-190, env-191, env-192, env-193, env-194, env-195, env-196, env-197, env-198, env-199, env-20, env-200, env-201, env-202, env-203, env-204, env-214, env-215, env-216, env-217, env-218, env-219, env-220, env-221, env-223, env-224, env-225, env-228, env-23, env-235, env-236, env-237, env-238, env-239, env-24, env-240, env-241, env-242, env-243, env-244, env-245, env-247, env-25, env-251, env-252, env-253, env-254, env-255, env-256, env-261, env-263, env-271, env-274, env-277, env-28, env-282, env-283, env-284, env-289, env-29, env-294, env-296, env-30, env-300, env-303, env-304, env-306, env-31, env-312, env-313, env-32, env-325, env-33, env-332, env-333, env-334, env-335, env-34, env-340, env-342, env-343, env-344, env-346, env-35, env-352, env-356, env-358, env-36, env-364, env-365, env-366, env-367, env-37, env-372, env-374, env-376, env-377, env-378, env-379, env-38, env-380, env-381, env-382, env-383, env-39, env-391, env-393, env-394, env-395, env-40, env-404, env-405, env-407, env-409, env-41, env-415, env-416, env-417, env-42, env-424, env-427, env-428, env-43, env-430, env-44, env-45, env-456, env-459, env-46, env-460, env-461, env-465, env-47, env-472, env-475, env-478, env-479, env-48, env-480, env-481, env-482, env-484, env-49, env-496, env-499, env-50, env-500, env-505, env-506, env-51, env-513, env-515, env-518, env-52, env-524, env-528, env-529, env-53, env-530, env-533, env-536, env-539, env-54, env-540, env-541, env-542, env-543, env-544, env-545, env-546, env-547, env-548, env-549, env-55, env-550, env-551, env-556, env-56, env-57, env-58, env-581, env-584, env-585, env-586, env-587, env-588, env-59, env-590, env-591, env-60, env-601, env-602, env-603, env-604, env-605, env-606, env-607, env-608, env-609, env-61, env-610, env-611, env-612, env-614, env-615, env-616, env-617, env-618, env-619, env-62, env-620, env-621, env-622, env-623, env-624, env-625, env-626, env-627, env-628, env-629, env-630, env-631, env-632, env-633, env-634, env-635, env-636, env-637, env-638, env-639, env-640, env-641, env-642, env-643, env-644, env-645, env-646, env-647, env-649, env-65, env-650, env-654, env-655, env-656, env-658, env-659, env-66, env-660, env-661, env-662, env-663, env-664, env-665, env-666, env-667, env-668, env-669, env-67, env-670, env-671, env-672, env-673, env-674, env-675, env-678, env-68, env-685, env-687, env-688, env-689, env-69, env-690, env-691, env-692, env-693, env-694, env-695, env-697, env-701, env-702, env-703, env-704, env-705, env-706, env-707, env-708, env-712, env-713, env-714, env-715, env-716, env-717, env-718, env-719, env-720, env-721, env-722, env-723, env-728, env-730, env-731, env-732, env-739, env-740, env-741, env-750, env-764, env-773, env-777, env-778, env-779, env-782, env-783, env-784, env-786, env-792, env-794, env-795, env-796, env-805, env-806, env-807, env-809, env-810, env-811, env-812, env-813, env-814, env-815, env-816, env-817, env-818, env-819, env-820, env-823, env-841, env-844, env-85, env-850, env-853, env-854, env-863, env-867, env-871, env-879, env-884, env-89, env-895, env-896, env-897, env-898, env-899, env-90, env-904, env-905, env-906, env-907, env-908, env-909, env-91, env-910, env-911, env-912, env-913, env-914, env-915, env-916, env-917, env-918, env-919, env-92, env-920, env-925, env-926, env-927, env-928, env-929, env-93, env-930, env-931, env-933, env-934, env-935, env-936, env-937, env-938, env-939, env-94, env-940, env-941, env-942, env-943, env-944, env-945, env-946, env-947, env-95, env-951, env-952, env-954, env-955, env-956, env-959, env-96, env-962, env-963, env-964, env-965, env-966, env-969, env-97, env-974, env-975, env-976, env-980, env-987, env-988, env-989, env-99, env-991, env-998

alignment positions $1 \cdots 79$

```
env-1   CACCGGUUUCAACAGGAAACUUACAGCGAAGACCUUGCCAAUAAAUAAAAUAGUUGUACUUUUGCGCGCCGAUUAUUAU
env-2   CGUUCAGAUACACAAAAAGUUGCAAAAAAGUUUUGUCGGUUCACAGAAAGUUCGUACCUUUGCACGCCGAUUAUUAUCA
env-3   ACGCCGCAAAAUCACGAAAAAAAAGACAAGUAGAUAUUUGCCAUGAAUAAAAAAAAAUAGUACUUUUGCACGCCGAUUAUU
env-4   CCAAAGAAGUAGCAAAAAUAAGUAAAAAAGUUUUGUCAGUUCGUGAAAAAGCCGUACCUUUGCACGCCGAUUAUUAUCA
env-5   AUAAUAUAUAAACAAACAUAACAACACGCCUCACAGCAUAAGAAUACUCAUUAUAUAUAUGUAACUUUGCAAACUGGAU
env-6   AAUGAAAGUGCGAAAAAAACUUAAAAAAAUCUUUGUUGGAUGGGGGAAAAGUGCUAACUUUGCAAGCCGAUUAUUAUCAA

        ...............................................................................
        ...............................................................................
        ●●●●●●●●●●●●●●●●●●●●●●●●●●●●●●●●●●●●●●●●●●●●●●●●●R●●●●●●●●●●Y●Y●●●●●●●●●●●●●Y●●
```

```
env-7     AGUAUGAAAGUGCGAAAAAAACUUAAAAAAUCUUGGUCAGAUGGUGGAAAAGUGCUAACUUUGCAAGCCGAUUAUUAUC
env-8     AACGAAUUACGGCAAAAAAUCAUAAAGAAAUCAUCGCCACAUAAAAAAUAAAAUUGUAAUUUUGCACACCGAUUAUUAUC
env-9     AACGAAUUACGGCAAAAAAUCAUAAAGAAAUCAUCGCCACAUAAAAAAUAAAAUCGUAAUUUUGCGCACCGAUUAUUAUC
env-10    AACGAAUUACGGCAAAAAAUCAUAAAGAAAUCAUCGCCACAUAAAAAAUAAAAUUGUAAUUUUGCGCACCGAUUAUUAUC
env-11    AAAAACCAGCAAAAAAGUCGCGGCAGGGGUUUGCUGUUCUGUGAAAAAGUCAUACCUUUGCACGCCGAUUAUUAUCAAG
env-12    CCUUAAAUAAAAAUAAUUGGCUGUAAACCAUUGUGGUUUGGCAUAAAAAAUCGUAACUUUGCAAGCCGAUUAUUAUCAAA
env-13    GAAAUCUGACAAAAAAAAACGCUCAGCUUUGCUUGCUGAAUAAAAAGAAAGUCGUACUUUUGCACGCCGAUUAUUAUCA
env-14    CAGGGAAGAAAAGAAAAACAACGGGGGGAACCUUGCCAUUCCAAUGAAUUUUAGUACUUUUGCACGCCGAUUAUUAUCA
env-15    AAAACAUAAAAAAUGCUAUCGUUGCAUAAAUUUUCAUCUUUUUUAUAAGGAAAAAGUAUUAACUUUGCACCCCGAAAUCG
env-16    GGGGGAAAAGCAAAAAAAAAGCAAAAAAUGCUUGCCAAUUCAGGAAAAAGCAGUACCUUUGCAAGCCGAUUAUUAUCAA
env-17    UAUAAUACUGAUAAAAAAAGUUCUUUUUUUGUUGGCUGUUUGGUAAUAAAAUAGUAAUUUUCCAAGCCGAUUAUUAUCAA
env-18    AAGAAAAGGUGAAAAAGUUUGUGGAUUUUGUUGGACGUUUGAUUGAAAAGUAGUAAUUUUGCAAGCCGAUUAUUAUCAA
env-21    AAGAAAGGGAGAAAAAGUUUGUGGAUUUUGUUGGCCGUUUGAUUGAAAAGUAGUAAUUUUGCAAGCCGAUUAUUAUCAA
env-22    AAGAAAGGGAGAAAAAGUUUGUGGAUUUUGUUGGCCGUUUGAUUGAAAAGUAGUAAUUUUGCAAGCCGAUUAUUAUCAA
env-26    AAGAAAGGGAGAAAAAGUUUGUGGAUUUUGUUGGCCGUUUGAUUGAAAAGUAGUAAUUUUGCAAGCCGAUUAUUAUCAA
env-27    AAGAAAGGGAGAAAAAGUUUGUGGAUUUUGUUGGCCGUUUGAUUGAAAAGUAGUAAUUUUGCAAGCCGAUUAUUAUCAA
env-63    AAGAAAGGGAGAAAAAGUUUGUGGAUUUUGUUGGCCGUUUGAUUGAAAAGUAGUAAUUGUAGAAGCCGAUUAUUAUCAA
env-64    AAGAAAGGGAGAAAAAGUUUGUGGAUUUUGUUGGCCGUUUGAUUGAAAAGUAGUAAUUUUGCAAGCCGAUUAUUAUCAA
env-70    AAUGUGCUCCAAACAAAAAAAAUCGGCAAAAAAACUUGUCAAUUCACGAAAAAGCAGUACCUUUGCAAGCCGAUUAUUAU
env-71    GUGAGAAGAAAAAAUUUUUGCAAAAAUACUUGUGGAUUCACGAAAAAGCAGUACCUUUGCAAGCCGAUUAUUAUCAGAG
env-72    UUUCACAUAAAAAAAAGUUACGCAAAACGCUUGCUGAAUGGCAUAAAAGUCGUACCUUUGCACGCCGAUUAUUAUCAAGG
env-73    ACUAACUUUUUCUCAAAAAAAAAUACCUAAAUCGCUUGUCAAUUCACGAAAAAGCAGUACCUUUGCAAGCCGAUUAUUAU
env-74    ..........AUUAAAAAAAAACGAGAAAUUCCUUUGCCAUGUCAGCGGGAAACCGUACCUUUGCAAGCCGAUUAUUAUC
env-75    AAUUUUUUGUGAAAUAAUCGGUUAGAAAUGCUUGUUAUGUGACUAUAAAUUACUAUCUUUGCAAGCCGAUUAUUAUCAA
env-76    AUUUUUUUAUAAAAAUAAUCGGUGCAUUUUGCUUGUUAUGUCCCUAUAAAUUACUAUCUUUGCAAGCCGAUUAUUAUCA
env-77    CUAUUUUAUGAUUCAUUUUCAUAAAAAAAUGCUUGCCAGUUUCAGAAAAAGUGGUACCUUUGCACGCCGAUUAUUAUCAA
env-78    GCACCUAAGGAAGAAAAAAACUGCCCGCAACGCUUGCAGAUUAGGGAAAAAGUUGUACCUUUGCACGCCGAUUAUUAUCA
env-79    GUAGCGAGCCCUUCAAAAAAAGCACGAAAUGCUUUGUUAUGUAACAGAAAAUGACUACCUUUGCAAGCCGAUUAUUAUC
env-80    GACUGUGUCAUGGGAAAAAUAUCGGCCAAAAACUUUGUGGUUCAGGGAAAAAUGACUACCUUUGCAAGCCGAUUAUUAU
Pbr-3-1   AGUAUUUAAAACGUCUUUUUCUAAAGAAUGUGUAGAAGGGUUAUUUCAAAUAAAAUAUGUACUUUUGCACUCUGAAUUG
env-81    AGUAUUUAAAACGUCUUUUUCUAAAGAAUGUGUAGAAGGGUUAUUUCAAAUAAAAUAUGUACUUUUGCACUCUGAAUUG
env-82    AGUAUAAAUAUUAAUAUGCUAAAAGAAUAUGCUUUCUUGAAUAUCAAUUAAUAGUUGUACCUUUGCAGUCCGAUUAUUA
env-83    UUUGAAAAUAAACAAAAAACACGAAAAAACUUUUGUCAGAAUAUAGGAAAGUCGUAUCUUUGCACGCCGAUUAUUAUCG
env-84    GCAAAACCUCUGAAAAUAUUUAUGAUAAACAUGCGACGGAAUGUAAAUAAAUUCGUACUUUUGCACGCCGAUUAUUAUC
env-86    GCAAAACCUCUGAAAAUAUUUAUGAUAAACAUGCGACGGAAUGUAAAUAAAUUCGUACUUUUGCACGCCGAUUAUUAUC
env-87    GCCUCAAGAAGAAAAAAAGUAUCACCCGGGCUUGCAGAAUAAGGAAAAAGUUGUACCUUUGCAAGCCGAUUAUUAUCAA
Pal-1-1   AAUUUAUAAAAUGACAUACUUGCAAGACAAGUGUUGAUGCUCGUUUUAAAUAAAAUAUGUACCUUUGCACACCGAAUUG
env-88    CCAUGAAAAGAAGAUUUUCAAGUUCACAAGCUUGGCUAUUUCGCAGAAUAUAAGUACAUUUGCAUGCCGAUUAUUAUCA
env-98    CCAUGAAAAGAAGAUUUUCAAGUUCACAAGCUUGGCUAUUUCGCAGAAUAUAAGUACAUUUGCAUGCCGAUUAUUAUCA
env-126   ..............................CUUGGCUAUUUCGCAGAAUAUAAGUACAUUUGCAUGCCGAUUAUUAUCA
env-129   CCAUGAAAAGAAGAUUUUCAAGUUCACAAGCUUGGCUAUUUCGCAGAAUAUAAGUACAUUUGCAUGCCGAUUAUUAUCA
env-132   CCAUGAAAAGAAGAUUUUCAAGUUCACAAGCUUGGCUAUUUCGCAGAAUAUAAGUACAUUUGCAUGCCGAUUAUUAUCA
env-148   CCAUGAAAAGAAGAUUUUCAAGUUCACAAGCUUGGCUAUUUCGCAGAAUAUAAGUACAUUUGCAUGCCGAUUAUUAUCA
env-155   CCAUGAAAAGAAGAUUUUCAAGUUCACAAGCUUGGCUAUUUCGCAGAAUAUAAGUACAUUUGCAUGCCGAUUAUUAUCA
env-157   CCAUGAAAAGAAGAUUUUCAAGUUCACAAGCUUGGCUAUUUCGCAGAAUAUAAGUACAUUUGCAUGCCGAUUAUUAUCA
env-158   UAAAUAAAUAAUCACAUUCAUAGACAAUCACUUAUCCUUAUAAUAAAAAGAAAGUAAUACCUUUGUAGUCCGAUUAUUA
env-159   UAAAUAAAUAAUCACAUUCAUAGACAAUCACUUAUCCUUAUAAUAAAAAGAAAGUAAUACCUUUGUAGUCCGAUUAUUA
env-160   UAAAUAAAUAUACACAAGCAAACACAACCAUAUCUCCUUAUAAUAAAAAGAAAGUAAUACCUUUGUAGUCCGAUUAUUA
env-163   .................................CUCCUUAUAAUAAAAAGAAAGUAAUACCUUUGUAGUCCGAUUAUUA
env-164   NNNNNNNNNNNNNNNNNNNNNNNNNNNNNCACUUAUCCUUAUAAUAAAAAGAAAGUAAUACCUUUGUAGUCCGAUUAUUA
env-165   UAAAUAAAUAAUCACAUUCAUAGACAAUCACUUAUCCUUAUAAUAAAAAGAAAGUAAUACCUUUGUAGUCCGAUUAUUA
env-166   UAAAUAAAUAAUCACAUCCACACACAAUCACUUAUCCUUAUAAUAAAAAGAAAGUAAUACCUUUGUAGUCCGAUUAUUA
env-167   UAAAUAAAUAAUCACACCCACAGACAAUCACUUAUCCUUAUAAUAAAAAGAAAGUAAUACCUUUGUAGUCCGAUUAUUA
env-168   UAAAUAAAUAAUUACAUCCAUAGACAAUCACUUAUCCUUAUAAUAAAAAGAAAGUAAUACCUUUGUAGUCCGAUUAUUA
env-169   .......AUAAUCAUAUCCACACACAAUCACUUAUCCUUAUAAUAAAAAGAAAGUAAUACCUUUGUAGUCCGAUUAUUA
env-170   UAAAUAAAUAACCACAUCCACACACAAUCACUUAUCCUUAUAAUAAAAAGAAAGUAAUACCUUUGUAGUCCGAUUAUUA
env-171   ..........................AUCACUUAUCCUUAUAAUAAAAAGAAAGUAAUACCUUUGUAGUCCGAUUAUUA
env-205   UAAAUAAAUAAUCGCAUCCAUAGAUAAUCACUUAUCCUUAUAAUAAAAAGAAAGUAAUACCUUUGUAGUCCGAUUAUUA
env-206   UAAAUAAAUAAUCACAUCCGCGCACAAUCACUUAUCCUUAUAAUAAAAAGAAAGUAAUACCUUUGUAGUCCGAUUAUUA
env-207   UAAAUAAAUAAUCACAUCUAUAGACAAUCACUUAUCCUUAUAAUAAAAAGAAAGUAAUACCUUUGUAGUCCGAUUAUUA
env-208   UAAAUAAAUAAUCACAGUCAUAGACAAUCACUUAUCCUUAUAAUAAAAAGAAAGUAAUACCUUUGUAGUCCGAUUAUUA
env-209   UAAAUAAAUAAUCACAUCCACGAACAACCACUUAUCCUUAUAAUAAAAAGAAAGUAAUACCUUUGUAGUCCGAUUAUUA
env-210   .................CCAUAGACAAUCACUUAUCCUUAUAAUAAAAAGAAAGUAAUACCUUUGUAGUCCGAUUAUUA
env-211   AAUCGCUCCUUUUACACAUAUAUUAUAUAUAAGGGNNNNNNNNNNNNNNNAAAGUAAUACCUUUGUAGUCCGAUUAUUA
env-212   UAAAUAAAUAAUCACAUCCAUAGACAAUCACUUAUCCUUAUAAUAAAAAGAAAGUAAUACCUUUGUAGUCCGAUUAUUA
env-213   ...........UCGCAUCCACAAACAACCACUUAUCCGUAUAAUAAAAAGAAAGUAAUACCUUUGUAGUCCGAUUAUUA
env-222   ...........................................................CUUUGUAGUCCGAUUAUUA
env-226   UAAAUAAAUAAUCACAUCCAUAGACAACCACUUAUCCUUAUAAUAAAAAGAAAGUAAUACCUUUGUAGUCCGAUUAUUA
env-227   UAAAUAAAUAAUCACAUCCACAGAUAAUCACUUAUCCUUAUAAUAAAAAGAAAGUAAUACCUUUGUAGUCCGAUUAUUA
env-229   UAAAUAAAUAAUCACAUCCACAGAUAAUCACUUAUCCUUAUAAUAAAAAGAAAGUAAUACCUUUGUAGUCCGAUUAUUA
env-230   UAAAUAAAUAAUCGCAUCCAUAGAUAAUCACUUAUCCUUAUAAUAAAAAGAAAGUAAUACCUUUGUAGUCCGAUUAUUA
env-231   UAAAUAAAUAAUCACAUCCAUAGAUAAUCACUUAUCCUUAUAAUAAAAAGAAAGUAAUACCUUUGUAGUCCGAUUAUUA
env-232   UAAAUAAAUAAUCAUGUCCACAAACAACCACUUAUCCUUAUAAUAAAAAGAAAGUAAUACCUUUGUAGUCCGAUUAUUA
env-233   UAAAUAAAUAAUCACAUCCACAGAUAAUCACUUAUCCUUAUAAUAAAAAGAAAGUAAUACCUUUGUAGUCCGAUUAUUA
env-234   .........................AAUCACUUAUCCUUAUAAUAAAAAGAAAGUAAUACCUUUGUAGUCCGAUUAUUA
env-246   UAAAUAAAUAAUCACAUCCACGAACAACCACUUAUCCUUAUAAUAAAAAGAAAGUAAUACCUUUGUAGUCCGAUUAUUA
env-248   UAAAUAAAUAAUUACAUCCACAGAUAAUCACUUAUCCUUAUAAUAAAAAGAAAGUAAUACCUUUGUAGUCCGAUUAUUA
env-249   UAAAUAAAUAAUCGCAUCCAUAGAUAAUCACUUAUCCUUAUAAUAAAAAGAAAGUAAUACCUUUGUAGUCCGAUUAUUA
env-250   ..................CAUAGACAAUCACUUAUCCUUAUAAUAAAAAGAAAGUAAUACCUUUGUAGUCCGAUUAUUA
env-257   AAAAAUGUUAAUAAUAAAUAAAUAAUCACACCCAUAGACAAUCANNNNNNNNNNNNNNNNNNNNNNNNNNNNAUUAUUA
env-258   UAAAUAAAUAAUCACAUUCAUAGACAAUCACUUAUCCUUAUAAUAAAAAGAAAGUAAUACCUUUGUAGUCCGAUUAUUA
env-259   UAAAUAAAUAAUCACAUCCAUAGAUAAUCACUUAUCCUUAUAAUAAAAAGAAAGUAAUACCUUUGUAGUCCGAUUAUUA
env-260   UAAAUAAAUAAUCACAUCCACACACAAUCACUUAUCCUUAUAAUAAAAAGAAAGUAAUACCUUUGUAGUCCGAUUAUUA
env-262   GAUUGUAAAAUAAGGAAAUCAUAAAAAGUCUAUUGCCUCUUUAAGAAAAGAAAUUGCUAUCUUUGUAGUCCGAUUAUUA

          ..............................................................................
          ..............................................................................
          ●●●●●●●●●●●●●●●●●●●●●●●●●●●●●●●●●●●●●●●●●●●●●●●●●R●●●●●●●●●●Y●Y●●●●●●●●●●●●●Y●●
```

```
env-264   AAUUGCAAAAUAAGAAAGUUGUAAAAAGCCUAUUACCUCUUUAAGAAAAGAAAUUGCUAUCUUUGUAGUCCGAUUAUUA
env-265   AACCGUAAAAUAAGGAAAUCAUAAAAAGCCUAUUACCUCUUUAAGAAAAGAAAUUGCUAUCUUUGUAGUCCGAUUAUUA
env-266   AUGUGCCCGCGCGCGGAAAUACAUAAAAUUAUUUGGUAGUUAGGGCGCUAUUUCGUACCUUUGCAAGCCGAUUAUUAUC
env-267   UUGAAAACUUCAGAAAAUUGUUGAAAAACUUUGCCGGUUCGUCAAAAAUUAGUACUUUUGCAAGCCGAUUAUUAUCAAG
Bsp-2-1   UUGAAAACUUCAGAAAAUUGUUGAAAAACUUUGCCAGUUCGUCAAAAAUUAGUACUUUUGCAAGCCGAUUAUUAUCAAG
env-268   AUAAAUAUAAACAAUAUCAUUGUGGAGCCUUGAUAGCCUCUCGGCCUGAAUAAAAUAUGUAACUUUGCAUAUCGAAUUG
env-269   AUAAAUAUAAACAAUAUCAUUGUGGAGUCUUGAUUGCCUCUCGACCUGAAUAAAAUAUGUAACUUUGCACAUCGAAUUG
env-270   AUAAAUAUAAACAAUGGCAUUGCAGAGCCAUGAUAGCCUCUCGGCCUGAAUAAAAUAUGUAACUUUGCAUAUCGAAUUG
env-272   AUAAAUAUAAACAAUGGCAUUGCAGAGCCAUGAUAGCCUCUCGGCCUGAAUAAAAUAUGUAACUUUGCACAUCGAAUUG
env-273   AUAAAUAUAAACAAUGGCAUUGCGGAGCCAUGAUAGCCUCUUGGCCUGAAUAAAAUAUGUAACUUUGCACAUCGAAUUG
env-275   AUAAAUAUAAACAAUAUCAUUGUGGAGCCUUGAUAGCCUCUCGGCCUGAAUAAAAUAUGUAACUUUGCACAUCGAAUUG
env-276   AUAAAUAUAAACAAUGGCAUUGCAGAGCCAUGAUAGCCUCUCGGCCUGAAUAAAAUAUGUAACUUUGCACAUCGAAUUG
env-278   AUAAUCGUAAAUAUUCGGUUUCGCCGCUUUUCAGGCAGGAGUCUGCUUAAAUAAAUACGUAUCUUUGCAAUCCGAAUUG
env-279   AUAAUCGUAAAUAUUCAGUUUCGCCGCUUUUCAGGCAGGAGUCUGCCUAAAUAAAUAUGUAUCUUUGCAAUUCGAAUUG
env-280   AUAAUCGUAAAUAUUCAGUUUCGUCGCUUUUCAGGCAGGAGUCUGCCUAAAUAAAUAUGUAUCUUUGCAAUCCGAAUUG
env-281   AUAAUCGUAAAUAUUCAGUUUCGCCGCUUUUCAGGCAGGAGUCUGCCUAAAUAAAUAUGUAUCUUUGCAAUCCGAAUUG
env-285   UAAUCGUAAAUCUUCAGUUUUGCGACGUUUCCUGCAAGAGGUAAGCUAAAUAAAAUAUGUAACUUUGCAUUCCGAAUUG
env-286   UAAUCGUAAAUCUUCAGUUUUGCGACGUUUCCUGCAAGAGGUAAGCUAAAUAAAAUAUGUAACUUUGCAUUCCGAAUUG
env-287   UAAUCGUAAAUCUUCGGUUUUGUGACGUUUCCUGCAAGAGGUAAGCUAAAUAAAAUAUGUAACUUUGCAUUCCGAAUUG
env-288   UAAUCGUAAAUCUUUAGUUUUGCGACGUUUCGUGCAAGAGGUAAGCUAAAUAAAAUAUGUAACUUUGCAUUCCGAAUUG
env-290   UAAUCGUAAAUCUUCAGUUUUGCGACGUUUCCUGCAAGAGGUAAGCUAAAUAAAAUAUGUAACUUUGCAUUCCGAAUUG
env-291   UAAUCGUAAAUCUUCAGUUUUGCGACGUUUCCUGCAAGAGGUAAGCUAAAUAAAAUAUGUAACUUUGCAUUCCGAAUUG
env-292   ................................UGCAAGAGGUAAGCUAAAUAAAAUAUGUAACUUUGCAUUCCGAAUUG
env-293   UAAUCGUAAAUCUUCAGUUUUGCGACGUUUUGUGCAAGAGGUAAGCUAAAUAAAAUAUGUAACUUUGCAUUCCGAAUUG
env-295   UAAUCGUAAAUCUUUAGUUUUGCGACGUUUCGUGCAAGAGGUAAGCUAAAUAAAAUAUGUAACUUUGCAUUCCGAAUUG
env-297   UAAUCGUAAAUCUUCAGUUUUGCGACGUUUCGUGCAAGAGGUAAGCUAAAUAAAAUAUGUAACUUUGCAUUCCGAAUUG
env-298   UAAUCGUAAAUCUUCAGUUUUGCGACGUUUCCUGCAAGAGGUAAGCUAAAUAAAAUAUGUAACUUUGCAUUCCGAAUUG
env-299   UAAUCGUAAAUCUUCGGUUUUGCGACGUUUCCUGCAAGAGGUAAGCUAAAUAAAAUAUGUAACUUUGCAUUCCGAAUUG
env-301   UAAUCGUAAAUCUUCAGUUUUGUGACGUUUCCUGCAAGAGGUAAGCUAAAUAAAAUAUGUAACUUUGCAUUCCGAAUUG
env-302   UAAUCGUAAAUCUUCAGUUUUACGACGUUUCCUGCAAGAGGUAAGCUAAAUAAAAUAUGUAACUUUGCAUUCCGAAUUG
env-305   UAAUCGUAAAUCUUCAGUUUUGCGACGUUUCCUGCAAGAGGUAAGCUAAAUAAAAUAUGUAACUUUGCAUUCCGAAUUG
env-307   UAAUCGUAAAUCUUCAGUUUUGCGACGUUUCGUGCAAGAGGUAAGCUAAAUAAAAUAUGUAACUUUGCAUUCCGAAUUG
env-308   UAAUCGUAAAUCUUCAGUUUUGCGACGUUUCGUGCAAGAGGUAAGCUAAAUAAAAUAUGUAACUUUGCAUUCCGAAUUG
env-309   UAAUCGUAAAUCUUCAGUUUUGCGACGUUUUAUGCAAGAGGUAAGCUAAAUAAAAUAUGUAACUUUGCAUUCCGAAUUG
env-310   UAAUCGUAAAUCUUCAGUUUUGCGACGUUUUGUGCAAGAGGUAAGCUAAAUAAAAUAUGUAACUUUGCAUUCCGAAUUG
env-311   UAAUUGUAAAUAUAUGGCUUGACAACGUUUAAGACAAGGGCUAACCUAAAUAAAAUAUGUAACUUUGCAUUCCGAAUUG
env-314   UAAUCGUAAAUCUUCGGUUUUGCGACGUUUCCUGCAAGAGGUAAGCUAAAUAAAAUAUGUAACUUUGCAUUCCGAAUUG
env-315   AUAAUCGUAAAUCUUUAGUUUUGCGACGUUUUGUGCAAGAUGUAAGCUAAAUAAAAUAUGUAACUUUGCAUUCCGAAUU
env-316   UAAUCAAUAAACAGAAGCAGAAAAGAACGCUCCACAGACAAGCGUCUCAAAUAAAAGUGGUAACUUUGCAAACCGAUUG
env-317   UAAUCAAUAAACAAAGGCAGAAAAGACCAUUCCACCGGCGAGUGUCUCAAAUAAAAGUGGUAACUUUGCAAACCGAUUG
env-318   UAAUCAAUAAACAAAGGCNGAAAAAACCAUUCCACCGGCGAGUGUCUCAAAUAAAAGUGGUAACUUUGCAAACCGAUUG
env-319   AAUAAUCAUAAAUCAACCUUAUCACAAGACUUGCAGAAAGCUCAUUUACAAUAAAAUAUGUACCUUUGCACCCCGAUUG
Psp-3-1   AUUGAGUUAAUAAUUCAUAAAUAUAGUAUCUUCUCCUCGCGCGAUCUUAAAUAAAAGUUGUACCUUUGCACACCGAUUG
env-320   AAUAUUCGUAAAAAAUGCAGGUCACACUCAUCCGCCCCGUGGCACUGCCAUAUAAAAUAUGUAACUUUGCACCCCGACUU
env-321   AAUAUUCGUAAAAAAUGCAGGUCGCGUCCAUCCGCCCCGUGGCACUGCCAUAUAAAAUGUGUAACUUUGCACCCCGACUU
env-322   AAUAUUCGUAAAAAAUGCAGGUCGCGUCCAUCCGCCCCGUGGCACUGCCAUAUAAAAUAUGUAACUUUGCACCCCGACUU
env-323   AAUAUUCGUAAAAAAUGCAGGUCGCGUCCAUCCGCCCCGUGGCACUGCCAUAUAAAAUAUGUAACUUUGCACCCCGACUU
env-324   AAUAUUCGUAAAAAAUGCAGGUCGCGUCCAUCCGCCCCGUGGCACUGCCAUAUAAAAUAUGUAACUUUGCACCCCGACUU
env-326   AAUAUUCGUAAAAAAUGCAGGUCGCGUCCAUCCACCCCGUGGCACUGCCAUAUAAAAUAUGUAACUUUGCACCCCGACUU
env-327   AAUAUUCGUAAAAAAUGCAGGUCGCGUCCAUCCGCCCCGUGGCACUGCAAUAUAAAAUAUGUAACUUUGCACCCCGACUU
env-328   AAUAUUCGUAAAAAAUGCAGGUCGCAUCCAUUCGCCCCGAGGCACUGCCAUAUAAAAUAUGUAACUUUGCACCCCGACUU
env-329   AAUAUUCGUAAAAAAUGCAGGCCACACCCAUCCGCCCCGUGGCACUGCCAUAUAAAAUAUGUAACUUUGCACCCCGACUU
env-330   AAUAUUCGUAAAAAAUGCAGGUCACAUCCACUCGCCCCGUGGCACUGCCAUAUAAAAUAUGUAACUUUGCACCCCGACUU
env-331   AAUAUUCGUAAAAAAUGCAGGUCGCGUCCAUCCGCCCCGUGGCACUGCCAUAUAAAAUAUGUAACUUUGCACCCCGACUU
env-336   AAUAUUCGUAAAAAAUGCAGGUCGCGUCCAUUCGCCCCGUGGCACUGCCAUAUAAAAUAUGUAACUUUGCACCCCGACUU
env-337   AAUAUUUGUAAAAAAUGCAGGUCGCGUCCAUCCGCCCCGUGGCACUGCCAUAUAAAAUAUGUAACUUUGCACCCCGACUU
env-338   AAUAUUCGUAAAAAAUGCAGGCCACACCCAUCCGCCCCGUGGCGCUGCCAUAUAAAAUAUGUAACUUUGCACCCCGACUU
env-339   AAUAUUCGUAAAAAAUGCAGGCCACGCCCAUCCGCCCCGUGGCACUGCCAUAUAAAAUAUGUAACUUUGCACCCCGACUU
env-341   AAUAUUCGUAAAAAAUGCAGGUCGCGUCCAUUCGCCCCGUGGCACUGCCAUAUAAAAUAUGUAACUUUGCACCCCGACUU
env-345   AAUAUUCGUAAAAAAUGCAGGCCACGCCCACCCGCCCCGUGGCACUGCCAUAUAAAAUAUGUAACUUUGCACCCCGACUU
env-347   AAUAUUCGUAAAAAAUGCAGGCCACACCCAUCCGCCCCGUGGCACUGCCAUAUAAAAUAUGUAACUUUGCACCCCGACUU
env-348   AAUAUUCGUAAAAAAUGCAGGCCACAUCCAUUCGCCCCGUGGCACUGCCAUAUAAAAUAUGUAACUUUGCACCCCGACUU
env-349   AAGUUUUUAAUUCAGCCGUUAUUGAUGGAUAGAGACGCGCGUUUCUAUCCAUAAAAUAUGUAACUUUGCAAACCGAUUG
Pma-3-1   AAGUUUUUAAUUCAGCCGUUAUUGAUGGAUAGAGACGCGCGUUUCUAUCCAUAAAAUAUGUAACUUUGCAAACCGAUUG
env-350   AAGUUUCUAAUUCGGCCAUUAUUGAUGGAUAGAGACGCGCGUUUCUAUCCAUAAAAUAUGUAACUUUGCAAACCGAUUG
env-351   AAGUUUUUAAUUCAGCCGUUAUUGAUGGAUAGAGACGCGCGUUUCUAUCCAUAAAAUAUGUAACUUUGCAAACCGAUUG
Pma-1-1   AAGUUUCUAAUUCAGCCGUUAUUGAUGGAUAGAGACGCGCGUUUCUAUCCAUAAAAUAUGUAACUUUGCAAACCGAUUG
env-353   AAGUUUUUAAUUCGGCCGUUAUUGAUGGAUAGAGACGCGCGUUUCUAUCCAUAAAAUAUGUAACUUUGCAAACCGAUUG
env-354   AAGUUUUUAAUUCGACCGUUAUUGAUGGAUAGAGACGCGCGUUUCUAUCCAUAAAAUAUGUAACUUUGCAAACCGAUUG
env-355   AAGUUUUUAAUUCAGCCGUUAUUGAUGGAUAGAGACGCGCGUUUCUAUCCAUAAAAUAUGUAACUUUGCAAACCGAUUG
env-357   AUAUUCGUAAUCUGAAUGCUUUUGCCUGCUAAUACUUUUCGAUUCUUCCUAUAAAAUAUGUAUCUUUGCAAACCGAUUA
env-359   AUAUUCGUAACCAGGAUGCUUUUGUCUGCUAAUGCUUUUCGAUUCUUCCUAUAAAAUAUGUAUCUUUGCAAACCGAUUA
env-360   AUAUUCGUAAUCAGAAUGCUUUUGUCUGCUAAUACUUUUCGGUUCUUCCUAUAAAAUAUGUAUCUUUGCAAACCGAUUA
env-361   AUAUUCGUAAUCAGAAUGCUUUUGUCUGCUAAUACUUUUCGGUUCUUCCUAUAAAAUAUGUAUCUUUGCAAACCGAUUA
env-362   AUAUUCGUAAUCAGAAUGCUUUUGCCUGCUAAUACUUUUCGGUUCUUCCUAUAAAAUAUGUAUCUUUGCAAACCGAUUA
env-363   AUAUUCGUAACCAGAAUGCUUUUGUCUGCUAAUACUUUUCGGUUCUUCCUAUAAAAUAUGUAUCUUUGCAAACCGAUUA
env-368   AUAUUCGUAAUCAGGAUGCUUUUGUCUGCUAAUACUUUUCGGUUCUUCCUAUAAAAUAUGUAUCUUUGCAAACCGAUUA
env-369   AUAUUCGUAACCGGGAUGCUUUUGUCUGCUAAUGCUUUUCGGUUCUUCCUAUAAAAUAUGUAUCUUUGCAAACCGAUUA
env-370   AUAUUCGUAACCAGGAUGCUUUUGUCUGCUAAUGCUUUUCGGUUCUUCCUAUAAAAUAUGUAUCUUUGCAAACCGAUUA
env-371   AUAUUCGUAAUCAGAAUGCUUUUGUCUGCUAAUACUUUUCGGUUCUUUCUAUAAAAUAUGUAUCUUUGCAAACCGAUUA
env-373   AUAUUCGUAAUCAGGAUGCUUUUGUCUGCUAAUACUUUUCGGUUCUUCCUAUAAAAUAUGUAUCUUUGCAAACCGAUUA
env-375   .........................................GUUCUUCCUAUAAAAUAUGUAUCUUUGCAAACCGAUUA
env-384   AUAUCCGUAAUCAGAAUACUUUUGUCUGCUAAUACUUUUCGGUUCUUCCUAUAAAAUAUGUAUCUUUGCAAACCGAUUA
env-385   AUAUUCGUAAUCAGAAUGCUUUUGCCUGCUAAUACUUUUCGGUUCUUCCUAUAAAAUAUGUAUCUUUGCAAACCGAUUA

          ...............................................................................
          ...............................................................................
          ●●●●●●●●●●●●●●●●●●●●●●●●●●●●●●●●●●●●●●●●●●●●●●●●●R●●●●●●●●●●Y●Y●●●●●●●●●●●●●●Y●●
```

```
env-386  AUAUUCGUAAUCAGGAUGCUUUUGUCUGCUAAUACUUUUCGGUUCUUCCUAUAAAAUAUGUAUCUUUGCAAACCGAUUA
env-387  AUAUUCAUAAUUGGAAUGCUUUUGCCUGCUAAUACUUUUCGGUUCUUCCUAUAAAAUAUGUAUCUUUGCAAACCGAUUA
env-388  AUAUUUGUAAUUGGGAUGCUUUUAUCUGCUCAUACUUUUCGGUUCUUCCUAUAAAAUAUGUAUCUUUGCAAACCGAUUA
env-389  AUAUUCGUAACCAGAAUGCUUUUGUCUGCUAAUGCUUUUCGGUUCUUCCUAUAAAAUAUGUAUCUUUGCAAACCGAUUA
env-390  ...........................................................................AUUA
env-392  AUAUUCGUAACCAGGAUGCUUUUGUCUGCUAAUGCUUUUCGAUUCUUCCUAUAAAAUAUGUAUCUUUGCAAACCGAUUA
env-396  AUAUUCGUAACCAGAAUGCUUUUGUCUGCUAAUGCUUUUCGGUUCUUCCUAUAAAAUAUGUAUCUUUGCAAACCGAUUA
env-397  AUAUUUGUAAUUGGAAUGCUUUUAUCUGCUCAUACUUUUCGGUUCUUCCUAUAAAAUAUGUAUCUUUGCAAACCGAUUA
env-398  AUAUUCGUAACCGGGAUGCUUUUGUCUGCUCAUACUUUUCGGUUCUUCCUAUAAAAUAUGUAUCUUUGCAAACCGAUUA
env-399  UUGUGUAUCUUGGGUUGUAGGGAUUGAUUUCAUGAAAUGAAUAAUAUUANNNNNNNNNNGUAUCUUUGCAAACCGAUUA
env-400  AUAUUCGUAACCAGAAUGCUUUUGUCUGCUAAUGCUUUUCGAUUCUUCCUAUAAAAUAUGUAUCUUUGCAAACCGAUUA
env-401  AUAUUCGUAAUCUGAAUGCUUUUGCCUGCUAAUACUUUUCGAUUCUUCCUAUAAAAUAUGUAUCUUUGCAAACCGAUUA
env-402  AUAUUUGUAAUUGGAAUGCUUUUAUCUGCUCAUACUUUUCGGUUCUUCCUAUAAAAUAUGUAUCUUUGCAAACCGAUUA
env-403  ..................CUUUUGUCUGCUAAUACUUUUCGGUUCUUCCUAUAAAAUAUGUAUCUUUGCAAACCGAUUA
env-406  ...................................CUUUUCGGUUCUUCCUAUAAAAUAUGUAUCUUUGCAAACCGAUUA
env-408  UUAUGUAAUUAGAAUAGAUUUACAGUCUAUUACAUUAUGCUUCUUUCUUUAAAAUAUGUACCUUUGCAAACCGAUUGCU
env-410  UUGUGUAAUUGGGAUAGAUUUACAGUCUAUUACAUUAUGCUUCUUUCUUUAAAAUAUGUACCUUUGCAAACCGAUUGCU
env-411  UUGUGUAAUUAGAAUAGAUUUACAGUCUAUUACAUUAUGCUUCUUUCUUUAAAAUAUGUACCUUUGCAAACCGAUUGCU
env-412  UAUUCGUAACCAGAAUACUUUUGUCUGCCAAUGCUUUUCGAUUCUUCCUAUAAAAUAUGUAUCUUUGCAAACCGAUUAC
Por-6-1  GUUAUGUAAUUAGAAUGGAUUUGCAGUCUAUUACAUUAUGCUUCUUUCUUUAAAAUAUGUACCUUUGCAAACCGAUUGC
Por-5-1  GUUAUGUAAUUAGAAUGGAUUUGCAGUCUAUUACAUUAUGCUUCUUUCUUUAAAAUAUGUACCUUUGCAAACCGAUUGC
env-413  GUUAUGUAAUUAGAAUAGAUUUACAGUCUAUUACAUUAUGCUUCUUUCUUUAAAAUAUGUACCUUUGCAAACCGAUUGC
env-414  GUUAUGUAAUUAGAAUGGAUUUGCAGUCUAUUACAUUAUGCUUCUUUCUUUAAAAUAUGUACCUUUGCAAACCGAUUGC
env-418  GUUAUGUAAUUAGAAUGGAUUUGCAGUCUAUUACAUUAUGCUUCUUUCUUUAAAAUAUGUACCUUUGCAAACCGAUUGC
env-419  GUUGUGUAAUUAGAAUGGAUUUACAGUCUAUUACAUUAUGCUUCUUUCUUUAAAAUAUGUACCUUUGCAAACCGAUUGC
env-420  .......................................GCUUCUUUCUUUAAAAUAUGUACCUUUGCAAACCGAUUGC
env-421  GUUAUGUAAUUAGAAUAGAUUUACAGUCUAUUACAUUAUGCUUCUUUCUUUAAAAUAUGUACCUUUGCAAACCGAUUGC
env-422  GUUGUGUAAUUAGAAUAGAUUUGCAGUCUAUUACAUUAUGCUUCUUUCUUUAAAAUAUGUACCUUUGCAAACCGAUUGC
env-423  GUUAUAUAAUUAGAAUGGAUUUGCAGUCUAUUACAUUAUGCUUCUUUCUUUAAAAUAUGUACCUUUGCAAACCGAUUGC
Psp-8-1  AUAUAUAUAAGAUGAGGUGUUUUAGGGGGCACUGUGAAUACAUCCCUCUCAUAAAAUAUGUAACUUUGCACACCGAUUG
env-425  AUAUAUAUAAGACGAGGCGUUUUAGGGGGCGCUGUGAAUACAUCCCUCUCAUAAAAUAUGUAACUUUGCACACCGAUUG
env-426  AUAUAUAUAAGACGAGGCGUUUUAGGGGGCGCUGUGAAUACAUCCCUCUCAUAAAAUAUGUAACUUUGCAUACCGAUUG
env-429  AUAUAUAUAAGACGAGGCGUUUUAGGGGGCGCUGUGAAUACAUCCCUCUCAUAAAAUAUGUAACUUUGCAUACCGAUUG
env-431  AUAUAUAUAAGAUGAGGUGUUUUAGGGGGCGCUGUGAAUACAUCCCUCUCAUAAAAUAUGUAACUUUGCACACCGAUUG
env-432  AUAUAUAUAAGAUGAGGUGUUUUAGGGGGCGCUGUGAAUACAUCCCUCUCAUAAAAUAUGUAACUUUGCAUACCGAUUG
env-433  AUAUAUGUAAGAUGAGGUGUUUUAGGGGGCACUAUGAAUACAUCCCUCUCAUAAAAUAUGUAACUUUGCAAACCGAUUG
env-434  AUAUAUAUAAGAUGAGGUGUUUUAGGGGGCGCUGUGAAUACAUCCCUCUCAUAAAAUAUGUAACUUUGCAUACCGAUUG
env-435  AUAUAUAUAAGAUGAGGUGUUUUAGGGGGUACUGUGAAUACAUCCCUCUCAUAAAAUAUGUAACUUUGCAUACCGAUUG
env-436  AUAUAUGUAAUAUAUGGUACUUUGGAGGGCGUUCCGAGUGCAUCCCUGUUAUAAAAUAUGUAACUUUGCACACCGAUUG
env-437  ....AUGUAAUAUAUGGUACUUUGGAGGGCGUUCCGAGUGCAUCCCUGUUAUAAAAUAUGUAACUUUGCACACCGAUUG
env-438  AUAUAUAUAAGAUGAGGUGUUUUAGGGGGCACUGUGAAUACAUCCCUCUCAUAAAAUAUGUAACUUUGCACACCGAUUG
env-439  AUAUAUAUAAGAUGAGGCGUUUUAGGGGGUACUGUGAAUACAUCCCUCUCAUAAAAUAUGUAACUUUGCACACCGAUUG
env-440  AUAUAUGUAAUAUGUAGUGCUUUGGAGGGCGUUCCGGGUGCAUCCCUGUUAUAAAAUAUGUAUCUUUGCACGCCGAUUG
env-441  AUAUAUGUAAUAUAUGGUGCUUUGAAGGGUGUUCCGGGUGCAUCCCUGUUAUAAAAUAUGUAUCUUUGCACGCCGAUUG
env-442  AUAUAUGUAAUAUAUGGUGCUUUGGAGGGCGUUCCGAGUGCAUCCCUGUUAUAAAAUAUGUAUCUUUGCACGCCGAUUG
env-443  AUAUAUGUAAUAUAUGGCGCUUUGGAGGGUGUUCCGAGUGCAUCCCUGUUAUAAAAUAUGUAUCUUUGCACGCCGAUUG
Pou-1-1  AUAUAUGUAAUAUAUGGUGCUUUGGAGGGCGUCCCGAGUGCAUCCCUGUUAUAAAAUAUGUAUCUUUGCACGCCGAUUG
env-444  AUAUAUGUAAUAUAUAGUGCUUUGGAGAGCGUUCCGGGUGCAUCCCUGUUAUAAAAUAUGUAUCUUUGCACGCCGAUUG
env-445  AUAUAUGUAAUAUAUAGUGCUUUGGAGGGUGUUCCGAGUGCAUCCCUGUUAUAAAAUAUGUAUCUUUGCACGCCGAUUG
env-446  AUAUAUGUAAUAUAUGGUGCUUUGGAGGGUGCUCAGGGUGCAUCCCUGUUAUAAAAUAUGUAUCUUUGCACGCCGAUUG
env-447  AUAUAUGUAAUAUAUGGUGCUUUGGAGGGCGUUCCGAGUGCAUCCCUGUUAUAAAAUAUGUAUCUUUGCACGCCGAUUG
Pou-2-1  AUAUAUGUAAUAUAUGGUACUUUGGAGGGCGUUCCGAGUGCAUCUCUGUUAUAAAAUAUGUAUCUUUGCACGCCGAUUG
env-448  AAUCAUAAUUCAUACAUUUUUUCUGUAUAAAUACCUUGUAAUUAAAAUAAAAUACAUACUUUUGCAGACCGAUUAUUAU
env-449  UGUUUUGUAAAAUAAAAGCCGCGUUCUCCAUGCCGUGGCCCCAUCUCGUCAUAAAAUAUGUAACUUUGCAGUCCGAUUG
Pba-2-1  AUGUUUUGUAAAAUAAAGCCGCGUUCUCCAUGCCGUGGCCCCAUCUCCUCAUAAAAUAUGUAACUUUGCAGUCCGAUUG
Pba-1-1  UGUUUUGUAAAAUAAAAGCCGCGUUCUCCAUGCCGUGGCCCCAUCUCGUCAUAAAAUAUGUAACUUUGCAGUCCGAUUG
env-450  UGUUUUGUAAUUUGUGUAGUGUGAAAUAUGUCCCGUGAAUCAAACUCGUAAUAAAAUAUGUAACUUUGCAGUCCGAUUG
Pde-2-1  UCAUAAAAGGAAAGUCGUGGCAUGCCCGCGGUGCUGCUACUCUAAUUAAAAUGUGUACUUUUGCAGACCGAUUAUUAAA
Pbe-1-1  CUUGUUAAAACAAAGCUCUGUGGGAGUGUUUUGUUAGUAUGCACGAAAUAAAAUAGGUAAUUUUGCAGACCGAUUGUUU
env-451  UUUCAUAGUCGGAAAUUGAUAGGUGAGGCUUUUACUAUAUAAAAUGUGUACCUUUGCAGUCCGAUUAUUAAAAUUGUUG
env-452  ACAUAAAACAUUGCUUCCGUAGGCUCCUUCGGAGGCUCAUCACCAUAUUAAAUACCUACCUUUGCAGUCCGAUUAUUAU
env-453  AGGUAGAAAACGCAAAUUUUUAUCGAAAAAAGUUGUUGAUUAAUAAAAAAUGCAUACUUUUGCAGUCCGAUUAUUAUUU
env-454  AUUCUUAACGCUUGAUCUCUCCUCUUCUCCUCGGGUUCGUUUUCCCAUAGAAAUACGUACCUUUGCAGUCCGAUUAUUA
env-455  AUUCUUAACGCUUGAUCUCUCCUCUUCUCCUCGGGUUCGUUUUCCCAUAGAAAUACGUACCUUUGCAGUCCGAUUAUUA
env-457  UUCGUAAAAUCUACUUUUCUUUCGCAGUAGGUUGGCUUGUUGCUCUAUAUAAAUAUGUAACUUUGCAGUCCGAUUAUUA
env-458  UUCGUAAAAUCUACUUUUCUUUCACAGUGGGUUGGCUUGUUGCUCUAUAUAAAUAUGUAACUUUGCAGUCCGAUUAUUA
env-462  UUCGUAAAAUCUACUUUUCUUUCGCAGUGGGUUGGCUUGUUGCUCUAUAUAAAUAUGUAACUUUGCAGUCCGAUUAUUA
env-463  UUCGUAAAAUGCACUUUUCUUUCGCAGUGGGUUGGUUUGUUGCUCUAUAUAAAUAUGUAACUUUGCAGUCCGAUUAUUA
env-464  UUCGUAAAAUACACUUUUCUUUCGCAGUGGGUUGGUUUGUUGCUCUAUAUAAAUAUGUAACUUUGCAGUCCGAUUAUUA
env-466  UAUAAAAAUCGCAUUUUAGUAAGCGAAAACGAGCAUUUAAUCACCGGAAAAUGCGUACUUUUGCAGACCGAUUUUAUCC
env-467  UACAUAAAAACAACAUAUUUAACAUCCCGUCAUAUAUUAAAUAAUGAGAAAAAGCAGUACCUUUGCAAUCCGAUUAUUU
env-468  AUAUUAAAUUGUGUGGUUUCUGUGACCAUCAGGAUGCGAUUAUGAAUAAAAAAUAUGUAACUUUGCAGUCCGAUUAUUA
env-469  AUGUUAAAUGUGAUGUCUUGGAAUCUUGGAGAGUGCGUUAAAAGGAUAAAAAUGAGUACCUUUGCAGUCCGAUUAUUAC
env-470  ............................................................UUUGCACACCGAUUAUUUU
Ppa-3-1  UAACUGUAAAUUUAAGGAUUGCGUCAUGCUCUUGCAUCAUGAAGUAAGAAUAAAAUAUGUACCUUUGCACACCGAUUAC
env-471  ...........................................GUAAGAAUAAAAUAUGUACCUUUGCACACCGAUUAC
env-473  UAACUAUAAAUUCAAGGAUUGAGCCAUGCUCUUGCAUCAUGAAGUAAGAAUAAAAUAUGUACCUUUGCACACCGAUUAC
env-474  UAACUGUAAAUUUAAGGAUUGCGUCAUGCUCUUGCAUCAUGAAGUAAGAAUAAAAUAUGUACCUUUGCACACCGAUUAC
env-476  UAACUGUAAAUUUAAGGAUUGCAUCAUGCUCUUGCAUCAUGAAGUAAGAAUAAAAUAUGUACCUUUGCACACCGAUUAC
env-477  ........................CAUGCUCUUGCAUCAUGAAGUAAGAAUAAAAUAUGUACCUUUGCACACCGAUUAC
env-483  AAACUAUAAAAAUCGCUUAUAAAAGCGAAUCGGCGCUUGCGGUAUGAAUAAAAAGUUGUACCUUUGCACCCCGAAACGG
env-485  AAACUAUAAAAAUCGCUUAUAAAAGCGAAUCGGUGUUUGUGGUAUGAAUAAAAAGUUGUACCUUUGCACCCCGAAACGG
env-486  CAUAAUUAUUCGUUUUCAAGGAUUGGUGAUGGCGGGACUCUGAAAGAAAAGCAGUACCUUUGCAGUCCGAUUAUUACCA
env-487  ..........................................................................GAUUU

         ...............................................................................
         ...............................................................................
         ●●●●●●●●●●●●●●●●●●●●●●●●●●●●●●●●●●●●●●●●●●●●●●●●●R●●●●●●●●●●Y●Y●●●●●●●●●●●●●Y●●
```

```
env-488   AAUAAGUAUAAAUCUAUGCAAAUAAUCCGCUUUUACGCUCCACACCUUAUUUAAAAUGUGUACCUUUGCACCUCGAUUU
env-489   CCUUAAUUUGAAACCAUCUAUUCCUCUUUACAUUUCCUCAUAAUAAAUAAAGUGAGUACCUUUGCAGUCCGAUUAUUAU
env-490   UUGUAAAAAACAGGAGGAUUGGUGCUAUAGUUGUGUUUUCACAUCAUUUAAAUUAGUACCUUUGUAGUCCGAUUAUUAU
env-491   UUAUAAAAAAUGGAAGGAUUGGUGCUGUAGUUGUGUUUUCACAUCAUUUAAAUUAGUACCUUUGUAGUCCGAUUAUUAU
env-492   UUAUAAAAAAUAGGAGGAUAGGUGCUGUAGUUGUGUUUUCACAUCAUUUAAAUUAGUACCUUUGUAGUCCGAUUAUUAU
env-493   UCAUAAAAAAUGGAAGGGUUGGUGCUGUAGUUGUGUUUUCACAUCAUUUAAAUUAGUACCUUUGUAGUCCGAUUAUUAU
Pam-1-1   UUAUAAAAAAUGGAAGGAUUGGUGCUGUAGUUGUGUUUUCACAUCAUUUAAAUUAGUACCUUUGUAGUCCGAUUAUUAU
Pam-3-1   UUAUAAAAAAUGGAGGGAUUGGUGCUGUAGUUGUGUUUUCACAUCAUUUAAAUUAGUACCUUUGUAGUCCGAUUAUUAU
Pbi-1-1   AUUUAUAAAAAUACUAUCUCUCAAAAUGCUAUCUCUCUUUUCACAUCAUUAAUUAGUACCUUUGCAGUCCGAUUAUUAU
Pve-1-1   UCGUAAAUAAUACAGAAUGAUUAUAGGAAAAGUAUAUAUAUAAGGUAGGAAAUAAUUACCUUUGCAGUCCGAUUAUUAU
Pve-3-1   UCGUAAAUAACAUAGAAUGAUUAUAGGAAAAGUAUAUAUAUAAGGUAGAAAACAAUUACCUUUGCAGUCCGAUUAUUAU
env-494   UCGUAAAUAACGCACAAUGAUUAUAGGAAAAGUAUAUAUAUAAGGUAGAAAACAAUUACCUUUGCAGUCCGAUUAUUAU
env-495   UCAUAAACAAUACAUAUCUAUGCUAUUCAAACCUUUUAUAUAAGGUUGAAAAUAAGUACCUUUGCAGUCCGAUUAUUAA
env-497   UCAUAAACAACACAUAUCUAUCCUAUUCAAACCUUUUAUAUAAGGUUGAAAAUAAGUACCUUUGCAGUCCGAUUAUUAA
Psc-1-1   AUAUAAACAUUACAUACUUACACGAGGUAACAUUAGUGUAUAAGGUUGAAAAUAAGUACCUUUGCAGUCCGAUUAUUAA
env-498   AUAUAAACAUUACAUACUUACACGAGGUAACAUUAGUGUAUAAGGUUGAAAAUAAGUACCUUUGCAGUCCGAUUAUUAA
Psp-6-1   ACCUAAAAAUCAUACACUUAAACAAGCUAACAUUAUUAUAUAAGGUUGAAAAUAAGUACCUUUGCAGUCCGAUUAUUAA
Pme-3-1   AAUCAUAAACUACAUAUAAUCUUACUAUCCAAAAAAUUAUAUAAGGUUGAAAAAAAGUACCUUUGCAGUCCGAUUAUUA
env-501   UGUAUAAAUUAUUUAUAACGAUGAUUGCCAUUCUUAUAUAUAAGGUUGGAAAUGACUACCUUUGCAGUCCGAUUAUUAU
env-502   UAUAUAAAUUAUUUAUAACGAUGAUUGCCAUUCUUAUAUAUAAGGUUGGAAAUGACUACCUUUGCAGUCCGAUUAUUAU
env-503   UAUAUAAAUUAUUUACAACGGUGAUUGCCAUUCUUAUAUAUAAGGUUGGAAAUGACUACCUUUGCAGUCCGAUUAUUAU
env-504   UAUAUAAAUUAUUUAUAACGAUGAUUGCCAUUCUUAUAUAUAAGGUUGGAAAUGACUACCUUUGCAGUCCGAUUAUUAU
env-507   UAUAUAAAUUAUUUAUAACGAUGAUUGCCAUUCUUAUAUAUAAGGUUGGAAAUGACUACCUUUGCAGUCCGAUUAUUAU
Phi-2-1   UAUAUAAAUUAUUUAUAACGAUGAUUGCCAUUCUUAUAUAUAAGGUUGGAAAUGACUAUCUUUGCAGUCCGAUUAUUAU
env-508   UAUAUAAAUUAUUUAUAACGAUGAUUGCCAUUCUUAUAUAUAAGGUUGGAAAUGACUACCUUUGCAGUCCGAUUAUUAU
env-509   UAUAUAAAUUAUUUAUAACGAUGAUUGCCAUUCUUAUAUAUAAGGUUGGAAAUGACUACCUUUGCAGUCCGAUUAUUAU
Phi-2-2   UAUAUAAAUUAUUUAUAACGAUGAUUGCCAUUCUUAUAUAUAAGGUUGGAAAUGACUACCUUUGCAGUCCGAUUAUUAU
env-510   UCAUAAACUACAUAUAAUCUUACUAUCCAAAACAAUUAUAUAAGGUUGAAAAUAAGUACCUUUGCAGUCCGAUUAUUAA
env-511   UCAUAAACUACAUAUAAUCUUACUAUCCAAAACAAUUAUAUAAGGUUGAAAAUAAGUACCUUUGCAGUCCGAUUAUUAA
env-512   UCAUAAACUACAUAUAAUCUUACUAUCCAAAACAAUUAUAUAAGGUUGAAAAUAAGUACCUUUGCAGUCCGAUUAUUAA
env-514   CAUAAACUACAUACAAUCCUACCAUUCAAAACAAUUAUAUAAGGUUGAAAAUAAGUACCUUUGCAGUCCGAUUAUUAAA
env-516   CAUAAACUACAUACAAUCCUACCAUUCAAAACAAUUAUAUAAGGUUGAAAAUAAGUACCUUUGCAGUCCGAUUAUUAAA
env-517   CCUAAACUACAUACAAUCCUACCAUUCAAAACAAUUAUAUAAGGUUGAAAAUAAGUACCUUUGCAGUCCGAUUAUUAAA
env-519   CAUAAACAACACAUAUCUAUCCUAUUCAAACCUUUUAUAUAAGGUUGAAAAUAAGUACCUUUGCAGUCCGAUUAUUAAA
Psp-5-1   UCAUAAAAAAUACGUAGCUAUGCUAUUCAAACCUUUUAUAUAAGGUUGAAAAUAAGUACCUUUGCAGUCCGAUUAUUAA
env-520   UCAUAAACAAUACGUAGCUAUGCUAUUCAAACCUUUUAUAUAAGGUUGAAAAUAAGUAUCUUUGCAGCCCGAUUAUUAA
env-521   UCAUAAACAAUACAUAUCUAUGCUAUUCAAACCUUUUAUAUAAGGUUGAAAAUAAGUACCUUUGCAGUCCGAUUAUUAA
env-522   UCAUAAACAACACAUAUCUAUGCUAUUCAAACCUUUUAUAUAAGGUUGAAAAUAAGUACCUUUGCAGUCCGAUUAUUAA
env-523   UCAUAAACUACAUAUAAUCUUACUAUCCAAAACAAUUAUAUAAGGUUGAAAAUAAGUACCUUUGCAGUCCGAUUAUUAA
env-525   UCAUAAACUACAUAUAAUCUUACUAUCCAAAACAAUUAUAUAAGGUUGAAAAUAAGUACCUUUGCAGUCCGAUUAUUAA
env-526   NNNNNNNNNCACAUAAUCCUACUAUCCAAAACAAUUAUAUAAGGUUGAAAAUAAGUACCUUUGCAGUCCGAUUAUUAA
env-527   .............................................................................AA
env-531   UCAUAAACUACACAUAAUCCUACUAUCCAAAACAAUUAUAUAAGGUUGAAAAUAAGUACCUUUGCAGUCCGAUUAUUAA
env-532   UCAUAAACUACAUAUAAUCCUACUAUCCAAAACAAUUAUAUAAGGUUGAAAAUAAGUACCUUUGCAGUCCGAUUAUUAA
env-534   UCAUAAACUACAUAUAAUCUUACUAUCCAAAACAAUUAUAUAAGGUUGAAAAUAAGUACCUUUGCAGUCCGAUUAUUAA
env-535   UCAUAAACUACAUAUAAUCCUACUAUCCAAAACAAUUAUAUAAAGUUGAAAAUAAGUACCUUUGCAGUCCGAUUAUUAA
env-537   UCAUAAACUACAUAUAAUCCUACUAUCCAAAACAAUUAUAUAAGGUUGAAAAUAAGUACCUUUGCAGUCCGAUUAUUAA
env-538   UCAUAAACUACAUAUAAUCUUACUAUCCAAAACAAUUAUAUAAGGUUGAAAAUAAGUACCUUUGCAGUCCGAUUAUUAA
env-552   UCAUAAACUACAUAUAAUCUUACUAUCCAAAACAAUUAUAUAAGGUUGAAAAUAAGUACCUUUGCAGUCCGAUUAUUAA
env-553   UCAUAAACAACACAUAUCUAUCCUAUUCAAACCUUUUAUAUAAGGUUGAAAAUAAGUACCUUUGCAGUCCGAUUAUUAA
env-554   CAUAAACUACAUAUAAUCCUACUAUCCAAAACAAUUAUAUAACGUUGAAAAUAAGUACCUUUGCAGUCCGAUUAUUAAA
env-555   UCAUAAACUACAUAUAAUCUUACUAUCCAAAACAAUUAUAUAAGGUUGAAAAUAAGUACCUUUGCAGUCCGAUUAUUAA
env-557   UCAUAAACUACAUAUAAUCCUACUAUCCAAAACAAUUAUAUAAGGUUAAAAAUAAGUACCUUUGCAGUCCGAUUAUUAA
env-558   UCAUAAACUACAUAUAAUCUUACUAUCCAAAACAAUUAUAUAAGGUUGAAAAUAAGUACCUUUGCAGUCCGAUUAUUAA
env-559   UCAUAAACUACAUAUAAUCUUACUAUCCAAAACAAUUAUAUAAGGUUGAAAAUAAGUACCUUUGCAGUCCGAUUAUUAA
env-560   UCAUAAACUAUAUAUAAUCCUACUACCCAAAACAAUUAUAUAAGGUUGAAAAUAAGUACCUUUGCAGUCCGAUUAUUAA
env-561   UCAUAAACUACAUAUAAUCCUACUAUCCAAAACAAUUAUAUAAGGUUGAAAAUAAGUACCUUUGCAGUCCGAUUAUUAA
Pfu-1-1   AUCAUAAACAACACGCCAUCAAACAUUCAAAUAUAAUUAUAAAUGUAGAAAAUAAGUACCUUUGCAGUCCGAUUAUUAU
Psp-24-1  AAUCGUAAAUAACACAGAAUGGUUAUAGGAAAUCAUAUAUAUAAGGUAGAAAACAAUUACCUUUGCAGUCCGAUUAUUA
env-562   AUCGUAAAUAACACAGAAUGAUUAUAGGAAAAGUAUAUAUAUAAGGUAGAAAACAAUUACCUUUGCAGUCCGAUUAUUA
env-563   AAUCGUAAAUAACACAGAAUGAUUAUAGGAAAUCAUAUAUAUAAGGUAGAAAACAAUUACCUUUGCAGUCCGAUUAUUA
env-564   AAUCGUAAAUAACACAGAAUGAUUAUAGGAAAUCAUAUAUAUAAGGUAGAAAACAAUUACCUUUGCAGUCCGAUUAUUA
Pde-3-1   CGUAAAAAGCAAACGUCGGUUCCUUGCUCCUUAUAGAAUGAGGUUGGAAAUGAGUACCUUUGCAGUCCGAUUAUUAUGG
env-565   CGUAAAAAGCAAACGUCGGUCCCUUGCUCCUUAUAGAAUGAGGUUGGAAAUGAGUACCUUUGCAGUCCGAUUAUUAUGG
Pde-4-1   CGUAAAAAGCAAGCGUCGGUCCCUUGCUCCUUAUAGAAUGAGGUUGGAAAUGAGUACCUUUGCAGUCCGAUUAUUAUGG
Pde-5-1   CGUAAAAAGCAAACGUCGGUCCCUUGCUCCUUAUAGAAUGAGGUUGGAAAUGAGUACCUUUGCAGUCCGAUUAUUAUGG
env-566   CGUAAAAAGCAAACGCCGGUCCCUUGCUCCUUAUAGAAUGAGGUUGGAAAUGAGUACCUUUGCAGUCCGAUUAUUAUGG
env-567   CGUAAAAAGCAAACGCCGGUCCCUUGCUCCUUAUAGAAUGAGGUUGGAAAUGAGUACCUUUGCAGUCCGAUUAUUAUGG
Pde-6-1   CGUAAAAAGCAAACGCCGGUCCCUUGCUCCUUAUAGAAUGAGGUUGGAAAUGAGUACCUUUGCAGUCCGAUUAUUAUGG
env-568   AUCGUAAAUAAUGGGUGCGGAAAACAUCACCCUUAUAAUAUAAGGUUGGAAAUAAGUACCUUUGCAGUCCGAUUAUUAU
env-569   AUCGUAAAUAAUGGGUGCGGAAAACAUCACCCUUAUAAUAUAAGGUUGGAAAUAAGUACCUUUGCAGUCCGAUUAUUAU
Pmu-1-1   AUCGUAAAUAAUGGGUGCGGAAAACAUCACCCUUAUAAUAUAAGGUUGGAAAUAAGUACCUUUGCAGUCCGAUUAUUAU
env-570   UCAUAAACUUAUAAGUUUGGAGAUGGUAUAGAGCAAUCUCUUAUACAUAAAAUGUAUAUCUUUGCAGUCCGAUUAUUUC
env-571   AAUUUAUAAAUCCGUUCGUUUUGUCUCCUGCUGCCCUUGUCUGAUUUAUAAAUAAUGAGUAAUUUUGCAAUCCGAUUGA
env-572   AAUAUAUAAAACGAUUUGUCUUGCAUCCCGUCGUUCUUGUCGGAUUUAUAAAUAAUAGGUAAUUUUGCAAUCCGAUUGA
env-573   AAUAUAUAAAACGAUUUGUCUUGCAUCCCGUCGUUCUUGUCGGAUUUAUAAAUAAUAGGUAAUUUUGCAAUCCGAUUGA
env-574   GUUUAAAACUGUAACACUUGCUUUGUCGGAUACUUCUCUAUGCCGAAUAAAAUGAGUAAUUUUGCAGACCGAUUAUUUC
env-575   ....UAAAUAUAGAUUUUUUGGUUGUGUUUGAUAGUUCAGUCAUCUAUAAAAUAAGUACUUUUGCAUACCGAUUAUUUU
env-576   UUUUUAAAUAUUGGCUUUUUGGUUGUUUUUGAUCGUUUAGUCAUCUGUAAAAUAAGUACUUUUGCAUACCGAUUAUUUU
env-577   ................CCGAGCAGACGCAACAUGCAAGUAUUGUAAGAAAUAUGUGUACCUUUGCAGUCCGAUUAUUUC
env-578   UACGUAAAAGACUAAUUAGCUUUUAAGGUAGUAUUUCCAAAAGUUUCCUAAAUGUGUACUUUUGCGCACCGAUUAUUAU
env-579   UACGUAAAAGACUAAUUAGCUUUUAAGGUAGUAUUUCCAAAAGUUUCCUAAAUGUGUACUUUUGCGCACCGAUUAUUAU
env-580   UACGUAAAAGACUAAUUAGCUUUUAAGGUAGUAUUUCCAAAAGUUUCCUAAAUGUGUACUUUUGCGCACCGAUUAUUAU
env-582   UACGUAAAAGACUAAUUAGCUUUUAAGGUAGUAUUUCCAAAAGUUUCCUAAAUGUGUACUUUUGCGCACCGAUUAUUAU

          ...............................................................................
          ...............................................................................
          ●●●●●●●●●●●●●●●●●●●●●●●●●●●●●●●●●●●●●●●●●●●●●●●●●R●●●●●●●●●●Y●Y●●●●●●●●●●●●●Y●●
```

```
Pin-1-1   AUAAUUAAUAUCAAUGCUUUCACGCUGUUAUGCGUUUCGUACCUAAUAAAAAAUGAGUAAUUUUGCAGACCGAUUAUUU
env-583   AUGAUUAAGAUAUAAGCAUCUACGGAUUUAUCCACUUUGUAAAUAAUAAAAAAUGAGUAAUUUUGCGAACCGAUUAUUU
env-589   AUGAUUAAGAUAUAAGCAUCUACGGAUUUAUCCACUUUGUAAAUAAUAAAAAAUGAGUAAUUUUGCGAACCGAUUAUUU
env-592   AUAAUUAAGAUAUAAGCGUCUACGGAUUUAUCCACUUUGUAAAUAAUAAAAAAUGAGUAAUUUUGCGAACCGAUUAUUU
env-593   AUAAUUAAGAUAUAAGCGUCUACGGAUUUAUCCACUUUGUAAAUAAUAAAAAAUGAGUAAUUUUGCGAACCGAUUAUUU
env-594   AUAAUUAAGAUAUAAGCGUCUGUGGAUUUAUCCACUUUGUAAAUAAUAAAAAAUGAGUAAUUUUGCGCACCGAUUAUUU
env-595   AUAAUUAAGAUAUAAGCGUCUACGGAUUUAUCCACUUUGUAAAUAAUAAAAAAUGAGUAAUUUUGCGAACCGAUUAUUU
env-596   AUGAUUAAGAUAUAAGCAUCUACGGAUUUAUCCACUUUGUAAAUAAUAAAAAAUGAGUAAUUUUGCGAACCGAUUAUUU
env-597   AUAAUUAAGAUAUAAGCAUCUACGGAUUUAUCCACUUUGUAAAUAAUAAAAAAUGAGUAAUUUUGCGAACCGAUUAUUU
env-598   AUAAUUAAGAUAUAAGCAUCUACGGAUUUAUCCACUUUGUAAAUAAUAAAAAAUGAGUAAUUUUGCGAACCGAUUAUUU
env-599   AUAAUUAAGAUAUAAGCGUCUACGGAUUUAUCCACUUUGUAAAUAAUAAAAAAUGAGUAAUUUUGCGAACCGAUUAUUU
env-600   AUAAUUAAGAUAUAAGCAUCUGCGGAUUUAUCCACUUUGUAAAUAAUAAAAAAUGAGUAAUUUUGCGAACCGAUUAUUU
env-613   AUAAUUAAGAUAUAAGCAUCUACGGAUUUAUCCACUUUGUAAAUAAUAAAAAAUGAGUAAUUUUGCGAACCGAUUAUUU
env-648   AUAAUUAAGAUAUAAGCGUCUGUGGAUUUAUCCACUUUGUAAAUAAUAAAAAAUGAGUAAUUUUGCGCACCGAUUAUUU
env-651   AUGAUUAAGAUAUAAGCAUCUGCGGAUUUAUCCACUUUGUAAAUAAUAAAAAAUGAGUAAUUUUGCGAACCGAUUAUUU
env-652   AUAAUUAAGAUAUAAGCAUCUGCGGAUUUAUCCACUUUGUAAAUAAUAAAAAAUGAGUAAUUUUGCGAACCGAUUAUUU
env-653   AUAAUUAAGAUAUAAGCAUCUACGGAUUUAUCCACUUUGUAAAUAAUAAAAAAUGAGUAAUUUUGCGAACCGAUUAUUU
env-657   AUAAUUAAGAUAUAAGCGUCUACGGAUUUAUCCACUUUGUAAAUAAUAAAAAAUGAGUAAUUUUGCGAACCGAUUAUUU
env-676   AUAAUAAAUGAACGGAAUUAAUCCCUGACGAUAGUGAUUCUGGUAUAUAAAAUGAGUAAUUUUGCAGACCGAUUAUUUU
Pco-3-1   AUAAUAAAUGAACGGAAUUAAUCCCUGACGAUAGUGAUUCUGGUAUAUAAAAUGAGUAAUUUUGCAGACCGAUUAUUUU
Pni-1-1   AUAAUUAAUAAGCAGACUUUCGCAGAGUUAUACACUUUGUAACCAAUAAAAAAUGAGUAAUUUUGCAGACCGAUUAUUC
env-677   AUGAUUAAUAAGCAAACUUUCGCAGAGUUAUACACUUUGUAACCAAUAAAAAAUGAGUAAUUUUGCAGACCGAUUAUUC
env-679   AUGAUUAAUAAGUAAACUUUCGCAGAGUUAUACACUUUGUAACCAAUAAAAAAUGAGUAAUUUUGCAGACCGAUUAUUC
env-680   AUGAUUAAUAAGUAAACUUUCGCAGAGUUAUACACUUUGUAACCAAUAAAAAAUGAGUAAUUUUGCAGACCGAUUAUUC
env-681   AUAAUUAAUAAGCAAACUUUCGCAGAGUUAUACACUUUGUAACCAAUAAAAAAUGAGUAAUUUUGCAGACCGAUUAUUC
env-682   AUGAUUAAUAAGUAGAUUUUCGCAGAGUUAUACACUUUGUAACCAAUAAAAAAUGAGUAAUUUUGCAGACCGAUUAUUC
env-683   AUGAUUAAUAAGUAGACUUUCGCAGAGUUAUACACUUUGUAACCAAUAAAAAAUGAGUAAUUUUGCAGACCGAUUAUUC
env-684   AUGAUUAAUAAGCAAACUUUCGCAGAGUUAUACACUUUGUAACCAAUAAAAAAUGAGUAAUUUUGCAGACCGAUUAUUC
env-686   AUAAUUAAUAAGCAGACUUUCGCAGAGUUAUACACUUUGUAACCAAUAAAAAAUGAGUAAUUUUGCAGACCGAUUAUUC
env-696   AUAAUUAAUAAGCAAACUUUCGCAGAGUUAUACACUUUGUAACCAAUAAAAAAUGAGUAAUUUUGCAGACCGAUUAUUC
env-698   AUGAUUAAUAAGCAAACUUUCGCAGAGUUAUACACUUUGUAACCAAUAAAAAAUGAGUAAUUUUGCAGACCGAUUAUUC
env-699   AUGAUUAAUAAGCAAACUUUCGCAGAGUUAUACACUUUGUAACCAAUAAAAAAUGAGUAAUUUUGCAGACCGAUUAUUC
Pni-3-1   AUGAUUAAUAAGCAGACUUUCGCAGAGUUAUACACUUUGUAACCAAUAAAAAAUGAGUAAUUUUGCAGACCGAUUAUUC
env-700   AUGAUUAAUAAGUAAACUUUCGCAGAGUUAUACACUUUGUAACCAAUAAAAAAUGAGUAAUUUUGCAGACCGAUUAUUC
env-709   AUAUUUAAUACAAAUACUUUCACGCGGCUACACAUUUCGUAGCUGAUAAAAAAUGAGUAAUUUUGCAGACCGAUUAUUU
env-710   UAUUUAAUACAAAUACUUUCAUGCGGCUACACAUUUCGUAGCUGAUAAAAAAAUGAGUAAUUUUGCAGACCGAUUAUUU
env-711   AUAUUUAAUACAAAUACUUUCAUGCGGCUACACAUUUCGUAGCUGAUAAAAAAUGAGUAAUUUUGCAGACCGAUUAUUU
env-724   ...............................................AAAAAAUGAGUAAUUUUGCAGACCGAUUAUUU
Pau-1-1   AUAUUUAAUACAAAUACUUUCACGCGGCUACACAUUUCGUAGCUGAUAAAAAAUGAGUAAUUUUGCAGACCGAUUAUUU
Psp-9-1   UAUCUAAAAACGGCAAAUUCGGAUAUAAGGGAGAAUGGCAACAUAAAUAAAAAUGAGUAAUUUUGCAGACCGAUUAUUU
env-725   AUAAUUAAUACCAACGCUUUCACGCUGUUAUGCGUUUCGUACCUAAUAAAAAAUGAGUAAUUUUGCAGACCGAUUAUUU
env-726   AUAAUUAAUACCAACGCUUUCACGCUGUUAUGCGUUUCGUACCUAAUAAAAAAUGAGUAAUUUUGCAGACCGAUUAUUU
env-727   AUAAUUAAUAUCAAUACUUUCACACUGUUAUGCGUUUCGUACCUAAUAAAAAAUGAGUAAUUUUGCAGACCGAUUAUUU
env-729   AUAAUUAAUACCAACGCUUUCACGCUGUUAUGCGUUUCGUACCUAAUAAAAAAUGAGUAAUUUUGCAGACCGAUUAUUU
env-733   AUAAUUAAUAUCAACGCUUUCACGCUGUUAUGCGUUUCGUACCUAAUAAAAAAUGAGUAAUUUUGCAGACCGAUUAUUU
env-734   AUAAUUAAUACCAACGCUUUCACGCUGUUAUGCGUUUCGUACCUAAUAAAAAAUGAGUAAUUUUGCAGACCGAUUAUUU
env-735   AUGAUUAAUACCAACGCUUUCACGCUGUUAUGCGUUUCGUACCUAAUAAAAAAUGAGUAAUUUUGCAGACCGAUUAUUU
env-736   AUAAUUAAUACCAACACUUUCACGCUGUUAUGCGUUUCAUACCUAAUAAAAAAUGAGUAAUUUUGCAGACCGAUUAUUU
Pin-3-1   AUAAUUAAUACCAAUGCUUUCGCGCUGUUAUGCGUUUCGUACCUAAUAAAAAAUGAGUAAUUUUGCAGACCGAUUAUUU
Pfa-1-1   AUAAUUAAUACUAACACUUUCGCACUGUUAUGCGUUUUGUAGCUAAUAAAAAAUGAGUAAUUUUGCAGACCGAUUAUUU
env-737   AUAAUUAAUACCAACGCUUUCACGCUGUUAUGCGUUUCGUACCUAAUAAAAAAUGAGUAAUUUUGCAGACCGAUUAUUU
env-738   UAAUUAAUACCAACGCUUUCACGCUGUUAUGCGUUUCGUACCUAAAUAAAAAAUGAGUAAUUUUGCAGACCGAUUAUUU
env-742   AUAAUUAAUACCAACGCUUUCACGCUGUUAUGCGUUUCGUACCUAAUAAAAAAUGAGUAAUUUUGCAGACCGAUUAUUU
Pdi-3-1   AUUUGUAAAUAAUUCCCUUUGGCGGCUGCAUCGCAACUCUACCUAAUAAAAAAUGAGUAAUUUUGCAGACCGAUUAUUU
env-743   AUUUGUAAAUAAUUCCCUUUGGCGGCUGCAUCGCAACUCUACCUAAUAAAAAAUGAGUAAUUUUGCAGACCGAUUAUUU
Pdi-2-1   AUUUGUAAAUAAUUACCUUUGGCGGCUGCAUCGCAACUCUACCUAAUAAAAAAUGAGUAAUUUUGCAGACCGAUUAUUU
Pdi-1-1   AUUUGUAAAUAAUUCCCUUUGGCGGCUGCAUCGCAACUCUACCUAAUAAAAAAUGAGUAAUUUUGCAGACCGAUUAUUU
env-744   AUUAUAAAAGAUAGGGGGCUGCGGCUCGUAACUUACACAUAAUCAAAUAAAAAUGUGUAAUUUUGCAGUCCGAUUAUUU
env-745   AAUUAUAAAAGAUAAGGACUACGGAGCGUAACUUACACAUAAUCAAAUAAAAAUGUGUAAUUUUGCAGUCCGAUUAUUU
env-746   AAUUAUAAAAGAUAAGGACUGCGGCUCGUAACUUGCACAUAAUCAAAUAAAAAUGUGUAAUUUUGCAGUCCGAUUAUUU
env-747   UAUUAUAAAAGAUAGGGGCUGCGGCUCGUAACUUACACAUAAUCAAAUAAAAAUGUGUAAUUUUGCAGUCCGAUUAUUU
env-748   AAUUAUAAAAGAUAGGGGCUGCGACUCGUAACUUACACAUAAUCAAAUAAAAAUGUGUAAUUUUGCAGUCCGAUUAUUU
env-749   AAUUAUAAAAGAUAGGGGCUGCGGUUCGUAACUUACACAUAAUCAAAUAAAAAUGUGUAAUUUUGCAGUCCGAUUAUUU
env-751   UAUUAUAAAAGAUAGGGACUGCGGCUCGUAACUUACACAUAAUCAAAUAAAAAUGUGUAAUUUUGCAGUCCGAUUAUUU
env-752   UAUUAUAAAAGAUAGGGGCUGCGGCUCGUAACUUACACAUAAUCAAAUAAAAAUGUGUAAUUUUGCAGUCCGAUUAUUU
env-753   AAUUAUAAAAGAUAAGGACUACGGAGCGUAACUUACACAUAAUCAAAUAAAAAUGUGUAAUUUUGCAGUCCGAUUAUUU
env-754   AAUUAUAAAAGAUAAGGACUGCGGCUCGUAACUUACACAUAAUCAAAUAAAAAUGUGUAAUUUUGCAGUCCGAUUAUUU
env-755   AAUCAUAAAAGAUGGGGGCUGUGACUCGUAACUUACACAUAAUCAAAUAAAAAUGUGUAAUUUUGCAGUCCGAUUAUUU
env-756   UAUUAUAAAAGAUAGGGACUGCGGCUCGUAACUUACACAUAAUCAAAUAAAAAUGUGUAAUUUUGCAGUCCGAUUAUUU
env-757   AACUAUAAAAGAUAAGGAGGUGGAAUCGUAACCUACACAUAAUCAAAUAAAAAUGUGUAAUUUUGCAGUCCGAUUAUUU
env-758   AUUCAUAAAAGAUGGGGUGCAGGACUCGUAACAUUCACAUAAUCAAAUAAAAAUGUGUAAUUUUGCAGUCCGAUUAUUU
env-759   UUCAUAAAAGUUUGGGGGUUAAAACUCGUAACCUACACAUAAUCAAAUAAAAAUGUGUAAUUUUGCAGUCCGAUUAUUU
env-760   AAUUAUAAAAGAUAGGGGCUGCGGCUCGUAACUUACACAUAAUCAAAUAAAAAUGUGUAAUUUUGCAGUCCGAUUAUUU
env-761   AUUUAUAAAAAUUUUGGUUGAAAACUCGUAACUUACACAUAAUUAAAUUUAAAUGUGUACUUUUGCAGUCCGAUUAUAU
env-762   AUUUAUAAAAGUUUUGAUUGAAAACUCGUAACUUACACAUAAUCAAAUAAAAAUGUGUAAUUUUGCAGUCCGAUUAUUU
env-763   AUUCAUAAAAGUUUGGGGUUAAAACUCGUAACUUACACAUAAUUAAAUAAAAAUGUGUAAUUUUGCAGUCCGAUUAUUU
env-765   GCAGCUGAUAGGAUAUGUGCAGUAGCCUUGCUAUGUGGCGUGAAAAGAUGUNNNNNNNNNNNNUGCAGUCCGAUUAUUU
env-766   AUUUAUAAAAGUUUUGAUUGAAAACUCGUAACCUACACAUAAUCAAAUAAAAAUGUGUAAUUUUGCAGUCCGAUUAUUU
env-767   AUUCAUAAAAGUUUGGGGUUAAAACUCGUAACAUACACAUAAUUAAAUAAAAAUGUGUAAUUUUGCAGUCCGAUUAUUU
env-768   UCUAUAAAAGUAAAACACUUGAGUUUGUAACUUACACAUAAUUAAAUAAAAAUGUGUACUUUUGCAGUCCGAUUAUUUA
env-769   UCCGUAAAAAUAAUACUUGAGGACUGCAGAGUUACACAUAAUUAAAUAAAAAUGUGUACUUUUGCAGUCCGAUUAUUUG
env-770   UCUAUAAAAGUAAAGCACUUGAGUUUGUAACUUACACAUAAUUAAAUAAAAAUGUGUACUUUUGCAGUCCGAUUAUUUA
env-771   UCUAUAAAAGUAAAACACUUGAGUUUGUAACUUACACAUAAUUAAAUAAAAAUGUGUACUUUUGCAGUCCAAUUAUUUA
env-772   UCUAUAAAAGUAAAACACUUGAGUUUGUAACUUACACAUAAUUAAAUAAAAAUGUGUACUUUUGCAGUCCGAUUAUUUG

          ...............................................................................
          ...............................................................................
          ●●●●●●●●●●●●●●●●●●●●●●●●●●●●●●●●●●●●●●●●●●●●●●●●●R●●●●●●●●●●Y●Y●●●●●●●●●●●●●Y●●
```

env-774	AAUUAUAAAAAUUUUGGUUGAAACUUCGUAACUUACACAUAAUGAAAUAAAAAGGGUACUGUUGCAGUCCGAUUAUUUG
env-775	UCUAUAAAAGUAAAACGCUUGAGUUUGUAACUUACACAUAAUUAAAUAAAAAUGUGUACUUUUGCAGUCCGAUUAUUUG
env-776	UCUAUAAAAGUAAAACGCUUGAGUUUGUAACUUACACAUAAUUAAAUAAAAAUGUGUACUUUUGCAGUCCGAUUAUUUG
env-780	UUUAUAAAAAUUUUGGUUGAAAACUCGUAACUUACACAUAAUUAAAUAAAAAUGUGUACUUUUGCAGUCCGAUUAUUUG
env-781	AUUUAUAAAAGUUUGGGGUUAAAACUCGUAACCUACACAUAAUCAAAUAAAAAUGUGUAAUUUUGCAGUCCGAUUAUUU
env-785	AUUCAUAAAAGUUUGGGGUUAAAACUCGUAACCUACACAUAAUCAAAUAAAAAUGUGUAAUUUUGCAGUCCGAUUAUUU
env-787	AUUCAUAAAAGUUUGGGAUUAAAACUCGUAACCUACACAUAAUCAAAUAAAAAUGUGUAAUUUUGCAGUCCGAUUAUUU
env-788	AUUUAUAAAAGUUUGGGGUUAAAACUCCUAACCUACACAUAAUCAAAUAAAAAUGUGUAAUUUUGCAGUCCGAUUAUUU
env-789	AUUCAUAAAAGUUURGGGUUAAAACUCGUAACCUACACAUAAUCAAAUAAAAAUGUGUAAUUUUGCAGUCCGAUUAUUU
env-790	AUUYAUAAAAGUUUAGGGUUAAAACUCGUAACCUACACAUAAUCAAAUAAAAAUGUGUAAUUUUGCAGUCCGAUUAUUU
env-791	AUUCAUAAAAGUUUGGGGUUAAAACUCGUAACCUACACAUAAUCAAAUAAAAAUGUGUAAUUUUGCAGUCCGAUUAUUU
env-793	AUUUAUAAAAGUUUAGGGUUAAAACUCGUAACCUACACAUAAUCAAAUAAAAAUGUGUAAUUUUGCAGUCCGAUUAUUU
env-797	AUUUAUAAAAGUUUAGGGUUAAAACUCGUAACCUACACAUAAUCAAAUAAAAAUGUGUAAUUUUGCAGUCCGAUUAUUU
env-798	AUUUAUAAAAGUUUAGGGUUAAAACUCGUAACCUACACAUAAUCAAAUAAAAAUGUGUAAUUUUGCAGUCCGAUUAUUU
env-799	AUUCAUAAAAGAUGGGGUGCAAGACUCGUAACCUACACAUAAUCAAAUAAAAAUGUGUAAUUUUGCAGUCCGAUUAUUU
env-800	.CAUAAAAAGUUUGGGGUUAAAAACUCGUAACCUACACAUAAUCAAAUAAAAAUGUGUAAUUUUGCAGUCCGAUUAUUU
env-801	NNNNNNNNNNNUUUAGGGUUAAAACUCGUAACCUACACAUAAUCAAAUAAAAAUGUGUAAUUUUGCAGUCCGAUUAUUU
env-802	UUUAUAAAAAGUUUAGGGUUAAAACUCGUAACCUACACAUAAUCAAAUAAAAAUGUGUAAUUUUGCAGUCCGAUUAUUU
env-803	AUUUAUAAAAGUUUAGGGUUAAAACUCGUAACCUACACAUAAUCAAAUAAAAAUGUGUAAUUUUGCAGUCCGAUUAUUU
env-804	AUUCAUAAAAGUUUGGGAUUAAAACUCGUAACCUACACAUAAUCAAAUAAAAAUGUGUAAUUUUGCAGUCCGAUUAUUU
env-808	AUUCAUAAAAGUUUGGGGUUAAAACUCGUAACCUACACAUAAUCAAAUAAAAAUGUGUAAUUUUGCAGUCCGAUUAUUU
env-821	UUYAUAAAAGUUURGGGUUAAAAACUCGUAACCUACACAUAAUCAAAUAAAAAUGUGUAAUUUUGCARUCCGAUUAUUU
env-822	AUUUAUAAAAAUUCAGGGUUAAAACUCGUAACCUACACAUAAUCAAAUAAAAAUGUGUAAUUUUGCAGUCCGAUUAUUU
env-824	AUUUAUAAAAGUUUAGGGUUAAAACUCGUAACCUACACAUAAUCAAAUAAAAAUGUGUAAUUUUGCAGUCCGAUUAUUU
env-825	AUUUAUAAAAGUUUAGGGUUAAAACUCGUAACCUACACAUAAUCAAAUAAAAAUGUGUAAUUUUGCAGUCCGAUUAUUU
env-826	AUUCAUAAAAGUUURGGGUUAAAACUCGUAACCUACACAUAAUCAAAUAAAAAUGUGUAAUUUUGCAGUCCGAUUAUUU
env-827	AUUCAUAAAAGUUUGGGGUUAAAAAUCGUAACCUACACAUAAUCAAAGAAAAAUGUGUAAUUUUGCAGUCCGAUUAUUU
env-828	....................................ACAUAAUCAAAUAAAAAUGUGUAAUUUUGCAGUCCGAUUAUUU
env-829	AUUCAUAAAAGUUUGGGGUUAAAACUCGUAACCUACACAUAAUCAAAUAAAAAUGUGUAAUUUUGCAGUCCGAUUAUUU
env-830	UCAUAAAAGUUUGGGGUUAAAACUCGUAACCUACACAUAAUCAAAUAAAAAAUGUGUAAUUUUGCARUCCGAUUAUUUC
env-831	AUUCAUAAAAGUUUGAGAUUAAAACUCGUAACCUACACAUAAUCAAAUAAAAAUGUGUAAUUUUGCAGUCCGAUUAUUU
env-832	....GUGUAAUUUUGCAGUCCGAUUAUUU
env-833	AAUAUUCAUAAGUUUAGGGUUAAAACUCGUAACCUACACAUAAUCAAAUAAAAUGUGUAAUUUUGCAGUCCGAUUAUUU
env-834	AUUUAUAAAAUACCUGCUUUUAAGUUUGUAACCUACACAAAAUCAAAUAAAAAUGUGUAAUUUUGCAGUCCGAUUAUUU
env-835	AUUCAUAAAAGUUUGGGGUUAAAACUCGUAACCUACACAUAAUCAAAAAAAUAUGUGUAAUUUUGCAGUCCGAUUAUUU
env-836	UUUCAUAAAAGUUUGGGUUAAAAACUCGUAACCUACACAUAAUCAAAUAAAAAUGUGUAAUUUUGCAGUCCGAUUAUUU
env-837	UUUUAUAAAAGUUUGGGUUUAAAACUCGUAACCUACACAUAAUCAAAUAAAAAUGUGUAAUUUUGCAGUCCGAUUAUUU
Pco-2-1	UUUUAUAAAAGUUUGGGUUUGAAACUCGUAACCUACACAUAAUCAAAUAAAAAUGUGUAACUUUGCAGUCCGAUUAUUU
env-838	UUUAUAAAAGUUUGGGGUUAAGACUCGUAACCUACACAUAAUCAAAUAAAAAAUGUGUAAUUUUGCAGUCCGAUUAUUU
env-839	AUUUAUAAAAGUUUGGGAUUAAAACUCGUAACCUACACAUAAUCAAAUAAAAAUGUGUAAUUUUGCAGUCCGAUUAUUU
env-840	AUUCAUAAAAGUUUGGGGUUAAGACUCGUAACCUACACAUAAUCAAAUAAAAAUGUGUAAUUUUGCAGUCCGAUUAUUU
env-842	AUUCAUAAAAGUUUGGGGUUAAAACUCGUAACCUACACAUAAUCAAAAAAAUAUGUGUAAUUUUGCAGUCCGAUUAUUU
env-843	AUUUAUAAAAGUUUGGGGUUAAAACUCGUAACCUACACAUAAUCAAAUAAAAAUGUGUAAUUUUGCAGUCCGAUUAUUU
Por-8-1	AUGAUUAAAUAGAUAAUUUUAAUCAUUUCUUAUUUUCCAACUUACGAUUAUACUUCGUACCUUUGCAGUCCGAUUAUUU
Psp-21-1	AAUAAUAAAAUAGGAGAUUUGUAAUAGCUCUGAUUAUACCAUGUAAAUAAAACGUCGUAACUUUGCAGUCCGAUUAUUU
env-845	NNNNNNNNNNNNNNNNNNNUGCUCCCAUGCUGUCUUCGCCAUUCGGCAGAAUCUUCGUACCUUUGCAGUCCGAUUAUU
env-846	AAUAUUAAAUCAACACCUUUUACCCCCAUGCUAUCUUCGCCAUUCGGCAGAAUCUUCGUACCUUUGCAGUCCGAUUAUU
env-847	GUUAAAUGUUUAGCCAAAUUACGCAUUACGCAUUACGCUUUACGCAUUAAAAUCGUACCUUUGCAGUCCGAUUAUUACA
env-848	ACGUAACAACAAAGUUAAAUAGCUGUCGCACUUAUUAGUUAAAGCAUAUUAAUAUGUACCUUUGCAGUCCGAUUAUUAU
env-849	ACGUAACAACAAAGUUAAAUAGCUGUCGCACUUAUUAGUUAAAGCAUAUUAAUAUGUACCUUUGCAGUCCGAUUAUUAU
env-851	GCGUAACGACAAAGUUAAAUAGCUGUCGCACUUAUUAGUUAAAGCAUAUUAAUAUGUACCUUUGCAGUCCGAUUAUUAU
env-852	ACGUAACGACAAAGUUAAAUAGCCAUCGUACUUAUUAGUUAAAGCAUAUUAAUAUGUACCUUUGCAGUCCGAUUAUUAU
env-855	ACGUAACGGUAAAGUUAAAUAGCUGUCGCACUUAUUAGUUAAAGCAUAUUAAUAUGUACCUUUGCAGUCCGAUUAUUAU
env-856	ACAUAACGAUAAAGUUAAAUAGCUGUCGCACUUAUUAGUUAAAGCAUAUUAAUACGUACCUUUGCAGUCCGAUUAUUAU
env-857	ACAUAACGAUAAAGUUAAAUAGCUGUCGCACUUAUUAGUUAAAGCAUAUUAAUAUGUACCUUUGCAGUCCGAUUAUUAU
env-858	ACAUAACGAUAAAGUUAAAUAGCUGUCGCACUUAUUAGUUAAAGCAUAUUAAUACGUACCUUUGCAGUCCGAUUAUUAU
env-859	ACGUAACGACAAAGUUAAAUAGCUGUCGCACUUAUUAGUUAAAGCAUAUUAAUAUGUACCUUUGCAGUCCGAUUAUUAU
env-860	ACAUAACGACAAGGUUAAAUAUGCGCCGCAUCUAUUAGUUAAAGCAUAUUAAUAUGUACCUUUGCAGUCCGAUUAUUAU
env-861	GCGUAACGACAAAGUUAAAUAGCUGUCGCACUUAUUAGUUAAAGCAUAUUAAUAUGUACCUUUGCAGUCCGAUUAUUAU
env-862	ACAUAACGGCAAAGUUAAAUAGCAAUCGCACUUAUUAGUUAAAGCAUAUUAAUAUGUACCUUUGCAGUCCGAUUAUUAU
env-864	ACGUAACGGCAAAGUUAAAUAUGCGCCGCAUUUAUUAGUUAAAGCAUAUUAAUAUGUACCUUUGCAGUCCGAUUAUUAU
env-865	ACGUAAUGACAAAGUUAAAUAUGCGCCGCAUUUAUUAGUUAAAGCAUAUUAAUAUGUACCUUUGCAGUCCGAUUAUUAU
env-866	ACAUAACGAUAAGGUUAAAUAGCUGUCGCACUUAUUAGUUAAAGCAUAUUAAUAUGUACCUUUGCAGUCCGAUUAUUAU
env-868	ACAUAACGAUAAGGUUAAAUAGCUGUCGCUCUUAUUAGUUAAAGCAUAUUAAUAUGUACCUUUGCAGUCCGAUUAUUAU
env-869	ACGUAACGACAAGGUUAAAUAUGCGCCGCAUUUAUUAGUUAAAGCAUAUUAAUAUGUACCUUUGCAGUCCGAUUAUUAU
env-870	ACAUAACGGCAAAGUUAAAUAUGCGCCGCAUUUAUUAGUUAAAGCAUAUUAAUAUGUACCUUUGCAGUCCGAUUAUUAU
env-872	AUUUAUCGUGNNNNNNNNNNNNNNNNNNNNNNNNNNNNNNNNNNNNNNNNNNNAAUACGUACCUUUGCAGUCCGAUUAUUAU
env-873	ACAUAACGGUAAAGUUAAAUAGCCAUCGCACUUAUUAGUUAAAGCAUAUUAAUACGUACCUUUGCAGUCCGAUUAUUAU
env-874	AUAUAACGACAAAGUUAAAUAGCUGUCGCACUUAUUAGUUAAAGCAUAUUAAUAUGUACCUUUGCAGUCCGAUUAUUAU
env-875	ACAUAACGGCAAGGUUAAAUAGCUGUCGCACUUAUUAGUUAAAGCAUAUUAAUAUGUACCUUUGCAGUCCGAUUAUUAU
env-876	ACAUAACGGCAAGGUUAAAUAGCUGUCGCACUUAUUAGUUAAAGCAUAUUAAUAUGUACCUUUGCAGUCCGAUUAUUAU
Psp-25-1	ACGUAACGAUAAAGUUAAAUAGCUGUCGCACUUAUUAGUUAAAGCAUAUUAAUACGUACCUUUGCAGUCCGAUUAUUAU
env-877	ACAUAACGACAAAGUUAAAUAGCUGUCGCACUUAUUAGUUAAAGCAUAUUAAUAUGUACCUUUGCAGUCCGAUUAUUAU
env-878	ACAUAAUGGCAAAGUUAAAUAGCAAUCGCACUUAUUAGUUAAAGCAUAUUAAUAUGUACCUUUGCAGUCCGAUUAUUAU
env-880	ACAUAACGACAAAGUUAAAUAGCCAUCGUACUUAUUAGUUAAAGCAUAUUAAUAUGUACCUUUGCAGUCCGAUUAUUAU
env-881	ACGUAACGAUAAAGUUAAAUAGCUGUCGCACUUAUUAGUUAAAGCAUAUUAAUACGUACCUUUGCAGUCCGAUUAUUAU
env-882	ACAUAACGGCAAAGUUAAAUAGCUGUCGCACUUAUUAGUUAAAGCAUAUUAAUAUGUACCUUUGCAGUCCGAUUAUUAU
env-883	ACGUAACGAUAAAGUUAAAUAGCUGUCGCACUUAUUAGUUAAAGCAUAUUAAUAUGUACCUUUGCAGUCCGAUUAUUAU
env-885	ACAUAACGGUAAAGUUAAAUAGCCAUCGCACUUAUUAGUUAAAGCAUAUUAAUACGUACCUUUGCAGUCCGAUUAUUAU
env-886	AGCGUAACGACAAAGUUAAAUAGCUGUCGCACUUAUUAGUUAAAGCACAUUAAUUCGUACCUUUGCAGUCCGAUUAUUA
env-887	UAUAUAACGCUAAGGUUAAAUAUGCGAUGCGCUUAUUAGUUAAAGCAUAUUAAUGCGUACCUUUGCAGUCCGAUUAUUA
env-888	UAUAUAACGCUAAGGUUAAAUAUGCGAUGCACUUAUUAGUUAAAGCAUAUUAAUGCGUACCUUUGCAGUCCGAUUAUUA
env-889	UAUAUAACUCUAAGGUUAAAUAUGCGAUGCGCUUAUUAGUUAAAGCAUAUUAAUGCGUACCUUUGCAGUCCGAUUAUUA
env-890	UAUAUAACGCUAAGGUUAAAUAUGCGAUGCGCUUAUUAGUUAAAGCAUAUUAAUGCGUACCUUUGCAGUCCGAUUAUUA
	....
	....
	●●●R●●●●●●●●●●●Y●Y●●●●●●●●●●●●●Y●●

env-891	UAUAUAACUCUAAGGUUAAAUAUGCGAUGCGUUUAUUAGUUAAAGCAUAUUAAUGCGUACCUUUGCAGUCCGAUUAUUA
env-892	UAUAUAAUGCUAAGGUUAAAUAUGCGAUGCGUUUAUUAGUUAAAGCAUAUUAAUGCGUACCUUUGCAGUCCGAUUAUUA
env-893	UAUAUAACGCUAAGGUUAAAUAUGCGAUAUGCUUAUUAGUUAAAGCAUAUUAAUGCGUACCUUUGCAGUCCGAUUAUUA
env-894	UAUAUAACGCUAAGGUUAAAUAUGCGAUGCGUUUAUUAGUUAAAGCAUAUUAAUGCGUACCUUUGCAGUCCGAUUAUUA
env-900	UAUAUAACGCUAAGGUUAAAUAUGCGAUGCGUUUAUUCGUUAAAGCAUAUUAAUGCGUACCUUUGCAGUCCGAUUAUUA
env-901	UAUAUAACGCUAAGGUUAAAUAUGCGAUGCGUUUAUUCGUUAAAGCAUAUUAAUGCGUACCUUUGCAGUCCGAUUAUUA
env-902	UAUAUAACGCUAAGGUUAAAUAUGCGAUGCGUUUAUUCGUUAAAGCAUAUUAAUGCGUACCUUUGCAGUCCGAUUAUUA
Psp-26-1	UAUAUAACGCUAAGGUUAAAUAUGCGAUGCGUUUAUUAGUUAAAGCAUAUUAAUGUGUACCUUUGCAGUCCGAUUAUUA
env-903	UAUAUAACGCUAAGGUUAAAUAUGCGAUGCGCUUAUUAGUUAAAGCAUAUUAAUGCGUACCUUUGCAGUCCGAUUAUUA
env-921	AACGUAACGACAAAGUUAAAUAGCUGUCGUACUUAUUAGUUAAAGCACAUUAAUUCGUACCUUUGCAGUCCGAUUAUUA
env-922	AGCGUAACGACAAAGUUAAAUAACUGUCGCACUUAUUAGUUAAAGCACAUUAAUUCGUACCUUUGCAGUCCGAUUAUUA
env-923	AACGUAACAACAAAGUUAAAUAGCUGUCGCACUUAUUAGUUAAAGCACAUUAAUUCGUACCUUUGCAGUCCGAUUAUUA
env-924	AGCGUAACGACAAAGUUAAAUAGCUGUCGCACUUAUUAGUUAAAGCACAUUAAUUCGUACCUUUGCAGUCCGAUUAUUA
env-932	AACGUAACGACAAAGUUAAAUAGCUGUCGCACUUAUUAGUUAAAGCACAUUAAUUCGUACCUUUGCAGUCCGAUUAUUA
env-948	ACGUAACGACAAAGUUAAAUAGCUGUCGUACUUAUUAGUUAAAGCACAUUAAUUCGUACCUUUGCAGUCCGAUUAUUAU
env-949	AACGUAACAACAAAGUUAAAUAGCUGUCGCACUUAUUAGUUAAAGCACAUUAAUUCGUACCUUUGCAGUCCGAUUAUUA
env-950	AGCGUAACGACAAAGUUAAAUAACUGUCGCACUUAUUAGUUAAAGCACAUUAAUUCGUACCUUUGCAGUCCGAUUAUUA
env-953	AACGUAACGACAAAGUUAAAUAGCUGUCGCACUUAUUAGUUAAAGCACAUUAAUUCGUACCUUUGCAGUCCGAUUAUUA
env-957	AACGUAACGGCAAAGUUAAAUAGCUGUCGCACUUAUUAGUUAAAGCACAUUAAUUCGUACCUUUGCAGUCCGAUUAUUA
env-958	AACGUAACGGCAAAGUUAAAUAGCUGUCGCACUUAUUAGUUAAAGCACAUUAAUUCGUACCUUUGCAGUCCGAUUAUUA
env-960	UUCAUUAAUAUUACUUUCCCCUCGCCGUUCCCGUUUGCUCCCGCUGAAUAAAUUAAGUAAUUUUGCAGUCCGAUUAUUU
env-961	UUCAUUAAUAUUACUUUCCCCGCGCCGUUCCCGUUUGCUCCCGCUGAAUAAAUUAAGUAAUUUUGCAGUCCGAUUAUUU
env-967	UAAUUCUAAAUAUUCGCCGAUGCUUGCUGUAUGUCUCUUUUAUAUAAUAAAAAAUGUGUAAUUUUGCAGACCGAUUAUU
env-968	UAAUUCUAAAUAUUCGCCGAUGCUUGCUGUAUGUCUCUUUUAUAUAAUAAAAAAUGUGUAAUUUUGCAGACCGAUUAUU
env-970	UAAUUCUAAAUAUUCGCCAAUGCUUGCUGUAUGUCUCUUUUAUAUAAUAAAAAAUGUGUAAUUUUGCAGACCGAUUAUU
env-971	UAAUUCUAAAUAUUCGCCAAUGCGUGCUGUAUGUCUCUUUUAUAUAAUAAAAAAUGUGUAAUUUUGCAGACCGAUUAUU
env-972	UAAUUCUAAAUAUUCGUUGCUGCUUAUUGUAUAUCACUUCUAUAUAAUAAAAAAUGUGUAAUUUUGCAGACCGAUUAUU
env-973	....AUAAAAAAUGUGUAAUUUUGCAGACCGAUUAUU
env-977	UAAUUCUAAAUAUUCGUUGCCGCUUAUUGUAUAUCACUUCUAUAUAAUAAAAAAUGUGUAAUUUUGCAGACCGAUUAUU
env-978	UAAUUCUAAAUAUUCGUUGCUGCUUAUUGCAUAUUACUUCUAUAUAAUAAAAAAUGUGUAAUUUUGCAGACCGAUUAUU
env-979	UAAUUCUAAAUAUUCGUUGCUGCUUAUCGUAUCUCACUUCUAUAUAAUAAAAAAUGUGUAAUUUUGCAGACCGAUUAUU
env-981	UAAUUCUAAAUAUUCGUUGCUGCUUAUUGUAUAUCACUUCUAUAUAAUAAAAAAUGUGUAAUUUUGCAGACCGAUUAUU
env-982	UAAUUCUAAAUAUUCGUUGCUGCUUAUUGCAUGUUACUUCUAUAUAAUAAAAAAUGUGUAAUUUUGCAGACCGAUUAUU
env-983	UAAUUCUAAAUAUUCGUUGCCGCUUAUUGCAUAUCACUUCUAUAUAAUAAAAAAUGUGUAAUUUUGCAGACCGAUUAUU
env-984	UAAUUCUAAAUAUUCGUUGCUGCUUAUUGCAUAUUACUUCUAUACAAUAAAAAAUGUGUAAUUUUGCAGACCGAUUAUU
env-985	UAACUUUAAAUAUUACACUUUGUGAGCUGUCUUGUUUUUGUAUAUAAUAAAUAAUGAGUAAUUUUGCGGACCGAUUAUU
env-986	UAAUUUUAAAUAUUACGCUUUGUGAGCUGUCUUGUUUUUGUAUAUAAUAAAUAAUGAGUAAUUUUGCGGACCGAUUAUU
env-990	UAAAUGUAAAUAAGCGGGCUGCUUAAAAGCAUGAUUUCUUUAUAUAAUAAAAAAUGAGUAAUUUUGCGGACCGAUUAUU
env-992	UUAAUUAAAAAAAUGAACACUUUAAGUUACAAGACUAUUUCCGUAAAUAAGGAAACAGCUAANNNNNNNNNNGAUUAUU
env-993	UAAACGUAAAUAUACAGUAUGCUUAAAAGCAUCAUUUCUCUAUAUAAUAAAAAAUGAGUAAUUUUGCGGACCGAUUAUU
env-994	UUUAUUAAUAUUAUAUUCUUCCCCGAUUCCGUUCUUGCUUUCAAAAAAUAAAAUAUGUAAUUUUGCAGUCCGAUUAUUU
Pbu-3-1	UAGUAUUUAAACGAUUGUGUUCUUGAUGCGAUAAUGGUGCGUUGUUUUUAAUAAAAUAUGUAACUUUGCACUCCGAUUG
Pbu-1-1	UAGUGUUUAAACGAUUGUAUUCUUGAUGCGAUAAUGGUGCGUUGUUUUUAAUAAAAUAUGUAACUUUGCACUCCGAUUG
Pbu-2-1	UAGUGUUUAAACGAUUGUGUUCUUGAUGUGAUAAUGGUGCGUUGUUUUUAAUAAAAUAUGUAACUUUGCACUCCGAUUG
Psp-12-1	UAGUGUUUAAACGAUUGUGUUCUUGAUGCGAUAAUGGUGCGUUGUUUUUAAUAAAAUAUGUAACUUUGCACUCCGAUUG
env-995	UCAUCCAAAUAGGAUAAAAAAGUCAUUUCUUGUUUGCGAAACACAUAAAAUGCGUACCUUUGCAGUCCGAUUAUUACAU
env-996	AUAAAUCUUACGAUAAAUUGUGAUGCUUUUUGUUUGCGAUAUAUAUAAAAUGCGUACCUUUGCAGUCCGAUUAUUACAA
env-997	UCAAAAAAUAGUUCUCCCUUACACAUCUCCCGUUAAAUAUAAGUCUGAAAUGUUUUGUAUCUUUGCAGUCCGAUUAUUU
env-999	UACAUAAAAUACACACAUUUGACCUUCAGAAGUAAAUAACAAAAGAAAAAGUAUAAGUAACUUUGCAGACCGAUUAUUU
Por-1-1	UUAUAAAAUUGGUUUUAAGCGUAUCUUAUACAGUACUCUAAUCUGAAAAAUAUACGUACUUUUGCAGACCGAUUAUUUU
env-1001	UUAUAAAAUUGGUUUUAAGCGUAUCUUAUACAGUACUCUAAUCUGAAAAAUAUACGUACUUUUGCAGACCGAUUAUUUU
Por-2-1	UUAUAAAAUUGGUUUUAAGCGUAUCUUAUACAGUACUCUAAUCUGAAAAAUAUACGUACUUUUGCAGACCGAUUAUUUU
env-1002	AGAUAUAAUUAAACGGGCUGGAUUGUACCUUUUCUUUGCACAUUACUAUUAAAUAUGUAUUUUUGCAGACCGAUUAUUU
env-1003	AGAUAUAAUUAAAAGGGCUGGAUAGUACCUUUUCUUUGCACUUUAAUGUUAAAUAUGUAUUUUUGCAGACCGAUUAUUU
Pti-3-1	AGAUAUAAUUAAACAGGCUGGUUUGUACCUUUUCUUUGCACUUUACAGUUAAAUAUGUAUUUUUGCAGACCGAUUAUUU
env-1004	AGAUAUAAUUAAACGGGCUGGAUUGUACCUUUUCUUUGCACAUUACUAUUAAAUAUGUAUUUUUGCAGACCGAUUAUUU
Pti-1-1	AGAUAUAAUUAAGCGUGCGGGGUUGUAUCUUUUCUUUGCACUUUACUAUUAAAUAUGUACUUUUGCAGACCGAUUAUUU
Pti-2-1	AGAUAUAAUUAAGCGUGCGGGGUUGUAUCUUUUCUUUGCACUUUACUAUUAAAUAUGUACUUUUGCAGACCGAUUAUUU
env-1005	AGAUAUAAUUAAACGGGCUGGAUUGUACCUUUUCUUUGCACAUUACUAUUAAAUAUGUACUUUUGCAGACCGAUUAUUU
env-1006	AUUAUAAAAUAAUGGCUAUUGAGAGAGUCUAAUGUUGGCUAUGUUUAUAUAUAUGUGUAUUUUUGCAGACCGAUUAUUU
Psp-4-1	AUUAUAAAAUAAUAGCUAUUGAGAGAGUCUAAUGUUGGCUAUGUUUAUAGAUAUGUGUAUUUUUGCAGACCGAUUAUUU
env-1007	AUUAUAAAAUAGCAGCUAUUAAGAGAGCCUAAUGUUGGCUAUGUUAAUAGAUAUGUGUAUUUUUGCAGACCGAUUAUUU
Pbu-5-1	AUUAUAAAAUAAUAGCUAUUAAGAGAGUCUAAUGUUGGCUAUGUUUAUAGAUAUGUGUAUUUUUGCAGACCGAUUAUUU
Pbu-4-1	AUUAUAAAAUAACGGCUAUUAAGAGAGCCUAAUGUUGGCUAUGUUUAUAGAUAUGUGUAUUUUUGCAGACCGAUUAUUU
env-1008	UACAUAAUUCACAAGAUUUCAUCCUGGGUCUUACCUGUGUUUUAUUUAUAAAAUAUGUAACUUUGCAGUCCGAUUAUUU
Pen-1-1	UUCGUAAUUUAUAGCAUCUCGUUUUUUGCCCGACUAGCGUUUCUCUUAUAAAAUAUGUAACUUUGCAGUCCGAUUAUUU
env-1009	UACAUAAUUCACAAGAUUUCAUCCUGGGUCUUACCUGUGUUUUAUUUAUAAAAUAUGUAACUUUGCAGUCCGAUUAUUU
env-1017	....UGCAGUCCGAUUAUUU
env-1020	UACAUAAUUCACAAAAUUUCAUCCUGGGUCUUACCUGUGUUUUAUUUAUAAAAUAUGUAACUUUGCAGUCCGAUUAUUU
Ppl-2-1	UGCAUAAUUCACAAGAUUUCAUCCUGGGUCUUACCUGUGUUUUAUUUAUAAAAUAUGUAACUUUGCAGUCCGAUUAUUU
env-1021	UUCAUAAAUCUUCAUCAUUGGGAGCAUCCCGAUAAUGGCUGAUAUAAAAAUAAUGUGUAAUUUUGCACGCCGAUUAUUU
env-1022	UUCAUAAAUAUUCAUCAUUGGAAGCGCCUCGAUAAUGGCUGAUAUAAAAAUAAUGUGUAAUUUUGCACGCCGAUUAUUU
env-1023	UUCAUAAAUCUUCAUCAUUGGGAGCGUCUCGAUAAUGGCUGAUAUAAAAAUAAUGUGUAAUUUUGCACGCCGAUUAUUU
env-1024	GAUUUUAAAUCAAAUAGCUUCAUGCGCUCAUAACUUGCUGAUAUAAGAUAUAAUGUGUAAUUUUGCAGACCGAUUAUUU
env-1025	GUUUUUAAAUCAAAUGGUCUCAUGCGCUCAUAACUUGCUGAUAUAAGAUAUAAUGUGUAAUUUUGCAGACCGAUUAUUU
env-1027	AUGUUAAAAAACCGUAACCAACAAGCGAAAAUGAGUAUAUAUUCAAACAUUACUUCGUACCUUUGCAGUCCGAUUAUUU
Pma-4-1	GUGUUAAAAAACCAUAACUAAUAAGCAAAAAUGAGUAUGUAUUCAAACAUUACUUCGUACCUUUGCAGUCCGAUUAUUU
env-1028	....CUUUGCAGUCCGAUUAUUU
env-1029	CACAAAUUUAAAAUUUAGAGGGCAUCUGUUUUUCAUUACGAUAUGAAAAAUGAGUACCUUUGCAGUCCGAUUAUUAUUU
env-1031	CUCAAUUUGAAAAUAUUUGAUGGUGUAUGUUUUUCAAUACGCGAUGAAAAAUGUGUACCUUUGCAGUCCGAUUAUUAUU
env-1032	UUGUUUAAAUUUCGUCUUUUUCGCGUUUUAUUACGCGCGUAGAAGGGUUGGAAUGUGUACUUUUGCAGACCGAUUAUUU
env-1033	UUGUUUAAAUUUCGUCUUUUUCGCGUUUUAUUACGCGCGUAGAAGCGUUGGAAUGUGUACUUUUGCAGACCGAUUAUUU
env-1034	AUCCUAAUUAUCAGCCCCUUACCCACCUCCAUUCUUGGCCCAAUAAAAGAUAAUGUGUAAUUUUGCAGACCGAUUAUUU
env-1035	UCUAUAAAUAUGAUUGUCCGAUGGCAGAAUGAUAACAUGCAGGUAAAAUAAAAUAAGUACUUUUGCAGACCGAUUAUUU
	....
	....
	●●R●●●●●●●●●●Y●Y●●●●●●●●●●●●●Y●●

```
env-1037  ..............AGCUUUAAGAAGAGCUAUCGAUGUGAAAAAUAAAUAAAAUGUGUACCUUUGCAGUCCGAUUAUUA
env-1038  AAUAUAAUACAACACAUUCAACGCCCUUCCCUGCGAACGAGUUAUAAUAAAAAUAUGUACCUUUGCAGUCCGAUUAUUA
env-1039  AAUAUAAUACAACACAUUCCGCGCCCUUCCCUGCGAACGAGUUAUAAUAAAAAUAUGUACCUUUACAGUCCGAUUAUUA
env-1040  AAUAUAAUACAACACAUUCAACGCCCUUCCCAGCGAACGAGUUAUAAUAAAAAUAUGUACCUUUGCAGUCCGAUUAUUA
env-1043  AAUAUAAUACAGCACAUUCAACGCCCUUCCCUGCGAACGAGUUAUAAUAAAAAUAUGUACCUUUGCAGUCCGAUUAUUA
env-1044  AAUAUAAUACAGCACAUUCAGCGCCCUUCCCUGCGAACGAGUUAUAAUAAAAAUAUGUACCUUUGCAGUCCGAUUAUUA
env-1046  AAUAUAAUACAACACAUUCAACGCCCUUCCCUGCGAACGAGUUAUAAUAAAAAUAUGUACCUUUGCAGUCCGAUUAUUA
env-1052  AAUAUAACUAAAAGGUAUUAAAAGCAUACAGCUAUUCAUUCUUAUUAAUAAAAUAUGUACCUUUGCAGUCCGAUUAUUA
env-1053  AAUAUAAUACAACACAUUCAACGCCCUUCCCUGCGAACGAGUUAUAAUAAAAAUGUGUACCUUUGCAGUCCGAUUAUUA
Psp-2-1   AAUAUAAUACAACACGUUCAACGCCCUUCCCUGCGAACGAGUUAUAAUAAAAAUAUGUACCUUUGCAGUCCGAUUAUUA
env-1054  AAUAUAAUACAACACGUUCAGCGCCCUUCCCUGCGAACGAGUUAUAAUAAAAAUAUGUACCUUUGCAGUCCGAUUAUUA
env-1055  AAUAUAAUACAACACAUUCCGCGCCCUUCCCUGCGAACGAGUUAUAAUAAAAAUAUGUACCUUUGCAGUCCGAUUAUUA
env-1056  AAUAUAAUACAACACAUUCAGCGCCCUUCCCUGCGAACGAGUUAUAAUAAAAAUAUGUACCUUUGCAGUCCGAUUAUUA
env-1058  AAUGUUAAUGUUUUAUAAUUUGUGGUUUUGAGCAUCUCGUAAUCCUUAUAAAAUUAGUACCUUUGCAGUCCGAUUAUUA
env-1059  ....................................................AGUAGUACCUUUGCAGUCCGAUUAUUA
env-1060  CCGCUAAAAAUUAUUAAUUUCGUUUUCUCCCUAACCUCACAUAAUAAAUAAAAGUAGUACCUUUGCAGUCCGAUUAUUA
env-1061  UCAUAAAAAUAAAAUUCCAACAAGUCUUGUAUUAUAAAUAUAAAGGAAAAAUUCAUACCUUUGCAAUCCGAUUAUUAAA
Psp-13-1  UCAUAAAAAUAAAAUCCUAACGAGUCUUGUAUUAUAAAUAUAAAGGAAAAAUUCAUACCUUUGCAAUCCGAUUAUUAAA
env-1062  UCAUAAAAAUAAAAUUCUAACGAGUCUUGUAUUAUAAAUAUAAAGGAAAAAUUCAUACCUUUGCAAUCCGAUUAUUAAA
Psp-17-1  UCAUAAAAAUAAAAUCCUAACAAGUCUUGUAUUAUAAAUAUAAAGGAAAAAUUCAUACCUUUGCAAUCCGAUUAUUAAA
env-1063  UCAUAAAAAUAAAACUCUAACGAGUCUUGUAUUAUAAAUAUAAAGGAAAAAUUCAUACCUUUGCAAUCCGAUUAUUAAA
Psp-15-1  UCAUAAAAAUAAAAUUCUAACGAGUCUUGUAUUAUAAAUAUAAAGGAAAAAUUCAUACCUUUGCAAUCCGAUUAUUAAA
Psp-16-1  UCAUAAAAAUAAAAUUCUAACGAGUCUUGUAUUAUAAAUAUAAAGGAAAAAUUCAUACCUUUGCAAUCCGAUUAUUAAA
env-1064  AUAUAAAAACAAAGCUUACAUUCUUCCAUAUAUAAUGUACAUAUAAAAAUUAUUCAUACCUUUGCAAUCCGAUAAUUUA
env-1065  AUAUAAAAUCACAGUUUGCAUUCUUUCAUAUAUAAUGUACAUACAAAAAUUAUUCAUACCUUUGCAAUCCGAUUAUUUA
env-1066  AUAUAAAAACAAAGCUUACAUUCUUCCAUAUAUAAUGUACAUAUAAAAAUUAUUCAUACCUUUGCAAUCCGAUUAUUUA
env-1067  .................UUUCUUGGUGCACUGAAUUCAGCGGACCAAUAAAAAUGUGUACCUUUGCAGUCCGAUUAUUA
Hse-1-1   CUUAAUCCAUGGGCGUUUUCACCGUUUUGUCGUGCGUUGUACGUCAUAAAAUGUGUAAUUUUGCAGAACCGAUUAUUUC
env-1068  AAUAUAAAACAAAGGUCGCACCGGCGUCCAAUCACAUUUUAUUAUGGAGAAAAAUACUACCUUUGCAGACCGAUUAUUG
env-1069  GCAUUUUCUUCACGCGUGAGGAGAUGCAUUUGCGAGAAAAUAAGUAAUUUUGCAGUCCGAUUAAUUCAAUCCGAUUAAA
env-1071  AAUAAUCACAAAAACGCAAUAUCAAGACCUACAUUAUGCUGCUAUGAAAAGAAAAAGCUGUACCUUUGCAGUCCGAUUA
env-1072  AACCUUAAAAGAUUGGUUCUUAAGCAGAAUGACGGACAGGUUUUCACCUGAAAACCACUACUUUUGCAGUCCAAACAAA
env-1074  ACCUUAAAAGAUUGAUUCUUAAGCAGAAUGACGGACAGGUUUUCAUCUGAAAACCACUACUUUUUGCAGUCCAAACAAA
env-1075  AACCUUAAAAGAUUGAUUCUUAAGCAGAAUGACGGACAGGUUUUCAUCUGAAAACCACUACUUUUGCAGUCCAAACAAA
env-1076  UUCAUAAAAAAACGGCGUCGAUGGUAGGAGAUUUAUUGCCGAUAGACAAUUAAUUAUUACCUUUGCAGUCCGAUUAUUAU
env-1077  ........................................................UACCUUUGCAGUCCGAUUAUUAU
env-1078  AAUUAUCAUAAAAGUUUGGUUUCGGAAGGGGAAAGGGCUUCGCAUAUUAACAAAUAUGUGUACCUUUGCAGUCCGAUUA
env-1079  AAUAUCUAUAAAAUACCUAUCCUGCGUGGGUGGUGCUUUCUUAGAAAAGAAAAUUCUUCGUACCUUUGCAGUCCGAUUU
env-1080  AUAAUCAUAAAUCUUUCAUCGCUUAUCGCUUAUCACUUGUUGCUUGUCAUUUAAAAUAUGUACCUUUGCAGUCCGAUUU
env-1081  AAUAAUCAUAAUCCGAGGCAGAUUCUUCACGCUUCACUCUUCACUCUUCACUUAAAAUAUGUACCUUUGCAGUCCGAUU
env-1083  AAUAAUCAUAAUCCGAGGCAGAUUCUUCACGCUUCACUCUUCACUCUUCACUUAAAAUAUGUACCUUUGCAGUCCGAUU
env-1084  AAAAACAUAAAAGAGACGUUUUUCGUUGUUACGCGUACAUUUAAAAGAAGAAAAAGCCGUACCUUUGCAGUCCGAUUAC
env-1085  ..........................................................GUACCUUUGCAGUCCGAUUAU
env-1086  AUAAUAUUAAAUCUUUAACUUAUCCAGUUUCUUAUUUUCCAGAUUCAAGAGAAAAGCUGUACCUUUGCAGUCCGAUUAU
env-1087  AAUUACAGCUGAUUGGCUCUAUUUCACUCUUUUCACUUUCCACUUUUCACUUUUCUUCGUACCUUUGCAGUCCGAUUAU
env-1088  AUAAUAUUAAAUCUUUAACUUAUCCAGUUCUUCGUUUCCUAGAUUCAAGAGAAAAACCGUACCUUUGCAGUCCGAUUAU
env-1089  AUAAUAUUAAAUCUUUAACUUAUCCAGUUUCUUGUUUUCAUGAUUCAAGAGAAAAGCCGUACCUUUGCAGUCCGAUUAU
env-1090  AUAAUCCUAAAAAUACACCUAUUUGCUUUCCUGACGUACAUUAUUAAGGAGAAAAGCCGUACCUUUGCAGUCCGAUUAU
env-1091  AAUAAUCAUAAAAAUUCUUUUCGUCGGACUGCAAAGGCGCGCACGUAUAAUAAAAUAUGUGUACCUUUGCAGUCCGAUUA
env-1092  AAAAUAUAAAUCUUUACUUUCAAUGGCUUUCUCGUGCCGUGAGAAAGUAGAAAAAGUAGUACCUUUGCAGUCCGAUUAU
env-1093  AAGUACGCCUUUUGCCCUCUUUUUUCACUUUUCACUUUUCACUUUUCAUUUUUUCGUCGUACCUUUGCAGUCCGAUUAU
env-1094  AAUAACACCAUUUUUACUUCCUUUAAGGCUCCUACUCUACUCAUUUCUCGUUUUUUAGCAGUACCUUUGCAGUCCGAUU
env-1095  AUUCAUAAAUCUUUGACUUCUGACAGGUUUCCUCGCGCGAUAUUAGAAAGAAAUAGCGUACCUUUGCAGUCCGAUUAUU
env-1096  AUAAUCAUAAAUCUUCCGUACGCCAGACUAAAAUAGCCUCGCGUACGGAAAAAUGUUCGUACCUUUGCAGUCCGAUUAU
env-1097  AAAUGCAAGCUUGAAGCCUAUUUAACAUUCCGCACCACACUUUUUUCAAUGAAAAACCGUACCUUUGCAGUCCGAUUAU
env-1098  AUAAUCAUAAAAGUUCGUGAAUAAAGAGGGUAAAGACACUCUAAAAUACAAAAUAUGUGUACCUUUGCACGCCGAUUAU
Psp-7-1   AAAAAUAUAAAUAUCUAUUUCUUGGACUGCAAAGGUGUGCAUGAAUAAAUAAAUAUGUGUACCUUUGCAGUCCGAUUAU
env-1099  UUUGUCUAAAUUUGACGCUUUUUUCUUUUGCGCGUACCUUAUAUAAUAAAAAUAGUUAGUACCUUUGCACACCGAUUUA
env-1100  AUUUGUCUAAAUUUGACGCUUUUUUCUUUUGCGCGUACCUUAUAUAAUAAAAAUAGUUAGUACCUUUGCACACCGAUUU
Psp-11-1  AUUUGUCUAAAUUUGACGCUUUUUUCUUUUGCGCGUACAUUAUAUAAUAAAAAUAGUUAGUACCUUUGCACACCGAUUU
Psp-19-1  AUUUCUCUAAAUUUGACGCUUUUUUCUUUUGCGCGUACCUUAUAUAAUAAAAAUAGUUAGUACCUUUGCACACCGAUUU
env-1101  AUUUCUCUAAAUUUAGCGCUUUUUUCUUUUGCGCGUACAUUAUAUAAUAAAAAUAGUUAGUACCUUUGCACACCGAUUU
Pru-1-1   AUUUGUCUAAAUUUGACGCUUUUUUCUUUUGCGCGUACCUUAUAUAAUAAAAAUAGUUAGUACCUUUGCACACCGAUUU
env-1102  AUAAUCAUAAAUAGUAAUAUCCAUAGCUGUCGCAGGCACGUAAAUUAAGAAAUAGUUCGUACCUUUGCAGUCCGAUUAU
env-1103  AAAUAUAAAUAACAGGGCGCUAAAAGGCAAAUAUAUCGUGUAAAACGCGAAAAAGCUGUACCUUUGCAGUCCGAUUAUU
env-1104  UAAUCAUAAAUACAGACUAUAAAUCCCAAUUCUCAAUUCUCAAUUCUUAAUUUAUUAGUACCUUUGCAGUCCGAUUAUU
Pbr-1-1   AACUAUAAUUUUAUGGUUACUGCAUGGAUGUAUGCUUCUUAAUUUCAAUUAAUGUGAGUACCUUUGCAGUCCGAUUAUU
Pbr-2-1   AACUAUAAUUUUAUGGUUGCUGCAUGGAUGUAUGCUUCUUAAUUUCAAUUAAUGUGAGUACCUUUGCAGUCCGAUUAUU
env-1105  AAAAUAAUUGCCGAGGUAAAAACGCGGCAAACCCUCUUACAGUCUCAAUAAAAAUAAUUACCUUUGCAGUCCGAUUAUU
env-1106  AAAACGUAAAUGUUAAGGUAGUGAGAGAUUCCAUCGCGCGAUAUAUGACGAAAAAGCAGUACCUUUGCAGUCCGAUUAU
env-1107  AAUCGUAAAAGUUGGGUCCUGGCUGGCUGCUGAUGGUCGAUGAAUGGCAGAAAAGUAGUACCUUUGCAGUCCGAUUAUU
env-1108  AAGCAUAAAUGUUUAGCUUCAAAAAUAUUAUAGGAGCCUGUAAAAGAAGAAAAAGUAGUACCUUUGCAGUCCGAUUUAG
env-1109  CUUUUAUAAAAAUACACUUUCCCGCUCGCUUCCGUAAGCGGAAUCAUCAUUUAAUUUGUACCUUUGCAGUCCGAUUUAA
env-1110  AAUCAUAAAUCUUAUGCCGUUUGCUGUUCUCGGUUUCUCGUUUUCCAUUUAAACUGCGUACCUUUGCAGUCCGAUUUUG
env-1111  AAUCAUAAUUUGAUGGUCUUUUCUCCUUUCUCAUUACUUCUUUCUCAUAUUAUCUCACUACCUUUGCAGUCCGAUUUUU
env-1112  AUCAUAAAAGUUGCGUCCUAAUAGCUGCGUUAUACCUCAUAUAAUGAGAAAAAGCAGUACCUUUGCAGUCCGAUUAUUG
env-1113  AAGUGCAGUUUUGCGCUCGCGCGAUACCCCUUAAAUGGAAAAAUAUGUGUAAUUUUGCACUCCGAUUUCCGAUUAUCGC
env-1114  UAUGCGGUUGCUUAGCUGGCAUGAUAGCCUCUAAGUAAAAAAAUAUGUGUAAUUUUGCACUCCGAUUUCCGAUUAUCGC
env-1115  ACUGCACUAAUGAAGGUCAUAGCAUAGCUACUAUGUCAAUAAAUAUGUGUAAUUUUGCACUCCGAUUUCCGAUUAUCGC
env-1116  AUAAAUUAGACAGCAAACACAGCAACGGAAACAACAUAUUCCAUAUAAAAUAUGUACUUUUGCAGUCCGAUUAUUAAAA
env-1117  AUACGUAAAAGAUGAAACAAAAUGUGUCGCGCGUAUAUUAAAUAAUGAGAAAAGCCGUAACUUUGCAGUCCGAUUAUUU
env-1118  UUUCUUAAAAAUACAGACUGCAAGGAAACUCAUUGCACAGAAGUGCGGAAAAAUGAGUACCUUUGCAGUCCGAUUAUUA
env-1119  UUUCUUAAAAAUACGGACUGCAAGGAAACUCAUUGCACAGAAGUGCGGAAAAAUGAGUACCUUUGCAGUCCGAUUAUUA
env-1120  AAAACGUAAAUCUGAUGAUUUCAUAAAAGUUCACGCGCGAUAUUUAAAAUAAAAUGCUUACCUUUGCAGUCCGAAAUCA

          ...............................................................................
          ...............................................................................
          ●●●●●●●●●●●●●●●●●●●●●●●●●●●●●●●●●●●●●●●●●●●●●●●●●R●●●●●●●●●●Y●Y●●●●●●●●●●●●●Y●●
```

```
env-1122    AAAACGUAAUCUGAUGAUUUCAUAAAAGUUCACGCGCGAAUAUUUAAAAAUAAAUGCUUACCUUUGCAGUCCGAAAUCA
env-1123    CAGCCAAGCAAACAGUCAGAAAAUCAUCAAAUUGUUUUCUUGUUUCGAAAAAGUAUCUACCUUUGCAGUCCGAUUAUUA
env-1125    AAAACAUAAAAGUUGCGCUUUCGCUCGCCGUGUCAAUCGCAAUGCGAAGAAAAAUCAGUACCUUUGCAGUCCGAUUAUU
env-1126    AAAACGUAAAUCUUAAUUUGUUUGCCCCUCUUUGGAUUGGUAAACGAAGAAAAAGCCGUACCUUUGCAGUCCGAUUAUU
env-1127    AAAACGUAAAUCUCACACUGUUUGCCCCUCUUUGGGUGGUUAAACGGAGAAAAAUGAGUACCUUUGCAGUCCGAUUAUU
env-1129    UGCAUAAAUAUUAUAGCAGAAUAGGGUGCAUGUUACGCAUAUUAUAAAAAUAAUGUGUAAUUUUGCAGUCCGAUUAUUU
env-1130    UUUGCUGAGGGAAUGAAAAAUUUUAAAUAUAUUUGGUGUAUUCGGUGAUUUUUCGUACCUUUGCAAGCCGAUUAUUAUC
env-1131    GUAUACUCGCGGUUGAAAAUUUGGAAAAGUUUUGUUAGUUACGCUUUUUUUCGUACCUUUGCACGCCGAUUAUUAUCA
env-1132    UGUAUGCAUGCGCGAAAAAAACAAGGAAAGUUUUGCCAGUUAAAAGAAUUUUCGUACCUUUGCACGCCGAUUAUUAUCA
env-1133    AUGCCAAGAAGAAAAAAAGUCUUGCCUGUGCUUGCAGAAUUGGAAAAAGUUGUACCUUUGCACGCCGAUUAUUAUCAA
env-1134    GUGGUUCGGAGACAAAAAAGAAAGGAAACUUUUGGUCAGUUAAUAUAAAAAUCGUACCUUUGCAAGCCGAUUAUUAUCA
env-1135    CCUGAAAAUUAUUUUUUUCAGUGGAAAUGCUUGCCGUUUCCGGAAAAAGUCGUACCUUUGCACGCCGAUUAUUGUCUAC
env-1136    CAAUAAUUAAUAAAAAAAGCAAGGAGAUGCUUGUCGGUUCAGAAAAAAGUUGUACCUUUGCACGCCGAUUAUUGUCUAA
env-1137    UCAACGAUUCCAUAUGAAAAAUAGGAGGAAGUUUGGCUGUUUCAGUAGAAAUGCGUAAUUUUGCAUGCCGAUUAUUAUC
env-1138    UCGACGAUUCUAUAUGAAAAAUAGGAGAGAGUUUGGCUGUUUCAGUAGAAAUGCGUAAUUUUGCAUGCCGAUUAUUAUC
env-1139    UCAACGAUUCCAUAUGAAAAAUAGGAGGAAGUUUGGCUGUUUCAGUAGAAAUGCGUAAUUUUGCAUGCCGAUUAUUAUC
env-1140    UCGACGAUUCUAUAUGAAAAAUAGGAGGAAGUUUGGCUGUUUCAGUAGAAAUGCGUAAUUUUGCAUGCCGAUUAUUAUC
env-1141    .......................................................GUAAUUUUGCAUGCCGAUUAUUAUC
env-1143    UCGACGAUUCUAUAUGAAAAAUAGGAGGAAGUUUGGCUGUUUCAGUAGAAAUGCGUAAUUUUGCAUGCCGAUUAUUAUC
env-1144    UCGACGAUUCUAUAUGAAAAAUAGGAGGAAGUUUGGCUGUUUCAGUAGAAAUGCGUAAUUUUGCAUGCCGAUUAUUAUC
env-1145    UCGACGAUUCUAUAUGAAAAAUAGGAGGAAGUUUGGCUGUUUCAGUAGAAAUGCGUAAUUUUGCAUGCCGAUUAUUAUC
env-1147    CGCCGACAUGCGCUUUUUGCCCUCACCUGCUUGGCUAUUUCGCAGAAUAUAAGUACAUUUGCAUGCCGAUUAUUAUCAA
env-1148    CGCCGACAUGCGCUUUUCGCCCUCACCUGCUUGGCUAUUUCGCAGAAUAUAAGUACAUUUGCAUGCCGAUUAUUAUCAA
env-1149    CGCCGACAUGCGCUUUUCGCCCUCACCUGCUUGGCUAUUUCGCAGAAUAUAAGUACAUUUGCAUGCCGAUUAUUAUCAA
env-1151    .......CUGCGCUUUUCGCCCUCACCUUCUUGGCUAUUUCGCAGAAUAUAAGUACAUUUGCAUGCCGAUUAUUAUCAA
env-1153    CGUCGACCUUCGCUUUUCGCCCUCACCGUCUUGGCUAUUUCGCAGAAUAUAAGUACAUUUGCAUGCCGAUUAUUAUCAA
env-1154    CGUCGACCUGCGCUUUUCGCCCUCACGUUCUUGGCUAUUUCGCAGAAUAUAAGUACAUUUGCAUGCCGAUUAUUAUCAA
env-1155    CGUCGACCUGCGCUUUUCGCCCUCACCUUCUUGGCUAUUUCGCAGAAUAUAAGUACAUUUGCAUGCCGAUUAUUAUCAA
env-1156    CAUGAAAAAAAGAUUUUCAAGUUCACAAGCUUGGCUAUUUCGCAGAAUAUAAGUACAUUUGCAUGCCGAUUAUUAUCAA
env-1157    CGUCGACCUGCGCUUUUCGCCCUCACCUUCUUGGCUAUUUCGCAGAAUAUAAGUACAUUUGCAUGCCGAUUAUUAUCAA
env-1158    CGUCGACCUGCGCUUUUCGCCCUCACCUUCUUGGCUAUUUCGCAGAAUAUAAGUACAUUUGCAUGCCGAUUAUUAUCAA
env-1159    CGCCGACAUGCGCUUUUCGCCCUCACCUGCUUGGCUAUUUCGCAGAAUAUAAGUACAUUUGCAUGCCGAUUAUUAUCAA
env-1163    CGCCGACCUGCGCUUUUCGCCCUCACCUUCUUGGCUAUUUCGCAGAAAAUAAGUACAUUUGCAUGCCGAUUAUUAUCAA
env-1164    CAUGAAAAGAAGAUUUUCAAGUUCACAAGCUUGGCUAUUUCGCAGAAUAUAAGUACAUUUGCAUGCCGAUUAUUAUCAA
env-1165    CGUCGACCAGCGCUUUUCGCCCUCACCUUCUUGGCUAUUUCGCAGAAUAUAAGUACAUUUGCAUGCCGAUUAUUAUCAA
env-1166    CGUCGACCUGCGCUUUUCGUCCUCACCUUCUUGGCUAUUUCGCAGAAUAUAAGUACAUUUGCAUGCCGAUUAUUAUCAA
env-1167    .......................................................ACAUUUGCAUGCCGAUUAUUAUCAA
env-1192    CAUGAAAAGAAGAUUUUCAAGUUCACAAGCUUGGCUAUUUCGCAGAAUAUAAGUACAUUUGCAUGCCGAUUAUUAUCAA
env-1193    UAAGUUUUGCAUUUAUAGAAGGAAAUGUUUGGUAGAUUCGUGGAAAAUAAGUAAUUUUGCAUGCCGAUUAUUAUCAAGU
env-1194    AAGUUUUGCAUUUAUAGAGGGAAAUGUUUGGUAGAUUCGUGGAAAAUAAGUAAUUUUGCAUGCCGAUUAUUAUCAAGUU
Psp-1-1     AAGUUUUGCAUUUAUAGAGGGAAAUGUUUGGUAGAUUCGUGGAAAAUAAGUAAUUUUGCAUGCCGAUUAUUAUCAAGUU
env-1195    AAGUUUUGCAUUUAUAGAGGGAAAUGUUUGGUAGAUUCGUGGAAAAUAAGUAAUUUUGCAUGCCGAUUAUUAUCAAGUU
env-1197    AAGUUUUGCAUUUAUAGAGGGAAAUGUUUGGUAGAUUCGUGGAAAAUAAGUAAUUUUGCAUGCCGAUUAUUAUCAAGUU
env-1203    AAGUUUUGCAUUUAUAGAAGGAAAUGUUUGGUAGAUUCGUGGAAAAUAAGUAAUUUUGCAUGCCGAUUAUUAUCAAGUU
env-1205    AAGUUUUGCAUUUAUGGAAGGAAAUGUUUGGUAGAUUCGUGGAAAAUAAGUAAUUUUGCAUGCCGAUUAUUAUCAAGUU
env-1206    ........................................................UUUUGCAUGCCGAUUAUUAUCAAG
env-1211    CUAAGUUUUGCAUUUAUAGAAGGAAAUGUUUGGUAGAUUCGUGGAAAAUAAGUAAUUUUGCAUGCCGAUUAUUAUCAAG
env-1213    GGGGGAGAAGUUGCUUUUCUCCGAGAAAUGUUUGGUGGUAUCACGAGAAAUAAGUAAUUUUGCAUGCCGAUUAUUAUCA
env-1216    GGCGUUUGCAGUGCUUUUCUCCGAGAAAUGUUUGGUGGAAUCAUGAGAAAUGAGUAAUUUUGCGUGCCGAUUAUUAUCA
env-1223    ...................UCUGAGAAAUGUUUGGUGGAAUCAUGAGAAAUGAGUAAUUUUGCGUGCCGAUUAUUAUCA
env-1224    GGCGUUUGCAGUGCUUUUCUCUGAGAAAUGUUUGGUGGAAUCAUGAGAAAUGAGUAAUUUUGCGUGCCGAUUAUUAUCA
env-1228    GUGUGAUAGUGUGCUUUUCUCUGAGAAAUGUUUGGUGGUAUCAUGAGAAAUGAGUAAUUUUGCGUGCCGAUUAUUAUCA
env-1233    GGCGUUUGCAGUGCUUUUCUCUGAGAAAUGUUUGGUGGAAUCAUGAGAAAUGAGUAAUUUUGCGUGCCGAUUAUUAUCA
env-1243    GGCGUUUGCAGUGCUUUUCUCCGAGAAAUGUUUGGUGGAAUCAUGAGAAAUGAGUAAUUUUGCGUGCCGAUUAUUAUCA
env-1244    GGGUGUUAUAGUGCUUUUCUCUGAGAAAUGUUUGGUGGAAUCAUGAGAAAUGAGUAAUUUUGCAUGCCGAUUAUUAUCA
env-1245    GGGUGUUAUAGUGCUUUUCUCUGAGAAAUGUUUGGUGGAAUCAUGAGAAAUGAGUAAUUUUGCAUGCCGAUUAUUAUCA
env-1250    GUGUGAUAGUGUGCUUUUCUCUGAGAAAUGUUUGGUGGAAUCAUGAGAAAUGAGUAAUUUUGCGUGCCGAUUAUUAUCA
env-1251    GUGUGAUAGUGUGCUUUUCUCUGAGAAAUGUUUGGUGGUAUCAUGAGAAAUGAGUAAUUUUGCGUGCCGAUUAUUAUCA
env-1254    .............................................GAGAAAUGAGUAAUUUUGCGUGCCGAUUAUUAUCA
env-1256    GGCGUUUGCAGUGCUUUUCUCCGAGAAAUGUUUGGUGGUGUCACGAGAAAUAAGUAAUUUUGCAUGCCGAUUAUUAUCA
env-1258    GGGUAUUCUAGUGCUUUUCUAUGAGAAAAGUUUGGCAGAAUCAGGAGAAAUGAGUAAUUUUGCGUGCCGAUUAUUAUCA
env-1259    GGGUGUUAUAGUGCUUUUCUCUGAGAAAAGUUUGGCAGAAUCAGGAGAAAUGAGUAAUUUUGCGUGCCGAUUAUUAUCA
Pmu-2-1     UUAUUUAUAAUUAAACCAAAUAAUAAUAAUUCAUUCUUUUCCGCAUCAACUGAUAUGUGUACCUUUGCACUGCUGAUUG
Pde-1-1     CUUAAUCCAUGGGCGUUUUCACCGUUUUGCCGUGCGUUGUACGUCAUAAAAUAUGUAAUUUUGCAGAACCGAUUAUUUC
            ................................................................................
            ................................................................................
            ●●●●●●●●●●●●●●●●●●●●●●●●●●●●●●●●●●●●●●●●●●●●●●●●●R●●●●●●●●●●Y●Y●●●●●●●●●●●●●Y●●
```

alignment positions 80 · · · 259

```
env-1       CAAGGGU.......................ACCCUUAAUGCCCAACCAAGAGUUAAUAGUU..AC..GUCC.....UAUUUUGGCCUGGACG.UAAUGGUUGGUAAGCUCUUGGAAAGAUAAACAUUAGUAGUUACAAUUUAAUUCAAACAAAAGUGGACACUUUAAGUUACAAGACCA
env-2       AGGGAGGCGC..AGAU...........GAUCCUCCC....AAAGUGGGCGUGUAGAUAGCG..CGU.UGUU..........UUGGCC.GACAC.GUGUGGUCUGUG.AAUCGCUCAGAGAAAAAACUACAUCUCCAUCGUGGCCGCUCUCCUUUGCGAGGUGGUGUACGAUAGUGGAGUG
env-3       AUCUGGC.....CGGCCAAAA.........GCCAGC.....CACGCCGCGGAUUAAUAGUU..GC..AUCC....GUUUUAUUGGCCAGGAUG.UAAUGGUUAGGU.AAUAUUCGUGGCAAAAGAUAAACAUUAGUAGUUACAAUUUAAAACAAACAAAAGUGGACACUUUAAGUUACAA
env-4       GAGAGAGUC...AUUA............G.UUCUCU....CAAAAGGGUGUGUAGAUAGCGU..G..AACU..........UUGGCC.GGUUC..UGUGGUCUGUG.AAUCACUCAAAUAAACUAAUCUCCGCCGCUAAUCCCGAAAGGGAGAACGGUAAGGGAGUGAUUUUUCGUGAUG
env-5       UGGGGCGACACUCUU..........AGAAUCGCUCC....AGCAUGAGCGUGUUAAUAGUG..UC..GCGU..........AAGGCC.AUGCG.UCAUGGUUAGGG.AAUCGCCCAUGCGUUCAGGGUUAAAGCCAUGGAUGGUCUCUUUUAGACUGGAGGCACCGCGAGGCCCCUGUUC
env-6       GGGAUGC.....GAAAA.............GCGUCC....UCUACAAGUGUGUAGAUAGCGU.CC..AUA...........UUGGCC..UAUG.GCGUGGUCUGUG.AAUCACUUGUGAAAAUGAAGAAUUGAACAAUUAAUUAGUAGUAACAAAAUCAAGAAUUUAAAGAUGAACACUU
env-7       AGGGGACGC...GAAA............GUG.CCCC....CAUAUAAGUGUGUAGAUAGCGU.CC..AUA...........UUGGCC..UGUG.GCGUGGUCUGUG.AAUCACUUGUAAAAAAACAAGAAUUGAACAAUUAAUUAGUAGUAACAAAAAUUUUAGAGAUGAACACUUUAAG
env-8       AACAGGC.....AACAAAAA..........GCCUGU....UAAUCCCACGUGUUAAUAGCU..GU..AUCUAUUAAAGAAUUUGGCC.UGAUG.CAGUGGUUAGUG.AAUCGUGGUGCGUCGUCAUGCGGCGCAAAAAAACUUUAUGGC...............................
env-9       AACAGGC.....AACAAAAA..........GCCUGU....UAAUCCCACGUGUUAAUAGCU..GU..AUCUAUUAAAGAAUUUGGCC.UGAUG.CAGUGGUUAGUG.AAUCGUGGUGCGUCAUCAUGCGGCGCAAAAAAAAACUUUAUGGCAAACCCGAAGAAAAACUUAGAACGGAAGG
env-10      AACAGGC.....AACAAAAA..........GCCUGU....UAAUCCCACGUGUUAAUAGCU..GU..AUCUAUUAAAGAAUUUGGCC.UGAUG.CAGUGGUUAGUG.AAUCGUGGUGCGUCGUCAUGCGGCGCAAAAAAACUUUAUGGCAAACCCGAAGAAAAACUUAGAACGGAAGGCAA

            .<<<<<<<<<<<.............>>>>>>>>>>>................<<<<<.<<<..<<..<<<<<...............>>>>>>.>>>>.>>>>>.......................................................................................
            .222222.1111.............1111.222222................02200.122..22..2222................22222.2221.00220.......................................................................................
            ●GGGG●○-----R●●●●○○○----------○●CCCC----●●●●●●●●YGUGU●AAUAGY●--●Y--RYYU----------UUGGCC-GRRY●-●●RYGGUU●GUG-AAUCR●●●●●●●RR●●R●●●●●●●●●●●●●●●●●●●●●●●●●●●●●●●●●●●●●●●●●●●●●●●●●●●●●●●●
             5′                                                                                                    3′
```

```
env-11    AGGGCAC.....AAAU..............GUGCCU....UCGCGACUCGUGUGGAUAGCC..U.A.UCCU..........UUGGCC.AGGA..AGACGGUCCGUG.AAUCGUGUCGAGUGAGAGUAAAGUAACAAACAGAAUCAAGAGUAAAAUCAAGUUAGAAUGAAUACUUUAAGUU
env-12    AAGACU......GUAAU..............GGUCU...AAAAUUCUGUGCGUUAAUAGUC..UU..GCCU..........UUGGCC.GGGCA..GAUGGUUAGGA.ACACGGAAAAAGAAAAAAGUUUUUAGUAGUAAUAAUUAAAAGAAACAAAAUGGACACUUUAAGUUACAAGACC
env-13    GACCCCCUC...GCAA............GAGGAGGU....UAAAACUGUGUGUUAAUAGUU..GCU.GUCC.......UUUUUGGCCUGGGCA.CAAUGGUUAGUG.AAUCGCAGUAUAAAACAGAGAAACAAUAAAAACUU......................................
env-14    AGUGGGCC....UUUG.............GGCCUGC.......GUCUGCGUGUUAAUAGUCU.UU..CCCG..........UUGGCC.CGGGG.AGAUGGUUAGUG.AAUCGCAAAGAAAAAGUUUUUUAGUAGUAAAAUUUUAAAAAGUCAAACAAGAGUGGACACUUUAAGUUACAAG
env-15    GAUGGGGAGU..UCUU...........AGACCCCAU......GGAUGUCGUGUUAAUAGCC..GU..GCUU..........UUGGCC.GAGUG.CGGUGGUUAGUG.AAUCGGCACCCAGCGAGACGUAUCGUAUUUACCGGCGAGUGCUUUCCAAGACUAAGAAGCAUUGUCUCGAUAG
env-16    AGAGCC......CCUUAAAA...........GGCUC....AUUAUCUGCGUGUUAAUAGCUU.UU..CUCA..........UUGGCC.UGGGA.AAGUGGUUAGUG.AAUCGCAGAAACAAACAGCGCGCUGGUGAGAGCCCAGCAGAAAGAAACAUUAGUAGUACAACAAAAACAAGUU
env-17    AAAGACU.....GUAAG.............AGUCUU....AAAAUCUGUGUGUUAAUAGCA..UU..UUCA..........UUGGCC.UGGA..AUGCGGUUAGUG.AAUCACAGAAGCAAGAAAUUAAAGUUUUUUUAGUAGUAACAAUUAUUAUUUUAAAAUUUAGAAUGGAUACUUU
env-18    AAAGAC......GAUAAAA............GUCUU....AGAACCUGCGUGUUAAUAGCU..CU..UUCA..........UUGGCC.UGGAA.GGGCGGUUAGGG.AAUCGCAGAAAAGGAAAAUUAAAGUUUUUUUAGUAGUAACAAUUUUAUUUUUAAAUUAAAGAAUGGAUACUUU
env-21    AAAGAC......GAUAAAA............GUCUU....AGAACCUGCGUGUUAAUAGCU..CU..UUCA..........UUGGCC.UGAAA.GGGUGGUUAGGG.AAUCGCAGAAAAAGGAAUUUAAAGUUUUUUUAGUAGUAACAAUUUUAUUUUAAAUUAAAGAAUGAAUACUUUA
env-22    AAAGAC......GAUAAAA............GUCUU....AGAACCUGCGUGUUAAUAGCU..CU..UUCA..........UUGGCC.UGAAA.GGGUGGUUAGGG.AAUCGCAGAAAAAGGAAUUUAAAGUUUUUUUAGUAGUAACAAUUUUAUUUUUAAAUUAAAGAAUGAAUACUUU
env-26    AAAGAC......GAUAAAA............GUCUU....AGAACCUGCGUGUUAAUAGCU..CU..UUCA..........UUGGCC.UGGAA.GGGUGGUUAGGG.AAUCGCAGAAAAAGGAAUUUAAAGUUUUUUUAGUAGUAACAAUUUUAUUUUGAAUUAAAGAAUGAAUACUUUA
env-27    AAAGAC......GAUAAAA............GUCUU....AGAACCUGCGUGUUAAUAGCU..CU..UUCA..........UUGGCC.UGGAA.GGGUGGUUAGGG.AAUCGCAGAAAAAGGAAUUUAAAGUUUUUUUAGUAGUAACAAUUUUAUUUUUAAAUUAAAGAAUGAAUACUUU
env-63    AAAGAC......GAUAAAA............GUCUU....AGAACCUGCGUGUUAAUAGCU..CU..UUCA..........UUGGCC.UGGAA.GGGUGGUUAGGG.AAUCGCAGAAAAAGGAAUUUAAAGUUUUUUUAGUAGUAACAAUUUUAUUUUUAAAUUAAAGAAUGAAUACUUU
env-64    AAAGAC......GAUAAAA............GUCUU....AGAACCUGCGUGUUAAUAGCU..CU..UUCA..........UUGGCC.UGGAA.GGGUGGUUAGGG.AAUCGCAGAAAAAGGAAUUUAAAGUUUUUUUAGUAGUAACAA...............................
env-70    CAGAGUCACC..CUUAA..........GGUG.CUCU.....UUUUCUGCGUGUUAAUAGCUU.UU..CUCA..........UUGGCC.UGUGA.AAGCGGUUAGUG.AAUCGCAGAAUGCAAAAAAGCGUGCCGGUGCAAGCCCGGCUAAAGAGACACAUUAAUAAUAUUAGUAGUAACA
env-71    AAGCCC......UUAAU..............GGGCU...CUCUUUCUGCGUGUUAAUAGCUU.UU..CUC...........UUGGCCU.GGGA.AAGCGGUUAGUG.AAUCGCAGUACAGACGAAAAAGCGUGCCGGUGGAAGCCCGGCAGUAGAAACAUUAGUAGUAACAAACAAGAAG
env-72    AAGCCC......GUAAU..............GGGCU...CGCAUUCUGCGUGUUAAUAGCGU.UU..UCCG..........AUGGCC.UGGGA.GCGUGGUUAGUG.AAUCGCAAAUGCACACCAUCGCCGCCCCCGUGGGCGGCAGCAAACAACAAUCAGUAGUAAAAAACAACUUAGU
env-73    CAGGGUGUG...GUGUAA..........CACACUCU.....AAAUCUGCGUGUUAAUAGCUU.UU..CUCA..........UUGGCC.UGGGA.GAGCGGUUAGUG.AAUCGCAGACCGACAAAAGCGCGCCGGUGCAAGCCCGGCGGUGUGACACAUUAAUUAUUUAUAGUAGUAACAA
env-74    GAGAGUC.....CGCUAAAA..........GACUCU.....GUAUCUGCGUGUUAAUAGCG..UA..UCCU..........AUGGCCUGGGA..ACGUGGUUAGUG.AAUCGCAGAAGUGACACCGCGAGGGCUCGUGAAGCCUAAAAGUCGGGGCGCGGCCCGAAAAGAACUUAGUAGU
env-75    AUAGGC......AAUAAAA............GCCUA....UUAAUCUGCAUGUUAAUAGUC..UG..CUCU..........UUGGCC.GGA.C.AGACGGUUAGUG.AGUGCAGAUAAUGUAUAAUUAAUUUUUAGUAGUAACAAUUUAUUAUUAGUUUUAAGAAUGGAUACUUUAAGUU
env-76    GAUAGGC.....AUUAAAC...........GCCUAU.....UAAUCUGCAUGUUAAUAGUC..UG..CUCU..........UUGGCC.GGA.C.AGACGGUUAGUG.AGUGCAGAUCAUGAAUAAUUAUUUUUAGUAGUAACAAUUUUUUAUUAUUAGUUUUAAGAAUGGAUACUUUAAG
env-77    AAGGGGCA....AAUUG............UGCCCCU......GUAGUGCGUGUGAAUAGGG..UG..UUCU..........UUGGCC.AGAC..AUCUGGUUCGUG.AAUCGCACAUGAGGAUGAAUGCCUGACCCGUCUGGCGAGAGAAAAGAAGACGUGGAAGCAGGAAGCCUCGGAA
env-78    AAGGGCC.....CCCAGAAA..........GGCCCU.....CUCGGAGCGUGUGAAUAGUC...C..GUCU........CAUUGGCCUGAACG..GACGGUUCGGG.AAUCGCACCGACAAAAAGAGAAAUAAAACAAGGUAGAAACAAAAACAAAAGAGACAAAGAAUGGAUACUUUGA
env-79    GAGGAGA.....GCGAAA............UCUCCU....CUGCGCUGUGUGUUAAUAGUU..GG..GCUU..........UUGGUC.GAGCC.UAAUGGUUAGUG.AAUCACGGCAUUAAGACAAAAAUAAAAGUAGUAACAAACAACAUCUAAAAACAAAAAUGGAGACUUUGAGUUU
env-80    CGAGGAG.....CGUCAGAAA.........CUCCUC......AAACCGUGUGUUAAUAGUU..GU..GUCU..........UUGGUC.GGGCG.CAACGGUUAGGG.AAUCACGGAAUUAAACGAAAGAAAAUAAUUAGUAGUAACAACAACAAAAACAAACAAUGGAGACUUUAAGUUU
Pbr-3-1   UGAAGAUGC...AUCUUA..........GAGUCUUC..ACAGCUUUCUUGUGUUAAUCGUG..UU..AUCU..........UUGGCC.GGGUA.UCACUGUUAGUG.AAUCAGGAAAGCGGCAGAGAGAAUGCUGUGGAUGGUCUCUUUUAGACUGGAGGCGCCGCGAGGUCUCUGCUCG
env-81    UGAAGAUGC...AUCUUA..........GAGUCUUC..ACAGCUUUCUUGUGUUAAUCGUG..UU..AUCU..........UUGGCC.GGGUA.UCACUGUUAGUG.AAUCAAGAAAGCGGCAGAGAGAAUGCUGUGGAUGGUCUCUUUUAGACUGGAGGCGCCGCGAGGUCUCUGCUCG
env-82    GGGGAGGUUC..UUA............GAAUC.CCC....GCGGGUGACGUGUAAAUAGCGU.UU..GUCU..........UUGGCC.GGACU.ACGUGGUUUGUG.AAUCGUCAUUCAAACAAUUGUGAAUGCGGCAGUGCCGCAAAAUUAAAAAACUGUUUUUUAGUAAAAUUAAUUU
env-83    AGGGGACAU...GAAA............AUG.CCCC....CAAAUCAGUGUGUGAAUAGCG..AAA.UCCU.........AUUAGCC.GGGA..ACGCGGUUCGUG.AAGCACUGAAGCGUGACGGACAAGAAGACGUCUCGCGAUUAAAACCACAGGCAACUGUGGACAGAGAUUAAUA
env-84    AGCAGGC.....AGAAA.............ACCUGC......AAAGCGCGUGUUAAUAGUA..AC..GCC.......AAUUUUGGCC.GGGCG.UUAUGGUUAGUG.AAUCGCGAUUAAAGCUAUGAACGCAUUAAUUAUCAAAUGCAUUCAUGCAGUUCGAAAGACGGUGCAAAAAUCG
env-86    AGCAGGC.....AGAAA.............GCCUGC......AAAGCGCGUGUUAAUAGUA..AC..GCC.......AAUUUUGGCC.GGGCG.UUAUGGUUAGUG.AAUCGCGAUUAAAGCUAUGAACGCAUUAAUUAUCAAAUGCAUUCAUGCGGUUCGAAAGACGGUGCAAAAAUCG
env-87    AGUACCCC....CAAA.............GGGGUGC....AAAAGAGGCGUGUGAAUAGUC..C...GCAU........CAUUGGCCUGUAC..GGACGGUUCGUG.AAUCGCUUCGACAACAUUGAGAAAUAAACAAGGUAGUAACAAAACAAAAAGUUUAGACAAAGAAUGGAUACUU
Pal-1-1   UGAAGGU..CUACGCU.........UAGAAGCCUUC...GCGCAGAGCCGUGUUAAUAGUA..CU..UCUU..........CUGGCC.GAGAA.GUGCGGUUAGUG.AAUCGGUGAAGCUACAGAGAGUAAUGCUGUGGAUGGUCUCUUUUAGACUGGAGGCGCCGCGAGGUCUCUGUAA
env-88    AUGUGG......CUCAUUUGCAA........CCACA........CUCGCGUGUUAAUAGCC..GG..GUUU........CAUUGGCCGAAACA.CGGUGGUUAGUG.AAUCGCGAGAACGAUAACUUAAUUGAAAUAGGAGCUCGCUCCUUAGUAACUACAAAACUACAUCAAAAAGAAU
env-98    AUGUGGC.....UCAUUUGCA.........GCCACA........CUCGCGUGUUAAUAGCC..GG..GUUU........CAUUGGCCGAAACA.CGGUGGUUAGUG.AAUCGCGAGAACGAUAACUUAAUUGAAAUAGGAGCUCGCUCCUUAGUAACUACAAAACUACAUCAAAAAGAAU
env-126   AUGUGGC.....UCAUUUGCA.........GCCACA........CUCGCGUGUUAAUAGCC..GG..GUUU........CAUUGGCCGAAAAA.CGGUGGUUAGUG.AAUCGCGAGAACGAUAACUUAAUUGAAAUAGGAGCUCGCUCCUUAGUAACUACAAAACUACAUCAAAAAGAAU
env-129   AUGUGGC.....UCAUUUGCA.........GCCACA........CUCGCGUGUUAAUAGCC..GG..GUUU........CAUUGGCCGAAAAA.CGGUGGUUAGUG.AAUCGCGAGAACGAUAACUUAAUUGAAAUAGGAGCUCGCUCCUUAGUAACUACAAAACUACAUCAAGAAGAAU
env-132   AUGUGGC.....UCAUUUGCA.........GCCACA........CUCGCGUGUUAAUAGCC..GG..GUUU........CAUUGGCCGAAAAA.CGGUGGUUAGUG.AAUCGCGAGAACGAUAACUUAAUUGAAAUAGGAGCUCGCUCCUUAGUAACUACAAAACUACAUCAAAAAGAAU
env-148   AUGUGGC.....UCAUUUGCA.........GCCACA........CUCGCGUGUUAAUAGCC..GG..GUUU........CAUUGGCCGAAAAA.CGGUGGUUAGUG.AAUCGCGAGAACGAUAACUUAAUUGAAAUAGGAGCUCGCUCCUUAGUAACUACAAAACUACAUUAAAAAGAAU
env-155   AUGUGGC.....UCAUUUGCA.........GCCACA........CUCGCGUGUUAAUAGCC..GG..GUUU........CAUUGGCCGAAAAA.CGGUGGUUAGUG.AAUCGCGAGAACGAUAACUUAAUUGAAACAGGAGCUCGCUCCUUAGUAACUACAAAACUACAUCAAAAAGAAU
env-157   AUGUGGC.....UCAUUUGCA.........GCCACA........CUCGCGUGUUAAUAGCC..GG..GUUU........CAUUGGCCGAAAAA.CGGUGGUUAGUG.AAUCGCGAGAACGAUAACUUAAUUGAAAUAGGAGCUCGCUCCUUAGUAACUACAAAACUACAUCAAAAAAGAA
env-158   CGGGG...CUA.AACU..........UAGUAACCCC....UGUGUAGAAGUGUUAAUAGUU..GAA.ACUU..........AAGGCC.AGGCU.CAACGGUUAGUG.AAUCGACUGCACA............................................................
env-159   CGGGG...CUA.AACU..........UAGUAACCCC....UGUGUAGAAGUGUUAAUAGUU..GAA.ACUU..........AAGGCC.AGGCU.CAACGGUUAGUG.AAUCGACUGCACAAAAGAAUUAAAAACGUUUUUUAGUAGUAAUAUUUAAUCAAAAGAAUGAACACUUUAAGUU
env-160   CGGGG...CUA.AACU..........UAGUAACCCC....UGUGUAGAAGUGUUAAUAGUUG.AU..GUUU..........AAGGCC.AGACA.CAACGGUUAGUG.AAUCGACUGCACAAAAGAAUUAAAAACGUUUUUUAGUAGUAAUAUUUAAUCAAAAGAAUGAACACUUUAAGUU
env-163   CGGGG...CUA.AACU..........UAGUAACCCC....UGUGUAGAAGUGUUAAUAGUUG.AU..GUUU..........AAGGCC.AGACA.CAACGGUUAGUG.AAUCGACUGCACAAAAGAAUUAAAAACGUUUUUUAGUAGUAAUAUUUAAUCAAAAGAAUGAACACUUUAAGUU
env-164   CGGGG...CUA.AACU..........UAGUAACCCC....UGUGUAGAAGUGUUAAUAGUUG.AA..GCUU..........AAGGCC.AGGCA.CAACGGUUAGUG.AAUCGACUGCACAAAAGAAUUAAAAACGUUUUUUAGUAGUAAUAUUUAAUCAAAAGAAUGAACACUUUAAGUU
env-165   CGGGG...CUA.AACU..........UAGUAACCCC....UGUGUAGAAGUGUUAAUAGUUG.AA..GCUU..........AAGGCC.AGGCU.CAACGGUUAGUG.AAUCGACUGCACAAAAGAAUUAAAAACGUUUUUUAGUAGUAAUAUUUAAUCAAAAGAAUGAACACUUUAAGUU
env-166   CGGGG...CUA.AACU..........UAGUAACCCC....UGUGUAGAAGUGUUAAUAGUUG.AA..GCUU..........AAGGCC.AGGCU.CAACGGUUAGUG.AAUCGACUGCACAAAAGAAUUAAAAACGUUUUUUAGUAGUAAUAUUUAAUCAAAAGAAUGAACACUUUAAGUU
env-167   CGGGG...CUA.AACU..........UAGUAACCCC....UGUGUAGAAGUGUUAAUAGUUG.AA..GCUU..........AAGGCC.AGGCU.CAACGGUUAGUG.AAUCGACUGCACAAAAGAAUUAAAAACGUUUUUUAGUAGUAAUAUUUAAUCAAAAGAAUGAACACUUUAAGUU
env-168   CGGGG...CUA.AACU..........UAGUAACCCC....UGUGUAGAAGUGUUAAUAGUUG.AA..GCUU..........AAGGCC.AGGCU.CAACGGUUAGUG.AAUCGACUGCACAAAAGAAUUAAAAACGUUUUUUAGUAGUAAUAUUUAAUCAAAAGAAUGAACACUUUAAGUU
env-169   CGGGG...CUA.AACU..........UAGUAACCCC....UGUGUAGAAGUGUUAAUAGUUG.AA..GCUU..........AAGGCC.AGGCU.CAACGGUUAGUG.AAUCGACUGCACAAAAGAAUUAAAAACGUUUUUUAGUAGUAAUAUUUAAUCAAAAGAAUGAACACUUUAAGUU
env-170   CGGGG...CUA.AACU..........UAGUAACCCC....UGUGUAGAAGUGUUAAUAGUUG.AA..GCUU..........AAGGCC.AGGCU.CAACGGUUAGUG.AAUCGACUGCACAAAAGAAUUAAAAACGUUUUUUAGUAGUAAUAUUUAAUCAAAAGAAUGAACACUUUAAGUU
env-171   CGGGG...CUA.AACU..........UAGUAACCCC....UGUGUAGAAGUGUUAAUAGUUG.AA..GCUU..........AAGGCC.AGGCU.CAACGGUUAGUG.AAUCGACUGCACAAAAGAAUUAAAAACGUUUUUUAGUAGUAAUAUUUAAUCAAAAGAAUGAACACUUUAAGUU
env-205   CGGGG...CUA.AACU..........UAGUAACCCC....UGUGUAGAAGUGUUAAUAGUUG.AA..GCUU..........AAGGCC.AGGCU.CAACGGUUAGUG.AAUCGACUGCACAAAAGAAUUAAAAACGUUUUUUAGUAGUAAUAUUUAAUCAAAAGAAUGAACACUUUAAGUU
env-206   CGGGG...CUA.AACU..........UAGUAACCCC....UGUGUAGAAGUGUUAAUAGUUG.AA..GCUU..........AAGGCC.AGGCU.CAACGGUUAGUG.AAUCGACUGCACAAAAGAAUUAAAAACGUUUUUUAGUAGUAAUAUUUAAUCAAAAGAAUGAACACUUUAAGUU
env-207   CGGGG...CUA.AACU..........UAGUAACCCC....UGUGUAGAAGUGUUAAUAGUUG.AA..GCUU..........AAGGCC.AGGCU.CAACGGUUAGUG.AAUCGACUGCACAAAAGAAUUAAAAACGUUUUUUAGUAGUAAUAUUUAAUCAAAAGAAUGAACACUUUAAGUU
env-208   CGGGG...CUA.AACU..........UAGUAACCCC....UGUGUAGAAGUGUUAAUAGUUG.AA..GCUU..........AAGGCC.AGGCU.CAACGGUUAGUG.AAUCGACUGCACAAAAGAAUUAAAAACGUUUUUUAGUAGUAAUAUUUAAUCAAAAGAAUGAACACUUUAAGUU
env-209   CGGGG...CUA.AACU..........UAGUAACCCC....UGUGUAGAAGUGUUAAUAGUUG.AA..GCUU..........AAGGCC.AGGCU.CAACGGUUAGUG.AAUCGACUGCACAAAAGAAUUAAAAACGUUUUUUAGUAGUAAUAUUUAAUCAAAAGAAUGAACACUUUAAGUU
env-210   CGGGG...CUA.AACU..........UAGUAACCCC....UGUGUAGAAGUGUUAAUAGUUG.AA..GCUU..........AAGGCC.AGGCU.CAACGGUUAGUG.AAUCGACUGCACAAAAGAAUUAAAAACGUUUUUUAGUAGUAAUAUUUAAUCAAAAGAAUGAACACUUUAAGUU
env-211   CGGGG...CUA.AACU..........UAGUAACCCC....UGUGUAGAAGUGUUAAUAGUUG.AA..GCUU..........AAGGCC.AGGCU.CAACGGUUAGUG.AAUCGACUGCACAAAAGAAUUAAAAACGUUUUUUAGUAGUAAUAUUUAAUCAAAAGAAUGAACACUUUAAGUU
env-212   CGGGG...CUA.AACU..........UAGUAACCCC....UGUGUAGAAGUGUUAAUAGUUG.AA..GCUU..........AAGGCC.AGGCU.CAACGGUUAGUG.AAUCGACUGCACAAAAGAAUUAAAAACGUUUUUUAGUAGUAAUAUUUAAUCAAAAGAAUGAACACUUUAAGUU
env-213   CGGGG...CUA.AACU..........UAGUAACCCC....UGUGUAGAAGUGUUAAUAGUUG.AA..GCUU..........AAGGCC.AGGCU.CAACGGUUAGUG.AAUCGACUGCACAAAAGAAUUAAAAACGUUUUUUAGUAGUAAUAUUUAAUCAAAAGAAUGAACACUUUAAGUU
env-222   CGGGG...CUA.AACU..........UAGUAACCCC....UGUGUAGAAGUGUUAAUAGUUG.AA..GCUU..........AAGGCC.AGGCU.CAACGGUUAGUG.AAUCGACUGCACAAAAGAAUUAAAAACGUUUUUUAGUAGUAAUAUUUAAUCAAAAGAAUGAACACUUUAAGUU
env-226   CGGGG...CUA.AACU..........UAGUAACCCC....UGUGUGGAAGUGUUAAUAGUUG.AU..GCUU..........AAGGCC.AGGCG.CAACGGUUAGUG.AAUCGACUGCACAAAAGAAUUAAAAACGUUUUUUAGUAGUAAUAUUUAAUCAAAAGAAUGAACACUUUAAGUU
env-227   CGGGG...CUA.AACU..........UAGUAACCCC....UGUGCGGAAGUGUUAAUAGUUG.AU..GCUU..........AAGGCC.AGGCA.CAACGGUUAGUG.AAUCGACUGCACAAAAGAAUUAAAAACGUUUUUUAGUAGUAAUAUUUAAUCAAAAGAAUGAACACUUUAAGUU
env-229   CGGGG...CUA.AACU..........UAGUAACCCC....UGUGUGGAAGUGUUAAUAGUUG.AU..GCUU..........AAGGCC.AGGCA.CAACGGUUAGUG.AAUCGACUGCACAAAAGAAUUAAAAACGUUUUUUAGUAGUAAUAUUUAAUCAAAAGAAUGAACACUUUAAGUU
env-230   CGGGG...CUA.AACU..........UAGUAACCCC....UGUGUGGAAGUGUUAAUAGUUG.AU..GCUU..........AAGGCC.AGGCA.CAACGGUUAGUG.AAUCAACUGCACAAAAGAAUUAAAAACGUU...........................................
env-231   CGGGG...CUA.AACU..........UAGUAACCCC....UGUGUGGAAGUGUUAAUAGUUG.AU..GCUU..........AAGGCC.AGGCA.CAACGGUUAGUG.AAUCAACUGCACAAAAGAAUUAAAAACGUUUUUUAGUAGUAAUAUUUAAUCAAAAGAAUGAACACUUUAAGUU
env-232   CGGGG...CUA.AACU..........UAGUAACCCC....UGUGUGGAAGUGUUAAUAGUUG.AU..GCUU..........AAGGCC.AGGCA.CAACGGUUAGUG.AAUCGACUGCACAAAAGAAUUAAAAACGUUUUUUAGUAGUAAUAUUUAAUCAAAAGAAUGAACACUUUAAGUU
env-233   CGGGG...CUA.AACU..........UAGUAACCCC....UGUGUGGAAGUGUUAAUAGUUG.AU..GCUU..........AAGGCC.AGGCA.CAACGGUUAGUG.AAUCGACUGCACA............................................................
env-234   CGGGG...CUA.AACU..........UAGUAACCCC....UGUGUGGAAGUGUUAAUAGUUG.AU..GCUU..........AAGGCC.AGGCA.CAACGGUUAGUG.AAUCGACUGCACAAAAGAAUUAAAAACGUUUUUUAGUAGUAAUAUUUAAUCAAAAGAAUGAACACUUUAAGUU
env-246   CGGGG...CUA.AACU..........UAGUAACCCC....UGUGUGGAAGUGUUAAUAGUUG.AU..GCUU..........AAGGCC.AGGCA.CAACGGUUAGUG.AAUCAACUGCACAAAAGAAUUAAAAACGUUUUUUAGUAGUAAUAUUUAAUCAAAAGAAUGAACACUUUAAGUU
env-248   CGGGG...CUA.AACU..........UAGUAACCCC....UGUGUGGAAGUGUUAAUAGUUG.AU..GCUU..........AAGGCC.AGGCA.CAACGGUUAGUG.AAUCGACUGCACA............................................................
env-249   CGGGG...CUA.AACU..........UAGUAACCCC....UGUGUGGAAGUGUUAAUAGUUG.AU..GCUU..........AAGGCC.AGGCA.CAACGGUUAGUG.AAUCGACUGCACAAAAGAAUUAAAAACGUUUUUUAGUAGUAAUAUUUAAUCAAAAGAAUGAACACUUUAAGUU
env-250   CGGGG...CUA.AACU..........UAGUAACCCC....UGUGUAGAAGUGUUAAUAGUUG.AU..GCUU..........AAGGCC.AGGCA.CAACGGUUAGUG.AAUCGACUGCACAAAAGAAUUAAAAACGUUUUUUAGUAGUAAUAUUUAAUCAAAAGAAUGAACACUUUAAGUU
env-257   CGGGG...CUA.AACU..........UAGUAACCCC....UGUGUAGAAGUGUUAAUAGUUG.AU..GCUU..........AAGGCC.AGGCA.CAACGGUUAGUG.AAUCGACUGCACAAAAGAAUUAAAAACGUUUUUUAGUAGUAAUAUUUAAUCAAAAGAAUGAACACUUUAAGUU
env-258   CGGGG...CUA.AACU..........UAGUAACCCC....UGUGUAGAAGUGUUAAUAGUUG.AU..GCUU..........AAGGCC.AGGCA.CAACGGUUAGUG.AAUCGACUGCACAAAAGAAUUAAAAACGUUUUUUAGUAGUAAUAUUUAAUCAAAAGAAUGAACACUUUAAGUU
env-259   CGGGG...CUA.AACU..........UAGUAACCCC....UGUGUAGAAGUGUUAAUAGUUG.AU..GCUU..........AAGGCC.AGGCA.CAACGGUUAGUG.AAUCGACU.................................................................
env-260   CGGGG...CUA.AACU..........UAGUAACCCC....UGUGUAGAAGUGUUAAUAGUUG.AU..GCUU..........AAGGCC.AGGCA.CAACGGUUAGUG.AAUCGACUGCACAAAAGAAUUAAAAACGUUUUUUAGUAGUAAUAUUUAAUCAAAAGAAUGAACACUUUAAGUU
env-262   CGGGG...CUA.AACU..........UAGUAACCCC....AGCACAAAAGUGUUAAUAGUAA.AG..GCUU..........CUGGCC.AAGUC.UUACGGUUAGUG.AAUCUGCUGUGCAAGAAAAAUAAUAAAUUGUUUUUUAGUAGUAAUAUCAUUAAAAGAAUGAACACAUUAAGUU
env-264   CGGGG...CUA.AACU..........UAGUAACCCC....AGCACAAAAGUGUUAAUAGUAA.AG..GCUU..........CUGGCC.AAGUC.UUACGGUUAGUG.AAUCUGCUGUGCAAGAAAAAUAAUAAAUUGUUUUUUAGUAGUAAUAUCAUUAAAAGAAUGAACACAUUAAGUU
env-265   CGGGG...CUA.AACU..........UAGUAACCCC....AGCACAAAAGUGUUAAUAGUAA.AG..GCUU..........CUGGCC.AAGUC.UUACGGUUAGUG.AAUCUGCUGUGCAAGAAAAAUAAUAAAUUGUUUUUUAGUAGUAAUAUCAUUAAAAGAAUGAACACAUUAAGUU
env-266   AGGGGUGCC...UUUUU...........GGC.CCCC.....GAAUCUGUGUGUGAAUAGCCCAUU..UCCA..........UUGGCC.UGGAU.GGGCGGUUCGGG.AAUCACAGAGGAAGCGAGGCUGCUUAGUGCCUCGAAACGUAAUAAAGUAUAGUAACGAACAUUAAACAACUGA

          .<<<<<<<<<<<.............>>>>>>>>>>>................<<<<<.<<<..<<..<<<<<...............>>>>>>.>>>>.>>>>>............................................................................
          .222222.1111.............1111.222222................02200.122..22..2222................22222.2221.00220............................................................................
          •GGGG•◦-----R•••◦◦◦◦----------◦•CCCC----◦◦•••••••YGUGU•AAUAGY•--•Y--RYYU---------UUGGCC-GRRY•-••RYGGUU•GUG-AAUCR•••••••RR••R••••••••••••••••••••••••••••••••••••••••••••••••••••••
           5′                                                                                                        3′
```

```
env-267  ACAGGC......GUAAAG.............GCUUG....UCAAAUAGCGUGUUAAUAGCU..UU..GCUU..........UUGGCC.GGGCG..AGUGGUUAGUG.AAUCGCUAUAAAGAAAAAUUAGUAGUAAACAAAUUAAAAAAUUAAAGAAACUGAACACUUUAAGUUACAAGAC
Bsp-2-1  ACAAGC......GUAAAG.............GCUUG....UCAAAUAGCGUGUUAAUAGCU..UU..GCUU..........UUGGCC.GGGCG..AGUGGUUAGUG.AAUCGCUAUAAAGAAAAAUUAGUAGUAAACAAAUUAAAAAAUUAAAGAAAGUGAACACUUUAAGUUACAAGAC
env-268  UGAAGAU.....AAUACUUAGA........AUCUUC..AUAGCUUCUUCGUGUUAAUAGUG..AU..AUUC..........AUGGCC.GAAUA.UCAUGGUUAGUG.AAUCGUAGAAGCGCCGGGGAUAAUGCUGUGGAUGGUCUCUUUUAGACUG........................
env-269  UGAAGAU.....AAUACUUAGA........AUCUUC..AUAGCUUCUUCGUGUUAAUAGUG..AU..AUUC..........AUGGCC.GAAUA.UUAUGGUUAGUG.AAUCGUAGAAGCGCCGGGGAUAAUGCUGUGGAUGGUCUCUU................................
env-270  UGAAGAU.....AACACUUAGA........AUCUUC..ACAGCUUCUUCGUGUUAAUAGUG..AU..AUUC..........AUGGCC.GAAUA.UUAUGGUUAGUG.AAUCGUAGAAGCGCCGGGGAUAAUGCUGUGGAUGGUCUCUUUUAGACUGGAGGCGCCGCGAGGUCCCUGCCCA
env-272  UGAAGAU.....AACACUUAGA........AUCUUC..ACAGCUUCUUCGUGUUAAUAGUG..AU..AUUC..........AUGGCC.GAAUA.UUAUGGUUAGUG.AAUCGUAGAAGCGCCGGGGAUAAUGCUGUGGAUGGUCUCUUUUAGACUG........................
env-273  UGAAGAU.....AAUACUUAGA........AUCUUC..ACAGCUUCUUCGUGUUAAUAGUG..AU..AUUC..........AUGGCC.GAAUA.UUAUGGUUAGUG.AAUCGUAGAAGCGCCGGGGAUAAUGCUGUGGAUGGUCUCUUUUAGACUG........................
env-275  UGAAGAU.....AAUACUUAGA........AUCUUC..ACAGCUUCUUCGUGUUAAUAGUG..AU..AUUC..........AUGGCC.GAAUA.UUAUGGUUAGUG.AAUCGUAGAAGCGCCGGGGAUAAUGCUGUGGAUGGUCUCUU................................
env-276  UGAAGAU.....AAUACUUAGA........AUCUUC..ACAGCUUCUUCGUGUUAAUAGUG..AU..AUUC..........AUGGCC.GAAUA.UUAUGGUUAGUG.AAUCGUAGAAGCGCCGGGGAUAAUGCUGUGGAUGGUCUCUUUUAGACUG........................
env-278  CGAAGA......GAAGACUUAGAA.......UCUUC..GCAGCUCCCUCGUGUUAAUAGUG..AC..AUUC..........AUGGCC.GGAUG.UCAUGGUUAGUG.AAUCGCCGGAGCGUCAGGGAAAAUGCUGUGGAUGGUCUCUUUU..............................
env-279  CGAAGA......GAAGACUUAGAA.......UCUUC..GCAGCUCCCUCGUGUUAAUAGUG..AC..AUUC..........AUGGCC.GGAUG.UCAUGGUUAGUG.AAUCGCCGGAGCGUCAGGGAAAAUGCUGUGGAUGGUCUCUUUUAGACUGGAGGCGCCGCGAGGUCUCUGCUCA
env-280  CGAAGA......GAAGACUUAGAA.......UCUUC..GCAGCUCCCUCGUGUUAAUAGUG..AC..AUUC..........AUGGCC.GGAUG.UCAUGGUUAGUG.AAUCGCCGGAGCGUCAGGGAAAAUGCUGUGGAUGGUCUCUUUUAGACUG........................
env-281  CGAAGA......GAAGACUUAGAA.......UCUUC..GCAGCUCCCUCGUGUUAAUAGUG..AC..AUUC..........AUGGCC.GGAUG.UCAUGGUUAGUG.AAUCGCCGGAGCGUCAGGGAAAAUGCUGUGGAUGGUCUCUUUUAGA..........................
env-285  CGAGGA......UAACGCUUAGAU.......UCUUC..GCAGCUCUUUCGUGUUAAUAGUG..GU..AUUC..........AUGGCC.GAAUG.UCAUGGUUAGUG.AAUCGUUAGAGCGUCAGGGAUAAUGCUGUGGAUGGUCUCUUUUAGACUGGAGGCGCCGCGAGGUUUCUGUUAU
env-286  CGAGGA......UAACGCUUAGAU.......UCUUC..GCAGCUCUUUCGUGUUAAUAGUG..GU..AUUC..........AUGGCC.GAAUG.UCAUGGUUAGUG.AAUCGUUAGAGCGUCAGGGAUAAUGCUGUGGAUGGUCUCUUUUAGACUG........................
env-287  CGAGGA......UAACGCUUAGAU.......UCUUC..GCAGCUCUUUCGUGUUAAUAGUG..GU..AUUC..........AUGGCC.GAAUA.UCAUGGUUAGUG.AAUCGUUAGAGCGUCAGGGAUAAUGCUGUGGAUGGUCUCUUUUAGACUGGAGGCGCCGCGAGGUCCCUGCUCA
env-288  CGAGGA......UAACGCUUAGAU.......UCUUC..GCAGCUCUUUCGUGUUAAUAGUG..GU..AUUC..........AUGGCC.GAAUA.UCAUGGUUAGUG.AAUCGUUAGAGCGUCAGGGAUAAUGCUGUGGAUGGUCUCUUUUAGACUGGAGGCGCCGCGAGGUCCCUGCUCA
env-290  CGAGGA......UAACGCUUAGAU.......UCUUC..GCAGCUCUUUCGUGUUAAUAGUG..GU..AUUC..........AUGGCC.GAAUA.UCAUGGUUAGUG.AAUCGUUAGAGCGUCAGGGAUAAUGCUGUGGAUGGUCUCUUUUAGACUGGAGGCGCCGCGAGGUCCCUGCUCA
env-291  CGAGGA......UAACGCUUAGAU.......UCUUC..GCAGCUCUUUCGUGUUAAUAGUG..GU..AUUC..........AUGGCC.GAAUA.UCAUGGUUAGUG.AAUCGUUAGAGCGUCAGGGAUAAUGCUGUGGAUGGUCUCUUUUAGACUGGAGGCGCCGCGAGGUCCCUGCCCA
env-292  CGAGGA......UAACGCUUAGAU.......UCUUC..GCAGCUCUUUCGUGUUAAUAGUG..GU..AUUC..........AUGGCC.GAAUA.UCAUGGUUAGUG.AAUCGUUAGAGCGUCAGGGAUAAUGCUGUGGAUGGUCUCUUUUAGACUGNNNNNNNNNNNNNNNNNNNNNNNN
env-293  CGAGGA......UAACGCUUAGAU.......UCUUC..GCAGCUCUUUCGUGUUAAUAGUG..GU..AUUC..........AUGGCC.GAAUA.UCAUGGUUAGUG.AAUCGUUAGAGCGUCAGGGAUAAUGCUGUGGAUGGUCUCUUUUAGACUGGAGGCGCCGCGAGGUCCCUGCUCA
env-295  CGAGGA......UAACGCUUAGAU.......UCUUC..GCAGCUCUUUCGUGUUAAUAGUG..GU..AUUC..........AUGGCC.GAAUA.UCAUGGUUAGUG.AAUCGUUAGAGCGUCAGGGAUAAUGCUGUGGAUGGUCUCUUUUAGACUG........................
env-297  CGAGGA......UAACGCUUAGAU.......UCUUC..GCAGCUCUUUCGUGUUAAUAGUG..GU..AUUC..........AUGGCC.GAAUA.UCAUGGUUAGUG.AAUCGUUAGAGCGUCAGGGAUAAUGCUGUGGAUGGUCUCUU................................
env-298  CGAGGA......UAACGCUUAGAU.......UCUUC..GCAGCUCUUUCGUGUUAAUAGUG..GU..AUUC..........AUGGCC.GAAUA.UCAUGGUUAGUG.AAUCGUUAGAGCGUCAGGGAUAAUGCUGUGGAUGGUCUCUUUUAGACUGGAGGCGCCGCGAGGUCCCUGCUCA
env-299  CGAGGA......UAACGCUUAGAU.......UCUUC..GCAGCUCUUUCGUGUUAAUAGUG..GU..AUUC..........AUGGCC.GAAUA.UCAUGGUUAGUG.AAUCGUUAGAGCGUCAGGGAUAAUGCUGUGGAUGGUCUCUUUUAGACUGGAGGCGCCGCGAGGU.........
env-301  CGAGGA......UAACGCUUAGAU.......UCUUC..GCAGCUCUUUCGUGUUAAUAGUG..GU..AUUC..........AUGGCC.GAAUA.UCAUGGUUAGUG.AAUCGUUAGAGCGUCAGGGAUAAUGCUGUGGAUGGUCUCUUUU..............................
env-302  CGAGGA......UAACGCUUAGAU.......UCCUC..GCAGCUCUUUCGUGUUAAUAGUG..GU..AUUC..........AUGGCC.GGAUA.UCAUGGUUAGUG.AAUCGUUAGAGCGUCAGGGAUAAUGCUGUGGAUGGUCUCUUUUAGACUGGAGGCGCCGCGAGGU.........
env-305  CGAGGA......UAACGCUUAGAU.......UCUUC..GCAGCUCUUUCGUGUUAAUAGUG..GU..AUUC..........AUGGCC.GGAUA.UCAUGGUUAGUG.AAUCGUCAGAGCGUCAGGGAUAAUGCUGUGGAUGGUCUCUUUUAGACUGGAGGCGCCGCGAGGUCCCUGCUCA
env-307  CGAGGA......UAACGCUUAGAU.......UCUUC..GCAGCUCUUUCGUGUUAAUAGUG..GU..AUUC..........AUGGCC.GGAUA.UCAUGGUUAGUG.AAUCGUCAGAGCGUCAGGGAUAAUGCUGUGGAUGGUCUCUUUUAGACUGGAGGCGCCGCGAGGUCCCUGCUCA
env-308  CGAGGA......UAACGCUUAGAU.......UCUUC..GCAGCUCUUUCGUGUUAAUAGUG..GU..AUUC..........AUGGCC.GGAUA.UCAUGGUUAGUG.AAUCGUUAGAGCGUCAGGGAUAAUGCUGUGGAUGGUCUCUUUUAGACUGGAGGCGCCGCGAGGUCCCUGCUCA
env-309  CGAGGA......UAACGCUUAGAU.......UCUUC..GCAGCUCUUUCGUGUUAAUAGUG..GU..AUUC..........AUGGCC.GGAUA.UCAUGGUUAGUG.AAUCGUUAGAGCGUCAGGGAUAAUGCUGUGGAUGGUCUCUUUUAGACUGGAGGCGCCGCGAGGUCCCUGCUCA
env-310  CGAGGA......UAACGCUUAGAU.......UCUUC..GCAGCUCUUUCGUGUUAAUAGUG..GU..AUUC..........AUGGCC.GGAUA.UCAUGGUUAGUG.AAUCGUCAGAGCGUCAGGGAUAAUGCUGUGGAUGGUCUCUUUUAGACUG........................
env-311  CGAGGA......UAACGCUUAGAU.......UCUUC..GCAGCUCUUUCGUGUUAAUAGUG..GU..AUUC..........AUGGCC.GGAUA.UCAUGGUUAGUG.AAUCGUCAGAGCGUCAGGGAUAAUGCUGUGGAUGGUCUCUU................................
env-314  CGAGGA......UAACGCUUAGAU.......UCUUC..GCAGCUCUUUCGUGUUAAUAGUG..GU..AUUC..........AUGGCC.GGAUA.UCAUGGUUAGUG.AAUCGUCAGAGCGUCAGGGAUAAUGCUGUGGAUGGUCUCUUUUAG...........................
env-315  GCGAAGA.....UAACGCUUAGAU......UCUUCG...CAGCUCUUUCGUGUUAAUAGUG..GU..AUUC..........AUGGCC.GGAUA.UCAUGGUUAGUG.AAUCGUCAGAGCGUCAGGGAUAAUGCUGUGGAUGGUCUCUUUUAGACUGGAGGCGCCGCGAGGUCCUUGUUCA
env-316  UGGGAGUGC...AUCUUA..........GAGCUUCC..GCAGCCGUGAGGUGUUAAUAGUCU.GU..CUC.........AUUUGGCCGAGACA.CGACGGUUAGUG.AAUCUUCACCGGCAUCAGAGAUAAUGCUGUGGAUGGUU...................................
env-317  UGGGAGU.....GUGUCUUAGA........GCUUCC..GCAGCCGUGAGGUGUUAAUAGUCU.GU..CUC.........AUUUGGCCGAGACA.CGACGGUUAGUG.AAUCUUCAUCGGCAUCAGAGAUAAUGCUGUGGAUGGUCUCUUUUAGACUGGAGGCGCCGCGAGGUCUCUGUUA
env-318  UGGGAGU.....GUGUCUUAGA........GCUUCC..ACAGCCGUGAGGUGUUAAUAGUCU.GU..CUC.........AUUUGGCCGAGACA.CGACGGUUAGUG.AAUCUUCAUCGGCAUCAGAGAUAAUG...............................................
env-319  UGGGCGUGUU..CUU............AGCACGGUC..GCAGCAAAGUCGUGUUAAUAGCG..AU..GCCU..........UUGGCC.AGACA.UCGUGGUUAGUG.AAUCGGCUUCGCAUCAGAGAUAAAGCUGUGGAUGGUCUCUUUUAGACUGGAGGCGCCGCGAGGUCUCUGUAUA
Psp-3-1  UGGGGACGC...GUCUUA..........GAAUCCCC..GCAGCCCGAUGGUGUUAAUAGCU..CU..UUCC..........AUGGCC.GGGAA.GAGUGGUUAGUG.AAUCCAGCGGACAUCAGAGAGAAUGCUGUGGAUGGUCUCUUUUAGACUGGAGGCGCCGCGAGGUCUCUGUUAU
env-320  UGGUAGC.....UUGCUUAGAA........GUUGCC...GCGCCGGAUAGUGUUAAUAGUC..GU..GCCU..........UUGGCC.GGACA.CGAUGGUUAGGG.AAUCUGUCGGGCAGUCGCAGCGGGAAUAAUGCUGGAUGGUCUCUUUUAGACUGGAGGCGCCGUGAGGUUUCCG
env-321  UGGCAGC.....UUGCUUAGAA........GUUGCC...GCGCCGGAUAGUGUUAAUAGUC..GU..GCCU..........UUGGCC.GGACA.CGACGGUUAGGG.AAUCUGUCGGGCAGUCGCAGCGGGAAUAAUGCUGGAUGGUCUCUUUUAGACUGGAGGCGCCGUGAGGUUUCCG
env-322  UGGCAGC.....UUGCUUAGAA........GUUGCC...GCGCCGGAGAGUGUUAAUAGUC..GU..GCCU..........UUGGCC.GGACA.CGAUGGUUAGGG.AAUCUGUCGGGCAGUCGCAGCGGGAAUAAUGCUGGAUGGUCUCUUUUAGACUGGAGGCGCCGUGAGGUUUCCG
env-323  UGGCACCUU...GCUUAG..........AAGUUGCC...GCGCCGGAUAGUGUUAAUAGUC..GU..GCCU..........UUGGCC.GGACA.CGAUGGUUAGGG.AAUCUGUCGGGCAGUCGCAGCGGGAAUAAUGCU........................................
env-324  UGGCUGCUU...GCUUAG..........AAGUUGCC...GCGCCGGAUAGUGUUAAUAGUC..GU..GCCU..........UUGGCC.GGACA.CGAUGGUUAGGG.AAUCUGUCGGGCAGUCGCAGCGGGAAUAAUGCUGGAUGGUCUCUUUUAGACUGGAGGCGCCGUGAGGUUUCCG
env-326  UGGCAGCUU...GCUUAG..........AAGUUGCC...GCGCCGGAUAGUGUUAAUAAUC..GU..GCCU..........UUGGCC.GGACA.CGAUGGUUAGGG.AAUCUGUCGGGCAGUCGCAGCGGGAAUAAUGCUGGAUGGUCUCUUUUAGACUGGAGGCGCCGUGAGGUUUCCG
env-327  UGGCAGCUU...GCUUAG..........AAGUUGCC...GCGCCGGAUAGUGUUAAUAGUC..GU..GCCU..........UUGGCC.GGACA.CGAUGGUUAGGG.AAUCUGUCGGGCAGUCGCAGCGGGAAUAAUGCUGGAUGGUCUCUUUUAGACUGGAGGCGCCGUGAGGUUUCCG
env-328  UGGCAGCUU...GCUUAG..........AAGUUGCC...GCGCCGGAUAGUGUUAAUAGUC..GU..GCCU..........UUGGCC.GGACA.CGAUGGUUAGGG.AAUCUGUCAGGCAGUCGCAGCGGGAAUAAUGCUGGAUGGUCUCUUUUAGACUGGA..................
env-329  UGGCAGCUU...GCUUAG..........AAGUUGCC...GCGCCGGAUAGUGUUAAUAGUC..GU..GCCU..........UUGGCC.GGACA.CGAUGGUUAGGG.AAUCUGUCGGGCAGUCGCAGCGGGAAUAAUGCUGGAUGGUCUCUUUUAGACUGGAGGCGCCGUGAGGUUUCCG
env-330  UGGCAGCUU...GCUUAG..........AAGUUGCC...GCGCCGGAUAGUGUUAAUAGUC..GU..GCCU..........UUGGCC.GGACA.CGAUGGUUAGGG.AAUCUGUCGGGCAGUCGCAGCGGGAAUAAUGCUGGAUGGUCUCUUUUAGACUGGAGGCGCCGUGAGGUUUCCG
env-331  UGGCAGCUU...GCUUAG..........AAGUUGCC...GCGCCGGAUAGUGUUAAUAGUC..GU..GCCU..........UUGGCC.GGACA.CGAUGGUUAGGG.AAUCUGUCGGGCAGUCGCAGCGGGAAUAAUGCUGGAUGGUCUCUUUUAGACUGGAGGCGCCGUGAGGUUUCCG
env-336  UGGCAGCUU...GCUUAG..........AAGUUGCC...GCGCCGGAUAGUGUUAAUAGUC..GU..GCCU..........UUGGCC.GGACA.CGAUGGUUAGGG.AAUCUGUCGGGCAGUCGCAGCGGGAAUAAUGCUAGAUGGUCUCUUUUAGACUGGAGGCGCCGUGAGGUUUCCG
env-337  UGGCAGCUU...GCUUAG..........AAGUUGCC...GCGCCGGAUAGUGUUAAUAGUC..GU..GCCU..........UUGGCC.GGACA.CGAUGGUUAGGG.AAUCUGUCGGGCAGUCGCAGCGGGAAUAAUGCUGGAUGGUCUCUUUUAGACUGGAGGCGCCGUGAGGUUUCCG
env-338  UGGCAGCUU...GCUUAG..........AAGUUGCC...GCGCCGGAUAGUGUUAAUAGUC..GU..GCCU..........UUGGCC.GGACA.CGAUGGUUAGGG.AAUCUGUCGGGCAGUCGCAGCGGGAAUAAUGCUGGAUGGUCUCUUUUAGACUGGAGGCGCCGUGAGGUUUCCG
env-339  UGGCAGCUU...GCUUAG..........AAGUUGCC...GCGCCGGAUAGUGUUAAUAGUC..GU..GCCU..........UUGGCC.GGACA.CGAUGGUUAGGG.AAUCUGUCGGGCAGUCGCAGCGGGAAUAAUGCUGGAUGGUCUCUUUUAGACUGGAGGCGCCGUGAGGUUUCCG
env-341  UGGCAGCUU...GCUUAG..........AAGUUGCC...GCGCCGGAUAGUGUUAAUAGUC..GU..GCCU..........UUGGCC.GGACA.CGAUGGUUAGGG.AAUCUGUCGGGCAGUCGCAGCGGGAAUAAUGCUGGAUGGUCUCUUUUAGACUGGAGGCGCCGUGAGGUUUCCG
env-345  UGGCAGCUU...GCUUAG..........AAGUUGCC...GCGCCGGAUAGUGUUAAUAGUC..GU..GCCU..........UUGGCC.GGACA.CGAUGGUUAGGG.AAUCUGUCGGGCAGUCGCAGCGGGAAUAAUGCUGGAUGGUCUCUUUUAGACUGGAGGCGCCGUGAGGUUUCCG
env-347  UGGCAGCUU...GCUUAG..........AAGUUGCC...GCGCCGGAUAGUGUUAAUAGUC..GU..GCCU..........UUGGCC.GGACA.CGAUGGUUAGGG.AAUCUGUCGGGCAGUCGCAGCGGGAAUAAUGCUGGAUGGUCUCUUUUAGACUGGAGGCGCCGUGAGGUUUCCG
env-348  UGGCAGCUU...GCUUAG..........AAGUUGCC...GCGCCGGAUAGUGUUAAUAGUC..GU..GCCU..........UUGGCC.GGACA.CGAUGGUUAGGG.AAUCUGUCGGGCAGUCGCAGCGGGAAUAAUGCUGGAUGGUCUCUUUUAGACUGGAGGCGCCGUGAGGUUUCCG
env-349  UCGGGAAU....AAGCUUAGG........AUUCCCG..GCAGUUUGGCAGUGUUAAUAGCC..UU..UUCU..........UUGGCC.GGAAU.UGGCGGUUAGUG.AAUCUGUCAGACAGCAGAAAGAAAGCUGUGGAUGGUCUCUUUUAGA...........................
Pma-3-1  UCGGGAAU....AAGCUUAGA........AUUCCCG..GUAGUUUGGCAGUGUUAAUAGCC..UU..UUCU..........UUGGCC.GGAAU.UGGCGGUUAGUG.AAUCUGUCAGACAGCAGAAAGAAAGCUGUGGAUGGUCUCUUUUAGACUGGAGGCGCCGCGAGGUUUCUGUUAU
env-350  UCGGGAAU....AAGCUUAGA........AUUCCCG..GCAGUUUGGCAGUGUUAAUAGCC..UU..UUCU..........UUGGCC.GGAAU.UGGCGGUUAGUG.AAUCUGUCAGACAGCAGAAAGAAAGCUGUGGAUGGUCUCUUUUAGACUGGAGGCGCCGCGAGGUUUCUGUUAU
env-351  UCGGGAAU....AAGCUUAGA........AUUCCCG..GCAGUUUGGCAGUGUUAAUAGCC..UU..UUCU..........UUGGCC.GGAAU.UGGCGGUUAGUG.AAUCUGUCAGACAGCAGAAAGAAAGCUGUGGAUGGUCUCUUUUAGACUGGAGGCGCCGCGAGGUUUCUGUUAU
Pma-1-1  UCGGGAAU....AAGCUUAGA........AUUCCCG..GCAGUUUGGCAGUGUUAAUAGCC..UU..UUCU..........UUGGCC.GGAAU.UGGCGGUUAGUG.AAUCUGUCAGACAGCAGAAAGAAAGCUGUGGAUGGUCUCUUUUAGACUGGAGGCGCCGCGAGGUUUCUGUUAU
env-353  UCGGGAAU....AAGCUUAGA........AUUCCCG..GCAGUUUGGCAGUGUUAAUAGCC..UU..UUCU..........UUGGCC.GGAAU.UGGCGGUUAGUG.AAUCUGUCAGACAGCAGAAAGAAAGCUGUGGAUGGUCUCUUUUAGA...........................
env-354  UCGGGAAU....AAGCUUAGA........AUUCCCG..GCAGUUUGGCAGUGUUAAUAGCC..UU..UUCU..........UUGGCC.GGAAU.UGGCGGUUAGUG.AAUCUGUCAGACAGCAGAAAGAAAGCUGUGGAUGGUCUCUUUUAGA...........................
env-355  UCGGGAAU....AAGCUUAGA........AUUCCCG..GCAGUUUGGCAGUGUUAAUAGCC..UU..UUCU..........UUGGCC.GGAAU.UGGCGGUUAGUG.AAUCUGUCAGACAGCAGAAAGAAAGCUGUGGAUGGUCUCUUUUAGA...........................
env-357  CUGGGAGAGC..GCUUG..........GAAUUCCCA..GUAGUUUAGCAGUGUUAAUAGCU..UU..UUCU..........UUGGCC.AGAUU.AGGCGGUUAGUG.AAUCUGUUAAACAACAGAACGUAAAGCUGUGGAUGGUCUCUUUUAGACUGGAGGCGCCGCGAGGUUUCUGUUA
env-359  CUGGGAGAGC..GCUUG..........GAAUUCCCA..GUAGUUUAGCAGUGUUAAUAGCU..UU..UUCU..........UUGGCC.AGAUU.AGGCGGUUAGUG.AAUCUGUUAAACAACAGAACGUAAAGCUGUGGAUGGUCUCUUUUAGACUGGAGGCGCCGCGAGGUUUCUGUUA
env-360  CUGGGAGAGC..GCUUG..........GAAUUCCCA..GUAGUUUAGCAGUGUUAAUAGCU..UU..UUCU..........UUGGCC.AGAUU.AGGCGGUUAGUG.AAUCUGUUAAACAACAGAA......................................................
env-361  CUGGGAGAGC..GCUUG..........GAAUUCCCA..GUAGUUUAGCAGUGUUAAUAGCU..UU..UUCU..........UUGGCC.AGAUU.AGGCGGUUAGUG.AAUCUGUUAAACAACAGAACGUAAAGCUGUGGAUGGUCUCUUUUAGACUGGAGGCGCCGCGAGGUUUCUGUUA
env-362  CUGGGAGAGC..GCUUG..........GAAUUCCCA..GUAGUUUAGCAGUGUUAAUAGCU..UU..UUCU..........UUGGCC.AGAUU.AGGCGGUUAGUG.AAUCUGUUAAACAACAGAACGUAAAGCUGUGGAUGGUCUCUUUUAGACUG.......................
env-363  CUGGGAGAGC..GCUUG..........GAAUUCCCA..GUAGUUUAGCAGUGUUAAUAGCU..UU..UUCU..........UUGGCC.AGAUU.AGGCGGUUAGUG.AAUCUGUUAAACAACAGAACGUAAAGCUGUGGAUGGUCUCUUUUAGACUG.......................
env-368  CUGGGAGAGC..GCUUG..........GAAUUCCCA..GUAGUUUAGCAGUGUUAAUAGCU..UU..UUCU..........UUGGCC.AGAUU.AGGCGGUUAGUG.AAUCUGUUAAACAACAGAACGUAAAGCUGUGGAUGGUCUCUUUUAGACUGGAGGCGCCGCGAGGUUUCUGUUA
env-369  CUGGGAGAGC..GCUUG..........GAAUUCCCA..GUAGUUUAGCAGUGUUAAUAGCU..UU..UUCU..........UUGGCC.AGAUU.AGGCGGUUAGUG.AAUCUGUUAAACAACAGAACGUAAAGCUGUGGAUGGUCUCUUUUAGACUGGAGGCGCCGCGAGGUUUCUGUUA
env-370  CUGGGAGAGC..GCUUG..........GAAUUCCCA..GUAGUUUAGCAGUGUUAAUAGCU..UU..UUCU..........UUGGCC.AGAUU.AGGCGGUUAGUG.AAUCUGUUAAACAACAGAACGUAAAGCUGUGGAUGGUCUCUUUUAGACUGGAGGCGCCGCGAGGUUUCUGUUA
env-371  CUGGGAGAGC..GCUUG..........GAAUUCCCA..GUAGUUUAGCAGUGUUAAUAGCU..UU..UUCU..........UUGGCC.AGAUU.AGGCGGUUAGUG.AAUCUGUUAAACAACAGAACGUAAAGCUGUGGAUGGUCUCUUUUAGACUGGAGGCGCCGCGAGGUUUCUGUUA
env-373  CUGGGAGAGC..GCUUG..........GAAUUCCCA..GUAGUUUAGCAGUGUUAAUAGCU..UU..UUCU..........UUGGCC.AGAUU.AGGCGGUUAGUG.AAUCUGUUAAACAACAGAACGUAAAGCUGUGGAUGGUCUCUUUUAGACUG.......................
env-375  CUGGGAGAGC..GCUUG..........GAAUUCCCA..GUAGUUUAGCAGUGUUAAUAGCU..UU..UUCU..........UUGGCC.AGAUU.AGGCGGUUAGUG.AAUCUGUUAAACAACAGAACGUAAAGCUGUGGAUGGUCUCUUUUAGACUGGAGGCGCCGCGAGGUUUCUGUUA
env-384  CUGGGAGAGC..GCUUG..........GAAUUCCCA..GUAGUUUAGCAGUGUUAAUAGCU..UU..UUCU..........UUGGCC.AGAUU.AGGCGGUUAGUG.AAUCUGUUAAACAACAGAACGUAAAGCUGUGGAUGGUCUCUUUUAGACUGGAGGCGCCGCGAGGUUUCUGUUA
env-385  CUGGGAGAGC..GCUUG..........GAAUUCCCA..GUAGUUUAGCAGUGUUAAUAGCU..UU..UUCU..........UUGGCC.AGAUU.AGGCGGUUAGUG.AAUCUGUUAAACAACAGAACGUAAAGCUGUGGAUGGUCUCUUUUAGACUGGAGGCGCCGCGAGGUUUCUGUUA
env-386  CUGGGAGAGC..GCUUG..........GAAUUCCCA..GUAGUUUAGCAGUGUUAAUAGCU..UU..UUCU..........UUGGCC.AGAUU.AGGCGGUUAGUG.AAUCUGUUAAACAACAGAACGUAAAGCUGUGGAUGGUCUCUUUUAGA..........................
env-387  CUGGGAGAGC..GCUUG..........GAAUUCCCA..GUAGUUUAGCAGUGUUAAUAGCU..UU..UUCU..........UUGGCC.AGAUU.AGGCGGUUAGUG.AAUCUGUUAAACAACAGAACGUAAAGCUGUGGAUGGUCUCUUUUAGACUGGAGGCGCCGCGAGGUUUCUGUUA

         .<<<<<<<<<<<.............>>>>>>>>>>>................<<<<<.<<<..<<..<<<<<...............>>>>>>.>>>>.>>>>>............................................................................
         .222222.1111.............1111.222222................02200.122..22..2222................22222.2221.00220............................................................................
         •GGGG•○-----R••••○○○----------○•CCCC----••••••••YGUGU•AAUAGY•--•Y--RYYU----------UUGGCC-GRRY•-••RYGGUU•GUG-AAUCR••••••••RR••R•••••••••••••••••••••••••••••••••••••••••••••••••••••••
          5′                                                                                                      3′
```

```
env-388   CUGGGAGAGC..GCUUG..........GAAUUCCCA..GUAGUUUAGCAGUGUUAAUAGCU..UU..UUCU..........UUGGCC.AGAUU.AGGCGGUUAGUG.AAUCUGUUAAACAACAGAACGUAAAGCUGUGGAUGGUCUCUUUUAGACUG.......................
env-389   CUGGGAGAGC..GCUUG..........GAAUUCCCA..GUAGUUUAGCAGUGUUAAUAGCC..UU..UUCU..........UUGGCC.AGAUU.AGGCGUUUAGUG.AAUCUGUUAAACAACAGAACGUAAAGCUGUGGAUGGUCUCUUUUAGACUGGAGGCGCCGCGAGGUUUCUGUUA
env-390   CUGGGAGAGC..GCUUG..........GAAUUCCCA..GUAGUUUAGCAGUGUUAAUAGCC..UU..UUCU..........UUGGCC.AGAUU.AGGUGGUUAGUG.AAUCUGUUAAACAACAGAACGUAAAGCUGUGGAUGGUCUCUUUUAGACUGGAGGCGCCGCGAGGUUUCUGUUA
env-392   CUGGGAGAGC..GCUUG..........GAAUUCCCA..GUAGUUUAGCAGUGUUAAUAGCC..UU..UUCU..........UUGGCC.AGAUU.AGGCGGUUAGUG.AAUCUGUUAAACAACAGAACGUAAAGCUGUGGAUGGUCUCUUUUAGACUGGAGGCGCCGCGAGGUUUCUGUUA
env-396   CUGGGAGAGC..GCUUG..........GAAUUCCCA..GUAGUUUAGCAGUGUUAAUAGCC..UU..UUCU..........UUGGCC.AGAUU.AGGCGGUUAGUG.AAUCUGUUAAACAACAGAACGUAAAGCUGUGGAUGGUCUCUUUUAGACUGGAGGCGCCGCGAGGUUUCUGUUA
env-397   CUGGGAGAGC..GCUUG..........GAAUUCCCA..GUAGUUUAGCAGUGUUAAUAGCC..UU..UUCU..........UUGGCC.AGAUU.AGGCGGUUAGUG.AAUCUGUUAAACAACAGAACGUAAAG...............................................
env-398   CUGGGAGAGC..GCUUG..........GAAUUCCCA..GUAGUUUAGCAGUGUUAAUAGCC..UU..UUCU..........UUGGCC.AGAUU.AGGCGGUUAGUG.AAUCUGUUAAACAACAGAACGUAAAGCUGUGGAUGGUCUCUUUUAGACUGGAGGCGCCGCGAGGUUUCUGUUA
env-399   CUGGGAGAGC..GCUUG..........GAAUUCCCA..GUAGUUUAGCAGUGUUAAUAGCC..UU..UUCU..........UUGGCC.AGAUU.AGGCGGUUAGUG.AAUCUGUUAAACAACAGAACGUAAAGCUGUGGAUGGUCUCUUUUAGACUGGAGGCGCCGCGAGGUUUCUGUUA
env-400   CUGGGAGAGC..GCUUG..........GAAUUCCCA..GUAGUUUAGCAGUGUUAAUAGCC..UU..UUCU..........UUGGCC.AGAUU.AGGCGGUUAGUG.AAUCUGUUAAACAACAGAACGUAAAGCUGUGGAUGGUCUCUUUUAGACUGGAGGCGCCGCGAGGUUUCUGUUA
env-401   CUGGGAGAGC..GCUUG..........GAAUUCCCA..GUAGUUUAGCAGUGUUAAUAGCC..UU..UUCU..........UUGGCC.AGAUU.AGGCGGUUAGUG.AAUCUGUUAAACAACAGAACGUAAAGCUGUGGAUGGUCUCUUUUAGACUGGAGGCGCCGCGAGGUUUCUGUUA
env-402   CUGGGAGAGC..GCUUG..........GAAUUCCCA..GUAGUUUAGCAGUGUUAAUAGCC..UU..UUCU..........UUGGCC.AGAUU.AGGCGGUUAGUG.AAUCUGUUAAACAACAGAACGUAAAGCUGUGGAUGGUCUCUUUUAGACUGGAGGCGCCGCGAGGUUUCUGUUA
env-403   CUGGGAGAGC..GCUUG..........GAAUUCCCA..GUAGUUUAGCAGUGUUAAUAGCC..UU..UUCU..........UUGGCC.AGAUU.AGGCGGUUAGUG.AAUCUGUUAAACAACAGAACGUAAAGCUGUGGAUGGUCUCUUUUAGACUGGAGGCGCCGCGAGGUUUCUGUUA
env-406   CUGGGAGAGC..GCUUG..........GAAUUCCCA..GUAGUUUAGCAGUGUUAAUAGCC..UU..UUCU..........UUGGCC.AGAUU.AGGCGGUUAGUG.AAUCUGUUAAACAACAGAACGUAAAGCUGUGGAUGGUCUCUUUUAGACUGGAGGCGCCGCGAGGUUUCUGUUA
env-408   GGGAAA......GCACUUGGAA.........UUUCC.GGCAGUCUGGCAGUGUUAAUAGCC..UU..UUCU..........UUGGCC.GGAUA.AGGCGGUUAGUG.AAUCUGUCGGACAACAGAACAAAAGCUGUGGAUGGUCUCUUUUAGACUGGAGGCGCCGCGAGGUUUCUGUUAU
env-410   GGGAAA......GCACUUGGAA.........UUUCC.GGCAGUCUGGCAGUGUUAAUAGCC..UU..UUCU..........UUGGCC.GGAUA.AGGCGGUUAGUG.AAUCUGUCGGACAACAGAACAAAAGCUGUGGAUGGUCUCUUUUAGACUGGAGGCGCCGCGAGGUUUCUGUUAU
env-411   GGGAAA......GCACUUGGAA.........UUUCC.GGCAGUCUGGCAGUGUUAAUAGCC..UU..UUCU..........UUGGCC.GGAUA.AGGCGGUUAGUG.AAUCUGUCGGGCAACAGAACAAAAGCUGUGGAUGGUCUCUUUUAGACUGGAGGCGCCGCGAGGUUUCUGUUAU
env-412   UGGGAGAGC...GCUUG...........GAAUUCCC.UGUAGUUUAGCAGUGUUAAUAGCC..UU..UUCU..........UUGGCC.AGAUU.AGGCGGUUAGUG.AAUCUGUUAAACAACAGAACGUAAAGCUGUGGAUGGUCUCUUUUAGACUGGAGGCGCCGCGAGGUUUCUGUUA
Por-6-1   UGGGAAAGC...GCUUG...........GAAUUUCC.GGCAGUCUGGCAGUGUUAAUAGCC..UU..UUCU..........UUGGC..GGAUA.AGGCGGUUAGUG.AAUUUGUCGGACAACAGGACAAAAGCUGUGGAUGGUCUCUUUUAGACUGGAGGCGCCGCGAGGUUUCUGUUAU
Por-5-1   UGGGAAAGC...GCUUG...........GAAUUUCC.GGCAGUCUGGCAGUGUUAAUAGCC..UU..UUCU..........UUGGCC.GGAUA.AGGCGGUUAGUG.AAUUUGUCGGACAACAGGACAAAAGCUGUGGAUGGUCUCUUUUAGACUGGAGGCGCCGCGAGGUUUCUGUUAU
env-413   UGGGAAAGC...GCUUG...........GAAUUUCC.GGCAGUCUGGCAGUGUUAAUAGCC..UU..UUCU..........UUGGCC.GGAUA.AGGCGGUUAGUG.AAUCUGUCGGACAACAGAACAAAAGCUGUGGAUGGUCUCUUUUAGACUG........................
env-414   UGGGAAAGC...GCUUG...........GAAUUUCC.GGCAGUCUGGCAGUGUUAAUAGCC..UU..UUCU..........UUGGCC.GGAUA.AGGCGGUUAGUG.AAUCUGUCGGACAACAGAACAAAAGCUGUGGAUGGUCUCUUUUAGACUGGAGGCGCCGCGAGGUUUCUGUUAU
env-418   UGGGAAAGC...GCUUG...........GAAUUUCC.GGCAGUCUGGCAGUGUUAAUAGCC..UU..UUCU..........UUGGCC.GGAUA.AGGCGGUUAGUG.AAUCUGUCGGACAACAGAACAAAAGCUGUGGAUGGUCUCUUUUA.............................
env-419   UGGGAAAGC...GCUUG...........GAAUUUCC.GGCAGUCUGGCAGUGUUAAUAGCC..UU..UUCU..........UUGGCC.GGAUA.AGGCGGUUAGUG.AAUCUGUCGGACAACAGAACAAAAGCUGUGGAUGGUCUCUUUUA.............................
env-420   UGGGAAAGC...GCUUG...........GAAUUUCC.GGCAGUCUGGCAGUGUUAAUAGCC..UU..UUCU..........UUGGCC.GGAUA.AGGCGGUUAGUG.AAUCUGUCGGACAACAGAACAAAAGCUGUGGAUGGUCUCUUUUAGACUGGAGGCGCCGCGAGGUCUUUGUUAU
env-421   UGGGAAAGC...GCUUG...........GAAUUUCC.GGCAGUCUGGCAGUGUUAAUAGCC..UU..UUCU..........UUGGCC.GGAUA.AGGCGGUUAGUG.AAUCUGUCGGACAACAGAACAAAAGCUGUGGAUGGUCUCUUUUAGACUGGAGGCGCCGCGAGGUUUCUGUUAU
env-422   UGGGAAAGC...GCUUG...........GAAUUUCC.GGCAGUCUGGCAGUGUUAAUAGCC..UU..UUCU..........UUGGCC.GGAUA.AGGCGGUUAGUG.AAUCUGUCGGACA............................................................
env-423   UGGGAAAGC...GCUUG...........GAAUUUCC.GGCAGUCUGGCAGUGUUAAUAGCC..UU..UUCU..........UUGGCC.GGAUA.AGGCGGUUAGUG.AAUCUGUCGGACAACAGAACAAAAGCUGUGGAUGGUCUCUUUUA.............................
Psp-8-1   UUGAGAAAGC..GCUUA..........GAAUUCUCA..GCAGUUCAGUGGUGUUAAUAGCG..UU..CUC..........UCUGGCC.UGAUG.ACGCGGUUAGUG.AAUCUGCUGAUCAGCAAAGCAAAAGCUGUGGAUGGUCUCUUUUAGACUGGAGGCGCCGCGAGGUCUUUGUUAU
env-425   UUGAGAAAGC..GCUUA..........GAAUUCUCA..GCAGUUCAGUGGUGUUAAUAGCG..UU..CUCU..........CCGGCC.AGAUG.ACGCGGUUAGUG.AAUCUGCUGAUCAGCAAAGCAAAAGCUGUGGAUGGUCUCUUUUAGACUGGAGGCGCCGCGAGGUCUUUGUUAU
env-426   UUGAGAAAGC..GCUUA..........GAAAUCUCA..GCAGUUCAGUGGUGUUAAUAGCG..UU..CUCU..........CUGGCC.AGAUG.ACGCGGUUAGUG.AAUCUGCUGAUCAGCAAAGCAAAAGCUGUGGAUGGUCUCUUUUAGA...........................
env-429   UUGAGAAAGC..GCUUA..........GAAAUCUCA..GCAGUUCAGUGGUGUUAAUAGCG..UU..CUCU..........CUGGCC.AGAUG.ACGCGGUUAGUG.AAUCUGCUGAUCAGCAAAGCAAAAGCUGUGGAUGGUCUCUUUUAGACUGGAGGCGCCGCGAGGUCUUUGUUAU
env-431   UUGAGAAAGC..GCUUA..........GAAAUCUCA..GCAGUUCAGUGGUGUUAAUAGCG..UU..CUCU..........CUGGCC.AGAUG.ACGCGGUUAGUG.AAUCUGCUGAUCAGCAAAGCAAAAGCUGUGGAUGGUCUCUUUUAGACUGGAGGCGCCGCGAGGUCUUUGUUAU
env-432   UUGAGAAAGC..GCUUA..........GAAAUCUCA..GCAGUUCAGUGGUGUUAAUAGCG..UU..CUCU..........CUGGCC.AGAUG.ACGCGGUUAGUG.AAUCUGCUGAUCAGCAAAGCAAAAGCUGUGGAUGGUCUCUUUUAGACUGGAGGCGCCGCGAGGUCUUUGUUAU
env-433   UUGAGAAAGC..GCUUA..........GAAAUCUCA..GCAGUUCAGUGGUGUUAAUAGCG..UU..CUCU..........CUGGCC.AGAUG.ACGCGGUUAGUG.AAUCUGCUGAUCAGCAAAGCAAAAGCUGUGGAUGGUCUCUUUUAGACUGGAGGCGCCGCGAGGUC........
env-434   UUGAGAAAGC..GCUUA..........GAAAUCUCA..GCAGUUCAGUGGUGUUAAUAGCG..UU..CUCU..........CUGGCC.AGAUG.ACGCGGUUAGUG.AAUCUGCUGAUCAGCAAAGCAAAAGCUGUGGAUGGUCUCUUUUAGA...........................
env-435   UUGAGAAAGC..GCUUA..........GAAAUCUCA..GCAGUUCAGUGGUGUUAAUAGCG..UU..CUCU..........CUGGCC.AGAUG.ACGCGGUUAGUG.AAUCUACUGAUCAGCAAAGCAAAAGCUGUGGAUGGUCUCUUUUAGACUGGAGGCGCCGCGAGGUCUUUGUUAU
env-436   UUGAGAAAGC..GCUUA..........GAAUUCUCA..GCGGUUCAGUGGUGUUAAUAGCG..UU..CUCU..........CUGGCC.AGAUG.ACGCGGUUAGUG.AAUCUGCUGAUCAGCAAAGCAAAAGCUGUGGAUGGUCUCUUUUAGACUGGAGGCGCCGCGAGGUCUUUGUUAU
env-437   UUGAGAAAGC..GCUUA..........GAAUUCUCA..GCGGUUCAGUGGUGUUAAUAGCG..UU..CUCU..........CUGGCC.AGAUG.ACGCGGUUAGUG.AAUCUGCUGAUCAGCAAAGCAAAAGCUGUGGAUGGUCUCUUUUAGACUGGAGGCGCCGCGAGGUCUUUGUUAU
env-438   UUGAGAAAGC..GCUUA..........GAAUUCUCA..GCAGUUCAGUGGUGUUAAUAGCG..UU..CUCU..........CUGGCC.AGAUG.ACGCGGUUAGUG.AAUCUGCUGAUCAGCAAAGCAAAAGCUGUGGAUGGUCUCUUUUAGACUGGAGGCGCCGCGAGGUCUUUGUUAU
env-439   UUGAGAAAGC..GCUUA..........GAAUUCUCA..GCAGUUCAGUGGUGUUAAUAGCG..UU..CUCU..........CUGGCC.AGAUG.ACGCGGUUAGUG.AAUCUGCUGAUCAGCAAAGCAAAAGCUGUGGAUGGUCUCUUUUAGACUGGAGGCGCCGCGAGGUCUUUGUUAU
env-440   UUGAGAA.....AGUGCUUAGAA.......UUCUCA..GCAGUUCAGUGGUGUUAAUAGCG..UU..CUCU..........CUGGCC.AGAUG.ACGCGGUUAGUG.AAUCUGCUGAUCAGCAAAGCAAAAGCUGUGGAUGGUCUCUUUUAGACUGGAGGCGCCGCGAGGUCUUUGUUAU
env-441   UUGAGAA.....AGUGCUUAGAA.......UUCUCA..GCAGUUCAGUGGUGUUAAUAGCG..UU..CUCU..........CUGGCC.AGAUG.ACGCGGUUAGUG.AAUCUGCUGAUCAGCAAAGCAAAAGCUGUGGAUGGUCUCUUUUA.............................
env-442   UUGAGAA.....AGUGCUUAGAA.......UUCUCA..GCAGUUCAGUGGUGUUAAUAGCG..UU..CUCU..........CUGGCC.AGAUG.ACGCGGUUAGUG.AAUCUGCUGAUCAGCAAAGCAAAAGCUGUGGAUGGUCUCUUUUAGACUGGAGGCGCCGCGAGGUCU.......
env-443   UUGAGAA.....AGUGCUUAGAA.......UUCUCA..GCAGUUCAGUGGUGUUAAUAGCG..UU..CUCU..........CUGGCC.AGAUG.ACGCGGUUAGUG.AAUCUGCUGAUCAGCAAAGCAAAAGCUGUGGAUGGUCUCUUUUAGA...........................
Pou-1-1   UUGAGAA.....AGUGCUUAGAA.......UUCUCA..GCAGUUCAGUGGUGUUAAUAGCG..UU..CUCU..........CUGGCC.AGAUG.ACGCGGUUAGUG.AAUCUGCUGAUCAGCAAAGCAAAAGCUGUGGAUGGUCUCUUUUAGACUGGAGGCGCCGCGAGGUCUUUGUUAU
env-444   UUGAGAA.....AGUGCUUAGAA.......UUCUCA..GCAGUUCAGUGGUGUUAAUAGCG..UU..CUCU..........CUGGCC.AGAUG.ACGCGGUUAGUG.AAUCUGCUGAUCAGCAAAGCAAAAGCUGUGGAUGGUCUCUUUUA.............................
env-445   UUGAGAA.....AGUGCUUAGAA.......UUCUCA..GCAGUUCAGUGGUGUUAAUAGCG..UU..CUCU..........CUGGCC.AGAUG.ACGCGGUUAGUG.AAUCUGCUGAUCAGCAAAGCAAAAGCUGUGGAUGGUCUCUUUUAGACUGGAGGCGCCGCGAGGUCUUUGUUAU
env-446   UUGAGAA.....AGUGCUUAGAA.......UUCUCA..GCAGUUCAGUGGUGUUAAUAGCG..UU..CUCU..........CUGGCC.AGAUG.ACGCGGUUAGUG.AAUCUGCUGAUCAACAAAGCAAAAGCUGUGGAUGGUCUCUUUUAGACUGGAGGCGCCGCGAGGUCUUUGUUAU
env-447   UUGAGAA.....AGUGCUUAGAA.......UUCUCA..GCAGUUCAGUGGUGUUAAUAGCG..UU..CUCU..........CUGGCC.AGAUG.ACGCGGUUAGUG.AAUCUGCUGAUCAGCAAAGCAAAAGCUGUGGAUGGUCUCUUUUAGACUGGAGGCGCCGCGAGGUCUUUGUUAU
Pou-2-1   UUGAGAA.....AGUGCUUAGAA.......UUCUCA..GCAGUUCAGUGGUGUUAAUAGCG..UU..CUCU..........CUGGCC.AGAUG.ACGCGGUUAGUG.AAUCUGCUGAUCAGCAAAGCAAAAGCUGUGGAUGGUCUCUUUUAGACUGGAGGCGCCGCGAGGUCUUUGUUAU
env-448   UUGGGG......AACACUUAGAA........CCCCA....CGUACAGAAGUGUUAAUAGCG..GU..GCUU..........UUGGCC.GAACA.UCGUGGUUAGUG.AAUCGUCUGUACAAGCUUGAUAAAAAAAAACGUUUUUUAGUAGUAAAUUUUCAAAAUUAGAAUGAACACAUUAA
env-449   UGGGAAUAC...GCUUAG..........G.AUUCUC..AUAGCGUGGUCGUGUUAAUAGUU..CU..UCUU..........UUGGCC.GAGGA.GAGCGGUUAGUG.AAUCGGCCGUCGCAUCAGAGAUAAUGCUGUGGAUGGUCUCUUUUAGACUGGAGG...................
Pba-2-1   UGGGAAUAC...GCUUAG..........G.AUUCUC..AUAGCGUGGUCGUGUUAAUAGUU..CU..UCUU..........UUGGCC.GAGGA.GAGCGGUUAGUG.AAUCGGCCGUCGCAUCAGAGAUAAUGCUGUGGAUGGUCUCUUUUAGACUGGAGGCGCCGCGAGGUCUCUGUUA
Pba-1-1   UGGGAAUAC...GCUUAG..........G.AUUCUC..AUAGCGUGGUCGUGUUAAUAGUU..CU..UCUU..........UUGGCC.GAGGA.GAGCGGUUAGUG.AAUCGGCCGUCGCAUCAGAGAUAAUGCUGUGGAUGGUCUCUUUUAGACUGGAGGCGCCGCGAGGUCUCUGUUA
env-450   UGGGAAUAC...GCUUAG..........G.AUUCUC..AUAGCGCGGUCGUGUUAAUAGCU..UU..UCUU..........UUGGCC.GAGAA.GAGCGGUUAGGG.AAUCGGCUGUCGCGUCAGAGAUAAUGCUGUGGAUGGUCUCUUUUAGACUGGAGGCGCCGCGAGGUCUCUGUUA
Pde-2-1   UGGAGGUU....GACUUAG..........AGCCUCC....GGCACGUGCGUGUUAAUAGUGU.CA..GCUU..........UUGGCC.GAGCU.GUACGGUUAGUG.AAUCUCACGGGCAACAAAAGAAAACGUUUUUUAGUAGUAAAAUUAUUAAUUUUUUAGAAUGGACACUUUAAGU
Pbe-1-1   CAGGGGC.....ACGCUUAGA.........GCCCCU.....CGCACCUUGUGUUAAUAGUG..AU..GCCU..........UUGGCC.GGGCA.UCACGGUUAGUG.AAUCAAGGGUGCAGCAGAGCGAAAGCCAUGGAUGGUCUCUUUUAGACUGGAGGCGCCGUGAGGUCUCUGUAUA
env-451   AAGGGG......AAACCUUAGAA........CCCCU.....UUGCGAGAGUGUUAAUAGUCA.AU..AGCU...........UGGCC.GGCUU.UGACGGUUAGUG.AAUCGCUCGCAAGAAAAAAUUAAGUUUUUUAGUAGUAAAAUUAAAAUUUUAAAUGGACACUUUAAGUUACAAG
env-452   UGGGGG......AAGGCUUAGAG........CCCCC....AGCCCGGGAGUGUUAAUAGUG..CU..GCA...........UUGGCC.AUGCA.CCACGGUUAGUG.AAUCGCCCGUGCAAAGUUCAAGAGCCUGAAGCGAGAAGUUUCAGGUGUGCGUUCUUGAGCGUCAGCCCGAGUC
env-453   GGGGGGU.....GGCUUAGA..........GCCCCC...GCUUGCGGAAGUGUUAAUAGUA..GU..GUUU..........UUGGCC.GGACA.UUACGGUUAGUG.AAUCGACCGCAAAUAAAGAGAAAAAACGUUUUUUAGUAGUAAUUACAAACAUUAAAAUGAACACUUUAAGUUA
env-454   UGGAGGUU....AACUUAG..........GACCUCC..GCGUACAGUUCGUGUUAAUAGUC..AC..AUCU..........UUGGCC.AGGUG.UGACGGUUAGUG.AAUCGGCUGUCGAAGAAAAACGUUUUUUAGUAGUAUUACAAUUUUAAAAUUGAAAAUGGACACUUUAAGUUAC
env-455   UGGAGGUU....AACUUAG..........GACCUCC..GCGUACAGUUCGUGUUAAUAGUC..AU..AUCU..........UUGGCC.AGGUG.UGACGGUUAGUG.AAUCGGCUGUCGAAGAAAAACGUUUUUUAGUAGUAUUACAAUUUUAAAAUUGAAAAUGGACACUUUAAGUUAC
env-457   CGGGGGU.....AACUUAGG..........GCCUCC....AUACAGUUCGUGUUAAUAGUU..GU..ACUU..........UUGGCC.GAGUG.CAGCGGUUAGUG.AAUCGGCUGUAAAAAUAAGAUAUAAACGUUUUUUAGUAGUAUUACAAUUUUAAUUUAAAAUGGACACUUUAAG
env-458   CGGGGGU.....AACUUAGG..........GCCUCC...ACUACAGUUCGUGUUAAUAGUU..GU..ACUU..........UUGGCC.GAGUG.CAACGGUUAGUG.AAUCGGCUGUAAAAAC.........................................................
env-462   CGGGGGU.....AACUUAGG..........GCCUCC...ACUACAGUUCGUGUUAAUAGUU..GU..ACUU..........UUGGCC.GAGUG.CAACGGUUAGUG.AAUCGGCUGUAAAACAAGAUAUAAACGUUUUUUAGUAGUAUUACAAUUUUAAUUUAAAAUGGACACUUUAAGU
env-463   CGGGGGU.....AACUUAGG..........GCCUCC...ACUACAGUUCGUGUUAAUAGUU..GU..ACUU..........UUGGCC.GAGUG.CAACGGUUAGUG.AAUCGGCUGUAAAACAAGAUAUAAACGUUUUUUAGUAGUAUUACAAUUUUAAUUUAAAAUGGACACUUUAAGU
env-464   CGGGGGU.....AACUUAGG..........GCCUCC...ACUACAGUUCGUGUUAAUAGUU..GU..ACUU..........UUGGCC.GAGUG.CAACGGUUAGUG.AAUCGGCUGUAAAAACAAGAUAUAAACGUUUUUUAGUAGUAUUACAAUUUUAAUUUAAAAUGGACACUUUAAG
env-466   UGAGGGCAC...GCUUA...........GAGCCCUC......GUCGGGCGUGUUAAUAGCGA.CU..GUC.........AUUUGGCCUGGACA.GCGUGGUUAGAG.AAUCGCUCGAAGACAUUCGAACACCGCAGGGGAACGCCGUUUCUCAUGCAGGUUAUUUGUGUGUCAUCACCAA
env-467   CUGGGG......AACACUUAGAGC.......CCCCA.....CGGACGUAGUGUUAAUAGUCG.CU..GUCU..........UUGGCC.GGGCA.CGACGGUUAGUG.AAUCGACGUCCAAACGGCAGAAAGCACACAUGCCAUAUAUAUUAAUGUAUGACAAAGUCUGUAAAAAGGCGUG
env-468   CGAGGGAGU...ACUUA...........GGUCCCUC....AGAAGCGGUGUGUUAAUAGUG..AU..GCUU..........UUGGCC.AGGCA.UGACGGUUAGUG.AAUCACGGCUUCACGACACAGAAAAUUGUUUUUUAGUAGUAAAUUUAAAAUUUGAAAUGAACACUUUAAG...
env-469   CGGGGGAGU...ACUUAG..........GC.CCCCC....AGGAGCGACGUGUUAAUAGCU..GU..GCUU..........UUGGCC.AGGCA.UAGUGGUUAGUG.AAUCGAAGCCCCAACGAAGAAAAAACUGUUUUUUAGUAGUAAACCAAAAGAAAAUGAACACUUUAAGUUACAA
env-470   UUGGGUAC....ACUUA............GAGCCCA..GUUUGGAGGCAGUGUUAAUAGUC..GA..GCGU..........UUGGCC.GCGC..CGAUGGUUAGUG.AAUCUGCGUUCAGCAAAAAUGUUUUUUAGUAGUAAUAAAAUUUUCUAAAAGUCAAGAAUGGACACUUUAAGUU
Ppa-3-1   AGGGG.......CGACUCUUAGAAA.......CCCC.GGUAGUGCAAUGGUGUUAAUAGCU..UU..ACUU..........UUGGCC.GAGUA.UCGCAGUUAGUG.AAUCUAUUGCACGGCAGAGAAAAAGCUAUGGAUGGUCUCUUUUAGACUGGAGGCGCCGUGAGGUCUCUGUUAU
env-471   AGGGG.......CGACUCUUAGAAA.......CCCC.AGUAGUGCAAUGGUGUUAAUAGCC..UU..ACUU..........UUGGCC.GAGUA.UCGCAGUUAGUG.AAUCUAUUGCACGGCAGAGAAAAAGCUAUGGAUGGUCUCUUUUAGACUGGAGGCGCCGUGAGGUCUCUGUUAU
env-473   AGGGG.......CGACUCUUAGAAA.......CCCC.AGUAGUGCAAUGGUGUUAAUAGCU..UU..ACUU..........UUGGCC.GAGUA.UCGCAGUUAGUG.AAUCUAUUGCACGGCAGAGAAAAAGCUUUGGAUGGUCUCUUUUAGACUGGAGGCGCCGAGAGGUCUCUGUUAU
env-474   AGGGG.......CGACUCUUAGAAA.......CCCC.AGUAGUGCAAUGGUGUUAAUAGCU..UU..ACUU..........UUGGCC.GAGUA.UCGCAGUUAGUG.AAUCUAUUGCACGGCAGAGAAAAAGCUUUGGAUGGUCUCUUUUAGACUGGAGGCGCCGAGAGGUCUCUGUUAU
env-476   AGGGGCGACU..CUU............AGGAACCCC.AGUAGUGCAAUGGUGUUAAUAACU..UU..ACUU..........UUGGCC.GAGUA.UCGCAGUUAGUG.AAUCUAUUGCACGGCAGAGCAAAAGCUUUGGAUGGUCUCUUUUAGACUGGAGGCGCCGAGAGGUCUCUGUUAU
env-477   AGGGGCGACU..CUU............AGGAACCCC.AGUAGUGCAAUGGUGUUAAUAACU..UU..ACUU..........UUGGCC.GAGUA.UCGCAGUUAGUG.AAUCUAUUGCACGGCAGAGCAAAAGCUUUGGAUGGUCUCUUUUAGACUGGAGGCGCCGAGAGGUCUCUGUUAU
env-483   AUGGGG......GAAGUCUUAGAA.......CCCCA.....UGGAUGCCGUGUUAAUAGCG..AU..GCCU..........UUGGCC.GGGCA.UGGCGGUUAGUG.AAUCGGCAUUCACGAGACGCAUCGCAUAAAGACCGGCGAGUGCCUUCCAAGACUAAAAGGCAUAGUCUCGAUA
env-485   AUGGGG......GAAGUCUUAGAA.......CCCCA.....UGGAUGCCGUGUUAAUAGCU..GU..GCCU..........UUGGCC.GGGCA.UGGCGGUUAGUG.AAUCGGCAUUCACGAGACGCAUCGCAUAAAGACCGGCGAGUGCCUUCCAAGACUAAAAGGCAUAGUCUCGAUA
env-486   AGGGGGU.....AAAUCCUUAGA.......GCCCCC....AGCGUGGCCGUGUUAAUAGUG..GU..GUCU..........UUGGCC.GGGCA.UCAUGGUUAGUG.AAUCUGCUGCGCAGUCAUCGAGGUUUUGUAAGCGGGAGCACCCAGUGGCUCGCUUGCUUGUUUGUGUGCGCUG
env-487   AGGGGA......AAACACUUAGAA.......UCCCC...GUGGACUAGAGUGUGAAUAGCA..AC..GUCU..........UUGGCC.GGACG.UCGUGGUUCGGG.AAUCGCUGGUUCAACCAUCCGCAACACGCCUGUUUACAGGCCCCGCGGAGCGCACGUUAGACUAACAACCGGG
env-488   AGGGGAAACU..CUU............AGAAUCCCC...GUGAACAGGGGUGUGAAUAGCG..GC..GUCU..........UUGGCC.GGGCG.AAGCGGUUCGUG.AAUCGUCUGUUCAAGCACAAGUUAGACUAACAGCCGGGAGUUAGACCUCGGAUGGGAGUCAAUGUGCGUACAU

          .<<<<<<<<<<<.............>>>>>>>>>>>................<<<<<.<<<..<<..<<<<<................>>>>>>.>>>>.>>>>>............................................................................
          .222222.1111.............1111.222222................02200.122..22..2222.................22222.2221.00220............................................................................
          ●GGGG●○-----R●●●○○○○----------○●CCCC----○○●●●●●●YGUGU●AAUAGY●--●Y--RYYU----------UUGGCC-GRRY●-●●RYGGUU●GUG-AAUCR●●●●●●●RR●●R●●●●●●●●●●●●●●●●●●●●●●●●●●●●●●●●●●●●●●●●●●●●●●●●●●●●●●●●
           5′                                                                                                             3′
```

```
env-489   UGGGGUACU...CUU.............AGAACCCC.....GGAGAAGCGUGUUAAUAGUC..GU..GCUU.........UUGGCC.GGGCA.CGAUGGUUAGGG.AAUCGCCCUCCAAAGUAUGAAGAAAUUGUUUUUUAGUAGUAAAAUUUUUAAAUCGAAAUGAACACAUUAAGUU
env-490   GGGGAUUUU...CUU.............AGAAUCCC....GUACGUAACAUGUUAAUAGCA..UG..GUUU.........UUGGCC.GAGCU.GUGUGGUUAGGUAAAGCAUGUGCGUGAGUUAAUAAAAACGUUUUUUAGUAGUAAAUUUAAUUAAAAAAAUGAACACUUUAAGUUAC
env-491   GGGGAUUUU...CUU.............AGAAUCCC....GUACGUAACAUGUUAAUAGCA..UG..GUUU.........UUGGCC.GAGCU.GUGUGGUUAGGUAAAGCAUGUGCGUGAAUUAAUAAAAACGUUUUUUAGUAGUAAAUUUAAUUAAAAAAAUGAACACUUUAAGUUAU
env-492   GGGGAUUUU...CUU.............AGAAUCCC....GUACGUAACAUGUUAAUAGCA..UG..GUUU.........UUGGCC.GAGCU.GUGUGGUUAGGUAAAGCAUGUGCGUGAGUUAAUAAAAACGUUUUUUAGUAGUAAAUUUAAUUAAAAAAAUGAACACUUUAAGUUAC
env-493   GGGGAUUUU...CUU.............AGAAUCCC....GUACGUAACAUGUUAAUAGCA..UG..GUUU.........UUGGCC.GAGCU.GUGUGGUUAGGUAAAGCAUGUGCGUGAAUUAAUAAAAACGUUUUUUAGUAGUAAAUUUAAUUAAAAAAAUGAACACUUUAAGUUAC
Pam-1-1   GGGGAUUUU...CUU.............AGAAUCCC....GUACGUAACAUGUUAAUAGCA..UG..GUUU.........UUGGCC.GAGCU.GUGUGGUUAGGUAAAGCAUGUGCGUGAAUUAAUAAAAACGUUUUUUAGUAGUAAAUUUAAUUAAAAAAAUGAACACUUUAAGUUAC
Pam-3-1   GGGGAUUUU...CUU.............AGAAUCCC....GUACGUAACAUGUUAAUAGCA..UG..GUUU.........UUGGCC.GAGCU.GUGUGGUUAGGUAAAGCAUGUGCGUGAAUUAAUAAAAACGUUUUUUAGUAGUAAAUUUAAUUAAAAAAAUGAACACUUUAAGUUAC
Pbi-1-1   GGGGAUUUU...CUU.............AGAAUCCC....AUACGUUACGUGUUAAUAACA..UA..GUCU.........UUGGCC.GGGCU.AUGUGGUUAGGAAAAGCAUGUGCGUGAAUUAAUAAAAACGUUUUUUAGUAGUAAAAUUUAAUUUUUAAAAUGGACACUUUAAGUUA
Pve-1-1   AGGGGUACU...CUU.............AGAGCCCC....AUACGAUGCAUGUUAAUAGUG..UU..GUCU.........UUGGCC.GGGCG.ACAUGGUUAGGUAAGGCAUGUUCGUGAAUAGAAAACGUUUUUUAGUAGUAAAAUUAUUAAUUUUUUAGAAUGGACACUUUAAGUUA
Pve-3-1   AGGGGUACU...CUU.............AGAGCCCC....AUACGAUACAUGUUAAUAGUG..UU..GUCU.........UUGGCC.GGGCG.ACAUGGUUAGGAAAAGCAUGAUCGUGAAAUAGAAAACGUUUUUUAGUAGUAAAAUUAUUAAUUUUUUAGAAUGGACACUUUAAGUU
env-494   AGGGGUACU...CUU.............AGAGCCCC....AUACGAUGCAUGUUAAUAGUG..UU..GUCU.........UUGGCC.GGGCG.ACAUGGUUAGGGAAAGCAUGAUCGUGAAAUAGAAAACGUUUUUUAGUAGUAAAAU...............................
env-495   CGGGG.......AAACACUUAGAA........CCCC....AUACGAUACGUGUUAAUAGUG..UU..GUCU.........UUGGCC.GGACA.GCAUGGUUAGGUCAAGCACAGUCGUAGAAAUAGAAAACGUUUUUUAGUAGUAAAAUUAUUAAUUUUUUAGAAUGAACACUUUAAGU
env-497   UGGGG.......AUACACUUAGAA........CCCC....AUACGAUACGUGUUAAUAGUG..UU..GUCU.........UUGGCC.GGGCA.GCAUGGUUAGGUCAAGCACAGUCGUAAAAAUAGAAAACGUUUUUUAGUAGUAAAAUUAUUAAUUUUUUAGAAUGGACACUUUAAGU
Psc-1-1   AGGGG.......GAAUUCUUAGAA........CCCC....AUACGAUACGUGUUAAUAGUG..UU..GUCU.........UUGGCC.GGGCA.AUAUGGUUAGGAAAAGCACAAUCGUAGAAAUAGAAAACGUUUUUUAGUAGUAAAAUUAUUAAUUUUUUAGAAUGGACACUUUAAGUU
env-498   AGGGG.......GAAUUCUUAGAA........CCCC....AUACGAUACGUGUUAAUAGUG..UU..GUCU.........UUGGCC.GGGCA.AUAUGGUUAGGAAAAGCACAAUCGUAGAAAUAGAAAACGUUUUUUA.........................................
Psp-6-1   AGGGG.......GAAUUCUUAGAA........CCCC....AUACGAUGCGUGUUAAUAGUG..UU..GUCU.........UUGGCC.GGGCA.ACAUGGUUAGGAAAAGCACAGUCGUAGAAACAGAAAACGUUUUUUAGUAGUAAAAUUAUUAAUUUUUUUGAAUGGACACUUUAAGU
Pme-3-1   AGGGG.......AAUUUUCUUAGAA.......CCCC....AUACGAUACGUGUUAAUAGUG..UU..GUCU.........UUGGCC.GGGCA.GCAUGGUUAGGUCAAGCAUUGUCGUAAAAAUAGAAAACGUUUUUUAGUAGUAAAAUUAUUAAUUUUUUAGAAUGGACACUUUAAGU
env-501   AGGGGU......AAUCUUAGA..........GCCCC....AUACGAUUCAUGUUAAUAGUG..UU..GUCU.........UUGGCC.GGGCA.GCAUGGUUAGGUCAAGCACAGUCGUAGAAAUAGAAAACGUUUUUUAGUAGUAAAAUUAUUAAUUUUUUAGAAUGGACACUUUAAGU
env-502   AGGGGU......AAUCUUAGA..........GCCCC....AUACGAUUCAUGUUAAUAGUG..UU..GUCU.........UUGGCC.GGGCA.GCAUGGUUAGGU.AAACACAUGAUCGUAAAAUAGAAAACGUUUUUUAGUAGU..................................
env-503   AGGGGU......AAUCUUAGA..........GCCCC....AUACGAUUCAUGUUAAUAGUG..UU..GUCU.........UUGGCC.GGGCG.GCAUGGUUAGGU.AAACACAUGAUCGUAAAAUAGAAAACGUUUUUUAGUAGU..................................
env-504   AGGGGU......AAUCUUAGA..........GCCCC....AUACGAUUCAUGUUAAUAGUG..UU..GUCU.........UUGGCC.GGGCG.GCAUGGUUAGGU.AAACACAUGGUCGUAAAAUAGAAAACGUUUUUUAGUAGUAAAAUUAUUAAUUUUUUAGAAUGGACACUUUAAG
env-507   AGGGGU......AAUCUUAGA..........GCCCC....AUACGAUUCAUGUUAAUAGUG..UU..GUCU.........UUGGCC.GGGCG.GCAUGGUUAGGU.AAACACAUGGUCGUAAAAUAGAAAACGUUUUUUAGUAGU..................................
Phi-2-1   AGGGGU......AAUCUUAGA..........GCCCC....AUACGAUUCAUGUUAAUAGUG..UU..GUCU.........UUGGCC.GGGCG.GCAUGGUUAGGU.AAACACAUGGUCGUAAAAUAGAAAACGUUUUUUAGUAGUAAAAUUAUUAAUUUUUUAGAAUGGACACUUUAAG
env-508   AGGGGU......AAUCUUAGA..........GCCCC....AUACGAUUCAUGUUAAUAGUG..UU..GUCU.........UUGGCC.GGGCG.GCAUGGUUAGGU.AAACACAUGGUCGUAAAAUAGAAAACGUUUUUUAGUAGUAAAAUUAUUAAUUUUUUAGAAUGGACACUUUAAG
env-509   AGGGGU......AAUCUUAGA..........GCCCC....AUACGAUUCAUGUUAAUAGUG..UU..GUCU.........UUGGCC.GGGCG.GCAUGGUUAGGU.AAACACAUGGUCGUAAAAUAGAAAACGUUUUUUAGUAGUAAAAUUAUUAAUUUUUUAGAANNNNNNNNNAGCC
Phi-2-2   AGGGGU......AAUCUUAGA..........GCCCC....AUACGAUUCAUGUUAAUAGUG..UU..GUCU.........UUGGCC.GGGCG.GCAUGGUUAGGU.AAACACAUGGUCGUUAAAUAGAAAACGUUUUUUAGUAGUAAAAUUAUUAAUUUUUUAGAAUGGACACUUUAAG
env-510   GGGGGAUUU...UCUU............AGAACCCC....AUAUGAUACGUGUUAAUAGUG..UU..GUCU.........UUGGCC.GGGCA.GCAUGGUUAGGUCAAGCACAGUCGUAAAAAUAGAAAACGUUUUUUAGUAGUAAAAUUAUUAAUUUUUUAGAAUGGACACUUUAAGU
env-511   GGGGGAUUU...UCUU............AGAGCCCC....AUACGAUACGUGUUAAUAGUG..UU..GUCU.........UUGGCC.GGGCA.GCAUGGUUAGGUCAAGCACAGUCGUAAAAACAGAAAACGUUUUUUAGUAGUAAAAUUAUUAAUUUUUUAGAAUGGACACUUUAAGU
env-512   GGGGGAUUU...UCUU............AGAACCCC....AUACGAUACGUGUUAAUAGUG..UU..GUCU.........UUGGCC.AGGCA.GCAUGGUUAGGUCAAGCACAGUCGUAAAAAUAGAAAACGUUUUUUAGUAGUAAAAUUAUUAAUUUUUUAGAAUGGACACUUUAAGU
env-514   GGGGGAUUU...UCUU............AGAACCCC....AUACGAUACGUGUUAAUAGUG..UU..GUCU.........UUGGCC.GGGCA.GCAUGGUUAGGACAAGCACCGUCGUAGAAAUAGAAAACGUUUUUUAGUAGUAAAAUUAUUAAUUUUUUAGAAUGGACACUUUAAGU
env-516   GGGGGAUUU...UCUU............AGAACCCC....AUACGAUACGUGUUAAUAGUG..UU..GUCU.........UUGGCC.GGGCA.GCAUGGUUAGGACAAGCACAGUCGUAGAAAUAGAAAACGUUUUUUAGUAGUA.................................
env-517   GGGGGAUUU...UCUU............AGAACCCC....AUACGAUUCGUGUUAAUAGUG..UU..GUCU.........UUGGCC.GGGCA.GCAUGGUUAGGACAAGCACAGUCGUAGAAAUAGAAAACGUUUUUUAGUAGUAAAAUUAUU.........................
env-519   GGGGGAUUU...UCUU............AGAACCCC....AUACGAUACGUGUUAAUAGUG..UU..GUCU.........UUGGCC.GGACA.GCAUGGUUAGGUAAAGCACAGUCGUAGAAAUAGAAAACGUUUUUUAGUAGUAAAAUUAUUAAUUUUUUAGAAUGGACACUUUAAGU
Psp-5-1   CGGGGAU.....ACACUUAG..........AACCCC....AUACGAUACGUGUUAAUAGUG..UU..GUCU.........UUGGCC.GGACA.GCAUGGUUAGGUCAAGCACAGUCGUAGAAAUAGAAAACGUUUUUUAGUAGUAAAAUUAUUAAUUUUUUAGAAUGGACACUUUAAGU
env-520   CGGGGAU.....ACACUUAG..........AACCCC....AUACGAUACGUGUUAAUAGUG..UU..GUCU.........UUGGCC.GGACA.GCAUGGUUAGGUCAAGCACAGUCGUAGAAAUAGAAAACGUUUUUUAGUAGUAAAAUUAUUAAUUUUUUAGAAUGAACACUUUAAGU
env-521   CGGGGAU.....ACACUUAG..........AACCCC....AUACGAUACGUGUUAAUAGUG..UU..GUCU.........UUGGCC.GGACA.GCAUGGUUAGGUCAAGCACAGUCGUAGAAAUAGAAAACGUUUUUUAGUAGUAAAAUUAUUAAUUUUUUAGAAUGAACACUUUAAGU
env-522   CGGGGAU.....ACACUUAG..........AACCCC....AUACGAUACGUGUUAAUAGUG..UU..GUCU.........UUGGCC.GGACA.GCAUGGUUAGGUCAAGCACAGUCGUAGAAAUAGAAAACGUUUUUUAGUAGUAAAAUUAUUAAUUUUUUAGAAUGAACACUUUAAGU
env-523   GGGGGAU.....UUUCUUAG..........AACCCC....AUACGAUACGUGUUAAUAGUG..CU..GUCU.........UUGGCC.GGGCA.GCAUGGUUAGGUCAAGCACAGUCGUAGAAAUAGAAAACGUUUUUUAGUAGUAAAAUUAUUAAUUUUUUAGAAUGGACACUUUAAGU
env-525   GGGGGAU.....UUUCUUAG..........AACCCC....AUACGAUACGUGUUAAUAGUG..CU..GUCU.........UUGGCC.GGGCA.GCAUGGUUAGGUCAAGCACAGUCGUAGAAAUAGAAAACGUUUUUUAGUAGUNNNNNNNNNNNNNNNNNNNNNNNNNNNNNNNNNNU
env-526   GGGGGAU.....UUUCUUAG..........AACCCC....AUACGAUACGUGUUAAUAGUG..UU..GUCU.........UUGGCC.GGGCA.GCAUGGUUAGGUCAAGCACAGUCGUAAAAAUAGAAAACGUUUUUUAGUAGUAAAAUUAUUAAUUUUUUAGAAUGGACACUUUAAGUU
env-527   GGGGGAU.....UUUCUUAG..........AACCCC....AUACGAUACGUGUUAAUAGUG..UU..GUCU.........UUGGCC.GGGCA.GCAUGGUUAGGUCAAGCACAGUCGUAAAAAUAGAAAACGUUUUUUAGUAGUAAAAUUAUUAAUUUUUUAGAAUGGACACUUUAAGU
env-531   GGGGGAU.....UUUCUUAG..........AACCCC....AUACGAUACGUGUUAAUAGUG..UU..GUCU.........UUGGCC.GGGCA.GCAUGGUUAGGUCAAGCACAGUCGUAAAAAUAGAAAACGUUUUUUAGUAGUAAAAUUAUUAAUUUUUUAGAAUGGACACUUUAAGU
env-532   GGGGGAU.....UUUCUUAG..........AACCCC....AUACGAUACGUGUUAAUAGUG..UU..GUCU.........UUGGCC.GGGCA.GCAUGGUUAGGUCAAGCACAGUCGUAAAAAUAGAAAACGUUUUUUAGUAGUAAAAUUAUUAAUUUUUUAGAAUGGACACUUUAAGUU
env-534   GGGGGAU.....UUUCUUAG..........AACCCC....AUACGAUACGUGUUAAUAGUG..UU..GUCU.........UUGGCC.GGGCA.GCAUGGUUAGGUCAAGCACAGUCGUAAAAAUAGAAAACGUUUUUUAGUAGUAAAAUUAUUAAUUUUUUAAAAUGGACACUUUAAGU
env-535   GGGGGAU.....UUUCUUAG..........AACCCC....AUACGAUACGUGUUAAUAGUG..UU..GUCU.........UUGGCC.GGGCA.GCAUGGUUAGGUCAAGCACAGUCGUAAAAAUAGAAAACGUUUUUUAGUAGUAAAAUUAUUAUUUUUUUAGAAUGGACACUUUAAGU
env-537   GGGGGAU.....UUUCUUAG..........AACCCC....AUACGAUACGUGUUAAUAGUG..UU..GUCU.........UUGGCC.GGGCA.GCAUGGUUAGGUCAAGCACAGUCGUAAAAAUAGAAAACGUUUUUUAGUAGUAAAAUUAUUAAUUUUUUAGAAUGGACACUUUAAGU
env-538   GGGGGAU.....UUUCUUAG..........AACCCC....AUACGAUACGUGUUAAUAGUG..UU..GUCU.........UUGGCC.GGGCA.GCAUGGUUAGGUCAAGCACAGUCGUAAAAAUAGAAAACGUUUUUUAGUAGUAAAAUUAUUAAUUUUUUAGAAUGGACACUUUAAGU
env-552   GGGGGAU.....UUUCUUAG..........AACCCC....AUACGAUACGUGUUAAUAGUG..UU..GUCU.........UUGGCC.GGGCA.GCAUGGUUAGGUCAAGCACAGUCGUAAAAAUAGAAAACGUUUUUUAGUAGUAAAAUUAUUAAUUUUUUAGAAUGGACACUUUAAGUU
env-553   GGGGGAU.....UUUCUUAG..........AACCCC....AUACGAUACGUGUUAAUAGUG..UU..GUCU.........UUGGCC.GGGCA.GCAUGGUUAGGUCAAGCACAGUCGUAAAAAUAGAAAACGUUUUUUAGUAGUA.................................
env-554   GGGGGAU.....UUUCUUAG..........AACCCC....AUACGAUACGUGUUAAUAGUG..UU..GUCU.........UUGGCC.GGGCA.GCAUGGUUAGGUCAAGCACAGUCGUAAAAAUAGAAAACGUUUUUUAGUAGUAAAAUUAUUAAUUUUUUAGAAUGGACACUUUAAGU
env-555   GGGGGAU.....UUUCUUAG..........AACCCC....AUACGAUACGUGUUAAUAGUG..UU..GUCU.........UUGGCC.GGGCA.GCAUGGUUAGGUCAAGCACAGUCGUAGAAAUAGAAAACGUUUUUUAGUAGUAAAAUUAUUAAUUUUUUAGAAUGGACACUUUAAGU
env-557   GGGGGAU.....UUUCUUAG..........AACCCC....AUACGAUACGUGUUAAUAGUG..UU..GUCU.........UUGGCC.GGGCA.GCAUGGUUAGGUCAAGCACAGUCGUAAAAAUAGAAAACGUUUUUUAGUAGUAAAAUUAUUAAUUUUUUAGAAUGGACACUUUAAGU
env-558   GGGGGAU.....UUUCUUAG..........AACCCC....AUACGAUACGUGUUAAUAGUG..UU..GUCU.........UUGGCC.GGGCA.GCAUGGUUAGGUCAAGCACAGUCGUAAAAAUAGAAAACGUUUUUUAGUAGUAAAAUUAUUAAUUUUUUAGAAUGGACANNNNNNNN
env-559   GGGGGAU.....UUUCUUAG..........AACCCC....AUACGAUACGUGUUAAUAGUG..UU..GUCU.........UUGGCC.GGGCA.GCAUGGUUAGGUCAAGCACAGUCGUAAAAAUAGAAAACGUUUUUUAGUAGUAAAAUUAUUAUUUUUUUAGAAUGGACACUUUAAGU
env-560   GGGGGAU.....UUUCUUAG..........AACCCC....AUACGAUACGUGUUAAUAGUG..UU..GUCU.........UUGGCC.GGGCA.GCAUGGUUAGGUCAAGCACAGUCGUAAAAAUAGAAAACGUUUUUUAGUAG..................................
env-561   GGGGGAU.....UUUCUUAG..........AACCCC....AUACGAUACGUGUUAAUAGUG..UU..GUCU.........UUGGCC.GGGCA.GCAUGGUUAGGUCAAGCACAGUCGUAAAAAUAGAAAACGUUUUUUAGUAGUAAAAUUAUUAAUUUUUUAAAAUGGACACUUUAAGU
Pfu-1-1   AGGGGU......AGUCUUAGA..........ACCCC....GUACGAUGCGUGUUAAUAGUG..UU..GUCU.........UUGGCC.GGGCA.GCAUGGUUAGGAAAGGCACAGUCGUAAAACAGAAAACGUUUUUUAGUAGUAAAAUUAUUAAUUUUUUAGAAUGGACACUUUAAGUU
Psp-24-1  UAGGGGU.....ACUCUUAGA........GCCCCA.....UACGAAUCAUGUUAAUAGUG..UU..GUCU.........UUGGCC.GGGCG.ACAUGGUUAGGCAAAGCAUGUUCGUGAAAUAGAAAACGUUUUUUAGUAGUAAAAUUAUUAAUUUUUUAAAAUGGACACUUUAAGUU
env-562   UAGGGGU.....ACUCUUAGA........GCCCCA.....UACGAAUCAUGUUAAUAGUG..UU..GUCU.........UUGGCC.GGGCG.ACAUGGUUAGGCAAAGCAUGUUCGUGAAAUAGAAAACGUUUUUUAGUAGU..................................
env-563   UAGGGGU.....ACUCUUAGA........GCCCCA.....UACGAAUCAUGUUAAUAGUG..UU..GUCU.........UUGGCC.GGGCG.ACAUGGUUAGGCAAAGCAUGUUCGUGAAAUAGAAAACGUUUUUUAGUAGU..................................
env-564   UAGGGGUACU..CUU............AGAGCCCCU.....UACGAAUCAUGUUAAUAGUG..UU..GUCU.........UUGGCC.GGGCG.ACAUGGUUAGGCAAAGCAUAUUCGUGAAAUAGAAAACGUUUUUUAGUAGUAAAAUUAUUAAUUUUUUAAAAUGGACACUUUAAGUU
Pde-3-1   UGGGGUAC....ACUUA............GAACCCC...CGUACGGUCUGUGUUAAUAGUG..CU..GUCU.........UUGGCC.GGGCA.GCAUGGUUAGGUUAAGCGCAGCCGUAAAAAAGAAAACGUUUUUUAGUAGUAAAAUUAUUAAUUUUCAAGAAUGGACACUUUAAGUU
env-565   UGGGGUAC....ACUUA............GAACCCC...CGUACGGUCUGUGUUAAUAGUG..CU..GUCU.........UUGGCC.GGGCA.GCAUGGUUAGGUCAAGCGCAGCCGUAAAAAGAAAACGUUUUUUAGUAGUAAAAU...............................
Pde-4-1   UGGGGUAC....ACUUA............GAACCCC...CGUACGGUCUGUGUUAAUAGUG..CU..GUCU.........UUGGCC.GGGCA.GCAUGGUUAGGUCAAGCGCAGCCGUAAAAAAGAAAACGUUUUUUAGUAGUAAAAUUAUUAAUUUUUAAGAAUGGACACUUUAAGUU
Pde-5-1   UGGGGUAC....ACUUA............GAACCCC...CGUACGGUCUGUGUUAAUAGUG..CU..GUCU.........UUGGCC.GGGCA.GCAUGGUUAGGUCAAGCGCAGCCGUAAAAAGAAAACGUUUUUUAGUAGUAAAAUUAUUAAUUUUUAAGAAUGGACACUUUAAGUUA
env-566   UGGGGUAC....ACUUA............GAACCCC...CGUACGGUCUGUGUUAAUAGUG..CU..GUCU.........UUGGCC.GGGCA.GCAUGGUUAGGUCAAGCGCAGCCGUAAAAAGAAAACGUUUUUUAGUAGUAAAUUUAUUAAUUUUUAAGAAUGGACACUUUAAGUUA
env-567   UGGGGUAC....ACUUA............GAACCCC...UGUACGGUCUGUGUUAAUAGUG..CU..GUCU.........UUGGCC.GGGCA.GCAUGGUUAGGUCAAGCGCAGCCGUAAAAAAGAAAACGUUUUUUAGUAGUAAAAUUAUUAAUUUUUAAGAAUGGACACUUUAAGUU
Pde-6-1   UGGGGUAC....ACUUA............GAACCCC...CGUACGGUCUGUGUUAAUAGUG..CU..GUCU.........UUGGCC.GGGCA.GCAUGGUUAGGUCAAGUGCAGCCGUAAAAAGAAAACGUUUUUUAGUAGUAAAAUUAUUAAUUUUUAAGAAUGGACACUUUAAGUUA
env-568   UGUGGGUAC...ACUUA...........GAGCCC.C....GUACGGCUCGUGUUAAUAGUG..CU..GUCU.........UUGGCC.GGGCA.GCAUGGUUAGGACAAGCACGGUCGUAGAAAGAAAACGUUUUUUAGUAGUAAAAU...............................
env-569   UGUGGGUAC...ACUUA...........GAGCCC.C....GUACGGCUCGUGUUAAUAGUG..CU..GUCU.........UUGGCC.GGGCA.GCAUGGUUAGGACAAGCACGGUCGUAGAAAGAAAACGUUUUUUAGUAGUAAAAUUAUUAAUUUUUUAGAAUGGACACUUUAAGUUA
Pmu-1-1   UGUGGGUAC...ACUUA...........GAGCCC.C....GUACGGCUCGUGUUAAUAGUG..CU..GUCU.........UUGGCC.GGGCA.GCAUGGUUAGGACAAGCACGGUCGUAGAAAGAAAACGUUUUUUAGUAGUAAAAUUAUUAAUUUUUUAGAAUGGACACUUUAAGUUA
env-570   AGGGGCACU...CUU.............AGAACCCC....UGUGUCGUUGUGUUAAUAGCGC.UU..GCUU..........UGGCC.GAGCA.UCGUGGUUAGUG.AAUCAAUGGCA..............................................................
env-571   UUGGGGCAC...GCUUA...........GAGCCCCA...AGACAUGGUUGUGUUAAUAGCG..UC..GCCU.........UUGGCC.GGACG.GCGCGGUUAGUG.AAUCAGCCGUGCGGCAGAGCGAAAGCCAUGGAUGGUCUCUUUUAGACUGGAG....................
env-572   UUGGGGCAC...GCUUA...........GAGCCCCA...AGGCAUGGUUGUGUUAAUAGCG..UC..GCCU.........UUGGCC.GGACG.GCGUGGUUAGUG.AAUCAAUCGUGUAGCAGAGCGAAAGCUAUGGAUGGUCUCUUUUAGACUGGAGGCGCCGUGAGGUCUCUGCUGG
env-573   UUGGGGCAC...GCUUA...........GAGCCCCA...AGGCAUGGUUGUGUUAAUAGCG..UC..GCCU.........UUGGCC.GGACG.GCGUGGUUAGUG.AAUCAAUCGUGUAGCAGAGCGAAAGCUAUGGAUGGUCUCUUUUAGA...........................
env-574   CGGGGCAC....GCUUA............GAGCCCC...ACGCACAGCAGUGUUAAUAGCG..GC..GCCU.........UUGGCC.GGACG.UCGUGGUUAGUG.AAUCGGCCGUGCAGCAGAGCUAACGCCAUGGAUGGUCUCUUUUAGACUGGAGGCGCCGUGAGGUCUCUGUCAA
env-575   UGGGGCAC....GCUUA............GAGUCCC....UUGCGCAUUGUGUUAAUAGUG..GU..GCCU.........UUGGCC.GGACA.UCGUGGUUAGUG.AAUCAAUGGCGCGACAGAGCGAAAGCCAUGGAUGGUCUCUUUUAGACUGGAGGCGCCGUGAGGUCUCUGUUUU
env-576   UGGGGCAC....GCUUA............GAGUCCC....UUGCGCGUUGUGUUAAUAGCG..AU..GCCU.........UUGGCC.GGACA.UCGUGGUUAGUG.AAUCAAUGGCGCGACAGAGCGAAAGCCAUGGAUGGUCUCUUUUAGACUGGAGGCGCCGUGAGGUCUCUGUUUU
env-577   UGGGGU......GAACUUAGA..........GCCCC...GUGGAUGUGCGUGUUAAUAGCC..GU..GCCU.........UUGGCC.GGACA.UGGUGGUUAGUG.AAUCGUCGUCCGCAGAGAAAAUGUUAAUUAACAAAAGGUUUUUUAGUAGUUUUUUAUUUAGAAAUGAACACUU
env-578   UGAGGGC.....AGUCUUAGA.........GCCCUU....GGCACAUACGUGUUAAUAGUGUCAU..GCUU.........UUGGCC.GAGCA.ACAUGGUUAGUG.AAUCGUAUGAGCAAAAGAAUUAGAAAACGUUUUUUAGUAGUAAAAUUUAAUUUCACAGAAUGGACACUUUAAG
env-579   UGAGGGC.....AGUCUUAGA.........GCCCUU....GGCACAUACGUGUUAAUAGUGUCAU..GCUU.........UUGGCC.GAGCA.ACAUGGUUAGUG.AAUCGUAUGAGCAAAAGAAUUAGAAAACGUUUUUUAGUAGUAAAA...........................
env-580   UGAGGGC.....AGUCUUAGA.........GCCCUU....GGCACAUGCGUGUUAAUAGUGUCAU..GCUU.........UUGGCC.GAGCA.ACAUGGUUAGUG.AAUCGUAUGAGCAAAAGAAUUAGAAAACGUUUUUUAGUAGUAAAAUUUAAUUUCACAGAAUGGACACUUUAAG
env-582   UGAGGGC.....AGUCUUAGA.........GCCCUU....AGCACAUGCGUGUUAAUAGUGUCAU..GCUU.........UUGGCC.GAGCA.ACAUGGUUAGUG.AAUCGUAUGAGCAAAAGAAUUAGAAAACGUUUUUUAGUAGUAAAAUUUAAUUUCACAGAAUGGACACUUUAAG

          .<<<<<<<<<<<.............>>>>>>>>>>>................<<<<<.<<<..<<..<<<<<..............>>>>>>.>>>>.>>>>>...........................................................................
          .222222.1111.............1111.222222................02200.122..22..2222...............22222.2221.00220...........................................................................
          ●GGGG●○-----R●●●○○○○----------○●CCCC----○○●●●●●●YGUGU●AAUAGY●--●Y--RYYU---------UUGGCC-GRRY●-●●RYGGUU●GUG-AAUCR●●●●●●●RR●●R●●●●●●●●●●●●●●●●●●●●●●●●●●●●●●●●●●●●●●●●●●●●●●●●●●●●●●●●
           5′                                                                                                         3′
```

```
Pin-1-1  CGAGGG......UGAGCUUAGAU........CCCUC...AGCACGUUGUGUGUUAAUAGUG..UU..GCUU..........UUGGCC.GAGCA.GCAUGGUUAGUG.AAUCCGACGGGCAAGAAUUAAAAACGUUUUUUAGUAGUAAAAUUUUAAAAUUAAAAAUGAACACUUUAAGUUA
env-583  CGAGAGU....GAACUUAGA.........ACUCUC...AGCACAUUGUGUGUUAAUAGCG..UU..ACUU..........UUGGCC.GGGUU.ACGUGGUUAGAG.AAU.GCAAUGAGCAAGUAUUAAAAACGUUUUUUAGUAGUAAAAUUUAAAAUUUAAAAAUGGACACUUUAAGUU
env-589  CGAGAGU....GAACUUAGA.........ACUCUC...AGCACAUUGUGUGUUAAUAGCG..UU..GCUU..........UUGGCC.GGGUU.ACGUGGUUAGAG.AAU.GCAAUGAGCAAGUAUUAAAAACGUUUUUUAGUAGUAAAAUUUAAAAUUUAAAAAUGGACACUUUAAGUU
env-592  CGAGAGU....GAACUUAGA.........ACUCUC...AGCACAUUGUGUGUUAAUAGCG..UU..GCUU..........UUGGCC.GGGUU.ACGUGGUUAGAG.AAU.GCAAUGAGCAAGUAUUAAAAACGUUUUUUAGUAGUAAAAUUUAAAANAGGAAACAGCUAAGAAAGAGUG
env-593  CGAGAGU....GAACUUAGA.........ACUCUC...AGCACAUUGUGUGUUAAUAGCG..UU..GCUU..........UUGGCC.GGGUU.ACGUGGUUAGAG.AAU.GCAAUGAGCAAGUAUUAAAAACGUUUUUUAGUAGUAAAAUUUAAAAUUUAAAAAUGGACACUUUAAGUU
env-594  CGAGAGU....GAACUUAGA.........ACUCUC...AGCACAUUGUAUGUUAAUAGCG..UU..ACUU..........UUGGCC.GGGUU.ACGUGGUUAGAG.AAU.GCAAUGAGCAAGUAUNNNNNNNNNNNNNNAGGAAACAGCUAAGAAAGAGUGGGUAGUAGUAGAUGCUAC
env-595  CGAGAGU....GAACUUAGA.........ACUCUC...AGCACAUUGUAUGUUAAUAGCG..UU..ACUU..........UUGGCC.GGGUU.ACGUGGUUAGAG.AAU.GCAAUGAGCAAGUAUUAAAAACGUUUUUUAGUAGUAAAAUUUAAAAUUUAAAAAUGGACACUUUAAGUU
env-596  CGAGAGU....GAACUUAGA.........ACUCUC...AGCACAUUGUAUGUUAAUAGCG..UU..ACUU..........UUGGCC.GGGUU.ACGUGGUUAGAG.AAU.GCAAUGAGCAAGUAUUAAAAACGUUUUUUAGUAGUAAAAUUUAAAAUUUAAAAAUGGACACUUUAAGUU
env-597  CGAGAGU....GAACUUAGA.........ACUCUC...AGCACAUUGUAUGUUAAUAGCG..UU..ACUU..........UUGGCC.GGGUU.ACGUGGUUAGAG.AAU.GCAAUGAGCAAGUAUNAGGAAACAGCUAAGAAAGAGUGGGUAGUAGUAGAUGCUACAGACCAGAUAGUU
env-598  AGAGAGU....GAACUUAGA.........ACUCUC...AGCACAUUGUAUGUUAAUAGCG..UU..ACUU..........UUGGCC.GGGUU.ACGUGGUUAGAG.AAU.GCAAUGAGCAAGUA..............................................................
env-599  CGAGAGU....GAACUUAGA.........ACUCUC...AGCACAUUGUAUGUUAAUAGCG..UU..ACUU..........UUGGCC.GGGUU.ACGUGGUUAGAG.AAU.GCAAUGAGCAAGUAUUAAAAACGUUUUUUAGUAGUAAAAUUUAAAAUUUAAAAAUGGACACUUUAAGUU
env-600  CGAGAGU....GAACUUAGA.........ACUCUC...AGCACAUUGUAUGUUAAUAGCG..UU..ACUU..........UUGGCC.GGGUU.ACGUGGUUAGAG.AAU.GCAAUGAGCAAGUAUUAAAAACGUUUUUUAGUAGUAAAAUUUAAAAUUUAAAAAUGGACACUUUAAGUU
env-613  CGAGAGU....GAACUUAGA.........ACUCUC...AGCACAUUGUAUGUUAAUAGCG..UU..ACUU..........UUGGCC.GGGUU.ACGUGGUUAGAG.AAU.GCAAUGAGCAAGUAUUAAAAACGUUUUUUAGUAGUAAAAUUUAAAAUUUAAAAAUGGACACUUUAAGUU
env-648  CGAGAGU....GAACUUAGA.........ACUCUC...AGCACAUUGUAUGUUAAUAGCG..UU..ACUU..........UUGGCC.GGGUU.ACGUGGUUAGAG.AAU.GCAAUGAGCAAGUAUUAAAAACGUUUUUUAGUAGUAAAAUUUAAAAUUUAAAAAUGGACACUUUAAGUU
env-651  CGAGAGU....GAACUUAGA.........ACUCUC...AGCACAUUGUAUGUUAAUAGCG..UU..ACUU..........UUGGCC.GGGUU.ACGUGGUUAGAG.AAU.GCAAUGAGCAAGUAUUAAAAACGUUUUUUAGUAGUAAAAUUUAAAAUUUAAAAAUGGACACUUUAAGUU
env-652  CGAGAGU....GAACUUAGA.........ACUCUC...AGCACAUUGUAUGUUAAUAGCG..UU..ACUU..........UUGGCC.GGGUU.ACGUGGUUAGAG.AAU.GCAAUGAGCAAGUAUUAAAAACGUUUUUUAGUAGUNAGUAGUAGAUGCUACAGACCAGAUAGUUGGUCG
env-653  CGAGAGU....GAACUUAGA.........ACUCUC...AGCACAUUGUAUGUUAAUAGCG..UU..ACUU..........UUGGCC.GGGUU.ACGUGGUUAGAG.AAU.GCAAUGAGCAAGUAUUAAAAACGUUUUUUAGUAGU.................................
env-657  CGAGAGU....GAACUUAGA.........ACUCUC...AGCACAUUGUAUGUUAAUAGCG..UU..ACUU..........UUGGCC.GGGUU.ACGUGGUUAGAG.AAU.GCAAUGAGCAAGUAU...........................................................
env-676  UGAGGGU....GUACUUAGA.........GCCCUC...UGCACGAUGUGUGUUAAUAGUAG.UG..GCUU..........UUGGCC.GAGCC.GCACGGUUAGAG.AAUCACACGGGCAAAAUUAGAAAACGUUUUUUAGUAGUAAAAUUAUUAAUUUUUUAGAAUGGACACUUUAAGU
Pco-3-1  CGAGGGU....GUACUUAGA.........GCCCUC...UGCACGAUGUGUGUUAAUAGUAG.UG..GCUU..........UUGGCC.GAGCC.GCACGGUUAGAG.AAUCACACGGGCAAAAUUAGAAAACGUUUUUUAGUAGUAAAAUUAUUAAUUUUUUAGAAUGGACACUUUAAGU
Pni-1-1  CGAGGGU....GAACUUAGA.........GCCCUC...AGCACGUUGUGUGUUAAUAGCG..CU..GCUU..........UUGGCC.AGGCU.GCGUGGUUAGUG.AAUCCGACGGGCAAGCAUUAAAAACGUUUUUUAGUAGUAAAAUUUUAAAAUUAAAAAUGGAUACUUUAAGUUA
env-677  CGAGGGU....GAACUUAGA.........ACCCUC...AGCACGUUGUGUGUUAAUAGCG..CU..GCUU..........UUGGCC.GGGCU.GCGUGGUUAGUG.AAUCCGACGGGCAAGCAUUAAAAACGUUUUUUAGUAGUAAAAUUUUAAAAUUAAAAAUGGAUACUUUAAGUUA
env-679  CGAGGGU....GAACUUAGA.........GCCCUC...AGCACGUUGUGUGUUAAUAGCG..CU..GCUU..........UUGGCC.GGGCU.GUGUGGUUAGUG.AAUCCGACGGGCAAGCAUUAAAAACGUUUUUUAGUAGUAAAAUUUUAAAAUUAAAAAUGGAUACUUUAAGUUA
env-680  CGAGGGU....GAACUUAGA.........GCCCUC...AGCACGUUGUGUGUUAAUAGCG..CU..GCUU..........UUGGCC.GGGCU.GCGUGGUUAGUG.AAUCCGACGGGCAAGCAUUAAAAACGUUUUUUAGUAGUAAAAUUUUAAAAUUAAAAAUGGACACUUUAAGUUA
env-681  CGAGGGU....GAACUUAGA.........GCCCUC...AGCACGUUGUGUGUUAAUAGCG..CU..GCUU..........UUGGCC.GGGCU.GCGUGGUUAGUG.AAUCCGACGGGCAAGCAUUAAAAACGUUUUUUAGUAGUAAAAUUUUAAAAUUAAAAAUGGACACUUUAAGUUA
env-682  CGAGGGU....GAACUUAGA.........GCCCUC...AGCACGUUGUGUGUUAAUAGCG..CU..GCUU..........UUGGCC.GGGCU.GCGUGGUUAGUG.AAUCCGACGGGCAAGCAUUAAAAACGUUUUUUAGUAGUAAAAUUUUAAAAUUAAAAAUGGAUACUUUAAGUUA
env-683  CGAGGGU....GAACUUAGA.........GCCCUC...AGCACGUUGUGUGUUAAUAGCG..CU..GCUU..........UUGGCC.GGGCU.GCGUGGUUAGUG.AAUCCGACGGGCAAGCAUUAAAAACGUUUUUUAGUAGUAAAAUUUUAAAAUUAAAAAUGGAUACUUUAAGUUA
env-684  CGAGGGU....GAACUUAGA.........GCCCUC...AGCACGUUGUGUGUUAAUAGCG..CU..GCUU..........UUGGCC.GGGCU.GCGUGGUUAGUG.AAUCCGACGGGCAAGCAUUAAAAACGUUUUUUAGUAGUAAAAUUUUAAAAUUAAAAAUGGAUACUUUAAGUUA
env-686  CGAGGGU....GAACUUAGA.........GCCCUC...AGCACGUUGUGUGUUAAUAGCG..CU..GCUU..........UUGGCC.GGGCU.GCGUGGUUAGUG.AAUCCGACGGGCAAGCAUUAAAAACGUUUUUUAGUAGUAAAAUUUUAAAAUUAAAAAUGGAUACUUUAAGUUA
env-696  CGAGGGU....GAACUUAGA.........GCCCUC...AGCACGUUGUGUGUUAAUAGCG..CU..GCUU..........UUGGCC.GGGCU.GCGUGGUUAGUG.AAUCCGACGGGCAAGCAUUAAAAACGUUUUUUAGUAGUAAAAUUUUAAAAUUAAAAAUGGAUACUUUAAGUUA
env-698  CGAGGGU....GAACUUAGA.........GCCCUC...AGCACGUUGUGUGUUAAUAGCG..CU..GCUU..........UUGGCC.GGGCU.GCGUGGUUAGUG.AAUCCGACGGGCAAGCAUUAAAAACGUUUUUUA.........................................
env-699  CGAGGGU....GAACUUAGA.........GCCCUC...AGCACGUUGUGUGUUAAUAGCG..CU..GCUU..........UUGGCC.GGGCU.GCGUGGUUAGUG.AAUCCGACGGGCAAGCAUUAAAAACGUUUUUUAGUAGUAAAAUUUUAAAAUUAAAAAUGGAUACU........
Pni-3-1  CGAGGGU....GAACUUAGA.........GCCCUC...AGCACGUUGUGUGUUAAUAGCG..CU..GCUU..........UUGGCC.GGGCU.GCGUGGUUAGUG.AAUCCGACGGGCAAGCAUUAAAAACGUUUUUUAGUAGUAAAAUUUUAAAAUUAAAAAUGGAUACUUUAAGUUA
env-700  CGAGGGU....GAACUUAGA.........GCCCUC...AGCACGUUGUGUGUUAAUAGCG..CU..GCUU..........UUGGCC.GGGCU.GCGUGGUUAGUG.AAUCCGACGGGCAAGCAUUAAAAACGUUUUUUAGUAGUAAA................................
env-709  CGAGGGU....GAACUUAGA.........GCCCUC...AGCACGUUGUGUGUUAAUAGUG..CC..GCUU..........UUGGCC.GGGCU.GCAUGGUUAGUG.AAUCCAACGAGCAAGAAUUAAAAACGUUUUUUAGUAGUAAA................................
env-710  CGAGGGU....GAACUUAGA.........GCUCUC...AGCACGUUGUGUGUUAAUAGUG..CC..GCUU..........UUGGCC.GGGCU.GCAUGGUUAGUG.AAUCCAACGAGCAAGAAUUAAAAACGUUUUUUAGUAGUAAAAUUUUAAAAUUAAAAAUGAACACUUUAAGUUA
env-711  CGAGGGU....GAACUUAGA.........GCUCUC...AGCACGUUGUGUGUUAAUAGUG..CC..GCUU..........UUGGCC.GGGCU.GCAUGGUUAGUG.AAUCCAACGAGCAAGAAUUAAAAACGUUUUUUAGUAGUAAAAUUUUAAAAUUAAAAAUGAACACUUUAAGUUA
env-724  CGAGGGU....GAACUUAGA.........GCUCUC...AGCACGUUGUGUGUUAAUAGUG..CC..GCUU..........UUGGCC.GGGCU.GCAUGGUUAGUG.AAUCCAACGAGCAAGAAUUAAAAACGUUUUUUAGUAGUAAAAUUUUAAAAUUAAAAAUGAACACUUUAAGUUA
Pau-1-1  CGAGGGU....GAACUUAGA.........GCUCUC...AGCACGUUGUGUGUUAAUAGUG..CC..GCUU..........UUGGCC.GGGCU.GCAUGGUUAGUG.AAUCCAACGAGCAAGAAUUAAAAACGUUUUUAGUAGUAAAAUUUUAAAAAUUAAAAAAUUGAAACACAUUUA
Psp-9-1  CGAGGGU....GUACUUAGA.........GCCCUC...AGCACGUGGUGUGUUAAUAGUG..CU..GCUU..........UUGGCC.GAGCU.GCACGGUUAGUG.AAUCACACGGGCAAAGAACAAAAACGUUUUUUAGUAGUAAAAUUAAAAUUUAAAAAUGGACACUUUAAGUUAC
env-725  CGAGGGU....GAACUUAGA.........GCCCUC...AGCACGUUGUGUGUUAAUAGUG..UU..GCUU..........UUGGCC.GAGCA.GUAUGGUUAGUG.AAUCCGACGGGCAAGAAUUAAAAACGUUUUUUAGUAGUAAAAUUUUAAAAUUAAAAAUGAACACUUUAAGUUA
env-726  CGAGGGU....GAACUUAGA.........GCCCUC...AGCACGUUGUGUGUUAAUAGUG..UU..GCUU..........UUGGCC.GAGCA.GUAUGGUUAGUG.AAUCCGACGGGCAAGAAUUAAAAACGUUUUUUAGUAGUAAAAUUUUAAAAUUAAAAAUGGAUACUUUAAGUUA
env-727  CGAGGGU....AAGCUUAGA.........GCCCUC...AGCACGUUGUGUGUUAAUAGUG..UU..GCUU..........UUGGCC.GAGCU.GCAUGGUUAGUG.AAUCCGACGGGCAAGAAUUAAAAACGUUUUUUAGUAGUAAA................................
env-729  CGAGGGU....GAGCUUAGA.........GCCCUC...GGCACGUUGUGUGUUAAUAGUG..UU..GCUU..........UUGGCC.GAGCU.GCAUGGUUAGUG.AAUCCGACGGGCAAGAAUUAAAAACGUUUUUUAGUAGUAAAAUUUUAAAAUUAAAAAUGAACACUUUAAGUUA
env-733  CGAGGGU....GAGCUUAGA.........GCCCUC...AGCACGUUGUGUGUUAAUAGUG..UU..GCUU..........UUGGCC.GAGCU.GCAUGGUUAGUG.AAUCCGACGGGCAAGAAUUAAAAACGUUUUUUAGUAGUAAAAUUUUAAAAUUAAAAAUGAACACUUUAAGUUA
env-734  CGAGGGU....GAGCUUAGA.........GCCCUC...AGCACGUUGUGUGUUAAUAGUG..UU..GCUU..........UUGGCC.GAGCU.GCAUGGUUAGUG.AAUCCGACGGGCAAGAAUUAAAAACGUUUUUUAGUAGUAAAAUUUUAAAAUUAAAAAUGAACACUUUAAGUUA
env-735  CGAGGGU....GAGCUUAGA.........GCCCUC...AGCACGUUGUGUGUUAAUAGUG..UU..GCUU..........UUGGCC.GAGCU.GCAUGGUUAGUG.AAUCCGACGGGCAAGAAUUAAAAACGUUUUUUAGUAGUAAAAUUUUAAAAUUAAAAAUGAACACUUUAAGUUA
env-736  CGAGGGU....GAGCUUAGA.........ACCCUC...AGCACGUUGUGUGUUAAUAGUG..UU..GCUU..........UUGGCC.GAGCU.GCAUGGUUAGUG.AAUCCGACGGGCAAGAAUUAAAAACGUUUUUUAGUAGUAAAAUUUUAAAAUUAAAAAUGAACACUUUAAGUUA
Pin-3-1  CGAGGGU....GAACUUAGA.........GCCCUC...AGCACGUUGUGUGUUAAUAGUG..UU..GCUU..........UUGGCC.GAGCU.GCAUGGUUAGUG.AAUCCGACGGGCAAGAAUUAAAAACGUUUUUUAGUAGUAAAAUUUUAAAAUUAAAAAUGAAUACUUUAAGUUA
Pfa-1-1  CGAGGGU....GAACUUAGA.........GCCCUC...AGCACGUUGUGUGUUAAUAGUG..UU..GCUU..........UUGGCC.GAGCU.GCAUGGUUAGUG.AAUCCGACGGGCAAGAAUUAAAAACGUUUUUAGUAGUAAAAUUUUAAAAUUAAAAUGAACACUUUAAGUUAU
env-737  CGAGGGU....GAACUUAGA.........GCCCUC...AGCACGUUGUGUGUUAAUAGUG..UU..GCUU..........UUGGCC.GAGCU.GCAUGGUUAGUG.AAUCCGACGGGCAAGAAUUAAAAACGUUUUUUAGUAGUAAAAUUUUAAAAUUAAAAAUGAACACUUUAAGUUA
env-738  CGAGGGU....GAACUUAGA.........GCCCUC...AGCACGUUGUGUGUUAAUAGUG..UU..GCUU..........UUGGCC.GAGCU.GCACGGUUAGUG.AAUCCGACGGGCAAGAAUUAAAAACGUUUUUUAGUAGUAAAAUUUUAAAAUUAAAAAUGAACACUUUAAGUUA
env-742  CGAGGGU....GAACUUAGA.........GCCCUC...AGCACGUUGUGUGUUAAUAGUG..UU..GCUU..........UUGGCC.GAGCU.GCACGGUUAGUG.AAUCCGACGGGCAAGAAUUAAAAACGUUUUUUAGUAGUAAAAUUUUAAAAUUAAAAAUGGAUACUUUAAGUUA
Pdi-3-1  CGAGGGU....GAACUUAGA.........ACCCUC...AGCUCGUUGUGUGUUAAUAGUG..UA..ACUU..........UUGGCC.GAGUC.GCAUGGUUAGUG.AAUCCAAUGAGCAAGAAUUAAAAACGUUUUUUAGUAGUAAAAUUUUAAAAUUUAAAUGAACACUUUAAGUUAC
env-743  CGAGGGU....GAACUUAGA.........ACCCUC...AGCUCGUUGUGUGUUAAUAGUG..UA..ACUU..........UUGGCC.GAGUC.GCAUGGUUAGUG.AAUCCAAUGAGCAAGAAU..............................................
Pdi-2-1  CGAGGGU....GAACUUAGA.........ACCCUC...AGCUUGUUGUGUGUUAAUAGUG..UA..ACUU..........UUGGCC.GAGUC.GCAUGGUUAGUG.AAUCCAAUGAGCAAGAAUUAAAAACGUUUUUUAGUAGUAAAAUUUUAAAAUUUAAAUGAACACUUUAAGUUAC
Pdi-1-1  CGAGGGU....GAACUUAGA.........ACCCUC...AGCUUGUUGUGUGUUAAUAGUG..UA..ACUU..........UUGGCC.GAGUC.GCAUGGUUAGUG.AAUCCAAUGAGCAAGAAUUAAAAACGUUUUUUAGUAGUAAAAUUUUAAAAUUUAAAAUGAACACUUUAAGUUAC
env-744  CGGGGAU....ACACUUAGA.........GUCCCC...UGGGUAUGGUGUGUUAAUAGCA..CA..UCCU..........GUGGCC.GGGAU.UUGCGGUUAGUG.AAUCACCUAUCCGAAAUUAAGUGAGUAAGAAAAGAUUAAAACGUUUUUUAGUAGUAAAUUUAAAUUUUAAAAU
env-745  CGGGGAU....ACACUUAGA.........GUCCCC...UGGGUAUGGUGUGUUAAUAGCA..CA..UCCU..........GUGGCC.GGGAU.UUGCGGUUAGUG.AAUCACCUAUCCGAAAUUAAGUGAGUAAAAAAAGAUUAAAACGUUUUUUAGUAGUAAAUUUAAAUUUUAAAAUG
env-746  CGGGGAU....ACACUUAGA.........GUCCCC...UGGGUAUGGUGUGUUAAUAGCA..CA..UCCU..........GUGGCC.GGGAU.UUGCGGUUAGUG.AAUCACCUAUCCGAAAAUUAAGUGAGUAAGAAAAGAUUAAAACGUUUUUUAGUAGUAAAUUUAAAUUUUAAAA
env-747  CGGGGAU....ACACUUAGA.........GUCCCC...UGGGUAUGGUGUGUUAAUAGCA..CA..UCCU..........GUGGCC.GGGAU.UUGCGGUUAGUG.AAUCACCUAUCCGAAAAUUAAGUGAGUAAGAAAAGAUUAAAACGUUUUUUAGUAGUAAAUUUAAAUUUUAAAA
env-748  CGGGGAU....ACACUUAGA.........GUCCCC...UGGGUAUGGUGUGUUAAUAGCA..CA..UCCU..........GUGGCC.GGGAU.UUGCGGUUAGUG.AAUCACCUAUCCGAAAUUAAGUGAGUAAGAAAAGAUUAAAACGUUUUUUAGUAGUAAAUUUAAAUUUUAAAAU
env-749  CGGGGAU....ACACUUAGA.........GUCCCC...UGGGUAUGGUGUGUUAAUAGCA..CA..UCCU..........GUGGCC.GGGAU.UUGCGGUUAGUG.AAUCACCUAUCCGAAAUUAAGUGAGUAAGAAAAGAUUAAAACGUUUUUUAGUAGUAAAUUUAAAUUUUAAAAU
env-751  CGGGGAU....ACACUUAGA.........GUCCCC...UGGGUAUGGUGUGUUAAUAGCA..CA..UCCU..........GUGGCC.GGGAU.UUGCGGUUAGUG.AAUCACCUAUCCGAAAUUAAGUGAGUAAGAAAAGAUUAAAACGUUUUUUAGUAGUAAAUUUAAAUUUUAAAAU
env-752  CGGGGAU....ACACUUAGA.........GUCCCC...UGGGUAUGGUGUGUUAAUAGCA..CA..UCCU..........GUGGCC.GGGAU.UUGCGGUUAGUG.AAUCACCUAUCCGAAAUUAAGUGAGUAAGAAAAGAUUAAAACGUUUUUUAGUAGUAAAUUUA..........
env-753  CGGGGAU....ACACUUAGA.........GUCCCC...UGGGUAUGGUGUGUUAAUAGCA..CA..UCCU..........GUGGCC.GGGAU.UUGCGGUUAGUG.AAUCACCUAUCCGAAAUUAAGUGAGUAAAAAGAUUAAAACGUUUUUUAGUAGUAAAUUUAAAUUUUAAAAUGGA
env-754  CGGGGAU....ACACUUAGA.........GUCCCC...UGGGUAUGGUGUGUUAAUAGCA..CA..UCCU..........GUGGCC.GGGAU.UUGCGGUUAGUG.AAUCACCUAUCCGAAAUUAAGUGAGUAAGAAAAGAUUAAAACGUUUUUUAGUAGUAAAUUUAAAUUUUAAAAU
env-755  CGGGGAU....ACACUUAGA.........GUCCCC...UGGGUAUGGUGUGUUAAUAGCA..CA..UCCU..........GUGGCC.GGGAU.UUGCGGUUAGUG.AAUCACCUAUCCGAAAUUAAGUGAGUAAGAAAAGAUUAAAACGUUUUUUAGUAGUAAAUUUANNNNNNNNUU
env-756  CGGGGAU....ACACUUAGA.........GUCCCC...UGGGUAUGGUGUGUUAAUAGCA..CA..UCCU..........GUGGCC.GGGAU.UUGCGGUUAGUG.AAUCACCUAUCCGAAAAUUAAGUGAGUAAGAAAAGAUUAAAACGUUUUUUAGUAGUAAAUUUAAAUUUUAAAA
env-757  CGGGGAUAC...ACUUA..........GAGUCCCC...UGGAUAGCGUGUGUUAAUAGCAA.CG..UCCU..........GUGGCC.GGGAU.UUGCGGUUAGUG.AAUCACCUAUCCAAAUAGUGAGUAAAAAGAUUAAAAACGUUUUUUAGUAGUAAUUUAAAUUUUAAAAUGGACA
env-758  CGGGGAUAC...ACUUA..........GAGUCCCC...UGGGUAUGGUGUGUUAAUAGCA..CG..UCCU..........GUGGCC.GGGAU.UUGCGGUUAGAG.AAUCACCUAUCCGAAACAUAAGUGAGUAAGAAAAAGAUUAAAAACGUUUUUUAGUAGUAAAUUUAAAUUUUAA
env-759  CGGGGAUAC...ACUUA..........GAGUCCCC...UGGAUAUGGUGUGUUAAUAGCA..CU..UCCU..........GUGGCC.GGGAU.UUGCGGUUAGUG.AAUCACCUAUCCGAAACAUAAGUGAAUUAAGAAAAAGAUUAAAAACGUUUUUUAGUAGUAAAUUUAAAUUUUA
env-760  CGGGGAUAC...GCUUA..........GAGUCCCC...UGGGUAUGGUGUGUUAAUAGCA..CA..UCCU..........GUGGCC.GGGAU.UUGCGGUUAGUG.AAUCACCUAUCCGAAAUUAAGUGAGUAAGAAAAGAUUAAAACGUUUUUUAGUAGUAAAUUUAAAUUUUAAAAU
env-761  CGGGGAUAC...ACUUA..........GAGUCCCC....UGGACUGGUGUGUUAAUAGCAA.UC..UCCU..........GUGGCC.GGGAA.UUGCGGUUAGUG.AAUCGCCUGUCUAAAAGUGAGUAAAAAGAUUUAAAAACGUUUUUUAGUAGU.......................
env-762  CGGGGAUAC...ACUUA..........GAGUCCCC....UGGAUUGGUGUGUUAAUAGCAA.UG..UCCU..........GUGGCC.GGGAU.UUGCGGUUAGAG.AAUCACUUGUCCGAAAUAGUGAGUAAAAAGAUUAAAAACGUUUUUUAGUAGUAAUUUAAAUUUUAAAAUGGAC
env-763  CGGGGAUAC...ACUUA..........GAGUCCCC....UGGACUGGUGUGUUAAUAGCAA.UG..UCCU..........GUGGCC.GGGAU.UUGCGGUUAGAG.AAUCACCUGUCCGAAAUAGUGAGUAAAAAGAUUAAAAACGUUUUUUAGUAGUAAUUUAAAUUUUAAAAUGGAC
env-765  CGGGGAUAC...ACUUA..........GAGUCCCC....UGGACUGGUGUGUUAAUAGCAA.UG..UCCU..........GUGGCC.GGGAU.UUGCGGUUAGAG.AAUCACCUGUCCGAAAUAGUGAGUAAAAAGAUUAAAAACGUUUUUUAGU........................
env-766  CGGGGAUAC...ACUUA..........GAGUCCCC....UGGACUGGUGUGUUAAUAGCAA.UG..UCCU..........GUGGCC.GGGAU.UUGCGGUUAGAG.AAUCACCUGUCCGAAAUAGUGAGUAAAAAGAUUAAAAACGUUUUUUAGUAGUAAUUUAAAUUUUAAAAUGGAC
env-767  CGGGGAUAC...ACUUA..........GAGUCCCC....UGGACUGGUGUGUUAAUAGCAA.UG..UCCU..........GUGGCC.GGGAU.UUGCGGUUAGAG.AAUCACCUGUCCGAAAUAGUGAGUAAAAAGAUUAAAAACGUUUUUUAGUAGUAAUUUAAAUUUUAAAAUGGAC
env-768  CGGGGAUAC...GCUUA..........GAGUCCCC...UGGGCAGUGUGUGUUAAUAGCAA.UC..UCCU..........GUGGCC.GGGAA.UUGCGGUUAGUG.AAUCGCCUGUCUAAAAGUGAGUAAAAAGAUUUAAAAACGUUUUUUAGUAGUAAUUUAAAAUUUAAAAUGGACA
env-769  CGGGGAUAC...GCUUA..........GAGUCCCC...UGGGCAGUGUGUGUUAAUAGCAA.UC..UCCU..........GUGGCC.GGGAA.UUGCGGUUAGUG.AAUCGCCUGUCUAAAAGUGAGUAAAAAGAUUUAAAAACGUUUUUUAGUAGUAA....................
env-770  CGGGGAUAC...GCUUA..........GAGUCCCC...UGGGCAGUGUGUGUUAAUAGCAA.UC..UCCU..........GUGGCC.GGGAA.UUGCGGUUAGUG.AAUCGCCUGUCUAAAAGUGAGUAAAAAGAUUUAAAAACGUUUUUUAGUAGUAAUUUAAAAUUUAAAAUGGACA
env-771  CGGGGAUAC...GCUUA..........GAGUCCCC...UGGGCAGUGUGUGUUAAUAGCAA.UC..UCCU..........GUGGCC.GGGAA.UUGCGGUUAGUG.AAUCGCCUGUCUAAAAGUGAGUAAAAAGAUUUAAAAACGUUUUUUAGUAGUAAUUUAAAAUUUAAAAUGGACA

         .<<<<<<<<<<<...........>>>>>>>>>>>................<<<<<.<<<..<<..<<<<<..................>>>>>>.>>>>.>>>>>...............................................................
         .222222.1111...........1111.222222................02200.122..22..2222...................22222.2221.00220...............................................................
         •GGGG•∘-----R•••∘∘∘∘----------∘•CCCC----∘∘••••••YGUGU•AAUAGY•--•Y--RYYU---------UUGGCC-GRRY•-••RYGGUU•GUG-AAUCR•••••••RR••R•••••••••••••••••••••••••••••••••••••••••••
          5′                                                                                                           3′
```

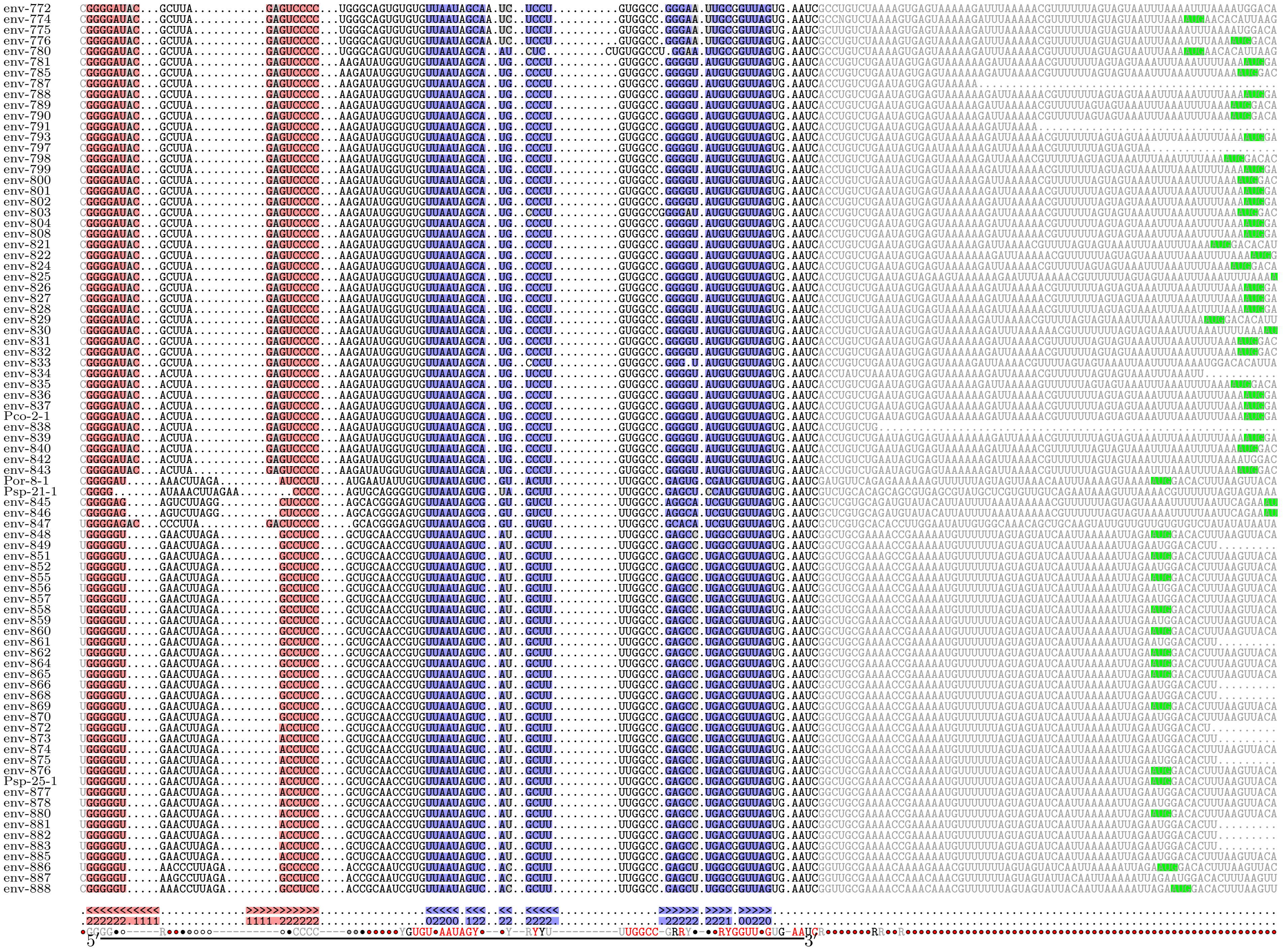

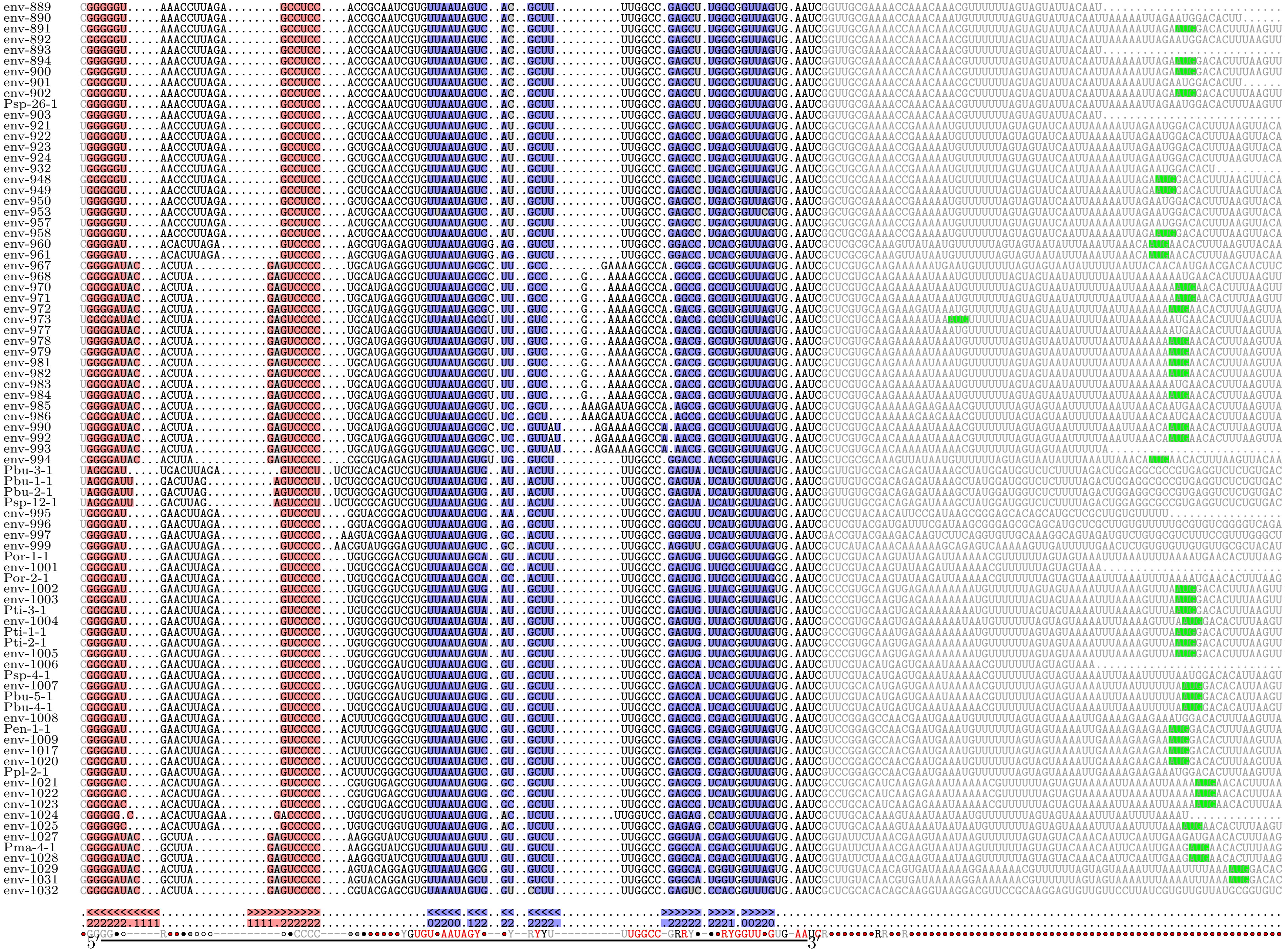

```
env-1033 CGGGGAUAC...ACUUA..........GAGUCCCC....CGUACGAGCGUGUUAAUAGUG..GU..CCUU.........UUGGCC.GAGUC.CCACGGUUUGUG.AAUCGCUCGCACACAGCAAGGUAAGGACGUUCCGCAAGGAGUGCAGUUCCUUAUCGUGUUGUUAUGCGGUGUC
env-1034 CGGGGAUAC...ACUUA..........GAGUCCCC....AGUAUACGAGUGUUAAUAGUG..UU..GCCC.........UUGGCC.GAGCU.GCACGGUUAGUG.AAUCGCGUGUACACAAAGAGAAAUAACAAUUGUUUUUUAGUAGUAAAAUUUAAAUUUAAAUUUAACACUUUA
env-1035 CGGGGAUAC...ACUUA..........GAGUCCCC....AGUAUAAGCGUGUUAAUAGUG..GU..GCUU.........UUGGCC.GAGCA.UCACGGUUAGUG.AAUCGCUUGUGCAUCAAAGUAUUAAUGUUUUUUAGUAGUAAAAUUUUAAAUUAGUAUUUGACACUUUAAGUUA
env-1037 CGGGGAUAC...ACUUA..........GAGUCCCC....UGUGCGGUCGUGUUAAUAGUG..GU..GCAU.........UUGGCC.GUGCC.CCAUGGUUAGUG.AAUCGGUCGUGCAAAGCAAGCCUUCGCGGUGAAUGAAUACAGAGCGAGGCCCAAGAAAGAAAAUGUUUUUAG
env-1038 AGGGG.......AUAACUCUUAGAA.......CCCC.....GGAACGGAGUGUUAAUAGUC..GU..ACUU.........UUGGCC.GAGUG.CGUCGGUUAGUA.AAUCGCCGUUCUAGCAAUAAUGAAUUUAAAAACAUUUUUUAGUAGUAAAUUUUAAAAUUAAAAUUUAACACUUU
env-1039 AGGGG.......AUAACUCUUAGAA.......CCCC.....GGAACGGAGUGUUAAUAGUC..GU..ACUU.........UUGGCC.GAGUG.CGACGGUUAGUG.AAUCGCCGUUCAAGCAAUAAUGAAUUUAAAAACGUUUUUUAGUAGUAAUUUUAAAAUUAAAAUUUAACACUUU
env-1040 AGGGG.......AUAACUCUUAGAA.......CCCC.....GGAACGGAGUGUUAAUAGUC..GU..ACUU.........UUGGCC.GAGUG.CGACGGUUAGUG.AAUCGCCGUUCAAGCAAUAAUGAAUUUAAAAACGUUUUUUAGUAGUAAUUUUAAAAUUAAAAUUUAACACUUU
env-1043 AGGGG.......AUAACUCUUAGAA.......CCCC.....GGAACGGAGUGUUAAUAGUC..GU..ACUU.........UUGGCC.GAGUG.CGACGGUUAGUG.AAUCGCCGUUCAAGCAAUAAUGAAUUUAAAAACGUUUUUUAGUAGUAAUUUUAAAAUUAAAAUUUAACACUUU
env-1044 AGGGG.......AUAACUCUUAGAA.......CCCC.....GGAACGGAGUGUUAAUAGUC..GU..ACUU.........UUGGCC.GAGUG.CGACGGUUAGUG.AAUCGCCGUUCAAGCAAUAAUGAAUUUAAAAACGUUUUUUAGUAGUAAUUUUAAAAUUAAAAUUUAACACUUU
env-1046 AGGGG.......AUAACUCUUAGAA.......CCCC.....GGAACGGAGUGUUAAUAGUC..GU..ACUU.........UUGGCC.GAGUG.CGACGGUUAGUG.AAUCGCCGUUCAAGCAAUAAUGAAUUUAAAAACGUUUUUUAGUAGUAAUUUUAAAAUUAAAAUUUAACACUUU
env-1052 AGGGG.......AUAACUCUUAGAA.......CCCC.....GGAACGGAGUGUUAAUAGUC..GU..ACUU.........UUGGCC.GAGUG.CGACGGUUAGUG.AAUCGCCGUUCAACAAGAGAAAUUAAAAACGUUUUUUAGUAGUAAUUUUAAAAUUAAAAUUUAACACUUUAAG
env-1053 AGGGG.......AUAACUCUUAGAA.......CCCC.....GGAACGGAGUGUUAAUAGUC..GU..ACUU.........UUGGCC.GAGUG.CGACGGUUAGUG.AAUCGCCGUUCAAGCAAUAAUGAAUUUAAAAACGUUUUUUAGUAGUAAUUUUAAAAUUAAAAUUUAACACUUU
Psp-2-1  AGGGG.......AUAACUCUUAGAA.......CCCC.....GGAACGGAGUGUUAAUAGUC..GU..ACUU.........UUGGCC.GAGUG.CGACGGUUAGUG.AAUCGCCGUUCAAGCAAUAAUGAAUUUAAAAACGUUUUUUAGUAGUAAUUUUAAAAUUAAAAUUUAACACUUU
env-1054 AGGGG.......AUAACUCUUAGAA.......CCCC.....GGAACGGAGUGUUAAUAGUC..GU..ACUU.........UUGGCC.GAGUG.CGACGGUUAGUG.AAUCGCCGUUCAAGCAAUAAUGAAUUUAAAAACGUUUUUUAGUAGUAAUUUUAAAAUUAAAAUUUAACACUUU
env-1055 AGGGG.......AUAACUCUUAGAA.......CCCC.....GGAACGGAGUGUUAAUAGUC..GU..ACUU.........UUGGCC.GAGUG.CGACGGUUAGUG.AAUCGCCGUUCAAGCAAUAAUGAAUUUAAAAACGUUUUUUAGUAGUAAUUUUAAAAUUAAAAUUUAACACUUU
env-1056 AGGGG.......AUAACUCUUAGAA.......CCCC.....GGAACGGAGUGUUAAUAGUC..GU..ACUU.........UUGGCC.GAGUG.CGACGGUUAGUG.AAUCGCCGUUCAAGCAAUAAUGAAUUUAAAAACGUUUUUUAGUAGUAAUUUUAAAAUUAAAAUUUAACACUUU
env-1058 CGGGG.......AUACACUUAGAA........CCCC....AGGAAAGGCGUGUUAAUAGCG..GU..GCUU.........UUGGCC.AGGCA.CAGCGGUUAGUG.AAUCGUCAUUCCACGAGAAACGUAAAAAUUGUUUUUUAGUAGUAAAUUUUAAAAUUUGUUUAACACUUUAAG
env-1059 CGGGG.......AUACACUUAGAA........CCCC....AGGAGCGGCGUGUUAAUAGUC..GU..GCUU........UUUGGCC.AGGCG.CGAUGGUUAGUG.AAUCGUCAUUCCACCUUAUUUAAAUUAAAUUGUUUUUUAGUAGUAAAAUUUUAAUUUCGAAUUUAACACUUUA
env-1060 CGGGG.......AUACACUUAGAA........CCCC....AGGAGCGGCGUGUUAAUAGUC..GU..GCUU.........UUGGCC.AGGCG.CGAUGGUUAGUG.AAUCGUCAUUCCACCUUAUUUAAAUUAAAUUGUUUUUUAGUAGUAAAAUUUUAAUUUCGAAUUUAACACUUUA
env-1061 CGGGG.......AAAGACUUAGAA........CCCC....ACGAACGUGGUGUUAAUAGUU..GU..ACUU.........UUGGCC.GAGUG.CGACGGUUAGUG.AAUCGACGUUCAAACGGUUUUCAUAAACCUCUAACCUUAUAUUAUAAAUAGGACAGACGGAAUAUGAAAAGCG
Psp-13-1 CGGGG.......AAAGACUUAGAA........CCCC....ACGAACGUAGUGUUAAUAGUU..GU..ACUU.........UUGGCC.GAGUG.CGACGGUUAGUG.AAUCGACGUUCAAACGGUUUUCAUAAACCUCUAACCUUAUAUUAUAAAUAGGACAGACGGAAUAUGAAAAGCG
env-1062 CGGGG.......AAAGACUUAGAA........CCCC....ACGAACGUAGUGUUAAUAGUU..GU..ACUU.........UUGGCC.GAGUG.CGACGGUUAGUG.AAUCGACGUUCAAACGGUUUUCAUAAACCUCUAACCUUAUAUUAUAAAUAGGACAGACGGAAUAUGAAAAGCG
Psp-17-1 CGGGG.......AAAGACUUAGAA........CCCC....ACGAACGUAGUGUUAAUAGUU..GU..ACUU.........UUGGCC.GAGUG.CGACGGUUAGUG.AAUCGACGUUCAAACGGUUUUCAUAAACCUCUACACCUUAUAUUAUAAAUAGGACAGACGGAAUAUGAAAAGC
env-1063 CGGGG.......AAAGACUUAGAA........CCCC....ACGAACGUAGUGUUAAUAGUU..GU..ACUU.........UUGGCC.GAGUG.CGACGGUUAGUG.AAUCGACGUUCAAACGGUUUUCAUAAACCUCUACACCUUAUAUUAUAAAUAGGACAGGCGGAAUAUGAAAAGC
Psp-15-1 CGGGG.......AAAGACUUAGAA........CCCC....ACGAACGUAGUGUUAAUAGUU..GU..ACUU.........UUGGCC.GAGUG.CGACGGUUAGUG.AAUCGACGUUCAAACGGUUUUCAUAAACCUCUAACCUUAUAUUAUAAAUAGGACAGACGGAAUAUGAAAAGCG
Psp-16-1 CGGGG.......AAAGACUUAGAA........CCCC....ACGAACGUAGUGUUAAUAGUU..GU..ACUU.........UUGGCC.GAGUG.CGACGGUUAGUG.AAUCGACGUUCAAACGGUUUUCAUAAACCUCUACACCUUAUAUUAUAAAUAGGACAGGCGGAAUAUGAAAAGC
env-1064 UGGGG.......AAACACUUAGAA........CCCC....GUGAGCGUUGUGUUAAUAGUC..GU..GCUU.........UUGGCC.GAGUG.CGACGGUUAGUG.AAUCAGCGUUCAAACGACUUUCCCGGCAUGCGUGAUUUUACCGCAUAUAUAAUAUAUAAGGUAAAGGCAAGAGU
env-1065 UGGGG.......AAACACUUAGAA........CCCC....GUGAGCGUUGUGUUAAUAGUC..GU..GCUU.........UUGGCC.GAGUG.CGACGGUUAGUG.AAUCAGCGUUCAAACGACUUUCCCGGCAUGCGCAUUUUACCGCAUAUAUUAUAUAUAAGGUAAAGGCAAGAG.
env-1066 UGGGG.......AAACACUUAGAA........CCCC....GUGAGCGUUGUGUUAAUAGUC..GU..GCUU.........UUGGCC.GAGUG.CGACGGUUAGUG.AAUCAGCGUUCAAACGACUUUCCCGGCAUGCGUGAUUUUACCGCAUAUAUAAUAUAUAAGGUAAAGGCAAGAGU
env-1067 CGGGGAU.....GCACUUAG..........AACCCC....AGGAGCGGCGUGUUAAUAGUG..GU..GCUU.........UUGGCC.AGGCA.CGACGGUUAGUG.AAUCGCCAUUCCAAGUGAAGUAAAAAUUGUUUUUUAGUAGUAAAUUCAAAAUUCGAAUUUAACACUUUAAGUU
Hse-1-1  CGGGGC......ACACUUAGA..........GCCCC....AUACACGACCGUGUUAAUAGUC..GC..GCCU.........UUGGCC.GGACG.CGACGGUUAGUG.AAUCGGCCGUGGGGCAGGGCAAAAGCCAUGGAUGGUCUCUUUUAGACUGGAGGCGCCGUGAGGUCUCUGCUUG
env-1068 CGGGGAU.....AAUCCUUAGA........GUCCCC....AUAGGGCGGUGUGUUAAUAGCC..AU..GUCU.........UUGGCC.AGGCC.UGGCGGUUAGUG.AAUCGCCGUCCAACCCUGUGGCAACGCCGCAGGCGAUAAUGAACAAAUGUUUUUUAGUAGUAAAAUUAAAAGU
env-1069 CGGGGA......GAGACUUAGAA........UCCCC...UUCGGGCAUUGUGUAAAUAGCG..CC..GCCU.........UUGGCC.AGACG.UCGCGGUUUGAG.AAUCAUUGCUCGGCAACCAACCCAGGAUGAAAGUGGGCGACCACUGACAGCCUUGUGGUUAUUUUGUGUGUUU
env-1071 UGGGGA......AAGGCUUAGAA........UCCCC..GCGAAAUAGCUGUGUGAAUAGCG..GC..GUCU.........UUGGCC.GGACG.AAGCGGUUCGUG.AAUCAGCAUUUCAGCAUACGUUAGACUUGCAGCCGGGCGUUAGACCUCGGAUGUGGUUUGUAUGUGUGUAAA
env-1072 CGGGGA......GAGUCUUAGAA........UCCCC....GCCGGUCCCGUGUAAAUAGUC..GU..GCCU.........UUGGCC.GGACA.UGACGGUUUGUG.AAUCGGGGCAAGAGCAUGACUUGGCGCCCAAUUAGACACUGCCAAGGCCAAGAUUUCUGAGGAAUCGGAUUGC
env-1074 CGGGGA......GAGUCUUAGAA........UCCCC....GCCGGUCCCGUGUAAAUAGUC..GU..GCCU.........UUGGCC.GGACG.UGACGGUUUGUG.AAUCGGGGCAAGAGCAUGACUUGGCGCCCAAUUAGACACUGCCAAGGCCAAGAUUCUGAGGAAUCGGAUUGC
env-1075 CGGGGA......GAGUCUUAGAA........UCCCC....GCCGGUCCCGUGUAAAUAGUC..GU..GCCU.........UUGGCC.GGACG.UGACGGUUUGUG.AAUCGGGGCAAGAGCAUGACUUGGCGCCCAAUUAGACACUGCCAAGGCCAAGAUUCUGAGGAAUCGGAUUGC
env-1076 UGGGGA......GAGGCUUAGAA........UCCCC....GCAGACGGCGUGUUAAUAGCA..GU..GUCU.........UUGGCC.GGGCA.UUGUGGUUAGUG.AAUCUCCGUCUGACACAGCAAGACUACCGUGAGGAUUCCGGAGAAGG..........................
env-1077 UGGGGA......GAGGCUUAGAA........UCCCC....GCAGACGGCGUGUUAAUAGCA..GU..GUCU.........UUGGCC.GGGCA.UUGUGGUUAGUG.AAUCUCCGUCUGACACAGCAAGACUACCGUGAGGAUUCCGGAGAAGGGUUCCGAGGGGCAGUUUGUUGUGAGU
env-1078 UUGGGGA.....GAGACUUAGAA.......UCCCCA....CGGGUUGAUGUGUGAAUAGCU..GU..GUCU.........UUGGCC.GGGCG.CGGUGGUUCGAG.AAUCGUCAGUCCAAGGCGCCGUCCCGCCAGGAAUCGUCUGAUUCCGAAGCGUACGUUAGACUUGCAACCGAGA
env-1079 AGGGGA......GAGGCUUAGAA........UCCCC...GUGAAUCCUGGUGUGAAUAGUG..GU..GUCU.........UUGGCC.GGGCA.CAACGGUUUGUG.AAUCGUCGGUUCAGCAAUCCCCGUAAGGGAGUUAUCCCUGCGGAACGCUCGUUAGACUUGCAGCCGGGAGUUA
env-1080 AGGGGA......GAGUCUUAGAA........UCCCC...GCGAGAUAUUGUGUGAAUAGCG..GC..GUCU.........UUGGCC.GGGCG.AAGCGGUUCGUG.AAUCAGUGUCUCUGGUAUCCGCCCGAUGUGGGAUUCGUCCCCACGGGAACGCUCGUUAGACUUGCAGCCGGGA
env-1081 UGGGGA......GAGUCUUAGAA........UCCCC...GCGAGAUAUUGUGUGAAUAGCG..GC..GUCU.........UUGGCC.GGGCG.AAGCGGUUCGUG.AAUCA..........................................................................
env-1083 UGGGGA......GAGUCUUAGAA........UCCCC...GCGAGAUAUUGUGUGAAUAGCG..GC..GUCU.........UUGGCC.GGGCG.AAGCGGUUCGUG.AAUCAGUGUCUCUGGUAUCCGCCCGAUGUGGGAUUCGUCCCCUCGGGAGCGCCCGUUAGACUUACAGCCGGG
env-1084 AGGGGA......GAGACUUAGAA........UCCCC....GUGAGCCGCGUGUGAAUAGCG..GU..GUCU.........UUGGCC.GGGCA.UCGUGGUUCGUG.AAUCGCUGUUCAGCACACUCGUUAGACUGCAGCAGAGCGUUAGACCUCGUAGCGGGUUUGUGUGUGCAAGU
env-1085 UGGGGA......GAGACUUAGAA........UCCCC....GUGAAUCUUGUGUAAAUAGUG..GU..GUCU.........UUGGCC.GGGCA.UUACGGUUUGUG.AAUCAGGGUUCAGCAUAGACGUUAGACUACGAGCAGGGAGUUAGACCCUGUGCGUGGUGAUGUUUGUGCAUA
env-1086 UGGGGA......GAGACUUAGAA........UCCCC....GUGAACCGUGUGUAAAUAGCG..GU..GUCU.........UUGGCC.GGGCG.CUGCGGUUUGUG.AAUCACGAUUCAGCCGAAAGGCACAGACGUUAGACUGCUGCGGAGCGUUAGACUCUCUGCCGU..........
env-1087 UGGGGGA.....GAGACUUAGAA.......UCCCCC....GUGGACCUUGUGUAAAUAGUA..GU..GUCU.........UUGGCC.GGGCA.UUGCGGUUUGUG.AAUCAUGGUUCAGC....................................................................
env-1088 UGGGGA......GAGGCUUAGAA........UCCCC....GUGGACCUUGUGUGAAUAGUA..GU..GUCU.........UUGGCC.GGGCA.UUGCGGUUUGUG.AAUCAUGGUUCAGC....................................................................
env-1089 UGGGGA......GAGGCUUAGAA........UCCCC....GUGAGCCGUGUGUAAAUAGUG..GU..GUCU.........UUGGCC.GGGCA.UUGCGGUUUGUG.AAUCAUGGU.........................................................................
env-1090 UGGGGGA.....AAGACUUAGAA.......UCCUCC....GUGAGCUAUGUGUAAAUAGUG..GU..GUCU.........UUGGCC.GGGCA.UUGCGGUUUGUG.AAUCAUGGU.........................................................................
env-1091 UUGAGGA.....GAGACUUAGAA.......UCCUCA....UGGAUUGAGGUGUGAAUAGCG..GC..GUCU.........UUGGCC.GGGCG.CAGUGGUUCGUG.AAUCGUCAGUCCAGCAA.................................................................
env-1092 UGGGGA......GAGGCUUAGAA........UCCCC...GUGAGCUGGAGUGUGAAUAGCG..GU..GUCU.........UUGGCC.AGGCG.UAGCGGUUCGUG.AAUCGCCAGUUCAGCAGACGUUAGACUUGCAGCCGGGCGACUGACCCCGGAUGUGGGUUUUACGUGUGUGUA
env-1093 CGGGGA......GAGACUUAGAA........UCCCC...GUGAAUCUCCGUGUGAAUAGCG..GC..GUCU.........UUGGCC.GGGCA.UUGUGGUUCGUG.AAUCGUUGGUUCAGUGUACGUUAGACUACUUGCGGAGCGUUAGACCUUCGCUGGUGGUUAUUUGUGCGCAUAA
env-1094 UGGGGA......GAGACUUAGAA........UCCCC.GUUGAAUGAGCGUGUGAAUAGCG..GC..GUCU.........UUGGCC.GGACG.UGGUGGUUCGUG.AAUCGCUGAUUCGAAAGUCCUGAAAGGCUCAAAAGCCCGUAGGGAGCGUGCGUUAGACUAACAGCCGGGAGUU
env-1095 GGGGGA......GAGACUUAGAA........UCCCC...GUGAAUCAGCGUGUGAAUAGCG..GC..GUCU.........UUGGCC.GGGCG.UGGCGGUUCGUG.AAUCGCAGGUUCGAGAAUCCCGAGUGGGCUCCUGAGUGGGGCCCUACGGCGCGCACGUUAGACUACCGCCGG
env-1096 UGGGGA......GAGACUUAGAA........UCCCC...GUGGAUUGAGGUGUGAAUAGCG..GC..GUCU.........UUGGCC.GGGCG.CAGUGGUUCGUG.AAUCGCUGGUCCAGACUGCCCUAAGGGAGUAUUCCCGAUGGGGACGUUCGUUAGACUUGCAACCAGGCGCUGC
env-1097 GGGGGA......GAGACUUAGAA........UCCCC...GUGAGUCAGGGUGUGAAUAGCG..GC..GUCU.........UUGGCC.GGGCG.CGGUGGUUCGUG.AAUCGCUGGUUCAGUGCACGUUAGACUACUUGCAGGGAGUUAGACCUCUGCCAGUGGUAUCUGUGCGCAUAAG
env-1098 UGGGGA......GAGACUUAGAA........UCCCC...GUGAAUCAGGGUGUGAAUAGCA..GC..GUCU.........UUGGCC.GGGCG.UAGUGGUUCGUG.AAUCGCUGGUUCAUCAGUGGAAUCAAUAGGUUCCAUGCGUACGUUAGACUUGCAGCCGAGUGUUAGACCUCGG
Psp-7-1  UGGGGA......GAGACUUAGAA........UCCCC...GUGAAUCAGGGUGUGAAUAGCG..GC..GUCU.........UUGGCC.GGGCG.CAGUGGUUCGUG.AAUCGCUGGUUCAUCAGCGGAAUCGUAUGAUUCCGGCGUUCGUUAGACUUGCAACCGAGUGUUAGACCUCGG
env-1099 GGGGGA......GAGACUUAGAA........UCCCC...GUGAAAUGUUGUGUGAAUAGCG..GC..GUCU.........UUGGCC.GGGCA.UGGUGGUUCGUG.AAUCAGCGUUUCUUGCGACGUUAGACUGACAACCGGGAGUUAGACCCUGGGAGUGAGUUUAUGCGCGUACAUA
env-1100 AGGGGA......GAGACUUAGAA........UCCCC...GUGAAAUGUUGUGUGAAUAGCG..GU..GUCU.........UUGGCC.GGGCA.UGGUGGUUCGUG.AAUCAGCGUUUCUUGCGACGUUAGACUGACAACCGGGAGUUAGACCCUGGGAGUGAGUUUAUGCGCGUACAUA
Psp-11-1 AGGGGA......GAAACUUAGAA........UCCCC...GUGAAAUGUUGUGUGAAUAGCG..GU..GUCU.........UUGGCC.GGGCA.UGGCGGUUCGUG.AAUCAGCGUUUCAAUGAUCUCCGUUAGGGAAUUAUCCCAGCGGGUGCGACGUUAGACUGACAACCGGGAGUUA
Psp-19-1 AGGGGA......GAGGCUUAGAA........UCCCC...GUGAAAUGUUGUGUGAAUAGCG..GU..GUCU.........UUGGCC.GGGCA.UGGCGGUUCGUG.AAUCAGCGUUUCAAUGAUCCCCGUCAGGGAAUUAACCCGAGGGGGUGCGACGUUAGACUGACAACCGGGAGUU
env-1101 AGGGGA......GAGACUUAGAA........UCCCC...GUGAAAUGUUGUGUGAAUAGCG..GU..GUCU.........UUGGCC.GGGCA.UGGCGGUUCGUG.AAUCAGCGUUUCAAUGAUCCCCGUCAGGGAAUCAGCCCUACGGG...........................
Pru-1-1  AGGGGA......GAGACUUAGAA........UCCCC...GUGAAAUGUUGUGUGAAUAGCG..GU..GUCU.........UUGGCC.GGGCA.UGGCGGUUCGUG.AAUCAGCGUUUCUUGCGACGUUAGACUGACAACCGGGAGUUAGACCCUGGGAGUGAGUUUAUGCGCGUACAUA
env-1102 UGGGGA......GAGACUUAGAA........UCCCC...UUGAGUCUUGGUGUGAAUAGCG..GU..GUCU.........UUGGCC.GGGCA.UGGCGGUUCGUG.AAUCCUUGGUUCAAUGAUCCCCGUCAGGGAAUCUCCCCAACGGGAGCGUUCGUUAGACUUGCAGCCG......
env-1103 AGGGGA......GAGUCUUAGAA........UCCCC...ACGAGAUGGCGUGUAAAUAGCGU.UU..GUCU.........UUGGCC.GGACA.GCGUGGUUUGUG.AAUCGUCGCUCAGCCUGCGGUGAGAUACGCAAAAACCUAUUUAAAAAACUGUUUUUUAGUAAAUUAAGAUUUU
env-1104 CGGGGA......GAGUCUUAGAA........UCCCC....AAGGGCGCCGUGUAAAUAGUGA.CU..GUCU.........UUGGCC.GGACG.GCAUGGUUUGUG.AAUCGGCGUUCAACAAGCGAACUUUAUAAAGUAAAAGAACUGUUUUUUAGUAAAUUAAACGAAGAAUGAACA
Pbr-1-1  GGGGGA......GAGUCUUAGAA........UCCCC....ACGGGCGUCGUGUAAAUAGUGA.CU..GUCU.........UUGGCC.GGAUG.GCACGGUUUGGG.AAUCGGCGUUCACACAAUCAACUUUAUAAAGUAAAAAACUGUUUUUUAGUAAAUUUAUUUAAGAUUUAACACU
Pbr-2-1  CGGGGA......GAGUCUUAGAA........UCCCC....ACGGGCGUCGUGUAAAUAGUGA.CU..GUCU.........UUGGCC.GGAUG.GCACGGUUUGUG.AAUCGGCGUUCACACAAUCAACUUUAUAAAGUAAAAAACUGUUUUUUAGUAAAUUUAUUUAAGAUUUAACACU
env-1105 CGGGGA......GAGUCUUAGAA........UCCCC....ACGGGCGUCGUGUAAAUAGUGA.UU..GUCU.........UUGGCC.GGACA.GCACGGUUUGUG.AAUCGGCGUUCAAAGCAUUAAAACUUUAUAAAGUAGAACAUCUGUUUUUUAGUAUUUAAAAUU..........
env-1106 CGGGGA......GAGUCUUAGAA........UCCCC...ACGGGACGUCGUGUAAAUAGUGA.CU..GCCU.........UUGGCC.AGACA.GCACGGUUUGUG.AAUCGGCG....................................................................
env-1107 AGGGGA......GAGUCUUAGAA........UCCCC....GUGGGCGUCGUGUAAAUAGCGC.CU..GUCU.........UUGGCC.GGACA.GCGUGGUUUGUG.AAUCGGCGUUCA................................................................
env-1108 UGGGGA......GAGUCUUAGAA........UCCCC....GUGGGCGUCGUGUAAAUAGCGU.CC..GUCU.........UUGGCC.GGACG.GCGUGGUUUGUG.AAUCGGC.....................................................................
env-1109 CGGGGA......GAGACUUAGAA........UCCCC..AUAGGGUCAGUGUGUAAAUAGCU..GU..GUCU.........UUGGCC.GGGCG.UAACGGUUUGUG.AAUCACUGAGUCAGUGACGGUGCAAGUCCGUCGGUGCAUAUCGUUAGACUACCAUGCAGAGAGUUAGACCUCU
env-1110 AGGGGA......GAGUCUUAGAA........UCCCC..ACAGAACGAGUGUGUAAAUAGUG..GC..GUCU.........UUGGCC.GGGCG.CAACGGUUUGUG.AAUCACUGGUUCAAGGACGGUGAGAGUCCGUCGCAGCGCAUUCGUUAGACUACCGUGCGGAGAGUUAGACCUC
env-1111 AGGGGA......GAGUCUUAGAA........UCCCC..ACAGAGCGAGUGUGUAAAUAGUG..GU..GUCU.........UUGGCC.GGGCA.CAACGGUUUGUG.AAUCACUGGUUCCAGGGCGGUGAAAGUCCGCCGUAGCGUAUCGUUAGACUACCGUGCAGGGAGUUAGACCUCU
env-1112 CGGGGAG.....GGUCUUAGA..........CUCCCC.....ACGAGUGUCGUGUAAAUAGUGC.UU..GUCU.........UUGGCC.GGAUG.GCACGGUUUGUG.AAUCGCGCUCAGUAAACAAUAUUUUAGGUAAAACUGUUUUUUAGUAAAAUUAAACGAAGAAUUUAACACUU
env-1113 UGGGGAAUU...ACUUA..........GGUUCCCC..GUGGGUGAUGUGUGUUAAUAGUC..GU..GCGU.........UUGGCC.GCGC..CGACGGUUAGUG.AAUCCGCACCCAGAGAGGCUAAGGGUGAAUUAGACUGCGCCUAAGGCCGAAAAGAAGUUUUUUAGUAGUUAAA
env-1114 AGGGGA......GAGACUUAGAA........UCCCC..ACGGGUGAUGUGUGUUAAUAGUC..GU..GCGU.........CUGGCC.GCGC..CGAUGGUUAGUG.AAUCGCACCCAGAGAGACAUUAUAUAUAAGGUGAAUUAGACUGCGCCUUGUCUCUUGCUUUAAUAAUUAACG
env-1115 AGGGGA......AAGACUUAGAA........UCCCC..AUGGGUGAUGUGUGUUAAUAGUU...A..GCGU.........UUGGCC.GCGCU..GACGGUUAGUG.AAUCGCACCCAGAGAGACAUAUAAUAUAAGGUGAAUUAGACUACGCCUUGUCUCUCGCAUUAAUUUUAAUUG
env-1116 CGGGGUAC....GCUUA..........GAGCCCC..ACCGUGCGGAAGUGUUAAUAGUG..GU..ACUU.........UUGGCC.GAGUA.CAACGGUUAGUG.AAUCGACCGCACAGAGAUAGAAACAUUAAAGAAAAACGUUUUUUAGUAGUAAUUUUAAAAUUAGAUUUAACAC

         .<<<<<<<<<<<............>>>>>>>>>>>...............<<<<<.<<<..<<..<<<<<..............>>>>>>.>>>>.>>>>>.................................................................................
         .222222.1111............1111.222222...............02200.122..22..2222...............22222.2221.00220...................................................................................
         •GGGG•∘-----R•••∘∘∘∘---------∘•CCCC---∘∘•••••YGUGU•AAUAGY•--•Y--RYYU---------UUGGCC-GRRY•-••RYGGUU•GUG-AAUCR•••••••RR••R•••••••••••••••••••••••••••••••••••••••••••••
          5′                                                                                                                         3′
```

```
env-1117  CGGGGAAC....ACUUA............GAUCCCC....AUGGCCGUAGUGUUAAUAGUC..AU..GUCU..........CUGGCC.GGGCA.UGGCGGUUAGUG.AAUCUGCGUCCAGACGGCAGACAGCCGGAGAGCGACGGUUCUUCCGGCGGUUGGUCGUGAGAAAGAAUUGAAA
env-1118  UGGGGCAC....ACUUA............GAACCCC...GCGAGCGAUGGUGUUAAUAGUC..AU..GCGU..........UUGGCC.GCGCA..GACGGUUAGUG.AAUCCGCGUUCGAAACAAAUGUUUUUUAGUAGUAUUUUUAAAAAGAUUUUUAAAGUUAAAUGAACACUUUAAG
env-1119  UGGGGCAC....ACUUA............GAACCCC...GCGAGCGAUGGUGUUAAUAGUC..AU..GCGU..........UUGGCC.GCGCA..GACGGUUAGUG.AAUCCGCGUUCGAAACAAUUGUUUUUUAGUAGUAUUUUUAAAAAGAUUUUUAAAGUUAAAUGAACACUUUAAG
env-1120  CGGGGG......AAGUCUUAG..........CCCCC..CGCCGGGCAAAGUGUUAAUAGCG..CC..GCUUG..........UGGC.CGAGCG.UCGCGGUUAGUG.AAUCUGCGCCCGACCGAGAGUCUCCCGAGACUAAGAGCCUCUCGCAAAGAAUGUUUUUUAGUAGUAUAAAUUU
env-1122  CGGGGG......AAGUCUUACG.........CCCCC..CGCCGGGCAAAGUGUUAAUAGCG..CC..GCUUG..........UGGC.CGAGCG.UCGCGGUUAGUG.AAUCUGCGCCCGACCGAGAGUCUCCCGAGACUAAGAGCCUCUCGCAAAGAAUGUUUUUAGUAGUAUAAAUUUU
env-1123  UGGAGCG.....AGUCUUAGA.........CGCUCC....GGUGCUGACGUGUUAAUAGUG..GC..AUCU..........UUGGCC.GGGUGACCAUGGUUAGUG.AAUCGAAAGCACAGCGACACAUAAGGUGAGCAGCACAGCGUUCGCUCUGUUUGUUGUGCGCCUGCGUGUCGAA
env-1125  UGGGGGAU....AGCUUAG..........GUCCUCC....GCUCUUGGCGUGUUAAUAGCG..UU..GCUU..........UUGGCC.GAGCA.UCGUGGUUAGUG.AAUCGUCAGGAAAGAGACAACAGAAAAGAAAACGUUUUUUAGUAGUAAUUUUUUAACUUAGAAAUGAACACAU
env-1126  UGAAGGAU....GUCUUAG..........GUCCUUC....GGUCUCGUCGUGUUAAUAGCU..GU..ACUU..........UUGGCC.GAGUG.CGGUGGUUAGUG.AAUCGUCGGGGCAAGCAAAGAGUUUUUUAGUAGUAAAGAAAGAAUUAGAGUUUAGAAAUGAACACGUUAAGUU
env-1127  UGAAGGAU....GUCUUAG..........GUCCUUC....GGUCUCGUCGUGUUAAUAGCG..GU..ACUU..........UUGGCC.GAGUG.CAGUGGUUAGUG.AAUCGUCGGGGCAAGCAAAAAGUUUUUUAGUAGUAAAAAGUAUUUUUAGAGUUAGAAAUGAACACGUUAAGUU
env-1129  CGGGGAU.....GUGCUUAGA.........GUCCCC...AGGAUGUGGCGUGUUAAUAGAG..GUA.UUCU..........GUGGCC.GGAAG.CCUUGGUUAGUG.AAUCGCCUGUCCAAAGAAAAGUGAGAUUAUAAAAACGUUUUUUAGUAGUAAAAUUUAAAAGUUAAAAAUGGAC
env-1130  AGGGCAC.....CCCAAAAA..........GUGCCC......AAACUGCGUGUGAAUAGCC..CGU.UUCA..........UUGGCC.UGGAC.GGACGGUUCGUG.AAUCGCAGAGAAUCAAGAAGUUAAACGUAGAAAACGAACAUUAAAAGAAAUGGACACACUGAGUCAGAAGACC
env-1131  GGGGU..ACC..CUCU...........GGUAUACCC.....AAAUCUGCGUGUAAAUAGUC..CGUUUCCA..........UUGGCC.UGGAC.GGGCGGUUUGUG.AAUCGCAGAACAAGUAGUAAUGAACAAUAAAGAAUUAAAAAACUAAAAUGGACACACUUAGUCAGAAGACCAC
env-1132  GGGGUACCC...CUCUGA..........GCGUACCC.......AUCUGCGUGUGAAUAGUC..CGUUUCCA..........UUGGCC.UGGAC.GGGUGGUUCGUG.AAUCGCAGAAGAGAACAAAAAACGUAGUAACGAACAAAAGAAACUAAAAAACAAAAAAUGGACACACUGAGUC
env-1133  AGGACCC.....CUAAA.............GGGCCC....UCUCGAAGCGUGUGAAUAGUC..CGC.AUCA..........UUGGCC.UGAAC.GGACGGUUCGUG.AAUCGCUCGACAAAAUGAGAAAUAAACUAGGUAGAAACAAACAAAAGUAAGACAAAGAAUGGAUACUUUGAGU
env-1134  AGAGGGG.....CUAAAAUA..........CCCCUC......AAACUGCGUGUAAAUAGUU..CGUUUCCA..........UUGGCC.UGGAC.GGAUGGUUUGUG.AAUCGCAGAAAACAAGAAAAAUUAAACGUAGUAACGAACAAAACAAAAGAAAUGGACACACUGAGUCAGAAGA
env-1135  AGGGGC......GAAUUAU............GCCCC......AAAGUGCGUGUGAAUAGUU..CG..CUCU..........UUGGCC.AGA.C.GGACGGUUCGUG.AAUCGCGCUGAAAAUGAACUUAGUAGAAAACAAAAAGAAAAAACAAAAUGGACACAUUGAGUUACAAGACCAC
env-1136  CGGGGC......AAAAACA............GCCCC......AAAGUGCGUGUGAAUAGUG..U...GCUU.........AUUGGCC.AGGU..ACACGGUUCGUG.AAUCGCGCUGAAGAAGAGUUAAAUUUAGUAGAGAACAAAAAGAAAAGAACAAAGAAAUGGACACAUUGAGUUA
env-1137  AAGUGGCCUC..UUUU...........GAGG.CACU.......AUCUGCGUGUUAAUAGCU..AC..GUUC.........UUUGGCC.GAACG.UGGUGGUUAGUG.AAUCGCAGAGAAAAGUGAUAAUUUAUUAGAUUUAGGGAUUAAUUCCCCUUAGUAACUACAAACAAUUAACAAC
env-1138  AAGUGGCCUC..UUUU...........GAGG.CACU.......AUCUGUGUGUUAAUAGCU..GC..GUUC.........UUUGGCC.GAACG.UGGUGGUUAGUG.AAUCGCAGAGAAAAGUGAUAAUUUAUUAGAUUUAGGGAUUAAUUCCCCUUAGUAACUACAAACAAUUAACAAC
env-1139  AAGUGGCCUC..UUUU...........GAGG.CACU.......AUCUGUGUGUUAAUAGCU..GC..GUUC.........UUUGGCC.GAACG.UGGUGGUUAGUG.AAUCGCAGAGAAAAAUGAUAAUUUAUUAGAUUUAGGGAAUCAAUUCCCCUUAGUAACUACAAACAAUUAACAA
env-1140  AAGUGGCCUC..UUUU...........GAGG.CGCU.......AUCUGUGUGUUAAUAGCU..GC..GUUC.........UUUGGCC.GAACG.UGGUGGUUAGUG.AAUCGCAGAGAAAAAUGAUAAUUUAUUAGAUUUAGGGAAUCAAUUCCCCUUAGUAACUACAAACAAUUAACAA
env-1141  AAGUGGCCUC..UUUU...........GAGG.CGCU.......AUCUGUGUGUUAAUAGCU..AC..GUUC.........UUUGGCC.GAACG.UGGUGGUUAGUG.AAUCGCAGAGAAAAAUGAUAAUUUAUUAGAAUUAGGGAUUCAAUUCCCCUUAGUAACUACAAACAAUUAACAA
env-1143  AAGUGGCCUC..UUUU...........GAGG.CGCU.......AUCUGUGUGUUAAUAGCU..AC..GUUC.........UUUGGCC.GAACG.UGGUGGUUAGUG.AAUCGCAGAGAAAAAUGAUAAUUUAUUAGAUUUAGGGAUUCAAUUCCCCUUAGUAACUACAAACAAUUAACAA
env-1144  AAGUGGCCUC..UUUU...........GAGG.CGCU.......AUCUGUGUGUUAAUAGCU..AC..GUUC.........UUUGGCC.GAACG.UGGUGGUUAGUG.AAUCGCAGAGAAAAAUGAUAAUUUAUUAGAUUUAGGGAAUCAAUUCCCCUUAGUAACUACAAACAAUUAACAA
env-1145  AAGUGGCCUC..UUUU...........GAGG.CGCU.......AUCUGUGUGUUAAUAGCU..AC..GUUC.........UUUGGCC.GAACG.UGGUGGUUAGUG.AAUCGCAGAGAAAAAUGAUAAUUUAUUAGAAUUAGGGAUUCAAUUCCCCUUAGUAACUACAAACAAUUAACAA
env-1147  AGUGCCUC....AAAA.............GAGUCAC.....G..GCCGCGUGUUAAUAGCC..GA..GUUU........CAUUGGCCGAAAAU.CGGUGGUUAGUG.AAUCGCGGAAACGAUAACUUAAUUGAAUUAGGAGUACGCUCCUUAGUAACUACAAAAAUCUCAACAAAAAGAA
env-1148  AGUGCCUC....AAAA.............GAGUCAC.....G..GCCGCGUGUUAAUAGCC..GA..GUUU........CAUUGGCCGAAAAU.CGGUGGUUAGUG.AAUCGCGGAAACGAUAACUUAAUUGAAUUAGGAGUACGCUCCUUAGUAACUACAAAAUCUCAACAAAAAGAAU
env-1149  AGUGCCUC....AAAA.............GAGUCAC.....G..GCCGCGUGUUAAUAGCC..GA..GUUU........CAUUGGCCGAAAAU.CGGUGGUUAGUG.AAUCGCGGAAACGAUAACUUAAUUGAAUUAGGAGUACGCUCCUUAGUAACUACAAAAAUCUCAACAAAAAGAA
env-1151  AGUGC.CUC...AUAA............GAGAGCAC.......AGCCGCGUGUUAAUAGCC..GA..GUUUC........AUUGGCCGAAAAU.CGGUGGUUAGUG.AAUCGCGGAAACGAUAACUUAAUUGAAAUAGGAGUACGCUCCUUAGUAACUACAAAAAUCUCAACAAAAAGAA
env-1153  AGUGC.CUC...AAAA............GAGUGCAC.......GGCCGCGUGUUAAUAGCC..GA..GUUUC........AUUGGCCGAAAAU.CGGUGGUUAGUG.AAUCGCGGAAACGAUAACUUAAUUGAAUUAGGAGUACGCUCCUUAGUAACUACAAAAAUCUCAACAAAAAGAA
env-1154  AGUGC.CUC...AAAA............GAGUGCAC.......GGCCGCGUGUUAAUAGCC..GA..GUUUC........AUUGGCCGAAAAU.CGGUGGUUAGUG.AAUCGCGGAAACGAUAACUUAAUUGAAAUAGGAGUACGCUCCUUAGUAACUACAAAAUCUCAACAAAAAGAAU
env-1155  AGUGC.CUC...AAAA............GAGUGCAC.......GGCCGCGUGUUAAUAGCC..GA..GUUUC........AUUGGCCGAAAAU.CGGUGGUUAGUG.AAUCGCGGAAACGNNNNNNNNAAGCUACAAGACCAUUUCUGCCAACAAGGAAACCGCACAGAAAGAGUGGGUC
env-1156  AGUGC.CUC...AAAA............GAGUGCAC.......GGCCGCGUGUUAAUAGCC..GA..GUUUC........AUUGGCCGAAAAU.CGGUGGUUAGUG.AAUCGCGGAAACGAUAACUUAAUUGAAUUAGGAGUACGCUCCUUAGUAACUACAAAAAUCUCAACAAAAAGAA
env-1157  AGUGC.CUC...AAAA............GAGUGCAC.......GGCCGCGUGUUAAUAGCC..GA..GUUUC........AUUGGCCGAAAAU.CGGUGGUUAGUG.AAUCGCGGAAACGAUAACUUAAUUGAAAUAGGAGUACGCUCCUUAGUAACUACAAAAUCUCAACAAAAAGAAU
env-1158  AGUGC.CUC...AAAA............GAGUGCAC.......GGCCGCGUGUUAAUAGCC..GA..GUUUC........AUUGGCCGAAAAU.CGGUGGUUAGUG.AAUCGCGGAAACGAUAACUUAAUUGAAUUAGGAGUACGCUCCUUAGUAACUACAAAAAUCUCAACAAAAAGAA
env-1159  AGUGC.CUC...AAAA............GAGUGCAC.......GGCCGCGUGUUAAUAGCC..GA..GUUUC........AUUGGCCGAAAAU.CGGUGGUUAGUG.AAUCGCGGAAACGAUAACUUAAUUGAAUUAGGAGUACGCUCCUUAGUAACUACAAAAAUCUCAACAAAAAGAA
env-1163  AGUGC.CUC...AAAA............GAGUGCAC.......GGCCGCGUGUUAAUAGCC..GA..GUUUC........AUUGGCCGAAAAU.CGGUGGUUAGUG.AAUCGCGGAAACGAUAACUUAAUUGAAAUAGGAGUACGCUCCUUAGUAACUACAAAAAUCUCAACAAAAAGAA
env-1164  AGUGC.CUC...AAAA............GAGUGCAC.......GGCCGCGUGUUAAUAGCC..GA..GUUUC........AUUGGCCGAAAAU.CGGUGGUUAGUG.AAUCGCGGAAACGAUAACUUAAUUGAAAUAGGAGUACGCUCCUUAGUAACUACAAAAAUCUCAACAAAAAGAA
env-1165  AGUGC.CUC...AAAA............GAGUGCAC.......GGCCGCGUGUUAAUAGCC..GA..GUUUC........AUUGGCCGAAAAU.CGGUGGUUAGUG.AAUCGCGGAAACGAUAACUUAAUUGAAAUAGGAGUACGCUCCUUAGUAACUACAAAAAUCUCAACAAAAAGAA
env-1166  AGUGC.CUC...AAAA............GAGUGCAC.......GGCCGCGUGUUAAUAGCC..GA..GUUUC........AUUGGCCGAAAAU.CGGUGGUUAGUG.AAUCGCGGAAACGAUAACUUAAUUGAAUCUAGGAGUACGCUCCUUAGUAACUACAAAAAUCUCAACAAAAAGA
env-1167  AGUGC.CUC...AAAA............GAGUGCAC.......GGCCGCGUGUUAAUAGCC..GA..GUUUC........AUUGGCCGAAAAU.CGGUGGUUAGUG.AAUCGCGGAAACGAUAACUUAAUUGAAAUAGGAGUACGCUCCUUAGUAACUACAAAAAUCUCAACAAAAAGAA
env-1192  UGUGGCU.C...AUUU............GCAGCCAC.......ACUCGCGUAUUAAUAGCC..GG..GUUU........CAUUGGCC.GAAAAACGGUGGUUAGUG.AAUCGCGAGAACGAUAACUUAAUUGAAAUAGGAGCUCGCUCCUUAGUAANNNNNNNNNNNNNNAAGCUACAAGA
env-1193  UGCCCCA.....AUUG..............UGGGGU....UACACUCACGUGUUAAUAGCC..UA..GUUC.........UUUGGCC.GAAUU.UGGUGGUUAGUG.AAUCGUGAGAAAAAGAUAAUUCAUUGAUUUAUGGGCAUUUGCCCACUUAGUAACUACAAAUUAAUCACUUAGA
env-1194  GCCCCAA.....UUU...............UUGGGG..GUUACACUCACGUGUUAAUAGCC..UA..GUUC.........UUUGGCC.GAACU.UGGUGGUUAGUG.AAUCGUGAGAAAAAGAUAAUUCAUUGAUUUAUGGGCAUUUGCCCACUUAGUAACUACAAAUUAAUCACUUAGA
Psp-1-1   GCCCCA......AUUUUG.............UGGGG..GUUACACUCACGUGUUAAUAGCC..UA..GUUC.........UUUGGCC.GAACU.UGGUGGUUAGUG.AAUCGUGAGAAAAAGAUAAUUCAUUGAUUUAUGGGCAUUUGCCCACUUAGUAACUACAAAUUAAUCACUUAGA
env-1195  GCCCCA......AUUG...............UGGGG..GUUACACUCACGUGUUAAUAGCC..UA..GUUC.........UUUGGCC.GAACU.UGGUGGUUAGUG.AAUCGUGAGAAAAAGAUAAUUCAU.................................................
env-1197  GCCCCA......AUUG...............UGGGG..GUUACACUCACGUGUUAAUAGCC..UA..GUUC.........UUUGGCC.GAACU.UGGUGGUUAGUG.AAUCGUGAGAAAAAGAUAAUUCAUUGAUUUAUGGGCAUUUGCCCACUUAGUAACUACAAAUUAAUCACUUAGA
env-1203  GCCCCA......AUUG...............UGGGG..GUUACACUCACGUGUUAAUAGCC..UA..GUUC.........UUUGGCC.GAACU.UGGUGGUUAGUG.AAUCGUGAGAAAAAGAUAAUUCAUUGAUUUAUGGGCAUUUGCCCACUUAGUAACUACAAAUUAAUCACUUAGA
env-1205  GCCCCA......AUUG...............UGGGG..GUUACACUCACGUGUUAAUAGCC..UA..GUUC.........UUUGGCC.GAACU.UGGUGGUUAGUG.AAUCGUGAGAAAAAGAUAAUUCAUUGAUUUAUGGGCAUUUGCCCACUUAGUAACUACAAAUUAAUCACUUAGA
env-1206  UUGCCCC.....AAUUGUGG..........GGGGUA......CACUCACGUGUUAAUAGCC..UA..GUUC.........UUUGGCC.GAACU.UGGUGGUUAGUG.AAUCGUGAGAAAAAGAUAAUUCAUUGAUUUAUGGGCAUUUGCCCACUUAGUAACUACAAAUUAAUCACUUAGA
env-1211  UUGCCCC.....AAUUGUGG..........GGGGUA......CACUCACGUGUUAAUAGCC..UA..GUUC.........UUUGGCC.GAACU.UGGUGGUUAGUG.AAUCGUGAGAAAAAGAUAAUUCAUUG...............................................
env-1213  AGUUGCCCCA..AUUG...........UGGGGUUAC.......ACUCACGUGUUAAUAGCC..UU..GUUC.........UUUGGCC.GAACU.UGGUGGUUAGUG.AAUCGUGAGAAAAAGAUAAUUCAUUGAUUUAUGGGCAUUCGCCCACUUAGUAACUACAAAUUAAUCACUUAGA
env-1216  AGUUACCUC...GUUUU...........GAGGUUGC.......ACUUGCGUGUUAAUAGCC..AA..GUUC.........UUUGGCC.GAAUU.UGGUGGUUAGAG.AAUCGCAAGAAAAAGAUAAUUUAUUGAUUUAUGGGCGAAAGCCCACUUAGUAACUACAAAUUAAUCAUUAGAA
env-1223  AGUUACCUC...GUUUU...........GAGGUUGC.......ACUUGCGUGUUAAUAGCC..AA..GUUC.........UUUGGCC.GAAUU.UGGUGGUUAGAG.AAUCGCAAGAAAAAGAUAAUUUAUUGAUUUAUGGGCGAAAGCCCACUUAGUAACUACAAAUUAAUCAUUAGAA
env-1224  AGUUACCUC...GUUUU...........GAGGUUGC.......ACUUGCGUGUUAAUAGCC..AA..GUUC.........UUUGGCC.GAAUU.UGGUGGUUAGAG.AAUCGCAAGAAAAAGAUAAUUUAUUGAUUUAUGGGCGAAAGCCCACUUAGUAACUACAAAUUAAUCAUUAGAA
env-1228  AGUUACCUCA..UUU............UGAGGUUGC.......ACUUGCGUGUUAAUAGCC..AA..GUUC.........UUUGGCC.GAAUU.UGGUGGUUAGAG.AAUCGCAAGAAAAAGAUAAUUUAUUGAUUUAUGG.......................................
env-1233  AGUUACCUCA..UUU............UGAGGUUGC.......ACUUGCGUGUUAAUAGCC..AA..GUUC.........UUUGGCC.GAAUU.UGGUGGUUAGAG.AAUCGCAAGAAAAAGAUAAUUUAUUGAUUUAUGGGCGAAAGCCCACUUAGUAACUACAAAUUAAUCAUUAGAA
env-1243  AGUUACCUCA..UUU............UGAGGUUGC.......ACUUGCGUGUUAAUAGCC..AA..GUUC.........UUUGGCC.GAAUU.UGGUGGUUAGAG.AAUCGCAAGAAAAAGAUAAUUUAUUGAUUUAUGGGCGAAAGCCCACUUAGUAACUACAAAUUAAUCAUUAGAA
env-1244  AGUUGCCUCA..UUU............UGAGGUUGC.......ACUUGCGUGUUAAUAGCC..AA..GUUC.........UUUGGCC.GAAUU.UGGUGGUUAGAG.AAUCGCAAGAAAAAGAUAAUUUAUUGAUUUA..........................................
env-1245  AGUUGCCUCA..UUU............UGAGGUUGC.......ACUUGCGUGUUAAUAGCC..AA..GUUC.........UUUGGCC.GAAUU.UGGUGGUUAGAG.AAUCGCAAGAAAAAGAUAAUUUAUUGAUUUAUGGGCGAAAGCCCACUUAGUAACUACAAAUUAAUCAUUUAGA
env-1250  AGUUGCCUCA..UUU............UGAGGUUGC.......ACUUGCGUGUUAAUAGCC..AA..GUUC.........UUUGGCC.GAAUU.UGGUGGUUAGAG.AAUCGCAAGAAAAAGAUAAUUUAUUGAUUUAUGGGCGAAUGCCCACUUAGUAACUACAAAUUAAUCAUUUAGA
env-1251  AGUUGCCUCA..UUU............UGAGGUUGC.......ACUUGCGUGUUAAUAGCC..AA..GUUC.........UUUGGCC.GAAUU.UGGUGGUUAGAG.AAUCGCAAGAAAAAGAUAAUUUAUUGAUUUAUGGGCGAAAGCCCACUUAGUAACUACAAAUUAAUCAUUUAGA
env-1254  AGUUGCCUCA..UUU............UGAGGUUGC.......ACUUGCGUGUUAAUAGCC..AA..GUUC.........UUUGGCC.GAAUU.UGGUGGUUAGAG.AAUCGCAAGAAAAAGAUAAUUUAUUGAUUUAUGGGCGAAUGCCCACUUAGUAACUACAAAUUAAUCAUUUAGA
env-1256  AGUUGCCUCA..UUUU...........UGAGGUUGC.......ACUUGCGUGUUAAUAGCC..AA..GCUC.........UUUGGCC.GAACU.UGGUGGUUAGAG.AAUCGCAAGAAAAAGAUAAUUUAUUGAUUUAUGGGCAUUCGCCCACUUAGUAACUACAAAUUAAUCACUUAGA
env-1258  AGUUGCCUC...UUUUU...........GAGGUCGC.......ACUUGCGUGUUAAUAGCC..AA..GCUC.........UUUGGCC.GAACU.UGGUGGUUAGAG.AAUCGCAAGAAAAAGAUAAUUUAUUGAUUUAUGGGCGAAAGCCCACUUAGUAACUACAAAUUAAUCAUUUAGA
env-1259  AGUUGCCUC...UUUUU...........GAGGUCGC.......ACUUGCGUGUUAAUAGCC..AA..GCUC.........UUUGGCC.GAACU.UGGUGGUUAGAG.AAUCGCAAGAAAAAGAUAAUUUAUUGAUUUAUGGGCGAAAGCCCACUUAGUAACUACAAAUUAAUCAUUUAGA
Pmu-2-1   GGGCAGCU....GUCUUAG..........AGCUGCC..ACGGCAUUGGCGUGUUAAUAGGG..CU..GUGC..........AUGGCC.GUACA.GUCUGGUUAGAG.AAUCGCCCGUGCGCUCAGGGUUAAAGCUGUGGAUGGUCUCUUUUAGACUGGAGGCACCGCGAGGCCCCUGUUC
Pde-1-1   CGGGGC......ACACUUAGA..........GCCCC...AUACACGACCGUGUUAAUAGUC..GC..GCCU..........UUGGCC.GGACG.CGACGGUUAGUG.AAUCGGCCGUGGGGCAGGGCAAAAGCCAUGGAUGGUCUCUUUUAGACUGGAGGCGCCGUGAGGUCUCUGCCUG
          .<<<<<<<<<<<.............>>>>>>>>>>>................<<<<<.<<<..<<..<<<<<...............>>>>>>.>>>>.>>>>>..............................................................................
          .222222.1111.............1111.222222................02200.122..22..2222................22222.2221.00220..............................................................................
          ●GGGG●○-----R●●●○○○○----------○●CCCC----○○●●●●●●YGUGU●AAUAGY●--●Y--RYYU----------UUGGCC-GRRY●-●●RYGGUU●GUG-AAUCR●●●●●●●RR●●R●●●●●●●●●●●●●●●●●●●●●●●●●●●●●●●●●●●●●●●●●●●●●●●●●●●●●●●●
           5′                                                                                                          3′
```

alignment positions 260 · · · 267

```
env-1     UUUCUGCU
env-2     UGUUUUUA
env-3     GACCAUUU
env-4     UUAAUGUA
env-5     AUCUGAAG
env-6     UAAGUUUC

          ........
          ........
          ●●●●R●●●
```

env-7	UUUCAAGA
env-8	....
env-9	CAAGCCUA
env-10	GCCUAAUG
env-11	ACAAGACC
env-12	AU......
env-13	....
env-14	ACCAUUUC
env-15	UAAACGAU
env-16	UAACAAAA
env-17	AAGUUACA
env-18	AAGUUACA
env-21	AGUUACAA
env-22	AAGUUACA
env-26	AGUUACAA
env-27	AAGUUACA
env-63	AAGUUACA
env-64	....
env-70	AACAGAAG
env-71	UAUUAACA
env-72	UAAGAAAC
env-73	ACAGAAGU
env-74	AAAACAAA
env-75	ACAAGACC
env-76	UUACAAGA
env-77	AGAUAAAA
env-78	GCUACAAG
env-79	CAAGACCA
env-80	CAAGACCA
Pbr-3-1	UUCAAUAG
env-81	UUCAAUAG
env-82	UAAGAAUG
env-83	AUUAAAAG
env-84	UCAUGAAU
env-86	UCAUGAAU
env-87	UGAGCUAC
Pal-1-1	GAUUCGAC
env-88	GGACACUU
env-98	GGACACUU
env-126	GGACACUU
env-129	GGACACUU
env-132	GGACACUU
env-148	GGACACUU
env-155	GGACACUU
env-157	UGGACACU
env-158	....
env-159	ACAAAACC
env-160	ACAAAACC
env-163	ACAAAACC
env-164	ACAAAACC
env-165	ACAAAACC
env-166	ACAAAACC
env-167	ACAAAACC
env-168	ACAAAACC
env-169	ACAAAACC
env-170	ACAAAACC
env-171	ACAAAACC
env-205	ACAAAACC
env-206	ACAAAACC
env-207	ACAAAACC
env-208	ACAAAACC
env-209	ACAAAACC
env-210	ACAAAACC
env-211	ACAAAACC
env-212	ACAAAACC
env-213	ACAAAACC
env-222	ACAAAACC
env-226	ACAAAACC
env-227	ACAAAACC
env-229	ACAAAACC
env-230	....
env-231	ACAAAACC
env-232	ACAAAACC
env-233	....
env-234	ACAAAACC
env-246	ACAAAACC
env-248	....
env-249	ACAAAACC
env-250	ACAAAACC
env-257	ACAAAACC
env-258	ACAAAACC
env-259	....
env-260	ACAAAACC
env-262	ACAAAACC
	....
	....
	●●●●R●●●

env-264	ACAAAACC
env-265	ACAAAACC
env-266	AAUGGACA
env-267	CAUUUCCG
Bsp-2-1	CAUUUCCG
env-268	....
env-269	....
env-270	UUCAAACC
env-272	....
env-273	....
env-275	....
env-276	....
env-278	....
env-279	UUCAGACA
env-280	....
env-281	....
env-285	CAUUGAAU
env-286	....
env-287	UUCAGGUA
env-288	UUCAGGUA
env-290	UUCAAGUA
env-291	UUCAAACC
env-292	NNNNNNNN
env-293	UUCAGGUA
env-295	....
env-297	....
env-298	UUCAGGUA
env-299	....
env-301	....
env-302	....
env-305	UUCAGGUA
env-307	UUCAGGUA
env-308	UUCAGGUA
env-309	UUCAGGUA
env-310	....
env-311	....
env-314	....
env-315	UUCAAGUA
env-316	....
env-317	UCACAAAG
env-318	....
env-319	UUCAAUAC
Psp-3-1	CAUGUGUU
env-320	CUAUAUCA
env-321	CUAUAUCA
env-322	CUAUAUCA
env-323	....
env-324	CUAUAUCA
env-326	CUAUAUCA
env-327	CUAUAUCA
env-328	....
env-329	CUAUAUCA
env-330	CUAUAUCA
env-331	CUAUAUCA
env-336	CUAUAUCA
env-337	CUAUAUCA
env-338	CUAUAUCA
env-339	CUAUAUCA
env-341	CUAUAUCA
env-345	CUAUAUCA
env-347	CUAUAACA
env-348	CUAUAUCA
env-349	....
Pma-3-1	CAAAGCAU
env-350	CAAAGCAU
env-351	CAAAGCAU
Pma-1-1	CAAAGCAU
env-353	....
env-354	....
env-355	....
env-357	UCAUUGAU
env-359	UCAUUGAU
env-360	....
env-361	UCAUUGAU
env-362	....
env-363	....
env-368	UCAUUGAU
env-369	UCAUUGAU
env-370	UCAUUGAU
env-371	UCAUUGAU
env-373	....
env-375	UCAUUGAU
env-384	UCAUUGAU
env-385	UCAUUGAU
	....
	....
	●●●●R●●●

env-386	....
env-387	UCAUUGAU
env-388	....
env-389	UCAUUGAU
env-390	UCAUUGAU
env-392	UCAUUGAU
env-396	UCAUUGAU
env-397	....
env-398	UCAUUGAU
env-399	UCAUUGAU
env-400	UCAUUGAU
env-401	UCAUUGAU
env-402	UCAUUGAU
env-403	UCAUUGAU
env-406	UCAUUGAU
env-408	CGAAAAGU
env-410	CGAAAAGU
env-411	CGAAAAGU
env-412	UCAUUGAU
Por-6-1	CGAAAAGU
Por-5-1	CGAAAAGU
env-413	....
env-414	CGAAAAGU
env-418	....
env-419	....
env-420	CGAAGGUA
env-421	CGAAAAGU
env-422	....
env-423	....
Psp-8-1	CGAAGGUA
env-425	CGAAGGUA
env-426	....
env-429	CGAAGGUA
env-431	CGAAGGUA
env-432	CGAAGGUA
env-433	....
env-434	....
env-435	CGAAGGUA
env-436	CGAAGGUA
env-437	CGAAGGUA
env-438	CGAAGGUA
env-439	CGAAGGUA
env-440	CGAAGGUA
env-441	....
env-442	....
env-443	....
Pou-1-1	CGAAGGUA
env-444	....
env-445	CGAAGGUA
env-446	CGAAGGUA
env-447	CGAAGGUA
Pou-2-1	CGAAGGUA
env-448	GUUACAAG
env-449	....
Pba-2-1	UCGAAUAC
Pba-1-1	UCGAAUAC
env-450	UCGAAUGU
Pde-2-1	UACAAGAC
Pbe-1-1	AAUAAGGU
env-451	ACUAUUUC
env-452	GCACAAGC
env-453	CAAGACUG
env-454	AAGACUAU
env-455	AAGACUAU
env-457	UUAUAAAA
env-458	....
env-462	UAUAAAAC
env-463	UAUAAAAC
env-464	UUAUAAAA
env-466	AAUGAGUU
env-467	UGGCGGUA
env-468	....
env-469	GACAAUUU
env-470	ACAAGACA
Ppa-3-1	AACAAUGU
env-471	AAUAAUGU
env-473	AAUAAUGU
env-474	AAUAAUGU
env-476	AAUAAUGU
env-477	AAUAAUGU
env-483	UAAGGAAC
env-485	UAAUAGGA
env-486	AGCAAUAC
env-487	AGUUAGAC
	....
	....
	●●●●R●●●

env-488	AACAAAUU
env-489	ACAAGACU
env-490	AAGACUAU
env-491	AAGACUAU
env-492	AAGACUAU
env-493	AAGACUAU
Pam-1-1	AAGACUAU
Pam-3-1	AAGACUAU
Pbi-1-1	CAAGACUA
Pve-1-1	CAAGACUA
Pve-3-1	ACAAGACU
env-494	....
env-495	UACAAGAC
env-497	UACAAGAC
Psc-1-1	ACAAGACU
env-498	....
Psp-6-1	UACAAGAC
Pme-3-1	UACAAGAC
env-501	UACAAGAC
env-502	....
env-503	....
env-504	UUACAAGA
env-507	....
Phi-2-1	UUACAAGA
env-508	UUACAA..
env-509	AACUUUCA
Phi-2-2	UUACAAGA
env-510	UACAAGAC
env-511	UACAANNN
env-512	UACAAGAC
env-514	UACAAGAC
env-516	....
env-517	....
env-519	UACAAGAC
Psp-5-1	UACAAGAC
env-520	UACAAGAC
env-521	UACAAGAC
env-522	UACAAGAC
env-523	UACAA...
env-525	CUUUGCUC
env-526	ACAAGACU
env-527	UACAAGAC
env-531	UACAAGAC
env-532	ACAAGACU
env-534	UACA....
env-535	UACAAGAC
env-537	UACAAGAC
env-538	UACAAGAC
env-552	ACAAGACU
env-553	....
env-554	UACAAGAC
env-555	UACAAGAC
env-557	UACAAGAC
env-558	NNNNNNNN
env-559	UACAAGAC
env-560	....
env-561	UACAAGAC
Pfu-1-1	ACAAGACU
Psp-24-1	ACAAGACU
env-562	....
env-563	....
env-564	ACA.....
Pde-3-1	ACAAGACU
env-565	....
Pde-4-1	ACAAGACU
Pde-5-1	CAAGACUA
env-566	CAAGA...
env-567	ACAAGA..
Pde-6-1	CAAGACUA
env-568	....
env-569	CAA.....
Pmu-1-1	CAAGACUA
env-570	....
env-571	....
env-572	AAUUAAUA
env-573	....
env-574	UAACAGGU
env-575	AUCAGGUA
env-576	AUCAGGUA
env-577	UAAGUUAC
env-578	UUAUAAGA
env-579	....
env-580	UUAUAAGA
env-582	UUAUAAGA
	....
	....
	●●●●R●●●

Pin-1-1	UAAGACUA
env-583	AU......
env-589	AUAAGACU
env-592	GGUAGUAG
env-593	AUAAGACU
env-594	AGACCAGA
env-595	AUAAGACU
env-596	AU......
env-597	GGUCGCCU
env-598	....
env-599	AUAAGAC.
env-600	AUAAGACU
env-613	AUAAGACU
env-648	AUAAGACU
env-651	AUAAGACU
env-652	CCUCUGCU
env-653	....
env-657	....
env-676	UACAAGAC
Pco-3-1	UACAAGAC
Pni-1-1	UAAGACUA
env-677	UAAGACUA
env-679	UAAGACUA
env-680	CAAGACU.
env-681	CA......
env-682	UAAGACUA
env-683	UAAGACUA
env-684	UAAGACUA
env-686	UAAGACUA
env-696	UAAGACUA
env-698	....
env-699	....
Pni-3-1	UAAGACUA
env-700	....
env-709	....
env-710	UAAGACUA
env-711	UAAGACUA
env-724	UAAGACUA
Pau-1-1	AGUUAUUA
Psp-9-1	AAGACUAU
env-725	UAAGACUA
env-726	UAAGACUA
env-727	....
env-729	UAAGACUA
env-733	UAAGACUA
env-734	UAAGACUA
env-735	UAAGACUA
env-736	UAAGACUA
Pin-3-1	UAAGACUA
Pfa-1-1	AAGACUAU
env-737	UAAGACUA
env-738	UAAGACUA
env-742	UAAGACUA
Pdi-3-1	AAGACUAU
env-743	....
Pdi-2-1	AAGACUAU
Pdi-1-1	AAGACUAU
env-744	GGACACA.
env-745	GACACAUU
env-746	UGGACACA
env-747	UGGACACA
env-748	GGACACAU
env-749	GGACACAU
env-751	GGACACAU
env-752	....
env-753	CACAUUAA
env-754	GGACACAU
env-755	GUAAUCGA
env-756	UGGACACA
env-757	CAUUAAGU
env-758	AAUGGACA
env-759	AAAUGGAC
env-760	GGACACAU
env-761	....
env-762	ACAUUAAG
env-763	ACAUUAAG
env-765	....
env-766	ACAUUAAG
env-767	ACAUUAAG
env-768	CAUUAAGU
env-769	....
env-770	CAUUAAGU
env-771	CAUUAAGU
env-772	CAUUAAGU
	....
	....
	••••R•••

env-774	UUUCAAGA
env-775	CAUUAAGU
env-776	CAUUAAGU
env-780	UUUCAAGA
env-781	CACAUUAA
env-785	ACAUUAAG
env-787	....
env-788	CACAUUAA
env-789	CAUUAAGU
env-790	CAUUAAGU
env-791	....
env-793	CACAUUAA
env-797	....
env-798	AUUAAGUU
env-799	CACAUUAA
env-800	ACAUUAAG
env-801	CACAUUAA
env-802	CACAUUAA
env-803	ACAUUAAG
env-804	CACAUUAA
env-808	CACAUUAA
env-821	UAAGUUAC
env-822	ACACAUUA
env-824	CAUUAAGU
env-825	UGGACACA
env-826	CACAUUAA
env-827	CACAUUAA
env-828	CACAUUAA
env-829	AAGUUACA
env-830	GGACACAU
env-831	ACAUUAAG
env-832	ACAUUAAG
env-833	AGUUACAA
env-834	....
env-835	CAUUAAGU
env-836	CACAUUAA
env-837	CACAUUAA
Pco-2-1	CACAUUAA
env-838	....
env-839	CACAUUAA
env-840	ACAUUAAG
env-842	ACAUUAAG
env-843	ACAUUAAG
Por-8-1	AGACCAUU
Psp-21-1	AUCAAAUC
env-845	GGAUACUU
env-846	GGAUACUU
env-847	AGGUGGAA
env-848	AGACUAUU
env-849	....
env-851	AGACUAUU
env-852	A.......
env-855	AGACUAUU
env-856	AGACUGUU
env-857	A.......
env-858	AGACUAUU
env-859	AGACUAUU
env-860	AGACUAUU
env-861	....
env-862	AGACUAUU
env-864	AGACUAUU
env-865	AGACUAUU
env-866	....
env-868	....
env-869	AGACUAUU
env-870	AGACUAUU
env-872	....
env-873	....
env-874	AGACUAUU
env-875	....
env-876	AGACUAUU
Psp-25-1	AGACUAUU
env-877	AGACUAUU
env-878	A.......
env-880	AGACUAUU
env-881	....
env-882	....
env-883	....
env-885	AGACUAUU
env-886	AAGACUAU
env-887	ACAAGACU
env-888	ACAAGACU
env-889	....
env-890	....
	....
	....
	●●●●R●●●

env-891	ACAAGACU
env-892	ACAAGACU
env-893	....
env-894	ACAAGACU
env-900	ACAAGACU
env-901	....
env-902	ACAAGACU
Psp-26-1	ACAAGACU
env-903	....
env-921	A.......
env-922	AGACUAUU
env-923	A.......
env-924	AGACUAUU
env-932	....
env-948	AGACUAUU
env-949	AGACUAUU
env-950	A.......
env-953	A.......
env-957	A.......
env-958	AGACUAUU
env-960	GACUGUUU
env-961	GACUGUUU
env-967	AGCCUAGA
env-968	ACAAGACU
env-970	ACAAGACU
env-971	ACAAGACU
env-972	CAAGACUA
env-973	CAAGACUA
env-977	CAAGACUA
env-978	CAAGACUA
env-979	CAAGACUA
env-981	CAAGACUA
env-982	CAAGACUA
env-983	CAAGACUA
env-984	CAAGACUA
env-985	CAAGACUA
env-986	CAAGACUA
env-990	CAAGACUA
env-992	CAAGACUA
env-993	....
env-994	GACUGUUU
Pbu-3-1	UGUAAUUA
Pbu-1-1	UGUAAUUA
Pbu-2-1	UGUAAUUA
Psp-12-1	UGUAAUUA
env-995	....
env-996	GAAAGUAA
env-997	UGUCGAAA
env-999	UAUAAUAU
Por-1-1	UUUCAAAA
env-1001	....
Por-2-1	UUUCAAAA
env-1002	AUAAGACU
env-1003	AUAAGACU
Pti-3-1	AUAAGACU
env-1004	UAUAAGAC
Pti-1-1	AUAAGACU
Pti-2-1	AUAAGACU
env-1005	AUAAGACU
env-1006	....
Psp-4-1	UACAAGAC
env-1007	UACAAGAC
Pbu-5-1	UACAAGAC
Pbu-4-1	UACAAGAC
env-1008	NNNNNNNN
Pen-1-1	CAAAACUA
env-1009	CAAAACUA
env-1017	CAAAACUA
env-1020	CAAAACUA
Ppl-2-1	CAAAACUA
env-1021	GUUACAAG
env-1022	GUUACAAG
env-1023	GUUACAAG
env-1024	....
env-1025	UACAAGAC
env-1027	UUACAAGA
Pma-4-1	UUACAAGA
env-1028	UUACAAGA
env-1029	UUUAAGUU
env-1031	UUUAAGUU
env-1032	UAUUGAAG
env-1033	UAUUGAAG
env-1034	AGUUACAA
env-1035	CAAGACUA
	....
	....
	●●●●R●●●

env-1037	UAGUAAAU
env-1038	AAGUUACA
env-1039	AAGUUACA
env-1040	AAGUUACA
env-1043	AAGUUACA
env-1044	AAGUUACA
env-1046	AAGUUACA
env-1052	UUACAAAA
env-1053	AAGUUACA
Psp-2-1	AAGUUACA
env-1054	AAGUUACA
env-1055	AAGUUACA
env-1056	AAGUUACA
env-1058	UUACAAGA
env-1059	AGUUACAA
env-1060	AGUUACAA
env-1061	UGAAGAAA
Psp-13-1	UGAAGAAA
env-1062	UGAAGAAA
Psp-17-1	GUGAAGAA
env-1063	GUGAAGAA
Psp-15-1	UGAAGAAA
Psp-16-1	GUGAAGAA
env-1064	UGGAAAGA
env-1065	....
env-1066	UGGAAAGA
env-1067	ACAAGACU
Hse-1-1	CAUCAAGG
env-1068	UAAAAUGA
env-1069	AACAAUUC
env-1071	AUAAUUAA
env-1072	UCGGAUGC
env-1074	UCGGAUGC
env-1075	UCGGAUGC
env-1076	....
env-1077	GAUUAUUU
env-1078	GUUAGACC
env-1079	GACCUCGG
env-1080	GUUAGACC
env-1081	....
env-1083	AGUUAGAC
env-1084	AAGAGAUA
env-1085	AGAGAGUA
env-1086	....
env-1087	....
env-1088	....
env-1089	....
env-1090	....
env-1091	....
env-1092	AUAUAAAU
env-1093	GAGAGAAA
env-1094	AGACCUCG
env-1095	GAGUUAGA
env-1096	GCCUGGGA
env-1097	AGAACAAA
env-1098	AUGUGGGU
Psp-7-1	UGUGAGUU
env-1099	UUUAAUUA
env-1100	UUUAAUUA
Psp-11-1	GACCCUGG
Psp-19-1	AGACCCUG
env-1101	....
Pru-1-1	UUUAAUUA
env-1102	....
env-1103	UUGAAAUG
env-1104	CUUUAAGU
Pbr-1-1	UUAAGUUA
Pbr-2-1	UUAAGUUA
env-1105	....
env-1106	....
env-1107	....
env-1108	....
env-1109	GUGGGUGG
env-1110	UGCGGGUG
env-1111	GCGGGUGG
env-1112	UAAGUUAC
env-1113	AAUUAUCG
env-1114	UGAUAAAA
env-1115	UGAUAAAA
env-1116	UUUAAGUU
env-1117	AUGUUUUU
env-1118	UUACAAGA
env-1119	UUACAAGA
env-1120	UAAGAAUU
	....
	....
	●●●●R●●●

```
env-1122   AAGAAUUA
env-1123   CGAAAUAA
env-1125   UAAGUUAC
env-1126   ACAAGACU
env-1127   ACAAGACU
env-1129   ACAUUAAG
env-1130   ACUUAUGU
env-1131   UAAUGUGA
env-1132   AGAAGACC
env-1133   UACAAGAC
env-1134   CCACAAAC
env-1135   AAAUGUGA
env-1136   CAAGACCA
env-1137   UUAGAAUG
env-1138   UUAGAAUG
env-1139   CUUAGAAU
env-1140   CUUAGAAU
env-1141   CUUAGAAU
env-1143   CUUAGAAU
env-1144   CUUAGAAU
env-1145   CUUAGAAU
env-1147   UGGACACU
env-1148   GGACACUU
env-1149   UGGACACU
env-1151   UGGACACU
env-1153   UGGACACU
env-1154   GGACACUU
env-1155   AUCGUUGA
env-1156   UGGACACU
env-1157   GGACACUU
env-1158   UGGACACU
env-1159   UGGACACU
env-1163   UGGACACU
env-1164   UGGACACU
env-1165   UGGACACU
env-1166   AUGGACAC
env-1167   UGGACACU
env-1192   CCAUUUCU
env-1193   AUGGAUAC
env-1194   AUGGAUAC
Psp-1-1    AUGGAUAC
env-1195   ........
env-1197   AUGGAUAC
env-1203   AUGGAUAC
env-1205   AUGGAUAC
env-1206   AUGGAUAC
env-1211   ........
env-1213   AUGGAUAC
env-1216   UGGAUACU
env-1223   UGGAUACU
env-1224   UGGAUACU
env-1228   ........
env-1233   UGGAUACU
env-1243   UGGAUACU
env-1244   ........
env-1245   AUGGAUAC
env-1250   AUGGAUAC
env-1251   AUGGAUAC
env-1254   AUGGAUAC
env-1256   AUGGAUAC
env-1258   AUGGAUAC
env-1259   AUGGAUAC
Pmu-2-1    GUCAACAG
Pde-1-1    GUAUCAAG
           ........
           ........
           ••••R•••
```

4 eL15-Euryarchaeota

4.1 Taxa

The taxonomy of each organism containing a putative eL15-Euryarchaeota RNA is listed, with abbreviations identifying each hit (e.g., "Eco-1-1" and "Eco-1-2" might hypothetically represent two distinct RNAs in *E. coli*). The abbreviations will be used to identify each individual eL15-Euryarchaeota RNA in Sections 4.2 and 4.4:.

**abbrev. of hits**	**taxonomy of species**
har-1-1	halophilic archaeon DL31 Archaea
ume-1-1	Archaea Euryarchaeota environmental samples uncultured methanogenic archaeon RC-I
h3A-1-1	Archaea Euryarchaeota Halobacteria Halobacteriales haloarchaeon 3A1_DGR
Hsa-1-1	Archaea Euryarchaeota Halobacteria Halobacteriales Haloarculaceae *Halapricum salinum*
Hsp-1-1	Archaea Euryarchaeota Halobacteria Halobacteriales Haloarculaceae *Haloarcula sp.* CBA1115
CHa-1-1	Archaea Euryarchaeota Halobacteria Halobacteriales Halobacteriaceae *Candidatus* Halobonum tyrrellensis G22
Hci-1-1	Archaea Euryarchaeota Halobacteria Halobacteriales Halobacteriaceae *Haladaptatus cibarius* D43
Hpa-1-1 to Hpa-1-2	Archaea Euryarchaeota Halobacteria Halobacteriales Halobacteriaceae *Haladaptatus paucihalophilus* DX253
Hje-1-1	Archaea Euryarchaeota Halobacteria Halobacteriales Halobacteriaceae *Halalkalicoccus jeotgali* B3
Hsu-1-1	Archaea Euryarchaeota Halobacteria Halobacteriales Halobacteriaceae *Halanaeroarchaeum sulfurireducens*
Hac-1-1 to Hac-1-2	Archaea Euryarchaeota Halobacteria Halobacteriales Halobacteriaceae *Halarchaeum acidiphilum* MH1-52-1
Ham-1-1	Archaea Euryarchaeota Halobacteria Halobacteriales Halobacteriaceae *Haloarcula amylolytica* JCM 13557
Har-1-1	Archaea Euryarchaeota Halobacteria Halobacteriales Halobacteriaceae *Haloarcula argentinensis* DSM 12282
Hca-1-1	Archaea Euryarchaeota Halobacteria Halobacteriales Halobacteriaceae *Haloarcula californiae* ATCC 33799
Hhi-1-1	Archaea Euryarchaeota Halobacteria Halobacteriales Halobacteriaceae *Haloarcula hispanica* ATCC 33960
Hhi-2-1	Archaea Euryarchaeota Halobacteria Halobacteriales Halobacteriaceae *Haloarcula hispanica* N601
Hja-1-1	Archaea Euryarchaeota Halobacteria Halobacteriales Halobacteriaceae *Haloarcula japonica* DSM 6131
Hma-1-1	Archaea Euryarchaeota Halobacteria Halobacteriales Halobacteriaceae *Haloarcula marismortui* ATCC 43049
Hsa-2-1	Archaea Euryarchaeota Halobacteria Halobacteriales Halobacteriaceae *Haloarcula salaria*
Hsi-1-1	Archaea Euryarchaeota Halobacteria Halobacteriales Halobacteriaceae *Haloarcula sinaiiensis* ATCC 33800
Hva-1-1	Archaea Euryarchaeota Halobacteria Halobacteriales Halobacteriaceae *Haloarcula vallismortis* ATCC 29715
Hsa-3-1	Archaea Euryarchaeota Halobacteria Halobacteriales Halobacteriaceae *Halobacterium salinarum* R1
Hsp-2-1	Archaea Euryarchaeota Halobacteria Halobacteriales Halobacteriaceae *Halobacterium sp.* DL1
Hsp-3-1	Archaea Euryarchaeota Halobacteria Halobacteriales Halobacteriaceae *Halobacterium sp.* NRC-1
Hla-1-1 to Hla-1-2	Archaea Euryarchaeota Halobacteria Halobacteriales Halobacteriaceae *Halobiforma lacisalsi* AJ5
Hni-1-1	Archaea Euryarchaeota Halobacteria Halobacteriales Halobacteriaceae *Halobiforma nitratireducens* JCM 10879
Hha-1-1 to Hha-1-2	Archaea Euryarchaeota Halobacteria Halobacteriales Halobacteriaceae *Halococcus hamelinensis* 100A6
Hmo-1-1	Archaea Euryarchaeota Halobacteria Halobacteriales Halobacteriaceae *Halococcus morrhuae* DSM 1307
Hsa-4-1	Archaea Euryarchaeota Halobacteria Halobacteriales Halobacteriaceae *Halococcus saccharolyticus* DSM 5350
Hsa-5-1	Archaea Euryarchaeota Halobacteria Halobacteriales Halobacteriaceae *Halococcus salifodinae* DSM 8989
Hth-1-1	Archaea Euryarchaeota Halobacteria Halobacteriales Halobacteriaceae *Halococcus thailandensis* JCM 13552
Hal-1-1	Archaea Euryarchaeota Halobacteria Halobacteriales Halobacteriaceae *Haloferax alexandrinus* JCM 10717
Hde-1-1	Archaea Euryarchaeota Halobacteria Halobacteriales Halobacteriaceae *Haloferax denitrificans* ATCC 35960
Hel-1-1	Archaea Euryarchaeota Halobacteria Halobacteriales Halobacteriaceae *Haloferax elongans* ATCC BAA-1513
Hgi-1-1	Archaea Euryarchaeota Halobacteria Halobacteriales Halobacteriaceae *Haloferax gibbonsii* ATCC 33959
Hla-2-1	Archaea Euryarchaeota Halobacteria Halobacteriales Halobacteriaceae *Haloferax larsenii* JCM 13917
Hlu-1-1	Archaea Euryarchaeota Halobacteria Halobacteriales Halobacteriaceae *Haloferax lucentense* DSM 14919
Hme-1-1 to Hme-1-2	Archaea Euryarchaeota Halobacteria Halobacteriales Halobacteriaceae *Haloferax mediterranei* ATCC 33500
Hmu-1-1	Archaea Euryarchaeota Halobacteria Halobacteriales Halobacteriaceae *Haloferax mucosum* ATCC BAA-1512
Hpr-1-1	Archaea Euryarchaeota Halobacteria Halobacteriales Halobacteriaceae *Haloferax prahovense* DSM 18310
Hsp-4-1	Archaea Euryarchaeota Halobacteria Halobacteriales Halobacteriaceae *Haloferax sp.* ATCC BAA-644
Hsp-5-1	Archaea Euryarchaeota Halobacteria Halobacteriales Halobacteriaceae *Haloferax sp.* ATCC BAA-645
Hsp-6-1	Archaea Euryarchaeota Halobacteria Halobacteriales Halobacteriaceae *Haloferax sp.* ATCC BAA-646
Hsp-7-1	Archaea Euryarchaeota Halobacteria Halobacteriales Halobacteriaceae *Haloferax sp.* BAB2207
Hsu-2-1	Archaea Euryarchaeota Halobacteria Halobacteriales Halobacteriaceae *Haloferax sulfurifontis* ATCC BAA-897
Hvo-1-1	Archaea Euryarchaeota Halobacteria Halobacteriales Halobacteriaceae *Haloferax volcanii* DS2
Hbo-1-1	Archaea Euryarchaeota Halobacteria Halobacteriales Halobacteriaceae *Halogeometricum borinquense* DSM 11551
Hsa-6-1	Archaea Euryarchaeota Halobacteria Halobacteriales Halobacteriaceae *Halogranum salarium* B-1
Hka-1-1	Archaea Euryarchaeota Halobacteria Halobacteriales Halobacteriaceae *Halomicrobium katesii* DSM 19301
Hmu-2-1	Archaea Euryarchaeota Halobacteria Halobacteriales Halobacteriaceae *Halomicrobium mukohataei* DSM 12286
Hxa-1-1	Archaea Euryarchaeota Halobacteria Halobacteriales Halobacteriaceae *Halopiger xanaduensis* SH-6
Hwa-1-1	Archaea Euryarchaeota Halobacteria Halobacteriales Halobacteriaceae *Haloquadratum walsbyi* C23
Hwa-2-1	Archaea Euryarchaeota Halobacteria Halobacteriales Halobacteriaceae *Haloquadratum walsbyi* DSM 16790
Hti-1-1	Archaea Euryarchaeota Halobacteria Halobacteriales Halobacteriaceae *Halorhabdus tiamatea* SARL4B
Hut-1-1	Archaea Euryarchaeota Halobacteria Halobacteriales Halobacteriaceae *Halorhabdus utahensis* DSM 12940
Hai-1-1	Archaea Euryarchaeota Halobacteria Halobacteriales Halobacteriaceae *Halorubrum aidingense* JCM 13560
Har-2-1	Archaea Euryarchaeota Halobacteria Halobacteriales Halobacteriaceae *Halorubrum arcis* JCM 13916
Hca-2-1	Archaea Euryarchaeota Halobacteria Halobacteriales Halobacteriaceae *Halorubrum californiensis* DSM 19288
Hco-1-1	Archaea Euryarchaeota Halobacteria Halobacteriales Halobacteriaceae *Halorubrum coriense* DSM 10284
Hdi-1-1	Archaea Euryarchaeota Halobacteria Halobacteriales Halobacteriaceae *Halorubrum distributum* JCM 10118
Hdi-2-1	Archaea Euryarchaeota Halobacteria Halobacteriales Halobacteriaceae *Halorubrum distributum* JCM 9100
Hho-1-1	Archaea Euryarchaeota Halobacteria Halobacteriales Halobacteriaceae *Halorubrum hochstenium* ATCC 700873
Hko-1-1	Archaea Euryarchaeota Halobacteria Halobacteriales Halobacteriaceae *Halorubrum kocurii* JCM 14978
Hla-3-1	Archaea Euryarchaeota Halobacteria Halobacteriales Halobacteriaceae *Halorubrum lacusprofundi* ATCC 49239

Hli-1-1	Archaea Euryarchaeota Halobacteria Halobacteriales Halobacteriaceae *Halorubrum lipolyticum* DSM 21995
Hli-2-1	Archaea Euryarchaeota Halobacteria Halobacteriales Halobacteriaceae *Halorubrum litoreum* JCM 13561
Hsa-7-1	Archaea Euryarchaeota Halobacteria Halobacteriales Halobacteriaceae *Halorubrum saccharovorum* DSM 1137
Hsp-8-1	Archaea Euryarchaeota Halobacteria Halobacteriales Halobacteriaceae *Halorubrum sp.* T3
Hte-1-1	Archaea Euryarchaeota Halobacteria Halobacteriales Halobacteriaceae *Halorubrum tebenquichense* DSM 14210
Hte-2-1	Archaea Euryarchaeota Halobacteria Halobacteriales Halobacteriaceae *Halorubrum terrestre* JCM 10247
Hpa-2-1	Archaea Euryarchaeota Halobacteria Halobacteriales Halobacteriaceae *Halosarcina pallida* JCM 14848
Hca-3-1	Archaea Euryarchaeota Halobacteria Halobacteriales Halobacteriaceae *Halosimplex carlsbadense* 2-9-1
Hli-3-1	Archaea Euryarchaeota Halobacteria Halobacteriales Halobacteriaceae *Haloterrigena limicola* JCM 13563
Hsa-8-1	Archaea Euryarchaeota Halobacteria Halobacteriales Halobacteriaceae *Haloterrigena salina* JCM 13891
Hth-2-1	Archaea Euryarchaeota Halobacteria Halobacteriales Halobacteriaceae *Haloterrigena thermotolerans* DSM 11522
Htu-1-1	Archaea Euryarchaeota Halobacteria Halobacteriales Halobacteriaceae *Haloterrigena turkmenica* DSM 5511
Has-1-1	Archaea Euryarchaeota Halobacteria Halobacteriales Halobacteriaceae *Halovivax asiaticus* JCM 14624
Nae-1-1	Archaea Euryarchaeota Halobacteria Halobacteriales Halobacteriaceae *Natrialba aegyptia* DSM 13077
Nas-1-1	Archaea Euryarchaeota Halobacteria Halobacteriales Halobacteriaceae *Natrialba asiatica* DSM 12278
Nch-1-1	Archaea Euryarchaeota Halobacteria Halobacteriales Halobacteriaceae *Natrialba chahannaoensis* JCM 10990
Nhu-1-1	Archaea Euryarchaeota Halobacteria Halobacteriales Halobacteriaceae *Natrialba hulunbeirensis* JCM 10989
Nma-1-1	Archaea Euryarchaeota Halobacteria Halobacteriales Halobacteriaceae *Natrialba magadii* ATCC 43099
Nta-1-1	Archaea Euryarchaeota Halobacteria Halobacteriales Halobacteriaceae *Natrialba taiwanensis* DSM 12281
Nal-1-1	Archaea Euryarchaeota Halobacteria Halobacteriales Halobacteriaceae *Natrinema altunense* JCM 12890
Nga-1-1	Archaea Euryarchaeota Halobacteria Halobacteriales Halobacteriaceae *Natrinema gari* JCM 14663
Npa-1-1	Archaea Euryarchaeota Halobacteria Halobacteriales Halobacteriaceae *Natrinema pallidum* DSM 3751
Npe-1-1	Archaea Euryarchaeota Halobacteria Halobacteriales Halobacteriaceae *Natrinema pellirubrum* DSM 15624
Nsp-1-1	Archaea Euryarchaeota Halobacteria Halobacteriales Halobacteriaceae *Natrinema sp.* J7-2
Nve-1-1	Archaea Euryarchaeota Halobacteria Halobacteriales Halobacteriaceae *Natrinema versiforme* JCM 10478
Ngr-1-1	Archaea Euryarchaeota Halobacteria Halobacteriales Halobacteriaceae *Natronobacterium gregoryi* SP2
Nam-1-1	Archaea Euryarchaeota Halobacteria Halobacteriales Halobacteriaceae *Natronococcus amylolyticus* DSM 10524
Nje-1-1	Archaea Euryarchaeota Halobacteria Halobacteriales Halobacteriaceae *Natronococcus jeotgali* DSM 18795
Noc-1-1	Archaea Euryarchaeota Halobacteria Halobacteriales Halobacteriaceae *Natronococcus occultus* SP4
Nin-1-1	Archaea Euryarchaeota Halobacteria Halobacteriales Halobacteriaceae *Natronolimnobius innermongolicus* JCM 12255
Nmo-1-1	Archaea Euryarchaeota Halobacteria Halobacteriales Halobacteriaceae *Natronomonas moolapensis* 8.8.11
Nph-1-1	Archaea Euryarchaeota Halobacteria Halobacteriales Halobacteriaceae *Natronomonas pharaonis* DSM 2160
Nba-1-1	Archaea Euryarchaeota Halobacteria Halobacteriales Halobacteriaceae *Natronorubrum bangense* JCM 10635
Nsu-1-1	Archaea Euryarchaeota Halobacteria Halobacteriales Halobacteriaceae *Natronorubrum sulfidifaciens* JCM 14089
Nti-1-1	Archaea Euryarchaeota Halobacteria Halobacteriales Halobacteriaceae *Natronorubrum tibetense* GA33
Ssp-1-1	Archaea Euryarchaeota Halobacteria Halobacteriales Halobacteriaceae *Salinarchaeum sp.* Harcht-Bsk1
Hag-1-1	Archaea Euryarchaeota Halobacteria Halobacteriales Halococcaceae *Halococcus agarilyticus*
Hse-1-1	Archaea Euryarchaeota Halobacteria Halobacteriales Halococcaceae *Halococcus sediminicola*
Hru-1-1	Archaea Euryarchaeota Halobacteria Haloferacales Haloferacaceae *Halobellus rufus*
Hal-2-1	Archaea Euryarchaeota Halobacteria Haloferacales Haloferacaceae *Haloferax alexandrinus*
Hgi-2-1	Archaea Euryarchaeota Halobacteria Haloferacales Haloferacaceae *Haloferax gibbonsii*
Hme-1-1 to Hme-1-2	Archaea Euryarchaeota Halobacteria Haloferacales Haloferacaceae *Haloferax mediterranei* ATCC 33500
Hsp-9-1	Archaea Euryarchaeota Halobacteria Haloferacales Haloferacaceae *Haloferax sp.* ATB1
Hna-1-1	Archaea Euryarchaeota Halobacteria Haloferacales Haloferacaceae *Haloplanus natans* DSM 17983
Hru-2-1	Archaea Euryarchaeota Halobacteria Haloferacales Halorubraceae *Halolamina rubra*
Hez-1-1	Archaea Euryarchaeota Halobacteria Haloferacales Halorubraceae *Halorubrum ezzemoulense* DSM 17463
Hha-2-1	Archaea Euryarchaeota Halobacteria Haloferacales Halorubraceae *Halorubrum halophilum*
Hsa-9-1	Archaea Euryarchaeota Halobacteria Haloferacales Halorubraceae *Halorubrum saccharovorum*
Hsp-10-1	Archaea Euryarchaeota Halobacteria Haloferacales Halorubraceae *Halorubrum sp.* BV1
Hdj-1-1	Archaea Euryarchaeota Halobacteria Natrialbales Natrialbaceae *Halopiger djelfimassiliensis*
Hgo-1-1	Archaea Euryarchaeota Halobacteria Natrialbales Natrialbaceae *Halopiger goleimassiliensis*
Hsa-10-1	Archaea Euryarchaeota Halobacteria Natrialbales Natrialbaceae *Halopiger salifodinae*
Hla-4-1	Archaea Euryarchaeota Halobacteria Natrialbales Natrialbaceae *Halostagnicola larsenii* XH-48
Hsp-11-1	Archaea Euryarchaeota Halobacteria Natrialbales Natrialbaceae *Halostagnicola sp.* A56
Hje-2-1	Archaea Euryarchaeota Halobacteria Natrialbales Natrialbaceae *Haloterrigena jeotgali* A29
Nal-2-1	Archaea Euryarchaeota Halobacteria Natrialbales Natrialbaceae *Natrinema altunense*
Nsp-2-1	Archaea Euryarchaeota Halobacteria Natrialbales Natrialbaceae *Natrinema sp.* J7-1
Mar-1-1	Archaea Euryarchaeota Methanobacteria Methanobacteriales Methanobacteriaceae *Methanobacterium arcticum*
Mfo-1-1	Archaea Euryarchaeota Methanobacteria Methanobacteriales Methanobacteriaceae *Methanobacterium formicicum*
Mfo-2-1	Archaea Euryarchaeota Methanobacteria Methanobacteriales Methanobacteriaceae *Methanobacterium formicicum* DSM 3637
Msp-1-1	Archaea Euryarchaeota Methanobacteria Methanobacteriales Methanobacteriaceae *Methanobacterium sp.* AL-21
Msp-2-1	Archaea Euryarchaeota Methanobacteria Methanobacteriales Methanobacteriaceae *Methanobacterium sp.* MB1
Msp-3-1	Archaea Euryarchaeota Methanobacteria Methanobacteriales Methanobacteriaceae *Methanobacterium sp.* SMA-27
Msp-4-1	Archaea Euryarchaeota Methanobacteria Methanobacteriales Methanobacteriaceae *Methanobacterium sp.* SWAN-1
Mve-1-1	Archaea Euryarchaeota Methanobacteria Methanobacteriales Methanobacteriaceae *Methanobacterium veterum*

Mor-1-1	Archaea Euryarchaeota Methanobacteria Methanobacteriales Methanobacteriaceae *Methanobrevibacter oralis* JMR01
Msm-1-1	Archaea Euryarchaeota Methanobacteria Methanobacteriales Methanobacteriaceae *Methanobrevibacter smithii*
Msm-2-1	Archaea Euryarchaeota Methanobacteria Methanobacteriales Methanobacteriaceae *Methanobrevibacter smithii* ATCC 35061
Msm-3-1	Archaea Euryarchaeota Methanobacteria Methanobacteriales Methanobacteriaceae *Methanobrevibacter smithii* DSM 2374
Msm-4-1	Archaea Euryarchaeota Methanobacteria Methanobacteriales Methanobacteriaceae *Methanobrevibacter smithii* DSM 2375
Msm-5-1	Archaea Euryarchaeota Methanobacteria Methanobacteriales Methanobacteriaceae *Methanobrevibacter smithii* TS145A
Msm-6-1	Archaea Euryarchaeota Methanobacteria Methanobacteriales Methanobacteriaceae *Methanobrevibacter smithii* TS145B
Msm-7-1	Archaea Euryarchaeota Methanobacteria Methanobacteriales Methanobacteriaceae *Methanobrevibacter smithii* TS146A
Msm-8-1	Archaea Euryarchaeota Methanobacteria Methanobacteriales Methanobacteriaceae *Methanobrevibacter smithii* TS146B
Msm-9-1	Archaea Euryarchaeota Methanobacteria Methanobacteriales Methanobacteriaceae *Methanobrevibacter smithii* TS146C
Msm-10-1	Archaea Euryarchaeota Methanobacteria Methanobacteriales Methanobacteriaceae *Methanobrevibacter smithii* TS146E
Msm-11-1	Archaea Euryarchaeota Methanobacteria Methanobacteriales Methanobacteriaceae *Methanobrevibacter smithii* TS147A
Msm-12-1	Archaea Euryarchaeota Methanobacteria Methanobacteriales Methanobacteriaceae *Methanobrevibacter smithii* TS147B
Msm-13-1	Archaea Euryarchaeota Methanobacteria Methanobacteriales Methanobacteriaceae *Methanobrevibacter smithii* TS147C
Msm-14-1	Archaea Euryarchaeota Methanobacteria Methanobacteriales Methanobacteriaceae *Methanobrevibacter smithii* TS94A
Msm-15-1	Archaea Euryarchaeota Methanobacteria Methanobacteriales Methanobacteriaceae *Methanobrevibacter smithii* TS94B
Msm-16-1	Archaea Euryarchaeota Methanobacteria Methanobacteriales Methanobacteriaceae *Methanobrevibacter smithii* TS94C
Msm-17-1	Archaea Euryarchaeota Methanobacteria Methanobacteriales Methanobacteriaceae *Methanobrevibacter smithii* TS95A
Msm-18-1	Archaea Euryarchaeota Methanobacteria Methanobacteriales Methanobacteriaceae *Methanobrevibacter smithii* TS95B
Msm-19-1	Archaea Euryarchaeota Methanobacteria Methanobacteriales Methanobacteriaceae *Methanobrevibacter smithii* TS95C
Msm-20-1	Archaea Euryarchaeota Methanobacteria Methanobacteriales Methanobacteriaceae *Methanobrevibacter smithii* TS95D
Msm-21-1	Archaea Euryarchaeota Methanobacteria Methanobacteriales Methanobacteriaceae *Methanobrevibacter smithii* TS96A
Msm-22-1	Archaea Euryarchaeota Methanobacteria Methanobacteriales Methanobacteriaceae *Methanobrevibacter smithii* TS96B
Msm-23-1	Archaea Euryarchaeota Methanobacteria Methanobacteriales Methanobacteriaceae *Methanobrevibacter smithii* TS96C
Mae-1-1	Archaea Euryarchaeota Methanococci Methanococcales Methanococcaceae *Methanococcus aeolicus* Nankai-3
Mvo-1-1	Archaea Euryarchaeota Methanococci Methanococcales Methanococcaceae *Methanococcus voltae* A3
Mco-1-1	Archaea Euryarchaeota Methanomicrobia Methanocellales Methanocellaceae *Methanocella conradii* HZ254
CMe-1-1	Archaea Euryarchaeota Methanomicrobia Methanomicrobiales Generaincertae sedis *Candidatus* Methanoregula boonei 6A8
Mpa-1-1	Archaea Euryarchaeota Methanomicrobia Methanomicrobiales Generaincertae sedis *Methanosphaerula palustris* E1-9c
Mma-1-1	Archaea Euryarchaeota Methanomicrobia Methanomicrobiales Methanomicrobiaceae *Methanoculleus marisnigri* JR1
Mse-1-1	Archaea Euryarchaeota Methanomicrobia Methanomicrobiales Methanomicrobiaceae *Methanoculleus sediminis*
Msp-5-1	Archaea Euryarchaeota Methanomicrobia Methanomicrobiales Methanomicrobiaceae *Methanoculleus sp.* MH98A
Mli-1-1	Archaea Euryarchaeota Methanomicrobia Methanomicrobiales Methanomicrobiaceae *Methanofollis liminatans* DSM 4140
Mta-1-1	Archaea Euryarchaeota Methanomicrobia Methanomicrobiales Methanoregulaceae *Methanolinea tarda* NOBI-1
Mhu-1-1	Archaea Euryarchaeota Methanomicrobia Methanomicrobiales Methanospirillaceae *Methanospirillum hungatei* JF-1
CMe-2-1	Archaea Euryarchaeota Methanomicrobia Methanosarcinales Candidatus Methanoperedenaceae *Candidatus* Methanoperedens nitroreducens
Mbu-1-1	Archaea Euryarchaeota Methanomicrobia Methanosarcinales Methanosarcinaceae *Methanococcoides burtonii* DSM 6242
Mme-1-1	Archaea Euryarchaeota Methanomicrobia Methanosarcinales Methanosarcinaceae *Methanococcoides methylutens*
Mme-2-1	Archaea Euryarchaeota Methanomicrobia Methanosarcinales Methanosarcinaceae *Methanococcoides methylutens* MM1
Mev-1-1	Archaea Euryarchaeota Methanomicrobia Methanosarcinales Methanosarcinaceae *Methanohalobium evestigatum* Z-7303
Mma-2-1	Archaea Euryarchaeota Methanomicrobia Methanosarcinales Methanosarcinaceae *Methanohalophilus mahii* DSM 5219
Mps-1-1	Archaea Euryarchaeota Methanomicrobia Methanosarcinales Methanosarcinaceae *Methanolobus psychrophilus* R15
Mti-1-1	Archaea Euryarchaeota Methanomicrobia Methanosarcinales Methanosarcinaceae *Methanolobus tindarius* DSM 2278
Mho-1-1	Archaea Euryarchaeota Methanomicrobia Methanosarcinales Methanosarcinaceae *Methanomethylovorans hollandica* DSM 15978
Mzh-1-1	Archaea Euryarchaeota Methanomicrobia Methanosarcinales Methanosarcinaceae *Methanosalsum zhilinae* DSM 4017
Mac-1-1	Archaea Euryarchaeota Methanomicrobia Methanosarcinales Methanosarcinaceae *Methanosarcina acetivorans* C2A
Mba-1-1	Archaea Euryarchaeota Methanomicrobia Methanosarcinales Methanosarcinaceae *Methanosarcina barkeri* 227
Mba-2-1	Archaea Euryarchaeota Methanomicrobia Methanosarcinales Methanosarcinaceae *Methanosarcina barkeri* 3
Mba-3-1	Archaea Euryarchaeota Methanomicrobia Methanosarcinales Methanosarcinaceae *Methanosarcina barkeri* CM1
Mba-4-1	Archaea Euryarchaeota Methanomicrobia Methanosarcinales Methanosarcinaceae *Methanosarcina barkeri* MS
Mba-5-1	Archaea Euryarchaeota Methanomicrobia Methanosarcinales Methanosarcinaceae *Methanosarcina barkeri str.* Fusaro
Mba-6-1	Archaea Euryarchaeota Methanomicrobia Methanosarcinales Methanosarcinaceae *Methanosarcina barkeri str.* Wiesmoor
Mho-2-1	Archaea Euryarchaeota Methanomicrobia Methanosarcinales Methanosarcinaceae *Methanosarcina horonobensis* HB-1 = JCM 15518
Mla-1-1	Archaea Euryarchaeota Methanomicrobia Methanosarcinales Methanosarcinaceae *Methanosarcina lacustris* Z-7289
Mma-3-1 to Mma-3-56	Archaea Euryarchaeota Methanomicrobia Methanosarcinales Methanosarcinaceae *Methanosarcina mazei*
Mma-4-1	Archaea Euryarchaeota Methanomicrobia Methanosarcinales Methanosarcinaceae *Methanosarcina mazei* C16
Mma-5-1	Archaea Euryarchaeota Methanomicrobia Methanosarcinales Methanosarcinaceae *Methanosarcina mazei* Go1
Mma-6-1	Archaea Euryarchaeota Methanomicrobia Methanosarcinales Methanosarcinaceae *Methanosarcina mazei* LYC
Mma-7-1	Archaea Euryarchaeota Methanomicrobia Methanosarcinales Methanosarcinaceae *Methanosarcina mazei* S-6
Mma-8-1	Archaea Euryarchaeota Methanomicrobia Methanosarcinales Methanosarcinaceae *Methanosarcina mazei* SarPi
Mma-9-1	Archaea Euryarchaeota Methanomicrobia Methanosarcinales Methanosarcinaceae *Methanosarcina mazei* WWM610
Msi-1-1	Archaea Euryarchaeota Methanomicrobia Methanosarcinales Methanosarcinaceae *Methanosarcina siciliae* C2J
Msi-2-1	Archaea Euryarchaeota Methanomicrobia Methanosarcinales Methanosarcinaceae *Methanosarcina siciliae* T4/M
Mso-1-1	Archaea Euryarchaeota Methanomicrobia Methanosarcinales Methanosarcinaceae *Methanosarcina soligelidi*
Msp-6-1	Archaea Euryarchaeota Methanomicrobia Methanosarcinales Methanosarcinaceae *Methanosarcina sp.* 1.H.A.2.2

Msp-7-1	Archaea Euryarchaeota Methanomicrobia Methanosarcinales Methanosarcinaceae *Methanosarcina sp.* 1.H.T.1A.1
Msp-8-1	Archaea Euryarchaeota Methanomicrobia Methanosarcinales Methanosarcinaceae *Methanosarcina sp.* 2.H.A.1B.4
Msp-9-1	Archaea Euryarchaeota Methanomicrobia Methanosarcinales Methanosarcinaceae *Methanosarcina sp.* 2.H.T.1A.15
Msp-10-1	Archaea Euryarchaeota Methanomicrobia Methanosarcinales Methanosarcinaceae *Methanosarcina sp.* 2.H.T.1A.3
Msp-11-1	Archaea Euryarchaeota Methanomicrobia Methanosarcinales Methanosarcinaceae *Methanosarcina sp.* 2.H.T.1A.6
Msp-12-1	Archaea Euryarchaeota Methanomicrobia Methanosarcinales Methanosarcinaceae *Methanosarcina sp.* 2.H.T.1A.8
Msp-13-1	Archaea Euryarchaeota Methanomicrobia Methanosarcinales Methanosarcinaceae *Methanosarcina sp.* Kolksee
Msp-14-1	Archaea Euryarchaeota Methanomicrobia Methanosarcinales Methanosarcinaceae *Methanosarcina sp.* MTP4
Msp-15-1	Archaea Euryarchaeota Methanomicrobia Methanosarcinales Methanosarcinaceae *Methanosarcina sp.* WH1
Msp-16-1	Archaea Euryarchaeota Methanomicrobia Methanosarcinales Methanosarcinaceae *Methanosarcina sp.* WWM596
Mth-1-1	Archaea Euryarchaeota Methanomicrobia Methanosarcinales Methanosarcinaceae *Methanosarcina thermophila* TM-1
Mva-1-1	Archaea Euryarchaeota Methanomicrobia Methanosarcinales Methanosarcinaceae *Methanosarcina vacuolata* Z-761
Msh-1-1	Archaea Euryarchaeota Methanomicrobia Methanosarcinales Methermicoccaceae *Methermicoccus shengliensis* DSM 18856
Pfe-1-1	Archaea Euryarchaeota Thermococci Thermococcales Thermococcaceae *Palaeococcus ferrophilus* DSM 13482
Ppa-1-1	Archaea Euryarchaeota Thermococci Thermococcales Thermococcaceae *Palaeococcus pacificus* DY20341
Pab-1-1	Archaea Euryarchaeota Thermococci Thermococcales Thermococcaceae *Pyrococcus abyssi* GE5
Pfu-1-1	Archaea Euryarchaeota Thermococci Thermococcales Thermococcaceae *Pyrococcus furiosus* COM1
Pfu-2-1	Archaea Euryarchaeota Thermococci Thermococcales Thermococcaceae *Pyrococcus furiosus* DSM 3638
Pho-1-1	Archaea Euryarchaeota Thermococci Thermococcales Thermococcaceae *Pyrococcus horikoshii* OT3
Psp-1-1	Archaea Euryarchaeota Thermococci Thermococcales Thermococcaceae *Pyrococcus sp.* NA2
Psp-2-1	Archaea Euryarchaeota Thermococci Thermococcales Thermococcaceae *Pyrococcus sp.* ST04
Pya-1-1	Archaea Euryarchaeota Thermococci Thermococcales Thermococcaceae *Pyrococcus yayanosii* CH1
Tba-1-1	Archaea Euryarchaeota Thermococci Thermococcales Thermococcaceae *Thermococcus barophilus* MP
Teu-1-1	Archaea Euryarchaeota Thermococci Thermococcales Thermococcaceae *Thermococcus eurythermalis*
Tga-1-1	Archaea Euryarchaeota Thermococci Thermococcales Thermococcaceae *Thermococcus gammatolerans* EJ3
Tko-1-1	Archaea Euryarchaeota Thermococci Thermococcales Thermococcaceae *Thermococcus kodakarensis* KOD1
Tli-1-1	Archaea Euryarchaeota Thermococci Thermococcales Thermococcaceae *Thermococcus litoralis* DSM 5473
Tna-1-1	Archaea Euryarchaeota Thermococci Thermococcales Thermococcaceae *Thermococcus nautili*
Ton-1-1	Archaea Euryarchaeota Thermococci Thermococcales Thermococcaceae *Thermococcus onnurineus* NA1
Tpa-1-1	Archaea Euryarchaeota Thermococci Thermococcales Thermococcaceae *Thermococcus paralvinellae*
Tsi-1-1	Archaea Euryarchaeota Thermococci Thermococcales Thermococcaceae *Thermococcus sibiricus* MM 739
Tsp-1-1	Archaea Euryarchaeota Thermococci Thermococcales Thermococcaceae *Thermococcus sp.* 4557
Tsp-2-1	Archaea Euryarchaeota Thermococci Thermococcales Thermococcaceae *Thermococcus sp.* AM4
Tsp-3-1	Archaea Euryarchaeota Thermococci Thermococcales Thermococcaceae *Thermococcus sp.* CL1
Tsp-4-1	Archaea Euryarchaeota Thermococci Thermococcales Thermococcaceae *Thermococcus sp.* PK
Tzi-1-1	Archaea Euryarchaeota Thermococci Thermococcales Thermococcaceae *Thermococcus zilligii* AN1
env-1 to env-713	environmental samples

4.2 Gene contexts

Each eL15-Euryarchaeota RNA (indicated by "RNA→") is listed. For each hit, the downstream genes predicted to reside in a regulated operon are listed. If the nearest downstream gene is encoding in the opposite strand (and therefore presumed to not be a part of a regulated operon), then that gene is still depicted. Some environmental sequences and some RefSeq entries lack gene annotations, and so no genes are available for such sequences. The direction of each gene is indicated with an arrow (→), and each predicted conserved domain in the gene is named. Conserved domains associated with more than one eL15-Euryarchaeota RNA are assigned a color; other domains are gray. Information about these conserved domains is given in Section 4.3. The accession of the sequence containing each eL15-Euryarchaeota RNA is given in the column named "Seq. accession". Accessions beginning with "NC_", "NS_", "NW_" or "NZ_" are contained in RefSeq. Other accession refer to environmental samples. Nucleotide coordinates are given for the 5′ and 3′ boundaries of each eL15-Euryarchaeota RNA. If the 5′ coordinate is greater than the 3′ coordinate, the RNA is present on the reverse-complement strand of the containing genomic DNA sequence. Each hit is denoted by an abbreviation (like "Eco-1-1") that refers to a taxonomy given in Section 4.1.

**abbrev.**	**Seq. accession**	**5′ at**	**3′ at**	**genes**

env-1	JGI24721J44947_10004045	-	13735	13690	**RNA**→ PRK04243 (PRK04243)Ribosomal_L15e (pfam00827)→<br>PRK14555 (PRK14555)RNA_binding (pfam01877)→ RPP1 (COG1603)PRK00912 (PRK00912)→<br>PRK03717 (PRK03717)POP5 (COG1369)→ PRK03996 (PRK03996)proteasome_alpha_archeal (cd03756)→<br>Sdo1 (COG1500)PRK13760 (PRK13760)→ Rrp4 (COG1097)PRK04163 (PRK04163)→<br>RNase_PH_archRRP41 (cd11366)PRK03983 (PRK03983)→<br>RNase_PH_archRRP42 (cd11365)PRK04282 (PRK04282)→ RPL43A (COG1997)rpl37ae (PRK03976)→<br>RPC10 (COG1996)rpoP (PRK00398)→ Brix (smart00879)IMP4 (COG2136)→<br>Pcc1 (COG2892)Pcc1 (pfam09341)→ gimC_beta (TIGR02338)GimC (COG1382)→
env-2	JGI24721J26819_10075606	-	1121	1075	**RNA**→ PRK04243 (PRK04243)Ribosomal_L15e (pfam00827)→<br>PRK14555 (PRK14555)RNA_binding (pfam01877)→ PRK00912 (PRK00912)→
Mma-2-1	NC_014002.1	+	819315	819361	**RNA**→ PRK04243 (PRK04243)Ribosomal_L15e (pfam00827)→<br>PRK14555 (PRK14555)RNA_binding (pfam01877)→ PRK00912 (PRK00912)RNase_P_p30 (pfam01876)→<br>PRK03717 (PRK03717)RNase_P_Rpp14 (pfam01900)→<br>PRK03996 (PRK03996)proteasome_alpha_archeal (cd03756)→ Sdo1 (COG1500)PRK13760 (PRK13760)→<br>Rrp4 (COG1097)PRK04163 (PRK04163)→ RNase_PH_archRRP41 (cd11366)PRK03983 (PRK03983)→<br>RNase_PH_archRRP42 (cd11365)PRK04282 (PRK04282)→ rpl37ae (PRK03976)Ribosomal_L37ae (pfam01780)<br>RPC10 (COG1996)rpoP (PRK00398)→ IMP4 (COG2136)PRK00933 (PRK00933)→<br>Pcc1 (COG2892)Pcc1 (pfam09341)→ hypo→
env-3	APHM01012746.1	-	3591	3541	**RNA**→ PRK04243 (PRK04243)Ribosomal_L15e (pfam00827)→
env-4	LTJ07As871_J07ABscf098854	+	859526	859576	**RNA**→ PRK04243 (PRK04243)Ribosomal_L15e (pfam00827)→
Hha-1-1	NZ_AJRK01000373.1	+	10855	10927	**RNA**→ PRK04243 (PRK04243)Ribosomal_L15e (pfam00827)→<br>PRK04243 (PRK04243)Ribosomal_L15e (pfam00827)→ FrnE (COG2761)Thioredoxin_4 (pfam13462)→<br>FrnE (COG2761)Thioredoxin_4 (pfam13462)→
Hha-1-2	NZ_AOMB01000014.1	+	37016	37088	**RNA**→ PRK04243 (PRK04243)Ribosomal_L15e (pfam00827)→ FrnE (COG2761)Thioredoxin_4 (pfam13462)–
env-5	SS_3KL_010_SOIL_10297861	-	306	257	**RNA**→ ←hypo
env-6	SS_3KL_010_SOIL_10032413	-	1596	1549	**RNA**→ PRK04243 (PRK04243)Ribosomal_L15e (pfam00827)→
env-7	APHM01002430.1	+	679	749	**RNA**→ PRK04243 (PRK04243)Ribosomal_L15e (pfam00827)→<br>PRK10089 (PRK10089)tRNA_bindingDomain (cd02153)→
env-8	LTJ07As871_J07ABscf098776	-	39554	39484	**RNA**→ PRK04243 (PRK04243)Ribosomal_L15e (pfam00827)→<br>PRK10089 (PRK10089)tRNA_bindingDomain (cd02153)→
env-9	GSLNARP_GFPJP1N02HFTYD	-	319	187	**RNA**→ PRK04243 (PRK04243)Ribosomal_L15e (pfam00827)→
env-10	GSLNARP_GFPJP1N02IOKU2	+	246	309	**RNA**→ PRK04243 (PRK04243)Ribosomal_L15e (pfam00827)→
Hsa-6-1	NZ_ALJD01000003.1	-	716758	716671	**RNA**→ PRK04243 (PRK04243)Ribosomal_L15e (pfam00827)→<br>PRK04243 (PRK04243)Ribosomal_L15e (pfam00827)→
h3A-1-1	NZ_KK033114.1	+	399056	399132	**RNA**→ PRK04243 (PRK04243)Ribosomal_L15e (pfam00827)→
env-11	P_1C_Sed_2_UnCty_1134943	+	65	158	**RNA**→ RPL15A (COG1632)PRK04243 (PRK04243)→
env-12	P_1C_Liq_2_UnCty_1030158	-	327	255	**RNA**→ RPL15A (COG1632)PRK04243 (PRK04243)→
env-13	P_A23_Liq_2_Fmt_1019611	-	352	280	**RNA**→ RPL15A (COG1632)PRK04243 (PRK04243)→
env-14	P_1C_Sed_2_UnCty_1034182	+	193	285	**RNA**→ PRK04243 (PRK04243)Ribosomal_L15e (pfam00827)→
env-15	ADL5mRS1u_contig05278	-	96	32	**RNA**→
har-1-1	NC_015954.1	+	2102369	2102433	**RNA**→ PRK04243 (PRK04243)Ribosomal_L15e (pfam00827)→
env-16	ADL20m3uS_contig11659	-	8862	8798	**RNA**→ PRK04243 (PRK04243)Ribosomal_L15e (pfam00827)→
env-17	2140918017-4741	-	11103	11039	**RNA**→ PRK04243 (PRK04243)Ribosomal_L15e (pfam00827)→
env-18	2140918027-00084	-	11554	11490	**RNA**→ PRK04243 (PRK04243)Ribosomal_L15e (pfam00827)→
env-19	P_A23_Liq_1_Fmt_1000005	+	6223	6278	**RNA**→ PRK04243 (PRK04243)Ribosomal_L15e (pfam00827)→<br>ThiF_MoeB_HesA_family (cd00757)adenyl_thiF (TIGR02356)→
env-20	P_A23_Liq_3_Fmt_1000005	+	8005	8060	**RNA**→ PRK04243 (PRK04243)Ribosomal_L15e (pfam00827)→<br>ThiF_MoeB_HesA_family (cd00757)adenyl_thiF (TIGR02356)→
env-21	P_A23_Liq_2_Fmt_1000009	-	43137	43082	**RNA**→ PRK04243 (PRK04243)Ribosomal_L15e (pfam00827)→<br>ThiF_MoeB_HesA_family (cd00757)adenyl_thiF (TIGR02356)→
Hwa-2-1	NC_008212.1	+	526505	526560	**RNA**→ PRK04243 (PRK04243)Ribosomal_L15e (pfam00827)→<br>ThiF_MoeB_HesA_family (cd00757)adenyl_thiF (TIGR02356)→<br>PRK08762 (PRK08762)ThiF_MoeB_HesA_family (cd00757)→
env-22	GSLNARP_contig12467	-	1205	1150	**RNA**→ PRK04243 (PRK04243)Ribosomal_L15e (pfam00827)→<br>PRK08762 (PRK08762)ThiF_MoeB_HesA_family (cd00757)→

Hwa-1-1	NC_017459.1	+	520849	520904	**RNA**→ PRK04243 (PRK04243)Ribosomal_L15e (pfam00827)→
					ThiF_MoeB_HesA_family (cd00757)adenyl_thiF (TIGR02356)→
					PRK08762 (PRK08762)ThiF_MoeB_HesA_family (cd00757)→
env-23	APHM01021410.1	+	37065	37122	**RNA**→ PRK04243 (PRK04243)Ribosomal_L15e (pfam00827)→
					PRK08762 (PRK08762)ThiF_MoeB_HesA_family (cd00757)→
env-24	LTJ07As871_J07ABscf098855	+	941476	941533	**RNA**→ PRK04243 (PRK04243)Ribosomal_L15e (pfam00827)→
env-25	APHM01021802.1	+	241797	241852	**RNA**→ PRK04243 (PRK04243)Ribosomal_L15e (pfam00827)→
					ThiF_MoeB_HesA_family (cd00757)adenyl_thiF (TIGR02356)→
env-26	LTJ07As871_J07ABscf098874	+	856037	856092	**RNA**→ PRK04243 (PRK04243)Ribosomal_L15e (pfam00827)→
					ThiF_MoeB_HesA_family (cd00757)adenyl_thiF (TIGR02356)→
env-27	JXWS01023088.1	-	1497	1442	**RNA**→ PRK04243 (PRK04243)Ribosomal_L15e (pfam00827)→
					ThiF_MoeB_HesA_family (cd00757)adenyl_thiF (TIGR02356)→
env-28	P_A23_Liq_2_Fmt_1049687	+	77	131	**RNA**→ PRK04243 (PRK04243)Ribosomal_L15e (pfam00827)→
env-29	P_A23_Liq_1_Fmt_1040915	-	222	168	**RNA**→ PRK04243 (PRK04243)Ribosomal_L15e (pfam00827)→
env-30	P_A23_Liq_3_Fmt_1034622	+	56	110	**RNA**→ RPL15A (COG1632)PRK04243 (PRK04243)→
env-31	GSLNARP_GFPJP1N02J0WUN	+	62	117	**RNA**→ RPL15A (COG1632)PRK04243 (PRK04243)→
Hru-1-1	NZ_BBJO01000020.1	+	43857	43911	**RNA**→ PRK04243 (PRK04243)Ribosomal_L15e (pfam00827)→
env-32	JXWS01113690.1	+	469	523	**RNA**→ RPL15A (COG1632)PRK04243 (PRK04243)→
env-33	GSLNARP_GGGT6729_b1	+	216	270	**RNA**→ RPL15A (COG1632)PRK04243 (PRK04243)→
Hpa-2-1	NZ_AOIV01000027.1	-	154749	154689	**RNA**→ PRK04243 (PRK04243)Ribosomal_L15e (pfam00827)→
env-34	GSLNAS_contig09763	+	194	284	**RNA**→ RPL15A (COG1632)PRK04243 (PRK04243)→
env-35	SL_5KL_010_BRINE_10000873	+	7980	8030	**RNA**→ PRK04243 (PRK04243)Ribosomal_L15e (pfam00827)→
env-36	SL_6KL_011_BRINE_1000559	+	8034	8084	**RNA**→ PRK04243 (PRK04243)Ribosomal_L15e (pfam00827)→
env-37	SL_4KL_010_BRINE_10001420	-	8559	8509	**RNA**→ PRK04243 (PRK04243)Ribosomal_L15e (pfam00827)→
env-38	SL_7KL_010_BRINE_10000923	-	9479	9429	**RNA**→ PRK04243 (PRK04243)Ribosomal_L15e (pfam00827)→
env-39	GSLNARP_contig11045	+	688	814	**RNA**→ PRK04243 (PRK04243)Ribosomal_L15e (pfam00827)→
env-40	APHM01004060.1	-	1754	1627	**RNA**→ PRK04243 (PRK04243)Ribosomal_L15e (pfam00827)→
env-41	LTJ07As871_J07ABscf087768	+	8333	8460	**RNA**→ PRK04243 (PRK04243)Ribosomal_L15e (pfam00827)→
Hac-1-1	NZ_BATA01000010.1	-	40529	40466	**RNA**→ PRK04243 (PRK04243)Ribosomal_L15e (pfam00827)→
Hac-1-2	NZ_BANO01000201.1	+	3030	3093	**RNA**→ PRK04243 (PRK04243)Ribosomal_L15e (pfam00827)→
env-42	JXWS01007612.1	+	354	427	**RNA**→ PRK04243 (PRK04243)Ribosomal_L15e (pfam00827)→
env-43	P_A23_Liq_1_Fmt_1012496	+	495	568	**RNA**→ PRK04243 (PRK04243)Ribosomal_L15e (pfam00827)→
env-44	P_A23_Liq_3_Fmt_1032483	-	283	210	**RNA**→ RPL15A (COG1632)PRK04243 (PRK04243)→
env-45	P_1C_Liq_3_UnCty_1034822	+	91	164	**RNA**→ RPL15A (COG1632)PRK04243 (PRK04243)→
env-46	P_1C_Liq_1_UnCty_1087024	-	228	155	**RNA**→ PRK04243 (PRK04243)Ribosomal_L15e (pfam00827)→
Hna-1-1	NZ_KE386573.1	+	519442	519497	**RNA**→ PRK04243 (PRK04243)Ribosomal_L15e (pfam00827)→
env-47	P_1C_Sed_2_UnCty_1011141	+	185	240	**RNA**→ PRK04243 (PRK04243)Ribosomal_L15e (pfam00827)→
env-48	GSLSAAI_contig05270	+	171	218	**RNA**→ PRK04243 (PRK04243)Ribosomal_L15e (pfam00827)→
					PRK08762 (PRK08762)ThiF_MoeB_HesA_family (cd00757)→
env-49	GSLSAAI_contig13583	-	272	225	**RNA**→ PRK04243 (PRK04243)Ribosomal_L15e (pfam00827)→
env-50	GSLSAAI_contig05339	-	621	574	**RNA**→ PRK04243 (PRK04243)Ribosomal_L15e (pfam00827)→
env-51	GSLSAAI_contig11105	+	1628	1675	**RNA**→ PRK04243 (PRK04243)Ribosomal_L15e (pfam00827)→
					PRK08762 (PRK08762)ThiF_MoeB_HesA_family (cd00757)→
env-52	GSLSAAI_contig11811	-	21562	21515	**RNA**→ PRK04243 (PRK04243)Ribosomal_L15e (pfam00827)→
					PRK08762 (PRK08762)ThiF_MoeB_HesA_family (cd00757)→ PRK05399 (PRK05399)MutS_I (pfam01624)→
					MUTSd (smart00533)MutS (COG0249)→ MUTSac (smart00534)MutS (COG0249)→
					HATPase_c (smart00387)HATPase_c (cd00075)→ DNA_mis_repair (pfam01119)MutL_C (pfam08676)→
env-53	PR_CR_10_Liq_1_inCR_1003279	-	5806	5759	**RNA**→ PRK04243 (PRK04243)Ribosomal_L15e (pfam00827)→
					PRK08762 (PRK08762)ThiF_MoeB_HesA_family (cd00757)→
					MUTSac (smart00534)MUTSd (smart00533)MutS (COG0249)→ MutL (COG0323)mutL (PRK00095)→
env-54	PR_CR_10_Liq_3_inCR_1104247	-	209	162	**RNA**→ RPL15A (COG1632)PRK04243 (PRK04243)→
env-55	SL_6KL_011_BRINE_1017367	+	1682	1774	**RNA**→ PRK04243 (PRK04243)Ribosomal_L15e (pfam00827)→
env-56	SL_4KL_010_BRINE_10011168	-	1073	981	**RNA**→ PRK04243 (PRK04243)Ribosomal_L15e (pfam00827)→
env-57	SL_6KL_011_BRINE_1001181	-	10407	10313	**RNA**→ PRK04243 (PRK04243)Ribosomal_L15e (pfam00827)→

env-58	SL_5KL_010_SED_10002058	-	10404	10310	**RNA**→ PRK04243 (PRK04243)Ribosomal_L15e (pfam00827)→
env-59	SL_8KL_010_SED_10019608	+	2296	2390	**RNA**→ PRK04243 (PRK04243)Ribosomal_L15e (pfam00827)→
env-60	SL_4KL_010_BRINE_10010798	-	3106	3012	**RNA**→ PRK04243 (PRK04243)Ribosomal_L15e (pfam00827)→
env-61	SL_6KL_011_BRINE_1000334	+	4498	4594	**RNA**→ PRK04243 (PRK04243)Ribosomal_L15e (pfam00827)→
env-62	SL_8KL_010_SED_10044433	-	1782	1686	**RNA**→ PRK04243 (PRK04243)Ribosomal_L15e (pfam00827)→
env-63	SL_6KL_011_BRINE_1254331	+	116	210	**RNA**→
env-64	SL_5KL_010_BRINE_10001910	-	7669	7575	**RNA**→ PRK04243 (PRK04243)Ribosomal_L15e (pfam00827)→
env-65	SL_4KL_010_BRINE_10067600	-	209	106	**RNA**→ RPL15A (COG1632)PRK04243 (PRK04243)→
env-66	SL_6KL_011_BRINE_1036440	-	1105	1005	**RNA**→ PRK04243 (PRK04243)Ribosomal_L15e (pfam00827)→
Hai-1-1	NZ_AOJI01000011.1	+	21092	21169	**RNA**→ PRK04243 (PRK04243)Ribosomal_L15e (pfam00827)→
Hco-1-1	NZ_AOJL01000012.1	+	32123	32204	**RNA**→ PRK04243 (PRK04243)Ribosomal_L15e (pfam00827)→
Hsp-8-1	NZ_JH815329.1	+	137323	137405	**RNA**→ PRK04243 (PRK04243)Ribosomal_L15e (pfam00827)→
Hte-1-1	NZ_AOJD01000051.1	+	33749	33831	**RNA**→ PRK04243 (PRK04243)Ribosomal_L15e (pfam00827)→
Hho-1-1	NZ_AOJO01000019.1	-	62900	62818	**RNA**→ PRK04243 (PRK04243)Ribosomal_L15e (pfam00827)→
env-67	P_1C_Liq_3_UnCty_1013660	-	413	331	**RNA**→ RPL15A (COG1632)PRK04243 (PRK04243)→
env-68	P_1C_Liq_1_UnCty_1013722	-	796	714	**RNA**→ PRK04243 (PRK04243)Ribosomal_L15e (pfam00827)→
env-69	P_1C_Liq_2_UnCty_1006954	-	774	692	**RNA**→ PRK04243 (PRK04243)Ribosomal_L15e (pfam00827)→
Hca-2-1	NZ_AOJK01000041.1	+	102530	102612	**RNA**→ PRK04243 (PRK04243)Ribosomal_L15e (pfam00827)→
env-70	P_A23_Liq_1_Fmt_1003600	-	1435	1353	**RNA**→ PRK04243 (PRK04243)Ribosomal_L15e (pfam00827)→
env-71	P_A23_Liq_3_Fmt_1003343	+	1237	1319	**RNA**→ PRK04243 (PRK04243)Ribosomal_L15e (pfam00827)→
env-72	P_A23_Liq_2_Fmt_1003882	-	1692	1610	**RNA**→ PRK04243 (PRK04243)Ribosomal_L15e (pfam00827)→
Hez-1-1	NZ_ATXS01000005.1	+	87176	87258	**RNA**→ PRK04243 (PRK04243)Ribosomal_L15e (pfam00827)→
Hli-2-1	NZ_AOJF01000031.1	-	113040	112960	**RNA**→ PRK04243 (PRK04243)Ribosomal_L15e (pfam00827)→
Har-2-1	NZ_AOJJ01000054.1	-	111488	111408	**RNA**→ PRK04243 (PRK04243)Ribosomal_L15e (pfam00827)→
Hdi-1-1	NZ_AOJN01000039.1	-	113054	112974	**RNA**→ PRK04243 (PRK04243)Ribosomal_L15e (pfam00827)→
Hte-2-1	NZ_AOIW01000042.1	-	36897	36817	**RNA**→ PRK04243 (PRK04243)Ribosomal_L15e (pfam00827)→
Hdi-2-1	NZ_AOJM01000058.1	-	27117	27037	**RNA**→ PRK04243 (PRK04243)Ribosomal_L15e (pfam00827)→
env-73	P_1C_Liq_1_UnCty_1078077	-	117	36	**RNA**→ hypo→
Hli-1-1	NZ_AOJG01000017.1	-	108989	108896	**RNA**→ PRK04243 (PRK04243)Ribosomal_L15e (pfam00827)→
Hha-2-1	NZ_BBJP01000018.1	+	13726	13819	**RNA**→ PRK04243 (PRK04243)Ribosomal_L15e (pfam00827)→
Hsa-9-1	NZ_JNFH02000023.1	+	21503	21596	**RNA**→ PRK04243 (PRK04243)Ribosomal_L15e (pfam00827)→
env-74	ADL20m3uS_contig07384	+	1833	1926	**RNA**→ PRK04243 (PRK04243)Ribosomal_L15e (pfam00827)→
env-75	2140918017-2486	+	2582	2675	**RNA**→ PRK04243 (PRK04243)Ribosomal_L15e (pfam00827)→
env-76	2140918027-05980	-	1029	936	**RNA**→ PRK04243 (PRK04243)Ribosomal_L15e (pfam00827)→
Hla-3-1	NC_012029.1	+	432908	433001	**RNA**→ PRK04243 (PRK04243)Ribosomal_L15e (pfam00827)→
Hko-1-1	NZ_AOJH01000069.1	+	3726	3819	**RNA**→ PRK04243 (PRK04243)Ribosomal_L15e (pfam00827)→
env-77	GQKUMWE01AOE6H	-	136	43	**RNA**→ hypo→
env-78	GQKUMWE02FUCCT	+	205	298	**RNA**→ hypo→
Hsa-7-1	NZ_AOJE01000060.1	+	111633	111732	**RNA**→ PRK04243 (PRK04243)Ribosomal_L15e (pfam00827)→
env-79	P_1C_Liq_2_UnCty_1069180	-	260	182	**RNA**→ PRK04243 (PRK04243)Ribosomal_L15e (pfam00827)→
env-80	P_A23_Liq_1_Fmt_1002719	-	2388	2327	**RNA**→ PRK04243 (PRK04243)Ribosomal_L15e (pfam00827)→
env-81	P_A23_Liq_3_Fmt_1003378	-	2423	2362	**RNA**→ PRK04243 (PRK04243)Ribosomal_L15e (pfam00827)→
env-82	P_A23_Liq_2_Fmt_1009559	-	879	818	**RNA**→ PRK04243 (PRK04243)Ribosomal_L15e (pfam00827)→
env-83	P_1C_Sed_2_UnCty_1017312	+	398	479	**RNA**→ PRK04243 (PRK04243)Ribosomal_L15e (pfam00827)→
env-84	P_1C_Liq_1_UnCty_1034993	+	194	275	**RNA**→ RPL15A (COG1632)PRK04243 (PRK04243)→
env-85	P_1C_Liq_2_UnCty_1023529	-	401	320	**RNA**→ RPL15A (COG1632)PRK04243 (PRK04243)→
env-86	P_1C_Liq_3_UnCty_1021498	-	346	265	**RNA**→ RPL15A (COG1632)PRK04243 (PRK04243)→
env-87	P_1C_Sed_2_UnCty_1004595	+	1788	1869	**RNA**→ PRK04243 (PRK04243)Ribosomal_L15e (pfam00827)→
env-88	P_1C_Sed_2_UnCty_1006549	+	274	355	**RNA**→ PRK04243 (PRK04243)Ribosomal_L15e (pfam00827)→
env-89	SL_6KL_011_BRINE_1064466	-	575	484	**RNA**→ PRK04243 (PRK04243)Ribosomal_L15e (pfam00827)→
env-90	SL_6KL_011_BRINE_1008323	-	510	419	**RNA**→ RPL15A (COG1632)PRK04243 (PRK04243)→
env-91	SL_6KL_011_BRINE_1063445	+	299	393	**RNA**→ RPL15A (COG1632)PRK04243 (PRK04243)→

env-92	SL_6KL_011_BRINE_1001542	-	7921	7827	**RNA**→ PRK04243 (PRK04243)Ribosomal_L15e (pfam00827)→
env-93	SL_6KL_011_BRINE_1006605	+	4992	5093	**RNA**→ RPL15A (COG1632)PRK04243 (PRK04243)→
env-94	SL_6KL_011_BRINE_1010435	-	890	821	**RNA**→ PRK04243 (PRK04243)Ribosomal_L15e (pfam00827)→
Hmu-1-1	NZ_AOLN01000018.1	+	760151	760214	**RNA**→ PRK04243 (PRK04243)Ribosomal_L15e (pfam00827)→
Hel-1-1	NZ_AOLK01000011.1	-	499306	499243	**RNA**→ PRK04243 (PRK04243)Ribosomal_L15e (pfam00827)→
Hla-2-1	NZ_AOLI01000030.1	-	9206	9143	**RNA**→ PRK04243 (PRK04243)Ribosomal_L15e (pfam00827)→
Hde-1-1	NZ_AOLP01000017.1	+	194706	194766	**RNA**→ PRK04243 (PRK04243)Ribosomal_L15e (pfam00827)→
Hal-2-1	NZ_LK053000.1	-	1797714	1797654	**RNA**→ PRK04243 (PRK04243)Ribosomal_L15e (pfam00827)→
Hpr-1-1	NZ_AOLG01000009.1	-	95328	95268	**RNA**→ PRK04243 (PRK04243)Ribosomal_L15e (pfam00827)→
Hgi-1-1	NZ_AOLJ01000011.1	+	406353	406413	**RNA**→ PRK04243 (PRK04243)Ribosomal_L15e (pfam00827)→
Hgi-2-1	NZ_CP011947.1	-	470623	470563	**RNA**→ PRK04243 (PRK04243)Ribosomal_L15e (pfam00827)→
Hsp-9-1	NZ_JPES01000007.1	-	10173	10113	**RNA**→ PRK04243 (PRK04243)Ribosomal_L15e (pfam00827)→
Hsu-2-1	NZ_AOLM01000018.1	+	49470	49530	**RNA**→ PRK04243 (PRK04243)Ribosomal_L15e (pfam00827)→
Hsp-6-1	NZ_AOLD01000031.1	+	8987	9047	**RNA**→ PRK04243 (PRK04243)Ribosomal_L15e (pfam00827)→
Hsp-4-1	NZ_AOLF01000036.1	-	67323	67263	**RNA**→ PRK04243 (PRK04243)Ribosomal_L15e (pfam00827)→
Hsp-5-1	NZ_AOLE01000023.1	+	9123	9183	**RNA**→ PRK04243 (PRK04243)Ribosomal_L15e (pfam00827)→
Hme-1-1	NZ_AOLO01000007.1	+	387185	387264	**RNA**→ PRK04243 (PRK04243)Ribosomal_L15e (pfam00827)→
Hme-1-2	NC_017941.2	-	494773	494694	**RNA**→ PRK04243 (PRK04243)Ribosomal_L15e (pfam00827)→
Hru-2-1	NZ_BBJN01000010.1	-	27162	27075	**RNA**→ PRK04243 (PRK04243)Ribosomal_L15e (pfam00827)→
env-95	P_A23_Liq_1_Fmt_1000360	+	2899	2970	**RNA**→ PRK04243 (PRK04243)Ribosomal_L15e (pfam00827)→
env-96	P_A23_Liq_3_Fmt_1000054	+	17691	17762	**RNA**→ PRK04243 (PRK04243)Ribosomal_L15e (pfam00827)→
env-97	P_A23_Liq_2_Fmt_1000005	+	59053	59124	**RNA**→ PRK04243 (PRK04243)Ribosomal_L15e (pfam00827)→
env-98	JXWS01130832.1	-	481	414	**RNA**→ RPL15A (COG1632)PRK04243 (PRK04243)→
env-99	APHM01022051.1	+	3638	3699	**RNA**→ PRK04243 (PRK04243)Ribosomal_L15e (pfam00827)→
env-100	LTJ07As871_J07ABscf098878	+	1502510	1502571	**RNA**→ PRK04243 (PRK04243)Ribosomal_L15e (pfam00827)→
CHa-1-1	NZ_ASGZ01000052.1	+	24839	24927	**RNA**→ PRK04243 (PRK04243)Ribosomal_L15e (pfam00827)→ GldE (TIGR03520)DUF21 (pfam01595)→
env-101	SL_4KL_010_BRINE_10493865	-	158	111	**RNA**→ RPL15A (COG1632)PRK04243 (PRK04243)→
env-102	SL_6KL_011_BRINE_1000129	+	15997	16046	**RNA**→ PRK04243 (PRK04243)Ribosomal_L15e (pfam00827)→<br>PRK08762 (PRK08762)ThiF_MoeB_HesA_family (cd00757)→ NikR (COG0864)NikR_C (pfam08753)→
env-103	SL_4KL_010_BRINE_10001303	+	2439	2488	**RNA**→ PRK04243 (PRK04243)Ribosomal_L15e (pfam00827)→<br>PRK08762 (PRK08762)ThiF_MoeB_HesA_family (cd00757)→ NikR (COG0864)NikR_C (pfam08753)→
env-104	SL_4KL_010_BRINE_10001201	+	2902	2951	**RNA**→ PRK04243 (PRK04243)Ribosomal_L15e (pfam00827)→<br>ThiF_MoeB_HesA_family (cd00757)adenyl_thiF (TIGR02356)→ NikR (COG0864)NikR_C (pfam08753)→
env-105	SL_5KL_010_BRINE_10000041	+	10173	10222	**RNA**→ PRK04243 (PRK04243)Ribosomal_L15e (pfam00827)→
env-106	SL_6KL_011_BRINE_1002627	-	6475	6426	**RNA**→ PRK04243 (PRK04243)Ribosomal_L15e (pfam00827)→
env-107	SL_5KL_010_SED_10011446	+	130	179	**RNA**→ PRK04243 (PRK04243)Ribosomal_L15e (pfam00827)→
env-108	SL_8KL_010_SED_10489198	-	248	199	**RNA**→ PRK04243 (PRK04243)Ribosomal_L15e (pfam00827)→
env-109	SL_4KL_010_BRINE_10000898	+	3047	3096	**RNA**→ PRK04243 (PRK04243)Ribosomal_L15e (pfam00827)→
env-110	SL_6KL_011_BRINE_1001896	-	92	43	**RNA**→ ←hypo
env-111	SL_4KL_010_BRINE_10030276	+	2918	2967	**RNA**→ hypo→
env-112	P_1C_Liq_3_UnCty_1038130	-	250	201	**RNA**→ PRK04243 (PRK04243)Ribosomal_L15e (pfam00827)→
env-113	P_1C_Liq_2_UnCty_1042794	+	154	203	**RNA**→ PRK04243 (PRK04243)Ribosomal_L15e (pfam00827)→
env-114	P_1C_Liq_1_UnCty_1078870	+	144	193	**RNA**→ PRK04243 (PRK04243)Ribosomal_L15e (pfam00827)→
env-115	P_A23_Liq_3_Fmt_1006374	-	68	19	**RNA**→
env-116	JXWS01040251.1	+	738	787	**RNA**→
env-117	P_A23_Liq_3_Fmt_1005856	+	1663	1712	**RNA**→
env-118	P_A23_Liq_2_Fmt_1003095	-	2041	1992	**RNA**→ PRK04243 (PRK04243)Ribosomal_L15e (pfam00827)→
env-119	APHM01020319.1	-	3958	3909	**RNA**→ PRK04243 (PRK04243)Ribosomal_L15e (pfam00827)→
env-120	LTJ07As871_J07ABscf098871	+	227603	227652	**RNA**→ PRK04243 (PRK04243)Ribosomal_L15e (pfam00827)→
env-121	P_A23_Liq_1_Fmt_1002579	-	586	537	**RNA**→ PRK04243 (PRK04243)Ribosomal_L15e (pfam00827)→
env-122	P_A23_Liq_1_Fmt_1006552	-	756	707	**RNA**→ PRK04243 (PRK04243)Ribosomal_L15e (pfam00827)→
env-123	P_A23_Liq_2_Fmt_1001062	+	420	469	**RNA**→ PRK04243 (PRK04243)Ribosomal_L15e (pfam00827)→
env-124	GSLNARP_contig28426	+	697	746	**RNA**→ PRK04243 (PRK04243)Ribosomal_L15e (pfam00827)→

env-125	P_A23_Liq_3_Fmt_1000673	-	2025	1976	**RNA**→ PRK04243 (PRK04243)Ribosomal_L15e (pfam00827)→
env-126	ADL20m3uS_contig11627	+	9912	9962	**RNA**→ PRK04243 (PRK04243)Ribosomal_L15e (pfam00827)→
env-127	ADL5mRS1u_contig00042	-	12742	12692	**RNA**→ PRK04243 (PRK04243)Ribosomal_L15e (pfam00827)→
env-128	2140918027-00046	-	29012	28962	**RNA**→ PRK04243 (PRK04243)Ribosomal_L15e (pfam00827)→
env-129	P_1C_Sed_2_UnCty_1108461	+	263	312	**RNA**→ PRK04243 (PRK04243)Ribosomal_L15e (pfam00827)→
env-130	ADL20m3uS_contig09914	+	12790	12839	**RNA**→ PRK04243 (PRK04243)Ribosomal_L15e (pfam00827)→
env-131	ADL5mRS1u_contig00058	-	5253	5204	**RNA**→ PRK04243 (PRK04243)Ribosomal_L15e (pfam00827)→
env-132	2140918017-271	-	51697	51648	**RNA**→ PRK04243 (PRK04243)Ribosomal_L15e (pfam00827)→
env-133	2140918017-8071	-	5288	5239	**RNA**→ PRK04243 (PRK04243)Ribosomal_L15e (pfam00827)→
env-134	2140918027-00195	-	5210	5161	**RNA**→ PRK04243 (PRK04243)Ribosomal_L15e (pfam00827)→
env-135	SL_4KL_010_BRINE_10074326	-	160	113	**RNA**→ PRK04243 (PRK04243)Ribosomal_L15e (pfam00827)→
env-136	SL_6KL_011_BRINE_1020994	-	164	117	**RNA**→ PRK04243 (PRK04243)Ribosomal_L15e (pfam00827)→
env-137	SL_6KL_011_BRINE_1123850	+	243	291	**RNA**→ PRK04243 (PRK04243)Ribosomal_L15e (pfam00827)→
env-138	SL_8KL_010_SED_10020664	-	519	471	**RNA**→ PRK04243 (PRK04243)Ribosomal_L15e (pfam00827)→
env-139	SL_8KL_010_SED_10132433	-	343	294	**RNA**→ RPL15A (COG1632)PRK04243 (PRK04243)→
env-140	SL_5KL_010_SED_10120051	+	387	435	**RNA**→
env-141	SL_5KL_010_BRINE_10075440	-	64	16	**RNA**→ hypo→
env-142	SL_5KL_010_SED_10001678	-	13040	12992	**RNA**→ PRK04243 (PRK04243)Ribosomal_L15e (pfam00827)→<br>MCP_signal (cd11386)MA (smart00283)PRK10935 (PRK10935)HAMP (pfam00672)→<br>ThiF_MoeB_HesA_family (cd00757)adenyl_thiF (TIGR02356)→ PRK04460 (PRK04460)ParD (COG3609)→<br>TIGR00697 (TIGR00697)Vut_1 (pfam02592)→
env-143	SL_7KL_010_BRINE_10383585	+	15	63	**RNA**→ RPL15A (COG1632)PRK04243 (PRK04243)→
env-144	SL_5KL_010_SED_10002997	-	7066	7018	**RNA**→ PRK04243 (PRK04243)Ribosomal_L15e (pfam00827)→<br>MA (smart00283)Tar (COG0840)PRK10935 (PRK10935)HAMP (pfam00672)→<br>ThiF_MoeB_HesA_family (cd00757)adenyl_thiF (TIGR02356)→ PRK04460 (PRK04460)ParD (COG3609)→<br>TIGR00697 (TIGR00697)Vut_1 (pfam02592)→ YhaK (COG1741)→
env-145	SL_5KL_010_BRINE_10088461	+	207	254	**RNA**→ PRK04243 (PRK04243)Ribosomal_L15e (pfam00827)→
env-146	SL_6KL_011_BRINE_1001921	-	5758	5709	**RNA**→ PRK04243 (PRK04243)Ribosomal_L15e (pfam00827)→<br>MA (smart00283)Tar (COG0840)HAMP (pfam00672)→<br>ThiF_MoeB_HesA_family (cd00757)adenyl_thiF (TIGR02356)→ PRK04460 (PRK04460)ParD (COG3609)→<br>TIGR00697 (TIGR00697)Vut_1 (pfam02592)→ YhaK (COG1741)→
env-147	SL_6KL_011_BRINE_1002375	-	67	20	**RNA**→
env-148	SL_4KL_010_BRINE_10002475	+	10303	10350	**RNA**→ RPL15A (COG1632)PRK04243 (PRK04243)→
env-149	SL_5KL_010_BRINE_10000147	-	15060	15012	**RNA**→ PRK04243 (PRK04243)Ribosomal_L15e (pfam00827)→<br>MA (smart00283)Tar (COG0840)HAMP (pfam00672)→<br>ThiF_MoeB_HesA_family (cd00757)adenyl_thiF (TIGR02356)→ PRK04460 (PRK04460)ParD (COG3609)→<br>TIGR00697 (TIGR00697)Vut_1 (pfam02592)→
env-150	SL_6KL_011_BRINE_1000125	+	13899	13947	**RNA**→ PRK04243 (PRK04243)Ribosomal_L15e (pfam00827)→<br>MA (smart00283)Tar (COG0840)HAMP (pfam00672)→<br>ThiF_MoeB_HesA_family (cd00757)adenyl_thiF (TIGR02356)→ PRK04460 (PRK04460)ParD (COG3609)→<br>TIGR00697 (TIGR00697)Vut_1 (pfam02592)→
env-151	SL_4KL_010_BRINE_10000557	+	10772	10820	**RNA**→ PRK04243 (PRK04243)Ribosomal_L15e (pfam00827)→<br>MA (smart00283)Tar (COG0840)HAMP (pfam00672)→<br>ThiF_MoeB_HesA_family (cd00757)adenyl_thiF (TIGR02356)→ PRK04460 (PRK04460)ParD (COG3609)→<br>TIGR00697 (TIGR00697)Vut_1 (pfam02592)→
env-152	SL_4KL_010_BRINE_10273491	-	277	229	**RNA**→ ←hypo
env-153	P_A23_Liq_1_Fmt_1052201	-	109	60	**RNA**→ hypo→
env-154	P_A23_Liq_3_Fmt_1050467	+	211	260	**RNA**→ hypo→
env-155	P_A23_Liq_2_Fmt_1033762	+	198	247	**RNA**→ PRK04243 (PRK04243)Ribosomal_L15e (pfam00827)→
env-156	APHM01021601.1	-	2022	1973	**RNA**→ PRK04243 (PRK04243)Ribosomal_L15e (pfam00827)→
env-157	LTJ07As871_J07ABscf098869	-	39692	39643	**RNA**→ PRK04243 (PRK04243)Ribosomal_L15e (pfam00827)→
env-158	JXWU01132649.1	-	361	312	**RNA**→ RPL15A (COG1632)PRK04243 (PRK04243)→
env-159	SL_4KL_010_BRINE_10652442	-	116	67	**RNA**→
Hsp-10-1	NZ_KN050825.1	+	204974	205055	**RNA**→ PRK04243 (PRK04243)Ribosomal_L15e (pfam00827)→

Hse-1-1	NZ_BBMP01000007.1	-	359177	359090	**RNA**→ PRK04243 (PRK04243)Ribosomal_L15e (pfam00827)→ DsbA_FrnE (cd03024)Thioredoxin_4 (pfam13462)→
Hmo-1-1	NZ_AOMC01000142.1	-	76777	76718	**RNA**→ PRK04243 (PRK04243)Ribosomal_L15e (pfam00827)→ DsbA_FrnE (cd03024)Thioredoxin_4 (pfam13462)→
Hth-1-1	NZ_AOMF01000174.1	-	199637	199578	**RNA**→ PRK04243 (PRK04243)Ribosomal_L15e (pfam00827)→
Nae-1-1	NZ_AOIP01000031.1	+	430123	430196	**RNA**→ PRK04243 (PRK04243)Ribosomal_L15e (pfam00827)→
Nta-1-1	NZ_AOIL01000013.1	+	203216	203289	**RNA**→ PRK04243 (PRK04243)Ribosomal_L15e (pfam00827)→
Nas-1-1	NZ_AOIO01000020.1	-	6198	6125	**RNA**→ PRK04243 (PRK04243)Ribosomal_L15e (pfam00827)→
env-160	GSLNAS_F7I09SK02JROTM	-	174	92	**RNA**→ ←hypo
env-161	GSLNARP_GFPJP1N02H548U	+	202	278	**RNA**→ RPL15A (COG1632)PRK04243 (PRK04243)→
env-162	SL_8KL_010_SED_10010026	+	6182	6238	**RNA**→ RPL15A (COG1632)PRK04243 (PRK04243)→
env-163	SL_4KL_010_BRINE_10000926	+	6176	6232	**RNA**→ PRK04243 (PRK04243)Ribosomal_L15e (pfam00827)→
env-164	SL_5KL_010_BRINE_10000029	+	1081	1137	**RNA**→ PRK04243 (PRK04243)Ribosomal_L15e (pfam00827)→
env-165	SL_6KL_011_BRINE_1183487	+	36	92	**RNA**→ RPL15A (COG1632)PRK04243 (PRK04243)→
env-166	SL_5KL_010_SED_10001187	-	8518	8462	**RNA**→ PRK04243 (PRK04243)Ribosomal_L15e (pfam00827)→
env-167	SL_4KL_010_BRINE_10007163	+	5927	5983	**RNA**→ PRK04243 (PRK04243)Ribosomal_L15e (pfam00827)→
env-168	SL_4KL_010_BRINE_10050687	-	1683	1627	**RNA**→ PRK04243 (PRK04243)Ribosomal_L15e (pfam00827)→
env-169	SL_8KL_010_SED_10059739	-	1479	1423	**RNA**→ PRK04243 (PRK04243)Ribosomal_L15e (pfam00827)→
env-170	SL_5KL_010_BRINE_10000179	+	3935	3995	**RNA**→ PRK04243 (PRK04243)Ribosomal_L15e (pfam00827)→
env-171	SL_6KL_011_BRINE_1206958	-	208	148	**RNA**→ RPL15A (COG1632)PRK04243 (PRK04243)→
Hla-4-1	NZ_CP007055.1	-	515819	515738	**RNA**→ PRK04243 (PRK04243)Ribosomal_L15e (pfam00827)→
Hsp-11-1	NZ_JMIP02000020.1	+	43217	43284	**RNA**→ PRK04243 (PRK04243)Ribosomal_L15e (pfam00827)→
Hsp-3-1	NC_002607.1	-	153435	153378	**RNA**→ PRK04243 (PRK04243)Ribosomal_L15e (pfam00827)→
Hsa-3-1	NC_010364.1	-	153597	153540	**RNA**→ PRK04243 (PRK04243)Ribosomal_L15e (pfam00827)→
env-172	SL_4KL_010_BRINE_10049878	+	1656	1711	**RNA**→ RPL15A (COG1632)PRK04243 (PRK04243)→
Hsa-1-1	NZ_BBMO01000001.1	-	1407520	1407456	**RNA**→ PRK04243 (PRK04243)Ribosomal_L15e (pfam00827)→
env-173	ADL20m3uS_GLR497S01AM9VW	-	353	303	**RNA**→ RPL15A (COG1632)PRK04243 (PRK04243)→
env-174	2140918017-22757	-	213	163	**RNA**→ PRK04243 (PRK04243)Ribosomal_L15e (pfam00827)→
env-175	GSLNAS_F7I09SK02IM9H7	+	19	70	**RNA**→ PRK04243 (PRK04243)Ribosomal_L15e (pfam00827)→
env-176	P_1C_Sed_2_UnCty_1039923	-	750	700	**RNA**→ PRK04243 (PRK04243)Ribosomal_L15e (pfam00827)→
Hpa-1-1	NZ_AEMG01000025.1	-	56166	56103	**RNA**→ PRK04243 (PRK04243)Ribosomal_L15e (pfam00827)→ PRK04243 (PRK04243)Ribosomal_L15e (pfam00827)→ DUF1211 (pfam06736)→ DUF1211 (pfam06736)→
Hpa-1-2	NZ_AQXI01000001.1	-	2637650	2637587	**RNA**→ PRK04243 (PRK04243)Ribosomal_L15e (pfam00827)→ DUF1211 (pfam06736)→
env-177	SL_4KL_010_BRINE_10364003	-	267	151	**RNA**→ RPL15A (COG1632)PRK04243 (PRK04243)→
env-178	SL_4KL_010_BRINE_10015962	-	1055	974	**RNA**→ PRK04243 (PRK04243)Ribosomal_L15e (pfam00827)→
env-179	SL_4KL_010_BRINE_10475926	+	13	62	**RNA**→ RPL15A (COG1632)PRK04243 (PRK04243)→
env-180	SL_4KL_010_BRINE_10013350	-	4649	4598	**RNA**→ PRK04243 (PRK04243)Ribosomal_L15e (pfam00827)→
env-181	SL_4KL_010_BRINE_10006798	-	2037	1873	**RNA**→ PRK04243 (PRK04243)Ribosomal_L15e (pfam00827)→
env-182	SL_4KL_010_BRINE_10145615	+	98	148	**RNA**→ PRK04243 (PRK04243)Ribosomal_L15e (pfam00827)→
Ssp-1-1	NC_021313.1	+	780000	780118	**RNA**→ PRK04243 (PRK04243)Ribosomal_L15e (pfam00827)→
env-183	JXWU01070236.1	-	388	249	**RNA**→ PRK04243 (PRK04243)Ribosomal_L15e (pfam00827)→
Nph-1-1	NC_007426.1	-	125427	125378	**RNA**→ PRK04243 (PRK04243)Ribosomal_L15e (pfam00827)→
env-184	SL_4KL_010_BRINE_10509292	+	104	196	**RNA**→ RPL15A (COG1632)PRK04243 (PRK04243)→
Hgo-1-1	NZ_HG315690.1	-	2398586	2398507	**RNA**→ PRK04243 (PRK04243)Ribosomal_L15e (pfam00827)→ PRK15005 (PRK15005)USP_Like (cd00293)→ ars_reduc_gluta (TIGR02689)LMWPc (pfam01451)→
Hxa-1-1	NC_015666.1	+	1583029	1583107	**RNA**→ PRK04243 (PRK04243)Ribosomal_L15e (pfam00827)→
env-185	SL_4KL_010_BRINE_10004067	+	1089	1146	**RNA**→ PRK04243 (PRK04243)Ribosomal_L15e (pfam00827)→ PRK15005 (PRK15005)USP_Like (cd00293)→
env-186	SL_5KL_010_BRINE_10021750	+	302	359	**RNA**→ PRK04243 (PRK04243)Ribosomal_L15e (pfam00827)→ PRK15005 (PRK15005)USP_Like (cd00293)→
env-187	SL_8KL_010_SED_10621895	-	164	104	**RNA**→ RPL15A (COG1632)PRK04243 (PRK04243)→
Nin-1-1	NZ_AOHZ01000075.1	-	121628	121567	**RNA**→ PRK04243 (PRK04243)Ribosomal_L15e (pfam00827)→
env-188	SL_5KL_010_BRINE_10005738	-	1188	1131	**RNA**→ PRK04243 (PRK04243)Ribosomal_L15e (pfam00827)→
env-189	SL_6KL_011_BRINE_1053602	+	271	328	**RNA**→ PRK04243 (PRK04243)Ribosomal_L15e (pfam00827)→
env-190	SL_8KL_010_SED_10028257	-	828	771	**RNA**→ PRK04243 (PRK04243)Ribosomal_L15e (pfam00827)→

env-191	SL_4KL_010_BRINE_10066144	-	810	753	**RNA**→ PRK04243 (PRK04243)Ribosomal_L15e (pfam00827)→
env-192	SS_3KL_010_SOIL_10028172	-	1924	1854	**RNA**→ ←hypo
env-193	SL_3KL_010_SED_10125154	+	137	207	**RNA**→ PRK04243 (PRK04243)Ribosomal_L15e (pfam00827)→
Nje-1-1	NZ_AOIA01000114.1	+	146675	146744	**RNA**→ PRK04243 (PRK04243)Ribosomal_L15e (pfam00827)→
Nam-1-1	NZ_AOIB01000026.1	+	119391	119460	**RNA**→ PRK04243 (PRK04243)Ribosomal_L15e (pfam00827)→
Noc-1-1	NC_019974.1	-	1797311	1797242	**RNA**→ PRK04243 (PRK04243)Ribosomal_L15e (pfam00827)→
env-194	GSLNARP_GFPJP1N02GQ822	+	449	495	**RNA**→
env-195	SL_4KL_010_BRINE_10350018	-	232	148	**RNA**→ RPL15A (COG1632)PRK04243 (PRK04243)→
env-196	SL_4KL_010_BRINE_10097165	-	841	785	**RNA**→ PRK04243 (PRK04243)Ribosomal_L15e (pfam00827)→
env-197	SL_5KL_010_BRINE_10001159	-	6520	6463	**RNA**→ PRK04243 (PRK04243)Ribosomal_L15e (pfam00827)→
env-198	SL_5KL_010_SED_10028357	+	365	422	**RNA**→ PRK04243 (PRK04243)Ribosomal_L15e (pfam00827)→
env-199	SL_6KL_011_BRINE_1084724	-	464	407	**RNA**→ RPL15A (COG1632)PRK04243 (PRK04243)→
env-200	SL_8KL_010_SED_10260913	+	164	221	**RNA**→ RPL15A (COG1632)PRK04243 (PRK04243)→
env-201	SL_4KL_010_BRINE_10003303	+	1476	1533	**RNA**→ PRK04243 (PRK04243)Ribosomal_L15e (pfam00827)→
env-202	GSLNAS_F7I09SK02F8ECV	-	271	209	**RNA**→ RPL15A (COG1632)PRK04243 (PRK04243)→
Hla-1-1	NZ_AGFZ01000059.1	-	22715	22654	**RNA**→ hypo→ hypo→ PRK04243 (PRK04243)Ribosomal_L15e (pfam00827)→ PRK04243 (PRK04243)Ribosomal_L15e (pfam00827)→
Hla-1-2	NZ_AOLZ01000073.1	-	22804	22743	**RNA**→ hypo→ PRK04243 (PRK04243)Ribosomal_L15e (pfam00827)→
Hva-1-1	NZ_AOLQ01000004.1	-	73870	73819	**RNA**→ PRK04243 (PRK04243)Ribosomal_L15e (pfam00827)→
Hsi-1-1	NZ_AOLR01000005.1	+	181787	181840	**RNA**→ PRK04243 (PRK04243)Ribosomal_L15e (pfam00827)→
Hma-1-1	NC_006396.1	+	1849657	1849710	**RNA**→ PRK04243 (PRK04243)Ribosomal_L15e (pfam00827)→
Ham-1-1	NZ_AOLW01000010.1	-	93451	93398	**RNA**→ PRK04243 (PRK04243)Ribosomal_L15e (pfam00827)→
env-203	P_A23_Liq_1_Fmt_1025305	+	182	235	**RNA**→ PRK04243 (PRK04243)Ribosomal_L15e (pfam00827)→
env-204	P_A23_Liq_3_Fmt_1015036	-	204	151	**RNA**→ PRK04243 (PRK04243)Ribosomal_L15e (pfam00827)→
env-205	P_A23_Liq_2_Fmt_1015236	+	488	541	**RNA**→ PRK04243 (PRK04243)Ribosomal_L15e (pfam00827)→
Hca-1-1	NZ_AOLS01000038.1	-	57729	57676	**RNA**→ PRK04243 (PRK04243)Ribosomal_L15e (pfam00827)→
Hsa-2-1	NZ_JXAO01000004.1	+	115750	115803	**RNA**→ hypo→
Hhi-2-1	NC_023013.1	+	2490914	2490967	**RNA**→ PRK04243 (PRK04243)Ribosomal_L15e (pfam00827)→
Hhi-1-1	NC_015948.1	+	2479481	2479534	**RNA**→ PRK04243 (PRK04243)Ribosomal_L15e (pfam00827)→
Hsp-1-1	NZ_CP010529.1	+	1092738	1092791	**RNA**→ PRK04243 (PRK04243)Ribosomal_L15e (pfam00827)→
Hja-1-1	NZ_AOLY01000007.1	-	129303	129250	**RNA**→ PRK04243 (PRK04243)Ribosomal_L15e (pfam00827)→
Har-1-1	NZ_AOLX01000025.1	-	30531	30480	**RNA**→ PRK04243 (PRK04243)Ribosomal_L15e (pfam00827)→
env-206	GSLNAS_F7I09SK02I6FWL	+	298	351	**RNA**→ PRK04243 (PRK04243)Ribosomal_L15e (pfam00827)→
Hmu-2-1	NC_013202.1	-	272589	272518	**RNA**→ PRK04243 (PRK04243)Ribosomal_L15e (pfam00827)→
Hka-1-1	NZ_KB905378.1	+	614167	614238	**RNA**→ PRK04243 (PRK04243)Ribosomal_L15e (pfam00827)→
env-207	SL_6KL_011_BRINE_1037538	-	695	635	**RNA**→ ←hypo
env-208	SL_6KL_011_BRINE_1012586	-	1128	1038	**RNA**→ PRK04243 (PRK04243)Ribosomal_L15e (pfam00827)→
env-209	SL_4KL_010_BRINE_10018197	+	2238	2328	**RNA**→ PRK04243 (PRK04243)Ribosomal_L15e (pfam00827)→
env-210	SL_6KL_011_BRINE_1090145	+	342	402	**RNA**→ PRK04243 (PRK04243)Ribosomal_L15e (pfam00827)→
env-211	SL_4KL_010_BRINE_10055587	-	1221	1161	**RNA**→ PRK04243 (PRK04243)Ribosomal_L15e (pfam00827)→
env-212	SL_6KL_011_BRINE_1018015	-	1651	1591	**RNA**→ PRK04243 (PRK04243)Ribosomal_L15e (pfam00827)→
env-213	SL_4KL_010_BRINE_10006569	+	5039	5099	**RNA**→ PRK04243 (PRK04243)Ribosomal_L15e (pfam00827)→
env-214	SL_6KL_011_BRINE_1094071	-	230	150	**RNA**→ RPL15A (COG1632)PRK04243 (PRK04243)→
env-215	GSLSAAI_GFP0ZZ301EUXSI	+	466	528	**RNA**→ ←hypo
env-216	SL_4KL_010_BRINE_10104860	+	909	969	**RNA**→ RPL15A (COG1632)PRK04243 (PRK04243)→
env-217	GSLNARP_contig03416	-	112	53	**RNA**→
env-218	GSLNAS_F7I09SK02HZKWC	-	504	445	**RNA**→ RPL15A (COG1632)PRK04243 (PRK04243)→
env-219	GSLNAS_F7I09SK02GL76P	-	421	362	**RNA**→ RPL15A (COG1632)PRK04243 (PRK04243)→
env-220	P_A23_Liq_1_Fmt_1037775	+	87	146	**RNA**→ PRK04243 (PRK04243)Ribosomal_L15e (pfam00827)→
Nmo-1-1	NC_020388.1	+	780497	780546	**RNA**→ PRK04243 (PRK04243)Ribosomal_L15e (pfam00827)→
env-221	APHM01007304.1	+	2549	2595	**RNA**→ PRK04243 (PRK04243)Ribosomal_L15e (pfam00827)→ PRK08671 (PRK08671)MetAP2 (cd01088)→
env-222	LTJ07As871_J07ABscf089578	-	54536	54490	**RNA**→ PRK04243 (PRK04243)Ribosomal_L15e (pfam00827)→

env-223	GSLNARP_contig28880	+	3300	3346	**RNA**→ PRK04243 (PRK04243)Ribosomal_L15e (pfam00827)→ PRK08671 (PRK08671)APP_MetAP (cd0106
env-224	APHM01013436.1	+	115	161	**RNA**→ PRK04243 (PRK04243)Ribosomal_L15e (pfam00827)→
env-225	LTJ07As871_J07ABscf098875	+	1708553	1708599	**RNA**→ PRK04243 (PRK04243)Ribosomal_L15e (pfam00827)→
env-226	SL_4KL_010_BRINE_10204549	+	36	97	**RNA**→ RPL15A (COG1632)PRK04243 (PRK04243)→
env-227	JXWU01037046.1	-	538	484	**RNA**→ RPL15A (COG1632)PRK04243 (PRK04243)→
env-228	SS_3KL_010_SOIL_10003299	+	3588	3637	**RNA**→ PRK04243 (PRK04243)Ribosomal_L15e (pfam00827)→
env-229	SS_3KL_010_SOIL_10003445	+	3378	3426	**RNA**→ PRK04243 (PRK04243)Ribosomal_L15e (pfam00827)→
Hci-1-1	NZ_JDTH01000002.1	-	1455055	1454992	**RNA**→ PRK04243 (PRK04243)Ribosomal_L15e (pfam00827)→
Hut-1-1	NC_013158.1	-	2429098	2429049	**RNA**→ PRK04243 (PRK04243)Ribosomal_L15e (pfam00827)→
env-230	GSLNARP_GFPJP1N02FZ9S5	+	70	153	**RNA**→ PRK04243 (PRK04243)Ribosomal_L15e (pfam00827)→
env-231	SL_8KL_010_SED_10278449	-	167	118	**RNA**→ RPL15A (COG1632)PRK04243 (PRK04243)→
env-232	SL_4KL_010_BRINE_10001615	-	5266	5217	**RNA**→ PRK04243 (PRK04243)Ribosomal_L15e (pfam00827)→
env-233	SL_6KL_011_BRINE_1000077	-	3468	3419	**RNA**→ PRK04243 (PRK04243)Ribosomal_L15e (pfam00827)→ hypo→ hypo→
env-234	SL_6KL_011_BRINE_1009755	-	3271	3210	**RNA**→ PRK04243 (PRK04243)Ribosomal_L15e (pfam00827)→
env-235	SL_8KL_010_SED_10678060	-	174	125	**RNA**→ RPL15A (COG1632)PRK04243 (PRK04243)→
env-236	SL_4KL_010_BRINE_10654803	+	125	172	**RNA**→ RPL15A (COG1632)PRK04243 (PRK04243)→
Nba-1-1	NZ_AOHY01000007.1	+	209062	209133	**RNA**→ PRK04243 (PRK04243)Ribosomal_L15e (pfam00827)→
env-237	SL_3KL_010_SED_11113557	-	168	97	**RNA**→ RPL15A (COG1632)PRK04243 (PRK04243)→
Nsu-1-1	NZ_AOHX01000026.1	+	190456	190532	**RNA**→ PRK04243 (PRK04243)Ribosomal_L15e (pfam00827)→
Npa-1-1	NZ_AOII01000068.1	+	9874	9946	**RNA**→ PRK04243 (PRK04243)Ribosomal_L15e (pfam00827)→
Nal-2-1	NZ_JNCS01000001.1	+	188067	188139	**RNA**→ PRK04243 (PRK04243)Ribosomal_L15e (pfam00827)→
Nal-1-1	NZ_AOIK01000025.1	-	13704	13632	**RNA**→ PRK04243 (PRK04243)Ribosomal_L15e (pfam00827)→
Nga-1-1	NZ_AOIJ01000043.1	+	26494	26566	**RNA**→ PRK04243 (PRK04243)Ribosomal_L15e (pfam00827)→
Nsp-2-1	NZ_AJVG01000013.1	+	23678	23750	**RNA**→ PRK04243 (PRK04243)Ribosomal_L15e (pfam00827)→
Nsp-1-1	NC_018224.1	+	1880458	1880530	**RNA**→ PRK04243 (PRK04243)Ribosomal_L15e (pfam00827)→
Hdj-1-1	NZ_HG315684.1	-	683477	683408	**RNA**→ PRK04243 (PRK04243)Ribosomal_L15e (pfam00827)→
Hli-3-1	NZ_AOIT01000029.1	-	228185	228113	**RNA**→ PRK04243 (PRK04243)Ribosomal_L15e (pfam00827)→
Hje-2-1	NZ_JDTG01000014.1	-	528274	528203	**RNA**→ PRK04243 (PRK04243)Ribosomal_L15e (pfam00827)→
Hth-2-1	NZ_AOIR01000063.1	-	74675	74604	**RNA**→ PRK04243 (PRK04243)Ribosomal_L15e (pfam00827)→
Hsa-10-1	NZ_JROF01000002.1	+	114940	115012	**RNA**→ PRK04243 (PRK04243)Ribosomal_L15e (pfam00827)→
env-238	SL_5KL_010_BRINE_10014771	+	1979	2040	**RNA**→ PRK04243 (PRK04243)Ribosomal_L15e (pfam00827)→
env-239	SL_5KL_010_BRINE_10003441	+	1496	1557	**RNA**→ PRK04243 (PRK04243)Ribosomal_L15e (pfam00827)→
env-240	SL_6KL_011_BRINE_1002462	-	7480	7419	**RNA**→ PRK04243 (PRK04243)Ribosomal_L15e (pfam00827)→
env-241	SL_8KL_010_SED_10108063	-	214	153	**RNA**→ PRK04243 (PRK04243)Ribosomal_L15e (pfam00827)→
env-242	SL_4KL_010_BRINE_10000366	+	1598	1659	**RNA**→ PRK04243 (PRK04243)Ribosomal_L15e (pfam00827)→
Hni-1-1	NZ_AOMA01000119.1	+	3513	3588	**RNA**→ PRK04243 (PRK04243)Ribosomal_L15e (pfam00827)→
Nch-1-1	NZ_AOIN01000080.1	+	5529	5592	**RNA**→ PRK04243 (PRK04243)Ribosomal_L15e (pfam00827)→
Nhu-1-1	NZ_AOIM01000013.1	+	217184	217247	**RNA**→ PRK04243 (PRK04243)Ribosomal_L15e (pfam00827)→
Nve-1-1	NZ_AOID01000026.1	-	208079	208007	**RNA**→ PRK04243 (PRK04243)Ribosomal_L15e (pfam00827)→
Hsa-8-1	NZ_AOIS01000035.1	+	13267	13339	**RNA**→ PRK04243 (PRK04243)Ribosomal_L15e (pfam00827)→
Htu-1-1	NC_013743.1	-	2869593	2869521	**RNA**→ PRK04243 (PRK04243)Ribosomal_L15e (pfam00827)→
env-243	SL_8KL_010_SED_10556862	-	191	117	**RNA**→ RPL15A (COG1632)PRK04243 (PRK04243)→
Hca-3-1	NZ_AOIU01000004.1	+	46196	46248	**RNA**→ PRK04243 (PRK04243)Ribosomal_L15e (pfam00827)→
env-244	JXWS01111537.1	-	180	72	**RNA**→ ←Form_Nir_trans (pfam01226)
env-245	SL_4KL_010_BRINE_10017818	-	3866	3781	**RNA**→ PRK04243 (PRK04243)Ribosomal_L15e (pfam00827)→ PRK15005 (PRK15005)USP_Like (cd00293)→
env-246	GSLNAS_F7I09SK02G2VWW	+	218	297	**RNA**→ PRK04243 (PRK04243)Ribosomal_L15e (pfam00827)→
env-247	SL_5KL_010_BRINE_10003121	+	300	385	**RNA**→ PRK04243 (PRK04243)Ribosomal_L15e (pfam00827)→
env-248	SL_5KL_010_BRINE_10044475	-	707	612	**RNA**→ PRK04243 (PRK04243)Ribosomal_L15e (pfam00827)→
env-249	SL_4KL_010_BRINE_10064659	+	812	918	**RNA**→ RPL15A (COG1632)PRK04243 (PRK04243)→ RPL15A (COG1632)PRK04243 (PRK04243)→
env-250	GSLNARP_GFPJP1N02IRMJO	+	320	372	**RNA**→ PRK04243 (PRK04243)Ribosomal_L15e (pfam00827)→
env-251	APHM01020421.1	+	442	499	**RNA**→ PRK04243 (PRK04243)Ribosomal_L15e (pfam00827)→
env-252	LTJ07As871_J07ABscf098852	-	1211893	1211836	**RNA**→ PRK04243 (PRK04243)Ribosomal_L15e (pfam00827)→

env-253	GSLNARP_GFPJP1N02H78XW	+	40	86	**RNA**→ RPL15A (COG1632)PRK04243 (PRK04243)→
Hsu-1-1	NZ_CP008874.1	-	516965	516913	**RNA**→ PRK04243 (PRK04243)Ribosomal_L15e (pfam00827)→
Hsp-2-1	NZ_CP007060.1	-	692145	692080	**RNA**→ PRK04243 (PRK04243)Ribosomal_L15e (pfam00827)→
Has-1-1	NZ_AOIQ01000019.1	+	72263	72320	**RNA**→ PRK04243 (PRK04243)Ribosomal_L15e (pfam00827)→
env-254	SS_3KL_010_SOIL_10043763	-	169	95	**RNA**→ RPL15A (COG1632)PRK04243 (PRK04243)→
Hag-1-1	NZ_BAFM01000013.1	+	96977	97051	**RNA**→ PRK04243 (PRK04243)Ribosomal_L15e (pfam00827)→ FrnE (COG2761)Thioredoxin_4 (pfam13462)→
Hsa-4-1	NZ_AOMD01000030.1	+	286601	286667	**RNA**→ PRK04243 (PRK04243)Ribosomal_L15e (pfam00827)→ hypo→ hypo→
					FrnE (COG2761)Thioredoxin_4 (pfam13462)→
Hsa-5-1	NZ_AOME01000076.1	+	93813	93880	**RNA**→ PRK04243 (PRK04243)Ribosomal_L15e (pfam00827)→ FrnE (COG2761)Thioredoxin_4 (pfam13462)→
env-255	CERQ01166877.1	-	487	436	**RNA**→ RPL15A (COG1632)PRK04243 (PRK04243)→
env-256	CETH01173410.1	+	12810	12860	**RNA**→ PRK04243 (PRK04243)Ribosomal_L15e (pfam00827)→ PreA (COG1146)Fer4_7 (pfam12838)→
env-257	CETM01021463.1	-	36859	36809	**RNA**→ PRK04243 (PRK04243)Ribosomal_L15e (pfam00827)→ PreA (COG1146)Fer4_7 (pfam12838)→
env-258	CEUQ01031760.1	+	6382	6432	**RNA**→ PRK04243 (PRK04243)Ribosomal_L15e (pfam00827)→ PreA (COG1146)Fer4_7 (pfam12838)→
env-259	CEUS01376465.1	+	263	313	**RNA**→ RPL15A (COG1632)PRK04243 (PRK04243)→
env-260	CEUY01138208.1	-	851	801	**RNA**→ PRK04243 (PRK04243)Ribosomal_L15e (pfam00827)→ hypo→
env-261	CEUZ01171605.1	+	1156	1206	**RNA**→ RPL15A (COG1632)PRK04243 (PRK04243)→
env-262	JCVI_SCAF_1096628214146	-	161	111	**RNA**→
env-263	CENY01171985.1	-	10152	10102	**RNA**→ PRK04243 (PRK04243)Ribosomal_L15e (pfam00827)→ PreA (COG1146)Fer4_13 (pfam13370)→
env-264	CEOQ01117861.1	+	720	770	**RNA**→ RPL15A (COG1632)PRK04243 (PRK04243)→
env-265	CEUL01353856.1	+	7	57	**RNA**→ PRK04243 (PRK04243)Ribosomal_L15e (pfam00827)→ PreA (COG1146)Fer4_7 (pfam12838)→
env-266	CENY01152858.1	+	14934	14985	**RNA**→ PRK04243 (PRK04243)Ribosomal_L15e (pfam00827)→ PreA (COG1146)Fer4_9 (pfam13187)→
Mae-1-1	NC_009635.1	-	1562691	1562646	**RNA**→ PRK04243 (PRK04243)Ribosomal_L15e (pfam00827)→
env-267	PBDCA2_FIDWTPW02QZKOZ	+	178	232	**RNA**→ hypo→ RPL15A (COG1632)PRK04243 (PRK04243)→
env-268	JGI24422J19971_10000787	-	11928	11884	**RNA**→ PRK04243 (PRK04243)Ribosomal_L15e (pfam00827)→
env-269	JGI2172J19969_10313737	-	395	351	**RNA**→ RPL15A (COG1632)PRK04243 (PRK04243)→
env-270	LGVF01069618.1	-	525	473	**RNA**→ PRK04243 (PRK04243)Ribosomal_L15e (pfam00827)→
env-271	LGVE01065530.1	-	193	142	**RNA**→ PRK04243 (PRK04243)Ribosomal_L15e (pfam00827)→
env-272	LGVF01108952.1	+	1406	1457	**RNA**→ PRK04243 (PRK04243)Ribosomal_L15e (pfam00827)→
env-273	LGVF01076430.1	-	3041	2990	**RNA**→ PRK04243 (PRK04243)Ribosomal_L15e (pfam00827)→
env-274	JGI24721J26819_10009006	+	6561	6611	**RNA**→ PRK04243 (PRK04243)Ribosomal_L15e (pfam00827)→ PRK12277 (PRK12277)RPL13 (COG4352)→
					PRK14555 (PRK14555)RNA_binding (pfam01877)→ RPP1 (COG1603)PRK00912 (PRK00912)→
					PRK03717 (PRK03717)RNase_P_Rpp14 (pfam01900)→
env-275	JGI24721J44947_10030901	-	78	28	**RNA**→
env-276	JGI24721J44947_10028419	+	536	586	**RNA**→ PRK04243 (PRK04243)Ribosomal_L15e (pfam00827)→ PRK12277 (PRK12277)RPL13 (COG4352)→
					PRK14555 (PRK14555)RNA_binding (pfam01877)→ RPP1 (COG1603)PRK00912 (PRK00912)→
					PRK03717 (PRK03717)RNase_P_Rpp14 (pfam01900)→
env-277	JGI24721J44947_10022851	+	2001	2047	**RNA**→ PRK04243 (PRK04243)Ribosomal_L15e (pfam00827)→ PRK12277 (PRK12277)RPL13 (COG4352)→
					PRK14555 (PRK14555)RNA_binding (pfam01877)→ hypo→ RPP1 (COG1603)PRK00912 (PRK00912)→
					PRK03717 (PRK03717)RNase_P_Rpp14 (pfam01900)→
env-278	JGI20130J15003_1001487	+	10993	11039	**RNA**→ PRK04243 (PRK04243)Ribosomal_L15e (pfam00827)→ PRK12277 (PRK12277)RPL13 (COG4352)→
					PRK14555 (PRK14555)RNA_binding (pfam01877)→ RPP1 (COG1603)PRK00912 (PRK00912)→
					PRK03717 (PRK03717)RNase_P_Rpp14 (pfam01900)→
env-279	JGI20130J15133_1001487	+	10993	11039	**RNA**→ PRK04243 (PRK04243)Ribosomal_L15e (pfam00827)→ PRK12277 (PRK12277)RPL13 (COG4352)→
					PRK14555 (PRK14555)RNA_binding (pfam01877)→ RPP1 (COG1603)PRK00912 (PRK00912)→
					PRK03717 (PRK03717)RNase_P_Rpp14 (pfam01900)→
env-280	JGI24721J26819_10027382	-	1728	1682	**RNA**→ PRK04243 (PRK04243)Ribosomal_L15e (pfam00827)→ PRK12277 (PRK12277)RPL13 (COG4352)→
					PRK14555 (PRK14555)RNA_binding (pfam01877)→ PRK00912 (PRK00912)RNase_P_p30 (pfam01876)→
env-281	JGI2172J19969_10022280	+	1906	1952	**RNA**→ RPL15A (COG1632)PRK04243 (PRK04243)→
env-282	SMTZ1_10021852	+	5904	5950	**RNA**→ RPL15A (COG1632)PRK04243 (PRK04243)→
env-283	JGI20214J14112_1187723	+	274	322	**RNA**→
env-284	WSSedA2C_c189965	+	176	224	**RNA**→ RPL15A (COG1632)PRK04243 (PRK04243)→
env-285	WSSedA2Ca3_1189965	+	176	224	**RNA**→ RPL15A (COG1632)PRK04243 (PRK04243)→
env-286	WSSedB1B2_c027137	-	242	194	**RNA**→ PRK04243 (PRK04243)Ribosomal_L15e (pfam00827)→
env-287	JGI24721J26819_10037939	+	1348	1398	**RNA**→ PRK04243 (PRK04243)Ribosomal_L15e (pfam00827)→ PRK12277 (PRK12277)RPL13 (COG4352)→

env-288	JGI24721J44947_10000270	-	47013	46963	**RNA**→ PRK04243 (PRK04243)Ribosomal_L15e (pfam00827)→ PRK12277 (PRK12277)RPL13 (COG4352)→
env-289	JGI2160J19893_10105365	-	280	230	**RNA**→ RPL15A (COG1632)PRK04243 (PRK04243)→
env-290	JGI2171J19970_10224488	+	281	331	**RNA**→ RPL15A (COG1632)PRK04243 (PRK04243)→
env-291	JGI2173J19968_10055779	+	876	926	**RNA**→ RPL15A (COG1632)PRK04243 (PRK04243)→
env-292	SMTZ1_10000331	-	33118	33068	**RNA**→ PRK04243 (PRK04243)Ribosomal_L15e (pfam00827)→ PRK12277 (PRK12277)RPL13 (COG4352)→ PRK14555 (PRK14555)RNA_binding (pfam01877)→ RPP1 (COG1603)PRK00912 (PRK00912)→ PRK03717 (PRK03717)RNase_P_Rpp14 (pfam01900)→
env-293	JGI2171J19970_10312620	+	332	382	**RNA**→ RPL15A (COG1632)PRK04243 (PRK04243)→
env-294	SMTZ1_10014562	-	4684	4634	**RNA**→ PRK04243 (PRK04243)Ribosomal_L15e (pfam00827)→ PRK12277 (PRK12277)RPL13 (COG4352)→ PRK14555 (PRK14555)RNA_binding (pfam01877)→ RPP1 (COG1603)PRK00912 (PRK00912)→ PRK03717 (PRK03717)RNase_P_Rpp14 (pfam01900)→
env-295	WSSedA1TJGI_c051973	+	58	106	**RNA**→ RPL15A (COG1632)PRK04243 (PRK04243)→
env-296	WSSedA1Ba3_1047821	-	762	714	**RNA**→ PRK04243 (PRK04243)Ribosomal_L15e (pfam00827)→ hypo→
env-297	JGI20171J14444_1002001	+	1726	1771	**RNA**→ PRK04243 (PRK04243)Ribosomal_L15e (pfam00827)→
env-298	JGI20165J15291_1047466	-	228	183	**RNA**→ PRK04243 (PRK04243)Ribosomal_L15e (pfam00827)→
env-299	JGI20164J15296_1001389	+	654	699	**RNA**→ PRK04243 (PRK04243)Ribosomal_L15e (pfam00827)→
env-300	JGI20163J15578_10000609	-	5418	5373	**RNA**→ PRK04243 (PRK04243)Ribosomal_L15e (pfam00827)→
env-301	JGI20164J15296_1023034	-	338	292	**RNA**→ RPL15A (COG1632)PRK04243 (PRK04243)→
env-302	JGI20164J15296_1001061	+	1474	1520	**RNA**→ PRK04243 (PRK04243)Ribosomal_L15e (pfam00827)→
env-303	JGI20164J15296_1003383	-	130	84	**RNA**→
env-304	WSSedA2C_c120722	-	217	169	**RNA**→ PRK04243 (PRK04243)Ribosomal_L15e (pfam00827)→
env-305	WSSedA1Ba2_1101229	-	82	34	**RNA**→ hypo→
env-306	WSSedA1Ba3_1039983	-	339	291	**RNA**→ RPL15A (COG1632)PRK04243 (PRK04243)→
env-307	WSSedA2Ca3_1120722	-	217	169	**RNA**→ PRK04243 (PRK04243)Ribosomal_L15e (pfam00827)→
env-308	JREQ01002797.1	+	594	639	**RNA**→ RPL15A (COG1632)PRK04243 (PRK04243)→
env-309	JREQ01001068.1	+	594	639	**RNA**→ RPL15A (COG1632)PRK04243 (PRK04243)→ RPL15A (COG1632)PRK04243 (PRK04243)→
env-310	JREQ01004635.1	-	265	221	**RNA**→ PRK04243 (PRK04243)Ribosomal_L15e (pfam00827)→
env-311	JGI24422J19971_10442158	+	33	79	**RNA**→ RPL15A (COG1632)PRK04243 (PRK04243)→
env-312	JGI2172J19969_10165153	+	155	200	**RNA**→ RPL15A (COG1632)PRK04243 (PRK04243)→
env-313	JGI2173J19968_10019714	-	204	159	**RNA**→ PRK04243 (PRK04243)Ribosomal_L15e (pfam00827)→
env-314	JGI2171J19970_10593475	+	159	204	**RNA**→ PRK04243 (PRK04243)Ribosomal_L15e (pfam00827)→
env-315	JGI24422J19971_10429142	-	262	217	**RNA**→ PRK04243 (PRK04243)Ribosomal_L15e (pfam00827)→
env-316	SMTZ1_10013484	-	9454	9409	**RNA**→ PRK04243 (PRK04243)Ribosomal_L15e (pfam00827)→ PRK14555 (PRK14555)RNA_binding (pfam01877)→ RPP1 (COG1603)PRK00912 (PRK00912)→ PRK03717 (PRK03717)RNase_P_Rpp14 (pfam01900)→
env-317	JGI2172J19969_10374243	-	221	175	**RNA**→ PRK04243 (PRK04243)Ribosomal_L15e (pfam00827)→
env-318	JGI2173J19968_10119450	-	690	644	**RNA**→ hypo→ PRK04243 (PRK04243)Ribosomal_L15e (pfam00827)→
env-319	JGI2171J19970_10117997	-	822	776	**RNA**→ hypo→ PRK04243 (PRK04243)Ribosomal_L15e (pfam00827)→ RNA_binding (pfam01877)→
env-320	SMTZ1_10010538	-	5263	5217	**RNA**→ PRK04243 (PRK04243)Ribosomal_L15e (pfam00827)→ PRK14555 (PRK14555)RNA_binding (pfam01877)→ RPP1 (COG1603)PRK00912 (PRK00912)→ PRK03717 (PRK03717)RNase_P_Rpp14 (pfam01900)→
env-321	TB_LI09_3_1376570	+	4	50	**RNA**→ PRK04243 (PRK04243)Ribosomal_L15e (pfam00827)→
env-322	LGVD01087542.1	-	817	771	**RNA**→ RPL15A (COG1632)PRK04243 (PRK04243)→ RPL15A (COG1632)PRK04243 (PRK04243)→ RNA_binding (pfam01877)→
env-323	JGI24024J18818_10296176	-	207	161	**RNA**→ hypo→
env-324	SMTZ1_10000031	+	24101	24147	**RNA**→ PRK04243 (PRK04243)Ribosomal_L15e (pfam00827)→ PRK14555 (PRK14555)RNA_binding (pfam01877)→ PRK00912 (PRK00912)RNase_P_p30 (pfam01876)→ PRK03717 (PRK03717)RNase_P_Rpp14 (pfam01900)→
env-325	JGI2173J19968_10468069	-	221	175	**RNA**→ PRK04243 (PRK04243)Ribosomal_L15e (pfam00827)→
env-326	LGVF01282102.1	-	355	309	**RNA**→ RPL15A (COG1632)PRK04243 (PRK04243)→
env-327	LGVE01222292.1	-	389	343	**RNA**→ RPL15A (COG1632)PRK04243 (PRK04243)→
env-328	LGOV01067113.1	+	3689	3735	**RNA**→ PRK04243 (PRK04243)Ribosomal_L15e (pfam00827)→ PRK14555 (PRK14555)RNA_binding (pfam01877)→ PRK00912 (PRK00912)RNase_P_p30 (pfam01876)→ POP5 (COG1369)→

Pab-1-1	NC_000868.1	+	818641	818684	**RNA**→ PRK04243 (PRK04243)Ribosomal_L15e (pfam00827)→<br>hydrog_prot (TIGR00072)H2MP_MemB-H2evol (cd06067)→
Psp-2-1	NC_017946.1	-	888644	888600	**RNA**→ PRK04243 (PRK04243)Ribosomal_L15e (pfam00827)→
Tga-1-1	NC_012804.1	+	1735891	1735934	**RNA**→ PRK04243 (PRK04243)Ribosomal_L15e (pfam00827)→
Tsp-3-1	NC_018015.1	-	513605	513561	**RNA**→ PRK04243 (PRK04243)Ribosomal_L15e (pfam00827)→
Tsp-2-1	NC_016051.1	-	1259118	1259075	**RNA**→ PRK04243 (PRK04243)Ribosomal_L15e (pfam00827)→
Tna-1-1	NZ_CP007264.1	+	1944164	1944207	**RNA**→ PRK04243 (PRK04243)Ribosomal_L15e (pfam00827)→
Teu-1-1	NZ_CP008887.1	+	1002663	1002706	**RNA**→ PRK04243 (PRK04243)Ribosomal_L15e (pfam00827)→
env-329	3300001592_11448690	+	60	103	**RNA**→ PRK04243 (PRK04243)Ribosomal_L15e (pfam00827)→
Tsp-4-1	NZ_KE387149.1	+	630216	630259	**RNA**→ PRK04243 (PRK04243)Ribosomal_L15e (pfam00827)→
Tsi-1-1	NC_012883.1	+	1077704	1077747	**RNA**→ PRK04243 (PRK04243)Ribosomal_L15e (pfam00827)→
Tli-1-1	NC_022084.1	+	51943	51986	**RNA**→ PRK04243 (PRK04243)Ribosomal_L15e (pfam00827)→
env-330	JGI24019J35510_1000020	-	119249	119205	**RNA**→ PRK04243 (PRK04243)Ribosomal_L15e (pfam00827)→
Tba-1-1	NC_014804.1	+	122047	122091	**RNA**→ PRK04243 (PRK04243)Ribosomal_L15e (pfam00827)→
Tpa-1-1	NZ_CP006965.1	+	229409	229452	**RNA**→ PRK04243 (PRK04243)Ribosomal_L15e (pfam00827)→
Ppa-1-1	NZ_CP006019.1	+	356037	356081	**RNA**→ PRK04243 (PRK04243)Ribosomal_L15e (pfam00827)→ COG1325 (COG1325)PRK14555 (PRK14555)<br>PRK03892 (PRK03892)RPP1 (COG1603)→ PRK03717 (PRK03717)POP5 (COG1369)→<br>TIGR00341 (TIGR00341)DUF389 (pfam04087)→
Pfu-2-1	NC_003413.1	-	849639	849596	**RNA**→ PRK04243 (PRK04243)Ribosomal_L15e (pfam00827)→
Pfu-1-1	NC_018092.1	-	651734	651691	**RNA**→ PRK04243 (PRK04243)Ribosomal_L15e (pfam00827)→
Pho-1-1	NC_000961.1	+	891553	891596	**RNA**→ PRK04243 (PRK04243)Ribosomal_L15e (pfam00827)→<br>PRK04243 (PRK04243)Ribosomal_L15e (pfam00827)→<br>hydrog_prot (TIGR00072)H2MP_MemB-H2evol (cd06067)→
Psp-1-1	NC_015474.1	+	1545307	1545351	**RNA**→ PRK04243 (PRK04243)Ribosomal_L15e (pfam00827)→<br>hydrog_prot (TIGR00072)H2MP_MemB-H2evol (cd06067)→
Pya-1-1	NC_015680.1	+	224726	224769	**RNA**→ PRK04243 (PRK04243)Ribosomal_L15e (pfam00827)→
Tko-1-1	NC_006624.1	-	1280545	1280502	**RNA**→ PRK04243 (PRK04243)Ribosomal_L15e (pfam00827)→
Tsp-1-1	NC_015865.1	-	869933	869890	**RNA**→ PRK04243 (PRK04243)Ribosomal_L15e (pfam00827)→
Pfe-1-1	NZ_LANF01000010.1	-	104355	104312	**RNA**→ PRK04243 (PRK04243)Ribosomal_L15e (pfam00827)→
Ton-1-1	NC_011529.1	+	120097	120140	**RNA**→ PRK04243 (PRK04243)Ribosomal_L15e (pfam00827)→ COG1325 (COG1325)PRK14555 (PRK14555)<br>PRK03892 (PRK03892)RPP1 (COG1603)→
Tzi-1-1	NZ_AJLF01000001.1	-	197475	197432	**RNA**→ PRK04243 (PRK04243)Ribosomal_L15e (pfam00827)→<br>PRK04243 (PRK04243)Ribosomal_L15e (pfam00827)→
env-331	JGI24721J44947_10092924	+	379	432	**RNA**→ PRK04243 (PRK04243)Ribosomal_L15e (pfam00827)→ PRK12277 (PRK12277)RPL13 (COG4352)→<br>PRK14555 (PRK14555)RNA_binding (pfam01877)→ PRK00912 (PRK00912)RNase_P_p30 (pfam01876)→
env-332	JGI24721J26819_10019109	+	3371	3424	**RNA**→ PRK04243 (PRK04243)Ribosomal_L15e (pfam00827)→ PRK12277 (PRK12277)RPL13 (COG4352)→<br>PRK14555 (PRK14555)RNA_binding (pfam01877)→ PRK00912 (PRK00912)RNase_P_p30 (pfam01876)→
env-333	JGI24422J19971_10125801	-	282	234	**RNA**→ RPL15A (COG1632)PRK04243 (PRK04243)→
env-334	JGI24721J26819_10947299	+	173	218	**RNA**→
env-335	JGI24721J44947_10473589	-	321	276	**RNA**→ PRK04243 (PRK04243)Ribosomal_L15e (pfam00827)→
env-336	JGI24721J26819_10001491	+	21482	21529	**RNA**→ PRK04243 (PRK04243)Ribosomal_L15e (pfam00827)→ PRK12277 (PRK12277)RPL13 (COG4352)→<br>PRK14555 (PRK14555)RNA_binding (pfam01877)→ RPP1 (COG1603)PRK00912 (PRK00912)→<br>PRK03717 (PRK03717)RNase_P_Rpp14 (pfam01900)→
env-337	JGI24721J44947_10032959	-	4920	4873	**RNA**→ PRK04243 (PRK04243)Ribosomal_L15e (pfam00827)→ PRK12277 (PRK12277)RPL13 (COG4352)→<br>PRK14555 (PRK14555)RNA_binding (pfam01877)→ RPP1 (COG1603)PRK00912 (PRK00912)→<br>PRK03717 (PRK03717)RNase_P_Rpp14 (pfam01900)→
env-338	TB_LI09_3_1044718	+	801	846	**RNA**→ PRK04243 (PRK04243)Ribosomal_L15e (pfam00827)→
env-339	JGI24721J26819_10004799	-	3984	3939	**RNA**→ PRK04243 (PRK04243)Ribosomal_L15e (pfam00827)→ PRK12277 (PRK12277)RPL13 (COG4352)→<br>PRK14555 (PRK14555)RNA_binding (pfam01877)→ RPP1 (COG1603)PRK00912 (PRK00912)→<br>PRK03717 (PRK03717)RNase_P_Rpp14 (pfam01900)→
env-340	JGI24721J44947_10004827	-	9632	9587	**RNA**→ PRK04243 (PRK04243)Ribosomal_L15e (pfam00827)→ PRK12277 (PRK12277)RPL13 (COG4352)→<br>PRK14555 (PRK14555)RNA_binding (pfam01877)→ RPP1 (COG1603)PRK00912 (PRK00912)→<br>PRK03717 (PRK03717)RNase_P_Rpp14 (pfam01900)→
env-341	JGI20130J15003_1091000	+	354	398	**RNA**→ PRK04243 (PRK04243)Ribosomal_L15e (pfam00827)→
env-342	JGI20130J15133_1091000	+	354	398	**RNA**→ PRK04243 (PRK04243)Ribosomal_L15e (pfam00827)→

env-343	JGI24721J44947_10014353	-	2950	2906	**RNA**→ PRK04243 (PRK04243)Ribosomal_L15e (pfam00827)→ PRK12277 (PRK12277)RPL13 (COG4352)→ PRK14555 (PRK14555)RNA_binding (pfam01877)→ RPP1 (COG1603)PRK00912 (PRK00912)→ PRK03717 (PRK03717)RNase_P_Rpp14 (pfam01900)→
env-344	JGI20130J15003_1005034	+	5585	5630	**RNA**→ PRK04243 (PRK04243)Ribosomal_L15e (pfam00827)→ PRK12277 (PRK12277)RPL13 (COG4352)→ PRK14555 (PRK14555)RNA_binding (pfam01877)→ PRK00912 (PRK00912)RNase_P_p30 (pfam01876)→ RPP1 (COG1603)PRK00912 (PRK00912)→ PRK03717 (PRK03717)RNase_P_Rpp14 (pfam01900)→
env-345	JGI20130J15133_1005034	+	5585	5630	**RNA**→ PRK04243 (PRK04243)Ribosomal_L15e (pfam00827)→ PRK12277 (PRK12277)RPL13 (COG4352)→ PRK14555 (PRK14555)RNA_binding (pfam01877)→ PRK00912 (PRK00912)RNase_P_p30 (pfam01876)→ RPP1 (COG1603)PRK00912 (PRK00912)→ PRK03717 (PRK03717)RNase_P_Rpp14 (pfam01900)→
env-346	JGI24721J26819_10047889	-	1496	1451	**RNA**→ PRK04243 (PRK04243)Ribosomal_L15e (pfam00827)→ PRK12277 (PRK12277)RPL13 (COG4352)→ PRK14555 (PRK14555)RNA_binding (pfam01877)→ Acyl-CoA_dh_N (pfam02771)→
env-347	JGI24721J44947_10203480	+	445	490	**RNA**→ PRK04243 (PRK04243)Ribosomal_L15e (pfam00827)→
env-348	JREQ01006527.1	-	305	258	**RNA**→ PRK04243 (PRK04243)Ribosomal_L15e (pfam00827)→
env-349	JGI2172J19969_10000042	-	4265	4219	**RNA**→ RPL15A (COG1632)PRK04243 (PRK04243)→ PRK12277 (PRK12277)RPL13 (COG4352)→ PRK14555 (PRK14555)RNA_binding (pfam01877)→ RPP1 (COG1603)PRK00912 (PRK00912)→ PRK03717 (PRK03717)RNase_P_Rpp14 (pfam01900)→
env-350	JGI2173J19968_10000091	+	26877	26923	**RNA**→ RPL15A (COG1632)PRK04243 (PRK04243)→ PRK12277 (PRK12277)RPL13 (COG4352)→ PRK14555 (PRK14555)RNA_binding (pfam01877)→ PRK00912 (PRK00912)RNase_P_p30 (pfam01876)→
env-351	JGI2171J19970_10013243	-	1906	1860	**RNA**→ RPL15A (COG1632)PRK04243 (PRK04243)→ PRK12277 (PRK12277)RPL13 (COG4352)→ PRK14555 (PRK14555)RNA_binding (pfam01877)→ PRK00912 (PRK00912)RNase_P_p30 (pfam01876)→
env-352	JGI24422J19971_10240577	+	294	340	**RNA**→ RPL15A (COG1632)PRK04243 (PRK04243)→
env-353	JGI24422J19971_10004830	+	6226	6272	**RNA**→ PRK04243 (PRK04243)Ribosomal_L15e (pfam00827)→ PRK12277 (PRK12277)RPL13 (COG4352)→ PRK14555 (PRK14555)RNA_binding (pfam01877)→ PRK00912 (PRK00912)RNase_P_p30 (pfam01876)→ PRK03717 (PRK03717)POP5 (COG1369)→
env-354	JGI2172J19969_10181609	-	256	210	**RNA**→ PRK04243 (PRK04243)Ribosomal_L15e (pfam00827)→
env-355	JGI24422J19971_10009670	-	3736	3690	**RNA**→ PRK04243 (PRK04243)Ribosomal_L15e (pfam00827)→ PRK12277 (PRK12277)RPL13 (COG4352)→ PRK14555 (PRK14555)RNA_binding (pfam01877)→ RPP1 (COG1603)PRK00912 (PRK00912)→ PRK03717 (PRK03717)RNase_P_Rpp14 (pfam01900)→
env-356	JGI2172J19969_10028492	+	394	440	**RNA**→ PRK04243 (PRK04243)Ribosomal_L15e (pfam00827)→ PRK12277 (PRK12277)RPL13 (COG4352)→ PRK14555 (PRK14555)RNA_binding (pfam01877)→ PRK00912 (PRK00912)→
env-357	JGI24422J19971_10492128	-	124	78	**RNA**→ ←hypo
env-358	JGI24422J19971_10371442	+	38	84	**RNA**→ RPL15A (COG1632)PRK04243 (PRK04243)→
env-359	JGI24019J35510_1000149	+	57719	57764	**RNA**→ PRK04243 (PRK04243)Ribosomal_L15e (pfam00827)→ PRK12277 (PRK12277)RPL13 (COG4352)→ PRK14555 (PRK14555)RNA_binding (pfam01877)→ PRK00912 (PRK00912)RNase_P_p30 (pfam01876)→
env-360	JGI24020J35080_1000786	+	20256	20301	**RNA**→
env-361	JGI24020J35080_1002222	-	8791	8746	**RNA**→ PRK04243 (PRK04243)Ribosomal_L15e (pfam00827)→ PRK12277 (PRK12277)RPL13 (COG4352)→ PRK14555 (PRK14555)RNA_binding (pfam01877)→ PRK00912 (PRK00912)RNase_P_p30 (pfam01876)→ PRK03717 (PRK03717)RNase_P_Rpp14 (pfam01900)→
env-362	JGI24422J19971_10000253	-	1868	1822	**RNA**→ PRK04243 (PRK04243)Ribosomal_L15e (pfam00827)→ PRK12277 (PRK12277)RPL13 (COG4352)→ PRK14555 (PRK14555)RNA_binding (pfam01877)→ PRK00912 (PRK00912)RNase_P_p30 (pfam01876)→
env-363	JGI26339J46600_10000361	+	3851	3897	**RNA**→ PRK04243 (PRK04243)Ribosomal_L15e (pfam00827)→ PRK12277 (PRK12277)RPL13 (COG4352)→ PRK14555 (PRK14555)RNA_binding (pfam01877)→ RPP1 (COG1603)PRK00912 (PRK00912)→ PRK03717 (PRK03717)RNase_P_Rpp14 (pfam01900)→
env-364	JGI24025J20009_10035112	-	1739	1692	**RNA**→ PRK04243 (PRK04243)Ribosomal_L15e (pfam00827)→
env-365	JGI24422J19971_10447265	-	312	266	**RNA**→ RPL15A (COG1632)PRK04243 (PRK04243)→
env-366	JGI24422J19971_10005604	-	2467	2422	**RNA**→ hypo→ PRK04243 (PRK04243)Ribosomal_L15e (pfam00827)→ PRK12277 (PRK12277)RPL13 (COG4352)→ PRK14555 (PRK14555)RNA_binding (pfam01877)→ RPP1 (COG1603)PRK00912 (PRK00912)→ PRK03717 (PRK03717)RNase_P_Rpp14 (pfam01900)→
env-367	JGI2171J19970_10311510	+	423	468	**RNA**→ hypo→
env-368	JGI24422J19971_10296037	-	379	334	**RNA**→ hypo→ RPL15A (COG1632)PRK04243 (PRK04243)→
env-369	JGI24422J19971_10083467	+	1365	1411	**RNA**→ RPL15A (COG1632)PRK04243 (PRK04243)→
env-370	JGI24422J19971_10004103	-	1823	1778	**RNA**→ hypo→ PRK04243 (PRK04243)Ribosomal_L15e (pfam00827)→ PRK12277 (PRK12277)RPL13 (COG4352)→ PRK14555 (PRK14555)RNA_binding (pfam01877)→ PRK00912 (PRK00912)RNase_P_p30 (pfam01876)→
env-371	JGI2172J19969_10042350	+	1081	1126	**RNA**→ hypo→ RPL15A (COG1632)PRK04243 (PRK04243)→
env-372	JGI2173J19968_10455679	-	124	79	**RNA**→ hypo→

env-373	JGI2171J19970_10583661	+	269	314	**RNA**→ hypo→
env-374	SMTZ1_10006093	+	9757	9802	**RNA**→ hypo→ PRK04243 (PRK04243)Ribosomal_L15e (pfam00827)→ PRK12277 (PRK12277)RPL13 (COG4352)→ PRK14555 (PRK14555)RNA_binding (pfam01877)→ PRK00912 (PRK00912)RNase_P_p30 (pfam01876)→ PRK03717 (PRK03717)RNase_P_Rpp14 (pfam01900)→
env-375	JGI24025J20009_10133169	-	100	54	**RNA**→ ←hypo
env-376	JGI24422J19971_10019282	-	678	632	**RNA**→ PRK04243 (PRK04243)Ribosomal_L15e (pfam00827)→
env-377	JGI24025J20009_10005934	-	75	29	**RNA**→
env-378	JGI24025J20009_10025887	+	2637	2683	**RNA**→
env-379	JGI24025J20009_10011070	+	4857	4903	**RNA**→
env-380	JGI24025J20009_10053632	+	1424	1470	**RNA**→ ←hypo
env-381	JGI24422J19971_10774031	-	188	142	**RNA**→ PRK04243 (PRK04243)Ribosomal_L15e (pfam00827)→
env-382	JGI2171J19970_10721007	-	71	25	**RNA**→
env-383	JGI24025J20009_10067810	-	1101	1055	**RNA**→ PRK04243 (PRK04243)Ribosomal_L15e (pfam00827)→ PRK12277 (PRK12277)RPL13 (COG4352)→ COG1325 (COG1325)PRK14555 (PRK14555)→
env-384	JGI24025J20009_10468419	-	121	75	**RNA**→
env-385	JGI2172J19969_10020584	-	1314	1266	**RNA**→ hypo→ PRK04243 (PRK04243)Ribosomal_L15e (pfam00827)→ PRK12277 (PRK12277)RPL13 (COG4352)→ PRK14555 (PRK14555)RNA_binding (pfam01877)→
env-386	JGI24422J19971_10090327	-	1116	1068	**RNA**→ PRK04243 (PRK04243)Ribosomal_L15e (pfam00827)→ PRK12277 (PRK12277)RPL13 (COG4352)→ COG1325 (COG1325)PRK14555 (PRK14555)→
env-387	JGI24422J19971_10132992	-	55	7	**RNA**→ hypo→
env-388	JGI24721J26819_10042702	+	773	820	**RNA**→ PRK04243 (PRK04243)Ribosomal_L15e (pfam00827)→ PRK12277 (PRK12277)RPL13 (COG4352)→ PRK14555 (PRK14555)RNA_binding (pfam01877)→ RPP1 (COG1603)PRK00912 (PRK00912)→
env-389	JGI24721J44947_10035793	+	3282	3329	**RNA**→ PRK04243 (PRK04243)Ribosomal_L15e (pfam00827)→ PRK12277 (PRK12277)RPL13 (COG4352)→ PRK14555 (PRK14555)RNA_binding (pfam01877)→
env-390	JGI24721J26819_10782576	-	189	142	**RNA**→ PRK04243 (PRK04243)Ribosomal_L15e (pfam00827)→
env-391	JGI24721J44947_11017790	-	189	142	**RNA**→ PRK04243 (PRK04243)Ribosomal_L15e (pfam00827)→
env-392	GBSCES77__Contig_41123	-	1948	1904	**RNA**→ PRK04243 (PRK04243)Ribosomal_L15e (pfam00827)→ PRK12277 (PRK12277)RPL13 (COG4352)→ PRK14555 (PRK14555)RNA_binding (pfam01877)→ RPP1 (COG1603)PRK00912 (PRK00912)→
env-393	GBSCES77__Contig_16200	-	2430	2386	**RNA**→ PRK04243 (PRK04243)Ribosomal_L15e (pfam00827)→ PRK12277 (PRK12277)RPL13 (COG4352)→ PRK14555 (PRK14555)RNA_binding (pfam01877)→ RPP1 (COG1603)PRK00912 (PRK00912)→ PRK03717 (PRK03717)RNase_P_Rpp14 (pfam01900)→
env-394	GBSCES77__Contig_3030	+	791	835	**RNA**→ PRK04243 (PRK04243)Ribosomal_L15e (pfam00827)→ PRK12277 (PRK12277)RPL13 (COG4352)→ PRK14555 (PRK14555)RNA_binding (pfam01877)→ RPP1 (COG1603)PRK00912 (PRK00912)→ PRK03717 (PRK03717)RNase_P_Rpp14 (pfam01900)→
env-395	GBSCES77__Contig_34313	+	671	715	**RNA**→ PRK04243 (PRK04243)Ribosomal_L15e (pfam00827)→ PRK12277 (PRK12277)RPL13 (COG4352)→ PRK14555 (PRK14555)RNA_binding (pfam01877)→ RPP1 (COG1603)PRK00912 (PRK00912)→ PRK03717 (PRK03717)RNase_P_Rpp14 (pfam01900)→
env-396	GBSCES77__Contig_24520	-	2097	2053	**RNA**→ PRK04243 (PRK04243)Ribosomal_L15e (pfam00827)→ PRK12277 (PRK12277)RPL13 (COG4352)→ PRK14555 (PRK14555)RNA_binding (pfam01877)→ RPP1 (COG1603)PRK00912 (PRK00912)→
env-397	GBSCES77__Contig_45658	+	559	603	**RNA**→ PRK04243 (PRK04243)Ribosomal_L15e (pfam00827)→ PRK12277 (PRK12277)RPL13 (COG4352)→ PRK14555 (PRK14555)RNA_binding (pfam01877)→ RPP1 (COG1603)PRK00912 (PRK00912)→ PRK03717 (PRK03717)RNase_P_Rpp14 (pfam01900)→
env-398	GBSCES77__Contig_11719	-	2430	2386	**RNA**→ PRK04243 (PRK04243)Ribosomal_L15e (pfam00827)→ PRK12277 (PRK12277)RPL13 (COG4352)→ PRK14555 (PRK14555)RNA_binding (pfam01877)→ RPP1 (COG1603)PRK00912 (PRK00912)→ PRK03717 (PRK03717)RNase_P_Rpp14 (pfam01900)→
env-399	GBSCES77__Contig_6779	+	671	715	**RNA**→ PRK04243 (PRK04243)Ribosomal_L15e (pfam00827)→ PRK12277 (PRK12277)RPL13 (COG4352)→ PRK14555 (PRK14555)RNA_binding (pfam01877)→ RPP1 (COG1603)PRK00912 (PRK00912)→
env-400	GBSCES77__Contig_20802	-	2430	2386	**RNA**→ PRK04243 (PRK04243)Ribosomal_L15e (pfam00827)→ PRK12277 (PRK12277)RPL13 (COG4352)→ PRK14555 (PRK14555)RNA_binding (pfam01877)→ RPP1 (COG1603)PRK00912 (PRK00912)→ PRK03717 (PRK03717)RNase_P_Rpp14 (pfam01900)→
env-401	GBSCES77__Contig_29831	-	2430	2386	**RNA**→ PRK04243 (PRK04243)Ribosomal_L15e (pfam00827)→ PRK12277 (PRK12277)RPL13 (COG4352)→ PRK14555 (PRK14555)RNA_binding (pfam01877)→ RPP1 (COG1603)PRK00912 (PRK00912)→ PRK03717 (PRK03717)RNase_P_Rpp14 (pfam01900)→
env-402	JGI20130J15003_1003712	-	4413	4369	**RNA**→ PRK04243 (PRK04243)Ribosomal_L15e (pfam00827)→ PRK12277 (PRK12277)RPL13 (COG4352)→ PRK14555 (PRK14555)RNA_binding (pfam01877)→ RPP1 (COG1603)PRK00912 (PRK00912)→ PRK03717 (PRK03717)RNase_P_Rpp14 (pfam01900)→

env-403	JGI20130J15133_1003712	-	4413	4369	**RNA**→ PRK04243 (PRK04243)Ribosomal_L15e (pfam00827)→ PRK12277 (PRK12277)RPL13 (COG4352)→ PRK14555 (PRK14555)RNA_binding (pfam01877)→ RPP1 (COG1603)PRK00912 (PRK00912)→ PRK03717 (PRK03717)RNase_P_Rpp14 (pfam01900)→
env-404	JGI24721J26819_10021412	-	955	911	**RNA**→ PRK04243 (PRK04243)Ribosomal_L15e (pfam00827)→ PRK12277 (PRK12277)RPL13 (COG4352)→
env-405	JGI24721J44947_10036157	+	3704	3748	**RNA**→ PRK04243 (PRK04243)Ribosomal_L15e (pfam00827)→ PRK12277 (PRK12277)RPL13 (COG4352)→
env-406	JGI20130J15003_1001502	+	15316	15361	**RNA**→ PRK04243 (PRK04243)Ribosomal_L15e (pfam00827)→ PRK12277 (PRK12277)RPL13 (COG4352)→ PRK14555 (PRK14555)RNA_binding (pfam01877)→ RPP1 (COG1603)PRK00912 (PRK00912)→ PRK03717 (PRK03717)RNase_P_Rpp14 (pfam01900)→
env-407	JGI20130J15133_1001502	+	15316	15361	**RNA**→ PRK04243 (PRK04243)Ribosomal_L15e (pfam00827)→ PRK12277 (PRK12277)RPL13 (COG4352)→ PRK14555 (PRK14555)RNA_binding (pfam01877)→ RPP1 (COG1603)PRK00912 (PRK00912)→ PRK03717 (PRK03717)RNase_P_Rpp14 (pfam01900)→
env-408	JGI24721J26819_10470799	+	270	315	**RNA**→
env-409	JGI24721J44947_10040429	+	409	454	**RNA**→ PRK04243 (PRK04243)Ribosomal_L15e (pfam00827)→ PRK12277 (PRK12277)RPL13 (COG4352)→ PRK14555 (PRK14555)RNA_binding (pfam01877)→ RPP1 (COG1603)PRK00912 (PRK00912)→ PRK03717 (PRK03717)RNase_P_Rpp14 (pfam01900)→
env-410	JGI20130J15003_1014452	-	2679	2634	**RNA**→ PRK04243 (PRK04243)Ribosomal_L15e (pfam00827)→ PRK12277 (PRK12277)RPL13 (COG4352)→ PRK14555 (PRK14555)RNA_binding (pfam01877)→ PRK00912 (PRK00912)RNase_P_p30 (pfam01876)→ PRK03717 (PRK03717)RNase_P_Rpp14 (pfam01900)→
env-411	JGI20130J15133_1014452	-	2679	2634	**RNA**→ PRK04243 (PRK04243)Ribosomal_L15e (pfam00827)→ PRK12277 (PRK12277)RPL13 (COG4352)→ PRK14555 (PRK14555)RNA_binding (pfam01877)→ PRK00912 (PRK00912)RNase_P_p30 (pfam01876)→ PRK03717 (PRK03717)RNase_P_Rpp14 (pfam01900)→
env-412	JGI24721J26819_10066975	-	1900	1855	**RNA**→ PRK04243 (PRK04243)Ribosomal_L15e (pfam00827)→ PRK12277 (PRK12277)RPL13 (COG4352)→ PRK14555 (PRK14555)RNA_binding (pfam01877)→ PRK00912 (PRK00912)RNase_P_p30 (pfam01876)→
env-413	JGI24721J44947_10053920	-	3312	3267	**RNA**→ RPL15A (COG1632)PRK04243 (PRK04243)→ RPL15A (COG1632)PRK04243 (PRK04243)→ PRK12277 (PRK12277)RPL13 (COG4352)→ PRK14555 (PRK14555)RNA_binding (pfam01877)→ PRK00912 (PRK00912)RNase_P_p30 (pfam01876)→ PRK03717 (PRK03717)RNase_P_Rpp14 (pfam01900)→
env-414	JGI24721J26819_10007452	+	1045	1090	**RNA**→ PRK04243 (PRK04243)Ribosomal_L15e (pfam00827)→ PRK12277 (PRK12277)RPL13 (COG4352)→ PRK14555 (PRK14555)RNA_binding (pfam01877)→ RPP1 (COG1603)PRK00912 (PRK00912)→ PRK03717 (PRK03717)RNase_P_Rpp14 (pfam01900)→
env-415	JGI24721J44947_10012689	+	1045	1091	**RNA**→ PRK04243 (PRK04243)Ribosomal_L15e (pfam00827)→ PRK12277 (PRK12277)RPL13 (COG4352)→ PRK14555 (PRK14555)RNA_binding (pfam01877)→ RPP1 (COG1603)PRK00912 (PRK00912)→ PRK03717 (PRK03717)RNase_P_Rpp14 (pfam01900)→
env-416	JGI24721J26819_10000566	+	1047	1092	**RNA**→ PRK04243 (PRK04243)Ribosomal_L15e (pfam00827)→ PRK12277 (PRK12277)RPL13 (COG4352)→ PRK14555 (PRK14555)RNA_binding (pfam01877)→ RPP1 (COG1603)PRK00912 (PRK00912)→ PRK03717 (PRK03717)RNase_P_Rpp14 (pfam01900)→
env-417	JGI24721J44947_10000929	-	39531	39486	**RNA**→ PRK04243 (PRK04243)Ribosomal_L15e (pfam00827)→ PRK12277 (PRK12277)RPL13 (COG4352)→ PRK14555 (PRK14555)RNA_binding (pfam01877)→ RPP1 (COG1603)PRK00912 (PRK00912)→ PRK03717 (PRK03717)RNase_P_Rpp14 (pfam01900)→
env-418	JGI20130J15003_1040014	+	320	364	**RNA**→ PRK04243 (PRK04243)Ribosomal_L15e (pfam00827)→ PRK12277 (PRK12277)RPL13 (COG4352)→
env-419	JGI20130J15133_1040014	+	320	364	**RNA**→ PRK04243 (PRK04243)Ribosomal_L15e (pfam00827)→ PRK12277 (PRK12277)RPL13 (COG4352)→
env-420	JGI24721J26819_10008710	-	5175	5131	**RNA**→ PRK04243 (PRK04243)Ribosomal_L15e (pfam00827)→ PRK12277 (PRK12277)RPL13 (COG4352)→ PRK14555 (PRK14555)RNA_binding (pfam01877)→ RPP1 (COG1603)PRK00912 (PRK00912)→ PRK03717 (PRK03717)RNase_P_Rpp14 (pfam01900)→
env-421	JGI24721J44947_10014925	-	5175	5131	**RNA**→ PRK04243 (PRK04243)Ribosomal_L15e (pfam00827)→ PRK12277 (PRK12277)RPL13 (COG4352)→ PRK14555 (PRK14555)RNA_binding (pfam01877)→ RPP1 (COG1603)PRK00912 (PRK00912)→ PRK03717 (PRK03717)RNase_P_Rpp14 (pfam01900)→
env-422	JGI20130J15003_1006708	+	320	364	**RNA**→ PRK04243 (PRK04243)Ribosomal_L15e (pfam00827)→ PRK12277 (PRK12277)RPL13 (COG4352)→ PRK14555 (PRK14555)RNA_binding (pfam01877)→ RPP1 (COG1603)PRK00912 (PRK00912)→ PRK03717 (PRK03717)RNase_P_Rpp14 (pfam01900)→
env-423	JGI20130J15133_1006708	+	320	364	**RNA**→ PRK04243 (PRK04243)Ribosomal_L15e (pfam00827)→ PRK12277 (PRK12277)RPL13 (COG4352)→ PRK14555 (PRK14555)RNA_binding (pfam01877)→ RPP1 (COG1603)PRK00912 (PRK00912)→ PRK03717 (PRK03717)RNase_P_Rpp14 (pfam01900)→
env-424	JGI24422J19971_10000261	-	3582	3531	**RNA**→ PRK04243 (PRK04243)Ribosomal_L15e (pfam00827)→ PRK12277 (PRK12277)RPL13 (COG4352)→ PRK14555 (PRK14555)RNA_binding (pfam01877)→ RPP1 (COG1603)PRK00912 (PRK00912)→ PRK03717 (PRK03717)RNase_P_Rpp14 (pfam01900)→
env-425	JGI2173J19968_10016998	+	1834	1885	**RNA**→ PRK04243 (PRK04243)Ribosomal_L15e (pfam00827)→ PRK12277 (PRK12277)RPL13 (COG4352)→

env-426	SMTZ1_10000482	+	8104	8155	**RNA**→ PRK04243 (PRK04243)Ribosomal_L15e (pfam00827)→ PRK12277 (PRK12277)RPL13 (COG4352)→ PRK14555 (PRK14555)RNA_binding (pfam01877)→ RPP1 (COG1603)PRK00912 (PRK00912)→ PRK03717 (PRK03717)RNase_P_Rpp14 (pfam01900)→
env-427	JGI2171J19970_10271144	-	353	302	**RNA**→ RPL15A (COG1632)PRK04243 (PRK04243)→
env-428	SMTZ1_10032630	-	6487	6436	**RNA**→ ←hypo
env-429	JGI2172J19969_10033071	+	406	457	**RNA**→ ←hypo
env-430	JGI2171J19970_10083154	-	519	468	**RNA**→ ←hypo
env-431	JGI24422J19971_10005257	+	6290	6341	**RNA**→ PRK04243 (PRK04243)Ribosomal_L15e (pfam00827)→ PRK12277 (PRK12277)RPL13 (COG4352)→ PRK14555 (PRK14555)RNA_binding (pfam01877)→ PRK00912 (PRK00912)RNase_P_p30 (pfam01876)→
env-432	JGI24422J19971_10022574	+	1413	1464	**RNA**→ PRK04243 (PRK04243)Ribosomal_L15e (pfam00827)→ PRK12277 (PRK12277)Ribosomal_L13e (pfam01294)→ PRK14555 (PRK14555)RNA_binding (pfam01877)→ RPP1 (COG1603)PRK00912 (PRK00912)→ PRK03717 (PRK03717)RNase_P_Rpp14 (pfam01900)→
env-433	JGI2172J19969_10002202	-	186	135	**RNA**→ PRK04243 (PRK04243)Ribosomal_L15e (pfam00827)→
env-434	JGI2172J19969_10005325	+	99	150	**RNA**→ PRK04243 (PRK04243)Ribosomal_L15e (pfam00827)→ PRK12277 (PRK12277)RPL13 (COG4352)→ PRK14555 (PRK14555)RNA_binding (pfam01877)→ RPP1 (COG1603)PRK00912 (PRK00912)→ PRK03717 (PRK03717)RNase_P_Rpp14 (pfam01900)→
env-435	JGI2173J19968_10000262	+	3449	3500	**RNA**→ PRK04243 (PRK04243)Ribosomal_L15e (pfam00827)→ PRK12277 (PRK12277)RPL13 (COG4352)→ PRK14555 (PRK14555)RNA_binding (pfam01877)→ RPP1 (COG1603)PRK00912 (PRK00912)→ PRK03717 (PRK03717)RNase_P_Rpp14 (pfam01900)→
env-436	JGI2171J19970_10002036	+	2023	2074	**RNA**→ PRK04243 (PRK04243)Ribosomal_L15e (pfam00827)→ PRK12277 (PRK12277)RPL13 (COG4352)→ PRK14555 (PRK14555)RNA_binding (pfam01877)→ RPP1 (COG1603)PRK00912 (PRK00912)→ PRK03717 (PRK03717)RNase_P_Rpp14 (pfam01900)→
env-437	JGI24422J19971_10243708	+	627	678	**RNA**→
env-438	JGI24721J44947_10007540	-	2578	2525	**RNA**→ PRK04243 (PRK04243)Ribosomal_L15e (pfam00827)→ PRK12277 (PRK12277)RPL13 (COG4352)→ PRK14555 (PRK14555)RNA_binding (pfam01877)→ RPP1 (COG1603)PRK00912 (PRK00912)→ PRK03717 (PRK03717)RNase_P_Rpp14 (pfam01900)→
env-439	JGI24721J26819_10005385	+	10350	10403	**RNA**→ PRK04243 (PRK04243)Ribosomal_L15e (pfam00827)→ PRK12277 (PRK12277)RPL13 (COG4352)→ PRK14555 (PRK14555)RNA_binding (pfam01877)→ RPP1 (COG1603)PRK00912 (PRK00912)→ PRK03717 (PRK03717)RNase_P_Rpp14 (pfam01900)→
env-440	JGI2172J19969_10330441	+	133	180	**RNA**→ RPL15A (COG1632)PRK04243 (PRK04243)→
env-441	JGI24422J19971_10230678	-	541	494	**RNA**→ PRK04243 (PRK04243)Ribosomal_L15e (pfam00827)→
env-442	JGI24422J19971_10330695	-	518	473	**RNA**→ PRK04243 (PRK04243)Ribosomal_L15e (pfam00827)→
env-443	JGI2171J19970_10787211	-	272	227	**RNA**→ RPL15A (COG1632)PRK04243 (PRK04243)→
env-444	JGI2172J19969_10018448	+	977	1022	**RNA**→ PRK04243 (PRK04243)Ribosomal_L15e (pfam00827)→
env-445	SMTZ1_10005895	-	12232	12187	**RNA**→ hypo→ PRK04243 (PRK04243)Ribosomal_L15e (pfam00827)→
env-446	JGI2171J19970_10523375	-	233	185	**RNA**→ PRK04243 (PRK04243)Ribosomal_L15e (pfam00827)→
env-447	JGI24721J26819_10011755	+	847	896	**RNA**→ PRK04243 (PRK04243)Ribosomal_L15e (pfam00827)→ PRK12277 (PRK12277)RPL13 (COG4352)→ PRK14555 (PRK14555)RNA_binding (pfam01877)→ RPP1 (COG1603)PRK00912 (PRK00912)→ PRK03717 (PRK03717)RNase_P_Rpp14 (pfam01900)→
env-448	JGI24721J44947_10000482	-	52170	52121	**RNA**→ PRK04243 (PRK04243)Ribosomal_L15e (pfam00827)→ PRK12277 (PRK12277)RPL13 (COG4352)→ PRK14555 (PRK14555)RNA_binding (pfam01877)→ RPP1 (COG1603)PRK00912 (PRK00912)→ PRK03717 (PRK03717)RNase_P_Rpp14 (pfam01900)→
env-449	JGI2173J19968_10000733	+	4783	4829	**RNA**→ PRK04243 (PRK04243)Ribosomal_L15e (pfam00827)→
env-450	JGI2172J19969_10000762	-	8526	8480	**RNA**→ PRK04243 (PRK04243)Ribosomal_L15e (pfam00827)→
env-451	JGI2171J19970_10187156	-	317	271	**RNA**→ RPL15A (COG1632)PRK04243 (PRK04243)→
env-452	JGI24422J19971_10000606	+	10914	10960	**RNA**→ PRK04243 (PRK04243)Ribosomal_L15e (pfam00827)→
env-453	NCBI_PBSM_READ_68102390	+	1020	1063	**RNA**→
Mvo-1-1	NC_014222.1	-	1392579	1392536	**RNA**→ PRK04243 (PRK04243)Ribosomal_L15e (pfam00827)→
env-454	SakLake120mDRAFT_c0000339	+	19224	19273	**RNA**→ RPL15A (COG1632)PRK04243 (PRK04243)→ RPP1 (COG1603)PRK00912 (PRK00912)→ RNase_P_Rpp14 (pfam01900)→ PRK03996 (PRK03996)proteasome_alpha_archeal (cd03756)→ Sdo1 (COG1500)PRK13760 (PRK13760)→ rpl37ae (PRK03976)Ribosomal_L37ae (pfam01780)→

ume-1-1	NC_009464.1	-	994905	994839	**RNA**→ PRK04243 (PRK04243)Ribosomal_L15e (pfam00827)→ COG1325 (COG1325)PRK14555 (PRK14555)<br>PRK00912 (PRK00912)RNase_P_p30 (pfam01876)→ PRK03717 (PRK03717)RNase_P_Rpp14 (pfam01900)→<br>PRK03996 (PRK03996)proteasome_alpha_archeal (cd03756)→ Sdo1 (COG1500)PRK13760 (PRK13760)→<br>Rrp4 (COG1097)PRK04163 (PRK04163)→ RNase_PH_archRRP41 (cd11366)PRK03983 (PRK03983)→<br>RNase_PH_archRRP42 (cd11365)PRK04282 (PRK04282)→ rpl37ae (PRK03976)Ribosomal_L37ae (pfam01780)<br>RPC10 (COG1996)rpoP (PRK00398)→ Pcc1 (COG2892)Pcc1 (pfam09341)→<br>gimC_beta (TIGR02338)Prefoldin_2 (pfam01920)→ hypo→
CMe-2-1	NZ_JMIY01000003.1	+	337389	337437	**RNA**→ PRK04243 (PRK04243)Ribosomal_L15e (pfam00827)→<br>PRK14555 (PRK14555)RNA_binding (pfam01877)→
Mev-1-1	NC_014253.1	-	592639	592588	**RNA**→ PRK04243 (PRK04243)Ribosomal_L15e (pfam00827)→<br>PRK14555 (PRK14555)RNA_binding (pfam01877)→ PRK00912 (PRK00912)RNase_P_p30 (pfam01876)→<br>PRK03717 (PRK03717)POP5 (COG1369)→ PRK03996 (PRK03996)proteasome_alpha_archeal (cd03756)→<br>Sdo1 (COG1500)PRK13760 (PRK13760)→ hypo→ hypo→ hypo→
env-455	WSSedA2C_c207490	+	72	126	**RNA**→ PRK04243 (PRK04243)Ribosomal_L15e (pfam00827)→
env-456	WSSedA2Ca3_1207490	+	72	126	**RNA**→ PRK04243 (PRK04243)Ribosomal_L15e (pfam00827)→
env-457	WSSedA1TJGI_c024695	+	624	678	**RNA**→ PRK04243 (PRK04243)Ribosomal_L15e (pfam00827)→
env-458	WSSedA1Ba2_1000622	+	1087	1141	**RNA**→ PRK04243 (PRK04243)Ribosomal_L15e (pfam00827)→ RPP1 (COG1603)PRK00912 (PRK00912)→<br>PRK03717 (PRK03717)RNase_P_Rpp14 (pfam01900)→<br>PRK03996 (PRK03996)proteasome_alpha_archeal (cd03756)→ Sdo1 (COG1500)PRK13760 (PRK13760)→<br>rpl37ae (PRK03976)Ribosomal_L37ae (pfam01780)→
env-459	WSSedA1Ba3_1003811	+	2343	2397	**RNA**→ PRK04243 (PRK04243)Ribosomal_L15e (pfam00827)→ RPP1 (COG1603)PRK00912 (PRK00912)→<br>PRK03717 (PRK03717)RNase_P_Rpp14 (pfam01900)→
env-460	JGI26437J51864_10646690	+	117	172	**RNA**→
env-461	WSSedB1B2_c010830	+	452	507	**RNA**→ RPL15A (COG1632)PRK04243 (PRK04243)→
env-462	JGI20214J14112_1483376	+	5	54	**RNA**→ PRK04243 (PRK04243)Ribosomal_L15e (pfam00827)→
Mzh-1-1	NC_015676.1	-	1281814	1281760	**RNA**→ PRK04243 (PRK04243)Ribosomal_L15e (pfam00827)→<br>PRK14555 (PRK14555)RNA_binding (pfam01877)→ RPP1 (COG1603)PRK00912 (PRK00912)→<br>PRK03717 (PRK03717)RNase_P_Rpp14 (pfam01900)→<br>PRK03996 (PRK03996)proteasome_alpha_archeal (cd03756)→ Sdo1 (COG1500)PRK13760 (PRK13760)→<br>Rrp4 (COG1097)PRK04163 (PRK04163)→ RNase_PH_archRRP41 (cd11366)PRK03983 (PRK03983)→<br>RNase_PH_archRRP42 (cd11365)PRK04282 (PRK04282)→ rpl37ae (PRK03976)Ribosomal_L37ae (pfam01780)<br>RPC10 (COG1996)rpoP (PRK00398)→ Brix (smart00879)IMP4 (COG2136)→<br>Pcc1 (COG2892)Pcc1 (pfam09341)→ gimC_beta (TIGR02338)Prefoldin_2 (pfam01920)→<br>PRK14538 (PRK14538)DHH (pfam01368)→ PaaK (COG1541)AMP-binding_C_2 (pfam14535)→
env-463	JGI24020J35080_1007792	+	2787	2832	**RNA**→ RPL15A (COG1632)PRK04243 (PRK04243)→
env-464	JGI24019J35510_1000013	+	160353	160398	**RNA**→ PRK04243 (PRK04243)Ribosomal_L15e (pfam00827)→<br>PRK14555 (PRK14555)RNA_binding (pfam01877)→ RPP1 (COG1603)PRK00912 (PRK00912)→<br>PRK03717 (PRK03717)RNase_P_Rpp14 (pfam01900)→<br>PRK03996 (PRK03996)proteasome_alpha_archeal (cd03756)→ Sdo1 (COG1500)PRK13760 (PRK13760)→<br>Rrp4 (COG1097)PRK04163 (PRK04163)→ RNase_PH_archRRP41 (cd11366)PRK03983 (PRK03983)→<br>RNase_PH_archRRP42 (cd11365)PRK04282 (PRK04282)→ rpl37ae (PRK03976)Ribosomal_L37ae (pfam01780)<br>Pcc1 (COG2892)Pcc1 (pfam09341)→ gimC_beta (TIGR02338)GimC (COG1382)→
env-465	JGI24724J26744_10037895	-	1732	1679	**RNA**→ PRK04243 (PRK04243)Ribosomal_L15e (pfam00827)→<br>ABC_ATPase (cd00267)AAA (smart00382)NapF (COG1145)Fer4_7 (pfam12838)→
env-466	LWFCAn_GLQU6AQ01BL2KP	-	265	213	**RNA**→ PRK04243 (PRK04243)Ribosomal_L15e (pfam00827)→
env-467	JGI24730J26740_1001090	+	9520	9567	**RNA**→ PRK04243 (PRK04243)Ribosomal_L15e (pfam00827)→<br>PRK14555 (PRK14555)RNA_binding (pfam01877)→
env-468	JGI24730J26740_1002557	-	5350	5303	**RNA**→ PRK04243 (PRK04243)Ribosomal_L15e (pfam00827)→<br>PRK14555 (PRK14555)RNA_binding (pfam01877)→
env-469	taComm3_C6796	+	6853	6906	**RNA**→ PRK04243 (PRK04243)Ribosomal_L15e (pfam00827)→<br>PRK00912 (PRK00912)RNase_P_p30 (pfam01876)→ PRK03717 (PRK03717)RNase_P_Rpp14 (pfam01900)→<br>PRK03996 (PRK03996)proteasome_alpha_archeal (cd03756)→ Sdo1 (COG1500)PRK13760 (PRK13760)→<br>rpl37ae (PRK03976)Ribosomal_L37ae (pfam01780)→ RPC10 (COG1996)rpoP (PRK00398)→

env-470	JGI12104J13512_1001091	+	6473	6526	**RNA**→ PRK04243 (PRK04243)Ribosomal_L15e (pfam00827)→ PRK00912 (PRK00912)RNase_P_p30 (pfam01876)→ PRK03717 (PRK03717)RNase_P_Rpp14 (pfam01900)→ PRK03996 (PRK03996)proteasome_alpha_archeal (cd03756)→ Sdo1 (COG1500)PRK13760 (PRK13760)→ rpl37ae (PRK03976)Ribosomal_L37ae (pfam01780)→ RPC10 (COG1996)rpoP (PRK00398)→
env-471	JGI11944J13513_1001226	+	6477	6530	**RNA**→ PRK04243 (PRK04243)Ribosomal_L15e (pfam00827)→ PRK00912 (PRK00912)RNase_P_p30 (pfam01876)→ PRK03717 (PRK03717)RNase_P_Rpp14 (pfam01900)→ PRK03996 (PRK03996)proteasome_alpha_archeal (cd03756)→ Sdo1 (COG1500)PRK13760 (PRK13760)→ rpl37ae (PRK03976)Ribosomal_L37ae (pfam01780)→ RPC10 (COG1996)rpoP (PRK00398)→
Mta-1-1	NZ_AGIY02000001.1	+	329402	329455	**RNA**→ PRK04243 (PRK04243)Ribosomal_L15e (pfam00827)→ PRK00912 (PRK00912)RNase_P_p30 (pfam01876)→ PRK00912 (PRK00912)RNase_P_p30 (pfam01876)→ PRK03717 (PRK03717)RNase_P_Rpp14 (pfam01900)→ PRK03996 (PRK03996)proteasome_alpha_archeal (cd03756)→ Sdo1 (COG1500)PRK13760 (PRK13760)→ rpl37ae (PRK03976)Ribosomal_L37ae (pfam01780)→ RPC10 (COG1996)rpoP (PRK00398)→
env-472	TB_LI09_4_10122878	+	413	459	**RNA**→ RPL15A (COG1632)PRK04243 (PRK04243)→
env-473	TB_LI09_4_10800703	+	157	203	**RNA**→ hypo→
env-474	BDMC2_contig02027	-	540	492	**RNA**→ RPL15A (COG1632)PRK04243 (PRK04243)→ PRK04243 (PRK04243)Ribosomal_L15e (pfam00827)→
Mpa-1-1	NC_011832.1	-	2661123	2661072	**RNA**→ PRK04243 (PRK04243)Ribosomal_L15e (pfam00827)→ RPP1 (COG1603)PRK00912 (PRK00912)→ PRK03717 (PRK03717)RNase_P_Rpp14 (pfam01900)→ PRK03996 (PRK03996)proteasome_alpha_archeal (cd03756)→ Sdo1 (COG1500)PRK13760 (PRK13760)→ rpl37ae (PRK03976)Ribosomal_L37ae (pfam01780)→ RPC10 (COG1996)rpoP (PRK00398)→ IMP4 (COG2136)PRK00933 (PRK00933)→ Pcc1 (COG2892)PRK14887 (PRK14887)→ yhbS (COG3153)Acetyltransf_7 (pfam13508)→
env-475	JGI20225J20221_1000037	+	100793	100845	**RNA**→ PRK04243 (PRK04243)Ribosomal_L15e (pfam00827)→ PRK00912 (PRK00912)RNase_P_p30 (pfam01876)→ PRK03717 (PRK03717)RNase_P_Rpp14 (pfam01900)→ PRK03996 (PRK03996)proteasome_alpha_archeal (cd03756)→ Sdo1 (COG1500)PRK13760 (PRK13760)→ rpl37ae (PRK03976)Ribosomal_L37ae (pfam01780)→ RPC10 (COG1996)rpoP (PRK00398)→ Pcc1 (COG2892)Pcc1 (pfam09341)→ gimC_beta (TIGR02338)Prefoldin_2 (pfam01920)→
env-476	WSSedL1Cam_c0003	-	103534	103482	**RNA**→ PRK04243 (PRK04243)Ribosomal_L15e (pfam00827)→ PRK00912 (PRK00912)RNase_P_p30 (pfam01876)→ PRK03717 (PRK03717)RNase_P_Rpp14 (pfam01900)→ PRK03996 (PRK03996)proteasome_alpha_archeal (cd03756)→ Sdo1 (COG1500)PRK13760 (PRK13760)→ rpl37ae (PRK03976)Ribosomal_L37ae (pfam01780)→ RPC10 (COG1996)rpoP (PRK00398)→ Pcc1 (COG2892)Pcc1 (pfam09341)→ gimC_beta (TIGR02338)Prefoldin_2 (pfam01920)→
env-477	JGI24729J20445_1007040	+	2798	2848	**RNA**→ ←hypo
env-478	JGI24730J26740_1000148	-	22730	22680	**RNA**→ PRK04243 (PRK04243)Ribosomal_L15e (pfam00827)→ RPP1 (COG1603)PRK00912 (PRK00912)→ PRK03717 (PRK03717)RNase_P_Rpp14 (pfam01900)→ PRK03996 (PRK03996)proteasome_alpha_archeal (cd03756)→ Sdo1 (COG1500)PRK13760 (PRK13760)→ rpl37ae (PRK03976)Ribosomal_L37ae (pfam01780)→ RPC10 (COG1996)rpoP (PRK00398)→ PRK00933 (PRK00933)→ Pcc1 (COG2892)Pcc1 (pfam09341)→
env-479	3300000558_10037240	-	1302	1247	**RNA**→ PRK04243 (PRK04243)Ribosomal_L15e (pfam00827)→ PRK00912 (PRK00912)RNase_P_p30 (pfam01876)→
env-480	3300000568_10759524	-	62	6	**RNA**→
env-481	3300000032_0445971	-	908	852	**RNA**→ PRK04243 (PRK04243)Ribosomal_L15e (pfam00827)→ PRK00912 (PRK00912)→
env-482	3300000568_10762646	-	251	195	**RNA**→ PRK04243 (PRK04243)Ribosomal_L15e (pfam00827)→
env-483	3300000568_10812672	+	106	162	**RNA**→ PRK04243 (PRK04243)Ribosomal_L15e (pfam00827)→
env-484	3300000568_10718528	+	26	82	**RNA**→ PRK04243 (PRK04243)Ribosomal_L15e (pfam00827)→
env-485	3300001567_10000590	+	50837	50893	**RNA**→ PRK04243 (PRK04243)Ribosomal_L15e (pfam00827)→ PRK00912 (PRK00912)RNase_P_p30 (pfam01876)→ PRK03717 (PRK03717)RNase_P_Rpp14 (pfam01900)→ PRK03996 (PRK03996)proteasome_alpha_archeal (cd03756)→ Sdo1 (COG1500)PRK13760 (PRK13760)→ rpl37ae (PRK03976)Ribosomal_L37ae (pfam01780)→ RPC10 (COG1996)rpoP (PRK00398)→ Pcc1 (COG2892)Pcc1 (pfam09341)→
env-486	3300000568_10082522	+	368	421	**RNA**→ PRK04243 (PRK04243)Ribosomal_L15e (pfam00827)→
env-487	3300001567_10000995	-	27743	27691	**RNA**→ PRK04243 (PRK04243)Ribosomal_L15e (pfam00827)→ PRK00912 (PRK00912)RNase_P_p30 (pfam01876)→ PRK03717 (PRK03717)RNase_P_Rpp14 (pfam01900)→ PRK03996 (PRK03996)proteasome_alpha_archeal (cd03756)→ Sdo1 (COG1500)PRK13760 (PRK13760)→ rpl37ae (PRK03976)Ribosomal_L37ae (pfam01780)→ RPC10 (COG1996)rpoP (PRK00398)→ Pcc1 (COG2892)Pcc1 (pfam09341)→

env-488	JGI26437J51864_10005597	-	1881	1829	**RNA**→ PRK04243 (PRK04243)Ribosomal_L15e (pfam00827)→ RPP1 (COG1603)PRK00912 (PRK00912)→ PRK03717 (PRK03717)RNase_P_Rpp14 (pfam01900)→
env-489	lwComb_BCIB16813_x1	+	697	749	**RNA**→
env-490	WSSedA1Ba3_1028801	+	186	238	**RNA**→ PRK04243 (PRK04243)Ribosomal_L15e (pfam00827)→ PRK00912 (PRK00912)RNase_P_p30 (pfam01876)→
env-491	WSSedA1Ba3_1008109	+	557	609	**RNA**→ PRK04243 (PRK04243)Ribosomal_L15e (pfam00827)→ PRK00912 (PRK00912)RNase_P_p30 (pfam01876)→ PRK03717 (PRK03717)RNase_P_Rpp14 (pfam01900)→ Proteasome_A_N (smart00948)PRE1 (COG0638)→
env-492	WSSedA1TJGI_c023358	-	396	344	**RNA**→ RPL15A (COG1632)PRK04243 (PRK04243)→
env-493	WSSedA1Ba_1000153	-	7580	7528	**RNA**→ PRK04243 (PRK04243)Ribosomal_L15e (pfam00827)→ PRK00912 (PRK00912)RNase_P_p30 (pfam01876)→ PRK03717 (PRK03717)RNase_P_Rpp14 (pfam01900)→ PRK03996 (PRK03996)proteasome_alpha_archeal (cd03756)→ Sdo1 (COG1500)PRK13760 (PRK13760)→ rpl37ae (PRK03976)Ribosomal_L37ae (pfam01780)→ RPC10 (COG1996)rpoP (PRK00398)→ Pcc1 (COG2892)Pcc1 (pfam09341)→
env-494	WSSedA1Ba2_1001361	-	527	475	**RNA**→ RPL15A (COG1632)PRK04243 (PRK04243)→
env-495	JGI26437J51864_10016078	-	877	823	**RNA**→ PRK04243 (PRK04243)Ribosomal_L15e (pfam00827)→ PRK00912 (PRK00912)→
env-496	LWFCAn_GLQU6AQ01EP314	-	386	333	**RNA**→ RPL15A (COG1632)PRK04243 (PRK04243)→
env-497	JGI24729J20445_1011542	+	712	765	**RNA**→ PRK04243 (PRK04243)Ribosomal_L15e (pfam00827)→ PRK00912 (PRK00912)RNase_P_p30 (pfam01876)→
env-498	JGI24728J21555_1031680	-	431	378	**RNA**→ RPL15A (COG1632)PRK04243 (PRK04243)→
env-499	3300001580_10004398	-	7500	7446	**RNA**→ PRK04243 (PRK04243)Ribosomal_L15e (pfam00827)→ RPP1 (COG1603)PRK00912 (PRK00912)→ PRK03717 (PRK03717)RNase_P_Rpp14 (pfam01900)→ PRK03996 (PRK03996)proteasome_alpha_archeal (cd03756)→ Sdo1 (COG1500)PRK13760 (PRK13760)→ rpl37ae (PRK03976)Ribosomal_L37ae (pfam01780)→ RPC10 (COG1996)rpoP (PRK00398)→ Pcc1 (COG2892)Pcc1 (pfam09341)→ gimC_beta (TIGR02338)Prefoldin_2 (pfam01920)→ spoT_relA (TIGR00691)SpoT (COG0317)→
env-500	JGI24729J20445_1004823	-	1184	1131	**RNA**→ PRK04243 (PRK04243)Ribosomal_L15e (pfam00827)→ RPP1 (COG1603)PRK00912 (PRK00912)→
env-501	JGI24732J26686_1002952	-	3151	3098	**RNA**→ PRK04243 (PRK04243)Ribosomal_L15e (pfam00827)→ RPP1 (COG1603)PRK00912 (PRK00912)→ PRK03717 (PRK03717)RNase_P_Rpp14 (pfam01900)→ PRK03996 (PRK03996)proteasome_alpha_archeal (cd03756)→ Sdo1 (COG1500)PRK13760 (PRK13760)→
env-502	JGI24732J26686_1094881	-	48	1	**RNA**→
env-503	JGI24729J20445_1001141	+	9710	9763	**RNA**→ PRK04243 (PRK04243)Ribosomal_L15e (pfam00827)→ RPP1 (COG1603)PRK00912 (PRK00912)→
env-504	JGI24732J26686_1139212	-	359	306	**RNA**→ RPL15A (COG1632)PRK04243 (PRK04243)→
env-505	TB_GS10_10_10036752	+	1072	1125	**RNA**→ PRK04243 (PRK04243)Ribosomal_L15e (pfam00827)→ RPP1 (COG1603)PRK00912 (PRK00912)→
env-506	JGI24730J26740_1018719	+	53	106	**RNA**→ PRK04243 (PRK04243)Ribosomal_L15e (pfam00827)→ RPP1 (COG1603)PRK00912 (PRK00912)→ RNase_P_Rpp14 (pfam01900)→
env-507	SakLake120mDRAFT_c0042489	-	330	276	**RNA**→ ←hypo
env-508	3300001605_10380848	+	193	245	**RNA**→ PRK04243 (PRK04243)Ribosomal_L15e (pfam00827)→
env-509	3300000032_0318192	-	212	159	**RNA**→ PRK04243 (PRK04243)Ribosomal_L15e (pfam00827)→
env-510	3300001580_10019781	-	923	870	**RNA**→ PRK04243 (PRK04243)Ribosomal_L15e (pfam00827)→ hypo→
env-511	3300000558_10012475	+	6222	6275	**RNA**→ RPL15A (COG1632)PRK04243 (PRK04243)→
env-512	3300001567_10108382	+	569	622	**RNA**→ PRK04243 (PRK04243)Ribosomal_L15e (pfam00827)→
env-513	3300001580_10224100	+	558	611	**RNA**→ RPL15A (COG1632)PRK04243 (PRK04243)→
env-514	JGI26437J51864_10657425	-	97	44	**RNA**→ hypo→
env-515	3300001580_10162759	+	554	608	**RNA**→ RPL15A (COG1632)PRK04243 (PRK04243)→
env-516	3300001567_10107871	+	571	625	**RNA**→ PRK04243 (PRK04243)Ribosomal_L15e (pfam00827)→
env-517	JGI24728J21555_1000054	-	38560	38510	**RNA**→ PRK04243 (PRK04243)Ribosomal_L15e (pfam00827)→ RPP1 (COG1603)PRK00912 (PRK00912)→ PRK03717 (PRK03717)RNase_P_Rpp14 (pfam01900)→ PRK03996 (PRK03996)proteasome_alpha_archeal (cd03756)→ Sdo1 (COG1500)PRK13760 (PRK13760)→ rpl37ae (PRK03976)Ribosomal_L37ae (pfam01780)→
env-518	JGI24728J21555_1000137	-	38579	38529	**RNA**→ PRK04243 (PRK04243)Ribosomal_L15e (pfam00827)→ RPP1 (COG1603)PRK00912 (PRK00912)→ PRK03717 (PRK03717)RNase_P_Rpp14 (pfam01900)→ PRK03996 (PRK03996)proteasome_alpha_archeal (cd03756)→ Sdo1 (COG1500)PRK13760 (PRK13760)→ rpl37ae (PRK03976)Ribosomal_L37ae (pfam01780)→

env-519	JGI24728J21555_1000138	+	5955	6005	**RNA**→ PRK04243 (PRK04243)Ribosomal_L15e (pfam00827)→ RPP1 (COG1603)PRK00912 (PRK00912)→ PRK03717 (PRK03717)RNase_P_Rpp14 (pfam01900)→ PRK03996 (PRK03996)proteasome_alpha_archeal (cd03756)→ Sdo1 (COG1500)PRK13760 (PRK13760)→ rpl37ae (PRK03976)Ribosomal_L37ae (pfam01780)→
env-520	JGI24728J21555_1000139	+	5947	5997	**RNA**→ PRK04243 (PRK04243)Ribosomal_L15e (pfam00827)→ RPP1 (COG1603)PRK00912 (PRK00912)→ PRK03717 (PRK03717)RNase_P_Rpp14 (pfam01900)→ PRK03996 (PRK03996)proteasome_alpha_archeal (cd03756)→ Sdo1 (COG1500)PRK13760 (PRK13760)→ rpl37ae (PRK03976)Ribosomal_L37ae (pfam01780)→
env-521	JGI24728J21555_1000140	-	38568	38518	**RNA**→ PRK04243 (PRK04243)Ribosomal_L15e (pfam00827)→ RPP1 (COG1603)PRK00912 (PRK00912)→ PRK03717 (PRK03717)RNase_P_Rpp14 (pfam01900)→ PRK03996 (PRK03996)proteasome_alpha_archeal (cd03756)→ Sdo1 (COG1500)PRK13760 (PRK13760)→ rpl37ae (PRK03976)Ribosomal_L37ae (pfam01780)→
env-522	SL_5KL_010_SED_10012660	-	440	389	**RNA**→ RPL15A (COG1632)PRK04243 (PRK04243)→
env-523	LCWY01000735.1	-	1783	1732	**RNA**→ PRK04243 (PRK04243)Ribosomal_L15e (pfam00827)→ PRK00912 (PRK00912)RNase_P_p30 (pfam01876)→ PRK03717 (PRK03717)RNase_P_Rpp14 (pfam01900)→
env-524	LCWZ01000388.1	-	3340	3289	**RNA**→ PRK04243 (PRK04243)Ribosomal_L15e (pfam00827)→ PRK00912 (PRK00912)RNase_P_p30 (pfam01876)→ PRK03717 (PRK03717)RNase_P_Rpp14 (pfam01900)→ PRK03996 (PRK03996)proteasome_alpha_archeal (cd03756)→ PRK13760 (PRK13760)SBDS (pfam01172)→ Sdo1 (COG1500)SBDS_C (pfam09377)→ hypo→
env-525	SL_5KL_010_SED_10011794	+	2661	2714	**RNA**→ PRK04243 (PRK04243)Ribosomal_L15e (pfam00827)→
env-526	SL_3KL_010_SED_10302459	+	187	240	**RNA**→ RPL15A (COG1632)PRK04243 (PRK04243)→
env-527	LCWZ01004261.1	-	1068	1015	**RNA**→ RPL15A (COG1632)PRK04243 (PRK04243)→ PRK00912 (PRK00912)RNase_P_p30 (pfam01876)→
env-528	JGI2065J20421_1127642	+	79	128	**RNA**→ PRK04243 (PRK04243)Ribosomal_L15e (pfam00827)→
env-529	2013877062	+	961	1010	**RNA**→ PRK04243 (PRK04243)Ribosomal_L15e (pfam00827)→ PRK00912 (PRK00912)RNase_P_p30 (pfam01876)→
env-530	BMHB3a_c117030	+	389	438	**RNA**→ PRK04243 (PRK04243)Ribosomal_L15e (pfam00827)→
env-531	BMHB3a_c11385	-	71	22	**RNA**→ hypo→
env-532	JGI24730J26740_1000056	+	41101	41154	**RNA**→ PRK04243 (PRK04243)Ribosomal_L15e (pfam00827)→ PRK00912 (PRK00912)RNase_P_p30 (pfam01876)→ PRK03717 (PRK03717)RNase_P_Rpp14 (pfam01900)→ PRK03996 (PRK03996)proteasome_alpha_archeal (cd03756)→ Sdo1 (COG1500)PRK13760 (PRK13760)→ rpl37ae (PRK03976)Ribosomal_L37ae (pfam01780)→ RPC10 (COG1996)rpoP (PRK00398)→ Pcc1 (COG2892)Pcc1 (pfam09341)→ gimC_beta (TIGR02338)GimC (COG1382)→
env-533	JGI24730J26740_1000097	-	49515	49462	**RNA**→ PRK04243 (PRK04243)Ribosomal_L15e (pfam00827)→ PRK00912 (PRK00912)RNase_P_p30 (pfam01876)→ PRK03717 (PRK03717)RNase_P_Rpp14 (pfam01900)→ PRK03996 (PRK03996)proteasome_alpha_archeal (cd03756)→ Sdo1 (COG1500)PRK13760 (PRK13760)→ rpl37ae (PRK03976)Ribosomal_L37ae (pfam01780)→ RPC10 (COG1996)rpoP (PRK00398)→ Pcc1 (COG2892)Pcc1 (pfam09341)→ gimC_beta (TIGR02338)GimC (COG1382)→
CMe-1-1	NC_009712.1	-	1906137	1906085	**RNA**→ PRK04243 (PRK04243)Ribosomal_L15e (pfam00827)→ PRK00912 (PRK00912)RNase_P_p30 (pfam01876)→ PRK03717 (PRK03717)RNase_P_Rpp14 (pfam01900)→ PRK03996 (PRK03996)proteasome_alpha_archeal (cd03756)→ Sdo1 (COG1500)PRK13760 (PRK13760)→ rpl37ae (PRK03976)Ribosomal_L37ae (pfam01780)→ RPC10 (COG1996)rpoP (PRK00398)→ Pcc1 (COG2892)Pcc1 (pfam09341)→ PRK09343 (PRK09343)GimC (COG1382)→
env-534	PRSSGFe2_Sequence0000007570	-	3311	3258	**RNA**→ PRK04243 (PRK04243)Ribosomal_L15e (pfam00827)→ PRK00912 (PRK00912)RNase_P_p30 (pfam01876)→ PRK03717 (PRK03717)RNase_P_Rpp14 (pfam01900)→ PRK03996 (PRK03996)proteasome_alpha_archeal (cd03756)→ Sdo1 (COG1500)PRK13760 (PRK13760)→
env-535	JGI26339J46600_10050974	+	104	156	**RNA**→ RPL15A (COG1632)PRK04243 (PRK04243)→ PRK00912 (PRK00912)→
env-536	SL_3KL_010_SED_10003966	-	9606	9558	**RNA**→ PRK04243 (PRK04243)Ribosomal_L15e (pfam00827)→ RPP1 (COG1603)PRK00912 (PRK00912)→ PRK03717 (PRK03717)RNase_P_Rpp14 (pfam01900)→ PRK03996 (PRK03996)proteasome_alpha_archeal (cd03756)→ Sdo1 (COG1500)PRK13760 (PRK13760)→ rpl37ae (PRK03976)Ribosomal_L37ae (pfam01780)→ RPC10 (COG1996)rpoP (PRK00398)→ IMP4 (COG2136)PRK00933 (PRK00933)→ Pcc1 (COG2892)Pcc1 (pfam09341)→ PRK09343 (PRK09343)GimC (COG1382)→
env-537	SL_3KL_010_SED_10034542	+	3130	3178	**RNA**→ RPL15A (COG1632)PRK04243 (PRK04243)→

env-538	JGI12104J13512_1003036	-	8013	7965	RNA→ PRK04243 (PRK04243)Ribosomal_L15e (pfam00827)→
					PRK00912 (PRK00912)RNase_P_p30 (pfam01876)→ PRK03717 (PRK03717)RNase_P_Rpp14 (pfam01900)→
					PRK03996 (PRK03996)proteasome_alpha_archeal (cd03756)→ Sdo1 (COG1500)PRK13760 (PRK13760)→
					rpl37ae (PRK03976)Ribosomal_L37ae (pfam01780)→
env-539	JGI11944J13513_1000334	+	17500	17548	RNA→ PRK04243 (PRK04243)Ribosomal_L15e (pfam00827)→
					PRK00912 (PRK00912)RNase_P_p30 (pfam01876)→ PRK03717 (PRK03717)RNase_P_Rpp14 (pfam01900)→
					PRK03996 (PRK03996)proteasome_alpha_archeal (cd03756)→ Sdo1 (COG1500)PRK13760 (PRK13760)→
					rpl37ae (PRK03976)Ribosomal_L37ae (pfam01780)→
env-540	PCEOT_contig21575	-	446	398	RNA→ RPL15A (COG1632)PRK04243 (PRK04243)→
Mhu-1-1	NC_007796.1	+	2543565	2543613	RNA→ PRK04243 (PRK04243)Ribosomal_L15e (pfam00827)→ RPP1 (COG1603)PRK00912 (PRK00912)→
					PRK03717 (PRK03717)RNase_P_Rpp14 (pfam01900)→
					PRK03996 (PRK03996)proteasome_alpha_archeal (cd03756)→ Sdo1 (COG1500)PRK13760 (PRK13760)→
					rpl37ae (PRK03976)Ribosomal_L37ae (pfam01780)→ RPC10 (COG1996)rpoP (PRK00398)→
					PRK00933 (PRK00933)→ Pcc1 (COG2892)Pcc1 (pfam09341)→ PRK09343 (PRK09343)Prefoldin_2 (pfam0192
					hypo→ hypo→ hypo→
					PRK14960 (PRK14960)CARDB (pfam07705)NosD (pfam05048)Beta_helix (pfam13229)DUF4832 (pfam16116)-
					PRK14960 (PRK14960)CARDB (pfam07705)NosD (pfam05048)Beta_helix (pfam13229)DUF4832 (pfam16116)-
					PRK14960 (PRK14960)CARDB (pfam07705)NosD (pfam05048)Beta_helix (pfam13229)DUF4832 (pfam16116)-
env-541	JGI24730J26740_1152247	-	310	262	RNA→ PRK04243 (PRK04243)Ribosomal_L15e (pfam00827)→
env-542	3300001580_10002056	+	12087	12136	RNA→ PRK04243 (PRK04243)Ribosomal_L15e (pfam00827)→
					PRK00912 (PRK00912)RNase_P_p30 (pfam01876)→ PRK03717 (PRK03717)RNase_P_Rpp14 (pfam01900)→
					PRK03996 (PRK03996)proteasome_alpha_archeal (cd03756)→ Sdo1 (COG1500)PRK13760 (PRK13760)→
					rpl37ae (PRK03976)Ribosomal_L37ae (pfam01780)→
env-543	JGI26437J51864_10233459	-	229	178	RNA→ PRK04243 (PRK04243)Ribosomal_L15e (pfam00827)→
env-544	JGI24730J26740_1087991	-	503	453	RNA→ RPL15A (COG1632)PRK04243 (PRK04243)→
env-545	JGI24731J21663_1016982	-	1473	1423	RNA→ PRK04243 (PRK04243)Ribosomal_L15e (pfam00827)→ RPP1 (COG1603)PRK00912 (PRK00912)→
					hypo→
env-546	JGI24732J26686_1102613	-	402	352	RNA→ RPL15A (COG1632)PRK04243 (PRK04243)→
Mli-1-1	NZ_CM001555.1	+	718118	718169	RNA→ PRK04243 (PRK04243)Ribosomal_L15e (pfam00827)→
					PRK04243 (PRK04243)Ribosomal_L15e (pfam00827)→ PRK00912 (PRK00912)RNase_P_p30 (pfam01876)→
					PRK00912 (PRK00912)RNase_P_p30 (pfam01876)→ PRK03717 (PRK03717)RNase_P_Rpp14 (pfam01900)→
					PRK03717 (PRK03717)RNase_P_Rpp14 (pfam01900)→
					PRK03996 (PRK03996)proteasome_alpha_archeal (cd03756)→
					PRK03996 (PRK03996)proteasome_alpha_archeal (cd03756)→ Sdo1 (COG1500)PRK13760 (PRK13760)→
					Sdo1 (COG1500)PRK13760 (PRK13760)→ rpl37ae (PRK03976)Ribosomal_L37ae (pfam01780)→
					rpl37ae (PRK03976)Ribosomal_L37ae (pfam01780)→ RPC10 (COG1996)rpoP (PRK00398)→
					RPC10 (COG1996)rpoP (PRK00398)→ IMP4 (COG2136)PRK00933 (PRK00933)→
					IMP4 (COG2136)PRK00933 (PRK00933)→ Pcc1 (COG2892)Pcc1 (pfam09341)→
					Pcc1 (COG2892)Pcc1 (pfam09341)→ gimC_beta (TIGR02338)GimC (COG1382)→
					gimC_beta (TIGR02338)GimC (COG1382)→
env-547	JGI24712J26585_10534437	+	165	211	RNA→
env-548	JGI24711J26586_10063235	+	193	239	RNA→ PRK04243 (PRK04243)Ribosomal_L15e (pfam00827)→
					PRK00912 (PRK00912)RNase_P_p30 (pfam01876)→
env-549	JGI24709J26583_10002362	+	11095	11141	RNA→ RPL15A (COG1632)PRK04243 (PRK04243)→ PRK00912 (PRK00912)RNase_P_p30 (pfam01876)→
					PRK03717 (PRK03717)RNase_P_Rpp14 (pfam01900)→
					PRK03996 (PRK03996)proteasome_alpha_archeal (cd03756)→ Sdo1 (COG1500)PRK13760 (PRK13760)→
					rpl37ae (PRK03976)Ribosomal_L37ae (pfam01780)→
env-550	JGI24710J26742_10002715	-	4756	4710	RNA→ RPL15A (COG1632)PRK04243 (PRK04243)→ PRK00912 (PRK00912)RNase_P_p30 (pfam01876)→
					PRK03717 (PRK03717)RNase_P_Rpp14 (pfam01900)→
					PRK03996 (PRK03996)proteasome_alpha_archeal (cd03756)→ Sdo1 (COG1500)PRK13760 (PRK13760)→
					rpl37ae (PRK03976)Ribosomal_L37ae (pfam01780)→
env-551	JGI24500J29687_10032170	+	390	436	RNA→ RPL15A (COG1632)PRK04243 (PRK04243)→ PRK00912 (PRK00912)RNase_P_p30 (pfam01876)→
					PRK03717 (PRK03717)RNase_P_Rpp14 (pfam01900)→
					PRK03996 (PRK03996)proteasome_alpha_archeal (cd03756)→ Sdo1 (COG1500)PRK13760 (PRK13760)→

Mse-1-1	NZ_JXOJ01000004.1	+	7876	7922	**RNA**→ hypo→ PRK04243 (PRK04243)Ribosomal_L15e (pfam00827)→ PRK00912 (PRK00912)RNase_P_p30 (pfam01876)→ PRK03717 (PRK03717)RNase_P_Rpp14 (pfam01900)→ PRK03996 (PRK03996)proteasome_alpha_archeal (cd03756)→ Sdo1 (COG1500)PRK13760 (PRK13760)→ rpl37ae (PRK03976)Ribosomal_L37ae (pfam01780)→ RPC10 (COG1996)rpoP (PRK00398)→ IMP4 (COG2136)PRK00933 (PRK00933)→ Pcc1 (COG2892)Pcc1 (pfam09341)→
Mma-1-1	NC_009051.1	-	1680920	1680874	**RNA**→ PRK04243 (PRK04243)Ribosomal_L15e (pfam00827)→ PRK00912 (PRK00912)RNase_P_p30 (pfam01876)→ PRK03717 (PRK03717)RNase_P_Rpp14 (pfam01900)→ PRK03996 (PRK03996)proteasome_alpha_archeal (cd03756)→ Sdo1 (COG1500)PRK13760 (PRK13760)→ rpl37ae (PRK03976)Ribosomal_L37ae (pfam01780)→ RPC10 (COG1996)rpoP (PRK00398)→ IMP4 (COG2136)PRK00933 (PRK00933)→ Pcc1 (COG2892)Pcc1 (pfam09341)→
Msp-5-1	NZ_JMIO01000049.1	+	18527	18573	**RNA**→ PRK04243 (PRK04243)Ribosomal_L15e (pfam00827)→ RPP1 (COG1603)PRK00912 (PRK00912)→ PRK03717 (PRK03717)RNase_P_Rpp14 (pfam01900)→ PRK03996 (PRK03996)proteasome_alpha_archeal (cd03756)→ Sdo1 (COG1500)PRK13760 (PRK13760)→ rpl37ae (PRK03976)Ribosomal_L37ae (pfam01780)→ RPC10 (COG1996)rpoP (PRK00398)→ IMP4 (COG2136)PRK00933 (PRK00933)→ hypo→ Pcc1 (COG2892)Pcc1 (pfam09341)→
env-552	JGI24712J26585_10228661	+	308	354	**RNA**→ PRK04243 (PRK04243)Ribosomal_L15e (pfam00827)→
env-553	JGI24707J26582_10055176	-	84	39	**RNA**→ ←hypo
env-554	JGI24708J26588_10022103	-	1842	1797	**RNA**→ PRK04243 (PRK04243)Ribosomal_L15e (pfam00827)→ RPP1 (COG1603)PRK00912 (PRK00912)→ PRK03717 (PRK03717)RNase_P_Rpp14 (pfam01900)→
env-555	JGI24712J26585_10005651	+	1497	1542	**RNA**→ PRK04243 (PRK04243)Ribosomal_L15e (pfam00827)→ RPP1 (COG1603)PRK00912 (PRK00912)→ PRK03717 (PRK03717)RNase_P_Rpp14 (pfam01900)→ PRK03996 (PRK03996)proteasome_alpha_archeal (cd03756)→ Sdo1 (COG1500)PRK13760 (PRK13760)→ Sdo1 (COG1500)PRK13760 (PRK13760)→ rpl37ae (PRK03976)Ribosomal_L37ae (pfam01780)→
env-556	JGI24710J26742_10007141	+	2776	2821	**RNA**→ PRK04243 (PRK04243)Ribosomal_L15e (pfam00827)→ RPP1 (COG1603)PRK00912 (PRK00912)→ PRK03717 (PRK03717)RNase_P_Rpp14 (pfam01900)→ PRK03996 (PRK03996)proteasome_alpha_archeal (cd03756)→ Sdo1 (COG1500)PRK13760 (PRK13760)→ rpl37ae (PRK03976)Ribosomal_L37ae (pfam01780)→
env-557	JGI24502J29692_10004908	+	2312	2357	**RNA**→ PRK04243 (PRK04243)Ribosomal_L15e (pfam00827)→ RPP1 (COG1603)PRK00912 (PRK00912)→ PRK03717 (PRK03717)RNase_P_Rpp14 (pfam01900)→ PRK03996 (PRK03996)proteasome_alpha_archeal (cd03756)→ Sdo1 (COG1500)PRK13760 (PRK13760)→ rpl37ae (PRK03976)Ribosomal_L37ae (pfam01780)→
env-558	JGI24503J29689_10004405	+	2321	2366	**RNA**→ PRK04243 (PRK04243)Ribosomal_L15e (pfam00827)→ RPP1 (COG1603)PRK00912 (PRK00912)→ PRK03717 (PRK03717)RNase_P_Rpp14 (pfam01900)→ PRK03996 (PRK03996)proteasome_alpha_archeal (cd03756)→ Sdo1 (COG1500)PRK13760 (PRK13760)→ rpl37ae (PRK03976)Ribosomal_L37ae (pfam01780)→
env-559	JGI24707J26582_10007021	+	73	118	**RNA**→ PRK04243 (PRK04243)Ribosomal_L15e (pfam00827)→ RPP1 (COG1603)PRK00912 (PRK00912)→ PRK03717 (PRK03717)RNase_P_Rpp14 (pfam01900)→ PRK03996 (PRK03996)proteasome_alpha_archeal (cd03756)→ Sdo1 (COG1500)PRK13760 (PRK13760)→ rpl37ae (PRK03976)Ribosomal_L37ae (pfam01780)→
env-560	JGI24708J26588_10445994	-	46	1	**RNA**→
env-561	JGI24711J26586_10009933	-	1842	1797	**RNA**→ PRK04243 (PRK04243)Ribosomal_L15e (pfam00827)→ RPP1 (COG1603)PRK00912 (PRK00912)→ PRK03717 (PRK03717)RNase_P_Rpp14 (pfam01900)→
env-562	JGI24709J26583_10016669	+	2203	2248	**RNA**→ PRK04243 (PRK04243)Ribosomal_L15e (pfam00827)→ PRK00912 (PRK00912)RNase_P_p30 (pfam01876)→ RPP1 (COG1603)PRK00912 (PRK00912)→ PRK03717 (PRK03717)RNase_P_Rpp14 (pfam01900)→
env-563	JGI24500J29687_10006466	+	556	601	**RNA**→ PRK04243 (PRK04243)Ribosomal_L15e (pfam00827)→ RPP1 (COG1603)PRK00912 (PRK00912)→ PRK03717 (PRK03717)RNase_P_Rpp14 (pfam01900)→ Proteasome_A_N (smart00948)PRE1 (COG0638)→
env-564	3300000568_10010387	+	293	339	**RNA**→ PRK04243 (PRK04243)Ribosomal_L15e (pfam00827)→
env-565	3300000032_0492979	+	132	178	**RNA**→
env-566	3300000558_10049236	+	193	239	**RNA**→ PRK04243 (PRK04243)Ribosomal_L15e (pfam00827)→ PRK00912 (PRK00912)RNase_P_p30 (pfam01876)→ PRK03717 (PRK03717)RNase_P_Rpp14 (pfam01900)→ PRK03996 (PRK03996)proteasome_alpha_archeal (cd03756)→ Sdo1 (COG1500)PRK13760 (PRK13760)→ rpl37ae (PRK03976)Ribosomal_L37ae (pfam01780)→
env-567	3300000032_0511128	+	210	256	**RNA**→
env-568	3300000032_0037457	-	630	584	**RNA**→ PRK04243 (PRK04243)Ribosomal_L15e (pfam00827)→
env-569	3300001592_10229257	+	50	96	**RNA**→ PRK04243 (PRK04243)Ribosomal_L15e (pfam00827)→

env-570	3300000507_100942	+	577	624	**RNA**→ PRK04243 (PRK04243)Ribosomal_L15e (pfam00827)→ PRK00912 (PRK00912)RNase_P_p30 (pfam01876)→ PRK03717 (PRK03717)RNase_P_Rpp14 (pfam01900)→ PRK03996 (PRK03996)proteasome_alpha_archeal (cd03756)→ Sdo1 (COG1500)PRK13760 (PRK13760)→ rpl37ae (PRK03976)Ribosomal_L37ae (pfam01780)→
env-571	JGI24713J26584_10001369	+	2226	2272	**RNA**→ RPL15A (COG1632)PRK04243 (PRK04243)→ RPP1 (COG1603)PRK00912 (PRK00912)→ PRK03717 (PRK03717)RNase_P_Rpp14 (pfam01900)→ PRK03996 (PRK03996)proteasome_alpha_archeal (cd03756)→ Sdo1 (COG1500)PRK13760 (PRK13760)→ rpl37ae (PRK03976)Ribosomal_L37ae (pfam01780)→
env-572	JGI24714J26587_10001520	-	13960	13913	**RNA**→ RPL15A (COG1632)PRK04243 (PRK04243)→ RPP1 (COG1603)PRK00912 (PRK00912)→ PRK03717 (PRK03717)RNase_P_Rpp14 (pfam01900)→ PRK03996 (PRK03996)proteasome_alpha_archeal (cd03756)→ Sdo1 (COG1500)PRK13760 (PRK13760)→ rpl37ae (PRK03976)Ribosomal_L37ae (pfam01780)→
env-573	JGI24504J29685_1025823	+	107	154	**RNA**→ RPL15A (COG1632)PRK04243 (PRK04243)→
env-574	3300001592_10015540	-	752	702	**RNA**→ PRK04243 (PRK04243)Ribosomal_L15e (pfam00827)→
env-575	3300000032_0000829	+	1108	1158	**RNA**→ PRK04243 (PRK04243)Ribosomal_L15e (pfam00827)→ PRK00912 (PRK00912)RNase_P_p30 (pfam01876)→ PRK03717 (PRK03717)RNase_P_Rpp14 (pfam01900)→ PRK03996 (PRK03996)proteasome_alpha_archeal (cd03756)→ Sdo1 (COG1500)PRK13760 (PRK13760)→ rpl37ae (PRK03976)Ribosomal_L37ae (pfam01780)→ RPC10 (COG1996)rpoP (PRK00398)→ PRK00933 (PRK00933)→ Pcc1 (COG2892)PRK14887 (PRK14887)→ gimC_beta (TIGR02338)GimC (COG138[illegible]
env-576	3300000558_12043292	+	53	104	**RNA**→ PRK04243 (PRK04243)Ribosomal_L15e (pfam00827)→
env-577	3300000568_10821221	-	73	23	**RNA**→ hypo→
env-578	3300001567_10009047	+	577	629	**RNA**→ PRK04243 (PRK04243)Ribosomal_L15e (pfam00827)→ RPP1 (COG1603)PRK00912 (PRK00912)→ PRK03717 (PRK03717)RNase_P_Rpp14 (pfam01900)→ PRK03996 (PRK03996)proteasome_alpha_archeal (cd03756)→ Sdo1 (COG1500)PRK13760 (PRK13760)→ rpl37ae (PRK03976)Ribosomal_L37ae (pfam01780)→
env-579	3300001592_10165112	+	124	175	**RNA**→ PRK04243 (PRK04243)Ribosomal_L15e (pfam00827)→ PRK00912 (PRK00912)RNase_P_p30 (pfam01876)→
env-580	3300001567_10003826	+	5679	5730	**RNA**→ PRK04243 (PRK04243)Ribosomal_L15e (pfam00827)→ RPP1 (COG1603)PRK00912 (PRK00912)→ PRK03717 (PRK03717)RNase_P_Rpp14 (pfam01900)→ PRK03996 (PRK03996)proteasome_alpha_archeal (cd03756)→ Sdo1 (COG1500)PRK13760 (PRK13760)→ rpl37ae (PRK03976)Ribosomal_L37ae (pfam01780)→
env-581	3300000032_0010281	-	14322	14272	**RNA**→ PRK04243 (PRK04243)Ribosomal_L15e (pfam00827)→ RPP1 (COG1603)PRK00912 (PRK00912)→ PRK03717 (PRK03717)RNase_P_Rpp14 (pfam01900)→ PRK03996 (PRK03996)proteasome_alpha_archeal (cd03756)→ Sdo1 (COG1500)PRK13760 (PRK13760)→ rpl37ae (PRK03976)Ribosomal_L37ae (pfam01780)→ RPC10 (COG1996)rpoP (PRK00398)→
env-582	3300000558_11443494	-	245	195	**RNA**→ PRK04243 (PRK04243)Ribosomal_L15e (pfam00827)→
env-583	3300000568_10668294	-	19377	19327	**RNA**→ PRK04243 (PRK04243)Ribosomal_L15e (pfam00827)→ RPP1 (COG1603)PRK00912 (PRK00912)→ PRK03717 (PRK03717)RNase_P_Rpp14 (pfam01900)→ PRK03996 (PRK03996)proteasome_alpha_archeal (cd03756)→ Sdo1 (COG1500)PRK13760 (PRK13760)→ rpl37ae (PRK03976)Ribosomal_L37ae (pfam01780)→ RPC10 (COG1996)rpoP (PRK00398)→
env-584	3300001580_10001349	-	19377	19327	**RNA**→ PRK04243 (PRK04243)Ribosomal_L15e (pfam00827)→ RPP1 (COG1603)PRK00912 (PRK00912)→ PRK03717 (PRK03717)RNase_P_Rpp14 (pfam01900)→ PRK03996 (PRK03996)proteasome_alpha_archeal (cd03756)→ Sdo1 (COG1500)PRK13760 (PRK13760)→ rpl37ae (PRK03976)Ribosomal_L37ae (pfam01780)→ RPC10 (COG1996)rpoP (PRK00398)→
env-585	JGI12104J13512_1001025	+	6838	6890	**RNA**→ RPL15A (COG1632)PRK04243 (PRK04243)→ PRK00912 (PRK00912)RNase_P_p30 (pfam01876)→ PRK03717 (PRK03717)RNase_P_Rpp14 (pfam01900)→ PRK03996 (PRK03996)proteasome_alpha_archeal (cd03756)→ Sdo1 (COG1500)PRK13760 (PRK13760)→ rpl37ae (PRK03976)Ribosomal_L37ae (pfam01780)→ RPC10 (COG1996)rpoP (PRK00398)→
env-586	JGI11944J13513_1001244	+	5853	5905	**RNA**→ RPL15A (COG1632)PRK04243 (PRK04243)→ PRK00912 (PRK00912)RNase_P_p30 (pfam01876)→ PRK03717 (PRK03717)RNase_P_Rpp14 (pfam01900)→ PRK03996 (PRK03996)proteasome_alpha_archeal (cd03756)→ Sdo1 (COG1500)PRK13760 (PRK13760)→ rpl37ae (PRK03976)Ribosomal_L37ae (pfam01780)→ RPC10 (COG1996)rpoP (PRK00398)→
env-587	taComm3_C10759	-	580	529	**RNA**→ PRK04243 (PRK04243)Ribosomal_L15e (pfam00827)→
env-588	3300001592_10896941	-	243	192	**RNA**→ PRK04243 (PRK04243)Ribosomal_L15e (pfam00827)→
env-589	2227094145	-	323	269	**RNA**→ ←hypo
env-590	2227164185	+	1201	1252	**RNA**→ ←hypo

env-591	2226969280	+	1286	1337	**RNA**→ RPL15A (COG1632)PRK04243 (PRK04243)→
env-592	2227071043	+	381	432	**RNA**→ PRK04243 (PRK04243)Ribosomal_L15e (pfam00827)→
env-593	IMNBL1_c0001816	-	461	410	**RNA**→ RPL15A (COG1632)PRK04243 (PRK04243)→
env-594	JGI20163J15578_10187233	-	825	772	**RNA**→ PRK04243 (PRK04243)Ribosomal_L15e (pfam00827)→
env-595	JGI996J12353_1000527	-	5644	5593	**RNA**→ PRK04243 (PRK04243)Ribosomal_L15e (pfam00827)→
					PRK14555 (PRK14555)RNA_binding (pfam01877)→ RPP1 (COG1603)PRK00912 (PRK00912)→
env-596	2227035939	+	2727	2778	**RNA**→ RPL15A (COG1632)PRK04243 (PRK04243)→
env-597	2227538537	-	5296	5245	**RNA**→ PRK04243 (PRK04243)Ribosomal_L15e (pfam00827)→
					PRK14555 (PRK14555)RNA_binding (pfam01877)→ RPP1 (COG1603)PRK00912 (PRK00912)→
env-598	IMNBL1_c0047771	-	55	4	**RNA**→
env-599	2226957925	-	288	237	**RNA**→ RPL15A (COG1632)PRK04243 (PRK04243)→
env-600	IMNBL1_c0018460	-	1848	1797	**RNA**→ PRK04243 (PRK04243)Ribosomal_L15e (pfam00827)→
					PRK14555 (PRK14555)RNA_binding (pfam01877)→ RPP1 (COG1603)PRK00912 (PRK00912)→
env-601	JGI994J12354_10008059	+	6285	6336	**RNA**→ PRK04243 (PRK04243)Ribosomal_L15e (pfam00827)→
					PRK14555 (PRK14555)RNA_binding (pfam01877)→ RPP1 (COG1603)PRK00912 (PRK00912)→
env-602	2227477430	-	52	1	**RNA**→
env-603	2227211080	-	136	82	**RNA**→
env-604	2227517699	+	3271	3325	**RNA**→ hypo→
env-605	IMNBL1_c0279633	+	244	298	**RNA**→ PRK04243 (PRK04243)Ribosomal_L15e (pfam00827)→
env-606	JGI994J12354_10081286	-	57	3	**RNA**→ ←hypo
env-607	2227618780	-	56	2	**RNA**→
env-608	IMNBL1_c0051091	+	1242	1296	**RNA**→ hypo→
env-609	JGI994J12354_10078750	-	57	3	**RNA**→
env-610	IMNBL1_c0049435	+	1175	1227	**RNA**→ PRK04243 (PRK04243)Ribosomal_L15e (pfam00827)→
env-611	2227139717	-	365	311	**RNA**→ ←hypo
env-612	JGI994J12354_10115912	-	1015	961	**RNA**→ PRK04243 (PRK04243)Ribosomal_L15e (pfam00827)→
					PRK14555 (PRK14555)RNA_binding (pfam01877)→
env-613	IMNBL1_c0291723	+	33	87	**RNA**→ RPL15A (COG1632)PRK04243 (PRK04243)→
env-614	IMNBL1_c0045419	+	1265	1319	**RNA**→ PRK04243 (PRK04243)Ribosomal_L15e (pfam00827)→
env-615	JGI994J12354_10079817	-	170	116	**RNA**→ PRK04243 (PRK04243)Ribosomal_L15e (pfam00827)→
env-616	3300000594_10398	+	31	76	**RNA**→ PRK04243 (PRK04243)Ribosomal_L15e (pfam00827)→
					PRK14555 (PRK14555)RNA_binding (pfam01877)→ PRK00912 (PRK00912)RNase_P_p30 (pfam01876)→
Mco-1-1	NC_017034.1	+	356660	356705	**RNA**→ PRK04243 (PRK04243)Ribosomal_L15e (pfam00827)→
					PRK14555 (PRK14555)RNA_binding (pfam01877)→ RPP1 (COG1603)PRK00912 (PRK00912)→
					BcsA (COG1215)glyco_like_mftF (TIGR04283)→
env-617	SL_5KL_010_SED_10351708	+	176	228	**RNA**→
env-618	SL_8KL_010_SED_10426921	+	67	119	**RNA**→ PRK04243 (PRK04243)Ribosomal_L15e (pfam00827)→
Mps-1-1	NC_018876.1	+	2608031	2608076	**RNA**→ PRK04243 (PRK04243)Ribosomal_L15e (pfam00827)→ COG1325 (COG1325)PRK14555 (PRK14555)
					RPP1 (COG1603)PRK00912 (PRK00912)→ PRK03717 (PRK03717)RNase_P_Rpp14 (pfam01900)→
					PRK03996 (PRK03996)proteasome_alpha_archeal (cd03756)→ Sdo1 (COG1500)PRK13760 (PRK13760)→
					Rrp4 (COG1097)PRK04163 (PRK04163)Pup (pfam05639)→
					RNase_PH_archRRP41 (cd11366)PRK03983 (PRK03983)→
					RNase_PH_archRRP42 (cd11365)PRK04282 (PRK04282)→ rpl37ae (PRK03976)Ribosomal_L37ae (pfam01780)
					RPC10 (COG1996)rpoP (PRK00398)→
env-619	JGI25321J50212_10071092	-	517	472	**RNA**→ RPL15A (COG1632)PRK04243 (PRK04243)→
env-620	3300001567_10059738	+	145	190	**RNA**→ PRK04243 (PRK04243)Ribosomal_L15e (pfam00827)→ COG1325 (COG1325)PRK14555 (PRK14555)
Mti-1-1	NZ_AZAJ01000001.1	-	571920	571858	**RNA**→ PRK04243 (PRK04243)Ribosomal_L15e (pfam00827)→
					PRK14555 (PRK14555)RNA_binding (pfam01877)→ PRK00912 (PRK00912)RNase_P_p30 (pfam01876)→
					PRK03717 (PRK03717)POP5 (COG1369)→ PRK03996 (PRK03996)proteasome_alpha_archeal (cd03756)→
					Sdo1 (COG1500)PRK13760 (PRK13760)→ Rrp4 (COG1097)PRK04163 (PRK04163)→
					RNase_PH_archRRP41 (cd11366)PRK03983 (PRK03983)→
					RNase_PH_archRRP42 (cd11365)PRK04282 (PRK04282)→ rpl37ae (PRK03976)Ribosomal_L37ae (pfam01780)
					RPC10 (COG1996)rpoP (PRK00398)→
env-621	2231757710	+	31	79	**RNA**→ PRK04243 (PRK04243)Ribosomal_L15e (pfam00827)→

Mba-2-1	NZ_CP009517.1	-	1228703	1228652	**RNA**→ PRK04243 (PRK04243)Ribosomal_L15e (pfam00827)→ COG1325 (COG1325)PRK14555 (PRK14555) RPP1 (COG1603)PRK00912 (PRK00912)→ PRK03717 (PRK03717)POP5 (COG1369)→
env-622	B4_c_Cons7318	+	381	432	**RNA**→ PRK04243 (PRK04243)Ribosomal_L15e (pfam00827)→
env-623	JGI20225J20221_1000854	-	92739	92688	**RNA**→ PRK04243 (PRK04243)Ribosomal_L15e (pfam00827)→ COG1325 (COG1325)PRK14555 (PRK14555) RPP1 (COG1603)PRK00912 (PRK00912)→ PRK03717 (PRK03717)POP5 (COG1369)→
env-624	3300000507_111820	-	641	590	**RNA**→ PRK04243 (PRK04243)Ribosomal_L15e (pfam00827)→
env-625	JGI24714J26587_10012885	-	2658	2606	**RNA**→ PRK04243 (PRK04243)Ribosomal_L15e (pfam00827)→ COG1325 (COG1325)PRK14555 (PRK14555) RPP1 (COG1603)PRK00912 (PRK00912)→ PRK03717 (PRK03717)POP5 (COG1369)→
env-626	JGI24712J26585_10034019	-	1360	1308	**RNA**→ PRK04243 (PRK04243)Ribosomal_L15e (pfam00827)→ COG1325 (COG1325)PRK14555 (PRK14555) RPP1 (COG1603)PRK00912 (PRK00912)→
env-627	JGI24711J26586_10062120	+	435	487	**RNA**→ PRK04243 (PRK04243)Ribosomal_L15e (pfam00827)→
Mho-2-1	NZ_CP009516.1	-	3291833	3291782	**RNA**→ PRK04243 (PRK04243)Ribosomal_L15e (pfam00827)→ COG1325 (COG1325)PRK14555 (PRK14555) RPP1 (COG1603)PRK00912 (PRK00912)→ PRK03717 (PRK03717)POP5 (COG1369)→
env-628	JGI24731J21663_1002020	-	12930	12879	**RNA**→ PRK04243 (PRK04243)Ribosomal_L15e (pfam00827)→ COG1325 (COG1325)PRK14555 (PRK14555) RPP1 (COG1603)PRK00912 (PRK00912)→ PRK03717 (PRK03717)POP5 (COG1369)→
env-629	JGI24731J21663_1031455	-	306	255	**RNA**→ RPL15A (COG1632)PRK04243 (PRK04243)→
env-630	JGI12419J13241_1124316	+	56	108	**RNA**→ RPL15A (COG1632)PRK04243 (PRK04243)→
Mth-1-1	NZ_CP009501.1	-	381745	381693	**RNA**→ PRK04243 (PRK04243)Ribosomal_L15e (pfam00827)→ COG1325 (COG1325)PRK14555 (PRK14555) RPP1 (COG1603)PRK00912 (PRK00912)→ PRK03717 (PRK03717)POP5 (COG1369)→
Msp-13-1	NZ_CP009524.1	-	1406720	1406669	**RNA**→ PRK04243 (PRK04243)Ribosomal_L15e (pfam00827)→ COG1325 (COG1325)PRK14555 (PRK14555) RPP1 (COG1603)PRK00912 (PRK00912)→ PRK03717 (PRK03717)POP5 (COG1369)→
Mva-1-1	NZ_CP009520.1	-	1438747	1438696	**RNA**→ COG4585 (COG4585)→ PRK04243 (PRK04243)Ribosomal_L15e (pfam00827)→ COG1325 (COG1325)PRK14555 (PRK14555)→ RPP1 (COG1603)PRK00912 (PRK00912)→ PRK03717 (PRK03717)POP5 (COG1369)→
Mba-4-1	NZ_CP009528.1	-	2670750	2670699	**RNA**→ PRK04243 (PRK04243)Ribosomal_L15e (pfam00827)→ COG1325 (COG1325)PRK14555 (PRK14555) RPP1 (COG1603)PRK00912 (PRK00912)→ PRK03717 (PRK03717)POP5 (COG1369)→
Mba-1-1	NZ_CP009530.1	+	476717	476768	**RNA**→ PRK04243 (PRK04243)Ribosomal_L15e (pfam00827)→ COG1325 (COG1325)PRK14555 (PRK14555) RPP1 (COG1603)PRK00912 (PRK00912)→ PRK03717 (PRK03717)POP5 (COG1369)→
Mba-3-1	NZ_CP008746.1	+	468339	468390	**RNA**→ PRK04243 (PRK04243)Ribosomal_L15e (pfam00827)→ COG1325 (COG1325)PRK14555 (PRK14555) RPP1 (COG1603)PRK00912 (PRK00912)→ PRK03717 (PRK03717)POP5 (COG1369)→
env-631	BMHB3a_c34562	+	135	186	**RNA**→
env-632	JGI2065J20421_1016166	+	872	923	**RNA**→ PRK04243 (PRK04243)Ribosomal_L15e (pfam00827)→ PRK14555 (PRK14555)RNA_binding (pfam01877)→
env-633	B3_all_c_Cons125890	+	461	512	**RNA**→ PRK04243 (PRK04243)Ribosomal_L15e (pfam00827)→
Mla-1-1	NZ_CP009515.1	+	2312125	2312176	**RNA**→ PRK04243 (PRK04243)Ribosomal_L15e (pfam00827)→ COG1325 (COG1325)PRK14555 (PRK14555) RPP1 (COG1603)PRK00912 (PRK00912)→ PRK03717 (PRK03717)POP5 (COG1369)→
Mba-5-1	NC_007355.1	+	3156160	3156211	**RNA**→ PRK04243 (PRK04243)Ribosomal_L15e (pfam00827)→ COG1325 (COG1325)PRK14555 (PRK14555) RPP1 (COG1603)PRK00912 (PRK00912)→ PRK03717 (PRK03717)POP5 (COG1369)→
Mba-6-1	NZ_CP009526.1	-	2909575	2909524	**RNA**→ PRK04243 (PRK04243)Ribosomal_L15e (pfam00827)→ COG1325 (COG1325)PRK14555 (PRK14555) RPP1 (COG1603)PRK00912 (PRK00912)→ PRK03717 (PRK03717)POP5 (COG1369)→
Msp-7-1	NZ_JJQY01000019.1	-	28722	28671	**RNA**→ PRK04243 (PRK04243)Ribosomal_L15e (pfam00827)→ COG1325 (COG1325)PRK14555 (PRK14555) RPP1 (COG1603)PRK00912 (PRK00912)→ PRK03717 (PRK03717)POP5 (COG1369)→
Msp-8-1	NZ_JJOV01000053.1	-	61527	61476	**RNA**→ PRK04243 (PRK04243)Ribosomal_L15e (pfam00827)→ COG1325 (COG1325)PRK14555 (PRK14555) RPP1 (COG1603)PRK00912 (PRK00912)→ PRK03717 (PRK03717)POP5 (COG1369)→
Msp-10-1	NZ_JJOX01000057.1	-	29319	29268	**RNA**→ PRK04243 (PRK04243)Ribosomal_L15e (pfam00827)→ COG1325 (COG1325)PRK14555 (PRK14555) RPP1 (COG1603)PRK00912 (PRK00912)→ PRK03717 (PRK03717)POP5 (COG1369)→
Msp-11-1	NZ_JJOY01000041.1	-	29252	29201	**RNA**→ PRK04243 (PRK04243)Ribosomal_L15e (pfam00827)→ COG1325 (COG1325)PRK14555 (PRK14555) RPP1 (COG1603)PRK00912 (PRK00912)→ PRK03717 (PRK03717)POP5 (COG1369)→
Msp-6-1	NZ_JJQL01000038.1	-	47137	47086	**RNA**→ PRK04243 (PRK04243)Ribosomal_L15e (pfam00827)→ COG1325 (COG1325)PRK14555 (PRK14555) RPP1 (COG1603)PRK00912 (PRK00912)→ PRK03717 (PRK03717)POP5 (COG1369)→
Msp-12-1	NZ_JJOZ01000046.1	-	28637	28586	**RNA**→ PRK04243 (PRK04243)Ribosomal_L15e (pfam00827)→ COG1325 (COG1325)PRK14555 (PRK14555) RPP1 (COG1603)PRK00912 (PRK00912)→ PRK03717 (PRK03717)POP5 (COG1369)→
Msp-9-1	NZ_JJOW01000294.1	-	20540	20489	**RNA**→ PRK04243 (PRK04243)Ribosomal_L15e (pfam00827)→ COG1325 (COG1325)PRK14555 (PRK14555) RPP1 (COG1603)PRK00912 (PRK00912)→ PRK03717 (PRK03717)POP5 (COG1369)→
Msp-16-1	NZ_CP009503.1	+	3735833	3735884	**RNA**→ PRK04243 (PRK04243)Ribosomal_L15e (pfam00827)→ COG1325 (COG1325)PRK14555 (PRK14555) RPP1 (COG1603)PRK00912 (PRK00912)→ PRK03717 (PRK03717)POP5 (COG1369)→
Msp-15-1	NZ_CP009504.1	-	2456027	2455976	**RNA**→ PRK04243 (PRK04243)Ribosomal_L15e (pfam00827)→ COG1325 (COG1325)PRK14555 (PRK14555) RPP1 (COG1603)PRK00912 (PRK00912)→ PRK03717 (PRK03717)POP5 (COG1369)→

env-634	JGI24731J21663_1041287	+	802	854	**RNA**→ ←hypo
Mso-1-1	NZ_JQLR01000001.1	+	2442416	2442467	**RNA**→ PRK04243 (PRK04243)Ribosomal_L15e (pfam00827)→ COG1325 (COG1325)PRK14555 (PRK14555)<br>RPP1 (COG1603)PRK00912 (PRK00912)→ PRK03717 (PRK03717)POP5 (COG1369)→
Mma-5-1	NC_003901.1	+	3119311	3119362	**RNA**→ PRK04243 (PRK04243)Ribosomal_L15e (pfam00827)→ COG1325 (COG1325)PRK14555 (PRK14555)<br>RPP1 (COG1603)PRK00912 (PRK00912)→ PRK03717 (PRK03717)POP5 (COG1369)→
Mma-6-1	NZ_CP009513.1	+	3747266	3747317	**RNA**→ PRK04243 (PRK04243)Ribosomal_L15e (pfam00827)→ COG1325 (COG1325)PRK14555 (PRK14555)<br>RPP1 (COG1603)PRK00912 (PRK00912)→ PRK03717 (PRK03717)POP5 (COG1369)→
Mma-8-1	NZ_CP009511.1	-	2504533	2504482	**RNA**→ PRK04243 (PRK04243)Ribosomal_L15e (pfam00827)→ COG1325 (COG1325)PRK14555 (PRK14555)<br>RPP1 (COG1603)PRK00912 (PRK00912)→ PRK03717 (PRK03717)POP5 (COG1369)→
Mma-7-1	NZ_CP009512.1	-	2625933	2625882	**RNA**→ PRK04243 (PRK04243)Ribosomal_L15e (pfam00827)→ COG1325 (COG1325)PRK14555 (PRK14555)<br>RPP1 (COG1603)PRK00912 (PRK00912)→ PRK03717 (PRK03717)POP5 (COG1369)→
Mma-3-1	NZ_JJPB01000050.1	+	11936	11987	**RNA**→ PRK04243 (PRK04243)Ribosomal_L15e (pfam00827)→ COG1325 (COG1325)PRK14555 (PRK14555)<br>RPP1 (COG1603)PRK00912 (PRK00912)→ PRK03717 (PRK03717)POP5 (COG1369)→
Mma-3-2	NZ_JJQI01000092.1	-	16421	16370	**RNA**→ PRK04243 (PRK04243)Ribosomal_L15e (pfam00827)→ COG1325 (COG1325)PRK14555 (PRK14555)<br>RPP1 (COG1603)PRK00912 (PRK00912)→ PRK03717 (PRK03717)POP5 (COG1369)→
Mma-3-3	NZ_JJQC01000141.1	-	42369	42318	**RNA**→ PRK04243 (PRK04243)Ribosomal_L15e (pfam00827)→ COG1325 (COG1325)PRK14555 (PRK14555)<br>RPP1 (COG1603)PRK00912 (PRK00912)→ PRK03717 (PRK03717)POP5 (COG1369)→
Mma-3-4	NZ_JJPX01000042.1	+	11441	11492	**RNA**→ PRK04243 (PRK04243)Ribosomal_L15e (pfam00827)→ COG1325 (COG1325)PRK14555 (PRK14555)<br>RPP1 (COG1603)PRK00912 (PRK00912)→ PRK03717 (PRK03717)POP5 (COG1369)→
Mma-3-5	NZ_JJQA01000093.1	-	42369	42318	**RNA**→ PRK04243 (PRK04243)Ribosomal_L15e (pfam00827)→ COG1325 (COG1325)PRK14555 (PRK14555)<br>RPP1 (COG1603)PRK00912 (PRK00912)→ PRK03717 (PRK03717)POP5 (COG1369)→
Mma-3-6	NZ_JJPN01000151.1	+	11194	11245	**RNA**→ PRK04243 (PRK04243)Ribosomal_L15e (pfam00827)→ COG1325 (COG1325)PRK14555 (PRK14555)<br>RPP1 (COG1603)PRK00912 (PRK00912)→ PRK03717 (PRK03717)POP5 (COG1369)→
Mma-3-7	NZ_JJOS01000070.1	+	20296	20347	**RNA**→ PRK04243 (PRK04243)Ribosomal_L15e (pfam00827)→ COG1325 (COG1325)PRK14555 (PRK14555)<br>RPP1 (COG1603)PRK00912 (PRK00912)→ PRK03717 (PRK03717)POP5 (COG1369)→
Mma-3-8	NZ_JJPG01000087.1	+	11936	11987	**RNA**→ PRK04243 (PRK04243)Ribosomal_L15e (pfam00827)→ COG1325 (COG1325)PRK14555 (PRK14555)<br>RPP1 (COG1603)PRK00912 (PRK00912)→ PRK03717 (PRK03717)POP5 (COG1369)→
Mma-3-9	NZ_JJPP01000085.1	+	31236	31287	**RNA**→ PRK04243 (PRK04243)Ribosomal_L15e (pfam00827)→ COG1325 (COG1325)PRK14555 (PRK14555)<br>RPP1 (COG1603)PRK00912 (PRK00912)→ PRK03717 (PRK03717)POP5 (COG1369)→
Mma-3-10	NZ_JJQB01000132.1	-	42369	42318	**RNA**→ PRK04243 (PRK04243)Ribosomal_L15e (pfam00827)→ COG1325 (COG1325)PRK14555 (PRK14555)<br>RPP1 (COG1603)PRK00912 (PRK00912)→ PRK03717 (PRK03717)POP5 (COG1369)→
Mma-3-11	NZ_JJQG01000125.1	+	110287	110338	**RNA**→ PRK04243 (PRK04243)Ribosomal_L15e (pfam00827)→ COG1325 (COG1325)PRK14555 (PRK14555)<br>RPP1 (COG1603)PRK00912 (PRK00912)→ PRK03717 (PRK03717)POP5 (COG1369)→
Mma-3-12	NZ_JJPF01000029.1	-	32163	32112	**RNA**→ PRK04243 (PRK04243)Ribosomal_L15e (pfam00827)→ COG1325 (COG1325)PRK14555 (PRK14555)<br>RPP1 (COG1603)PRK00912 (PRK00912)→ PRK03717 (PRK03717)POP5 (COG1369)→
Mma-3-13	NZ_JJQR01000115.1	+	47927	47978	**RNA**→ PRK04243 (PRK04243)Ribosomal_L15e (pfam00827)→ COG1325 (COG1325)PRK14555 (PRK14555)<br>RPP1 (COG1603)PRK00912 (PRK00912)→ PRK03717 (PRK03717)POP5 (COG1369)→
Mma-3-14	NZ_JJQW01000009.1	-	43611	43560	**RNA**→ PRK04243 (PRK04243)Ribosomal_L15e (pfam00827)→ COG1325 (COG1325)PRK14555 (PRK14555)<br>RPP1 (COG1603)PRK00912 (PRK00912)→ PRK03717 (PRK03717)POP5 (COG1369)→
Mma-3-15	NZ_JJPO01000048.1	+	11937	11988	**RNA**→ PRK04243 (PRK04243)Ribosomal_L15e (pfam00827)→ COG1325 (COG1325)PRK14555 (PRK14555)<br>RPP1 (COG1603)PRK00912 (PRK00912)→ PRK03717 (PRK03717)POP5 (COG1369)→
Mma-3-16	NZ_JJQX01000051.1	+	8135	8186	**RNA**→ PRK04243 (PRK04243)Ribosomal_L15e (pfam00827)→ COG1325 (COG1325)PRK14555 (PRK14555)<br>RPP1 (COG1603)PRK00912 (PRK00912)→ PRK03717 (PRK03717)POP5 (COG1369)→
Mma-3-17	NZ_JJQE01000025.1	-	33596	33545	**RNA**→ PRK04243 (PRK04243)Ribosomal_L15e (pfam00827)→ COG1325 (COG1325)PRK14555 (PRK14555)<br>RPP1 (COG1603)PRK00912 (PRK00912)→ PRK03717 (PRK03717)POP5 (COG1369)→
Mma-3-18	NZ_JJRA01000090.1	-	35290	35239	**RNA**→ PRK04243 (PRK04243)Ribosomal_L15e (pfam00827)→ COG1325 (COG1325)PRK14555 (PRK14555)<br>RPP1 (COG1603)PRK00912 (PRK00912)→ PRK03717 (PRK03717)POP5 (COG1369)→
Mma-3-19	NZ_JJRB01000023.1	-	35592	35541	**RNA**→ PRK04243 (PRK04243)Ribosomal_L15e (pfam00827)→ COG1325 (COG1325)PRK14555 (PRK14555)<br>RPP1 (COG1603)PRK00912 (PRK00912)→ PRK03717 (PRK03717)POP5 (COG1369)→
Mma-3-20	NZ_JJOT01000028.1	+	142723	142774	**RNA**→ PRK04243 (PRK04243)Ribosomal_L15e (pfam00827)→ COG1325 (COG1325)PRK14555 (PRK14555)<br>RPP1 (COG1603)PRK00912 (PRK00912)→ PRK03717 (PRK03717)POP5 (COG1369)→
Mma-3-21	NZ_JJQS01000135.1	-	34925	34874	**RNA**→ PRK04243 (PRK04243)Ribosomal_L15e (pfam00827)→ COG1325 (COG1325)PRK14555 (PRK14555)<br>RPP1 (COG1603)PRK00912 (PRK00912)→ PRK03717 (PRK03717)POP5 (COG1369)→
Mma-3-22	NZ_JJPC01000138.1	+	16225	16276	**RNA**→ PRK04243 (PRK04243)Ribosomal_L15e (pfam00827)→ COG1325 (COG1325)PRK14555 (PRK14555)<br>RPP1 (COG1603)PRK00912 (PRK00912)→ PRK03717 (PRK03717)POP5 (COG1369)→
Mma-3-23	NZ_JJOU01000016.1	-	43691	43640	**RNA**→ PRK04243 (PRK04243)Ribosomal_L15e (pfam00827)→ COG1325 (COG1325)PRK14555 (PRK14555)<br>RPP1 (COG1603)PRK00912 (PRK00912)→ PRK03717 (PRK03717)POP5 (COG1369)→

Mma-3-24	NZ_JJPS01000107.1	-	6231	6180	**RNA**→ PRK04243 (PRK04243)Ribosomal_L15e (pfam00827)→ COG1325 (COG1325)PRK14555 (PRK14555) RPP1 (COG1603)PRK00912 (PRK00912)→ PRK03717 (PRK03717)POP5 (COG1369)→
Mma-3-25	NZ_JJPY01000103.1	-	42289	42238	**RNA**→ PRK04243 (PRK04243)Ribosomal_L15e (pfam00827)→ COG1325 (COG1325)PRK14555 (PRK14555) RPP1 (COG1603)PRK00912 (PRK00912)→ PRK03717 (PRK03717)POP5 (COG1369)→
Mma-3-26	NZ_JJOR01000097.1	+	48050	48101	**RNA**→ PRK04243 (PRK04243)Ribosomal_L15e (pfam00827)→ COG1325 (COG1325)PRK14555 (PRK14555) RPP1 (COG1603)PRK00912 (PRK00912)→ PRK03717 (PRK03717)POP5 (COG1369)→
Mma-3-27	NZ_JJQD01000017.1	+	37333	37384	**RNA**→ PRK04243 (PRK04243)Ribosomal_L15e (pfam00827)→ COG1325 (COG1325)PRK14555 (PRK14555) RPP1 (COG1603)PRK00912 (PRK00912)→ PRK03717 (PRK03717)POP5 (COG1369)→
Mma-3-28	NZ_JJQM01000066.1	+	47345	47396	**RNA**→ PRK04243 (PRK04243)Ribosomal_L15e (pfam00827)→ COG1325 (COG1325)PRK14555 (PRK14555) RPP1 (COG1603)PRK00912 (PRK00912)→ PRK03717 (PRK03717)POP5 (COG1369)→
Mma-3-29	NZ_JJPA01000100.1	-	34395	34344	**RNA**→ PRK04243 (PRK04243)Ribosomal_L15e (pfam00827)→ COG1325 (COG1325)PRK14555 (PRK14555) RPP1 (COG1603)PRK00912 (PRK00912)→ PRK03717 (PRK03717)POP5 (COG1369)→
Mma-3-30	NZ_JJPR01000056.1	-	42079	42028	**RNA**→ PRK04243 (PRK04243)Ribosomal_L15e (pfam00827)→ COG1325 (COG1325)PRK14555 (PRK14555) RPP1 (COG1603)PRK00912 (PRK00912)→ PRK03717 (PRK03717)POP5 (COG1369)→
Mma-3-31	NZ_JJPH01000044.1	-	32163	32112	**RNA**→ PRK04243 (PRK04243)Ribosomal_L15e (pfam00827)→ COG1325 (COG1325)PRK14555 (PRK14555) RPP1 (COG1603)PRK00912 (PRK00912)→ PRK03717 (PRK03717)POP5 (COG1369)→
Mma-3-32	NZ_JJPT01000015.1	-	33938	33887	**RNA**→ PRK04243 (PRK04243)Ribosomal_L15e (pfam00827)→ COG1325 (COG1325)PRK14555 (PRK14555) RPP1 (COG1603)PRK00912 (PRK00912)→ PRK03717 (PRK03717)POP5 (COG1369)→
Mma-3-33	NZ_JJPW01000129.1	-	35393	35342	**RNA**→ PRK04243 (PRK04243)Ribosomal_L15e (pfam00827)→ COG1325 (COG1325)PRK14555 (PRK14555) RPP1 (COG1603)PRK00912 (PRK00912)→ PRK03717 (PRK03717)POP5 (COG1369)→
Mma-3-34	NZ_JJQQ01000052.1	-	35254	35203	**RNA**→ PRK04243 (PRK04243)Ribosomal_L15e (pfam00827)→ COG1325 (COG1325)PRK14555 (PRK14555) RPP1 (COG1603)PRK00912 (PRK00912)→ PRK03717 (PRK03717)POP5 (COG1369)→
Mma-3-35	NZ_JJQZ01000044.1	-	45155	45104	**RNA**→ PRK04243 (PRK04243)Ribosomal_L15e (pfam00827)→ COG1325 (COG1325)PRK14555 (PRK14555) RPP1 (COG1603)PRK00912 (PRK00912)→ PRK03717 (PRK03717)POP5 (COG1369)→
Mma-3-36	NZ_JJPI01000027.1	+	21733	21784	**RNA**→ PRK04243 (PRK04243)Ribosomal_L15e (pfam00827)→ COG1325 (COG1325)PRK14555 (PRK14555) RPP1 (COG1603)PRK00912 (PRK00912)→ PRK03717 (PRK03717)POP5 (COG1369)→
Mma-3-37	NZ_JJPQ01000027.1	+	11208	11259	**RNA**→ PRK04243 (PRK04243)Ribosomal_L15e (pfam00827)→ COG1325 (COG1325)PRK14555 (PRK14555) RPP1 (COG1603)PRK00912 (PRK00912)→ PRK03717 (PRK03717)POP5 (COG1369)→
Mma-3-38	NZ_JJQH01000164.1	-	45155	45104	**RNA**→ PRK04243 (PRK04243)Ribosomal_L15e (pfam00827)→ COG1325 (COG1325)PRK14555 (PRK14555) RPP1 (COG1603)PRK00912 (PRK00912)→ PRK03717 (PRK03717)POP5 (COG1369)→
Mma-3-39	NZ_JJPD01000048.1	+	11958	12009	**RNA**→ PRK04243 (PRK04243)Ribosomal_L15e (pfam00827)→ COG1325 (COG1325)PRK14555 (PRK14555) RPP1 (COG1603)PRK00912 (PRK00912)→ PRK03717 (PRK03717)POP5 (COG1369)→
Mma-3-40	NZ_JJPJ01000102.1	-	35123	35072	**RNA**→ PRK04243 (PRK04243)Ribosomal_L15e (pfam00827)→ COG1325 (COG1325)PRK14555 (PRK14555) RPP1 (COG1603)PRK00912 (PRK00912)→ PRK03717 (PRK03717)POP5 (COG1369)→
Mma-3-41	NZ_JJPK01000008.1	+	160849	160900	**RNA**→ PRK04243 (PRK04243)Ribosomal_L15e (pfam00827)→ COG1325 (COG1325)PRK14555 (PRK14555) RPP1 (COG1603)PRK00912 (PRK00912)→ PRK03717 (PRK03717)POP5 (COG1369)→
Mma-3-42	NZ_JJPZ01000008.1	+	11958	12009	**RNA**→ PRK04243 (PRK04243)Ribosomal_L15e (pfam00827)→ COG1325 (COG1325)PRK14555 (PRK14555) RPP1 (COG1603)PRK00912 (PRK00912)→ PRK03717 (PRK03717)POP5 (COG1369)→
Mma-3-43	NZ_JJQF01000099.1	-	13178	13127	**RNA**→ PRK04243 (PRK04243)Ribosomal_L15e (pfam00827)→ COG1325 (COG1325)PRK14555 (PRK14555) RPP1 (COG1603)PRK00912 (PRK00912)→ PRK03717 (PRK03717)POP5 (COG1369)→
Mma-3-44	NZ_JJPL01000149.1	+	76874	76925	**RNA**→ PRK04243 (PRK04243)Ribosomal_L15e (pfam00827)→ COG1325 (COG1325)PRK14555 (PRK14555) RPP1 (COG1603)PRK00912 (PRK00912)→ PRK03717 (PRK03717)POP5 (COG1369)→
Mma-3-45	NZ_JJPV01000067.1	-	142282	142231	**RNA**→ PRK04243 (PRK04243)Ribosomal_L15e (pfam00827)→ COG1325 (COG1325)PRK14555 (PRK14555) RPP1 (COG1603)PRK00912 (PRK00912)→ PRK03717 (PRK03717)POP5 (COG1369)→
Mma-3-46	NZ_JJPM01000067.1	+	8095	8146	**RNA**→ PRK04243 (PRK04243)Ribosomal_L15e (pfam00827)→ COG1325 (COG1325)PRK14555 (PRK14555) RPP1 (COG1603)PRK00912 (PRK00912)→ PRK03717 (PRK03717)POP5 (COG1369)→
Mma-3-47	NZ_JJPU01000129.1	-	54102	54051	**RNA**→ PRK04243 (PRK04243)Ribosomal_L15e (pfam00827)→ COG1325 (COG1325)PRK14555 (PRK14555) RPP1 (COG1603)PRK00912 (PRK00912)→ PRK03717 (PRK03717)POP5 (COG1369)→
Mma-3-48	NZ_JJQU01000233.1	+	21011	21062	**RNA**→ PRK04243 (PRK04243)Ribosomal_L15e (pfam00827)→ COG1325 (COG1325)PRK14555 (PRK14555) RPP1 (COG1603)PRK00912 (PRK00912)→ PRK03717 (PRK03717)POP5 (COG1369)→
Mma-3-49	NZ_JJPE01000037.1	-	32163	32112	**RNA**→ PRK04243 (PRK04243)Ribosomal_L15e (pfam00827)→ COG1325 (COG1325)PRK14555 (PRK14555) RPP1 (COG1603)PRK00912 (PRK00912)→ PRK03717 (PRK03717)POP5 (COG1369)→
Mma-3-50	NZ_JJQK01000170.1	+	11911	11962	**RNA**→ PRK04243 (PRK04243)Ribosomal_L15e (pfam00827)→ COG1325 (COG1325)PRK14555 (PRK14555) RPP1 (COG1603)PRK00912 (PRK00912)→ PRK03717 (PRK03717)POP5 (COG1369)→
Mma-3-51	NZ_JJQO01000228.1	+	47525	47576	**RNA**→ PRK04243 (PRK04243)Ribosomal_L15e (pfam00827)→ COG1325 (COG1325)PRK14555 (PRK14555) RPP1 (COG1603)PRK00912 (PRK00912)→ PRK03717 (PRK03717)POP5 (COG1369)→
Mma-3-52	NZ_JJQJ01000089.1	-	22779	22728	**RNA**→ PRK04243 (PRK04243)Ribosomal_L15e (pfam00827)→ COG1325 (COG1325)PRK14555 (PRK14555) RPP1 (COG1603)PRK00912 (PRK00912)→ PRK03717 (PRK03717)POP5 (COG1369)→

Mma-3-53	NZ_JJQP01000172.1	+	39888	39939	**RNA**→ PRK04243 (PRK04243)Ribosomal_L15e (pfam00827)→ COG1325 (COG1325)PRK14555 (PRK14555) RPP1 (COG1603)PRK00912 (PRK00912)→ PRK03717 (PRK03717)POP5 (COG1369)→
Mma-3-54	NZ_JJQN01000109.1	+	11937	11988	**RNA**→ PRK04243 (PRK04243)Ribosomal_L15e (pfam00827)→ COG1325 (COG1325)PRK14555 (PRK14555) RPP1 (COG1603)PRK00912 (PRK00912)→ PRK03717 (PRK03717)POP5 (COG1369)→
Mma-3-55	NZ_JJQT01000136.1	-	6188	6137	**RNA**→ PRK04243 (PRK04243)Ribosomal_L15e (pfam00827)→ COG1325 (COG1325)PRK14555 (PRK14555) RPP1 (COG1603)PRK00912 (PRK00912)→ PRK03717 (PRK03717)POP5 (COG1369)→
Mma-3-56	NZ_JJQV01000102.1	-	35394	35343	**RNA**→ PRK04243 (PRK04243)Ribosomal_L15e (pfam00827)→ COG1325 (COG1325)PRK14555 (PRK14555) RPP1 (COG1603)PRK00912 (PRK00912)→ PRK03717 (PRK03717)POP5 (COG1369)→
Mma-9-1	NZ_CP009509.1	+	2909485	2909536	**RNA**→ PRK04243 (PRK04243)Ribosomal_L15e (pfam00827)→ COG1325 (COG1325)PRK14555 (PRK14555) RPP1 (COG1603)PRK00912 (PRK00912)→ PRK03717 (PRK03717)POP5 (COG1369)→
Mma-4-1	NZ_CP009514.1	+	1235377	1235428	**RNA**→ PRK04243 (PRK04243)Ribosomal_L15e (pfam00827)→ COG1325 (COG1325)PRK14555 (PRK14555) RPP1 (COG1603)PRK00912 (PRK00912)→ PRK03717 (PRK03717)POP5 (COG1369)→
Mac-1-1	NC_003552.1	-	2189802	2189751	**RNA**→ PRK04243 (PRK04243)Ribosomal_L15e (pfam00827)→ COG1325 (COG1325)PRK14555 (PRK14555) RPP1 (COG1603)PRK00912 (PRK00912)→ PRK03717 (PRK03717)POP5 (COG1369)→
Msi-2-1	NZ_CP009506.1	-	3147028	3146977	**RNA**→ PRK04243 (PRK04243)Ribosomal_L15e (pfam00827)→ COG1325 (COG1325)PRK14555 (PRK14555) RPP1 (COG1603)PRK00912 (PRK00912)→ PRK03717 (PRK03717)POP5 (COG1369)→
env-635	WSSedB2T_c104918	+	74	125	**RNA**→ RPL15A (COG1632)PRK04243 (PRK04243)→
Msi-1-1	NZ_CP009508.1	-	2149794	2149743	**RNA**→ PRK04243 (PRK04243)Ribosomal_L15e (pfam00827)→ COG1325 (COG1325)PRK14555 (PRK14555) RPP1 (COG1603)PRK00912 (PRK00912)→ PRK03717 (PRK03717)POP5 (COG1369)→
env-636	M940CN_1003840	-	2106	2055	**RNA**→ PRK04243 (PRK04243)Ribosomal_L15e (pfam00827)→ COG1325 (COG1325)PRK14555 (PRK14555) RPP1 (COG1603)PRK00912 (PRK00912)→ PRK03717 (PRK03717)RNase_P_Rpp14 (pfam01900)→
Msp-14-1	NZ_CP009505.1	-	2602304	2602254	**RNA**→ ←hypo
env-637	3300001567_10002926	-	2776	2730	**RNA**→ PRK04243 (PRK04243)Ribosomal_L15e (pfam00827)→ COG1325 (COG1325)PRK14555 (PRK14555) RPP1 (COG1603)PRK00912 (PRK00912)→ PRK03717 (PRK03717)POP5 (COG1369)→ PRK03996 (PRK03996)proteasome_alpha (cd01911)→
env-638	DHTCA2_contig15787	+	370	416	**RNA**→
env-639	AMWB01080572.1	+	1326	1372	**RNA**→ PRK04243 (PRK04243)Ribosomal_L15e (pfam00827)→
env-640	3300001580_10062933	+	412	459	**RNA**→ PRK04243 (PRK04243)Ribosomal_L15e (pfam00827)→ PRK14555 (PRK14555)RNA_binding (pfam01877)→ RPP1 (COG1603)PRK00912 (PRK00912)→
Mho-1-1	NC_019977.1	+	1869779	1869826	**RNA**→ PRK04243 (PRK04243)Ribosomal_L15e (pfam00827)→ PRK14555 (PRK14555)RNA_binding (pfam01877)→ RPP1 (COG1603)PRK00912 (PRK00912)→ PRK03717 (PRK03717)POP5 (COG1369)→ PRK03996 (PRK03996)proteasome_alpha_archeal (cd03756)→ Sdo1 (COG1500)PRK13760 (PRK13760)→ Rrp4 (COG1097)PRK04163 (PRK04163)→ RNase_PH_archRRP41 (cd11366)PRK03983 (PRK03983)PRK05658 (PRK05658)→ RNase_PH_archRRP42 (cd11365)PRK04282 (PRK04282)→ rpl37ae (PRK03976)Ribosomal_L37ae (pfam01780)→ RPC10 (COG1996)rpoP (PRK00398)→ Brix (smart00879)IMP4 (COG2136)→ Pcc1 (COG2892)Pcc1 (pfam09341)→ gimC_beta (TIGR02338)GimC (COG1382)→ PRK05427 (PRK05427)DHH (pfam01368)→ PaaK (COG1541)AMP-binding_C_2 (pfam14535)→
env-641	AMWB01126899.1	+	489	536	**RNA**→ PRK04243 (PRK04243)Ribosomal_L15e (pfam00827)→ PRK14555 (PRK14555)RNA_binding (pfam01877)→ PRK00912 (PRK00912)RNase_P_p30 (pfam01876)→ PRK03717 (PRK03717)POP5 (COG1369)→ PRK03996 (PRK03996)proteasome_alpha_archeal (cd03756)→
Mbu-1-1	NC_007955.1	+	176502	176552	**RNA**→ PRK04243 (PRK04243)Ribosomal_L15e (pfam00827)→ PRK14555 (PRK14555)RNA_binding (pfam01877)→ PRK00912 (PRK00912)RNase_P_p30 (pfam01876)→ PRK03717 (PRK03717)RNase_P_Rpp14 (pfam01900)→
env-642	AZIB01003834.1	+	91	141	**RNA**→ PRK04243 (PRK04243)Ribosomal_L15e (pfam00827)→ PRK14555 (PRK14555)RNA_binding (pfam01877)→
Mme-2-1	NZ_CP009518.1	+	794999	795052	**RNA**→ PRK04243 (PRK04243)Ribosomal_L15e (pfam00827)→ COG1325 (COG1325)PRK14555 (PRK14555)→ RPP1 (COG1603)PRK00912 (PRK00912)→ PRK03717 (PRK03717)RNase_P_Rpp14 (pfam01900)→
Mme-1-1	NZ_JRHO01000010.1	+	86627	86680	**RNA**→ PRK04243 (PRK04243)Ribosomal_L15e (pfam00827)→ COG1325 (COG1325)PRK14555 (PRK14555)→ PRK00912 (PRK00912)RNase_P_p30 (pfam01876)→ PRK03717 (PRK03717)POP5 (COG1369)→
Msh-1-1	NZ_JONQ01000008.1	+	45804	45849	**RNA**→ RPL15A (COG1632)PRK04243 (PRK04243)→ PRK14555 (PRK14555)RNA_binding (pfam01877)→ RPP1 (COG1603)PRK00912 (PRK00912)→
env-643	HCF12C_96986	-	646	605	**RNA**→ PRK04243 (PRK04243)Ribosomal_L15e (pfam00827)→
env-644	AUXO016695324.1	-	1274	1233	**RNA**→ PRK04243 (PRK04243)Ribosomal_L15e (pfam00827)→
env-645	JGI12104J13512_1011404	-	2541	2501	**RNA**→ PRK04243 (PRK04243)Ribosomal_L15e (pfam00827)→ PRK14555 (PRK14555)RNA_binding (pfam01877)→ RPP1 (COG1603)PRK00912 (PRK00912)→ PRK03717 (PRK03717)POP5 (COG1369)→

env-646	3300001580_10676249	+	109	149	RNA→ PRK04243 (PRK04243)Ribosomal_L15e (pfam00827)→
env-647	08_07_CONTROL_DNA_scaffold24236_7	-	6049	6007	RNA→ PRK04243 (PRK04243)Ribosomal_L15e (pfam00827)→ PRK14555 (PRK14555)RNA_binding (pfam01877)→
env-648	08_07_XOS_DNA_scaffold878_1	+	2078	2120	RNA→ PRK04243 (PRK04243)Ribosomal_L15e (pfam00827)→
env-649	O8_19_POLYDEXT_DNA_scaffold34267_3	-	167	125	RNA→ PRK04243 (PRK04243)Ribosomal_L15e (pfam00827)→
env-650	JGI994J12354_10354549	-	293	251	RNA→ PRK04243 (PRK04243)Ribosomal_L15e (pfam00827)→
env-651	JGI24713J26584_10000181	-	19302	19259	RNA→ PRK04243 (PRK04243)Ribosomal_L15e (pfam00827)→ COG1325 (COG1325)PRK14555 (PRK14555)→ RPP1 (COG1603)PRK00912 (PRK00912)→ PRK03717 (PRK03717)POP5 (COG1369)→ PRK03996 (PRK03996)proteasome_alpha_archeal (cd03756)→ Sdo1 (COG1500)PRK13760 (PRK13760)→ Rrp4 (COG1097)PRK04163 (PRK04163)→ RNase_PH_archRRP41 (cd11366)PRK03983 (PRK03983)→ RNase_PH_archRRP42 (cd11365)PRK04282 (PRK04282)→
env-652	JGI24714J26587_10000218	+	19819	19862	RNA→ PRK04243 (PRK04243)Ribosomal_L15e (pfam00827)→ COG1325 (COG1325)PRK14555 (PRK14555)→ RPP1 (COG1603)PRK00912 (PRK00912)→ PRK03717 (PRK03717)POP5 (COG1369)→ PRK03996 (PRK03996)proteasome_alpha_archeal (cd03756)→ Sdo1 (COG1500)PRK13760 (PRK13760)→ Rrp4 (COG1097)PRK04163 (PRK04163)→ RNase_PH_archRRP41 (cd11366)PRK03983 (PRK03983)→ RNase_PH_archRRP42 (cd11365)PRK04282 (PRK04282)→
env-653	JGI24504J29685_1000272	-	114106	114063	RNA→ PRK04243 (PRK04243)Ribosomal_L15e (pfam00827)→ COG1325 (COG1325)PRK14555 (PRK14555)→ RPP1 (COG1603)PRK00912 (PRK00912)→ PRK03717 (PRK03717)POP5 (COG1369)→ PRK03996 (PRK03996)proteasome_alpha_archeal (cd03756)→ Sdo1 (COG1500)PRK13760 (PRK13760)→ Rrp4 (COG1097)PRK04163 (PRK04163)→ RNase_PH_archRRP41 (cd11366)PRK03983 (PRK03983)→ RNase_PH_archRRP42 (cd11365)PRK04282 (PRK04282)→
env-654	JGI24504J29685_1000273	+	20634	20677	RNA→ PRK04243 (PRK04243)Ribosomal_L15e (pfam00827)→ COG1325 (COG1325)PRK14555 (PRK14555)→ RPP1 (COG1603)PRK00912 (PRK00912)→ PRK03717 (PRK03717)POP5 (COG1369)→ PRK03996 (PRK03996)proteasome_alpha_archeal (cd03756)→ Sdo1 (COG1500)PRK13760 (PRK13760)→ Rrp4 (COG1097)PRK04163 (PRK04163)→ RNase_PH_archRRP41 (cd11366)PRK03983 (PRK03983)→ RNase_PH_archRRP42 (cd11365)PRK04282 (PRK04282)→
env-655	JGI24505J29691_1005987	+	2006	2049	RNA→ PRK04243 (PRK04243)Ribosomal_L15e (pfam00827)→ COG1325 (COG1325)PRK14555 (PRK14555)→ RPP1 (COG1603)PRK00912 (PRK00912)→ PRK03717 (PRK03717)POP5 (COG1369)→ PRK03996 (PRK03996)proteasome_alpha_archeal (cd03756)→ Sdo1 (COG1500)PRK13760 (PRK13760)→ Rrp4 (COG1097)PRK04163 (PRK04163)→ RNase_PH_archRRP41 (cd11366)PRK03983 (PRK03983)→ RNase_PH_archRRP42 (cd11365)PRK04282 (PRK04282)→
env-656	RUMENNODE_3862964_1	+	19057	19104	RNA→ hypo→ PRK04243 (PRK04243)Ribosomal_L15e (pfam00827)→ PRK14555 (PRK14555)RNA_binding (pfam01877)→ hypo→
env-657	AUXO010246320.1	+	2947	2993	RNA→
env-658	RUMENNODE_3957055_1	+	1849	1895	RNA→ PRK04243 (PRK04243)Ribosomal_L15e (pfam00827)→ PRK14555 (PRK14555)RNA_binding (pfam01877)→
Mve-1-1	NZ_JQJK01000008.1	+	40819	40860	RNA→ PRK04243 (PRK04243)Ribosomal_L15e (pfam00827)→ PRK14555 (PRK14555)RNA_binding (pfam01877)→ RPP1 (COG1603)PRK00912 (PRK00912)→ PRK03717 (PRK03717)RNase_P_Rpp14 (pfam01900)→
Mar-1-1	NZ_JQKN01000001.1	+	40819	40860	RNA→ PRK04243 (PRK04243)Ribosomal_L15e (pfam00827)→ PRK14555 (PRK14555)RNA_binding (pfam01877)→ RPP1 (COG1603)PRK00912 (PRK00912)→ PRK03717 (PRK03717)RNase_P_Rpp14 (pfam01900)→
env-659	JGI24731J21663_1049964	-	360	319	RNA→ RPL15A (COG1632)PRK04243 (PRK04243)→
env-660	B3_all_c_Cons195859	+	487	528	RNA→ RPL15A (COG1632)PRK04243 (PRK04243)→
Msp-4-1	NC_015574.1	+	503676	503718	RNA→ PRK04243 (PRK04243)Ribosomal_L15e (pfam00827)→ PRK14555 (PRK14555)RNA_binding (pfam01877)→ RPP1 (COG1603)PRK00912 (PRK00912)→
env-661	2014734625	+	11972	12018	RNA→ PRK04243 (PRK04243)Ribosomal_L15e (pfam00827)→ PRK14555 (PRK14555)RNA_binding (pfam01877)→ RPP1 (COG1603)PRK00912 (PRK00912)→
env-662	ANASMECb_4085297_C2853	+	11972	12018	RNA→ PRK04243 (PRK04243)Ribosomal_L15e (pfam00827)→ PRK14555 (PRK14555)RNA_binding (pfam01877)→ RPP1 (COG1603)PRK00912 (PRK00912)→
env-663	3300001580_10131017	-	51	5	RNA→
env-664	3300001592_10138106	-	985	939	RNA→ PRK04243 (PRK04243)Ribosomal_L15e (pfam00827)→ PRK14555 (PRK14555)RNA_binding (pfam01877)→
env-665	JGI24712J26585_10348213	-	47	4	RNA→ hypo→

env-666	JGI24709J26583_10002022	-	8087	8044	**RNA**→ PRK04243 (PRK04243)Ribosomal_L15e (pfam00827)→ PRK14555 (PRK14555)RNA_binding (pfam01877)→ RPP1 (COG1603)PRK00912 (PRK00912)→ PRK03717 (PRK03717)RNase_P_Rpp14 (pfam01900)→ PRK03996 (PRK03996)proteasome_alpha_archeal (cd03756)→ Sdo1 (COG1500)PRK13760 (PRK13760)→ Rrp4 (COG1097)PRK04163 (PRK04163)→ RNase_PH_archRRP41 (cd11366)PRK03983 (PRK03983)→ RNase_PH_archRRP42 (cd11365)PRK04282 (PRK04282)→
env-667	JGI24710J26742_10000204	+	18022	18065	**RNA**→ PRK04243 (PRK04243)Ribosomal_L15e (pfam00827)→ PRK14555 (PRK14555)RNA_binding (pfam01877)→ RPP1 (COG1603)PRK00912 (PRK00912)→ PRK03717 (PRK03717)RNase_P_Rpp14 (pfam01900)→ PRK03996 (PRK03996)proteasome_alpha_archeal (cd03756)→ Sdo1 (COG1500)PRK13760 (PRK13760)→ Rrp4 (COG1097)PRK04163 (PRK04163)→ RNase_PH_archRRP41 (cd11366)PRK03983 (PRK03983)→ RNase_PH_archRRP42 (cd11365)PRK04282 (PRK04282)→
env-668	JGI24500J29687_10014623	+	1789	1832	**RNA**→ hypo→ PRK04243 (PRK04243)Ribosomal_L15e (pfam00827)→ PRK14555 (PRK14555)RNA_binding (pfam01877)→ RPP1 (COG1603)PRK00912 (PRK00912)→ PRK03717 (PRK03717)RNase_P_Rpp14 (pfam01900)→ PRK03996 (PRK03996)proteasome_alpha_archeal (cd03756)→ Sdo1 (COG1500)PRK13760 (PRK13760)→ Rrp4 (COG1097)PRK04163 (PRK04163)→ RNase_PH_archRRP41 (cd11366)PRK03983 (PRK03983)→ RNase_PH_archRRP42 (cd11365)PRK04282 (PRK04282)→
env-669	JGI24501J29690_1010023	+	224	267	**RNA**→ hypo→ PRK04243 (PRK04243)Ribosomal_L15e (pfam00827)→ PRK14555 (PRK14555)RNA_binding (pfam01877)→ RPP1 (COG1603)PRK00912 (PRK00912)→ PRK03717 (PRK03717)RNase_P_Rpp14 (pfam01900)→ PRK03996 (PRK03996)proteasome_alpha_archeal (cd03756)→ Sdo1 (COG1500)PRK13760 (PRK13760)→ Rrp4 (COG1097)PRK04163 (PRK04163)→ RNase_PH_archRRP41 (cd11366)PRK03983 (PRK03983)→ RNase_PH_archRRP42 (cd11365)PRK04282 (PRK04282)→
env-670	JGI24711J26586_10150374	-	270	227	**RNA**→ PRK04243 (PRK04243)Ribosomal_L15e (pfam00827)→
env-671	JGI24731J21663_1027378	-	1115	1069	**RNA**→ PRK04243 (PRK04243)Ribosomal_L15e (pfam00827)→ PRK14555 (PRK14555)RNA_binding (pfam01877)→
env-672	JGI24729J20445_1009801	+	789	835	**RNA**→ PRK04243 (PRK04243)Ribosomal_L15e (pfam00827)→ PRK14555 (PRK14555)RNA_binding (pfam01877)→ RPP1 (COG1603)PRK00912 (PRK00912)→
Mfo-2-1	NZ_AMPO01000006.1	+	4040	4086	**RNA**→ PRK04243 (PRK04243)Ribosomal_L15e (pfam00827)→ PRK04243 (PRK04243)Ribosomal_L15e (pfam00827)→ PRK14555 (PRK14555)RNA_binding (pfam01877)→ PRK14555 (PRK14555)RNA_binding (pfam01877)→ RPP1 (COG1603)PRK00912 (PRK00912)→ RPP1 (COG1603)PRK00912 (PRK00912)→
env-673	JGI20214J14112_1380804	-	244	198	**RNA**→ RPL15A (COG1632)PRK04243 (PRK04243)→
env-674	JGI24731J21663_1005210	+	4307	4353	**RNA**→ PRK04243 (PRK04243)Ribosomal_L15e (pfam00827)→ PRK14555 (PRK14555)RNA_binding (pfam01877)→ RPP1 (COG1603)PRK00912 (PRK00912)→
Msp-2-1	NC_023044.1	+	529281	529327	**RNA**→ PRK04243 (PRK04243)Ribosomal_L15e (pfam00827)→ PRK14555 (PRK14555)RNA_binding (pfam01877)→ RPP1 (COG1603)PRK00912 (PRK00912)→ PRK03717 (PRK03717)RNase_P_Rpp14 (pfam01900)→ PRK03996 (PRK03996)proteasome_alpha_archeal (cd03756)→ Sdo1 (COG1500)PRK13760 (PRK13760)→ Rrp4 (COG1097)PRK04163 (PRK04163)→ RNase_PH_archRRP41 (cd11366)PRK03983 (PRK03983)→ RNase_PH_archRRP42 (cd11365)PRK04282 (PRK04282)→
Mfo-1-1	NZ_CP006933.1	-	1785014	1784968	**RNA**→ PRK04243 (PRK04243)Ribosomal_L15e (pfam00827)→ PRK14555 (PRK14555)RNA_binding (pfam01877)→ RPP1 (COG1603)PRK00912 (PRK00912)→ PRK03717 (PRK03717)POP5 (COG1369)→ PRK03996 (PRK03996)proteasome_alpha_archeal (cd03756)→ Sdo1 (COG1500)PRK13760 (PRK13760)→ Rrp4 (COG1097)PRK04163 (PRK04163)→ RNase_PH_archRRP41 (cd11366)PRK03983 (PRK03983)→ RNase_PH_archRRP42 (cd11365)PRK04282 (PRK04282)→
env-675	PRSSGFe2_Sequence0000019669	+	951	992	**RNA**→ PRK04243 (PRK04243)Ribosomal_L15e (pfam00827)→ PRK14555 (PRK14555)RNA_binding (pfam01877)→
env-676	JGI12104J13512_1023983	-	1071	1030	**RNA**→ PRK04243 (PRK04243)Ribosomal_L15e (pfam00827)→ PRK14555 (PRK14555)RNA_binding (pfam01877)→
env-677	JGI11944J13513_1001397	-	15451	15410	**RNA**→ PRK04243 (PRK04243)Ribosomal_L15e (pfam00827)→ PRK14555 (PRK14555)RNA_binding (pfam01877)→ PRK00912 (PRK00912)RNase_P_p30 (pfam01876)→ PRK03717 (PRK03717)POP5 (COG1369)→
env-678	3300001592_10385652	-	169	128	**RNA**→ PRK04243 (PRK04243)Ribosomal_L15e (pfam00827)→

Msp-1-1	NC_015216.1	-	2225955	2225907	RNA→ PRK04243 (PRK04243)Ribosomal_L15e (pfam00827)→ PRK14555 (PRK14555)RNA_binding (pfam01877)→ RPP1 (COG1603)PRK00912 (PRK00912)→ RPP1 (COG1603)PRK00912 (PRK00912)→ PRK03717 (PRK03717)RNase_P_Rpp14 (pfam01900)→
env-679	JGI2065J20421_1000005	+	287784	287832	RNA→ PRK04243 (PRK04243)Ribosomal_L15e (pfam00827)→ PRK14555 (PRK14555)RNA_binding (pfam01877)→ RPP1 (COG1603)PRK00912 (PRK00912)→
env-680	WSSedB2Ca_1123116	-	192	151	RNA→ PRK04243 (PRK04243)Ribosomal_L15e (pfam00827)→
Msp-3-1	NZ_JQLY01000001.1	+	314798	314839	RNA→ PRK04243 (PRK04243)Ribosomal_L15e (pfam00827)→ PRK14555 (PRK14555)RNA_binding (pfam01877)→ RPP1 (COG1603)PRK00912 (PRK00912)→ PRK03717 (PRK03717)POP5 (COG1369)→
env-681	B3_all_c_Cons30296	+	231	272	RNA→ PRK04243 (PRK04243)Ribosomal_L15e (pfam00827)→
env-682	B3_all_c_Cons42794	+	295	336	RNA→ RPL15A (COG1632)PRK04243 (PRK04243)→
env-683	SL_5KL_010_SED_10126917	-	228	187	RNA→ PRK04243 (PRK04243)Ribosomal_L15e (pfam00827)→
Mor-1-1	NZ_HG796200.1	-	398447	398403	RNA→ PRK04243 (PRK04243)Ribosomal_L15e (pfam00827)→ PRK14555 (PRK14555)RNA_binding (pfam01877)→
Msm-14-1	NZ_AELU01000003.1	-	71106	71058	RNA→ PRK04243 (PRK04243)Ribosomal_L15e (pfam00827)→ PRK14555 (PRK14555)RNA_binding (pfam01877)→ YurZ (COG0599)→
Msm-4-1	NZ_DS996911.1	+	39506	39554	RNA→ PRK04243 (PRK04243)Ribosomal_L15e (pfam00827)→ PRK14555 (PRK14555)RNA_binding (pfam01877)→
env-684	scaffold116291_3_V1.CD-8	-	666	618	RNA→ PRK04243 (PRK04243)Ribosomal_L15e (pfam00827)→
env-685	4491416.3_NODE_1031	+	40283	40331	RNA→ PRK04243 (PRK04243)Ribosomal_L15e (pfam00827)→ PRK14555 (PRK14555)RNA_binding (pfam01877)→ YurZ (COG0599)→
env-686	scaffold101749_2_MH0011	+	6770	6818	RNA→ PRK04243 (PRK04243)Ribosomal_L15e (pfam00827)→ PRK14555 (PRK14555)RNA_binding (pfam01877)→ YurZ (COG0599)→
env-687	scaffold45427_5_O2.UC-12	+	989	1037	RNA→ PRK04243 (PRK04243)Ribosomal_L15e (pfam00827)→
env-688	scaffold45612_1_V1.CD-9	-	1221	1173	RNA→ PRK04243 (PRK04243)Ribosomal_L15e (pfam00827)→ PRK14555 (PRK14555)RNA_binding (pfam01877)→
env-689	scaffold99220_1_V1.UC-8	-	16733	16685	RNA→ PRK04243 (PRK04243)Ribosomal_L15e (pfam00827)→ PRK14555 (PRK14555)RNA_binding (pfam01877)→
env-690	UnmappedStool_Broad_scaffold_1133206	+	660	708	RNA→ PRK04243 (PRK04243)Ribosomal_L15e (pfam00827)→ PRK14555 (PRK14555)RNA_binding (pfam01877)→
env-691	DLM022_scaffold90350_1	+	654	702	RNA→ PRK04243 (PRK04243)Ribosomal_L15e (pfam00827)→ PRK14555 (PRK14555)RNA_binding (pfam01877)→ YurZ (COG0599)→
env-692	ADJS01014849.1	-	6089	6041	RNA→ PRK04243 (PRK04243)Ribosomal_L15e (pfam00827)→ PRK14555 (PRK14555)RNA_binding (pfam01877)→
env-693	SRS014235_C3325073	+	168	216	RNA→ PRK04243 (PRK04243)Ribosomal_L15e (pfam00827)→
env-694	SRS014979_C3300304	-	2323	2275	RNA→ PRK04243 (PRK04243)Ribosomal_L15e (pfam00827)→ PRK14555 (PRK14555)RNA_binding (pfam01877)→
Msm-1-1	NZ_CCXV01000062.1	-	3684	3636	RNA→ PRK04243 (PRK04243)Ribosomal_L15e (pfam00827)→ PRK14555 (PRK14555)RNA_binding (pfam01877)→
Msm-19-1	NZ_AELZ01000004.1	-	93410	93362	RNA→ PRK04243 (PRK04243)Ribosomal_L15e (pfam00827)→ PRK14555 (PRK14555)RNA_binding (pfam01877)→
Msm-11-1	NZ_AELR01000006.1	-	74375	74327	RNA→ PRK04243 (PRK04243)Ribosomal_L15e (pfam00827)→ PRK14555 (PRK14555)RNA_binding (pfam01877)→
Msm-23-1	NZ_AEMD01000013.1	-	30363	30315	RNA→ PRK04243 (PRK04243)Ribosomal_L15e (pfam00827)→ PRK14555 (PRK14555)RNA_binding (pfam01877)→
Msm-12-1	NZ_AELS01000005.1	-	55986	55938	RNA→ PRK04243 (PRK04243)Ribosomal_L15e (pfam00827)→ PRK14555 (PRK14555)RNA_binding (pfam01877)→
Msm-13-1	NZ_AELT01000005.1	-	60811	60763	RNA→ PRK04243 (PRK04243)Ribosomal_L15e (pfam00827)→ PRK14555 (PRK14555)RNA_binding (pfam01877)→
Msm-22-1	NZ_AEMC01000004.1	-	93153	93105	RNA→ PRK04243 (PRK04243)Ribosomal_L15e (pfam00827)→ PRK14555 (PRK14555)RNA_binding (pfam01877)→
Msm-21-1	NZ_AEMB01000003.1	-	93149	93101	RNA→ PRK04243 (PRK04243)Ribosomal_L15e (pfam00827)→ PRK14555 (PRK14555)RNA_binding (pfam01877)→
Msm-20-1	NZ_AEMA01000007.1	-	93143	93095	RNA→ PRK04243 (PRK04243)Ribosomal_L15e (pfam00827)→ PRK14555 (PRK14555)RNA_binding (pfam01877)→
Msm-10-1	NZ_AELQ01000008.1	-	55971	55923	RNA→ PRK04243 (PRK04243)Ribosomal_L15e (pfam00827)→ PRK14555 (PRK14555)RNA_binding (pfam01877)→

Msm-18-1	NZ_AELY01000006.1	-	93416	93368	**RNA**→ PRK04243 (PRK04243)Ribosomal_L15e (pfam00827)→
					PRK14555 (PRK14555)RNA_binding (pfam01877)→
Msm-9-1	NZ_AELO01000021.1	-	3502	3454	**RNA**→ PRK04243 (PRK04243)Ribosomal_L15e (pfam00827)→
					PRK14555 (PRK14555)RNA_binding (pfam01877)→
Msm-5-1	NZ_AEKU01000009.1	-	30411	30363	**RNA**→ PRK04243 (PRK04243)Ribosomal_L15e (pfam00827)→
					PRK14555 (PRK14555)RNA_binding (pfam01877)→
Msm-17-1	NZ_AELX01000007.1	-	93439	93391	**RNA**→ RPL15A (COG1632)PRK04243 (PRK04243)→ hypo→ hypo→
					PRK14555 (PRK14555)RNA_binding (pfam01877)→
Msm-8-1	NZ_AELN01000005.1	-	30375	30327	**RNA**→ PRK04243 (PRK04243)Ribosomal_L15e (pfam00827)→
					PRK14555 (PRK14555)RNA_binding (pfam01877)→
Msm-2-1	NC_009515.1	-	288824	288776	**RNA**→ PRK04243 (PRK04243)Ribosomal_L15e (pfam00827)→
					PRK14555 (PRK14555)RNA_binding (pfam01877)→ YurZ (COG0599)→
Msm-3-1	NZ_GG704759.1	-	1679032	1678984	**RNA**→ PRK04243 (PRK04243)Ribosomal_L15e (pfam00827)→
					PRK14555 (PRK14555)RNA_binding (pfam01877)→
env-695	scaffold43629_1_V1.CD-14	+	589	637	**RNA**→ RPL15A (COG1632)PRK04243 (PRK04243)→
Msm-16-1	NZ_AELW01000005.1	-	70811	70763	**RNA**→ PRK04243 (PRK04243)Ribosomal_L15e (pfam00827)→
					PRK14555 (PRK14555)RNA_binding (pfam01877)→ YurZ (COG0599)→
Msm-15-1	NZ_AELV01000008.1	-	55039	54991	**RNA**→ PRK04243 (PRK04243)Ribosomal_L15e (pfam00827)→
					PRK14555 (PRK14555)RNA_binding (pfam01877)→ YurZ (COG0599)→
Msm-6-1	NZ_AELL01000010.1	-	30405	30357	**RNA**→ PRK04243 (PRK04243)Ribosomal_L15e (pfam00827)→
					PRK14555 (PRK14555)RNA_binding (pfam01877)→
Msm-7-1	NZ_AELM01000013.1	-	30412	30364	**RNA**→ PRK04243 (PRK04243)Ribosomal_L15e (pfam00827)→
					PRK14555 (PRK14555)RNA_binding (pfam01877)→
env-696	JGI26339J46600_10088026	-	219	169	**RNA**→ PRK04243 (PRK04243)Ribosomal_L15e (pfam00827)→
env-697	JGI24730J26740_1120805	+	4	51	**RNA**→ RPL15A (COG1632)PRK04243 (PRK04243)→
env-698	LGVF01127568.1	+	648	697	**RNA**→
env-699	LGOV01033634.1	-	1312	1261	**RNA**→ PRK04243 (PRK04243)Ribosomal_L15e (pfam00827)→
					PRK14555 (PRK14555)RNA_binding (pfam01877)→ PRK00912 (PRK00912)RNase_P_p30 (pfam01876)→
env-700	LGVE01048678.1	-	619	570	**RNA**→ PRK04243 (PRK04243)Ribosomal_L15e (pfam00827)→
env-701	LGVF01407700.1	+	445	496	**RNA**→ RPL15A (COG1632)PRK04243 (PRK04243)→
env-702	LGVE01104961.1	-	973	923	**RNA**→ PRK04243 (PRK04243)Ribosomal_L15e (pfam00827)→
env-703	LGVF01373635.1	-	2320	2270	**RNA**→ PRK04243 (PRK04243)Ribosomal_L15e (pfam00827)→
env-704	LGVE01091035.1	+	101	150	**RNA**→ PRK04243 (PRK04243)Ribosomal_L15e (pfam00827)→
env-705	LGVF01174437.1	-	990	941	**RNA**→ PRK04243 (PRK04243)Ribosomal_L15e (pfam00827)→
env-706	LGVE01094124.1	+	632	681	**RNA**→ PRK04243 (PRK04243)Ribosomal_L15e (pfam00827)→
env-707	JGI24024J18818_10368636	+	225	274	**RNA**→
env-708	LGVF01194351.1	-	1341	1292	**RNA**→ PRK04243 (PRK04243)Ribosomal_L15e (pfam00827)→
env-709	PBDCA2_contig12847	-	760	713	**RNA**→ PRK04243 (PRK04243)Ribosomal_L15e (pfam00827)→
					PRK14555 (PRK14555)RNA_binding (pfam01877)→
env-710	JGI2161J19892_1008470	-	767	717	**RNA**→ RPL15A (COG1632)PRK04243 (PRK04243)→
env-711	JGI2172J19969_10002521	+	546	596	**RNA**→ RPL15A (COG1632)PRK04243 (PRK04243)→
env-712	JGI24422J19971_10970220	+	95	145	**RNA**→ RPL15A (COG1632)PRK04243 (PRK04243)→
env-713	JGI2160J19893_10000003	-	51272	51222	**RNA**→ RPL15A (COG1632)PRK04243 (PRK04243)→
Ngr-1-1	NC_019792.1	-	2417457	2417388	**RNA**→ PRK04243 (PRK04243)Ribosomal_L15e (pfam00827)→
Hti-1-1	NC_021921.1	-	2128296	2128249	**RNA**→ PRK04243 (PRK04243)Ribosomal_L15e (pfam00827)→
Npe-1-1	NC_019962.1	-	782478	782407	**RNA**→ PRK04243 (PRK04243)Ribosomal_L15e (pfam00827)→
Hsp-7-1	NZ_ANPG01000166.1	-	1829	1769	**RNA**→ PRK04243 (PRK04243)Ribosomal_L15e (pfam00827)→
Hlu-1-1	NZ_AOLH01000025.1	+	31699	31759	**RNA**→ PRK04243 (PRK04243)Ribosomal_L15e (pfam00827)→
Hal-1-1	NZ_AOLL01000011.1	+	546240	546300	**RNA**→ PRK04243 (PRK04243)Ribosomal_L15e (pfam00827)→
Hvo-1-1	NC_013967.1	-	495895	495835	**RNA**→ PRK04243 (PRK04243)Ribosomal_L15e (pfam00827)→
Hje-1-1	NC_014297.1	+	2507160	2507224	**RNA**→ PRK04243 (PRK04243)Ribosomal_L15e (pfam00827)→
					ThiF_MoeB_HesA_family (cd00757)adenyl_thiF (TIGR02356)→
Hbo-1-1	NC_014729.1	+	2535983	2536036	**RNA**→ PRK04243 (PRK04243)Ribosomal_L15e (pfam00827)→
Nma-1-1	NC_013922.1	-	988031	987966	**RNA**→ PRK04243 (PRK04243)Ribosomal_L15e (pfam00827)→
Nti-1-1	NZ_KB913017.1	+	2799470	2799545	**RNA**→ PRK04243 (PRK04243)Ribosomal_L15e (pfam00827)→

4.3 Conserved domains

Conserved domains found in protein-coding genes listed in Section 4.2 are shown below, with the first sentence in their description from the Conserved Domain Database (if any). Conserved domains associated with more than one eL15-Euryarchaeota RNA are assigned a color, while others are shown in gray. The number in parentheses after the colored domain name is the number of occurrences in Section 4.2.

cd00075 (1) Histidine kinase-like ATPases; This family includes several ATP-binding proteins for example: histidine kinase, DNA gyrase B, topoisomerases, heat shock protein HSP90, phytochrome-like ATPases and DNA mismatch repair proteins
cd00267 (1) ATP-binding cassette transporter nucleotide-binding domain.
cd00293 (4) Usp: Universal stress protein family.
cd00757 (26) ThiF_MoeB_HesA.
cd01066 (1) A family including aminopeptidase P, aminopeptidase M, and prolidase.
cd01088 (1) Methionine Aminopeptidase 2.
cd01911 (1) proteasome alpha subunit.
cd02153 (2) The tRNA binding domain is also known as the Myf domain in literature.
cd03024 (2) DsbA family, FrnE subfamily; FrnE is a DsbA-like protein containing a CXXC motif.
cd03756 (76) proteasome_alpha_archeal.
cd06067 (3) Endopeptidases belonging to membrane-bound hydrogen evolving hydrogenase group. [NiFe]
cd11365 (19) RRP42 subunit of archaeal exosome.
cd11366 (19) RRP41 subunit of archaeal exosome.
cd11386 (1) Methyl-accepting chemotaxis protein (MCP), signaling domain.
COG0249 (3) DNA mismatch repair ATPase MutS [Replication, recombination and repair]
COG0317 (1) (p)ppGpp synthase/hydrolase, HD superfamily [Signal transduction mechanisms, Transcription]
COG0323 (1) DNA mismatch repair ATPase MutL [Replication, recombination and repair]
COG0599 (7) Uncharacterized conserved protein YurZ, alkylhydroperoxidase/carboxymuconolactone decarboxylase family [General function prediction only]
COG0638 (2) 20S proteasome, alpha and beta subunits [Posttranslational modification, protein turnover, chaperones]
COG0840 (5) Methyl-accepting chemotaxis protein [Cell motility, Signal transduction mechanisms]
COG0864 (3) Metal-responsive transcriptional regulator, contains CopG/Arc/MetJ DNA-binding domain [Transcription]
COG1097 (19) Exosome complex RNA-binding protein Rrp4, contains S1 and KH domains [Translation, ribosomal structure and biogenesis]
COG1145 (1) Ferredoxin [Energy production and conversion]
COG1146 (6) NAD-dependent dihydropyrimidine dehydrogenase, PreA subunit [Nucleotide transport and metabolism]
COG1215 (1) Glycosyltransferase, catalytic subunit of cellulose synthase and poly-beta-1,6-N-acetylglucosamine synthase [Cell motility]
COG1325 (106) Exosome subunit, RNA binding protein with dsRBD fold [Translation, ribosomal structure and biogenesis]
COG1369 (108) RNase P/RNase MRP subunit POP5 [Translation, ribosomal structure and biogenesis]
COG1382 (10) Prefoldin, chaperonin cofactor [Posttranslational modification, protein turnover, chaperones]
COG1500 (76) Ribosome maturation protein Sdo1 [Translation, ribosomal structure and biogenesis]
COG1541 (2) Phenylacetate-coenzyme A ligase PaaK, adenylate-forming domain family [Coenzyme transport and metabolism]
COG1603 (223) RNase P/RNase MRP subunit p30 [Translation, ribosomal structure and biogenesis]
COG1632 (147) Ribosomal protein L15E [Translation, ribosomal structure and biogenesis]
COG1741 (2) Redox-sensitive bicupin YhaK, pirin superfamily [General function prediction only]
COG1996 (35) DNA-directed RNA polymerase, subunit RPC12/RpoP, contains C4-type Zn-finger [Transcription]
COG1997 (1) Ribosomal protein L37AE/L43A [Translation, ribosomal structure and biogenesis]
COG2136 (11) rRNA maturation protein Rpf1, contains Brix/IMP4 (anticodon-binding) domain [Translation, ribosomal structure and biogenesis]
COG2761 (6) Predicted dithiol-disulfide isomerase, DsbA family [Posttranslational modification, protein turnover, chaperones]
COG2892 (25) tRNA threonylcarbamoyladenosine modification (KEOPS) complex, Pcc1 subunit [Translation, ribosomal structure and biogenesis]
COG3153 (1) Predicted N-acetyltransferase YhbS [General function prediction only]
COG3609 (6) Transcriptional regulator, contains Arc/MetJ-type RHH (ribbon-helix-helix) DNA-binding domain [Transcription]
COG4352 (80) Ribosomal protein L13E [Translation, ribosomal structure and biogenesis]
COG4585 (1) Signal transduction histidine kinase [Signal transduction mechanisms]
pfam00672 (6) HAMP domain.
pfam00827 (780) Ribosomal L15.
pfam01119 (1) DNA mismatch repair protein, C-terminal domain.
pfam01172 (1) Shwachman-Bodian-Diamond syndrome (SBDS) protein.
pfam01226 (1) Formate/nitrite transporter.
pfam01294 (1) Ribosomal protein L13e.
pfam01368 (2) DHH family.
pfam01451 (1) Low molecular weight phosphotyrosine protein phosphatase.
pfam01595 (1) Domain of unknown function DUF21.
pfam01624 (1) MutS domain I.
pfam01780 (58) Ribosomal L37ae protein family.
pfam01876 (70) RNase P subunit p30.
pfam01877 (163) RNA binding.
pfam01900 (136) Rpp14/Pop5 family.
pfam01920 (6) Prefoldin subunit.
pfam02592 (6) Putative vitamin uptake transporter.
pfam02771 (1) Acyl-CoA dehydrogenase, N-terminal domain.
pfam04087 (1) Domain of unknown function (DUF389).
pfam05048 (3) Periplasmic copper-binding protein (NosD).

pfam05639 (1) Pup-like protein. [EQ]
pfam06736 (3) Protein of unknown function (DUF1211).
pfam07705 (3) CARDB.
pfam08676 (1) MutL C terminal dimerisation domain.
pfam08753 (3) NikR C terminal nickel binding domain.
pfam09341 (23) Transcription factor Pcc1.
pfam09377 (1) SBDS protein C-terminal domain.
pfam12838 (5) 4Fe-4S dicluster domain.
pfam13187 (1) 4Fe-4S dicluster domain.
pfam13229 (3) Right handed beta helix region.
pfam13370 (1) 4Fe-4S single cluster domain of Ferredoxin I. [4Fe-4S]
pfam13462 (8) Thioredoxin.
pfam13508 (1) Acetyltransferase (GNAT) domain.
pfam14535 (2) AMP-binding enzyme C-terminal domain.
pfam16116 (3) Domain of unknown function (DUF4832).
PRK00095 (1) DNA mismatch repair protein; Reviewed
PRK00398 (35) DNA-directed RNA polymerase subunit P; Provisional
PRK00912 (296) ribonuclease P protein component 3; Provisional
PRK00933 (11) ribosomal biogenesis protein; Validated
PRK03717 (241) ribonuclease P protein component 2; Provisional
PRK03892 (2) ribonuclease P protein component 3; Provisional
PRK03976 (59) 50S ribosomal protein L37Ae; Reviewed
PRK03983 (19) exosome complex exonuclease Rrp41; Provisional
PRK03996 (77) proteasome subunit alpha; Provisional
PRK04163 (19) exosome complex RNA-binding protein Rrp4; Provisional
PRK04243 (927) 50S ribosomal protein L15e; Validated
PRK04282 (19) exosome complex RNA-binding protein Rrp42; Provisional
PRK04460 (6) nickel responsive regulator; Provisional
PRK05399 (1) DNA mismatch repair protein MutS; Provisional
PRK05427 (1) putative manganese-dependent inorganic pyrophosphatase; Provisional
PRK05658 (1) RNA polymerase sigma factor RpoD; Validated
PRK08671 (2) methionine aminopeptidase; Provisional
PRK08762 (10) molybdopterin biosynthesis protein MoeB; Validated
PRK09343 (3) prefoldin subunit beta; Provisional
PRK10089 (2) tRNA-binding protein; Provisional
PRK10935 (2) nitrate/nitrite sensor protein NarQ; Provisional
PRK12277 (81) 50S ribosomal protein L13e; Provisional
PRK13760 (76) putative RNA-associated protein; Provisional
PRK14538 (1) putative bifunctional signaling protein/50S ribosomal protein L9; Provisional
PRK14555 (267) hypothetical protein; Provisional
PRK14887 (2) KEOPS complex Pcc1-like subunit; Provisional
PRK14960 (3) DNA polymerase III subunits gamma and tau; Provisional
PRK15005 (4) universal stress protein F; Provisional
smart00283 (6) Methyl-accepting chemotaxis-like domains (chemotaxis sensory transducer).
smart00382 (1) ATPases associated with a variety of cellular activities.
smart00387 (1) Histidine kinase-like ATPases.
smart00533 (2) DNA-binding domain of DNA mismatch repair MUTS family.
smart00534 (2) ATPase domain of DNA mismatch repair MUTS family.
smart00879 (3) The Brix domain is found in a number of eukaryotic proteins.
smart00948 (2) Proteasome subunit A N-terminal signature Add an annotation.
TIGR00072 (3) hydrogenase maturation protease.
TIGR00341 (1) TIGR00341 family protein. [Hypothetical proteins, Conserved]
TIGR00691 (1) (p)ppGpp synthetase, RelA/SpoT family. [Cellular processes, Adaptations to atypical conditions]
TIGR00697 (6) conserved hypothetical integral membrane protein. [Hypothetical proteins, Conserved]
TIGR02338 (13) prefoldin, beta subunit, archaeal.
TIGR02356 (16) thiazole biosynthesis adenylyltransferase ThiF, E. [Biosynthesis of cofactors, prosthetic groups, and carriers, Thiamine]
TIGR02689 (1) arsenate reductase, glutathione/glutaredoxin type. [Cellular processes, Detoxification]
TIGR03520 (1) gliding motility-associated protein GldE.
TIGR04283 (1) transferase 2, rSAM/selenodomain-associated. [Unknown function, Enzymes of unknown specificity]

4.4 Multiple-sequence alignment

Each eL15-Euryarchaeota RNA is denoted by an abbreviation (like "Eco-1-1") that refers to a taxonomy given in Section 4.1. The alignment may include sequences containing the RNA motif, as well as flanking sequence. The eL15-Euryarchaeota RNA itself is denoted by the line underneath marked 5′ and 3′ on either end. Nucleotides in flanking sequences (i.e., not part of the motif) are written in gray letters. Stems of predicted rho-independent transcription terminators, if any, are shaded yellow. (Note: terminator predictions have not been analyzed manually, and many are likely to be false positives. Terminator predictions are those of the RNIE software.) Nucleotides predicted to function (as DNA) as transcription-factor binding sites, if any, are shaded in green. (But note: these predictions are manually annotated, so they might be under-predicted.) Annotated start codons, if any, are shaded green. (Note: start codons are frequently misannotated, especially in environmental samples.) Nucleotides proposed to basepair as part of the consensus structure are shaded in color when they comprise Watson-Crick or G-U pairs. Otherwise they are shaded gray. Conserved stems are also indicated at the bottom of the alignment by angle brackets, where matching < and > denote base-paired columns. Below these angle brackets, the symbol "2" denotes base pairs exhibiting covariation according to the statistically well-founded R-scape method. "1" denotes base pairs exhibiting covariation according to R2R's simplistic method. "0" denotes base pairs that are not observed to mutate and "?" denotes base pairs that have a significant frequency of non-canonical nucleotides for Watson-Crick or G-U pairs ($> 5\%$). Below these base pair annotation is the consensus sequence: "R" = "A" or "G", "Y" = "C" or "U", red nucleotides: nucleotide identity conserved more than 97% of the time, black nucleotides: 90%, gray nucleotides: 75%,

red circle (•): nucleotide is present 97% of the time, black circle (•): 90%, gray circle (•): 75%, white circle (◦): 50%. All percentages of sequences just described (e.g. 97% conserved) assume that sequences have been weighted by the GSC algorithm implemented by the Infernal software package.

Duplicate sequences: the following putative homologs are not shown in the alignment because their sequences are identical to a homolog already shown: Hac-1-2, Hal-1-1, Hdi-1-1, Hdi-2-1, Hez-1-1, Hha-1-2, Hhi-1-1, Hhi-2-1, Hla-1-2, Hla-3-1, Hlu-1-1, Hme-1-2, Hpa-1-2, Hsa-3-1, Hsp-1-1, Hsp-4-1, Hsp-5-1, Hte-2-1, Hvo-1-1, Hwa-2-1, Mar-1-1, Mba-1-1, Mba-3-1, Mba-6-1, Mho-1-1, Mhu-1-1, Mma-3-10, Mma-3-11, Mma-3-12, Mma-3-13, Mma-3-14, Mma-3-15, Mma-3-16, Mma-3-17, Mma-3-18, Mma-3-19, Mma-3-2, Mma-3-20, Mma-3-21, Mma-3-22, Mma-3-23, Mma-3-24, Mma-3-25, Mma-3-26, Mma-3-27, Mma-3-28, Mma-3-29, Mma-3-3, Mma-3-30, Mma-3-31, Mma-3-32, Mma-3-33, Mma-3-34, Mma-3-35, Mma-3-36, Mma-3-37, Mma-3-38, Mma-3-39, Mma-3-4, Mma-3-40, Mma-3-41, Mma-3-42, Mma-3-43, Mma-3-44, Mma-3-45, Mma-3-46, Mma-3-47, Mma-3-48, Mma-3-49, Mma-3-5, Mma-3-50, Mma-3-51, Mma-3-52, Mma-3-53, Mma-3-54, Mma-3-55, Mma-3-56, Mma-3-6, Mma-3-7, Mma-3-8, Mma-3-9, Mma-4-1, Mma-5-1, Mma-6-1, Mma-7-1, Mma-8-1, Mma-9-1, Msm-1-1, Msm-10-1, Msm-11-1, Msm-12-1, Msm-13-1, Msm-15-1, Msm-17-1, Msm-18-1, Msm-19-1, Msm-2-1, Msm-20-1, Msm-21-1, Msm-22-1, Msm-23-1, Msm-3-1, Msm-5-1, Msm-7-1, Msm-8-1, Msm-9-1, Msp-10-1, Msp-11-1, Msp-12-1, Msp-15-1, Msp-6-1, Msp-8-1, Msp-9-1, Nal-1-1, Nsp-1-1, Nsp-2-1, Pfu-1-1, env-100, env-103, env-106, env-107, env-108, env-109, env-111, env-113, env-118, env-120, env-123, env-124, env-127, env-128, env-13, env-131, env-132, env-133, env-134, env-143, env-150, env-151, env-154, env-155, env-157, env-163, env-165, env-166, env-167, env-168, env-17, env-171, env-18, env-190, env-191, env-198, env-20, env-200, env-204, env-205, env-209, env-21, env-211, env-222, env-225, env-24, env-240, env-241, env-242, env-252, env-257, env-258, env-259, env-26, env-260, env-261, env-263, env-264, env-265, env-272, env-275, env-279, env-280, env-282, env-285, env-288, env-292, env-294, env-300, env-303, env-305, env-306, env-307, env-313, env-314, env-315, env-316, env-320, env-335, env-337, env-340, env-342, env-343, env-345, env-346, env-347, env-350, env-351, env-352, env-355, env-357, env-36, env-361, env-368, env-37, env-372, env-373, env-374, env-38, env-389, env-391, env-393, env-394, env-395, env-396, env-397, env-398, env-399, env-4, env-400, env-401, env-403, env-404, env-405, env-407, env-409, env-41, env-411, env-415, env-417, env-419, env-421, env-423, env-426, env-430, env-431, env-434, env-435, env-436, env-437, env-44, env-445, env-448, env-451, env-452, env-456, env-458, env-459, env-46, env-464, env-468, env-470, env-471, env-478, env-482, env-483, env-493, env-494, env-498, env-501, env-504, env-510, env-518, env-519, env-520, env-521, env-523, env-524, env-526, env-527, env-533, env-54, env-548, env-549, env-550, env-551, env-554, env-555, env-556, env-557, env-558, env-56, env-562, env-566, env-569, env-572, env-573, env-58, env-582, env-583, env-584, env-586, env-59, env-592, env-593, env-60, env-600, env-601, env-614, env-615, env-618, env-62, env-626, env-627, env-632, env-648, env-649, env-652, env-653, env-654, env-655, env-662, env-666, env-667, env-668, env-669, env-677, env-678, env-68, env-687, env-688, env-689, env-69, env-690, env-691, env-692, env-693, env-694, env-703, env-705, env-71, env-711, env-712, env-713, env-72, env-75, env-76, env-8, env-81, env-82, env-85, env-88, env-96, env-97, har-1-1

alignment positions 1 · · · 180

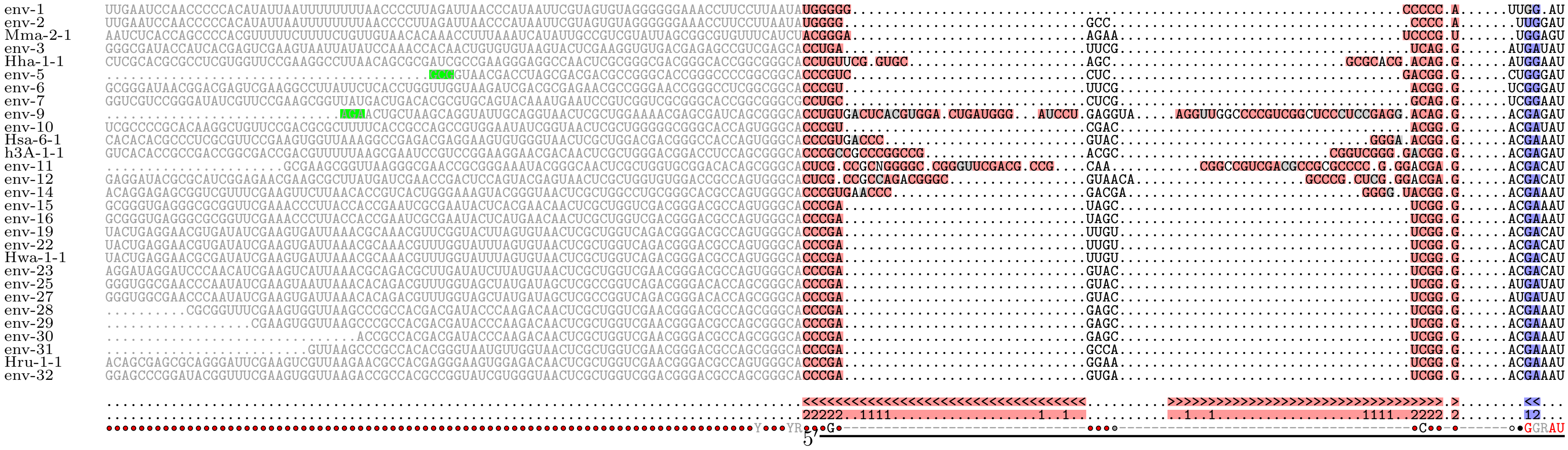

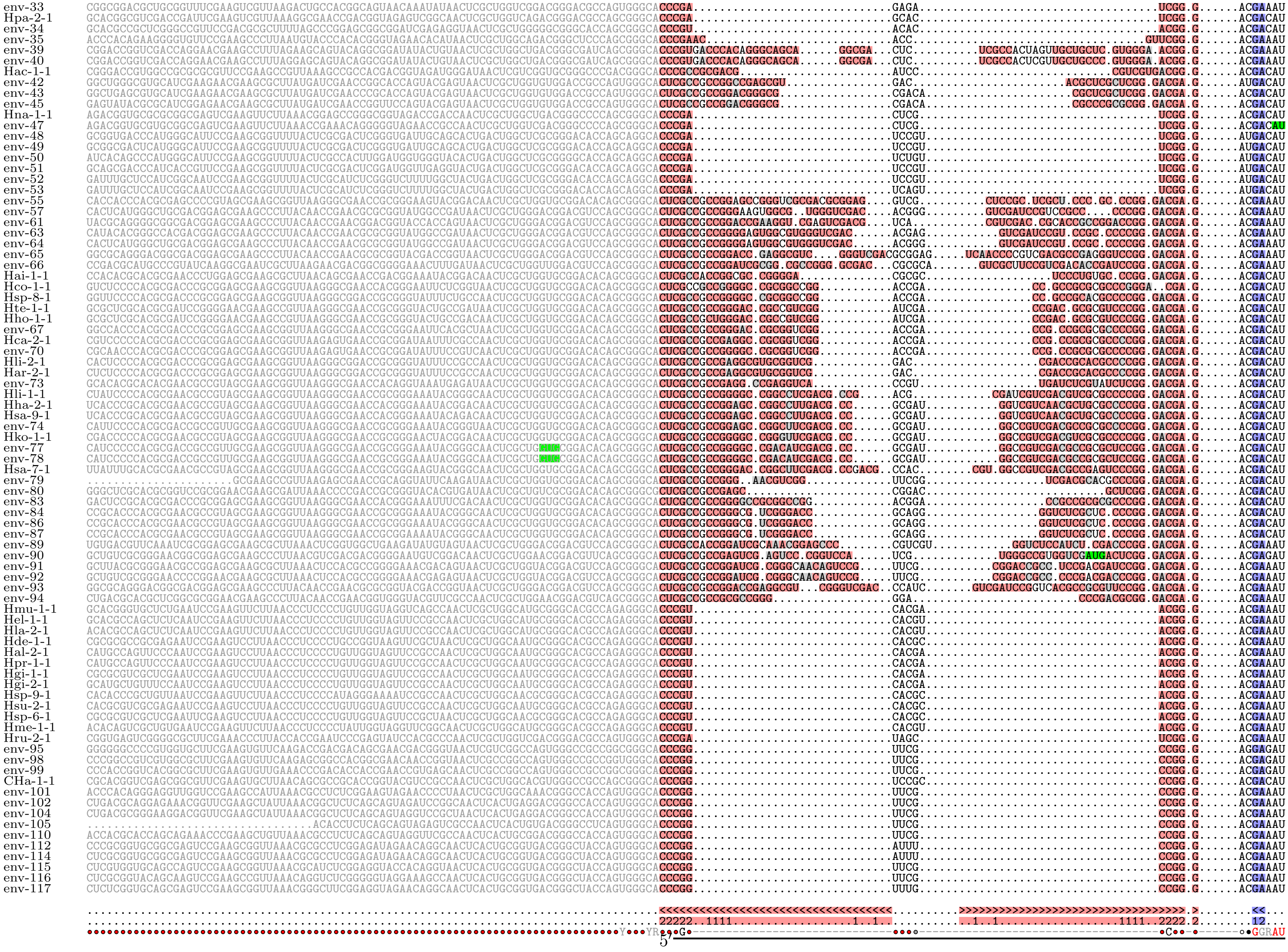
env-33
Hpa-2-1
env-34
env-35
env-39
env-40
Hac-1-1
env-42
env-43
env-45
Hna-1-1
env-47
env-48
env-49
env-50
env-51
env-52
env-53
env-55
env-57
env-61
env-63
env-64
env-65
env-66
Hai-1-1
Hco-1-1
Hsp-8-1
Hte-1-1
Hho-1-1
env-67
Hca-2-1
env-70
Hli-2-1
Har-2-1
env-73
Hli-1-1
Hha-2-1
Hsa-9-1
env-74
Hko-1-1
env-77
env-78
Hsa-7-1
env-79
env-80
env-83
env-84
env-86
env-87
env-89
env-90
env-91
env-92
env-93
env-94
Hmu-1-1
Hel-1-1
Hla-2-1
Hde-1-1
Hal-2-1
Hpr-1-1
Hgi-1-1
Hgi-2-1
Hsp-9-1
Hsu-2-1
Hsp-6-1
Hme-1-1
Hru-2-1
env-95
env-98
env-99
CHa-1-1
env-101
env-102
env-104
env-105
env-110
env-112
env-114
env-115
env-116
env-117
5'

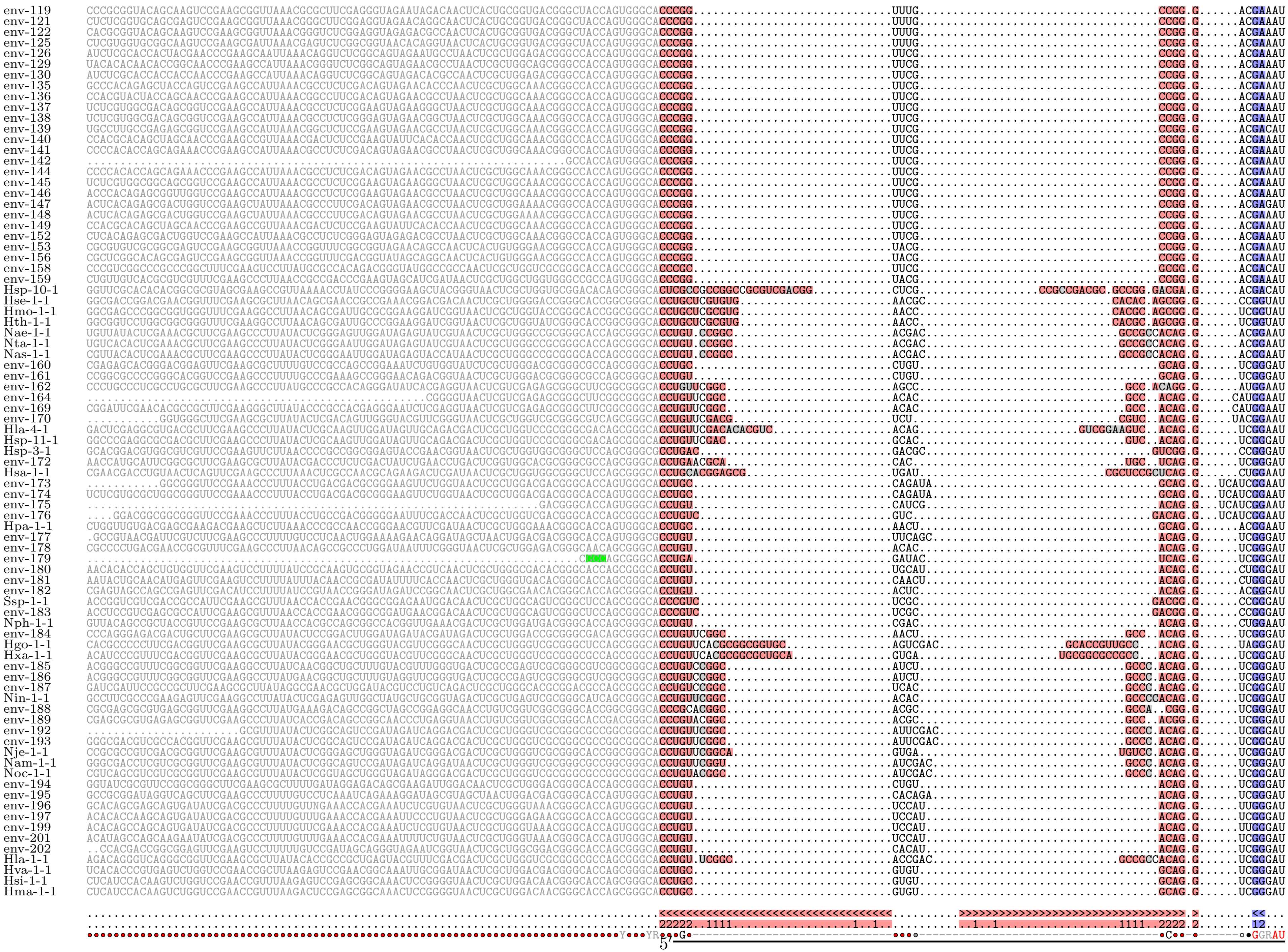
env-119
env-121
env-122
env-125
env-126
env-129
env-130
env-135
env-136
env-137
env-138
env-139
env-140
env-141
env-142
env-144
env-145
env-146
env-147
env-148
env-149
env-152
env-153
env-156
env-158
env-159
Hsp-10-1
Hse-1-1
Hmo-1-1
Hth-1-1
Nae-1-1
Nta-1-1
Nas-1-1
env-160
env-161
env-162
env-164
env-169
env-170
Hla-4-1
Hsp-11-1
Hsp-3-1
env-172
Hsa-1-1
env-173
env-174
env-175
env-176
Hpa-1-1
env-177
env-178
env-179
env-180
env-181
env-182
Ssp-1-1
env-183
Nph-1-1
env-184
Hgo-1-1
Hxa-1-1
env-185
env-186
env-187
Nin-1-1
env-188
env-189
env-192
env-193
Nje-1-1
Nam-1-1
Noc-1-1
env-194
env-195
env-196
env-197
env-199
env-201
env-202
Hla-1-1
Hva-1-1
Hsi-1-1
Hma-1-1
5′

```
Ham-1-1   UCACAUCCGCGAGUCUGGUCCGAACCGUUUAAGGCUCCGAACGGCAAACUCCGGGUAACUCGCUGGACGACGGGCACCAGCGGGCACCUGC..............................GUGU.....................................GCAG.G......UCGGGAU
env-203   UCACACCCGCGAGUCGGGUCCGAACCGCUUAAGAGUCCGAACGGCAAACUCCGGGUAACUCGCUGGACGACGGGCACCAGCGGGCACCUGC..............................GUGU.....................................GCAG.G......CCGGGAU
Hca-1-1   CUCAUCCACAAGUCUGGUCCGAACCGUUUAAGAGUCCGAGCGGCAAACUCCGGGGUAACUCGCUGGACGACGGGCACCAGCGGGCACCUGC..............................GUGU.....................................GCAG.G......UCGGGAU
Hsa-2-1   UCACAUCCGCGAGUCUGGUCCGAACCGUUUAAGACUCCGAACGGCAAACUCCGGGUAACUCGCUGGACGACGGGCACCAGCGGGCACCUGC..............................GUGU.....................................GCAG.G......UCGGGAU
Hja-1-1   CCCAUCCCUAAGUCCGGUCCGAACCGCUUAAGAGUCCGAACGGCAAACUCCGGAGUAACUCGCUGGACGACGGGCACCAGCGGGCACCUGC..............................GUGU.....................................GCAG.G......UCGGGAU
Har-1-1   CGCAUCCACGAGUCUGGUCCGAACCGCUUAAGAGUCCGAACGGCAAACUCCGGAGUAACUCGCUGGGCGACGGGCACCAGCGGGCACCUGC..............................GUGU.....................................GCAG.G......UCGGGAU
env-206   UGAGCGGCUCCCGGCGGAGUUCGAAGGCCUUUUGGCCGCCGGCGGGGUACCGCCUGUAACUCGCUGGAGACGGGCACCAGCGGGCACCUGC..............................CUGU.....................................GCAG.G......ACGGGAU
Hmu-2-1   AGCCCGUCUCGAUCAGCUUCGAAUGGCUUAAGGGCGCAAGCCGGGAAGCAGUGAGUAACUCGCUGGACGACGGGCACCAGCGGGCACCUGCAUGGACCG......................AUCGA............................CGGGUCGUACAG.G......UCGGGAU
Hka-1-1   AGACCGUCUCGAUCAGCUUCGAAUGGCUUAAGGGCGCAAGCCGGGAAGCAGUGGGUAACUCGCUGGACGACGGGCACCAGCGGGCACCUGCAUGGACCG......................AUCGA............................CGGGUCGUACAG.G......UCGGGAU
env-207   CUCAACGGACGGCGGGGUUUCGAAGCCCUUAACAGCGGUUGCGGUAAAGUUGGAGCAACUCGCCGGACGACGGGCACCGGUGGGCACCUGU..............................CUAC.....................................GCAG.G......UCGGGAU
env-208   ACCAGUUGUCGACCGCAUUUCGAAGCCCUUAACCCCUGCUGUGAUAAGUUUCGAACAACUCGCCGGACGACGGGCAUCGGCGGGCACCUGU..............................CUAC.....................................GCAG.G......UCGGGAU
env-210   CGCGGAAACCCGUUCGAUUUCGAAGUGUUUAACCCCGUCAGUCGUAAAAAUCAGGCAACUCGCUGGACGACGGGCACCAGCGGGCGCCUGU..............................CCAC.....................................ACAG.G......UCGGGAU
env-212   CGCAAGGCCCCGAUCGGUUUCGAAGUGUUUAACCCCGGCAGUCGUGAAUCUCCCGUAACUCGCUGGACGACGGGCACCAGCGGGCGCCUGU..............................CUAC.....................................GCAG.G......UCGGGAU
env-213   CGUGAGAUCCCGUUCGGUUUCGAAGUGUUUAACCCCGACAGUCGUGAAUCUCCCGUAACUCGCUGGACGACGGGCACCAGCGGGCGCCUGU..............................CUAC.....................................GCAG.G......UCGGGAU
env-214   CGACGUUUUGAGGAUACUUUCGAAGCCCUUAACCCCACUAGCGAAGAAUCUUAGUCAACGCGCCGGACGACGGGCACCGGUGGGCACCUGU..............................CUAC.....................................ACAG.G......UCGGAAU
env-215   AUCCGGCGCGUAAGACGUUUCGAAGCCCUUAACCGCGAUUAUUGUGAAGAGACGGUAACUCGCUGGACGACGGGCACCAGUGGGCACCUGU..............................CUAC.....................................GCAG.G......UCGGGAU
env-216   CGGCCAGUUCGAGCGCUUUCGAAGCGCUUAACCCCGACAAUACAGAAAAUCUGGGUAACUCGCUGGCGGACGGGCACCAGCGGGCACCUGU..............................CUAC.....................................GCAG.G......UCGGGAU
env-217   CUUGACCGUCACGGGCUUUCGAAGCCCUUAACCGACACAGCAGCGAAUUCCACGGUAACUCGCCGGAGGACGGGCACCGGUGGGCACCUGU..............................CUAC.....................................ACAG.G......UCGGGAU
env-218   ........................GCUUAACCGACACAGCGGUGAAUUUAAUCGUAACUCGCCGGACGACGGGCGACCGGUGGGCACCUGU..............................CUAC.....................................GCAG.G......UCGGGAU
env-219   CGUGGGUGCCACAGGCUUUCGAAGCGCUUAACCGACACAGCGGUGAAUUUAAUCGUAACUCGCCGGACGACGGGCACCGGUGGGCACCUGU..............................CUAC.....................................GCAG.G......UCGGGAU
env-220   CGUGGGUGCCGCAGGCUUUCGAAGCGCUUAACCGACACAGCGGUGAAUUUAAGCGUAACUCGCCGGACGACGGGCACCGGUGGGCACCUGU..............................CUAC.....................................GCAG.G......UCGGGAU
Nmo-1-1   CGUCGACCGCCACGGGCUUUCGAAGCGCUUAACCGACCCAGCGGUGAAUUCCGGGUAACGCGCCGGACGACGGGCACCGGUGGGCACCUGU..............................CUAC.....................................ACAG.G......UCGGGAU
env-221   GGACUCGGGCUGUACGGGGUUCGAAGCGUUUUUCCACGACGGCGAUGAACUGUGGGUAACUCGCUGGAGACGGGCACCAGCGGGCGCCUGU..............................CUGU.....................................ACAG.G......UCGGGAU
env-223   GUAUCGCCCUCCGGUGGGCUUCGAAGCGUUUUUUAUCCGAGACGCGAAAGUUCAGGUAACUCGCUGGAGACGGGCACCAGCGGGCACCUGU..............................CUGU.....................................ACAG.G......UCGGGAU
env-224   CACCACCGGUGUUCGGACCGCUUCGAAGUAUUUUAACUUAAAACUGAGAAGUAUAUACAACUCGCUGGGACGGGCACCAGCGGGCACCUGU..............................CUGU.....................................ACAG.G......UCGGGAU
env-226   ...................................................AGCGUAACUCGCUGGACGACGGGCACCAGUGGGCACCUGU..............................CCAC.....................................ACAG.G......UAGGGAU
env-227   .......................................AAUACGGAACUAUCGAUAACUCGCUGGACGACGGGCACCAGUGGGCACCUGC..............................GUGU.....................................GCAG.G......UCGGGAU
env-228   CGAAUCGAGCGGCGCGGUUCGAAGCUCUUAAACGCCCAGUGCGACGUAUCUCGGGUAGACUCGCUGGAGACGGGCACCAGCGGGCACCCGUC.............................GCG.....................................GACGG.G......CCGGGAU
env-229   CGAGGCGAGCGGCGCGGUUCGAAGCUCUUAACCCUCCCGUAGGACGUAGCUCUGGUAGACUCGCUGGGGACGGGCACCAGCGGGCACCCGUC.............................GCGC....................................GACGG.G......CCGGGAU
Hci-1-1   AUGUACGACCGUGCGAAAGACGAAGGCCUUAAACCCCCGAACGGGAUAUCUUGGGUAACUCGCUGGUCAACGGGCACCAGUGGGCACCUGC..............................AAUC.....................................GCAG.G......ACGGAAU
Hut-1-1   AUCUCACUCACGGAAGUUCGAAUUCCUUAAGAGGACGACCGAAGUAUGAGUGAGUAACUCGCUGGUCGGCGGGCAGCCAGCGGGCACCUGU..............................UUAG.....................................GCAG.G......UCGGAAU
env-230   .................UUCCGAAGCCCUUAGAACCCCGAGCGAAGAAGAGGCGUAUAACUCGUCGGUCGCGGGCACCGGCGGGCACCUGC..............................UACG.....................................GCAG.G......ACGGAAU
env-231   GGAGUCUCGCAGGUGCUUUCGAAGGUCUUAAACUCCGAACAGCGGUAUUCCAGAGUAACUCGCUGGACGACGGGCACCAGCGGGCACCUGUC.............................AUC.....................................GACAG.G......ACGGAAU
env-232   GAUGGAUCGACGCUGCUUUCGAAGGCCUUAAACUCCAAACGGCGGUACUCACGGGUAACUCGCUGGACGACGGGCACCGGCGGGCACCUGUC.............................AUC.....................................GACAG.G......ACGGAAU
env-233   GACGUAUCGAGGGGUCUUUCGAAGGUCUUAAACUCCGAACGACGGUAUCCAACUGUAACUCGCUGGACGACGGGCACCAGCGGGCACCUGUC.............................AUC.....................................GACAG.G......ACGGAAU
env-234   AACAGCCGCAGCCUCACUUCGAAGCCCUUUUGUCCACAAAUCGGAAAGGAGAGCACACCACGCUGGACGACGGGCACCAGCGGGCACCUGUC.............................ACAG....................................GACAG.G......ACGGGAU
env-235   ...........CCCUGUAUCGAAGUGCUUAAGCGAAUCUCAUGGGUAAAUUAUAGUAUCUCGCUGGAAGACGGGCACCAGCGGGCACCUGUC.............................UCU.....................................GACAG.G......UCGGGAU
env-236   CUGACGCCCGGGGGUUUCGAAGCGCUUAAACGAAGCACAGGGGAAUAUAUCAUAAGUUCUCGCUGGCGGACGGGCACCAGCGGGCACCUGUC.............................AUC.....................................GACAG.G......CCGGGAU
Nba-1-1   ACAGCCACUUCCGGAGUUCGAAGCCCUUAUACUCGAGAGUUGGAUAGACUGUCGUAGACUCGCUGGGUCGCGGGCGUCAGCGGGCACCUGU.GCA..........................GCAUUG...............................UGCUACAG.G......UCGGGAU
env-237   ACUGCCGUUCCCGGAGUUCGAAGCCCUUAUACUCGAGAGUUGGAUAGAUUGUCGUAGACUCGCUGGGUCGCGGGCGUCAGCGGGCACCUGU.GCA..........................GCAUUG...............................UGCUACAG.G......UCGGGAU
Nsu-1-1   CCGUCUGGUCCGGGAGUUCGAAGGCCUUAUACUCGAGAGUUGGGUAGAUGGUCGUAGACUCGCUGGGUCGCGGGCGUCAGCGGGCACCUGCGCAGCAC.......................GUCUCU............................GUGCUA..CAG.G......UCGGGAU
Npa-1-1   CACGGGCGUUCUCGGAGUUCGAAGCCCUUAUACUCGAGAGUUGGGAAGUGUGAGUAGACUCGCUGGUUCGCGGGCAUCAGCGGGCACCUGU..CCGA........................CGG................................UCGGUCACAG.G......UCGGGAU
Nal-2-1   CCACGGGCGUCUCGGAGUUCGAAGCCCUUAUACUCGAGAGUUGGGAAGUGUGAGUAGACUCGCUGGUUCGCGGGCAUCAGCGGGCACCUGU..CCGA........................CGG................................UCGGUCACAG.G......UCGGGAU
Nga-1-1   CAUGGCUGUUCUCGGAGUUCGAAGCCCUUAUACUCGAGAGUUGGGAAGUGUGAGUAGACUCGCUGGUUCGCGGGCAUCAGCGGGCACCUGU..CCGA........................CGG................................UCGGUCACAG.G......UCGGGAU
Hdj-1-1   ACGCAGGGUGUGGCCGGUUCGAAGCGCUUAUACUCGAGAGCUAGAUAGUUACGGUAGACUCGCUGGUUCGCGGGCAUCAGCGGGCACCUGCACGGC.........................UUU.................................GCC..GCAG.G......UCGGGAU
Hli-3-1   GUAGCGCGCUCGGGGAGUUCGAAGCCCUUAUACUCGAGAGUUGGGUAGAUCCAGUAGACUCGCUGGUUCGCGGGCAUCAGCGGGCACCUGCACGGU.........................ACCGG...............................GCC..GCAG.G......UCGGGAU
Hje-2-1   ACGCCGCGUUCCCGGAGUUCGAAGCGCUUAUACUCGAGAGCCGGGAAGAGAGGGUAGACUCGCUGGUUCGCGGGCAUCAGCGGGCACCUGCACGACG........................CCG................................CGUCA..CAG.G......UCGGGAU
Hth-2-1   ACGCCGCGUUCCCGGAGUUCGAAGCGCUUAUACUCGAGAGCCGGGAAGAGUGGGUAGACUCGCUGGUUCGCGGGCAUCAGCGGGCACCUG...CACG........................ACGC...............................CGUGUCACAG.G......UCGGGAU
Hsa-10-1  CGUUACCGUCGGCGUCGUUCGAAGCCCUUAUACUCGAGAGUUGGGUAGGUCCGGUAGACUCGCUGGUUCGCGGGCAUCAGCGGGCACCUGUACGACG........................CCG................................CGUC..ACAG.G......UCGGGAU
env-238   GCCGUUUGUUUCGACGCUUCGAAGCGCUUAUACACGAACGCUGGAUAGAACCGACAGACUCGCUGGGCUGCGGGCACCAGCGGGCACCUGU.UCGGC........................UCGAC..............................GCCGCCACAG.G......ACGGGAU
env-239   GCCGUUUGUUUCGACGCUUCGAAGCGCUUAUACACGAACGCUGGAUAGAACCGACAGACUCGCUGGGCUGCGGGCACCAGUGGGCACCUGU.UCGGC........................UCGAC..............................GCCGCCACAG.G......ACGGGAU
Hni-1-1   CACGCCACUUCCGAUGGUUCGAAGCGCUUAUACACGGCGGCUGGGUACGUUUCGACGACUCGCUGGGUCGCGGGCACCAGCGGGCACCUGU.UCGGC........................AACCGAC............................GCCGCCACAG.G......CCGGGAU
Nch-1-1   CAACACGCUCCGCCUGCUUCGAAGCCCUUAUACUCGGGAGUUGAAUAGGUGAAACUGACUCGCUGGAUCGCGGGCACCAGCGGGCACCUGU.UCGGC........................ACGCGAC............................GCCGCCACAG.G......ACGGGAU
Nhu-1-1   CGACACGGUCCGCACGCUUCGAAGCCCUUAUACUCGGGAGUUGAAUAGGUGAAACCGACUCGCUGGAUCGCGGGCACCAGCGGGCACCUGU.UCGGC........................ACACGAC............................GCCGCCACAG.G......ACGGGAU
Nve-1-1   CACGGCGUCUCACGGAGUUCGAAGCCCUUAUACUCGAGAGUUGGGAAGGGUGAGUAGACUCGCUGGUUCGCGGGCAUCAGCGGGCACCUGU.ACGGC........................ACC................................GCCGCCACAG.G......UCGGGAU
Hsa-8-1   GCGGCGUGGCCGCGCAGUUCGAAGCCCUUAUACUCGAGAGUUGGGAAGAGAGAGUAGACUCGCUGGGUCGCGGGCGCCAGCGGGCACCUGU.ACGGC........................ACC................................GCCGCCACAG.G......UCGGGAU
Htu-1-1   GCUCGCCGUUCCCGCAGUUCGAAGCCCUUAUACUCGAGAGUUGGGAAGAGAAGGUAGACUCGCUGGGUCGCGGGCGCCAGCGGGCACCUGU.ACGGC........................AUU................................GCCGCCACAG.G......UCGGGAU
env-243   GUGACCGGCCACGGAGUUCGAAGGCCUUAUACUCGAGAGUUGGCUAUCACGACGUAGACUCGCUGAGUCGCGGGCAUCAGCGGGCACCUGU.UCGGCG.......................CGG...............................CGCCACCACAG.G......UCGGGAU
Hca-3-1   GGUGCGCCGUCGCGAGUUCGGAUCGCUUAAGGGCGCGAGCGGGGAACUGGCGAGUAACUCGCUGGGCGGUCGGGCACCAGCGGGCGCCUGC..............................CUGU.....................................GCAG.G......UCGGGAU
env-244   AACCCACGCCAGCGGAGUUCGAAGUGUUUAACCCCGGGACCGGGGAAGGACGGGGUAACUCGCUGGUCGACGGGCACCAGCGGGCACCUGCCGCGUCGCGUUUCGAGGGG...........ACG...................CCCCUCG...CGCGAUCCA.CAG.G......ACGGGAU
env-245   AAUACGGCGAGCACGUGGUUCGAAGCGCUUAUAUUUGCGACGCUGAUAGCUGCUGUUGACUUACUGGCGGCGGGCACCAGUGGGCACCUGCACGG..........................GUUCGA...............................CC..GCAG.G......UCGGGAU
env-246   CGAGCGGGGCGGCGCCACUCCGAAGCUCUUUUGCCCGAAAGCGGGGAAGAGAGGAUAACUCGCUGGGACGCGGGCGCCAGCGGGCACCUGC..............................CUGU.....................................GCAG.G......UCGGGAU
env-247   UGUGGCUAAACCGGCGGUUCGAAGCCCUUAUACUCACCACUCCGGUAGCUGUGAGUGACUGACUGGACGACGGGCACCAGUGGGCACCUGCACGG..........................CUAGA................................CC..GCAG.G......UCGGGAU
env-248   AAAUCGGAUACUGACGCUUCGAAGCGCUUAUACUCGCCACUCCGGUAGCUCUGGGUGACUGACUGGACGGCGGGCACCAGUGGGCACCUGCACGG..........................CUAGA................................CC..GCAG.G......UCGGGAU
env-249   AAAGCGGAUACAGACGCUUCGAAGCGCUUAUACUCGCCACUCCGGUAGCUCUGGGUGACUGACUGGACGGCGGGCACCAGUGGGCACCUGCACGG..........................CUAGA................................CC..GCAG.G......UCGGGAU
env-250   GUUCCGGGACCGGCCGAGUUCGAAGCGUUUUAGCCCGGCACUCGAGUAGGUUGUGUAUCUCGCUGGCGGACGGGCACCAGCGGGCGCCUGC..............................GUGU.....................................GCAG.G......UCGGGAU
env-251   UCCGGGACCGGCCGACGAGUUCGAAGCGUUUUAGCCUGGCACUCGCAUAACUGUCCAUCUCGCUGGCGAACGGGCACCAGCGGGCGCCUGC..............................GUGU.....................................GCAG.G......UCGGAAU
env-253   ...............................................AUUUCUGAUAACUCGCUGGCGGACGGGCACCAGCGGGCACCUGC..............................CUGU.....................................GCAG.G......CCGGGAU
Hsu-1-1   AACCGGCCUGACCGCGAUUCGAAGUCCUUUUAGUCGAACCGCGCCGAUCAUACGAUAUCUCGCUGGUGAGCGGGCUCCAGCGGGCUCCCGA..............................UAGCC....................................UCGG.G......ACGGGAU
Hsp-2-1   CCUUCGACGUCGAGUAGUUCCGAAGUUCUUAACCCCGACCGAUUCGAACUCAGGGUAACUCGCUGGCGAGCGGGCUCCAGCGGGCUCCUGAC.............................CACA....................................GUCAG.G......ACGGGAU
Has-1-1   ACUGCGGACGACAGCGCUUCGAAGCCCUUAUGAAACGACGUUCGGUAGUAUGGGUAGCUACGCUGGCGGCGGGCACCAGCGGGCACCCGUACGG..........................GUUAU................................CC..ACGG.G......UUGGAAU
env-254   CCGUACACCGCCGCUCGCUUCGAAGCCCUUAUGAAACCGGACGGGAAACCAUCGGUAGACUCGUCGGCAGCGGGCACCGACGGGCACCCGUACGGC.........................AACC................................GCCC.ACGG.G......CCGGGAU
Hag-1-1   GACCGGGUCGAUGGAGGAUGUUUCGAAGCCCUUAACAGCGCCACUGGCGGAGUUCCGGUGACUCGCCGGGCGGGCACCGGCGGGCACCUGCG.UGCGA.......................UGU...............................UCGCAUUGCAG.G......UCGGAAU
Hsa-4-1   UCGGCCAAGGACCGUGGCGUUCCGAAGCGCUUAACAGCGCCACUGGCGGAGAUCCAGUGACUCGCUGGGACGGGCACCGGCGGGCACCUGCG.............................UGAUAC..................................UGCAG.G......UCGGAAU
Hsa-5-1   CCGGACGAGGACCGCGGCGUUCCGAAGCGCUUAACAGCGCCACUGGCGGAGAUCCAGUGACUCGCUGGGACGGGCACCGGCGGGCACCUGUGG............................CGAUA..................................CUGCAG.G......UCGGAAU
env-255   UCACUGAUGUUUGUUUCGAAGUGCUUAAAAGGGUCGACAGCUGCAUGAGGAGUACAGAUUCGCCGGGAUGCGGAACCGGCGGGGCGCCUGU..............................AUAAC....................................UCAG.G.UUGGUGCGGGAU
env-256   CGAUUGACGUCUGUUUCGAAGUGCUUAAAAGGAUCGAAAGCUGCAUGAGGAGUACAGAUUCGCCGGAAUGCGGAACCGGUGGGGCGCCUGU..............................AUAAC....................................UCAG.G.UUGAUGCGGGAU
env-262   ................................................................................GGGGCGCCUGU..............................AUAAC....................................UCAG.G.UUGAUGCGGGAU
env-266   CUAAGGACAUUUUUUUUCGAAGUGCUUAAAAGGGUCGGAAGCUGCAUGAUGAAUACAGAUUCGUCGGGUAGCGGAACCGGCGGGGCGCCUGU..............................AUAGA....................................UCAG.G.UUGACGCGGGAU
Mae-1-1   AACCGUUAAAUUAAUAUACUAAAUACCAUACAAGAUAAUGGGCAUAUUAUUACAUAUAGGAUAGAAAUAAAAACAACAGACAAUAACGGGA..............................GUCAAA...................................UCCC.G.....UCGGGCAU
env-267   GGGCGAAGGCUAUCGUUGCGGGGCCGGUUUAUUCUGCGAAAUAUUUAUAUUAUAGUACCUGUUUAUUGUACCCACAGUUUCGUUCGCGGGA..............................UCCG.....................................UCCC.G......CCGGGAU
env-268   UGAAAGAAGCUAGCCUAGUGGAUAAGCCAUUGAGAACUUGAAAAGGUUUAUUAACAAUCCACUGGCUUCAUACCUACGAGCUAUCUACGGA..............................UGUCGU...................................UCCG.U.......UGGGAU
env-269   .....................................................ACAAUCCACUGGCUUCAUACCUACGAGCUAUCUACGGA..............................UGUCGU...................................UCCG.U.......UGGGAU

          ......................................................................................<<<<<<<<<<<<<<<<<<<<<<<<<<<<<<<<<<<...........>>>>>>>>>>>>>>>>>>>>>>>>>>>>>>>>>>.>........<<...
          ......................................................................................22222..1111..................1..1.............1..1...................1111..2222.2........12...
          ●●●●●●●●●●●●●●●●●●●●●●●●●●●●●●●●●●●●●●●●●●●●●●●●●●●●●●●●●●●●●●●●●●●●●●●●●●●●●●●●Y●●●YR●●●G●------------------------------●●●○-------------------------------------●C●●-●------○●GGRAU
                                                                                                5′
```

```
env-270  CUUGUAGAUAAAGAUUAUGAUCGCCCUGGGGAGUUUUUCAGACAUACGUUUAAUAGUAAACACAUCCAAAAAGCACACUUCAUCGGCGGG...............................AAUGCG...................................CCC.G......CAGGGAU
env-271  GCUUGUAGAUAAAGAUUAUGAUUGCCUGAUGAGUUUUCCAGACAUACGUUUAAUAAUAAACACAUCCAAAAAGCACACUUCAUCGGCGGG...............................AAUGCG...................................CCC.G......CAGGGAU
env-273  GCUUGUAGAUAAAGAUUCUGAUCGCCUGAUGAGUUUUCCAGACAUACGUUUAAUAAUAAACACAUCCAAAAAGCACACUUCAUCGGCGGG...............................AAUGCG...................................CCC.G......CAGGGAU
env-274  AGAUAACCACAUGUUUUAAGCUAAGCCUUAAUAUUUUAUUGGAAAAGUUUAUUUCACUGCAAAGUGCUAUGAAACAGAAGCACUGGCGGAU..............................AAAUC...................................GUCC.G......CUGGGAU
env-276  AGGGCUGAAGAUUAGAGAUUUUGGCGCGCGAUAUUUUAUUGGAAAAGUUUAUUUCACUGCAAAGUGCUAUGAAACAGAAGCACUGGCGGAU..............................AAAUC...................................GUCC.G......CUGGGAU
env-277  GAAAGCAGAGCUUCAAAGCAAACAGCCACAAACGUGAUUGGGAAAAGUUUAUUUUGCCGCUUGAUGCUAUGAAUCAGAACCAUGGGCGGAAA.............................AGGG...................................UUUCC.G......CUGGGAU
env-278  GAAAGCAGAGCUUCAAAGCAAACAGCCACAAACGUGAUUGGGAAAAGUUUAUUUUGCCGCUUGAUGCUAUGAAUCAGAACCAUAGGCGGAAA.............................AGGG...................................UUUCC.G......CUGGGAU
env-281  AGUAACCAGCUAAAACUUCAAUGUGGCUGAUUCCAGCUUUUGAAAAGGUUUAUUAGUAGUUACGUUGUUUUAGCUAGAAGCCGCUGGCGGA..............................CUUGGA..................................UCCG.U.......UGGAAU
env-283  AUUAACAGACGCAAUCGAUUGUUUUAGGCUCUAUGAUGCGAGAAAGGUUUAUUUCAUUGCAGGGCACUAUGAUGUGAAAUUGCAGGCGGACA.............................ACCG...................................UGUCC.G......UUGGGAU
env-284  UAGAAAAGCCCCAACAAAGCGUAAGAGCUCCGUUUCCGCUGGAAAGGUUUAUCUCAUUCAAGGGCACUAUGUUGUGAAAUUGCAGGCGGACA.............................ACCG...................................UGUCC.G......UUGGGAU
env-286  AUUAACAGACGCAAUCGAUUGUUUUAGGCUCUAUGAUGCGAGAAAGGUUUAUUUCAUUGCAGGGCACUAUGAUGUGAAAUUGCAGGCGGACA.............................ACCG...................................UGUCC.G......UUGGGAU
env-287  CUGCUUUACCCCGAUUUGGGUUGGGAGCGGUGUUUCUUGUGGAAAAGGUUUAUUUCAGUGCAUGCCGUUUCCUUGGGAACUGCAGGCGGAC..............................UAACUU..................................GUCC.G......UUGGGAU
env-289  GAAACCAAAGUAAACGCUAGAAAAUGUUUGAAACAUCAUUAGAAAGGUUUAUUUUUAAGCACGGUGCUAGCAUUUAAAAUUGCAGGCGGAC............................CAUCAGA...................................GUCC.G......UUGGGAU
env-290  AUAAAAGGGAGGGGGUACGGUUUCCGAGCAAUAGAGGUUUGGAAAGGUUUAUUUUUAAGCAGGGUUUUAAAAUGUAAACCUGCAGGCGGAC............................CAUCAGA...................................GUCC.G......UUGGGAU
env-291  CAAAAGGGGUUAACGGCUAGAAAAUGUUUGAAACAUCAUCGGAAAGGUUUAUUUUUAAGCGUGGCGGUAUCAUGUAAAAUUGCAGGCGGAC............................CAACUGU...................................GUCC.G......UUGGGAU
env-293  GGAAAGCAAAGUAACCGCUAGAAAUGUUAGAAACAUCAUUGGAAAGGUUUAUUUUUAAGCUUGUUGCUAGCAUUUAAAAUUGCAGGCGGACA.............................AUCUG..................................UGUCC.G......CUGGGAU
env-295  .............................UUGGCGGUGCGGAAAGGUUUAUUUCAUUGCAGGGCACUAUGAUGUGAAAUUGCAGGCGGAC.............................ACCAUA...................................GUCC.G......UUGGGAU
env-296  UUUGAGAGGCGAAUUAGGCUGUUUUGGACUUGGCGGUGCGGAAAGGUUUAUUUCAUUGCAGGGCACUAUGAUGUGAAAUUGCAGGCGGAC.............................ACCAUA...................................GUCC.G......UUGGGAU
env-297  AAAAUAAAAAAAAUAUCUGCGAACUUGCUGUUUAUUUCUUGGAAAGGUUUAUUUUAUGCAAUAACACUCUGAUGUAAAAAUACAGGCGGACA.............................ACA....................................UGUCC.G......UAGGGAU
env-298  .........................................GAAAGGUUUAUUUUAUGCAAUAACCAUCUGAUAUGAAAAUACAGGCGGACA.............................ACA....................................UGUCC.G......UAGGGAU
env-299  ACAAAAUAUUUUACCAUUUUGUAUUUUGCUUUACUUUUUUGGAAAGGUUUAUUUUAUGCAAUAACCAUCUGUACAAAAAUACAGGCGGACA.............................ACA....................................UGUCC.G......UAGGGAU
env-301  ...........................................AAGGUUUAUUUUAUGCAAUAGCAAUCUGAUGUAAAAAUACAGGCGGAC..............................AAACA..................................CGUCC.G......UAGGGAU
env-302  UGGAAGUUAGGGAUUAACGUGUCAAGGGUGUCUGUUUUUUGGAAAGGUUUAUUUUAUGCAAUGAUGAUUGGAUGUAAAAAUACAGGCGGACA.............................AACA...................................UGUCC.G......UAGGGAU
env-304  CUUAGCCAAGCAAAAUUACUUAAUUAAUCUUUAAUCAUCAAGAAAGGUUUAUUUUGUUGGUUCGUUUAUUGAUGUGAAAUUGCUGGCGGACA.............................ACA....................................UGUCC.G......UUGGGAU
env-308  AAUGAGUAGCGAAAGUUUCGGUUUAUUAGGUUUCAGCUUUGAAAAAGUUUAUUAAUUGUUAUGUUGUCUUAGCUACAAGCCACUGGCGGAC..............................ACGG....................................GUUC.G......UUGGAAU
env-309  AAUGAGUAGCGAAAGUUUCGGUUUAUUAGGUUUCAGCUUUGAAAAAGUUUAUUAAUUGUUAUGUUGUCUUAGCUACAAGCCACUGGCGGAC..............................AUGG....................................GUCC.G......UUGGAAU
env-310  AAAUGAGUAGCGAAAGUCUCGGUUUAUUAGGUUUCAGUUUUGAAAAAGUUUAUUAGUUGUUAUGUUGUCUUAGCUACAAGCCACUGCGGAC..............................AUGG....................................GUCC.G......UUGGAAU
env-311  ......................................................GUUGUUAUGUUGUCUUAGCUACAAGCCACUGGCGGAC..............................AUGG....................................GUCC.G......UUGGAAU
env-312  AGAGUGAGAGGUUAAACCAAUCGCUAAGCCUUUAAGUAGACGAGGAUGGGUGUGGGUGGCUUUAGGCCACCAUUUCCUUUGUUUAGGCGGA..............................UACG....................................UCCG.C.......AGGGAU
env-317  AGCCAAGUUGGGCCAAUCGCUACACCUAAAAGUAAAUGAAAAGGNAGAAGCACUGGCUGAAGAGCCACACCAUUUCCUUUGUUUCGGCGGA..............................UACG....................................UCCG.C......AAGGGAU
env-318  .....................ACACCUAAAAGUAAAUGAAAAGGGAGAAGCACUGGCUGAAGAGCCACACCAUUUCCUUUGUUUCGGCGGA..............................UACG....................................UCCG.C......AAGGGAU
env-319  AGCCAAGUUGGGCCAAUCGCUACACCUAAAAGUAAAUGAAAAGGGAGAAGCACUGGCUGAAGAGCCACACCAUUUCCUUUGUUUCGGCGGA..............................UACG....................................UCCG.C......AAGGGAU
env-321  ...................................................................................UUGGCGGA..............................UACG....................................UCCG.C......GCGGGAU
env-322  GGACAAAGCGUUACACCUUUAAGCGAAUGUCCUCGAGUUGGCUUGGUCACGGCAUGCGUGCCGGACCAGACCAUUCCUUUGUGUUGGCGGA..............................UGCG....................................UCCG.C......ACGGGAU
env-323  GGACAAAGCGUUACACCUUUAAGCAAAUGUCCUCGAGUUGGCUUGGUCACGGCAUGCAUGCCGUACCAGACCAUUCCUUUGUGUUGGCGGA..............................UGCG....................................UCCG.C......ACGGGAU
env-324  CAGAGCGUUACACCUUUAAGCAAAUAGCUUAGGUGACCUCUAGGCUCAGAUCUAUAUUCUAGAGUCCUACCAUUUCCUUUGUGUUGGCGGA..............................UACG....................................UCCG.C......ACGGGAU
env-325  CAGAGCGUUACACCUUUAAGCAAAUAGCUUAGGUGACCUCUAGGCUCAGAUCUAUAUUCUAGAGUCNUACCAUUUCCUUUGUGUUGGCGGA..............................UACG....................................UCCG.C......ACGGGAU
env-326  UCGCUAACUCUUUAAGCGAAUAUCAAAGGACAGGGAUUGGCUUUGACCAGGAAUGCUUGGUCAGGCCAAACCGUUCCUUUGUGUUGGCGGA..............................UACG....................................UCCG.C......AAGGGAU
env-327  UCGCUAACUCUUUAAGCGAAUAUCAAAGGACAGGGAUUGGCUUUGACCAGGAAUGCUUGGUCAGGCCAAACCGUUCCUUUGUGUUGGCGGA..............................UACG....................................UCCG.C......AAGGGAU
env-328  UCGCUAACUCUUUAAGCGAAUAUCAAAGGACAGGGAUUGGCUUUGACCAGGAAUGCUUGGUCAGGCCAAACCGUUCCUUUGUGUUGGCGGA..............................UACG....................................UCCG.C......AAGGGAU
Pab-1-1  GAUUAUUCACCAAUUUAAGCACUUCGAUGCUGGAUCCAAAAUUUUUAUAUAGUGAUUGAAGGUUCUGGGUGUUGGAAUUAAAUUGGACUAA..............................AUAAA....................................ACG.U.........GGAU
Psp-2-1  UAUAAAUAAGACCUGAACAAAGAUUAUGUGGAUGCCUAAAGCACCAAAACCCUUAAAAAUUCUCCAUCCAACAUCGCCCGAACUUGGCGGG..............................GUAAA...................................CCCG.C.........GGAU
Tga-1-1  UCAAGGAAAUGAUCAAGGAAAACGGGGUGCACCCGCUUGGAUGAGCGCAACCCUUAAAAACCAACCCUCGAAACCCGACCGACUAGGCGGG..............................UUCG....................................CCCG.C.........GGAU
Tsp-3-1  AGGCGAGUUUCUGAAGGCGCAUUUCCAUCACCGCACUGAGUACUCCGCAAUCCUUAAAAACUCAACCUCGAACCUCGACCGACUGGGCGGG..............................CUCAG...................................CCCG.C.........GGAU
Tsp-2-1  UCGAGGAAAUGAUCAGGGAAAACGGUGUUCAUCCGGUUGGGUGAGCGCAACCCUUAAAAACCAACCCUCGAAACCCGACCGACUAGGCGGG..............................UUCG....................................CCCG.C.........GGAU
Tna-1-1  GGAGGAUGAGCCCCAACCGUUUUCCCGUCAUCGACGUUAGUCCUCCGCAACCCUUAAAAACCAACCCUCGAAACUCGACCGACUAGGCGGG..............................UUCG....................................CCCG.C.........GGAU
Teu-1-1  GACGAGGCGUCUGAGUUUUUCUCUGACCAUCAUAUCCAGUUAAUCCGCAACCCUUAAAAACUAACCCUCGAAACUCGACCGACUGGGCGGG..............................UUCG....................................CCCG.C.........GGAU
env-329  ...........................CUUUUUAAAUAAUGAGGCGCAACUUUUAAAAAGCAACAACCUUUAUUCACCACGACUAGGCGGU..............................GGAA....................................ACCG.C.........GGAU
Tsp-4-1  CACCACCCACUCAAGAUUUGCGGCUUGCUUUUUAAAUAAUGAGGCGCAACUUUUAAAAAGCAACAACCUUUAUUCACCACGACUAGGCGGU..............................GGAA....................................ACCG.C.........GGAU
Tsi-1-1  UCACCACCAACUUGAAAUUCAUCACUCACUUUUUAAAUAUACAGCGCAACUUUUAAAAAGCAACCUUGUUUAUUCACCUUGACUAGGCGGU..............................GAAA....................................ACCG.C.........GGAU
Tli-1-1  CACCACCCAUUCAAGAUUUGAGGCUUGCUUUUUAAAUAAUGAGGCGCAACUUUUAAAAAGCAACACCCUUUAUUCACCACGACUAGGCGGU..............................GGAA....................................ACCG.C.........GGAU
env-330  ACUACUCUUGGAUCGCAAAAGGAAAGUGCAAACUUAGAAUCCAUCGUAACCUUUAAAAACCUAAACUCUAAACUCACCACGACUAAGCGGG..............................UUUAG...................................CCCG.C.........GGAU
Tba-1-1  AGUUGUGGUUGUUUGAGUAUCUUUGGAAUUUUGAUAGGAAAAAUCGCAACUUUUAAAAACUCAGACUCUAAACUCACCUCGACCAGGCGGG..............................GGAAA...................................CCCG.C.........GGAU
Tpa-1-1  AGUAUUUAAAAGUUGUGGUCGUUCUCAGUUUGAACAGAAGGAAUCGCAACUUUUAAAAACUCAAACUCUAAACUCACCUCGACUAGGCGGG..............................GGAA....................................CCCG.C.........GGAU
Ppa-1-1  UUCGACCACCUAAAUACCUCAUCUCUUGGAUGUAUUAACUCUUCCGCAACCUUUAAAAACUAGAACUCUAAACUCACCACGACUAGGCGGG..............................GGAAA...................................CCCG.C.........GGAU
Pfu-2-1  UUACUUGUGAGUAAAUCGAUGCGUGACUUAAGGUUCUUUUAACUCCGCAAUGCUUAAAAACUCACCUUCCAACAUCCACCGACUUGGCGGG..............................GUAAA...................................CCCG.C.........GGAU
Pho-1-1  AUUUAAGAAUUUCGAUGCGAGACUUAAGGUUAAGUCUUUUAACUCCGCAACGCUUAAAAACUCACCUUCCAACAUCCUUCGACUUGGCGGG..............................GUCAA...................................CCCG.C.........GGAU
Psp-1-1  UUGAGAGGUCUAUUAUCAUGGAACACACCAAAUGCAUAUAGCAUGCGUAACACUUAAAAAGUCAGCUACCCAACUUGUCCCGACUAAGCGGG..............................GUUAA...................................CCCG.C.........GGAU
Pya-1-1  AUGGGAACCUCUUACAUACUCGAGAAGCCAUUCUAAUUCAACACCCGCAAUCCUUAAAAACUCUUCAUCCAACUUCGCCCGACUAGGCGGG..............................GUCAA...................................CCCG.C.........GGAU
Tko-1-1  CAGCCAGUUUUUCUAUGCGCAUGGACACCACCGGUUCUGGUAUUCCGCAACCCUUAAAAACUCACCCUCGAACCACGACCGACUAGGCGGG..............................GCAGA...................................CCCG.C.........GGAU
Tsp-1-1  AAACCAGGUUGCUGAGCCGCAUUUCAUCACCUCUAUCCAUUAUUCCGCAACCCUUAAAAACUCAACCUCGAACCUCGACCGACUAGGCGGG..............................GCAAA...................................CCCG.C.........GGAU
Pfe-1-1  GCUGUUCACAGGGUUUGAGAACAUUUGAGGUUCAGCACCUAAUUCCGCAACCCUUAAAAACCCAACCUCGAACUUCCCAUGACUUGGCGGG..............................GUAGA...................................CCCG.C.........GGAU
Ton-1-1  AAGUGAGCUUGUUGAGGCGCAUGGUUUUCACCGCUUCGACUACUCCGCAACUUUUAAAAACCUAAUCUCGAACUUCGCCCGACUAGGCGGG..............................GCAAA...................................CCCG.C.........GGAU
Tzi-1-1  GAUUCACGUGAUAAAAGGGUAAGCUCCAGCGCCCACCUCAGACAUCGCAACCCUUAAAAACCCAGCCUCCAACUCGGCCUGACUCGGCGGG..............................UUCAG...................................CCCG.C.........GGAU
env-331  CUUAAUUUCGUUAAAUUUUGAAGAGCUAAUUUGCUUUUGUUGGAAAAGUUUAUUUCAUUGUUAAGUGUGUUAUGAACAAAGCGUUGGCGGAU.............................AAAC...................................AUCCG.C.......UGGAAU
env-332  CUUAAUUUCGUUAAAUUUUGAAGAGCUAAUUUGCUUUUGUUGGAAAAGUUUAUUUCAUUGUUAAGUGUGUUAUGAACAAAGCGUUGGCGGAU.............................AAAC...................................AUCCG.C.......UGGAAU
env-333  AAAUCAUAGCAAAAAUAACUAAACUGACUAACUUCUAAUGAAAAAGCUUUAUUAACCGUCAAAUCGUUCAUAGGUGCAAUUUGCGGGCGGAU.............................AAUA...................................AUCCG.U.......AGGGAU
env-334  CUCCAAGUCAAGAAGGCCGUGCUUGUGAACAAAUGAGCAUCUGGAAAGGUUUAUCUUGAUGCUUAAUGCUAUGAGUCAAGCUGCUGGCGGAC.............................GAAA...................................GUCCG.C.......AGGGAU
env-336  UAUAAAUAGAUCUAAUAGUUUCUUGUUUAUAUUGUUCUAUUGGAAAAGUUUAUUUUACUGCAUAGAAGUAUGAAUAGCCGCUACUGGCGGAC.............................UAACU..................................GUCCG.C.......UGGGAU
env-338  UUCCUAGUCAAGAAAGCCGUGCUUGAAACCAAGUGACAAUCUGGAAAGGUUUAUCUUGAUGCGAAGUGCUAUGAACCAAGCUACUGGCGGAU.............................CAAA...................................GUCCG.C.......AGGGAU
env-339  AUUUGCGAGGUUAGAAAGGCUUCGUAAGCGUUUACCUAUUUAAGAAAGGUUUAUUUUGUUGAGCGGACUUUCUUUGAAAGUCAAGGGCGGA..............................GAAAA...................................UCCG.C.......UGGGAU
env-341  AGGCGAGACUUGAGAAUAAGUGAAAUAGUUGCAGUAAUUUUGGAAAAGUUUAUUUUGCUGGUUAAUGCAAUCAAUGAAGGCUAAUGGCGGA..............................GUAAA...................................UCCG.C.......UGGGAU
env-344  AAGCGAGACUUGAGAAUAAGUGAAAUAGUUGCAGUAAUUUUGGAAAAGUUUAUUUUGCUGGUUAAUGGAAUCAAUGAAGGCUGAUGGCGGA..............................GUAAA...................................UCCG.C.......UGGGAU
env-348  GAAACUUGAAAACAAAUAGGAUAUUGCUCGCUGAGUGUUCUGGAAAAGUUUAUUUUGUUGCAUGACACUAUGAAUAAAAGCUGAUGGCGGAUG............................UAA...................................UGUCCG.C.......UGGGAU
env-349  AAUCUAAAAAUAAGAAAUUCGCGGAAAGAUUUCAAAUUGCUGGAAAGGUUUAUUUUAUUGUGUGAAGCUAUGGAUUAAAGCUAUUGGCGGAU.............................AAUU...................................GUCCG.C.......UGGGAU
env-353  UUCGAUUUUUUGUAACGAAAGAGAAACUAAAAAAACUGCUGGAAAAGUUUAUUUUGUUGUGUGAAGCUAUGAAUCAAAAGCUACUGGCGGA..............................UAGGUG..................................UCCG.C.......UGGGAU
env-354  CAAGGAAUAUGCGCUUUAAAAAAAAAGAAACUGGAAUCUGCUGGAAAGGUUUAUUUUGCUGCGUGAAACUAUGAACUAAAGCAUUGGCGGA..............................UAGAUG..................................UCCG.C.......UGGGAU
env-356  UCAAGGAAUAUGCGCUUUUGAAAAAGAAACUGGAAUCUGCUGGAAAGGUUUAUUUUGCUGCGUGAAACUAUGAACUAAAGCUAUUGGCGGA..............................UAGAUG..................................UCCG.C.......UGGGAU
env-358  .................................................UAUUUUGUUGUGUGAAGCUAUGAAUCAAAAGCUACUGGCGGA..............................UAGGUG..................................UCCG.C.......UGGGAU
env-359  UUGUUCGAAAAGCUAAACUUGAAAGUAAAUAAAUUGUUUUAGGAAAAGUUUAUUUUGUCGCAGAUUGCUAUGAAUCAAAACCAAUGGCGGAU.............................AAAU...................................GUCCG.C.......UGGAAU
env-360  UUGUUCGAAAAGCUAAACUUGAAAGUAAAUAAAUUGUCUUAGGAAAAGUUUAUUUUGUUGCAGAUUGCUAUGAAUCAAAACCAAUGGCGGAU.............................AAAU...................................GUCCG.C.......UGGAAU
env-362  AGGAAUGUUGCCGCUUUGGCGAAAAGAAAGUAGAAUCUGCUGGAAAAGUUUAUCUUGCUGCGUGAAGCUAUGAAUUAAAGCUAUUGGCGGAU.............................AGGU...................................GUCCG.C.......UGGGAU
env-363  GCAAUCGGACUGCAAUGAAUGAUUUGCCACUGUCUUAGUCUGGAAAGGUUUAUUUUGUGGCUAGGUGCUAUCAAACAGAGCUAUUGGCGGAU.............................AGGU...................................GUCCG.C.......AGGGAU

         ......................................................................................<<<<<<<<<<<<<<<<<<<<<<<<<<<<<<<<<<<..........>>>>>>>>>>>>>>>>>>>>>>>>>>>>>>>>>>.>.........<<...
         ......................................................................................22222..1111.................1..1............1..1.................1111..2222.2.........12...
         ●●●●●●●●●●●●●●●●●●●●●●●●●●●●●●●●●●●●●●●●●●●●●●●●●●●●●●●●●●●●●●●●●●●●●●●●●●●●●Y●●●YR●●●G●-----------------------------●●●○-------------------------------------●C●●-●------○●GGRAU
                                                                                               5′
```

```
env-364  CUGAGGCAAGGCUUGAAAAUAAACCAGAGUGUGACUGAAGCCGGAAAAGUUUAUUAUGUUGCGUGAAGCUAUGAAUCAAGCUAUCGGCGGAUG..........AAAG..........GUCCG.C......UGGGAU
env-365  AGGAAUAUCGUCGAUUUGGAAAAAAGAAACUGGAAUCUUCUGGAAAGGUUUAUUUUGCUGCAUGAAACUAUGAACUAAAGCUAUUGGCGGAU...........AGAU..........GUCCG.C......UGGGAU
env-366  CAAAGCAAAGCUUGAAAACAAUUGAAUGUGCCUGAGAUUCUGGAAAGGUUUAUUUUGAUGCGUGGAACUAUGAAUCAAAGCUACUGGCGGAC...........GGGU..........GUCCG.C......UGGGAU
env-367  AAAAGCGGGGCUUGAAAACAAUUGAAUGUGCCUGAGAUUCUGGAAAAGUUUAUUUUGAUGCGUGAAACUAUGAAUCAAAGCUACUGGCGGAU...........AGGU..........GUCCG.C......UGGGAU
env-369  AGCAAAACUUGAAAACAAAUGAACAUUGCUAGCUGAGUACUGGAAAAGUUUAUUUUGUUGCAUGAAGCUAUGAAUUAAAGCUACUGGCGGAU...........AGGU..........GUCCG.C......UGGGAU
env-370  CAAAGCGGAGCUUGAAAACAAUUGAAUGUGCCUGAGAUUCUGGAAAGGUUUAUUUUGAUGCGUGAAACUAUGAAUCAAAGCUACUGGCGGAU...........GGGU..........GUCCG.C......UGGGAU
env-371  CAAAGCGGAGCUUGAAAACAAUUGAAUGUACCUGAGAUUCUGGAAAGGUUUAUUUUGAUGCGUGAAACUAUGAAUCAAAGCUACUGGCGGAU...........GGGU..........GUCCG.C......UGGGAU
env-375  ACCACGCUAUUAAAUUUUUCAAAGAACANCCCCAUUUAACUGGAAAAGUUUAUUUCACUGUAAGAUGCUAUGAAUNAAAACAAUUGGCGGAU...........AAAA..........GUCCG.C......UGGGAU
env-376  UCUAUCACGCUAUUAAAUUUUUCAAACAGUCCCCUUUAGCUGGAAAAGUUUAUUUCAUUGUAAGAUGCUAUGAAUCAAAACAAUUGGCGGAU...........AGAA..........GUCCG.C......UGGGAU
env-377  GUUACCACGCUAUUAAAUUUUUCAAACAGCCCCCUUUAUCUGGAAAAGUUUAUUUCACUGUAAGAUGCUAUCAAUCAAAACAAUUGGCGGA............UGAAAG.........UCCG.C......UGGGAU
env-378  ACCAUACUAUUAAAUUUUUCAAAGAACAGCCCCAUUCAACUGGAAAAGUUUAUUUCACUGUAAGAUGCUAUGAAUCAAAACAAUUGGCGGA............UGAAAG.........UCCG.C......UGGGAU
env-379  AACUUUAGAAAGCGAAAACAGAUAGAGAAUGAAAACCUAUUGGAAAAGUUUAUUUCAUUGUAAGAUGCUAUCAAUCAAAACAAUUGGCGGA............UGAAAG.........UCCG.C......UGGGAU
env-380  UCUACCACGCUAUUAAAUUUUUCAAAUAGUCCCUUUUAGCUGGAAAAGUUUAUUUCAUUGUAAGAUGUUAUGAAUCAAAACAAUUGGCGGA............UGAAAG.........UCCG.C......UGGGAU
env-381  UCUACCACGCUAUUAAAUUUUUCAAACAGUCCUCUUUAGCUGGAAAAGUUUAUUUCAUUGUAAGAUGCUAUGAAUCAAAACAAUUGGCGGA............UAAAAG.........UCCG.C......UGGGAU
env-382  ACCGCACUAUUAAAUUUUUCAAAGAACAGCCCCAUUUAGCUGGAAAAGUUUAUUUCAUUGUAAGAUGCUAUCAAUCAAAACAAUUGGCGGA............UAAAAG.........UCCG.C......UGGGAU
env-383  UCUACCACGCUAUUAAAUUUUUCAAACAGUCCCUUUUAGCUGGAAAAGUUUAUUUCAUUGUAAGAUGCUAUGAAUUAAAACAAUUGGCGGA............UAAAAG.........UCCG.C......UGGGAU
env-384  UCUACCACGCUAUUAAAUUUUUCAAACAGUCCCUUUUAGCUGGAAAAGUUUAUUUCACUGUAAGAUGCUAUGAAUAAAAACAAUUGGCGGA............UAAAAA.........UCCG.C......UGGAAU
env-385  GGCGAAGCUUGAAAACAAUUGAACAUUGCCAGUUUAGCGCUGGAAAAGUUUAUUUUGCCGCGUGAAGCUAUGAAUCAAAGCUACUGGCGGAU...........AAGU..........GUCCG.U......UGGAAU
env-386  GGCGAAGCUUGAAGACAAAUGAACAUUGCCAGUUUAGCGCUGGAAAAGUUUAUUUUGCCGUGUGAAGGUAUGAAUCAAAGCUACUGGCGGAU...........AAGU..........GUCCG.U......UGGAAU
env-387  GGCGAAGCUUGAAAACAAUUGAACAUUGCCAGUUUAGCGUUGGAAAAGUUUAUUUUGCCGCGUGAAGCUAUGAAUCAAAGCUACUGGCGGAU...........AAGU..........GUCCG.U......UGGAAU
env-388  GUAAAAUUAACUUGAAGAAAAUGAAUAAGUGACUGUUUUCUGGAAAAGUUUAUUUUACUGCAUGAUGCUAUGAAUCAAAGCUAUUGGCGGA............UUAGAUG........UCCG.C......UGGGAU
env-390  GUAAAAUUAACUAAAAGAAAAUGAAUAAGUGACUGUUUUCUGGAAAAGUUUAUUUUACUGCAUGAUGCUAUGAAUCAAAGCUAUUGGCGGA............UUAGACA........UCCG.C......UGGGAU
env-392  GGCCGUGCUUGAGGAUAAGCCGAAAUAGGCUGUUCGCCUUUGGAAAAGUUUAUUUUGUCGGCUUGUGAUUGAUUGAGAGCCGAGCGGCGGA............CUUCA..........UCCG.C......UGGGAU
env-402  GGCCGUGCUUGAGGAUAAGCCGAAAUAGGCUGUUCGCCUUUGGAAAAGUUUAUUUUGUCGGCUUGUGAAUGAUUGAGAGCCGAGCGGCGGA............CUUCA..........UCCG.C......UGGGAU
env-406  GUUAAAAAAGCUGUGCUUGAGGCUAAAAAUGGUUAACUUGCUGGAAAAGUUUAUUUUUGUGGUUGGGCAAUCAUUGAGAGCCAGUGGCGGAU...........AGAA..........AUCCG.C......UGGGAU
env-408  GUUAAAAAAGCUGUGCUUGAGACUAAAAAUGGUUAACUUGCUGGAAAAGUUUAUUUUUGUGGUUGGGCAAUCAUUGAGAGCCAGUGGCGGAU...........GGAA..........AUCCG.C......UGGGAU
env-410  UUAAAAAGGCCGUGCUUGAGGCUAAGACCUAACAGCCUUGUUGGAAAAGUUUAUUUUGUUGGUUGGGGAAUCAUUGAGAGCCAGUGGCGGAU...........GAGA..........AUCCG.C......UGGGAU
env-412  ..........................................................AAUCAUUGAGAGCCAGUGGCGGAU...........GAGA..........AUCCG.C......UGGGAU
env-413  .......................................................GGGAAUCAUUGAGAGCCAGUGGCGGAU...........GAGA..........AUCCG.C......UGGGAU
env-414  AGAGCUUGAAGACAAGACAAGAUAGGCGCCUCAAGCUUUUUGGAAAAGUUUAUUUUGCUGGUUGGCGAAUCAUUGAAAGCCGAGCGGCGGAC...........UUU...........GUCCG.C......UGGGAU
env-416  GAGCUUGCAGACAAGGCAAAAUAGGCACUUCAAGCCCUUUUGGAAAAGUUUAUUUUGCUGGUUGGCGAAUCAUUGAAAGCCGAGCGGCGGAC...........UUU...........GUCCG.C......UGGGAU
env-418  GCUGAACUUGAGGACAAGCCGAAAUAGGCAGUUUGCCUCUUGGAAAAGUUUAUUUUGCUGGCUUGUGAAUGAUUGAGAGCCGAGCGGCGGAC...........AAG...........GUCCG.C.....UAGG.AU
env-420  AGCUGAACUUGAGGACAAGCCAAAAUAGGCAGUUUGCCUUUGGAAAAGUUUAUUUUGCUGGCUUGUGAAUGAUUGAGAGCCGAGCGGCGGAC...........AAG...........GUCCG.C......UGGGAU
env-422  GCUGAACUUGAGGACAAGCCAAAAUAGGCGGUUUGCCUUUUGGAAAAGUUUAUUUUGCUGGCUUGUGAAUGAUUGAGAGCCGAGCGGCGGAC...........AAG...........GUCCG.C......UGGGAU
env-424  UCAAGAAAUCUUGAAGGCUAGAAAAUGUUUGAAACAUCAUAGAAAAGGUUUAUUUUUAAGCACUGCGCUAACAUUCAAAAUUGCAGGCGGACU..........ACCAGA........GUCCG.C......UGGGAU
env-425  UUUAUAUAAGAGAGAGUAGUCUAAUACAGUAAUAGCGUUUUGGAAAGGUUUAUUUUUAGGCAUGGUGCUAAGAUGUAAAAUUGCAGGCGGAC...........CAGCAGA.......GUCCG.C......UGGGAU
env-427  UUUAUAUAAGAGAGAGUAGUCUAAUACAGCAAUAGCGUUUUGGAAAGGUUUAUUUUUAGGCAUGGUGCUAAGAUGUAAAAUUGCAGGCGGAC...........CAGCAGA.......GUCCG.C......UGGGAU
env-428  GCUUAAGAACCUUUCGUCACACGCACAGCGCUAGCGUUCUAGAAAAGGUUUAUUUUUAACCACGGCGCUAACAUUUAAAAUUGCAGGCGGAC...........CAACCGU.......GUCCG.C......UGGGAU
env-429  GUAUAGGGAGGCUAUAGUCUCUGCAGAGCACUAGGGUUCUGGAAAAAGUUUAUUUUUAACCACGGCGCUAACAUUCAAAAUUGCAGGCGGAC...........CAGCUGU.......GUCCG.C......UGGGAU
env-432  AUAUAGAGGGGCUAUAGUCUCCGCAGAGCACUAGUGUUCUGGAAAGGUUUAUUUUUAACCACGGCGCUAACAUUGCAGAAUUGCAGGCGGAC...........CAACCGU.......GUCCG.C......UGGGAU
env-433  AUAUAGAGGGGCUAUAGUCACCGCAGAGCACCAGAGUUCUGGAAAAGGUUUAUUUUUAACCACGGCGCUAACAUUCAAAAUUGCAGGCGGAC...........CAACCGU.......GUCCG.C......UGGGAU
env-438  UCAUUUUAAUUCGCACUUCUGAAGAGUGAUUUGACUUUUGUUGGAAAAGUUUAUUUCAUAGGUAAGUGUUAUGAAACAAAACAUUGGCGGAU...........AACA..........GUCCG.C......UGGAAU
env-439  UCAUUUUAAUUCGCACUUCUGAAGAGUGAUUUGACUUUUGUUGGAAAAGUUUAUUUCAUAGGUAACUGUUAUGAAACAAAACAUUGGCGGAU...........AACA..........GUCCG.C......UGGAAU
env-440  GGUUGUAGCUAAUAAAUUCAUUGUAGAAGGCUGACUGCGCGCGCGCAAAAGAUUUAUUAACAUUCGUCAUGUGUUACUGGCUGCUGGCGGAU...........GCCAA.........AUCCG.U......UGGAAU
env-441  UUGAACUGUGGCUAAUAAAUUCAUUGCUGGCGUAUGUUUAGAAAAAGCUUUAUUAGGAUUCUCAUUGUGUUAGCUACAAGCUGUUGGCGGAU...........GCUCG.........GUCCG.C......UGGAAU
env-442  ......GCUGUGGUUAAUAAAUUCAUUGCUUGUGCCUGUUGAGAAAAGCUUUAUUAGGAUUCUUGGCUUGUUACCUAANGCUGUUGGCGGAU...........AUU...........GUCCG.C......UGGAAU
env-443  ..............................................UUUUUUAGGAUUCUUGGCUUGUUACCUAAAGCUGUUGGCGGAU...........AUU...........GUCCG.C......UGGAAU
env-444  CCUAGUCUGUGGCUAAUAAAUUCACUGCUGGUCCAAGCUUAGAAAAAGCUUUAUUAGGACUCUUCAUUUGUUACCUAAAAUUGUUAGCGGA............CACGA..........UCCG.C......UGGAAU
env-446  CCUCAGCUGCGCACNCANUCGCUUGGACCCACGAAGCAUCAGAAAAAACUUUAUUAGGAUUCCUUGGGUGUAAGCUAGGACUACAGGCGGAUC..........CUUC..........GAUCCG.U......UGGGAU
env-447  CUACGGAUUUAUCCGUGGGUAGCUGACGUUAUGCUCUGUGCUGGAAAGGUUUAUUUUUAACCGUCUGAUUAGCAUGUGCGUUGCUUGCGGACG..........GUGA..........UGUCCG.C......UUGGGAU
env-449  CUCAGUGUAGGUAGUAAGCNCGCGUCGGCGGCGACGAAGCCGAAAAAGCUUUAAUAGACGUCUCGUUUGUUAGCUACAAACUAUUGGCGGAUG..........GGC...........CAUCCG.C......UGGAAU
env-450  CUCAGUGUAGGUAGUAAGCCCGCGUCGGCGGCGACGAAGCCGAAAAAGCUUUAAUAGACGUCUCGUUUGUUAGCUACAAACUAUUGGCGGAUG..........GGC...........CAUCCG.C......UGGAAU
env-453  ACGGAAACCAAGGGGCCAGGGGCCGGGGGGGCCCAAAAAAUCCCAACAGGUCGUGAAUUUUUGGGGAAACCCCCAAAACUUUGGGGGGGGG............CAGAA..........CCCG.C........GGGAA
Mvo-1-1  UUUUUAACAAUAUAUUUUUUGAACGUUAACUUAAUAUACUUGAGUGUCUUAAUUAGGUAAGAUAACCUAAAAGGUUCUCACAUUAACGGGA............GUAA...........UCCG.G....UAGGGAAU
env-454  UUUUUGACACUGAGAAAGAACAGAAGCACGUGAAUCGGUGACUUCGAUAGGUAUAUGUAGCAAUAACUGCAAACCAUACUGGAAUCACGGGA...........UCCG..........UCCCG.G.....AGAGGAAU
ume-1-1  CAUGUGCCAGCAGGCUGCUUUAGCGCCCGGACAAUACGAAAUAUUUAUAUUAUAGGACCUGUUUAUUGUUCCAACGGUUUCAGUCGCGGGA............UACGU..........UCCG.G......CCGGGAU
CMe-2-1  UGCUGAAGGAAUUGAAAUAAGAACCGAAGGUGGACGAAGAUUCAAGAAAUCUUUAACUACAAUGAAAUAGCUUAAAGUCCCUGGUAGCGGGA...........GAAAA.........UCCCG.C.......AGGAAU
Mev-1-1  UUGUAUUUUUGUAUGAUAAUAUAACUCAGUAUAGUUGCAUAAAAAACUUUAAAUCAUAUUAAGUUUGUAGUGCACUUGUUUCAUUUACGGGA...........UGUUA.........UCCCG.U.......CGGAAU
env-455  ..............GCUCUNAUGGGUCCNGNAACUGCUUCUGCCGGAGUACUUUAAUAGACCUCCGGUUAAAGUAUGUGGGAACAUGGGA............UCCG...........UCCC.A..UUGAAAGGAAU
env-457  ACACGGGUGCGAGUUGCUCUCAUGGGUCCCGCAACUGCUUCUGCCGGAGUACUUUAAUAGACCUCCGGUUAAAGUAUGUGGGAACAUGGGA............UCCG...........UCCC.A..UUGAAAGGAAU
env-460  GCGCAAAGGCUCGUGAUGCUGAUGGCUCCGUCAACUGCCCGUGCCGGAGUACUUUAAUAGACCUCCGGUUAAAGUAUGUGGGAACAUGGGA............UCCG...........UCCC.A..UUGAAAGGAAU
env-461  AACAAAGUGCUCGUAGUUCUGAUGGUUCCGGCACCUGCUUCUUCCGGAGUACUUUAAUAGACCUCCGGUUAAAGUAUGUGGGAACAUGGGA............UCCG...........UCCC.A..UUGAAAGGAAU
env-462  ..............................................................................GUCGCGGGA............GUGA...........UCCC.G......CCGGAAU
Mzh-1-1  ACAAAUGCUACUGCAUUACAUAGAAACCUGAACAAAACACAAAAACUUAAAUGCAAUCAGGUAUGUAGUAGCGGAUGUUUCAUUAUCGGGA............UAUAU..........UCCC.G......UAGGGAU
env-463  UCAAAGGAAUAAAAAACUUUGGACAUUCCCUAGAACGUCAAUAGUAACAUUUAAAUUCGAGAAUUACAUUUGAAACACUGAAGUGGCGGGA............GAGA...........UCCC.G......CUGGGAU
env-465  AUUUACUACUAUCCUUGCAGAUGAGAAGUACCUUUUCAUUUAGAUAGCUUUAAUACUGAACGGACUGCACUUCAUAUACCCUAACGUGGGA............GAGA...........UCCC.G......CUGGAAU
env-466  AGGGCGUAUAAUUCCUCUGGCAGCAAUGGAUUGACACGAUAAUGCCGGUGUACUUUAUUACCCUGCCAGUCCAAUUUUAUGGGAAUGCGGG............AUUCGUA........CCCG.U...UGAAAGGAAU
env-467  AAGCUUUAAAUACAUUGGGGUAAAAAUUCUGAUUGUACCUGGGAUAUCUUUAAUUACAAUGAAAUAGUUUAAAGCUUCUCAAGCGGCGGGA............GGAGA..........UCCC.G......CAGGGAU
env-469  CAUCGGGGAAGAGUGACCGGAAAGGGCACUCUCCAUUCCUGUAACCGCGCACUUUAAGUGCGUCCCUGCCGAUUCAUAUAGGUCUUACGGGA...........UGCG..........UCCCG.U.....CAAGGAAU
Mta-1-1  CAUCGGGGAAGAGUGACCGGAAAGGGCACUCUCCAUUCCUGCAACCGCGCACUUUAAGUGCGUCCCUGCCGAUUCAUAUAGGUCUUACGGGA...........UGCG..........UCCCG.U.....CAAGGAAU
env-472  CCUCACGCUCUCCGCGGGACACGCAAAACCGCCCGGUUCAGGGCAAAGCAUAUAAGCCGCGCAUCCCAACGGGGCGCCCCUAGUGCCGGGG............CCCG...........CCCC.G......GUGGGAU
env-473  AGCCAGAAAGGCACAAAAAGCACGUAAAACCGCCCGCUUCGGGACAAAGCAUAUAAACAGCCCUCACAAUGGGGCGCCCCUAGUGCCGGGG............UACG...........CCCC.G......GUGGGAU
env-474  ACCGUCUUCCCCCAUCUGUGAAUUUCUCGCCCAAAGAUACCGAAGCCCAUUGUUUUAAUACCCCAUCCAUCAUCGAAAUAUACAGACCACGGGAU...........AGU...........AUCCC.G.....AAAGGAAU
Mpa-1-1  GCGAUAUCCGGGUGAUCGAGAAGAUCAAGGGCUGACCAGGAUCACCGCUGUUAUUAAUAACCGGGUGGUCGACAUAUAAAGGAAUACGGGA............UCCG...........UCCC.G....UAAAGGAAU
env-475  GGCAGUGAACUCCCCCUGCCAAUUAUGGUCUCUCUCCGGCACCUGCCGAGUACUUUAAUACCCCUUCUGUCCAAUUCUAUAGAACCGCGGGA...........UUCG..........UCCCG.U...UAAAAGGAAU
env-476  GUCAUUAUUGCCGGUUAUAAGUGUCCAUGAAUGGAAUGAAUCCGUCCCCUCCCUUUAUUACCUCCUCCAUCCAAUUCUAUAGAACCGCGGGA...........UUCG..........UCCCG.U...UAAAAGGAAU
env-477  CGAUAAGGGUGCAGGCAGGAACCCCGGGAUACCAGGUUUCCGGCACCAUUCUUUUAAUAGGGCCCCGGUUAAUGUAUUAAGGAACACGGGA............UCCG...........UCCC.G....UUAAGGAAU
env-479  ACGGCGAAAAAGGGCGCGUGUUAAUUAUUCCGGUCUUUCUCCAGCCCCCACUCUUUAUUACCCCCAUAUCGUAGUAUAUAGGAAUGCGGGA...........UACG..........UCCCG.UGUUGAAAAGGAAU
env-480  GCGCAAGAGUACCUGAUAGUAUAAUGAUCCCAGCUGAUGCAGGAGCCGGUGUAUUUAAUAUGGCUCCGGUUAAAGUAUAUAGGAAUGCGGGA...........UACG..........UCCCG.U...UGAAAGGAAU
env-481  GCGCAAGAGUACCUGAUAGUAUAAUGAUCCCCGGAGAUGCAGGAGCCGGUGUAUUUAAUACGGCUCCGGUUAAAGUAUAUGGGAAUGCGGGA...........UACG..........UCCCG.U...UGAAAGGAAU
env-484  ........................................................GGCUCCGGUUAAAGUAUAUAGGAAUGCGGGA...........UACG..........UCCCG.U...UGAAAGGAAU
env-485  GCGCAAGAGUACUUGAUAGUAUAAUGAUCCCCGGAGAUGCAGGAGCCGGUGUAUUUAAUACGGCUCCGGUUAAAGUAUAUGGGAAUGCGGGA...........UACG..........UCCCG.U...UGAAAGGAAU
env-486  CACAAAGGUCCCUGACACCAGGAUCACCCGGAAGGGAGGAGGAGCCGGAGUACUUUAAUAGCCCUCCGGUUAAAGUAUGUGGGAAUACGGGA...........UCUG..........UCCCG.U...UGAAAGGAAU

         ........................................................................................<<<<<<<<<<<<<<<<<<<<<<<<<<<<<<<<<<<..........>>>>>>>>>>>>>>>>>>>>>>>>>>>>>>>>>>.>........<<...
         ........................................................................................22222..1111..................1..1............1..1..................1111..2222.2........12...
         ●●●●●●●●●●●●●●●●●●●●●●●●●●●●●●●●●●●●●●●●●●●●●●●●●●●●●●●●●●●●●●●●●●●●●●●●●●●●●●●●●●Y●●●YR●●●G●-----------------------------●●●○----------------------------------●C●●-●------○●GGRAU
                                                                                                5'
```

```
env-487  UUGAAAAGCGCAAAGGCUGAUCAGGGAUCCUCCGGGAAGAGGAGCCGGAGUACUUUAAUAUCCGCCCGGUUAAAGUAUGUGGGAAUACGGGA..........UCCG..........UCCCG.U...UGAAAGGAAU
env-488  AGCGCAAGGGUACCUGAUUAUCAAAUCCUCUUGAUGAGCAGGAGCCGGAGUACUUUAAUAGCCCUCGGGUUAAAGUAUGUGGGAAUACGGGA..........UCCG..........UCCCG.U...UGAAAGGAAU
env-489  GCGCAAGGGCCCUUAAUUAUCAGAAUCCUCUGGAGUUGCAGGAGCCGGAGUACUUUAAUAGCCCUCCGGUUAAAGUAUGUGAGAAUACGGGA..........UCCG..........UCCCG.U...UGAAAGGAAU
env-490  AUUGAAAAGUGCAAGGGCACUUAGUUAUCAAAUCCUCUGGAAUGCCGGAGUACUUUAAUAGCCAUCCGGUUAAAGUAUGUGGGAAUACGGGA..........UCCG..........UCCCG.U...UGAAAGGAAU
env-491  AGCGUAAGGGCACCUAAUUAUCAAAUCCUCUGGAGUUGCAGGAGCCGGAGUACUUUAAUAGCCCUCCGGUUAAAGUAUGUGGGAAUACGGGA..........UCCG..........UCCCG.U...UGAAAGGAAU
env-492  AGCGAAAGGGCACCUAAUUAUCAAAUCCUCUGGAGUUGCAGGAGCCGGAGUACUUUAAUAGCCCUCCGGUUAAAGUAUGUGGGAAUACGGGA..........UCCG..........UCCCG.U...UGAAAGGAAU
env-495  CGAAAGGGUUCCGCUGUAUAGAUCCCCUGCGGCAACGGGAAGAGCCGGUGUACUUUAAUAGUCCACCGGUUAAAGUAUGUGGGAAUGCGGGA..........UGCG..........UCCCG.U...UGAAAGGAAU
env-496  ............................GGACGAUAAGCCGUUGCCGCAGUUCUUUAAUAUCCAUCCACCCUAUCUAUAAAGGCAUACGGGA..........UGCG..........UCCCG.U...UGAAAGGAAU
env-497  UCCGGUUUUUAGUCUUCCAUUUUUGACUUUUUUCUUGCCGGGGCGGGAGAGCUUUAAUAUCACGUGUAUCCUAGUAUACAGGAACACGGGA..........UCCG..........UCCC.G.UUGGAAAGGAAU
env-499  ACGCAAUAUGCAGCACGCAUACUAUCUCCAGUCCGCCAUCCCCCCGCCUCCUUUAUUACCCCCUUCAUCCAAUGUAUACAGGCAUACGGGA..........UUCG..........UCCC.G.UAGAGAAGGAAU
env-500  UGAACAGGUAAGAGAGACGGUCUCAGAACAGAUGGCCCGGAUGCCACAGCUCUUUAAUAUCCGUCCACCCUAUCUAUACAGGCAUACGGGA..........UCCG..........UCCC.G.GUGAAAAGGAAU
env-502  GUUUGGAGAGAUGGUGGGUACCCCAUCGGGCGAUAAGCCACUGCCACAGCUCUUUAAUAUCCGUCCACCCUAUCUAUACAGGCAUACGGGA..........UCCG..........UCCC.G.GUGAAAAGGAAU
env-503  ........................................................AUCCGUCCACCCUAUCUAUACAGGCAUACGGGA..........UCCG..........UCCC.G.AUGAAAAGGAAU
env-505  CGGAUUAUUUAUCGACAAAGUGGUGCCUGACCUUGCCCAAUUGCCACAGUUCUUUAAUAUCCGUCCAUCCUAUCUAUACAGGCAUACGGGA..........UCCG..........UCCC.G.AUGAAAAGGAAU
env-506  ...................................UGAUUUUUACCCCACUCUUUAUAUCCCUAUUUAUCCUAUGUAUACAGGCAUACGGGA..........UACUU.........UCCC.G.UAGACAAGGAAU
env-507  GGGUAUGUUCAGAACUGUGUUUUUCAGUUUUAAUGACCCCCGCUCCCCAUCCUUUAUUACCCUCCAUAUCCUAGUAUAUAGGAAUACGGGAU.........ACAA..........GUCCC.G.UGUAAAAGGAAU
env-508  CGGCGAAAAAGGGCGCGUGUUAAUUAUUCCGGUCUUUCUCCAGCCCCCACUCUUUAUUACCCCCCAUAUCGUAGUAUAUAGGAAUGCGGGA..........UACG..........UCCC.G.UGUUGAAGGAAU
env-509  .............CACCUCCCACGAGUCCACAAUAACUCCCGCCCCCAACUCUUUAUAACCCCCCUCAUCGUAGUAUAUAGGAAUGCGGGA..........UACG..........UCCC.GUAUGAAAAGGAAU
env-511  CAUACUGUUUCUGCACCUCCCACGAGUCCACAAUAACUCCCGCCCCCAACUCUUUAUAACCCCCCUCAUCGUAGUAUAUAGGAAUGCGGGA..........UACG..........UCCC.GUAUGAAAAGGAAU
env-512  UCUUCCGCAAAUAAUCCUCGAGAGAAAAAUGAGUAUCCCCACUCCCCAACUCUUUAUUACCCCCAAUGUCGUAGUAUAUAGGAAUGCGGGA..........UACG..........UCCC.GUGUGAAAAGGAAU
env-513  GAUCAAAAAACCGCACCAAUCAUCACCGUUCCUCUGCCUUGUGCCUGCAUCUUUUAAUACCCCUCAGGUUGUAGUAUAUAGGAAUGCGGGA..........UACG..........UCCC.GUGUGAAAAGGAAU
env-514  UUCUCCUGCAAAUAAUCCUCGAGGGAAAAAAUGAGUAUCCCCACCCCAACUCUUUAUAACCCCCCUCAUCGUAGUAUAUAGGAAUGCGGGA..........UACG..........UCCC.GUAUGAAAAGGAAU
env-515  GAUCAAAAAACCGCACNAAUCAUCACCGUUCCUCUGCCUUGUGCCUGCAUCUUUUAAUACCCCUCAGGUUGUAGUAUAUAGGAAUGCGGGAU.........AAC...........GUCCC.GUAUGAAAAGGAAU
env-516  UCUUCCGCAAAUAAUCCUCGAGAGAAAAAUGAGUAUCCCCACUCCCCAACUCUUUAUUACCCCCCAUAUCGUAGUAUAUAGGAAUGCGGGAU.........AAA...........GUCCC.GUGUGAAAAGGAAU
env-517  AUAUCAAAGUUGAAGAGAAACAAAAAGGGCACCACCUUUAAGGGUACGGCAAUAAAUAGCAGCGCACCCCCAUAAACUAAGGAAGACGGGA..........CCAG..........UCCC.G....UUAAGGAAU
env-522  UAUUGAAGGAAUUUCAGUACUUUCAAAAGAGAAAGGGACGCCGGAACCAAUCCUUUAAGUCCACAGGUGACGAAGAAUACAGAACACGGGA..........GCUU..........UCCC.G....UUAAGGAAU
env-525  UUGAAGGAAUCUCGGUUGUGAGGAAGGAGAAAAGCCUUGCUGAACCAUCAUCCUUUAAGUCUGCCGGUGACGAAUAAUAAAGAACACGGGA..........UCGC..........UCCC.G....UCAAGGAAU
env-528  ........GCUCUGUCCCGUUUGAAUUUUUCCACUGAAAAUUGCCCUCUCCCUUUAUUACCUCCUCCAUCCAAUUCUAUAGAACCGCGGGA..........UUCG..........UCCC.G..UUCAAAGGAAU
env-529  UCAUUCCUGCCGGUCACAGGUGUCUGUUAAUGGAAUGAAACCGUCCCCUCCCUUUAUUACCUCCUCCAUCCAAUUCUAUAGAACCGCGGGA..........UUCG..........UCCC.G..UUCAAAGGAAU
env-530  CAAUAACCUCUUUUUGGCAUCCCGGAAGAUCCCACGGGAUCUGCCCUAGUACUUUAAUACCCCUCCUGUCCAAUUCUAUAGAACCGCGGGA..........UUCG..........UCCC.G..UUCAAAGGAAU
env-531  GAAAACGAGCUCUGUCCCGUUUGAAUUUUUCCACUGAAAAUUGCCCUCUCCCUUUAUUACCUCCUCCAUCCAAUUCUAUAGAACCGCGGGA..........UUCG..........UCCC.G..UUCAAAGGAAU
env-532  ACUUGUUUCAGAAUUUUCAUGAAAAAUAUCCCGCUGAAAAUUGCCCUCUCCCUUUAUUACCUCCUCCAUCCAAUUCUAUAGAACCGCGGGA..........UUCG..........UCCC.G.UUACAAAGGAAU
CMe-1-1  GGGAAAGUGUGGUCUCCUUGUCAGUGAAGUGCUUGUGAAAUGGCCUGCGUACUUUAAUAUCCCCUUAUCCAAUUCUAUAGAACCGCGGGA..........UUCG..........UCCC.G.UUACAAAGGAAU
env-534  UCAUUUUUGCCGGUCAUUGGUGUCUGUGAAUGGCAGGAAUCCGCCCUCUCCCUUUAUUACCUCCUCCAUCCAAUUCUAUAGAACUGCGGGA..........UUCG..........UCCC.G.UUAAAAAGGAAU
env-535  GUGCCGUAAUCNCCCACUCUUUGUCCGCUUUUUCCCGUGCAACGCCGCGUACUUUAAUAUCCUCCCUGUCCAAUUCUAUAGAACUACGGGA..........UCCG..........UCCC.G.UUCUAAAGGAAU
env-536  UUGAGGGGAUCCGGGUUCUUUUAAAAGAGAAGGGCACCGCGUAACCAGACAUCUUUAUCUGCGCUCGUAACGAACAAAUACGCACACGGGA..........UCUA..........UCCC.G....UAAAGGAAU
env-537  UUGAGGGAGUUCGUGUUCUGUCAAAAGAGAAGGGCACCGCAUAACCAGACACCUUUAUCUGCGCUUGUAGCGAACAAAUACGCACACGGGA..........UCUU..........UCCC.G....UAAAGGAAU
env-538  AGGGUACUUGAAAAAGUAAAACAGGACCUGUAAGGCAGUCAUCAAGAGCAGAUAUAUAUCCUGUAUGAGAGACCUUAAUGGACUUUCGGGA..........UCCG..........UCCC.G.....UUAGGAAU
env-539  GGGUACUUGAAAAAGUAAAGCAGGACCUGUAAGGCAGUCAUCAGGAGCAGGUAUAUAUCCUGUCUAAGUGACCUUAAAUGGACUUUCGGGA..........UCCG..........UCCC.G.....UUAGGAAU
env-540  GGGUACUUGAAAAAGUAAAACAGGACCUGUAAGGCAGUCAUCAAGAGCAGAUAUAUAUCCUGUAUGAGAGACCUGUAAUGGACUUUCGGGA..........UCCG..........UCCC.G.....UUAGGAAU
env-541  ............AGUUGUAGAGAAGAUAAAACAUGGUCUCUCAGAGACAAAUUAAAUAUCUGUCAGAGUGACCUGUAAUGGACUCUCGGGA..........UUAG..........UCCC.G.....UUAGGUAU
env-542  UGCUCGAGAAAAGGAAAGGGUAGGGGCAGGCUCCGUUCCCGCGGGCAUGGCUUUAAGAAGCAUCCUGCCGAUAUAUACUGGUCUUACGGGA..........UCCG..........UCCC.G....UGAAGGAAU
env-543  UGCUCAGGAAACAGAAAGGAUAGGGGGCAGAUCCUUUCCCGCGGGCAUGAAUUUAAGAACCUUCCCGCAGAUAUAUACAGGUCUUACGGGA..........UCCG..........UCCC.G....UGAAGGAAU
env-544  ..................................................................CCACAAUAUAAGGAAUACGGGA..........UACG..........UCCC.G.....UAAGGAAU
env-545  AUCGAGGGGAUCCGGGUCGUGGAGAAGGUCAAGGGCUUGCCCGGGGCGCAGGCUUAAAAACCCUGGCGCCGAGAUAUAAAGGCACUCGGGA..........UCCG..........UCCC.G...UGCAAGGAAU
env-546  ............................UCAAGGGCGAGCCCGGGGCGCAGGCUUAAGAACCCUGGCGCCGAGAUAUAAAGGCACUCGGGA..........UCCG..........UCCC.G...UGCAAGGAAU
Mli-1-1  CGAGGGCAUCCGCGUGAUCGAGAAACGGAAGGGCGCGCUCUGACCGAGCGAUUAAUAUCUCUCUGGAACAUACCAAUACAGACCUGCGGGA..........UCAG..........UCCC.G....UGAAGGAAU
env-547  AUAUCCGCGUCAUCGAGAAACGAAAAGGAAGCAGUUGAGCAGGAAGGAAUCCUUUAUUUAUCCUCCGGUCGACCUGUUAAGAAUCACGGGA..........UACG..........UCCC.G....GGAAGGAAU
Mse-1-1  GGAGGGUCAAUAUACCGGAGCCCCCGGUGGGCGCGGGACCCUGGGGGAAUCCUUUAUUUGUGCUCCCGUCAACCUGUUAGGAACCACGGGA..........UACG..........UCCC.G....AAAAGGAAU
Mma-1-1  GGAGGGUCAAUAUACCGGAGCCCCCGGUGGGCGCGGGACCCCGGGGGAAUCCUUUAUUUGCGCUCCCGUCAACCUGUUAGGAACCACGGGA..........UACG..........UCCC.G....AAAAGGAAU
Msp-5-1  GGAGAAACGUAAAGGGGUCUAGGUCUUCCUGCAGGCAGGGGGGAGGCAAUCCUUUAUUUGCGCUCCCGUCAACCUGUUAGGAACCACGGGA..........UACG..........UCCC.G....AAAAGGAAU
env-552  CCUGCGGAGCGGAGUUGGAGCCCCCGAUGGGCGCGGGACCCCGGGGGAAUCCUUUAUUUGUGCUCCCGUCAACCUGUUAGGAACCACGGGA..........UACG..........UCCC.G....GAAAGGAAU
env-553  GGGCAAGGCGACCCGCCAGAUCUCUUGCAGGCAGGCUGACCGAAAGGGAUCCUUUAUCUGUUCUCUCGCCAACCUGUUAAGAAUCACGGGA..........UACG..........UCCCUG......AAGGAAU
env-559  ..............GCCAGAUCUCUUGCAGGCAGGCUGACCGAAAGGGAUCCUUUAUCUGUUCUCUCGCCAACCUGUUAAGAAUCACGGGA..........UACG..........UCCCUG......AAGGAAU
env-560  GGGCAAGGCGGCCCGCCAGACCUCUUGCAGGCAGGCUGACCGGAAGGGAUCCUUUAUCUGUUCUCUCGCCAACCUGUUAAGAAUCACGGGA..........UACG..........UCCCUG......AAGGAAU
env-561  GGGCAAGGCGACCCGCCAGAUCUCUUGCAGGCAGGCUGACCGAAAGGGAUCCUUUAUCUGUUCUCUCGNCAACCUGUUAAGAAUCACGGGA..........UACG..........UCCCUG......AAGGAAU
env-563  GGGCAAGGCGACCUGCCAGAUCUCUUGCAGGCAGGCUGACCGAAAGGGAUCCUUUAUCUGUUCUCUCGCCAACCUGUUAAGAAUCACGGGA..........UACG..........UCCCUG......AAGGAAU
env-564  ACACGGUAUCUAUGGGGAAUCGCCCCCGUGGGGGAGGGGGAGACGGAAUCCUUUAUCAGCGCCCCCGUCGAUCUAUAUGGAGCCNACGGGA..........UACG..........UCCC.G....GAAAGGAAU
env-565  ACACGGUAUCUAUGGGGAAUCGCCCCCGUGGGGGAGGGGGAGACGGAAUCCUUUAUCAGCGCCCCCGUCGAUCUAUAUGGAGCCAACGGGA..........UACG..........UCCC.G....GAAAGGAAU
env-567  GACACGGUAUCUAUGGGGAAUCGCCCCCGGGGGGAGGGGGAGACGGAAUCCUUUAUCAGCGCCCCCGUCGAUCUAUAUGGAGCCCACGGGA..........UACG..........UCCC.G....GAAAGGAAU
env-568  ..................................................................................ACGGGA..........UACG..........UCCC.G....GAAAGGAAU
env-570  GCGCAAGGGCGUGUAGGUCUUCCUGCGAGGCUGUGGGGCGGCAAAAGCAAUCCUUUAUUUGCGCUCCCGUCAACCUGUUAGGAACCACGGGA..........UACG..........UCCCG.G....AAAGGAAU
env-571  AGCGCAAAGGGGUCUAGAUCUCCCUGCGGGUGGGUGCCGGCGAAGAGAAUCCUUUAUCUAUGCUCCCGUCAACCUGUUAAAGACCACGGGA..........UACG..........UCCC.G....GAAAGGAAU
env-574  AGGCAAUCCGGGUGAUUUUGAAAGAGAAAUGCUGAUACCUGCGGGCAUGGGUUUAUCACGCUUCCUGUUGACAUGAUAUGGUCUUGCGGGA..........UGCG..........UCCC.G....UGAAGGAAU
env-575  AGGCAAUCCGGGUGAUUUUGAAAGAGAAAUGCUGAUACNUGCGGGCAUGGGUUUAUCACGCUUCCUGUUGACAUGAUAUGGUCUGACGGGA..........UGCG..........UCCC.G....UGAAGGAAU
env-576  ..................................AUACCUGCGGGCAUGGGUUUAUCACGCUUCCUGUUGACAUGAUAUGGUCUGACGGGA..........UGCG..........UCCC.G....UGAAGGAAU
env-577  AGGCAAUCCGGGUGAUUUUGAAAGAGAAAUGCUGAUACCUGCGGGCAUGGGUUUAUCACGCUUCCUGUUGAUAUGAUAUGGUCUUGCGGGA..........UGCG..........UCCC.G....UGAAGGAAU
env-578  GGGAACGCCAGGAGAAUAGCAAUCCAUUCCACGAUCGGGAUCACCGCGCAGUUUAAGUGUCCACCCGUCAAAUAUUAAAGGUCUUACGGGA..........UUCG..........UCCC.G...GGAUAGGAAU
env-579  AUUGUUGAAGAAAAGCGCAAAGGUGUCUGAAGGACCUUCUUUCUUCAGUGUAUUUUAAUAUCCCACACGUCGACAUAGUGNGGUCUACGGGA..........UUUG..........UCCCG.G....AAAGGAAU
env-580  AUUGUUGAAGAAAAGCGCAAAGGUGUCUGAAGGUCCUGCGUUCUUCAGUGUAUUUUAAUAUCCCGCAGGUCGAUAUUGUGAGGUCUACGGGA..........UUCG..........UCCCG.G....AAAGGAAU
env-581  ACGAAUAGAGGGACCGGCUCCCUGGACCCCAACAGUCCCUGGACCGCGCAUUUUAAUACCCCGAUUGACGAUUCUUAUAGGUCUUGCGGGA..........UACG..........UCCC.G...CGAAGGAAU
env-585  UGGCAGGUGAGUGAUCAGGGCGGGACCCCUCCUUCCCCCCGACCGCGCACUUUAAGUGUGCCCCUGCCGAUUGAUAUAGGUCUUACGGGA..........UGCG..........UCCC.G...UCAAGGAAU
env-587  AGCGGUACCAGCGAGCAGUAAAAAAGUACGCAGGUAGCGCUGACCGCGCAGUUUAAUAUCCCUGCAGGCGACUUCUAUAGGUCUUACGGGA..........UGCG..........UCCC.G...UCAAGGAAU
env-588  .......................................................UCCCUGCAGGCGACUUCUAUAGGUCUUACGGGA..........UACG..........UCCC.G...UGAAGGAAU
env-589  GUAAGUUAAAUUAAAGACUUUUCGUGUAAAGCGAUUUAUCUAAAAACCUUUAAAUGCAAUGAACGGAUAUGACGCAUGUUUCAUUUCGGGA..........UCCG..........UCCC.G.....UUGGAAU
env-590  UUAUAUAGUUUCCUCUUUAUUUCAUUUUCCGGCUCUUUUUUAAAAACCUUUAAAUGCAAUGAACGGAUACGACGUAUGUUUCAUUUCGGGA..........UCCG..........UCCC.G.....UUGGAAU
env-591  UUAUAUAGUUUCCUCUUUAUUUCAUUUUCCGGUUCUUUUUUAAAAACCUUUAAAUGCAAUGAACGGAUACGACGUAUGUUUCAUUUCGGGA..........UACG..........UCCC.G.....UUGGAAU
env-594  GGUAUGUAUAUUUUUUGCAACGGCAGGUAAGUAUUGCGGUAAGUAACCUUUAAAUGCAAUGAACGAAUACAGCCAAUGUUUCAUUUCGGGA..........UCCG..........UCCC.G.....UUGGAAU
env-595  UUCAAAGGAAUUGAGGGGUUUUCGUGCAAAGCCUAAUUUUUAAAAACCUUUAAAUGCAAUGAACGGAUAUGACGCAUGUUUCAUUUCGGGA..........UCCG..........UCCC.G.....UUGGAAU
env-596  UUUUUGUUNAUUUUUCAGCUUCNUUUUGUCUUUUACUUUUUAAAAACCUUUAAAUGCAAUGAACGGAUAUGACGCAUGUUUCAUUUCGGGA..........UCCG..........UCCC.G.....UUGGAAU
env-597  UUUUUGUUCAUUUUUUUAGUUUCGUUUUGUCUUUUACUUUUUAAAAACCUUUAAAUGCAAUGAACGGAUAUGACGCAUGUUUCAUUUCGGGA..........UCCG..........UCCC.G.....UUGGAAU
env-598  UUGGUGGAAUUUGAAGUUGAAUGGUUUCACAAAUAAUUUUUAAAAACCUUUAAAUGCAAUGAACGGAUAUGACGCAUGUUUCAUUUCGGGA..........UCCG..........UCCC.G.....UUGGAAU
env-599  UUUUUGUUUAUUUUUCAGCUUCGUUUUGUCUUUUACUUUUUAAAAACCUUUAAAUGCAAUGAACGGAUAUGACGCAUGUUUCAUUUCGGGA..........UCCG..........UCCC.G.....UUGGAAU

         ..........................................................................................<<<<<<<<<<<<<<<<<<<<<<<<<<<<<<<<<<<<<..........>>>>>>>>>>>>>>>>>>>>>>>>>>>>>>>>>>>.>.........<<...
         ..........................................................................................22222..1111..................1..1............1..1...................1111..2222.2.........12...
         ..........................................................................Y...YR...G.-------------------------------...------------------------------------.C..-.-------..GGRAU
                                                                                                   5'
```

```
env-602   UUUUACACAACUUUGACUUUUUACACAAAGCCUACUUUUUAAAAACCUUUAAAUGCAAUGAACGGAUAUGACGCAUGUUUCAUUUCGGGA..............................UCCG...................................UCCC.G......UUGGAAU
env-603   UCUUUCAUUUCUUUUUCAUUUUUCUUUCUGUUUCUUUUUUUAAAAACCUUUAAAUGCAAUGAACGGAUAUGACGCAUGUUUCAUUUCGGGA..............................UCCG...................................UCCC.G......UUGGAAU
env-604   UUUUUUUCAUUUCUUUUUCAUUUUCCUUUCUGUUUUUUUUUUAAAAACCUUUAAAUGCAAUGAACGGAUAUGACGCAUGUUUCAUUUCGGGA..............................UCCG...................................UCCC.G......UUGGAAU
env-605   CUUUUCAUUUCCUUUUCAUUUUCCUUUCUGUUUCUUUUUUUAAAAACCUUUAAAUGCAAUGAACGGAUAUGACGCAUGUUUCAUUUCGGGA..............................UCCG...................................UCCC.G......UUGGAAU
env-606   UAUUUCAUUUUUGUUUUUUGUUUUUUGUUUUUUGUUUUUUUAAAAACCUUUAAAUGCAAUGAACGGUUAUGACGCAUGUUUCAUUUCGGGA..............................UCCG...................................UCCC.G......UUGGAAU
env-607   UUUUUAUUAUUUUUUAUUGCAUUUUUGUUUUUUGUUUUUUUAAAAACCUUUAAAUGCAAUGAACGGAUAUGACGCAUGUUUCAUUUCGGGA..............................UCCG...................................UCCC.G......UUGGAAU
env-608   GUUCUUAACUUGUUUUUCCUAUAUUUUUACUUCAUUUAUUUAAAAACCUUUAAAUGCAAUGAACGGAUAUGACGCAUGUUUCAUUUCGGGA..............................UCCG...................................UCCC.G......UUGGAAU
env-609   AUUGGUUGAAUUUGAAGUUGAAUGGUUUCGCAAAUCCCUUUAAAAACCUUUAAAUGCAAUGAACGGUUAUGACGCAUGUUUCAUUUCGGGA..............................UCCG...................................UCCC.G......UUGGAAU
env-610   UCUUUCAUUUCUUUUUCAUUUUUCUUUCUGUUUCUUUUUUUAAAAACCUUUAAAUGCAAUGAACGGAUAUGACGCAUGUUUCAUUUCGGGA..............................UCCG...................................UCCC.G......UUGGAAU
env-611   UUUUAUUUUUGUUUUUGUUUUUGUUUUUUAUUUUUUCUUUUAAAAACCUUUAAAUGCAAUGAACGGAUAUGACGCAUGUUUCAUUUCGGGA..............................UCCG...................................UCCC.G......UUGGAAU
env-612   .....................................UUUAAAAACCUUUAAAUGCAAUGAACGGUUAUGACGCAUGUUUCAUUUCGGGA..............................UCCG...................................UCCC.G......UUGGAAU
env-613   .....................................................UGCAAUGAACGGAUAUGACGCAUGUUUCAUUUCGGGA..............................UCCG...................................UCCC.G......UUGGAAU
env-616   .......................................................GCCUUCUAUUGGCCAUCGCAGUUUCAGCAGCGGGA..............................UGCG...................................UCCC.G......CCGGAAU
Mco-1-1   CCAUCGUUUUUUUAUUGCCAACCAUCUUAACACUUCGAAACUUUUAUAUGCUUGUAGCCUUCUAUUGGCCAUCGCAGUUUCAGCAGCGGGA..............................UGCG...................................UCCC.G......CCGGAAU
env-617   ...................UUUACUUUACAAUCUAUACAAAAACUUAAAUCCUAUCAGGUAAUUAGAUACCUCGUGUUUCAUUAUCGGGAU.............................AAA...................................AUCCC.G......UUGGAAU
Mps-1-1   CUUUUUUCUUUAACGAAGCAAGCCAGCUUAUUUUCCUGUAAGAAAGUUUAUAUCAUAUCAGGUGGGUACUAGUCCUGUUUCAUUGUCGGGA..............................UGUG...................................UCCC.G......UGGGAAU
env-619   AUUUUCGUGCUUCCCUGAAAAGCUUCAGGCUUUAUCCGGUAAGAAAGUUUAAAUCAUAUCAGGGGAUACUACACCUGUUUCAUUGUCGGGA..............................UAUG...................................UCCC.G......UGGGAAU
env-620   AUUUUCAUGCUUUCCCAAAAAGCCUCAGGCUUUAUCCGGUAAGAAAGUUUAAAUCAUAUCAGGGGAUACUACACCUGUUUCAUUGUCGGGA..............................UAUG...................................UCCC.G......UGGGAAU
Mti-1-1   UUUUUCUAUAGAAAAUUGUAUUUCUAUUCCCUGGUUGUGCAAAGGUUUAAAUUCAAUUGGGUUAAGUAGUAGUCCUGUUUCAUUGCCGGGA..............................UUAG...................................UCCC.G......UGGGAAU
env-621   .......................................................AUCAGGUUGGUACUAGUCCUGUUUCAUUGUCGGGA..............................UCUG...................................UCCC.G......UGGGAAU
Mba-2-1   CUUUGUCCAGGUAUUUCACUUUUAAUUGGCCCAUUCACGGGCAAAGCUUUAAAUCAGAUGGUUCAGUUUAUCCCAUGUUUCAUUUUCGGGAU.............................UAG...................................GUCCC.G......UUGGAAU
env-622   UUUUGAAGAAUUAUUUGCUUUUGAAAUGUCUUUGAGGGGGCAAAGCUUUAAAUCAGAUUAUUCCUUUGUAUCGCAUGUUUCAUUUUCGGGA..............................UUAGU..................................UCCC.G......UUGGAGU
env-623   AUAUUAUUUUGCUACAACUACUUAAUCGAGCUGUGUACGGGCAAAGCUUUAAAUCAGAUGGUUCAGUUUAUCCCAUGUUUCAUUUUCGGGAU.............................UAG...................................AUCCC.G......UUGGAAU
env-624   CUUCUUGUGACUUUUUUCUUCUAAAGAGACCUUGGUGGGGCAAAGCUUUAAAUCUGAUGAUUCAGUUGUAUCGCAUGUUUCAUUUUCGGGAU.............................UAG...................................GUCCC.G......UUGGGAU
env-625   CUCUUUUUGCGGAUUUUACCUUUAAUAGCUUUUGUAUGGGCAAAGCUUUAAAUCAAAUGAUUCAGUUUAUCGCAUGUUUCAUUUUUCGGGAU.............................UAG...................................GUCCC.G......UUGGAAU
Mho-2-1   UAGGGUUUAUCCGGGGUUUUCUGACCGUUUACUUUGCAGGCAAAGCUUUAAAUCAGAUGAUUCAGUUGUAUCGCAUGUUUCAUUUUCGGGAU.............................UAG...................................AUCCC.G......UUGGGAU
env-628   UUUUUGCUGAUUCUUUGCUCUCAGAGGGCCUGUGCAAGGGCAAAGCUUUAAAUCAGAUGAUUCAGUUGUAUCGCAUGUUUCAUUUUCGGGAU.............................UAG...................................GUCCC.G......UUGGAAU
env-629   CUUUGUCCAGGUAUUUCACUUUUAAUUAGCCCAUUCAUGGGCAAAGCUUUAAAUCAGAUGGUUCAGUUUAUCCCAUGUUUCAUUUUCGGGAU.............................UAG...................................GUCCC.G......UUGGAAU
env-630   ..............................UUUGCAUAGGCAAAGCUUUAAAUCAAAUGAUUCAGUUUAUCGCAUGUUUCAUUUUCGGGAU.............................UAG...................................AUCCC.G......UUGGAAU
Mth-1-1   GCUUUUUGUGGAUUUUUUACCCUUAAUAGCUUUUGCAUAGGCAAAGCUUUAAAUCAAAUGAUUCAGUUUAUCGCAUGUUUCAUUUUCGGGAU.............................UAG...................................AUCCC.G......UUGGAAU
Msp-13-1  UCUUUUUUGUGGAUUUUUCACUUAAUCGAGCUUUGUACGGGCAAAGCUUUAAAUCAAAUGGUUCAGUUUAUCGCAUGUUUCAUUUUCGGGAU.............................UCG...................................GUCCC.G......UUGGAAU
Mva-1-1   UCUUUUUUGUGGAUUUUUCACUUAAUCGAGCUUUGUACGGGCAAAGCUUUAAAUCAAAUGGUUCAGUUUAUCGCAUGUUUCAUUUUCGGGAU.............................UAG...................................GUCCC.G......UUGGAAU
Mba-4-1   CUUUUUGCGUAGAUUUUUCACUUAAUCGAGCUAUGUACGGGCAAAGCUUUAAAUCAGAUGGUUCAGUUUAUCCCAUGUUUCAUUUUCGGGAU.............................UAG...................................AUCCC.G......UUGGAAU
env-631   CUUUUUGUGUGGAUUCUUCACUUAAUCGAGCUGUGUACGGGCAAAGCUUUAAAUCAGAUGGUUCAGUUUAUCCCAUGUUUCAUUUUCGGGAU.............................UAG...................................AUCCC.G......UUGGAAU
env-633   CGGAUUUUUGAUUUCUACUUCUCAAAGGGCUUUUGGAGGGGCAAAGCUUUAAAUCAGAUGAUUCAGUUUAUCGCAUGUUUCAUUUUCGGGAU.............................UCG...................................AUCCC.G......UUGGAAU
Mla-1-1   UUUUUGCGGAUUUUUUGCUUUUAAAGGGUUUUUGAAAGGGCAAAGCUUUAAAUCAGAUGAUUCAGUUGUAUCGCAUGUUUCAUUUUCGGGAU.............................UAG...................................AUCCC.G......UCGGAAU
Mba-5-1   CUUUUUGCGUAGAGUUUUCACUUAAUCGAGCUGUUUAUGGGCAAAGCUUUAAAUCAGAUGGUUCAGUUUAUCCCAUGUUUCAUUUUCGGGAU.............................UAG...................................AUCCC.G......UUGGAAU
Msp-7-1   UUUUUGCGGAUUCUUUGCUUUUAAAGGGCUUUUGAAGGGGCAAAGCUUUAAAUCAGAUGAUUCAGUUGUAUCGCAUGUUUCAUUUUCGGGAU.............................UAG...................................AUCCC.G......UUGGAAU
Msp-16-1  UGAGGUUCACUCGGGUUUUUUCGGUCAUUUACUUUGCAGGCAAAGCUUUAAAUCAGAUGAUUCAGUUGUAUCGCAUGUUUCAUUUUCGGGAU.............................UAG...................................AUCCC.G......UUGGAAU
env-634   UUUUCUGCGGAUUUCUACCUUUAAAAGAGCUUUUACACGGGCAAAGCUUUAAAUCAAAUGAUUCAGUUUAUCGCAUGUUUCAUUUCCGGGAU.............................UAG...................................AUCCC.G......UUGGAAU
Mso-1-1   UGACUUAUUGACUGAUUCUUCCUGAAAGCUUUGAAGGGGGCAAAGCUUUAAAUCAAAUGAUUCAGUUGUAUCGCAUGUUUCAUUUUCGGGAU.............................UAG...................................GUCCC.G......UUGGGAU
Mma-3-1   CUGACUUAUUGACUGAUUCUUCCUGAAAGCUUUGAAGGGGGCAAAGCUUUAAAUCAAAUGAUUCAGUUUAUCGCAUGUUUCAUUUUCGGGAU.............................UAG...................................GUCCC.G......UUGGGAU
Mac-1-1   ACUGAGGGUCCUCCAGAUUUCAGGUAAUUUGCUCUUCAGGCAAAGCUUUAAAUCAGAUGAUUCAGUUGUAUCGCAUGUUUCAUUUUCGGGAU.............................UAG...................................GUCCC.G......UUGGAAU
Msi-2-1   ACUGAGGGUCCUCCAGAUUUCAGGUAAUUUGUUCUUCAGGCAAAGCUUUAAAUCAGAUGAUUCAGUUGUAUCGCAUGUUUCAUUUUCGGGAU.............................UAG...................................GUCCC.G......UUGGAAU
env-635   ............AGCCUCCUAUUAAAUGGCCAUGAAAAGGCAAAGCUUUAAAUCAGAUGAUUCAUUUGUAUUCAUGUUUCAUUUCCGGGAU.............................UCG...................................AUCCC.G......UUGGGAU
Msi-1-1   ACUGAGGGUCCUCCAGAUUUCAGGUAAUUUGUUCUUCAGGCAAAGCUUUAAAUCAGAUGAUUCAGUUGUAUCGCAUGUUUCAUUUUCGGGAU.............................UAG...................................GUCCC.G......UUGGAAU
env-636   ACUGAGGAUCCUCCAGAUUUCAGGUAAUUUGUUCUUCAGGCAAAGCUUUAAAUCAGAUGAUUCAGUUGUAUCGCAUGUUUCAUUUUCGGGAU.............................UAG...................................GUCCC.G......UUGGAAU
Msp-14-1  UUCAGGGGCCGUGGCUGUCCUGAGGGAGUUUUUUUGCGGGCAAAGCUUUAAAUCAGAUGAUUCAGUUCUAACGCAUGUUUCAUUUUCGGGA..............................UUAGC..................................UCCC.G......UAGGAAU
env-637   UAAAUUGUUAGAAUCGUAUUGUUUGACAGCUGGAAUUUAGUAAAAACAUUUAAAUCAUAUCAUGUGCGUAGUAAUCACGUUUCAUUCGGGA..............................GGAA...................................UCCC.G......UAGGAAU
env-638   UAAAAUGUUAGAAUCGUAUUGUUUGACAGCUGGAAUUUAGUAAAAACAUUUAAAUCAUAUCAUGUGCGUAGUAAUCACGUUUCAUUCGGGA..............................GGAA...................................UCCC.G......UAGGAAU
env-639   AUAGAACAUUGGAAUUGGUCGUUUAAAAGCUGAAAGUUAGCGAAAACAUUUAAAUCAUAUCAUGUGUGUACUAAUCACGUUUCAUUCGGGA..............................GGAA...................................UCCC.G......UAGGAAU
env-640   UAGAAUAUCUGGAAACAUAUGGUUUAAGAGAUACAUUUGGCAAAAACAUUUAAAUCAUAUCAUGUGUGUAGUAAUGUCGUUUCAUUCGGGA..............................GGGA...................................UCCC.G......UGGGAAU
env-641   UGACAUAAGAAUUGGAAGUAUGAUUUAAAAGGGCAUUUGGCAAAAACAUUUAAAUCAUAUCAUGUGCGUAGUAGUUUCGUUUCAUUCGGGA..............................GCUA...................................UCCC.G......UGGGAAU
Mbu-1-1   UACUGAUCAACAAUAUUAUUCUUUCAGUCCUGUCAGAUAAAAAGGUUUAAAUGCCAUAGUGUAUGUACUAAUCGAUGUUUCAUUGUCGGGAU.............................ACU...................................AUCCC.G......UGGGAAU
env-642   UGAUCGGUUAUGCCAUCAUCUUUUCGACUCUCUUGGAUACAAAGACUUAAAUGUCAUAGUGUAUGUAGUAAUCGAUGUUUCAUCGUCGGGAU.............................AAU...................................GUCCC.G......UAGGAGU
Mme-2-1   AUAUCAGGUAGCUCUGCCUAAUUUGUAUUUCUUCUCAUACAAAGCUUUAAAUGGUAUGUUGUAUGUACUAACCGAUGUUUCAUUGUCGGGAU.............................GAGA..................................GUCCC.G......UAGGAGU
Mme-1-1   AAAUCGAGUAGCUCUGCAUCUUUUGGUUUUCUUUCCAUACAAAGCUUUAAAUGCUAUGUUGUAUGUACUAACCGAUGUUUCAUUGUCGGGAU.............................GAGA..................................GUCCC.G......UAGGAGU
Msh-1-1   GCUUCGCAUACCCGCAAAACGUUAAGUGAAGUUCCCAUCGGUAUGGCAAACCUUUAAAAGCGAUGCCGAUGUUGGGUGCGUGGCUGCGGGA..............................GGUG...................................UCCC.G......CCGGGAU
env-643   UUCUAUGAUUUAAUAUAAUAUAAUGAGAUUAUAAUUUUUUAGGCAUAUCAUCUUUAACGAUAAUUUUUAUUAAAAAAACUCUUUAUGGGAU..............................AAU....................................AUCC.C.......UGG.AU
env-644   AUGGCGAUUAUUAUCUGAUUUUAUAAUUCUUAUAAAAAUCGAUAAUCUGCUGUAAAAUUAACGAUAAUUUUUAAAAAACUCUUUAUAGGAC..............................UAUU...................................GUCC.U.......UGG.AU
env-645   AACUCCUGUCAGGGCCCAAAACCUUUUUAAGGUGUUAUGACCAACACUUGGUUACUGUAAAUUUAAAGUAUAACCUACUUUUUUUAGGGAC..............................ACA....................................GUCC.C......AUGG.AU
env-646   AAUUCCUGUCAGGGCCCAAAACCUUUUUAAGGUGUUAUGACCAAGACUUGGUUACUGUAAAUUUAAAGUAUAACCUACUUUUUUUAGGGAC..............................ACU....................................GUCC.C......AUGG.AU
env-647   UAUUUUAAAAUUAACCAUCAUAUUUAUAAAUAAUUAAAACAUAUAUUUUACUGUCGUUAGUUUACUAACGAUUAACAACUUUUUUUGGGA...............................UAAAU...................................UCC.C.......UGG.AU
env-650   UAAGAAAAGGAAAACCUUUAUAAAUAAAUAUAACCAAUAUUUUAUUAUCAUUAAAAUAUUAAUGAUUAUUUUUUAAAUACUCUUUUAGGGA..............................UAAUU..................................UCCC.U.......UGG.AU
env-651   AUUUUUUCCUCUUAAACCCAUCAUUUCUGGCAGCUGACAGCAACCUUUUUAAAUGAUUACUCUAAAACUAAUAUCACCUAUUUUUCCGGGAU.............................CACG..................................AUCCC.A.......UGG.AU
env-656   UGCCAGUUUUAACGAUACCUUUAUAAAUAAACAAAACAAAAUAUUUUAUUACUGUUGUUUUAUGGCAGUGUUAAAAAUACUUUUUUUGGGGA.............................UAAG..................................UUCCC.A.....UAUGG.AU
env-657   AAUCUCUAAUUUCAACGAUACUUUUAUAAAUGCAUAAAACAAAUAUUUUAUUACUGUUAUUUUUAACAGUUAUAAAUUACUUUUUUUGGGA..............................UAAGU..................................UCCC.A.....UAUGG.AU
env-658   NNNNNNNNNNNNUAGCGAUACUUUUAUAAAUAAUUAAAACAAAUAUUUGAUUACUGUUAUUUUUAACAGUGCUAAAAUACUUUUUUUGGGA..............................UAAGU..................................UCCC.A.....UAUGG.AU
Mve-1-1   ACUUUUAAUUUUGGUUUAAAGCACAACCUUUUUAUACUAACUUAUUCUAAUAAUUGAUCAUCACCAAUGGUGAAAACAACUUUUUUUGGGAU.............................AUA...................................AUCCC.A.......CGG.AU
env-659   GAAUUUAAUUUAAUUAAACACAACCUUUUUAUGUAAACUUAAUCUAAUAAUGAUCAUCACUACUUUAGUGAAUACAAUCUUUUUUUGGGAU.............................UUA...................................AUCCC.A.......UGG.AU
env-660   AAUUUUUGCAAAAUAAACAUAACCUUUUUAAAUAGACCUGACCAAUAUUGAUCACCGCCAAAUUAGGCGAUUUAACCUACUUUUUUUGGGAC.............................CUG...................................GUCCC.A.......UGG.AU
Msp-4-1   GAAUAGCAACCUUUUUAAAUGCAUUUAACUAAUAAUGAUCAUCGCUAAUUUUUGGUGAUAUACUAAAUUAUAUAACCUAACUUUUUUGAGAU.............................GUAA..................................GUCUC.A.......UGG.AU
env-661   UUUUCACCCUCUGGAACAUAACCUUUUUAAGGGCAUGUGAUCAACAUUGUGUUACCGCCAAUAUGGCGACUGUGACCUACUUUUUUUGGGACC............................AAUUAU...............................GAUCCC.A.......UGG.AU
env-663   UUUUCACCCCUGGGAGCACAACCUUUUUAAGGACAUAUGAUCAACAUUGUGUUACCGCCAAUCUGGCGAGUGUGACCUACUUUUUUUGGGACC............................AAUUAU...............................GAUCCC.A.......UGG.AU
env-664   ....................CCUUUUUAAGGACAUAUGAUCAACAUUGUGUUACCGCCAAUCUGGCGAGUGUGACCUACUUUUUUUGGGACC............................AAUUAU...............................GAUCCC.A.......UGG.AU
env-665   ACACUUACCAGAAUCCAAACCUUUUUAAAGAGAUAUAACCAACAUUGUGUUACCGCCAAUCUGGCGUGUUUUGACCUACUUUUUUUUGGGAUC............................ACAG..................................GUCCC.A.......UGG.AU
env-670   ACACUUACCAGAAUCCAAACCUUUUUAAAGAGAUAUAACCAACAUUGUGUUACCGCCAAUCUGGCGUGUUUUGACCUACUUUUUUUUGGGAUC............................ACAG..................................GUCCC.A.......UGG.AU
env-671   AUUUUUACCCCGGAACACAACCUUUUUAAGGGCAUGUGAUCAACAUUGUGUUACCGCCAAUAUGGGCGUAUGUGACCUACUUUUUUUGGGACC............................AAUAAU...............................GAUCCC.A.......UGG.AU
env-672   GAUUUUUACCCCGGAACACAACCUUUUUAAGGGCAUGUGAUCAACAUUGUGUUACCGCCAAUAUGGCGUAUGUGACCUACUUUUUUUGGGACC............................AAUAAU...............................GAUCCC.A.......UGG.AU
Mfo-2-1   AUUUUAUGCCCCUGAACACAACCUUUUUAAGGGCAUGUGAUCAACAUUGUGUUACCGCCAAUAUGGCGUAAGUGACCUACUUUUUUUGGGACC............................CCUAAU...............................GAUCCC.A.......UGG.AU
env-673   ...........................................................AUAUGGCGUAUGUGACCUACUUUUUUUGGGACC............................CCUAAU...............................GAUCCC.A.......UGG.AU
env-674   UUUUUCAUCCGAGGAGCACAACCUUUUUAAGGGUAUGUGAUCAACAUUGUGUUACCGCCAAUCUGGCGGGUUUGACCUACUUUUUUUGGGACC............................UAUUAU...............................GAUCCC.A.......UGG.AU
Msp-2-1   UUUCCACUUCUCUAACACAAACCUUUUUAAGGGCAUGUGAUCAACAUUGUGUUACCGCCAAUAUGGCGUGUUUGACCUACUUUUUUUGGGACCA...........................GUAA................................UGAUCCC.A.......UGG.AU

          .....................................................................................<<<<<<<<<<<<<<<<<<<<<<<<<<<<<<<<<<<.........>>>>>>>>>>>>>>>>>>>>>>>>>>>>>>>>>>.>........<<...
          .....................................................................................22222..1111..................1..1............1..1..................1111..2222.2........12...
          ●●●●●●●●●●●●●●●●●●●●●●●●●●●●●●●●●●●●●●●●●●●●●●●●●●●●●●●●●●●●●●●●●●●●●●●●●●●●●●●Y●●●YR●●●G●------------------------------●●●○-----------------------------------●C●●-●------○●GGRAU
                                                                                             5′
```

```
Mfo-1-1     UUCCCUUAUCCCCAACACAAACCUUUUUAAGGGCAUGUGAUCAACAUUGUGUUACCGCCAAUCUGGCGUGUUUGACCUACUUUUUUUGGGACCA..........................GUUA................................UGAUCCC.A......UGG.AU
env-675     AAUGUCAAAUUUUUUAGAGCACAACCUUUUUAUACUUACUUAUUCUAAUAGUUGAUCAUCACCAACUGGUGACAAACAACUUUUUUUGGGA...........................AACAA...................................UCCC.A......UGG.AU
env-676     AAUGUUUUACAUUUAUCAUAACCUUUUUAAGUAGUGGUGAUCAAUAUUUAUCACCGCCAAUUUUGGCGAUAAAAAACAACUUUUUUUGGGA...........................UAAAU...................................UCCC.A......UGG.AU
Msp-1-1     AUCAAUAGUGAUCACCGCCAGUUUUGGCGAUUAAAUAAUUGACUAAAACUUGAUUGCUUACAUUGAAGUUAUAUAAAACUUUUUUUGGGAU..........................AAGUAAAACC.............................AUCCC.A......UGG.AU
env-679     UCAAUAUUGAUCACCGCCAGUUUUGGCGAUUAAAUCAAUUGAAUAAAACUUGAUUGCUUACAUUGAAGUUAUAUAAAACUUUUUUUGGGAU..........................AAGUAAAACC.............................AUCCC.A......UGG.AU
env-680     AUCAAUAUUUAUCACCGCCAAUUAUGGCGAAUAAAUCUAGUAAAUAGAACUUGAUUGCUUACAUUGAAGUUAUAUACAACUUUUUUUGGGAU..........................UUG....................................AUCCC.A......UGG.AU
Msp-3-1     AUCAAUAUUGAUCACCGCCAAUUCUGGCGAUUAAAUCUAGUAAAUAAAACUUGAUUGCUUACAUUGAAGUUAUAUACAACUUUUUUUGGGAU..........................AGU....................................AUCCC.A......UGG.AU
env-681     CAACAUUGAUCACCGCCAAUUCUGGCUAUUUAAAUUUAGUAGUUAAAACUUGAUUGCUUUACAUAGAAGUUAUAUACAACUUUUUUUGGGAU..........................AUU....................................AUCCC.A......UGG.AU
env-682     CAACAUUGAUCACCGCCAAUUUUGGCGAUUUAAAUUUAGAAGUUAAAACUUGAUUGCUUUACAUUGAAGUUAUUUACAACUUUUUUUGGGAU..........................AAU....................................AUCCC.A......UGG.AU
env-683     CGGUCUGUAUUUUUUAGCACAAACUUUUUAAGUAUUCAUGAUCAACAUUGAUCACCGCCAAUUGGUGGAUAAUAAAUACUCUUUUUAGGGAU..........................CAA....................................GUCCC.U......UGG.AU
Mor-1-1     UUUUAAUAAAGUCAAAUAAACGAUAUCUUUAUAAACAUAUAAUUGCUAAUAUUUAAUUACUGCCAAUUUGGUAGAAUAACUUUUUUUGGGA...........................UAUUU...................................UCCC.A.....UUGG.AU
Msm-14-1    CUUUAUAUGUUUAACCAUAACUUUAUAAAUAAACAAAACAAAUAUUUUAUUAUCGUUGAUUUAUUGACGAUUACAUAACUUUUUUUCGAGAC..........................AAU....................................GUCUC.A......UGG.AU
Msm-4-1     UCUUUAUAUGUUUAACCAUAACUUUAUAAAUAAACAAAACAAAUAUUUUAUUAUCGUUGAUUUAUUGACGAUUGCAUAACUUUUUUCGAGAC..........................AAU....................................GUCUC.A......UGG.AU
env-684     UCUUUAUAUGUUUAACCAUAACUUUAUAAAUAAACAAAACAAAUAUUUUAUUAUCGUUGAUUUAUUGACGAUUAUAUAACUUUUUUCGAGAC..........................AAU....................................GUCUC.A......UGG.AU
env-685     UCUUUAUAAAUUUGACCAAAACUUUAUAAAUAAAUAAAACAAAUAUUUUAUUAUCGUUAAUUUAUUGACGAUUACAUAACUUUUUUCGAGAC..........................AAU....................................GUCUC.A......UGG.AU
env-686     UCUUUAUAUGUUUAACCAUAACUUUAUAAAUAAACAAAACAAAUAUUUUAUUAUCGUUGAUUUAUUGACGAUUACAUAACUUUUUUCGAGAC..........................AAU....................................GUCUC.A......UGG.AU
env-695     UCUUUAUAUGUUUAACCAUAACUUUAUAAAUAAACAAAACAAAUAUUUUAUUAUCGUUAAUUUAUUGACGAUUACAUAACUUUUUUCGAGAC..........................AAU....................................GUCUC.A......UGG.AU
Msm-16-1    UCUUUAUAUGUUUAACCAUAACUUUAUAAAUAAACAAAACAAAUAUUUUAUUAUCGUUGAUUUAUUGACGAUUACAUAACUUUUUUCGAGAC..........................AAU....................................GUCUC.A......UGG.AU
Msm-6-1     CUUUAUAUGUUUAACCAUAACUUUAUAAAUAAACAAAACAAAUAUUUUAUUAUCGUUGAUUUAUUGACGAUUACAUAACUUUUUUUCGAGAC..........................AAU....................................GUCUC.A......UGG.AU
env-696     UGAAUUCCGAAUGUAGGGCAACAAUCUUUUUAUUAUUCGAAACGUUUAUAUGCUAAUACAAGUUAUUCUGCAAGGCAGUUUCAAUCGCGGGA..........................UACG...................................UCCCG.C......CGGAAU
env-697     ...........................................................................................UGGCGGGA...........................GAAAA...................................UCCC.G.....CAGGGAU
env-698     CGCUUACAUAUGAUCAAUGAUCUCUUACCCAACCUUUAUAACUCAUAAAAGUAUCUAUACCUUUUUACAACAAGCCAGUUUCAUCGCGGGAU..........................AAAU...................................AUCCC.G.....CAGGGAU
env-699     CACUUACAUAUGAUCAAUGAUCUCUUACCCAACCUUUAUAACCCAUAAAAGAAUUUAUAACCUUUUACAACAAGCCAGUUUCAUUGGCGGGA..........................UAAAAUC................................UCCCG.C......AGGGAU
env-700     AGCAGUGUGUAGUACCAAAUUCCAUCCCCAACCUUUAUUACCCAUUAAUGUAUCUAUAAUCCUCUCACAACAUGCCAGUUUCAUAGGCGGGA..........................UACG...................................UCCCG.C......AGGAAU
env-701     UCCAAAUUUCAUACCGAUGCAUCAUCCCCAACCUUUAUUACCCAUUAAUGUAUCUAUAAUCCACUCAAAACAUGCCAGUUUCAUAGGCGGGA..........................UACG...................................UCCCG.C......AGGGAU
env-702     UCCAAAUUUCAUACCGAUGCAUCAUCCCCAACCUUUAUUACCCAUUAACGUAUCUAUAAUCCACUCACAGCAUGCCAGUUUCAUAGGCGGGA..........................UACG...................................UCCCG.C......AGGGAU
env-704     ..........................................AUUAACGUAUCUAUAAUCCCCUUACAACAUGCCAGUUUCAUAGGCGGGA..........................UACG...................................UCCCG.C......AGGGAU
env-706     ACCAAAUUCCAUCCCGAUGCAUCAUCCCCAACAUUUAUUACCCAUUAACGUAUCUAUAAUCCCCUCACAAGAUGCCAGUUUCAUAGGCGGGA..........................UACG...................................UCCCG.C......AGGGAU
env-707     AUUAAAUUUCAUACCAAUACAUCAUCCCCAACAUUUAUUACCCAUUAAUGUAUCUAUAAUCCUCUCAGAACAUGCCAGUUUCAUAGGCGGGA..........................UACG...................................UCCCG.C......AGGGAU
env-708     NNNNNNNNNCAUCCCGAUGCAUCAUCCCCAACAUUUAUUACCCAUUAACGUAUCUAUAAUCCCCUCACAAGAUGCCAGUUUCAUAGGCGGGA..........................UACG...................................UCCCG.C......AGGGAU
env-709     UUUGCGCAGGAAGCCAUAAACCAGUUCAACAGUUUUACGAAAUAUUUAUAUCAUAGUACCUGAUUAUUCUGCCUACGGUUUCGUUCGCGGGA..........................UCCG...................................UCCCG.C......CGGAAU
env-710     GGAAUAACAAUCUUUUAAAUGGUUUUUUUCUGAUAAUUCAUAGAAAAGGUUUAUUUUUAAGCAAUGUUGUGAUUAAAAAUUGAAAACGGAC..........................CUGUAGA................................GUCCG.U......AGGGAU
Ngr-1-1     GCCGUGUCGGCGACGGUUCGAAGCGCUUAUACUCGAGAGUUGAGUAAAGACCGAUAGACUCGCUGUAUCGCGGGCGACAGCGGGCACCUGUACGGC..........................UUU...............................GCC..ACAG.G.....UCGGGAU
Hti-1-1     UCCCGGCCUGGCGGGAGUUCGAAUCGCUUAAGGGUGCCAGCACGGUAGUACCGAUAACUCGCUGGUCGGCGGGCAGCCAGCGGGCACCUGU..........................UUAG...................................GCAG.G.....ACGGAAU
Npe-1-1     UCGCUGCGUUCCCGGAGUUCGAAGCGCUUAUACCCGAGAGCCGGGAAGGGAGGGUAGACUCGCUGGUUCGCGGGCAUCAGCGGGCACCUG...CACG..........................ACGC...............................CGUGUCACAG.G.....UCGGGAU
Hsp-7-1     CGCGCGCCGUGAGAAUCCGAAGUCCUUAACCCUCCCCUGUUGGUAGUUCCGCCAACUCGCUGGCAACGCGGGCACGCCAGAGGGCACCCGU..........................CACGC..................................ACGG.G.....ACGAAAU
Hje-1-1     ACCGGCGAAACGACGCGGGACCGAAGGGCUUAACCGUGAUUCGGGGGUAGAUCCUCCACUCGCUGGAAACGGGCACCAGCAGGCACCUGCUACC..........................GUCG...............................GGUCGCAG.G.....CAGGAAU
Hbo-1-1     GACACGUGCCGGCAGAUUCGAAGUCGUUAAAGGCGAACCGAGGGAAGAGUCGGUAACUCGCUGGCCGGACGGGACGCCAGCGGGCACCCGA..........................GAAC...................................UCGG.G.....ACGAAAU
Nma-1-1     CAACAUGGUCCGCCCGCUUCGAAGCCCUUAUACUCGGGAGUUGAAUAGGUGAAACUGACUCGCUGGAUCGCGGGCACCAGCGGGCACCUGU.UCGGC..........................ACAUUCGAC.........................GCCGCCACAG.G.....ACGGGAU
Nti-1-1     AAGCACGCUCCCGGAGUUCGAAGCCCUUAUACUCGAGAGUUGGGUAGACUGUCGUAGACUCGCUGAGUCGCGGGCAUCAGCGGGCACCUGU.UCGGCG..........................UCGA..............................CGCCACCACAG.G.....UCGGGAU
            ...........................................................................................<<<<<<<<<<<<<<<<<<<<<<<<<<<<<<<<<<.........>>>>>>>>>>>>>>>>>>>>>>>>>>>>>>>>.>.........<<...
            ...........................................................................................22222..1111...................1..1...........1..1..............1111..2222.2.........12...
            ●●●●●●●●●●●●●●●●●●●●●●●●●●●●●●●●●●●●●●●●●●●●●●●●●●●●●●●●●●●●●●●●●●●●●●●●●●●●●●●●●●●Y●●●YR●●G●-----------------------------●●●○-----------------------------------●C●●-●------○●GGRAU
                                                                                                   5′
```

alignment positions 181 · · · 360

```
env-1       GU.GGCC...........................................................................................................................................................
env-2       GU.GGCC...........................................................................................................................................................
Mma-2-1     GU.GGCC...........................................................................................................................................................
env-3       GU.GGGUGA.........................................................................................................................................................
Hha-1-1     GU.GGCCUGUGCGGU...................................................................................................................................................
env-5       GU.GACCGG.........................................................................................................................................................
env-6       GU.GACCGU.........................................................................................................................................................
env-7       GU.GGCC..ACCCUCGUGGU..............................................................................................................................................
env-9       GU.GCCCAGA.CUAGC.GUC.CC...........................................................................................................................................
env-10      GU.GGCUCAAGCGGUCC.................................................................................................................................................
Hsa-6-1     GU.GGUUGCGC.GGCGAGGACGAC..........................................................................................................................................
h3A-1-1     GU.GGUUCCGCGGC....................................................................................................................................................
env-11      GU.GGCCGC.........................................................................................................................................................
env-12      GU.GGCCGG.........................................................................................................................................................
env-14      GU.GACG.CGGGAGACGUGACGUCUC........................................................................................................................................
env-15      GU.GGUCGGC.CGGGCGC................................................................................................................................................
env-16      GU.GGUCGGC.CGGGCGC................................................................................................................................................
env-19      GU.GGGAAUACGU.....................................................................................................................................................
env-22      GU.GGGAAUACGU.....................................................................................................................................................
Hwa-1-1     GU.GGGAAUACGU.....................................................................................................................................................
env-23      GU.GGGAAGACGUC....................................................................................................................................................
env-25      GU.GGGAAUGCGUC....................................................................................................................................................
env-27      GU.GGGAAUGCGUC....................................................................................................................................................
env-28      GU.GGGCCGGCG......................................................................................................................................................
env-29      GU.GGGCCGGCG......................................................................................................................................................
env-30      GU.GGGCCGGCG......................................................................................................................................................
env-31      GU.GGGUCGGCGU.....................................................................................................................................................
Hru-1-1     GU.GGGUCGGCGU.....................................................................................................................................................
env-32      GU.GGGUCGGCGU.....................................................................................................................................................
env-33      GU.GGGUCGACGU.....................................................................................................................................................
Hpa-2-1     GU.GGAGUGCGGUC....................................................................................................................................................
env-34      GU.GGCUC.............................................................GGGUGGUCC.......ACC.................GGACCACC.GCAA......CCGA..........GCGGC................GGA........
env-35      GU.GGCGG..........................................................................................................................................................

            ...<<<<<<<<<<<<<<<<<<<<<<<<<<<<<<<<<<<<<<<<<<<<<<<<<<<<<<<<<<<<<<<...<<<<<<<<<<<<<<<<............>>>>>>>>>>>>>>>>.....<<<<<<<...>>>>>>>.<<<<<<<<<<<<<<<<<<<<<<<<.......>>>>>
            ...22222.................1.1..000000000.0000000...000.000...00000.....1111111...100000.............000001...1111111.....000000....000000.1011..1.11....111.10000000.......00000
            GU-GRYY○-----------------------------------------------------------------------------------------------------------------------------------------------------------------------
```

```
env-39   GU.GGCCUGUCUGAG..CCGGG.CUC.......................................................................................................................................................
env-40   GU.GGCCUGUCUGAG..CCGGG..CUC......................................................................................................................................................
Hac-1-1  GU.GGUGCGC...........................................................................................................................................................................
env-42   GU.GGCCGG............................................................................................................................................................................
env-43   GU.GGCCGG............................................................................................................................................................................
env-45   GU.GGCCGG............................................................................................................................................................................
Hna-1-1  GU.AGC.CCGCCG........................................................................................................................................................................
env-47   GU.GGC.UCGCCG........................................................................................................................................................................
env-48   GU.GGCGC.............................................................................................................................................................................
env-49   GU.GGCGC.............................................................................................................................................................................
env-50   GU.GGCGC.............................................................................................................................................................................
env-51   GU.GGCGC.............................................................................................................................................................................
env-52   GU.GGCGC.............................................................................................................................................................................
env-53   GU.GGCGC.............................................................................................................................................................................
env-55   GU.GGCCC.............................................................................................................................................................................
env-57   GU.GGCCGC............................................................................................................................................................................
env-61   GU.GGCCG.............................................................................................................................................................................
env-63   GU.GGCCGC............................................................................................................................................................................
env-64   GU.GGCCGC............................................................................................................................................................................
env-65   GU.GGCCGC............................................................................................................................................................................
env-66   GU.GGCCGC............................................................................................................................................................................
Hai-1-1  GU.GGCCGC............................................................................................................................................................................
Hco-1-1  GU.GGCC..............................................................................................................................................................................
Hsp-8-1  GU.GGCC..............................................................................................................................................................................
Hte-1-1  GU.GGCC..............................................................................................................................................................................
Hho-1-1  GU.GGCC..............................................................................................................................................................................
env-67   GU.GGCC..............................................................................................................................................................................
Hca-2-1  GU.GGCC..............................................................................................................................................................................
env-70   GU.GGCC..............................................................................................................................................................................
Hli-2-1  GU.GGCC..............................................................................................................................................................................
Har-2-1  GU.GGCC..............................................................................................................................................................................
env-73   GU.GGCCGC............................................................................................................................................................................
Hli-1-1  GU.GGCCGC............................................................................................................................................................................
Hha-2-1  GU.GGCCGC............................................................................................................................................................................
Hsa-9-1  GU.GGCCGC............................................................................................................................................................................
env-74   GU.GGCCGC............................................................................................................................................................................
Hko-1-1  GU.GGCCGC............................................................................................................................................................................
env-77   GU.GGCCGC............................................................................................................................................................................
env-78   GU.GGCCGC............................................................................................................................................................................
Hsa-7-1  GU.GGCCGC............................................................................................................................................................................
env-79   GU.GGCC..............................................................................................................................................................................
env-80   GU.GGCC..............................................................................................................................................................................
env-83   GU.GGCC..............................................................................................................................................................................
env-84   GU.GGCCGC............................................................................................................................................................................
env-86   GU.GGCCGC............................................................................................................................................................................
env-87   GU.GGCCGC............................................................................................................................................................................
env-89   GU.GGCCGC............................................................................................................................................................................
env-90   GU.GGCCGC............................................................................................................................................................................
env-91   GU.GGCCGC............................................................................................................................................................................
env-92   GU.GGCCGC............................................................................................................................................................................
env-93   GU.GGCCGC............................................................................................................................................................................
env-94   GU.GGCCGC............................................................................................................................................................................
Hmu-1-1  GU.GGUC.AUCGAGGGA....................................................................................................................................................................
Hel-1-1  GU.GGUC.AUCGAGGGA....................................................................................................................................................................
Hla-2-1  GU.GGUC.AUCGAGGGA....................................................................................................................................................................
Hde-1-1  GU.GGUCU.CAGGGG......................................................................................................................................................................
Hal-2-1  GU.GGUCU.CAGGGG......................................................................................................................................................................
Hpr-1-1  GU.GGUCU.CAGGGG......................................................................................................................................................................
Hgi-1-1  GU.GGUCU.CAGGGG......................................................................................................................................................................
Hgi-2-1  GU.GGUCU.CAGGGG......................................................................................................................................................................
Hsp-9-1  GU.GGUCU.CAGGGG......................................................................................................................................................................
Hsu-2-1  GU.GGUCU.CAGGGG......................................................................................................................................................................
Hsp-6-1  GU.GGUCU.CAGGG.......................................................................................................................................................................
Hme-1-1  GU.GGCCGA.C.GGGGAAUUCCCCGUCUG........................................................................................................................................................
Hru-2-1  GU.GGUCGAAAGAGGCG..CGACG.CGCCU.......................................................................................................................................................
env-95   GU.GGUCUGGGUGGCGCGGAC................................................................................................................................................................
env-98   GU.GGUCUGGGUGACGCGG..................................................................................................................................................................
env-99   GU.GGUCUGGGUGAC......................................................................................................................................................................
CHa-1-1  GU.GGUCGGCGGGCG......UCGUCCG.........................................................................................................................................................
env-101  GU.GGUCG.............................................................................................................................................................................
env-102  GU.GGUCGG............................................................................................................................................................................
env-104  GU.GGUCGG............................................................................................................................................................................
env-105  GU.GGUCGG............................................................................................................................................................................
env-110  GU.GGUCGG............................................................................................................................................................................
env-112  GU.GGUCGG............................................................................................................................................................................
env-114  GU.GGUCGG............................................................................................................................................................................
env-115  GU.GGUCGG............................................................................................................................................................................
env-116  GU.GGUCGG............................................................................................................................................................................
env-117  GU.GGUCGG............................................................................................................................................................................
env-119  GU.GGUCGG............................................................................................................................................................................
env-121  GU.GGUCGG............................................................................................................................................................................
env-122  GU.GGUCGG............................................................................................................................................................................
env-125  GU.GGUCGG............................................................................................................................................................................

         ...<<<<<<<<<<<<<<<<<<<<<<<<<<<<<<<<<<<<<<<<<<<<<<<<<<<<<<<<<<<<<<<<<<<<...<<<<<<<<<<<<<<<<..............>>>>>>>>>>>>>>>>.....<<<<<<<...>>>>>>>.<<<<<<<<<<<<<<<<<<<<<<<<<<.......>>>>>
         ...22222...................1.1..000000000.0000000...000.000...00000.......1111111...100000..............000001...1111111.....000000....000000.1011..1.11....111.10000000.......00000
         GU-GRYY∘-------------------------------------------------------------------------------------------------------------------------------------------------------------------------------
```

```
env-126    GU.GGUCGGG....................................................................................................................................................................
env-129    GU.GGUCGGA....................................................................................................................................................................
env-130    GU.GGUCGG.....................................................................................................................................................................
env-135    GU.GGUCGA.....................................................................................................................................................................
env-136    GU.GGUCGA.....................................................................................................................................................................
env-137    GU.GGUCGA.....................................................................................................................................................................
env-138    GU.GGUCGA.....................................................................................................................................................................
env-139    GU.GGUCGA.....................................................................................................................................................................
env-140    GU.GGUCGA.....................................................................................................................................................................
env-141    GU.GGUCGA.....................................................................................................................................................................
env-142    GU.GGUCGA.....................................................................................................................................................................
env-144    GU.GGUCGA.....................................................................................................................................................................
env-145    GU.GGUCGA.....................................................................................................................................................................
env-146    GU.GGUCGA.....................................................................................................................................................................
env-147    GU.GGUCGA.....................................................................................................................................................................
env-148    GU.GGUCGA.....................................................................................................................................................................
env-149    GU.GGUCGA.....................................................................................................................................................................
env-152    GU.GGUCGA.....................................................................................................................................................................
env-153    GU.GGUCGG.....................................................................................................................................................................
env-156    GU.GGUCGG.....................................................................................................................................................................
env-158    GU.GGCCGGU....................................................................................................................................................................
env-159    GU.GGCCGCC....................................................................................................................................................................
Hsp-10-1   GU.AGCCGC.....................................................................................................................................................................
Hse-1-1    GU.GGCCU.GUGGCG.CGACCGU.............................................................................................................................................................
Hmo-1-1    GU.GGCCC......................................................................................................................................................................
Hth-1-1    GU.GGCCC......................................................................................................................................................................
Nae-1-1    GU.GAUCCG.CGAGG.....................................................................................................................................................................
Nta-1-1    GU.GAUCCG.CGGGGG....................................................................................................................................................................
Nas-1-1    GU.GAUCCG.CGGGGA....................................................................................................................................................................
env-160    GU.GGCU...............................................................GG.CGGGGUC.........UCU....................GACCCCG.GGCA..................GGCGG.....................CACCG.......
env-161    GU.GACCAGC...............................................................GGGGUC..........GAA.....................GACCCC.AAAA..................CGGCG.....................UCGC........
env-162    GU.GGGCC......................................................................................................................................................................
env-164    GU.GGGCC......................................................................................................................................................................
env-169    GU.GGGCC......................................................................................................................................................................
env-170    GU.GGACCGC....................................................................................................................................................................
Hla-4-1    GU.GGAACGCGAGAG.....................................................................................................................................................................
Hsp-11-1   GU.GGAACGCGGGAG.....................................................................................................................................................................
Hsp-3-1    GU.GAUUCUCGG..................................................................................................................................................................
env-172    GU.GAUCCU.....................................................................................................................................................................
Hsa-1-1    GU.GGUCGG.....................................................................................................................................................................
env-173    GU.GGUCU......................................................................................................................................................................
env-174    GU.GGUCU......................................................................................................................................................................
env-175    GU.GGUCCC.....................................................................................................................................................................
env-176    GU.GGUCC......................................................................................................................................................................
Hpa-1-1    GU.GGCGUGG..UCGAUU..................................................................................................................................................................
env-177    GU.GAACCG................................................................GGAGGGU...GAGAUCGAA..........GAUCUCGCAGCCCUCC....ACGAGAUUUCCAAUCUCGCCGGGG.....................CCUUCGA.....
env-178    GU.GACCUGCG..CGGCUUCGGCCGC..........................................................................................................................................................
env-179    GU.GAUCUC.....................................................................................................................................................................
env-180    GU.GAACUCC....................................................................................................................................................................
env-181    GU.GAACCGACGGCGGGUUUC.CCGCCUC..AUACUGGUGACUAUAAGAGCGUG.GAAAAUUCCGACCCU..............................................................................................................
env-182    GU.GAACCUG....................................................................................................................................................................
Ssp-1-1    GU.GACCC..............................................................UGGCCGACGGACGC.....CGAC...............GCGUUCGUCGG.GAGA..................GGCG.ACGCGC..GUGCCG.......GCA.........
env-183    GU.GACCC...UGGC..........................................................CGGCCGACGCC.....GAC................GGCGUCGGUCGGGGGA..................GGCGACGGUCGAGGCCGAUCGCACCGAUCCG..CGGUG
Nph-1-1    GU.GACCGGC....................................................................................................................................................................
env-184    GU.GAUUGACAC.ACCACUCGAGUC...........................................................................................................................................................
Hgo-1-1    GU.GAUCCCGA...................................................................................................................................................................
Hxa-1-1    GU.GAUCUCGA...................................................................................................................................................................
env-185    GU.GAUCUC.....................................................................................................................................................................
env-186    GU.GAUCUC.....................................................................................................................................................................
env-187    GU.GAUCUCGG...................................................................................................................................................................
Nin-1-1    GU.GAUCCCG....................................................................................................................................................................
env-188    GU.GAUCCGG....................................................................................................................................................................
env-189    GU.GAUCCGG....................................................................................................................................................................
env-192    GU.GAUCCG.CGGG......................................................................................................................................................................
env-193    GU.GAUCCG.CGGG......................................................................................................................................................................
Nje-1-1    GU.GAUCC.GCGGG......................................................................................................................................................................
Nam-1-1    GU.GAUCC.GCGGG......................................................................................................................................................................
Noc-1-1    GU.GAUCC.GCGGG......................................................................................................................................................................
env-194    GU.GGCCC......................................................................................................................................................................
env-195    GU.GAACC...GCGAGGUCGAAGAUCUC........................................................................................................................................................
env-196    GU.GAACUAUCGC.................................................................................................................................................................
env-197    GU.GAACUAUCGC.................................................................................................................................................................
env-199    GU.GAACUAUCGC.................................................................................................................................................................
env-201    GU.GAACUAUCGC.................................................................................................................................................................
env-202    GU.GAACCGC.CGAGG....................................................................................................................................................................
Hla-1-1    GU.GAUCCC.....................................................................................................................................................................
Hva-1-1    GU.GAGUCGC....................................................................................................................................................................
Hsi-1-1    GU.GAGUCGCGC..................................................................................................................................................................
Hma-1-1    GU.GAGUCGCGC..................................................................................................................................................................
Ham-1-1    GU.GAGUCGCGU..................................................................................................................................................................
env-203    GU.GAGUCGCGC..................................................................................................................................................................
Hca-1-1    GU.GAGUCGCGC..................................................................................................................................................................
Hsa-2-1    GU.GAGUCGCGC..................................................................................................................................................................

           ...<<<<<<<<<<<<<<<<<<<<<<<<<<<<<<<<<<<<<<<<<<<<<<<<<<<<<<<<<<<<<<<<<<<...<<<<<<<<<<<<<<<<..............>>>>>>>>>>>>>>>>.....<<<<<<<...>>>>>>>.<<<<<<<<<<<<<<<<<<<<<<<<<<.......>>>>>
           ...22222..................1.1..000000000.0000000...000.000...00000.......1111111...100000..............000001...1111111.....000000....000000.1011..1.11....111.10000000.......00000
           GU-GRYY∘-------------------------------------------------------------------------------------------------------------------------------------------------------------------------
```

```
Hja-1-1    GU.GAGUCGCGC........................................................................................................................................................................
Har-1-1    GU.GAGUCGCG.........................................................................................................................................................................
env-206    GU.GAGUCGCG.........................................................................................................................................................................
Hmu-2-1    GU.GACUCCGGA........................................................................................................................................................................
Hka-1-1    GU.GACUCCGGA........................................................................................................................................................................
env-207    GU.GACCCCCGCGG......................................................................................................................................................................
env-208    GU.GAUUCGCGUUCGUGGCCGACG..GCCGC....................................................................................................................................................
env-210    GU.GAUCC.CCAGCC.....................................................................................................................................................................
env-212    GU.GAUUCCC.AGC......................................................................................................................................................................
env-213    GU.GAUUCCC.AGCC.....................................................................................................................................................................
env-214    GU.GACUCCCG.CGGGGC...GGCCGU.........................................................................................................................................................
env-215    GU.GAUUCCACCGCC.....................................................................................................................................................................
env-216    GU.GAUUCCC..CGCC....................................................................................................................................................................
env-217    GU.GACUCCGCGGGC.....................................................................................................................................................................
env-218    GU.GACUCCGCGGGC.....................................................................................................................................................................
env-219    GU.GACUCCGCGGGC.....................................................................................................................................................................
env-220    GU.GACUCCGCGGGC.....................................................................................................................................................................
Nmo-1-1    GU.GACUCCG..........................................................................................................................................................................
env-221    GU.GGUCC............................................................................................................................................................................
env-223    GU.GGCCC............................................................................................................................................................................
env-224    GU.GGCCU............................................................................................................................................................................
env-226    GU.GACUCUCGGCGUC....................................................................................................................................................................
env-227    GU.GAGGCGGCG........................................................................................................................................................................
env-228    GU.GGCCGC...........................................................................................................................................................................
env-229    GU.GGCCG............................................................................................................................................................................
Hci-1-1    GU.GGCAGGCGGGAUUC...................................................................................................................................................................
Hut-1-1    GU.GGCCGGC..........................................................................................................................................................................
env-230    GU.GGCCGGCGGGGG..CGAAAGCCCCC........................................................................................................................................................
env-231    GU.GGCCGG...........................................................................................................................................................................
env-232    GU.GGCCGG...........................................................................................................................................................................
env-233    GU.GGCCGG...........................................................................................................................................................................
env-234    GU.GAACC..CCGAG.....................................................................................................................................................................
env-235    GU.GACUUC...........................................................................................................................................................................
env-236    GU.GACCC............................................................................................................................................................................
Nba-1-1    GU.GAUCC...GCGGGGC..................................................................................................................................................................
env-237    GU.GAUCC...GCGGGGC..................................................................................................................................................................
Nsu-1-1    GU.GAUCC...GCGGGGC..................................................................................................................................................................
Npa-1-1    GU.GAUCC...GCGGGGC..................................................................................................................................................................
Nal-2-1    GU.GAUCC...GCGGGGC..................................................................................................................................................................
Nga-1-1    GU.GAUCC...GCGGGGC..................................................................................................................................................................
Hdj-1-1    GU.GAUCC...GCGGGGC..................................................................................................................................................................
Hli-3-1    GU.GAUCC...GCGGGGC..................................................................................................................................................................
Hje-2-1    GU.GAUCC...GCGGGGC..................................................................................................................................................................
Hth-2-1    GU.GAUCC...GCGGGGC..................................................................................................................................................................
Hsa-10-1   GU.GAUCC...GCGGGGC..................................................................................................................................................................
env-238    GU.GAUCCC...........................................................................................................................................................................
env-239    GU.GAUCCC...........................................................................................................................................................................
Hni-1-1    GU.GAUCC..GCGGGGC...................................................................................................................................................................
Nch-1-1    GU.GAUCCGA..........................................................................................................................................................................
Nhu-1-1    GU.GAUCCGA..........................................................................................................................................................................
Nve-1-1    GU.GAUCC...GCGGGGC..................................................................................................................................................................
Hsa-8-1    GU.GAUCC..GC.GGGG...................................................................................................................................................................
Htu-1-1    GU.GAUCCG...CGGGG...................................................................................................................................................................
env-243    GU.GAUCC...GCGGGG...................................................................................................................................................................
Hca-3-1    GU.GAUCGCCG.........................................................................................................................................................................
env-244    GU.GAACCGCGCUCUCGGUUCGGAGGUC........................................................................................................................................................
env-245    GU.GAUC.C.ACGGGAGCGGUCUCG...........................................................................................................................................................
env-246    GU.GACC.GGCGAGACCUUCGGUCUCGU........................................................................................................................................................
env-247    GU.GAUC................................................................CG.CGAGUA..........CGG......................UACUCGAGUGA..................GGG........................ACCGC.......
env-248    GU.GAUCCG.................................................................CGAGCG..........CGAUA....................CGCUCGAGAGA..................GGUGACAG...................AUG.........
env-249    GU.GAUC................................................................CG.CGAGCGU.........GCAUGCGAUACGAG..........GCGCUCGAGAGA..................GGUGACAG...................CGG.........
env-250    GU.GACCC.CG.........................................................................................................................................................................
env-251    GU.GACCCUU..........................................................................................................................................................................
env-253    GU.GACCC............................................................................................................................................................................
Hsu-1-1    GU.GAUCCGC..........................................................................................................................................................................
Hsp-2-1    GU.GAUCCGUGAGGCGC...................................................................................................................................................................
Has-1-1    GU.GGGCCGG..........................................................................................................................................................................
env-254    GU.GGACCUGAUUCGAGG..................................................................................................................................................................
Hag-1-1    GU.GGCCAGCGUGGUCC...................................................................................................................................................................
Hsa-4-1    GU.GGCCAGCGUGGUCC...................................................................................................................................................................
Hsa-5-1    GU.GGCCAGCGUGGUCC...................................................................................................................................................................
env-255    GU.GGCU.............................................................................................................................................................................
env-256    GU.GGCU.............................................................................................................................................................................
env-262    GU.GGCU.............................................................................................................................................................................
env-266    GU.GGCU.............................................................................................................................................................................
Mae-1-1    GU.GGC..............................................................................................................................................................................
env-267    GU.GGACGUUCC........................................................................................................................................................................
env-268    GU.AAC..............................................................................................................................................................................
env-269    GU.AAC..............................................................................................................................................................................
env-270    GU.GACUGC...........................................................................................................................................................................
env-271    GU.GACUGC...........................................................................................................................................................................
env-273    GU.GACUGC...........................................................................................................................................................................
env-274    GU.AGCCUU...........................................................................................................................................................................

           ...<<<<<<<<<<<<<<<<<<<<<<<<<<<<<<<<<<<<<<<<<<<<<<<<<<<<<<<<<<<<<<<<<<<<...<<<<<<<<<<<<<<<<..............>>>>>>>>>>>>>>>>.....<<<<<<<...>>>>>>>.<<<<<<<<<<<<<<<<<<<<<<<<<<.......>>>>>
           ...22222..................1.1..000000000.0000000...000.000...00000......1111111...100000..............000001...1111111.....000000....000000.1011..1.11....111.10000000.......00000
           GU-GRYYo----------------------------------------------------------------------------------------------------------------------------------------------------------------------------
```

```
env-276  GU.AGCCUU.......................................................................................................................................................................
env-277  GU.AGCC.............................................................................................................................................................................
env-278  GU.AGCC.............................................................................................................................................................................
env-281  GU.GACC.............................................................................................................................................................................
env-283  GU.GGUCC............................................................................................................................................................................
env-284  GU.GGUCC............................................................................................................................................................................
env-286  GU.GGUCC............................................................................................................................................................................
env-287  GU.GGUCCC...........................................................................................................................................................................
env-289  GU.GGCCC............................................................................................................................................................................
env-290  GU.GGCCCA...........................................................................................................................................................................
env-291  GU.GGCCC............................................................................................................................................................................
env-293  GU.GGCCCU...........................................................................................................................................................................
env-295  GU.GGUCC............................................................................................................................................................................
env-296  GU.GGUCC............................................................................................................................................................................
env-297  GU.AGCC.............................................................................................................................................................................
env-298  GU.GGCC.............................................................................................................................................................................
env-299  GU.GGCC.............................................................................................................................................................................
env-301  GU.GGCC.............................................................................................................................................................................
env-302  GU.GGCC.............................................................................................................................................................................
env-304  GU.GGCCC............................................................................................................................................................................
env-308  GU.AACCU............................................................................................................................................................................
env-309  GU.AACCU............................................................................................................................................................................
env-310  GU.AACC.............................................................................................................................................................................
env-311  GU.AACC.............................................................................................................................................................................
env-312  GU.GGCCC............................................................................................................................................................................
env-317  GU.GGCCC............................................................................................................................................................................
env-318  GU.GGCCC............................................................................................................................................................................
env-319  GU.GGCCC............................................................................................................................................................................
env-321  GU.GGCC.............................................................................................................................................................................
env-322  GU.GGCCC............................................................................................................................................................................
env-323  GU.GGCCC............................................................................................................................................................................
env-324  GU.GGCC.............................................................................................................................................................................
env-325  GU.GGCC.............................................................................................................................................................................
env-326  GU.GGCCC............................................................................................................................................................................
env-327  GU.GGCCC............................................................................................................................................................................
env-328  GU.GGCCC............................................................................................................................................................................
Pab-1-1  GU.CCCC.............................................................................................................................................................................
Psp-2-1  GU.UCCC.............................................................................................................................................................................
Tga-1-1  GU.UCCG.............................................................................................................................................................................
Tsp-3-1  GU.UCCG.............................................................................................................................................................................
Tsp-2-1  GU.UCCG.............................................................................................................................................................................
Tna-1-1  GU.UCCG.............................................................................................................................................................................
Teu-1-1  GU.UCCG.............................................................................................................................................................................
env-329  GU.UCCG.............................................................................................................................................................................
Tsp-4-1  GU.UCCG.............................................................................................................................................................................
Tsi-1-1  GU.UCCG.............................................................................................................................................................................
Tli-1-1  GU.UCCG.............................................................................................................................................................................
env-330  GU.UCCG.............................................................................................................................................................................
Tba-1-1  GU.UCCG.............................................................................................................................................................................
Tpa-1-1  GU.UCCG.............................................................................................................................................................................
Ppa-1-1  GU.UCCG.............................................................................................................................................................................
Pfu-2-1  GU.CCCC.............................................................................................................................................................................
Pho-1-1  GU.CCCC.............................................................................................................................................................................
Psp-1-1  GU.CCCC.............................................................................................................................................................................
Pya-1-1  GU.UCCC.............................................................................................................................................................................
Tko-1-1  GU.UCCC.............................................................................................................................................................................
Tsp-1-1  GU.UCCC.............................................................................................................................................................................
Pfe-1-1  GU.UCCC.............................................................................................................................................................................
Ton-1-1  GU.UCCC.............................................................................................................................................................................
Tzi-1-1  GU.UCCC.............................................................................................................................................................................
env-331  GU.AACCCU...........................................................................................................................................................................
env-332  GU.AACCCU...........................................................................................................................................................................
env-333  GU.AACCC............................................................................................................................................................................
env-334  GU.GGCC.............................................................................................................................................................................
env-336  GU.AGCC.............................................................................................................................................................................
env-338  GU.GGCC.............................................................................................................................................................................
env-339  GU.AACC.............................................................................................................................................................................
env-341  GU.AACC.............................................................................................................................................................................
env-344  GU.AACC.............................................................................................................................................................................
env-348  GU.AGCC.............................................................................................................................................................................
env-349  GU.AGCC.............................................................................................................................................................................
env-353  GU.AGCC.............................................................................................................................................................................
env-354  GU.AGCC.............................................................................................................................................................................
env-356  GU.AGCC.............................................................................................................................................................................
env-358  GU.AGCC.............................................................................................................................................................................
env-359  GU.GGCC.............................................................................................................................................................................
env-360  GU.GGCC.............................................................................................................................................................................
env-362  GU.GGCC.............................................................................................................................................................................
env-363  GU.GGCC.............................................................................................................................................................................
env-364  GU.AGCC.............................................................................................................................................................................
env-365  GU.AGCC.............................................................................................................................................................................
env-366  GU.AGCC.............................................................................................................................................................................
env-367  GU.AGCC.............................................................................................................................................................................

         ...&lt;&lt;&lt;&lt;&lt;&lt;&lt;&lt;&lt;&lt;&lt;&lt;&lt;&lt;&lt;&lt;&lt;&lt;&lt;&lt;&lt;&lt;&lt;&lt;&lt;&lt;&lt;&lt;&lt;&lt;&lt;&lt;&lt;&lt;&lt;&lt;&lt;&lt;&lt;&lt;&lt;&lt;&lt;&lt;&lt;&lt;&lt;&lt;&lt;&lt;&lt;&lt;&lt;&lt;&lt;&lt;&lt;&lt;&lt;&lt;&lt;&lt;&lt;&lt;&lt;&lt;&lt;...&lt;&lt;&lt;&lt;&lt;&lt;&lt;&lt;&lt;&lt;&lt;&lt;&lt;&lt;&lt;&lt;..............&gt;&gt;&gt;&gt;&gt;&gt;&gt;&gt;&gt;&gt;&gt;&gt;&gt;&gt;&gt;&gt;.....&lt;&lt;&lt;&lt;&lt;&lt;&lt;...&gt;&gt;&gt;&gt;&gt;&gt;&gt;.&lt;&lt;&lt;&lt;&lt;&lt;&lt;&lt;&lt;&lt;&lt;&lt;&lt;&lt;&lt;&lt;&lt;&lt;&lt;&lt;&lt;&lt;&lt;&lt;&lt;&lt;.......&gt;&gt;&gt;&gt;&gt;
         ...22222..................1.1..000000000.0000000...000.000...00000.......1111111...100000..............000001...1111111.....000000....000000.1011..1.11....111.10000000.......00000
         GU-GRYY∘------------------------------------------------------------------------------------------------------------------------------------------------------------------------
```

```
env-369   GU.AGCC............................................................................................................................................................................
env-370   GU.GGCC............................................................................................................................................................................
env-371   GU.GGCC............................................................................................................................................................................
env-375   GU.AGCC............................................................................................................................................................................
env-376   GU.AGCC............................................................................................................................................................................
env-377   GU.AGCC............................................................................................................................................................................
env-378   GU.AGCC............................................................................................................................................................................
env-379   GU.AGCC............................................................................................................................................................................
env-380   GU.AGCC............................................................................................................................................................................
env-381   GU.AGCC............................................................................................................................................................................
env-382   GU.AGCC............................................................................................................................................................................
env-383   GU.AGCC............................................................................................................................................................................
env-384   GU.AGCC............................................................................................................................................................................
env-385   GU.AGCCUA..........................................................................................................................................................................
env-386   GU.AGCCUA..........................................................................................................................................................................
env-387   GU.AGCCUA..........................................................................................................................................................................
env-388   GU.AGCC............................................................................................................................................................................
env-390   GU.AGCC............................................................................................................................................................................
env-392   GU.AACC............................................................................................................................................................................
env-402   GU.AACC............................................................................................................................................................................
env-406   GU.AACC............................................................................................................................................................................
env-408   GU.AACC............................................................................................................................................................................
env-410   GU.AACC............................................................................................................................................................................
env-412   GU.AACC............................................................................................................................................................................
env-413   GU.AACC............................................................................................................................................................................
env-414   GU.AACCA...........................................................................................................................................................................
env-416   GU.AACCA...........................................................................................................................................................................
env-418   GU.AACC............................................................................................................................................................................
env-420   GU.AACC............................................................................................................................................................................
env-422   GU.AACC............................................................................................................................................................................
env-424   GU.AGCCC...........................................................................................................................................................................
env-425   GU.AGCCC...........................................................................................................................................................................
env-427   GU.AGCCC...........................................................................................................................................................................
env-428   GU.GGCCCA..........................................................................................................................................................................
env-429   GU.GGCCC...........................................................................................................................................................................
env-432   GU.GGCCC...........................................................................................................................................................................
env-433   GU.GGCCC...........................................................................................................................................................................
env-438   GU.AACCCU..........................................................................................................................................................................
env-439   GU.AACCCU..........................................................................................................................................................................
env-440   GU.AACCU...........................................................................................................................................................................
env-441   GU.AACCU...........................................................................................................................................................................
env-442   GU.AACCU...........................................................................................................................................................................
env-443   GU.AACCU...........................................................................................................................................................................
env-444   GU.AACCU...........................................................................................................................................................................
env-446   GU.AACCU...........................................................................................................................................................................
env-447   GU.AGCCU...........................................................................................................................................................................
env-449   GU.AACC............................................................................................................................................................................
env-450   GU.AACC............................................................................................................................................................................
env-453   AU.UCCC............................................................................................................................................................................
Mvo-1-1   GUAGC..............................................................................................................................................................................
env-454   GU.GGUCC...........................................................................................................................................................................
ume-1-1   GU.GGCCUAUCC.......................................................................................................................................................................
CMe-2-1   GU.GGCCC...........................................................................................................................................................................
Mev-1-1   GU.GACUCA..........................................................................................................................................................................
env-455   GUAGCUUUU..........................................................................................................................................................................
env-457   GUAGCUUUU..........................................................................................................................................................................
env-460   GUAGCUUUUU.........................................................................................................................................................................
env-461   GUAGCUUUUU.........................................................................................................................................................................
env-462   GU.GGCUUA..........................................................................................................................................................................
Mzh-1-1   GU.GAUUCGUU........................................................................................................................................................................
env-463   GU.GGCCA...........................................................................................................................................................................
env-465   GU.GGCCCUGA........................................................................................................................................................................
env-466   GU.AGCCC...........................................................................................................................................................................
env-467   GU.GGCUC...........................................................................................................................................................................
env-469   GU.GGCCCGG.........................................................................................................................................................................
Mta-1-1   GU.GGCCCGG.........................................................................................................................................................................
env-472   GU.GGCCC...........................................................................................................................................................................
env-473   GU.GGCCC...........................................................................................................................................................................
env-474   GU.AGCUU...........................................................................................................................................................................
Mpa-1-1   GU.AGCUCAA.........................................................................................................................................................................
env-475   GU.AGCCUG..........................................................................................................................................................................
env-476   GUAGCCUG...........................................................................................................................................................................
env-477   GUAGCACG...........................................................................................................................................................................
env-479   GUAGCCC............................................................................................................................................................................
env-480   GUAGCUCU...........................................................................................................................................................................
env-481   GUAGCUCU...........................................................................................................................................................................
env-484   GUAGCUCU...........................................................................................................................................................................
env-485   GUAGCUCU...........................................................................................................................................................................
env-486   GUAGCUC............................................................................................................................................................................
env-487   GUAGCUC............................................................................................................................................................................
env-488   GUAGCUC............................................................................................................................................................................
env-489   GUAGCUC............................................................................................................................................................................
env-490   GUAGCUC............................................................................................................................................................................

          ...<<<<<<<<<<<<<<<<<<<<<<<<<<<<<<<<<<<<<<<<<<<<<<<<<<<<<<<<<<<<<<<<<<...<<<<<<<<<<<<<<<<..............>>>>>>>>>>>>>>>>.....<<<<<<<...>>>>>>>.<<<<<<<<<<<<<<<<<<<<<<<<<<.......>>>>>
          ...22222.................1.1..000000000.0000000...000.000...00000.......1111111...100000..............000001...1111111.....000000....000000.1011..1.11....111.10000000.......00000
          GU-GRYYo-----------------------------------------------------------------------------------------------------------------------------------------------------------------------------
```

```
env-491   GUAGCUC...................................................................................................................................................................
env-492   GUAGCUC...................................................................................................................................................................
env-495   GUAGCUCUG.................................................................................................................................................................
env-496   GUAGCUCA..................................................................................................................................................................
env-497   GUAGCUCA..................................................................................................................................................................
env-499   GUAGCUCA..................................................................................................................................................................
env-500   GUAGCUCA..................................................................................................................................................................
env-502   GUAGCUCA..................................................................................................................................................................
env-503   GUAGCUCA..................................................................................................................................................................
env-505   GUAGCUCA..................................................................................................................................................................
env-506   GUAGCCC...................................................................................................................................................................
env-507   GUAGCCC...................................................................................................................................................................
env-508   GUAGCCC...................................................................................................................................................................
env-509   GUAGCCC...................................................................................................................................................................
env-511   GUAGCCC...................................................................................................................................................................
env-512   GUAGCCC...................................................................................................................................................................
env-513   GUAGCCC...................................................................................................................................................................
env-514   GUAGCCC...................................................................................................................................................................
env-515   GUAGCCC...................................................................................................................................................................
env-516   GUAGCCC...................................................................................................................................................................
env-517   GU.GGCCUG.................................................................................................................................................................
env-522   GU.GGCUU.C................................................................................................................................................................
env-525   GU.GGCUU.C................................................................................................................................................................
env-528   GU.AGCC...................................................................................................................................................................
env-529   GU.AGCC...................................................................................................................................................................
env-530   GU.AGCC...................................................................................................................................................................
env-531   GU.AGCC...................................................................................................................................................................
env-532   GUAGCCC...................................................................................................................................................................
CMe-1-1   GUAGCCC...................................................................................................................................................................
env-534   GUAGCCC...................................................................................................................................................................
env-535   GUAGCCC...................................................................................................................................................................
env-536   GU.GGCUC..................................................................................................................................................................
env-537   GU.GGCUC..................................................................................................................................................................
env-538   GU.AGCCUG.................................................................................................................................................................
env-539   GU.AGCCUG.................................................................................................................................................................
env-540   GU.AGCCUG.................................................................................................................................................................
env-541   GU.GGCCU..................................................................................................................................................................
env-542   GU.GGCGUC.................................................................................................................................................................
env-543   GU.GGCGUUU................................................................................................................................................................
env-544   GU.AGCCUG.................................................................................................................................................................
env-545   GU.GGCGG..................................................................................................................................................................
env-546   GU.GGCGG..................................................................................................................................................................
Mli-1-1   GU.GGCGC..................................................................................................................................................................
env-547   GU.GGCG...................................................................................................................................................................
Mse-1-1   GU.GGCG...................................................................................................................................................................
Mma-1-1   GU.GGCG...................................................................................................................................................................
Msp-5-1   GU.GGCG...................................................................................................................................................................
env-552   GU.GGCG...................................................................................................................................................................
env-553   GU.GGCG...................................................................................................................................................................
env-559   GU.GGCG...................................................................................................................................................................
env-560   GU.GGCG...................................................................................................................................................................
env-561   GU.GGCG...................................................................................................................................................................
env-563   GU.GGCG...................................................................................................................................................................
env-564   GU.GGCG...................................................................................................................................................................
env-565   GU.GGCG...................................................................................................................................................................
env-567   GU.GGCG...................................................................................................................................................................
env-568   GU.GGCG...................................................................................................................................................................
env-570   GU.GGCG...................................................................................................................................................................
env-571   GU.GGCG...................................................................................................................................................................
env-574   GU.GGCGUG.................................................................................................................................................................
env-575   GU.GGCGUG.................................................................................................................................................................
env-576   GU.GGCGUG.................................................................................................................................................................
env-577   GU.GGCGUG.................................................................................................................................................................
env-578   GU.GGCUCA.................................................................................................................................................................
env-579   GU.GGCUC..................................................................................................................................................................
env-580   GU.GGCUCA.................................................................................................................................................................
env-581   GU.GGCCCG.................................................................................................................................................................
env-585   GU.GGCCCG.................................................................................................................................................................
env-587   GU.GGCUCG.................................................................................................................................................................
env-588   GU.GGCUCG.................................................................................................................................................................
env-589   G..UAGCCGC................................................................................................................................................................
env-590   GU.GGCUG..................................................................................................................................................................
env-591   GU.GGCUG..................................................................................................................................................................
env-594   GU.GGCUGC.................................................................................................................................................................
env-595   GU.GGCUGC.................................................................................................................................................................
env-596   GU.GGCUGC.................................................................................................................................................................
env-597   GU.GGCUGC.................................................................................................................................................................
env-598   GU.GGCUGC.................................................................................................................................................................
env-599   GU.GGCUGC.................................................................................................................................................................
env-602   GU.GGCUGC.................................................................................................................................................................
env-603   GU.GGCCGC.................................................................................................................................................................
env-604   GU.GGCCGC.................................................................................................................................................................
env-605   GU.GGCCGC.................................................................................................................................................................

          ...<<<<<<<<<<<<<<<<<<<<<<<<<<<<<<<<<<<<<<<<<<<<<<<<<<<<<<<<<<<<<<<<<<<...<<<<<<<<<<<<<<<<.............>>>>>>>>>>>>>>>>.....<<<<<<<...>>>>>>>.<<<<<<<<<<<<<<<<<<<<<<<<<.......>>>>>
          ...22222..................1.1..000000000.0000000...000.000...00000.......1111111...100000.............000001...1111111.....000000....000000.1011..1.11...111.10000000.......00000
          GU-GRYY∘----------------------------------------------------------------------------------------------------------------------------------------------------------------------------------
```

```
env-606    GU.GGCCGC...................................................................................................................................................................
env-607    GU.GGCCGC...................................................................................................................................................................
env-608    GU.GGCCGC...................................................................................................................................................................
env-609    GU.GGCCGC...................................................................................................................................................................
env-610    GU.GGCCGC...................................................................................................................................................................
env-611    GU.GGCCGC...................................................................................................................................................................
env-612    GU.GGCCGC...................................................................................................................................................................
env-613    GU.GGCCGC...................................................................................................................................................................
env-616    GU.GGCUA....................................................................................................................................................................
Mco-1-1    GU.GGCUA....................................................................................................................................................................
env-617    GU.GAUCUUU..................................................................................................................................................................
Mps-1-1    GU.GGCUU....................................................................................................................................................................
env-619    GU.GGCUU....................................................................................................................................................................
env-620    GU.GGCUU....................................................................................................................................................................
Mti-1-1    G..UAGCUUUUG................................................................................................................................................................
env-621    G..UAGCUU...................................................................................................................................................................
Mba-2-1    G..UAGUCCUG.................................................................................................................................................................
env-622    GU.GACCCUG..................................................................................................................................................................
env-623    GU.GGUCCUG..................................................................................................................................................................
env-624    GU.GGUCCUG..................................................................................................................................................................
env-625    GU.GGUCCUGU.................................................................................................................................................................
Mho-2-1    GU.GGUCCUG..................................................................................................................................................................
env-628    GU.GGUCCUG..................................................................................................................................................................
env-629    GU.GGUCCUG..................................................................................................................................................................
env-630    GU.GGUCCUGU.................................................................................................................................................................
Mth-1-1    GU.GGUCCUGU.................................................................................................................................................................
Msp-13-1   GU.GGUCCUG..................................................................................................................................................................
Mva-1-1    GU.GGUCCUG..................................................................................................................................................................
Mba-4-1    GU.GGUCCUG..................................................................................................................................................................
env-631    GU.GGUCCUG..................................................................................................................................................................
env-633    GU.GGUCCUG..................................................................................................................................................................
Mla-1-1    GU.GGUCCUG..................................................................................................................................................................
Mba-5-1    GU.GGUCCUG..................................................................................................................................................................
Msp-7-1    GU.GGUCCUG..................................................................................................................................................................
Msp-16-1   GU.GGUCCUG..................................................................................................................................................................
env-634    GU.GGUCCUGC.................................................................................................................................................................
Mso-1-1    G.UGGUCCUG..................................................................................................................................................................
Mma-3-1    G.UGGUCCUG..................................................................................................................................................................
Mac-1-1    G.UGGCCCUG..................................................................................................................................................................
Msi-2-1    G.UGGCCCUG..................................................................................................................................................................
env-635    GU.GGCCUUG..................................................................................................................................................................
Msi-1-1    GUGGCCCUG...................................................................................................................................................................
env-636    GUGGCCCUG...................................................................................................................................................................
Msp-14-1   GU.GGCUCG...................................................................................................................................................................
env-637    GU.GGUCC....................................................................................................................................................................
env-638    GU.GGUCC....................................................................................................................................................................
env-639    G..UAGUCC...................................................................................................................................................................
env-640    G..UAGCC....................................................................................................................................................................
env-641    GUGGCCCA....................................................................................................................................................................
Mbu-1-1    G.UGGCUGAA..................................................................................................................................................................
env-642    GU.GGCUGAA..................................................................................................................................................................
Mme-2-1    GU.GGCCGAAG.................................................................................................................................................................
Mme-1-1    GU.GGCCGAAG.................................................................................................................................................................
Msh-1-1    GU.GGCUC....................................................................................................................................................................
env-643    GU.GGCU.....................................................................................................................................................................
env-644    GU.GGC......................................................................................................................................................................
env-645    GU.GGC......................................................................................................................................................................
env-646    GU.GGC......................................................................................................................................................................
env-647    GU.GGCC.....................................................................................................................................................................
env-650    GU.GGCU.....................................................................................................................................................................
env-651    GU.GGC......................................................................................................................................................................
env-656    GU.GGCC.....................................................................................................................................................................
env-657    GU.GGCC.....................................................................................................................................................................
env-658    GU.GGCC.....................................................................................................................................................................
Mve-1-1    GU.GGC......................................................................................................................................................................
env-659    GU.GGC......................................................................................................................................................................
env-660    GU.GGC......................................................................................................................................................................
Msp-4-1    GU.GGC......................................................................................................................................................................
env-661    GU.GGC......................................................................................................................................................................
env-663    GU.GGC......................................................................................................................................................................
env-664    GU.GGC......................................................................................................................................................................
env-665    GU.GGC......................................................................................................................................................................
env-670    GU.GGC......................................................................................................................................................................
env-671    GU.GGC......................................................................................................................................................................
env-672    GU.GGC......................................................................................................................................................................
Mfo-2-1    GU.GGC......................................................................................................................................................................
env-673    GU.GGC......................................................................................................................................................................
env-674    GU.GGC......................................................................................................................................................................
Msp-2-1    GU.GGC......................................................................................................................................................................
Mfo-1-1    GU.GGC......................................................................................................................................................................
env-675    GU.GGC......................................................................................................................................................................
env-676    GU.GGC......................................................................................................................................................................
Msp-1-1    GU.GGC......................................................................................................................................................................

           ...&lt;&lt;&lt;&lt;&lt;&lt;&lt;&lt;&lt;&lt;&lt;&lt;&lt;&lt;&lt;&lt;&lt;&lt;&lt;&lt;&lt;&lt;&lt;&lt;&lt;&lt;&lt;&lt;&lt;&lt;&lt;&lt;&lt;&lt;&lt;&lt;&lt;&lt;&lt;&lt;&lt;&lt;&lt;&lt;&lt;&lt;&lt;&lt;&lt;&lt;&lt;&lt;&lt;&lt;&lt;&lt;&lt;&lt;&lt;&lt;&lt;&lt;&lt;&lt;&lt;&lt;&lt;...&lt;&lt;&lt;&lt;&lt;&lt;&lt;&lt;&lt;&lt;&lt;&lt;&lt;&lt;&lt;&lt;..............&gt;&gt;&gt;&gt;&gt;&gt;&gt;&gt;&gt;&gt;&gt;&gt;&gt;&gt;&gt;&gt;.....&lt;&lt;&lt;&lt;&lt;&lt;&lt;...&gt;&gt;&gt;&gt;&gt;&gt;&gt;.&lt;&lt;&lt;&lt;&lt;&lt;&lt;&lt;&lt;&lt;&lt;&lt;&lt;&lt;&lt;&lt;&lt;&lt;&lt;&lt;&lt;&lt;&lt;&lt;&lt;&lt;.......&gt;&gt;&gt;&gt;&gt;
           ...22222..................1.1..000000000.0000000...000.000...00000.......1111111...100000..............000001...1111111.....000000....000000.1011..1.11....111.10000000.......00000
           GU-GRYY∘-----------------------------------------------------------------------------------------------------------------------------------------------------------------------------
```

```
env-679     GU.GGC..........................................................................
env-680     GU.GGC..........................................................................
Msp-3-1     GU.GGC..........................................................................
env-681     GU.GGC..........................................................................
env-682     GU.GGC..........................................................................
env-683     GU.GGC..........................................................................
Mor-1-1     GU.GGCU.........................................................................
Msm-14-1    GU.GGCUUUU......................................................................
Msm-4-1     GU.GGCUUUU......................................................................
env-684     GU.GGCUUUU......................................................................
env-685     GU.GGCUUUU......................................................................
env-686     GU.GGCUUUU......................................................................
env-695     GU.GGCUUUU......................................................................
Msm-16-1    GU.GGCUUUU......................................................................
Msm-6-1     GU.GGCUUUU......................................................................
env-696     GU.GGCUUA.......................................................................
env-697     GU.GGCCU........................................................................
env-698     GU.GGCCU........................................................................
env-699     GU.GGCCU........................................................................
env-700     GU.GGCC.........................................................................
env-701     GU.GGCCC........................................................................
env-702     GU.GGCCC........................................................................
env-704     GU.GGCCC........................................................................
env-706     GU.GGCCC........................................................................
env-707     GU.GGCCC........................................................................
env-708     GU.GGCCC........................................................................
env-709     GU.GGCCC........................................................................
env-710     GU.GGCUU........................................................................
Ngr-1-1     GU.GAUC.CG.CGGGGC...............................................................
Hti-1-1     GU.GGCCGC.......................................................................
Npe-1-1     GU.GAUCC...GCGGGGC..............................................................
Hsp-7-1     GU.GGUCU.CAGGGG.................................................................
Hje-1-1     GU.GGUCCGCGC....................................................................
Hbo-1-1     GU.GGAGGGCGU....................................................................
Nma-1-1     GU.GAUCCGA......................................................................
Nti-1-1     GU.GAUCC...GCGGGG...............................................................
            ...<<<<<<<<<<<<<<<<<<<<<<<<<<<<<<<<<<<<<<<<<<<<<<<<<<<<<<<<<<<<<<<<<<<<...<<<<<<<<<<<<<<<<..............>>>>>>>>>>>>>>>>.....<<<<<<<...>>>>>>>.<<<<<<<<<<<<<<<<<<<<<<<<<<.......>>>>>
            ...22222..................1.1..000000000.0000000...000.000...00000.......1111111...100000..............000001...1111111.....000000....000000.1011..1.11....111.10000000.......00000
            GU-GRYY∘----------------------------------------------------------------------------------------
```

alignment positions 361 · · · 512

```
env-1       .....................CAUUUGGA.................................................................GGCU.GAACAAAUCAUUAGGGUGAAAUAUC..........................AUG
env-2       .....................CAUUUGGA.................................................................GGCU.GAACAAAUCAUUAGGGUGAAAUAUC..........................AUG
Mma-2-1     .....................UUUAU....................................................................GGCU.GAACCGUCAAUGAAUGAAACAAGGUGAAAUC...................AUG
env-3       .....................AUACAG.................................................................UCACUC.GAAUCGAAACCGCUCGUCAGAGACAACA.....................AUG
Hha-1-1     .....................CGGG.............................................................ACCGCAC.GGCU.GAACCACCAACGCCCGGAGGCACAUC.........................AUG
env-5       .....................UUGA...................................................................CCGGUU.GAACCACUAACGCCCCGGGAACCGAAAGACC...................AUG
env-6       .....................UUC....................................................................ACGGUU.GAACCACCAACGCCCCGGGAAGGUACACCAA...................AUG
env-7       .....................GAAGGG.....................................................ACCGCCCCGGAACGGCU.GAACCAUCGAGCCCGCGGUGACAACA........................AUG
env-9       .....................GACUCGAA................................................GGAGGCUGCUGGAUCUGGGU.GAAUCGCAAGCGCUCGUGGAAUACACA.......................AUG
env-10      .....................GAC..........................................................GGACCACGAGGGCC.GAAUCGCAAGCGCCCGCGGUAGACCA.........................AUG
Hsa-6-1     .....................GACGU..................................................GUCGUCUUCGUUGGCGGAACU.GAAUCGCAAGCGUCCGUGGUGAUUACA.......................AUG
h3A-1-1     .....................GUC.......................................................GUCGCGGGACU.GAAUCGCAAGCGUCCGUGGACAACCC..........................AUG
env-11      .....................UUC....................................................................GCGGCU.GAAUCGCAACCGUCCGCGGACAACAU..........................AUG
env-12      .....................AGA....................................................................CCGGCC.GAAUCGCAAGCGUCCACAGGCAACC...........................AUG
env-14      .....................CGG.....................................................GAGGCGGCCCACCGCCGACGUU.GAAUCGCAAGCGCCCGCGGUGAAACAU.......................AUG
env-15      .....................AAC................................................................GCGCCCCUGCCGACU.GAAUCGCAAGCGCCCGCAAUCUAACC..........................AUG
env-16      .....................AAC................................................................GCGCCCCUGCCGACU.GAAUCGCAAGCGCCCGCAAUCUAACC..........................AUG
env-19      .....................CAUUG...................................................................ACGU..UCCC.GAAUCACAAGCGCCCGUGGUGAAAACAG........................AUG
env-22      .....................CAUUG...................................................................ACGU..UCCC.GAAUCACAAGCGCCCGUGGUGAAAACAG........................AUG
Hwa-1-1     .....................CAUUG...................................................................ACGU..UCCC.GAAUCACAAGCGCCCGUGGUGAAAACAG........................AUG
env-23      .....................AAU.....................................................................GACGUCUUCCC.GAAUCGCAAGCGCCCGUGGUGAAAACAG........................AUG
env-25      .....................AUU.....................................................................GACGUC..CCC.GAAUCGCAAGCGCCCGUGGUGAAAAUAC........................AUG
env-27      .....................AUU.....................................................................GACGUC..CCC.GAAUCGCAAGCGCCCGUGGUGAAAAUAC........................AUG
env-28      .....................UCGAC.......................................................................CGUC.GCCC.GAAUCGCAAGCGCCCGUGGUGACACCA.........................AUG
env-29      .....................UCGAC.......................................................................CGUC.GCCC.GAAUCGCAAGCGCCCGUGGUGACACCA.........................AUG
env-30      .....................UCGAC.......................................................................CGUC.GCCC.GAAUCGCAAGCGCCCGUGGUGACACCA.........................AUG
env-31      .....................CGA.......................................................................ACGCCCGCCC.GAAUCGCAAGCGCCCGUGGUGACACCA.........................AUG
Hru-1-1     .....................CGA.......................................................................ACGCCG.CCC.GAAUCGAAAGCGCCCGUGGUGAAACCA.........................AUG
env-32      .....................CGA.......................................................................ACGUCG.CCC.GAAUCGCAAGCGCCCGUGGUGAAACCA.........................AUG
env-33      .....................CGA.......................................................................ACGUCG.CCC.GAAUCGCAAGCGCCCGUGGUGAAACCA.........................AUG
Hpa-2-1     .....................GACGGUA..................................................................GAGCGC.CUCC.GAAUCGCAAGCGCCCGUGGUGAAACU..........................AUG
env-34      ............GCCGC....GAGGGGA........................................................................GAGCU.GAAUCGCAAGCGCCCGCGGUACACCA..........................AUG
env-35      .....................UAUAU..........................................................................CCGCU.GAAUCGCAAGCGCCCGUGGUGAUACAU.........................AUG
env-39      .....................CGCCGU.....................................................GAACCCCGACUCUCAAUCAGGCU.GAAUCGCAAGCGUCCGUGGUACACAUC.........................AUG
env-40      .....................CACGGU....................................................GAACCCCCGACUCUCAAUCAGGCU.GAAUCGCAAGCGUCCGUGGAACACAUC.........................AUG
Hac-1-1     .....................GUC........................................................................GCGCACU.GAAUCGCAAGCGCCCGCGGAACACACU.........................AUG
env-42      .....................AGA..........................................................................CCGGCC.GAAUCGCAAGCGUCCGCAGGCACCC...........................AUG

            >>>>>>>>>>>>>>>>>>>>>...........>>>>>>>>>>>>>>>>>>>>>>>>>>>>>>>>>>>>>>>>>>>>>>>>>>>>>>>>>....>>..................................................
            001.111....11.1..1101...........:...00000...000.000...0000000.000000000..1.1...................22222....21..................................................
            --------------------∙∙∙∘---------------------------------------------------------------∘RRYU-GAACCR∙RRR∙∙∙∙∙∙∙∙R∙∙∙∙∙∙∙∘∘--------------------AUG
                                                                                                              3′
```

```
env-43     ....................GGA.................................................................CCGGCC.GAAUCGCAAGCGUCCGCAGGCAACC.........................AUG
env-45     ....................GGA.................................................................CCGGCC.GAAUCGCAAGCGUCCGCAGGCAACC.........................AUG
Hna-1-1    ....................ACUU.........................................................CGGCGGCGCC.GAAUCGCAAGCGCCCGUGGGUAACUC.......................AUG
env-47     ....................ACUN.........................................................CGGCGACGCU.GAAUCGCAAGCGCCCGUGGUGAAACCC.....................AUG
env-48     ....................AGGU..............................................................GCGCU.GAAUCGCAACGGCCCGCGGUGAAACC........................AUG
env-49     ....................AGGU..............................................................GCGCU.GAAUCGCAACGGCCCGCGGUGAAACC........................AUG
env-50     ....................AGAU..............................................................GCGCU.GAAUCGCAACGGCCCGCGGUGAAACC........................AUG
env-51     ....................AGAU..............................................................GCGCU.GAAUCGCAACGGCCCGCGGUGAAACC........................AUG
env-52     ....................AGAC..............................................................GCGCU.GAAUCGCAAGCGCCCGCGGUGAAACC........................AUG
env-53     ....................AGAC..............................................................GCGCU.GAAUCGCAAGCGCCCGCGGUGAAACC........................AUG
env-55     ....................UUC...............................................................GGGCU.GAAUCGCAACCGUCCGCGGACACACCA.......................AUG
env-57     ....................AUGC.............................................................GCGGCU.GAAUCGCAAACGUCCGUGGACAACACCC.....................AUG
env-61     ....................CAUGCA.............................................................CGGCU.GAAUCGCAAACGUCCGUGGACACGACC.......................AUG
env-63     ....................AUGC.............................................................GCGGCU.GAAUCGCAAACGUCCGUGGACAACACCC.....................AUG
env-64     ....................AUGC.............................................................GCGGCU.GAAUCGCAAACGUCCGUGGACAACACCC.....................AUG
env-65     ....................AUGC.............................................................GCGGCU.GAAUCGCAACCGUCCGUGGACACGAACC.....................AUG
env-66     ....................CUC..............................................................GCGGCU.GAAUCGCAACCGUCCGUGGACAACCA.......................AUG
Hai-1-1    ....................CUC..............................................................GCGGCU.GAAUCGCAACCGUCCGCGGAUACACCA......................AUG
Hco-1-1    ....................CCUCGC..............................................................GGCU.GAAUCGCAACCGUCCGCAGACACACA.......................AUG
Hsp-8-1    ....................CCUCGC..............................................................GGCU.GAAUCGCAACCGUCCGCGGACACACA.......................AUG
Hte-1-1    ....................CCUCGC..............................................................GGCU.GAAUCGCAACCGUCCGCGGACACACA.......................AUG
Hho-1-1    ....................CCUCGC..............................................................GGCU.GAAUCGCAACCGUCCGCGGACACACA.......................AUG
env-67     ....................CCUCGC..............................................................GGCU.GAAUCGCAACCGUCCGCGGACACACA.......................AUG
Hca-2-1    ....................CCUCGC..............................................................GGCU.GAAUCGCAACCGUCCGCGCAACACACA......................AUG
env-70     ....................CCUCGC..............................................................GGCU.GAAUCGCAACCGUCCGCGGACAACA.........................AUG
Hli-2-1    ....................CCUCGC..............................................................GGCU.GAAUCGCAACCGUCCGCGCAACACACA......................AUG
Har-2-1    ....................CCUCGC..............................................................GGCU.GAAUCGCAACCGUCCGCGCAACACACA......................AUG
env-73     ....................CUC..............................................................GCGGCU.GAAUCGCAACCGUCCGCGGACAACAU.......................AUG
Hli-1-1    ....................UUC..............................................................GCGGCU.GAAUCGCAACCGUCCGCGGACAACAU.......................AUG
Hha-2-1    ....................UUC..............................................................GCGGCU.GAAUCGCAACCGUCCGCGGACAACAU.......................AUG
Hsa-9-1    ....................UUC..............................................................GCGGCU.GAAUCGCAACCGUCCGCGGACAACAU.......................AUG
env-74     ....................UUC..............................................................GCGGCU.GAAUCGCAACCGUCCGCGGACAACAU.......................AUG
Hko-1-1    ....................UUC..............................................................GCGGCU.GAAUCGCAACCGUCCGCGGACAACAU.......................AUG
env-77     ....................UUC..............................................................GCGGCU.GAAUCGCAACCGUCCGCGGACAACAU.......................AUG
env-78     ....................CUC..............................................................GCGGCU.GAAUCGCAACCGUCCGCGGACAACAU.......................AUG
Hsa-7-1    ....................CUC..............................................................GCGGCU.GAAUCGCAACCGUCCGCGGACAACAU.......................AUG
env-79     ....................CCUCGC..............................................................GGCU.GAAUCGCAACCGUCCGCGGACAACAU.......................AUG
env-80     ....................CUCGC...............................................................GGCU.GAAUCGCAACCGUCCGCACACACACA.......................AUG
env-83     ....................CCUCGC..............................................................GGCU.GAAUCGCAACCGUCCGCAGACACACA.......................AUG
env-84     ....................UUC..............................................................GCGGCU.GAAUCGCAACCGUCCGCGGACACACCA......................AUG
env-86     ....................UUC..............................................................GCGGCU.GAAUCGCAACCGUCCGCGGACACACAU......................AUG
env-87     ....................UUC..............................................................GCGGCU.GAAUCGCAACCGUCCGCGGACACACAU......................AUG
env-89     ....................CUC..............................................................GCGGCU.GAAUCGCAAUCGUCCGUGGACGAUACA.......................AUG
env-90     ....................CUC..............................................................GCGGCU.GAAUCGCAAGCGUCCGUGGACAACACA.......................AUG
env-91     ....................CUC..............................................................GCGGCU.GAAUCGCAAGCGUCCGUGGACAACACA.......................AUG
env-92     ....................CUC..............................................................GCGGCU.GAAUCGCAAGCGUCCGUGGACGAUACA.......................AUG
env-93     ....................AUGC.............................................................GCGGCU.GAAUCGCAAACGUCCGUGGACACGAACC.....................AUG
env-94     ....................UUC..............................................................GCGGCU.GAAUCGCAAGCGUCCGUGGACACGACA.......................AUG
Hmu-1-1    ....................GUG......................................................UCCCUCCACCGACU.GAAUCGCAAGCGCCCGCGGUGAUAUACA....................AUG
Hel-1-1    ....................UGG......................................................UCCCUCAACCGACU.GAAUCGCAAGCGCCCGCGGUGAUAUACA....................AUG
Hla-2-1    ....................UGG......................................................UCCCUCAUUCGACU.GAAUCGCAAGCGCCCGCGGUGAUAUACA....................AUG
Hde-1-1    ....................GAUU.......................................................CCCCGACGGACU.GAAUCGCAAGCGCCCGCGGUGAUACACA....................AUG
Hal-2-1    ....................AGUA.......................................................CCCCGACGGACU.GAAUCGCAAGCGCCCGCGGUGAUAUACA....................AUG
Hpr-1-1    ....................AGUU.......................................................CCCCGACGGACU.GAAUCGCAAGCGCCCGCGGUGAUAUACA....................AUG
Hgi-1-1    ....................AGUU.......................................................CCCCGACGGACU.GAAUCGCAAGCGCCCGCGGUGAUAUACA....................AUG
Hgi-2-1    ....................AGUU.......................................................CCCCGACGGACU.GAAUCGCAAGCGCCCGCGGUGAUAUACA....................AUG
Hsp-9-1    ....................AGUU.......................................................CCCCGACGGACU.GAAUCGCAAGCGCCCGCGGUGAUACACA....................AUG
Hsu-2-1    ....................AGUU.......................................................CCCCGACGGACU.GAAUCGCAAGCGCCCGCGGUGAUAUACA....................AUG
Hsp-6-1    ....................AAGUUC.......................................................CCCGACGGACU.GAAUCGCAAGCGCCCGCGGUGAUACACA....................AUG
Hme-1-1    ....................GGG...................................CGGACG.......CCCCAGAAUGGCU.GAAUCGCAAGCGCCCGCGGUGAUAUACA....................AUG
Hru-2-1    ....................CCCG...................................AGGCGGCGGAGCCGCCGACAUUCGACU.GAAUCGCAACCGCCCGCACAGAACC.......................AUG
env-95     ....................GGC..................................................GUUCGCGCCGCCACGACU.GAAUCGCAAGCGCCCGCAGGAGACACU.....................AUG
env-98     ....................ACU....................................................CCGCGUCGUCGCGACU.GAAUCGCAAGCGCCCGCAGGAGACACU.....................AUG
env-99     ....................GGACG.....................................................GUCGCCACGACU.GAAUCGCAAGCGCCCGCAGGAACACU.......................AUG
CHa-1-1    ....................ACGCCCGAGGG........................................CGGGGGACCCGCCCGACCGCCGACU.GAAUCGCAAGCGCCCGCGGAGCAACCC.............AUG
env-101    ....................UCGUU..............................................................CGACU.GAAUCGCAAGCGCCCGAGGAUACCA.......................AUG
env-102    ....................GUGAU.............................................................CCGACU.GAAUCGCAAGCGCCCGAGGAUAACCU......................AUG
env-104    ....................GUGAU.............................................................CCGACU.GAAUCGCAAGCGCCCGAGGAUAACCA......................AUG
env-105    ....................AUGAA.............................................................CCGACU.GAAUCGCAAGCGCCCGAGGACAAUCU......................AUG
env-110    ....................AUGAA.............................................................CCGACU.GAAUCGCAAGCGCCCGAGGACAAUCU......................AUG
env-112    ....................CCUGA.............................................................CCGACU.GAAUCGCAAGCGCCCGAGGACAACU.......................AUG
env-114    ....................CCUGA.............................................................CCGACU.GAAUCGCAAGCGCCCGAGGACAACU.......................AUG
env-115    ....................CCUGA.............................................................CCGACU.GAAUCGCAAGCGCCCGAGGACAACU.......................AUG
env-116    ....................CCUGA.............................................................CCGACU.GAAUCGCAAGCGCCCGAGGACAACU.......................AUG
env-117    ....................CUUGA.............................................................CCGACU.GAAUCGCAAGCGCCCGAGGACAACU.......................AUG
env-119    ....................CCUGA.............................................................CCGACU.GAAUCGCAAGCGCCCGAGGACAACU.......................AUG
env-121    ....................CCUGA.............................................................CCGACU.GAAUCGCAAGCGCCCGAGGACAACU.......................AUG
env-122    ....................CCUGA.............................................................CCGACU.GAAUCGCAAGCGCCCGAGGACAACU.......................AUG
env-125    ....................CCUGA.............................................................CCGACU.GAAUCGCAAGCGCCCGAGGACAACU.......................AUG
env-126    ....................AUUU.............................................................UCCGACU.GAAUCGCAAGCGCCCGAGGAUAACA.......................AUG
env-129    ....................AUU..............................................................UCCGACU.GAAUCGCAAGCGCCCGAGGACAACU.......................AUG
env-130    ....................GAUUU.............................................................CCGACU.GAAUCGCAAGCGCCCGAGGACAACA.......................AUG
env-135    ....................GUU..............................................................UCGACU.GAAUCGCAAGCGCCCGAGGACAACA.......................AUG

           >>>>>>>>>>>>>>>>>>>>...........>>>>>>>>>>>>>>>>>>>>>>>>>>>>>>>>>>>>>>>>>>>>>>>>>>>>>>....>>.............................................
           001.111....11.1..1101...............00000...000.000...0000000.000000000..1.1..................22222....21.............................................
           --------------------●●●○-------------------------------------------------------------○RRYU-GAACCR●RRR●●●●●●●●R●○○○○○○○○-------------------AUG
                                                                                                              3′
```

```
env-136   .....................AUU.....................................................................UCGACU.GAAUCGCAAGCGCCCGAGGACAAAUA.......................AUG
env-137   .....................CUGU....................................................................UCGACU.GAAUCGCAAGCGCCCGAGGACAACCA.......................AUG
env-138   .....................CUCU....................................................................UCGACU.GAAUCGCAAGCGCCCGAGGACAAACA.......................AUG
env-139   .....................CUCCU...................................................................UCGACU.GAAUCGCAAGCGCCCGAGGACAAACA.......................AUG
env-140   .....................CUCC....................................................................UCGACU.GAAUCGCAAGCGCCCGAGGACAAACA.......................AUG
env-141   .....................CUCC....................................................................UCGACU.GAAUCGCAAGCGCCCGAGGACAAAC...........................
env-142   .....................CUCC....................................................................UCGACU.GAAUCGCAAGCGCCCGAGGACAAACA.......................AUG
env-144   .....................CUCC....................................................................UCGACU.GAAUCGCAAGCGCCCGAGGACAAACA.......................AUG
env-145   .....................CUU.....................................................................UCGACU.GAAUCGCAAGCGCCCGAGGACAAACA.......................AUG
env-146   .....................CUUGU...................................................................UCGACU.GAAUCGCAAGCGCCCGAGGACAACCA.......................AUG
env-147   .....................CGU.....................................................................UCGACU.GAAUCGCAAGCGCCCGAGGACAACCA.......................AUG
env-148   .....................CGU.....................................................................UCGACU.GAAUCGCAAGCGCCCGAGGACAACCA.......................AUG
env-149   .....................CUCC....................................................................UCGACU.GAAUCGCAAGCGCCCGAGGAUAACA........................AUG
env-152   .....................CUCU....................................................................UCGACU.GAAUCGCAAGCGCCCGAGGAAAACA........................AUG
env-153   .....................CUCGA...................................................................CCGACU.GAAUCGCAAGCGCCCGACGAACU..........................AUG
env-156   .....................CUUGA...................................................................CCGACU.GAAUCGCAAGCGCCCGAGACAACU.........................AUG
env-158   .....................UCG....................................................................ACCGGCU.GAAUCGCAAGCGCCCGCGGUGAAACG.......................AUG
env-159   .....................UCC....................................................................GGCGGCU.GAAUCGCAAGCGCCCGCGGUGAACCAC......................AUG
Hsp-10-1  .....................CUC.....................................................................GCGGCU.GAAUCGCAACCGUCCGCGAACACACA.......................AUG
Hse-1-1   .....................GCG.......................................................GCGGUCGUCGCCCGAGGGCU.GAACCACCAACGCCCGCAGAUAACC........................AUG
Hmo-1-1   .....................UCAC.....................................................................GGGCU.GAACCCUCAACGCCCGCAGACAACC........................AUG
Hth-1-1   .....................UCAC.....................................................................GGGCU.GAACCCGCAACGCCCGCAGACAACC........................AUG
Nae-1-1   .....................CAGCGC............................................................CCUCGCCGGGUU.GAACCAUCACCGCCCGCGGUGAAUACA......................AUG
Nta-1-1   .....................AUCG.............................................................UCCUCGCCGGGUU.GAACCAUCACCGCCCGCGGUGAAUACA......................AUG
Nas-1-1   .....................AUCG.............................................................UCCUCGCCGGGUU.GAACCAUCACCGCCCGCGGUGAAUACA......................AUG
env-160   ................CCGCCGGAG......................................................................AGCU.GAACCACACAAGGCCCGCACGCCAACC......................AUG
env-161   ................CGCCG..........................................................................GGUU.GAACCACGCAAGGCCCGCACGCCAACA......................AUG
env-162   .....................CUCGA....................................................................GGCCU.GAACCAACGACGCCCGCGGAUACACU.......................AUG
env-164   .....................CUCGA....................................................................GGCCU.GAACCAACGACGCCCGCGGACAACCU.......................AUG
env-169   .....................CUCGA....................................................................GGCCU.GAACCAACGACGCCCGCGGACAACCU.......................AUG
env-170   .....................UCUC...................................................................GUGGUCU.GAACCGGGAAACGCCCGCAGGUAAAACG.....................AUG
Hla-4-1   .....................AUC...............................................................CUCUCGAGUUCU.GAACCAGGAAACGCCCGCAGCAAAACACA....................AUG
Hsp-11-1  .....................ACU...............................................................CUCUCGAGUUCU.GAACCAGGAAACGCCCGCAGGUAUACA......................AUG
Hsp-3-1   .....................UGCG.................................................................CCGGGAAUU.GAACCACACAACGCCCGCGGACACAGAACA...................AUG
env-172   .....................CUCG....................................................................AGGAUU.GAACCAGGAACUGCCCGCAGCACAACU......................AUG
Hsa-1-1   .....................GCGA....................................................................CCGACU.GAACCACUCGACGCCCGCGGUGAAAUC......................AUG
env-173   .....................UCG......................................................................GGACU.GAACCGGCCAACGCCCGUGGUGAUACACG....................AUG
env-174   .....................UCG......................................................................GGACU.GAACCGGCCAACGCCCGUGGUGAUACACG....................AUG
env-175   .....................UCC.....................................................................GGGACU.GAACCGGCAAACGCCCGUGGUGAUAGACC....................AUG
env-176   .....................CUCG.....................................................................GGACU.GAACCGGCAAACGCCCGUGGUGAUAGACC....................AUG
Hpa-1-1   .....................GUG............................................................AAUCGAUUCCACGCU.GAACCACUCAACGCCCGUGGUGAUAGACC....................AUG
env-177   ................CCCCGCA......................................................................UGGUUU.GAACCACGCAACGCCCGUGGUGAUAGACAU...................AUG
env-178   .....................GGGGAG.................................................GCGGAUC.GGCCGCCGAACGGUU.GAACCACACGACGCCCGUGGUGAACACACU...................AUG
env-179   .....................UCAC....................................................................GGGAUU.GAACCAGGAAACGCCCGCAAGAAAACC......................AUG
env-180   .....................GCUC...................................................................GGAGUUU.GAACCGUACAACGCCCGUGGGUGAAACCACA..................AUG
env-181   .....................ACGGG........GGUCGGAUA.UUCACACAAAUUUAUAGCCACCAGUAUCAGUGGUGGCGAAGCCACCCAG.GGUUU.GAACCACACAACGCCCGUGGGUGAUACCAC...................AUG
env-182   .....................AAUC...................................................................CAGGUUU.GAACCACUCAACGCCCGUGGGUGAAACCACA..................AUG
Ssp-1-1   ..CGGCACCACCGCG.ACGCCGAUC.....................................................................GGGUU.GAACCACCAAACGCCCGAGGAGAAUACA.....................AUG
env-183   CGACCGGGCCCGACCGCUGCCGAUC.....................................................................GGGUU.GAACCACCAAACGCCCGAGGAGAACACA.....................AUG
Nph-1-1   .....................UCC....................................................................GCCGGUU.GAACCACGAAACGCCCGUGGUGAACAUC.....................AUG
env-184   .....................CCGAGAACU...............................................GACUCGAGUGGGCGUGAAAAUU.GAACCAGAAAACGCCCGCAGAGAAUACCA....................AUG
Hgo-1-1   .....................AAC...................................................................UCGGGAUU.GAACCAGGAAACGCCCGCGGGUGAAACC.....................AUG
Hxa-1-1   .....................AGU...................................................................UCGGGAUU.GAACCAGGAAACGCCCGCAGGUGAUAAUC....................AUG
env-185   .....................ACUC....................................................................GAGAUU.GAACCAGGAAACGCCCGCAGAAACCU.......................AUG
env-186   .....................ACUC....................................................................GAGAUU.GAACCAGGAAACGCCCGCAGAAACCU.......................AUG
env-187   .....................GAC...................................................................GCGGGAUU.GAACCAGGAAACGUCCGCGGGUGAAACU.....................AUG
Nin-1-1   .....................GAGGU...................................................................CGGGAUU.GAACCAGGAAACGCCCGCGGUGAUUACC.....................AUG
env-188   .....................AGG....................................................................CCGGAUU.GAACCAGGAAACGCCCGCGGUGAACACG.....................AUG
env-189   .....................AGG....................................................................CCGGAUU.GAACCAGGAAACGCCCGCGGUGAACACG.....................AUG
env-192   .....................GACGA..............................................................CCCGCCGGGUU.GAACCAGGAAACGUCCGCAGUGAUCACG.....................AUG
env-193   .....................GACGA..............................................................CCCGCCGGGUU.GAACCAGGAAACGUCCGCAGUGAUCACG.....................AUG
Nje-1-1   .....................GUCGA..............................................................CCCGCUGGGUU.GAACCAGGAAACGCCCGCAGUGAUCACG.....................AUG
Nam-1-1   .....................GCCGG..............................................................CCCGCUGGGUU.GAACCAGGAAACGCCCGCAGUGAUCACG.....................AUG
Noc-1-1   .....................ACCGA..............................................................CCCGCUGGGUU.GAACCAGGAAACGCCCGCAGUGAUCACG.....................AUG
env-194   .....................GCGA.....................................................................GGGCU.GAACCAGGCAACG.......................................
env-195   .....................GCC..................................................GAGAUUGGAAAUCUCGCUACGGUUU.GAACCACGCAACGCCCGUGGUGAUAGACAU...................AUG
env-196   .....................CCC.................................................................GCGAUGGUUU.GAACCACGCAACGCCCGUGGUGAUACCA.....................AUG
env-197   .....................ACCC................................................................GCGAUGGUUU.GAACCACGCAACGCCCGUGGUGAUAGCA.....................AUG
env-199   .....................ACCC................................................................GCGAUGGUUU.GAACCACGCAACGCCCGUGGUGAUAACA.....................AUG
env-201   .....................ACCC................................................................GCGAUGGUUU.GAACCACGCAACGCCCGUGGUGAUAACA.....................AUG
env-202   .....................ACGU.............................................................CCCCGUGCGGUUU.GAACCACGCAACGCCCGUGGUGACAAUC.....................AUG
Hla-1-1   .....................CUUC....................................................................GGGAUU.GAACCAGGAAACGCCCGCGGGUGAUAACC....................AUG
Hva-1-1   .....................AGACC..................................................................GCGACUU.GAACCACGCAACGCCCGUGGUGAAUACAU....................AUG
Hsi-1-1   .....................CGC..................................................................GCGCGACUU.GAACCACGCAACGCCCGUGGUGAAUACAU....................AUG
Hma-1-1   .....................CGC..................................................................GCGCGACUU.GAACCACGCAACGCCCGUGGUGAAUACAU....................AUG
Ham-1-1   .....................CGA..................................................................GCGCGACUU.GAACCACGCAACGCCCGUGGUGAAUACAU....................AUG
env-203   .....................CGA..................................................................GCGCGACUU.GAACCACGCAACGCCCGUGGUGAAUACAU....................AUG
Hca-1-1   .....................CGA..................................................................GCGCGACUU.GAACCACGCAACGCCCGUGGUGAAUACAU....................AUG
Hsa-2-1   .....................CGA..................................................................GCGCGACUU.GAACCACGCAACGCCCGUGGUGAAUACAU....................AUG
Hja-1-1   .....................CGA..................................................................GCGCGACUU.GAACCACGCAACGCCCGUGGUGAAUACAU....................AUG
Har-1-1   .....................AAA...................................................................CGCGACUU.GAACCACGCAACGCCCGUGGUGAAUACAU....................AUG
env-206   .....................GACCG.................................................................CGCGACUU.GAACCACGCAACGCCCGAGGUGAAACCC.....................AUG
Hmu-2-1   .....................CGAU.................................................................UCCGGCGUU.GAACCACGCAACGCCCGUGGUGAUACACAC...................AUG

          >>>>>>>>>>>>>>>>>>>>>...........>>>>>>>>>>>>>>>>>>>>>>>>>>>>>>>>>>>>>>>>>>>>>>>>>>>>>>>>>>>>>....>>...............................................
          001.111....11.1..1101...............00000...000.000...0000000.000000000..1.1..................22222....21...............................................
          ---------------------●●●○--------------------------------------------------------------------○RRYU-GAACCR●RRR●●●●●●●●R●●●●●●●○○-------------------AUG
                                                                                                                 3′
```

```
Hka-1-1    ....................GGAU.............................................................UCCGGCGUU.GAACCACGCAACGCCCGUGGUGAUACACAC.................AUG
env-207    ....................GCGGUU..........................................................CCGCGGGGUU.GAACCACGCAACGCCCGUGGUGAAACC....................AUG
env-208    ....................GAGGAG.........................................GCGGCACCGCCG.CC.GAAACGGAAUU.GAACCACACAACGCCCGUGGUGAAACC....................AUG
env-210    ....................GUCGG.........................................................GGCUCGAGGAUU.GAACCAGCAAACGCCCGUGGUGAAACC....................AUG
env-212    ....................CGUCGAA........................................................GCUCGGGAAUU.GAACCACACAACGCCCGUGGUGAAACC....................AUG
env-213    ....................GUCGG.........................................................GGCUCGGGAAUU.GAACCACACAACGCCCGUGGUGAAACC....................AUG
env-214    ....................GAGA..............................................ACGGCCACAGCGCCGAUGGGGGUU.GAACCACACAACGCCCGUGGUGACACAU...................AUG
env-215    ....................GCAC.........................................................GGAUCCGGGAAUU.GAACCACACAACGCCCGUGGUGAAACU....................AUG
env-216    ....................GGAU.........................................................GGCGUCGGGAAUU.GAACCACACGACGCCCGUGGUGAAACC....................AUG
env-217    ....................GGC...........................................................GCUCGCGGGGUU.GAACCAUACAACGCCCGUGGUGAAACC....................AUG
env-218    ....................GGC...........................................................GCUCGCGGGGUU.GAACCAUACAACGCCCGUGGUGAAACC....................AUG
env-219    ....................GGC...........................................................GCUCGCGGGGUU.GAACCAUACAACGCCCGUGGUGAAACC....................AUG
env-220    ....................GGC...........................................................GCUCGCGGGGUU.GAACCAUACAACGCCCGUGGUGAAACC....................AUG
Nmo-1-1    ....................CGG................................................................CGGAGUU.GAACCACACAACGCCCGUGGUGAAACU....................AUG
env-221    ....................GAAG.................................................................GGGCU.GAACCAGCAAACGCCCGUGGUGAAACAG...................AUG
env-223    ....................GCGA.................................................................GGGCU.GAACCAGACAACGCCCGUGGUGAGACAG...................AUG
env-224    ....................ACGU.................................................................GGGCU.GAACCAACAAACGCCCGUGUGGUGAGACCA.................AUG
env-226    ....................UUU..........................................................GGCGUCGAGAGUU.GAACCAUGCAACGCCCGUGGUGAAACC....................AUG
env-227    ....................ACCA.............................................................CGCCGCCUU.GAACCACACAACGCCCGUGGUGAUACC....................AUG
env-228    ....................AGAC................................................................GCGGCU.GAACCACGAAACGCCCGUGGUGAAACACG..................AUG
env-229    ....................CACA.................................................................CGGCU.GAACCACCAAACGCCCGUGGUGAAACACG..................AUG
Hci-1-1    ....................GUA.........................................................GAAUCCUGCCCGCU.GAACCACUCAACGCCCGUGGUGAUAGACC..................AUG
Hut-1-1    ....................GGA................................................................GCCGGCU.GAACCACCCAACGCCCGCGGUGAAAGAU...................AUG
env-230    ....................GGAGA............................................GGCGGC..ACGACCGCCGACCGGCU.GAACCACGCAACGCCCGCGGUGACAAC....................AUG
env-231    ....................GCUC................................................................CCGGCU.GAACCACCCAACGCCCGUGGUGAAACAU...................AUG
env-232    ....................ACUC................................................................CCGGCU.GAACCACGCAACGCCCGUGGUGAAACAU...................AUG
env-233    ....................ACUC................................................................CCGGCU.GAACCACCCAACGCCCGUGGUGAAACAU...................AUG
env-234    ....................ACCGA.........................................................CUCGCACGGUUU.GAACCACUCGACGCCCGUGGUGAGACAAC..................AUG
env-235    ....................ACAC................................................................GGAGUU.GAACCACUUGACGCCCGUGGUGAUAAACU..................AUG
env-236    ....................ACGC.................................................................GGGUU.GAACCACCUGACGCCCGUGGUGAUAUAC...................AUG
Nba-1-1    ....................AUG........................................................GCCCCGGUCAGGAUU.GAACCAGGAAACGCCCGCGGGAACAUACA..................AUG
env-237    ....................AUG........................................................GCCCCGGUCAGGAUU.GAACCAGGAAACGCCCGCGGGAACAUACA..................AUG
Nsu-1-1    ....................AUG........................................................GCCCCGGUCGGGAUU.GAACCAGAAAACGCCCGCGGGAACAUACA..................AUG
Npa-1-1    ....................ACGC.......................................................GCUCCGGUCGGGAUU.GAACCACGAAACGCCCGCGGGUGAAUACAG.................AUG
Nal-2-1    ....................GCGC.......................................................GCCCCGGUCGGGAUU.GAACCACGAAACGCCCGCGGGUGAAUACAG.................AUG
Nga-1-1    ....................ACAC.......................................................GCUCCGGUCGGGAUU.GAACCACGAAACGCCCGCGGUGAAUACAG..................AUG
Hdj-1-1    ....................AUG........................................................GCCCCGGUCGGGAUU.GAACCAGGAAACGCCCGCGGGUGAACAUAC.................AUG
Hli-3-1    ....................AUGU.......................................................GCCCCGGUCGGGAUU.GAACCAGGAAACGCCCGCGGGUGAAUACG..................AUG
Hje-2-1    ....................UCG........................................................GCCCCGGUCGGGAUU.GAACCAGCAAACGCCCGCGGGUGAAUACA..................AUG
Hth-2-1    ....................UUG........................................................GCCCCGGUCGGGAUU.GAACCAGCAAACGCCCGCGGGUGAAUACA..................AUG
Hsa-10-1   ....................GCCG.......................................................GCCCCGGUCAGGAUU.GAACCAGGAAACGCCCGCGAGUGAUAAUAC.................AUG
env-238    ....................UGGUU...............................................................GGGAUU.GAACCACGAAACGCCCGCGGUGAUUACG...................AUG
env-239    ....................UGGUU...............................................................GGGAUU.GAACCACGAAACGCCCGCGGUGAUUACG...................AUG
Hni-1-1    ....................CCC.........................................................GCCCCGUUGGGAUU.GAACCAGGAAACGCCCGCGGUGAUACAU...................AUG
Nch-1-1    ....................CAC................................................................UCGGAUU.GAACCAGGAAACGCCCGCGGUGAUAAUCA..................AUG
Nhu-1-1    ....................CGC................................................................UCGGAUU.GAACCAGGAAACGCCCGCGGUGACAAUCA..................AUG
Nve-1-1    ....................UCG........................................................GCCCCGGUCGGGAUU.GAACCAGCAAACGCCCGCGGAGAAUACA...................AUG
Hsa-8-1    ....................ACGGG.......................................................CCCCGGCCAGGAUU.GAACCAGGAAACGCCCGCGGUGAUACAC...................AUG
Htu-1-1    ....................ACCGC.......................................................CCCCGGUCGGGAUU.GAACCAGGAAACGCCCGCGGUGAUACAC...................AUG
env-243    ....................ACCGA.......................................................CCCCGGUCGGGAUU.GAACCAGGCAACGCCCGCGGUGAUAGCU...................AUG
Hca-3-1    ....................GCGA..............................................................CGGCGGUU.GAACCACGCAACGCCCGGCGGUGAACACCC.................AUG
env-244    ....................GGA................................................GACCUCC........CGCGGUUU.GAACCACUCGACGCCCGUGGUGAUAGCCC..................AUG
env-245    ....................GAGA................................................CGAG.CCGUCACCGGCGUGAUU.GAACCACUCAUUGCCCGCGGUGAUACCA...................AUG
env-246    ....................GGAG..............................................GCGGCACCG.....CCGCCGGGUU.GAACCACGCAAGGCCCGCACGCCAACC....................AUG
env-247    .................CCCGAGGC.................................................................GAUU.GAACCAAGAACGCCCGUAGGUACACU.....................AUG
env-248    ............CUGUCACCGA................................................................CGC.GAUU.GAACCAGGAAAGCCCGCAGGCAAACU.....................AUG
env-249    ............UUGUCGCCGAGGC.................................................................GAUU.GAACCAGGAAAGCCCGCAGGCAAACU.....................AUG
env-250    ....................UUCCG.............................................................UGAGGGUU.GAACCACACACGCCCGUGGUGACAGAUC...................AUG
env-251    ....................UCUCAGCCUAC........................................................GAGGGUU.GAACCACUCACGCCCGUGGUGACAGAUU...................AUG
env-253    ....................GCGA.................................................................GGGUU.GAACCACGAACGCCCGUGGUGACAGAC....................AUG
Hsu-1-1    ....................CUCGA..............................................................GCGGAUU.GAACCACCAAACGCCCGCGAGACAAUC....................AUG
Hsp-2-1    ....................UGC.........................................................GUGCCUCGCGGAUU.GAACCACGCAACGCCCGCGGACAACAC....................AUG
Has-1-1    ....................CUGA...............................................................CCGGUCU.GAACCAGGAACGCCCGCGGUGAUACAC....................AUG
env-254    ....................ACGU.......................................................CCUCGAUGC.GGUCU.GAACCACGAACGCCCGCGGUGAAACG.....................AUG
Hag-1-1    ....................AUC.........................................................GGGCCACGC.GGCU.GAACCACCAACGCCCGCAGACAACAC.....................AUG
Hsa-4-1    ....................CCC.........................................................GGACCACGC.GGCU.GAACCAUCAACGCCCGCAAACAACAC.....................AUG
Hsa-5-1    ....................GGU.........................................................GGACCACGC.GGCU.GAACCAUCAACGCCCGCAGCAACC.......................AUG
env-255    ....................UUCAC.................................................................GGCU.GAACCGUUAAGAAUCCGCGAGGUUAGCAA..................AUG
env-256    ....................UUAC..................................................................GGCU.GAACCGUUAAGAAUCCGCGAGGUUAGCAA..................AUG
env-262    ....................UUAC..................................................................GGCU.GAACCGUUAAGAAUCCGCGAGGUUAGCAA..................AUG
env-266    ....................UUUGU.................................................................GGCU.GAACCGUUAAGAAUCCGCGAGGUUAAGAA..................AUG
Mae-1-1    ....................AAGA...................................................................GCU.GAACCCAUAAACAACAAUUACAAUAACUUCCAACAACCAAUUAUUAUAUG
env-267    ....................UGAU.............................................................GGAACGUUU.GAACCACGAAACGCGAAUGCACAGGAGACACGAAACUA.........AUG
env-268    ....................AGAUG..................................................................GUU.GAACCACAAAUGGAGGGGAGAUUCCACUAG.................AUG
env-269    ....................AGAUG..................................................................GUU.GAACCACAAAUGGAGGGGAGAUUCCACUAG.................AUG
env-270    ....................GAAAAAAA............................................................GCAGUU.GAACCAUAAAAUAGGUGAAUUUGUAA.....................AUG
env-271    ....................GAAAAAG.............................................................GCAGUU.GAACCAUAAAAUAGGUGAAUUUGUAA.....................AUG
env-273    ....................GAAAAAG.............................................................GCAGUU.GAACCAUAAAAUAGGUGAAUUUGUAA.....................AUG
env-274    ....................ACAAA...............................................................AAGGCU.GAACCAUAAACUAAUUGAGAGGUGAAAAU..................AUG
env-276    ....................ACAAA...............................................................AAGGCU.GAACCAUAAACUAAUUGAGAGGUGAAAAU..................AUG
env-277    ....................GCAG..................................................................GGCU.GAACCGUAAACAAGUGUGAGGUGAAAA....................AUG
env-278    ....................GCAG..................................................................GGCU.GAACCGUAAACAAGUGUGAGGUGAAAA....................AUG
env-281    ....................UUAAU.................................................................GGUU.GAACCGUAGACUUGAAGGAGAGUUGAGA...................AUG

           >>>>>>>>>>>>>>>>>>>>..........>>>>>>>>>>>>>>>>>>>>>>>>>>>>>>>>>>>>>>>>>>>>>>>>>>>>>>>>>>>>....>>.............................................
           001.111...11.1..1101..............00000..000.000...0000000.000000000..1.1................22222....21.............................................
           --------------------●●●○---------------------------------------------------------------○RRYU-GAACCR●RRR●●●●●●●●R●○○○○○○○○------------------AUG
                                                                                                           3′
```

```
env-283  ....................UUCG.......................................................GGACU.GAACCAUAAGCAAACAAUACAGAUAGGAUGAAAAAUAU..........AUG
env-284  ....................UUCG.......................................................GGACU.GAACCAUAAGCAAACAAUACAGAUAGGAUGAAAAAUAU..........AUG
env-286  ....................UUCG.......................................................GGACU.GAACCAUAAGCAAACAAUACAGAUAGGAUGAAAAACAU..........AUG
env-287  ....................UUUG......................................................GGGACU.GAACCGCAAACAGAAAAAACAACAAAAAGUGAUUAAAC..........AUG
env-289  ....................AGUAC......................................................GGGCU.GAACCGCAAACAUAAGAUUAGAGGUGAAAAAAGAAAG...........AUG
env-290  ....................UAU.......................................................UGGGCU.GAACCGUAAACUUAAGAUUAGAGGUGAAAAAAGAAAG...........AUG
env-291  ....................AAACA......................................................GGGCU.GAACCGAAAACAUAAACUGAGAGGUGAAAAAGGAAAG...........AUG
env-293  ....................AAC.......................................................GGGGCU.GAACCGUAAACUUAAGAUGUGAGGUGAAAGAAGAAAG...........AUG
env-295  ....................UUCG.......................................................GGACU.GAACCGUAAGCAACAAAACACAUAGGAGAAAAAAUUUAA.........AUG
env-296  ....................UUCG.......................................................GGACU.GAACCGUAAGCAACAAAACACAUAGGAGAAAAAAUUUAA.........AUG
env-297  ....................GAAA........................................................GGCU.GAACCGUAAACAAAUAAUACACAUAAUAAACAUAG.............GUG
env-298  ....................GAAA........................................................GGCU.GAACCGUAAACAAUUAACACACAGGUGAAGAAACAAA...........AUG
env-299  ....................GAAA........................................................GGCU.GAACCGUAAACAAUUAACACACAGGUGAAGAAACAAA...........AUG
env-301  ....................GAAA........................................................GGCU.GAACCAUAAACAAAUAAUACUUAG........................GUG
env-302  ....................GAGA........................................................GGCU.GAACCAUAAACAAGUAAUACUUAGGUGAAAAAC...............AUG
env-304  ....................CACGU.......................................................GGGCU.GAACCGUAAGCAUAAAACACAUUGGAGGUAAAAAAU............AUG
env-308  ....................UAA........................................................AGGUU.GAACCGUAAACGUAAAGGAGAAUUAGACA..................AUG
env-309  ....................UAA........................................................AGGUU.GAACCGUAAACGUAAAGGAGGAUUUAGAAACGGCUUACAAGUAU....UUG
env-310  ....................UAAA........................................................GGUU.GAACCGUAAACGUAAAGGAGGAU.........................UUG
env-311  ....................CUUAGA......................................................GGUU.GAACCGUAAGAAUAGGAGAAUUAGAGA.....................AUG
env-312  ....................CAGA.......................................................GGGCU.GAACCACAAACAAAGCGAAUAGAUCACAACAGAGG.............AUG
env-317  ....................GCAG.......................................................GGGCU.GAACCACAAACAAAGCGAAUGAAUUACAGCAAAGG.............AUG
env-318  ....................GCAG.......................................................GGGCU.GAACCACAAACAAAGCGAAUGAAUUACAGCAAAGG.............AUG
env-319  ....................GCAG.......................................................GGGCU.GAACCACAAACAAAGCGAAUGAAUUACAGCAAAGG.............AUG
env-321  ....................CUCUGA......................................................GGCU.GAACCACAAACAAAGUGAAUCUAUUACGACAAAGG.............AUG
env-322  ....................UUAG.......................................................GGGCU.GAACCACAAACAAAGUGAAUAUAUUACGACAAAGG.............AUG
env-323  ....................UUAG.......................................................GGGCU.GAACCACAAACAAAGCGAAUAUAUUACGACAAAGG.............AUG
env-324  ....................CAUAGA......................................................GGCU.GAACCACAAACAAAGUGAAUAUAGUUACAGCAAAGG............AUG
env-325  ....................CAUAGA......................................................GGCU.GAACCACAAACAAAGUGAAUAUAGUUACAGCAAAGG............AUG
env-326  ....................AUUA.......................................................GGGCU.GAACCACAAACAAAGCGAAUUUAAUCACUGCAAAGGAAGGCAA.....AUG
env-327  ....................AUUA.......................................................GGGCU.GAACCACAAACAAAGCGAAUUAAAUCACGGCAAAGGAAGGCAA.....UUG
env-328  ....................AUUA.......................................................GGGCU.GAACCACAAACAAAGCGAAUUUAAUCACGGCAAAGGAAGGCAA.....UUG
Pab-1-1  ....................GUAAU........................................................GGGG.GAACCACGAGGGAAGGAGGGAAAGAA......................AUG
Psp-2-1  ....................GAGUU........................................................GGGA.GAACCACAAACAGGAGGGAAAAGGG.......................AUG
Tga-1-1  ....................CUCUG........................................................CGGA.GAACCACAAACAAGGAAGGAGGGUGCAAG...................AUG
Tsp-3-1  ....................CACUG........................................................CGGA.GAACCACAAACGUGGAAGGAGGGUGCAAG...................AUG
Tsp-2-1  ....................CUCUG........................................................CGGA.GAACCACAAACGUGGGAGGGAAUUGAA.....................AUG
Tna-1-1  ....................CUCUG........................................................CGGA.GAACCACAAACGUGGGAGGGAAUUGAA.....................AUG
Teu-1-1  ....................CUCUG........................................................CGGA.GAACCACAAACAGGGAAGAGGUGAAAGAG...................AUG
env-329  ....................CUCUG........................................................CGGA.GAACCACAAACAGCGAAAGAUGAGGUGGAAAAA...............AUG
Tsp-4-1  ....................CUCUG........................................................CGGA.GAACCACAAACAGCGAAAGAUGAGGUGGAAAAA...............AUG
Tsi-1-1  ....................CUCUA........................................................CGGA.GAACCAUAAACAGUGAAAGACGAGGUGAAAAAA...............AUG
Tli-1-1  ....................CUCUG........................................................CGGA.GAACCACAAACAGUGAAGAACGAGGUGAAAAAG...............AUG
env-330  ....................CUCUG........................................................CGGA.GAACCACAAACAGUGAAAGCGAGGUGAAGAAG................AUG
Tba-1-1  ....................CUCUG........................................................CGGA.GAACCACAAACAGCGAAAGCGAGGUGGAAGGA................AUG
Tpa-1-1  ....................CUCUG........................................................CGGA.GAACCACAAACAGCGAAAGCGAGGUGGAAAGA................AUG
Ppa-1-1  ....................CUAAG........................................................CGGA.GAACCAUAAACAGCAAAAACGAGGUGAAAAAG................AUG
Pfu-2-1  ....................GGAU.........................................................GGGG.GAACCACAAACUAGGGAGGGAGAGCA......................AUG
Pho-1-1  ....................GGAU.........................................................GGGG.GAACCACAAACCAGGGAGGGAGAGCA......................AUG
Psp-1-1  ....................GAGCC........................................................GGGG.GAACCACAAACUAGGGAGGGAGCAAG......................AUG
Pya-1-1  ....................GGAC.........................................................GGGA.GAACCACAAACGAGGAAGGGGUGAAAGGA...................AUG
Tko-1-1  ....................GGAA.........................................................GGGA.GAACCACAAACAGGGAAGAGGUGAACGAG...................AUG
Tsp-1-1  ....................GUAA.........................................................GGGA.GAACCACAAACAUGGAAGAGGUGAACGAG...................AUG
Pfe-1-1  ....................GUAA.........................................................GGGA.GAACCACAAACAUGGAAGGAGGGAACGAG...................AUG
Ton-1-1  ....................GUAA.........................................................GGGA.GAACCACAAACGAGGAAGGAGGGAGCAAG...................AUG
Tzi-1-1  ....................GGAA.........................................................GGGA.GAACCACAAACGGGGAAGAGGUGUGAAAG...................AUG
env-331  ....................ACAAGCAA.....................................................AGGGUU.GAACCAUAAACUAAGAAGGAGGAAUGCUUUAAA...............AUG
env-332  ....................AUAAGCAA.....................................................AGGGUU.GAACCAUAAACUAAGAAGGAGGAAUGCUUUAAA...............AUG
env-333  ....................AAACG.......................................................GGGUU.GAACCGCAAACAAAGGAGGAACAAUGAAU...................AUG
env-334  ....................GCAA.........................................................GGCU.GAACCAUAAACAACCGAACACACGUGAACUGAA...............AUG
env-336  ....................AUAUA........................................................GGCU.GAACCGUAAACUGUUGAGGAGUGAAACUAGAA................AUG
env-338  ....................GCAA.........................................................GGCU.GAACCAUAAACAAGUGGCACAAGUGAACAAAG................AUG
env-339  ....................GUUUA........................................................GGUU.GAACCAUAAACUAGUGGAGGUGAGA.......................AUG
env-341  ....................GAAA.........................................................GGUU.GAACCGUAAACUAGAAGCGAGGUGAAAAAGGAAA..............AUG
env-344  ....................GAAAA........................................................GGUU.GAACCGUAAACUAGAAGCGAGGUGAAAAAGGAAA..............AUG
env-348  ....................GUAUA........................................................GGCU.GAACCAUAAACCAAGAAAGCGAGGUGAAAAA.................AUG
env-349  ....................GCAUA........................................................GGCU.GAACCAUAAACCGAAAAGGCGAAGUGAAAAA.................AUG
env-353  ....................GUAUA........................................................GGCU.GAACCAUAAACUAAGAACGAGGUGAAAAA...................AUG
env-354  ....................GUAUA........................................................GGCU.GAACCGAAAACUAGAAGCGAGGUGGAAAA...................AUG
env-356  ....................GUAUA........................................................GGCU.GAACCAUAAACUAAAAGCGAGGUGGAAAA...................AUG
env-358  ....................GUAUA........................................................GGCU.GAACCAUAAACCAAGAGAGCGAGGUGAAAAA.................AUG
env-359  ....................UUAA.........................................................GGCU.GAACCAUAAACUAAAAACGAGGUGAAAAA...................AUG
env-360  ....................UUAA.........................................................GGCU.GAACCAUAAACUAAAAACGAGGUGAAAAA...................AUG
env-362  ....................GCGUA........................................................GGCU.GAACCAUAAACUAAAAGCGAGGUGGAAAA...................AUG
env-363  ....................GAGAA........................................................GGCU.GAACCAUAAACUAACGUUGAGGUUGAAAGAAA................UUG
env-364  ....................GUGCA........................................................GGCU.GAACCGUAAACCAAAAGCGAGGUGAAAAUGA.................AUG
env-365  ....................GUACA........................................................GGCU.GAACCGAAAACUAAAAGCGAGGUGGAAAA...................AUG
env-366  ....................UAUG.........................................................GGCU.GAACCAUAAACUAAAAGGCGAGGUAAAAAA..................AUG
env-367  ....................UUUA.........................................................GGCU.GAACCAUAAACUGAAUGGCGAGAUGAAAAA..................AUG
env-369  ....................GUAUU........................................................GGCU.GAACCAUAAACUAAAAGGCGAGGUGAAAAA..................AUG
env-370  ....................UAUA.........................................................GGCU.GAACCAUAAACUAAAUGGCGAGGUGAAAAA..................AUG
env-371  ....................UAUA.........................................................GGCU.GAACCAUAAACUAAAUGGCGAGGUGAAAAA..................AUG
env-375  ....................GCAUA........................................................GGCU.GAACCAUAAACUAAAGAGUGAGGUGAAAAA..................AUG

         >>>>>>>>>>>>>>>>>>>>..........>>>>>>>>>>>>>>>>>>>>>>>>>>>>>>>>>>>>>>>>>>>>>>>>>>>>>>>>....>>..........................................
         001.111....11.1..1101..........  ....00000...000.000...0000000.000000000..1.1..................22222....21..........................................
         --------------------●●●○------------------------------------------------------○RRYU-GAACCR●RRR●●●●●●●●R●●●●●●●○○---------------------AUG
                                                                                                   3′
```

```
env-376  .....................GCGUA.......................................................................GGCU.GAACCAUAAACUAAAGAGUGAGGUGAAAAA...................AUG
env-377  .....................GCAUA.......................................................................GGCU.GAACCAUAAACUAAAGAGUGAGGUGAAAAA...................AUG
env-378  .....................GCAUA.......................................................................GGCU.GAACCAUAAACUAAAGAGUGAGGUGAAAAA...................AUG
env-379  .....................GCGUA.......................................................................GGCU.GAACCAUAAACUAAAGAGUGAGGUGAAAAA...................AUG
env-380  .....................GCGUA.......................................................................GGCU.GAACCAUAAACUAAAGAGUGAGGUGAAAAA...................AUG
env-381  .....................GCAGA.......................................................................GGCU.GAACCAUAAACUAAAGAGUGAGGUGAAAAA...................AUG
env-382  .....................GCAUA.......................................................................GGCU.GAACCAUAAACUAAAGAGUGAGGUGAAAAA...................AUG
env-383  .....................GCAUA.......................................................................GGCU.GAACCAUAAAGUAAAGAGUGAGGUGAAAAA...................AUG
env-384  .....................GCAUA.......................................................................GGCU.GAACCAUAAACUAAAGAGUGAGGUGAAAAA...................AUG
env-385  .....................UAA.......................................................................UAGGCU.GAACCAUAAACUAAGAGGCGAGGUGAGAGA...................AUG
env-386  .....................UAA.......................................................................UAGGCU.GAACCAUAAACUAAGAGGCGAGGUGAGAGA...................AUG
env-387  .....................UAA.......................................................................UAGGCU.GAACCAUAAACUAAGA.....................................
env-388  .....................GUAAA.......................................................................GGCU.GAACCAUAAACUAGUGUGAGGUGAAUUAAA...................AUG
env-390  .....................GUAAA.......................................................................GGCU.GAACCAUAAACUAGUGUGAGGUGAAUAAA....................AUG
env-392  .....................GCAU........................................................................GGUU.GAACCAUAAACUAGUGCGAGGUGAAAAG.....................AUG
env-402  .....................GCAU........................................................................GGUU.GAACCAUAAACCAGUGCGAGGUGAAAAG.....................AUG
env-406  .....................GUUU........................................................................GGUU.GAACCAUAAACAAUAAUGAGGUGAAAAACAA..................AUG
env-408  .....................GUUU........................................................................GGUU.GAACCAUAAACAAUAAUGAGGUGAAAAACAA..................AUG
env-410  .....................AUUU........................................................................GGUU.GAACCAUAAACCGUGGGCGAGGUGAAAAAG...................UUG
env-412  .....................AUUU........................................................................GGUU.GAACCAUAAACUGUGGCGAGGUGAAAAAGG...................UUG
env-413  .....................AUUU........................................................................GGUU.GAACCAUAAACUGUGGCGAGGUGAAAAAGG...................UUG
env-414  .....................UCA........................................................................UGGUU.GAACCGUAAACUAAGCGAGGUGAAAAA......................AUG
env-416  .....................UCA........................................................................UGGUU.GAACCGUAAACUGAAGCGAGGUGAAAAA.....................AUG
env-418  .....................GUAA........................................................................GGUU.GAACUAUAAACUGGUGCGAGGUGAAAAG.....................GUG
env-420  .....................GUAA........................................................................GGUU.GAACCAUAAACUAGUGUGAGGUGAAAAA.....................GUG
env-422  .....................GUAA........................................................................GGUU.GAACCAUAAACUAGUGUGAGGUGAAAAA.....................GUG
env-424  .....................AAACC......................................................................GGGCU.GAACCGCAAACAUAAGAUGAGAGGUGAAAAAAGAAAG............AUG
env-425  .....................AGCAC......................................................................GGGCU.GAACCGUAAACGUAAGAUGAGAGGUGAAAAAAGAAAG............AUG
env-427  .....................AGCAC......................................................................GGGCU.GAACCGUAAACGUAAGAUGAGAGGUGAAAAAAGAAAG............AUG
env-428  .....................AAC.......................................................................UGGGCU.GAACCGUAAGCAUAAAAUGUGAGGUGAAAAAAGAAG.............AUG
env-429  .....................AGCAC......................................................................GGGCU.GAACCGUAAACAUUAGAUGUGAGGUGAAAAAAGAGAG............AUG
env-432  .....................AACAC......................................................................GGGCU.GAACCGCAAACAUAAGAUGUGAGGUGAAAAAAGAAAG............AUG
env-433  .....................AAACC......................................................................GGGCU.GAACCGCAAACAUAAGAUGAGAGGUGAAAAAAGAAAG............AUG
env-438  .....................AAUCAAAA..................................................................AGGGUU.GAACCAUAAACUNAAAAGUGAUUAAAA......................AUG
env-439  .....................AAUCAAAA..................................................................AGGGUU.GAACCAUAAACUGAAAAGUGAUUAAAA......................AUG
env-440  .....................AUA........................................................................AGGUU.GAACCAUAAACAGGAGAGCAGAUAGG.......................AUG
env-441  .....................UUA........................................................................AGGUU.GAACCACAAACUGGAGAAUGCAUAA........................AUG
env-442  .....................UUA........................................................................AGGUU.GAACCGUAAACUGGAGAAUACGUAAG.......................AUG
env-443  .....................UUA........................................................................AGGUU.GAACCGUAAACUGGAGAAUACGUAAG.......................AUG
env-444  .....................UAA........................................................................AGGUU.GAACCACAAACUGAGGAACCAUAA.........................AUG
env-446  .....................UUA........................................................................AGGUU.GAACCUCAAACUGAGGAAUUGGA..........................AUG
env-447  .....................UGA........................................................................AGGCU.GAACCGUUAAACCGUUGAGGAGGAAACAUA..................GUG
env-449  .....................GUAA........................................................................GGUU.GAACCGUUAAGCUGGAGAUCACUUAG.......................AUG
env-450  .....................GUAA........................................................................GGUU.GAACCGUUAAGCUGGAGAUCACUUAG.......................AUG
env-453  .....................GCAAU.......................................................................UGGG.GAAC.AAAAACGGGGAGGAGGGGAGAAAAAGGA.....................
Mvo-1-1  .....................GAAU...........................................................................GCCGAACCCGGAAACAAAAUAUUGACUACAGUGAGUGAUAAA..........AUG
env-454  .....................UUCU.......................................................................GGACU.GAACCUAGGUGAAAAUAAUGGUACGAUCAAUGU..............AUG
ume-1-1  .....................AUAUUCAUCAUGACA.........................................................GGAUAGGUU.GAACCAGGAAACACUAUUCAAAGGAGACACGAAACUA...........AUG
CMe-2-1  .....................AUAA.......................................................................GGGCU.GAACCGUAAAACUUAGGUGAAUACAAUGAAAUCA..............AUG
Mev-1-1  .....................AAAUA.....................................................................UGAGUU.GAACCAUGAAACUAAAAUGAAACCAGGUGAGAAAA.............UUG
env-455  .....................UAAA......................................................................AAAAGCCGAACCUUAGGUGAAUAUC..............................AUG
env-457  .....................UAAA......................................................................AAAAGCCGAACCUUAGGUGAAUAUC..............................AUG
env-460  .....................AAA......................................................................AAAAAGCCGAACCUCAGGUGAAUAUC..............................AUG
env-461  .....................AAA......................................................................AAAAAGCCGAACCUUAGGUGAAUAUC..............................AUG
env-462  .....................AGACC.....................................................................UAAGCU.GAACCCUUGAAACUUUUAUCGGAGACGAAACUUA..............AUG
Mzh-1-1  .....................AAACC...................................................................GAUGAAUU.GAACCAUGAAACUAAGAUGAAACAAAGGUGACCAAA............UUG
env-463  .....................UUU........................................................................UGGCU.GAACCGUAAAACUGCGGAUGGUGAGUGGG...................GUG
env-465  .....................AAAAAG...................................................................UCGGGCU.GAACCGUGAAAGGUGAUUAAAAG........................AUG
env-466  .....................GUAA.......................................................................GGGCU.GAACCUUGAGGUGACACUAA...........................AUG
env-467  .....................AACA.......................................................................GAGCU.GAACCGUAAAACUUUAGGUGAAUACA.....................AUG
env-469  .....................UCUU.....................................................................CCGGGCU.GAACCUGAAAGGUGUAAAAAUGGCACGAUCA...............AUG
Mta-1-1  .....................UCUU.....................................................................CCGGGCU.GAACCUGAAAGGUGUAAAA...........................AUG
env-472  .....................GCGA.......................................................................GGGCU.GAACCGUAAAACCAUGGAGCAACGCC.....................AUG
env-473  .....................GAAA.......................................................................GGGCU.GAACCGUAAAACCAUGGAGCAAUGCC.....................AUG
env-474  .....................AUCC.......................................................................AAGCU.GAACCUUGAGGUGAACCGAUUCAAUGGCAAAAUCA.............AUG
Mpa-1-1  .....................AAU......................................................................UUGAGCU.GAACCUUGGUGAAGAUCAUGUCAAAAUCUAUGU..............AUG
env-475  .....................UCA.......................................................................CAGGCU.GAACCUUGAGGUGUUAUAACAC.........................AUG
env-476  .....................UUA........................................................................CAGGCCGAACCUUGAGGUGUUAUAACAC.........................AUG
env-477  .....................GAAA.......................................................................CGUGCCGAACCUGAGGUGAAAUCCAUAUGGCAAAAUCA...............AUG
env-479  .....................UCUUU.......................................................................GGGCCGAACCUNAGGUGAUUACC............................AUG
env-480  .....................CAAAAAA....................................................................GGGGCCGAACCUUAGGUGAUU.................................
env-481  .....................CAAAAAA....................................................................GGGGCCGAACCUUAGGUGAUUACC............................AUG
env-484  .....................CAAAAAA....................................................................GGGGCCGAACCUUAGGUGAUUACC............................AUG
env-485  .....................CAAAAAA....................................................................GGGGCCGAACCUUAGGUGAUUACC............................AUG
env-486  .....................GUUAAA......................................................................GAGCCGAACCUUAGGUGAUUAUC............................AUG
env-487  .....................GUAAA.......................................................................GAGCCGAACCUUAGGUGAUUAUC............................AUG
env-488  .....................CACGU.......................................................................GAGCCGAACCUUAGGAGAUCAAC............................AUG
env-489  .....................CACGU.......................................................................GAGCCGAACCUUAGGAGAUCAAC............................AUG
env-490  .....................UACGU.......................................................................GAGCCGAACCUUAGGUGAUUAUC............................AUG
env-491  .....................CACGU.......................................................................GAGCCGAACCUUAGGUGAGUAUC............................AUG
env-492  .....................CACGU.......................................................................GAGCCGAACCUUAGGUGAGUAUC............................AUG
env-495  .....................UAA........................................................................CAGAGCCGAACCUUAGGUGANUAUC............................AUG
env-496  .....................GCAA........................................................................UGAGCCGAACCUUAGGUGAUCACC............................AUG

         >>>>>>>>>>>>>>>>>>>>>..........>>>>>>>>>>>>>>>>>>>>>>>>>>>>>>>>>>>>>>>>>>>>>>>>>>>>>>>>>>>>>>>>>>>>....>>.............................................
         001.111....11.1..1101...........
....00000...000.000...0000000.000000000..1.1..................22222....21.............................................
         ---------------------●●●○-------------------------------------------------------------------------○RRYU-GAACCR●RRR●●●●●●●●R●○○○○○○○○-------------------AUG
                                                                                                                      3′
```

```
env-497   ....................CCAA..........UGAGCCGAACCUUACAGGUGAUCAGACUAUGGUUAAAUCG...............AUG
env-499   ....................GGAAA.........UGAGCCGAACCUUAGGUGAUUACCAUGGUAAAAUCA...................AUG
env-500   ....................GCAA..........UGAGCCGAACCUUAGGUGAUUACCAUGGUAAAAUCA...................AUG
env-502   ....................GCAA..........UGAGCCGAAC.............................................
env-503   ....................GCAA..........UGAGCCGAACCUUAGGUGAUUACCAUGGUAAAAUCG...................AUG
env-505   ....................GCAA..........UGAGCCGAACCUUAGGUGAUUACCAUGGUAAAAUCG...................AUG
env-506   ....................UCUUU..........GGGCCGAACCUUAGGUGAUUACC................................AUG
env-507   ....................UCUUU..........GGGCCGAACCUUAGGUGAUUACCAUGGUAAAAUCG...................AUG
env-508   ....................UCUUU..........GGGCCGAACCUCAGGUGAUUACCAUGGUAAAAUCA...................AUG
env-509   ....................UCUUU..........GGGCCGAACCUUAGGUGAUCACCAUGGUAAAAUCG...................AUG
env-511   ....................UCUUU..........GGGCCGAACCUUAGGUGAUCACCAUGGUAAAAUCG...................AUG
env-512   ....................UCUUU..........GGGCCGAACCUUAGGUGAUCACCAUGGUAAAAUCG...................AUG
env-513   ....................UCUUU..........GGGCCGAACCUUAGGUGAUCACCAUGGUAAAAUCG...................AUG
env-514   ....................UCUUU..........GGGCCGAACCUUAGGUGAUUACCAUGGUAAAAUCA...................AUG
env-515   ....................UCUUU..........GGGCCGAACCUUAGGUGAUCACCAUGGUAAAAUCG...................AUG
env-516   ....................UCUUU..........GGGCCGAACCUUAGGUGAUCACC................................AUG
env-517   ....................AUAU..........CAGGCU.GAACCUUUGGAGAGAUC................................AUG
env-522   ....................AUUG.........GCAAGCC.GAACCUGGAGCAUAAU.................................AUG
env-525   ....................AAUUCA.......GUAAGCC.GAACCUGGAGAAUAAC.................................AUG
env-528   ....................UUACC..........GGCU.GAACCUUGAGGUGUCAAUAACACAUGGUAAAAUCA..............AUG
env-529   ....................UUACC..........GGCU.GAACCUUGAGGUGUCAAUAACACAUGGUAAAAUCA..............AUG
env-530   ....................UUACC..........GGCU.GAACCUUGAGGUGUCAAUAACACAUGGUAAAAUCA..............AUG
env-531   ....................UUACC..........GGCU.GAACCUUGAGGUGUCAAUAACACAUGGUAAA...................
env-532   ....................UUACUU.........GGGCCGAACCUUGAGGUGUUAUAACACAUGGUAAAAUCG...............AUG
CMe-1-1   ....................GUCAU..........GGGCCGAACCUUGAGGUGUUAUCGCACAUGGUAAAAUCG...............AUG
env-534   ....................UUACGU.........GGGCCGAACCUUGAGGUGUUAUAACACAUGGUAAAAUCG...............AUG
env-535   ....................GCGAU..........GGGCCGAACCUUGAGGUGUUAUAACAGAUGGUAAAAUCG...............AUG
env-536   ....................AAAA..........GAGCU.GAACCUGGAGCAACUAUGUCACGAUCU.......................AUG
env-537   ....................AUAC..........GAGCU.GAACCUGGAGCAACUAUGUCACGAUCA.......................AUG
env-538   ....................AUU..........CAGGCU.GAACCUGGUGAACCGC..................................AUG
env-539   ....................AUU..........CAGGCU.GAACCUGGUGAACCGC..................................AUG
env-540   ....................AUU..........CAGGCU.GAACCUGGUGAACCGC..................................AUG
env-541   ....................GAAAA.........AGGCU.GAACCUGGAGAAUUUAUGUCAAAAUCGAUGUAUUCGU.............AUG
env-542   ....................UCU..........GACGCU.GAACCUGAUGGUGAUCUACCAUGGCGAAAUCGAUGU.............AUG
env-543   ....................UUC.........AGACGCU.GAACCUGAGGCUGAUUGUCUAUGGCGAAAUCGAUGU.............AUG
env-544   ....................AAAAA.........UAGGCU.GAACCUUGGAGAUAUC.................................AUG
env-545   ....................UUCAU..........CCGCU.GAACCUCAGGUGAUCGCA...............................AUG
env-546   ....................UUCAU..........CCGCU.GAACCUCAGGUGAUCGCA...............................AUG
Mli-1-1   ....................GAAACCA........GCGCU.GAACCUGAAGGUGAUUAUCAAUGGCAAAGUCAAUGU.............AUG
env-547   ....................GAAA............CGCU.GAACCUGAGGUGAUUUGAAAAAUGGCAAAAUCGAUGU............AUG
Mse-1-1   ....................GAAA............CGCU.GAACCUGAGGUGAUUCUAAACAUGGCAAAAUCGAUGU............AUG
Mma-1-1   ....................GAAA............CGCU.GAACCUGAGGUGAUUCUAAACAUGGCAAAAUCGAUGU............AUG
Msp-5-1   ....................GAAA............CGCU.GAACCUGAGGUGAUUCUAAACAUGGCAAAAUCGAUGU............AUG
env-552   ....................GAAA............CGCU.GAACCUGAGGUGAUUCUAAACAUGGCAAAAUCGAUGU............AUG
env-553   ....................GAAA............CGCU.GAACCUGAGGUGAUUUGUGAAUGACAAAAUCG.................AUG
env-559   ....................GAAA............CGCU.GAACCUGAGGUGAUUUGUGAAUGACAAAAUCG.................AUG
env-560   ....................GAAA............CGCU.GAACCUGAGG.........................................
env-561   ....................GAAA............CGCU.GAACCUGAGGUGAUUUGUGAAUGACAAAAUCG.................AUG
env-563   ....................GAAA............CGCU.GAACCUGAGGUGAUUUGUGAAUGACAAAAUCG.................AUG
env-564   ....................GAAA............CGCU.GAACCUGAGGUGAUUGAGAGCAUGGCAAAAUCGAUGU............AUG
env-565   ....................GAAA............CGCU.GAACCUGAGGUGAUUGAGAGCAUGGCAAAAUCGAUGU............AUG
env-567   ....................GAAA............CGCU.GAACCUGAGGUGAUUGAGAGCAUGGCAAAAUCGAUGU............AUG
env-568   ....................GAAA............CGCU.GAACCUGAGGUGAUUGAGAGCAUGGCAAAAUCGAUGU............AUG
env-570   ....................GCAA............CGCU.GAACCUGAGGUGAUUCUAAACAUGGCAAAAUCGAUGU............AUG
env-571   ....................GAAA............CGCU.GAACCUGAGGUGAUAAAAGAAUGGCAAAGUCAAUGU.............AUG
env-574   ....................GGAA..........CACGCU.GAACCUGGAUGGUGACAGAGCAUGNCGAAAUCGAUGU............AUG
env-575   ....................GCAA..........CACGCU.GAACCUGGAUGGUGACAGAGUAUGUCGAAAUCGAUGU............AUG
env-576   ....................GCAA..........CACGCUUGAACCUGGAUGGUGACAGAGUAUGUCGAAAUCGAUGU............AUG
env-577   ....................GGAA..........CACGCU.GAACCUGGAUGUGACAGAGC..............................AUG
env-578   ....................UCUAC.........UGAGCU.GAACCUGAAGGUGAAGAAAU.............................AUG
env-579   ....................UCCAUU.........GAGCU.GAACCUGAGGUGAUUUGUUAUGGCAAAAUCGAUGU..............AUG
env-580   ....................CCAU..........UGAGCU.GAACCUGAGGUGAUUUGUAAAUGGCAAAAUCG.................AUG
env-581   ....................UCUU..........CGGGCU.GAACCUGUAGGUGCAAGAAUC............................AUG
env-585   ....................UUCCAU........CGGGCU.GAACCUGAAAGGUGUGAGAA.............................AUG
env-587   ....................UCUUU.........CGAGCU.GAACCUGAAAGGUGCAAGAACC...........................AUG
env-588   ....................UCUUU.........CGAGCU.GAACCUGAAAGGUGCAAGAACC...........................AUG
env-589   ....................GGAAAAAAAG...GCGGCUG..AACCGUGAAACUCUCUUAUUAAAG.........................GUG
env-590   ....................UAAUAACGU.......CAGCU.GAACCGUGAAACUGUAUAAAAUUGGUGAAACAACA..............AUG
env-591   ....................UAAUAACGU.......CAGCU.GAACCGUGAAACUGUAUAAAAUUGGUGAAACAACA..............AUG
env-594   ....................UAAAAGCAU.....GCGGCU.GAACCGUGAAACAAUCAUAAAUUGGUGAAACACA................AUG
env-595   ....................AAAAACA.......GUAGCU.GAACCGUGAAACUAUAAACUAUUGGUGAAACAAA................AUG
env-596   ....................AAAAACA.......GUAGCU.GAACCGUGAAACUAUAAACUAUUGGUGAAACAAA................AUG
env-597   ....................AAAAACA.......GUAGCU.GAACCGUGAAACUAUAAACUAUUGGUGAAACAAA................AUG
env-598   ....................AAAAACA.......GUAGCU.GAACCGUGAAACU.......................................
env-599   ....................AAAAACA.......GUAGCU.GAACCGUGAAACUAUAAACUAUUGGUGAAACAAA................AUG
env-602   ....................AAAAACA.......GUAGCU.GAACCGUGAA..........................................
env-603   ....................GAAAAAAAAG....GCGGCU.GAACCGUGAAACUCUCUUAUUAAAGGUGAAACAAA...............AUG
env-604   ....................GGAAAAAAAG....GCGGCU.GAACCGUGAAACUCUCUUAUUAAAGGUGAAACAAA...............AUG
env-605   ....................GGAAAAAAAG....GCGGCU.GAACCGUGAAACUCUCUUAUUAAAGGUGAAACAAA...............AUG
env-606   ....................AGAAAAAAAG....GCGGCU.GAACCGUGAAAC........................................
env-607   ....................AGAAAAAAAG....GCGGCU.GAACCGUGAAA.........................................
env-608   ....................AGAAAAAAAG....GCGGCU.GAACCGUGAAACUCUCUU..................................
env-609   ....................AGAAAAAAAG....GCGGCU.GAACCGUGAAAC........................................

          >>>>>>>>>>>>>>>>>>>>>..........>>>>>>>>>>>>>>>>>>>>>>>>>>>>>>>>>>>>>>>>>>>>>>>>>>....>>..........
          001.111....11.1..1101...........00000..000.000...0000000.000000000..1.1.................22222....21..........
          --------------------●●●○----------------------------○RRYU-GAACCR●RRR●●●●●●●●R●●●●●●●○○-------------------AUG
                                                                          3′
```

```
env-610   .....................GAUAAAAG................................................................GCGGCU.GAACCGUGAAACUCUAUUAUUAUAGGUGAAACAAA..............AUG
env-611   .....................AGAAAAAAA..............................................................GGCGGCU.GAACCGUGAAACUCUCUUACUAAAGGUGAAACAAA..............AUG
env-612   .....................AGAAAAAAA..............................................................GGCGGCU.GAACCGUGAAACUCUCUUAUUUAAAGGUGAAACAAA.............AUG
env-613   .....................AGAAAAAAA..............................................................GGCGGCU.GAACCGUGAAACUCUCUUAUUUAAAGGUGAAACAAA.............AUG
env-616   .....................ACA......................................................................UAGCU.GAACCGUGAAACCAUGAUGGAGAAUCGAAAC..................AUG
Mco-1-1   .....................ACA......................................................................UAGCU.GAACCGUGAAACCAUGAUGGAGAAUCGAAAC..................AUG
env-617   .....................AAAGA..................................................................AAAGAUU.GAACCAUGAAACUAAAAAUGGAACAGGUGAAUAAUU.............UUG
Mps-1-1   .....................UUG......................................................................AAGCU.GAACCGUGAAACAAAGAAAUGAAACAAGGUGAACGUAU...........UUG
env-619   .....................UUG......................................................................AAGCU.GAACCGUGAAACACAGAAAUGAAACAAGGUGAAUAUAU...........UUG
env-620   .....................UUG......................................................................AAGCU.GAACCGUGAAACACAGAAAUGAAACAAGGUGA.................AUG
Mti-1-1   .....................UAAUAAUUAGAAGC.......................................................CAAAAGCUG..AACCGUGAAACUAGAAAUGAAACUUAG.....................GUG
env-621   .....................UGAUAU..................................................................AAGCUG..AACCGUGAAACAAGAAGAUGAAACAAG.....................GUG
Mba-2-1   .....................CAGG..................................................................CAGGGCUG..AACCGUGAAACAUAAGUGAAACUUG.......................GUG
env-622   .....................CAGA...................................................................CAGGGUU.GAACCGUGAAACAUAAAUGAAACUUGGUGUUUUAA..............UUG
env-623   .....................CAGG...................................................................CAGGGCU.GAACCGUGAAACAAAGUGAAACUUG........................GUG
env-624   .....................CAGG...................................................................CAGGACU.GAACCGUGAAACGUAAGUGAAACUUG.......................GUG
env-625   .....................AGG...................................................................GCAGGGCU.GAACCAUGAAACAAAAGUGAAACUUG.......................GUG
Mho-2-1   .....................CUGG...................................................................CAGGGCU.GAACCGUGAAACAUAAGUGAAACUUG.......................GUG
env-628   .....................CUGG...................................................................CAGGGCU.GAACCGUGAAACAUAAGUGAAACCUG.......................GUG
env-629   .....................CAAG...................................................................CAGGGCU.GAACCGUGAAACAUAAGUGAAACUUG.......................GUG
env-630   .....................AGG...................................................................GCAGGGCU.GAACCGUGAAACAAAAGUGAAACUUG.......................GUG
Mth-1-1   .....................AGG...................................................................GCAGGGCU.GAACCGUGAAACAAAAGUGAAACUUG.......................GUG
Msp-13-1  .....................CAGG...................................................................CAGGGCU.GAACCGUGAAACAAAAGUGAAACCUG.......................GUG
Mva-1-1   .....................CAGG...................................................................CAGGGCU.GAACCGUGAAACAAAAGUGAAACCUG.......................GUG
Mba-4-1   .....................CUGG...................................................................CAGGGCU.GAACCGUGAAACAAAAGUGAAACCUG.......................GUG
env-631   .....................CAGG...................................................................CAGGGCU.GAACCGUGAAACAAAAGUGAAACCUG.......................GUG
env-633   .....................CAGG...................................................................CAGGGCU.GAACCGUGAAACAUAAGUGAAACUUG.......................GUG
Mla-1-1   .....................CAGG...................................................................CAGGGCU.GAACCAUGAAACAUAAGUGAAACUUG.......................GUG
Mba-5-1   .....................CAGG...................................................................CAGGGCU.GAACCGUGAAACAAAAGUGAAACUUG.......................GUG
Msp-7-1   .....................CAGG...................................................................CAGGGCU.GAACCAUGAAACAUAAGUGAAACUUG.......................GUG
Msp-16-1  .....................CAGG...................................................................CAGGGCU.GAACCGUGAAACAUAAGUGAAACUUG.......................GUG
env-634   .....................AAG...................................................................GCAGGGCU.GAACCGUGAAACAAAAGUGAAACUUG.......................GUG
Mso-1-1   .....................CUGG...................................................................CAGGGCUG.AACCGUGAAACAUAAGUGAAACUUG.......................GUG
Mma-3-1   .....................CUGG...................................................................CAGGGCUG.AACCGUGAAACAUAAGUGAAACUUG.......................GUG
Mac-1-1   .....................CAGG...................................................................CAGGGCUG.AACCGUGAAACAAAAGUGAAACUUG.......................GUG
Msi-2-1   .....................CAGG...................................................................CAGGGCUG.AACCGUGAAACAAAAGUGAAACUUG.......................GUG
env-635   .....................CAGG...................................................................CAGGGCU.GAACCGUGAAGCAAAAAGUGAAACUUG......................GUG
Msi-1-1   .....................CAGG....................................................................CAGGGUUGAACCGUGAAACAAAAGUGAAACUUGGUGUUUAAA..............UUG
env-636   .....................CAGG....................................................................CAGGGUUGAACCGUGAAACAAAAGUGAAACUUGGUGUUUAAA..............UUG
Msp-14-1  .....................AGACC...................................................................CGGGCU.GAACCGUGAAACAAGAAGUGAAACUUGGUGUUAUAAAA...........UUG
env-637   .....................GACA.....................................................................GGACU.GAACCGUGAAACUGAAAAUGAAACAG.......................GUG
env-638   .....................GACA.....................................................................GGACU.GAACCGUGAAACUGAAAAUGAAACAG.......................GUG
env-639   .....................GAUA....................................................................GGACUG..AACCGUGAAACUUAAAAUGAAACAG.......................GUG
env-640   .....................GGAAAAA..................................................................GGCUG..AACCGUGAAACUAAAAAUGAAACAAG......................GUG
env-641   .....................AAA......................................................................UGGGUUGAACCGUGAAACUGAAAAUGAAACAAGGUGAACAUA.............UUG
Mbu-1-1   .....................AAC....................................................................UUCAGCUG.AACCGUGAAACUAACAUGAAACAAGGUGAAUAAA..............UUG
env-642   .....................AAC....................................................................UUCAGCU.GAACCGUGAAACUAACAUGAAACAAG.......................GUG
Mme-2-1   .....................UAA...................................................................CUUUGGCU.GAACCGUGAAACUAACAUGAAACUAAGGUGAAAAAU.............UUG
Mme-1-1   .....................UAA...................................................................CUUUGGCU.GAACCGUGAAACUAACGUGAAACAAGGUGAAUAAA..............UUG
Msh-1-1   .....................ACU......................................................................GAGCU.GAACCAUAAAGCAGAGAUGGAGGCACUGU....................AUG
env-643   .....................UUAA......................................................................AGCU.GAACGAGAGGUUAUAAAAUG.............................AUG
env-644   .....................GUAAA......................................................................GCU.GAACGAGAGGUUAUUAAAAUG............................AUG
env-645   .....................GAAA.......................................................................GCU.GAACGAAAAGAGGUGUUUUAA............................UUG
env-646   .....................GAAG.......................................................................GCU.GAACAAAAAGAGGUGUUUUAA............................UUG
env-647   .....................GAGAA.....................................................................GGCU.GAACAAAAAGGUGUUUAUU..............................AUG
env-650   .....................UUA........................................................................AGCU.GAACAAGAGGUGUUAAU................................UUG
env-651   .....................GUAAA......................................................................GCU.GAACAGAUAGAGGUGUAAUUA............................AUG
env-656   .....................CUAGU.....................................................................GGCU.GAACAAAAAGGUGUUAUUU..............................AUG
env-657   .....................CUCGU.....................................................................GGCU.GAACAAAAAGGUGUUAUUU..............................AUG
env-658   .....................CUCGU.....................................................................GGCU.GAACAAAAGGUGUUAUU................................AUG
Mve-1-1   .....................GCAA.......................................................................GCU.GAACAAAAGAGGUGUUUUA..............................AUG
env-659   .....................GCAA.......................................................................GCU.GAACAAAAGAGGUGUUUUA..............................AUG
env-660   .....................GCAA.......................................................................GCU.GAACAAAAGAGGUGUUUUA..............................AUG
Msp-4-1   .....................GAAA.......................................................................GCU.GAACAAAAGAGGUGUUUUA..............................AUG
env-661   .....................GAAA.......................................................................GCU.GAACAAAAGAGGUGUAUAUUAA...........................AUG
env-663   .....................GAAA.......................................................................GCU.GAACAAAAGAGGUG......................................
env-664   .....................GAAA.......................................................................GCU.GAACAAAAGAGGUGUAUAUUAA...........................AUG
env-665   .....................GAAA.......................................................................GCU.GAACAAAAAGAGG.......................................
env-670   .....................GAAA.......................................................................GCU.GAACAAAAAGAGGUGUAUUA.............................AUG
env-671   .....................GAAA.......................................................................GCU.GAACAAAAAGAGGUGUAUUUA............................AUG
env-672   .....................GAAA.......................................................................GCU.GAACAAAAAGAGGUGUAUUUA............................AUG
Mfo-2-1   .....................GAAA.......................................................................GCU.GAACAAAAAGAGGUGUAUUUA............................AUG
env-673   .....................GAAA.......................................................................GCU.GAACAAAAAGAGGUGUAUUUA............................AUG
env-674   .....................GAAA.......................................................................GCU.GAACAAAAAGAGGUGUAUAUUAA..........................AUG
Msp-2-1   .....................GAAA.......................................................................GCU.GAACAAAAAGAGGUGUAUAUUAA..........................AUG
Mfo-1-1   .....................GAAA.......................................................................GCU.GAACAAAAAGAGGUGUAUAUUAA..........................AUG
env-675   .....................GCAA.......................................................................GCU.GAACAAAGAGGUGUUUUUA..............................AUG
env-676   .....................GAAA.......................................................................GCU.GAACAAAGAGGUGUUUAA...............................AUG
Msp-1-1   .....................GCAA.......................................................................GCU.GAACAAAGAGGUGUAAUUUA.............................AUG
env-679   .....................GCAA.......................................................................GCU.GAACAAAGAGGUGUAAUUUA.............................AUG
env-680   .....................GCAA.......................................................................GCU.GAACAAAGAGGUGUUAAUUA.............................AUG
Msp-3-1   .....................GCAA.......................................................................GCU.GAACAAAGAGGUGUUAAGUA.............................AUG
env-681   .....................GCAA.......................................................................GCU.GAACAAAGAGGUGUUAAUUA.............................AUG

          >>>>>>>>>>>>>>>>>>>>>...........>>>>>>>>>>>>>>>>>>>>>>>>>>>>>>>>>>>>>>>>>>>>>>>>>>>>>>>>>>>>>>>>>>....>>...............................................
          001.111....11.1..1101...............00000...000.000...0000000.000000000..1.1..................22222....21...............................................
          ---------------------●●●○---------------------------------------------------------------------○RRYU-GAACCR●RRR●●●●●●●R●●●●●●●○○----------------------AUG
                                                                                                                 3′
```

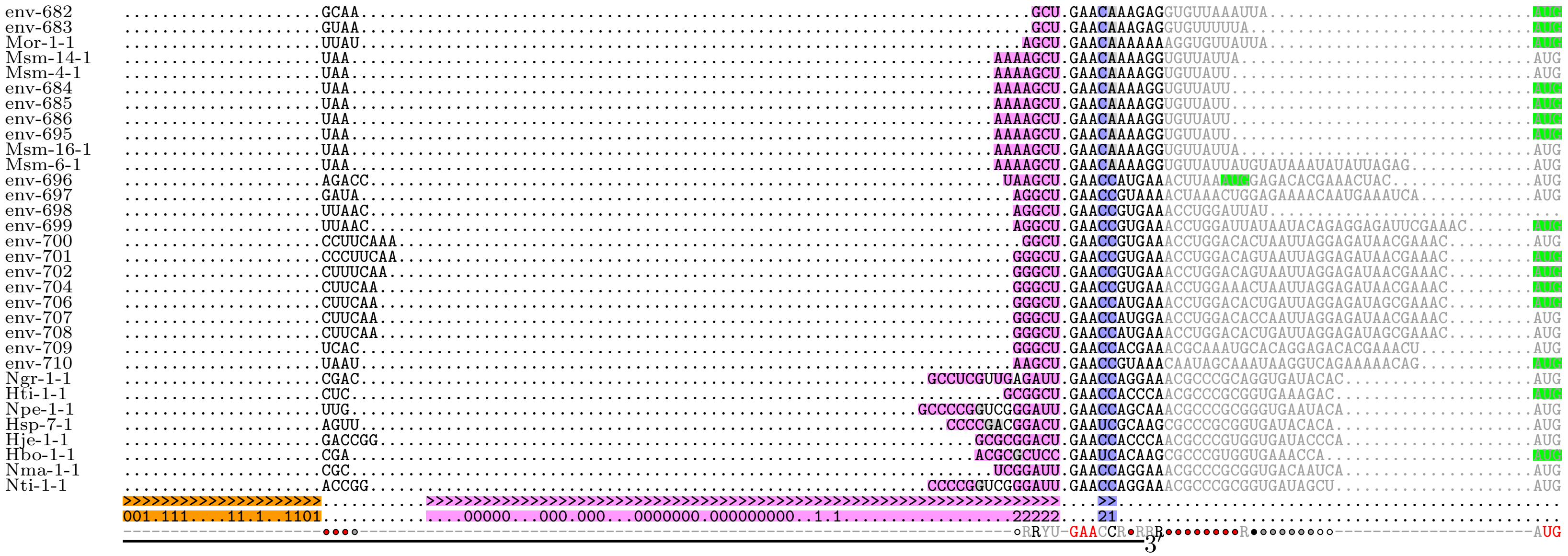

5 L17-Actino-Proteobacteria

5.1 Taxa

The taxonomy of each organism containing a putative L17-Actino-Proteobacteria RNA is listed, with abbreviations identifying each hit (e.g., “Eco-1-1” and “Eco-1-2” might hypothetically represent two distinct RNAs in *E. coli*). The abbreviations will be used to identify each individual L17-Actino-Proteobacteria RNA in Sections 5.3 and 5.5:.

abbrev. of hits	taxonomy of species
Aal-1-1	Bacteria Actinobacteria Actinobacteridae Actinomycetales Propionibacterineae Nocardioidaceae *Actinopolymorpha alba* DSM 45243
Tbi-1-1	Bacteria Actinobacteria Actinobacteridae Actinomycetales Pseudonocardineae Pseudonocardiaceae *Thermobispora bispora* DSM 43833
Sac-1-1	Bacteria Actinobacteria Actinobacteridae Actinomycetales Streptomycineae Streptomycetaceae *Streptomyces acidiscabies* 84-104
Saf-1-1	Bacteria Actinobacteria Actinobacteridae Actinomycetales Streptomycineae Streptomycetaceae *Streptomyces afghaniensis* 772
Sal-1-1	Bacteria Actinobacteria Actinobacteridae Actinomycetales Streptomycineae Streptomycetaceae *Streptomyces albulus* CCRC 11814
Sal-2-1	Bacteria Actinobacteria Actinobacteridae Actinomycetales Streptomycineae Streptomycetaceae *Streptomyces albus* J1074
Sau-1-1	Bacteria Actinobacteria Actinobacteridae Actinomycetales Streptomycineae Streptomycetaceae *Streptomyces aurantiacus* JA 4570
Sau-2-1	Bacteria Actinobacteria Actinobacteridae Actinomycetales Streptomycineae Streptomycetaceae *Streptomyces auratus* AGR0001
Sav-1-1	Bacteria Actinobacteria Actinobacteridae Actinomycetales Streptomycineae Streptomycetaceae *Streptomyces avermitilis* MA-4680
Sbi-1-1	Bacteria Actinobacteria Actinobacteridae Actinomycetales Streptomycineae Streptomycetaceae *Streptomyces bingchenggensis* BCW-1
Sbo-1-1 to Sbo-1-2	Bacteria Actinobacteria Actinobacteridae Actinomycetales Streptomycineae Streptomycetaceae *Streptomyces bottropensis* ATCC 25435
Sca-1-1	Bacteria Actinobacteria Actinobacteridae Actinomycetales Streptomycineae Streptomycetaceae *Streptomyces canus* 299MFChir4.1
Sca-2-1 to Sca-2-2	Bacteria Actinobacteria Actinobacteridae Actinomycetales Streptomycineae Streptomycetaceae *Streptomyces cattleya* NRRL 8057 = DSM 46488
Sch-1-1	Bacteria Actinobacteria Actinobacteridae Actinomycetales Streptomycineae Streptomycetaceae *Streptomyces chartreusis* NRRL 12338
Scl-1-1 to Scl-1-2	Bacteria Actinobacteria Actinobacteridae Actinomycetales Streptomycineae Streptomycetaceae *Streptomyces clavuligerus* ATCC 27064
Sco-1-1	Bacteria Actinobacteria Actinobacteridae Actinomycetales Streptomycineae Streptomycetaceae *Streptomyces coelicoflavus* ZG0656
Sco-2-1	Bacteria Actinobacteria Actinobacteridae Actinomycetales Streptomycineae Streptomycetaceae *Streptomyces coelicolor* A3(2)
Sco-3-1	Bacteria Actinobacteria Actinobacteridae Actinomycetales Streptomycineae Streptomycetaceae *Streptomyces collinus* Tu 365
Sda-1-1	Bacteria Actinobacteria Actinobacteridae Actinomycetales Streptomycineae Streptomycetaceae *Streptomyces davawensis* JCM 4913
Sfl-1-1	Bacteria Actinobacteria Actinobacteridae Actinomycetales Streptomycineae Streptomycetaceae *Streptomyces flavogriseus* ATCC 33331
Sfu-1-1	Bacteria Actinobacteria Actinobacteridae Actinomycetales Streptomycineae Streptomycetaceae *Streptomyces fulvissimus* DSM 40593

Sga-1-1	Bacteria Actinobacteria Actinobacteridae Actinomycetales Streptomycineae Streptomycetaceae *Streptomyces gancidicus* BKS 13-15
Sgh-1-1	Bacteria Actinobacteria Actinobacteridae Actinomycetales Streptomycineae Streptomycetaceae *Streptomyces ghanaensis* ATCC 14672
Sgl-1-1	Bacteria Actinobacteria Actinobacteridae Actinomycetales Streptomycineae Streptomycetaceae *Streptomyces globisporus* C-1027
Sgr-1-1	Bacteria Actinobacteria Actinobacteridae Actinomycetales Streptomycineae Streptomycetaceae *Streptomyces griseoaurantiacus* M045
Sgr-2-1	Bacteria Actinobacteria Actinobacteridae Actinomycetales Streptomycineae Streptomycetaceae *Streptomyces griseoflavus* Tu4000
Sgr-4-1	Bacteria Actinobacteria Actinobacteridae Actinomycetales Streptomycineae Streptomycetaceae *Streptomyces griseus subsp. griseus* NBRC 13350
Sgr-3-1	Bacteria Actinobacteria Actinobacteridae Actinomycetales Streptomycineae Streptomycetaceae *Streptomyces griseus* XylebKG-1
Shy-1-1	Bacteria Actinobacteria Actinobacteridae Actinomycetales Streptomycineae Streptomycetaceae *Streptomyces hygroscopicus* ATCC 53653
Shy-2-1	Bacteria Actinobacteria Actinobacteridae Actinomycetales Streptomycineae Streptomycetaceae *Streptomyces hygroscopicus subsp. jinggangensis* 5008
Shy-3-1	Bacteria Actinobacteria Actinobacteridae Actinomycetales Streptomycineae Streptomycetaceae *Streptomyces hygroscopicus subsp. jinggangensis* TL01
Sip-1-1	Bacteria Actinobacteria Actinobacteridae Actinomycetales Streptomycineae Streptomycetaceae *Streptomyces ipomoeae* 91-03
Sli-1-1	Bacteria Actinobacteria Actinobacteridae Actinomycetales Streptomycineae Streptomycetaceae *Streptomyces lividans* 1326
Sli-2-1 to Sli-2-2	Bacteria Actinobacteria Actinobacteridae Actinomycetales Streptomycineae Streptomycetaceae *Streptomyces lividans* TK24
Smo-1-1	Bacteria Actinobacteria Actinobacteridae Actinomycetales Streptomycineae Streptomycetaceae *Streptomyces mobaraensis* NBRC 13819 = DSM 40847
Sni-1-1	Bacteria Actinobacteria Actinobacteridae Actinomycetales Streptomycineae Streptomycetaceae *Streptomyces niveus* NCIMB 11891
Spr-1-1	Bacteria Actinobacteria Actinobacteridae Actinomycetales Streptomycineae Streptomycetaceae *Streptomyces pristinaespiralis* ATCC 25486
Spr-2-1	Bacteria Actinobacteria Actinobacteridae Actinomycetales Streptomycineae Streptomycetaceae *Streptomyces prunicolor* NBRC 13075
Spu-1-1	Bacteria Actinobacteria Actinobacteridae Actinomycetales Streptomycineae Streptomycetaceae *Streptomyces purpureus* KA281
Sri-1-1	Bacteria Actinobacteria Actinobacteridae Actinomycetales Streptomycineae Streptomycetaceae *Streptomyces rimosus subsp. rimosus* ATCC 10970
Sro-1-1	Bacteria Actinobacteria Actinobacteridae Actinomycetales Streptomycineae Streptomycetaceae *Streptomyces roseochromogenes subsp. oscitans* DS 12.976
Sro-2-1	Bacteria Actinobacteria Actinobacteridae Actinomycetales Streptomycineae Streptomycetaceae *Streptomyces roseosporus* NRRL 15998
Ssc-1-1	Bacteria Actinobacteria Actinobacteridae Actinomycetales Streptomycineae Streptomycetaceae *Streptomyces scabiei* 87.22
Ssc-2-1	Bacteria Actinobacteria Actinobacteridae Actinomycetales Streptomycineae Streptomycetaceae *Streptomyces scabrisporus* DSM 41855
Sso-1-1	Bacteria Actinobacteria Actinobacteridae Actinomycetales Streptomycineae Streptomycetaceae *Streptomyces somaliensis* DSM 40738
Ssp-1-1	Bacteria Actinobacteria Actinobacteridae Actinomycetales Streptomycineae Streptomycetaceae *Streptomyces sp.* 303MFCol5.2
Ssp-2-1	Bacteria Actinobacteria Actinobacteridae Actinomycetales Streptomycineae Streptomycetaceae *Streptomyces sp.* 351MFTsu5.1
Ssp-3-1	Bacteria Actinobacteria Actinobacteridae Actinomycetales Streptomycineae Streptomycetaceae *Streptomyces sp.* AA0539
Ssp-4-1	Bacteria Actinobacteria Actinobacteridae Actinomycetales Streptomycineae Streptomycetaceae *Streptomyces sp.* AA1529
Ssp-6-1	Bacteria Actinobacteria Actinobacteridae Actinomycetales Streptomycineae Streptomycetaceae *Streptomyces sp.* Amel2xE9
Ssp-5-1	Bacteria Actinobacteria Actinobacteridae Actinomycetales Streptomycineae Streptomycetaceae *Streptomyces sp.* ATexAB-D23
Ssp-7-1	Bacteria Actinobacteria Actinobacteridae Actinomycetales Streptomycineae Streptomycetaceae *Streptomyces sp.* BoleA5
Ssp-8-1	Bacteria Actinobacteria Actinobacteridae Actinomycetales Streptomycineae Streptomycetaceae *Streptomyces sp.* C
Ssp-17-1	Bacteria Actinobacteria Actinobacteridae Actinomycetales Streptomycineae Streptomycetaceae *Streptomyces sp.* CcalMP-8W
Ssp-9-1	Bacteria Actinobacteria Actinobacteridae Actinomycetales Streptomycineae Streptomycetaceae *Streptomyces sp.* CNB091
Ssp-10-1	Bacteria Actinobacteria Actinobacteridae Actinomycetales Streptomycineae Streptomycetaceae *Streptomyces sp.* CNQ766
Ssp-11-1	Bacteria Actinobacteria Actinobacteridae Actinomycetales Streptomycineae Streptomycetaceae *Streptomyces sp.* CNS335
Ssp-12-1	Bacteria Actinobacteria Actinobacteridae Actinomycetales Streptomycineae Streptomycetaceae *Streptomyces sp.* CNS615
Ssp-13-1	Bacteria Actinobacteria Actinobacteridae Actinomycetales Streptomycineae Streptomycetaceae *Streptomyces sp.* CNT302
Ssp-14-1	Bacteria Actinobacteria Actinobacteridae Actinomycetales Streptomycineae Streptomycetaceae *Streptomyces sp.* CNT372
Ssp-15-1	Bacteria Actinobacteria Actinobacteridae Actinomycetales Streptomycineae Streptomycetaceae *Streptomyces sp.* CNY228
Ssp-16-1	Bacteria Actinobacteria Actinobacteridae Actinomycetales Streptomycineae Streptomycetaceae *Streptomyces sp.* CNY243
Ssp-18-1	Bacteria Actinobacteria Actinobacteridae Actinomycetales Streptomycineae Streptomycetaceae *Streptomyces sp.* DvalAA-83
Ssp-49-1	Bacteria Actinobacteria Actinobacteridae Actinomycetales Streptomycineae Streptomycetaceae *Streptomyces sp. e14*
Ssp-19-1	Bacteria Actinobacteria Actinobacteridae Actinomycetales Streptomycineae Streptomycetaceae *Streptomyces sp.* FxanaC1
Ssp-20-1	Bacteria Actinobacteria Actinobacteridae Actinomycetales Streptomycineae Streptomycetaceae *Streptomyces sp.* FxanaD5
Ssp-21-1	Bacteria Actinobacteria Actinobacteridae Actinomycetales Streptomycineae Streptomycetaceae *Streptomyces sp.* GBA 94-10
Ssp-22-1	Bacteria Actinobacteria Actinobacteridae Actinomycetales Streptomycineae Streptomycetaceae *Streptomyces sp.* HCCB10043
Ssp-23-1	Bacteria Actinobacteria Actinobacteridae Actinomycetales Streptomycineae Streptomycetaceae *Streptomyces sp.* HGB0020
Ssp-25-1	Bacteria Actinobacteria Actinobacteridae Actinomycetales Streptomycineae Streptomycetaceae *Streptomyces sp.* HmicA12
Ssp-24-1	Bacteria Actinobacteria Actinobacteridae Actinomycetales Streptomycineae Streptomycetaceae *Streptomyces sp.* HPH0547
Ssp-26-1	Bacteria Actinobacteria Actinobacteridae Actinomycetales Streptomycineae Streptomycetaceae *Streptomyces sp.* KhCrAH-244
Ssp-27-1	Bacteria Actinobacteria Actinobacteridae Actinomycetales Streptomycineae Streptomycetaceae *Streptomyces sp.* KhCrAH-340
Ssp-28-1	Bacteria Actinobacteria Actinobacteridae Actinomycetales Streptomycineae Streptomycetaceae *Streptomyces sp.* LaPpAH-108
Ssp-29-1	Bacteria Actinobacteria Actinobacteridae Actinomycetales Streptomycineae Streptomycetaceae *Streptomyces sp.* LaPpAH-165
Ssp-30-1	Bacteria Actinobacteria Actinobacteridae Actinomycetales Streptomycineae Streptomycetaceae *Streptomyces sp.* LaPpAH-202
Ssp-31-1	Bacteria Actinobacteria Actinobacteridae Actinomycetales Streptomycineae Streptomycetaceae *Streptomyces sp.* LaPpAH-95
Ssp-32-1 to Ssp-32-2	Bacteria Actinobacteria Actinobacteridae Actinomycetales Streptomycineae Streptomycetaceae *Streptomyces sp.* Mg1
Ssp-33-1	Bacteria Actinobacteria Actinobacteridae Actinomycetales Streptomycineae Streptomycetaceae *Streptomyces sp.* MspMP-M5
Ssp-34-1	Bacteria Actinobacteria Actinobacteridae Actinomycetales Streptomycineae Streptomycetaceae *Streptomyces sp.* PAMC26508
Ssp-36-1	Bacteria Actinobacteria Actinobacteridae Actinomycetales Streptomycineae Streptomycetaceae *Streptomyces sp.* PsTaAH-124
Ssp-35-1	Bacteria Actinobacteria Actinobacteridae Actinomycetales Streptomycineae Streptomycetaceae *Streptomyces sp.* PVA 94-07
Ssp-37-1	Bacteria Actinobacteria Actinobacteridae Actinomycetales Streptomycineae Streptomycetaceae *Streptomyces sp.* R1-NS-10
Ssp-38-1	Bacteria Actinobacteria Actinobacteridae Actinomycetales Streptomycineae Streptomycetaceae *Streptomyces sp.* S4
Ssp-39-1	Bacteria Actinobacteria Actinobacteridae Actinomycetales Streptomycineae Streptomycetaceae *Streptomyces sp.* SA3_actF
Ssp-40-1	Bacteria Actinobacteria Actinobacteridae Actinomycetales Streptomycineae Streptomycetaceae *Streptomyces sp.* SA3_actG

Ssp-44-1	Bacteria Actinobacteria Actinobacteridae Actinomycetales Streptomycineae Streptomycetaceae *Streptomyces sp.* ScaeMP-e10
Ssp-45-1	Bacteria Actinobacteria Actinobacteridae Actinomycetales Streptomycineae Streptomycetaceae *Streptomyces sp.* SirexAA-E
Ssp-41-1	Bacteria Actinobacteria Actinobacteridae Actinomycetales Streptomycineae Streptomycetaceae *Streptomyces sp.* SPB74
Ssp-42-1	Bacteria Actinobacteria Actinobacteridae Actinomycetales Streptomycineae Streptomycetaceae *Streptomyces sp.* SPB78
Ssp-43-1	Bacteria Actinobacteria Actinobacteridae Actinomycetales Streptomycineae Streptomycetaceae *Streptomyces sp.* SS
Ssp-46-1	Bacteria Actinobacteria Actinobacteridae Actinomycetales Streptomycineae Streptomycetaceae *Streptomyces sp.* TOR3209
Ssp-47-1	Bacteria Actinobacteria Actinobacteridae Actinomycetales Streptomycineae Streptomycetaceae *Streptomyces sp.* Tu6071
Ssp-48-1	Bacteria Actinobacteria Actinobacteridae Actinomycetales Streptomycineae Streptomycetaceae *Streptomyces sp.* W007
Ssu-1-1	Bacteria Actinobacteria Actinobacteridae Actinomycetales Streptomycineae Streptomycetaceae *Streptomyces sulphureus* DSM 40104
Ssu-2-1	Bacteria Actinobacteria Actinobacteridae Actinomycetales Streptomycineae Streptomycetaceae *Streptomyces sulphureus* L180
Ssv-1-1	Bacteria Actinobacteria Actinobacteridae Actinomycetales Streptomycineae Streptomycetaceae *Streptomyces sviceus* ATCC 29083
Sts-1-1	Bacteria Actinobacteria Actinobacteridae Actinomycetales Streptomycineae Streptomycetaceae *Streptomyces tsukubaensis* NRRL18488
Stu-1-1	Bacteria Actinobacteria Actinobacteridae Actinomycetales Streptomycineae Streptomycetaceae *Streptomyces turgidiscabies* Car8
Sve-1-1	Bacteria Actinobacteria Actinobacteridae Actinomycetales Streptomycineae Streptomycetaceae *Streptomyces venezuelae* ATCC 10712
Svi-1-1	Bacteria Actinobacteria Actinobacteridae Actinomycetales Streptomycineae Streptomycetaceae *Streptomyces violaceusniger* SPC6
Svi-2-1	Bacteria Actinobacteria Actinobacteridae Actinomycetales Streptomycineae Streptomycetaceae *Streptomyces violaceusniger* Tu 4113
Svi-3-1	Bacteria Actinobacteria Actinobacteridae Actinomycetales Streptomycineae Streptomycetaceae *Streptomyces viridochromogenes* DSM 40736
Svi-4-1	Bacteria Actinobacteria Actinobacteridae Actinomycetales Streptomycineae Streptomycetaceae *Streptomyces viridochromogenes* Tue57
Svi-5-1	Bacteria Actinobacteria Actinobacteridae Actinomycetales Streptomycineae Streptomycetaceae *Streptomyces viridosporus* T7A
Svi-6-1	Bacteria Actinobacteria Actinobacteridae Actinomycetales Streptomycineae Streptomycetaceae *Streptomyces vitaminophilus* DSM 41686
Szi-1-1	Bacteria Actinobacteria Actinobacteridae Actinomycetales Streptomycineae Streptomycetaceae *Streptomyces zinciresistens* K42
Sba-1-1	Bacteria Actinobacteria Actinobacteridae Actinomycetales Streptomycineae *Streptomycetaceae bacterium* MP113-05
Nco-1-1	Bacteria Actinobacteria Actinobacteridae Actinomycetales Streptosporangineae Streptosporangiaceae *Nonomuraea coxensis* DSM 45129
Sro-3-1	Bacteria Actinobacteria Actinobacteridae Actinomycetales Streptosporangineae Streptosporangiaceae *Streptosporangium roseum* DSM 43021
Aat-1-1	Bacteria Actinobacteria Actinobacteridae Actinomycetales Streptosporangineae Thermomonosporaceae *Actinomadura atramentaria* DSM 43919
Afl-1-1	Bacteria Actinobacteria Actinobacteridae Actinomycetales Streptosporangineae Thermomonosporaceae *Actinomadura flavalba* DSM 45200
Aac-1-1	Bacteria Actinobacteria Catenulisporales Actinospicaceae *Actinospica acidiphila*
Nal-1-1	Bacteria Actinobacteria Propionibacteriales Nocardioidaceae *Nocardioides alkalitolerans* DSM 16699
Nha-1-1	Bacteria Actinobacteria Propionibacteriales Nocardioidaceae *Nocardioides halotolerans* DSM 19273
Nin-1-1	Bacteria Actinobacteria Propionibacteriales Nocardioidaceae *Nocardioides insulae* DSM 17944
Nlu-1-1	Bacteria Actinobacteria Propionibacteriales Nocardioidaceae *Nocardioides luteus*
Nsp-1-1	Bacteria Actinobacteria Propionibacteriales Nocardioidaceae *Nocardioides sp.* J54
Nsp-2-1	Bacteria Actinobacteria Propionibacteriales Nocardioidaceae *Nocardioides sp.* URHA0020
Nsp-3-1	Bacteria Actinobacteria Propionibacteriales Nocardioidaceae *Nocardioides sp.* URHA0032
Psi-1-1	Bacteria Actinobacteria Propionibacteriales Nocardioidaceae *Pimelobacter simplex*
Ssp-50-1	Bacteria Actinobacteria Pseudonocardiales Pseudonocardiaceae *Saccharothrix sp.* ST-888
Kau-1-1 to Kau-1-2	Bacteria Actinobacteria Streptomycetales Streptomycetaceae *Kitasatospora aureofaciens*
Kaz-1-1	Bacteria Actinobacteria Streptomycetales Streptomycetaceae *Kitasatospora azatica* KCTC 9699
Kme-1-1	Bacteria Actinobacteria Streptomycetales Streptomycetaceae *Kitasatospora mediocidica* KCTC 9733
Kph-1-1 to Kph-1-2	Bacteria Actinobacteria Streptomycetales Streptomycetaceae *Kitasatospora phosalacinea*
Kpu-1-1	Bacteria Actinobacteria Streptomycetales Streptomycetaceae *Kitasatospora purpeofusca*
Ksp-1-1	Bacteria Actinobacteria Streptomycetales Streptomycetaceae *Kitasatospora sp.* MBT63
Ksp-2-1	Bacteria Actinobacteria Streptomycetales Streptomycetaceae *Kitasatospora sp.* MBT66
Ksp-3-1	Bacteria Actinobacteria Streptomycetales Streptomycetaceae *Kitasatospora sp.* MY 5-36
Ksp-4-1	Bacteria Actinobacteria Streptomycetales Streptomycetaceae *Kitasatospora sp.* NRRL B-11411
Sal-3-1	Bacteria Actinobacteria Streptomycetales Streptomycetaceae *Streptacidiphilus albus*
Sal-4-1	Bacteria Actinobacteria Streptomycetales Streptomycetaceae *Streptacidiphilus albus* JL83
Sca-3-1	Bacteria Actinobacteria Streptomycetales Streptomycetaceae *Streptacidiphilus carbonis*
Sje-1-1	Bacteria Actinobacteria Streptomycetales Streptomycetaceae *Streptacidiphilus jeojiense*
Sor-1-1	Bacteria Actinobacteria Streptomycetales Streptomycetaceae *Streptacidiphilus oryzae* TH49
[pa-1-1	Bacteria Actinobacteria Streptomycetales Streptomycetaceae Streptomyces [Kitasatospora] papulosa
Asp-1-1	Bacteria Actinobacteria Streptomycetales Streptomycetaceae Streptomyces *Actinosporangium sp.* NRRL B-3428
Sac-2-1	Bacteria Actinobacteria Streptomycetales Streptomycetaceae *Streptomyces achromogenes subsp. achromogenes*
Sac-3-1	Bacteria Actinobacteria Streptomycetales Streptomycetaceae *Streptomyces acidiscabies*
Sah-1-1	Bacteria Actinobacteria Streptomycetales Streptomycetaceae *Streptomyces ahygroscopicus subsp. wuyiensis*
Sal-5-1	Bacteria Actinobacteria Streptomycetales Streptomycetaceae Streptomyces Streptomyces albidoflavus group *Streptomyces albidoflavus*
Sal-6-1	Bacteria Actinobacteria Streptomycetales Streptomycetaceae *Streptomyces alboflavus*
Sal-7-1	Bacteria Actinobacteria Streptomycetales Streptomycetaceae *Streptomyces alboviridis*
Sal-8-1	Bacteria Actinobacteria Streptomycetales Streptomycetaceae *Streptomyces albulus*
Sal-9-1	Bacteria Actinobacteria Streptomycetales Streptomycetaceae *Streptomyces albulus* PD-1
Sal-10-1	Bacteria Actinobacteria Streptomycetales Streptomycetaceae *Streptomyces albulus* ZPM
Sal-11-1	Bacteria Actinobacteria Streptomycetales Streptomycetaceae *Streptomyces albus*
Sal-12-1 to Sal-12-5	Bacteria Actinobacteria Streptomycetales Streptomycetaceae *Streptomyces albus subsp. albus*
Sam-1-1	Bacteria Actinobacteria Streptomycetales Streptomycetaceae *Streptomyces ambofaciens* ATCC 23877
San-1-1	Bacteria Actinobacteria Streptomycetales Streptomycetaceae *Streptomyces antibioticus*

San-2-1 to San-2-2	Bacteria Actinobacteria Streptomycetales Streptomycetaceae *Streptomyces anulatus*
Sat-1-1	Bacteria Actinobacteria Streptomycetales Streptomycetaceae *Streptomyces atratus*
Sat-2-1	Bacteria Actinobacteria Streptomycetales Streptomycetaceae *Streptomyces atroolivaceus*
Sau-3-1 to Sau-3-2	Bacteria Actinobacteria Streptomycetales Streptomycetaceae *Streptomyces aureocirculatus*
Sau-4-1	Bacteria Actinobacteria Streptomycetales Streptomycetaceae *Streptomyces aureus*
Sav-2-1 to Sav-2-2	Bacteria Actinobacteria Streptomycetales Streptomycetaceae *Streptomyces avermitilis* MA-4680 = NBRC 14893
Sav-3-1	Bacteria Actinobacteria Streptomycetales Streptomycetaceae *Streptomyces avicenniae*
Sba-2-1	Bacteria Actinobacteria Streptomycetales Streptomycetaceae *Streptomyces baarnensis*
Sbi-2-1 to Sbi-2-2	Bacteria Actinobacteria Streptomycetales Streptomycetaceae *Streptomyces bicolor*
Sbi-3-1 to Sbi-3-3	Bacteria Actinobacteria Streptomycetales Streptomycetaceae *Streptomyces bikiniensis*
Sbr-1-1	Bacteria Actinobacteria Streptomycetales Streptomycetaceae *Streptomyces brasiliensis*
Sca-4-1	Bacteria Actinobacteria Streptomycetales Streptomycetaceae *Streptomyces caatingaensis*
Sca-5-1 to Sca-5-2	Bacteria Actinobacteria Streptomycetales Streptomycetaceae *Streptomyces californicus*
Sca-6-1 to Sca-6-2	Bacteria Actinobacteria Streptomycetales Streptomycetaceae *Streptomyces capuensis*
Sca-7-1	Bacteria Actinobacteria Streptomycetales Streptomycetaceae *Streptomyces catenulae*
Sce-1-1	Bacteria Actinobacteria Streptomycetales Streptomycetaceae *Streptomyces celluloflavus*
Sce-2-1	Bacteria Actinobacteria Streptomycetales Streptomycetaceae *Streptomyces cellulosae*
Scy-1-1	Bacteria Actinobacteria Streptomycetales Streptomycetaceae *Streptomyces cyaneofuscatus*
Scy-2-1	Bacteria Actinobacteria Streptomycetales Streptomycetaceae *Streptomyces cyaneogriseus subsp. noncyanogenus*
Sde-1-1	Bacteria Actinobacteria Streptomycetales Streptomycetaceae *Streptomyces decoyicus*
Sdu-1-1	Bacteria Actinobacteria Streptomycetales Streptomycetaceae *Streptomyces durhamensis*
Ser-1-1	Bacteria Actinobacteria Streptomycetales Streptomycetaceae *Streptomyces erythrochromogenes*
Seu-1-1	Bacteria Actinobacteria Streptomycetales Streptomycetaceae *Streptomyces europaeiscabiei*
Sex-1-1	Bacteria Actinobacteria Streptomycetales Streptomycetaceae *Streptomyces exfoliatus*
Sex-2-1	Bacteria Actinobacteria Streptomycetales Streptomycetaceae *Streptomyces exfoliatus* DSM 41693
Sfl-2-1	Bacteria Actinobacteria Streptomycetales Streptomycetaceae *Streptomyces flaveolus*
Sfl-3-1	Bacteria Actinobacteria Streptomycetales Streptomycetaceae *Streptomyces flaveus*
Sfl-4-1	Bacteria Actinobacteria Streptomycetales Streptomycetaceae *Streptomyces flavidovirens* DSM 40150
Sfl-5-1	Bacteria Actinobacteria Streptomycetales Streptomycetaceae *Streptomyces flavochromogenes*
Sfl-6-1	Bacteria Actinobacteria Streptomycetales Streptomycetaceae *Streptomyces flavotricini*
Sfl-7-1	Bacteria Actinobacteria Streptomycetales Streptomycetaceae *Streptomyces flavovariabilis*
Sfl-8-1	Bacteria Actinobacteria Streptomycetales Streptomycetaceae *Streptomyces flavovirens*
Sfl-9-1	Bacteria Actinobacteria Streptomycetales Streptomycetaceae *Streptomyces floridae*
Sfr-1-1 to Sfr-1-2	Bacteria Actinobacteria Streptomycetales Streptomycetaceae *Streptomyces fradiae*
Sfu-2-1	Bacteria Actinobacteria Streptomycetales Streptomycetaceae *Streptomyces fulvoviolaceus*
Sfu-3-1	Bacteria Actinobacteria Streptomycetales Streptomycetaceae *Streptomyces fulvoviridis*
Sga-2-1	Bacteria Actinobacteria Streptomycetales Streptomycetaceae *Streptomyces galbus*
Sgl-2-1	Bacteria Actinobacteria Streptomycetales Streptomycetaceae *Streptomyces glaucescens*
Sgl-3-1 to Sgl-3-2	Bacteria Actinobacteria Streptomycetales Streptomycetaceae *Streptomyces globisporus subsp. globisporus*
Sgr-5-1	Bacteria Actinobacteria Streptomycetales Streptomycetaceae *Streptomyces graminis*
Sgr-6-1	Bacteria Actinobacteria Streptomycetales Streptomycetaceae *Streptomyces griseoflavus*
Sgr-7-1	Bacteria Actinobacteria Streptomycetales Streptomycetaceae *Streptomyces griseofuscus*
Sgr-8-1	Bacteria Actinobacteria Streptomycetales Streptomycetaceae *Streptomyces griseolus*
Sgr-9-1	Bacteria Actinobacteria Streptomycetales Streptomycetaceae *Streptomyces griseoluteus*
Sgr-10-1	Bacteria Actinobacteria Streptomycetales Streptomycetaceae *Streptomyces griseorubens*
Sgr-11-1	Bacteria Actinobacteria Streptomycetales Streptomycetaceae *Streptomyces griseus*
Sgr-12-1 to Sgr-12-9	Bacteria Actinobacteria Streptomycetales Streptomycetaceae *Streptomyces griseus subsp. griseus*
Sgr-13-1 to Sgr-13-5	Bacteria Actinobacteria Streptomycetales Streptomycetaceae *Streptomyces griseus subsp. rhodochrous*
Sha-1-1	Bacteria Actinobacteria Streptomycetales Streptomycetaceae *Streptomyces halstedii*
Shy-4-1	Bacteria Actinobacteria Streptomycetales Streptomycetaceae *Streptomyces hygroscopicus subsp. hygroscopicus*
Sia-1-1	Bacteria Actinobacteria Streptomycetales Streptomycetaceae *Streptomyces iakyrus*
Sir-1-1	Bacteria Actinobacteria Streptomycetales Streptomycetaceae *Streptomyces iranensis*
Ska-1-1 to Ska-1-2	Bacteria Actinobacteria Streptomycetales Streptomycetaceae *Streptomyces katrae*
Sla-1-1	Bacteria Actinobacteria Streptomycetales Streptomycetaceae *Streptomyces lavendulae*
Sla-2-1 to Sla-2-4	Bacteria Actinobacteria Streptomycetales Streptomycetaceae *Streptomyces lavendulae subsp. lavendulae*
Sla-3-1	Bacteria Actinobacteria Streptomycetales Streptomycetaceae *Streptomyces lavenduligriseus*
Sle-1-1	Bacteria Actinobacteria Streptomycetales Streptomycetaceae *Streptomyces leeuwenhoekii*
Sli-2-1 to Sli-2-2	Bacteria Actinobacteria Streptomycetales Streptomycetaceae *Streptomyces lividans* TK24
Slu-1-1	Bacteria Actinobacteria Streptomycetales Streptomycetaceae *Streptomyces luteus*
Sly-1-1	Bacteria Actinobacteria Streptomycetales Streptomycetaceae *Streptomyces lydicus*
Sly-2-1	Bacteria Actinobacteria Streptomycetales Streptomycetaceae *Streptomyces lydicus* A02
Sme-1-1	Bacteria Actinobacteria Streptomycetales Streptomycetaceae *Streptomyces mediolani*
Sme-2-1	Bacteria Actinobacteria Streptomycetales Streptomycetaceae *Streptomyces megasporus*
Smi-1-1	Bacteria Actinobacteria Streptomycetales Streptomycetaceae *Streptomyces mirabilis*
Smo-2-1	Bacteria Actinobacteria Streptomycetales Streptomycetaceae *Streptomyces monomycini*

Sna-1-1	Bacteria Actinobacteria Streptomycetales Streptomycetaceae *Streptomyces natalensis*
Sna-2-1	Bacteria Actinobacteria Streptomycetales Streptomycetaceae *Streptomyces natalensis* ATCC 27448
Sni-2-1	Bacteria Actinobacteria Streptomycetales Streptomycetaceae *Streptomyces niger*
Sno-1-1	Bacteria Actinobacteria Streptomycetales Streptomycetaceae *Streptomyces nodosus*
Sno-2-1	Bacteria Actinobacteria Streptomycetales Streptomycetaceae *Streptomyces novaecaesareae*
Soc-1-1	Bacteria Actinobacteria Streptomycetales Streptomycetaceae *Streptomyces ochraceiscleroticus*
Sod-1-1	Bacteria Actinobacteria Streptomycetales Streptomycetaceae *Streptomyces odonnellii*
Sol-1-1	Bacteria Actinobacteria Streptomycetales Streptomycetaceae *Streptomyces olindensis*
Sol-2-1 to Sol-2-2	Bacteria Actinobacteria Streptomycetales Streptomycetaceae *Streptomyces olivaceus*
Spe-1-1	Bacteria Actinobacteria Streptomycetales Streptomycetaceae *Streptomyces peruviensis*
Spe-2-1	Bacteria Actinobacteria Streptomycetales Streptomycetaceae *Streptomyces peucetius*
Spl-1-1 to Spl-1-2	Bacteria Actinobacteria Streptomycetales Streptomycetaceae *Streptomyces pluripotens*
Spu-2-1 to Spu-2-2	Bacteria Actinobacteria Streptomycetales Streptomycetaceae *Streptomyces puniceus*
Spu-3-1 to Spu-3-2	Bacteria Actinobacteria Streptomycetales Streptomycetaceae *Streptomyces purpeochromogenes*
Spy-1-1	Bacteria Actinobacteria Streptomycetales Streptomycetaceae *Streptomyces pyridomyceticus*
Sre-1-1	Bacteria Actinobacteria Streptomycetales Streptomycetaceae *Streptomyces resistomycificus*
Sri-2-1 to Sri-2-2	Bacteria Actinobacteria Streptomycetales Streptomycetaceae *Streptomyces rimosus*
Sri-3-1	Bacteria Actinobacteria Streptomycetales Streptomycetaceae *Streptomyces rimosus* R6-500
Sri-4-1 to Sri-4-20	Bacteria Actinobacteria Streptomycetales Streptomycetaceae *Streptomyces rimosus subsp. rimosus*
Sro-4-1	Bacteria Actinobacteria Streptomycetales Streptomycetaceae *Streptomyces roseosporus* NRRL 11379
Sro-5-1	Bacteria Actinobacteria Streptomycetales Streptomycetaceae *Streptomyces roseoverticillatus*
Sro-6-1	Bacteria Actinobacteria Streptomycetales Streptomycetaceae *Streptomyces roseus*
Sru-1-1 to Sru-1-2	Bacteria Actinobacteria Streptomycetales Streptomycetaceae *Streptomyces ruber*
Ssc-3-1 to Ssc-3-2	Bacteria Actinobacteria Streptomycetales Streptomycetaceae *Streptomyces scabiei*
Ssc-4-1 to Ssc-4-2	Bacteria Actinobacteria Streptomycetales Streptomycetaceae *Streptomyces sclerotialus*
Ssc-5-1	Bacteria Actinobacteria Streptomycetales Streptomycetaceae *Streptomyces scopuliridis* RB72
Sse-1-1	Bacteria Actinobacteria Streptomycetales Streptomycetaceae *Streptomyces seoulensis*
Ssp-51-1	Bacteria Actinobacteria Streptomycetales Streptomycetaceae *Streptomyces sp.* 142MFCol3.1
Ssp-52-1	Bacteria Actinobacteria Streptomycetales Streptomycetaceae *Streptomyces sp.* 150FB
Ssp-53-1	Bacteria Actinobacteria Streptomycetales Streptomycetaceae *Streptomyces sp.* 769
Ssp-55-1	Bacteria Actinobacteria Streptomycetales Streptomycetaceae *Streptomyces sp.* AcH 505
Ssp-54-1	Bacteria Actinobacteria Streptomycetales Streptomycetaceae *Streptomyces sp.* AW19M42
Ssp-56-1	Bacteria Actinobacteria Streptomycetales Streptomycetaceae *Streptomyces sp.* CNH099
Ssp-57-1	Bacteria Actinobacteria Streptomycetales Streptomycetaceae *Streptomyces sp.* CNH189
Ssp-58-1	Bacteria Actinobacteria Streptomycetales Streptomycetaceae *Streptomyces sp.* CNH287
Ssp-59-1	Bacteria Actinobacteria Streptomycetales Streptomycetaceae *Streptomyces sp.* CNQ-509
Ssp-60-1	Bacteria Actinobacteria Streptomycetales Streptomycetaceae *Streptomyces sp.* CNQ-525
Ssp-61-1	Bacteria Actinobacteria Streptomycetales Streptomycetaceae *Streptomyces sp.* CNQ329
Ssp-62-1	Bacteria Actinobacteria Streptomycetales Streptomycetaceae *Streptomyces sp.* CNQ431
Ssp-63-1	Bacteria Actinobacteria Streptomycetales Streptomycetaceae *Streptomyces sp.* CNQ865
Ssp-64-1	Bacteria Actinobacteria Streptomycetales Streptomycetaceae *Streptomyces sp.* CNR698
Ssp-65-1	Bacteria Actinobacteria Streptomycetales Streptomycetaceae *Streptomyces sp.* CNS606
Ssp-66-1	Bacteria Actinobacteria Streptomycetales Streptomycetaceae *Streptomyces sp.* CNS654
Ssp-67-1	Bacteria Actinobacteria Streptomycetales Streptomycetaceae *Streptomyces sp.* CNT318
Ssp-68-1	Bacteria Actinobacteria Streptomycetales Streptomycetaceae *Streptomyces sp.* CNT360
Ssp-69-1	Bacteria Actinobacteria Streptomycetales Streptomycetaceae *Streptomyces sp.* CNT371
Ssp-70-1	Bacteria Actinobacteria Streptomycetales Streptomycetaceae *Streptomyces sp.* CT34
Ssp-71-1	Bacteria Actinobacteria Streptomycetales Streptomycetaceae *Streptomyces sp.* DpondAA-B6
Ssp-73-1	Bacteria Actinobacteria Streptomycetales Streptomycetaceae *Streptomyces sp.* FxanaA7
Ssp-72-1	Bacteria Actinobacteria Streptomycetales Streptomycetaceae *Streptomyces sp.* FXJ7.023
Ssp-74-1	Bacteria Actinobacteria Streptomycetales Streptomycetaceae *Streptomyces sp.* GXT6
Ssp-75-1	Bacteria Actinobacteria Streptomycetales Streptomycetaceae *Streptomyces sp.* HNS054
Ssp-76-1	Bacteria Actinobacteria Streptomycetales Streptomycetaceae *Streptomyces sp.* JS01
Ssp-77-1	Bacteria Actinobacteria Streptomycetales Streptomycetaceae *Streptomyces sp.* KE1
Ssp-78-1	Bacteria Actinobacteria Streptomycetales Streptomycetaceae *Streptomyces sp.* KhCrAH-337
Ssp-79-1	Bacteria Actinobacteria Streptomycetales Streptomycetaceae *Streptomyces sp.* KhCrAH-40
Ssp-80-1	Bacteria Actinobacteria Streptomycetales Streptomycetaceae *Streptomyces sp.* M10
Ssp-81-1	Bacteria Actinobacteria Streptomycetales Streptomycetaceae *Streptomyces sp.* MBT28
Ssp-32-1 to Ssp-32-2	Bacteria Actinobacteria Streptomycetales Streptomycetaceae *Streptomyces sp.* Mg1
Ssp-82-1	Bacteria Actinobacteria Streptomycetales Streptomycetaceae *Streptomyces sp.* MNU77
Ssp-83-1	Bacteria Actinobacteria Streptomycetales Streptomycetaceae *Streptomyces sp.* MUSC 125
Ssp-84-1	Bacteria Actinobacteria Streptomycetales Streptomycetaceae *Streptomyces sp.* MUSC119T
Ssp-85-1	Bacteria Actinobacteria Streptomycetales Streptomycetaceae *Streptomyces sp.* MUSC136T
Ssp-86-1	Bacteria Actinobacteria Streptomycetales Streptomycetaceae *Streptomyces sp.* MUSC149T
Ssp-87-1	Bacteria Actinobacteria Streptomycetales Streptomycetaceae *Streptomyces sp.* MUSC164

Ssp-88-1	Bacteria Actinobacteria Streptomycetales Streptomycetaceae *Streptomyces sp.* NBRC 110027
Ssp-89-1	Bacteria Actinobacteria Streptomycetales Streptomycetaceae *Streptomyces sp.* NBRC 110035
Ssp-90-1	Bacteria Actinobacteria Streptomycetales Streptomycetaceae *Streptomyces sp.* NRRL B-11253
Ssp-91-1	Bacteria Actinobacteria Streptomycetales Streptomycetaceae *Streptomyces sp.* NRRL B-12105
Ssp-92-1	Bacteria Actinobacteria Streptomycetales Streptomycetaceae *Streptomyces sp.* NRRL B-1322
Ssp-93-1	Bacteria Actinobacteria Streptomycetales Streptomycetaceae *Streptomyces sp.* NRRL B-1347
Ssp-94-1	Bacteria Actinobacteria Streptomycetales Streptomycetaceae *Streptomyces sp.* NRRL B-1381
Ssp-95-1	Bacteria Actinobacteria Streptomycetales Streptomycetaceae *Streptomyces sp.* NRRL B-24051
Ssp-96-1	Bacteria Actinobacteria Streptomycetales Streptomycetaceae *Streptomyces sp.* NRRL B-24484
Ssp-97-1	Bacteria Actinobacteria Streptomycetales Streptomycetaceae *Streptomyces sp.* NRRL B-24720
Ssp-98-1	Bacteria Actinobacteria Streptomycetales Streptomycetaceae *Streptomyces sp.* NRRL B-3229
Ssp-99-1	Bacteria Actinobacteria Streptomycetales Streptomycetaceae *Streptomyces sp.* NRRL B-3253
Ssp-100-1	Bacteria Actinobacteria Streptomycetales Streptomycetaceae *Streptomyces sp.* NRRL F-2202
Ssp-101-1	Bacteria Actinobacteria Streptomycetales Streptomycetaceae *Streptomyces sp.* NRRL F-2305
Ssp-102-1	Bacteria Actinobacteria Streptomycetales Streptomycetaceae *Streptomyces sp.* NRRL F-2580
Ssp-103-1	Bacteria Actinobacteria Streptomycetales Streptomycetaceae *Streptomyces sp.* NRRL F-2664
Ssp-104-1	Bacteria Actinobacteria Streptomycetales Streptomycetaceae *Streptomyces sp.* NRRL F-2747
Ssp-105-1	Bacteria Actinobacteria Streptomycetales Streptomycetaceae *Streptomyces sp.* NRRL F-2799
Ssp-106-1	Bacteria Actinobacteria Streptomycetales Streptomycetaceae *Streptomyces sp.* NRRL F-2890
Ssp-107-1	Bacteria Actinobacteria Streptomycetales Streptomycetaceae *Streptomyces sp.* NRRL F-3218
Ssp-108-1	Bacteria Actinobacteria Streptomycetales Streptomycetaceae *Streptomyces sp.* NRRL F-3273
Ssp-109-1	Bacteria Actinobacteria Streptomycetales Streptomycetaceae *Streptomyces sp.* NRRL F-3307
Ssp-110-1	Bacteria Actinobacteria Streptomycetales Streptomycetaceae *Streptomyces sp.* NRRL F-4335
Ssp-111-1	Bacteria Actinobacteria Streptomycetales Streptomycetaceae *Streptomyces sp.* NRRL F-4428
Ssp-112-1	Bacteria Actinobacteria Streptomycetales Streptomycetaceae *Streptomyces sp.* NRRL F-4474
Ssp-113-1	Bacteria Actinobacteria Streptomycetales Streptomycetaceae *Streptomyces sp.* NRRL F-4835
Ssp-114-1	Bacteria Actinobacteria Streptomycetales Streptomycetaceae *Streptomyces sp.* NRRL F-5008
Ssp-115-1	Bacteria Actinobacteria Streptomycetales Streptomycetaceae *Streptomyces sp.* NRRL F-5053
Ssp-116-1	Bacteria Actinobacteria Streptomycetales Streptomycetaceae *Streptomyces sp.* NRRL F-5065
Ssp-117-1	Bacteria Actinobacteria Streptomycetales Streptomycetaceae *Streptomyces sp.* NRRL F-5123
Ssp-118-1	Bacteria Actinobacteria Streptomycetales Streptomycetaceae *Streptomyces sp.* NRRL F-5126
Ssp-119-1	Bacteria Actinobacteria Streptomycetales Streptomycetaceae *Streptomyces sp.* NRRL F-5135
Ssp-120-1	Bacteria Actinobacteria Streptomycetales Streptomycetaceae *Streptomyces sp.* NRRL F-5140
Ssp-121-1	Bacteria Actinobacteria Streptomycetales Streptomycetaceae *Streptomyces sp.* NRRL F-5193
Ssp-122-1	Bacteria Actinobacteria Streptomycetales Streptomycetaceae *Streptomyces sp.* NRRL F-525
Ssp-123-1	Bacteria Actinobacteria Streptomycetales Streptomycetaceae *Streptomyces sp.* NRRL F-5527
Ssp-124-1	Bacteria Actinobacteria Streptomycetales Streptomycetaceae *Streptomyces sp.* NRRL F-5555
Ssp-125-1	Bacteria Actinobacteria Streptomycetales Streptomycetaceae *Streptomyces sp.* NRRL F-5630
Ssp-126-1	Bacteria Actinobacteria Streptomycetales Streptomycetaceae *Streptomyces sp.* NRRL F-5635
Ssp-127-1	Bacteria Actinobacteria Streptomycetales Streptomycetaceae *Streptomyces sp.* NRRL F-5639
Ssp-128-1	Bacteria Actinobacteria Streptomycetales Streptomycetaceae *Streptomyces sp.* NRRL F-5650
Ssp-129-1	Bacteria Actinobacteria Streptomycetales Streptomycetaceae *Streptomyces sp.* NRRL F-5681
Ssp-130-1	Bacteria Actinobacteria Streptomycetales Streptomycetaceae *Streptomyces sp.* NRRL F-5702
Ssp-131-1	Bacteria Actinobacteria Streptomycetales Streptomycetaceae *Streptomyces sp.* NRRL F-5727
Ssp-132-1	Bacteria Actinobacteria Streptomycetales Streptomycetaceae *Streptomyces sp.* NRRL F-5917
Ssp-133-1	Bacteria Actinobacteria Streptomycetales Streptomycetaceae *Streptomyces sp.* NRRL F-6131
Ssp-134-1	Bacteria Actinobacteria Streptomycetales Streptomycetaceae *Streptomyces sp.* NRRL F-6628
Ssp-135-1	Bacteria Actinobacteria Streptomycetales Streptomycetaceae *Streptomyces sp.* NRRL F-6674
Ssp-136-1	Bacteria Actinobacteria Streptomycetales Streptomycetaceae *Streptomyces sp.* NRRL F-6676
Ssp-137-1	Bacteria Actinobacteria Streptomycetales Streptomycetaceae *Streptomyces sp.* NRRL F-6677
Ssp-138-1	Bacteria Actinobacteria Streptomycetales Streptomycetaceae *Streptomyces sp.* NRRL S-1022
Ssp-139-1	Bacteria Actinobacteria Streptomycetales Streptomycetaceae *Streptomyces sp.* NRRL S-104
Ssp-140-1	Bacteria Actinobacteria Streptomycetales Streptomycetaceae *Streptomyces sp.* NRRL S-118
Ssp-141-1	Bacteria Actinobacteria Streptomycetales Streptomycetaceae *Streptomyces sp.* NRRL S-1314
Ssp-142-1	Bacteria Actinobacteria Streptomycetales Streptomycetaceae *Streptomyces sp.* NRRL S-1448
Ssp-143-1	Bacteria Actinobacteria Streptomycetales Streptomycetaceae *Streptomyces sp.* NRRL S-146
Ssp-144-1	Bacteria Actinobacteria Streptomycetales Streptomycetaceae *Streptomyces sp.* NRRL S-15
Ssp-145-1	Bacteria Actinobacteria Streptomycetales Streptomycetaceae *Streptomyces sp.* NRRL S-1813
Ssp-146-1	Bacteria Actinobacteria Streptomycetales Streptomycetaceae *Streptomyces sp.* NRRL S-1824
Ssp-147-1	Bacteria Actinobacteria Streptomycetales Streptomycetaceae *Streptomyces sp.* NRRL S-1831
Ssp-148-1	Bacteria Actinobacteria Streptomycetales Streptomycetaceae *Streptomyces sp.* NRRL S-1868
Ssp-149-1	Bacteria Actinobacteria Streptomycetales Streptomycetaceae *Streptomyces sp.* NRRL S-1896
Ssp-150-1	Bacteria Actinobacteria Streptomycetales Streptomycetaceae *Streptomyces sp.* NRRL S-237
Ssp-151-1	Bacteria Actinobacteria Streptomycetales Streptomycetaceae *Streptomyces sp.* NRRL S-241
Ssp-152-1	Bacteria Actinobacteria Streptomycetales Streptomycetaceae *Streptomyces sp.* NRRL S-244

Ssp-153-1	Bacteria Actinobacteria Streptomycetales Streptomycetaceae *Streptomyces sp.* NRRL S-31
Ssp-154-1	Bacteria Actinobacteria Streptomycetales Streptomycetaceae *Streptomyces sp.* NRRL S-325
Ssp-155-1	Bacteria Actinobacteria Streptomycetales Streptomycetaceae *Streptomyces sp.* NRRL S-337
Ssp-156-1	Bacteria Actinobacteria Streptomycetales Streptomycetaceae *Streptomyces sp.* NRRL S-340
Ssp-157-1	Bacteria Actinobacteria Streptomycetales Streptomycetaceae *Streptomyces sp.* NRRL S-350
Ssp-158-1	Bacteria Actinobacteria Streptomycetales Streptomycetaceae *Streptomyces sp.* NRRL S-37
Ssp-159-1	Bacteria Actinobacteria Streptomycetales Streptomycetaceae *Streptomyces sp.* NRRL S-378
Ssp-160-1	Bacteria Actinobacteria Streptomycetales Streptomycetaceae *Streptomyces sp.* NRRL S-384
Ssp-161-1	Bacteria Actinobacteria Streptomycetales Streptomycetaceae *Streptomyces sp.* NRRL S-455
Ssp-162-1	Bacteria Actinobacteria Streptomycetales Streptomycetaceae *Streptomyces sp.* NRRL S-474
Ssp-163-1	Bacteria Actinobacteria Streptomycetales Streptomycetaceae *Streptomyces sp.* NRRL S-475
Ssp-164-1	Bacteria Actinobacteria Streptomycetales Streptomycetaceae *Streptomyces sp.* NRRL S-481
Ssp-165-1	Bacteria Actinobacteria Streptomycetales Streptomycetaceae *Streptomyces sp.* NRRL S-495
Ssp-166-1	Bacteria Actinobacteria Streptomycetales Streptomycetaceae *Streptomyces sp.* NRRL S-515
Ssp-167-1	Bacteria Actinobacteria Streptomycetales Streptomycetaceae *Streptomyces sp.* NRRL S-575
Ssp-168-1	Bacteria Actinobacteria Streptomycetales Streptomycetaceae *Streptomyces sp.* NRRL S-623
Ssp-169-1	Bacteria Actinobacteria Streptomycetales Streptomycetaceae *Streptomyces sp.* NRRL S-646
Ssp-170-1	Bacteria Actinobacteria Streptomycetales Streptomycetaceae *Streptomyces sp.* NRRL S-813
Ssp-171-1	Bacteria Actinobacteria Streptomycetales Streptomycetaceae *Streptomyces sp.* NRRL S-87
Ssp-172-1	Bacteria Actinobacteria Streptomycetales Streptomycetaceae *Streptomyces sp.* NRRL S-920
Ssp-173-1	Bacteria Actinobacteria Streptomycetales Streptomycetaceae *Streptomyces sp.* NRRL S-98
Ssp-174-1	Bacteria Actinobacteria Streptomycetales Streptomycetaceae *Streptomyces sp.* NRRL WC-3540
Ssp-175-1	Bacteria Actinobacteria Streptomycetales Streptomycetaceae *Streptomyces sp.* NRRL WC-3549
Ssp-176-1	Bacteria Actinobacteria Streptomycetales Streptomycetaceae *Streptomyces sp.* NRRL WC-3626
Ssp-177-1	Bacteria Actinobacteria Streptomycetales Streptomycetaceae *Streptomyces sp.* NRRL WC-3641
Ssp-178-1	Bacteria Actinobacteria Streptomycetales Streptomycetaceae *Streptomyces sp.* NRRL WC-3683
Ssp-179-1	Bacteria Actinobacteria Streptomycetales Streptomycetaceae *Streptomyces sp.* NRRL WC-3702
Ssp-180-1	Bacteria Actinobacteria Streptomycetales Streptomycetaceae *Streptomyces sp.* NRRL WC-3703
Ssp-181-1	Bacteria Actinobacteria Streptomycetales Streptomycetaceae *Streptomyces sp.* NRRL WC-3719
Ssp-182-1	Bacteria Actinobacteria Streptomycetales Streptomycetaceae *Streptomyces sp.* NRRL WC-3725
Ssp-183-1	Bacteria Actinobacteria Streptomycetales Streptomycetaceae *Streptomyces sp.* NRRL WC-3742
Ssp-184-1 to Ssp-184-2	Bacteria Actinobacteria Streptomycetales Streptomycetaceae *Streptomyces sp.* NRRL WC-3744
Ssp-185-1	Bacteria Actinobacteria Streptomycetales Streptomycetaceae *Streptomyces sp.* NRRL WC-3773
Ssp-186-1	Bacteria Actinobacteria Streptomycetales Streptomycetaceae *Streptomyces sp.* NRRL WC-3774
Ssp-187-1	Bacteria Actinobacteria Streptomycetales Streptomycetaceae *Streptomyces sp.* NRRL WC-3795
Ssp-188-1	Bacteria Actinobacteria Streptomycetales Streptomycetaceae *Streptomyces sp.* NTK 937
Ssp-189-1	Bacteria Actinobacteria Streptomycetales Streptomycetaceae *Streptomyces sp.* PCS3-D2
Ssp-190-1	Bacteria Actinobacteria Streptomycetales Streptomycetaceae *Streptomyces sp.* PRh5
Ssp-191-1	Bacteria Actinobacteria Streptomycetales Streptomycetaceae *Streptomyces sp.* RSD-27
Ssp-192-1	Bacteria Actinobacteria Streptomycetales Streptomycetaceae *Streptomyces sp.* SBT349
Ssp-193-1	Bacteria Actinobacteria Streptomycetales Streptomycetaceae *Streptomyces sp.* SolWspMP-sol2th
Ssp-194-1	Bacteria Actinobacteria Streptomycetales Streptomycetaceae *Streptomyces sp.* TAA040
Ssp-195-1	Bacteria Actinobacteria Streptomycetales Streptomycetaceae *Streptomyces sp.* TAA204
Ssp-196-1	Bacteria Actinobacteria Streptomycetales Streptomycetaceae *Streptomyces sp.* Tu 6176
Ssp-197-1	Bacteria Actinobacteria Streptomycetales Streptomycetaceae *Streptomyces sp.* UNC401CLCol
Ssp-198-1	Bacteria Actinobacteria Streptomycetales Streptomycetaceae *Streptomyces sp.* URHA0041
Ssp-199-1	Bacteria Actinobacteria Streptomycetales Streptomycetaceae *Streptomyces sp.* WM6386
Ssp-200-1	Bacteria Actinobacteria Streptomycetales Streptomycetaceae *Streptomyces sp.* WM6391
Ssp-201-1	Bacteria Actinobacteria Streptomycetales Streptomycetaceae *Streptomyces sp.* WMMB 714
Sst-1-1	Bacteria Actinobacteria Streptomycetales Streptomycetaceae *Streptomyces stelliscabiei*
Sto-1-1	Bacteria Actinobacteria Streptomycetales Streptomycetaceae *Streptomyces toyocaensis*
Sva-1-1	Bacteria Actinobacteria Streptomycetales Streptomycetaceae *Streptomyces variegatus*
Sva-2-1 to Sva-2-2	Bacteria Actinobacteria Streptomycetales Streptomycetaceae *Streptomyces varsoviensis*
Svi-7-1	Bacteria Actinobacteria Streptomycetales Streptomycetaceae *Streptomyces vietnamensis*
Svi-8-1	Bacteria Actinobacteria Streptomycetales Streptomycetaceae *Streptomyces vinaceus*
Svi-9-1 to Svi-9-2	Bacteria Actinobacteria Streptomycetales Streptomycetaceae *Streptomyces violaceoruber*
Svi-10-1	Bacteria Actinobacteria Streptomycetales Streptomycetaceae *Streptomyces violaceorubidus*
Svi-11-1	Bacteria Actinobacteria Streptomycetales Streptomycetaceae *Streptomyces violens*
Svi-12-1 to Svi-12-3	Bacteria Actinobacteria Streptomycetales Streptomycetaceae *Streptomyces virginiae*
Svi-13-1 to Svi-13-4	Bacteria Actinobacteria Streptomycetales Streptomycetaceae *Streptomyces viridochromogenes*
Swa-1-1	Bacteria Actinobacteria Streptomycetales Streptomycetaceae *Streptomyces wadayamensis*
Swe-1-1	Bacteria Actinobacteria Streptomycetales Streptomycetaceae *Streptomyces wedmorensis*
Sxa-1-1 to Sxa-1-2	Bacteria Actinobacteria Streptomycetales Streptomycetaceae *Streptomyces xanthophaeus*
Sxi-1-1	Bacteria Actinobacteria Streptomycetales Streptomycetaceae *Streptomyces xiamenensis*
Sxi-2-1	Bacteria Actinobacteria Streptomycetales Streptomycetaceae *Streptomyces xinghaiensis* S187

Sxy-1-1	Bacteria Actinobacteria Streptomycetales Streptomycetaceae *Streptomyces xylophagus*
Sya-1-1	Bacteria Actinobacteria Streptomycetales Streptomycetaceae *Streptomyces yangpuensis*
Sye-1-1	Bacteria Actinobacteria Streptomycetales Streptomycetaceae *Streptomyces yeochonensis* CN732
Sye-2-1	Bacteria Actinobacteria Streptomycetales Streptomycetaceae *Streptomyces yerevanensis*
Hcr-1-1	Bacteria Actinobacteria Streptosporangiales Streptosporangiaceae *Herbidospora cretacea*
Mro-1-1	Bacteria Actinobacteria Streptosporangiales Streptosporangiaceae *Microbispora rosea subsp. nonnitritogenes*
Msp-1-1	Bacteria Actinobacteria Streptosporangiales Streptosporangiaceae *Microbispora sp.* ATCC PTA-5024
Mgl-1-1	Bacteria Actinobacteria Streptosporangiales Streptosporangiaceae *Microtetraspora glauca*
Nca-1-1	Bacteria Actinobacteria Streptosporangiales Streptosporangiaceae *Nonomuraea candida*
Nsp-4-1	Bacteria Actinobacteria Streptosporangiales Streptosporangiaceae *Nonomuraea sp.* SBT364
Sam-2-1	Bacteria Actinobacteria Streptosporangiales Streptosporangiaceae *Streptosporangium amethystogenes*
Sro-7-1	Bacteria Actinobacteria Streptosporangiales Streptosporangiaceae *Streptosporangium roseum*
Ama-1-1	Bacteria Actinobacteria Streptosporangiales Thermomonosporaceae *Actinomadura madurae* LIID-AJ290
Ari-1-1	Bacteria Actinobacteria Streptosporangiales Thermomonosporaceae *Actinomadura rifamycini* DSM 43936
Sal-13-1	Bacteria Actinobacteria Streptosporangiales Thermomonosporaceae *Spirillospora albida*
[br-1-1	Bacteria Proteobacteria Betaproteobacteria Burkholderiales [Polyangium] brachysporum
Rge-1-1	Bacteria Proteobacteria Betaproteobacteria Burkholderiales *Rubrivivax gelatinosus* CBS
Age-1-1	Bacteria Proteobacteria Deltaproteobacteria Myxococcales Cystobacterineae Archangiaceae *Archangium gephyra*
Avi-1-1	Bacteria Proteobacteria Deltaproteobacteria Myxococcales Cystobacterineae Archangiaceae *Archangium violaceum* Cb vi76
Hmi-1-1	Bacteria Proteobacteria Deltaproteobacteria Myxococcales Cystobacterineae Archangiaceae *Hyalangium minutum*
Cfu-1-1	Bacteria Proteobacteria Deltaproteobacteria Myxococcales Cystobacterineae Cystobacteraceae *Cystobacter fuscus* DSM 2262
Sau-5-1	Bacteria Proteobacteria Deltaproteobacteria Myxococcales Cystobacterineae Cystobacteraceae *Stigmatella aurantiaca* DW4/3-1
Cco-1-1	Bacteria Proteobacteria Deltaproteobacteria Myxococcales Cystobacterineae Myxococcaceae *Corallococcus coralloides* DSM 2259
Mfu-1-1	Bacteria Proteobacteria Deltaproteobacteria Myxococcales Cystobacterineae Myxococcaceae *Myxococcus fulvus* 124B02
Mfu-2-1	Bacteria Proteobacteria Deltaproteobacteria Myxococcales Cystobacterineae Myxococcaceae *Myxococcus fulvus* HW-1
Mha-1-1	Bacteria Proteobacteria Deltaproteobacteria Myxococcales Cystobacterineae Myxococcaceae *Myxococcus hansupus*
Msp-2-1	Bacteria Proteobacteria Deltaproteobacteria Myxococcales Cystobacterineae Myxococcaceae *Myxococcus sp. (contaminant ex* DSM 436)
Mst-1-1	Bacteria Proteobacteria Deltaproteobacteria Myxococcales Cystobacterineae Myxococcaceae *Myxococcus stipitatus* DSM 14675
Mxa-1-1	Bacteria Proteobacteria Deltaproteobacteria Myxococcales Cystobacterineae Myxococcaceae *Myxococcus xanthus* DK 1622
Mxa-2-1	Bacteria Proteobacteria Deltaproteobacteria Myxococcales Cystobacterineae Myxococcaceae *Myxococcus xanthus* DZ2
Mxa-3-1	Bacteria Proteobacteria Deltaproteobacteria Myxococcales Cystobacterineae Myxococcaceae *Myxococcus xanthus* DZF1
env-1 to env-41	environmental samples

5.2 Notes

Superscript numbers are used to annotate any L17-Actino-Proteobacteria RNA that has special characteristics (described below). These numeric annotations will be used in Sections 5.3 and 5.5:. The meaning of the numbers are as follows.

[1] Left hairpin does not conform well to the characteristics of those in most other sequences.

5.3 Gene contexts

Each L17-Actino-Proteobacteria RNA (indicated by "RNA→") is listed. For each hit, the downstream genes predicted to reside in a regulated operon are listed. If the nearest downstream gene is encoding in the opposite strand (and therefore presumed to not be a part of a regulated operon), then that gene is still depicted. Some environmental sequences and some RefSeq entries lack gene annotations, and so no genes are available for such sequences. The direction of each gene is indicated with an arrow (→), and each predicted conserved domain in the gene is named. Conserved domains associated with more than one L17-Actino-Proteobacteria RNA are assigned a color; other domains are gray. Information about these conserved domains is given in Section 5.4. The accession of the sequence containing each L17-Actino-Proteobacteria RNA is given in the column named "Seq. accession". Accessions beginning with "NC_", "NS_", "NW_" or "NZ_" are contained in RefSeq. Other accession refer to environmental samples. Nucleotide coordinates are given for the 5′ and 3′ boundaries of each L17-Actino-Proteobacteria RNA. If the 5′ coordinate is greater than the 3′ coordinate, the RNA is present on the reverse-complement strand of the containing genomic DNA sequence. Each hit is denoted by an abbreviation (like "Eco-1-1") that refers to a taxonomy given in Section 5.1. (Superscript numbers refer to annotations listed in Section 5.2)

abbrev.	Seq. accession		5′ at	3′ at	genes
env-1	C687J26621_10007310	-	562	514	**RNA**→ RplQ (COG0203)rplQ (PRK05591)→ hypo→
env-2	C687J26621_10071554	-	1140	1092	**RNA**→ hypo→ RplQ (COG0203)rplQ (PRK05591)→ hypo→ hypo→
[br-1-1	NZ_CP011371.1	-	5726771	5726711	**RNA**→ RplQ (COG0203)rplQ (PRK05591)→
env-3	soil_Contig5330	+	734	782	**RNA**→ RplQ (COG0203)rplQ (PRK05591)→ hypo→ hypo→ hypo→
Nal-1-1	NZ_AUFN01000013.1	-	290198	290141	**RNA**→ RplQ (COG0203)rplQ (PRK05591)→ hypo→
Nsp-1-1	NZ_JAER01000028.1	+	47081	47133	**RNA**→ RplQ (COG0203)rplQ (PRK05591)→
Mxa-1-1	NC_008095.1	+	3870176	3870231	**RNA**→ RplQ (COG0203)rplQ (PRK05591)→
Mxa-2-1	NZ_AKYI02000032.1	+	15862	15917	**RNA**→ RplQ (COG0203)rplQ (PRK05591)→
Mxa-3-1	NZ_AOBT01000010.1	-	325703	325648	**RNA**→ RplQ (COG0203)rplQ (PRK05591)→
env-4	G312J29652_10012153	-	190	133	**RNA**→ RplQ (COG0203)rplQ (PRK05591)→
Cfu-1-1	NZ_ANAH02000033.1	-	26146	26089	**RNA**→ RplQ (COG0203)rplQ (PRK05591)→
Avi-1-1	NZ_JPMI01000295.1	+	16333	16389	**RNA**→ RplQ (COG0203)rplQ (PRK05591)→
Age-1-1	NZ_CP011509.1	+	5915327	5915383	**RNA**→ RplQ (COG0203)rplQ (PRK05591)→
Hmi-1-1	NZ_JMCB01000003.1	+	16437	16494	**RNA**→ RplQ (COG0203)rplQ (PRK05591)→
env-5	JGI12467J12023_1013060	+	231	287	**RNA**→ RplQ (COG0203)rplQ (PRK05591)→
Sau-5-1	NC_014623.1	+	4510803	4510859	**RNA**→ RplQ (COG0203)rplQ (PRK05591)→
Mfu-1-1	NZ_CP006003.1	-	6488472	6488416	**RNA**→ RplQ (COG0203)rplQ (PRK05591)→
Mst-1-1	NC_020126.1	-	5952427	5952371	**RNA**→ RplQ (COG0203)rplQ (PRK05591)→
Msp-2-1	NZ_AKXT02000011.1	-	416054	415998	**RNA**→ RplQ (COG0203)rplQ (PRK05591)→
Mha-1-1	NZ_CP012109.1	-	4586241	4586185	**RNA**→ RplQ (COG0203)rplQ (PRK05591)→
Mfu-2-1	NC_015711.1	+	5782024	5782080	**RNA**→ RplQ (COG0203)rplQ (PRK05591)→
env-6	JGI12467J12023_1015101	+	1120	1177	**RNA**→ RplQ (COG0203)rplQ (PRK05591)→
Cco-1-1	NC_017030.1	+	3764344	3764400	**RNA**→ RplQ (COG0203)rplQ (PRK05591)→
env-7	SRB_FXPBX6M01ETLHE	-	521	467	**RNA**→ RplQ (COG0203)rplQ (PRK05591)→
env-8	LWAnN_GIDYKCY01AF5CK	-	393	338	**RNA**→ RplQ (COG0203)rplQ (PRK05591)→
Ama-1-1	NZ_AWOO02000003.1	-	173008	172954	**RNA**→ rplQ (PRK05591)Ribosomal_L17 (pfam01196)→
env-9	topACOD_F95O9CU02FUYVO	+	189	240	**RNA**→ rplQ (PRK05591)Ribosomal_L17 (pfam01196)→
env-10	botACOD_contig00616	+	47	98	**RNA**→ rplQ (PRK05591)Ribosomal_L17 (pfam01196)→ PseudoU_synth_EcTruA (cd02570)truA (PRK00021)→
					AdoMet_MTases (cd02440)Methyltransf_25 (pfam13649)→
					AAA (smart00382)Uup (COG0488)ABC_tran_Xtn (pfam12848)→
Ssp-68-1	NZ_AUKO01000030.1	+	121030	121087	**RNA**→ rplQ (PRK05591)Ribosomal_L17 (pfam01196)→
[1]env-11	topACOD_F95O9CU01C589N	-	233	171	**RNA**→ rplQ (PRK05591)Ribosomal_L17 (pfam01196)→
env-12	DCrCPGB_c521527	+	223	278	**RNA**→ RplQ (COG0203)rplQ (PRK05591)→
Ssp-96-1	NZ_JOAI01000002.1	-	176853	176802	**RNA**→ rplQ (PRK05591)Ribosomal_L17 (pfam01196)→ PseudoU_synth_EcTruA (cd02570)truA (PRK00021)→
Ksp-1-1	NZ_JAIZ01000648.1	-	13562	13510	**RNA**→ rplQ (PRK05591)Ribosomal_L17 (pfam01196)→ PseudoU_synth_EcTruA (cd02570)truA (PRK00021)→
					AAA (smart00382)Uup (COG0488)→
Kme-1-1	NZ_JQLN01000005.1	+	2502927	2502979	**RNA**→ rplQ (PRK05591)Ribosomal_L17 (pfam01196)→ PseudoU_synth_EcTruA (cd02570)truA (PRK00021)→
					AAA (smart00382)Uup (COG0488)AAA_21 (pfam13304)→
Rge-1-1	NZ_AJFF01000004.1	+	16232	16281	**RNA**→ RplQ (COG0203)rplQ (PRK05591)→
Sca-3-1	NZ_BBPM01000018.1	-	109868	109813	**RNA**→ rplQ (PRK05591)Ribosomal_L17 (pfam01196)→ PseudoU_synth_EcTruA (cd02570)truA (PRK00021)→
Sal-3-1	NZ_BBPL01000005.1	+	86334	86389	**RNA**→ rplQ (PRK05591)Ribosomal_L17 (pfam01196)→
Sal-4-1	NZ_JQML01000001.1	+	5666634	5666689	**RNA**→ rplQ (PRK05591)Ribosomal_L17 (pfam01196)→
env-13	JGI24770J26754_10005505	-	4344	4287	**RNA**→ RplQ (COG0203)rplQ (PRK05591)PRK11855 (PRK11855)→
					PRK09434 (PRK09434)bac_FRK (cd01167)→ CarboxypepD_reg (pfam13620)→
Sav-3-1	NZ_JOEK01000005.1	-	58397	58339	**RNA**→ RplQ (COG0203)rplQ (PRK05591)→
[1]Ssp-195-1	NZ_AUKW01000004.1	-	554930	554861	**RNA**→ PRK13428 (PRK13428)rplQ (PRK05591)Ribosomal_L17 (pfam01196)→
					PseudoU_synth_EcTruA (cd02570)truA (PRK00021)→
Nsp-4-1	NZ_LAVL01000256.1	-	2722	2668	**RNA**→ rplQ (PRK05591)Ribosomal_L17 (pfam01196)→ PseudoU_synth_EcTruA (cd02570)truA (PRK00021)→
env-14	botACOD_F64RS5002G1I32	+	29	84	**RNA**→ rplQ (PRK05591)Ribosomal_L17 (pfam01196)→
Nco-1-1	NZ_KB903951.1	-	119743	119681	**RNA**→ rplQ (PRK05591)Ribosomal_L17 (pfam01196)→ PseudoU_synth_EcTruA (cd02570)truA (PRK00021)→
Sje-1-1	NZ_JOEH01000005.1	-	70730	70674	**RNA**→ rplQ (PRK05591)Ribosomal_L17 (pfam01196)→ PseudoU_synth_EcTruA (cd02570)truA (PRK00021)→
Kaz-1-1	NZ_JQMO01000003.1	+	3548080	3548135	**RNA**→ rplQ (PRK05591)Ribosomal_L17 (pfam01196)→

Kph-1-1	NZ_JNWZ01000014.1	-	38553	38499	RNA→ rplQ (PRK05591)Ribosomal_L17 (pfam01196)→ PseudoU_synth_EcTruA (cd02570)truA (PRK00021)→ AAA (smart00382)Uup (COG0488)→
Kph-1-2	NZ_JNYE01000006.1	+	139183	139237	RNA→ rplQ (PRK05591)Ribosomal_L17 (pfam01196)→
Sno-2-1	NZ_JNWQ01000041.1	-	6189	6134	RNA→ rplQ (PRK05591)Ribosomal_L17 (pfam01196)→
Ksp-3-1	NZ_LFVW01000009.1	+	42129	42184	RNA→ rplQ (PRK05591)Ribosomal_L17 (pfam01196)→ PseudoU_synth_EcTruA (cd02570)truA (PRK00021)→ AAA (smart00382)Uup (COG0488)AAA_21 (pfam13304)→
Spy-1-1	NZ_KL573373.1	+	195480	195535	RNA→ rplQ (PRK05591)Ribosomal_L17 (pfam01196)→
Ksp-4-1	NZ_JOGH01000001.1	-	191862	191808	RNA→ rplQ (PRK05591)Ribosomal_L17 (pfam01196)→ PseudoU_synth_EcTruA (cd02570)truA (PRK00021)→ AAA (smart00382)Uup (COG0488)→
Ssp-165-1	NZ_JZWY01000017.1	-	18645	18591	RNA→ rplQ (PRK05591)Ribosomal_L17 (pfam01196)→
Ssp-133-1	NZ_JOHN01000002.1	+	49938	49992	RNA→ rplQ (PRK05591)Ribosomal_L17 (pfam01196)→ PseudoU_synth_EcTruA (cd02570)truA (PRK00021)→ AAA (smart00382)Uup (COG0488)→
Ssp-50-1	NZ_JYJF01000115.1	-	10192	10138	RNA→ rplQ (PRK05591)Ribosomal_L17 (pfam01196)→ PseudoU_synth_EcTruA (cd02570)truA (PRK00021)→
Ssp-157-1	NZ_JOHO01000003.1	+	268650	268704	RNA→ rplQ (PRK05591)Ribosomal_L17 (pfam01196)→ PseudoU_synth_EcTruA (cd02570)truA (PRK00021)→ AAA (smart00382)Uup (COG0488)AAA_21 (pfam13304)→
Ssp-160-1	NZ_JOJA01000001.1	+	115341	115395	RNA→ rplQ (PRK05591)Ribosomal_L17 (pfam01196)→ PseudoU_synth_EcTruA (cd02570)truA (PRK00021)→
Ksp-2-1	NZ_JAIY01000008.1	-	87931	87877	RNA→ rplQ (PRK05591)Ribosomal_L17 (pfam01196)→ PseudoU_synth_EcTruA (cd02570)truA (PRK00021)→ AAA (smart00382)Uup (COG0488)→
Kpu-1-1	NZ_JODS01000006.1	+	94156	94210	RNA→ rplQ (PRK05591)Ribosomal_L17 (pfam01196)→
Ssp-183-1	NZ_JOCF01000001.1	-	67177	67123	RNA→ rplQ (PRK05591)Ribosomal_L17 (pfam01196)→ PseudoU_synth_EcTruA (cd02570)truA (PRK00021)→ AAA (smart00382)Uup (COG0488)AAA_21 (pfam13304)→
Sam-2-1	NZ_JOEQ01000010.1	-	266003	265948	RNA→ rplQ (PRK05591)Ribosomal_L17 (pfam01196)→ PseudoU_synth_EcTruA (cd02570)truA (PRK00021)→
Sro-7-1	NZ_JOEP01000065.1	+	28073	28126	RNA→ rplQ (PRK05591)Ribosomal_L17 (pfam01196)→ PseudoU_synth_EcTruA (cd02570)truA (PRK00021)→
Ssp-87-1	NZ_LAKD01000081.1	+	62702	62751	RNA→ rplQ (PRK05591)Ribosomal_L17 (pfam01196)→ PseudoU_synth_EcTruA (cd02570)truA (PRK00021)→
Ssp-190-1	NZ_JABQ01000020.1	+	41705	41754	RNA→ rplQ (PRK05591)Ribosomal_L17 (pfam01196)→ PseudoU_synth_EcTruA (cd02570)truA (PRK00021)→
Sir-1-1	NZ_LK022848.1	-	5397417	5397368	RNA→ rplQ (PRK05591)Ribosomal_L17 (pfam01196)→ PseudoU_synth_EcTruA (cd02570)truA (PRK00021)→
Shy-4-1	NZ_JOIK01000025.1	+	178719	178768	RNA→ rplQ (PRK05591)Ribosomal_L17 (pfam01196)→ PseudoU_synth_EcTruA (cd02570)truA (PRK00021)→
Sro-3-1	NC_013595.1	+	1131580	1131634	RNA→ rplQ (PRK05591)Ribosomal_L17 (pfam01196)→ PseudoU_synth_EcTruA (cd02570)truA (PRK00021)→
Nin-1-1	NZ_KE383928.1	+	830235	830308	RNA→ rplQ (PRK05591)Ribosomal_L17 (pfam01196)→
Nca-1-1	NZ_JOAG01000016.1	+	29392	29446	RNA→ rplQ (PRK05591)Ribosomal_L17 (pfam01196)→
[1]Ssp-192-1	NZ_LAVK01000035.1	+	24842	24902	RNA→ rplQ (PRK05591)Ribosomal_L17 (pfam01196)→ PseudoU_synth_EcTruA (cd02570)truA (PRK00021)→
Ssp-172-1	NZ_JODF01000016.1	-	82667	82612	RNA→ rplQ (PRK05591)Ribosomal_L17 (pfam01196)→ PseudoU_synth_EcTruA (cd02570)truA (PRK00021)→
Ssp-93-1	NZ_JOJM01000060.1	-	2808	2751	RNA→ rplQ (PRK05591)Ribosomal_L17 (pfam01196)→
Sau-3-1	NZ_JOAP01000029.1	+	78987	79043	RNA→ rplQ (PRK05591)Ribosomal_L17 (pfam01196)→
Sau-3-2	NZ_JOAR01000028.1	+	78983	79039	RNA→ rplQ (PRK05591)Ribosomal_L17 (pfam01196)→
Sal-6-1	NZ_JNXT01000010.1	-	3945	3889	RNA→ rplQ (PRK05591)Ribosomal_L17 (pfam01196)→ PseudoU_synth_EcTruA (cd02570)truA (PRK00021)→
Sru-1-1	NZ_JODN01000016.1	-	4008	3952	RNA→ rplQ (PRK05591)Ribosomal_L17 (pfam01196)→ PseudoU_synth_EcTruA (cd02570)truA (PRK00021)→
Sru-1-2	NZ_JOAQ01000016.1	-	4052	3996	RNA→ rplQ (PRK05591)Ribosomal_L17 (pfam01196)→ PseudoU_synth_EcTruA (cd02570)truA (PRK00021)→
Sau-1-1	NZ_AOPZ01000478.1	-	3734	3676	RNA→ ←hypo
Ssp-106-1	NZ_JOIG01000009.1	-	261110	261058	RNA→ rplQ (PRK05591)Ribosomal_L17 (pfam01196)→ PseudoU_synth_EcTruA (cd02570)truA (PRK00021)→
Sxi-1-1	NZ_CP009922.2	-	2234636	2234584	RNA→ rplQ (PRK05591)Ribosomal_L17 (pfam01196)→ PseudoU_synth_EcTruA (cd02570)truA (PRK00021)→
Ssp-3-1	NZ_ALNP01000008.1	+	75229	75281	RNA→ rplQ (PRK05591)Ribosomal_L17 (pfam01196)→ PseudoU_synth_EcTruA (cd02570)truA (PRK00021)→
env-15	JGI12419J13241_1048700	+	807	860	RNA→
env-16	JGI12341J12878_1001749	+	4790	4843	RNA→ rplQ (PRK05591)Ribosomal_L17 (pfam01196)→
env-17	JGI12298J13902_1000008	-	41137	41084	RNA→ rplQ (PRK05591)Ribosomal_L17 (pfam01196)→
Tbi-1-1	NC_014165.1	+	644331	644384	RNA→ rplQ (PRK05591)Ribosomal_L17 (pfam01196)→
env-18	JGI12419J13241_1045219	-	901	848	RNA→ rplQ (PRK05591)Ribosomal_L17 (pfam01196)→
Ssp-118-1	NZ_JOFZ01000006.1	-	529759	529704	RNA→ rplQ (PRK05591)Ribosomal_L17 (pfam01196)→
env-19	2200133452	+	1231	1291	RNA→ rplQ (PRK05591)Ribosomal_L17 (pfam01196)→
env-20	2200130945	-	4098	4038	RNA→ rplQ (PRK05591)Ribosomal_L17 (pfam01196)→
env-21	JGI12419J13241_1059560	-	305	245	RNA→ rplQ (PRK05591)Ribosomal_L17 (pfam01196)→
Sor-1-1	NZ_JQMQ01000005.1	+	4289657	4289704	RNA→ rplQ (PRK05591)Ribosomal_L17 (pfam01196)→ PseudoU_synth_EcTruA (cd02570)truA (PRK00021)→
Ssp-64-1	NZ_AZXC01000006.1	-	241043	240986	RNA→ rplQ (PRK05591)rpsP (PRK14521)Ribosomal_L17 (pfam01196)→ PseudoU_synth_EcTruA (cd02570)truA (PRK00021)→

Ssp-12-1	NZ_KB846744.1	-	49103	49046	**RNA**→ rplQ (PRK05591)Ribosomal_L17 (pfam01196)→ PseudoU_synth_EcTruA (cd02570)truA (PRK00021)→
Ssp-13-1	NZ_KB898271.1	-	505841	505784	**RNA**→ rplQ (PRK05591)Ribosomal_L17 (pfam01196)→ PseudoU_synth_EcTruA (cd02570)truA (PRK00021)→
Ssp-61-1	NZ_AXVU01000018.1	-	122599	122542	**RNA**→ rplQ (PRK05591)Ribosomal_L17 (pfam01196)→ PseudoU_synth_EcTruA (cd02570)truA (PRK00021)→
Ssp-59-1	NZ_CP011492.1	+	4931209	4931266	**RNA**→ rplQ (PRK05591)Ribosomal_L17 (pfam01196)→ PseudoU_synth_EcTruA (cd02570)truA (PRK00021)→
Ssp-16-1	NZ_KB897717.1	-	411625	411568	**RNA**→ rplQ (PRK05591)Ribosomal_L17 (pfam01196)→ PseudoU_synth_EcTruA (cd02570)truA (PRK00021)→
Ssp-10-1	NZ_KB897364.1	-	373832	373775	**RNA**→ rplQ (PRK05591)Ribosomal_L17 (pfam01196)→ PseudoU_synth_EcTruA (cd02570)truA (PRK00021)→
Ssp-11-1	NZ_KB897623.1	+	230842	230899	**RNA**→ rplQ (PRK05591)Ribosomal_L17 (pfam01196)→ PseudoU_synth_EcTruA (cd02570)truA (PRK00021)→
Ssp-63-1	NZ_AUKP01000008.1	+	18089	18146	**RNA**→ rplQ (PRK05591)Ribosomal_L17 (pfam01196)→ PseudoU_synth_EcTruA (cd02570)truA (PRK00021)→
Ssp-60-1	NZ_JNID01000001.1	+	69239	69296	**RNA**→ rplQ (PRK05591)Ribosomal_L17 (pfam01196)→ PseudoU_synth_EcTruA (cd02570)truA (PRK00021)→
Ssp-69-1	NZ_AZWZ01000009.1	-	266579	266522	**RNA**→ rplQ (PRK05591)Ribosomal_L17 (pfam01196)→ PseudoU_synth_EcTruA (cd02570)truA (PRK00021)→ hypo→
Ssp-56-1	NZ_AZWL01000015.1	-	17420	17364	**RNA**→ rplQ (PRK05591)Ribosomal_L17 (pfam01196)→ PseudoU_synth_EcTruA (cd02570)truA (PRK00021)→
Hcr-1-1	NZ_JODQ01000002.1	-	560025	559979	**RNA**→ rplQ (PRK05591)Ribosomal_L17 (pfam01196)→ PseudoU_synth_EcTruA (cd02570)truA (PRK00021)→
Msp-1-1	NZ_KI866524.1	-	954571	954521	**RNA**→ rplQ (PRK05591)Ribosomal_L17 (pfam01196)→ PseudoU_synth_EcTruA (cd02570)truA (PRK00021)→
Mro-1-1	NZ_JNZQ01000013.1	+	26247	26297	**RNA**→ rplQ (PRK05591)Ribosomal_L17 (pfam01196)→ PseudoU_synth_EcTruA (cd02570)truA (PRK00021)→
Ssp-171-1	NZ_JOGB01000027.1	-	2040	1986	**RNA**→ rplQ (PRK05591)Ribosomal_L17 (pfam01196)→ PseudoU_synth_EcTruA (cd02570)truA (PRK00021)→
Ssp-43-1	NZ_JH941042.1	+	296021	296076	**RNA**→ rplQ (PRK05591)Ribosomal_L17 (pfam01196)→ PseudoU_synth_EcTruA (cd02570)truA (PRK00021)→
Svi-13-1	NZ_LGUP01000370.1	-	89952	89897	**RNA**→ rplQ (PRK05591)Ribosomal_L17 (pfam01196)→ PseudoU_synth_EcTruA (cd02570)truA (PRK00021)→
Swe-1-1	NZ_JNWK01000006.1	-	89924	89869	**RNA**→ rplQ (PRK05591)Ribosomal_L17 (pfam01196)→ PseudoU_synth_EcTruA (cd02570)truA (PRK00021)→
Ssp-73-1	NZ_LACL01000017.1	+	268699	268754	**RNA**→ rplQ (PRK05591)Ribosomal_L17 (pfam01196)→
Ssc-3-1	NZ_KL997422.1	+	626325	626380	**RNA**→ rplQ (PRK05591)Ribosomal_L17 (pfam01196)→
Kau-1-1	NZ_JODU01000011.1	-	303136	303081	**RNA**→ rplQ (PRK05591)Ribosomal_L17 (pfam01196)→
Sgr-1-1	NZ_AEYX01000036.1	-	159801	159746	**RNA**→ rplQ (PRK05591)Ribosomal_L17 (pfam01196)→ rplQ (PRK05591)Ribosomal_L17 (pfam01196)→ PseudoU_synth_EcTruA (cd02570)truA (PRK00021)→ PseudoU_synth_EcTruA (cd02570)truA (PRK00021)→
Sto-1-1	NZ_JFCB01000003.1	+	275848	275903	**RNA**→ rplQ (PRK05591)Ribosomal_L17 (pfam01196)→
Ssp-114-1	NZ_JOHW01000009.1	-	153796	153741	**RNA**→ rplQ (PRK05591)Ribosomal_L17 (pfam01196)→ PseudoU_synth_EcTruA (cd02570)truA (PRK00021)→
Ssc-4-1	NZ_JODX01000002.1	+	54572	54627	**RNA**→ rplQ (PRK05591)Ribosomal_L17 (pfam01196)→ PseudoU_synth_EcTruA (cd02570)truA (PRK00021)→
Ssp-1-1	NZ_KB911732.1	-	106280	106225	**RNA**→ rplQ (PRK05591)Ribosomal_L17 (pfam01196)→
Sau-4-1	NZ_KL647039.1	-	228013	227958	**RNA**→ rplQ (PRK05591)Ribosomal_L17 (pfam01196)→ PseudoU_synth_EcTruA (cd02570)truA (PRK00021)→
Spr-1-1	NZ_CM000950.1	-	3302692	3302637	**RNA**→ hypo→ rplQ (PRK05591)Ribosomal_L17 (pfam01196)→ hypo→ PseudoU_synth_EcTruA (cd02570)truA (PRK00021)→
Ssp-189-1	NZ_JDUZ01000002.1	+	233594	233648	**RNA**→ rplQ (PRK05591)Ribosomal_L17 (pfam01196)→ PseudoU_synth_EcTruA (cd02570)truA (PRK00021)→ AAA (smart00382)Uup (COG0488)AAA_21 (pfam13304)→ DUF2029 (pfam09594)→
Sfl-6-1	NZ_JNXV01000034.1	+	16887	16941	**RNA**→ rplQ (PRK05591)Ribosomal_L17 (pfam01196)→
Ssp-103-1	NZ_JOFX01000039.1	+	152601	152655	**RNA**→ rplQ (PRK05591)Ribosomal_L17 (pfam01196)→
Ssp-111-1	NZ_JYJI01000223.1	+	17460	17514	**RNA**→ rplQ (PRK05591)Ribosomal_L17 (pfam01196)→
Ssp-159-1	NZ_JOBX01000009.1	+	138190	138244	**RNA**→ rplQ (PRK05591)Ribosomal_L17 (pfam01196)→
Ser-1-1	NZ_JNZG01000024.1	+	137960	138014	**RNA**→ rplQ (PRK05591)Ribosomal_L17 (pfam01196)→ PseudoU_synth_EcTruA (cd02570)truA (PRK00021)→ AAA (smart00382)Uup (COG0488)AAA_21 (pfam13304)→ DUF2029 (pfam09594)→
Sya-1-1	NZ_LBMK01000005.1	-	318921	318867	**RNA**→ rplQ (PRK05591)Ribosomal_L17 (pfam01196)→
Svi-12-1	NZ_LGUV01000155.1	-	24360	24306	**RNA**→ rplQ (PRK05591)Ribosomal_L17 (pfam01196)→ PseudoU_synth_EcTruA (cd02570)truA (PRK00021)→ AAA (smart00382)Uup (COG0488)AAA_21 (pfam13304)→ DUF2029 (pfam09594)→
Ssp-151-1	NZ_JOCY01000032.1	-	24441	24387	**RNA**→ rplQ (PRK05591)Ribosomal_L17 (pfam01196)→ PseudoU_synth_EcTruA (cd02570)truA (PRK00021)→ AAA (smart00382)Uup (COG0488)AAA_21 (pfam13304)→ DUF2029 (pfam09594)→
Ssp-110-1	NZ_JOFY01000047.1	-	27927	27873	**RNA**→ rplQ (PRK05591)Ribosomal_L17 (pfam01196)→ PseudoU_synth_EcTruA (cd02570)truA (PRK00021)→ AAA (smart00382)Uup (COG0488)AAA_21 (pfam13304)→ DUF2029 (pfam09594)→
Ssp-112-1	NZ_JOIB01000007.1	+	127472	127526	**RNA**→ rplQ (PRK05591)Ribosomal_L17 (pfam01196)→ PseudoU_synth_EcTruA (cd02570)truA (PRK00021)→ AAA (smart00382)Uup (COG0488)AAA_21 (pfam13304)→ DUF2029 (pfam09594)→
Ssp-102-1	NZ_JOIR01000010.1	+	206080	206134	**RNA**→ rplQ (PRK05591)Ribosomal_L17 (pfam01196)→ PseudoU_synth_EcTruA (cd02570)truA (PRK00021)→ AAA (smart00382)Uup (COG0488)AAA_21 (pfam13304)→ DUF2029 (pfam09594)→
Sla-2-1	NZ_JOBU01000029.1	+	79242	79296	**RNA**→ rplQ (PRK05591)Ribosomal_L17 (pfam01196)→
Ssp-92-1	NZ_JOHF01000013.1	+	131540	131594	**RNA**→ rplQ (PRK05591)Ribosomal_L17 (pfam01196)→
Ssp-139-1	NZ_JZWW01000181.1	-	23883	23829	**RNA**→ rplQ (PRK05591)Ribosomal_L17 (pfam01196)→
Ssp-173-1	NZ_JOJD01000018.1	-	24958	24904	**RNA**→ rplQ (PRK05591)Ribosomal_L17 (pfam01196)→

Svi-12-2	NZ_JOAK01000021.1	-	24986	24932	**RNA**→ rplQ (PRK05591)Ribosomal_L17 (pfam01196)→
Ssp-8-1	NZ_GG657750.1	+	4667597	4667651	**RNA**→ rplQ (PRK05591)Ribosomal_L17 (pfam01196)→ rplQ (PRK05591)Ribosomal_L17 (pfam01196)→
Svi-7-1	NZ_CP010407.1	+	5084066	5084122	**RNA**→ rplQ (PRK05591)Ribosomal_L17 (pfam01196)→ PseudoU_synth_EcTruA (cd02570)truA (PRK00021)→
Sgr-12-1	NZ_JNZT01000074.1	-	4616	4560	**RNA**→ rplQ (PRK05591)Ribosomal_L17 (pfam01196)→ PseudoU_synth_EcTruA (cd02570)truA (PRK00021)→
Sgr-12-2	NZ_JOEN01000002.1	-	181210	181154	**RNA**→ rplQ (PRK05591)Ribosomal_L17 (pfam01196)→ PseudoU_synth_EcTruA (cd02570)truA (PRK00021)→
Sfl-5-1	NZ_JNZO01000012.1	-	190895	190839	**RNA**→ rplQ (PRK05591)Ribosomal_L17 (pfam01196)→ PseudoU_synth_EcTruA (cd02570)truA (PRK00021)→
Sbi-3-1	NZ_JNWL01000008.1	+	233987	234043	**RNA**→ rplQ (PRK05591)Ribosomal_L17 (pfam01196)→ PseudoU_synth_EcTruA (cd02570)truA (PRK00021)→
Sbi-3-2	NZ_JOAU01000005.1	+	321503	321559	**RNA**→ rplQ (PRK05591)Ribosomal_L17 (pfam01196)→ PseudoU_synth_EcTruA (cd02570)truA (PRK00021)→
Sbi-3-3	NZ_JNXJ01000008.1	+	234102	234158	**RNA**→ rplQ (PRK05591)Ribosomal_L17 (pfam01196)→ PseudoU_synth_EcTruA (cd02570)truA (PRK00021)→
Sex-1-1	NZ_KL647056.1	+	327108	327164	**RNA**→ rplQ (PRK05591)Ribosomal_L17 (pfam01196)→ PseudoU_synth_EcTruA (cd02570)truA (PRK00021)→
Sgl-3-1	NZ_JODW01000006.1	-	235779	235723	**RNA**→ rplQ (PRK05591)Ribosomal_L17 (pfam01196)→ PseudoU_synth_EcTruA (cd02570)truA (PRK00021)→
Sgr-9-1	NZ_JOBE01000026.1	+	78758	78814	**RNA**→ rplQ (PRK05591)Ribosomal_L17 (pfam01196)→ PseudoU_synth_EcTruA (cd02570)truA (PRK00021)→
Ssp-121-1	NZ_JOHZ01000078.1	+	27510	27566	**RNA**→ rplQ (PRK05591)Ribosomal_L17 (pfam01196)→ PseudoU_synth_EcTruA (cd02570)truA (PRK00021)→
Sve-1-1	NC_018750.1	+	4750596	4750652	**RNA**→ rplQ (PRK05591)Ribosomal_L17 (pfam01196)→ PseudoU_synth_EcTruA (cd02570)truA (PRK00021)→
Sav-2-1	NZ_JZJK01000077.1	-	121007	120951	**RNA**→ rplQ (PRK05591)Ribosomal_L17 (pfam01196)→ PseudoU_synth_EcTruA (cd02570)truA (PRK00021)→
Sav-1-1	NC_003155.4	+	6003039	6003095	**RNA**→ rplQ (PRK05591)Ribosomal_L17 (pfam01196)→ PseudoU_synth_EcTruA (cd02570)truA (PRK00021)→
Sav-2-2	NZ_BAVY01000021.1	-	121478	121422	**RNA**→ rplQ (PRK05591)Ribosomal_L17 (pfam01196)→ PseudoU_synth_EcTruA (cd02570)truA (PRK00021)→
Mgl-1-1	NZ_JOFO01000009.1	+	79442	79498	**RNA**→ rplQ (PRK05591)Ribosomal_L17 (pfam01196)→ PseudoU_synth_EcTruA (cd02570)truA (PRK00021)→
Asp-1-1	NZ_JOGW01000014.1	-	37012	36956	**RNA**→ rplQ (PRK05591)Ribosomal_L17 (pfam01196)→
Stu-1-1	NZ_AEJB01000201.1	-	20150	20094	**RNA**→ rplQ (PRK05591)Ribosomal_L17 (pfam01196)→ PseudoU_synth_EcTruA (cd02570)truA (PRK00021)→
Sye-2-1	NZ_JNWJ01000097.1	+	561	617	**RNA**→ rplQ (PRK05591)Ribosomal_L17 (pfam01196)→ PseudoU_synth_EcTruA (cd02570)truA (PRK00021)→
Sdu-1-1	NZ_JNXR01000027.1	+	434	490	**RNA**→ rplQ (PRK05591)Ribosomal_L17 (pfam01196)→ PseudoU_synth_EcTruA (cd02570)truA (PRK00021)→
Sgh-1-1	NZ_DS999641.1	-	3242980	3242924	**RNA**→ rplQ (PRK05591)Ribosomal_L17 (pfam01196)→ rplQ (PRK05591)Ribosomal_L17 (pfam01196)→ PseudoU_synth_EcTruA (cd02570)truA (PRK00021)→ PseudoU_synth_EcTruA (cd02570)truA (PRK00021)→
Svi-5-1	NZ_JH993790.1	-	1541255	1541199	**RNA**→ rplQ (PRK05591)Ribosomal_L17 (pfam01196)→ PseudoU_synth_EcTruA (cd02570)truA (PRK00021)→
Ssp-153-1	NZ_JOCB01000058.1	+	23941	23997	**RNA**→ rplQ (PRK05591)Ribosomal_L17 (pfam01196)→ PseudoU_synth_EcTruA (cd02570)truA (PRK00021)→
Ssp-86-1	NZ_LAVA01000018.1	+	78943	78999	**RNA**→ rplQ (PRK05591)Ribosomal_L17 (pfam01196)→ PseudoU_synth_EcTruA (cd02570)truA (PRK00021)→
Sro-1-1	NZ_CM002285.1	-	3697722	3697666	**RNA**→ rplQ (PRK05591)Ribosomal_L17 (pfam01196)→ PseudoU_synth_EcTruA (cd02570)truA (PRK00021)→
Sgr-7-1	NZ_JOFU01000024.1	+	46860	46916	**RNA**→ rplQ (PRK05591)Ribosomal_L17 (pfam01196)→
Ssp-28-1	NZ_KB891987.1	+	241284	241340	**RNA**→ rplQ (PRK05591)Ribosomal_L17 (pfam01196)→ PseudoU_synth_EcTruA (cd02570)truA (PRK00021)→
Ssp-176-1	NZ_JOCA01000010.1	+	82614	82670	**RNA**→ rplQ (PRK05591)Ribosomal_L17 (pfam01196)→
Ssp-14-1	NZ_KB897780.1	-	288156	288100	**RNA**→ rplQ (PRK05591)Ribosomal_L17 (pfam01196)→
Sac-2-1	NZ_JODT01000001.1	-	203810	203754	**RNA**→ rplQ (PRK05591)Ribosomal_L17 (pfam01196)→ PseudoU_synth_EcTruA (cd02570)truA (PRK00021)→
Sbo-1-1	NZ_KB405078.1	-	1073332	1073276	**RNA**→ rplQ (PRK05591)Ribosomal_L17 (pfam01196)→
Sbo-1-2	NZ_KB911581.1	+	1052469	1052525	**RNA**→ rplQ (PRK05591)Ribosomal_L17 (pfam01196)→
Ssp-2-1	NZ_KB911779.1	-	228049	227993	**RNA**→ rplQ (PRK05591)Ribosomal_L17 (pfam01196)→ PseudoU_synth_EcTruA (cd02570)truA (PRK00021)→
Ssp-98-1	NZ_JOGP01000006.1	-	287417	287361	**RNA**→ rplQ (PRK05591)Ribosomal_L17 (pfam01196)→
Sac-3-1	NZ_KQ257828.1	+	111389	111445	**RNA**→ rplQ (PRK05591)Ribosomal_L17 (pfam01196)→ PseudoU_synth_EcTruA (cd02570)truA (PRK00021)→
Sac-1-1	NZ_AHBF01000045.1	-	10486	10430	**RNA**→ rplQ (PRK05591)Ribosomal_L17 (pfam01196)→ rplQ (PRK05591)Ribosomal_L17 (pfam01196)→ PseudoU_synth_EcTruA (cd02570)truA (PRK00021)→ PseudoU_synth_EcTruA (cd02570)truA (PRK00021)→
Sca-7-1	NZ_JODY01000013.1	-	8155	8099	**RNA**→ rplQ (PRK05591)Ribosomal_L17 (pfam01196)→ PseudoU_synth_EcTruA (cd02570)truA (PRK00021)→
Sgr-2-1	NZ_GG657758.1	-	3229434	3229378	**RNA**→ rplQ (PRK05591)Ribosomal_L17 (pfam01196)→ rplQ (PRK05591)Ribosomal_L17 (pfam01196)→
Ssp-105-1	NZ_JOIF01000015.1	+	61537	61593	**RNA**→ rplQ (PRK05591)Ribosomal_L17 (pfam01196)→ PseudoU_synth_EcTruA (cd02570)truA (PRK00021)→
Ssp-85-1	NZ_LBDA01000016.1	+	39978	40034	**RNA**→ rplQ (PRK05591)Ribosomal_L17 (pfam01196)→ PseudoU_synth_EcTruA (cd02570)truA (PRK00021)→
Sco-3-1	NC_021985.1	+	5098374	5098430	**RNA**→ rplQ (PRK05591)Ribosomal_L17 (pfam01196)→ PseudoU_synth_EcTruA (cd02570)truA (PRK00021)→
Sse-1-1	NZ_JNXP01000008.1	-	210569	210513	**RNA**→ rplQ (PRK05591)Ribosomal_L17 (pfam01196)→ PseudoU_synth_EcTruA (cd02570)truA (PRK00021)→
Sre-1-1	NZ_KL575664.1	-	76522	76466	**RNA**→ RplQ (COG0203)rplQ (PRK05591)→
Spe-1-1	NZ_KL575016.1	+	244282	244340	**RNA**→ rplQ (PRK05591)Ribosomal_L17 (pfam01196)→
Sbi-2-1	NZ_JOFS01000017.1	-	10150	10092	**RNA**→ rplQ (PRK05591)Ribosomal_L17 (pfam01196)→
Sbi-2-2	NZ_JOFR01000006.1	-	10415	10357	**RNA**→ rplQ (PRK05591)Ribosomal_L17 (pfam01196)→
Ssp-83-1	NZ_JUIG01000050.1	-	40198	40142	**RNA**→ rplQ (PRK05591)Ribosomal_L17 (pfam01196)→ PseudoU_synth_EcTruA (cd02570)truA (PRK00021)→
Spl-1-1	NZ_JTDH01000075.1	-	3887	3831	**RNA**→ rplQ (PRK05591)Ribosomal_L17 (pfam01196)→ PseudoU_synth_EcTruA (cd02570)truA (PRK00021)→
Spl-1-2	NZ_JUIF01000014.1	-	27221	27165	**RNA**→ rplQ (PRK05591)Ribosomal_L17 (pfam01196)→ PseudoU_synth_EcTruA (cd02570)truA (PRK00021)→

Sno-1-1	NZ_CP009313.1	+	4387573	4387629	RNA→ rplQ (PRK05591)Ribosomal_L17 (pfam01196)→
San-1-1	NZ_LGUX01000327.1	+	17957	18013	RNA→ rplQ (PRK05591)Ribosomal_L17 (pfam01196)→ PseudoU_synth_EcTruA (cd02570)truA (PRK00021)→
Sfl-3-1	NZ_JOCU01000004.1	+	188839	188895	RNA→ rplQ (PRK05591)Ribosomal_L17 (pfam01196)→ PseudoU_synth_EcTruA (cd02570)truA (PRK00021)→
Ssp-109-1	NZ_JOIC01000006.1	+	63381	63437	RNA→ rplQ (PRK05591)Ribosomal_L17 (pfam01196)→ PseudoU_synth_EcTruA (cd02570)truA (PRK00021)→
Sfl-2-1	NZ_JNWV01000004.1	+	188944	189000	RNA→ rplQ (PRK05591)Ribosomal_L17 (pfam01196)→ PseudoU_synth_EcTruA (cd02570)truA (PRK00021)→
Sla-3-1	NZ_JOBD01000005.1	+	187241	187297	RNA→ rplQ (PRK05591)Ribosomal_L17 (pfam01196)→ PseudoU_synth_EcTruA (cd02570)truA (PRK00021)→
Ssp-74-1	NZ_KI601353.1	-	672230	672174	RNA→ rplQ (PRK05591)Ribosomal_L17 (pfam01196)→ PseudoU_synth_EcTruA (cd02570)truA (PRK00021)→
Ssp-162-1	NZ_KL585947.1	+	251029	251085	RNA→ rplQ (PRK05591)Ribosomal_L17 (pfam01196)→
Ssp-164-1	NZ_KL585169.1	-	174791	174735	RNA→ rplQ (PRK05591)Ribosomal_L17 (pfam01196)→
Ssp-161-1	NZ_JOCT01000004.1	-	557487	557431	RNA→ rplQ (PRK05591)Ribosomal_L17 (pfam01196)→
Sgl-2-1	NZ_CP009438.1	+	4600285	4600341	RNA→ rplQ (PRK05591)Ribosomal_L17 (pfam01196)→
Ssp-143-1	NZ_JOAW01000083.1	+	21714	21772	RNA→ rplQ (PRK05591)Ribosomal_L17 (pfam01196)→
Ssp-36-1	NZ_KB892158.1	+	80443	80499	RNA→ rplQ (PRK05591)Ribosomal_L17 (pfam01196)→
Ssp-196-1	NZ_KK106988.1	+	235730	235786	RNA→ rplQ (PRK05591)Ribosomal_L17 (pfam01196)→
Ssp-200-1	NZ_JXWX01000073.1	-	12207	12151	RNA→ rplQ (PRK05591)Ribosomal_L17 (pfam01196)→
Sli-2-1	NZ_GG657756.1	-	3314931	3314875	RNA→ rplQ (PRK05591)Ribosomal_L17 (pfam01196)→ rplQ (PRK05591)Ribosomal_L17 (pfam01196)→
Sli-1-1	NZ_CM001889.1	+	5192600	5192656	RNA→ rplQ (PRK05591)Ribosomal_L17 (pfam01196)→
Sli-2-2	NZ_CP009124.1	-	3347250	3347194	RNA→ rplQ (PRK05591)Ribosomal_L17 (pfam01196)→
Sco-2-1	NC_003888.3	+	5143469	5143525	RNA→ rplQ (PRK05591)Ribosomal_L17 (pfam01196)→
Svi-9-1	NZ_JOCE01000019.1	-	83006	82950	RNA→ rplQ (PRK05591)Ribosomal_L17 (pfam01196)→
Sco-1-1	NZ_AHGS01001102.1	+	34290	34346	RNA→ rplQ (PRK05591)Ribosomal_L17 (pfam01196)→
Ssp-128-1	NZ_JOGV01000002.1	+	114246	114302	RNA→ rplQ (PRK05591)Ribosomal_L17 (pfam01196)→
Sol-2-1	NZ_JOFH01000023.1	-	178524	178468	RNA→ rplQ (PRK05591)Ribosomal_L17 (pfam01196)→ PseudoU_synth_EcTruA (cd02570)truA (PRK00021)→
Ssp-72-1	NZ_APIV01000001.1	-	622171	622115	RNA→ rplQ (PRK05591)Ribosomal_L17 (pfam01196)→ PseudoU_synth_EcTruA (cd02570)truA (PRK00021)→
Ssp-124-1	NZ_JOHJ01000006.1	+	93024	93080	RNA→ rplQ (PRK05591)Ribosomal_L17 (pfam01196)→
Ssp-147-1	NZ_JOGT01000009.1	-	100768	100712	RNA→ rplQ (PRK05591)Ribosomal_L17 (pfam01196)→
Ssp-126-1	NZ_JOGJ01000017.1	+	292846	292902	RNA→ rplQ (PRK05591)Ribosomal_L17 (pfam01196)→
Sce-2-1	NZ_JOEV01000018.1	-	142923	142867	RNA→ rplQ (PRK05591)Ribosomal_L17 (pfam01196)→ PseudoU_synth_EcTruA (cd02570)truA (PRK00021)→
Smo-1-1	NZ_AORZ01000051.1	-	35230	35174	RNA→ rplQ (PRK05591)Ribosomal_L17 (pfam01196)→ PseudoU_synth_EcTruA (cd02570)truA (PRK00021)→
Svi-9-2	NZ_JODI01000022.1	+	100216	100272	RNA→ rplQ (PRK05591)Ribosomal_L17 (pfam01196)→ PseudoU_synth_EcTruA (cd02570)truA (PRK00021)→
Sso-1-1	NZ_AJJM01000095.1	-	4025	3969	RNA→ rplQ (PRK05591)Ribosomal_L17 (pfam01196)→ rplQ (PRK05591)Ribosomal_L17 (pfam01196)→
Soc-1-1	NZ_JOAX01000002.1	+	178274	178330	RNA→ rplQ (PRK05591)Ribosomal_L17 (pfam01196)→ PseudoU_synth_EcTruA (cd02570)truA (PRK00021)→
Sni-2-1	NZ_JOFQ01000004.1	-	303573	303517	RNA→ rplQ (PRK05591)Ribosomal_L17 (pfam01196)→ PseudoU_synth_EcTruA (cd02570)truA (PRK00021)→
Svi-11-1	NZ_JOBH01000014.1	+	179111	179167	RNA→ rplQ (PRK05591)Ribosomal_L17 (pfam01196)→ PseudoU_synth_EcTruA (cd02570)truA (PRK00021)→
Sex-2-1	NZ_AZSS01000011.1	+	62972	63028	RNA→ rplQ (PRK05591)Ribosomal_L17 (pfam01196)→ PseudoU_synth_EcTruA (cd02570)truA (PRK00021)→
Ssp-6-1	NZ_KB912947.1	-	14396	14340	RNA→ rplQ (PRK05591)Ribosomal_L17 (pfam01196)→ PseudoU_synth_EcTruA (cd02570)truA (PRK00021)→
Ssp-101-1	NZ_JOFW01000029.1	-	52272	52216	RNA→ rplQ (PRK05591)Ribosomal_L17 (pfam01196)→
Ssp-49-1	NZ_GG753626.1	+	3590861	3590917	RNA→ rplQ (PRK05591)Ribosomal_L17 (pfam01196)→ rplQ (PRK05591)Ribosomal_L17 (pfam01196)→ PseudoU_synth_EcTruA (cd02570)truA (PRK00021)→ PseudoU_synth_EcTruA (cd02570)truA (PRK00021)→
Sal-11-1	NZ_CP010519.1	+	5393200	5393256	RNA→ rplQ (PRK05591)Ribosomal_L17 (pfam01196)→
Shy-2-1	NC_017765.1	+	6630461	6630517	RNA→ rplQ (PRK05591)Ribosomal_L17 (pfam01196)→ PseudoU_synth_EcTruA (cd02570)truA (PRK00021)→
Shy-3-1	NC_020895.1	+	6324153	6324209	RNA→ rplQ (PRK05591)Ribosomal_L17 (pfam01196)→ PseudoU_synth_EcTruA (cd02570)truA (PRK00021)→
Ssp-46-1	NZ_AGNH01000472.1	-	11085	11029	RNA→ rplQ (PRK05591)Ribosomal_L17 (pfam01196)→
Ssp-187-1	NZ_JOCG01000011.1	+	26447	26503	RNA→ rplQ (PRK05591)Ribosomal_L17 (pfam01196)→
Ssp-156-1	NZ_JOIY01000026.1	+	57550	57606	RNA→ rplQ (PRK05591)Ribosomal_L17 (pfam01196)→
Sgr-10-1	NZ_KL503830.1	+	4506022	4506078	RNA→ rplQ (PRK05591)Ribosomal_L17 (pfam01196)→
Aac-1-1	NZ_JNYX01000001.1	-	551094	551038	RNA→ rplQ (PRK05591)Ribosomal_L17 (pfam01196)→
Ssp-197-1	NZ_JMLN01000037.1	+	22969	23025	RNA→ rplQ (PRK05591)Ribosomal_L17 (pfam01196)→
Ssp-20-1	NZ_KB891532.1	+	21043	21099	RNA→ rplQ (PRK05591)Ribosomal_L17 (pfam01196)→
Ssp-123-1	NZ_JOHL01000062.1	-	14420	14364	RNA→ rplQ (PRK05591)Ribosomal_L17 (pfam01196)→
Ssp-141-1	NZ_JOHU01000049.1	-	16428	16372	RNA→ rplQ (PRK05591)Ribosomal_L17 (pfam01196)→
Sga-1-1	NZ_AOHP01000041.1	-	49677	49621	RNA→ rplQ (PRK05591)Ribosomal_L17 (pfam01196)→

Ssp-81-1	NZ_LARV01000087.1	+	36996	37052	**RNA**→ rplQ (PRK05591)Ribosomal_L17 (pfam01196)→
Ssp-113-1	NZ_KL591910.1	+	68579	68635	**RNA**→ rplQ (PRK05591)Ribosomal_L17 (pfam01196)→
Ssp-57-1	NZ_AZUS01000002.1	+	290899	290955	**RNA**→ rplQ (PRK05591)Ribosomal_L17 (pfam01196)→
Ssp-149-1	NZ_JOIV01000008.1	+	53324	53380	**RNA**→ rplQ (PRK05591)Ribosomal_L17 (pfam01196)→ PseudoU_synth_EcTruA (cd02570)truA (PRK00021)→
Ssp-178-1	NZ_JOJG01000009.1	-	357423	357367	**RNA**→ rplQ (PRK05591)Ribosomal_L17 (pfam01196)→ PseudoU_synth_EcTruA (cd02570)truA (PRK00021)→
Sah-1-1	NZ_JXYI02000085.1	+	175249	175305	**RNA**→ rplQ (PRK05591)Ribosomal_L17 (pfam01196)→ PseudoU_synth_EcTruA (cd02570)truA (PRK00021)→
Sal-8-1	NZ_CP007574.1	-	4322438	4322382	**RNA**→ rplQ (PRK05591)Ribosomal_L17 (pfam01196)→ PseudoU_synth_EcTruA (cd02570)truA (PRK00021)→
Sal-10-1	NZ_CP006871.1	-	4620900	4620844	**RNA**→ rplQ (PRK05591)Ribosomal_L17 (pfam01196)→ PseudoU_synth_EcTruA (cd02570)truA (PRK00021)→
Sal-1-1	NZ_AROY02000047.1	+	56170	56226	**RNA**→ rplQ (PRK05591)Ribosomal_L17 (pfam01196)→ PseudoU_synth_EcTruA (cd02570)truA (PRK00021)→
Sal-9-1	NZ_AXDB02000042.1	-	10629	10573	**RNA**→ rplQ (PRK05591)Ribosomal_L17 (pfam01196)→ PseudoU_synth_EcTruA (cd02570)truA (PRK00021)→
Svi-10-1	NZ_JODM01000001.1	+	289559	289615	**RNA**→ rplQ (PRK05591)Ribosomal_L17 (pfam01196)→
Sch-1-1	NZ_JH164842.1	-	142738	142680	**RNA**→ rplQ (PRK05591)Ribosomal_L17 (pfam01196)→
Sia-1-1	NZ_JNXI01000009.1	-	19967	19909	**RNA**→ rplQ (PRK05591)Ribosomal_L17 (pfam01196)→
Saf-1-1	NZ_KE354180.1	-	5829	5771	**RNA**→ rplQ (PRK05591)Ribosomal_L17 (pfam01196)→
Ssp-89-1	NZ_BBNN01000017.1	+	134818	134874	**RNA**→ rplQ (PRK05591)Ribosomal_L17 (pfam01196)→
Sva-1-1	NZ_JYJH01000002.1	+	426	482	**RNA**→ rplQ (PRK05591)Ribosomal_L17 (pfam01196)→
Sfl-7-1	NZ_JNXD01000002.1	-	531073	531017	**RNA**→ rplQ (PRK05591)Ribosomal_L17 (pfam01196)→
Ssp-158-1	NZ_JOIZ01000007.1	+	222840	222896	**RNA**→ rplQ (PRK05591)Ribosomal_L17 (pfam01196)→
Sro-5-1	NZ_JOFL01000019.1	-	115762	115706	**RNA**→ rplQ (PRK05591)Ribosomal_L17 (pfam01196)→
Ssp-116-1	NZ_JOHV01000022.1	-	72383	72327	**RNA**→ rplQ (PRK05591)Ribosomal_L17 (pfam01196)→ PseudoU_synth_EcTruA (cd02570)truA (PRK00021)→
env-22	JGI25153J46596_10079674	+	416	472	**RNA**→ rplQ (PRK05591)Ribosomal_L17 (pfam01196)→
Sam-1-1	NZ_CP012382.1	+	4777985	4778041	**RNA**→ rplQ (PRK05591)Ribosomal_L17 (pfam01196)→ PseudoU_synth_EcTruA (cd02570)truA (PRK00021)→
Ssp-51-1	NZ_AUKV01000055.1	+	421	477	**RNA**→ rplQ (PRK05591)Ribosomal_L17 (pfam01196)→ PseudoU_synth_EcTruA (cd02570)truA (PRK00021)→
Slu-1-1	NZ_KN039947.1	-	2174179	2174123	**RNA**→ rplQ (PRK05591)Ribosomal_L17 (pfam01196)→ PseudoU_synth_EcTruA (cd02570)truA (PRK00021)→
Sna-2-1	NZ_JRKI01000063.1	+	196029	196085	**RNA**→ rplQ (PRK05591)Ribosomal_L17 (pfam01196)→
Sna-1-1	NZ_KL570007.1	-	55047	54991	**RNA**→ rplQ (PRK05591)Ribosomal_L17 (pfam01196)→
Ssp-75-1	NZ_LDZX01000014.1	-	12861	12805	**RNA**→ rplQ (PRK05591)Ribosomal_L17 (pfam01196)→
env-23	JGI12419J13241_1019540	-	599	543	**RNA**→ rplQ (PRK05591)Ribosomal_L17 (pfam01196)→
Ssp-182-1	NZ_JOJH01000005.1	-	219931	219875	**RNA**→ rplQ (PRK05591)Ribosomal_L17 (pfam01196)→ PseudoU_synth_EcTruA (cd02570)truA (PRK00021)→
Scy-2-1	NZ_CP010849.1	+	4633351	4633407	**RNA**→ rplQ (PRK05591)Ribosomal_L17 (pfam01196)→ PseudoU_synth_EcTruA (cd02570)truA (PRK00021)→
Ssp-184-1	NZ_JNXK01000001.1	+	184828	184884	**RNA**→ rplQ (PRK05591)Ribosomal_L17 (pfam01196)→ PseudoU_synth_EcTruA (cd02570)truA (PRK00021)→
Ssp-184-2	NZ_JOCJ01000007.1	+	53530	53586	**RNA**→ rplQ (PRK05591)Ribosomal_L17 (pfam01196)→ PseudoU_synth_EcTruA (cd02570)truA (PRK00021)→
Svi-4-1	NZ_AMLP01000111.1	-	27781	27725	**RNA**→ rplQ (PRK05591)Ribosomal_L17 (pfam01196)→
Ssp-163-1	NZ_JOJB01000037.1	+	425	481	**RNA**→ rplQ (PRK05591)Ribosomal_L17 (pfam01196)→ PseudoU_synth_EcTruA (cd02570)truA (PRK00021)→
Ssp-177-1	NZ_KL579031.1	-	16656	16600	**RNA**→ rplQ (PRK05591)Ribosomal_L17 (pfam01196)→
Sol-1-1	NZ_JJOH01000017.1	+	251041	251097	**RNA**→ rplQ (PRK05591)Ribosomal_L17 (pfam01196)→
Svi-3-1	NZ_GG657757.1	+	5367559	5367615	**RNA**→ rplQ (PRK05591)Ribosomal_L17 (pfam01196)→ rplQ (PRK05591)Ribosomal_L17 (pfam01196)→
Szi-1-1	NZ_AGBF01000029.1	-	28163	28107	**RNA**→ rplQ (PRK05591)Ribosomal_L17 (pfam01196)→ rplQ (PRK05591)Ribosomal_L17 (pfam01196)→
Svi-13-2	NZ_LFNT01000154.1	+	521	577	**RNA**→ rplQ (PRK05591)Ribosomal_L17 (pfam01196)→
Svi-13-3	NZ_LGUR01000037.1	+	521	577	**RNA**→ rplQ (PRK05591)Ribosomal_L17 (pfam01196)→
Svi-13-4	NZ_LGUQ01000054.1	-	6858	6802	**RNA**→ rplQ (PRK05591)Ribosomal_L17 (pfam01196)→
Ssp-120-1	NZ_JOIA01001474.1	+	156	212	**RNA**→ rplQ (PRK05591)Ribosomal_L17 (pfam01196)→
Ssp-131-1	NZ_JOGX01000044.1	+	26042	26098	**RNA**→ rplQ (PRK05591)Ribosomal_L17 (pfam01196)→ PseudoU_synth_EcTruA (cd02570)truA (PRK00021)→
Sca-4-1	NZ_LFXA01000017.1	+	733261	733317	**RNA**→ rplQ (PRK05591)Ribosomal_L17 (pfam01196)→ PseudoU_synth_EcTruA (cd02570)truA (PRK00021)→
Sba-1-1	NZ_KI547048.1	+	192269	192328	**RNA**→ rplQ (PRK05591)Ribosomal_L17 (pfam01196)→
Ssp-65-1	NZ_AUFD01000010.1	-	185036	184977	**RNA**→ rplQ (PRK05591)Ribosomal_L17 (pfam01196)→
Sme-2-1	NZ_JODL01000029.1	-	20518	20462	**RNA**→ rplQ (PRK05591)Ribosomal_L17 (pfam01196)→ PseudoU_synth_EcTruA (cd02570)truA (PRK00021)→
Sgr-12-3	NZ_JNZI01000001.1	-	441683	441627	**RNA**→ rplQ (PRK05591)Ribosomal_L17 (pfam01196)→ PseudoU_synth_EcTruA (cd02570)truA (PRK00021)→
Sgr-12-4	NZ_JOGN01000053.1	-	20537	20481	**RNA**→ rplQ (PRK05591)Ribosomal_L17 (pfam01196)→ PseudoU_synth_EcTruA (cd02570)truA (PRK00021)→
Ssp-15-1	NZ_KB898225.1	+	40192	40248	**RNA**→ rplQ (PRK05591)Ribosomal_L17 (pfam01196)→ PseudoU_synth_EcTruA (cd02570)truA (PRK00021)→
Ssp-38-1	NZ_FR873697.1	-	1509259	1509203	**RNA**→ rplQ (PRK05591)Ribosomal_L17 (pfam01196)→ PseudoU_synth_EcTruA (cd02570)truA (PRK00021)→
Sal-5-1	NZ_JOII01000001.1	-	312744	312688	**RNA**→ rplQ (PRK05591)Ribosomal_L17 (pfam01196)→ PseudoU_synth_EcTruA (cd02570)truA (PRK00021)→

Ssp-99-1	NZ_JOGQ01000003.1	+	125525	125581	**RNA**→ rplQ (PRK05591)Ribosomal_L17 (pfam01196)→ PseudoU_synth_EcTruA (cd02570)truA (PRK00021)→
Ssp-77-1	NZ_LAYX01000002.1	-	159453	159397	**RNA**→ rplQ (PRK05591)Ribosomal_L17 (pfam01196)→ PseudoU_synth_EcTruA (cd02570)truA (PRK00021)→
Swa-1-1	NZ_JHDU01000025.1	-	87726	87670	**RNA**→ rplQ (PRK05591)Ribosomal_L17 (pfam01196)→ PseudoU_synth_EcTruA (cd02570)truA (PRK00021)→
Ssp-30-1	NZ_KB890704.1	-	981330	981274	**RNA**→ rplQ (PRK05591)Ribosomal_L17 (pfam01196)→ PseudoU_synth_EcTruA (cd02570)truA (PRK00021)→
Ssp-134-1	NZ_JOHX01000007.1	-	127009	126953	**RNA**→ rplQ (PRK05591)Ribosomal_L17 (pfam01196)→ PseudoU_synth_EcTruA (cd02570)truA (PRK00021)→
Ssp-62-1	NZ_JTCK01000085.1	+	32179	32235	**RNA**→ rplQ (PRK05591)Ribosomal_L17 (pfam01196)→ PseudoU_synth_EcTruA (cd02570)truA (PRK00021)→
Ssp-21-1	NZ_CM002271.1	+	4349395	4349451	**RNA**→ rplQ (PRK05591)Ribosomal_L17 (pfam01196)→ PseudoU_synth_EcTruA (cd02570)truA (PRK00021)→
Ssp-35-1	NZ_CM002273.1	+	4354309	4354365	**RNA**→ rplQ (PRK05591)Ribosomal_L17 (pfam01196)→ PseudoU_synth_EcTruA (cd02570)truA (PRK00021)→
Ssp-80-1	NZ_KN549146.1	+	250775	250831	**RNA**→ rplQ (PRK05591)Ribosomal_L17 (pfam01196)→ PseudoU_synth_EcTruA (cd02570)truA (PRK00021)→
Sgr-12-5	NZ_JOGU01000007.1	-	148467	148411	**RNA**→ rplQ (PRK05591)Ribosomal_L17 (pfam01196)→ PseudoU_synth_EcTruA (cd02570)truA (PRK00021)→
Sva-2-1	NZ_JOBF01000008.1	-	20052	19996	**RNA**→ rplQ (PRK05591)Ribosomal_L17 (pfam01196)→
Sva-2-2	NZ_JOFN01000040.1	-	18282	18226	**RNA**→ rplQ (PRK05591)Ribosomal_L17 (pfam01196)→
Ssp-58-1	NZ_AUKQ01000015.1	+	52320	52377	**RNA**→ rplQ (PRK05591)Ribosomal_L17 (pfam01196)→
Sal-12-1	NZ_JOID01000003.1	+	68800	68856	**RNA**→ rplQ (PRK05591)Ribosomal_L17 (pfam01196)→
Sfr-1-1	NZ_LGSP01000027.1	-	10382	10326	**RNA**→ rplQ (PRK05591)Ribosomal_L17 (pfam01196)→
Sfr-1-2	NZ_JNAD01000026.1	-	10369	10313	**RNA**→ rplQ (PRK05591)Ribosomal_L17 (pfam01196)→
Sxi-2-1	NZ_AFRP01001524.1	+	40341	40397	**RNA**→ rplQ (PRK05591)Ribosomal_L17 (pfam01196)→
Ssp-119-1	NZ_JOHR01000002.1	+	230095	230151	**RNA**→ rplQ (PRK05591)Ribosomal_L17 (pfam01196)→
Ssp-33-1	NZ_KB891885.1	-	239596	239541	**RNA**→ rplQ (PRK05591)Ribosomal_L17 (pfam01196)→ PseudoU_synth_EcTruA (cd02570)truA (PRK00021)→
Ssp-37-1	NZ_BARG01000058.1	-	624	569	**RNA**→ rplQ (PRK05591)Ribosomal_L17 (pfam01196)→
Ssp-170-1	NZ_JODG01000026.1	+	329	385	**RNA**→ rplQ (PRK05591)Ribosomal_L17 (pfam01196)→ PseudoU_synth_EcTruA (cd02570)truA (PRK00021)→ Ugd (COG1004)NDP-sugDHase (TIGR03026)→
Smi-1-1	NZ_KN050740.1	-	288662	288606	**RNA**→ rplQ (PRK05591)Ribosomal_L17 (pfam01196)→ PseudoU_synth_EcTruA (cd02570)truA (PRK00021)→
Ssp-138-1	NZ_JOIU01000016.1	+	88730	88786	**RNA**→ rplQ (PRK05591)Ribosomal_L17 (pfam01196)→ PseudoU_synth_EcTruA (cd02570)truA (PRK00021)→
Sga-2-1	NZ_JRHJ01000054.1	+	156382	156438	**RNA**→ rplQ (PRK05591)Ribosomal_L17 (pfam01196)→
env-24	JGI24740J21852_10018017	-	980	924	**RNA**→ rplQ (PRK05591)Ribosomal_L17 (pfam01196)→
env-25	JGI24739J22299_10011386	-	2321	2265	**RNA**→ rplQ (PRK05591)Ribosomal_L17 (pfam01196)→
env-26	JGI24737J22298_10030274	-	711	655	**RNA**→ rplQ (PRK05591)Ribosomal_L17 (pfam01196)→ hypo→
env-27	JGI24735J21928_10022680	+	1260	1316	**RNA**→ rplQ (PRK05591)Ribosomal_L17 (pfam01196)→
env-28	JGI24738J21930_10016389	+	901	957	**RNA**→ rplQ (PRK05591)Ribosomal_L17 (pfam01196)→
env-29	JGI24738J21930_10017841	-	666	610	**RNA**→ rplQ (PRK05591)Ribosomal_L17 (pfam01196)→
Sca-1-1	NZ_KB911586.1	+	111863	111919	**RNA**→ rplQ (PRK05591)Ribosomal_L17 (pfam01196)→ PseudoU_synth_EcTruA (cd02570)truA (PRK00021)→
Ssp-23-1	NZ_KE150428.1	+	48833	48889	**RNA**→ rplQ (PRK05591)Ribosomal_L17 (pfam01196)→ PseudoU_synth_EcTruA (cd02570)truA (PRK00021)→
Ssp-135-1	NZ_JNXF01000033.1	-	14094	14038	**RNA**→ rplQ (PRK05591)Ribosomal_L17 (pfam01196)→
Ssp-137-1	NZ_JOHP01000033.1	-	14040	13984	**RNA**→ rplQ (PRK05591)Ribosomal_L17 (pfam01196)→
Ssp-91-1	NZ_JNZW01000034.1	-	14086	14030	**RNA**→ rplQ (PRK05591)Ribosomal_L17 (pfam01196)→
Ssp-136-1	NZ_JOHS01000031.1	-	14033	13977	**RNA**→ rplQ (PRK05591)Ribosomal_L17 (pfam01196)→
Ssp-169-1	NZ_JODC01000003.1	-	760731	760675	**RNA**→ rplQ (PRK05591)Ribosomal_L17 (pfam01196)→
env-30	JGI24736J21556_1066001	+	409	465	**RNA**→
Ssv-1-1	NZ_CM000951.1	+	5617327	5617383	**RNA**→ rplQ (PRK05591)Ribosomal_L17 (pfam01196)→ hypo→ PseudoU_synth_EcTruA (cd02570)truA (PRK00021)→ hypo→
Ssp-186-1	NZ_JOBZ01000048.1	+	11344	11400	**RNA**→ rplQ (PRK05591)Ribosomal_L17 (pfam01196)→
Sda-1-1	NC_020504.1	-	4173417	4173361	**RNA**→ rplQ (PRK05591)Ribosomal_L17 (pfam01196)→ PseudoU_synth_EcTruA (cd02570)truA (PRK00021)→
Sgr-12-6	NZ_JOJE01000032.1	-	42148	42092	**RNA**→ rplQ (PRK05591)Ribosomal_L17 (pfam01196)→
Ssp-199-1	NZ_JXTE01000103.1	-	19424	19368	**RNA**→ rplQ (PRK05591)Ribosomal_L17 (pfam01196)→
env-31	JGI25160J50197_1027159	-	1290	1235	**RNA**→ rplQ (PRK05591)Ribosomal_L17 (pfam01196)→
Ssp-88-1	NZ_BBNO01000005.1	+	169561	169616	**RNA**→ rplQ (PRK05591)Ribosomal_L17 (pfam01196)→ PseudoU_synth_EcTruA (cd02570)truA (PRK00021)→
Ssp-19-1	NZ_AQWO01000012.1	-	42118	42063	**RNA**→ rplQ (PRK05591)Ribosomal_L17 (pfam01196)→ PseudoU_synth_EcTruA (cd02570)truA (PRK00021)→
Sfu-2-1	NZ_JOEY01000005.1	-	283078	283023	**RNA**→ rplQ (PRK05591)Ribosomal_L17 (pfam01196)→ PseudoU_synth_EcTruA (cd02570)truA (PRK00021)→ Glyco_hydro_26 (pfam02156)→
Sau-2-1	NZ_JH725387.1	+	3734051	3734106	**RNA**→ rplQ (PRK05591)Ribosomal_L17 (pfam01196)→
Sxy-1-1	NZ_JNWO01000013.1	+	27275	27330	**RNA**→ rplQ (PRK05591)Ribosomal_L17 (pfam01196)→
Ssp-122-1	NZ_JNXE01000005.1	+	80283	80338	**RNA**→ rplQ (PRK05591)Ribosomal_L17 (pfam01196)→

Spr-2-1	NZ_BARF01000005.1	+	85303	85358	**RNA**→ rplQ (PRK05591)Ribosomal_L17 (pfam01196)→
Ssp-84-1	NZ_LBMU01000061.1	-	29957	29901	**RNA**→ rplQ (PRK05591)Ribosomal_L17 (pfam01196)→
Sip-1-1	NZ_AEJC01000092.1	+	9205	9260	**RNA**→ rplQ (PRK05591)Ribosomal_L17 (pfam01196)→ PseudoU_synth_EcTruA (cd02570)truA (PRK00021)→
Sly-2-1	NZ_CP007699.1	+	4988677	4988732	**RNA**→ rplQ (PRK05591)Ribosomal_L17 (pfam01196)→ PseudoU_synth_EcTruA (cd02570)truA (PRK00021)→
Ssc-2-1	NZ_KB889574.1	-	55636	55581	**RNA**→ rplQ (PRK05591)Ribosomal_L17 (pfam01196)→
Sye-1-1	NZ_JQNR01000005.1	+	1956765	1956820	**RNA**→ rplQ (PRK05591)Ribosomal_L17 (pfam01196)→
Svi-6-1	NZ_KB904651.1	-	121812	121756	**RNA**→ rplQ (PRK05591)Ribosomal_L17 (pfam01196)→
Sni-1-1	NZ_CM002280.1	-	3417483	3417429	**RNA**→ rplQ (PRK05591)Ribosomal_L17 (pfam01196)→ PseudoU_synth_EcTruA (cd02570)truA (PRK00021)→
Ssu-2-1	NZ_AJTQ01000307.1	+	13549	13604	**RNA**→ RplQ (COG0203)rplQ (PRK05591)→
Ssu-1-1	NZ_KB905817.1	-	189202	189147	**RNA**→ RplQ (COG0203)rplQ (PRK05591)→ PseudoU_synth_EcTruA (cd02570)truA (PRK00021)→
Svi-2-1	NC_015957.1	+	705115	705170	**RNA**→ rplQ (PRK05591)Ribosomal_L17 (pfam01196)→ PseudoU_synth_EcTruA (cd02570)truA (PRK00021)→
Sfl-4-1	NZ_AUBE01000006.1	-	238624	238569	**RNA**→ rplQ (PRK05591)Ribosomal_L17 (pfam01196)→ PseudoU_synth_EcTruA (cd02570)truA (PRK00021)→
Spu-1-1	NZ_KB913030.1	+	4401513	4401568	**RNA**→ rplQ (PRK05591)Ribosomal_L17 (pfam01196)→ PseudoU_synth_EcTruA (cd02570)truA (PRK00021)→
Ssc-4-2	NZ_JOBC01000019.1	-	77220	77165	**RNA**→ rplQ (PRK05591)Ribosomal_L17 (pfam01196)→ PseudoU_synth_EcTruA (cd02570)truA (PRK00021)→
Shy-1-1	NZ_GG657754.1	-	4571299	4571244	**RNA**→ rplQ (PRK05591)Ribosomal_L17 (pfam01196)→ rplQ (PRK05591)Ribosomal_L17 (pfam01196)→
env-32	JGI25160J50197_1040719	-	1000	945	**RNA**→ rplQ (PRK05591)Ribosomal_L17 (pfam01196)→ truA (PRK00021)PseudoU_synth_1 (pfam01416)→
Sbi-1-1	NC_016582.1	+	7517266	7517321	**RNA**→ rplQ (PRK05591)Ribosomal_L17 (pfam01196)→
Afl-1-1	NZ_KB894099.1	-	208768	208711	**RNA**→ rplQ (PRK05591)Ribosomal_L17 (pfam01196)→
Ssp-55-1	NZ_JTIY01000001.1	-	1514095	1514041	**RNA**→ rplQ (PRK05591)Ribosomal_L17 (pfam01196)→
Ssp-152-1	NZ_JOCX01000005.1	+	52944	52998	**RNA**→ rplQ (PRK05591)Ribosomal_L17 (pfam01196)→
Ssp-104-1	NZ_JOIS01000020.1	-	101942	101888	**RNA**→ rplQ (PRK05591)Ribosomal_L17 (pfam01196)→
Ssp-166-1	NZ_JODE01000005.1	+	46633	46687	**RNA**→ rplQ (PRK05591)Ribosomal_L17 (pfam01196)→ PseudoU_synth_EcTruA (cd02570)truA (PRK00021)→
					AAA (smart00382)Uup (COG0488)AAA_21 (pfam13304)→ DUF2029 (pfam09594)→ DUF2029 (pfam09594)→
Ssp-167-1	NZ_JODJ01000029.1	+	46652	46706	**RNA**→ rplQ (PRK05591)Ribosomal_L17 (pfam01196)→ PseudoU_synth_EcTruA (cd02570)truA (PRK00021)→
					AAA (smart00382)Uup (COG0488)AAA_21 (pfam13304)→ DUF2029 (pfam09594)→ DUF2029 (pfam09594)→
Ssp-191-1	NZ_JWZS01000804.1	+	24884	24938	**RNA**→ rplQ (PRK05591)Ribosomal_L17 (pfam01196)→ PseudoU_synth_EcTruA (cd02570)truA (PRK00021)→
					AAA (smart00382)Uup (COG0488)AAA_21 (pfam13304)→ hypo→
Sxa-1-1	NZ_JNZH01000037.1	-	123110	123056	**RNA**→ rplQ (PRK05591)Ribosomal_L17 (pfam01196)→ PseudoU_synth_EcTruA (cd02570)truA (PRK00021)→
					AAA (smart00382)Uup (COG0488)→ DUF2029 (pfam09594)→
Sxa-1-2	NZ_JOFT01000047.1	+	32685	32739	**RNA**→ rplQ (PRK05591)Ribosomal_L17 (pfam01196)→ PseudoU_synth_EcTruA (cd02570)truA (PRK00021)→
					AAA (smart00382)Uup (COG0488)→ DUF2029 (pfam09594)→
Sla-1-1	NZ_JNXL01000004.1	+	16844	16898	**RNA**→ rplQ (PRK05591)Ribosomal_L17 (pfam01196)→
Svi-12-3	NZ_JNYC01000038.1	+	47873	47927	**RNA**→ rplQ (PRK05591)Ribosomal_L17 (pfam01196)→ PseudoU_synth_EcTruA (cd02570)truA (PRK00021)→
					AAA (smart00382)Uup (COG0488)AAA_21 (pfam13304)→
Sla-2-2	NZ_JOEW01000015.1	+	48662	48716	**RNA**→ rplQ (PRK05591)Ribosomal_L17 (pfam01196)→ PseudoU_synth_EcTruA (cd02570)truA (PRK00021)→
					AAA (smart00382)Uup (COG0488)AAA_21 (pfam13304)→ DUF2029 (pfam09594)→
Ssp-150-1	NZ_JODA01000027.1	-	24120	24066	**RNA**→ rplQ (PRK05591)Ribosomal_L17 (pfam01196)→ PseudoU_synth_EcTruA (cd02570)truA (PRK00021)→
					AAA (smart00382)Uup (COG0488)AAA_21 (pfam13304)→ DUF2029 (pfam09594)→
Ssp-32-1	NZ_DS570396.1	-	78156	78102	**RNA**→ rplQ (PRK05591)Ribosomal_L17 (pfam01196)→ rplQ (PRK05591)Ribosomal_L17 (pfam01196)→
					PseudoU_synth_EcTruA (cd02570)truA (PRK00021)→ PseudoU_synth_EcTruA (cd02570)truA (PRK00021)→
					AAA (smart00382)Uup (COG0488)AAA_21 (pfam13304)→
					AAA (smart00382)Uup (COG0488)AAA_21 (pfam13304)→ DUF2029 (pfam09594)→ DUF2029 (pfam09594)→
Ssp-32-2	NZ_CP011664.1	+	4787937	4787991	**RNA**→ rplQ (PRK05591)Ribosomal_L17 (pfam01196)→ PseudoU_synth_EcTruA (cd02570)truA (PRK00021)→
					AAA (smart00382)Uup (COG0488)AAA_21 (pfam13304)→ DUF2029 (pfam09594)→
Ska-1-1	NZ_JNZY01000017.1	+	138776	138830	**RNA**→ rplQ (PRK05591)Ribosomal_L17 (pfam01196)→
Sro-6-1	NZ_LFML01000045.1	+	17342	17396	**RNA**→ rplQ (PRK05591)Ribosomal_L17 (pfam01196)→
Ssp-194-1	NZ_AUKR01000012.1	+	193841	193895	**RNA**→ rplQ (PRK05591)Ribosomal_L17 (pfam01196)→ PseudoU_synth_EcTruA (cd02570)truA (PRK00021)→
env-33	LWSO_NODE_580	+	2805	2860	**RNA**→ rplQ (PRK05591)Ribosomal_L17 (pfam01196)→ PseudoU_synth_EcTruA (cd02570)truA (PRK00021)→
Ssp-181-1	NZ_JOCD01000044.1	-	26202	26147	**RNA**→ rplQ (PRK05591)Ribosomal_L17 (pfam01196)→
Ssp-7-1	NZ_KB892128.1	+	655	710	**RNA**→ rplQ (PRK05591)Ribosomal_L17 (pfam01196)→ PseudoU_synth_EcTruA (cd02570)truA (PRK00021)→
Ssp-140-1	NZ_KL591014.1	+	86235	86290	**RNA**→ rplQ (PRK05591)Ribosomal_L17 (pfam01196)→ PseudoU_synth_EcTruA (cd02570)truA (PRK00021)→
Svi-1-1	NZ_ASHX01000001.1	-	1241638	1241583	**RNA**→ rplQ (PRK05591)Ribosomal_L17 (pfam01196)→
Sca-2-1	NC_016111.1	+	3889184	3889239	**RNA**→ rplQ (PRK05591)Ribosomal_L17 (pfam01196)→ PseudoU_synth_EcTruA (cd02570)truA (PRK00021)→
Sca-2-2	NC_017586.1	+	3888995	3889050	**RNA**→ rplQ (PRK05591)Ribosomal_L17 (pfam01196)→ PseudoU_synth_EcTruA (cd02570)truA (PRK00021)→

Sts-1-1	NZ_AJSZ01000399.1	-	24785	24730	**RNA**→ rplQ (PRK05591)Ribosomal_L17 (pfam01196)→ rplQ (PRK05591)Ribosomal_L17 (pfam01196)→
Ssp-25-1	NZ_KB891797.1	+	527129	527185	**RNA**→ rplQ (PRK05591)Ribosomal_L17 (pfam01196)→ PseudoU_synth_EcTruA (cd02570)truA (PRK00021)→
Ska-1-2	NZ_JZWV01000003.1	+	52624	52679	**RNA**→ rplQ (PRK05591)Ribosomal_L17 (pfam01196)→ PseudoU_synth_EcTruA (cd02570)truA (PRK00021)→
					AAA (smart00382)Uup (COG0488)AAA_21 (pfam13304)→ DUF2029 (pfam09594)→
Smo-2-1	NZ_JNYL01000032.1	-	28345	28290	**RNA**→ rplQ (PRK05591)Ribosomal_L17 (pfam01196)→
Ssp-70-1	NZ_KN708638.1	-	3669055	3669000	**RNA**→ rplQ (PRK05591)Ribosomal_L17 (pfam01196)→
Sba-2-1	NZ_KL573554.1	+	64146	64201	**RNA**→ rplQ (PRK05591)Ribosomal_L17 (pfam01196)→ PseudoU_synth_EcTruA (cd02570)truA (PRK00021)→
Sme-1-1	NZ_JOJK01000001.1	-	178778	178723	**RNA**→ rplQ (PRK05591)Ribosomal_L17 (pfam01196)→ PseudoU_synth_EcTruA (cd02570)truA (PRK00021)→
Sgr-11-1	NZ_JYBE01000004.1	-	64041	63986	**RNA**→ rplQ (PRK05591)Ribosomal_L17 (pfam01196)→ PseudoU_synth_EcTruA (cd02570)truA (PRK00021)→
Sgl-1-1	NZ_JH689267.1	-	288602	288547	**RNA**→ rplQ (PRK05591)Ribosomal_L17 (pfam01196)→ PseudoU_synth_EcTruA (cd02570)truA (PRK00021)→
Ssp-76-1	NZ_JPWW01000002.1	+	1014580	1014635	**RNA**→ rplQ (PRK05591)Ribosomal_L17 (pfam01196)→ PseudoU_synth_EcTruA (cd02570)truA (PRK00021)→
Sgr-12-7	NZ_JOGA01000004.1	-	151218	151163	**RNA**→ rplQ (PRK05591)Ribosomal_L17 (pfam01196)→ PseudoU_synth_EcTruA (cd02570)truA (PRK00021)→
Sgl-3-2	NZ_JNZK01000003.1	-	116076	116021	**RNA**→ rplQ (PRK05591)Ribosomal_L17 (pfam01196)→ PseudoU_synth_EcTruA (cd02570)truA (PRK00021)→
Sgr-4-1	NC_010572.1	-	3318808	3318753	**RNA**→ rplQ (PRK05591)Ribosomal_L17 (pfam01196)→ PseudoU_synth_EcTruA (cd02570)truA (PRK00021)→
San-2-1	NZ_JNXS01000008.1	+	26146	26201	**RNA**→ rplQ (PRK05591)Ribosomal_L17 (pfam01196)→ PseudoU_synth_EcTruA (cd02570)truA (PRK00021)→
San-2-2	NZ_JOEZ01000006.1	-	175251	175196	**RNA**→ rplQ (PRK05591)Ribosomal_L17 (pfam01196)→ PseudoU_synth_EcTruA (cd02570)truA (PRK00021)→
Sgr-3-1	NZ_GL877172.1	-	3511493	3511438	**RNA**→ rplQ (PRK05591)Ribosomal_L17 (pfam01196)→ rplQ (PRK05591)Ribosomal_L17 (pfam01196)→
					PseudoU_synth_EcTruA (cd02570)truA (PRK00021)→ PseudoU_synth_EcTruA (cd02570)truA (PRK00021)→
Sgr-12-8	NZ_JOBR01000006.1	+	349597	349652	**RNA**→ rplQ (PRK05591)Ribosomal_L17 (pfam01196)→ PseudoU_synth_EcTruA (cd02570)truA (PRK00021)→
Ssp-9-1	NZ_KB899005.1	+	126861	126916	**RNA**→ rplQ (PRK05591)Ribosomal_L17 (pfam01196)→ PseudoU_synth_EcTruA (cd02570)truA (PRK00021)→
Seu-1-1	NZ_KQ257782.1	-	24925	24870	**RNA**→ rplQ (PRK05591)Ribosomal_L17 (pfam01196)→ PseudoU_synth_EcTruA (cd02570)truA (PRK00021)→
Ssp-82-1	NZ_JXUQ01000001.1	+	4992228	4992283	**RNA**→ rplQ (PRK05591)Ribosomal_L17 (pfam01196)→ PseudoU_synth_EcTruA (cd02570)truA (PRK00021)→
Sal-12-2	NZ_JOED01000012.1	+	368027	368082	**RNA**→ rplQ (PRK05591)Ribosomal_L17 (pfam01196)→ PseudoU_synth_EcTruA (cd02570)truA (PRK00021)→
Ssp-48-1	NZ_AGSW01000026.1	-	4221	4166	**RNA**→ rplQ (PRK05591)Ribosomal_L17 (pfam01196)→ rplQ (PRK05591)Ribosomal_L17 (pfam01196)→
					PseudoU_synth_EcTruA (cd02570)truA (PRK00021)→ PseudoU_synth_EcTruA (cd02570)truA (PRK00021)→
Sha-1-1	NZ_JOAZ01000028.1	-	8990	8935	**RNA**→ rplQ (PRK05591)Ribosomal_L17 (pfam01196)→ PseudoU_synth_EcTruA (cd02570)truA (PRK00021)→
Sgr-8-1	NZ_JOFC01000040.1	-	9004	8949	**RNA**→ rplQ (PRK05591)Ribosomal_L17 (pfam01196)→ PseudoU_synth_EcTruA (cd02570)truA (PRK00021)→
Ssp-45-1	NC_015953.1	+	4421046	4421101	**RNA**→ rplQ (PRK05591)Ribosomal_L17 (pfam01196)→ PseudoU_synth_EcTruA (cd02570)truA (PRK00021)→
Ssp-18-1	NZ_ARPI01000009.1	+	69587	69642	**RNA**→ rplQ (PRK05591)Ribosomal_L17 (pfam01196)→ PseudoU_synth_EcTruA (cd02570)truA (PRK00021)→
Ssp-71-1	NZ_AZVE01000001.1	-	707671	707616	**RNA**→ rplQ (PRK05591)Ribosomal_L17 (pfam01196)→ PseudoU_synth_EcTruA (cd02570)truA (PRK00021)→
Ssp-175-1	NZ_KL585382.1	+	76745	76800	**RNA**→ rplQ (PRK05591)Ribosomal_L17 (pfam01196)→ PseudoU_synth_EcTruA (cd02570)truA (PRK00021)→
Ssp-188-1	NZ_JJOB01000001.1	+	3882743	3882798	**RNA**→ rplQ (PRK05591)Ribosomal_L17 (pfam01196)→ PseudoU_synth_EcTruA (cd02570)truA (PRK00021)→
Ssp-52-1	NZ_JTHL01000001.1	+	5725538	5725593	**RNA**→ rplQ (PRK05591)Ribosomal_L17 (pfam01196)→
Sri-4-1	NZ_JOCQ01000089.1	-	23248	23193	**RNA**→ rplQ (PRK05591)Ribosomal_L17 (pfam01196)→
Ssp-145-1	NZ_JOHB01000009.1	+	199860	199915	**RNA**→ rplQ (PRK05591)Ribosomal_L17 (pfam01196)→
env-34	JGI25160J50197_1029655	-	174	119	**RNA**→
Sly-1-1	NZ_JNZA01000027.1	-	104738	104683	**RNA**→ rplQ (PRK05591)Ribosomal_L17 (pfam01196)→
Ssp-142-1	NZ_JOGE01000029.1	+	58699	58754	**RNA**→ rplQ (PRK05591)Ribosomal_L17 (pfam01196)→
Ssp-155-1	NZ_JOIX01000020.1	+	81701	81756	**RNA**→ rplQ (PRK05591)Ribosomal_L17 (pfam01196)→ PseudoU_synth_EcTruA (cd02570)truA (PRK00021)→
Ssp-53-1	NZ_CP003987.1	+	5490097	5490152	**RNA**→ rplQ (PRK05591)Ribosomal_L17 (pfam01196)→ PseudoU_synth_EcTruA (cd02570)truA (PRK00021)→
Sat-2-1	NZ_JNXG01000002.1	+	464448	464503	**RNA**→ rplQ (PRK05591)Ribosomal_L17 (pfam01196)→ PseudoU_synth_EcTruA (cd02570)truA (PRK00021)→
Ssp-95-1	NZ_JOAE01000005.1	-	277533	277478	**RNA**→ rplQ (PRK05591)Ribosomal_L17 (pfam01196)→ PseudoU_synth_EcTruA (cd02570)truA (PRK00021)→
Sfl-1-1	NC_016114.1	-	2965273	2965218	**RNA**→ rplQ (PRK05591)Ribosomal_L17 (pfam01196)→ PseudoU_synth_EcTruA (cd02570)truA (PRK00021)→
Ssp-34-1	NC_021055.1	+	4462927	4462982	**RNA**→ rplQ (PRK05591)Ribosomal_L17 (pfam01196)→ PseudoU_synth_EcTruA (cd02570)truA (PRK00021)→
[pa-1-1	NZ_JNYQ01000004.1	-	851086	851031	**RNA**→ rplQ (PRK05591)Ribosomal_L17 (pfam01196)→ PseudoU_synth_EcTruA (cd02570)truA (PRK00021)→
Sol-2-2	NZ_JNWM01000003.1	+	440846	440901	**RNA**→ rplQ (PRK05591)Ribosomal_L17 (pfam01196)→ PseudoU_synth_EcTruA (cd02570)truA (PRK00021)→
Sfu-3-1	NZ_JNXH01000004.1	-	821119	821064	**RNA**→ rplQ (PRK05591)Ribosomal_L17 (pfam01196)→ PseudoU_synth_EcTruA (cd02570)truA (PRK00021)→
Sfl-8-1	NZ_JOAB01000003.1	-	795932	795877	**RNA**→ rplQ (PRK05591)Ribosomal_L17 (pfam01196)→ PseudoU_synth_EcTruA (cd02570)truA (PRK00021)→
Ssp-154-1	NZ_JOIW01000004.1	-	790730	790675	**RNA**→ rplQ (PRK05591)Ribosomal_L17 (pfam01196)→ PseudoU_synth_EcTruA (cd02570)truA (PRK00021)→
Sce-1-1	NZ_JOEL01000031.1	+	46103	46158	**RNA**→ rplQ (PRK05591)Ribosomal_L17 (pfam01196)→
Ssc-5-1	NZ_JOEI01000008.1	+	148665	148720	**RNA**→ rplQ (PRK05591)Ribosomal_L17 (pfam01196)→ PseudoU_synth_EcTruA (cd02570)truA (PRK00021)→
Ssp-144-1	NZ_KL589850.1	+	71321	71376	**RNA**→ rplQ (PRK05591)Ribosomal_L17 (pfam01196)→ PseudoU_synth_EcTruA (cd02570)truA (PRK00021)→
Ssp-54-1	NZ_CBRG010000181.1	+	9589	9644	**RNA**→ rplQ (PRK05591)Ribosomal_L17 (pfam01196)→ PseudoU_synth_EcTruA (cd02570)truA (PRK00021)→

Sgr-13-1	NZ_JOFF01000044.1	-	4277	4222	**RNA**→ rplQ (PRK05591)Ribosomal_L17 (pfam01196)→ PseudoU_synth_EcTruA (cd02570)truA (PRK00021)→
Sfl-9-1	NZ_JOAC01000001.1	+	308726	308781	**RNA**→ rplQ (PRK05591)Ribosomal_L17 (pfam01196)→ PseudoU_synth_EcTruA (cd02570)truA (PRK00021)→
Sro-4-1	NZ_ABYX02000001.1	+	4766062	4766117	**RNA**→ rplQ (PRK05591)Ribosomal_L17 (pfam01196)→ PseudoU_synth_EcTruA (cd02570)truA (PRK00021)→
Ssp-22-1	NZ_AWOQ01000620.1	+	11739	11794	**RNA**→ rplQ (PRK05591)Ribosomal_L17 (pfam01196)→
Sro-2-1	NZ_DS999644.1	+	4713512	4713567	**RNA**→ rplQ (PRK05591)Ribosomal_L17 (pfam01196)→ rplQ (PRK05591)Ribosomal_L17 (pfam01196)→ PseudoU_synth_EcTruA (cd02570)truA (PRK00021)→ PseudoU_synth_EcTruA (cd02570)truA (PRK00021)→
Sal-12-3	NZ_JNWW01000015.1	-	121666	121611	**RNA**→ rplQ (PRK05591)Ribosomal_L17 (pfam01196)→ PseudoU_synth_EcTruA (cd02570)truA (PRK00021)→
Ssp-174-1	NZ_JOCW01000027.1	+	95259	95314	**RNA**→ rplQ (PRK05591)Ribosomal_L17 (pfam01196)→ PseudoU_synth_EcTruA (cd02570)truA (PRK00021)→
Ssp-107-1	NZ_JOIP01000001.1	+	298835	298890	**RNA**→ rplQ (PRK05591)Ribosomal_L17 (pfam01196)→ PseudoU_synth_EcTruA (cd02570)truA (PRK00021)→
Ssp-108-1	NZ_JOIO01000005.1	+	89666	89721	**RNA**→ rplQ (PRK05591)Ribosomal_L17 (pfam01196)→ PseudoU_synth_EcTruA (cd02570)truA (PRK00021)→
Sgr-13-2	NZ_LGUZ01000023.1	-	4252	4197	**RNA**→ rplQ (PRK05591)Ribosomal_L17 (pfam01196)→ PseudoU_synth_EcTruA (cd02570)truA (PRK00021)→
Spu-3-1	NZ_JOBG01000005.1	-	224288	224233	**RNA**→ rplQ (PRK05591)Ribosomal_L17 (pfam01196)→ PseudoU_synth_EcTruA (cd02570)truA (PRK00021)→
Sgr-13-3	NZ_JOFE01000006.1	+	95269	95324	**RNA**→ rplQ (PRK05591)Ribosomal_L17 (pfam01196)→ PseudoU_synth_EcTruA (cd02570)truA (PRK00021)→
Sgr-13-4	NZ_JOFD01000016.1	-	125262	125207	**RNA**→ rplQ (PRK05591)Ribosomal_L17 (pfam01196)→ PseudoU_synth_EcTruA (cd02570)truA (PRK00021)→
Spu-3-2	NZ_JODK01000012.1	+	27097	27152	**RNA**→ rplQ (PRK05591)Ribosomal_L17 (pfam01196)→ PseudoU_synth_EcTruA (cd02570)truA (PRK00021)→
Sbr-1-1	NZ_JNXB01000005.1	+	80235	80290	**RNA**→ rplQ (PRK05591)Ribosomal_L17 (pfam01196)→ PseudoU_synth_EcTruA (cd02570)truA (PRK00021)→
Sca-5-1	NZ_KL573598.1	-	234310	234255	**RNA**→ rplQ (PRK05591)Ribosomal_L17 (pfam01196)→ PseudoU_synth_EcTruA (cd02570)truA (PRK00021)→
Sca-5-2	NZ_KL569913.1	+	82305	82360	**RNA**→ rplQ (PRK05591)Ribosomal_L17 (pfam01196)→ PseudoU_synth_EcTruA (cd02570)truA (PRK00021)→
Sgr-13-5	NZ_JODO01000006.1	+	95248	95303	**RNA**→ rplQ (PRK05591)Ribosomal_L17 (pfam01196)→ PseudoU_synth_EcTruA (cd02570)truA (PRK00021)→
Ssp-129-1	NZ_JOHA01000009.1	+	79533	79588	**RNA**→ rplQ (PRK05591)Ribosomal_L17 (pfam01196)→ PseudoU_synth_EcTruA (cd02570)truA (PRK00021)→
Ssp-130-1	NZ_JOHD01000004.1	+	34514	34569	**RNA**→ rplQ (PRK05591)Ribosomal_L17 (pfam01196)→ PseudoU_synth_EcTruA (cd02570)truA (PRK00021)→
Ssp-100-1	NZ_JOIH01000008.1	-	104878	104823	**RNA**→ rplQ (PRK05591)Ribosomal_L17 (pfam01196)→ PseudoU_synth_EcTruA (cd02570)truA (PRK00021)→
Spu-2-1	NZ_JOFA01000009.1	+	27215	27270	**RNA**→ rplQ (PRK05591)Ribosomal_L17 (pfam01196)→ PseudoU_synth_EcTruA (cd02570)truA (PRK00021)→
Sgr-12-9	NZ_JOIT01000002.1	+	49057	49112	**RNA**→ rplQ (PRK05591)Ribosomal_L17 (pfam01196)→ PseudoU_synth_EcTruA (cd02570)truA (PRK00021)→
Spu-2-2	NZ_JOBQ01000009.1	+	27228	27283	**RNA**→ rplQ (PRK05591)Ribosomal_L17 (pfam01196)→ PseudoU_synth_EcTruA (cd02570)truA (PRK00021)→
Svi-8-1	NZ_JNYP01000002.1	+	382717	382772	**RNA**→ rplQ (PRK05591)Ribosomal_L17 (pfam01196)→
Ssp-66-1	NZ_JNLT01000004.1	-	111420	111365	**RNA**→ rplQ (PRK05591)Ribosomal_L17 (pfam01196)→ PseudoU_synth_EcTruA (cd02570)truA (PRK00021)→
Ssp-94-1	NZ_JOHG01000019.1	-	96015	95960	**RNA**→ rplQ (PRK05591)Ribosomal_L17 (pfam01196)→ PseudoU_synth_EcTruA (cd02570)truA (PRK00021)→
Ssp-17-1	NZ_KB890938.1	+	398321	398376	**RNA**→ rplQ (PRK05591)Ribosomal_L17 (pfam01196)→ PseudoU_synth_EcTruA (cd02570)truA (PRK00021)→
Ssp-193-1	NZ_ARPF01000004.1	-	200548	200493	**RNA**→ rplQ (PRK05591)Ribosomal_L17 (pfam01196)→ PseudoU_synth_EcTruA (cd02570)truA (PRK00021)→
Sat-1-1	NZ_JQJU01000001.1	+	70024	70079	**RNA**→ rplQ (PRK05591)Ribosomal_L17 (pfam01196)→ PseudoU_synth_EcTruA (cd02570)truA (PRK00021)→
Ssp-44-1	NZ_KB891296.1	-	1243298	1243243	**RNA**→ rplQ (PRK05591)Ribosomal_L17 (pfam01196)→ PseudoU_synth_EcTruA (cd02570)truA (PRK00021)→
Scy-1-1	NZ_JOEM01000001.1	-	651664	651609	**RNA**→ rplQ (PRK05591)Ribosomal_L17 (pfam01196)→ PseudoU_synth_EcTruA (cd02570)truA (PRK00021)→
Sfu-1-1	NC_021177.1	+	4972092	4972147	**RNA**→ rplQ (PRK05591)Ribosomal_L17 (pfam01196)→ PseudoU_synth_EcTruA (cd02570)truA (PRK00021)→
Sal-7-1	NZ_JNWU01000006.1	-	157973	157918	**RNA**→ rplQ (PRK05591)Ribosomal_L17 (pfam01196)→ PseudoU_synth_EcTruA (cd02570)truA (PRK00021)→
Ssp-168-1	NZ_KL647092.1	-	163335	163280	**RNA**→ rplQ (PRK05591)Ribosomal_L17 (pfam01196)→ PseudoU_synth_EcTruA (cd02570)truA (PRK00021)→
Ssp-97-1	NZ_JOJO01000001.1	+	71929	71984	**RNA**→ rplQ (PRK05591)Ribosomal_L17 (pfam01196)→ PseudoU_synth_EcTruA (cd02570)truA (PRK00021)→
Ssp-146-1	NZ_JOGZ01000044.1	-	52593	52538	**RNA**→ rplQ (PRK05591)Ribosomal_L17 (pfam01196)→ PseudoU_synth_EcTruA (cd02570)truA (PRK00021)→
Sod-1-1	NZ_LATD01000297.1	+	18268	18323	**RNA**→ rplQ (PRK05591)Ribosomal_L17 (pfam01196)→ PseudoU_synth_EcTruA (cd02570)truA (PRK00021)→
Sri-4-2	NZ_JOBO01000012.1	+	88122	88177	**RNA**→ rplQ (PRK05591)Ribosomal_L17 (pfam01196)→ PseudoU_synth_EcTruA (cd02570)truA (PRK00021)→
Sgr-6-1	NZ_LGUW01000024.1	+	68205	68259	**RNA**→ rplQ (PRK05591)Ribosomal_L17 (pfam01196)→ PseudoU_synth_EcTruA (cd02570)truA (PRK00021)→
Sri-4-3	NZ_JOBV01000009.1	+	106610	106664	**RNA**→ rplQ (PRK05591)Ribosomal_L17 (pfam01196)→ PseudoU_synth_EcTruA (cd02570)truA (PRK00021)→
Sri-4-4	NZ_JOES01000007.1	+	106606	106660	**RNA**→ rplQ (PRK05591)Ribosomal_L17 (pfam01196)→ PseudoU_synth_EcTruA (cd02570)truA (PRK00021)→
Sri-4-5	NZ_JNWX01000006.1	+	69237	69291	**RNA**→ rplQ (PRK05591)Ribosomal_L17 (pfam01196)→ PseudoU_synth_EcTruA (cd02570)truA (PRK00021)→
Sri-2-1	NZ_JMGX01000035.1	+	49554	49609	**RNA**→ rplQ (PRK05591)Ribosomal_L17 (pfam01196)→ PseudoU_synth_EcTruA (cd02570)truA (PRK00021)→
Kau-1-2	NZ_LGUY01000192.1	+	63444	63499	**RNA**→ rplQ (PRK05591)Ribosomal_L17 (pfam01196)→ PseudoU_synth_EcTruA (cd02570)truA (PRK00021)→
Ssp-179-1	NZ_JOCC01000033.1	+	69292	69347	**RNA**→ rplQ (PRK05591)Ribosomal_L17 (pfam01196)→ PseudoU_synth_EcTruA (cd02570)truA (PRK00021)→
Sri-4-6	NZ_JOEO01000033.1	-	28104	28049	**RNA**→ rplQ (PRK05591)Ribosomal_L17 (pfam01196)→ PseudoU_synth_EcTruA (cd02570)truA (PRK00021)→
Sri-4-7	NZ_JNYK01000036.1	-	28104	28049	**RNA**→ rplQ (PRK05591)Ribosomal_L17 (pfam01196)→ PseudoU_synth_EcTruA (cd02570)truA (PRK00021)→
Sri-4-8	NZ_JOBN01000012.1	+	209341	209396	**RNA**→ rplQ (PRK05591)Ribosomal_L17 (pfam01196)→ PseudoU_synth_EcTruA (cd02570)truA (PRK00021)→
Sri-4-9	NZ_JOCL01000017.1	+	107763	107818	**RNA**→ rplQ (PRK05591)Ribosomal_L17 (pfam01196)→ PseudoU_synth_EcTruA (cd02570)truA (PRK00021)→
Sri-4-10	NZ_JOCR01000028.1	+	69299	69354	**RNA**→ rplQ (PRK05591)Ribosomal_L17 (pfam01196)→ PseudoU_synth_EcTruA (cd02570)truA (PRK00021)→

Sri-4-11	NZ_JOCO01000017.1	+	107815	107870	RNA→ rplQ (PRK05591)Ribosomal_L17 (pfam01196)→ PseudoU_synth_EcTruA (cd02570)truA (PRK00021)→
Sri-4-12	NZ_JOBS01000018.1	+	107803	107858	RNA→ rplQ (PRK05591)Ribosomal_L17 (pfam01196)→ PseudoU_synth_EcTruA (cd02570)truA (PRK00021)→
Ssp-180-1	NZ_JOCH01000032.1	+	69299	69354	RNA→ rplQ (PRK05591)Ribosomal_L17 (pfam01196)→ PseudoU_synth_EcTruA (cd02570)truA (PRK00021)→
Sca-6-1	NZ_JNWP01000055.1	+	21186	21241	RNA→ rplQ (PRK05591)Ribosomal_L17 (pfam01196)→ PseudoU_synth_EcTruA (cd02570)truA (PRK00021)→
Sca-6-2	NZ_JOFM01000031.1	+	69322	69377	RNA→ rplQ (PRK05591)Ribosomal_L17 (pfam01196)→ PseudoU_synth_EcTruA (cd02570)truA (PRK00021)→
Sri-4-13	NZ_JOBT01000154.1	-	8051	7996	RNA→ rplQ (PRK05591)Ribosomal_L17 (pfam01196)→ PseudoU_synth_EcTruA (cd02570)truA (PRK00021)→
Sri-4-14	NZ_JOCM01000018.1	+	107761	107816	RNA→ rplQ (PRK05591)Ribosomal_L17 (pfam01196)→ PseudoU_synth_EcTruA (cd02570)truA (PRK00021)→
Sri-4-15	NZ_JOCP01000018.1	-	28099	28044	RNA→ rplQ (PRK05591)Ribosomal_L17 (pfam01196)→ PseudoU_synth_EcTruA (cd02570)truA (PRK00021)→
Sri-4-16	NZ_JNYR01000006.1	+	209435	209490	RNA→ rplQ (PRK05591)Ribosomal_L17 (pfam01196)→ PseudoU_synth_EcTruA (cd02570)truA (PRK00021)→
Sla-2-3	NZ_JOEX01000032.1	+	107616	107671	RNA→ rplQ (PRK05591)Ribosomal_L17 (pfam01196)→ PseudoU_synth_EcTruA (cd02570)truA (PRK00021)→
Sla-2-4	NZ_JOCN01000020.1	+	107890	107945	RNA→ rplQ (PRK05591)Ribosomal_L17 (pfam01196)→ PseudoU_synth_EcTruA (cd02570)truA (PRK00021)→
Sri-1-1	NZ_ANSJ01000031.1	+	47278	47333	RNA→ rplQ (PRK05591)Ribosomal_L17 (pfam01196)→ PseudoU_synth_EcTruA (cd02570)truA (PRK00021)→
Sri-3-1	NZ_JJNO01000013.1	+	209385	209440	RNA→ rplQ (PRK05591)Ribosomal_L17 (pfam01196)→ PseudoU_synth_EcTruA (cd02570)truA (PRK00021)→
Sri-2-2	NZ_JMGY01000011.1	+	209215	209270	RNA→ rplQ (PRK05591)Ribosomal_L17 (pfam01196)→ PseudoU_synth_EcTruA (cd02570)truA (PRK00021)→
Sri-4-17	NZ_JOET01000019.1	+	107682	107737	RNA→ rplQ (PRK05591)Ribosomal_L17 (pfam01196)→ PseudoU_synth_EcTruA (cd02570)truA (PRK00021)→
Ssp-90-1	NZ_JNWN01000026.1	-	28254	28199	RNA→ rplQ (PRK05591)Ribosomal_L17 (pfam01196)→
Spe-2-1	NZ_JOCK01000016.1	+	107552	107607	RNA→ rplQ (PRK05591)Ribosomal_L17 (pfam01196)→
Sri-4-18	NZ_JOBW01000066.1	+	21254	21309	RNA→ rplQ (PRK05591)Ribosomal_L17 (pfam01196)→ PseudoU_synth_EcTruA (cd02570)truA (PRK00021)→
Ssp-185-1	NZ_JOJI01000014.1	+	111307	111361	RNA→ rplQ (PRK05591)Ribosomal_L17 (pfam01196)→ PseudoU_synth_EcTruA (cd02570)truA (PRK00021)→
Sri-4-19	NZ_JOBP01000017.1	-	140212	140157	RNA→ rplQ (PRK05591)Ribosomal_L17 (pfam01196)→ PseudoU_synth_EcTruA (cd02570)truA (PRK00021)→
Sri-4-20	NZ_JOJJ01000026.1	+	70453	70508	RNA→ rplQ (PRK05591)Ribosomal_L17 (pfam01196)→ PseudoU_synth_EcTruA (cd02570)truA (PRK00021)→
Ssp-5-1	NZ_KB892002.1	-	454475	454420	RNA→ rplQ (PRK05591)Ribosomal_L17 (pfam01196)→ PseudoU_synth_EcTruA (cd02570)truA (PRK00021)→
Ssp-29-1	NZ_KB891785.1	-	425407	425352	RNA→ rplQ (PRK05591)Ribosomal_L17 (pfam01196)→ PseudoU_synth_EcTruA (cd02570)truA (PRK00021)→
Ssp-31-1	NZ_AQWQ01000002.1	-	425407	425352	RNA→ rplQ (PRK05591)Ribosomal_L17 (pfam01196)→ PseudoU_synth_EcTruA (cd02570)truA (PRK00021)→
Ssp-26-1	NZ_KB891594.1	-	382830	382775	RNA→ rplQ (PRK05591)Ribosomal_L17 (pfam01196)→ PseudoU_synth_EcTruA (cd02570)truA (PRK00021)→
Ssp-27-1	NZ_KB891406.1	-	1044742	1044687	RNA→ rplQ (PRK05591)Ribosomal_L17 (pfam01196)→ PseudoU_synth_EcTruA (cd02570)truA (PRK00021)→
Ssp-79-1	NZ_KI911349.1	+	472182	472237	RNA→ rplQ (PRK05591)Ribosomal_L17 (pfam01196)→ PseudoU_synth_EcTruA (cd02570)truA (PRK00021)→
Ssp-78-1	NZ_AZVF01000001.1	-	1050889	1050834	RNA→ rplQ (PRK05591)Ribosomal_L17 (pfam01196)→ PseudoU_synth_EcTruA (cd02570)truA (PRK00021)→
Aat-1-1	NZ_KB907214.1	-	233058	233000	RNA→ rplQ (PRK05591)Ribosomal_L17 (pfam01196)→
Ssp-117-1	NZ_JOHY01000071.1	-	9294	9239	RNA→ rplQ (PRK05591)Ribosomal_L17 (pfam01196)→ PseudoU_synth_EcTruA (cd02570)truA (PRK00021)→
Ssp-198-1	NZ_KL370770.1	+	196586	196640	RNA→ rplQ (PRK05591)Ribosomal_L17 (pfam01196)→ PseudoU_synth_EcTruA (cd02570)truA (PRK00021)→
Ssp-115-1	NZ_JOHT01000007.1	+	90429	90475	RNA→ rplQ (PRK05591)Ribosomal_L17 (pfam01196)→ PseudoU_synth_EcTruA (cd02570)truA (PRK00021)→
Ssp-148-1	NZ_JOGD01000029.1	+	57793	57839	RNA→ rplQ (PRK05591)Ribosomal_L17 (pfam01196)→ PseudoU_synth_EcTruA (cd02570)truA (PRK00021)→
Psi-1-1	NZ_CP009896.1	-	4283040	4282985	RNA→ rplQ (PRK05591)Ribosomal_L17 (pfam01196)→ IspH (COG0761)ispH (PRK01045)→ PRK11630 (PRK11630)SUA5 (COG0009)→
Ssp-4-1	NZ_ALAP01000018.1	-	152336	152283	RNA→ rplQ (PRK05591)Ribosomal_L17 (pfam01196)→ PseudoU_synth_EcTruA (cd02570)truA (PRK00021)→
Ssp-67-1	NZ_AUKN01000012.1	+	38678	38731	RNA→ rplQ (PRK05591)Ribosomal_L17 (pfam01196)→ PseudoU_synth_EcTruA (cd02570)truA (PRK00021)→
Ssp-201-1	NZ_LAND01000020.1	+	47240	47292	RNA→ rplQ (PRK05591)Ribosomal_L17 (pfam01196)→ PseudoU_synth_EcTruA (cd02570)truA (PRK00021)→
Aal-1-1	NZ_KB892746.1	+	281585	281636	RNA→ rplQ (PRK05591)Ribosomal_L17 (pfam01196)→ rplQ (PRK05591)Ribosomal_L17 (pfam01196)→ PseudoU_synth_EcTruA (cd02570)truA (PRK00021)→ AdoMet_MTases (cd02440)Methyltransf_25 (pfam13649)→ hypo→ hypo→
Sal-13-1	NZ_JOFJ01000024.1	-	50986	50914	RNA→ RplQ (COG0203)rplQ (PRK05591)→
Ari-1-1	NZ_AULB01000026.1	+	70386	70458	RNA→ rplQ (PRK05591)Ribosomal_L17 (pfam01196)→
Ssp-40-1	NZ_ADXA01000020.1	-	61314	61258	RNA→ rplQ (PRK05591)Ribosomal_L17 (pfam01196)→ rplQ (PRK05591)Ribosomal_L17 (pfam01196)→ PseudoU_synth_EcTruA (cd02570)truA (PRK00021)→ PseudoU_synth_EcTruA (cd02570)truA (PRK00021)→
Ssp-39-1	NZ_ADXB01001219.1	+	56	112	RNA→ rplQ (PRK05591)Ribosomal_L17 (pfam01196)→ rplQ (PRK05591)Ribosomal_L17 (pfam01196)→
Ssp-42-1	NZ_GG657742.1	-	2775010	2774954	RNA→ rplQ (PRK05591)Ribosomal_L17 (pfam01196)→ rplQ (PRK05591)Ribosomal_L17 (pfam01196)→ PseudoU_synth_EcTruA (cd02570)truA (PRK00021)→ PseudoU_synth_EcTruA (cd02570)truA (PRK00021)→
Ssp-47-1	NZ_CM001165.1	+	4663630	4663686	RNA→ ←hypo
Ssp-41-1	NZ_GG770539.1	+	4279054	4279112	RNA→ hypo→ rplQ (PRK05591)Ribosomal_L17 (pfam01196)→ PseudoU_synth_EcTruA (cd02570)truA (PRK00021)→ hypo→
Ssp-125-1	NZ_JOGO01000002.1	-	47335	47279	RNA→ rplQ (PRK05591)Ribosomal_L17 (pfam01196)→ PseudoU_synth_EcTruA (cd02570)truA (PRK00021)→
Nsp-3-1	NZ_JIAV01000015.1	-	114208	114159	RNA→ RplQ (COG0203)rplQ (PRK05591)rne (PRK10811)→

Nsp-2-1	NZ_KK211169.1	+	269460	269509	**RNA**→ RplQ (COG0203)rplQ (PRK05591)→
env-35	JGI12075J13256_1000799	-	577	527	**RNA**→ rplQ (PRK05591)Ribosomal_L17 (pfam01196)→
env-36	JGI12112J13074_1005592	-	506	456	**RNA**→ RplQ (COG0203)rplQ (PRK05591)→
env-37	JGI12271J12027_1176655	+	141	191	**RNA**→
env-38	JGI12396J12026_1276148	+	100	150	**RNA**→ hypo→
env-39	JGI12397J12024_1184993	+	151	201	**RNA**→
env-40	JGI12271J12027_1236889	+	33	83	**RNA**→ rplQ (PRK05591)Ribosomal_L17 (pfam01196)→
Nlu-1-1	NZ_JZDQ01000118.1	+	6491	6537	**RNA**→ rplQ (PRK05591)Ribosomal_L17 (pfam01196)→
env-41	JXWU01108663.1	+	303	347	**RNA**→ rplQ (PRK05591)Ribosomal_L17 (pfam01196)→
Nha-1-1	NZ_AUGT01000009.1	+	577134	577185	**RNA**→ RplQ (COG0203)rplQ (PRK05591)→
Sst-1-1	NZ_KQ257875.1	+	79579	79635	**RNA**→ rplQ (PRK05591)Ribosomal_L17 (pfam01196)→
Sle-1-1	NZ_LN831790.1	-	3535110	3535054	**RNA**→ rplQ (PRK05591)Ribosomal_L17 (pfam01196)→ PseudoU_synth_EcTruA (cd02570)truA (PRK00021)→
Sal-2-1	NC_020990.1	+	4279609	4279665	**RNA**→ rplQ (PRK05591)Ribosomal_L17 (pfam01196)→ PseudoU_synth_EcTruA (cd02570)truA (PRK00021)→
Ssp-24-1	NZ_KE150476.1	-	126365	126312	**RNA**→ ←hypo
Ssp-132-1	NZ_JOHQ01000016.1	+	22026	22079	**RNA**→ ←hypo
Ssp-127-1	NZ_JOGK01000040.1	+	21913	21966	**RNA**→ ←hypo
Sgr-5-1	NZ_JNWT01000058.1	+	21899	21952	**RNA**→ ←hypo
Sal-12-4	NZ_JODZ01000010.1	+	169503	169556	**RNA**→ ←hypo
Sal-12-5	NZ_JODR01000016.1	+	224011	224064	**RNA**→ ←hypo
Scl-1-1	NZ_CM001015.1	+	4251346	4251401	**RNA**→ rplQ (PRK05591)Ribosomal_L17 (pfam01196)→
Scl-1-2	NZ_CM000913.1	+	4257377	4257432	**RNA**→ rplQ (PRK05591)Ribosomal_L17 (pfam01196)→ rplQ (PRK05591)Ribosomal_L17 (pfam01196)→
Ssc-1-1	NC_013929.1	-	4144210	4144152	**RNA**→ rplQ (PRK05591)Ribosomal_L17 (pfam01196)→
Ssc-3-2	NZ_KL997514.1	+	21465	21523	**RNA**→ rplQ (PRK05591)Ribosomal_L17 (pfam01196)→
Sde-1-1	NZ_KL570245.1	+	195754	195809	**RNA**→ rplQ (PRK05591)Ribosomal_L17 (pfam01196)→

5.4 Conserved domains

Conserved domains found in protein-coding genes listed in Section 5.3 are shown below, with the first sentence in their description from the Conserved Domain Database (if any). Conserved domains associated with more than one L17-Actino-Proteobacteria RNA are assigned a color, while others are shown in gray. The symbols 'd' and 'u' (if any) to the left of the colored domain name indicate whether the domain occurs upstream or downstream of the RNA motif. Domains marked with lower-case 'd' occur (at least once) present downstream of some L17-Actino-Proteobacteria RNA in the opposite orientation. Domains marked with capital 'D' occur downstream and in the same orientation (i.e., a potential *cis*-regulatory arrangement). Domains marked with a 'u' occur upstream of the RNA in either orientation. If the 'd'/'u' symbols are missing, then the domain is downstream and in the same strand. (This mode is used for motifs predicted as *cis*-regulatory.) The number in parentheses after the colored domain name is the number of occurrences in Section 5.3.

cd01167 (1) Fructokinases (FRKs) mainly from bacteria and plants are enzymes with high specificity for fructose, as are all FRKs, but they catalyzes the conversion of fructose to fructose-6-phosphate, which is an entry point into glycolysis via conversion into glucose-6-phosphate. [or ketohexokinases (KHKs)]
cd02440 (2) S-adenosylmethionine-dependent methyltransferases (SAM or AdoMet-MTase), class I; AdoMet-MTases are enzymes that use S-adenosyl-L-methionine (SAM or AdoMet) as a substrate for methyltransfer, creating the product S-adenosyl-L-homocysteine (AdoHcy).
cd02570 (329) Eukaryotic and bacterial pseudouridine synthases similar to E.
COG0009 (1) tRNA A37 threonylcarbamoyladenosine synthetase subunit TsaC/SUA5/YrdC [Translation, ribosomal structure and biogenesis]
COG0203 (37) Ribosomal protein L17 [Translation, ribosomal structure and biogenesis]
COG0488 (29) ATPase components of ABC transporters with duplicated ATPase domains [General function prediction only]
COG0761 (1) 4-Hydroxy-3-methylbut-2-enyl diphosphate reductase IspH [Lipid transport and metabolism]
COG1004 (1) UDP-glucose 6-dehydrogenase [Cell wall/membrane/envelope biogenesis]
pfam01196 (524) Ribosomal protein L17.
pfam01416 (1) tRNA pseudouridine synthase.
pfam02156 (1) Glycosyl hydrolase family 26.
pfam09594 (19) Protein of unknown function (DUF2029).
pfam12848 (1) ABC transporter.
pfam13304 (21) AAA domain, putative AbiEii toxin, Type IV TA system.
pfam13620 (1) Carboxypeptidase regulatory-like domain.
pfam13649 (2) Methyltransferase domain.
PRK00021 (330) tRNA pseudouridine synthase A; Validated

PRK01045 (1) 4-hydroxy-3-methylbut-2-enyl diphosphate reductase; Reviewed
PRK05591 (561) 50S ribosomal protein L17; Validated
PRK09434 (1) aminoimidazole riboside kinase; Provisional
PRK10811 (1) ribonuclease E; Reviewed
PRK11630 (1) hypothetical protein; Provisional
PRK11855 (1) dihydrolipoamide acetyltransferase; Reviewed
PRK13428 (1) F0F1 ATP synthase subunit delta; Provisional
PRK14521 (1) 30S ribosomal protein S16; Provisional
smart00382 (29) ATPases associated with a variety of cellular activities.
TIGR03026 (1) nucleotide sugar dehydrogenase.

5.5 Multiple-sequence alignment

Each L17-Actino-Proteobacteria RNA is denoted by an abbreviation (like "Eco-1-1") that refers to a taxonomy given in Section 5.1. (Superscript numbers refer to annotations listed in Section 5.2) The alignment may include sequences containing the RNA motif, as well as flanking sequence. The L17-Actino-Proteobacteria RNA itself is denoted by the line underneath marked 5′ and 3′ on either end. Nucleotides in flanking sequences (i.e., not part of the motif) are written in gray letters. Stems of predicted rho-independent transcription terminators, if any, are shaded yellow. (Note: terminator predictions have not been analyzed manually, and many are likely to be false positives. Terminator predictions are those of the RNIE software.) Nucleotides predicted to function (as DNA) as transcription-factor binding sites, if any, are shaded in green. (But note: these predictions are manually annotated, so they might be under-predicted.) Annotated start codons, if any, are shaded green. (Note: start codons are frequently misannotated, especially in environmental samples.) Nucleotides proposed to basepair as part of the consensus structure are shaded in color when they comprise Watson-Crick or G-U pairs. Otherwise they are shaded gray. Conserved stems are also indicated at the bottom of the alignment by angle brackets, where matching $<$ and $>$ denote base-paired columns. Below these angle brackets, the symbol "2" denotes base pairs exhibiting covariation according to the statistically well-founded R-scape method. "1" denotes base pairs exhibiting covariation according to R2R's simplistic method. "0" denotes base pairs that are not observed to mutate and "?" denotes base pairs that have a significant frequency of non-canonical nucleotides for Watson-Crick or G-U pairs ($> 5\%$). Below these base pair annotation is the consensus sequence: "R" = "A" or "G", "Y" = "C" or "U", red nucleotides: nucleotide identity conserved more than 97% of the time, black nucleotides: 90%, gray nucleotides: 75%, red circle (•): nucleotide is present 97% of the time, black circle (•): 90%, gray circle (•): 75%, white circle (◦): 50%. All percentages of sequences just described (e.g. 97% conserved) assume that sequences have been weighted by the GSC algorithm implemented by the Infernal software package.

Duplicate sequences: the following putative homologs are not shown in the alignment because their sequences are identical to a homolog already shown: Kau-1-2, Kpu-1-1, Mha-1-1, Mxa-2-1, Mxa-3-1, Sac-1-1, Sal-1-1, Sal-10-1, Sal-12-4, Sal-12-5, Sal-4-1, Sal-8-1, Sal-9-1, San-2-2, Sau-3-2, Sav-1-1, Sav-2-2, Sbi-2-2, Sbi-3-2, Sbi-3-3, Sbo-1-2, Sbr-1-1, Sca-2-2, Sca-5-1, Sca-5-2, Sca-6-1, Sca-6-2, Scl-1-2, Sfl-2-1, Sfl-3-1, Sfl-7-1, Sfr-1-2, Sfu-3-1, Sga-1-1, Sgr-12-2, Sgr-12-8, Sgr-12-9, Sgr-13-2, Sgr-13-3, Sgr-13-4, Sgr-13-5, Sgr-5-1, Sgr-8-1, Shy-3-1, Sla-2-3, Sla-2-4, Sli-1-1, Sli-2-1, Sli-2-2, Sna-1-1, Sol-2-2, Spe-2-1, Spl-1-2, Spu-2-2, Spu-3-1, Spu-3-2, Sri-1-1, Sri-2-2, Sri-3-1, Sri-4-10, Sri-4-11, Sri-4-12, Sri-4-13, Sri-4-14, Sri-4-15, Sri-4-16, Sri-4-17, Sri-4-20, Sri-4-3, Sri-4-4, Sri-4-6, Sri-4-7, Sri-4-8, Sri-4-9, Sro-2-1, Sru-1-1, Sru-1-2, Ssc-3-2, Ssp-10-1, Ssp-100-1, Ssp-107-1, Ssp-108-1, Ssp-109-1, Ssp-11-1, Ssp-126-1, Ssp-127-1, Ssp-130-1, Ssp-132-1, Ssp-136-1, Ssp-137-1, Ssp-147-1, Ssp-148-1, Ssp-167-1, Ssp-173-1, Ssp-178-1, Ssp-179-1, Ssp-18-1, Ssp-180-1, Ssp-181-1, Ssp-184-2, Ssp-196-1, Ssp-22-1, Ssp-27-1, Ssp-31-1, Ssp-32-2, Ssp-34-1, Ssp-35-1, Ssp-60-1, Ssp-63-1, Ssp-67-1, Ssp-78-1, Ssp-79-1, Ssp-88-1, Ssp-91-1, Ssp-92-1, Ssp-99-1, Sva-2-2, Svi-12-2, Svi-13-3, Svi-13-4, Swe-1-1, Sxa-1-2, Sxi-1-1, Tbi-1-1, env-17, env-25, env-26, env-27, env-28, env-29, env-36

alignment positions 1 · · · 79

```
env-1     AGUGCUCGCUUCGCGCGGCCUGACCUUGGGUAUGAAGCUCGAAAACUGGCCGCCGGCCGGUCUCGACAAACACUGAACU
env-2     ..............................................UGGCCGCCGGCCGGUCUCGACAAACACUGAACU
[br-1-1   CGUGCGGCUCGAGAACUGGCCGCCGUCCGGCCUCGACAAGCGUUAAAAAAUCGGGGUGUCGGCUGCAGGCCGGCACCCC
env-3     GCGCGGCCUGACCUUGGGCAUGAAACUGGAAUCGUGGCCGCCCGCCGGCCUGGACAAACGUUAAUUCAACCCGGUGAAA
Nal-1-1   CGCAGGCCGCCCUCGCGGCGUACGGCGACGACGACGACGACGCGUUCGUCGAGGACGAGCAGUACUGAUCCAGCCCCAC
Nsp-1-1   CCUCCGCCUGGCGGCAAGACACGAGAGACGCAGCCGUCGGGUGGGGCCUGGCCCCGCCUGGCGGCAAGACAAGUGAAGA
Mxa-1-1   GCCUCCCCACGUGGCCGCGGUACGGCGGUCAGCGGCGCGAGGGGAGGGGGCUGGGACGUCUUCCGGUCCGAAUGACAGU
env-4     UGCUUUAACCCGCGCGUCCGGGGCGGCACGGCCGCAUGCCCCGGAUGGACGGGGGACCCUCACCGGUCCAAUACAUUGA
Cfu-1-1   GCGAGUACCUGCCCACCCGGGGCGGCAUGGUUUCAUGCCCCGGGACGAGCAGGGGGCCCUCACCGGCCCAACCCCGCAG
Avi-1-1   UCGCAGUACCUCGUCCCUCGGGGCGGCAUGGCCGUAUACCCCGGGGGAGGAGGGGGCCUUCCCAGGCCCGAUACAGCAA
Age-1-1   CCGCAGUCCCUCGUCCCUCGGGGCGGCAUGGCCGCAUACCCCGGGGGAGGAGGGGGCCCUCCCAGGCCCAACACAGCAA
Hmi-1-1   UCGGCCCGCGCCGUGACACGGCUUCACGCGGCGCGGGAAGAGGGGGCCCCUCCCCAUAAAAGUGGGGGCCUAUUGGCAA
```

```
env-5       UCUGAAGCACCUGCUCUCGCGCCGCGACACAGCCGCAGGCGGCGCGAGGGGAGGGGGCCCGUAAGGCCCAACAAGGCAA
Sau-5-1     UGUCGUACCUCACGCCUCGCCGCGACACGGCUUCACACGGCGGGGAGUGAGGGGGGCCCCUGAAAAGGCUCCUUGGCAA
Mfu-1-1     CCCCGCGCGCCCGCGACAUGGCGGCAAGCGGCGCGCGGAGGGAAGGAGCCCGGUGAAGUCCUCCGGGUUCGACGAGCAG
Mst-1-1     UUUCCUCCGUGCCCGCGACAUGGUUGCAGGCGGCGCGGAGGGAAGGGACCUGGUGAGACCCACCUGGUCCAAUCAGCAG
Msp-2-1     ACCUCCUCACGCGUCCGCGGGCACGGCGGUCAGCGGCGCGAGGGGAGGGGGCUGGGACGUCUUCCGGCCCGAAUGACAG
Mfu-2-1     GCCUCCCCACGCGGCCGCGGGCACGGCGGUCAGCGGCGCGAGGGGAGGGGGCCGGGACGUCUUCCAGCCCGAAUGACAG
env-6       CUCGGUCCACGCCGUGACACGGCUUCACGCGGCGCGGGCAGAGGGGGCCUCCCUCCAUAAACGGGAGGCCCAAUGGCAA
Cco-1-1     CACCUGCCUUGGCUGCCCACCGACACGGCUUCACGGUGGCGCGAAGGCAGGGGGCCCUGGUACACAGGGCCCGAUUCAA
env-7       ..........................................................GCCGCGAGGCACGGUCGACGAA
env-8       AGAACUGGCCGCCGCAGAACCUGGACAAGCGCUAGAGCUCUGACAAUACGAAAGGCGGCCCAAGUGCCGUCCCAGCGUG
Ama-1-1     GGACAACUACGGCGACGACGACCAGAGCUACGCCGAGACCGAGCAGUACUAGACACCCGAUUCCGGCGGCCGGGGGCAC
env-9       CGGAGUGCGACCAUGAGUACCGAGCACGAAGCGAGCGCGACCGUCAGUGCAGAGUCCGAGCGGCGCGCGGAUCCCAGAC
env-10      ...................................GCGCGACCGUCAGUGCUGAGUCCGAGCGGCGCGCCGAUCCCAGAC
1 Ssp-68-1  CUGCGGGCCGGCUGCGGCCUGCCGCUCCCACGCGGCGAAGCCGCAUGGGAGCACAGCCCCGCGCCCUUCGGGGGCGCA
env-11      AGACCGAGCAGUACUGACCGGACCGCGGCGGCCGGGGCCGCGUUCCCCGGCCGUCCGGGCAGCACAGGCGCGCGGGCGU
env-12      GCAGUUGGCGCGGUCACAACUACAGAUUGAUUGGCGGCUGCGGGCCGAAACUCUUGGUUUCGGGUUCGCUUCGCCACAC
Ssp-96-1    CUACGCGGAGACCGAGCAGUACUAAGAAUUCGGGACGGGGGCGGCCCUUCGCGGGCCGCCCCCGCCACCGUGCAAGUCG
Ksp-1-1     GCGGAGACCGAGCAGUACUAAGAGUUUGGGACGGGGGCGGCCCCUCGCGGACCGCCCCCGCUUCAAUGCACACAAGCCG
Kme-1-1     UUACGCGGAGACCGAGCAGUACUGAGUUUGGUCCGGGACGGCUCCCACCGGAGCCGUCCCGGUUCAUGAAGGAAAGUCG
Rge-1-1     AGCGUUGAGAUCGCGGGCCGCUCGCGGCUCGUCGUCGUAAGAACCGCUGCGGCUCCCGGACGGAGCCGCGGUGUCGAU
Sca-3-1     UGAUCCAGCACUGAUCACCGUUCAGGUGGUACGGGACCGCUCCGGCGGUCCCGUACUCUUGAUGGCUGUAUGACCAAGC
Sal-3-1     AGACCGAGCAGUACUGAGAAACACCUGGGUACGGGACCGCCUCGGCGGUCCCGUACUCUUGAAGGCUGUGCAACCAAGU
env-13      GACGACGACGACGACGCGUUCGUCGAGGACGAGCAGUACUGAUCCGUCCGGGAGGCUCCGCCCUCCCGGACACCCCCGA
1 Sav-3-1   GACGCGUUCGGCGCGGAGGACGACGGCGACGCGGGCUUCGUCGAGACCGAGCAGUACUGACCCCUCGGGGUCGCCAACC
Ssp-195-1   AGACCGAGCAGUACUGAUCCGACGGGGCGUGCGACCGGCCGCGGCCGGCCCCACGCCCCGGUCCCCGUCCGGCGAUCCG
Nsp-4-1     ACCGCGGUGGCCGGCGGCGGCUACGACGACGACGACAGCGCGUACGUCGAGACCGAGCAGUACUGAUUUUCCCGAUAGC
env-14      .....................................................GGAGAGGGGCCCGCGCGGGUGAACGUA
Nco-1-1     CGCGGUGGCCGGCGGCGGCUACGACGACGACGACAGCGCGUACGUCGAGACCGAGCAGUACUGAUCACACACUGAUAGC
Sje-1-1     GACCGAGCAGUACUGAGCCUGAUCGAGUGGUACGGGACCGCCCCGGCGGUCCCGUACCCUUGAAGGCUGUGCAACCAAG
Kaz-1-1     GGACGGGGGCGGCCCUCGGGUCGCCCCUGUUCGAUGCACACAGGGCUCUGCGGGGGACAACCCCCAGACCCCCACUAAG
Kph-1-1     GGCUACGCGGAGACCGAGCAGUACUGAGUUUCGACACCGGGGGCGGUCCUCGCCGGACCGCCCCCGGUGGCGUGAGAAG
Kph-1-2     GCUACGCGGAGACCGAGCAGUACUGAGUUUCGACACCGGGGGCAGCCCUUCGCCGGGCCGCCCCCGGUGGCGUGAGAAG
Sno-2-1     GGUUACGCCGAGACCGAGCAGUACUAAGAGAUUUGGCCGCGGGGGCGGUUCCACGGAGCCGCCCCCGUCGUCACGGAAG
Ksp-3-1     GCACUUCGCCGAGACCGAGCAGUACUGAGAGAAUUGGCCGCGGGGGCGGUUCCACGAACCGCCCCCGUCGUCGUGAAAG
Spy-1-1     GGUUACGCCGAGACCGAGCAGUACUAAGAGAUUUGGCCGCGGGGGCGGUUCCACGGAGCCGCCCCCGUCGUCACGGAAG
Ksp-4-1     AGACCGAGCAGUACUGAGUUCCCUGAAGUCUUGACGCCGGGGGCGGUUCCCAGUGGAUCGCCCCCGGGGCCAUGAAAAG
Ssp-165-1   GGUUACGCGGAGACCGAGCAGUACUGAGAGACUUGACCGCGGGGGCGGUUCCCCGGAACCGCCCCCGUCGUCGUGAAAG
Ssp-133-1   CGGGUUACGCGGAGACCGAGCAGUACUGAGAGACUUGACCGCGGGGCGGUUCCCCGGAACCGCCCCGCUGUCGUGAAAG
Ssp-50-1    AGGGCUACGCGGAGACCGAGCAGUACUAAGAGUUUGAGUCGGGGGCGACUCGCCACGGGUCGCCCCCGAUCCAUGCAAG
Ssp-157-1   GGCUACGCGGAGACCGAGCAGUACUAAGAGACUUGGCCGCGGGGGCGGUUCCCCGGAACCGCCCCCGUCGUCCUGGAAG
Ssp-160-1   GCUACGCGGAGACCGAGCAGUACUGAGUUUCGACACCGGGGGCGGUCCUUCGCCGGACCGCCUCCGGUGGCGUGAGAAG
Ksp-2-1     GGUUACGCGGAGACCGAGCAGUACUGAGAGACUUGACCGCGGGGGCGGUUCCAGGAACCGCCUCCGUUGUCGUGAAAG
Ssp-183-1   CGGGUUACGCGGAGACCGAGCAGUACUGAGAGACUUGACCGCGGGGCGGUUCCCCGGAACCGCCCCGCUGUCGUGAAAG
Sam-2-1     CCGAGCGCGGUGGCCGGUGGCGGCUACGACGACGACGACAGCGCCUACGUCGAGACCGAGCAGUACUGAUUUUUCAAUA
Sro-7-1     CCCAGCGCGGUGGCCGGCGGCGGCUACGACGACGACGACAGCGCGUACGUCGAGACCGAGCAGUACUGAUUUUUCUGUA
Ssp-87-1    CGGACGCCGGUUUCGUCGAGACCGAGCAGUACUAGAACCUCCCGGUCCGCUGUGGCUGAGGUCUUGUCCUCAAUCGCCG
Ssp-190-1   GCGGACGCCGGUUUCGUCGAGACCGAGCAGUACUAGAACCUGCUGGUCCCUGUGGUUGAGGUCUUGUCCUCAAUCGCCG
Sir-1-1     CGGACGCUGGAUUCGUCGAGACCGAGCAGUACUAGAACCUGCUGGUCCGCUGUGGUUGAGGUCUUGUCCUCAACCGCCG
Shy-4-1     CGGACGCCGGUUUCGUGGAGACCGAGCAGUACUAGAACCCGCUGGUCCGCUGUGGCUGAGGCCGCGUCCUCAGCCACCG
Sro-3-1     CCCCAGCGCGGUGGCCGGCGGCGGCUACGACGACGACGACAGCGCGUACGUCGAGACCGAGCAGUACUGAUUUUUCUGU
Nin-1-1     GUUCGUCGAGCCCGGCCGGUGGAUGCAAGCGGCACUGUCCACCGGCUGCCGCGCGACAACUGAAGAUCCGGUGGCCGAG
1 Nca-1-1   ACGACAGCGCGUACGUCGAGACCGAGCAGUACUGAUUCACCGUCAGCCCCCGCGUCUGAUCGCGGGGGCUCAAUAACGG
Ssp-192-1   UCGCGCCGCCACCGGUACCUGAUACGGCCGGUGCGGCUCGCCUCCGGUGGGGAAGCCGUGUGCCGCCCCCGCCGGGGCC
Ssp-172-1   GACGACGCCGACGCCGGGUUCGUCGAGACCGAGCAGUACUAAAUUCUCUGCGGACCGGCUCGGGCCGGUCGCGCAGUUC
Ssp-93-1    CGAGACCGAGCAGUACUAAGAGCUCGGGACCGGCCGCUUGAUUUUGGCUGCGCGUGCGUUGUGGUUGUUCGCGCAGUUC
Sau-3-1     CGAGACCGAGCAGUACUGAGAGCUCGGGCCUCGCGGUUCGAUUGUGGCUGCGGACCGGCUUCGGCCGGUCGCGCAGCUC
Sal-6-1     CGAGACCGAGCAGUACUGAGAGCUCGGGACUGGCGGAUUGAUUUUGGCUGCGCGUGCGUCUUGAUUGCGCGCGCAGUUC
Sau-1-1     UCGAGACCGAGCAGUACUAAGAGCUCGGGACUGACGGUUCGUUUUGGCUGCGGGGCCGUUGUGGCUGGUCGCGCAGUUC
Ssp-106-1   AGGACGACGGGGACACGGGCUUCGCGGAGACCGAGCAGUACUGACGAAGCGGUACUGAGCGGCCCCCGCGGCUGACAGU
Ssp-3-1     GACGCCUUCGGGGCCGAGGACGACGGGGACACGGGCUUCGCGGAGACCGAGCAGUACUGACGGUCCUGCGUCUCACAGU
env-15      GCGGCCCUCGGCACGGUGACCGCCCGGCCCGGCCGGGGCGGCACCGCCGCCAGCGGUCUGCCUGGCAAGCCAUGAAGGU
env-16      GCGGCCCUCGGCACGGUGACCGCCCGGCCCGGCCGGGGCGGCACCGCCGCCAGCGGUCUGCCUGGCAAGCCAUGAAGGU
env-18      ...................................................................AAGCCAUGAAGGU
Ssp-118-1   GCCGCGCGGCACCGCCGUGCUCGCGGCGUCACGGGGCGGCGUGCUGAACGCCGCACGCCGUACGCCGCACACACGAAGC
env-19      CACCGGGCGCCGGACUGCUCGGCGGUAGUGAGCCCUCCGGAUCGCGGCCACCGGCCCGUUCCGGGAGAUUGUCAAAGAG
env-20      CACCGGGCGCCGGACUGCUCGGCGGUAGUGAGCCCUCCGGAUCGCGGCCACCGGCCCGUUCCGGGAGAUUGUCAAAGAG
env-21      ACCGGACGCCCGGACUGUUCGGCGGCAGUGAGCCCUCCGGAUCGCGGUGACCGCCCGAUCCGGCAGACAUGUCAAAGAG
Sor-1-1     ACCGAGCAGUACUGAGAACACAUCGUCCCGUCGGCGUCCCCGCCGGCGUGAUGCCGUAAGUCGUACGGCGUGCGCGGAC
Ssp-64-1    GUGGCUGCCUCCGGGGCCACGGCCCCGGGCUCCGGGCGCGAGAGCCCGGCACAGAUGUCCCCGGUACCGCCGGGGGGUG
Ssp-12-1    GGGUGGCUGCCUCCGGGGCCACGGCCCCGGGCUCCGGGCGCGAGCCCGGCACAGAUGUCCCCGGCACCGCCGGGGGGUG
Ssp-13-1    GGGUGGCUGCCUCCGGGGCCACGGCCCCGGGCUCCGGGCGCGAGCCCGGCACAGAUGUCCCCGGCACCGCCGGGGGGUG
Ssp-61-1    CCCGACCUCCGCCGCCGACACCUUCGGCGCCGACGACGACGCCGACGCGGGCUUCGUCGAGACCGAGCAGUACUGAAGU
Ssp-59-1    CCCCACCUCGGCCGCCGACACCUUCGGCGCCGACGACGACGCCGACGCCGGCUUCGUCGAGACCGAGCAGUACUGAUGU
Ssp-16-1    CCCCACCUCGGCCGCCGACACCUUCGGCGCCGACGACGACGCCGACGCCGGCUUCGUCGAGACCGAGCAGUACUGAUGU
Ssp-69-1    CCCCACCUCGGCCGCCGACACCUUCGGCGCCGACGACGACGCCGACGCCGGCUUCGUCGAGACCGAGCAGUACUGAUGU
Ssp-56-1    GCCGCCGACACCUUCGGCGCCGAGGACGACGCCGACGCGGGCUUCGUCGAGACCGAGCAGUACUGAGCAGCACUGAUGU
Hcr-1-1     CGCUGUCGCCGGUGGCGGCUACGACGACGAGGACGGCGGCUUCAUCGAGACCGAGCAGUACUGAGUUUUCGCAGAUAGA
Msp-1-1     CGUCACCGAUCGCUGCCGGUAGGCACCACCGCAUCACCGGCCGCCCCGCGUCGUACGGCGUGGGGCGGCGAUGACAGCC
Mro-1-1     CUGAUCGCCCGUCUCCGAGCAGCGGCACCACCGUCUCGUGCCGCCCGUCCAGGGCGGCAUGAGGCGGCGAUGACAAGUC
Ssp-171-1   CCGACGCCGGCUUCGUCGAGACCGAGCAGUACUGAUCCACGCUUGCGGGAGGCCCGGGCACUGCCCGGGCCCCCCGUAC
Ssp-43-1    GAGCAGUACUAGUAGAUCGUCUGCGGGCCGGCUUCGGCCGGUCGCGCAGUUCCCCGCGCCCCUUACGGGGCGCGGGUCU
Svi-13-1    GAGCAGUACUAGUAGAUCGUCUGCGGGCCGGCUUCGGCCGGUCGCGCAGUUCCCCGCGCCCCUUACGGGGCGCGGGUCU
Ssp-73-1    CGUGGCUGGUCGCGCAGUUCCCCGCGCCCCUUACGGGGCGCGGUCCUCCGAGGCGCUUCGAAAGAGGAACCCCGGAUCU
Ssc-3-1     GGUGCGGGUGCGCUGUGGCUUGUCGCGCAGUUCCCCGUGCCCCUUCCGGGAGCGUCCCUUCGGGACUGCUCCCGGAUCU

            ................................................................................
            ................................................................................
            ●●●●●●●●●●●●●●●●●●●●●●●●●●●●●●●●●●●●●●●●●●●●●●●●●●●●●●●●●●●●●●●●●●●●●●●●●●●●●●●●
```

```
Kau-1-1     GGUUCGACGGUUCGUUUUGGCUGCCGGCCCGCUGUGGCUGGUCGCGCAGUUCCCCGCGCCCCUUUGGGGCGCGGGGUCU
Sgr-1-1     GCGCGGGGCGGCUGUGGCUUGACGCGCAGUUCCCCGUGCCCCUUCCGGGCACGGCCCCUCGGGGCCCGUGUCCGGAUCU
Sto-1-1     GGUGCGGGUGCGCUGUGGCUUGUCGCGCAGUUCCCCGCGCCCCUUCCGGGAGCGCCCCUUCGGGGCUGCUUCCGGAUCU
Ssp-114-1   GGCUGUGGCUGGUCGCGCAGUUCCCCGCGCCCCUUUGGGGCGCGGACCACUCCGGGCGUCCGAAAAGGCGCCCGGAUCU
Ssc-4-1     GGUGCGGGUGCACUGUGGCUGAGCGCGCAGUUCCCCGCGCCCCUUCCGGGAGCGCCCCUGCGGGGCGACUCCCGGAUCU
Ssp-1-1     CCGGGUGCGGGUGCGCUGUGGCUUGUCGCGCAGUUCCCCGCGCCCCUUCCGACUCGCCCCUUCGGGGCUCGUCGGAUCU
Sau-4-1     GACCGGCUGUGGCUGAUCGCGCAGUUCCCCGCGCCCCUUACGGGGCUCGGUACCUCCGGCGCUGAAAAGUGCCGGAUCU
Spr-1-1     CCGACGACGACGCGGACGCGGGCUUCGUCGAGACCGAGCAGUACUGAGAAGGCUUGCGGGGAGCUCCGGCUCCCCGCAC
Ssp-189-1   CGACGACGACGCGGACGCCGGUUUCGUCGAGACCGAGCAGUACUGAGAAAGCCUGCGGGGGGCCUCGCCCCCCGCACCC
Sfl-6-1     GACGACGACGCGGACGCCGGUUUCGUCGAGACCGAGCAGUACUGAGAAAGCUUGCGGGGGUCUCGCGCCCCCCGCACCC
Ssp-103-1   CGACGACGACGCGGACGCCGGUUUCGUCGAGACCGAGCAGUACUGAGAAAGCUUGCGGGGGGCCCCGCCCCCCGCACCC
Ssp-111-1   GACGACGACGCGGACGCCGGUUUCGUCGAGACCGAGCAGUACUGAGAAAGCUUGCGGGGGGCUCGCGCCCCCCGCACCC
Ssp-159-1   CGACGACGACGCGGACGCCGGUUUCGUCGAGACCGAGCAGUACUGAGAAAGCUUGCGGGGGGCUCGCGCCCCCCGCACC
Ser-1-1     GACGACGACGCGGACGCCGGUUUCGUCGAGACCGAGCAGUACUGAGAAAGCUUGCGGGGGGCUCACGCCCCCCGCACCC
Sya-1-1     GACGACGACGCGGACGCCGGUUUCGUCGAGACCGAGCAGUACUGAGAAAGCUUGCGGGGGGCUCGCGCCCCCCGUACCC
Svi-12-1    CCGACGACGACGCGGACGCCGGUUUCGUCGAGACCGAGCAGUACUAGAAAAGCUUGUGGGGGGCUCACGCCCCCCACAC
Ssp-151-1   CCGACGACGACGCGGACGCCGGUUUCGUCGAGACCGAGCAGUACUAGAAAAGCUUGUGGGGGGCUCGCGCCCCCCACAC
Ssp-110-1   GCCGACGACGACGCGGACGCCGGUUUCGUCGAGACCGAGCAGUACUAGAAAGCUUGUGGGGGGCUCGCGCCCCCCACAC
Ssp-112-1   GACGACGACGCGGACGCCGGUUUCGUCGAGACCGAGCAGUACUGAGAAAGCUUGCGGGGGGCUCACGCCCCCCGCACCC
Ssp-102-1   CCGACGACGACGCGGACGCCGGUUUCGUCGAGACCGAGCAGUACUAGAAAACUUGUGGGGGGCUCACGCCCCCCGCACC
Sla-2-1     CGACGACGACGCGGACGCCGGUUUCGUCGAGACCGAGCAGUACUGAGAAAGCUUGUGGGGGGCUCACGCCCCCCACACA
Ssp-139-1   CCGACGACGACGCGGACGCCGGUUUCGUCGAGACCGAGCAGUACUGAGAAAGCUUGUGGGGGGCUCGCGCCCCCCACAC
Ssp-8-1     CCGACGACGACGCGGACGCCGGUUUCGUCGAGACCGAGCAGUACUGAGAAAGCUUGCGGGGGGCUCGCCCCCCGCACCC
Svi-7-1     GACCGACGGUCCAGAUUUGGCUGCGGACCGGCUUCGGCUGGUCGCGCAGUUCCCCGCGCCCCUAACGGGGCGCGGGGUC
Sgr-12-1    UACUGACGGCCGCAGUUCCGCUGCCGGUCCGCUCCGGCUGACCGCGCAGUUCCCCGCGCCCCUCACGGGGCGCGGGGUC
Sfl-5-1     CCCGGGUGCGGGAGCGCUGUGCUUGAUCGCGCAGUUCCCCGCGCCCCUUCCGGGCGCCCCUUCGGGGGCUGCCCGGAUC
Sbi-3-1     GAUCGCGCUCACACGGCGGAGCCGUACAUCGGCUCCGCCCCGCGCCCCUUCCGGGCGCCUCCACGAGGCCUUCCGGAUC
Sex-1-1     AGCAGUACUAGUAGAUCGUCUGCGGGCCGGUUUCGGCUGGUCGCGCAGUUCCCCGCGCCCCUUAACAGGGGCGCGGGUC
Sgl-3-1     ACUGAGGUCGUAGUUCUUGCUGCGGACCGGCCUCGGCCGGUCACGCAGUUCCCCGCGCCCCUUCCGGGGGCGCGGGGUC
Sgr-9-1     GGACCGACGGUCCAGAUUCGCCUGCGGGCCGGCUGUGGCUGGUCGCGCAGUUCCCCGCGCCCCUGAGGGGGCGCGGGUC
Ssp-121-1   UCGGGUCCGUACUCGGGUGCGGGUGGACUGUCGCUGGUCGCGCAGUUCCCCGCGCCCCUUCCGGGGCCCUGCCCCGGUC
Sve-1-1     AGUACUAGUCGCAGUCUUGCUGCGGGCCGGCUUCGGCUGGUCGCGCAGUUCCCCGCGCCCCUGACCGGGGCGCGGGGUC
Sav-2-1     GGGUGCGGGUGCGCUGUGGCUUGUCGCGCAGUUCCCCGCGCCCCUUCCGGGGGCGCCUCUACGAGGCUGCCCCCGGAUC
Mgl-1-1     GGGUCCGUACUCGGGUGCGGGUGGACUGUGGCUGUUCGCGCAGUUCCCCGCGCCCCUUUCCGGGGCGUACGCCCCGGUC
Asp-1-1     GCGGACGCGGGUUUCGUGGAGACCGAGCAGUACUGAUCGGUCCCCCUUCCGGGAGCCGCCUUCGGGAGGCUCCCGGAUC
Stu-1-1     GGGUGCGGGUGCGCUGUGCUUGAUCGCGCAGUUCCUCGCGCCCCUUCCGGGAUCGCCCCUUCGUGGCUGCUCCCGGAUC
Sye-2-1     GCGGAGCCGCAAAUUGACACAGCCCGCGCCCCUGAAGGGGCGCGGGUGCUCCGGGCGCCUUGAAAAGGGUGCCCGGAUC
Sdu-1-1     CCGGCUUCGGCUGGUCGCGCAGUUCCCCGCGCCCCUUCGGGGGCGCGGGUCUUCCGGUGCUCGAAGGAGCGCCCGGAUC
Sgh-1-1     GAUCGGAUCCUAGGAAGCGGGCGCGCCAUGCGCGCCCGCUCCCCUUCCGGGAGCUCCCCCACGGGGUGGCUCCCGGAUC
Svi-5-1     GAUCGGAUCCUAGGAAGCGGGCGCGCCGUGCGCGCCCGCUCCCCUUCCGGGAGCUCCCCCACGGGGUGGCUCCCGGAUC
Ssp-153-1   CCGGGUUCGUCGAGACCGAGCAGUACUAGGAGCUCGGGACCGACGGUCCUCCGGGCGCCCCCGCGGGGACGCCCGGAUC
Ssp-86-1    CGAGGAUCGGCAGCACUGCCACUGCGGGUCGGCUGUGGCUGGUCGCGCAGUUCCCCGCGCCCCUCUGGGGCGCGGGGUC
Sro-1-1     ACGCGGACGCGGGUUUCGUGGAGACCGAGCAGUACUGAUCUCCACAGCCUCCGGGGUUCGCGUCAGCGGACCCCGGAUC
Sgr-7-1     GGGCCUCAGGGCUCGAUUUCGUCUGCCGGCCGGCUGCGGCUGGUCGCGCAGUUCCCCGCGCCCCUUCGGGGCGCGGGUC
Ssp-28-1    GGUGCGGGAGCGCUGUGCUUGAUCGCGCAGUUCCCCGCGCCCCUUUCCGAGCAGGCCCCCUUCGGGGCCCGCUCGGAUC
Ssp-176-1   CGUUUUUCGGUUGCGGGUCGGCUGUGGCUGGUCGCGCAGUUCCCCGCGUCCCUUCCAGGCCCCUUCGGGGGCCUGGAUC
Ssp-14-1    GCAGCCGCACAGGCAGCACCGCCCCGCGCCCCUGACGGUGCGCCCUUCCGGGGGCGCACCCUGACGGGGGGCGCGGGUC
Sac-2-1     CGACGCGGACGCGGGUUUCGUGGAGACCGAGCAGUACUGAGUCUCUGCUUCCGGGGUUCGCGUCAGCGGACCCCGGAUC
Sbo-1-1     CGGGCGCGGGUGCGCUGUGCUUGAUCGCGCAGUUCCCCGCGCCGCUUCCGGGCAGGCCCUUCGGGGCCUCGCCCGGAUC
Ssp-2-1     ACGCGGACGCCGGGUUCGUGGAGACCGAGCAGUACUAAGCGCUCCGCACCCCUUCCAGGCCCCUUCGGGGGCCUGGAUC
Ssp-98-1    CGGGUGCGCUGUGGCUUGUCGCGCAGUUCCCCGCGCCCCUUCCGGACGAUGCGCCCCUUCGGGGCUUGUCGUCCGGAUC
Sac-3-1     GCGCUGUGGCUUGUCGCGCAGUUCCCCGCGCCCCUAAGGGCGAUGCCCCUUUCCGGAGUUUCGUAAGAAGCCUCGGAUC
Sca-7-1     GCGGUUCCGUUCUUGCUGCGGGUCGGCUGUGGCCGGUCGCGCAGUUCCCCGCGCCCCUCUUUGCGAGGGGCGCGGGGUC
Sgr-2-1     CGUACUGCCCGUGCGGGCCAGCUGUGGCUGGUCGCGCAGUUCCCCGCGCCCCUUCCAGGCCCCUUCGGGGGCCUGGAUC
Ssp-105-1   GCGUCUGCGGCCGGCAGUGGCCGGUCGCCCAGUUCCCCGCGCCCCUUCCGAGCGGGCCCCUUCGGGGCCGGCUCGGAUC
Ssp-85-1    CGGCUGCGGCCGGCCGCGCGGUUCCCCGCGCCCCUUCGGGAGCGCGGGCCCUCCGGGAGUCCGCGAGGACUCCCGGAUC
Sco-3-1     CCGGCUGUGGCCGGUCGCGCAGUUCCCCGCGCCCCUUCGGGGGCGCGGACCCUCCGGGAUCGAACGUGAACCCCGGAUC
Sse-1-1     CGUCUGCGGGCCGGCUGUGACUGGUUCGCGCAGUUCCCCGCACCCCUUCCGAGCGGGCCCUUCAGGGCCUGCUCGGAUC
Sre-1-1     NNNGGCUGGUCGCGCAGUUCCCCGCGCCCCUUACGGGGCGCGGAUCCUCCGGGGUGCUUCGAAAGAGGCGCCCCGGAUC
Spe-1-1     AGCAGUACUGAUCGGUUUCGGCUGCGGGCCCGCUGUGACUGGUCACGCAGUUCCCCGCGCCCCUAUGGGGCGCGGGGUC
Sbi-2-1     GGACCGACUGCCGGUUCUUGGCUGCGGGCCCGCUGUGGCUGGUCGCGCAGUUCCCCGCGCCCCUUUGGGGCGCGGGGUC
Ssp-83-1    GACACGGACGCGGGUUUCGUGGAGACCGAGCAGUACUGAUCGGUACCCCUUCCGGGGGCCUCACCGAGGCCCCCGGAUC
Spl-1-1     GACACGGACGCGGGUUUCGUGGAGACCGAGCAGUACUGAUCGGUACCCCUUCCGGGGGCCUCACCGAGGCCCCCGGAUC
Sno-1-1     GUCCGUUGUGGCCGGUCGCGCAGUUCCCCGUGCCCCUUUGGGGCGCGGGGCCUCCGGGAGUCGUCAGGACGACCGGAUC
San-1-1     GUUUCUGCGGGCCGGUUGUGGCUGGCCGCGCAGUUCCCCGCGCCCCUUCCGGGGCGCCCCUUCGGGGACGCCCCGGAUC
Sla-3-1     CGUACUGCGUCUGCGGGCCGGUUGUGGCUGGUCGCGCAGUUCCCCGCGCCCCUUCCAGGCCCCUUCGGGGGCCUGGAUC
Ssp-74-1    GACGCGGACUCGGGUUUCGUGGAGACCGAGCAGUACUAGGUCUCCGUUCUCCGGGGCCUGCGACAGUGGGCCCCGGAUC
Ssp-162-1   NNNNNNNNNNNNNNNNNNNNNNNNNGGCUGGUCGCGCAGUUCCCCGCGCCCCUUCCAGGCCCCUUCGGGGGCCUGGAUC
Ssp-164-1   CGUACUGCGUCUGCGGGCCGGUCGUGGCUGGUCGCGCAGUUCCCCGCGCCCCUUCCAGGCCCCUUCGGGGGCCUGGAUC
Ssp-161-1   CGUACUGCGUCUGCGGGCCGGUCGUGGCUGGUCGCGCAGUUCCCCGCGCCCCUUCCAGGCCCCUUCGGGGGCCUGGAUC
Sgl-2-1     UGAGGGUCGCCGCGCCGUGUCGCGCGGUUGCCCGCACCCCGCUCCGAGCGCGGCCCCUCGCGGGGUCAGCGCCCGGAUC
Ssp-143-1   CUGGUCGCGCCCACGCGACGGAGUCGCAUAUCGACACCGCCCCGCGCCCCUUCCAGGCCCCCUACGGGGGGCCUGGAUC
Ssp-36-1    GUCCGAUGCCGGGUGCGGGGGCGCUGUGCCUGAUCGCGCAGUUCCCCGCGCCCCUUCCGGGGCUGGAAGGCCCCGGAUC
Ssp-200-1   GCGAAGCCGCACAGGGGUACAGCCCCGCGCCCCUUGGGGGUGCGGACCCUCCGGGGCGCUUGCGAAGGCGCCCCGGAUC
Sco-2-1     GCGAAGCCGCACAGGGGUACAGCCCCGCGCCCCUUUGGGGUGCGGACCCUCCGGGGCGCUUGCGAAGGCGCCCCGGAUC
Svi-9-1     GCGAAGCCGCACAGGGGUACAGCCCCGCGCCCCUUUGGGGUGCGGGCCCUCCGGGGCGCUUGCGAAGGCGCCCCGGAUC
Sco-1-1     GACGCGGACGCGGGCUUCGUGGAGACCGAGCAGUACUGAUCGGCACCACUUCCGGGGGCCUCGUGAAGGCCCCCGGAUC
Ssp-128-1   GGUUCGACGGUUCGUUUUCGACUGCCGGCCGGUUGUGGCCGGUCGCGCAGUUCCCCGUGCCCCAUGGGGGCGCGGGGUC
Sol-2-1     GGCUGCGGCUGGUCGCGCAGUUCUCCGAGCCCCUUCGGGGCGCGGAUCCUCCGGGGCGUCUGCGAAGACGCCCCGGAUC
Ssp-72-1    GGCUGUGGCUGGUCGCGCAGUUCCGCGUACCCCUUCGGGGCGCGGAUCCUCCGGGGCGUCUGCGAAGAGGCCCCGGAUC
Ssp-124-1   CGGCUGUGGCCGGUCGCGCAGUUCCCCGCGCCCCUUCGGGCGGGGACCCUCCGGGGCGUGUGAAAAGACGCCCCGGAUC
Sce-2-1     GACGCGGACGCGGGUUUCGUGGAGACCGAGCAGUACUGAUCGGUGCCACCUCCGGGGGUCUCGCGCAGACCCCCGGAUC
Smo-1-1     GGCCCGCUGUGGCUGGUCGCGCAGUUCCCCGCGCCCCUCGGGGCGCGGGCCCUUCCGGGCCCCGAAAGAGGCUCGGAUC
Svi-9-2     GACGCGGACGCGGGUUUCGUGGAGACCGAGCAGUACUGAUCGGUGCCACCUCCGGGGGUCUCGCGCAGACCCCCGGAUC
```

Sso-1-1	UCCCACGAGGUCCGACCGCUCCCGCGGACCGGCUCCGGCCGGCCGCGGAGCUCCCCGCGCCCCCCUGGGGGCGCGGGUC
Soc-1-1	GGCCUAACGGCCUGAUUUUGACUGCCGGCCCGCUGUGGCUGGUCGCGCAGUUCCCCGCGCCCCUUCGGGGGCGCGGGUC
Sni-2-1	GGCCUGACGGUCUGAUUUUGCUGCGGACCGGCUUCGGCUGGUCGCGCAGUUCCCCGCGCCCCUUACGGGGCGCGGGGUC
Svi-11-1	CGGCCCGCUGUGGCUGGUCGCGCCCACGCGGCAGAGCCGCAGAUCAACACCUUCCCGCGCCCCUUCGGGGGCGCGGGUC
Sex-2-1	UCCUGAGGAUCGAUUGACCCCUGCCGGCCGGUUUCGGCUGGUCGCGCAGUUCCCCGCGCCCCUUGUUGGGCGCGGGGUC
Ssp-6-1	GGGCGCGGGUGCGCUGUGCUUGAUCGCGCAGCUCCUCGCGUCCCUUCCGGGAGCGCCCCUUCGGGGCUGCUCCCGGAUC
Ssp-101-1	UCGGCUGUGGCCGGUCGCGCAGUUCCCCGCGCCCCUCUGGGGCGCGGACCCUCCGGGCGUCAGAAAUGGCGCCCGGAUC
Ssp-49-1	UAUCGACACCGCCCCGCGCCCCUGUCGGGAGCGCCCCUGCGGGGCGGCUCCCGACUCCUCCGGGGCGCGUCCCCGGAUC
Sal-11-1	CGGGUGCGGGUGCGCUGCGGUACUCACGCAGCUCCCCGCGCCCCUGUCCGGACCGCCCCUUCGGGGGCGGAUCCGGAUC
Shy-2-1	GGCCCGCCGGACCGUUUUCGGCUGCCGGCCCGCUGUGGCUGGUCGCGCAGUUCCCCGCGCCCCUUGCGGGCGCGGGGUC
Ssp-46-1	GUCCGUUGCGGCCGGUCGCGCAGUUCCCCGCGCGCUUCGGGGCGCGGAUCCUCCGGGGUGUCGAAAGGCGCCCCGGAUC
Ssp-187-1	GUCCGUCGUGGCCGGUCGCGCAGUUCCCCGCGCCCCUCGGGGCGCGGACCCUCCGGGGCGUCGAAAGGCGCCCCGGAUC
Ssp-156-1	UCGCGCAGUUCCCCGCGCCCCUGUCGGGACGCCCUUCGGGCUGCCCCGACACCCUCCGGAGCUGAAGAAGCUCCGGAUC
Sgr-10-1	GUUUCGGCUGGUCGCGCAGUUCCCCGCGCCCCUUCGGGGGCGCGGGUCCUCCAGGACGUCUGUGAAGGCGCUCUGGAUC
Aac-1-1	GUUUCGGCUGGUCGCGCAGUUCCCCGCGCCCCUUCGGGGGCGCGGGUCCUCCAGGACGUCUGUGAAGGCGCCCUGGAUC
Ssp-197-1	CGUACGCGGGUGCGGGUGCGCUGUGCUUGACCGCGCAGUUCCCCGCGCCCCUUUCCGGGCCCCUUCGGGGGUCCGGAUC
Ssp-20-1	CGUACGCGGGUGCGGGUGCGCUGUGCUUGACCGCGCAGUUCCCCGCGCCCCUUUCCGGGCCCCUUCGAGGGUCCGGAUC
Ssp-123-1	CCGGCUGUGGCUGGUCGCGCAGUUCCCCGCGCCCCUUCGGGGCGCGGACCCUCCGGGGCGUCGCAAGGCGCCCCGGAUC
Ssp-141-1	CCGGCUGUGGCUGGUCGCGCAGUUCCCCGCGCCCCUUCGGGGCGCGGACCCUCCGGGGCGUCGCAAGGCGCCCCGGAUC
Ssp-81-1	GUCCGUCGUGGCCGGUCGCGCAGUUCCCCGCGCCCUUCGGGGCGCGGAUCCUCCGGGGCGUCGAAAGGCGCCCCGGAUC
Ssp-113-1	GUCCGUUGUGGCCGGUCGCGCAGUUCCCCGCGCCCUUCGGGGCGCGGAUCCUCCGGGGCGUCGAAAGGCGCCCCGGAUC
Ssp-57-1	GUUUCGGCUGGUCGCGCGGUCCCCCGCGCCCCUUCGGGGGCGCGGGUCCUCCAGGACGUCGGUGAAGGCGUCCUGGAUC
Ssp-149-1	CGGAGCCGCAUAUGGGCACAGCCCCGCGACCCUUCGGGGCCGCGGACCCUCCGGGGCGCCUGACAAGGCGCCCCGGAUC
Sah-1-1	GGUCCUCGACCGGCCACGCGGUUCCCCGCGCCCCUUCUGGGGGCGCGGGUCCUCCGGGCCCCGAGCAGGGGUCCGGAUC
Svi-10-1	GUGCGGGUGCGCUGUGGCUUGUCGCGCAGUUCCCCGCGCCCCUUCCGAGCACGGUCCCUCCGGGACCUUUGCUCGGAUC
Sch-1-1	CGACGCGGACGCCGGGUUCGUGGAGACCGAGCAGUACUGAGUCUCUGCUUCCGGGGUCCGCGUCAGCGGACCCCGGAUC
Sia-1-1	CUCGGGUGCGGGUGCGCUGUGGCUUGUCGCGCAGUUCCCCGCGCCCCUUCCGAGCAGCCCCUUCGGGGCUGCUCGGAUC
Saf-1-1	CGACGCGGACGCGGGCUUCGUGGAGACCGAGCAGUACUGAGUCUCUGCUUCCGGGGUUCGCGUCAGCGGACCCCGGAUC
Ssp-89-1	GGUUCCGUUUCGGCUGGUCGCGCCCACGCGACGGAGCCGCACAUCGGCACCUCCCCGCGCCCCUGUGGGGCGCGGGGUC
Sva-1-1	GGUGCGGGAGCGCUGUGCUUGAUCGCGCAGUUUCCCGCGCCCCUUCCGGACGGGCCCCUGCGGGGGCCUCGUCCGGAUC
Ssp-158-1	CGCCCACGCGACGGAGUCGCAUAUCGAUACCGCCCGCGCCCCUUCCGAGCACGGUCCCUUCGGGACCUGUGCUCGGAUC
Sro-5-1	UGCGGCCCGGCUUCGGCUGGGCGCGCAGUUCCCCGCGCCCCUUACGGGGCGCGGACCCUCCGGGGCGCAGCCCCGGAUC
Ssp-116-1	UACUGAUUGACCCGGCCCGUCCGGCUCGAUCGGCGCUGACCGGUCCCGCCUCCGGGGGCCCCGCGCGGGCCCCCGGAUC
env-22	UGUGGAGACCGAGCAGUACUGAUCGGUCUCCGGCUGCGGCUGGUCGCGCAGCUCCCCGCGCCCCCCGGGGCGCGGGGUC
Sam-1-1	GGGUGCGGGUGGGCUGUGGCUGGUCGCGCAGUUCCCCGCGCCCCUUCCGGACGGGCCCCUUCGGGGCCCCGUCCGGAUC
Ssp-51-1	CGUACCCGGGCGCGGGUGCGCUGUGGCUGAUCGCGCAGUUCCCCGCGCCCCUUUCCAGGCCCCUUCGGGAGCCUGGAUC
Slu-1-1	GCGGAGUCGUAUACCGACACCGCCCCGCGCUCCUUCGGGGGCGCGGAUCCUCCGGGGCGCCCGAAAGGCGCCCCGGAUC
Sna-2-1	GGCCGGCUGUGGCUGGUCGCGCAGUUCCCCGCGCCCCUUACGGGGCGCGGCCCCUCCGGGCCCCACUGGGGCCCGGAUC
Ssp-75-1	CCGUACGCGGGUGCGGGUGCGCUGUGCUUGAUCGCGCAGUUCCCCGCGCCCCUCUCCGGGCCCCUGCGGGGCCCGGAUC
env-23	UGCGGGCGCGCCGCGCUUGAUCGCACGGCUCCCCGCACCUCUUCCGGGCGCGGUCCCCUCGCGGGCCCGUGCCCGGAUC
Ssp-182-1	CGGAGCCGCAUAUGGGCACAGCCCCGCGACCCUUCGGGGCCGCGGACCCUCCGGGGCGCCUGACAAGGCGCCCCGGAUC
Scy-2-1	GGGUGCGGGUGCGCUGUGCUUGAUCGCGCAGUUCCCCGCGCCCCCUCCGGGAGCGCCCCUCGGGGACGGCUCCCGGAUC
Ssp-184-1	CCGUUGUGGCUGGUCGCAGUUCCCCGCGACCCUUCGGGGCCGCGGACCCUCCGGGGUGCCUGACAAGGCGCCCCGGAUC
Svi-4-1	GCGGGUGCGCUGUGCCUGAUCGCGCAGUUCCCCGCGCCCCUUCCGGACGACAGCCCCUUCGGGGCUUGUCGCCCGGAUC
Ssp-163-1	GGGUGCGGGUGCGCUGUGCUUGAUCGCGCAGUUCCCCGCGCCCCUUCCGGGAGCGUCCCUUCGGGGCGGCUCCCGGAUC
Ssp-177-1	UCCGUUGUGGCUGGUCGCACAGUUCCCCGCGCCCCUUACGGGGCGCGGUCCCUUCCGGAGUUGAAAAGAACCCCGGAUC
Sol-1-1	UCCGUUGUGGCUGGUCGCACAGUUCCCCGCGCCCCUUACGGGGCGCGGUCCCUUCCGGAGUUGAAAAGAACCCCGGAUC
Svi-3-1	CGAUGUGGCUGAUCGCGCAGUUCCCCGCGCCCCUUACGGGGCGCGGAUCCUUCCGGGCGUCUGAAAGGACGCCCGGAUC
Szi-1-1	GCCCCCCGGGGCCGGUCGCGCGGGUUCCCGCACCCCUCGGGGCGCGGACCCUCCGGGCGUCCGACAGGGCGCCCGGAUC
Svi-13-2	UUCUGCGGGCCGGUUUCGGCUGGUCGCGCAGUUCCCCGCGCCCCUUCCGGGAGCGCCCCUUCGGGGCUGCUCCCGGAUC
Ssp-120-1	CUUCUGCGGGCCGGUUUCGGCUGGUCGCGCAGUUCCCCGCGCCCCUUCCGGGAGCGCCCUUCGGGGCCGCUCCCGGAUC
Ssp-131-1	UACUGACGGUCGUAGUUCUGCUGCGGGUCCGCUUCGGCUGAUCGCGCAGUUCCCCGCGCCCCUUACGGGGCACGGGGUC
Sca-4-1	GUACUAAGAGCUCGGGUCCGACCCACGGUUCUUGGGCGCGGGUGCGCUGUGGCUUGUCGCGCAGUUCCCCGCGCCCCUU
Sba-1-1	GCGGGCGGAACCGACGACGUCGGGUCCGCCCCGCCCGUGGGGCCUCCGGGACGACGUUCGGCCCGAACGUCGUCCCGGG
Ssp-65-1	GACUUCCGGCCUCCGGGCCGGGCGACCGGCCGGUUCACACCGGCCGGCUCCGGGGCGGCGGUACGCCGACCGUCCCGGG
Sme-2-1	GGCACUCCGACCGGGCGCGGUGCCGACCGCGCCCGGUCGGCCCACGCUUCCGGGGCCGCCGCUGCCGCGCGCUCCGGGU
Sgr-12-3	GGGUCCGUCGGACAGACUGAAGUCGGUCUGGGGCGGUUGAGGGGUCCGGGUGGCUCUUCGGAGUCGUUCGGGUCGAGAC
Sgr-12-4	UGGAAAAGAUGUCCUCAAUCGCCGGACGGGCUUUAAGUGGCUCGCUGGGCAGGCUUCUGGCCGACGGUUGAGGUGAGAC
Ssp-15-1	GGGUCCGUUGGACAGGCUGAAGUCGGUCUGGGGCGGUUGAGGGGUCCGGGUGGCUCUUCGGAGUCGUUCGGGUCGAGAC
Ssp-38-1	GGGUCUGUCGGACAGGCUGAAGUCGGUCUGGGGCGGUUGAGGGGUCCGGGUGGCUCUUCGGAGUCGUUCGGGUCGAGAC
Sal-5-1	GGGUCCGUCGGACAGGCUGCAGUCGGUCUGGGGCGGUUGAGGGGUCCGGGUGGCUCUUUGGAGUCGUUCGGGUCGAGAC
Ssp-77-1	AUGUCCUCAAUCGCCGGACGGGCUUAGUGGGGCUCGUCGGACGGGCUCAGUCGGGCCCGUCUCGCGGUUGAGGUGAGAC
Swa-1-1	GGGUCCGUCGGACAGGCUGAAGUCGGUCUGGGGCGGUUGAGGGGUCCGGGUGGCUCUUCGGAGUCGUUCGGGUCGAGAC
Ssp-30-1	GGGUCCGUCGGACGGGCUGAAGUUGGUCUGGGGCGGUUGAGGGGUCCGGGUGGCUCUUCGGAGUUGUUCGGGUCGGGAC
Ssp-134-1	GGGUUCGUCGGACAGGCUGAAGUUGGUCUGGGGCGGUUGAGGGGUCCGGGUGGCUCUUCGGAGUCGUUCGGGUCGAGAC
Ssp-62-1	UCGGGUGCGUGGCCGGUUGAAAAGAUGUCCUCAAUCGCCGGACGGGCUUUAUCUGGCACGUUUCGCGGUUGAGGGGGAC
Ssp-21-1	UUGAAAAGAUGUCCUCAAUCGCCGGACGGGCUUUAUGUGGCUCGCCGGACAGGCUUUUGGCCGACGGUUGAGGUGAGAC
Ssp-80-1	UUGAAAAGAUGUCCUCAAUCGCCGGACGGGCUUUAGGUGGCUCGCCGGGCAGGCUUUCGGCCGACGGUUGAGGUGAGAC
Sgr-12-5	UUGAGAAGAUGUCCUCAAUCGCCGGACGGGCUUUAGGUGGCUCGCCGGGCAGGCUUUCGGCCGACGGUUGAGGUGAGAC
Sva-2-1	UCCUCAGCUACGGCUGAGGCUUUGUCCUCAAGUGCCGGACGGGCUUGGUUUGGCCUGGGCCGUUCGGCGAACGCACGCU
Ssp-58-1	GUCGGGUCCCGGCCCGCACCCCCGCCCGCGGACCACGCACGGUCCGCGGGCAUUCCGGGGCGGCGCGUGCCGCCUCGGG
Sal-12-1	CUCACGGCUGAGGUGAGGGCGGAGCCGCCCGGCGCUCCGAGCCCGGACGCAAGCCUCCGGGAACCGCCGGUUCCCGGGU
Sfr-1-1	CGGCACCGGGUGCCGGGGGUGCCGCCCCGCCGGGCGCCCGGCCCGGACGCAAGCCUCCGGGAACCGCCGGUUCCCGGGU
Sxi-2-1	CCGGCACCGGGUGCCGGGGUGCCGCCCCGCCGGGCGCGCGGCCCGGACGCAAGCCUCCGGGAACCGCCGGUUCCCGGGU
Ssp-119-1	CGGCGCGGACGACGACGCGGACGCGGGCUUCGUGGAGACCGAGCAGUACUGAAACACCUCCGGAGCGUCAGCUCCGGGU
Ssp-33-1	UCGAGACCGAGCAGUACUGACGUCUCGGGGGCUCUGCCCCCGGACCCCCGCUCCUCAAACGCCGGAGGGGCUGGGUAUU
Ssp-37-1	GGCCGCUGCCGACGCCUUCGGCGCCGACGACGACGUGGACGCGGGUUUUGUGGAGACCGAGCAGUACUGAUCGGUUGUU
Ssp-170-1	GGCCGCUGCCGACGCCUUCGGCGCCGACGACGACGCGGACGCGGGUUUCGUGGAGACCGAGCAGUACUGAUCGGUCGUU
Smi-1-1	GCCGCUGCGGACGCGUUCGGCGCGGACGACGACGCGGACGCGGGUUUCGUGGAGACCGAGCAGUACUGAUCGGUCGUUU
Ssp-138-1	GGCCGCUGCCGACGCCUUCGGCGCCGACGACGACGCGGACGCGGGUUUCGUGGAGACCGAGCAGUACUGAUCGGUCGUU
Sga-2-1	CGACGCCUUCGGCGCCGACGACGACGCGGACGCGGGUUUCGUGGAGACCGAGCAGUACUGACCGUACUGACCGGUCGUU
env-24	CGCCGCCGCCGACGCCUUCGGCGCCGACGACGACGCGGACGCGGGUUUCGUGGAGACCGAGCAGUACUGACCGGUCUUU
Sca-1-1	GGCCGCCGCCGACGCCUUCGGCGCCGACGACGACGCGGACGCGGGUUUCGUGGAGACCGAGCAGUACUGAUCGGUCGUU
Ssp-23-1	GCGCCGACGACGACGCGGACGCGGGUUUCGUGGAGACCGAGCAGUACUGAGCCCUACGGGUCUGUGCUGAACGGUCGUU

```
Ssp-135-1  GGCCGCUGCCGACGCCUUCGGCGCCGACGACGACGCGGACGCGGGUUUCGUGGAGACCGAGCAGUACUGAUCGGUCGAU
Ssp-169-1  CGCCGCCGCCGACGCCUUCGGCGCCGACGACGACGCGGACGCGGGUUUCGUGGAGACCGAGCAGUACUGAUCGGUCUUU
env-30     CGCCGCCGCCGACGCCUUCGGCGCCGACGACGACGCGGACGCGGGUUUCGUGGAGACCGAGCAGUACUGACCGGUCUUU
Ssv-1-1    GCCGCUGCCGACGCCUUCGGCGCCGACGACGACGCGGACGCGGGUUUCGUGGAGACCGAGCAGUACUGAUCGGUCGUUU
Ssp-186-1  GCCGCUGCCGACGCCUUCGGCGCCGACGACGACGCGGACGCGGGUUUCGUGGAGACCGAGCAGUACUGAUCGGUCGUUU
Sda-1-1    ACGCCUUCGGCGCCGACGACGACGCGGACGCGGGUUUCGUGGAGACCGAGCAGUACUGAUCAGUACUUGAUCGGUCGAU
Sgr-12-6   CGCCGCCGCCGACGCCUUCGGCGCCGACGACGACGCGGACGCGGGUUUCGUGGAGACCGAGCAGUACUGAUCGGUUGUU
Ssp-199-1  CGCCGCCGCCGACGCCUUCGGUGCCGACGACGACGCGGACGCGGGUUUUGUGGAGACCGAGCAGUACUGAUCGGUCGUU
env-31     UCGCUGGUCGCGCAGUUCCCCGCGCCCCUUACAGGGCGCGGGGGUCCCGCCCCUCUCCUCGCGGGAGGGGCUGGUUGUU
Ssp-19-1   UGGCCGGUCGCGCAGUUCCCCGCGCCCUUUACAGGGCGCGGGGGUCCCGUCCCUCUCCUCGCAGGAGGGGCUGGUUGUU
Sfu-2-1    CGCCGCCGCCGACGCCUUCGGCGCCGACGACGACGCGGACGCGGGUUUCGUGGAGACCGAGCAGUACUGAUCGGUUGUU
Sau-2-1    AGACGGCCGGAUUGUGACUGCGCGGCCGCUGUGGCUGGUCGCGCAGUUCCCCGCGCCCCUUACGGGGGCGCUGGUUGUU
Sxy-1-1    CGCUGCCGCCGACGCCUUUGGCGCCGACGACGACGUGGACGCGGGUUUCGUGGAGACCGAGCAGUACUGAUCGGUUGUU
Ssp-122-1  CGCCGCCGCCGAUGCCUUUGGCGCCGACGACGAUGUGGACGCGGGUUUCGUGGAGACCGAGCAGUACUGAUCGGUUGUU
Spr-2-1    CGCUGCCGCCGACGCCUUUGGCGCCGACGACGACGUGGACGCGGGUUUCGUGGAGACCGAGCAGUACUGAUCGGUUGUU
Ssp-84-1   ACGCCUUCGGCGCCGACGACGACGCGGACGCGGGUUUCGUGGAGACCGAGCAGUACUGAUCAGUACCUGACCGGUCGUU
Sip-1-1    UCGGCGGUUCGUUUUAGGCUGCGCGACCGCUGUGGCUGGUCGCGCAGUUCCCCGCGCCCCUUACGGGGCGCUGGUUGUU
Sly-2-1    CUGACGGUCUGAUUGUGGCUGCGGGCCGGCUUCGGCCGGUCGCGCAGUUCCCCGCGCCCCUUUCGGGGCGCUGGUUGUU
Ssc-2-1    CGGACAGCUUCGGCGACGAGGACGACCACGGCUUCGCGGAGACCGAGCAGUACUGAGCUUCGCCGCGAGGCGUAGCCAC
Sye-1-1    CCCUUUCCCGGGUACACACCCGCGUCCUUCCGGACGUACCGCGCCCUGCGGGGCGCACUCCGCGCCCUCGGGCGCGUUG
Svi-6-1    GCGGACGCCUUCGGCACCGAGGACGACGCGGACGCCGGGUUCGUCGAGACCGAGCAGUACUGAGGCGACCCGCUGAAUA
Sni-1-1    CCGCCGCCGCCGACGCCUUCGGCGCGGACGACGACGCGGACGCCGGGUUCGUGGAGACCGAGCAGUACUGAAAAGCCUU
Ssu-2-1    GAGCCGUUCUCGGCCCGCGCACCCGGCGUCCGCCCGGUGCGCGGGCCGAGCGCCGUAGGCGCCGGUGGCGCGUACGGAU
Ssu-1-1    GAGCCGUUCUCGGCCCGCGCACCCGGCGUCCGCCCGGUGCGCGGGCCGAGCGCCGUAGGCGCCCGUGGCGCGUACGGAC
Svi-2-1    CUGAGGCGCUGUCCUCAAUCGCCGGACGGGCUUGAUUUUGCUGAGCUUGAUCGAGUCCGUGCGGCGGGCGACGAAAAUU
Sfl-4-1    CCGACCGCCGCCGCCGACGCCUUCGGCGCCGACGACGACGCGGACGCGGGUUUCGUCGAGACCGAGCAGUACUAGAACU
Spu-1-1    GGGCUUCGUCGAGACCGAGCAGUACUAAGCAGUACCCGGUGGUCCGGGGCCCGCGUGGCCCCGGACCACCGUAUGGACU
Ssc-4-2    CGCCUUCGGCGCCGACGACGACGCGGACGCCGGGUUCGUCGAGACCGAGCAGUACUGAGCCGACCGGCUCAGCGACACU
Shy-1-1    CCGACCGCCGCCGCGGACGCCUUCGGCGCGGACGAUGACGCGGAUGCCGGUUUCGUGGAGACCGAGCAGUACUGAUACC
env-32     ......GCCGCCGCCGACGCCUUCGGCGCGGACGACGACGCGGAUGCCGGUUUCGUGGAGACCGAGCAGUACUGAUACC
Sbi-1-1    GUUUCGUCGAGACCGAGCAGUACUGAGCCCUGGGGCCCCGAGCUUCGCGCUCGGGGCCCUGAGCUGAAGCCUGAAGACU
Afl-1-1    UUUCGGCAGUGCUGCUGCCGGGGGUCUGGGGGUUGUCCCCCCGAAGUGACACGCACAGUACUGACCCUCGGAGCACGAC
Ssp-55-1   CGCCGACGCCUUCGGCGCCGACGACGACGCGGACGCGGGUUUCGUGGAGACCGAGCAGUACUGAGCAGUCCCUCGAAUU
Ssp-152-1  CGACGCCUUCGGCGCCGACGACGACGCGGACGCCGGUUUCGUCGAGACCGAGCAGUACUGAGCAGCACCCGUAGAACUU
Ssp-104-1  CGACGCCUUCGGCGCCGACGACGACGCGGACGCCGGUUUCGUGGAGACCGAGCAGUACUGAGCAGCACCCGUUGAACUU
Ssp-166-1  CCGAGCAGUACUGAUUCAUCCCGCCGCCGGCGGCCGAGGCUUUCGCCCAGGUCGCCGGCCGGGCUUGAACCGAACACUU
Ssp-191-1  GCCGCCGCCGACGCCUUCGGCGCCGACGACGACGCGGACGCCGGUUUCGUCGAGACCGAGCAGUACUGAGAAACGACUU
Sxa-1-1    UUCGGCGCCGACGACGACGCGGACGCCGGUUUCGUCGAGACCGAGCAGUACUAGUCCCACCCCGCACCAACGAAGACUU
Sla-1-1    CGAGCAGUACUGAUCCAUCCCGCCGCCGGCGGCCGGGGCCUUCGGCCCGGGUCGCCGGCCGGGCUUGGACCCAAGACUU
Svi-12-3   GAGCAGUACUGAUCCGUCCCGCCGCCGGCGGCCGAGGUCUUCGGACCGCGGUCGCCGGCCGGGCUCGGAACCAAGACUU
Sla-2-2    CGACGCCUUCGGCGCCGACGACGACGCGGACGCCGGUUUCGUCGAGACCGAGCAGUACUAAGCAGCACCCGUAGAUCUU
Ssp-150-1  GAGCAGUACUGAUUCAUCCCGCCGCCGGCGGCCGAGGUCCGCGGACCGUGGUCGCCGGCCGGGCUUGGAACCAAGACUU
Ssp-32-1   CGACGCCUUCGGCGCCGACGACGACGCGGACGCCGGUUUCGUCGAGACCGAGCAGUACUAAGCACCACCCGUAGAACUU
Ska-1-1    GCCGCUGCCGACGCCUUCGGCGCCGACGACGACGCGGACGCCGGUUUCGUCGAGACCGAGCAGUACUAAGAAAAGACUU
Sro-6-1    GCCGCCGCCGACGCCUUCGGCGCCGACGACGACGCGGACGCCGGUUUCGUCGAGACCGAGCAGUACUAAGAGAAGACUU
Ssp-194-1  CGGACGGGGCCCGGCCCCGUCCGGAACCCCCUCGCGGGCGCUCCCGGACACGGAAGCACCCGCGAGGGGCACACGCACU
env-33     CCUUCGGCGCCGACGACGACGCGGACGCCGGCUUCGUCGAGACCGAGCAGUACUGAGCCGCACGGCUCGGGUGACAACU
Ssp-7-1    ACCUCCGCCGCCGACACCUUCGGUGCGGACGACGACGCCGACGCCGGCUUCGUCGAGACCGAGCAGUACUGAUCAGACU
Ssp-140-1  CGCGGGCUUCGUCGAGACCGAGCAGUACUGAUCCGCCGCUGAUCCCCUCACCGGGAUCAGCGCGGUUCAUGCGACAUCU
Svi-1-1    CGACCGCCGCCGCCGACGCCUUCGGCGCCGACGACGACGCGGACGCGGGCUUCGUCGAGACCGAGCAGUACUGAUCACU
Sca-2-1    CGACGCGUUCGGCGCGGACGACGACGACGCCGGCUUCGUCGAGACCGAGCAGUACUGAUCCGCGAGCGGUACUGAGACU
Sts-1-1    CCAGCGCCCCUAUCGGGGCACACCCUCCUGCGCCCGUGACGGGCGCAGGAACCCCCGCUUCUCGCGGGGCACACACACU
Ssp-25-1   ACCGCCGCCGCCGACGCCUUCGGCGCCGACGACGACGCGGACGCCGGUUUCGUGGAGACCGAGCAGUACUGAUGUGACU
Ska-1-2    CGCCGCCGCCGACGCCUUCGGCGCGGACGACGACGCCGACGCCGGCUUCGUCGAGACCGAGCAGUACUAAGAAAUGACU
Smo-2-1    UUCGGCCGGGCUGGAUGAAUGAGCCUCGCCGAGACUGGAAGCGUCGGGGUAGCCCGGCCGCUUCCAGUGCGAACGAUCU
Ssp-70-1   GUUUCGUGGAGACCGAGCAGUACUGAGCGUGACCGACUGGGGUCCGGGCGGUGUCCCGGGCCCCAGCCGUGUGAAAUCU
Sba-2-1    NNNNNNNNNNNNNNNNNNNNNNNNNNNNNNNNNNNNCGUCGGGCAGGCUGAAUUUGGCUGAUGUCCGGCAGCUAUGACU
Sme-1-1    AUCGCCGGACGGGCUUGAUUUUGGCUGAUGUCCGGCGUGAGCGUCGGGCGGGCUUGAAUGCCGUCCGGCACGUAUGACU
Sgr-11-1   AUCGCCGGACGGGCUUGAUUUCGGCUGAUGUCCGGCGUGAGCGUCGGGCGGGCUUGAAUGCCGUCCGGCACGUAUGACU
Sgl-1-1    UUGAGGGUGCCCCCGUGCCGGGGCUGAGGCUUUGUCCUCAAUCGCCGGACGGGUUUGAUUUUGUCCGGCAGCUAUGACU
Ssp-76-1   GCCGGACGGGCUUGAUUUUGGCUGAUGUCCGGCGUGAGCGUCGGGCGGGCUUGAAUGCGGCCGUCCGGCACGUAUGACU
Sgr-12-7   AAUCGCCGGACGGGCUGAAUUUGGCUGAUGUCCGGCGCGAACGUCGGGCGGGCUUGAACGCCGUCCGGCAGCUAUGACU
Sgl-3-2    CUCGAUCGCCGGACGGGCUUGUUUUCGGCUGAUGUCCGGCGAACGUCGGGCGGGCUGAUUCUGGCUCGCGCCGAAGACU
Sgr-4-1    UCGCGGGUGCCCCGGGCCGGGGCUGAGGCUCUGUCCUCAAACGCCGGGCGGGCUUGAUCGCCGCCCGGCAGCCAUGACU
San-2-1    CCGGACGGGCUUGAUUUCGGCUGAUGCCCGGCGAAAGUCGGGCGGGCUGGAAACAUCGAGCCCGACCGCACGUAUGACU
Sgr-3-1    UCGCGGGUGCCCCGGGCCGGGGCUGAGGCUUUGUCCUCAAACGCCGGGCGGGCUUGACCGCCGCCCGGCAGCCAUGACU
Ssp-9-1    UUGGCUUAUGUCCGGCGAGCGUCGGGCGGGCUUGAGUGUGGCUGAUGCCGGACUCCGAGCCCGAACGGCAGCUAUGACU
Seu-1-1    CCGGACAGGCUUGAUUUUGGCUGAUGUCCGGCGAACGUCGGGCGGGCUUGAAAGCCGUGCCCGACCGGCACGUAUGACU
Ssp-82-1   GGUUUUGUGGGUGCCCCCGGGCCGGGCUGAGGCUUUGUCCUCAAUCGCCGGACGGGCUUGCCGUCCGGCAGCUAUGACU
Sal-12-2   CGGACGGGCUUGAAUUUGGCUGAUGUCCGGCGUGAACGUCGGGCAGGCUGAAUUUGGCUGAUGUCCGGCAGCUAUGACU
Ssp-48-1   UCAAUCGCCGGACGGGCUUGAUUUUGGCUGAUGUCCGGCGAACGUCGGGCGGGCUGAUUCCGGCUCACGUCCGAAGACU
Sha-1-1    GAGGCCGUGUCCUCAGGCGCCGGACGGCCCGGUCUCGUGGCCCCCUGGGCCCGAUGACGGCCGUGCGGCACGCACGACU
Ssp-45-1   AGACCGAGCAGUACUGAGCCGUUCCCCGGCCGGGGCGCCCUGUGAGGGGCGUCCCCGUGCCGGGUGGGGCCGCACGACU
Ssp-71-1   GAGGCCGUGGCCUCAGGCGCCGGACGGACCGGUCUCGUGGCCCCCUGGGCCCGAUGACGGCCGUGCGGCACGCACGACU
Ssp-175-1  GACCGAGCAGUACUGACCGUGUUCCGGCAGGGGCGCCCGUUCUGACGGGUGCCCCCGUGCCGGAUCCGGCAGCAGGACU
Ssp-188-1  CGAGGCCGUGGCCUCAGGCGCCGGACGGACCGGUCUCGUGGCCCCCUGGGCCCGAUGACGGCCGUGCGGCACGCACGACU
Ssp-52-1   UUCGGCGCCGACGACGACGUGGACGCCGGGUUCGUGGAGACCGAGCAGUACUGAGCCGGUCGCACGAUCGCACGAAUCU
Sri-4-1    CGCCUUCGGCGCCGACGACGACGCGGACGCCGGUUUCGUGGAGACCGAGCAGUACUGAGCCCAAGGGCUCGCGCAAUCU
Ssp-145-1  GAGACCGAGCAGUACUGAGCCUGCGGGUUCGUUGCGGCUGAGGCUUUGUCCUCAAUCGCCGGACGGGCUGAUUUGAUCU
env-34     GCUUCGUGGAGACCGAGCAGUACUGAGUUUGUCCGGCGGAGGUCCGGGGUGUCCCCCCGGGCCUCCGCCGUGUGAGUCU
Sly-1-1    GUUUCGUGGAGACCGAGCAGUACUGAGUUUGUCCGGCUGGGGUCCGGGGCGCUGCCCCGGACCCCAGUCGUGUGAAUCU
Ssp-142-1  GGUUCGUGGAGACCGAGCAGUACUGAGUUCGACCGGCAGGGGUCCGGGAGCCUUUCCCGGGCCCUUGCCGUGUGCAUCU
Ssp-155-1  GGGUUCGUGGAGACCGAGCAGUACUGAGUUCGACCGGCAGGGGUCCGGGAGCCUUCCCGGGCCCCUGCCGUGUGCAUCU
Ssp-53-1   CUCGAACGCCGAACGGGCUGAUGCAUGAACCCCCUGCUGGGGGCCCGGGGGUCGUCCCCCGGGCGAAUGCAGUGUGACU
```

```
Sat-2-1      AGACCGAGCAGUACUGAGCCGCAUCCCGGACGGGGGUGCCUGAUCUUCCAGGUGCCCCGUGCCGGGCCAGUAGGGACU
Ssp-95-1     GAGCAGUACUGAUCCGCUUCCCGGCCAGGGGCGCCCGCUGACGCGGAGCCCCUGGGCCGGUCGCGGACCGUUCGAGACU
Sfl-1-1      GAGCAGUACUGAUCCGCUUCCCGGCCAGGGGCGCCCGCUCACGCGGAGCCCCUGGGCCGGUCGCGGACCGUUCAAGACU
Jpa-1-1      GAGCAGUACUGAUCCGCUUCCCGGCCAGGGGCGCCCGCUGACGCGGAGCCCCUGGGCCGGUCGCGGACCGUUCAAGACU
Sfl-8-1      GAGCAGUACUGAUCCGCUUCCCGGCCAGGGGCGCCCGCUCACGCGGAGCCCCUGGGCCGGUCGCGGACCGUUCAAGACU
Ssp-154-1    GAGCAGUACUGAUCCGCUUCCCGGCCAGGGGCGCCCGCUCACGCGGAGCCCCUGCGCCGGCCGCGGACCGUUCAAGACU
Sce-1-1      GACGCCUUCGGCGCCGACGACGACGCGGACGCCGGGUUCGUGGAGACCGAGCAGUACUGAGCCGCGGGCUCGUGAAUCU
Ssc-5-1      CGCGGGUUUCGUGGAGACCGAGCAGUACUAAGGGUUCCGGGUCCGGGGCUCGGACAGCCCGGACCGGCCUGAUUUCGCU
Ssp-144-1    UCCGGGGCGCCCCCGCCGGGGCCGAGGGCUUCGUCCUCGAUCGCCGGCCGGGCUCGGACGAGCCCGGCCGGCAAGAACU
Ssp-54-1     CGAGGGCUCUGUCCUCAGGCGCCGGACGGGCUUGAUAUUCGGGCUGAUGUCCGAUGACAGCCGUAAGGCUCGUAAGACU
Sgr-13-1     GCGCCCCGGGCCGGGGCUGAGGCUUGGUCCUCAAUCGCCGGACGGGCUUGAAUUCGGCCGCUGUCCGGCACGUACGACU
Sfl-9-1      GCGCCCCGGUCCGGGGCUGAGGCUUUGUCCUCAUUCGCCGGACGGGCUUGAAUUCGGCCGCUGUCCGGCACGUACGACU
Sro-4-1      GUGCCCCGUGCCGGGCUGAGGCUUUGUCCUCAAUCGCCGUUCGGGCUUGAUAUAUCAAGCCCGAACGGCAGCUAUGACU
Sal-12-3     AUCGCCGGACGGGCUUGAUUUUGGCUGAUGUCCGGCGUGGACGUCGGGCGGGCUUGAACAUCGUCCGGCAGCUAUGACU
Ssp-174-1    GCGCCCCGGGCCGGGGCUGAGGCUUUGUCCUCAAUCGCCGGACAGGCUUGAAUUCGGCCGCUGUCCGGCACGUACGACU
Ssp-129-1    GCGCCCCGGGCCGGGGCUGAGGCUUUGUCCUCAAUCGCCGGACAGGCUUGAAUUCGGCCGCUGUCCGGCACGUACGACU
Spu-2-1      GCGCCCCGGGCCGGGGCUGAGGCUUUGUCCUCAAUCGCCGGACAGGCUUGAAUUCGGCCGCUGUCCGGCACGUACGACU
Svi-8-1      GCGCCCCGGGCCGGGGCUGAGGCUUUGUCCUCAUUCGCCGGACAGGCUUGCAUUCGGCCGCUGUCCGGCACGUACGACU
Ssp-66-1     GUGCCCCGUGCCGGGCUGAGGCUUUGUCCUCAAUCGCCGGACGGGCUUGAUUUAUCAAGCCCGAACGGCAGCUAUGACU
Ssp-94-1     GGCGCCCCGGGCCGGGCUGAGGCUUUGUCCUCAAUCGCCGGACAGGCUUGAAUUCGGCCGCUGUCCGGCACGUACGACU
Ssp-17-1     CGAUGCCUUCGGUGCCGACGACGACGCGGACGCCGGUUUCGUGGAGACCGAGCAGUACUGAGCACCACCCCGUACGACU
Ssp-193-1    CGACGCCUUCGGUGCCGACGACGACGCGGACGCCGGUUUCGUGGAGACCGAGCAGUACUGAGCACCACCCCGUACGACU
Sat-1-1      CCUCAACAGCCGGACGGGCUUGAUUUCGGCCGAUGACUGAUCUCAUCGGCCGAGAUGGUGUCCGGAUGGCCCAUGGACU
Ssp-44-1     UUCGAGGGUGCCCCGGGCCGGGGCUGAGGCUUUGUCCUCAAUCGCCGGACGGGCCUGAUGUCGUCCGGCAGCCAUGACU
Scy-1-1      GGUGCCCCGGGCCGGGGCUGAGGCUUUGUCCUCAAUCGCCGGAUGGGCUGAAUGUGGCUGAUGUCCGGCAGCUAUGACU
Sfu-1-1      GGUUUCGAGGGUGCCCCGGGCCGGGGCUGAGGCUUUGUCCCCAAUCGCCGGACGGGCUUGAUGUCCGGCAGCUAUGACU
Sal-7-1      GUGCCCCCGUGCCGGGAUGAGGCUUUGUCCUCAAUCGCCGGACGGGCUUGAAUUUGGCUGAUGUCCGGCAGCUAUGACU
Ssp-168-1    GUGCCCCCGUGCCGGGAUGAGGCUUUGUCCUCAAUCGCCGGACGGGCUUGAAUUCGGCUGAUGUCCGGCAGCUAUGACU
Ssp-97-1     UAUCCGGGCGCCCGGACCGGGGUGAGGCUCCGGCCUCGAUAGCCGGACGGGCUCGAUGAGCCCCGACGGCCCAUGAUCU
Ssp-146-1    UUUCCGGGCGCCCCGACCGGGGUGAGGCUCCGGCCUCGAUCGCCGGACGGGCUCGAUGAGCCCCGACGGCCCAUGAUCU
Sod-1-1      CAGUACUGAGCGGCCCCGGGCCCGGGGCUGGGGCUUUGUCCUCAAUCGCCGGACGGGCUUGAUUUCGUCGCUUGAUUUU
Sri-4-2      GGCCCUGGGGCCUCGUCCUCAAUCUCCCCAGCUGCCGCUGGGAGGUGCCCCCAGGACGGGCUUGAAAUGAAAGAGACU
Sgr-6-1      CGGCCAGGUUUGAUGGAUGAAGCCGGCCGAAGCUGGAAGCGGCGGGGGCGACCCGGCCGCUUCCAGCGCGAUAAGAACU
Sri-4-5      CCGCCGUGUUCGGGUGCAGGCUUGAUCGGCCCUGGGGCCUCGUCCUCAAACGCCGGACGGGCUUGAAAUGAAAAAGACU
Sri-2-1      CAAACGCCGGACGGGCUGGAUAGGGAUGAGCUGCGCUCAGCCACGACAGCCCAGCGGGCCCCCGGCCCGCGUGCAAUCU
Ssp-90-1     GCCCGCCGUGUUCGGGUGCAGGCUUAUCGGCCUGCGGCCUCGUCCUCAAACGCCGGACGGGCUUGAAAUGAAAAAGACU
Sri-4-18     CGGCCAGGUUUGAUGGAUGAAGCCGGCCGAAGCUGGAAGCGGCGGGGGCGACCCGGCCGCUUCCAGCGCGAAAAGAACU
Ssp-185-1    CGGCCCUGAGGCCUCGUCCUCAAACUCCCCCAGCUACCGCUGGGAGGUGCCCCCAGGACGGGCUUGAAUCGAAAGAUCU
Sri-4-19     CGGCCAGGUUUGAUGGAUGAAGCCGGCCGAAGCUGGAAGCGGCGGGGGCGACCCGGCCGCUUCCAGCGCGAAAAGAACU
Ssp-5-1      UUGAUCUUCGGGCUGAGGCCCGAGAUUUCGGGUGCGGAUGCCGACUCGUGAGUGAGUCGGUCCGGCGACAGCACACACU
Ssp-29-1     UGUGGGCGCCCCGGCCCGGGUGAGGGCUUUUGUCCUCAAUCGCCGGACGGGCUUGAUCGGUCCGGCGCGAUCACACACU
Ssp-26-1     GCCGGACGGGCUUGAUUUUCGCCCGAUGCCGCGUGCGGAUCCGACUCGCGAGAGUCGGUCCGGCGACAGCACACACACU
Aat-1-1      GCAACGACCGCCGCGUCGUUUCUCAGUACCGCGGGGCUGUGGGGGUCGUCCCCCACAGGUGACACGCCACACGCAACAC
Ssp-117-1    GUGUAGUUGCCCCUCGGGGCAGAAUGGAAGCCUCCCAGGGCGGCAGCCCUGGGGACUCCGGGGCUCGGCCCCGGGACAC
Ssp-198-1    GGCGCCGACGACGACGUGGACGCGGGCUUCGUCGAGACCGAGCAGUACUGAGCCCCCCGAGGCGCAGCCUCGGACCCC
Ssp-115-1    UGGUCGCGCAGUUCCCCGCGCCCCUCUCGGGGCGCAUCCCCGUCGGGCGUUCCGUCCGGCGCGGGUUCGUGAGCCCGGU
Psi-1-1      CUCCCGACACGCUGCCGGCCUCGCGGUCGAGGUCCGCUGAGCGAGAGAUCGCUCGGCGGCCAGACCACCCACCCAAUGG
Ssp-4-1      CGCCGGGUUCGUCGAGACCGAGCAGUACUGAGCGACUGCCGACCGGCGGGGGCUCCGCCCGGAGCCCCCGCGAACCCCU
Ssp-201-1    CCUUCGGGCCCGCUCCCUGGCCGGGAAUCCGCCCGGUCCGGGAUUCCCCUCGGCCCUCGCGGACGAGGGGGCACCCCCU
Aal-1-1      CUCCUUCGACGAAGAGGACGAGAGCUUCGCGGAGACCGAGCAGUACUAACCCUCAACACAGUCCGCAACAACCGCCCCA
Sal-13-1     CGACCCGAGCGCGGCGGCCGACGCCUACAGCGACGACGACCAGAGCUACGCCGAGACCGAGCAGUACUAGAAACCCGAG
Ari-1-1      CGACCCGAGCGCGGCGGCCGACAACUACGGCGACGACGACCAGAGCUACGCCGAGACCGAGCAGUACUAGAACCCAGAU
Ssp-40-1     CGCUUCGCGCUCGUCCUCAAGCGCCGGACGGGCUGGAUUCUUUUCCGCCCGUACCGGAUUCCGGACUUCGGGCCGGAUC
Ssp-39-1     ..........................GGACGGGCUGGAUUCUUUUCCGCCCGUACCGGAUUCCGGACUUCGGGCCGGAUC
Ssp-42-1     CGCUUCGCGCUCGUCCUCAAGCGCCGGACGGGCUGGAUUCUUUUCCGCCCGUACCGGAUUCCGGACUUCGGGCCGGAUC
Ssp-47-1     CGCUUGGCGCUCGUCCUCAAGCGCCGGACGGGCUGGAUUCUUUUCCGCCCGUACCGGAUUCCGGACUUCGGGCCGGAUC
Ssp-41-1     CGCUUCGCGCUCGUUCUCAAGCGCCGGACGGGCUGGAAUUUUUCCCGCCCGUGCCGGAUUCCGGACUUCGGGCCGGAUC
Ssp-125-1    GCGCUUCGCGCUCGUCCUCAAGCGCCGGACGGGCUGGAUUCUUCCCGCCCGCACCGGAUUCCGGACUUCGGGCCGGAUC
Nsp-3-1      GACCCGCACGCCGCGCUCGCGGCGUACGGCGAUGACGACGACGACGCCUUCGUCGAGGACGAGCAGUACUGAUCCCAAA
Nsp-2-1      ACGACGCGUUCGUCGAGGACGAGCAGUACUGAUCCGCUCCUGAGGCAGGCGCUCGUGCGCCUGCCUCAGACCAGCCAAA
env-35       GACGCGUUCGUCGAGGACGAGCAGUACUGAGCACCACCUGAUCGAUCCCGCCGGGGCCGCAGCCGCGGCCCCGGCCAAA
env-37       GACCCGCAGGCUGCGCUCGCGGCGUACGGCGACGACGACGACGACGCGUUUGUCGAGGACGAGCAGUACUGAUCCCCAA
env-38       GACCCGCAGGCUGCGCUCGCGGCGUACGGCGACGACGACGACGACGCGUUUGUCGAGGACGAGCAGUACUGAUCCCCAA
env-39       GACCCGCAGGCUGCGCUCGCGGCGUACGGCGACGACGACGACGACGCGUUUGUCGAGGACGAGCAGUACUGAUCCCCAA
env-40       ...............................................GCGUCGAGGACGAGCAGUACUGAGAACGAAA
Nlu-1-1      UCCGCCGCCCUCGCCGCCUACGGUGACGAUGACGACGACGCGUUCGUCGAGGACGAGCAGUACUGAGUUUCCACCAAAA
env-41       CGUCGAGGACGAGCAGUACUGAGUUGUAGCGCCGGGCCAGGCGGCUUCGCCGCGGCCCGGCGUUAACCCAUAAAGAAAA
Nha-1-1      CGAGCAGCUCUGACUGGAUUGCGCGAGCGUGCCGGCCGAGCGCUGUGGUCGGCACCUCGCACCAGUCACACAAGUGAAA
Sst-1-1      CGGGUGCGGGUGCGCUGUGCUUGAUCGCGCGGUUCCCCGCACCCCUUCCGGGCAGGCCCUUCGGGGCCCUGCCCGGAUC
Sle-1-1      GGGUGCGGGUGCGCUGUGCUCGACCGCGCAGUUCCCCGCGCCCCCUCCGGGAGCGCCCCUCGGGGACGGCUCCCGGAUC
Sal-2-1      GGGUCCGUCGGACAGGCUGAAGUUGGUCUGGGGCGGUUGAGGGGUCCGGGUGGCUCUUUGGAGUCGUUCGGGUCGAGAC
Ssp-24-1     GCGGGCCGGCUGCGGCCGGCCGUGCGGCUCCCCGCGCCCCUUUCGGGGCACCCCGCGCCGCACGGGCGCGGGGCCGACU
Scl-1-1      GCGGGGACGGUGCGCCGCCGUUGUGGCUGAGCGCGCGGGUCCCUGCGCCCCUGGCGGGGCCUUCGGGCCCCGGCACACU
Ssc-1-1      GGACCGACUGCCGGUUCUUGGCUGCCGGCCCGCUGUGGCUGGUCGCGCAGUUCCCCGCGCCCCUAUGGGGCGCGGGGUC
Sde-1-1      GGUUCGUGGAGACCGAGCAGUACUGAGUUUGUCCGGCUGGGGUCCGGGGCCGCGUGCCCCGGGCCUCAGCCGUGAGCCU
             ...............................................................................
             ...............................................................................
             ●●●●●●●●●●●●●●●●●●●●●●●●●●●●●●●●●●●●●●●●●●●●●●●●●●●●●●●●●●●●●●●●●●●●●●●●●●●●●●●
```

alignment positions 80 · · · 259

```
.<<<<<<<<<<<<<<<<.......................>>>>>>>>>>>>>>>>.......<<<..<<<<<<<..........>>>>>>>..>>>...........................................................................................
.2222222221110000.......................0000111222222222.......122...221100..........001122...221...........................................................................................
YC●●●●●R●●-------R●YY-------------------------●●Y●●●●●G●●●----CUGA-C●CCGGUACCUGAYACGGCCGG●G--CAGR●●●●------------------------------●AGGAGARR●●●○-----AUGCC●●R●CCCRCCAAGGGYGCCCGYCU
5'                                                                                           3'
```

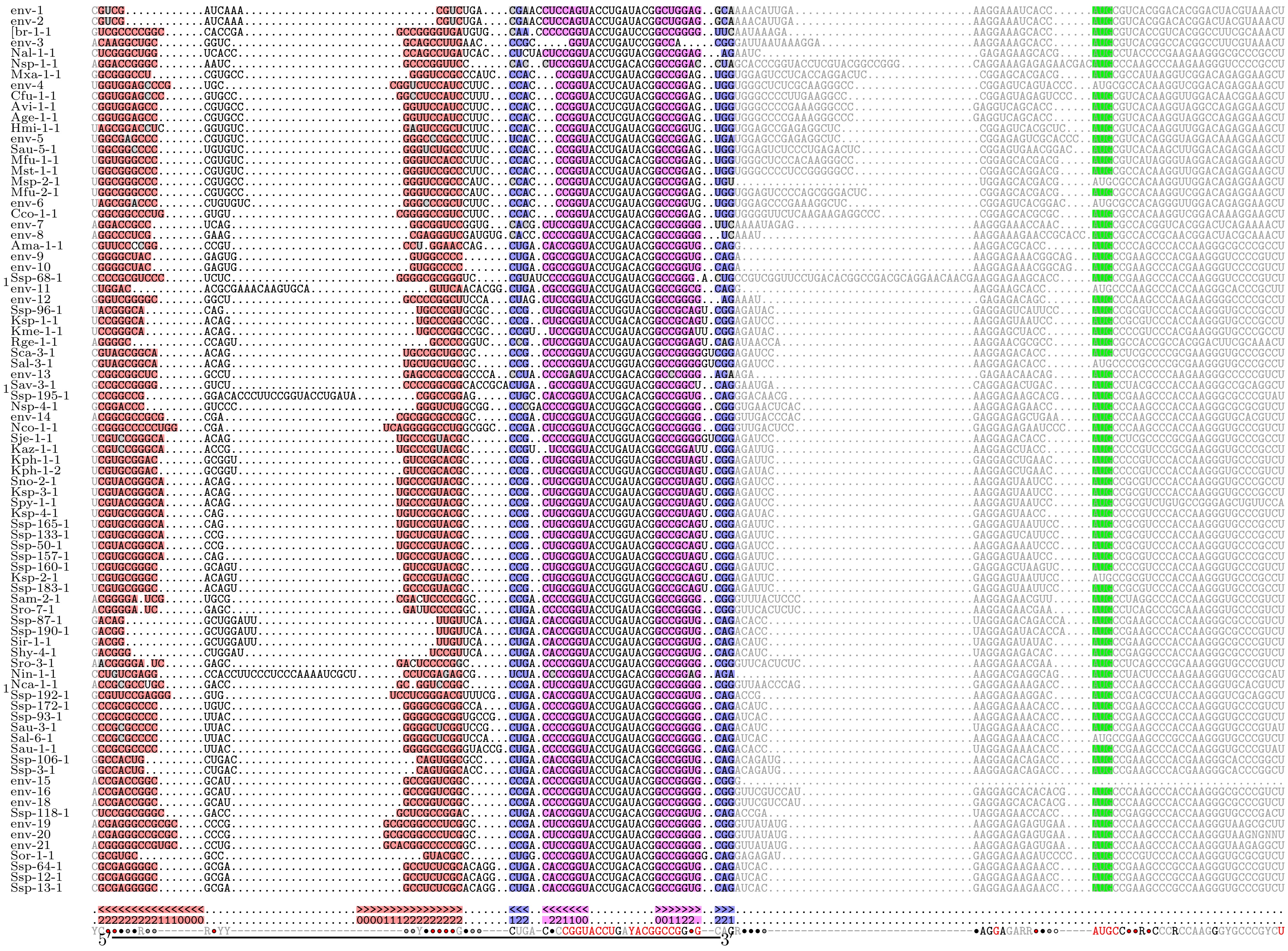
env-1
env-2
[br-1-1
env-3
Nal-1-1
Nsp-1-1
Mxa-1-1
env-4
Cfu-1-1
Avi-1-1
Age-1-1
Hmi-1-1
env-5
Sau-5-1
Mfu-1-1
Mst-1-1
Msp-2-1
Mfu-2-1
env-6
Cco-1-1
env-7
env-8
Ama-1-1
env-9
env-10
Ssp-68-1
env-11
env-12
Ssp-96-1
Ksp-1-1
Kme-1-1
Rge-1-1
Sca-3-1
Sal-3-1
env-13
Sav-3-1
Ssp-195-1
Nsp-4-1
env-14
Nco-1-1
Sje-1-1
Kaz-1-1
Kph-1-1
Kph-1-2
Sno-2-1
Ksp-3-1
Spy-1-1
Ksp-4-1
Ssp-165-1
Ssp-133-1
Ssp-50-1
Ssp-157-1
Ssp-160-1
Ksp-2-1
Ssp-183-1
Sam-2-1
Sro-7-1
Ssp-87-1
Ssp-190-1
Sir-1-1
Shy-4-1
Sro-3-1
Nin-1-1
Nca-1-1
Ssp-192-1
Ssp-172-1
Ssp-93-1
Sau-3-1
Sal-6-1
Sau-1-1
Ssp-106-1
Ssp-3-1
env-15
env-16
env-18
Ssp-118-1
env-19
env-20
env-21
Sor-1-1
Ssp-64-1
Ssp-12-1
Ssp-13-1
5′
3′

```
Ssp-61-1   CCCGGGGGGCG......CCU...........................CGCCCCCCGGACC....CCGAUCACCGGUACCUGAUACGGCCGGUG..CGGUA...................................AAGGAGAAACACC.....AUGCCGAAGCCCACCAAGGGCGCCCGACU
Ssp-59-1   CCCGGGGGGCG......CGA...........................CGCCCCCCGGACC....CCGAUCACCGGUACCUGACACGGCCGGUG..CGGUA...................................AAGGAGAAACACC.....AUGCCGAAGCCCACCAAGGGCGCCCGACU
Ssp-16-1   CCCGGGGGGCG......CUG...........................CGCCCCCCGGACC....CCGAUCACCGGUACCUGACACGGCCGGUG..CGGUA...................................AAGGAGAAACACC.....AUGCCGAAGCCCACCAAGGGCGCCCGACU
Ssp-69-1   CCCGGGGGGC.......ACUGC..........................GCCCCCCGGACC....CCGAUCACCGGUACCUGACACGGCCGGUG..CGGUA...................................AAGGAGAAACACC.....AUGCCGAAGCCCACCAAGGGCGCCCGACU
Ssp-56-1   CUCGGGGGGC.......CACC...........................GCCCCCCGAACC....CCGAUCACCGGUACCUGACACGGCCGGUG..CGGUA...................................AAGGAGAAACACC.....AUGCCGAAGCCCAGCAAGGGCGCCCGUAU
Hcr-1-1    ACGAUC...........GUCU...............................GAUCGGC.....CCGA.CCCCGGUACCUGAUACGGCCGGGG..CGGGUUAUCCA.............................AAGGAGCAUGUC......AUGCCCAAGCCCACCAAAGGCGCCCGUCU
Msp-1-1    ACGACCGG.........UACG.............................CCGGUCGGC.....CCGA.CCCCGGUACCUGACACGGCCGGGG..CGGGUUAUCCAU............................GAGGAGCAUCAC......AUGCCCAAGCCCACCAAGGGCCCCCGCUU
Mro-1-1    ACGACCGG.........UACG.............................CCGGUCGGC.....CCGA.CCCCGGUACCUGACACGGCCGGAG..CGGGUUAUCCAU............................GAGGAGCAUGAA......AUGCCCAAGCCCACCAAGGGCGCCCGUCU
Ssp-171-1  ACCCU.CCGGC......AAAA..........................GUCGGUAGGGA......CUGC.CACCGGUACCUGACACGGCCGGUG..CAGCACA.................................GAGGAGAAACACC.....AUGCCGCGUCCCGCUAAGGGCGCCCGCCU
Ssp-43-1   UCGA.CGGGC.......GACC...........................GCCCGCUCGGAUA...CUGA.CCCCGGUACCUGGUACGGCCGGGG..CAGACAUC................................AAGGAGAAACACC.....AUGCCGCGUCCCGCGAAGGGUGCCCGCCU
Svi-13-1   UCGA.CGGGC.......GACC...........................GCCCGCUCGGAUA...CUGA.CCCCGGUACCUGGUACGGCCGAGG..CAGACAUC................................AAGGAGAAACACC.....AUGCCGCGUCCCGCGAAGGGUGCCCGCCU
Ssp-73-1   UCGA.CGGGC.......AUCC...........................GCCCGCUCGGAUA...CUGA.CCCCGGUACCUGAUACGGCCGGGG..CAGACACC................................UAGGAGAAACACC.....AUGCCGAAGCCCACCAAGGGUGCCCGUCU
Ssc-3-1    UCGA.CGGGC.......AUCC...........................GCCCGCUCGGAUA...CUGA.CCUCGGUACCUGAUACGGCCGGGG..CAGACACC................................UAGGAGAAACACC.....AUGCCGAAGCCCACCAAGGGUGCCCGUCU
Kau-1-1    CCGA.CAGGC.......GACC...........................GCCUGCUCGGAUA...CUGA.CUCCGGUACCUGAUACGGCCGGGG..CAGACACC................................UAGGAGAAACACC.....AUGCCGAAGCCCACCAAGGGUGCCCGUCU
Sgr-1-1    UCGA.CAGGC.......GACC...........................GCCUGCUCGGAUA...CUGA.CUCCGGUACCUGAUACGGCCGGGG..CAGACACC................................UAGGAGAAACACC.....AUGCCGAGGCCCACCAAGGGUGCCCGUCU
Sto-1-1    CCGA.CAGGC.......GACC...........................GCCUGCUCGGAUA...CUGA.CUCCGGUACCUGAUACGGCCGGGG..CAGACACC................................UAGGAGAAACACC.....AUGCCGAGGCCCACCAAGGGUGCCCGUCU
Ssp-114-1  CCGA.CAGGC.......GACC...........................GCCUGCUCGGAUA...CUGA.CUCCGGUACCUGAUACGGCCGGGG..CAGACACC................................UAGGAGAAACACC.....AUGCCGAAGCCCACCAAGGGUGCCCGUCU
Ssc-4-1    CCGA.CAGGC.......GACC...........................GCCUGCUCGGAUA...CUGA.CUCCGGUACCUGAUACGGCCGGGG..CAGACACC................................UAGGAGAAACACC.....AUGCCGAAGCCCACCAAGGGUGCCCGUCU
Ssp-1-1    UCGA.CGGGU.......AAUC...........................GCCCGCUCGGACA...CUGA.CCUCGGUACCUGAUACGGCCGGGG..CAGACACU................................UAGGAGAAAGACC.....AUGCCGAAGCCCACCAAGGGUGCCCGUCU
Sau-4-1    UCGA.CGGGU.......AAUC...........................GCCCGCUCGGACA...CUGA.CCUCGGUACCUGAUACGGCCGGGG..CAGACACC................................UAGGAGAAACAUC.....AUGCCGAAGCCCGCCAAGGGUGCCCGUCU
Spr-1-1    ACCCUCC.GGC......GACC..........................GCCAGGAGGGAA.....CUGA.CACCGGUACCUGACACGGCCGGUG..CAGAUCAC................................AAGGAGAAACCCC.....AUGCCGCGUCCCACCAAGGGAGCCCGUUU
Ssp-189-1  ACCCCCUUGC.......GACC...........................GCUUGGGGGAA.....CUGA.CUCCGGUACCUGACACGGCCGGUG..CAGAUAUG................................AAGGAGUAACA.......AUGCCGCGUCCCGCAAAGGGUGCCCGCCU
Sfl-6-1    ACCCUCUCGC.......GACC...........................GCCUGAGGGAA.....CUGA.CACCGGUACCUGACACGGCCGGUG..CAGAUGUG................................AAGGAGUAACA.......AUGCCGCGUCCCGCAAAGGGUGCCCGCCU
Ssp-103-1  ACCCUCUCGC.......GACC...........................GCCUGAGGGAA.....CUGA.CACCGGUACCUGACACGGCCGGUG..CAGAUAUG................................AAGGAGUAACA.......AUGCCGCGUCCCGCAAAGGGUGCCCGCCU
Ssp-111-1  ACCCUCUCGC.......GACC...........................GCCUGAGGGAA.....CUGA.CACCGGUACCUGACACGGCCGGUG..CAGAUGUG................................AAGGAGUAACA.......AUGCCGCGUCCCGCAAAGGGUGCCCGCCU
Ssp-159-1  CCCCUCUCGC.......GACC...........................GCCUGAGGGAA.....CUGA.CUCCGGUACCUGACACGGCCGGUG..CAGAUUUG................................AAGGAGUAACA.......AUGCCGCGUCCCGCAAAGGGUGCCCGCCU
Ser-1-1    ACCCUCUCGC.......GACC...........................GCCUGAGGGAA.....CUGA.CUCCGGUACCUGACACGGCCGGUG..CAGAUUUG................................AAGGAGUAACA.......AUGCCGCGUCCCGCAAAGGGUGCCCGCCU
Sya-1-1    ACCCUCUCGC.......GACC...........................GCUUGAGGGAA.....CUGA.CUCCGGUACCUGACACGGCCGGUG..CAGAUUUG................................AAGGAGUAACA.......AUGCCGCGUCCCGCAAAGGGUGCCCGCCU
Svi-12-1   CCCCUCUCGC.......GACC...........................GCUUGAGGGAA.....CUGA.CUCCGGUACCUGACACGGCCGGUG..CAGAUUUG................................AAGGAGUAACA.......AUGCCGCGUCCCGCAAAGGGUGCCCGCCU
Ssp-151-1  CCCCUCUCGC.......GACC...........................GCUUGAGGGAA.....CUGA.CUCCGGUACCUGACACGGCCGGUG..CAGAUUUG................................AAGGAGUAACA.......AUGCCGCGUCCCGCAAAGGGUGCCCGCCU
Ssp-110-1  CCCCUCUCGC.......GACC...........................GCUUGAGGGAA.....CUGA.CUCCGGUACCUGACACGGCCGGUG..CAGAUUUG................................AAGGAGUAACA.......AUGCCGCGUCCCGCAAAGGGUGCCCGCCU
Ssp-112-1  ACCCUCUCGC.......GACC...........................GCUUGAGGGAA.....CUGA.CUCCGGUACCUGACACGGCCGGUG..CAGAUUUG................................AAGGAGUAACA.......AUGCCGCGUCCCGCAAAGGGUGCCCGCCU
Ssp-102-1  CCCCUCUCGC.......GACC...........................GCUUGAGGGAA.....CUGA.CUCCGGUACCUGACACGGCCGGUG..CAGAUUUG................................AAGGAGUAACA.......AUGCCGCGUCCCGCAAAGGGUGCCCGCCU
Sla-2-1    CCCCUCUCGC.......GACC...........................GCUUGAGGGAA.....CUGA.CACCGGUACCUGACACGGCCGGUG..CAGAUAUG................................AAGGAGUAACA.......AUGCCGCGUCCCGCAAAGGGUGCCCGCCU
Ssp-139-1  CCCCUCUCGC.......GACC...........................GCUUGAGGGAA.....CUGA.CACCGGUACCUGACACGGCCGGUG..CAGAUUUG................................AAGGAGUAACA.......AUGCCGCGUCCCGCAAAGGGUGCCCGCCU
Ssp-8-1    ACCCCCUCGC.......GACC...........................GCUGGGGGGAA.....CUGA.CACCGGUACCUGACACGGCCGGUG..CAGAUUUG................................AAGGAGUAACA.......AUGCCGCGUCCCGCAAAGGGUGCCCGCCU
Svi-7-1    UCCGA.CGGGU......GACC..........................GCCCGCUCGGACA....CUGA.CAUCGGUACCUCACACGGCCGGUG..CAGAUCAC................................AAGGAGAAACACC.....AUGCCGCGUCCCGCGAAGGGUGCCCGCCU
Sgr-12-1   UCCGA.CGGGU......GACC..........................GCCCGCUCGGACA....CUGA.CACCGGUACCUCACACGGCCGGUG..CAGAUCAC................................AAGGAGAAACACC.....AUGCCGCGUCCCGCGAAGGGUGCCCGCCU
Sfl-5-1    UCCGA.CGGGU......GACC..........................GCCCGCUCGGACA....CUGA.CAUCGGUACCUCAUACGGCCGGUG..CAGAUCAC................................AAGGAGAAACACC.....AUGCCGCGUCCCGCGAAGGGUGCCCGCCU
Sbi-3-1    UCCGA.CGGGU......GACC..........................GCCCGCUCGGACA....CUGA.CAUCGGUACCUCAUACGGCCGGUG..CAGAUCAC................................AAGGAGAAACACC.....AUGCCGCGUCCCGCGAAGGGUGCCCGCCU
Sex-1-1    UCCGA.CGGGU......GACC..........................GCCCGCUCGGACA....CUGA.CAUCGGUACCUCAUACGGCCGGUG..CAGAUCAC................................AAGGAGAAACACC.....AUGCCGCGUCCCGCGAAGGGUGCCCGCCU
Sgl-3-1    UCCGA.CGGGU......GACC..........................GCCCGCUCGGACA....CUGA.CACCGGUACCUCAUACGGCCGGUG..CAGAUCAC................................AAGGAGAAACACC.....AUGCCGCGUCCCGCGAAGGGUGCCCGCCU
Sgr-9-1    UCCGA.CGGGU......GACC..........................GCCCGCUCGGACA....CUGA.CACCGGUACCUCAUACGGCCGGUG..CAGAUCAC................................AAGGAGAAACACC.....AUGCCGCGUCCCGCGAAGGGUGCCCGCCU
Ssp-121-1  UCCGA.CGGGU......GACC..........................GCCCGCUCGGACA....CUGA.CACCGGUACCUCAUACGGCCGGUG..CAGAUCAC................................AAGGAGAAACACC.....AUGCCGCGUCCCGCGAAGGGUGCCCGCCU
Sve-1-1    UCCGA.CGGGC......ACUC..........................GCCCGCUCGGACA....CUGA.CAUCGGUACCUCAUACGGCCGGUG..CAGAUCAC................................AAGGAGAAACACC.....AUGCCGCGUCCCGCGAAGGGUGCCCGCCU
Sav-2-1    UCCGA.CGGGC......AAUC..........................GCCCGCUCGGACA....CUGA.CCUCGGUACCUGAUACGGCCGGGG..CAGACACC................................UAGGAGAAACACC.....AUGCCGAAGCCCACCAAGGGUGCCCGUCU
Mgl-1-1    UCCGA.CAGGU......AUCC..........................GCCUGCUCGGACA....CUGA.CUCCGGUACCUGACACGGCCGGGG..CAGACACU................................AGGAGAAACACCA.....AUGCCGCGUCCCACCAAGGGUGCCCGCCU
Asp-1-1    UCCGA.CGGAC......GACC..........................GUCCGCUCGGAUA....CUGA.CUCCGGUACCUAGUACGGCCGGGG..CAGCACAC................................AAGGAGAAACAUC.....AUGCCGAAGCCCGCCAAGGGUGCCCGUCU
Stu-1-1    UCCGA.CGGGU......GACC..........................GCCUGCUCGGACA....CUGA.CUCUGGUACCUGAUACGGCCGGGG..CAGACAAC................................AAGGAGAAAGAUC.....AUGCCGAAGCCCGCCAAGGGUGCCCGUCU
Sye-2-1    UCCGA.CAGGC......AGCC..........................GCCUGCUCGGAUA....CUGA.CCUCGGUACCUAGUACGGCCGGGG..CAGACACC................................UAGGAGAAGAACC.....AUGCCGAAGCCCGCCAAGGGUGCCCGUCU
Sdu-1-1    UCCGA.CAGAC......AACC..........................GUCUGCUCGGAUA....CUGA.CCCUGGUACCUGACACGGCCAGGG..CAGACACA................................CAGGAGAAGAAC......AUGCCGAAGCCCACCAAGGGUGCCCGUCU
Sgh-1-1    UCCGA.CAGAC......AACC..........................GUCUGCUCGGAUA....CUGA.CUCCGGUACCUGAUACGGCCGGGG..CAGACACA................................CAGGAGAAGAACC.....AUGCCGAAGCCCACCAAGGGUGCCCGUAU
Svi-5-1    UCCGA.CAGAC......AACC..........................GUCUGCUCGGAUA....CUGA.CUCCGGUACCUGAUACGGCCGGGG..CAGACACA................................CAGGAGAAGAACC.....AUGCCGAAGCCCACCAAGGGUGCCCGUAU
Ssp-153-1  UCCGA.CAGAC......AACC..........................GUCUGCUCGGAUA....CUGA.CUCCGGUACCUGACACGGCCGGGG..CAGACACA................................CAGGAGAAGAACC.....AUGCCGAGGCCCACCAAGGGUGCCCGUCU
Ssp-86-1   UCCGA.CAGAC......AACC..........................GUCUGCUCGGAUA....CUGA.CCUCGGUACCUGACACGGCCGGGG..CAGACACA................................CAGGAGAAGAACC.....AUGCCGAAGCCCACCAAGGGUGCCCGUCU
Sro-1-1    UCCGA.CAGAC......AACC..........................GUCUGCUCGGAUA....CUGA.CCCCGGUACCUGACACGGCCGGGG..CAGACACA................................CAGGAGAAGAACA.....AUGCCGAAGCCCACCAAGGGUGCCCGUCU
Sgr-7-1    UCCGA.CAGAC......AACC..........................GUCUGCUCGGAUA....CUGA.CCCCGGUACCUGACACGGCCGGGG..CAGACACA................................CAGGAGAAGAACU.....AUGCCGAAGCCCACCAAGGGUGCCCGUCU
Ssp-28-1   UCUGA.CAGAC......AACC..........................GUCUGCUCAGAUA....CUGA.CUCCGGUACCUGACACGGCCGGGG..CAGACACC................................UAGGAGAAACACC.....AUGCCGAAGCCUGCCAAGGGCGCCCGUCU
Ssp-176-1  UCCGA.CAGGC......AACC..........................GUCUGCUCGGAUA....CUGA.CUCCGGUACCUGAUACGGCCGGAG..CAGACACC................................UAGGAGAAAUACC.....AUGCCCAGGCCCACCAAGGGUGCCCGUAU
Ssp-14-1   UCCGA.CAGGU......AACC..........................GCCUGCUCGGAUA....CUGA.CACCGGUACCUGGUACGGCCGGUG..CAGACACC................................AAGGAGAAACACC.....AUGCCGCGUCCCACCAAGGGAGCCCGUUU
Sac-2-1    UCUGA.CAGGC......GACC..........................GCCUGCUCAGAUA....CUGA.CUCCGGUACCUGAUACGGCCGGGG..CAGACACC................................UAGGAGAAGAACC.....AUGCCGAAGCCCACCAAGGGUGCCCGUCU
Sbo-1-1    UCUGA.CAGGC......GACC..........................GUCUGCUCAGAUA....CUGA.CUCCGGUACCUAGUACGGCCGGGG..CAGACACC................................UAGGAGAAACACC.....AUGCCGAAGCCCACCAAGGGUGCCCGUCU
Ssp-2-1    UCUGA.CAGGC......GACC..........................GUCUGCUCAGAUA....CUGA.CUCCGGUACCUGGUACGGCCGGGG..CAGACACC................................UAGGAGAAACAUC.....AUGCCGAAGCCCACCAAGGGUGCCCGUCU
Ssp-98-1   UCUGA.CAGGC......GACC..........................GUCUGCUCAGAUA....CUGA.CUCCGGUACCUGGUACGGCCGGGG..CAGACACC................................UAGGAGAAACAUC.....AUGCCGAAGCCCACCAAGGGUGCCCGUCU
Sac-3-1    UCUGA.CAGGC......GACC..........................GUCUGCUCAGAUA....CUGA.CCCCGGUACCUCGUACGGCCGGGG..CAGACACCU...............................CAGGAGAAUGAA......AUGCCCAAGCCCACCAAGGGUGCCCGUCU
Sca-7-1    UCCGA.CAGAC......GUCC..........................GUCUGCUCGGAUA....CUGA.CCUCGGUACCUGAUACGGCCGGGG..CAGACACC................................UAGGAGAAACACC.....AUGCCGAAGCCCACCAAGGGUGCCCGUCU
Sgr-2-1    UCCGA.CAGAC......GACC..........................GUCUGCUCGGAUA....CUGA.CCCCGGUACCUGAUACGGCCGGGG..CAGACACA................................CAGGAGAGACAUC.....AUGCCGAGGCCCACCAAGGGUGCCCGUAU
Ssp-105-1  UCCGA.CGAGC......AACC..........................GCUCGCUCGGAUA....CUGA.CUCCGGUACCUGACACGGCCGGGG..CAGACACU................................GAGGAGAAACAUC.....AUGCCGAAGCCCUCCAAGGGUGCCCGUCU
Ssp-85-1   UCCGA.CGAGC......AACC..........................GCUCGCUCGGAUA....CUGA.CUUCGGUACCUGACACGGCCGGGG..CAGACACA................................CAGGAGAAGAACU.....AUGCCGAAGCCCACCAAGGGUGCCCGUCU
Sco-3-1    UCCGA.CGAGC......AACC..........................GCUCGCUCGGAUA....CUGA.CCUCGGUACCUGACACGGCCGGGG..CAGACACA................................CAGGAGAAGAACC.....AUGCCGAAGCCCACCAAGGGUGCCCGUCU
Sse-1-1    UCCGA.AGAGC......AACC..........................GCUCGCUCGGAUA....CUGA.CCUCGGUACCUGACACGGCCGGGG..CAGACACU................................UAGGAGAAACAUC.....AUGCCGAAGCCCGCCAAGGGUGCCCGUCU
Sre-1-1    UCCGA.CAGGC......GAUC..........................GCCUGCUCGGACA....CUGA.CCCCGGUACCUGAUACGGCCGGGG..CAGACACC................................UAGGAGAAACACC.....AUGCCGAAGCCCACCAAGGGUGCCCGUCU
Spe-1-1    UCCGA.CGAGC......AAACCC........................GCUCGCUCGGAUA....CUGA.CCCCGGUACCUGACACGGCCGGGG..CAGACACA................................CAGGAGAAGAACC.....AUGCCGAAGCCCACCAAGGGUGCCCGUCU
Sbi-2-1    UCCGA.CGAGC......AAACCC........................GCUCGCUCGGAUA....CUGA.CCCCGGUACCUGAUACGGCCGGGG..CAGACACA................................CAGGAGAAGAACA.....AUGCCGAAGCCCACCAAGGGUGCCCGUCU
Ssp-83-1   UCCGA.CGGGC......AACC..........................GCUCGCUCGGAUA....CUGA.CCCCGGUACCUGACACGGCCGGGG..CAGACACA................................CAGGAGAAGAAACA....AUGCCGAAGCCCACCAAGGGUGCCCGUCU
Spl-1-1    UCCGA.CGGGC......AACC..........................GCUCGCUCGGAUA....CUGA.CCCCGGUACCUGACACGGCCGGGG..CAGACACA................................CAGGAGAAGAAACA....AUGCCGAAGCCCACCAAGGGUGCCCGUCU
Sno-1-1    UCCGA.CGGGC......AACC..........................GCUCGCUCGGAUA....CUGA.CCCCGGUACCUGGUACGGCCGGGG..CAGACACC................................UAGGAGAAGAACC.....AUGCCGAAGCCCACCAAGGGUGCCCGUCU
San-1-1    UCCGA.CGAGC......AACC..........................GCUCGCUCGGAUA....CUGA.CCCCGGUACCUGAUACGGCCGGGG..CAGACACA................................CAGGAGAAGAACC.....AUGCCGAAGCCCACCAAGGGUGCCCGUCU
Sla-3-1    UCCGA.CGGGC......AACC..........................GCUCGCUCGGAUA....CUGA.CCCCGGUACCUGAUACGGCCGGGG..CAGAGAUA................................CAGGAGAUGGAAC.....AUGCCGAAGCCCACCAAGGGUGCCCGUCU
Ssp-74-1   UCCGA.CGGGC......AACC..........................GCUCGCUCGGAUA....CUGA.CCCCGGUACCUGAUACGGCCGGGG..CAGACACA................................CAGGAGAAGAACC.....AUGCCGAAGCCCACCAAGGGUGCCCGUCU
Ssp-162-1  UCCGA.CGGGC......AACC..........................GCUCGCUCGGAUA....CUGA.CCCCGGUACCUGAUACGGCCGGGG..CAGACACC................................UAGGAGAAACACC.....AUGCCGAAGCCCACCAAGGGUGCCCGUAU
Ssp-164-1  UCCGA.CGGGC......AACC..........................GCUCGCUCGGAUA....CUGA.CCCCGGUACCUGAUACGGCCGGGG..CAGACACC................................UAGGAGAAACACC.....AUGCCGAAGCCCACCAAGGGUGCCCGUAU
Ssp-161-1  UCCGA.CGGGC......AACC..........................GCUCGCUCGGAUA....CUGA.CCCCGGUACCUGAUACGGCCGGGG..CAGACACC................................UAGGAGAAACACC.....AUGCCGAAGCCCACCAAGGGUGCCCGUAU

           .<<<<<<<<<<<<<<<<........................>>>>>>>>>>>>>>>>.......<<<..<<<<<<<..........>>>>>>>..>>>.....................................................................................
           .2222222221110000........................0000111222222222.......122...221100..........001122...221.....................................................................................
           YC●●●●●R○○-------R●YY---------------------------○○Y●●●●●G●○○----CUGA-C●CCGGUACCUGAYACGGCCGG●G--CAGR●●●○-------------------------------●AGGAGARR●●○○-----AUGCC●●R●CCCRCCAAGGGYGCCCGYCU
           5′                                                                                 3′
```

```
Sgl-2-1    UCCGA.CGGGC......AACC.........................GCUCGCUCGGAUA....CUGA.CCCCGGUACCUGAUACGGCCGGGG..CAGACACA..............................CAGGAGAAGAACC....AUGCCGAAGCCCACCAAGGGUGCCCGUCU
Ssp-143-1  UCCGA.CGGGU......AAACCC.......................GCUCGCUCGGAUA....CUGA.CCCCGGUACCUGAUACGGCCGGGG..CAGACACC..............................UAGGAGAAGUUCC....AUGCCGAAGCCCACCAAGGGUGCCCGUAU
Ssp-36-1   UCCGA.CAGGC......GACC.........................GCCUGCUCGGAUA....CUGA.CUCCGGUACCUCGUACGGCCGGGG..CAGACACC..............................UAGGAGAAACAUC....AUGCCGAGGCCCACCAAGGGUGCCCGUCU
Ssp-200-1  UCCGA.CAGGC......GGCC.........................GCCUGCUCGGAUA....CUGA.CUUCGGUACCUGAUACGGCCGGGG..CAGACACC..............................UAGGAGAAACAUC....AUGCCGAAGCCCAGCAAGGGUGCCCGUCU
Sco-2-1    UCCGA.CAGGC......GGCC.........................GCCUGCUCGGAUA....CUGA.CUUCGGUACCUGAUACGGCCGGGG..CAGACACC..............................UAGGAGAAACAUC....AUGCCGAAGCCCAGCAAGGGUGCCCGUCU
Svi-9-1    UCCGA.CAGGC......GGCC.........................GCCUGCUCGGAUA....CUGA.CUUCGGUACCUGAUACGGCCGGGG..CAGACACC..............................UAGGAGAAACAUC....AUGCCGAAGCCCAGCAAGGGUGCCCGUCU
Sco-1-1    UCCGA.CAGGC......GGCC.........................GCCUGCUCGGAUA....CUGA.CUUCGGUACCUGAUACGGCCGGGG..CAGACACC..............................UAGGAGAAACACC....AUGCCGAAGCCCACCAAGGGUGCCCGUCU
Ssp-128-1  UCCGA.CAGGC......GGCC.........................GCCUGCUCGGAUA....CUGA.CCCCGGUACCUGAUACGGCCGGGG..CAGACACC..............................UAGGAGAAACACC....AUGCCGAAGCCCACCAAGGGUGCCCGUCU
Sol-2-1    UCCGA.CAGGC......GGCC.........................GCCUGCUCGGAUA....CUGA.CCUCGGUACCUGAUACGGCCGGGG..CAGACACC..............................UAGGAGAAACAUC....AUGCCGAAGCCCACCAAGGGUGCCCGUCU
Ssp-72-1   UCCGA.CAGGC......GGCC.........................GCCUGCUCGGAUA....CUGA.CCUCGGUACCUGAUACGGCCGGGG..CAGACACC..............................UAGGAGAAACAUC....AUGCCGAAGCCCACCAAGGGUGCCCGUCU
Ssp-124-1  UCCGA.CAGGC......GGCC.........................GCCUGCUCGGAUA....CUGA.CCUCGGUACCUGAUACGGCCGGGG..CAGACACC..............................UAGGAGAAACACC....AUGCCGAAGCCCACCAAGGGUGCCCGUCU
Sce-2-1    UCCGA.CGGGC......GACC.........................GCCCGCUCGGAUA....CUGA.CUCCGGUACCUGAUACGGCCGGGG..CAGACCCC..............................UAGGAGAAAGACC....AUGCCGAAGCCCACCAAGGGUGCCCGUCU
Smo-1-1    UCCGA.CGGGC......GACC.........................GCCCGCUCGGAUA....CUGA.CUCCGGUACCUGAUACGGCCGGGG..CAGACACC..............................UAGGAGAAACACC....AUGCCGCGUCCCACCAAGGGUGCCCGUCU
Svi-9-2    UCCGA.CGGGC......GACC.........................GCCCGCUCGGAUA....CUGA.CUCCGGUACCUGAUACGGCCGGGG..CAGACACC..............................UAGGAGAAAGACC....AUGCCGAAGCCCACCAAGGGUGCCCGUCU
Sso-1-1    UCCGA.CGGGC......GACC.........................GCCCGCUCGGAUA....CUGA.CCCCGGUACCUGGUACGGCCGGGG..CAGACACC..............................AAGGAGAAACACC....AUGCCGCGUCCCGCGAAGGGUGCCCGCCU
Soc-1-1    UCCGA.CGGGC......GACC.........................GCCCGCUCGGAUA....CUGA.CCCCGGUACCUGAUACGGCUGGGG..CAGACACA..............................GAGGAGAAACACC....AUGCCGCGUCCCGCCAAGGGUGCCCGUCU
Sni-2-1    UCCGA.CGGGC......GACC.........................GCCCGCUCGGAUA....CUGA.CCCCGGUACCUGAUACGGCUGGGG..CAGACACA..............................GAGGAGAAACACC....AUGCCGCGUCCCGCCAAGGGUGCCCGUCU
Svi-11-1   UCCGA.CGGGC......GACC.........................GCCCGCUCGGAUA....CUGA.CCCCGGUACCUGAUACGGCUGGGG..CAGACACA..............................GAGGAGAAACACC....AUGCCGCGUCCCGCCAAGGGUGCCCGUCU
Sex-2-1    UCCGA.CGGGU......GACC.........................GCCCGCUCGGAUA....CUGA.CCCCGGUACCUGAUACGGCCGGGG..CAGACACC..............................AAGGAGAAACACC....AUGCCGCGUCCCGCGAAGGGUGCCCGCCU
Ssp-6-1    UCCGA.CGGGC......GACC.........................GCCCGCUCGGAUA....CUGA.CCCCGGUACCUGAUACGGCCGGGG..CAGACACC..............................UAGGAGAAACACC....AUGCCGAAGCCCACCAAGGGUGCCCGUCU
Ssp-101-1  UCCGA.CGGGC......GACC.........................GCCCGCUCGGAUA....CUGA.CCCCGGUACCUGAUACGGCCGGGG..CAGACACC..............................UAGGAGAGACACC....AUGCCGAGGCCCACCAAGGGCGCCCGUAU
Ssp-49-1   UCCGA.CGGGC......GACC.........................GCCCGCUCGGAUA....CUGA.CCCCGGUACCUGAUACGGCCGGGG..CAGACACA..............................CAGGAGAAGAACC....AUGCCGAAGCCCACCAAGGGUGCCCGUCU
Sal-11-1   UCCGA.CGGGC......GACC.........................GCCUGCUCGGACA....CUGA.CCCCGGUACCUGAUACGGCCGGGG..CAGACAUG..............................GAGGAGCAACACC....AUGCCGAAGCCCACCAAGGGUGCCCGUUU
Shy-2-1    UCCGA.CAGGC......GACC.........................GCCUGCUCGGAUA....CUGA.CUCCGGUACCUGACACGGCCGGGG..CAGACACA..............................CAGGAGAAAGAAC....AUGCCGAAGCCCACCAAGGGUGCCCGUCU
Ssp-46-1   UCCGA.CAGGC......GACC.........................GCCUGCUCGGAUA....CUGA.CUCCGGUACCUGAUACGGCCGGGG..CAGACACC..............................UAGGAGAAUCACC....AUGCCGAAGCCCACCAAGGGUGCCCGUCU
Ssp-187-1  UCCGA.CAGGC......GACC.........................GCCUGCUCGGAUA....CUGA.CUCCGGUACCUGAUACGGCCGGGG..CAGACACC..............................UAGGAGAAUCACC....AUGCCGAAGCCCACCAAGGGUGCCCGUCU
Ssp-156-1  UCCGA.CAGGC......GACC.........................GCCUGCUCGGAUA....CUGA.CUCCGGUACCUGAUACGGCCGGGG..CAGACACC..............................UAGGAGAAACAUC....AUGCCGAGGCCCACCAAGGGUGCCCGUCU
Sgr-10-1   UCCGA.CAGGC......GACC.........................GCCUGCUCGGAUA....CUGA.CUCCGGUACCUGAUACGGCCGGGG..CAGACACA..............................CAGGAGAGUCACU....AUGCCGAGGCCCACCAAGGGUGCCCGUAU
Aac-1-1    UCCGA.CAGGC......GACC.........................GCCUGCUCGGAUA....CUGA.CUCCGGUACCUGAUACGGCCGGGG..CAGACACA..............................CAGGAGAGUCACU....AUGCCGAGGCCCACCAAGGGUGCCCGUAU
Ssp-197-1  UCCGA.CAGGC......GACC.........................GCCUGCUCGGAUA....CUGA.CUCCGGUACCUGAUACGGCCGGGG..CAGACACA..............................CAGGAGAGUCACU....AUGCCGAGGCCCACCAAGGGUGCCCGUCU
Ssp-20-1   UCCGA.CAGGC......GACC.........................GCCUGCUCGGAUA....CUGA.CUCCGGUACCUGAUACGGCCGGGG..CAGACACA..............................CAGGAGAGUCACU....AUGCCGAGGCCCACCAAGGGUGCCCGUCU
Ssp-123-1  UCCGA.CAGGC......GACC.........................GCCUGCUCGGAUA....CUGA.CUCCGGUACCUGAUACGGCCGGGG..CAGACACC..............................UAGGAGAUACACC....AUGCCGAGGCCCACCAAGGGUGCCCGUCU
Ssp-141-1  UCCGA.CAGGC......GACC.........................GCCUGCUCGGAUA....CUGA.CUCCGGUACCUGAUACGGCCGGGG..CAGACACC..............................UAGGAGAUACACC....AUGCCGAGGCCCACCAAGGGUGCCCGUCU
Ssp-81-1   UCCGA.CAGGC......GACC.........................GCCUGCUCGGAUA....CUGA.CUCCGGUACCUGAUACGGCCGGGG..CAGACACC..............................UAGGAGAAUCACC....AUGCCGAAGCCCACCAAGGGUGCCCGUCU
Ssp-113-1  UCCGA.CAGGC......GACC.........................GCCUGCUCGGAUA....CUGA.CUCCGGUACCUGAUACGGCCGGGG..CAGACACC..............................UAGGAGAAUCACC....AUGCCGAAGCCCACCAAGGGUGCCCGUCU
Ssp-57-1   UCCGA.CAGGC......GACC.........................GCCUGCUCGGAUA....CUGA.CUCCGGUACCUGAUACGGCCGGGG..CAGACACA..............................CAGGAGAGUCAUC....AUGCCGAGGCCCACCAAGGGUGCCCGUAU
Ssp-149-1  UCCGA.CAGGC......GACC.........................GCCUGCUCGGAUA....CUGA.CUCCGGUACCUGAUACGGCCGGGG..CAGACACA..............................CAGGAGAAGAACC....AUGCCGAAGCCCACCAAGGGUGCCCGUCU
Sah-1-1    UCCGA.CAGGC......AACC.........................GCCUGCUCGGAUA....CUGA.CUCCGGUACCUGAUACGGCCGGGG..CAGACGUUU.............................GAGGAGAAACACC....AUGCCGAAGCCCGCCAAGGGUGCCCGUCU
Svi-10-1   UCCGA.CAGGC......AACC.........................GCCUGCUCGGAUA....CUGA.CUCCGGUACCUGAUACGGCCGGGG..CAGACACC..............................UAGGAGAAACACC....AUGCCGAAGCCCACCAAGGGUGCCCGUUU
Sch-1-1    UCCGA.CAGGC......AAACCC.......................GUCUGCUCGGAUA....CUGA.CCCCGGUACCUGAUACGGCCGGGG..CAGACACC..............................UAGGAGAAACACC....AUGCCGAAGCCCACCAAGGGUGCCCGUCU
Sia-1-1    UCCGA.CAGGC......AAACCC.......................GCCUGCUCGGAUA....CUGA.CCCCGGUACCUGAUACGGCCGGGG..CAGACACC..............................UAGGAGAAGCACC....AUGCCGAAGCCCACCAAGGGUGCCCGUAU
Saf-1-1    UCCGA.CAGGC......AAACCC.......................GCCUGCUCGGAUA....CUGA.CCCCGGUACCUGAUACGGCCGGGG..CAGACACC..............................UAGGAGAAACACC....AUGCCGAAGCCCACCAAGGGUGCCCGUAU
Ssp-89-1   UCCGA.CAGGC......AACC.........................GCCUGCUCGGAUA....CUGA.CCCCGGUACCUGAUACGGCCGGGG..CAGACACC..............................UAGGAGAGACAGC....AUGCCGAAGCCCACCAAGGGUGCCCGUAU
Sva-1-1    UCCGA.CGGGC......AACC.........................GCCUGCUCGGAUA....CUGA.CCCCGGUACCUGAUACGGCCGGGG..CAGACACC..............................UAGGAGAAACACC....AUGCCGAAGCCCACCAAGGGUGCCCGUAU
Ssp-158-1  UCCGA.CGGGC......GACC.........................GCCUGCUCGGAUA....CUGA.CCCCGGUACCUGAUACGGCCGGGG..CAGACACC..............................UAGGAGAAACACC....AUGCCGAGGCCCACCAAGGGUGCCCGUAU
Sro-5-1    UCCGA.CAGGC......AACC.........................GCCUGCUCGGAUA....CUGA.CCUCGGUACCUGAUACGGCCGGGG..CAGACUA...............................AAGGAGAAACACC....AUGCCGCGUCCCACCAAGGGUGCCCGUCU
Ssp-116-1  UCCGA.CAGGC......GACC.........................GCCUGCUCGGAUA....CUGA.CCCCGGUACCUGGUACGGCCGGGG..CAGACACC..............................UAGGAGAAUCACC....AUGCCGAAGCCCGCCAAGGGUGCCCGUCU
env-22     UCCGA.CAGGC......GCCC.........................GCCUGCUCGGAUA....CUGA.CCUCGGUACCUGAUACGGCCGGGG..CAGACACA..............................CAGGAGAAGAACU....AUGCCGAAGCCCACCAAGGGUGCCCGUCU
Sam-1-1    UCCGA.CAGGC......GACC.........................GCCUGCUCGGAUA....CUGA.CCUCGGUACCUGAUACGGCCGGGG..CAGACACC..............................UAGGAGAAACACC....AUGCCGAAGCCCAGCAAGGGUGCCCGUAU
Ssp-51-1   UCCGA.CAGGC......GACC.........................GCCUGCUCGGAUA....CUGA.CCUCGGUACCUGAUACGGCCGGGG..CAGACACA..............................CAGGAGAAGAACA....AUGCCGAAGCCCACCAAGGGUGCCCGUCU
Slu-1-1    UCCGA.CAGGC......GACC.........................GCCUGCUCGGAUA....CUGA.CCUCGGUACCUGAUACGGCCGGGG..CAGACACC..............................UAGGAGAAUCACC....AUGCCGAAGCCCACCAAGGGUGCCCGUCU
Sna-2-1    UCCGA.CAGGC......GACC.........................GCCUGCUCGGAUA....CUGA.CCCCGGUACCUGAUACGGCCGGGG..CAGACACC..............................AAGGAGAAACACC....AUGCCGAAGCCUUCCAAGGGUGCCCGUCU
Ssp-75-1   UCCGA.CAGGC......GACC.........................GCCUGCUCGGAUA....CUGA.CCCCGGUACCUGAUACGGCCGGGG..CAGACACA..............................CAGGAGAGUCACU....AUGCCGAGGCCCACCAAGGGUGCCCGUCU
env-23     UCCGA.CAGGC......GACC.........................GCCUGCUCGGAUA....CUGA.CCCCGGUACCUGAUACGGCCGGGG..CAGACACC..............................UAGGAGAAACACG....AUGCCGAAGCCCACCAAGGGUGCCCGUCU
Ssp-182-1  UCCGA.CAGGC......GACC.........................GCCUGCUCGGAUA....CUGA.CCCCGGUACCUGAUACGGCCGGGG..CAGACACA..............................CAGGAGAAGAACC....AUGCCGAAGCCCACCAAGGGUGCCCGUCU
Scy-2-1    UCCGA.CAGGC......GACC.........................GCCUGCUCGGAUA....CUGA.CCCCGGUACCUGAUACGGCCGGGG..CAGACACC..............................UAGGAGAAACACC....AUGCCGAAGCCCACCAAGGGUGCCCGUCU
Ssp-184-1  UCCGA.CAGGC......GACC.........................GCCUGCUCGGAUA....CUGA.CCCCGGUACCUGAUACGGCCGGGG..CAGACACA..............................CAGGAGAAGAACC....AUGCCGAAGCCCACCAAGGGUGCCCGUCU
Svi-4-1    UCUGA.CAGGC......AACC.........................GCCUGCUCAGAUA....CUGA.CCCCGGUACCUGAUACGGCCGGGG..CAGACACA..............................CAGGAGAAGAACA....AUGCCGAAGCCCACCAAGGGUGCCCGUCU
Ssp-163-1  UCUGA.CAGAC......AACC.........................GUCUGCUCAGAUA....CUGA.CCCCGGUACCUGAUACGGCCGGGG..CAGACACC..............................UAGGAGAAGUUCC....AUGCCGAAGCCCACCAAGGGUGCCCGUAU
Ssp-177-1  UCUGA.CAGAC......AACC.........................GUCUGCUCAGAUA....CUGA.CCCCGGUACCUGAUACGGCCGGGG..CAGACACC..............................UAGGAGAAACACC....AUGCCGAAGCCCACCAAGGGUGCCCGUAU
Sol-1-1    UCUGA.CAGAC......AACC.........................GUCUGCUCAGAUA....CUGA.CCCCGGUACCUGAUACGGCCGGGG..CAGACACC..............................UAGGAGAAACACC....AUGCCGAAGCCCACCAAGGGUGCCCGUAU
Svi-3-1    UCUGA.CAGAC......AACC.........................GUCUGCUCAGAUA....CUGA.CCUCGGUACCUGAUACGGCCGGGG..CAGACACC..............................UAGGAGAAACACC....AUGCCGAAGCCCACCAAGGGUGCCCGUCU
Szi-1-1    UCCGA.CAGGU......AACC.........................GCCUGCUCGGAUA....CUGA.CCCCGGUACCUCAUACGGCCGGGG..CAGACACA..............................CAGGAGAUUGAC.....AUGCCGAAGCCCACCAAGGGUGCCCGUCU
Svi-13-2   UCCGA.CAGGU......AACC.........................GCCUGCUCGGAUA....CUGA.CCUCGGUACCUCAUACGGCCGGGG..CAGACAUA..............................CAGGAGAUUGAC.....AUGCCGAAGCCCACCAAGGGUGCCCGUCU
Ssp-120-1  UCCGA.CAGGU......AACC.........................GCCUGCUCGGAUA....CUGA.CCUCGGUACCUCAUACGGCCGGGG..CAGACAUA..............................CAGGAGAUUGAC.....AUGCCGAAGCCCACCAAGGGUGCCCGUCU
Ssp-131-1  UCCGA.CAGGU......AACC.........................GCCUGCUCGGAUA....CUGA.CACCGGUACCUCAUACGGCCGGUG..CAGAUCAC..............................AAGGAGAAACACC....AUGCCGCGUCCCGCGAAGGGUGCCCGCCU
Sca-4-1    UCCG.GCGGGC......AACC.........................GUCCGCUCGGAUA....CUGA.CUCCGGUACCUGAUACGGCCGGGG..CAGACAUC..............................AAGGAGAAACACC....AUGCCGCGUCCCACCAAGGGUGCCCGUCU
Sba-1-1    CCCGUGCGGAC......GACCU........................GUCCGUACGGACCC...CUGA.CACCGGUACCUGACACGGCCGGUG..CAGUAACC..............................AAGGAGAAGCACC....AUGCCGAAGCCCACCAAGGGUGCCCGUCU
Ssp-65-1   CCCGUGCGGACG.....GCC.........................CGUCCGUACGGACCC...CUGA.CACCGGUACCUGACACGGCCGGUG..CAGUAACC..............................AAGGAGAAGCACC....AUGCCGAAGCCCACCAAGGGUGCCCGUCU
Sme-2-1    UCCG.GCGGAC......ACAA.........................GUCCGUACGGACA....CUGA.CAUCGGUACCUGAUACGGCCGGUG..CAGACACGA.............................GAGGAACGGAUCA....AUGCCGAAGCCCCCCAAGGGCGCCCGCCU
Sgr-12-3   UCCG.GCGGAC......ACAA.........................GUUCGUGCGGACA....CUGU.CAUCGGUACCUGAUACGGCCGGUG..CAGAUCAC..............................AAGGAGAAAGACC....AUGCCGAAGCCCACCAAGGGUGCCCGUCU
Sgr-12-4   UCCG.GCGGAC......ACAA.........................GUUCGUGCGGACA....CUGU.CAUCGGUACCUGAUACGGCCGGUG..CAGAUCAC..............................AAGGAGAAAGACC....AUGCCGAAGCCCACCAAGGGUGCCCGUCU
Ssp-15-1   UCCG.GCGGAC......ACAA.........................GUUCGUGCGGACA....CUGU.CAUCGGUACCUGAUACGGCCGGUG..CAGAUCAC..............................AAGGAGAAAGACC....AUGCCGAAGCCCACCAAGGGUGCCCGUCU
Ssp-38-1   UCCG.GCGGAC......ACAA.........................GUUCGUGCGGACA....CUGU.CAUCGGUACCUGAUACGGCCGGUG..CAGAUCAC..............................AAGGAGAAAGACC....AUGCCGAAGCCCACCAAGGGUGCCCGUCU
Sal-5-1    UCCG.GCGGAC......ACAA.........................GUUCGUGCGGACA....CUGU.CAUCGGUACCUGAUACGGCCGGUG..CAGAUCAC..............................AAGGAGAAAGACC....AUGCCGAAGCCCACCAAGGGUGCCCGUCU
Ssp-77-1   UCCG.GCGGAC......ACAA.........................GUUCGUGCGGACA....CUGU.CAUCGGUACCUGAUACGGCCGGUG..CAGAUCAC..............................AAGGAGAAAGACC....AUGCCGAAGCCCACCAAGGGUGCCCGUCU
Swa-1-1    UCCG.GCGGAC......ACAA.........................GUUCGUGCGGACA....CUGU.CAUCGGUACCUGAUACGGCCGGUG..CAGAUCAC..............................AAGGAGAAAGACC....AUGCCGAAGCCCACCAAGGGUGCCCGUCU
Ssp-30-1   UCCG.GCGGAC......ACAA.........................GUUCGUGCGGACA....CUGU.CAUCGGUACCUGAUACGGCCGGUG..CAGAUCAC..............................AAGGAGAAAGACC....AUGCCGAAGCCCACCAAGGGUGCCCGUCU
Ssp-134-1  UCCG.GCGGAC......ACAA.........................GUUCGUGCGGACA....CUGU.CAUCGGUACCUGAUACGGCCGGUG..CAGAUCAC..............................AAGGAGAAAGACC....AUGCCGAAGCCCACCAAGGGUGCCCGUCU
Ssp-62-1   UCCG.GCGGAC......ACAA.........................GUUCGUGCGGACA....CUGU.CAUCGGUACCUGGUACGGCCGGUG..CAGAUCAC..............................AAGGAGAAAGACC....AUGCCGAAGCCCACCAAGGGUGCCCGUCU
Ssp-21-1   UCCG.GCGGAC......ACAA.........................GUUCGUGCGGACA....CUGU.CAUCGGUACCUGGUACGGCCGGUG..CAGAUCAC..............................AAGGAGAAAGACC....AUGCCGAAGCCCACCAAGGGUGCCCGUCU
Ssp-80-1   UCCG.GCGGAC......ACAA.........................GUUCGUGCGGACA....CUGU.CAUCGGUACCUGGUACGGCCGGUG..CAGAUCAC..............................AAGGAGAAAGACC....AUGCCGAAGCCCACCAAGGGUGCCCGUCU
Sgr-12-5   UCCG.GCGGAC......ACAA.........................GUUCGUGCGGACA....CUGU.CAUCGGUACCUGGUACGGCCGGUG..CAGAUCAC..............................AAGGAGAAAGACC....AUGCCGAAGCCCACCAAGGGUGCCCGUCU

           .<<<<<<<<<<<<<<<<.......................>>>>>>>>>>>>>>>>.......<<<..<<<<<<<..........>>>>>>>..>>>...............................................................................
           .2222222221110000.......................0000111222222222.......122...221100..........001122...221...............................................................................
           YC●●●●●R○○-------R●YY------------------------○○Y●●●●●G●○○----CUGA-C●CCGGUACCUGAYACGGCCGG●G--CAGR●●●●○-----------------------------●AGGAGARR●●○○-----AUGCC●●R●CCCRCCAAGGGYGCCCGYCU
            5′———————————————————————————————————————————————————————————————————————————————————3′
```

```
Sva-2-1    UCCG.GCGGGC......AAAC.........................GCUCGUACGGACA....CUGA.CACCGGUACCUGAUACGGCCGGUG..CAGAAAUG................................AAGGAGAAAGACC.....AUGCCGAAGCCCGCCAAGGGUGCCCGUCU
Ssp-58-1   CCCGUACGGGC......AGCA.........................GCCCGUGCGGACC....CUGA.CACCGGUACCUGAUACGGCCGGUG..CAGAGACCC..............................AAGGAGAAAGAC......AUGCCGAAGCCCACCAAGGGUGCCCGUCU
Sal-12-1   UCCG.GCGGGC......AUCA.........................GCCCGUACGGACA....CUGA.CACCGGUACCUGAUACGGCCGGUG..CAGAGACU...............................GAGGAGAAACAC......AUGCCGAAGCCCGCCAAGGGUGCCCGUCU
Sfr-1-1    UCCG.GCGGGC......ACGA.........................GCCCGUACGGACA....CUGA.CACCGGUACCUGAUACGGCCGGUG..CAGAGACU...............................GAGGAGACACAC......AUGCCGAAGCCCGCCAAGGGUGCCCGUCU
Sxi-2-1    UCCG.GCGGGC......ACGA.........................GCCCGUACGGACA....CUGA.CACCGGUACCUGAUACGGCCGGUG..CAGAGACU...............................GAGGAGACACAC......AUGCCGAAGCCCGCCAAGGGUGCCCGUCU
Ssp-119-1  UCCGG.CGGGU......AAUC.........................GCCCGUCCGGGAA....CUGA.CACCGGUACCUGAUACGGCCGGUG..CAGAUACG...............................AAGGAGUCACACCC....AUGCCGAAGCCCACCAAGGGUGCCCGUCU
Ssp-33-1   GCCGGUGAGC.......GCUC..........................GCUCAUCGGGUC....CUGA.CACCGGUACCUGAUACGGCCGGUG..CAGCAAUG...............................AAGGAGAUACACC.....AUGCCGAAGCCCACCAAGGGCGCCCGUCU
Ssp-37-1   GCCGGUGAGC.......GUCC..........................GCUCACCGGGUC....CUGA.CCCCGGUACCUCAUACGGCCGGGG..CAGAUACC...............................AAGGAGAAAGAA......AUGCCGAAGCCCACCAAGGGUGCCCGUCU
Ssp-170-1  GCCGGUGAGC.......AUCC..........................GCUCAUCGGGUUC...CUGA.CCCCGGUACCUGACACGGCCGGGG..CAGACACA...............................CAGGAGAAGAACA.....AUGCCGAAGCCCACCAAGGGUGCCCGUCU
Smi-1-1    GCCGGUGAGC.......ACCC..........................GCUCAUCGGGUUC...CUGA.CCCCGGUACCUGAUACGGCCGGGG..CAGACACA...............................CAGGAGAUAGAAC.....AUGCCGAAGCCCACCAAGGGUGCCCGUCU
Ssp-138-1  GCCGGUGAGC.......AUCC..........................GCUCAUCGGGUUC...CUGA.CCCCGGUACCUGAUACGGCCGGGG..CAGACACA...............................CAGGAGAAAGAAC.....AUGCCGAAGCCCACCAAGGGUGCCCGUCU
Sga-2-1    GCCGGUGAGC.......AUCC..........................GCUCAUCGGGUUC...CUGA.CCCCGGUACCUGAUACGGCCGGGG..CAGACACA...............................CAGGAGAAGAACA.....AUGCCGAAGCCCACCAAGGGUGCCCGUCU
env-24     GCCGGUGAGC.......AUCC..........................GCUCAUCGGGUUC...CUGA.CCCCGGUACCUGAUACGGCCGGGG..CAGACACA...............................CAGGAGAAGAACC.....AUGCCGAAGCCCACCAAGGGUGCCCGUCU
Sca-1-1    GCCGGUGAGC.......AUCC..........................GCUCAUCGGGUUC...CUGA.CCCCGGUACCUGAUACGGCCGGGG..CAGACACA...............................CAGGAGAUAGAAA.....AUGCCGAAGCCCACCAAGGGUGCCCGUAU
Ssp-23-1   GCCGGUGAGC.......AUCC..........................GCUCAUCGGGUUC...CUGA.CCCCGGUACCUGAUACGGCCGGGG..CAGACACA...............................CAGGAGAAAGAA......AUGCCGAAGCCCACCAAGGGUGCCCGUCU
Ssp-135-1  GCCGGUGAGC.......AUCC..........................GCUCAUCGGGUUC...CUGA.CCCCGGUACCUGAUACGGCCGGGG..CAGACACA...............................CAGGAGAAAGAAC.....AUGCCGAAGCCCACCAAGGGUGCCCGUCU
Ssp-169-1  GCCGGUGAGC.......AUCC..........................GCUCAUCGGGUUC...CUGA.CCCCGGUACCUGAUACGGCCGGGG..CAGACACA...............................CAGGAGAAGAACA.....AUGCCGAAGCCCACCAAGGGUGCCCGUCU
env-30     GCCGGUGAGC.......AUCC..........................GCUCAUCGGGUUC...CUGA.CCCCGGUACCUGAUACGGCCGGGG..CAGACACA...............................CAGGAGAAGAACC.....AUGCCGAAGCCCACCAAGGGUGCCCGUCU
Ssv-1-1    GCCGGUGAGC.......AUCC..........................GCUCAUCGGGUUC...CUGA.CCCCGGUACCUGAUACGGCCGGGG..CAGACACA...............................CAGGAGAUAGAAA.....AUGCCGAAGCCCACCAAGGGUGCCCGUAU
Ssp-186-1  GCCGGUGAGC.......AUCC..........................GCUCAUCGGGUUC...CUGA.CCCCGGUACCUGAUACGGCCGGGG..CAGACACA...............................CAGGAGAUAGAAA.....AUGCCGAAGCCCACCAAGGGUGCCCGUAU
Sda-1-1    GCCGGUGAGC.......AUCC..........................GCUCAUCGGGUUC...CUGA.CCCCGGUACCUGAUACGGCCGGGG..CAGACACA...............................CAGGAGAUUGAA......AAUGCCGAAGCCCACCAAGGGUGCCCGUC
Sgr-12-6   GCCGGUGAGC.......AUCC..........................GCUCACCGGGUUC...CUGA.CCCCGGUACCUGAUACGGCCGGGG..CAGAUACC...............................AAGGAGAAAGAA......AUGCCGAAGCCCGCCAAGGGUGCCCGUCU
Ssp-199-1  GCCGGUGAGC.......AUCC..........................GCUCACCGGGUUC...CUGA.CCCCGGUACCUGAUACGGCCGGGG..CAGACC.................................AAGGAGAAAGAA......AUGCCGAAGCCCACCAAGGGUGCCCGUCU
env-31     GCCGGUGAGC.......AACC..........................GCUCGCCGGGUC....CUGA.CCUCGGUACCUGAUACGGCCGGGG..CAGAUACC...............................AAGGAGAAAGAAC.....AUGCCGAAGCCCGCCAAGGGUGCCCGUCU
Ssp-19-1   GCCGGUGAGC.......AACC..........................GCUCACCGGGUC....CUGA.CCUCGGUACCUGAUACGGCCGGGG..CAGAUACC...............................AAGGAGAAAGAAA.....AUGCCGAAGCCCGCCAAGGGUGCCCGUCU
Sfu-2-1    GCCGGUGAGC.......AUCC..........................GCUCACCGGGUC....CUGA.CCCCGGUACCUGAUACGGCCGGGG..CAGAUGCC...............................AAGGAGAAAGAA......AUGCCGAAGCCCACCAAGGGUGCCCGUCU
Sau-2-1    GCCGGUGAGC.......AACC..........................GCUCACCGGGUC....CUGA.CCCCGGUACCUGAUACGGCCGGGG..CAGAUACC...............................AAGGAGAAAGAA......AUGCCGAAGCCCGCCAAGGGUGCCCGUCU
Sxy-1-1    GCCGGUGAGC.......AUCC..........................GCUCGCCGGGUC....CUGA.CCCCGGUACCUGAUACGGCCGGGG..CAGAUACC...............................AAGGAGAAAGAA......AUGCCGAAGCCCUCCAAGGGUGCCCGUCU
Ssp-122-1  GCCGGUGAGC.......GUCC..........................GCUCGCCGGGUC....CUGA.CCCCGGUACCUGAUACGGCCGGGG..CAGAUACC...............................AAGGAGAAAGAA......AUGCCGAAGCCCACCAAGGGUGCCCGUCU
Spr-2-1    GCCGGUGAGC.......GUCC..........................GCUCGCCGGGUC....CUGA.CCCCGGUACCUGAUACGGCCGGGG..CAGAUACC...............................AAGGAGAAAGAA......AUGCCGAAGCCCACCAAGGGUGCCCGUCU
Ssp-84-1   GCCGGUGGGC.......AUCC..........................GCUCACCGGGUUC...CUGA.CCCCGGUACCUGACACGGCCGGGG..CAGACACA...............................CAGGAGAAGAACC.....AUGCCGAAGCCCACCAAGGGUGCCCGUCU
Sip-1-1    GCCGGUGAGC.......AUCC..........................GCUCAUCGGGCC....CUGA.CCUCGGUACCUCAUACGGCCGGGG..CAGAUACC...............................AAGGAGAAAGAAA.....AUGCCGAAGCCCACCAAGGGUGCCCGUCU
Sly-2-1    GCCGGUGAGC.......GACC..........................GCUCAUCGGGUC....CUGA.CCCCGGUACCUAGUACGGCCGGGG..CAGAUACC...............................AAGGAGAAACACC.....AUGCCGAAGCCCUCCAAGGGUGCCCGUAU
Ssc-2-1    CACCCGGGCC.......GACC..........................GGCCCGGGUGGA....CUGA.CGCCGGUACCUGACACGGCCGGCG..CAGGAAUAUC.............................UAGGAGAGAAC.......AUGCCCAGGCCCACCAAGGGUCCGCGCCU
Sye-1-1    ACGGGUCGGC.......GGCC..........................GCCGACCCGCCA....CUGA.CACCGGUACCUGACACGGCCGGUG..CAGCGAAC...............................AAGGAGAAUGAC......AUGCCCCAGCCCGCGAAGGGUGCCCGCCU
Svi-6-1    UCGGCGGGGC.......AACCCC........................GCCCCGCCGAA.....CUGA.CACCGGUACCUGACACGGCCGGUG..CAGACCUG...............................GAGGAGAGACACAACC..AUGCCGAAGCCCACCAAGGGUCCCCGUCU
Sni-1-1    UCCGCGGAC........GGCC...........................GUUCGCGGGGAA...CUGA.CACCGGUACCUGAUACGGCCGGUG..CAGAUAUG...............................AAGGAGUAACACC.....AUGCCGAAGCCCGCCAAGGGUGCCCGUCU
Ssu-2-1    ACCGGCGGGGC......GCCC.........................GCCUCGUCGGC......CUGA.CACCGGUACCUGACACGGCCGGUG..CAGAGUUC...............................AAGGAGAGAGAAG.....AUGCCGAAGCCCACCAAGGGUCCCCGUCU
Ssu-1-1    UCCGGCGGGGC......GCCC.........................GCCUCGUCGGC......CUGA.CACCGGUACCUGACACGGCCGGUG..CAGAGUUC...............................AAGGAGAGAGAAG.....AUGCCGAAGCCCACCAAGGGUCCCCGUCU
Svi-2-1    UCCGGCGGGC.......GGCC..........................GCUCGCCGGUAA....CUGA.CACUGGUACCUGAUACGGCCGGUG..CAGACACC...............................UAGGAGAUACACCA....AUGCCGAAGCCCGCCAAGGGCGCCCGUCU
Sfl-4-1    UCCGGCGGGC.......AGCA..........................GCUCGCCGGUAA....CUGA.CACCGGUACCUGAUACGGCCGGUG..CAGACAC................................AAGGAGAAACACC.....AUGCCGCGUCCCGCAAAGGGUGCCCGUCU
Spu-1-1    UCCGGCGGGC.......GGUC..........................GCCCGUCGGUAA....CUGA.CACCGGUACCUGACACGGCCGGUG..CAGAGAC................................GAGGAGAAACACC.....AUGCCGCGUCCCACCAAGGGAGCCCGUCU
Ssc-4-2    UCCGGCGGGC.......GUCC..........................GCUCGUCGGUAA....CUGA.CACCGGUACCUGACACGGCCGGUG..CAGACAG................................AAGGAGAAACACC.....AUGCCGCGUCCCGCCAAGGGAGCCCGUCU
Shy-1-1    UCCGGCGGGC.......ACAC..........................GCUCGUCGGUAA....CUGA.CACCGGUACCUGAUACGGCCGGUG..CAGACAUC...............................UAGGAGAUACACC.....AUGCCGAAGCCCACCAAGGGCGCCCGUCU
env-32     UCCGGCGGGC.......ACAC..........................GCUCGUCGGUAA....CUGA.CACCGGUACCUGAUACGGCCGGUG..CAGACAUC...............................UAGGAGAUACACC.....AUGCCGAAGCCCGCCAAGGGCGCCCGUCU
Sbi-1-1    UCCGGCGGGC.......ACUC..........................GCCCGUCGGUAA....CUGA.CACCGGUACCUCAUACGGCCGGUG..CAGACCA................................AAGGAGAAACA.......AUGCCGAGGCCCACCAAGGGUGCCCGUCU
```
[truncated: 766,084 more chars]
