# Supplementary material for: Discovery of 20 novel ribosomal leader candidates in bacteria and archaea
Source: BMC Microbiol. 2020 May 24;20:130. doi: 10.1186/s12866-020-01823-6 (PMC7247131; doi:10.1186/s12866-020-01823-6)
Supplement: Supplementary file 1 — Additional file 1. Supplementary text, tables and figures. [file 12866_2020_1823_MOESM1_ESM.pdf]

Additional file 1: Supplementary text, tables and figures

For:

Discovery of 20 novel ribosomal leader candidates in bacteria and archaea

Iris Eckert[1] and Zasha Weinberg[1,*]

Supplementary Text

Candidate r-leaders are consistent with available transcriptomic data

Each of the 20 r-leader motifs has between 67 and 8,743 examples in various genomic locations in various organisms, with a total of 29,730 r-leader examples (Additional file 1: Table S2). We hypothesize that each r-leader example regulates its immediately downstream gene, and possibly additional co-transcribed genes. Thus, if our hypothesis is correct, each r-leader example should be located downstream of the transcription start site (TSS) for its regulated gene. By contrast, if the TSS occurs downstream of the r-leader, the r-leader would not be transcribed, which could suggest that our hypothesis is incorrect. A TSS could also be located within the r-leader such that important conserved features would not be transcribed. Such a TSS position would also contradict our hypothesis. Therefore, we wished to determine if TSS positions are consistent with our r-leader predictions.

TSS positions could, in principle, be determined on a genome-wide scale using standard RNA-seq experiments. However, such experiments do not provide reliable TSS predictions, because an apparent TSS in RNA-seq data could correspond to the 5′ end of a processed RNA [1]. Fortunately, differential RNA-seq (dRNA-seq) addresses this problem [1]. We thus searched (explained in next section, sub-heading “Searching for differential RNA-seq (dRNA-seq) datasets”) for studies that used dRNA-seq or related methods to determine TSS sites for organisms that contain a predicted r-leader. In some cases, no TSS was provided for the gene that we predicted is regulated by the relevant r-leader. We did not analyze these cases, since we cannot be certain where the relevant TSS is.

Ultimately, we found TSS positions for 11 distinct regulated genes out of the 29,730 r-leader examples (Figure S12, Table S3). These 11 regulated genes include examples of eight of the 20 motifs. (Some motifs have multiple examples with TSS data.) Most organisms lack published dRNA-seq results, and most of our motif examples occur in metagenomic sequences, which also lack dRNA-seq experiments. Therefore, only a small fraction of the total r-leader examples had usable TSS data, and we analyzed these available data.

As expected, the TSS sites for all 11 genes were positioned so as to transcribe the r-leader (Figure S12). In two cases, there were TSSes that would cause up to two nucleotides of the r-leader to be

skipped (Figure S12, see ”L31-Actinobacteria” and “S15-Halobacteria”). However, in both cases, the skipped nucleotides are not well conserved, and therefore likely do not belong to the r-leader. Thus, in all cases, conserved nucleotides and stems are located downstream of all TSSes

Searching for differential RNA-seq (dRNA-seq) datasets

To search for relevant dRNA-seq datasets, we enumerated genera in which many examples of each of the 20 novel r-leader motifs occur. We then searched for dRNA-seq data in these genera using multiple strategies. The most successful strategy was to use Google Scholar [2] to search for the names of the various genera containing the r-leader motifs within papers citing the study that introduced dRNA-seq [1]. We also performed *ad hoc* Google Scholar searches. Our remaining strategy was to search for RNA-seq studies within the Short Read Archive (SRA) [3] on the NCBI Web site, especially for the term “dRNA-seq”. However, we found that the type of experiment was not consistently stated in the SRA metadata.

Predicting the ligands of new r-leaders based on the ligands of previously established r-leader

As noted in the main text, we wish to generate a hypothesis for the ligand of each of the 20 newly found r-leaders. For r-leaders that are predicted to only regulate genes encoding one r-protein, it is clear that this r-protein is the most likely ligand. For r-leaders that regulate genes encoding multiple r-proteins, we proposed in the main text that the most likely ligand is whichever r-protein has previously been established as the ligand of an earlier r-leader. There is no experimentally established counter-example to this rule.

However, it is clear, given the relatively low number of r-leaders with validated ligands, that we cannot rule out the possibility that exceptions might be proven in the future. Also, whereas an *E. coli* r-leader exists whose ligand is L4, experiments suggested L4 does not bind the putative r-leader in *B. subtilis* that regulates similar genes [4]. While these results are suggestive, they do not establish that the ligand is not L4 [4]. First the experiments merely failed to show L4 binding, and did not validate a different ligand. Second, the experiments were conducted in a surrogate host. Despite these caveats, we believe that the use of previously established r-leader ligands is likely to provide good hypotheses for the ligands of newly predicted r-leaders.

Additional notes on motifs, including motifs not described in the main text

L2

We found an r-leader upstream of genes that encode L2 in Alphaproteobacteria (Figure S2). In *E. coli*, the L2 and L4 genes form an operon, and the L4 protein is the ligand of an r-leader. However, the Alphaproteobacterial operons lack the L4 gene. Therefore, we presume that the most likely ligand is that encoded by the immediately downstream genes, i.e., L2.

We regard this motif as a borderline prediction (Table 1), because of the relative lack of conserved nucleotides around the stems and relatively frequent non-conserved insertions (Additional files 2-4). Thus, although this motif likely functions as an r-leader, especially given the precedent that many bacterial ribosomal genes are regulated by r-leaders, the data are not as convincing as with other motifs.

L4

The L4-Archaeoglobi r-leader motif has many examples that overlap protein-coding genes, which is unusual for *cis*-regulatory RNAs. Our conclusion is that the motif is very likely to correspond to an RNA with a conserved secondary structure, and that the motif remains a credible r-leader candidate. However, in view of the unusual locations of these r-leaders, we have marked the motif's assignment as an r-leader as relatively borderline (Table 1). We explain our reasoning in detail in the following text.

Roughly 54% of L4-Archaeglobi r-leader examples (254 sequences) are found within the coding regions of genes predicted to encode RNA methyltransferases defined by Pfam [5] entry PF02598. The remaining 46% of L4-Archaeglobi r-leader examples (215 sequences) do not occur inside such genes, or in other types of genes. We believe many of the 254 r-leaders do, in fact, overlap a coding region, i.e., this overlap is not the result of a genome annotation error. This conclusion follows because of the large number of overlapping predictions, and since we observed insertions in multiples of three nucleotides among affected r-leader sequences. Such insertions suggest a coding function because each codon is three nucleotides. Additionally, of the 254 sequences that overlap these genes, there are zero stop codons in the third reading frame (i.e., starting with the third nucleotide in each sequence, which corresponds to the reading frame of the methyltransferase genes). Based on the number of codons and the frequencies of the four nucleotides, the expected number of stop codons in random nucleotides would be 217.

It also appears that many L4-Archaeoglobi motif examples do not overlap the gene, i.e., the absence of the gene is also not a genome annotation error. For example, we chose a non-overlapping motif example (in RefSeq accession NZ_CM001555.1) and compared it and its surrounding genomic nucleotides to a protein encoding an overlapping gene (protein accession WP_049993178.1) using BLASTX (which internally translates nucleotide sequences into amino acid sequences in all frames). We did not find any similarity with an E-value better than 1. This result provides additional evidence that this sequence does not code for an RNA methyltransferase like Pfam entry PF02598.

We also analyzed whether any of the 215 sequences that do not overlap a gene contained stop codons. Such stop codons would suggest the absence of coding function. We investigated all three reading frames on the sense strand, and found they all had many stop codons. In the first reading frame, there were 6,141 codons among the 215 sequences, of which 117 were stop codons. Based on the number of codons and frequencies of the four nucleotides, 206 stop codons would be expected. In the second reading frame, 313 of 6,104 codons were stop codons, versus an expected 212. In the third reading frame, 79 of 5,971 codons were stop codons, versus 200 expected. Overall, there is no clear evidence of coding function in these sequences.

One possible concern with this r-leader motif is that, in performing homology searches, we inadvertently included sequences that are not homologous. In particular, perhaps the L4-Archaeoglobi motif sequences that overlap the methyltransferase genes are not actually homologous to the motif sequences that do not overlap these genes. We therefore compared all overlapping sequences to all non-overlapping sequences using the blastn program from NCBI's BLAST [6] package with a word size of 7 and an E-value cutoff of 10^{-5}. Out of the 254 gene-overlapping sequences, 252 had a match to a non-overlapping sequence that BLAST could find. In the reverse direction, BLAST was able to match 75 L4-Archaeoglobi motifs examples that do not overlap a methyltransferase gene against motif examples that do overlap genes. Since BLAST is not designed for RNA and does not consider secondary structure, it is noteworthy that it is able to detect so many similarities. We also used the Infernal software [7] to train a covariance model on the motif examples that overlap genes, and used it to search the sequences of non-overlapping motif examples. We found 175 (out of 215) of the sequences with an E-value better than 10^{-5}, and 190 with an E-value better than 10^{-2}. Therefore, there is strong evidence that the motif examples that overlap genes are homologous to the motif examples that do not overlap genes.

Additionally, we found that an alignment of the 254 sequences that overlap genes exhibits covariation according to R-scape—including E-values better than 10^{-5}. An alignment of the 215 sequences that do not overlap RNA methyltransferase genes similarly exhibits covariation, with R-scape E-values below 10^{-5}. Thus, each group of motif examples independently has evidence of covariation.

Thus, the L4-Archaeoglobi motif likely represents an RNA that sometimes occurs within a protein-coding gene, and sometimes does not. The RNA could therefore regulate the downstream gene from transcripts that include the overlapping methyltransferase genes. Since 46% of L4-Archaeoglobi RNAs neither overlap nor are near to methyltransferase genes, it seems unlikely that the function of the RNA is primarily related to these protein-coding genes. However, the unusual arrangements of this motif reduce our confidence L4-Archaeoglobi RNAs function as r-leaders (as reflected in Table 1).

Another possibility to consider is that the motif does not function as a *cis*-regulatory RNA and does not have a conserved RNA secondary structure. However, the covariation information is significant both quantitatively (Table S2 and above text) and qualitatively. Therefore, we believe that the motif likely does function as an RNA.

L13

This motif consists of two adjacent hairpins: a 5´ and a 3´ hairpin (Figure S3). Although the 3´ hairpin in this motif is well supported by covariation, the 5´ hairpin is less clear, due to a relatively high variation in lengths of unpaired regions, and the relative absence of strong sequence conservation. This combination of features allows the possibility that the base pairs arise by chance. In this view (that the base pairs are not biological, but rather arise by chance), the variable lengths of unpaired regions give the computer great flexibility in finding spurious base pairs located somewhere in a relatively long sequence. Thus, such variable lengths can be associated with false-positive stems. Arguing against this view, however, we do notice that even sequences that share the same lengths exhibit covariation within the putative 5´

hairpin. Since the sequences have similar lengths, they appear to be related, and the covariation within these sequences is less likely to arise by chance. Moreover, if the 5´ hairpin truly occurs in these related sequences, then it is reasonable to assume that it occurs in other sequences. Therefore, this 5´ hairpin is likely of biological importance to the host, although our level of confidence is not as high as with other hairpins in the drawings of the various motifs.

L17

This motif (Figure S5) has a 5´ hairpin whose biological significance is not completely clear because of variation in lengths of unpaired regions and a relative lack of sequence conservation, similar to the case with the L13 r-leader motif, above. As with the L13 hairpin, the 5´ hairpin in the L17 r-leader motif exhibits covariation even among similar sequences. There are, however, two identical sequences in which no compelling stem can be found. We conclude that the hairpin in the L17 motif is likely to be biological, at least in most members of the motif.

L20

The r-leader is immediately upstream of the L20 gene and immediately downstream of the L35 gene. In *E. coli* and *B. subtilis*, the L35 gene occurs immediately upstream of the L20 gene, but in these organisms, the relevant r-leaders occur immediately upstream of the L35 gene. Thus, the order of the genes is the same, but the r-leader in Deltaproteobacteria occurs between the genes. Given the precedents that L20 is an r-leader ligand, we hypothesize that the new Deltaproteobacteria motif also binds L20, and that the position of the L35 gene is therefore of little importance.

L31

Zinc proteins and L31 motifs

To determine if the L31 motifs are likely to have a function related to the zinc binding properties of some L31 proteins, we analyzed the regulated proteins to determine how often the motifs regulated proteins that contained or did not contain the zinc-binding peptide motif. For the L31-Coriobacteria and L31-Firmicutes motifs, 60% and 67% of the regulated genes encode proteins with the zinc-binding element (Table S5). Thus, there is a mixture of zinc-binding and non-binding proteins encoded by the genes that are apparently regulated by these motifs. Thus, these two motifs probably have no relationship to zinc levels.

In contrast, the other three L31 motifs are consistently associated with one type of protein (Table S5), but the association is not consistent across these three motifs. In particular, while the motifs in Gammaproteobacteria and Actinobacteria are almost always associated with proteins that appear capable of binding zinc, the Corynebacteriacaea L31 motif only regulates genes encoding proteins that lack the zinc-binding pattern. It is possible that these three L31 motifs do act in zinc regulation, but it seems mostly likely that all five L31 motifs should have the same biochemical function. Moreover, we note that there is no organism that contains more than one type of these motifs. Therefore, it does not appear

to be the case that one motif regulates zinc-binding proteins, while another motif regulates proteins that do not bind zinc. Thus, if it is indeed the case that all five motifs have the same biochemical function, these data suggest that the L31 motifs are not likely to play a role in zinc homeostasis.

Other information on L31 r-leaders

Three motifs are found in the phylum Actinobacteria, specifically those distributed in the taxa Actinobacteria, Coriobacteria and Corynebacteriaceae (Table 1, "Lineage"). However, these three motifs occur in distinct organisms; no single organism contains more than one type of L31 motif.

Among the L31 motif examples in Firmicutes, the sequence CUU is found often immediately upstream of the conserved hairpin. This sequence could bind the downstream gene's Shine-Dalgarno sequence. However, a much larger number of motif instances lack the conserved CUU. Therefore, its significance is unclear. We have depicted a subset of L31 r-leaders in Firmicutes that have a CUU extension on their 5´ ends (Figure S7).

There is a potential stem in the L31-Gammaproteobacteria motif involving the SD sequence. The 5´ side of the stem involves nucleotides in the region depicted as "5-12 nt" (Fig. 2) and the GC dimer that occurs immediately 5´ to that region. The stem's 3´ side involves nucleotides in the SD sequence and the U nucleotide immediately 3´ it. We did not indicate this stem in drawings or alignments, because it is not supported by covariation. However, it is plausible and might form part of a regulatory mechanism.

The L31-Gammaproteobacteria motif has the sequence UUCUGUAU(5-7nucs)GCC 40-120 nucleotides upstream of many r-leaders. However, these sequences seem to be associated with upstream genes that encode a DEAD-like helicase. When these genes are absent, the sequence is not found. Therefore, we do not believe that this partially conserved sequence is related to the L31-Gammaproteobacteria r-leader motif.

S4

Although Firmicutes have only one previously established S4 r-leader, two distinct multiple-sequence alignments have been prepared of this r-leader (Figure S8). The first alignment to be published [8] was based on a comparative analysis of sequences upstream of S13 genes. A subsequent study [9] considered experimental data [10, 11] and adjusted the earlier alignment to conform to these experimental results. Both of these alignments exhibit a highly conserved GUAA sequence nearby to, and 5´ to a hairpin. In both alignments, there is a conserved GCU in the hairpin's terminal loop. In the earlier study [8], there is also a conserved YGA sequence 3´ to the hairpin, which is not aligned in the second study [9].

Our S4 motif in Fusobacteria has the GUAA sequence 5´ to a stem that contains a three-stem junction. (The motif has additional conserved nucleotides 5´ to the GUAA sequence, but this region is not conserved in the Firmicutes r-leader.) 3´ to the enclosing stem in the new Fusobacteria motif is a conserved YGA sequence, resembling the Firmicutes motif in the earlier study's [8] analysis. Oddly, the GUAA and YGA sequences are shifted in their positions relative to the stem when comparing the

Firmicutes motif to the Fusobacterial motif. This dissimilarity cannot be resolved by removing or adding base pairs. The Fusobacterial motif does not have a GCU sequence in either of the terminal loops that the stem encloses. However, one of the stems does have a conserved GUA sequence. It is not clear if these conserved sequences are related.

The four novel motifs differ in terms of which genes they regulate in addition to the S4 gene (*rpsD*), but the regulated genes for each new r-leader are similar to those of some previously published r-leaders. Two S4 r-leaders have been published. The Gammaproteobacteria S4-binding r-leader occurs immediately upstream of the S13 gene (*rpsM*), and S4 is encoded by the third gene in the regulated operon [12], with a gene encoding S11 in between. The relevant r-leader in Firmicutes is immediately upstream of the S4 gene, and the S13 and S11 genes are located elsewhere in the genome. Among the novel r-leaders, the Fusobacteria and Clostridia S4 motifs (Table 1) occur upstream of the S13 gene, with S11 and S4 genes located further downstream. These r-leader motifs are also immediately upstream of the L36 gene. These characteristics of the Fusobacteria and Clostridia S4 motifs are similar to those of the Gammaproteobacterial r-leader. Two other new r-leader motifs (in Bacteroidia and Flavobacteria; see Table 1) are found directly upstream of the S4 gene (*rpsD*), and genes encoding S13 and S11 are found immediately upstream of the r-leaders. This gene order is the same as in *E. coli,* but in that organism, the r-leader is upstream of the S13 gene.

S6:S18

The start codon downstream of S6:S18-Chlorobi r-leaders is conserved as UUG in annotated positions (Figure S9). Although gene annotations often contain incorrect start codons, the consistency of the predictions in all sequences suggests that the start codon was correctly annotated.

S15

The new motif upstream of S15 genes in Flavobacteria (Figure S10) has strong evidence to support its assignment as an RNA, but its function as an r-leader is less clear. The sequences belonging to the S15-associated motif in Flavobacteria contain a non-essential hairpin that exhibits covariation. The typical sequence pattern (TAxxTTTG) corresponding to a Flavobacteria promoter [13] occurs immediately upstream of the motif. The hairpin follows in most sequences, but many sequences have a short sequence (usually fewer than 6 nucleotides) that does not form base pairs. 3´ to the hairpin region are several conserved nucleotides that end in the start codon. Given the promoter sequence and the covariation in the hairpin, it is clear that this motif is transcribed as RNA. However, sequences missing the hairpin thereby lack any demonstrable secondary structure, while all r-leaders studied so far are believed to exhibit a conserved secondary structure in all cases. Moreover, we do not find any compelling similarity between the conserved nucleotides and the rRNA binding site for S15. Therefore, it is unclear whether this motif functions as an r-leader.

There are some positions within the archaeal motifs that could correspond to stacked G-C, G-U base pairs, one of the conserved features of the rRNA's S15 protein binding site. However, we judged the similarity to be uncompelling, given that this is such a simple pattern.

S16

The S16 motif in Flavobacteria (Figure S11) is consistently located upstream of genes encoding S16. Downstream of the S16 gene are usually *rimM* genes. *rimM* genes encode proteins involved in 16S rRNA processing, and do not encode an r-protein. Despite the presence of *rimM* genes, this motif likely functions as an S16-binding r-leader for two reasons. First, it is less clear that the *rimM* genes are part of the regulated operon, due to an inconsistent distance between the genes (often close to 300 nucleotides) and occasional cases in which the *rimM* gene is missing. If the *rimM* genes are, in fact, not part of the operon, then it is clear that they have no relevance for the function of the RNA motif.

Second, previously validated r-leaders are known to regulate genes that do not encode r-proteins [12]. For example, the experimentally validated L20 r-leader in *B. subtilis* is found immediately upstream of *infC* genes, which encode translation initiation factor 3 [12]. However, this r-leader binds the L20 r-protein, which is actually encoded by the third gene in the operon. Another similar example is that of the S6:S18 r-leaders validated in *E. coli* and *B.* subtilis [12]. This r-leader binds S6:S18, but regulates genes that encode single-strand-DNA-binding proteins. Thus, there are at least two established r-leaders that bind r-proteins, but regulate genes that do not encode r-proteins.

Thus, the *rimM* genes might not be part of the operon regulated by the S16-associated r-leaders. Moreover, if the *rimM* genes are part of the operon, this would not contradict the hypothesis that the motif is an S16 r-leader, given the precedents mentioned in the previous paragraph for L20 and S6:S18 r-leaders.

The S16 r-protein is not established or proposed as the ligand of any previously published r-leader.

Supplementary Tables and Figures

Table S1. Previously published or predicted r-leaders. This table is expanded from a previously published review paper [12], using data from Rfam [14] and a recent paper [15]. The table includes only motifs that are either experimentally confirmed or have a published predicted multiple-sequence alignment. “R-protein ligand”: the experimentally verified or proposed ligand protein, according to the original paper. Multiple r-leaders that exhibit distinct structures and bind the same protein ligand are listed in separate rows. In some cases, the ligand has not been proposed. In such cases, the products of the proximal genes in the downstream operon are listed. “Experimentally confirmed?”: “yes” if any confirmatory experiment was performed. Information on experimentally confirmed r-leaders is available in a previous review [12]. “Lineage”: the taxon that contains known examples of the r-leader. “Alignment citation”: citations reflect works that produced multiple-sequence alignments including multiple homologs. If such an alignment is not available, an earlier paper describing an r-leader in a single organism is given. “Rfam Database accession”: accessions are given for r-leaders present in the Rfam Database [14]. Special annotations: (a) Although the L7Ae protein is part of the archaeal ribosome, it is also present in many other RNA-protein complexes [16, 17], so it is at least not purely an r-protein. Thus, the assignment of this RNA as an r-leader depends on the precise definition of the term “r-leader”. We included it in the table, since the published r-leader has been shown to regulate the L7Ae gene by binding L7Ae. (b) The alignment in the Rfam Database is independent of the alignment present in the cited work. The alignments might complement each other. (c) The motif occurs downstream of the associated genes, and therefore likely functions in the 3´ UTR. This motif is therefore technically not an r-leader, even though it might bind the L17 r-protein and participate in feedback regulation. (d) No alignment is available for this r-leader, which is known only in one organism. (e) These alignments exhibit little or no covariation, except in a Rho-independent terminator, so the true secondary structure is unclear, and it is difficult to compare them to the other predicted S7 or S12 r-leaders. Therefore, it is uncertain if these are truly distinct structures. (f) S10 refers to the product of the first gene in an operon that contains many genes that each might encode the ligand of this gene. The correct ligand has not been experimentally determined.

R-protein ligand (confirmed or predicted)	Experimentally confirmed?	Lineage	Alignment citation	Rfam Database accession (if any)
L1	Yes	Archaea & Bacteria	[18]	
L4	Yes	Gammaproteobacteria	[18]	
L7Ae (a)	Yes	Archaea	[16, 17]	
L10	Yes	Bacteria	[18]	RF00557 (b)
L13, S9	No	Firmicutes	[8]	RF00555
L13	Yes	Gammaproteobacteria	[15]	
L17 (c)	No	Firmicutes	[19]	RF01708

L19	No	Firmicutes	[8]	RF00556
L20	Yes	Firmicutes	[9]	RF00558
L20	Yes	Gammaproteobacteria	[18]	
L20	Yes	*E. coli* (d)	[20]	
L21, L27	No	Firmicutes	[8]	RF00559
L25	Yes	Enterobacteria	[21]	
L28	No	Gammaproteobacteria	[15]	
L34	No	Gammaproteobacteria	[15]	
S1	No	Cyanobacteria	[22]	
S1	Yes	Gammaproteobacteria	[18]	
S2	No	*Pelagibacter*	[23]	RF01815
S2	Yes	Bacteria	[18]	RF00127 (b)
S4	Yes	Gammaproteobacteria	[18]	RF00140
S4	Yes	Firmicutes	[9]	
S6:S18	Yes	Bacteria (but not Chlorobi)	[24, 25]	
S7	Yes	Gammaproteobacteria	[18]	
S7, S12 (e)	No	*Pelagibacter*	[23]	RF01823
S7, S12 (e)	No	*Pseudomonas*	[26]	RF01773
S7, S12 (e)	No	*Rickettsia*	[26]	RF01774
S8	Yes	Gammaproteobacteria	[18]	
S10 (f)	No	Firmicutes	[8]	
S15	No	Actinobacteria	[27]	
S15	Yes	Alphaproteobacteria	[27]	
S15	No	*Chlamydia* (d)	[27]	
S15	Yes	Firmicutes	[9]	
S15	Yes	Gammaproteobacteria	[18]	RF00114
S15	Yes	*Thermus thermophilus* (d)	[28]	
S20	Yes	*E. coli* (d)	[29]	

Table S2. Base pair and covariation statistics of predicted r-leaders, including statistical support of RNA covariation from R-scape. Note: due to issues described in Methods, we additionally evaluated covariation information manually. This table provides the following information for each predicted r-leader (including the three r-leaders we found that strongly resemble previously established leaders). “Number of seqs.”: the number of instances of the r-leaders within the sequence databases we searched. Note: the 29,730 r-leader examples mentioned in the main text do not include the three motifs (L19-Flavobacteria, L25-Gammaproteobacteria and S10-Clostridia) that we concluded are very similar in conserved features to previously published r-leader motifs. “Avg. len.”: the average length in nucleotides of the motif. “All”: the total number of base pairs in the alignment. Note: this includes aligned columns that have gaps in the vast majority of motif members, since there is often significant variation in the lengths of stems. “Invariant”: the Watson-Crick or G-U base pair is not observed to change. (Corresponds to red shading in our diagrams.) “Covary (R2R)”: covariation is predicted by R2R, but not by R-scape. (Corresponds to blue shading in our diagrams.) “Covary (R-scape)”: covariation is predicted by R-scape (and possibly also by R2R). (Corresponds to green shading in our diagrams.) Covariation predicted by R-scape [30] is statistically significant and has an E-value < 0.05. (E-values are similar to p-values.) Note: base pairs in gappy columns are generally not drawn in the diagrams, but are reflected in the numbers in this table. Therefore, these numbers will generally be higher than the number of base pairs in the corresponding diagrams.

r-leader	Number of seqs.	Avg. len.	Number of base pairs			
			All	Invariant	Covary (R2R)	Covary (R-scape)
L2-Alphaproteobacteria	808	70	42	9	3	13
L4-Archaeoglobi	654	94	49	5	10	24
L13-Bacteroidia	1380	76	26	3	5	16
eL15-Euryarchaeota	1001	57	153	46	27	11
L17-Actino-Proteobacteria	548	56	26	6	6	13
L19-Flavobacteria	2361	28	9	3	1	5
L20-Deltaproteobacteria	202	68	25	4	12	6
L25-Gammaproteobacteria	6732	26	139	19	36	18
L31-Actinobacteria	1737	49	40	3	9	11
L31-Coriobacteria	303	33	8	1	2	4
L31-Corynebacteriaceae	360	76	51	5	10	8
L31-Firmicutes	8296	32	18	0	6	6
L31-Gammaproteobacteria	8743	54	27	4	8	11

S4-Bacteroidia	558	38	10	2	3	5
S4-Clostridia	496	65	65	3	26	17
S4-Flavobacteria	931	34	11	1	0	7
S4-Fusobacteriales	561	65	36	5	12	11
S6:S18-Chlorobi	67	78	31	4	14	6
S10-Clostridia	1294	86	41	4	19	13
S15-Flavobacteria	2449	62	26	7	10	6
S15-Halobacteria	285	67	55	14	7	11
S15-Methanomicrobia	194	79	49	21	10	7
S16-Flavobacteria	157	35	11	2	3	3

Table S3. Additional data on experimentally annotated transcription start sites (TSSes) and predicted r-leaders. This table presents data underlying and relating to Figure S12. “R-leader name”: as in Figure S12. “Organism (RefSeq sequence accession)”: the full name of the organism, and the RefSeq [31] accession of the relevant chromosome. “Citation”: a citation of the source of the TSS data. The coordinates in these papers refers to the RefSeq sequence accession given in the previous column. (In some cases, they refer to a sequence in the GenBank database [32] that is identical to the given RefSeq accession.) ”Upstream gene position”: the coordinates of the 5′ and 3′ ends of the upstream gene (which may encode a protein or a tRNA). If the 5′ coordinate (first number) is greater than the 3′ coordinate (second number), then the upstream gene is located on the reverse-complement strand of the RefSeq sequence. This information comes from the RefSeq genome annotation. “Position of TSS(es)”: list of TSS positions. This information comes from the given citation. “R-leader position”: the coordinates of the 5′ and 3′ ends of the r-leader regulating the given gene (next column). This information comes from our alignments (Additional file 2). “Regulated gene”: the coordinates of the 5′ and 3′ ends of the gene immediately downstream of the r-leader. We predicted that this gene is regulated by the r-leader. This information comes from the RefSeq genome annotation. “TSS pos.”: same meaning as in Figure S12. These values can be calculated from the “Position of TSS(es)” and “R-leader position” columns.

R-leader name	Organism (RefSeq sequence accession)	Citation	Upstream gene position	Position of TSS(es)	R-leader position	Regulated gene position	TSS pos.
eL15-Euryarchaeota	*Haloferax volcanii* DS2 (NC_013967.1)	[33]	497121-496045	495928	495895-495835	495816-495226	-33
eL15-Euryarchaeota	*Thermococcus kodakarensis* KOD1 (NC_006624.1)	[34]	1280610-1281680	1280551	1280545-1280502	1280482-1279898	-6
L20-Delta-proteobacteria	*Geobacter sulfurreducens* (NC_002939.5)	[35]	1664628-1664825	1664821, 1664819, 1664816	1664846-1664908	1664915-1665268	-25
L31-Actinobacteria	*Streptomyces coelicolor* A3(2) (NC_003888.3)	[36]	5828747-5829895	5829951	5829950-5829996	5830020-5830244	+1
L31-Coryne-bacteriaceae	*Coryne-bacterium glutamicum*	[37]	928712-928398	928804,928836,928840	928877-928932	928944-929210	-37

	ATCC 13032 (NC_006958.1)						
L31-Firmicutes	*Bacillus licheniformis* DSM 13 (NC_006322.1)	[38]	3780008-3781291	3779958	3779923-3779890	3779880-3779680	-35
L31-Firmicutes	*Staphylo-coccus aureus* USA300-ISMMS1 (NC_010079.1)	[39]	2237762-2236446	2236392	2236367-2236338	2236328-2236074	-25
L31-Gammaproteo-bacteria	*E. coli* (NC_000913.3)	[40]	4126810-4124612	4126908, 4126911, 4126913	4126954-4127006	4127013-4127225	-41
L31-Gammaproteo-bacteria	*Shewanella oneidensis* MR-1 (NC_004347.2)	[41]	4280501-4279464	4279371, 4279364	4279346-4279296	4279288-4279076	-18
S6:S18-Chlorobi	*Chlorobaculum tepidum* TLS (NC_002932.3)	[42]	2021087-2020326	2020246	2020240-2020165	2020134-2019739	-6
S15-Halobacteria	*Haloferax volcanii* DS2 (NC_013967.1)	[33]	1048559-1048630	1048442	1048444-1048382	1048314-1047847	+2

Table S4. R-leaders and their operons in *E. coli* and *B. subtilis*. Based on data compiled in [12]. The column “Distinct RNA structures?” refers to whether the *E. coli* and *B. subtilis* r-leaders belong to different structural classes. Question marks indicate cases where no relevant r-leader in *B. subtilis* has been established or its ligand has not been experimentally determined. Out of eight r-leaders in *E. coli* that regulate operons containing multiple genes, five of their protein ligands are also ligands of an r-leader in *B. subtilis*, and in two of these five cases, the r-leaders are not structurally related. In the remaining three cases, no *B. subtilis* r-leader is known for any of the r-proteins encoded by the operons. The data in this table support two conclusions regarding multi-gene operons, as described in the main text. First, for r-leaders that regulate multi-gene operons, the ligand is often not encoded by the immediately downstream gene. For example, the top row shows that the immediately downstream gene of the L1-binding r-leader in *E. coli* actually encodes L11. Thus, the ligand (L1) is not encoded by the immediately downstream gene (which encodes L11). Second, this table also agrees with the hypothesis that certain r-proteins are the target of multiple r-leaders in multiple organisms, even when the r-leaders exhibit distinct structures. For example, the fifth row concerns the two r-leader motifs that bind L20, one in *E. coli* and one in *B. subtilis*. In both cases, L20 is the ligand, even though L35 is also encoded by a gene in the regulated operon.

R-proteins encoded by operon in *E. coli* (in order of genes)	Ligand of r-leader in *E. coli*	R-proteins encoded by operon in *B. subtilis* (in order of genes)	Ligand of r-leader in *B. subtilis*	Distinct RNA structures?
L11, L1	L1	L1	L1	No
S10, L3, L4, L23, L2, S19, L22, S3, L16, L29, S17, L14, L24	L4	S10, L3, L4, L23, L2, S19, L22, S3, L16, L29, S17, L14, L24, L5, S14, S8, L6, L18, S5, L30, L15	?	?
L10:L7/L12	L10:L7/L12	L10:L7/L12	L10:L7/L12	No
L13, S9	L13		?	?
L35, L20	L20	L35, L20	L20	Yes
L25	L25		?	?
S1	S1		?	?
S2	S2	S2	S2	No
S13, S11, S4, L17	S4	S4	S4	Yes

S6:S18, L9	S6:S18	S6:S18	S6:S18	No
S7	S7		?	?
L5, S14, S8, L6, L18, S5, L30, L15	S8	S10, L3, L4, L23, L2, S19, L22, S3, L16, L29, S17, L14, L24, L5, S14, S8, L6, L18, S5, L30, L15	?	?
S15	S15	S15	S15	Yes
S20	S20		?	?

Table S5. Association of L31 r-leader motifs with CXXC zinc-binding peptide motifs. Some r-motifs (i.e., the L31 r-leader motifs in Coriobacteria and Firmicutes) do not appear to correlate with zinc-binding. These motifs are very unlikely to play a role in zinc homeostasis. The remaining three motifs are either consistently associated with zinc-binding or consistently associated with non-zinc-binding proteins. It is possible that one or more of these three motifs is a regulator related to zinc, but we find this possibility less likely in view of the fact that the five motifs are not consistent with each other. See Supplementary Text for details.

Motif Name	Number of regulated genes encoding complete proteins with CXXC (i.e., zinc-binding)	Number of regulated genes encoding complete proteins lacking CXXC (i.e., non-zinc-binding)	Percentage that contain CXXC
L31-Actinobacteria	2052	0	100.0%
L31-Coriobacteria	50	33	60.2%
L31-Corynebacteriaceae	0	100	0.0%
L31-Firmicutes	555	277	66.7%
L31-Gammaproteobacteria	500	1	99.8%

Table S6. Alignments from the Rfam Database [14] that were used to analyze binding sites within rRNAs.

Domain of life	rRNA molecule	Rfam accession
Bacteria and Archaea	5S	RF00001
Bacteria	16S	RF00177
Bacteria	23S	RF02541
Archaea	16S	RF01959
Archaea	23S	RF02540

Table S7. Papers used to analyze rRNA nucleotides that are known to interact with specific r-proteins. All r-proteins were analyzed using PyMol as described in Methods, but where a paper was available, we found that the PyMol results did not deviate significantly from the previously established interactions.

R-protein	Citations
L2	[43–45]
L3	[46, 47]
L4	[48, 49]
L6	[50, 51]
L13	None
L15	[52]
L17	None
L19	None
L20	[20, 53, 54]
L25	[55, 56]
L31	None
S4	[57–59]
S6	[24, 25]
S13	[60, 61]
S15	[12, 27, 62, 63]
S16	[64]

Figure S1 (next page)**.** New r-leader motifs whose structural features are essentially the same as a previously published r-leader. Left panel: novel motifs (this study); the text “novel” refers to the alignments produced in this work. Middle panel: relevant binding sites in rRNA. Right panel: previously published r-leaders binding the same r-proteins that were experimentally validated or computationally predicted, and that have published multiple-sequence alignments. Annotations are the same as in Figs. 2 and 3. Helix numbers refer to the same source as Fig. 3. All drawings of previously predicted r-leaders in all Figures S1-S11 use alignments that were included as supplementary data of the relevant paper or are available in the Rfam Database [14] (identifying information in Table S1). (**a**) L25 r-leader in Gammaproteobacteria. Our alignment is similar to the previously published alignment [21], but has additional potential hairpins and is present in a wider variety of Gammaproteobacteria. The binding site in 5S rRNA for L25 is shown, with yellow shading as in Fig. 3. The previously published alignment was not made available in machine-readable format, and is therefore not shown. (**b**) S10 r-leader in Fusobacteria (this study). Two disjoint S10-binding regions in 16S rRNA and the L4-binding region are shown in the middle column. S10 is encoded by the immediately downstream gene, and L4 is a previously established r-leader ligand [8]. The yellow shading of the r-leader motifs shows a possible similarity with the rRNA binding site for S10, based on our analysis. (**c**) L19 r-leader in Flavobacteria. The internal loop enclosed by a C-G and a U-A base pair significantly resembles the previously published Firmicutes motif [8]. Two relevant binding sites in different parts of 16S rRNA and in 23 rRNA are shown. The figure appears on the next page.

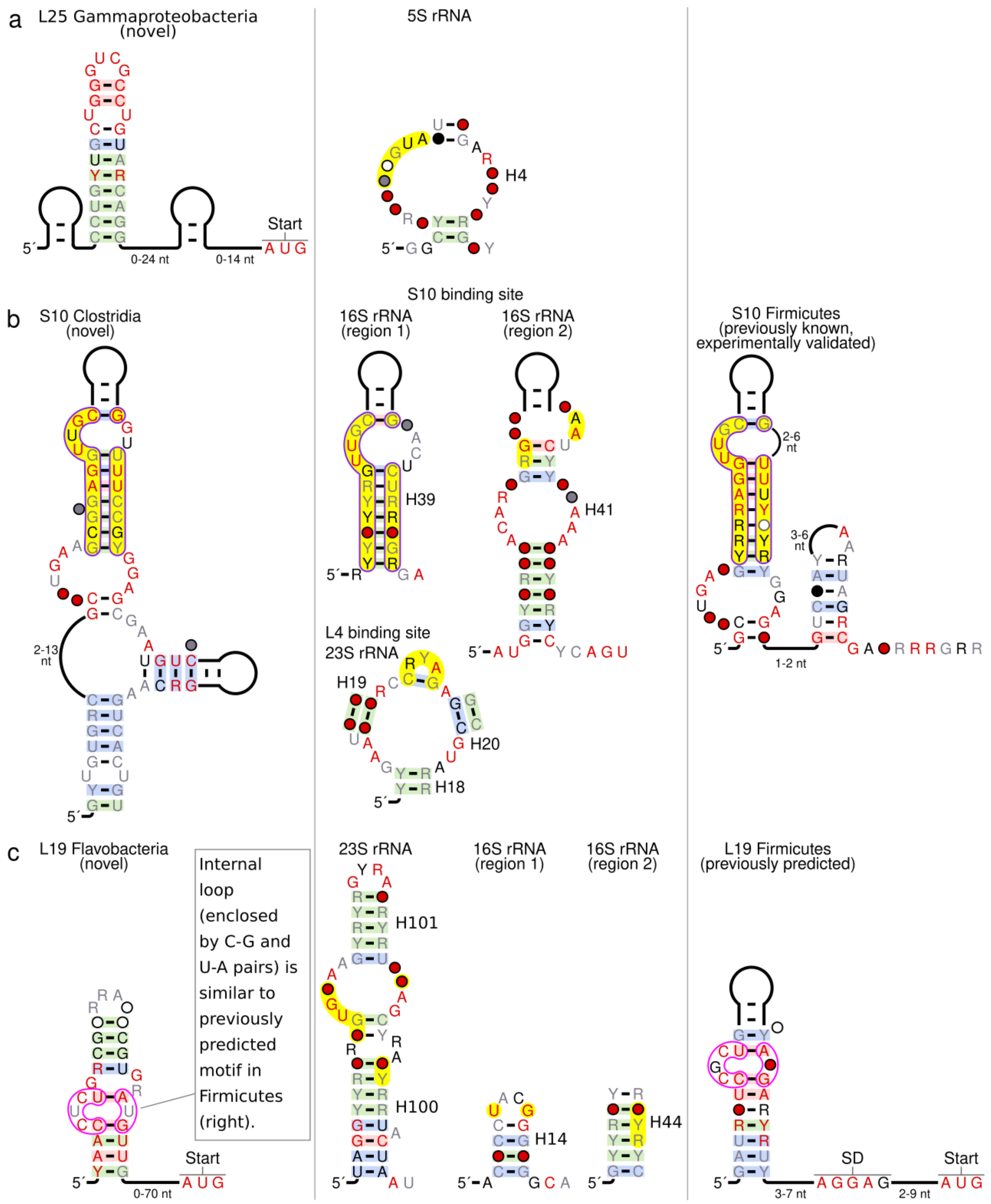

a
L25 Gammaproteobacteria
(novel)
Start
0-24 nt
0-14 nt
5S rRNA
H4
b
S10 Clostridia
(novel)
2-13 nt
S10 binding site
16S rRNA
(region 1)
H39
16S rRNA
(region 2)
H41
L4 binding site
23S rRNA
H19
H20
H18
S10 Firmicutes
(previously known,
experimentally validated)
2-6 nt
3-6 nt
1-2 nt
c
L19 Flavobacteria
(novel)
Internal loop (enclosed by C-G and U-A pairs) is similar to previously predicted motif in Firmicutes (right).
Start
0-70 nt
23S rRNA
H101
H100
16S rRNA
(region 1)
H14
16S rRNA
(region 2)
H44
L19 Firmicutes
(previously predicted)
SD
Start
3-7 nt
2-9 nt

Figure S2. Novel r-leaders, rRNA and previously established r-leaders related to r-proteins L2 and L4. The left, middle and right panels have the same meaning as in Figure S1. Annotations are the same as in Figs. 2 and 3. Helix numbers refer to the same source as Figure 3.

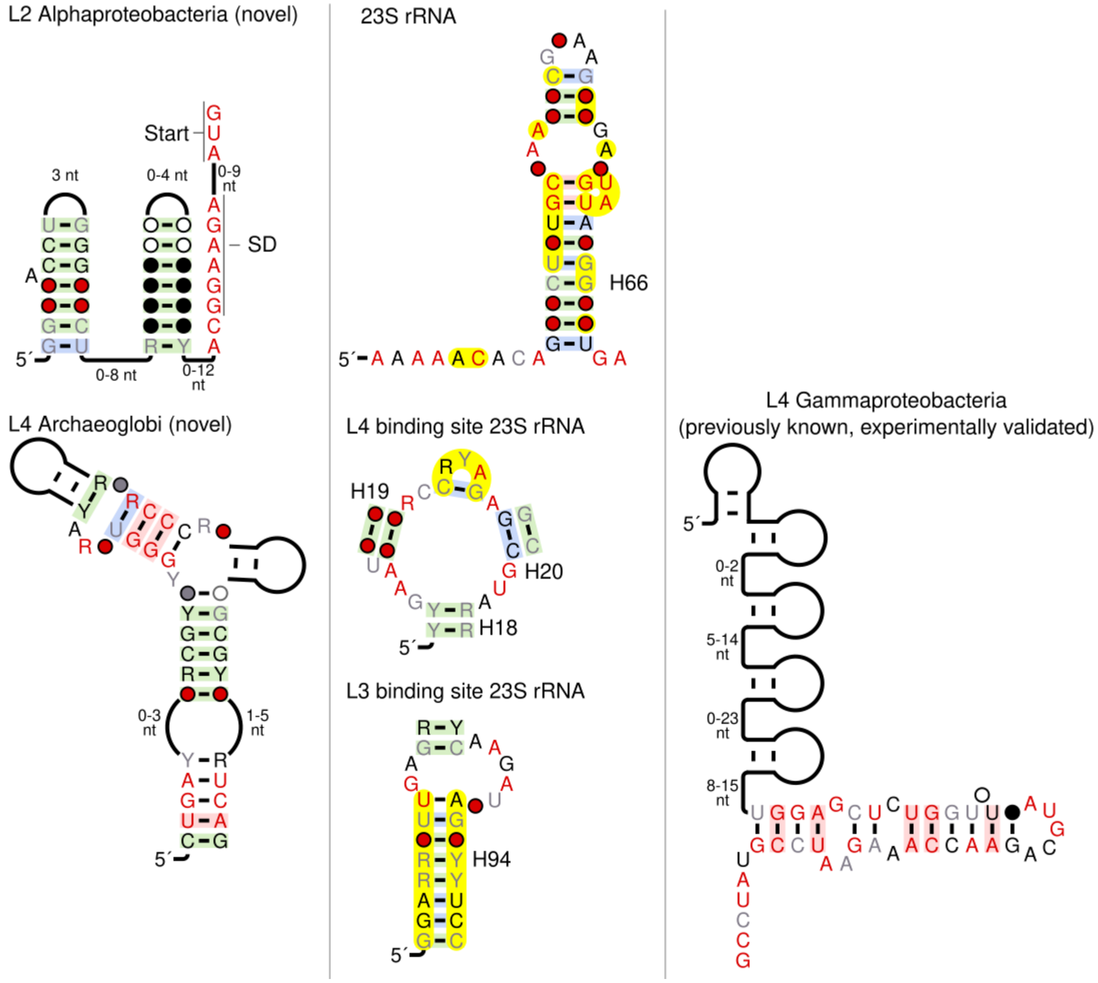

Figure S3. Novel r-leader, rRNA and previously established r-leaders related to r-protein L13. The left, middle and right panels have the same meaning as in Figure S1. Two disjoint regions of the rRNA make contact with the r-protein. Annotations are the same as in Figs. 2 and 3. Helix numbers refer to the same source as Fig. 3.

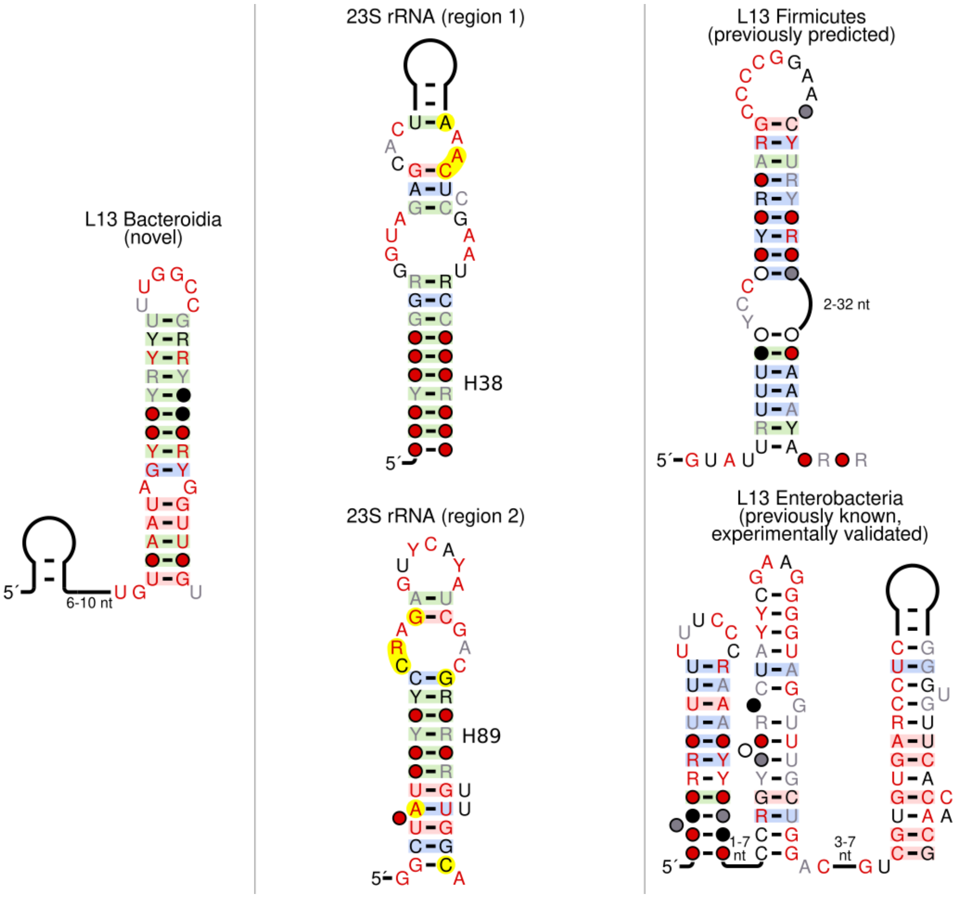

Figure S4. Novel r-leader, rRNA and previously established r-leaders related to r-protein eL15. The left, middle and (empty) right panels have the same meaning as in Figure S1. The right panel is empty because no eL15 r-leader has previously been established or predicted. The information in this figure is the same as in Fig. 3. We included it as a supplementary figure so that the supplementary figures are comprehensive. Annotations are the same as in Figs. 2 and 3. Helix numbers refer to the same source as Fig. 3.

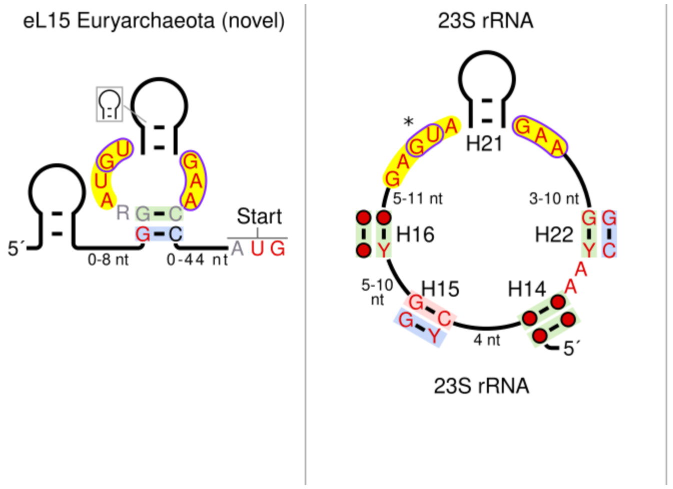

Figure S5. Novel r-leader, rRNA and previously established r-leaders related to r-protein L17. The left, middle and right panels have the same meaning as in Figure S1. The previously published L17 Downstream Element (L17DE) motif [19] is shown. However, if the L17DE motif does bind the L17 protein, it would function in the 3´ UTR, and therefore would not fit the strict definition of an r-leader. Annotations are the same as in Figs. 2 and 3. Helix numbers refer to the same source as Fig. 3.

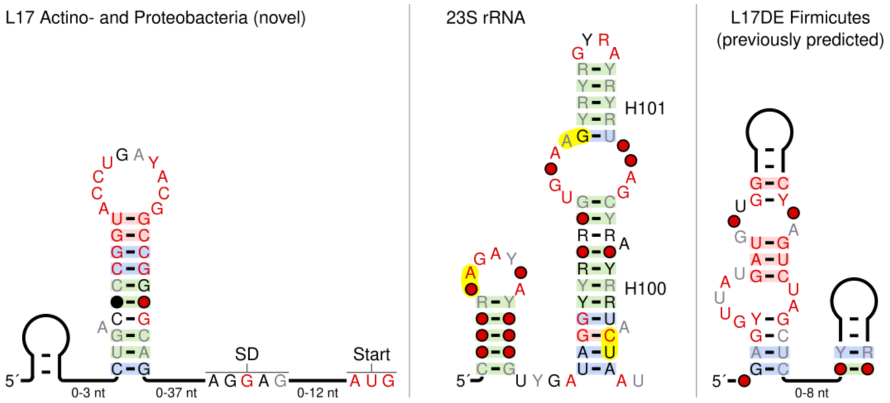

Figure S6. Novel r-leader, rRNA and previously established r-leaders related to r-protein L20. The left, middle and right panels have the same meaning as in Figure S1. An additional previously published L20 r-leader in Gammaproteobacteria is not shown because no alignment for it is available [12]. Annotations are the same as in Figs. 2 and 3. Helix numbers refer to the same source as Fig. 3.

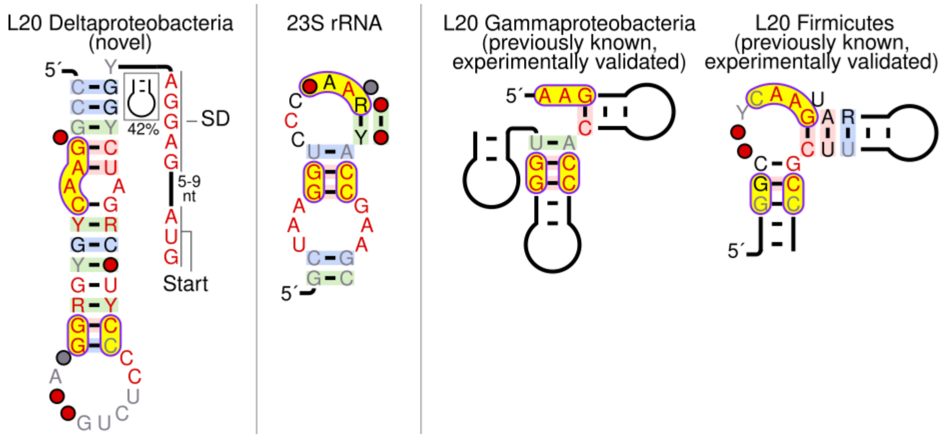

Figure S7. Novel r-leaders, rRNA and previously established r-leaders related to r-protein L31. The left, middle and right panels have the same meaning as in Figure S1. A version of the Firmicutes L31 motif with a conserved CUU sequence on its 5’ end is depicted. This version of the motif was not depicted in Fig. 2 because we are not persuaded that the CUU sequence is truly of biological significance (see Supplementary text). The right panel is empty, because no r-leaders for L31 have previously been published. Annotations are the same as in Figs. 2 and 3. Helix numbers refer to the same source as Fig. 3.

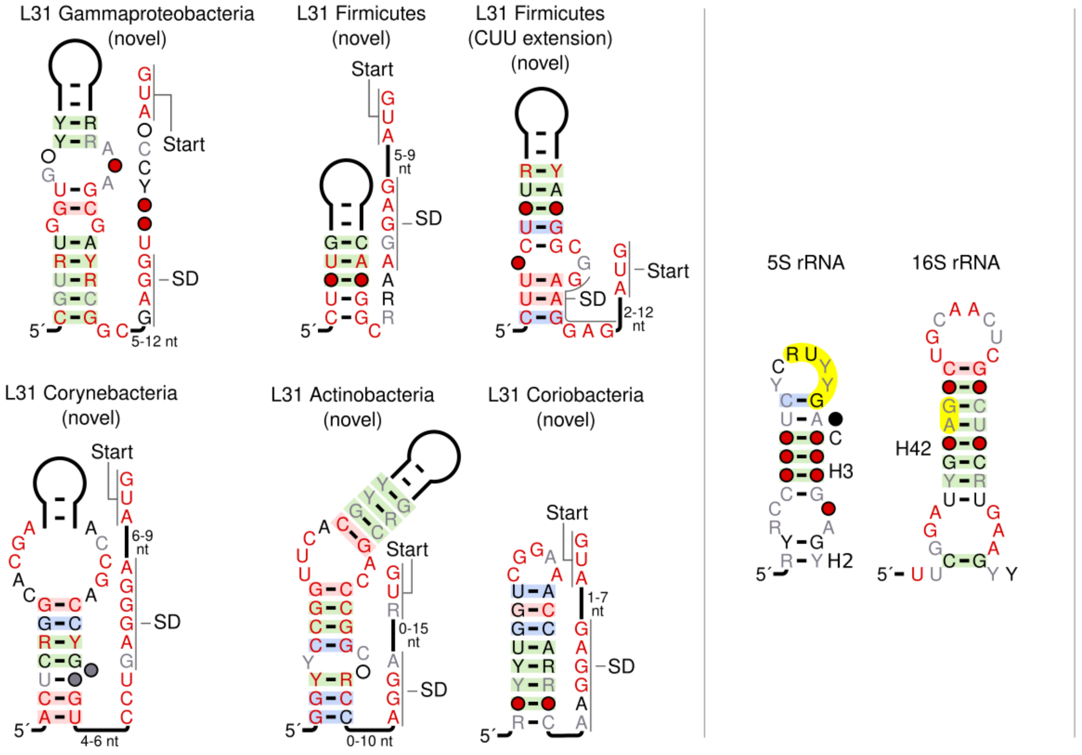

Figure S8 (next page)**.** Novel r-leaders, rRNA and previously established r-leaders related to r-protein S4. The left, middle and right panels have the same meaning as in Figure S1. An additional region of 16S rRNA that interacts with the S4 is shown that is not depicted in Fig. 3. This region was not depicted in Fig. 3 because it does not seem similar to any of the motifs. The rRNA binding site of S13 is also shown because two of the motifs are found immediately upstream of genes that encode S13. We did not, however, find any meaningful evidence of rRNA imitation related to S13. Two versions of an alignment of S4 leaders in Firmicutes have been published. We compared our motifs to both (see text). They are labelled in the figure as "(previously known, Yao, *et al.*)" [8] and "(previously known, Deiorio-Haggar, *et al.*)" [9]. Annotations are the same as in Figs. 2 and 3. Helix numbers refer to the same source as Fig. 3. The figure appears on the next page.

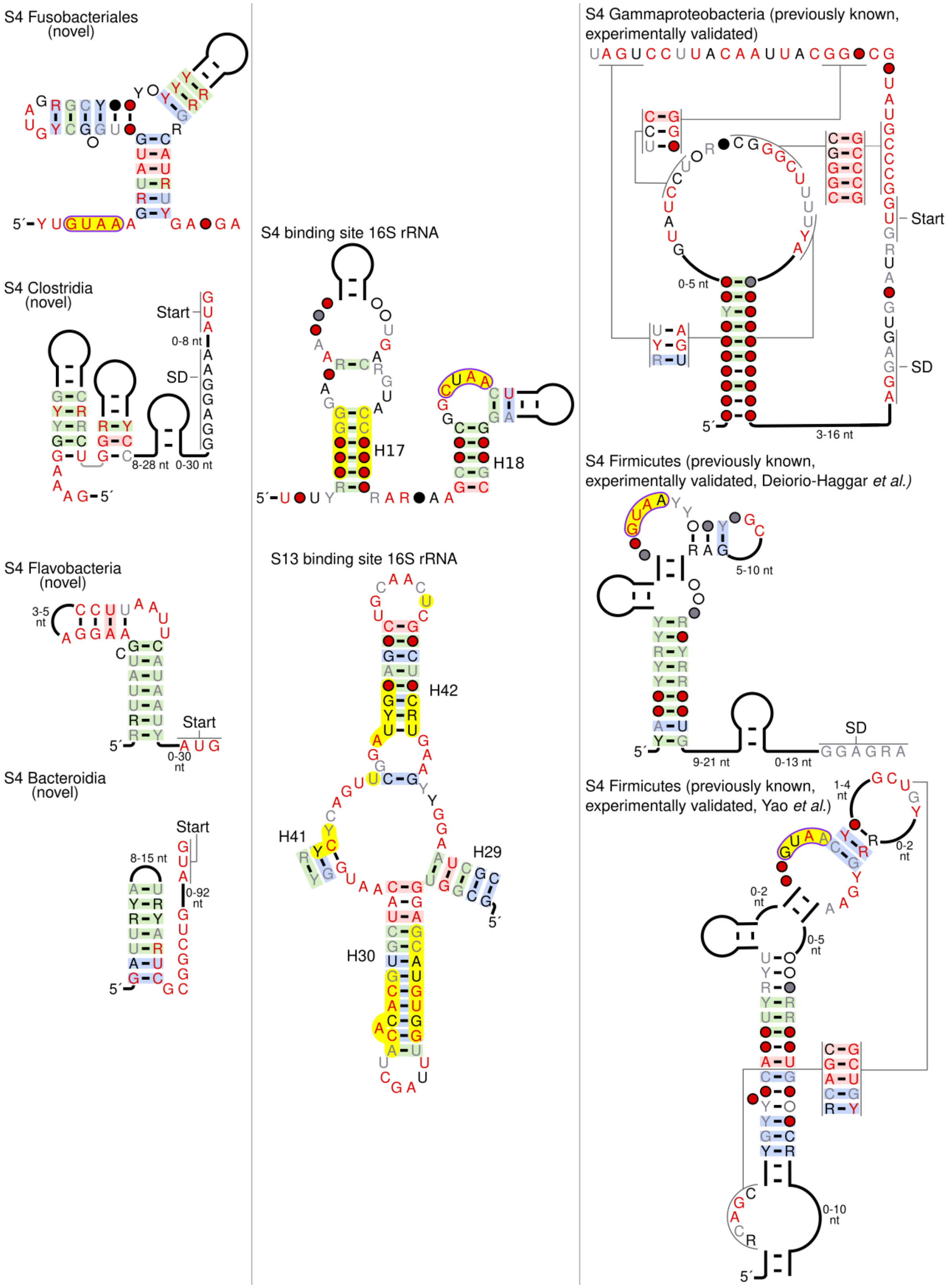
S4 Fusobacteriales (novel)
S4 Clostridia (novel)
Start
SD
8-28 nt
0-30 nt
0-8 nt
S4 Flavobacteria (novel)
3-5 nt
Start
0-30 nt
S4 Bacteroidia (novel)
Start
8-15 nt
0-92 nt
S4 binding site 16S rRNA
H17
H18
S13 binding site 16S rRNA
H42
H41
H29
H30
S4 Gammaproteobacteria (previously known, experimentally validated)
Start
SD
0-5 nt
3-16 nt
S4 Firmicutes (previously known, experimentally validated, Deiorio-Haggar et al.)
5-10 nt
SD
9-21 nt
0-13 nt
S4 Firmicutes (previously known, experimentally validated, Yao et al.)
1-4 nt
0-2 nt
0-2 nt
0-5 nt
0-10 nt

Figure S9. Novel r-leader, rRNA and previously established r-leaders related to r-proteins S6:S18. The left, middle and right panels have the same meaning as in Figure S1. Annotations are the same as in Figs. 2 and 3. Helix numbers refer to the same source as Fig. 3.

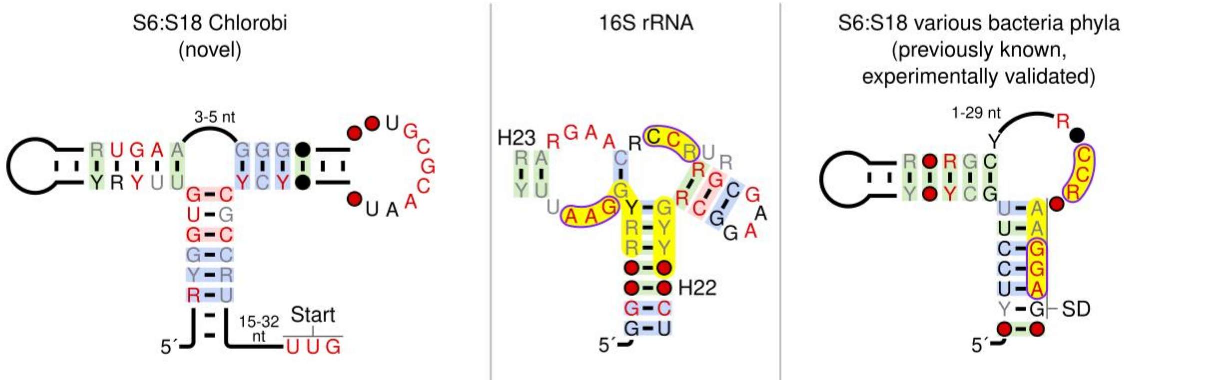

Figure S10. Novel r-leaders, rRNA and previously established r-leaders related to r-protein S15. The left, middle and right panels have the same meaning as in Figure S1. The putative promoter upstream of the Flavobacteria S15 r-leader motif (see Supplementary Text) is shown using DNA nucleotides (i.e., T instead of U), and there is a gap between the DNA sequence and the potential start of the RNA sequence. The hairpin depicted in cartoon form exhibits covariation, but is only present in 89% of the sequences. Archaeal and bacterial versions of the rRNA binding site are shown, due to the mixture of bacterial and archaeal motifs we found. The previously predicted S15 r-leader in *Thermus thermophilus* is not shown because no alignment is available. Annotations are the same as in Figs. 2 and 3. Helix numbers refer to the same source as Fig. 3.

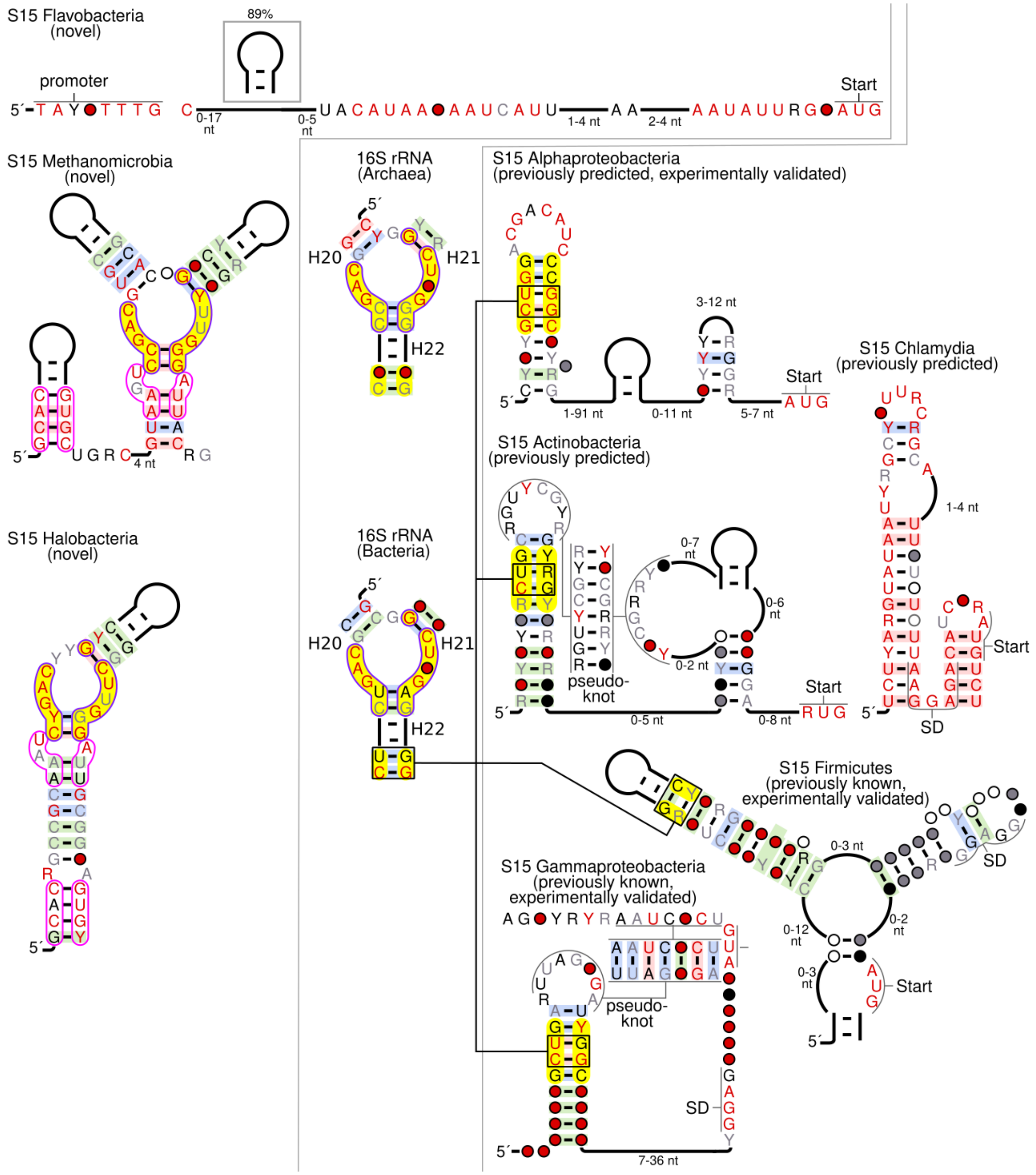

S15 Flavobacteria
(novel)
89%
promoter
Start
0-17 nt
0-5 nt
1-4 nt
2-4 nt
S15 Methanomicrobia
(novel)
4 nt
16S rRNA
(Archaea)
H20
H21
H22
S15 Alphaproteobacteria
(previously predicted, experimentally validated)
3-12 nt
1-91 nt
0-11 nt
5-7 nt
Start
S15 Chlamydia
(previously predicted)
1-4 nt
Start
SD
S15 Actinobacteria
(previously predicted)
0-7 nt
0-6 nt
0-2 nt
pseudo-
knot
0-5 nt
0-8 nt
Start
S15 Halobacteria
(novel)
16S rRNA
(Bacteria)
H20
H21
H22
S15 Firmicutes
(previously known,
experimentally validated)
0-3 nt
0-12 nt
0-2 nt
0-3 nt
SD
Start
S15 Gammaproteobacteria
(previously known,
experimentally validated)
pseudo-
knot
SD
7-36 nt

Figure S11. Novel r-leader, rRNA and previously established r-leaders related to r-protein S16. The left, middle and right panels have the same meaning as in Figure S1. No S16 r-leaders have previously been proposed. Annotations are the same as in Figs. 2 and 3. Helix numbers refer to the same source as Fig. 3.

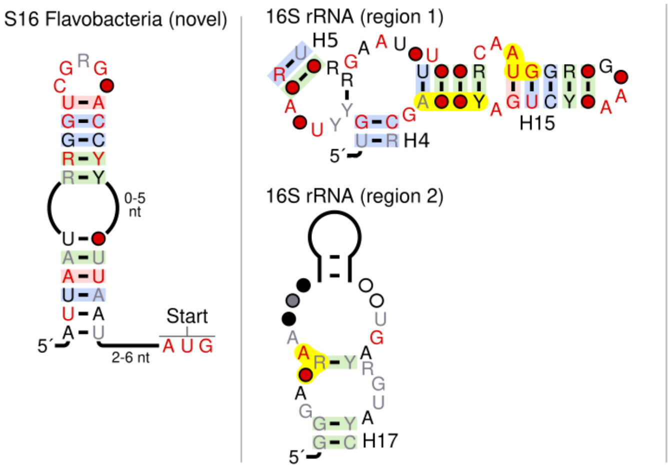

Figure S12. Experimentally determined transcription start sites (TSSes) are consistent with a *cis*-regulatory role for our r-leader motifs. We found experimentally annotated TSS data for 11 specific genes that we predicted to be regulated by an example of one of the 20 new r-leaders (see Additional file 1: Supplementary text, under sub-heading “Candidate r-leaders are consistent with available transcriptomic data”). “R-leader name”: predicted ligand and lineage of relevant r-leader (refers to Table 1). Some lineages are abbreviated. “Organism”: the organism in which TSS experiments were conducted. “TSS pos.”: position of the TSS relative to the 5′ end of the r-leader. If there are multiple TSSes, the TSS nearest the gene is used. Negative values mean that the TSS is upstream of the r-leader, so the r-leader is transcribed. Positive values mean that the given number of r-leader nucleotides would be skipped. These numbers are low (at most +2; see text). Genome cartoons are to scale (scale bar: lower right). Genes and r-leaders are depicted as arrows whose direction corresponds to their DNA strand. The TSS or TSSes for the putatively regulated gene are shown as vertical lines, with a thin arrow above them. The name of the regulated gene is given. Additional data, citations and underlying numbers are provided (Additional file 1: Table S3).

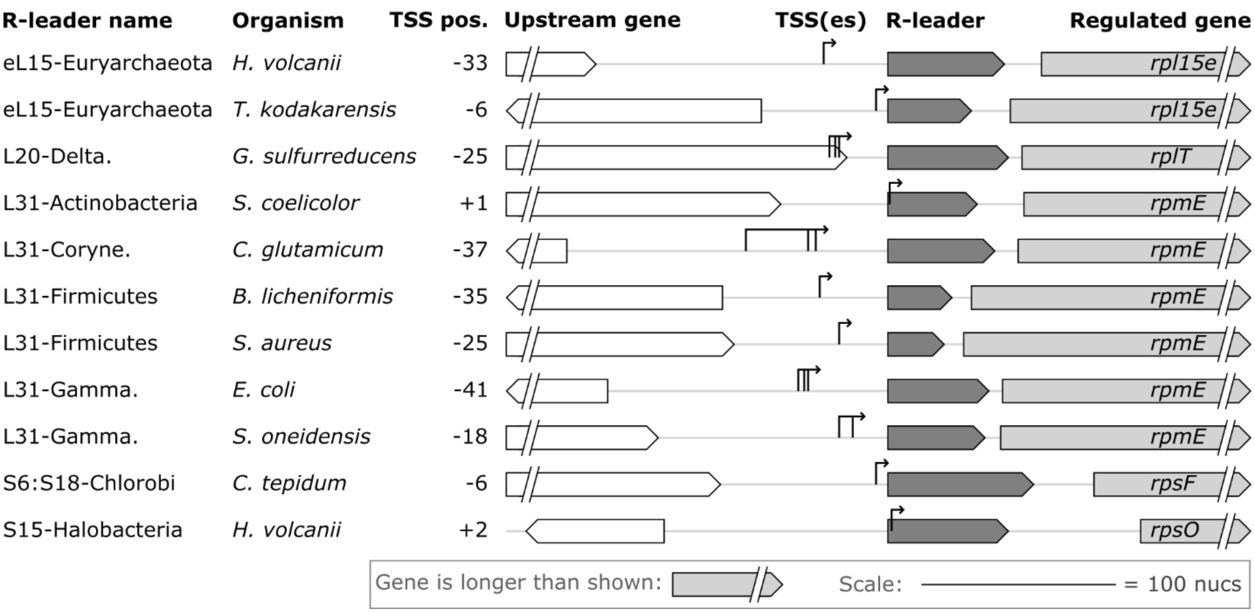

References

1. Sharma CM, Hoffmann S, Darfeuille F, Reignier J, Findeiss S, Sittka A, et al. The primary transcriptome of the major human pathogen *Helicobacter pylori*. Nature. 2010;464:250–5.
2. Google LLC. https://scholar.google.com/. Accessed 1 Nov 2019.
3. Database Resources of the National Center for Biotechnology Information. Nucleic Acids Res. 2017;45:D12-D17.

4. Li X, Lindahl L, Sha Y, Zengel JM. Analysis of the *Bacillus subtilis* S10 ribosomal protein gene cluster identifies two promoters that may be responsible for transcription of the entire 15-kilobase S10-*spc*-α cluster. J Bacteriol. 1997;179:7046–54.
5. Finn RD, Mistry J, Tate J, Coggill P, Heger A, Pollington JE, et al. The Pfam protein families database. Nucleic Acids Res. 2010;38:D211-22.
6. Altschul SF, Madden TL, Schaffer AA, Zhang J, Zhang Z, Miller W, Lipman DJ. Gapped BLAST and PSI-BLAST: a new generation of protein database search programs. Nucleic Acids Res. 1997;25:3389–402.
7. Nawrocki EP, Eddy SR. Infernal 1.1: 100-fold faster RNA homology searches. Bioinformatics. 2013;29:2933–5.
8. Yao Z, Barrick J, Weinberg Z, Neph S, Breaker R, Tompa M, Ruzzo WL. A computational pipeline for high-throughput discovery of *cis*-regulatory noncoding RNA in prokaryotes. PLoS Comput Biol. 2007;3:e126.
9. Deiorio-Haggar K, Anthony J, Meyer MM. RNA structures regulating ribosomal protein biosynthesis in bacilli. RNA Biol. 2013;10:1180–4.
10. Grundy FJ, Henkin TM. Characterization of the *Bacillus subtilis rpsD* regulatory target site. J Bacteriol. 1992;174:6763–70.
11. Grundy FJ, Henkin TM. The *rpsD* gene, encoding ribosomal protein S4, is autogenously regulated in *Bacillus subtilis*. J Bacteriol. 1991;173:4595–602.
12. Meyer MM. rRNA mimicry in RNA regulation of gene expression. Microbiol Spectr. 2018;6:RWR-0006-2017.
13. Bayley DP, Rocha ER, Smith CJ. Analysis of *cepA* and other *Bacteroides fragilis* genes reveals a unique promoter structure. FEMS Microbiol Lett. 2000;193:149–54.
14. Kalvari I, Argasinska J, Quinones-Olvera N, Nawrocki EP, Rivas E, Eddy SR, et al. Rfam 13.0: Shifting to a genome-centric resource for non-coding RNA families. Nucleic Acids Res. 2018;46:D335-D342.
15. Mustoe AM, Busan S, Rice GM, Hajdin CE, Peterson BK, Ruda VM, et al. Pervasive regulatory functions of mRNA structure revealed by high-resolution SHAPE probing. Cell. 2018;173:181-195.e18.
16. Huang L, Ashraf S, Lilley DMJ. The role of RNA structure in translational regulation by L7Ae protein in archaea. RNA. 2019;25:60–9.
17. Daume M, Uhl M, Backofen R, Randau L. RIP-Seq Suggests Translational Regulation by L7Ae in Archaea. MBio 2017. doi:10.1128/mBio.00730-17.
18. Fu Y, Deiorio-Haggar K, Anthony J, Meyer MM. Most RNAs regulating ribosomal protein biosynthesis in *Escherichia coli* are narrowly distributed to Gammaproteobacteria. Nucleic Acids Res. 2013;41:3491–503.

19. Weinberg Z, Wang JX, Bogue J, Yang J, Corbino K, Moy RH, Breaker RR. Comparative genomics reveals 104 candidate structured RNAs from bacteria, archaea, and their metagenomes. Genome Biol. 2010;11:R31.
20. Guillier M, Allemand F, Raibaud S, Dardel F, Springer M, Chiaruttini C. Translational feedback regulation of the gene for L35 in *Escherichia coli* requires binding of ribosomal protein L20 to two sites in its leader mRNA: A possible case of ribosomal RNA-messenger RNA molecular mimicry. RNA. 2002;8:878–89.
21. Aseev LV, Bylinkina NS, Boni IV. Regulation of the *rplY* gene encoding 5S rRNA binding protein L25 in *Escherichia coli* and related bacteria. RNA. 2015;21:851–61.
22. Weinberg Z, Barrick JE, Yao Z, Roth A, Kim JN, Gore J, et al. Identification of 22 candidate structured RNAs in bacteria using the CMfinder comparative genomics pipeline. Nucleic Acids Res. 2007;35:4809–19.
23. Meyer MM, Ames TD, Smith DP, Weinberg Z, Schwalbach MS, Giovannoni SJ, Breaker RR. Identification of candidate structured RNAs in the marine organism *'Candidatus* Pelagibacter ubique'. BMC Genomics. 2009;10:268.
24. Matelska D, Purta E, Panek S, Boniecki MJ, Bujnicki JM, Dunin-Horkawicz S. S6:S18 ribosomal protein complex interacts with a structural motif present in its own mRNA. RNA. 2013;19:1341–8.
25. Fu Y, Deiorio-Haggar K, Soo MW, Meyer MM. Bacterial RNA motif in the 5´ UTR of *rpsF* interacts with an S6:S18 complex. RNA. 2014;20:168–76.
26. Naville M, Gautheret D. Premature terminator analysis sheds light on a hidden world of bacterial transcriptional attenuation. Genome Res. 2010;11:R97.
27. Slinger BL, Deiorio-Haggar K, Anthony JS, Gilligan MM, Meyer MM. Discovery and validation of novel and distinct RNA regulators for ribosomal protein S15 in diverse bacterial phyla. BMC Genomics. 2014;15:657.
28. Serganov A, Polonskaia A, Ehresmann B, Ehresmann C, Patel DJ. Ribosomal protein S15 represses its own translation via adaptation of an rRNA-like fold within its mRNA. EMBO J. 2003;22:1898–908.
29. Parsons GD, Mackie GA. Expression of the gene for ribosomal protein S20: Effects of gene dosage. J Bacteriol. 1983;154:152–60.
30. Rivas E, Clements J, Eddy SR. A statistical test for conserved RNA structure shows lack of evidence for structure in lncRNAs. Nat Methods. 2017;14:45–8.
31. O'Leary NA, Wright MW, Brister JR, Ciufo S, Haddad D, McVeigh R, et al. Reference sequence (RefSeq) database at NCBI: current status, taxonomic expansion, and functional annotation. Nucleic Acids Res. 2016;44:D733-D745.
32. Benson DA, Cavanaugh M, Clark K, Karsch-Mizrachi I, Lipman DJ, Ostell J, Sayers EW. GenBank. Nucleic Acids Res. 2013;41:D36-42.

33. Babski J, Haas KA, Näther-Schindler D, Pfeiffer F, Förstner KU, Hammelmann M, et al. Genome-wide identification of transcriptional start sites in the haloarchaeon *Haloferax volcanii* based on differential RNA-Seq (dRNA-Seq). BMC Genomics. 2016;17:1–19.
34. Jäger D, Förstner KU, Sharma CM, Santangelo TJ, Reeve JN. Primary transcriptome map of the hyperthermophilic archaeon *Thermococcus kodakarensis*. BMC Genomics. 2014;15:684.
35. González G, Labastida A, Jímenez-Jacinto V, Vega-Alvarado L, Olvera M, Morett E, Juárez K. Global transcriptional start site mapping in *Geobacter sulfurreducens* during growth with two different electron acceptors. FEMS Microbiol Lett 2016. doi:10.1093/femsle/fnw175.
36. Jeong Y, Kim J-N, Kim MW, Bucca G, Cho S, Yoon YJ, et al. The dynamic transcriptional and translational landscape of the model antibiotic producer *Streptomyces coelicolor* A3(2). Nat Commun. 2016;7:11605.
37. Pfeifer-Sancar K, Mentz A, Rückert C, Kalinowski J. Comprehensive analysis of the *Corynebacterium glutamicum* transcriptome using an improved RNAseq technique. BMC Genomics. 2013;14:888.
38. Wiegand S, Dietrich S, Hertel R, Bongaerts J, Evers S, Volland S, et al. RNA-Seq of *Bacillus licheniformis*: Active regulatory RNA features expressed within a productive fermentation. BMC Genomics. 2013;14:1–20.
39. Choe D, Szubin R, Dahesh S, Cho S, Nizet V, Palsson B, Cho B-K. Genome-scale analysis of Methicillin-resistant *Staphylococcus aureus* USA300 reveals a tradeoff between pathogenesis and drug resistance. Sci Rep. 2018;8:2215.
40. Thomason MK, Bischler T, Eisenbart SK, Förstner KU, Zhang A, Herbig A, et al. Global transcriptional start site mapping using differential RNA sequencing reveals novel antisense RNAs in *Escherichia coli*. J Bacteriol. 2015;197:18–28.
41. Shao W, Price MN, Deutschbauer AM, Romine MF, Arkin AP. Conservation of transcription start sites within genes across a bacterial genus. MBio. 2014;5:e01398-14.
42. Hilzinger JM, Raman V, Shuman KE, Eddie BJ, Hanson TE. Differential RNA Sequencing Implicates Sulfide as the Master Regulator of S0 Metabolism in *Chlorobaculum tepidum* and Other Green Sulfur Bacteria. Appl Environ Microbiol 2018. doi:10.1128/AEM.01966-17.
43. Nakagawa A, Nakashima T, Taniguchi M, Hosaka H, Kimura M, Tanaka I. The three-dimensional structure of the RNA-binding domain of ribosomal protein L2; a protein at the peptidyl transferase center of the ribosome. EMBO J. 1999;18:1459–67.
44. Egebjerg J, Christiansen J, Garrett RA. Attachment sites of primary binding proteins L1, L2 and L23 on 23 S ribosomal RNA of *Escherichia coli*. J Mol Biol. 1991;222:251–64.
45. Gulle H, Hoppe E, Osswald M, Greuer B, Brimacombe R, Stöffler G. RNA-protein cross-linking in *Escherichia coli* 50S ribosomal subunits; determination of sites on 23S RNA that are cross-linked to proteins L2, L4, L24 and L27 by treatment with 2-iminothiolane. Nucleic Acids Res. 1988;16:815–32.
46. Uchiumi T, Sato N, Wada A, Hachimori A. Interaction of the Sarcin/Ricin Domain of 23 S Ribosomal RNA with Proteins L3 and L6. J Biol Chem. 1999;274:681–6.

47. Leffers H, Egebjerg J, Andersen A, Christensen T, Garrett RA. Domain VI of *Escherichia coli* 23 S ribosomal RNA. J Mol Biol. 1988;204:507–22.
48. Stelzl U, Zengel JM, Tovbina M, Walker M, Nierhaus KH, Lindahl L, Patel DJ. RNA-structural mimicry in *Escherichia coli* ribosomal protein L4-dependent regulation of the S10 operon. J Biol Chem. 2003;278:28237–45.
49. Allen T, Shen P, Samsel L, Liu R, Lindahl L, Zengel JM. Phylogenetic analysis of L4-mediated autogenous control of the S10 ribosomal protein operon. J Bacteriol. 1999;181:6124–32.
50. Spierer P, Zimmermann RA. RNA-protein interactions in the ribosome. J Mol Biol. 1976;103:647–53.
51. Spierer P, Zimmermann RA, Mackie GA. RNA-Protein Interactions in the Ribosome. Binding of 50-S-Subunit Proteins to 5′ and 3′ Terminal Segments of the 23-S RNA. Eur J Biochem. 1975;52:459–68.
52. Klein DJ, Moore PB, Steitz TA. The roles of ribosomal proteins in the structure assembly, and evolution of the large ribosomal subunit. J Mol Biol. 2004;340:141–77.
53. Choonee N, Even S, Zig L, Putzer H. Ribosomal protein L20 controls expression of the *Bacillus subtilis infC* operon via a transcription attenuation mechanism. Nucleic Acids Res. 2007;35:1578–88.
54. Guillier M, Allemand F, Dardel F, Royer CA, Springer M, Chiaruttini C. Double molecular mimicry in *Escherichia coli*: Binding of ribosomal protein L20 to its two sites in mRNA is similar to its binding to 23S rRNA. Mol Microbiol. 2005;56:1441–56.
55. Lu M, Steitz TA. Structure of *Escherichia coli* ribosomal protein L25 complexed with a 5S rRNA fragment at 1.8-Å resolution. Proc Natl Acad Sci USA. 2000;97:2023–8.
56. Stoldt M, Wöhnert J, Ohlenschläger O, Görlach M, Brown LR. The NMR structure of the 5S rRNA E-domain-protein L25 complex shows preformed and induced recognition. EMBO J. 1999;18:6508–21.
57. Davies C, Gerstner RB, Draper DE, Ramakrishnan V, White SW. The crystal structure of ribosomal protein S4 reveals a two-domain molecule with an extensive RNA-binding surface: One domain shows structural homology to the ETS DNA-binding motif. EMBO J. 1998;17:4545–58.
58. Vartikar JV, Draper DE. S4-16 S ribosomal RNA complex. J Mol Biol. 1989;209:221–34.
59. Draper DE. How do proteins recognize specific RNA sites? New clues from autogenously regulated ribosomal proteins. Trends Biochem. Sci. 1989;14:335–8.
60. Grondek JF, Culver GM. Assembly of the 30S ribosomal subunit: Positioning ribosomal protein S13 in the S7 assembly branch. RNA. 2004;10:1861–6.
61. Powers T, Stern S, Changchien L-M, Noller HF. Probing the assembly of the 3′ major domain of 16 S rRNA. J Mol Biol. 1988;201:697–716.
62. Philippe C, Portier C, Mougel M, Grunberg-Manago M, Ebel JP, Ehresmann B, Ehresmann C. Target site of *Escherichia coli* ribosomal protein S15 on its messenger RNA. J Mol Biol. 1990;211:415–26.
63. Serganov AA, Masquida B, Westhof E, Cachia C, Portier C, Garber M, et al. The 16S rRNA binding site of *Thermus thermophilus* ribosomal protein S15: Comparison with *Escherichia coli* S15, minimum site and structure. RNA. 1996;2:1124–38.

64. Adilakshmi T, Ramaswamy P, Woodson SA. Protein-independent folding pathway of the 16S rRNA 5′ domain. J Mol Biol. 2005;351:508–19.
